# Effect of Solvents on Proline Modified at the Secondary Sphere: A Multivariate Exploration

**DOI:** 10.1021/acs.joc.1c02778

**Published:** 2022-01-12

**Authors:** Danilo M. Lustosa, Shahar Barkai, Ido Domb, Anat Milo

**Affiliations:** Department of Chemistry, Ben-Gurion University of the Negev, Beer Sheva 84105, Israel

## Abstract

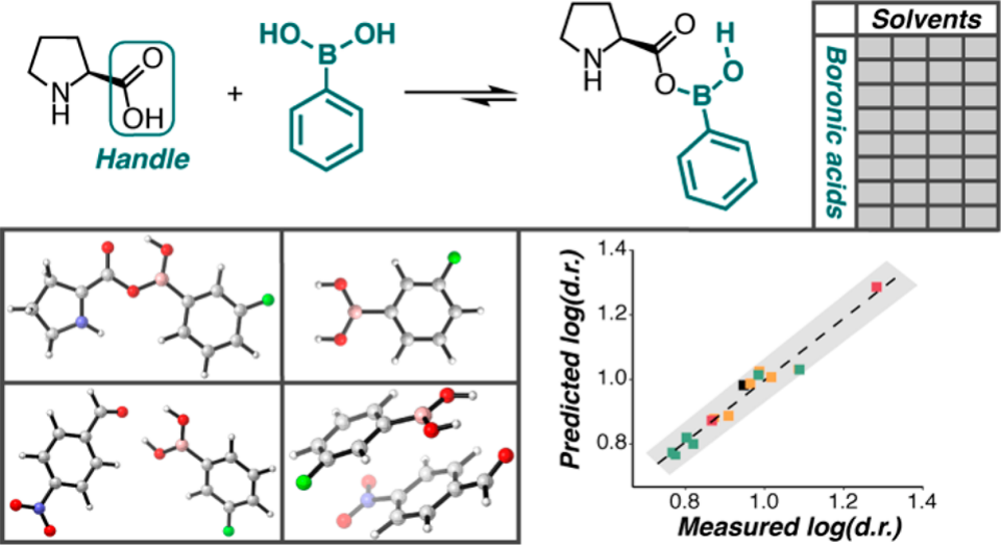

The critical influence of solvent effects on proline-catalyzed aldol reactions has been extensively described. Herein, we apply multivariate regression strategies to probe the influence of different solvents on an aldol reaction catalyzed by proline modified at its secondary sphere with boronic acids. In this system, both *in situ* binding of the boronic acid to proline and the outcome of the aldol reaction are impacted by the solvent-controlled microenvironment. Thus, with the aim of uncovering mechanistic insight and an ancillary aim of identifying methodological improvements, we designed a set of experiments, spanning 15 boronic acids in five different solvents. Based on hypothesized intermediates or interactions that could be responsible for the selectivity in these reactions, we proposed several structural configurations for the library of boronic acids. Subsequently, we compared the statistical models correlating the outcome of the reaction in different solvents with molecular descriptors produced for each of these proposed configurations. The models allude to the importance of different interactions in controlling selectivity in each of the studied solvents. As a proof-of-concept for the practicality of our approach, the models in chloroform ultimately led to lowering the ketone loading to only two equivalents while retaining excellent yield and enantio- and diastereo-selectivity.

## Introduction

The pursuit of reactions that deliver levels of selectivity similar to those provided by enzymes is at the core of asymmetric catalysis.^1^ The synthesis and derivatization of scaffolds that resemble enzymatic active sites have led to countless achievements, yet isolating active sites from their environment also comes with its drawbacks. Notably, in an enzyme, both the substrate and the active site have an ideal microenvironment to interact and react selectively.^2^ Conversely, in a reaction flask, the interaction between a substrate and molecular catalyst is controlled by the interplay between their steric and electronic characteristics, which in turn influences the way they assemble into a reactive intermediate. Further confounding the situation, this interplay is highly influenced by the surrounding solvent molecules.^3^ This stark difference is exemplified by the observation that many reactions that occur at room temperature enzymatically, oftentimes need low temperatures and long reaction times *in vitro* to afford desirable levels of selectivity.^4^ In organocatalysis, wherein selectivity is often governed by noncovalent interactions,^5^ solvents significantly impact the structural organization and degrees of freedom of intermediates and transition states.^1c,6−13^ This pronounced sensitivity of reaction outcomes to solvent changes has led to unusual solvent combinations.

**Figure 1:**
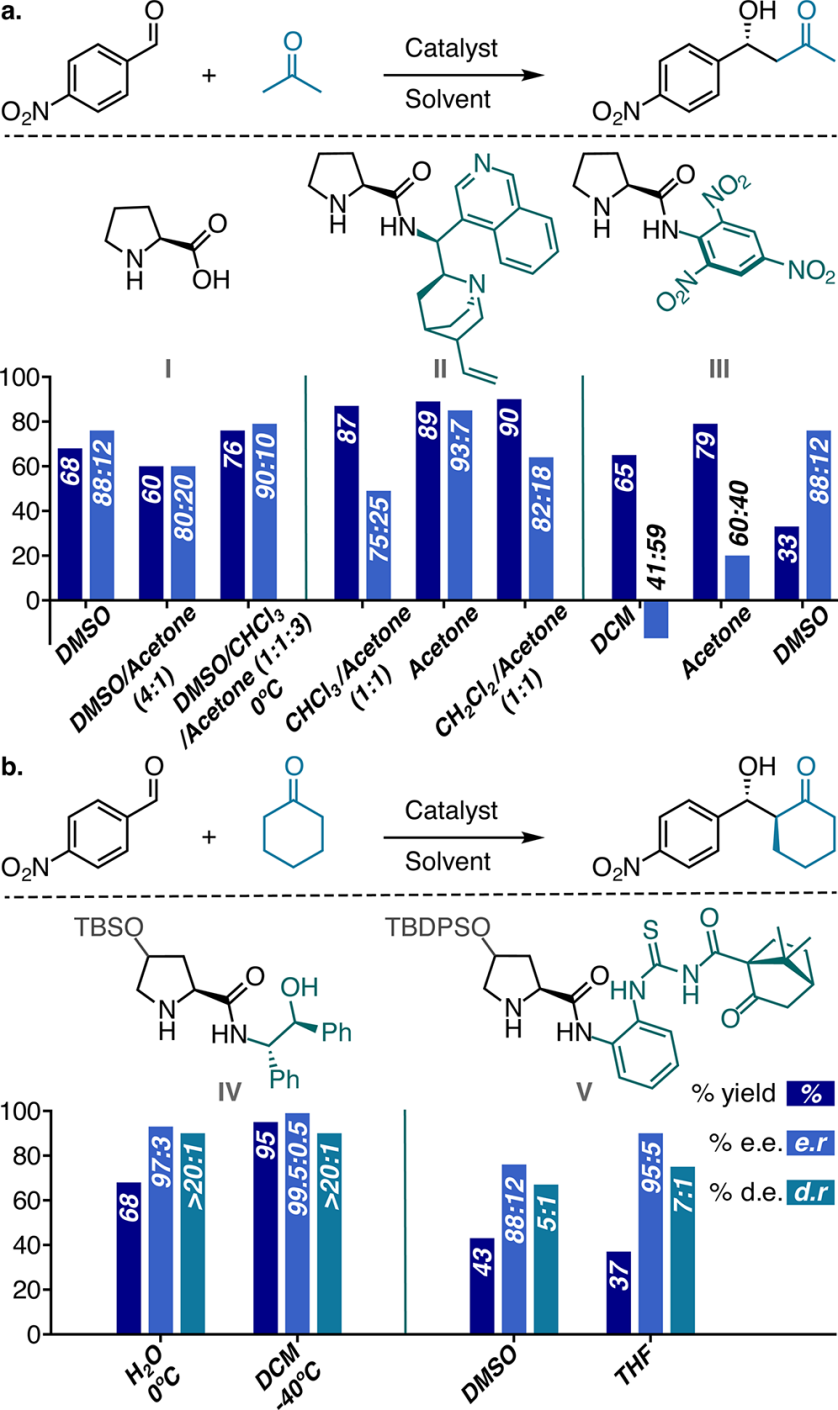
Solvent effects on the outcome of aldol reactions with different catalysts using (a) acetone as aldol donor and (b) cyclohexanone as aldol donor. The bar graphs represent % of yield (dark blue), % of enantiomeric excess (ee, light blue), and % of diastereomeric excess (de, teal) in each reaction; the values of yield, dr, and er are noted on their respective bars.

The role of solvent variation on proline-catalyzed aldol reactions was already described in the seminal work by List, Lerner and Barbas, who revealed ranges of 67:33 to 88:12 enantiomeric ratio (er) as a response to solvent variation (Figure 1.a, catalyst I).^1c,6^ Subsequently, List demonstrated that the addition of chloroform (CHCl_3_) to a dimethyl sulfoxide (DMSO)/acetone solvent system, could speed up the reaction, minimize elimination and increase er.^7^ Similar phenomena were observed for this reaction when using more structurally complex catalysts. Xiao and co-workers demonstrated that catalyst **II** in CHCl_3_ afforded the aldol adduct with 75:25 er, while increased er was obtained in acetone or a 1:1 mixture of acetone and dichloromethane (DCM). An even more drastic response to solvent variations was identified by Shirai and co-workers (Figure 1.a, catalyst **III**), who reported that DCM and acetone delivered opposite enantiomers.^9^ Furthermore, changing the solvent to DMSO modulated the er to 88:12. The impact of solvent on cyclic ketones as aldol donors is also marked (Figure 1.b). Gong and co-workers, for instance, demonstrated that by using catalyst **IV** with water as solvent, the aldol product of cyclohexanone was furnished with dr of >20:1 and er of 97:3 at O °C.^10^ In contrast, when the same reaction was performed in DCM, a low temperature of −40 °C was required to obtain the same level of diastereoselectivity.^11^ In addition, Chen and co-workers, observed a strong dependency between the solvent for these reactions and their enantioselectivity.^12^ Using catalyst **V** they observed an increase in enantioselectivity when moving from DMSO to THF.

**Figure 2:**
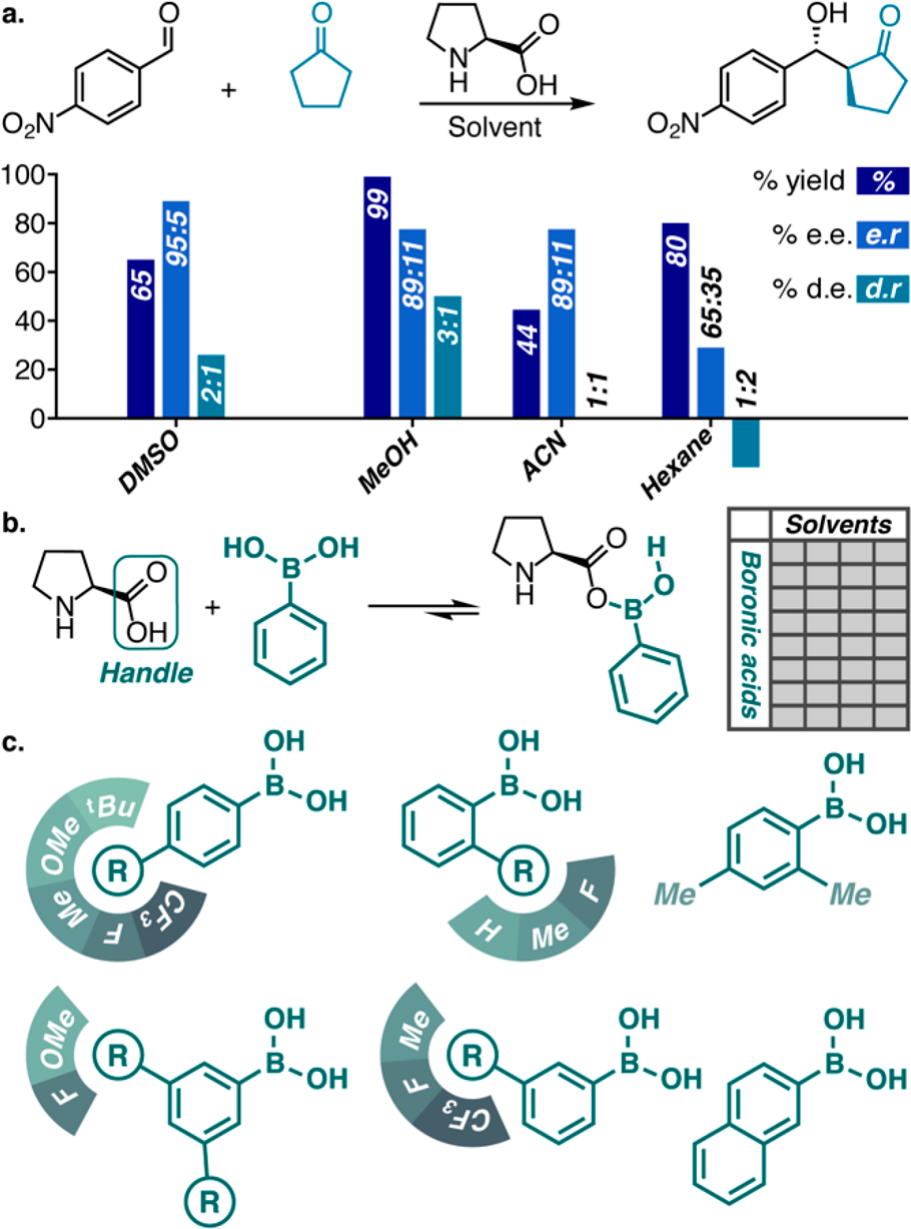
(a) Solvent effects on the outcome of proline catalyzed aldol reactions of cyclopentanone; (b) data set design based on a systematic variation of different solvents and different boronic acids as secondary sphere modifiers; (c) boronic acids selected for the data set based on their diverse substitution patterns as well as steric and electronic properties.

Cyclopentanone as aldol donor is much less studied compared to its 6-membered ring counterpart, which piqued our interest in its application, yet finding systematic results demonstrating solvent effects was challenging. Therefore, to test its sensitivity to solvent effects in proline catalysis, we conducted reactions in hexane, methanol and acetonitrile and compared them to those reported by Barbas in DMSO (Figure 2.a).^13^ We observed that the er could vary from 65:35 in hexane to 95:5 in DMSO. Additionally, the diastereoselectivity goes all the way from 3:1 in favor of the *anti*-product in methanol to 1:2 in favor of the *syn*-product in hexane. Whereas solvent clearly plays an important role in the selectivity and reactivity of amino-catalyzed aldol reactions, mechanistic studies that clearly describe the influence of different solvents on these reactions are scarce. Recently, Yang and co-workers evaluated the effect of organic solvents and water on proline catalyzed aldol reactions by explicitly placing a handful of solvent molecules around the solute to simulate a first solvation sphere.^14^ Yet generally, despite the great contributions DFT studies have provided to understanding solvent effects,^15^ modeling solvents is challenging.^16^ Moreover, the accuracy of computational methods is limited when small changes in energy reflect large changes in stereoselectivity.^17^

As part of our ongoing program on secondary sphere modified organocatalysts,^18^ we recently reported the application of amino catalysts in an aldol reaction between aromatic aldehydes and cyclopentanone (Figure 2.a).^19^ By modifying proline with boronic acids *in situ* we were able to rapidly access different organocatalyst derivatives without any synthetic effort. Taking advantage of the broad spread of data stemming from our secondary-sphere modification, we set out to study the influence of solvent effects on the aldol reaction. Solvent effects are of particular interest in secondary sphere modified catalysts, not only because of the additional degrees of structural freedom the modifier confers, but also due to the required binding stability under different reaction conditions. It is worth highlighting that the concept of secondary sphere modification is inspired by enzymatic catalysis, yet the modifiers impart less spatial constrains than an enzymatic microenvironment. Therefore, the role of solvent molecules is paramount in controlling the structure of intermediates and transition states that determine reactivity and selectivity.

## Results and Discussion

In our aforementioned work,^19^ the combination of proline with boronic acids led to unprecedent levels of diastereoselectivity, short reaction-times and a broad scope. The optimized conditions required 18 equiv of neat cyclopentanone with respect to the aldehyde because we were unable to identify a solvent that provided satisfactory yield or diastereoselectivity. It is often the case that proline-catalyzed aldol reactions are performed in neat ketone, which represents a formidable drawback for its practical use. Given our interest in understanding the intertwined effect of solvents on binding and reactivity in our system, we were keen to design a data set based on the systematic variation of both solvent and secondary sphere modifier (Figure 2.b). We were not discouraged by our previous solvent screening, because it was the result of a univariate optimization of a handful of solvents with a single boronic acid modifier. In contrast, our new data set was planned to deconvolute the effects of different secondary sphere modifiers in a diverse set of solvents. We postulated that this design would not only uncover mechanistic insight, but could potentially lead to methodological improvements.

**Figure 3:**
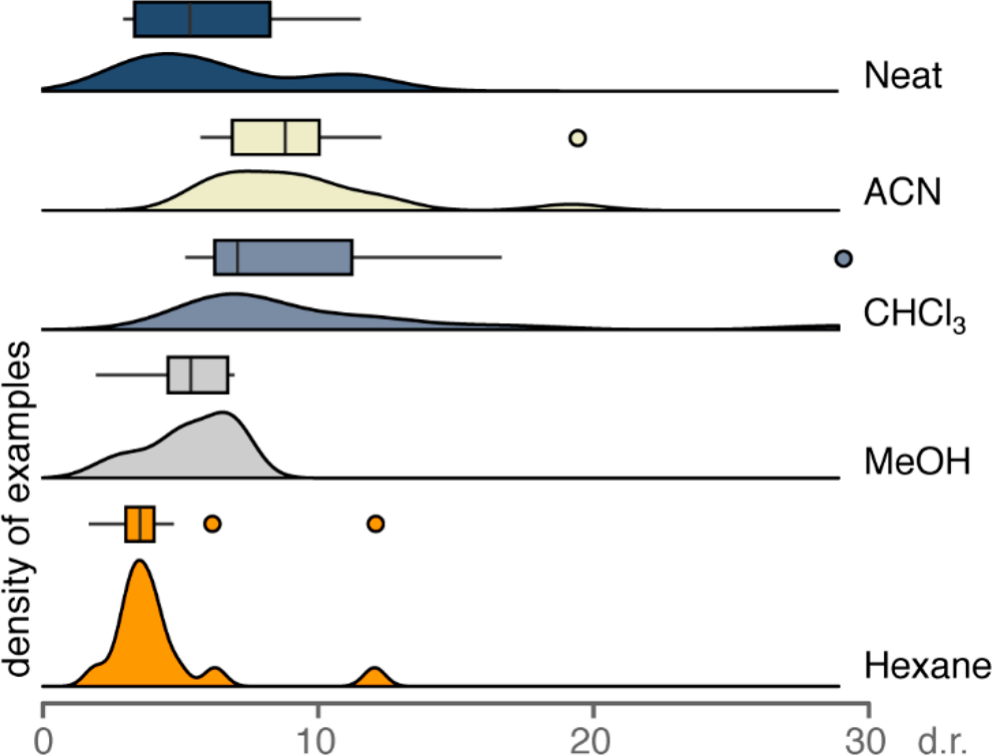
Probability densities (curves) and distributions (box plots) of the experimental dr values obtained in the tested solvents. The height of the curves reflects the probability of a given dr in each solvent, whereas the boxes represent the margin between the 25th and the 75th percentiles, with an inner line indicating the median and single points indicating outliers.

With the variance of our data set in mind, we aimed to compare our neat reaction conditions to reactions in hexane, CHCl_3_, acetonitrile (ACN), and methanol (MeOH). With the aim of obtaining a broad spread of results suitable for modeling, boronic acids with different steric, electronic, and substitution patterns were represented in the data set (Figure 2.c). With 5 solvents and 15 boronic acids, we designed a total of 75 reactions and 75 duplicates. All of these 150 reactions are independent of each other, which allows for their parallel execution. We first focused on dr values because these appeared to change significantly from solvent to solvent with different boronic acids. We note that dr values are well distributed for neat, ACN and CHCl_3_ conditions, whereas for hexane and MeOH they do not substantially vary as a function of boronic acid (see Figure 3).

**Figure 4:**
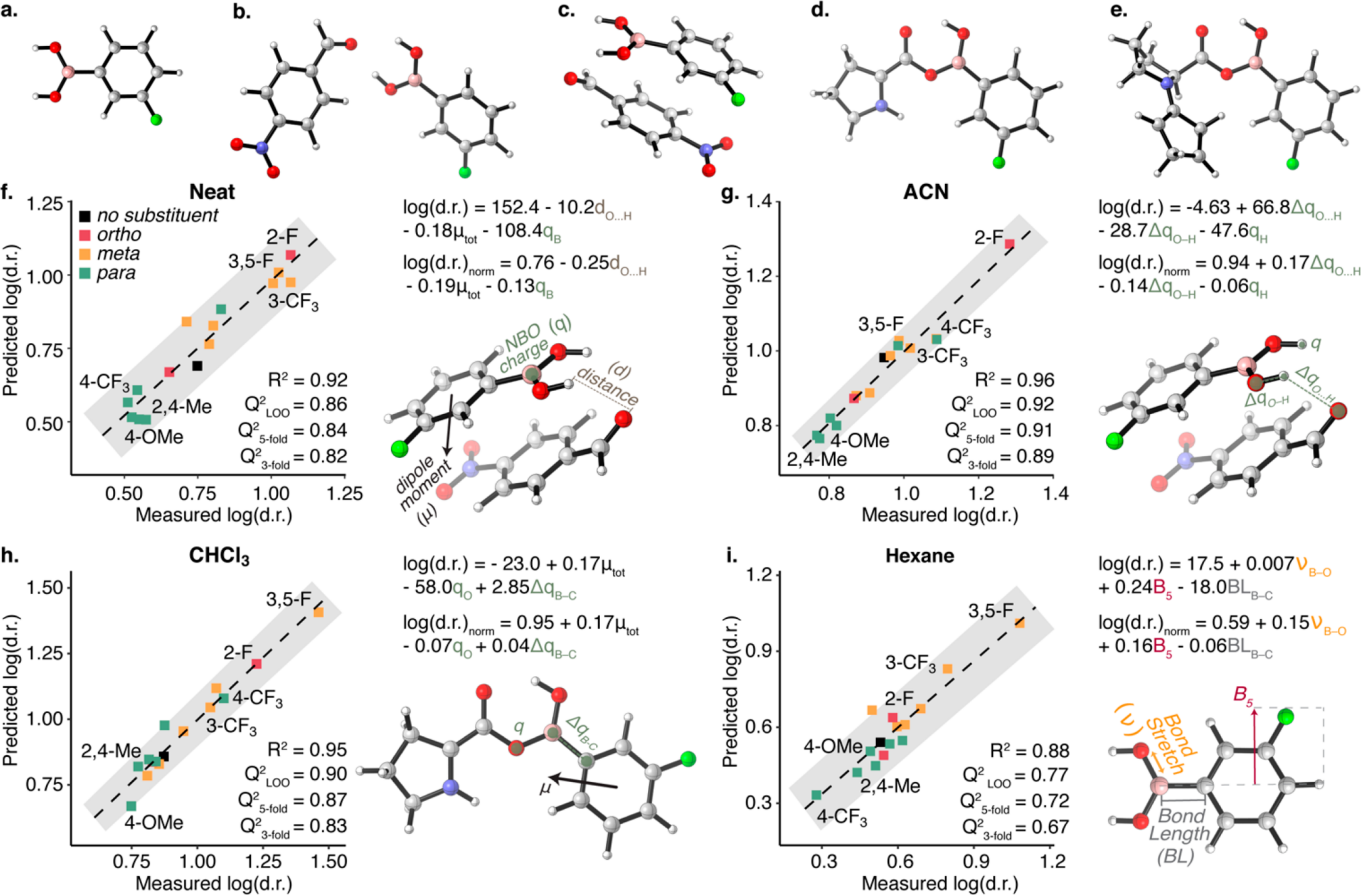
Several optimized structures used in parametrization: (a) boronic acid; (b) H-bonding interaction between boronic acid and 4-nitrobenzaldehyde; (c) π-interaction between boronic acid and 4-nitrobenzaldehyde; (d) boronic acid bound to proline; (e) boronic acid bound to enamine. The best fitted multivariate linear regression models for the dr with varying boronic acid substituents in (f) neat cyclopentanone (18 equiv), (g) acetonitrile (ACN) as solvent with 6.8 equiv of cyclopentanone; (h) CHCl_3_ as solvent with 6.8 equiv of cyclopentanone, (i) hexane as solvent with 6.8 equiv of cyclopentanone. Parameters that were identified as predictive for each system are presented with their corresponding structure. The goodness-of-fit of each model is indicated by *R*^2^ and *Q*^2^ for 3-fold (500 iterations), 5-fold (500 iterations), and leave-one-one (LOO) cross validations (see SI section 21 for details). The equations predicting log(dr) are added to the right of each plot for normalized and raw parameters.

As a part of our modeling strategy, molecular libraries of boronic acid derivatives were produced for several possible structural configurations. These configurations were selected based on mechanistic hypotheses from the proline-catalyzed aldol-reaction literature,^20^ as well as our own experience with noncovalent interactions and secondary sphere modifiers in organocatalysis.^18,19^ Each of these sets of molecular libraries represents an intermediate or interaction that may come into play in determining selectivity (Figure 4.a-e.).^21^ We postulated that different libraries could lead to better correlations with the experimental outcome in each solvent. Furthermore, we assumed that even if the same library would lead to the best models in different solvents, the specific parameters that appear in each model and their relative contribution would still be informative. This approach could serve to pinpoint which interaction contributes more significantly to the selectivity in a given solvent.

Molecular descriptors were extracted for each library, correlated against all of the experimental data and the best performing model was identified (a report including all models produced in this work is appended to the SI).^21^ The parameters used in this process were intended to capture both the electronic and steric variation due to different substitution patterns. The electronic nature of the structures is represented by NBO charges, and their steric nature is depicted by bond lengths, distances, and dihedral angles as well as Sterimol parameters. The directional components of the dipole moment and specific bond stretching frequencies are stereoelectronic hybrids affected by the location, size, mass, and electronic nature of the substituents. The components of the dipole moment for all of the structures were taken with respect to the center of the boronic acid aromatic ring. The models with the highest goodness-of-fit *Q*^2^ leave-one-out cross-validation values were identified for each proposed structure in each solvent system (see SI section 21 for a detailed explanation of cross validation). We decided to use this *Q*^2^ value as the selection criterion rather than the *R*^2^ because it not only reflects the goodness-of-fit but also the predictive robustness of each model, thus minimizing the likelihood of overfitted models being selected.

Under neat reaction conditions, wherein 18 equiv of cyclopentanone were used as both reactant and solvent, the model with the best fit was based on the boronic acid–aldehyde π interaction structure (Figure 4.f). The parameters that appeared in this model were the distance between the aldehyde’s oxygen and one of the hydrogens of B(OH)_2_, the overall dipole moment, and the charge on the boron atom. The best model for the reactions in ACN was also based on the boronic acid–aldehyde π interaction structure (Figure 4.g). The parameters that appeared in this model were the charge on one of the boronic acid hydrogens, the difference in charge between the other boronic acid hydrogen and oxygen, and the difference in charge between the same hydrogen and the aldehyde oxygen. The best model for the reaction in CHCl_3_ was based on the bound boronic acid–proline structure (Figure 4.h). The parameters that appeared in this model were the overall dipole moment, the charge on the oxygen that binds boronic acid with proline, and the difference in charge between the boron and carbon on the boronic acid. The best performing model in hexane had a lower *Q*^2^ value compared to the models in other solvents, which we attribute to the narrow distribution of results in this solvent (Figure 3). Nevertheless, the model stemming from the structure of the boronic acid alone provided a decent fit in hexane (Figure 4.i). The parameters that appeared in this model were the stretching frequency vibration between the boron and one of the oxygens, the bond length between the boron and carbon, and the B_5_ Sterimol parameter representing the maximal width of the boronic acid. All of the attempts to identify a model for the dr obtained in MeOH resulted in poor correlations. Based on the lack of correlation and the extremely low er values observed for this reaction, we tested whether there was a background reaction interfering with our catalytic process. Indeed, even in the absence of both proline and boronic acid, we observed a racemic aldol reaction in MeOH which led to poor dr and er values with all of the boronic acids (see Table S4).

**Figure 5:**
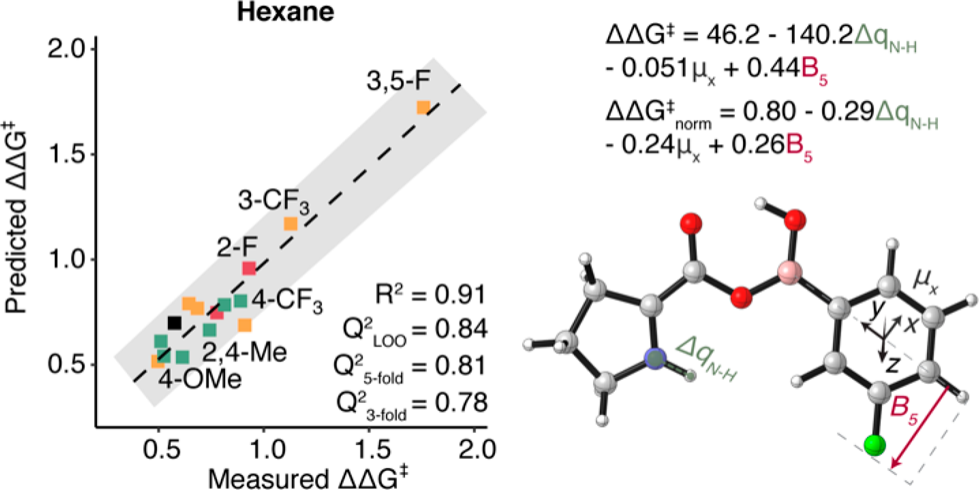
Multivariate linear regression model for enantioselectivity (in kcal/mol based on ΔΔ*G*^⧧^ = −*RT* ln[er]) with hexane as a solvent. Parameters identified as predictive for this system are presented with the corresponding optimized structure. The goodness-of-fit is indicated by *R*^2^ and *Q*^2^ for 3-fold (500 iterations), 5-fold (500 iterations), and leave-one-one (LOO) cross validations. The equations predicting ΔΔ*G*^⧧^ are added to the right of the plot for normalized and raw parameters.

In all of the solvents besides MeOH and hexane the er values were excellent for all boronic acids tested. In hexane, as opposed to MeOH, the er varied significantly as a result of the boronic acid structure. This variation enabled correlating the libraries of descriptors to enantioselectivity in hexane and the best fit was based on the bound boronic acid–proline structure (Figure 5). The parameters that appeared in this model were the difference in charges between the nitrogen and hydrogen atoms, the Sterimol B_5_ width on the boronic acid aryl ring, and the component of the dipole moment on the plane of the aryl ring in the direction of the *ortho* and *meta* positions. Based on the assumption that both stereocenters are formed in the same step, we were expecting to identify models stemming from the same structural configuration for both the enantio- and diastereo-selectivity. Nevertheless, these types of selectivity are controlled by the face from which and with which the aldehydes approach.^20^ Therefore, it is not unlikely that they may be decoupled because of the fine balance between possible spatial arrangements of the reaction components. Moreover, since the diastereoselectivity is fairly low in hexane showing a narrow distribution in response to changes in the structure of boronic acid, it could indicate an averaging across different configurations. Thus, the fairly good fit of the model with boronic acid alone could in fact also represent an averaging across different structures or interactions in which boronic acid takes part. Enantioselectivity, on the other hand, seems to be more significantly impacted by different boronic acids and is perhaps more stringently controlled by a specific interaction or intermediate represented by the structure of boronic acid bound to proline.

As stated above, we set out to study solvent effects with the intention of uncovering mechanistic aspects of our secondary-sphere guided aldol reaction, but in the process were also hoping to identify conditions with lower aldol donor loading. Gratifyingly, the excellent experimental results obtained using CHCl_3_ or ACN as solvents with only 6.8 equiv of cyclopentanone already surpassed our previously optimized neat conditions.^19^ Given these results, we wondered whether the models could provide an indication as to which of these systems could withstand further reduction in the amount of ketone without an erosion of selectivity. For the reaction in CHCl_3_ the bound boronic acid–proline structure provided the best fit (Figure 4.h), whereas in ACN it was the boronic acid–aldehyde π interaction structure that led to the best fit (Figure 4.g). We speculated that having a bound species in the best fitted model could indicate that covalently bound species rather than noncovalently interacting ones govern selectivity. If this assumption is correct, it would be easier to lower the ketone loading in the case where stronger interactions hold the selectivity determining units together, thus indicating toward CHCl_3_ as the more appropriate system. It is noted that for reactions in CHCl_3_ the boronic acid–aldehyde π interaction structure led to a decent fit as well, whereas the bound boronic acid–proline structure led to an excellent fit for the reactions in ACN (see Figures S11–S12 for details). We assume that this disparity could indicate that both types of interactions or species come into play in both cases, yet in CHCl_3_ a covalently bound proline–boronic acid species may play a more important role.

**Figure 6:**
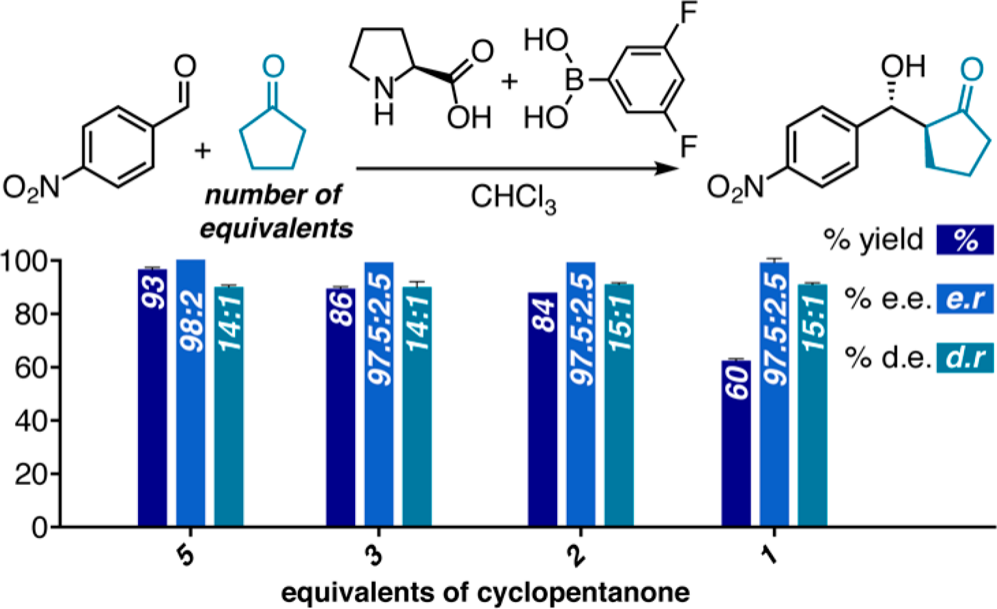
Evaluating the effect of the amount of cyclopentanone on the reaction outcome. Reactions were performed for 9 h using 0.5 mmol of aldehyde, 5 mol % of proline, and 10 mol % of 3,5-F-phenylboronic acid (for details, see Table S6).

Because the bound boronic acid–proline structure (Figure 4.d) provided an excellent fit in both cases, we turned to analyze the parameters that appear in each of these models. The most important parameter in the CHCl_3_ model was the total dipole moment (μ_tot_) on the boronic acid moiety (see Figure 4.h), whereas for ACN it was the difference in charge between the oxygen and carbon of the carbonyl on the proline moiety (see Figure S11). Carbonyl charges are tightly correlated with p*K*_a_ and Hammet values;^22^ therefore, we interpreted this parameter to reflect binding strength between the proline and boronic acids. This assumption led us to presume that binding in CHCl_3_ is strong enough to have less of an impact on selectivity because, in contrast to ACN, the most important parameter in the CHCl_3_ model did not signify binding. Taken together, these hypotheses led us to select the reaction in CHCl_3_ as a more appropriate candidate for lowering ketone loading. Indeed, when we lower the ketone loading enantio- and diastereoselectivity are preserved; however, the yields in 1 h reactions are diminished. When we moved to slightly higher reaction times we were able to lower the ketone loading to 2 equiv with excellent er and dr values (see Figure 6). We note that even a 1:1 ratio of aldehyde to ketone was possible with only a slight loss of yield (see Figure S6 for details).

**Figure 7:**
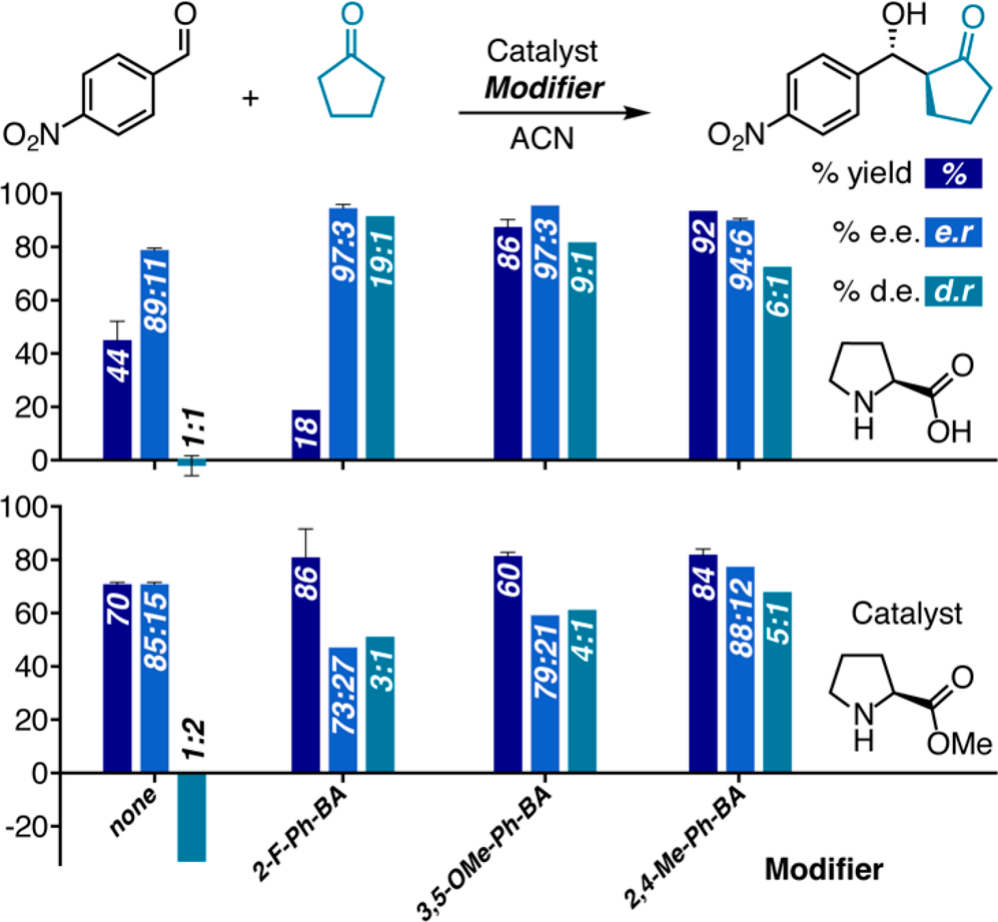
Influence of different boronic acid on reaction yield, er and dr of reactions catalyzed by proline (top) and methyl prolinate (bottom). Reactions were performed for 1h, using 0.3 mL of cyclopentanone (6.8 equiv), 0.5 mmol of aldehyde, 5 mol % of proline, and 10 mol % phenyl boronic acid derivatives (for details see SI, Table S8).

Taking into account all the models identified in the different solvents, we were surprised that the boronic acid–aldehyde π interaction structure provided the best fit under neat conditions and using ACN as solvent. These results were not in full agreement with our previous work,^19^ where both NMR and mass spectrometry suggested that the active catalyst in this system consisted of one proline unit bound to boronic acid(s). To address this discrepancy, we decided to measure mass spectra of boronic acid in the presence of proline and cyclopentanone in all of the tested solvents. Due to low solubility, this analysis was not possible in hexane; however, in ACN, CHCl_3_, and MeOH we were able to not only identify masses that can be attributed to boronic acid–proline adducts but also to their enamines (for details, see SI section 25). This result supports our hypothesis that the active catalyst could contain a boronic acid–proline adduct because such adducts seem to form a putative reactive intermediate in the catalytic cycle. To try to settle this result with a possible π interaction between the boronic acid and aldehydes, we designed a proline catalyst that cannot form an ester derivative with boronic acid. Thus, proline methyl ester was prepared and submitted to our reaction conditions in ACN with three different boronic acids that spread over the dr range (see Figure 7). In the reaction without the addition of boronic acid a 2:1 ratio was obtained in favor of the *syn*-diastereomer. Once boronic acid was added, the *anti-*diastereomer was predominantly formed; however, the differences in dr values for different boronic acids were not significant. Moreover, the trend in dr observed for this catalyst in the presence of different boronic acids is distinct from that observed with proline, where the same boronic acids spread the *syn*:*anti* ratio from 1:6 all the way up to 1:19.

These results suggest that a noncovalent interaction with boronic acid can indeed control selectivity; however the difference in trends may indicate that it is not necessarily the π interaction between unbound boronic acid and free aldehyde in both cases. For methyl prolinate, a covalent bond with boronic acid is ruled out, but besides a possible π interaction of unbound boronic acid with aldehyde, there could be an additional hydrogen-bonding interaction involving the oxygen of proline, or even a bridging hydrogen bonding interaction between boronic acid and the oxygens of the proline and aldehyde. Ultimately, these results strongly imply that the structures on which the models are based represent selectivity-controlling interactions rather than specific reaction intermediates. We note that in this case, where covalent binding to proline was blocked, the variation in dr was not significant, but the variation in er was. This is reminiscent of the er changes observed in hexane as solvent, indicating that the dr and er determination may be similarly decoupled in this system.

## Conclusions

In this work, we set out to study the influence of different solvents on proline-catalyzed aldol reactions, which are notoriously sensitive to solvent effects. Specifically, we studied their influence on reactions of proline modified *in situ* at its secondary sphere. These modifications were aimed at mimicking steric and electronic constrains that exist in the microenvironment of enzymatic active sites. However, the molecular control in these modified systems depends on the compatibility between catalytic activity and *in situ* binding of the modifier under reaction conditions. Accordingly, the impact of solvent effects here is 2-fold because they control both binding and catalysis. Yet at its core, this work echoes general issues of attaining enzymatic-like selectivity with molecular catalysts. Whereas some mechanistic aspects of this study may be specific to secondary sphere modified systems, the strategy that we developed to uncover them is broadly applicable. The models revealed statistical correlations with molecular descriptors of selected moieties and intermediates. It was also evident that the significance of each of these structures varies with the variation of solvent. For example, in ACN, we found that several of the modeled structures could lead to an excellent statistical correlation with the experimental data. This observation along with further mechanistic experiments with methyl prolinate strongly suggested that the proposed structures provided a partial picture of possible interactions, and that, in certain cases, several effects may work in concert to impart the observed selectivity. Furthermore, based on the models for ACN and CHCl_3_, we hypothesized that the latter would likely not lead to erosion of selectivity with the reduction in the amount of aldol donor. As a result, we were able to optimize the conditions of our reaction to only 2 equiv of cyclopentanone while maintaining excellent yield and enantiomeric and diastereomeric ratios.

## Experimental Section

Reactions were conducted according to general procedure 1 (GP1), general procedure 2 (GP2), and general procedure 3 (GP3); see below. With respect to the ketone/solvent ratio, we decided to start out by using 1/3 of cyclopentanone/solvent. This decision was taken because we hypothesized that in order to properly depict a solvent effect, the solvent should be used as the major component in the mixture. As every reaction used 0.8 mL of solvent + ketone mixture, we rounded up the 0.27 mL of ketone to 0.3 mL, leading to 6.8 equiv of ketone. It is important highlight that no less than 6.8 equiv of ketone was initially evaluated, as we knew from our previous study that drastically reducing the amount of ketone can lead to low conversions. It was also known that water had a marked effect on the diastereoselectivity and reactivity of these reaction.^19^ Thus, all the reactions were performed with the addition of 0.9 μL of water. In an attempt to address the effect of water, we also planned an additional set of reactions without water. Unfortunately, the conversion of starting materials and the yield of the products were so low that the NMR determination of the diastereomeric rates became challenging. As a result, we deemed these dr values untrustworthy for modeling.

### GP1—Subsets: Neat Conditions, Acetonitrile, and MeOH

A 5 mL vial, containing 5.8 g of proline (0.05 mmol) and a stirring bar, was placed under argon (with a balloon). To this flask, 25 mg of activated molecular sieves (3 Å) and a boroxine derivative (0.03 mmol) were also added. Next, 0.8 mL of a stock solution of internal standard 1,3,5-trimethoxybenzene (25 mg/mL, 0.15 M) and water (11.3 μL of water per 1 mL of solvent) in 37.5% V of cyclopentanone and 62.5% V of a given solvent was also added to the solids in the vial. The reaction was then allowed to stir for 1 h and after that quenched with a saturated solution of ammonium chloride. The organic phase was then collected, and the water phase was extracted once again with chloroform. The organic phases were then combined and dried over magnesium sulfate. Lastly, solvent was removed under reduced pressure, and the reaction crude was directly analyzed via ^1^H NMR for yield and diastereoselectivity determination and via chiral HPLC for determination of enantioselectivity.

### GP2—Subsets: Chloroform, Hexane

For the subsets in chloroform and hexane, due to low water solubility in this solvents, water was added to each individual reaction instead of being used as a component in the solvent stock solutions. A 5 mL vial, containing 5.8 g of proline (0.05 mmol) and a stirring bar, was placed under argon (with a balloon). To this flask was added 25 mg of activated molecular sieves (3 Å), and a boroxine derivative (0.03 mmol) were also added. Next, 0.8 mL of a stock solution of internal standard 1,3,5-trimethoxybenzene (25 mg/mL, 0.15 M) in 37.5% V of cyclopentanone and 62.5% V of a given solvent was also added to the solids in the vial. To this mixture was added 9 μL of water (0.5 mmol). The reaction was then allowed to stir for 1 h and after that quenched with a saturated solution of ammonium chloride. The organic phase was then collected, and the water phase was extracted once again with chloroform. The organic phases were then combined and dried over magnesium sulfate. Lastly, solvent was removed under reduced pressure, and the reaction crude was directly analyzed via ^1^H NMR for yield and diastereoselectivity determination and via chiral-HPLC for determination of enantioselectivity.

### GP3—Single Reactions

Unlike the reaction in different solvents, the reactions for studying the stoichiometry of ketone, those with methyl prolinate, and blank reactions were not performed in batch. These reactions were performed using the following procedure: proline (0.05 mmol, 5.8 mg), boroxine derivative (0.03 mmol), 1,3,5-trimethoxybenzene (20 mg, 0.12 mmol), 25 mg of molecular sieves 3 Å, 0.8 mL of cyclopentanone, and 9 μL of water were placed in a screw-capped vial under argon (with a balloon). The mixture was stirred for 15 min at ambient temperature followed by addition of aldehyde (0.5 mmol). After completion of the reaction, the reaction mixture was treated with saturated aqueous ammonium chloride solution, and the whole mixture was extracted 3 times with CHCl_3_. The organic layer was dried over sodium sulfate and concentrated to give a crude residue which was then purified via column chromatography over silica gel using hexane–ethyl acetate or hexane–DCM as an eluent to afford pure product. Diastereoselectivity and yield were determined by ^1^H NMR analysis of the crude aldol product. The ee of the aldol product was determined by chiral-phase HPLC analysis.

## Supporting Information Available

Excel sheet with all the results and parameters that were used to identify models (XLSX)
Report with the details of all of the best-fitted models identified in this work (PDF)
Detailed experimental and computational procedures, analyses, and characterizations (PDF)

## Notes

The raw data and code files are openly available in GitHub at https://github.com/Milo-group/SolEffectsJOC2022 and include all HPLC, NMR and HRMS traces, as well as all optimized xyz files, code, and data sets used for model identification.
