# Supplementary material for: Effect of Solvents on Proline Modified at the Secondary Sphere: A Multivariate Exploration
Source: J Org Chem. 2022 Jan 12;87(3):1850–7. doi: 10.1021/acs.joc.1c02778 (PMC9182215; doi:10.1021/acs.joc.1c02778)

Models Report

Neat

Only Boronic Acids

formula	R.sq	Q.sq	MAE
`output ~ X.2.9. + dip_z + B5`	0.8719851	0.7041997	0.0913893
`output ~ X.2.9. + dip_y + B5`	0.8797651	0.6979800	0.0858136

	Estimate	Std. Error	t value	Pr(>|t|)
(Intercept)	0.7602560	0.0212517	35.773953	0.0000000
X.2.9.	0.1854286	0.0234177	7.918324	0.0000072
dip_z	0.0719108	0.0224257	3.206622	0.0083556
B5	0.1186739	0.0229895	5.162081	0.0003123

3 & 5 fold CV

Q2	MAE
0.6765074	0.1068543

Q2	MAE
0.6995434	0.0954343

Top Ranked Full Model

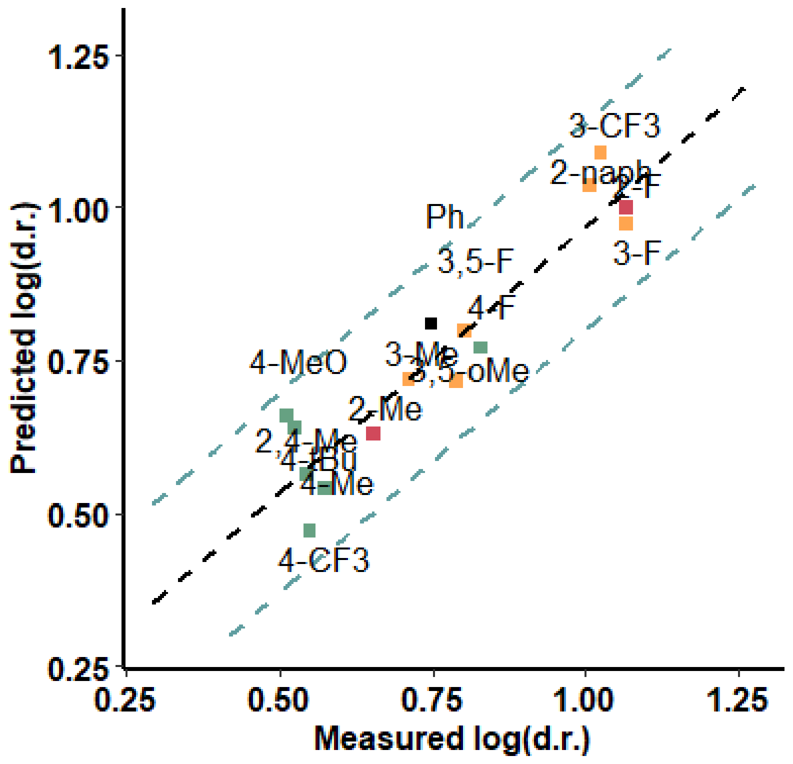

With Catalyst - Close to the active site

formula	R.sq	Q.sq	MAE
output ~ `Dist.14..18.` + `diff.O16.C17` + `L`	0.8064919	0.7229366	0.0866972
output ~ `X.17.18.` + `Dist.8..14.` + `NBO.N.19`	0.8172571	0.6967683	0.0975522

	Estimate	Std. Error	t value	Pr(>|t|)
(Intercept)	0.7602560	0.0261284	29.096958	0.0000000
Dist.14..18.	0.1909044	0.0334086	5.714222	0.0001353
diff.O16.C17	0.1736214	0.0335086	5.181403	0.0003031
L	-0.0882004	0.0271518	-3.248419	0.0077570

3 & 5 fold CV

Q2	MAE
0.6163099	0.1144414

Q2	MAE
0.6709842	0.0979151

Top Ranked Full Model

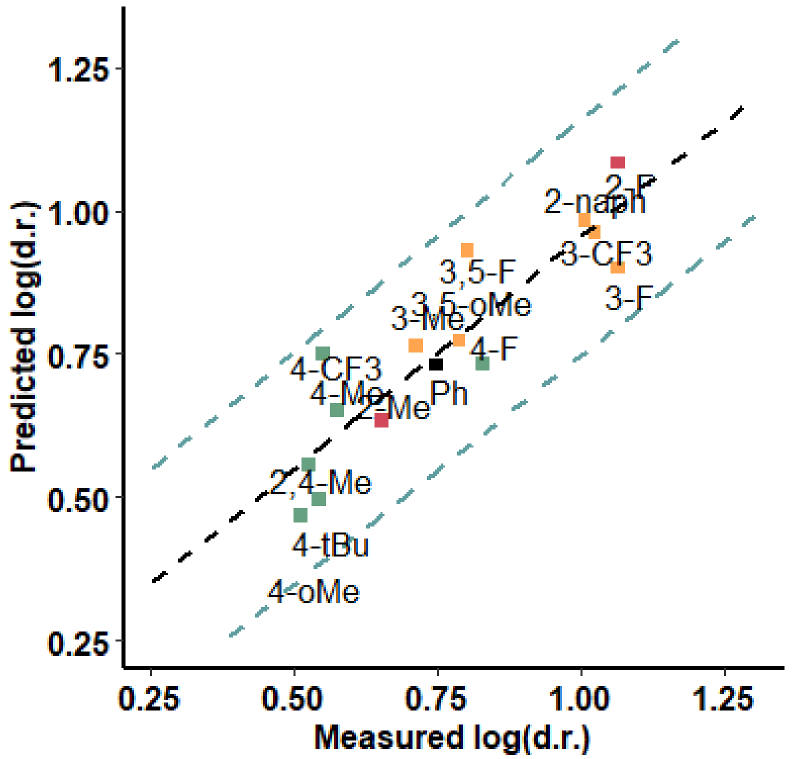

With Catalyst - far from the active site

formula	R.sq	Q.sq	MAE
output ~ dip_y + diff.C17.O18 + B5	0.8527290	0.7266616	0.0844181
output ~ dip_y + diff.H14.O18 + B5	0.8530859	0.7177136	0.0905937

	Estimate	Std. Error	t value	Pr(>|t|)
(Intercept)	0.7602560	0.0227940	33.353277	0.0000000
dip_y	0.1038558	0.0253244	4.101012	0.0017564
diff.C17.O18	-0.1751757	0.0251146	-6.975060	0.0000234
B5	0.0705326	0.0238044	2.963002	0.0129058

3 & 5 fold CV

Q2	MAE
0.6816969	0.0994453

Q2	MAE
0.7016755	0.0922093

Top Ranked Full Model

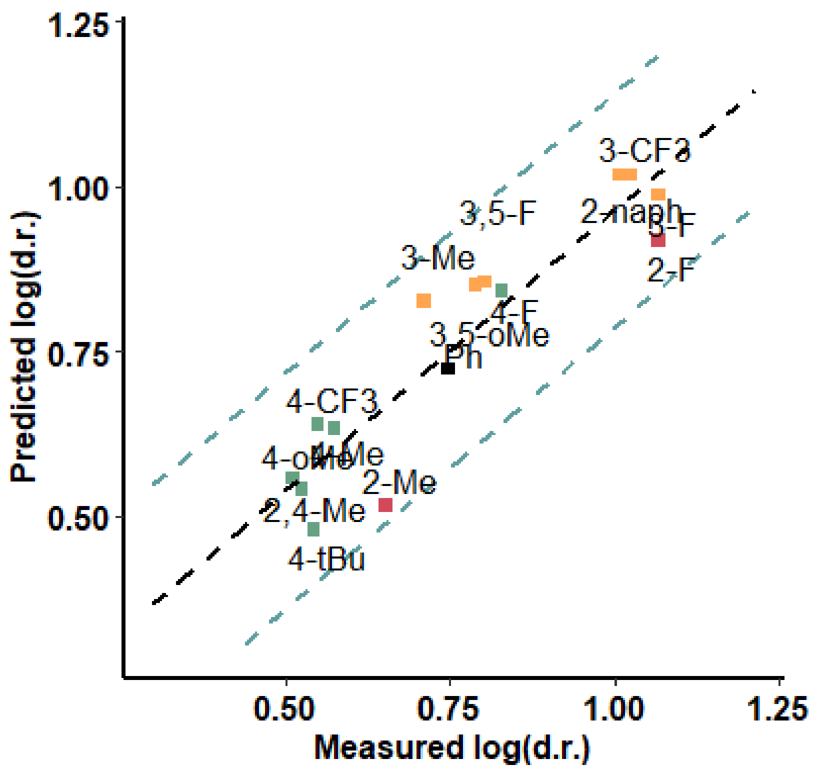

BA + Pentanone

formula	R.sq	Q.sq	MAE
output ~ X.1.2. + X.17.18. + Dist.17..18.	0.8314757	0.7407062	0.0807623
output ~ X.2.3. + X.2.9. + NBO.O.9	0.8148084	0.7324720	0.0748838

	Estimate	Std. Error	t value	Pr(>|t|)
(Intercept)	0.7602560	0.0243834	31.179262	0.0000000
X.1.2.	0.1329706	0.0305909	4.346735	0.0011617
X.17.18.	0.9213506	0.1753522	5.254287	0.0002708
Dist.17..18.	0.9177942	0.1751990	5.238583	0.0002774

3 & 5 fold CV

Q2	MAE
0.5954084	0.1111396

Q2	MAE

0.6587516	0.0944145

Top Ranked Full Model

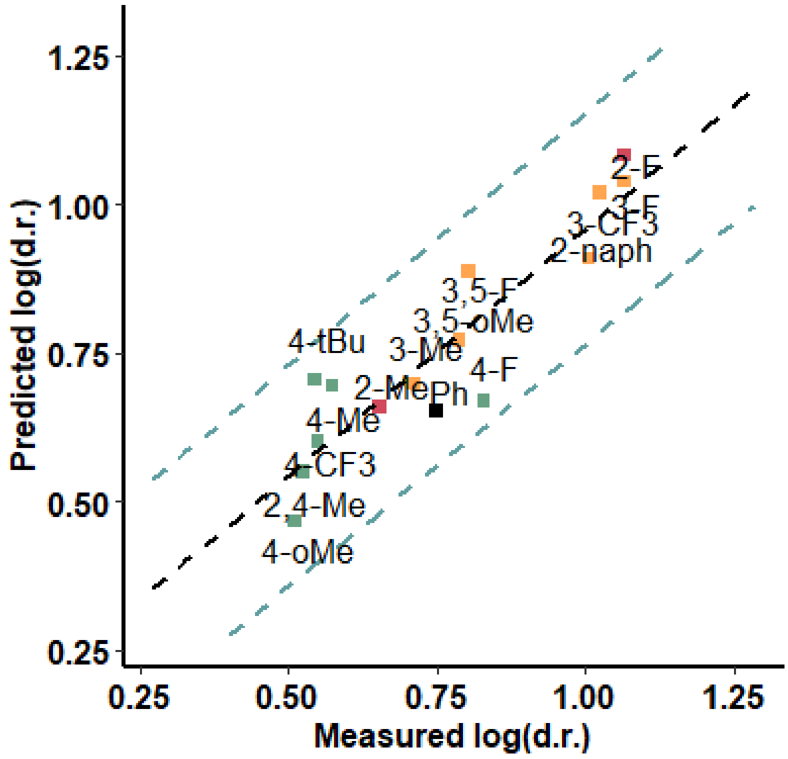

BA + Aldehyde (pi interaction) - oxygen side

formula	R.sq	Q.sq	MAE
output ~ X.2.9. + diff.O1.B2 + L	0.7458243	0.6309926	0.1066979
output ~ X.1.2. + Dist.18..20. + L	0.7355452	0.5825422	0.1140823

	Estimate	Std. Error	t value	Pr(>|t|)
(Intercept)	0.7602560	0.0299454	25.388093	0.0000000
X.2.9.	-0.0948999	0.0328423	-2.889565	0.0147173
diff.O1.B2	-0.1591638	0.0328524	-4.844811	0.0005150
L	-0.0958739	0.0311430	-3.078506	0.0104993

3 & 5 fold CV

Q2	MAE
0.5118772	0.1429641

Q2	MAE

0.5686433	0.1189537

Top Ranked Full Model

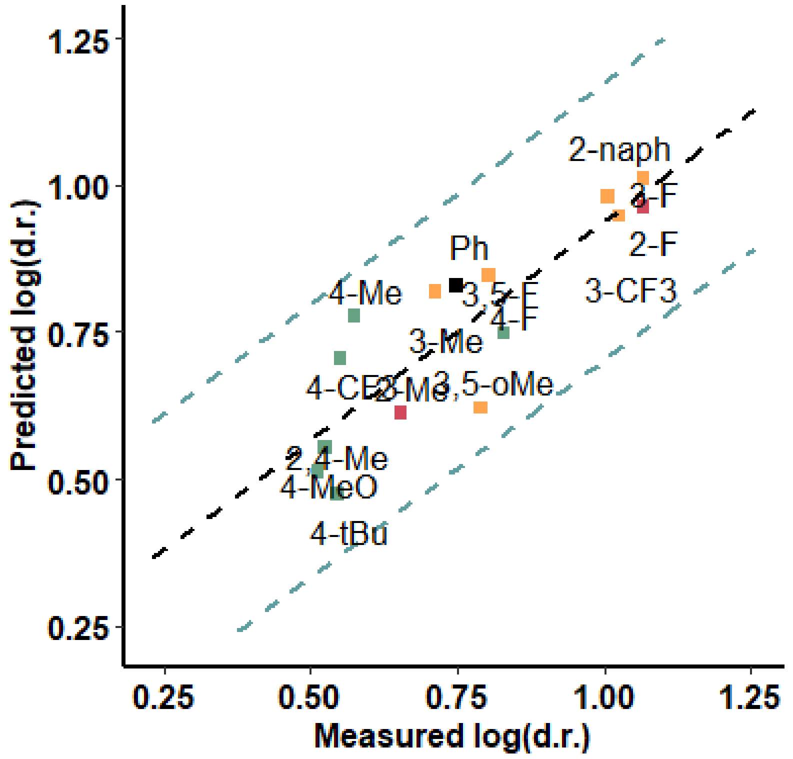

BA + Aldehyde - pi interaction - hydrogen side

formula	R.sq	Q.sq	MAE
output ~ Dist.10..17. + Total + NBO.B.2	0.9183105	0.8638877	0.0618977
output ~ Total + diff.B2.09 + diff.H10.017	0.8805636	0.8076407	0.0715706

	Estimate	Std. Error	t value	Pr(>|t|)
(Intercept)	0.7602560	0.0169764	44.783077	0.00e+00
Dist.10..17.	-0.2547901	0.0235603	-10.814390	3.00e-07
Total	-0.1856470	0.0232170	-7.996163	6.60e-06
NBO.B.2	-0.1294256	0.0195475	-6.621078	3.76e-05

3 & 5 fold CV

Q2	MAE
0.8197333	0.0739802

Q2	MAE
0.8477884	0.0666105

Top Ranked Full Model

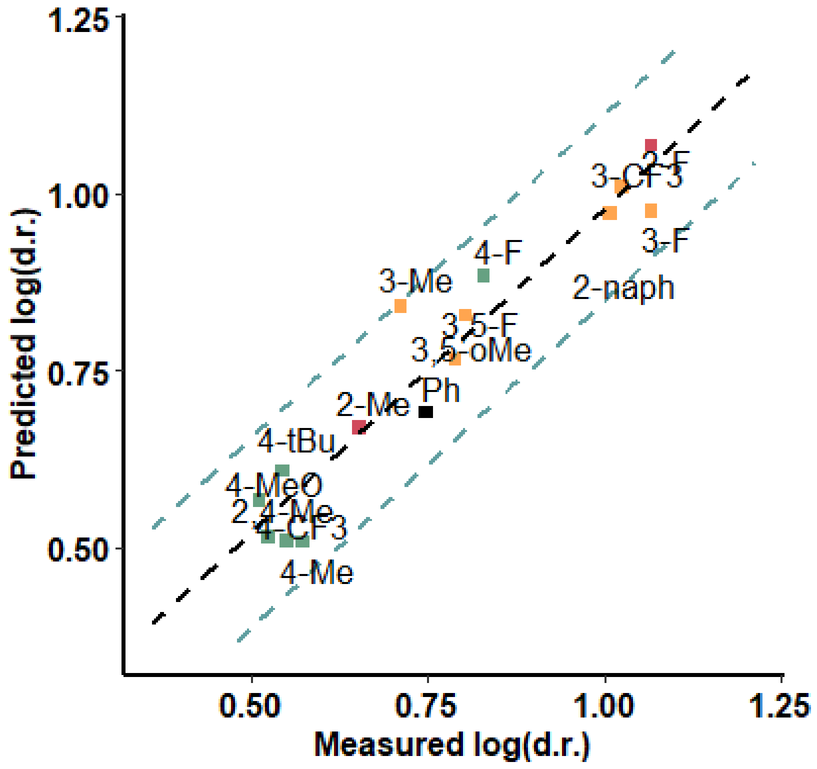

BA + Aldehyde - H bond - aldehyde side

formula	R.sq	Q.sq	MAE
output ~ para + dip_z + NBO.C.20	0.8322566	0.7040580	0.0891631
output ~ para + dip_z + diff.C18.C20	0.8215268	0.7007097	0.0928397

	Estimate	Std. Error	t value	Pr(>|t|)
(Intercept)	0.7602560	0.0243268	31.251759	0.0000000
para	0.1248137	0.0267514	4.665691	0.0006873
dip_z	-0.1035397	0.0260775	-3.970461	0.0021944
NBO.C.20	-0.1012133	0.0268804	-3.765316	0.0031257

3 & 5 fold CV

Q2	MAE
0.6415591	0.1071551

Q2	MAE
0.6689537	0.0981635

Top Ranked Full Model

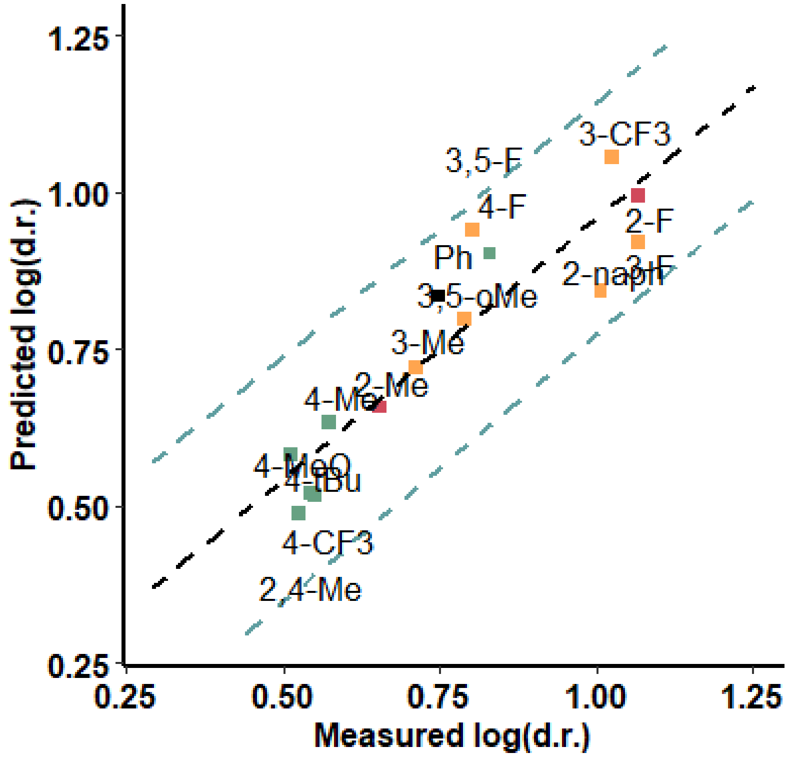

BA + Aldehyde - H bond - opposite to aldehyde

formula	R.sq	Q.sq	MAE
`output ~ Dist.2..9. + dip_z + B1`	0.7049477	0.5485687	0.1166106
`output ~ X.1.2. + NBO.H.10 + B5`	0.6769584	0.5081417	0.1209404

	Estimate	Std. Error	t value	Pr(>|t|)
(Intercept)	0.7602560	0.0322636	23.563924	0.0000000
Dist.2..9.	-0.1555179	0.0352004	-4.418073	0.0010318
dip_z	-0.0911710	0.0354767	-2.569885	0.0260557
B1	-0.0824066	0.0336808	-2.446692	0.0324356

3 & 5 fold CV

Q2	MAE
0.4693024	0.1518696

Q2	MAE
0.517652	0.124469

Top Ranked Full Model

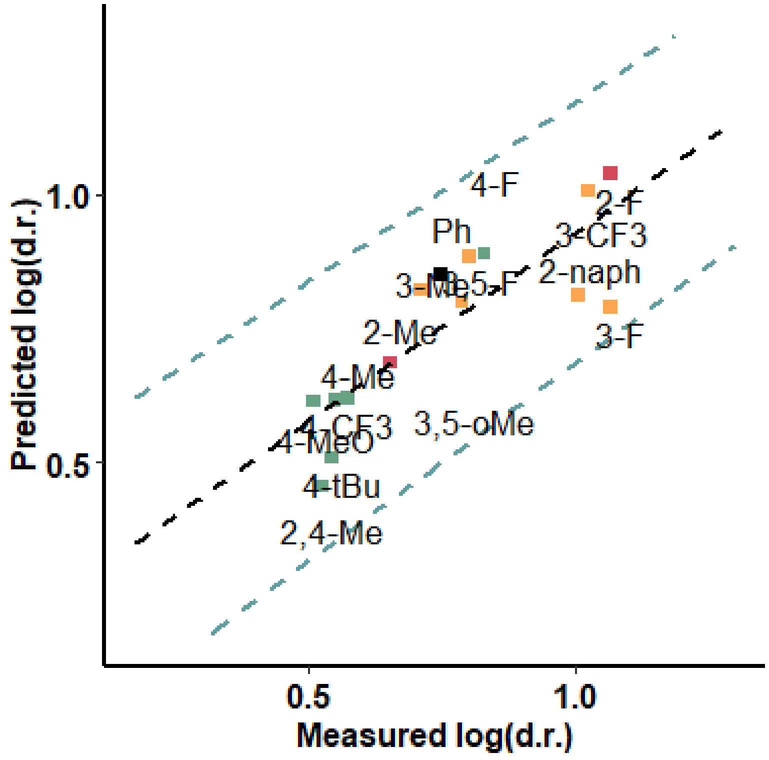

Enamine - far from the active site

formula	R.sq	Q.sq	MAE
output ~ para + dip_y + diff.C16.O36	0.7911133	0.6842042	0.0923034
output ~ para + Dist.1..2. + dip_y	0.7939364	0.6829495	0.0879622

	Estimate	Std. Error	t value	Pr(>|t|)
(Intercept)	0.7602560	0.0271468	28.005396	0.0000000
para	0.1324083	0.0290044	4.565117	0.0008098
dip_y	0.0809572	0.0284875	2.841850	0.0160287
diff.C16.O36	-0.0802905	0.0288250	-2.785447	0.0177303

3 & 5 fold CV

Q2	MAE
0.5728479	0.1203466

Q2	MAE
0.6284218	0.1048313

Top Ranked Full Model

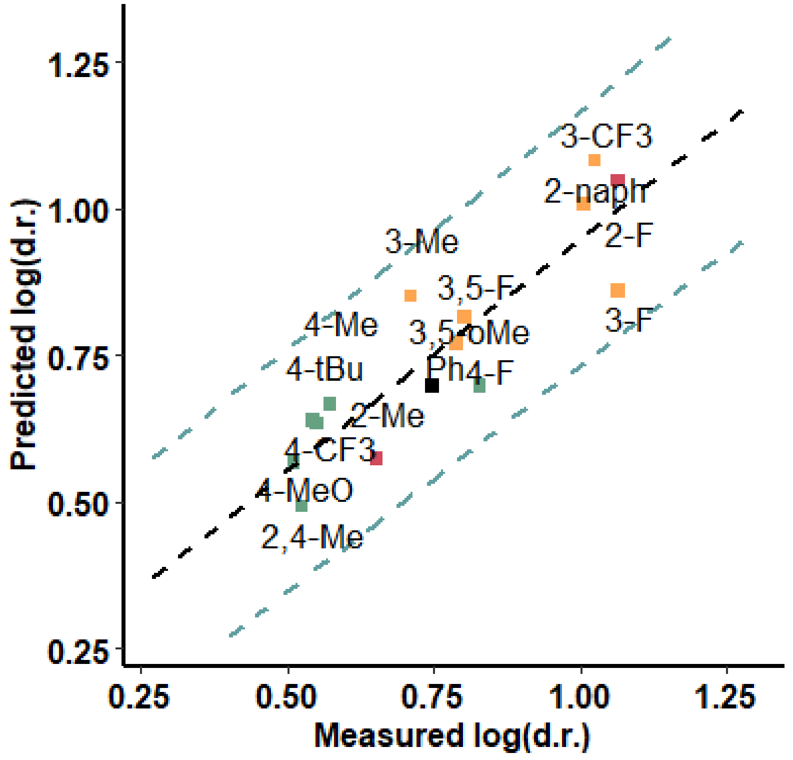

Enamine - close to the active site

formula	R.sq	Q.sq	MAE
`output ~ X.9.16. + Total + diff.O1.B2`	0.7664625	0.5255704	0.1235699
`output ~ X.16.36. + Total + diff.O1.B2`	0.7664625	0.5255704	0.1235699

	Estimate	Std. Error	t value	Pr(>|t|)
(Intercept)	0.7602560	0.0287039	26.486148	0.0000000
X.9.16.	0.2338055	0.0436509	5.356263	0.0002316
Total	-0.1881195	0.0436050	-4.314174	0.0012265
diff.O1.B2	0.0928054	0.0318789	2.911187	0.0141589

3 & 5 fold CV

Q2	MAE
0.4362723	0.1393731

Q2	MAE
0.4894538	0.1278434

Top Ranked Full Model

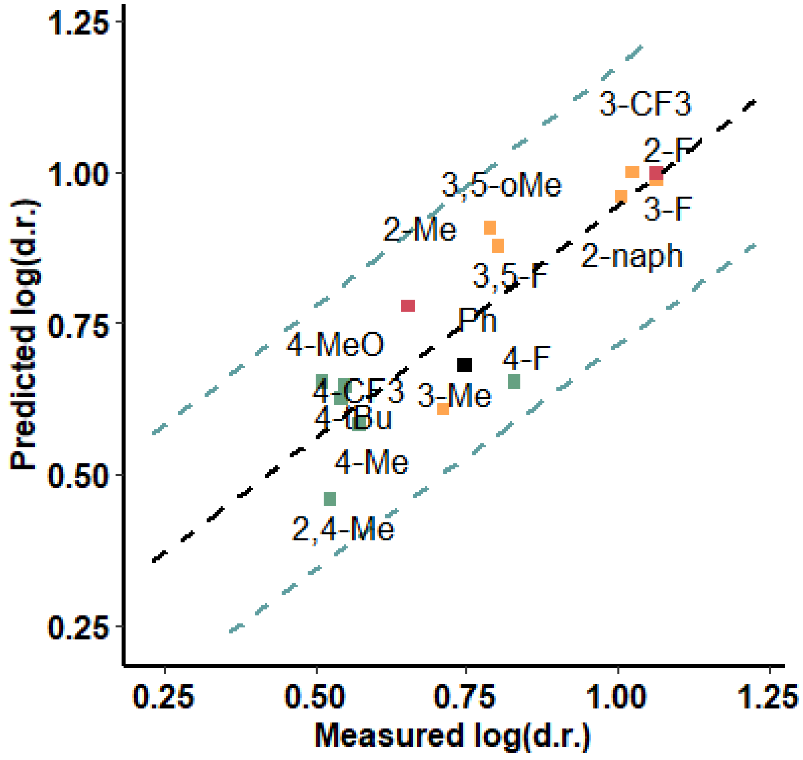

ACN

Only Boronic Acids

formula	R.sq	Q.sq	MAE
output ~ X.2.3. + Dist.2..9. + dip_y	0.8935605	0.8215973	0.0414026
output ~ X.2.3. + X.9.16. + Dist.2..9.	0.8792770	0.8187999	0.0414746

	Estimate	Std. Error	t value	Pr(>|t|)
(Intercept)	0.9445187	0.0132770	71.139424	0.0000000
X.2.3.	-0.0354619	0.0153233	-2.314238	0.0409917
Dist.2..9.	-0.1235038	0.0162124	-7.617872	0.0000104
dip_y	0.0307249	0.0153868	1.996832	0.0711893

3 & 5 fold CV

Q2	MAE

0.7646598	0.0573718

Q2	MAE
0.7911873	0.0498054

Top Ranked Full Model

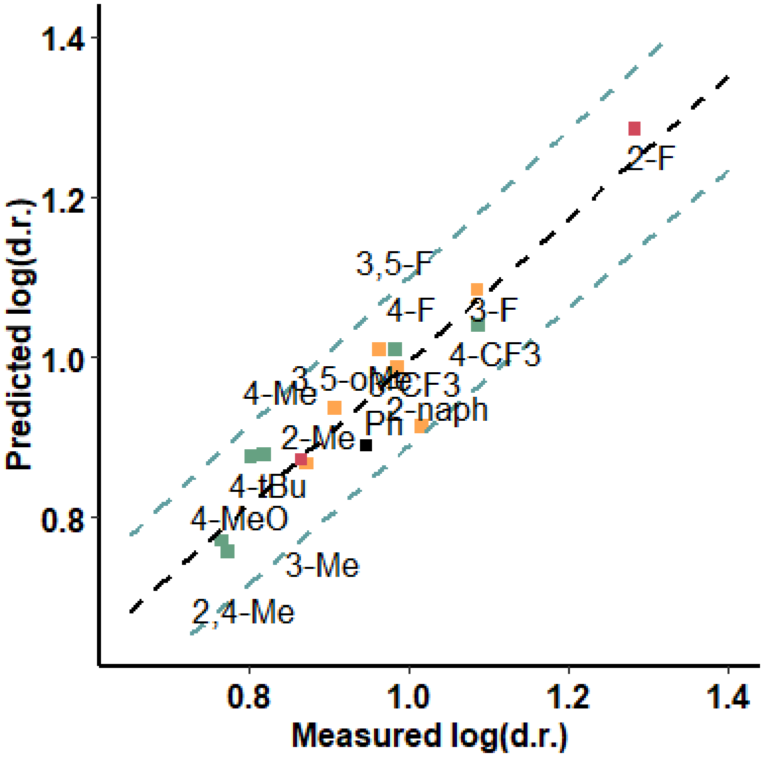

With Catalyst - Close to the active site

formula	R.sq	Q.sq	MAE
output ~ X.16.17. + Dist.1..8. + diff.O16.C17	0.8600127	0.7931926	0.0545321
output ~ X.8.14. + Dist.14..18. + B1	0.8553472	0.7896601	0.0506718

	Estimate	Std. Error	t value	Pr(>|t|)
(Intercept)	0.9445187	0.0152263	62.032233	0.0000000
X.16.17.	-0.1227704	0.0168988	-7.265025	0.0000161
Dist.1..8.	-0.0877933	0.0162548	-5.401069	0.0002163
diff.O16.C17	0.0442257	0.0164047	2.695913	0.0208081

3 & 5 fold CV

Q2	MAE
0.4477399	0.2169794

Q2	MAE
0.512109	0.137276

Top Ranked Full Model

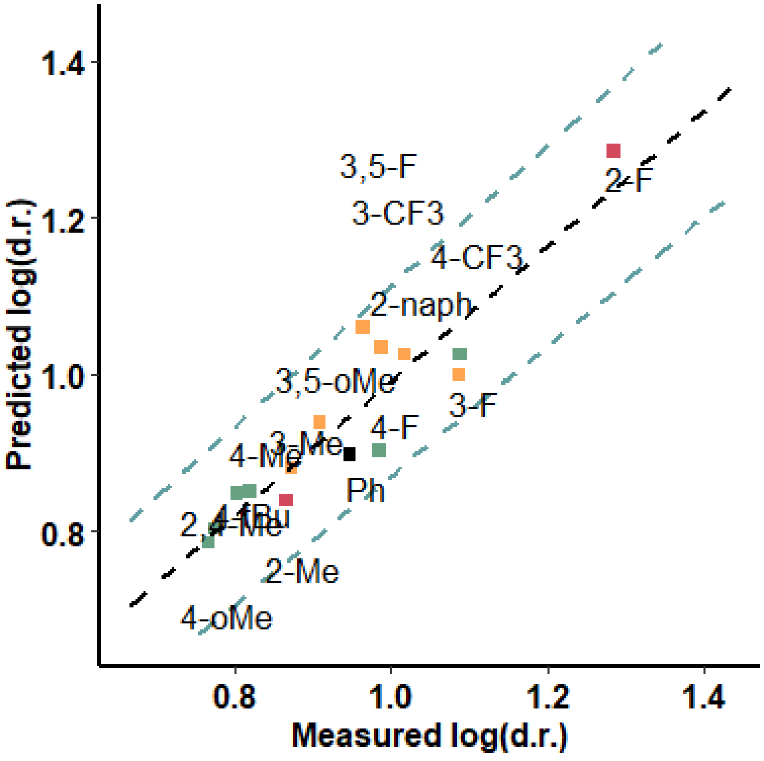

With Catalyst - far from the active site

formula	R.sq	Q.sq	MAE
output ~ `Dist.19..20.` + `diff.C17.O18` + `B5`	0.9153409	0.8689910	0.0425448
output ~ `Dist.16..17.` + `Dist.19..20.` + `diff.N19.H20`	0.9099579	0.8624879	0.0461414

	Estimate	Std. Error	t value	Pr(>|t|)
(Intercept)	0.9445187	0.0118409	79.767307	0.0000000
Dist.19..20.	0.1194359	0.0186648	6.399000	0.0000509
diff.C17.O18	-0.1903134	0.0177404	-10.727699	0.0000004
B5	-0.0332385	0.0134654	-2.468432	0.0312082

3 & 5 fold CV

Q2	MAE
0.8304527	0.059961

Q2	MAE
0.8541898	0.0461192

Top Ranked Full Model

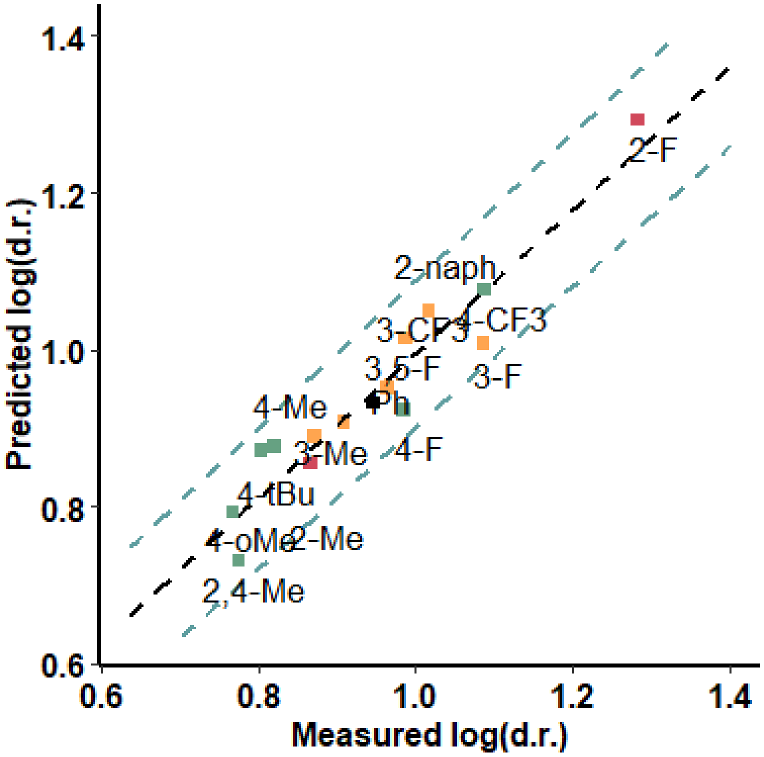

BA + Pentanone

formula	R.sq	Q.sq	MAE
output ~ Dist.1..2. + dip_x + diff.B2.09	0.8758965	0.8147647	0.0449457
output ~ dip_x + Total + NBO.0.1	0.8718930	0.8068999	0.0479210

	Estimate	Std. Error	t value	Pr(>|t|)
(Intercept)	0.9445187	0.0143364	65.882450	0.0000000
Dist.1..2.	-0.1630368	0.0215539	-7.564146	0.0000111
dip_x	0.0476885	0.0161251	2.957410	0.0130355

diff.B2.O9	0.0364514	0.0202817	1.797261	0.0997708

3 & 5 fold CV

Q2	MAE
0.6658149	0.0698803

Q2	MAE
0.744246	0.057417

Top Ranked Full Model

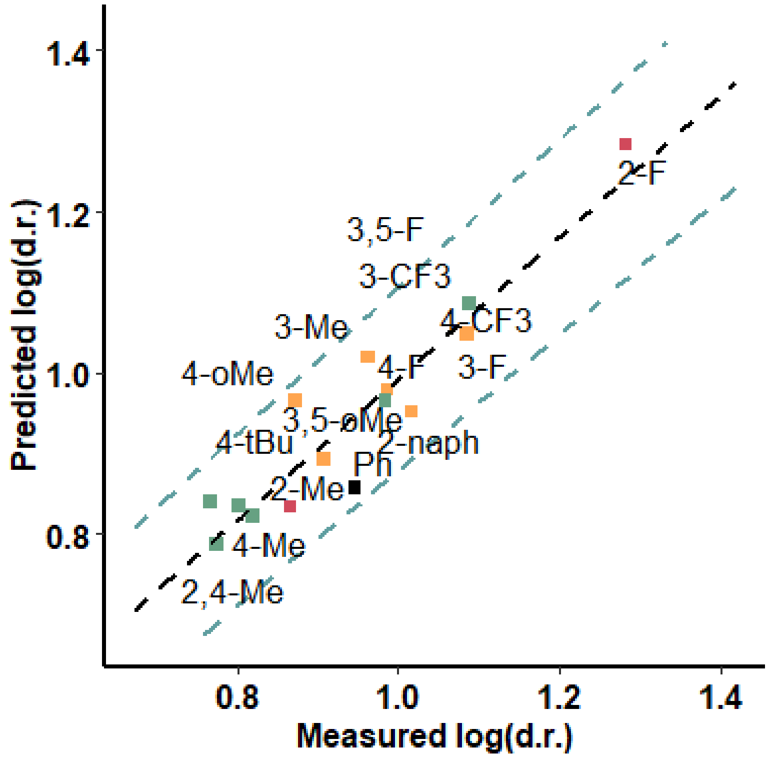

BA + Aldehyde (pi interaction) - oxygen side

formula	R.sq	Q.sq	MAE
output ~ X.2.9. + Dist.2..9. + B5	0.9368757	0.8932947	0.0362956
output ~ Dist.2..9. + dip_y + B1	0.9139520	0.8643921	0.0429315

	Estimate	Std. Error	t value	Pr(>|t|)
(Intercept)	0.9445187	0.0102246	92.376896	0.0000000
X.2.9.	-0.0485831	0.0112237	-4.328628	0.0011973
Dist.2..9.	-0.1253365	0.0108604	-11.540648	0.0000002

B5	-0.0345553	0.0113678	-3.039763	0.0112513

3 & 5 fold CV

Q2	MAE
0.8696749	0.0448942

Q2	MAE
0.8828007	0.0394001

Top Ranked Full Model

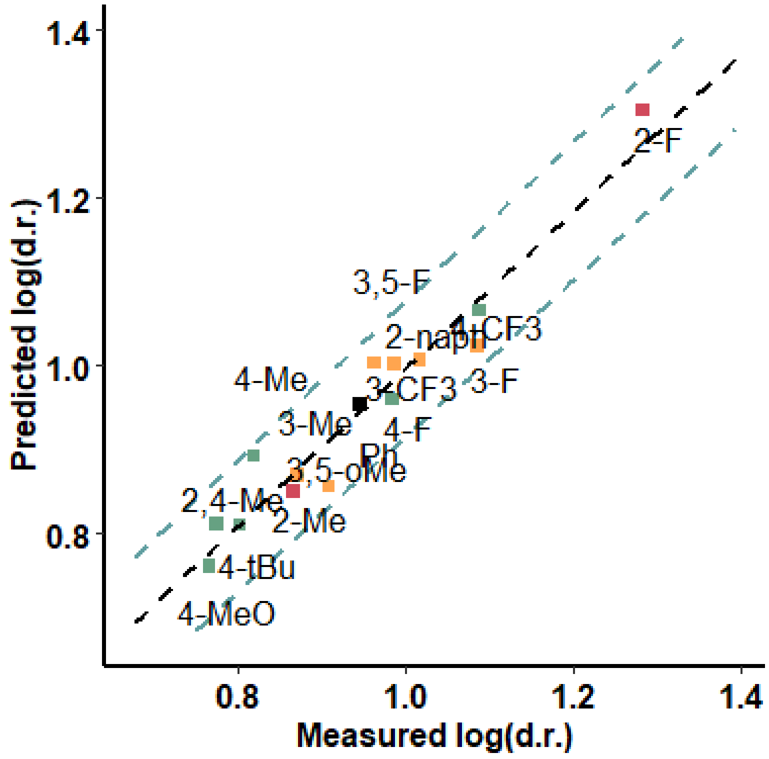

BA + Aldehyde (pi interaction) - hydrogen side

formula	R.sq	Q.sq	MAE
output ~ `NBO.H.16` + `diff.O1.H10` + `diff.H10.O17`	0.9561832	0.9243700	0.0315412
output ~ `NBO.O.1` + `NBO.H.16` + `diff.H10.O17`	0.9497616	0.9119918	0.0324338

	Estimate	Std. Error	t value	Pr(>|t|)
(Intercept)	0.9445187	0.0085186	110.876952	0.0000000

NBO.H.16	-0.0657890	0.0170136	-3.866840	0.0026222
diff.O1.H10	-0.1443473	0.0109816	-13.144510	0.0000000
diff.H10.O17	0.1730257	0.0183219	9.443641	0.0000013

3 & 5 fold CV

Q2	MAE
0.8977793	0.0411254

Q2	MAE
0.9114328	0.0339986

Top Ranked Full Model

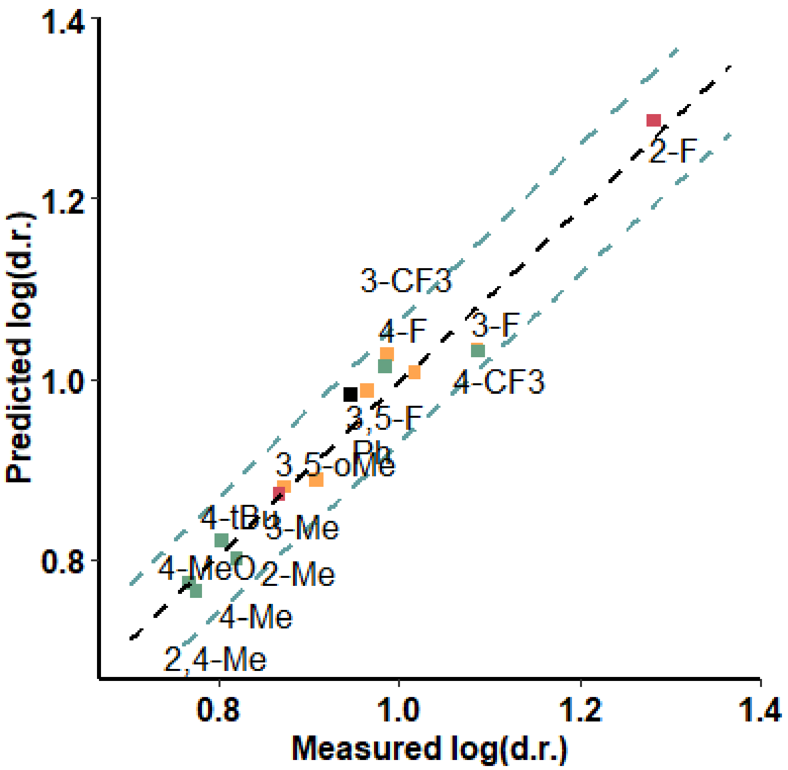

BA + Aldehyde - H bond - aldehyde side

formula	R.sq	Q.sq	MAE
output ~ NBO.O.9 + diff.H10.O17 + diff.C18.C20	0.8246279	0.6988750	0.0592294
output ~ Dist.9..16. + Total + NBO.O.9	0.8130284	0.6928567	0.0661306

	Estimate	Std. Error	t value	Pr(>|t|)

(Intercept)	0.9445187	0.0170423	55.421918	0.0000000
NBO.O.9	0.1657842	0.0364231	4.551622	0.0008279
diff.H10.O17	-0.2500693	0.0510407	-4.899414	0.0004721
diff.C18.C20	0.2868322	0.0432788	6.627546	0.0000372

3 & 5 fold CV

Q2	MAE
0.6173183	0.077972

Q2	MAE
0.6542058	0.0676776

Top Ranked Full Model

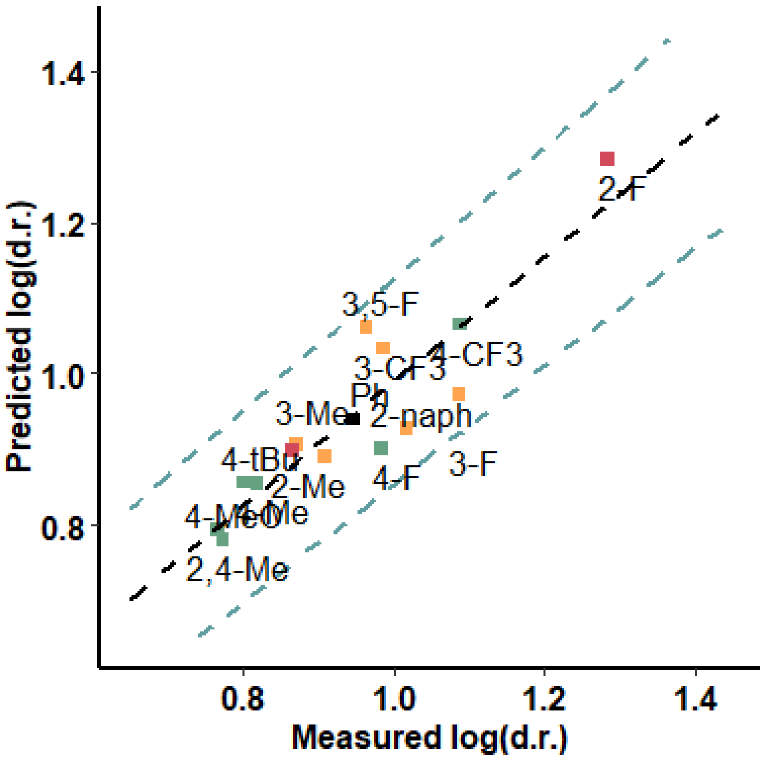

BA + Aldehyde - H bond - opposite to aldehyde

formula	R.sq	Q.sq	MAE
`output ~ NBO.C.18 + diff.O1.H10 + diff.H16.O17`	0.8573931	0.6414726	0.0681631

	Estimate	Std. Error	t value	Pr(>|t|)

(Intercept)	0.9445187	0.0153681	61.459842	0.0000000
NBO.C.18	0.5466142	0.0905860	6.034203	0.0000850
diff.O1.H10	-0.1228705	0.0363256	-3.382479	0.0061157
diff.H16.O17	-0.4613203	0.0859761	-5.365679	0.0002283

3 & 5 fold CV

Q2	MAE
0.6076123	0.0900551

Q2	MAE
0.6257414	0.0760585

Top Ranked Full Model

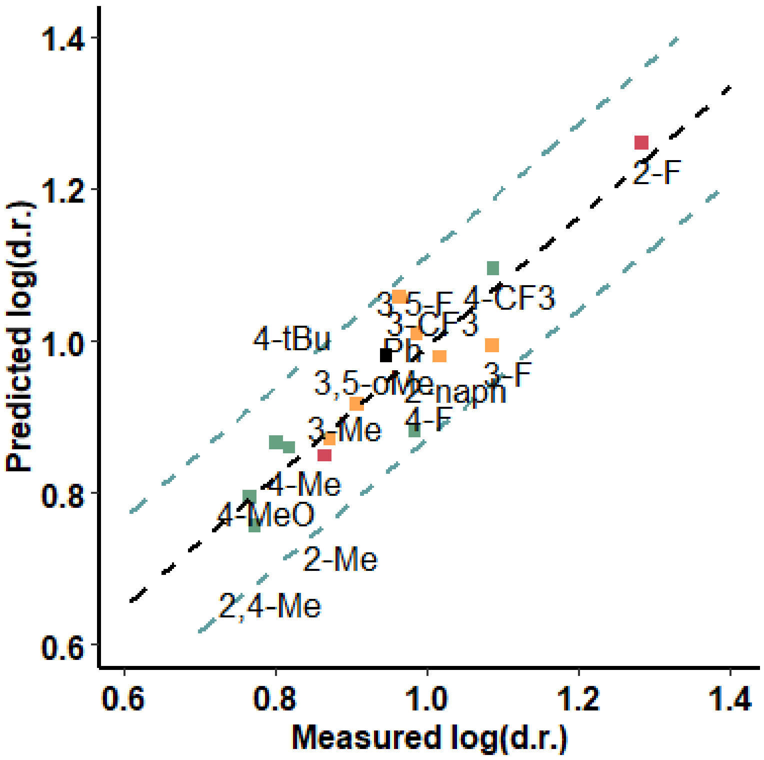

Enamine - far from the active site

formula	R.sq	Q.sq	MAE
output ~ para + `X.2.3.` + `Dist.1..2.`	0.8489350	0.7548529	0.0519191
output ~ `X.1.2.` + `Dist.1..2.` + `B1`	0.8067027	0.7277601	0.0562147

	Estimate	Std. Error	t value	Pr(>|t|)

(Intercept)	0.9445187	0.0158172	59.714511	0.0000000
para	0.0388101	0.0192972	2.011175	0.0694604
X.2.3.	0.0342529	0.0189875	1.803966	0.0986607
Dist.1..2.	-0.1194747	0.0167955	-7.113504	0.0000196

3 & 5 fold CV

Q2	MAE
0.6380305	0.0704364

Q2	MAE
0.7019644	0.0604399

Top Ranked Full Model

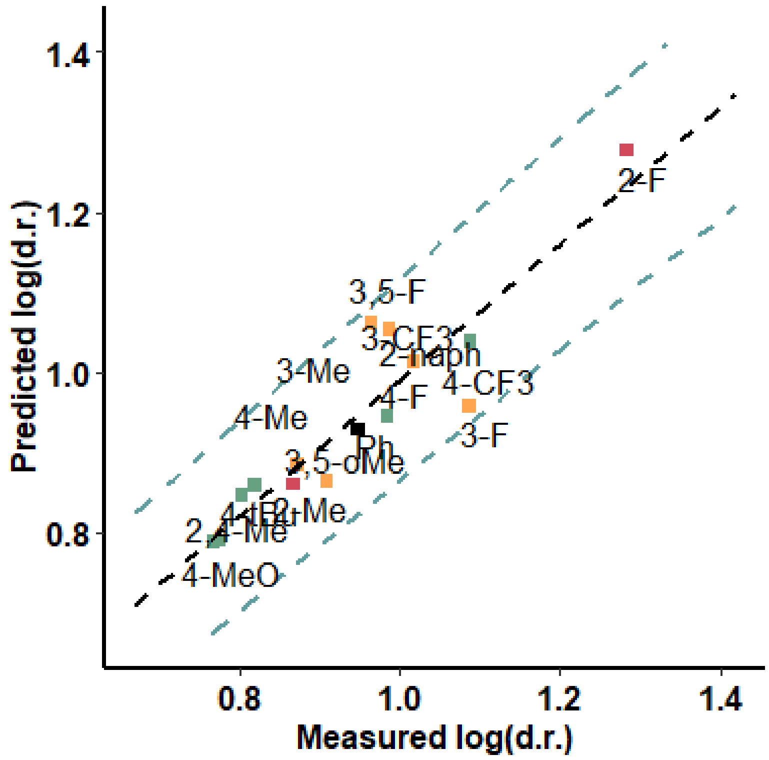

Enamine - close to the active site

formula	R.sq	Q.sq	MAE
output ~ `X.2.3. + Dist.1..10. + diff.O1.H10`	0.9368997	0.8842193	0.0410499
output ~ `X.2.3. + NBO.H.10 + diff.C16.O36`	0.9347878	0.8555366	0.0460048

	Estimate	Std. Error	t value	Pr(>|t|)
(Intercept)	0.9445187	0.0102227	92.394483	0.0000000
X.2.3.	0.0720869	0.0135318	5.327223	0.0002421
Dist.1..10.	0.1334003	0.0110452	12.077639	0.0000001
diff.O1.H10	0.0675968	0.0130683	5.172590	0.0003073

3 & 5 fold CV

Q2	MAE
0.8312262	0.0517943

Q2	MAE
0.8564135	0.0462081

Top Ranked Full Model

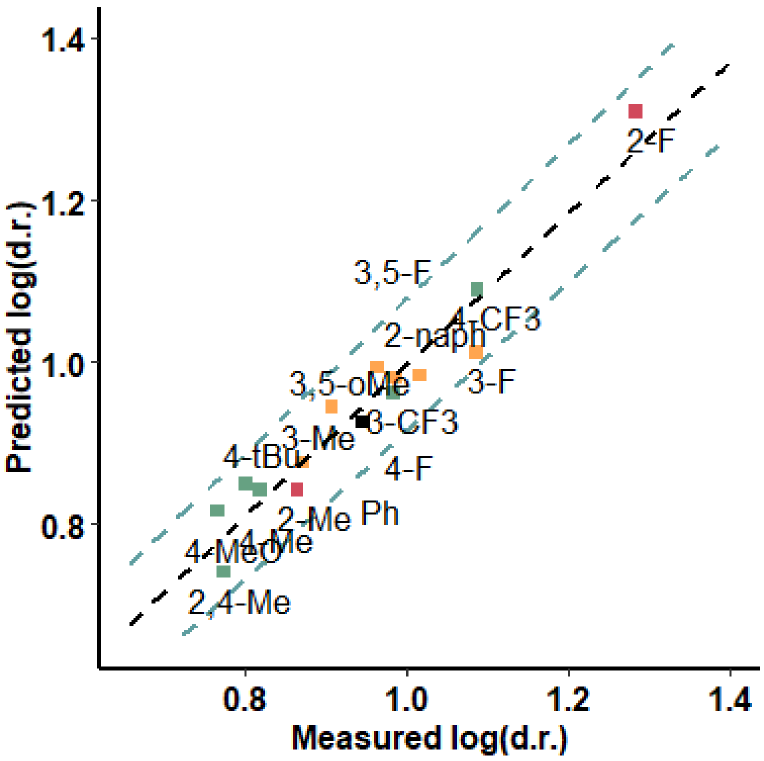

Methanol

Only Boronic Acids

formula	R.sq	Q.sq	MAE
output ~ cross + para + Total	0.6922134	0.3986021	0.0934931

output ~ X.9.16. + Dist.1..10. + L	0.6915081	0.3948789	0.1058145

	Estimate	Std. Error	t value	Pr(>|t|)
(Intercept)	0.7096271	0.0250421	28.337346	0.0000000
cross	0.1866896	0.0533714	3.497934	0.0049884
para	0.2248532	0.0490043	4.588434	0.0007795
Total	-0.1476787	0.0371808	-3.971914	0.0021889

3 & 5 fold CV

Q2	MAE
0.3405423	0.1101085

Q2	MAE
0.3618142	0.1010866

Top Ranked Full Model

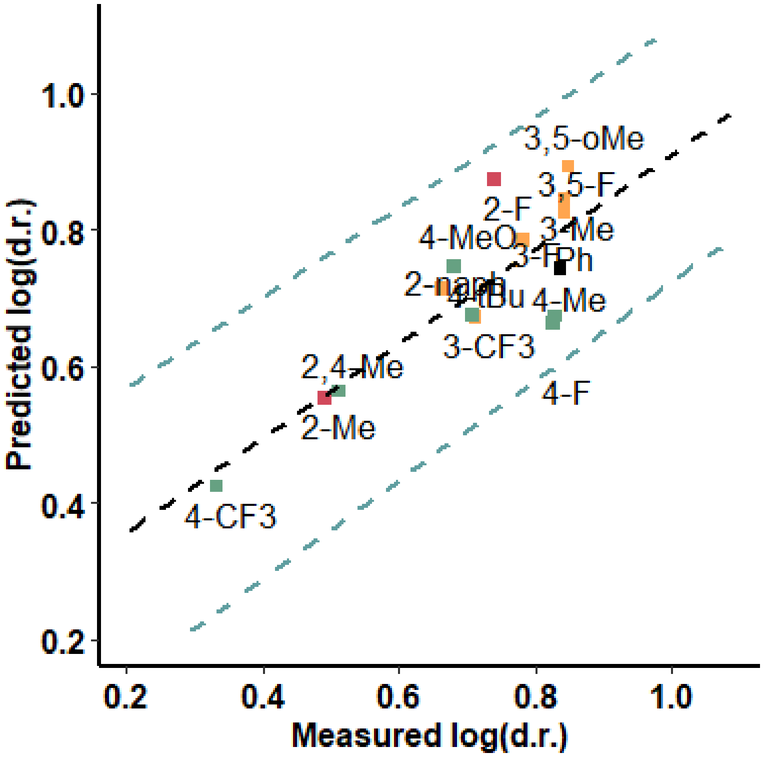

With Catalyst - Close to the active site

formula	R.sq	Q.sq	MAE
output ~ Dist.1..2. + NBO.B.1 + L	0.7550323	0.5366056	0.0850073

output ~ Dist.14..18. + dip_y + diff.O8.H14	0.7524695	0.4247401	0.0773804

	Estimate	Std. Error	t value	Pr(>|t|)
(Intercept)	0.7096271	0.0223409	31.763592	0.0000000
Dist.1..2.	-0.1322018	0.0284233	-4.651181	0.0007037
NBO.B.1	0.0781880	0.0242608	3.222809	0.0081184
L	-0.1467242	0.0295578	-4.963978	0.0004261

3 & 5 fold CV

Q2	MAE
0.4489777	0.1008164

Q2	MAE
0.496588	0.0914699

Top Ranked Full Model

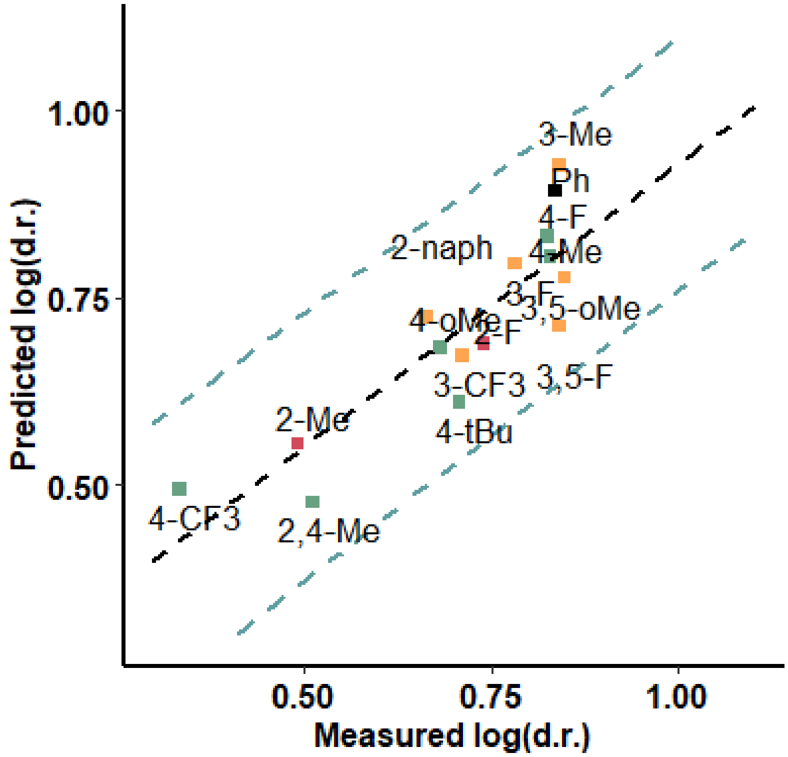

With Catalyst - far from the active site

formula	R.sq	Q.sq	MAE

`output ~ X.1.8. + dip_y + diff.O16.C17`	0.8272496	0.6117842	0.0801216
`output ~ X.1.8. + dip_y + NBO.O.18`	0.7782930	0.5571560	0.0884945

	Estimate	Std. Error	t value	Pr(>|t|)
(Intercept)	0.7096271	0.0187610	37.824608	0.0000000
X.1.8.	0.1047157	0.0200638	5.219139	0.0002859
dip_y	0.0981090	0.0197640	4.964023	0.0004261
diff.O16.C17	-0.0601550	0.0199994	-3.007843	0.0119117

3 & 5 fold CV

Q2	MAE
0.5515629	0.0894694

Q2	MAE
0.589942	0.0834045

Top Ranked Full Model

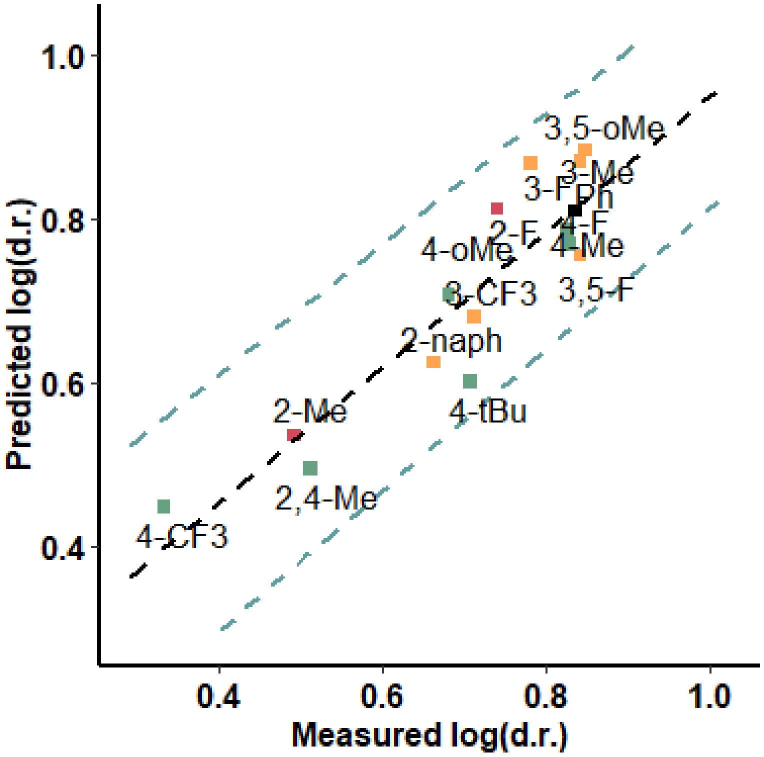

BA + Pentanone

formula	R.sq	Q.sq	MAE

output ~ Dist.2..3. + dip_y + L	0.7514926	0.5386066	0.0898684

	Estimate	Std. Error	t value	Pr(>|t|)
(Intercept)	0.7096271	0.0225017	31.536564	0.0000000
Dist.2..3.	-0.1226959	0.0283944	-4.321130	0.0012124
dip_y	0.0678104	0.0240155	2.823604	0.0165606
L	-0.1090695	0.0290876	-3.749693	0.0032116

3 & 5 fold CV

Q2	MAE
0.4783471	0.0988627

Q2	MAE
0.5008795	0.0951922

Top Ranked Full Model

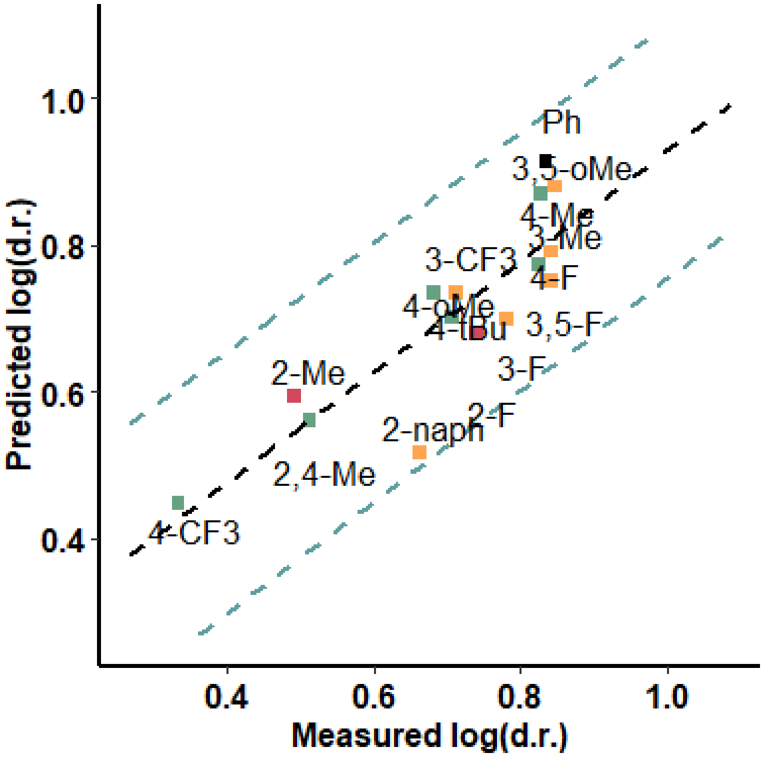

BA + Aldehyde (pi interaction) - oxygen side

formula	R.sq	Q.sq	MAE
output ~ Dist.2..3. + Dist.18..20. + L	0.7984201	0.6034807	0.0730294

output ~ Dist.9..16. + Dist.16..17. + L	0.7578872	0.5670328	0.0782136

	Estimate	Std. Error	t value	Pr(>|t|)
(Intercept)	0.7096271	0.0202661	35.015508	0.0000000
Dist.2..3.	-0.1876356	0.0314038	-5.974924	0.0000925
Dist.18..20.	0.1055824	0.0244039	4.326464	0.0012016
L	-0.1778186	0.0297389	-5.979325	0.0000920

3 & 5 fold CV

Q2	MAE
0.5397727	0.0856526

Q2	MAE
0.5732987	0.0778355

Top Ranked Full Model

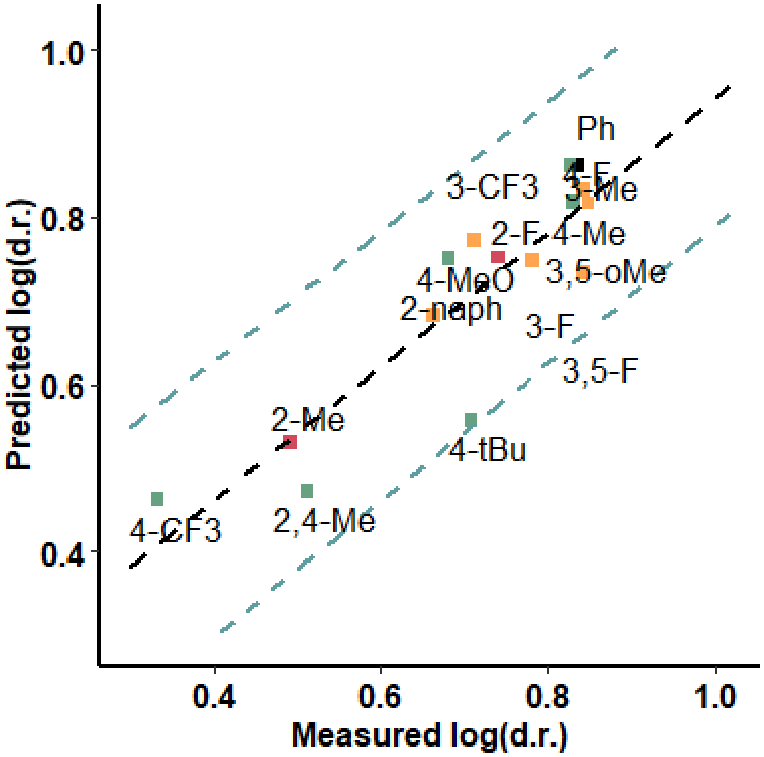

BA + Aldehyde (pi interaction) - hydrogen side

formula	R.sq	Q.sq	MAE
output ~ X.18.20. + NBO.H.10 + L	0.8016758	0.6515848	0.0684132

output ~ X.18.20. + Total + L	0.7784225	0.6172180	0.0769768

	Estimate	Std. Error	t value	Pr(>|t|)
(Intercept)	0.7096271	0.0201018	35.301747	0.0000000
X.18.20.	0.1214244	0.0216036	5.620573	0.0001555
NBO.H.10	-0.0637230	0.0220061	-2.895699	0.0145567
L	-0.1020940	0.0227999	-4.477832	0.0009347

3 & 5 fold CV

Q2	MAE
0.5830002	0.0884932

Q2	MAE
0.6133242	0.0741368

Top Ranked Full Model

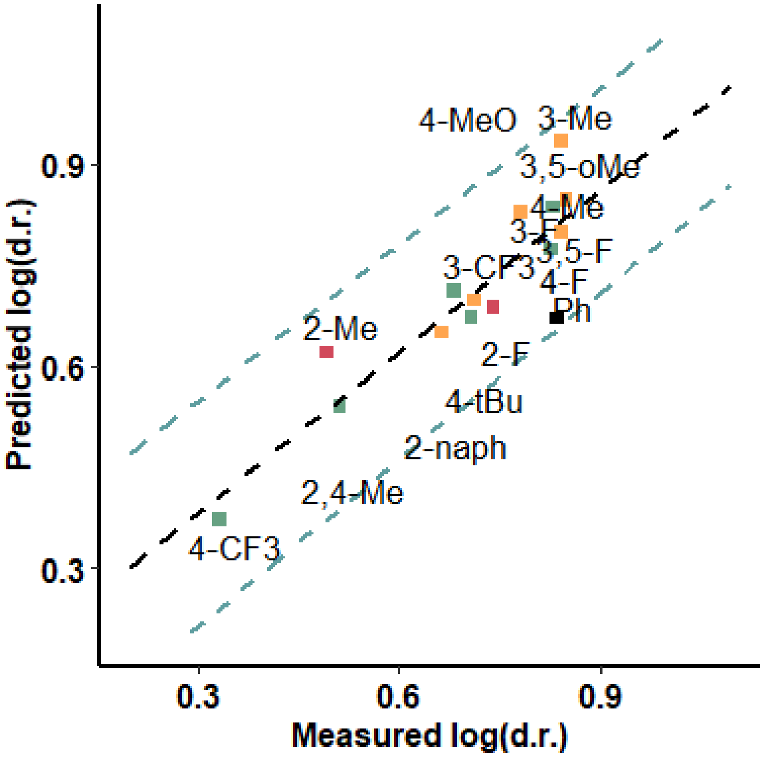

BA + Aldehyde - H bond - aldehyde side

formula	R.sq	Q.sq	MAE
output ~ Dist.18..20. + NBO.C.18 + L	0.7703468	0.5368018	0.0852736

output ~ X.2.9. + Dist.2..3. + L	0.7577096	0.5277069	0.0808464

	Estimate	Std. Error	t value	Pr(>|t|)
(Intercept)	0.7096271	0.0216313	32.805582	0.0000000
Dist.18..20.	-0.4975007	0.0990250	-5.023988	0.0003876
NBO.C.18	-0.5707275	0.1037945	-5.498630	0.0001866
L	-0.1501622	0.0286822	-5.235379	0.0002788

3 & 5 fold CV

Q2	MAE
0.4800409	0.0963722

Q2	MAE
0.5073712	0.090616

Top Ranked Full Model

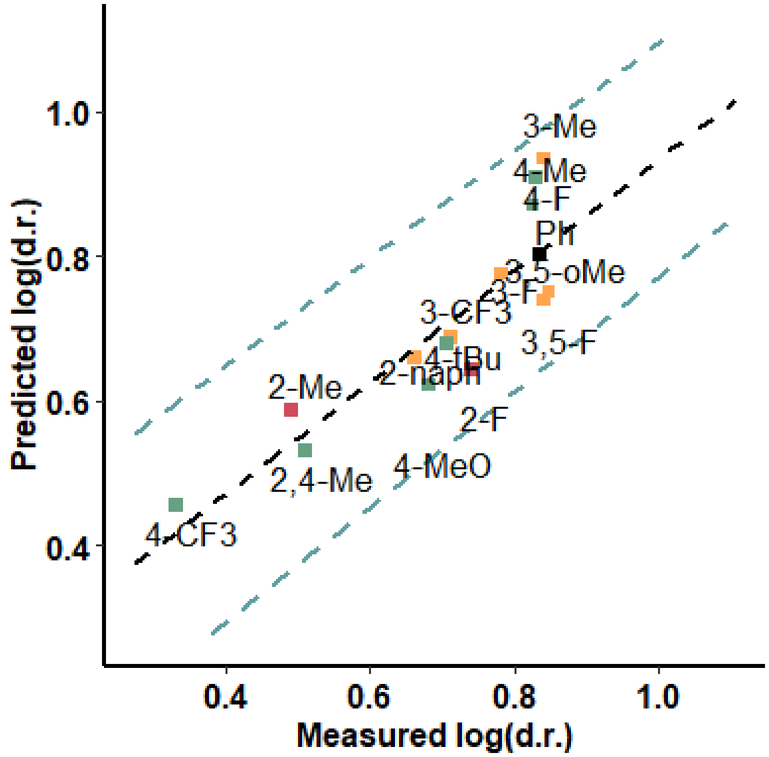

BA + Aldehyde - H bond - opposite to aldehyde

formula	R.sq	Q.sq	MAE
output ~ Dist.9..16. + diff.O1.B2 + L	0.7631709	0.5907987	0.0856327

output ~ X.18.20. + Dist.1..2. + diff.H16.O17	0.7649252	0.5407058	0.0844245

	Estimate	Std. Error	t value	Pr(>|t|)
(Intercept)	0.7096271	0.0219666	32.304754	0.0000000
Dist.9..16.	-0.2161021	0.0406119	-5.321157	0.0002444
diff.O1.B2	-0.1352282	0.0398357	-3.394651	0.0059854
L	-0.1208896	0.0273489	-4.420264	0.0010281

3 & 5 fold CV

Q2	MAE
0.3815917	0.1155288

Q2	MAE
0.4630401	0.1022524

Top Ranked Full Model

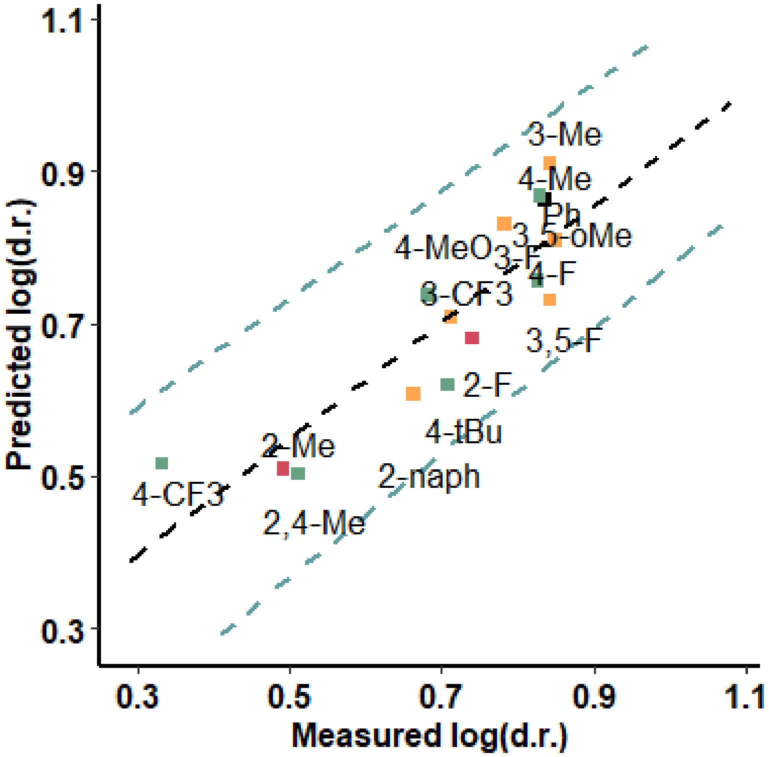

Enamine - far from the active site

formula	R.sq	Q.sq	MAE

output ~ Dist.10..36. + dip_x + L		0.7894706	0.5283876	0.0822182
output ~ X.9.16. + Dist.10..36. + L		0.7836696	0.5004415	0.0834403

	Estimate	Std. Error	t value	Pr(>|t|)
(Intercept)	0.7096271	0.0207111	34.263177	0.0000000
Dist.10..36.	0.1256495	0.0234486	5.358505	0.0002308
dip_x	-0.1169389	0.0251268	-4.653952	0.0007006
L	-0.1045710	0.0239384	-4.368340	0.0011206

3 & 5 fold CV

Q2	MAE
0.4702661	0.1159049

Q2	MAE
0.4887238	0.1022606

Top Ranked Full Model

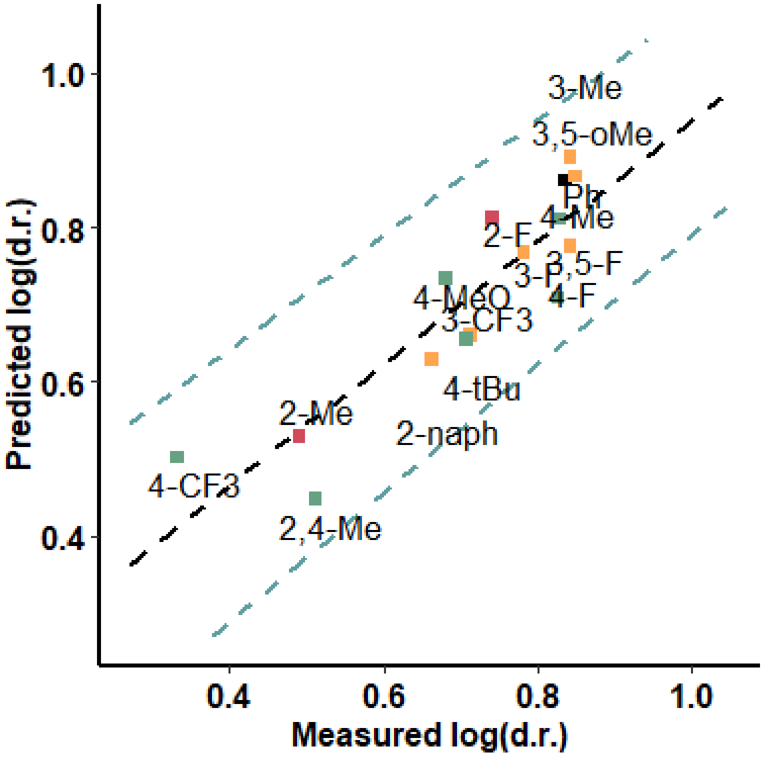

Enamine - close to the active site

formula	R.sq	Q.sq	MAE

output ~ X.2.9. + NBO.O.36 + L	0.7860717	0.6035341	0.0763721
output ~ Dist.2..3. + NBO.B.2 + L	0.7732674	0.5997343	0.0761633

	Estimate	Std. Error	t value	Pr(>|t|)
(Intercept)	0.7096271	0.0208776	33.989898	0.0000000
X.2.9.	0.1073357	0.0234353	4.580095	0.0007902
NBO.O.36	-0.0823806	0.0223876	-3.679742	0.0036275
L	-0.1103054	0.0240688	-4.582928	0.0007866

3 & 5 fold CV

Q2	MAE
0.5257874	0.0880121

Q2	MAE
0.558774	0.0827631

Top Ranked Full Model

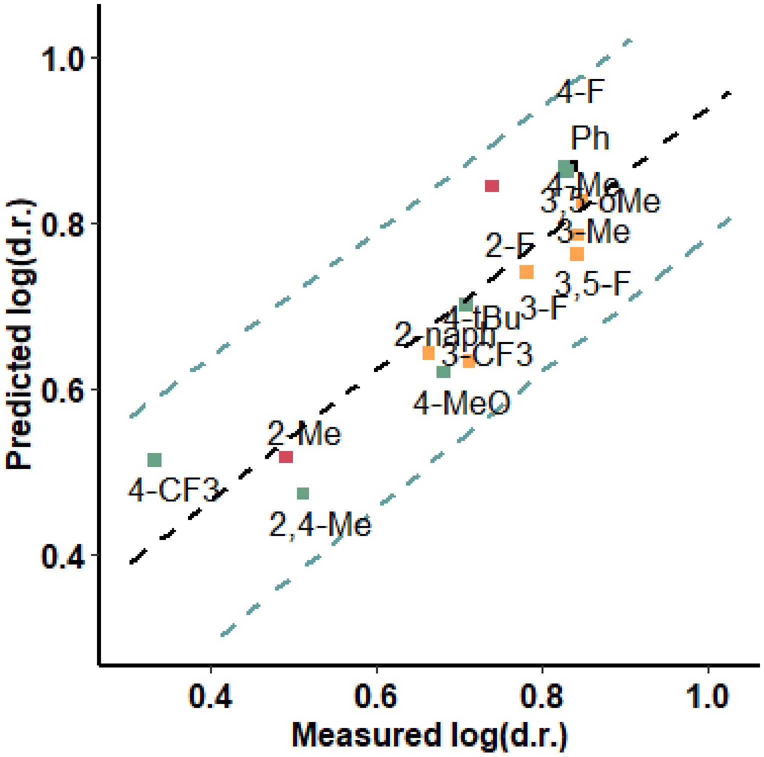

Chloroform

Only Boronic Acids

formula	R.sq	Q.sq	MAE
`output ~ Dist.1..2. + diff.B2.C3 + B5`	0.8470181	0.7188765	0.0873730
`output ~ Dist.1..2. + NBO.C.3 + B5`	0.8464943	0.7184720	0.0891002

	Estimate	Std. Error	t value	Pr(>|t|)
(Intercept)	0.9516937	0.0223869	42.511269	0.0000000
Dist.1..2.	-0.2543859	0.0337924	-7.527901	0.0000116
diff.B2.C3	0.2018311	0.0360556	5.597766	0.0001608
B5	0.1432921	0.0277945	5.155413	0.0003156

3 & 5 fold CV

Q2	MAE
0.5738378	0.1079603

Q2	MAE
0.6397147	0.0970273

Top Ranked Full Model

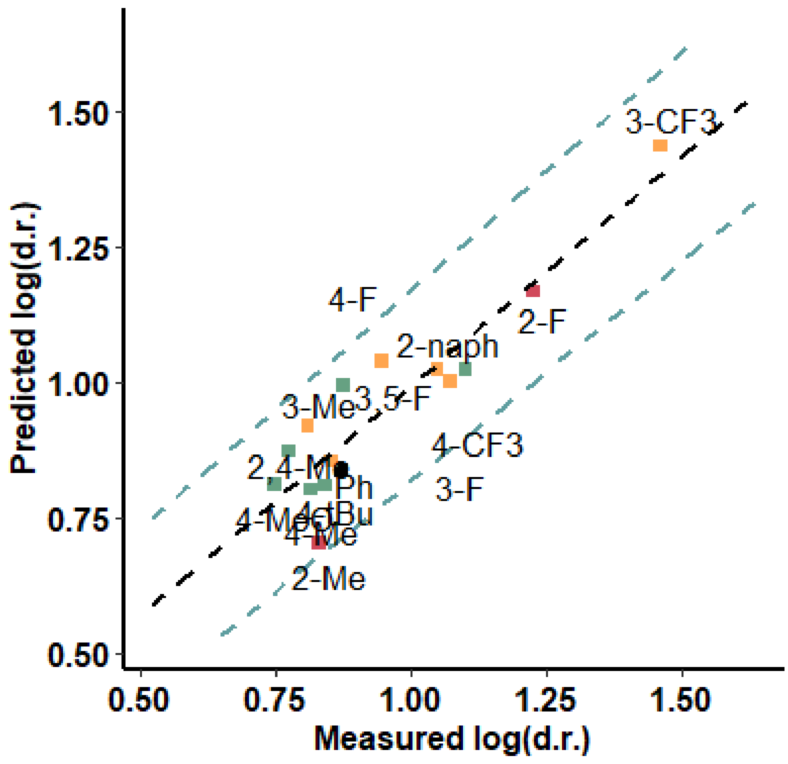

With Catalyst - Close

formula	R.sq	Q.sq	MAE
output ~ Dist.8..14. + dip_z + Total	0.9161020	0.8347970	0.0676771
output ~ Dist.17..18. + NBO.0.18 + diff.N19.H20	0.9184669	0.8230735	0.0673584

	Estimate	Std. Error	t value	Pr(>|t|)
(Intercept)	0.9516937	0.0165786	57.404791	0.0000000
Dist.8..14.	0.1402622	0.0181654	7.721413	0.0000091
dip_z	0.0593602	0.0183181	3.240514	0.0078668
Total	-0.0587418	0.0175020	-3.356288	0.0064059

3 & 5 fold CV

Q2	MAE
0.7466433	0.0813729

Q2	MAE
0.8017824	0.0727287

Top Ranked Full Model

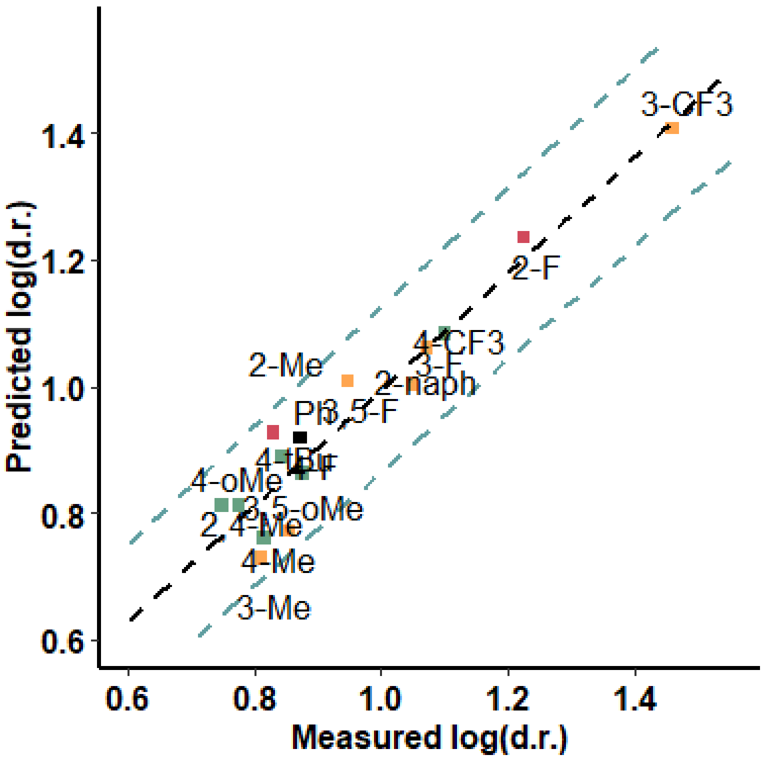

With Catalyst - far

formula	R.sq	Q.sq	MAE
output ~ Total + NBO.O.16 + diff.B1.C2	0.9501665	0.9023519	0.0470429
output ~ Total + NBO.C.2 + NBO.O.16	0.9494356	0.8966295	0.0496260

	Estimate	Std. Error	t value	Pr(>|t|)
(Intercept)	0.9516937	0.0127771	74.484101	0.0000000
Total	0.1691442	0.0140811	12.012146	0.0000001
NBO.O.16	-0.0698816	0.0143236	-4.878771	0.0004878
diff.B1.C2	0.0419038	0.0141754	2.956089	0.0130663

3 & 5 fold CV

Q2	MAE
0.8255142	0.0621238

Q2	MAE
0.8685743	0.0536012

Top Ranked Full Model

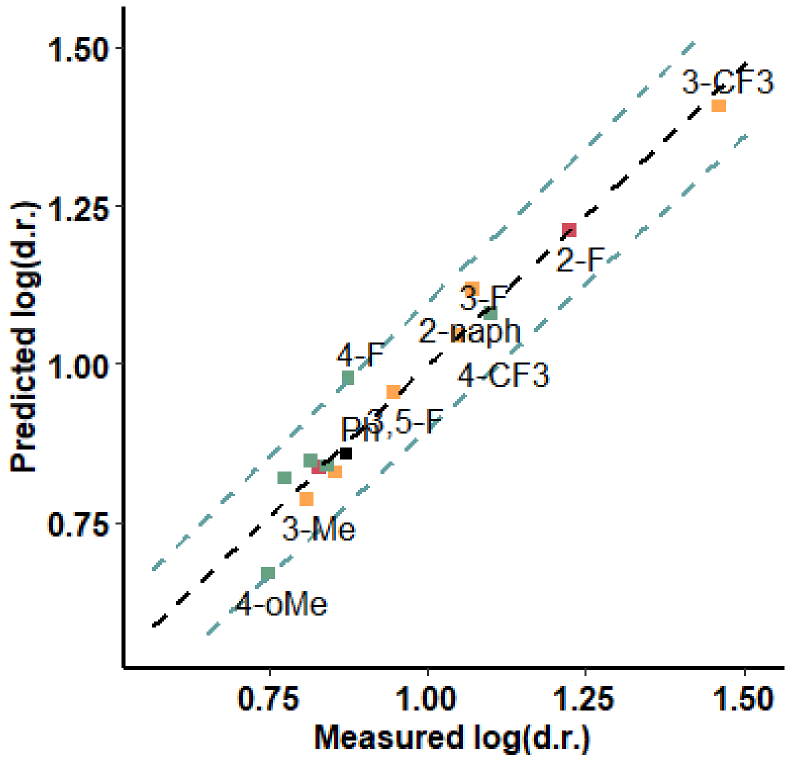

BA + Pentanone

formula	R.sq	Q.sq	MAE
output ~ Dist.2..9. + diff.B2.C3 + B5	0.8810873	0.7689940	0.0780754
output ~ Dist.2..9. + NBO.C.3 + B5	0.8812168	0.7679346	0.0791693

	Estimate	Std. Error	t value	Pr(>|t|)
(Intercept)	0.9516937	0.0197373	48.218083	0.0000000
Dist.2..9.	-0.2704470	0.0309097	-8.749574	0.0000028
diff.B2.C3	0.2206265	0.0331842	6.648551	0.0000362
B5	0.1134724	0.0232557	4.879337	0.0004874

3 & 5 fold CV

Q2	MAE
0.6725197	0.0924159

Q2	MAE
0.7231191	0.0841402

Top Ranked Full Model

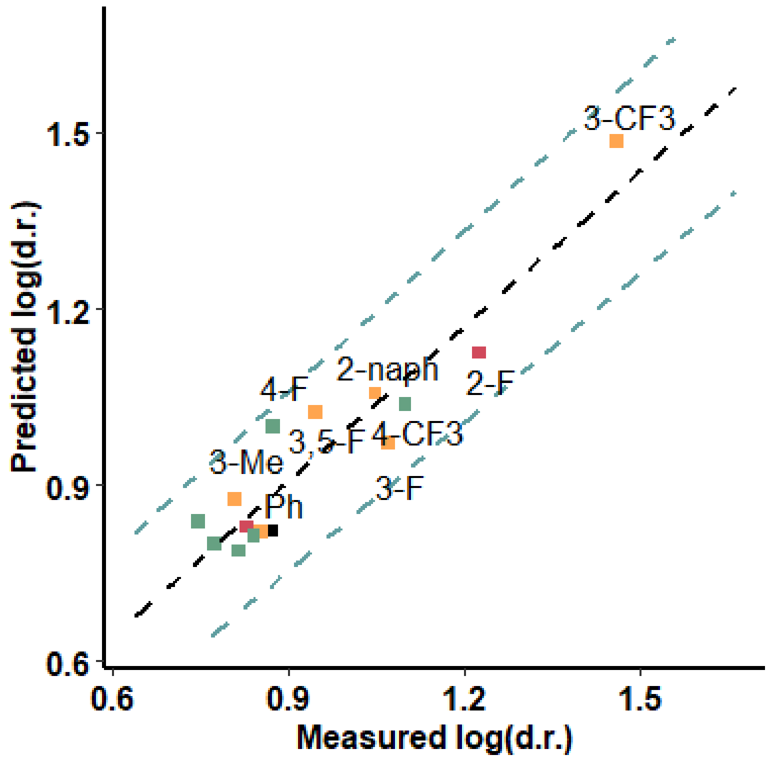

BA + Aldehyde (pi interaction) - oxygen side

formula	R.sq	Q.sq	MAE
output ~ Dist.17..18. + NBO.C.20 + diff.H16.O17	0.9051171	0.7532205	0.0768778
output ~ Dist.18..20. + Dist.10..17. + NBO.H.10	0.8878192	0.7508254	0.0750426

	Estimate	Std. Error	t value	Pr(>|t|)
(Intercept)	0.9516937	0.0176306	53.979641	0.0e+00
Dist.17..18.	-0.3246620	0.0369046	-8.797328	2.6e-06
NBO.C.20	-0.2777336	0.0338176	-8.212698	5.1e-06
diff.H16.O17	0.1655450	0.0213751	7.744749	8.9e-06

3 & 5 fold CV

Q2	MAE

0.7453368	0.0893858

Q2	MAE
0.7574629	0.0799352

Top Ranked Full Model

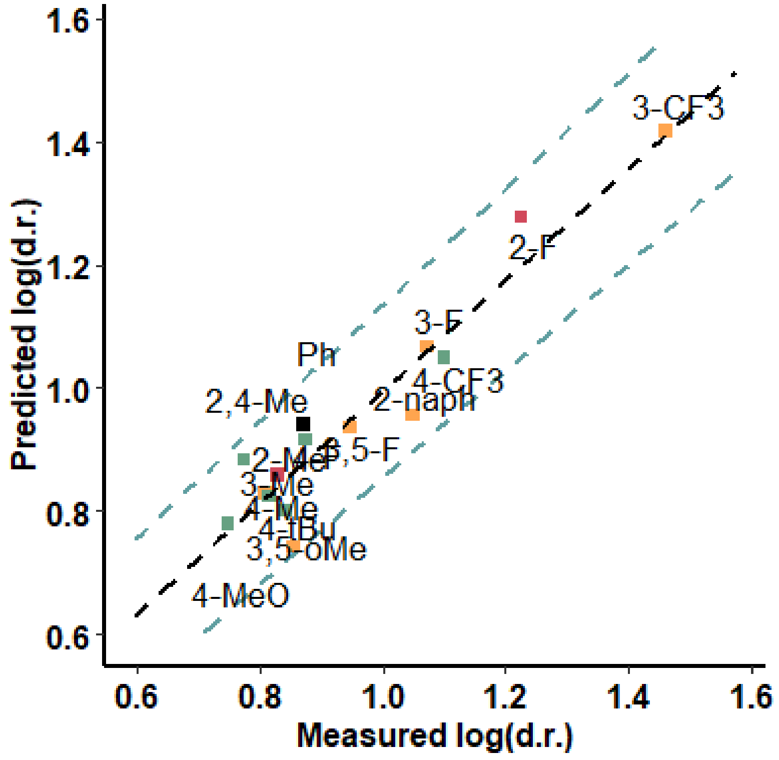

BA + Aldehyde (pi interaction) - hydrogen side

formula	R.sq	Q.sq	MAE
`output ~ Dist.1..10. + diff.B2.09 + B5`	0.8171098	0.6420217	0.0796085
`output ~ NBO.H.19 + diff.B2.09 + B5`	0.8153299	0.6405403	0.0937817

	Estimate	Std. Error	t value	Pr(>|t|)
(Intercept)	0.9516937	0.0244776	38.880229	0.0000000
Dist.1..10.	0.1030092	0.0273220	3.770194	0.0030993
diff.B2.09	-0.2235894	0.0330667	-6.761759	0.0000311
B5	0.1092360	0.0315904	3.457881	0.0053531

3 & 5 fold CV

Q2	MAE

0.5443901	0.1066903

Q2	MAE
0.6015224	0.089799

Top Ranked Full Model

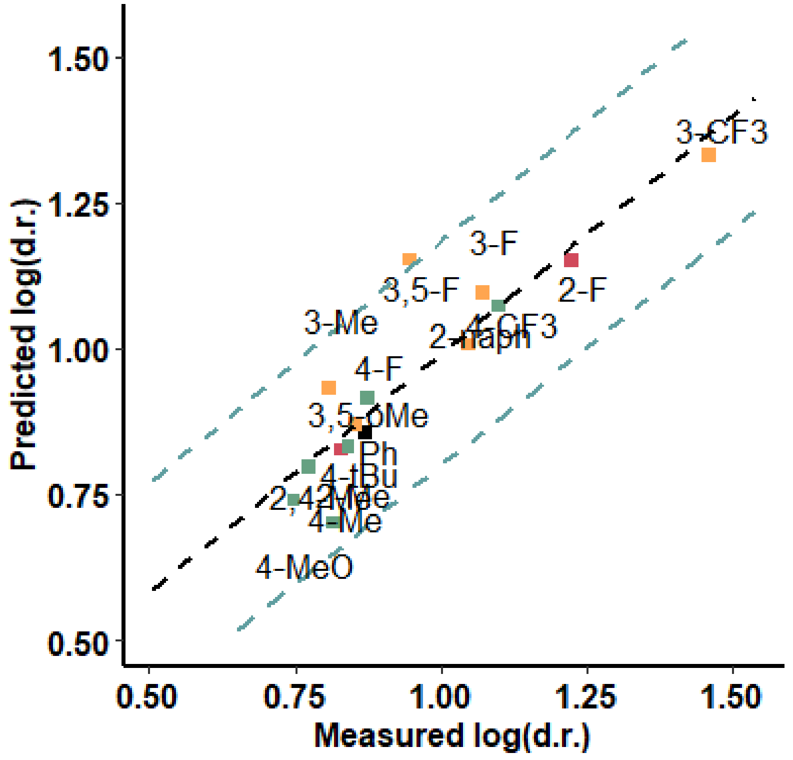

BA + Aldehyde - H bond - aldehyde side

formula	R.sq	Q.sq	MAE
`output ~ X.2.9. + Dist.16..17. + dip_y`	0.8682956	0.7800944	0.0759574
`output ~ X.2.9. + Dist.9..16. + dip_y`	0.8778245	0.7788217	0.0747310

	Estimate	Std. Error	t value	Pr(>|t|)
(Intercept)	0.9516937	0.0207718	45.816720	0.0000000
X.2.9.	0.1253820	0.0222387	5.638000	0.0001515
Dist.16..17.	-0.1084500	0.0215663	-5.028678	0.0003848
dip_y	0.1080663	0.0221957	4.868793	0.0004957

3 & 5 fold CV

Q2	MAE

0.7425314	0.1015883

Q2	MAE
0.7632797	0.0843984

Top Ranked Full Model

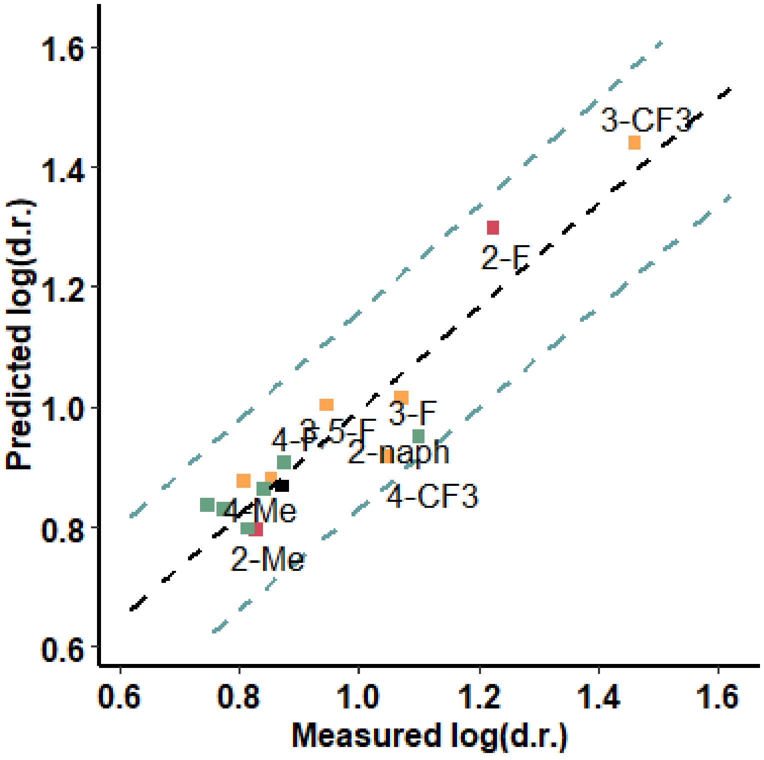

BA + Aldehyde - H bond - opposite to aldehyde

formula	R.sq	Q.sq	MAE
output ~ X.2.3. + X.17.18. + NBO.H.10	0.9345717	0.8829876	0.0622381
output ~ X.2.3. + X.17.18. + Dist.1..10.	0.8704286	0.7968104	0.0822063

	Estimate	Std. Error	t value	Pr(>|t|)
(Intercept)	0.9516937	0.0146405	65.004157	0.00e+00
X.2.3.	-0.1247698	0.0180257	-6.921787	2.51e-05
X.17.18.	-0.1598116	0.0189528	-8.432078	3.90e-06
NBO.H.10	0.2127128	0.0175766	12.102031	1.00e-07

3 & 5 fold CV

Q2	MAE
0.6757456	0.9079589

Q2	MAE
0.800003	0.4067611

Top Ranked Full Model

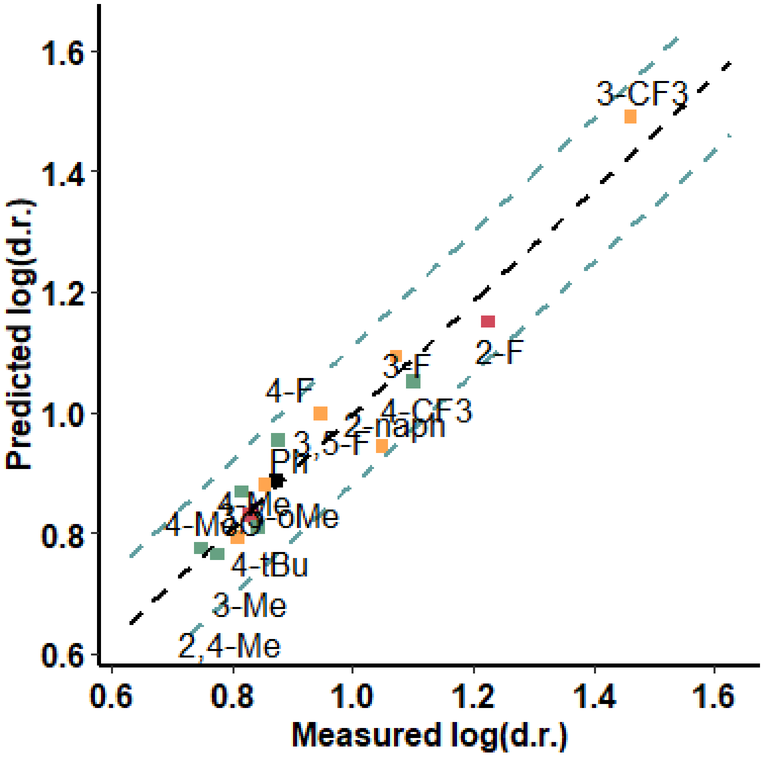

Enamine - far from the active site

formula	R.sq	Q.sq	MAE
output ~ para + dip_x + Total	0.9202811	0.8504402	0.0671754
output ~ para + dip_y + Total	0.9101539	0.8147319	0.0720714

	Estimate	Std. Error	t value	Pr(>|t|)
(Intercept)	0.9516937	0.0161605	58.890246	0.0000000
para	0.0806444	0.0178452	4.519109	0.0008733
dip_x	-0.2482640	0.0598172	-4.150379	0.0016154
Total	0.3861781	0.0587006	6.578779	0.0000398

3 & 5 fold CV

Q2	MAE
0.7961489	0.0754074

Q2	MAE
0.8240744	0.0704558

Top Ranked Full Model

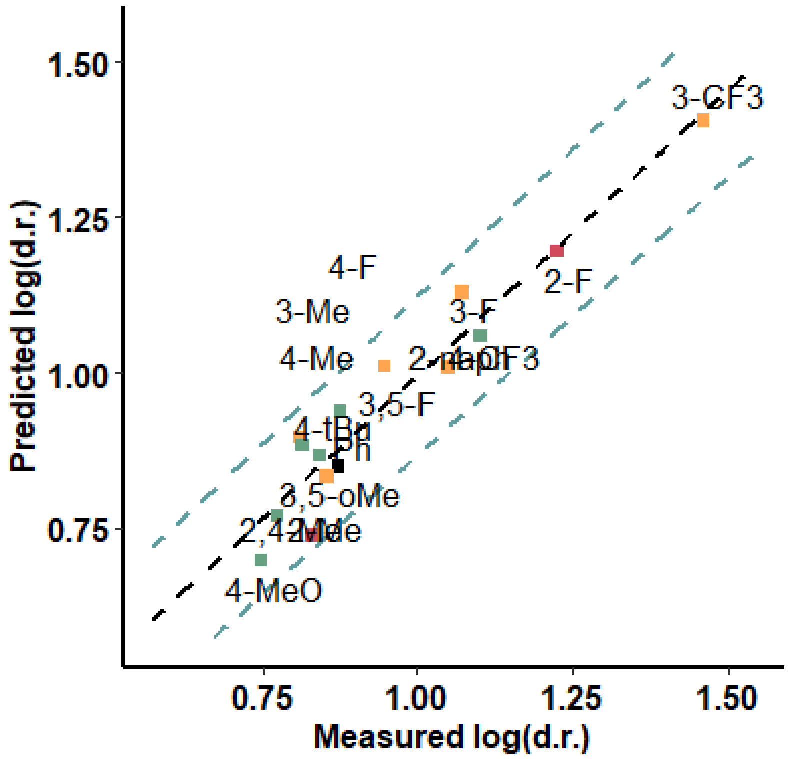

Enamine - close to the active site

formula	R.sq	Q.sq	MAE
`output ~ NBO.C.16 + diff.B2.09 + diff.N24.C25`	0.8554631	0.6905015	0.0855311

	Estimate	Std. Error	t value	Pr(>|t|)
(Intercept)	0.9516937	0.0217602	43.735566	0.0000000
NBO.C.16	0.3158147	0.0436727	7.231407	0.0000168
diff.B2.09	-0.1407929	0.0297186	-4.737532	0.0006119
diff.N24.C25	-0.2922294	0.0371606	-7.863950	0.0000077

3 & 5 fold CV

Q2	MAE
0.5221028	0.1197347

Q2	MAE
0.5996629	0.1036941

Top Ranked Full Model

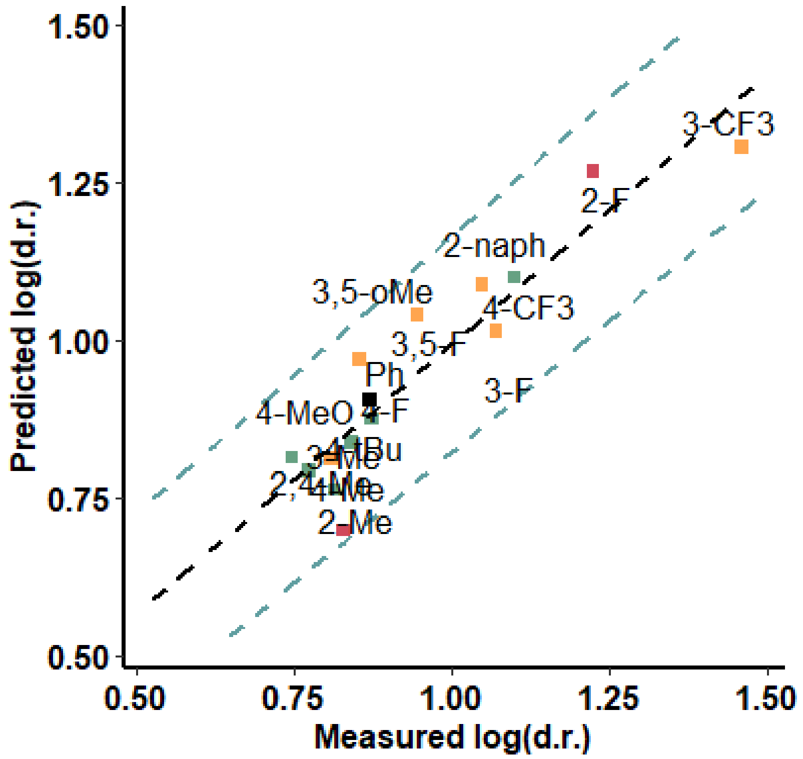

Hexane

Only Boronic Acids

formula	R.sq	Q.sq	MAE
output ~ X.2.9. + Dist.2..3. + B5	0.8755476	0.7691076	0.0643666
output ~ X.2.9. + Dist.9..16. + B5	0.8566010	0.6682341	0.0820453

	Estimate	Std. Error	t value	Pr(>|t|)
(Intercept)	0.5902181	0.0183355	32.189883	0.0000000
X.2.9.	0.1460368	0.0210299	6.944243	0.0000244
Dist.2..3.	-0.0630844	0.0217923	-2.894798	0.0145802

B5	0.1682191	0.0223758	7.517920	0.0000117

3 & 5 fold CV

Q2	MAE
0.666408	0.0827982

Q2	MAE
0.7185957	0.0722703

Top Ranked Full Model

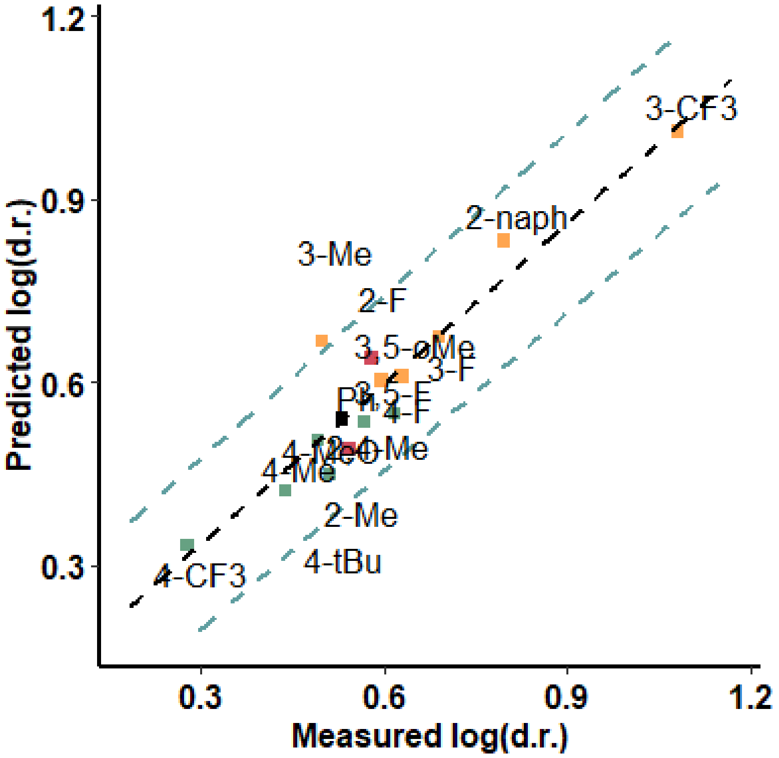

With Catalyst - Close to the active site

formula	R.sq	Q.sq	MAE
`output ~ Total + diff.C17.O18 + B5`	0.7809091	0.5620645	0.0942857
`output ~ Dist.1..2. + Dist.8..14. + B5`	0.7764460	0.5451110	0.0913185

	Estimate	Std. Error	t value	Pr(>|t|)
(Intercept)	0.5902181	0.0243278	24.261006	0.0000000
Total	-0.1259711	0.0297082	-4.240286	0.0013882
diff.C17.O18	-0.0816847	0.0292958	-2.788276	0.0176409

B5	0.0828415	0.0262542	3.155356	0.0091544

3 & 5 fold CV

Q2	MAE
0.4724348	0.1141425

Q2	MAE
0.5123817	0.101973

Top Ranked Full Model

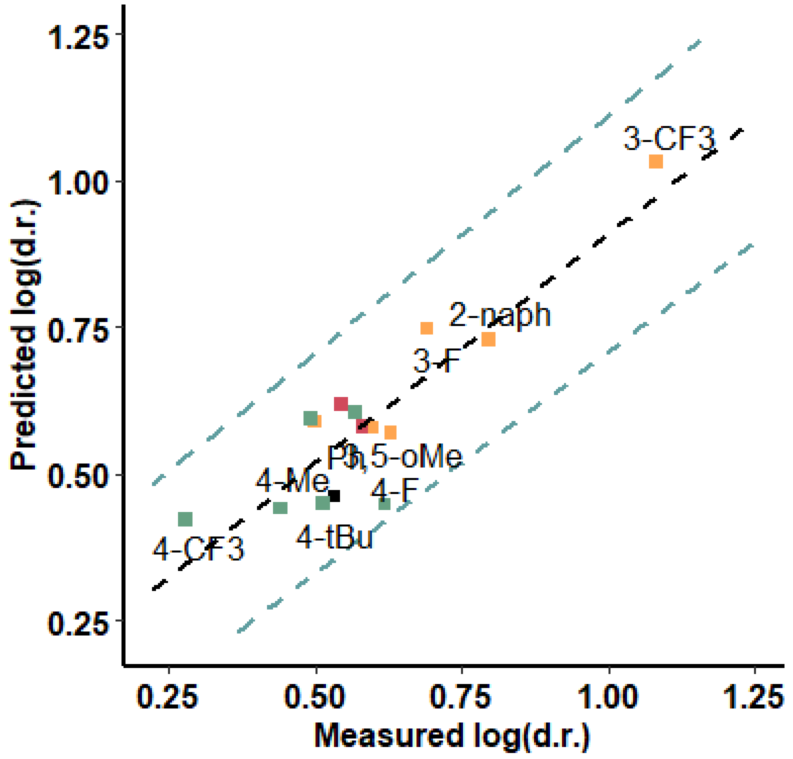

With Catalyst - far from the active site

formula	R.sq	Q.sq	MAE
output ~ Dist.1..2. + Total + B5	0.7616516	0.565349	0.0881043

	Estimate	Std. Error	t value	Pr(>|t|)
(Intercept)	0.5902181	0.0253745	23.260277	0.0000000
Dist.1..2.	-0.1008379	0.0352923	-2.857220	0.0155940
Total	0.1294074	0.0333966	3.874872	0.0025861
B5	0.1228454	0.0288244	4.261853	0.0013388

3 & 5 fold CV

Q2	MAE
0.4813282	0.1061982

Q2	MAE
0.5275744	0.0953394

Top Ranked Full Model

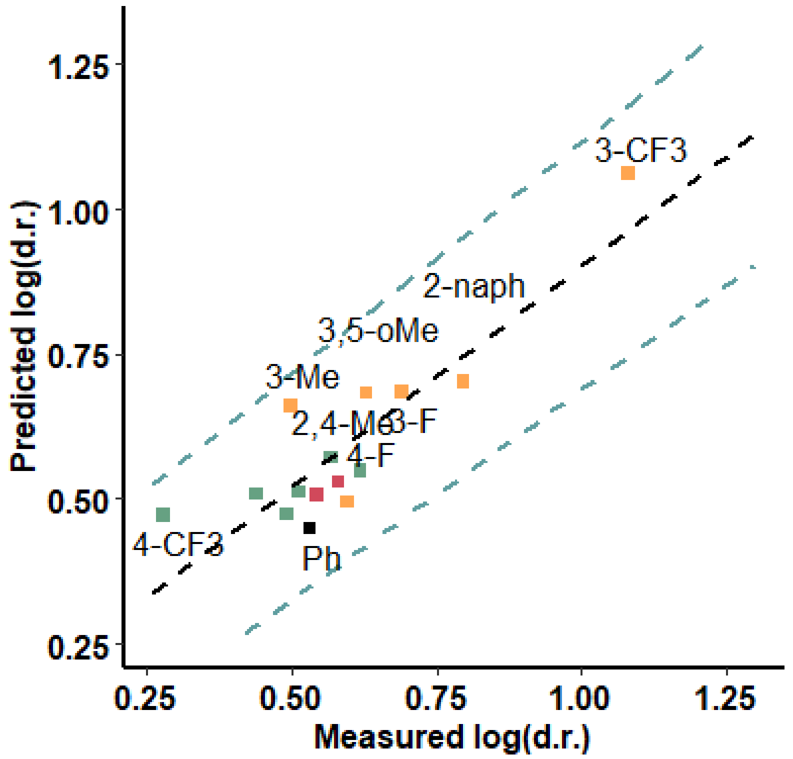

BA + Aldehyde (pi interaction) - oxygen side

formula	R.sq	Q.sq	MAE
`output ~ X.2.9. + X.1.10. + X.18.19.`	0.7995523	0.6454449	0.1756751
`output ~ X.18.19. + X.18.20. + diff.C18.C20`	0.7632680	0.5832786	0.2354729

	Estimate	Std. Error	t value	Pr(>|t|)
(Intercept)	0.5902181	0.0232698	25.364159	0.0000000
X.2.9.	-0.0525346	0.0248238	-2.116300	0.0579406
X.1.10.	-0.0789994	0.0255239	-3.095115	0.0101926

X.18.19.	0.1670208	0.0257086	6.496683	0.0000445

3 & 5 fold CV

Q2	MAE
0.568998	1.692854

Q2	MAE
0.5710617	1.291109

Top Ranked Full Model

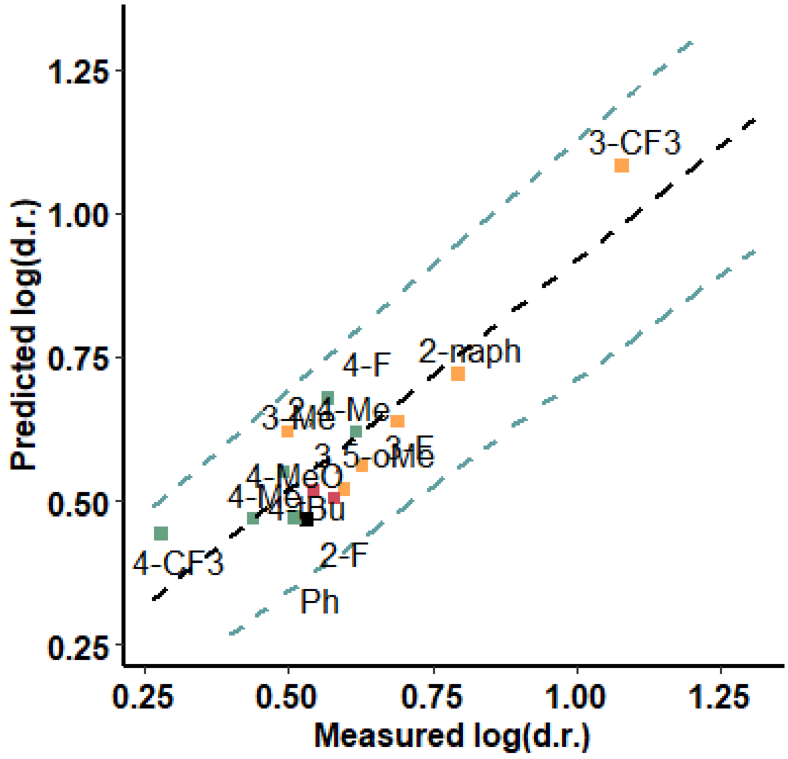

BA + Aldehyde (pi interaction) - hydrogen side

formula	R.sq	Q.sq	MAE
output ~ Dist.9..16. + Dist.17..18. + NBO.C.20	0.8320009	0.6121787	0.0894207
output ~ Dist.9..16. + Dist.17..18. + diff.C18.C20	0.8177483	0.6027992	0.0856343

	Estimate	Std. Error	t value	Pr(>|t|)
(Intercept)	0.5902181	0.0213032	27.705600	0.0000000

Dist.9..16.	-0.2378390	0.0447972	-5.309241	0.0002489
Dist.17..18.	0.5739637	0.0792099	7.246111	0.0000165
NBO.C.20	0.3993029	0.0566846	7.044294	0.0000214

3 & 5 fold CV

Q2	MAE
0.5765431	0.0956017

Q2	MAE
0.6045818	0.089529

Top Ranked Full Model

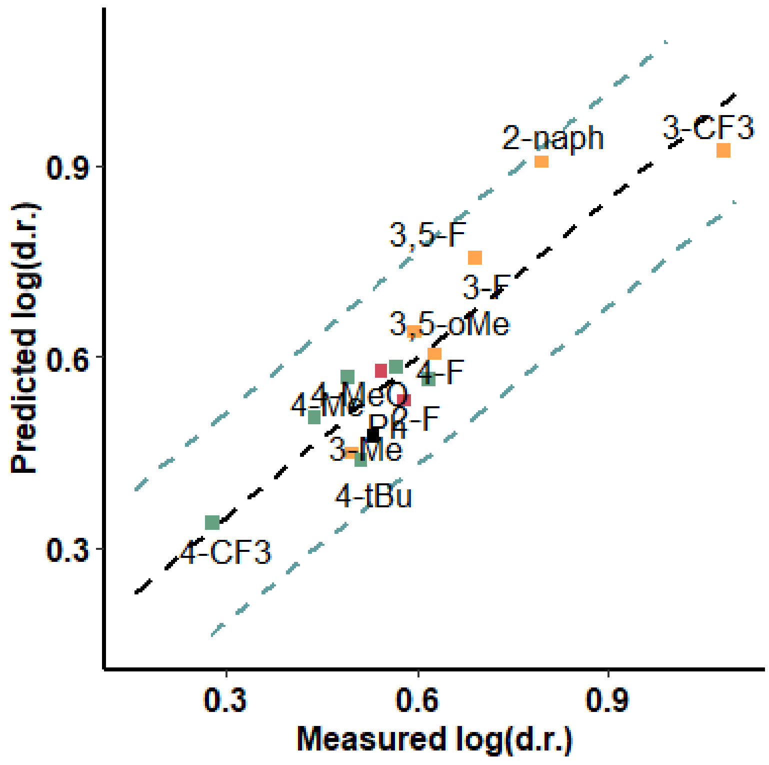

BA + Aldehyde - H bond - aldehyde side

formula	R.sq	Q.sq	MAE
`output ~ para + dip_z + B5`	0.6540133	0.3975334	0.0977147
`output ~ Dist.2..3. + NBO.C.20 + B5`	0.6864446	0.3596414	0.1086377

	Estimate	Std. Error	t value	Pr(>|t|)
(Intercept)	0.5902181	0.0305718	19.305956	0.0000000

para	0.0800794	0.0331397	2.416415	0.0342235
dip_z	-0.0586576	0.0343380	-1.708241	0.1156203
B5	0.0874714	0.0340409	2.569600	0.0260690

3 & 5 fold CV

Q2	MAE
0.3454542	0.1211554

Q2	MAE
0.3623049	0.1099832

Top Ranked Full Model

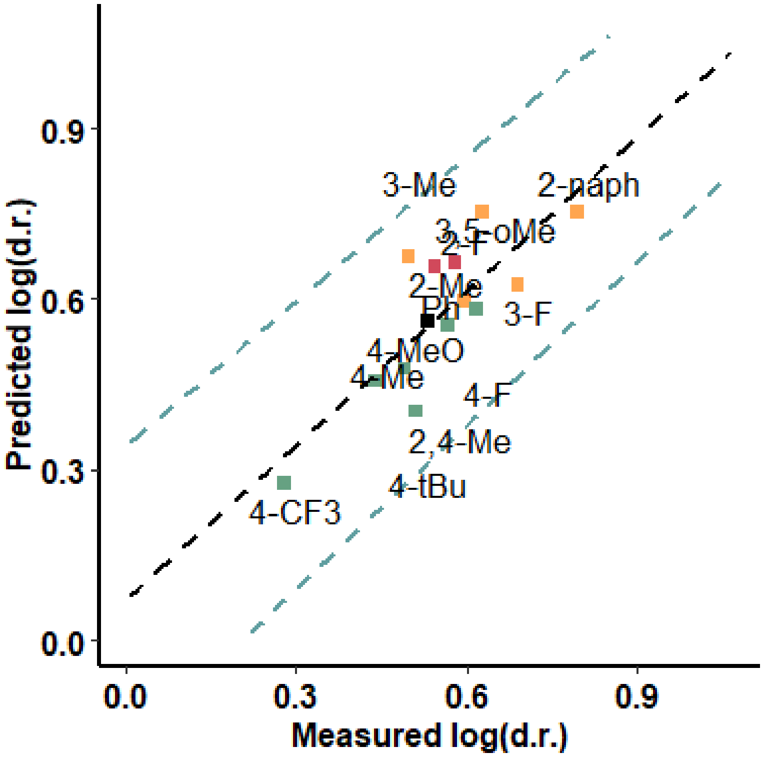

BA + Aldehyde - H bond - opposite to aldehyde

formula	R.sq	Q.sq	MAE
`output ~ X.2.3. + diff.C18.H19 + B5`	0.8033539	0.6259376	0.0880591

	Estimate	Std. Error	t value	Pr(>|t|)
(Intercept)	0.5902181	0.0230481	25.608157	0.0000000
X.2.3.	-0.0658658	0.0246705	-2.669820	0.0218010

diff.C18.H19	-0.1029966	0.0254818	-4.041973	0.0019419
B5	0.1640537	0.0259575	6.320084	0.0000568

3 & 5 fold CV

Q2	MAE
0.4603351	0.1200071

Q2	MAE
0.5333689	0.1026862

Top Ranked Full Model

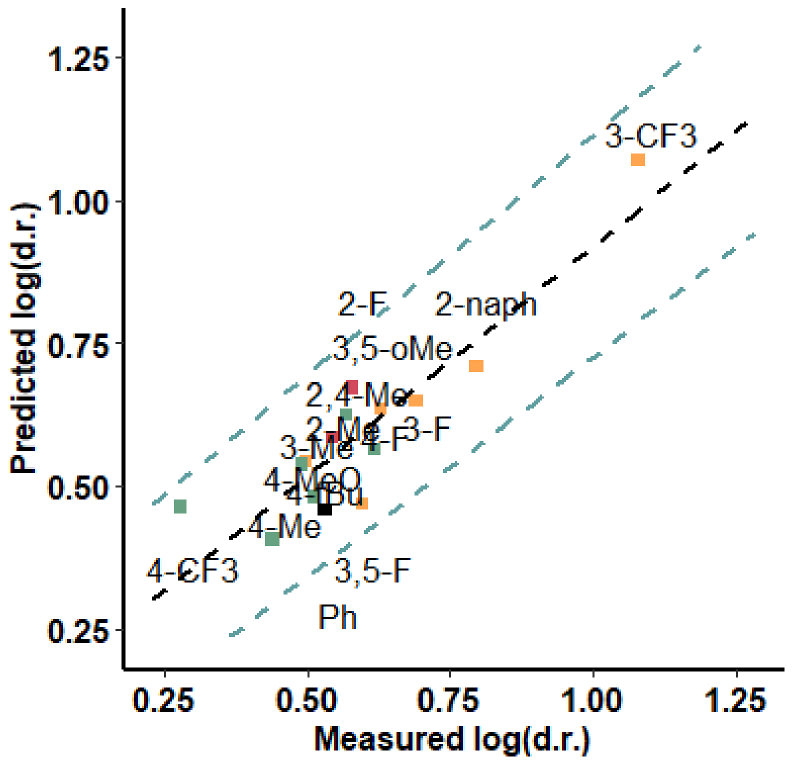

Enamine - far from the active site

formula	R.sq	Q.sq	MAE
output ~ X.1.10. + dip_z + B5	0.6959192	0.4826160	0.0944714
output ~ Dist.1..10. + dip_z + B5	0.7020307	0.4816768	0.0948664

	Estimate	Std. Error	t value	Pr(>|t|)
(Intercept)	0.5902181	0.0286606	20.593327	0.0000000
X.1.10.	0.0541858	0.0311904	1.737260	0.1102207

dip_z	-0.0984117	0.0315416	-3.120057	0.0097490
B5	0.1557786	0.0330449	4.714155	0.0006354

3 & 5 fold CV

Q2	MAE
0.297223	0.1537918

Q2	MAE
0.3472259	0.1283395

Top Ranked Full Model

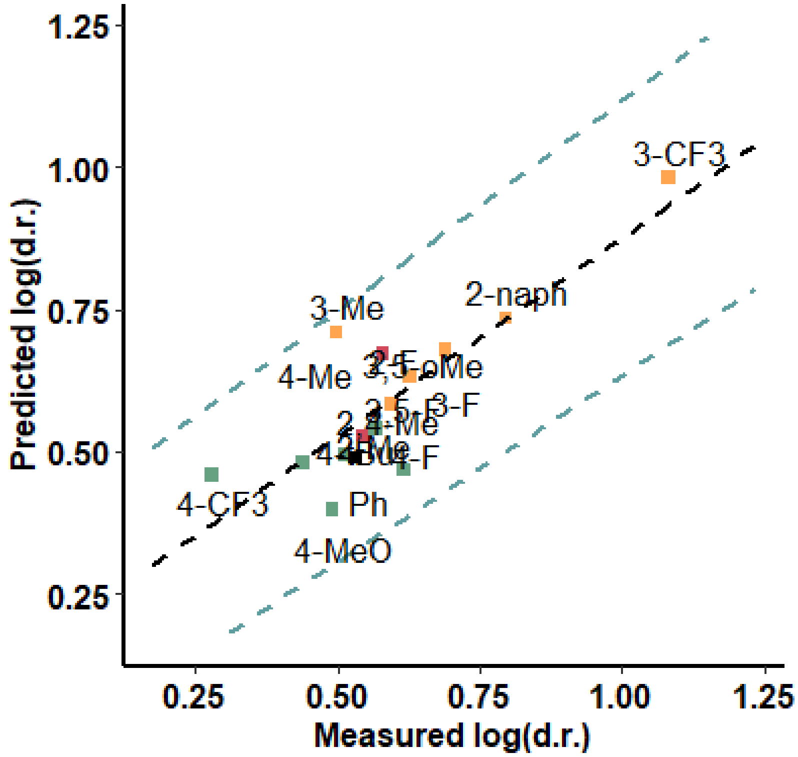

Enamine - close to the active site

formula	R.sq	Q.sq	MAE
output ~ Dist.10..36. + dip_z + NBO.C.16	0.6564753	0.4011530	0.0908784
output ~ dip_x + NBO.C.16 + NBO.C.25	0.7055401	0.3117577	0.1080938

	Estimate	Std. Error	t value	Pr(>|t|)
(Intercept)	0.5902181	0.0304628	19.375013	0.0000000

Dist.10..36.	0.1667968	0.0421312	3.958982	0.0022380
dip_z	0.0808861	0.0320510	2.523668	0.0282903
NBO.C.16	0.1559673	0.0420735	3.707017	0.0034591

3 & 5 fold CV

Q2	MAE
0.2949039	0.1317234

Q2	MAE
0.3310653	0.1075782

Top Ranked Full Model

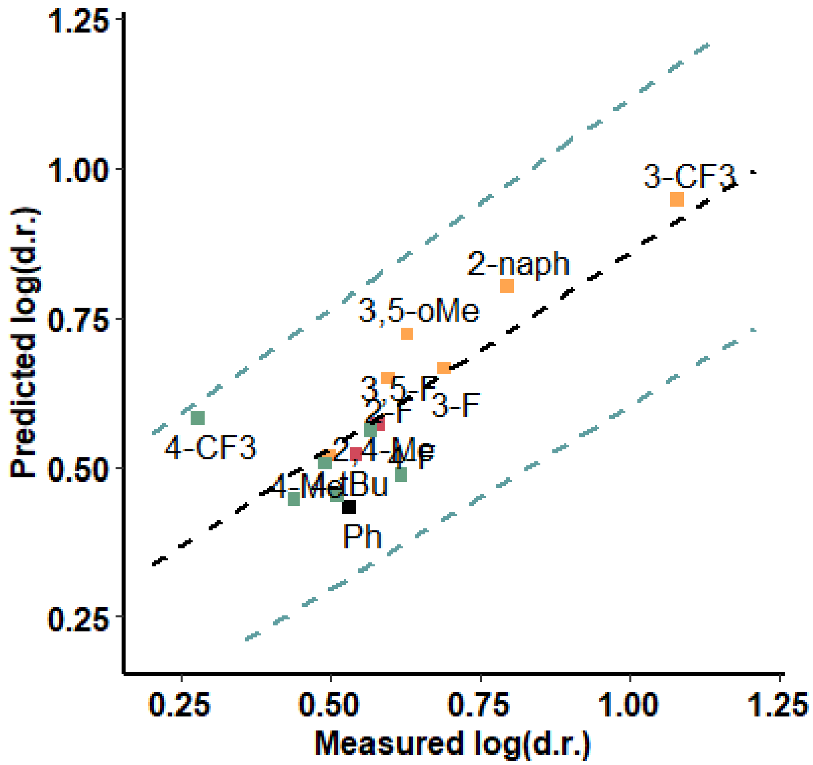

Hexane - against ee

Only Boronic Acids

formula	R.sq	Q.sq	MAE
output ~ NBO.H.16 + diff.B2.C3 + B5	0.8092831	0.5969891	0.1564650
output ~ NBO.C.3 + NBO.H.16 + B5	0.8061288	0.5901086	0.1563283

	Estimate	Std. Error	t value	Pr(>|t|)
(Intercept)	0.7999364	0.0408109	19.601048	0.00e+00
NBO.H.16	0.3526279	0.0586513	6.012276	8.77e-05
diff.B2.C3	0.2913971	0.0628399	4.637133	7.20e-04
B5	0.2346225	0.0476628	4.922548	4.55e-04

3 & 5 fold CV

Q2	MAE
0.5444704	0.1791977

Q2	MAE
0.5765664	0.1659519

Top Ranked Full Model

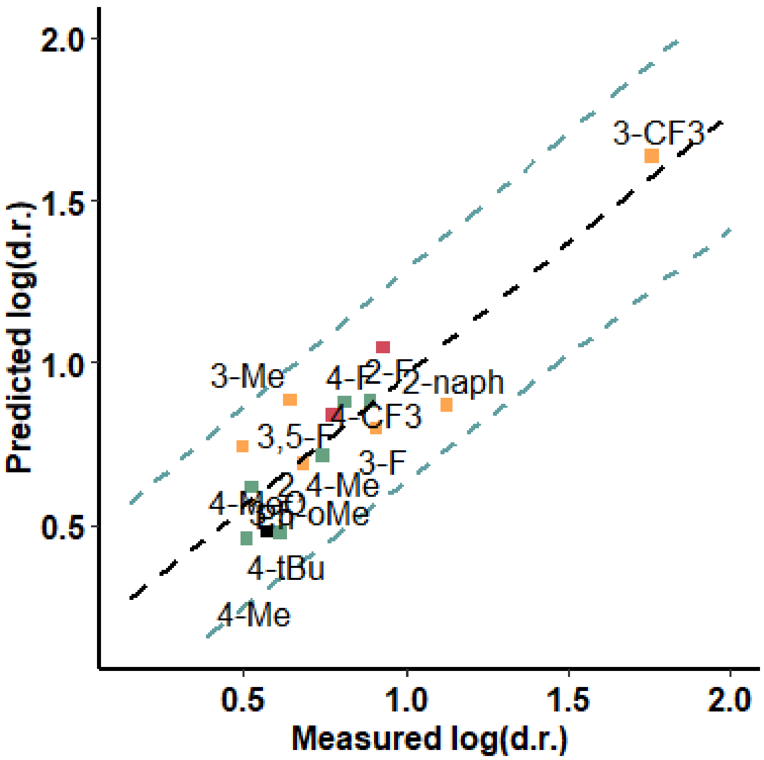

With Catalyst - Close to the active site

formula	R.sq	Q.sq	MAE
`output ~ dip_x + diff.N19.H20 + B5`	0.9110369	0.8371205	0.1031061

	Estimate	Std. Error	t value	Pr(>|t|)
(Intercept)	0.7999364	0.0278732	28.699141	0.00e+00
dip_x	-0.2439328	0.0361628	-6.745402	3.18e-05
diff.N19.H20	-0.2921078	0.0341089	-8.563989	3.40e-06
B5	0.2577144	0.0315807	8.160516	5.40e-06

3 & 5 fold CV

Q2	MAE
0.7837803	0.121837

Q2	MAE
0.8105981	0.1098384

Top Ranked Full Model

With Catalyst - far

formula	R.sq	Q.sq	MAE
`output ~ Total + NBO.O.16 + NBO.O.18`	0.8541393	0.5723166	0.1294332
`output ~ X.16.17. + Total + diff.O16.C17`	0.8599285	0.5507880	0.1476431

	Estimate	Std. Error	t value	Pr(>|t|)
(Intercept)	0.7999364	0.0356903	22.413247	0.0000000
Total	0.3500325	0.0643050	5.443314	0.0002029
NBO.O.16	-0.2088682	0.0504027	-4.143991	0.0016330
NBO.O.18	-0.2009904	0.0776564	-2.588200	0.0252190

3 & 5 fold CV

Q2	MAE
0.5218771	0.1684343

Q2	MAE
0.5576643	0.1431278

Top Ranked Full Model

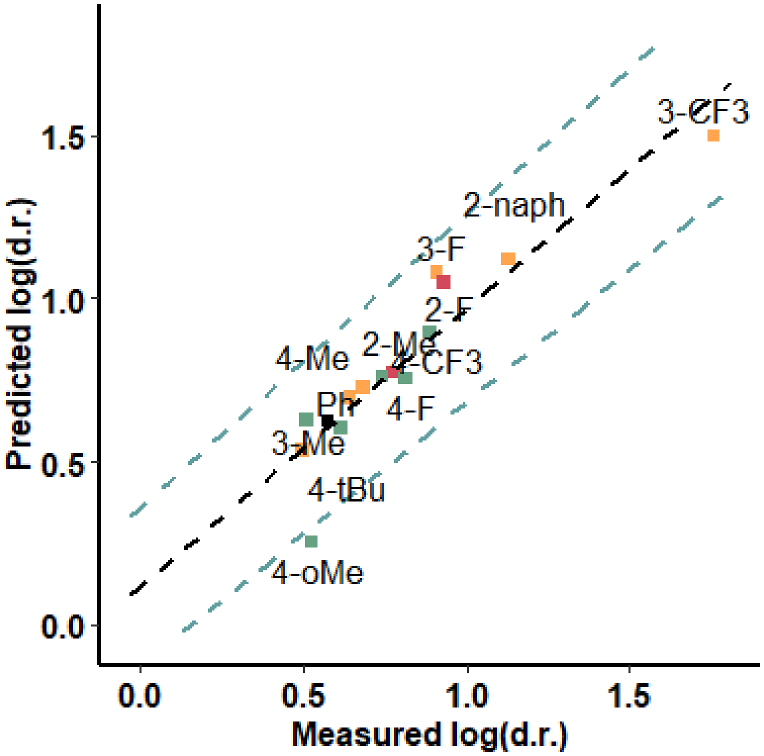

BA + Aldehyde (pi interaction) - oxygen side

formula	R.sq	Q.sq	MAE
output ~ X.1.2. + X.18.19. + NBO.H.10	0.8644711	0.6885338	3.846756
output ~ X.2.9. + X.18.19. + NBO.H.10	0.8839083	0.6885221	4.566467

	Estimate	Std. Error	t value	Pr(>|t|)
(Intercept)	0.7999364	0.0344031	23.251869	0.0000000
X.1.2.	-0.0965472	0.0367515	-2.627029	0.0235317
X.18.19.	0.2479915	0.0374194	6.627353	0.0000372
NBO.H.10	0.1216105	0.0376777	3.227650	0.0080488

3 & 5 fold CV

Q2	MAE
0.6867888	5.643921

Q2	MAE
0.6887585	4.541408

Top Ranked Full Model

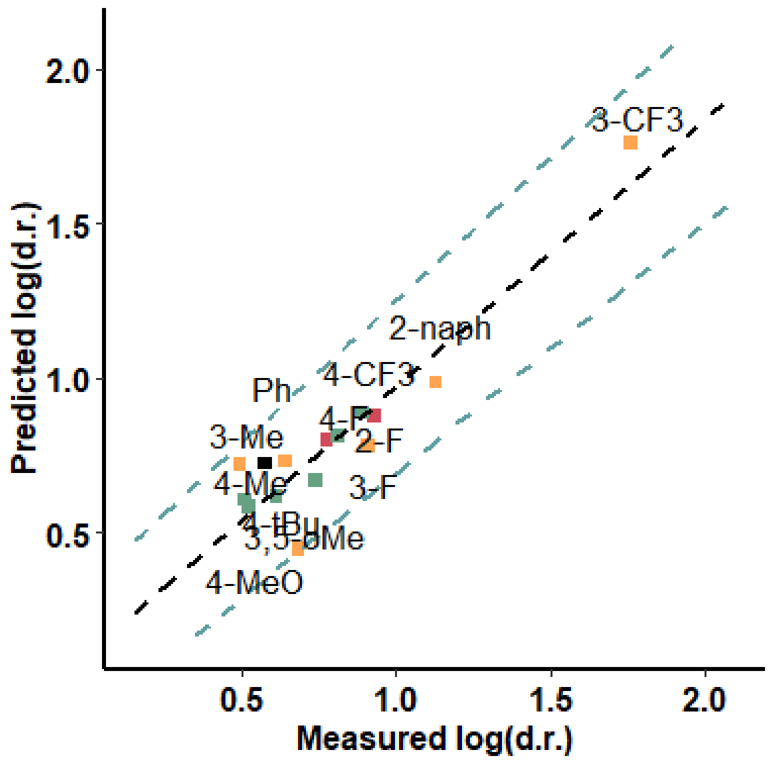

BA + Aldehyde (pi interaction) - hydrogen side

formula	R.sq	Q.sq	MAE
output ~ X.18.19. + diff.B2.09 + B5	0.7091732	0.3900531	0.1979070
output ~ Dist.17..18. + diff.B2.09 + B5	0.6915228	0.3553806	0.1948376

	Estimate	Std. Error	t value	Pr(>|t|)
(Intercept)	0.7999364	0.0503963	15.872914	0.0000000
X.18.19.	-0.1633268	0.0571441	-2.858155	0.0155679
diff.B2.09	-0.2115888	0.0673857	-3.139965	0.0094090
B5	0.2601548	0.0655114	3.971136	0.0021918

3 & 5 fold CV

Q2	MAE
0.3486724	0.2297474

Q2	MAE
0.3608696	0.209722

Top Ranked Full Model

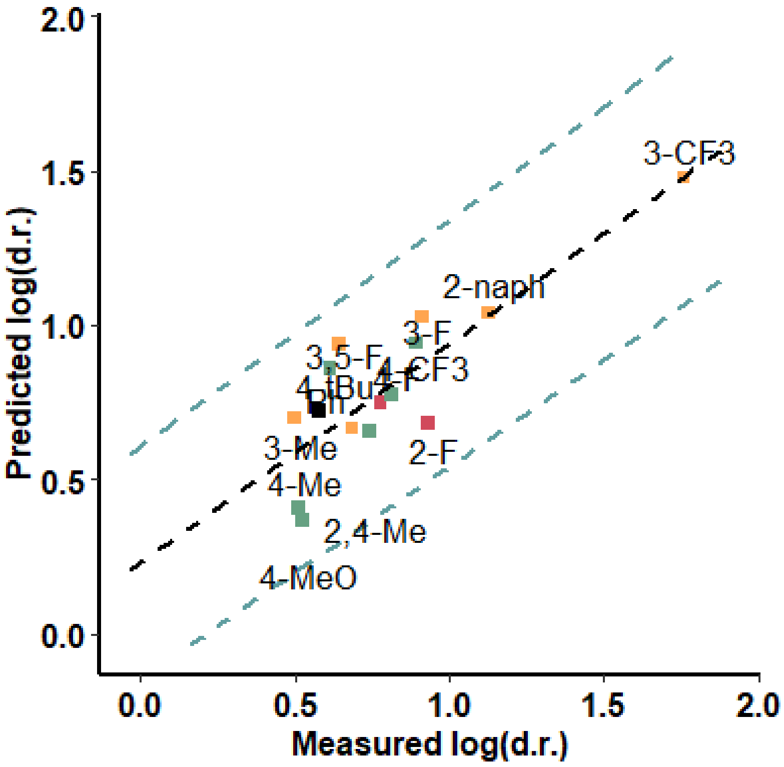

BA + Aldehyde - H bond - aldehyde side

formula	R.sq	Q.sq	MAE
output ~ Dist.18..20. + Dist.10..17. + diff.O1.B2	0.7811492	0.4855365	0.1775713
output ~ Dist.18..20. + diff.B2.C3 + B5	0.7866050	0.4683771	0.1730788

	Estimate	Std. Error	t value	Pr(>|t|)
(Intercept)	0.7999364	0.0437175	18.297839	0.0000000
Dist.18..20.	-0.4839826	0.0882849	-5.482053	0.0001913
Dist.10..17.	1.1066412	0.2740859	4.037570	0.0019566
diff.O1.B2	1.0434098	0.2826770	3.691173	0.0035559

3 & 5 fold CV

Q2	MAE
0.4510126	0.1972915

Q2	MAE
0.4635572	0.1871171

Top Ranked Full Model

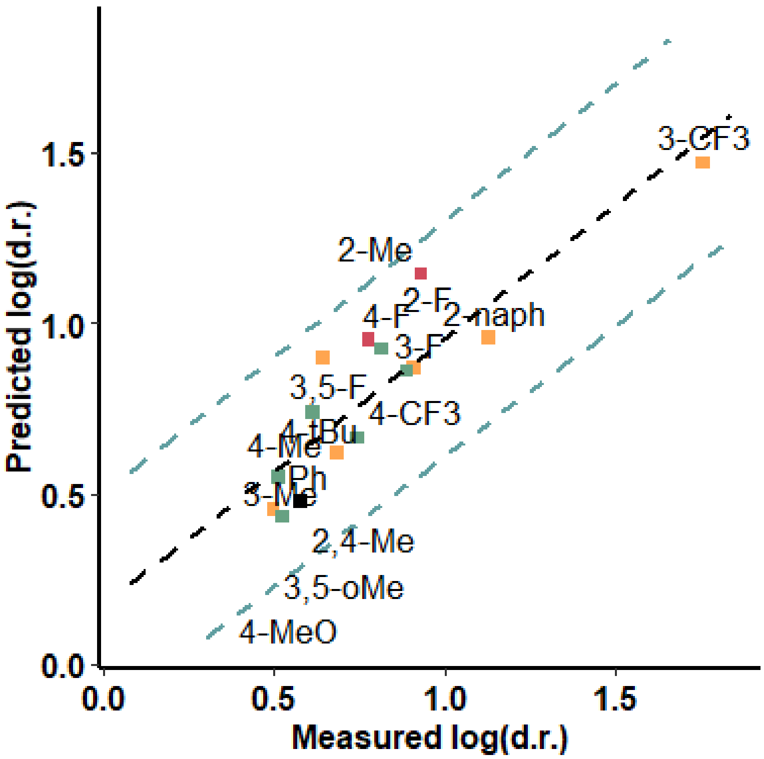

BA + Aldehyde - H bond - opposite to aldehyde

formula	R.sq	Q.sq	MAE
output ~ `X.2.3.` + `X.17.18.` + `Dist.10..17.`	0.853099	0.7342390	0.1113065
output ~ `X.2.3.` + `diff.C18.H19` + `B5`	0.853946	0.5654389	0.1492731

	Estimate	Std. Error	t value	Pr(>|t|)
(Intercept)	0.7999364	0.0358174	22.333740	0.00e+00
X.2.3.	-0.2776442	0.0458423	-6.056509	8.23e-05
X.17.18.	-0.3471624	0.0493848	-7.029747	2.18e-05
Dist.10..17.	-0.3085719	0.0460402	-6.702226	3.37e-05

3 & 5 fold CV

Q2	MAE
0.5650586	1.152972

Q2	MAE

0.6649241	0.2985166

Top Ranked Full Model

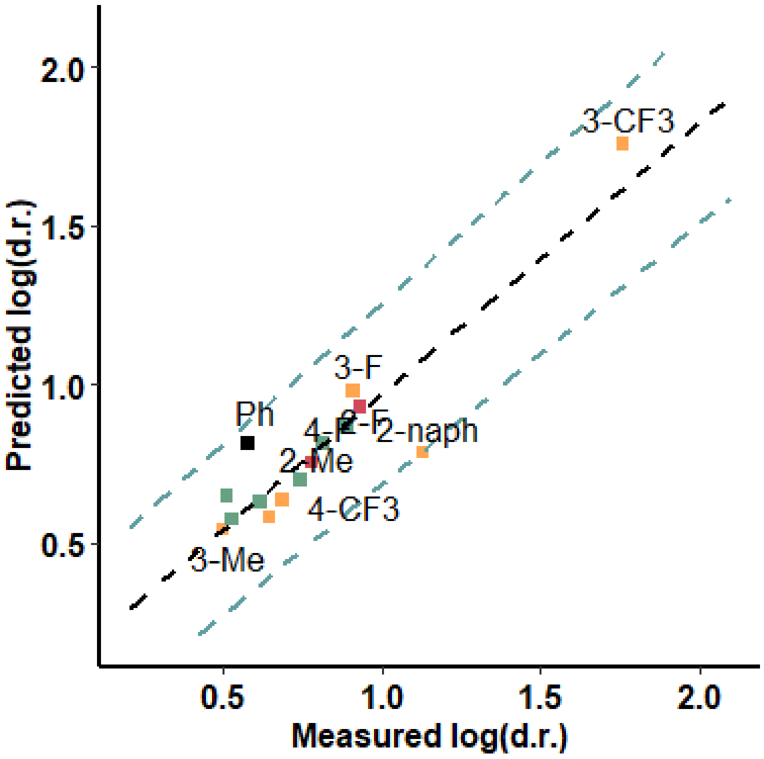

Enamine - far from the active site

formula	R.sq	Q.sq	MAE
output ~ Dist.2..3. + Total + B5	0.8079764	0.6314503	0.1518676
output ~ para + Dist.9..16. + Total	0.8272442	0.6160071	0.1394574

	Estimate	Std. Error	t value	Pr(>|t|)
(Intercept)	0.7999364	0.0409505	19.534241	0.0000000
Dist.2..3.	-0.1295211	0.0610840	-2.120377	0.0575323
Total	0.3180661	0.0575738	5.524495	0.0001795
B5	0.2053727	0.0484404	4.239699	0.0013896

3 & 5 fold CV

Q2	MAE
0.5498667	0.1999909

Q2	MAE

0.5811175	0.1579649

Top Ranked Full Model

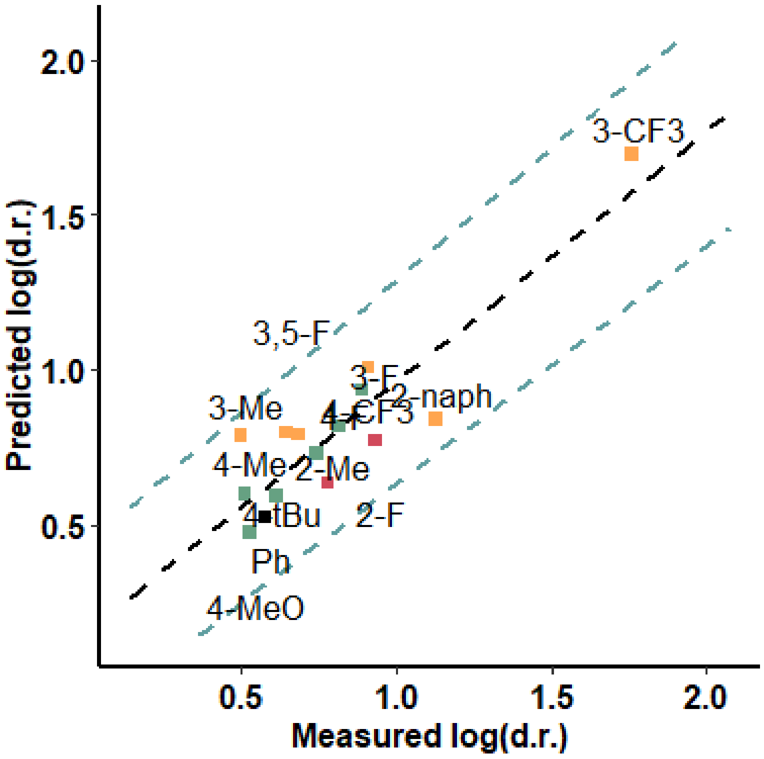

Enamine - close to the active site

formula	R.sq	Q.sq	MAE
output ~ NBO.B.2 + NBO.C.16 + diff.N24.C25	0.7885358	0.5136736	0.1664176
output ~ X.24.25. + dip_y + NBO.C.16	0.7685915	0.4037103	0.2019363

	Estimate	Std. Error	t value	Pr(>|t|)
(Intercept)	0.7999364	0.0429734	18.614674	0.0000000
NBO.B.2	-0.2185839	0.0669724	-3.263792	0.0075480
NBO.C.16	0.5533003	0.0922491	5.997894	0.0000895
diff.N24.C25	-0.5748252	0.0984986	-5.835870	0.0001132

3 & 5 fold CV

Q2	MAE
0.451171	0.1922528

Q2	MAE
0.4779782	0.1783278

Top Ranked Full Model

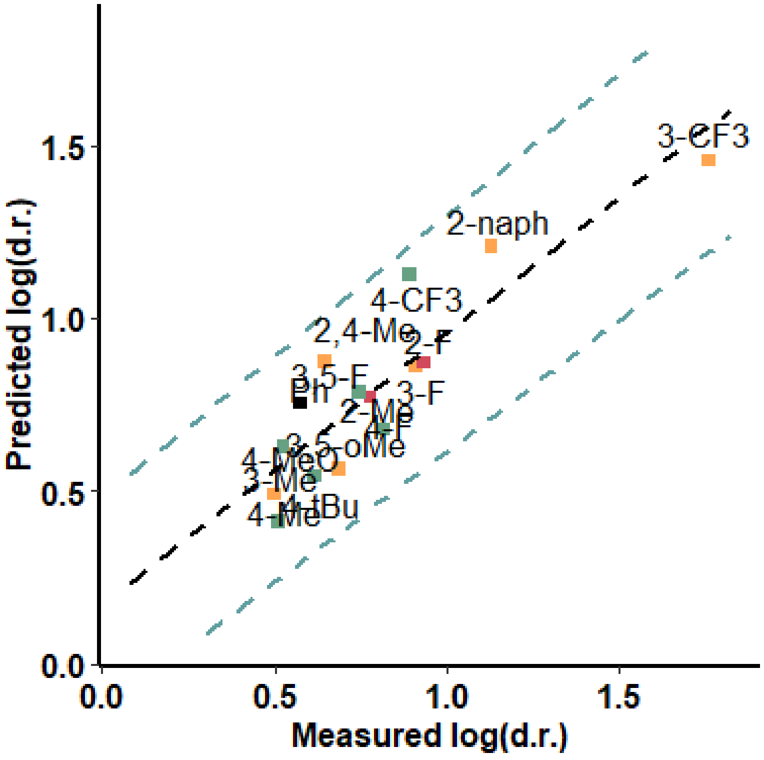

Supplement: Supplementary file 2 — jo1c02778_si_002.pdf [file jo1c02778_si_002.pdf]
