# Supplementary material for: Effect of Solvents on Proline Modified at the Secondary Sphere: A Multivariate Exploration
Source: J Org Chem. 2022 Jan 12;87(3):1850–7. doi: 10.1021/acs.joc.1c02778 (PMC9182215; doi:10.1021/acs.joc.1c02778)
Supplement: Supplementary file 3 — jo1c02778_si_003.pdf [file jo1c02778_si_003.pdf]

Support information

The effect of solvents on proline modified at the secondary sphere: A multivariate exploration

Danilo M. Lustosa, Shahar Barkai, Ido Domb, Anat Milo* Department of Chemistry, Ben-Gurion University of the Negev, Beer Sheva, Israel.

*anatmilo@bgu.ac.il.

Table of Contents

1. General Information S3

2. General Procedures S4

3. Building the data set: outcomes of reactions in different solvents. S7

4. Reaction optimization: Reducing the amount of ketone S12

5. Probing boronic acid influence with methyl prolinate S14

6. Characterization of Boroxines: S15

7. Characterization of (S)-2-((R)-hydroxy(4-nitrophenyl)methyl)cyclopentan-1-one: S19

8. HPLC Traces for Table S1. Boronic acid screening and blanks in acetonitrile S20

9. HPLC Traces for Table S2. Boronic acid screening and blanks in chloroform S37

10. HPLC Traces for Table S3. Boronic acid screening and blanks in neat cyclopentanone S54

11. HPLC Traces for Table S4. Boronic acid screening and blanks in MeOH S70

12. HPLC Traces for Table S5. Boronic acid screening and blanks in hexane S87

13. HPLC Traces for Table S6. Reducing amount of ketone with 3,5-F-phenyl boronic acid S104

14. HPLC Traces for Table S7. Reducing amount of ketone with 3-CF_3-phenyl boronic acid S116

15. HPLC Traces for Table S8. Evaluating the influence of boronic acid in methyl prolinate catalyzed aldol reaction S120

16. NMR for Boroxines S124

17. NMR of isolated (S)-2-((R)-hydroxy(4-nitrophenyl)methyl)cyclopentan-1-one S132

18. NMR traces for diastereoselectivity determination S133

18.1. NMR Traces for Table S1. Boronic acid Screening and blanks in acetonitrile S134

18.2. NMR Traces for Table S2. Boronic acid screening and blanks in chloroform S167

18.3. NMR Traces for Table S3. Boronic acid screening and blanks in neat cyclopentanone S201

18.4. NMR Traces for Table S4. Boronic acid screening and blanks in MeOH S235

18.5. NMR Traces for Table S5. Boronic acid screening and blanks in hexane S269

18.6. NMR Traces for Table S6. Reducing amount of ketone with 3,5-F-phenyl boronic acid S304

18.7. NMR Traces for Table S7. Reducing amount of ketone with 3-CF_3-phenyl boronic acid S328

18.8. NMR Traces for Table S8. Evaluating the influence of boronic acid in methyl prolinate catalyzed aldol reaction S336

18. High-resolution (HR)-MS experiments S344

19. NMR of isolated (S)-2-((R)-hydroxy(4-nitrophenyl)methyl)cyclopentan-1-one S352

20. References – Experimental Supporting Information S353

21. Multivariate Modelling Supporting Information S355

22. Optimized structures – xyz coordinates S367

23. References – Multivariate Modelling Supporting Information S556

1. General Information

Chemicals, unless differently mentioned, were purchased commercially from suppliers (Sigma Aldrich, Merck, Acros, Alfa Aesar) and used with no prior purification. Liquid aldehydes were distilled before use. Dry solvents were prepared from commercial grade solvents (AR), dried over activated molecular sieves (3Å) (20% m/v) for at least 3 days. Thin-layer chromatography (TLC) analysis was performed using Merck silica gel 60 F254 TLC plates. Detection was performed using UV-light (254 nm) or staining solutions (KMnO4 and p-anisaldehyde). Column chromatography was performed on Merck Silica Gel 60 Å 230 X 400 mesh. As eluents, petroleum ether (PE), ethyl acetate (EA) and dichloromethane (DCM) were commonly used. Nuclear magnetic resonance (NMR) spectra were recorded using Bruker DPX400 spectrometer. 1H and 13C chemical shifts are reported in parts per million and residual solvent peak is used as reference. For 1H NMR, the multiplicity of the pics are described as: s (singlet), d (doublet), t (triplet), q (quartet), m (multiplet). Solvent abbreviations are reported as follows: EtOAc = ethyl acetate, Hex = hexanes, DCM = dichloromethane, ACN = Acetonitrile, MeOH = methanol, THF = tetrahydrofuran. HR-MS data were obtained using a Thermoscientific LTQU XL Orbitrap HRMS equipped with APCI (atmospheric-pressure chemical ionization). HPLC grade solvent used for HPLC analysis: n-hexane, acetonitrile, isopropyl alcohol and water. Enantiomeric excesses were measured on a Shimadzu high performance liquid chromatography, LC-20A series chiral HPLC using Chiralpak IC (Chiralcel) column.

2. General Procedures

GP1 – Subsets: Neat conditions, Acetonirtile, MeOH

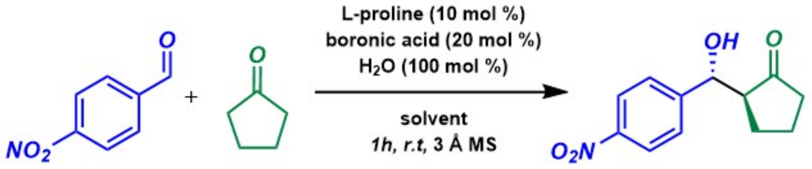

A 5 mL vial, containing 5.8 g of Proline (0.05 mmol) and a stirring bar was placed under argon (with a balloon). To this flask, 25 mg of activated molecular sieves (3 Å) and a boroxine derivative were also added (0.0333 mmol). Next, 0.8 mL of a stock solution of internal standard 1,3,5-trimethoxybenzene (25 mg/mL, 0.149 M) and water (11.3 □L of water per 1 mL of solvent) in 37.5% V of cyclopentanone and 62.5% V of a given solvent was also added to the solids in the vial. The mixture was stirred for 15 min at ambient temperature followed by addition of aldehyde (0.5 mmol). The reaction was then let to stir for 1 hour and after that quenched with a saturated solution of ammonium chloride. The organic phase was then reserved, and the water phase was extracted once again with chloroform. The organic phases were then combined and dried over magnesium sulfate. Lastly, solvent was removed under reduced pressure and the reaction crude was then dissolved in $CDCl_3$ and directly analyzed via 1H NMR for yield and diastereoselectivity determination. From this solution, 0.05 mL was sampled into HPLC vials. The solvent was again removed under high vacuum and the remaining crude was diluted with 1 mL of isopropanol and analyzed via chiral-HPLC for determination of enantioselectivity - Conditions: Chiralpak IC, Eluent: 10% IPA in Hexane; Flow rate: 1 ml/min. 254 nm.

GP2 – Subsets: Chloroform, Hexane

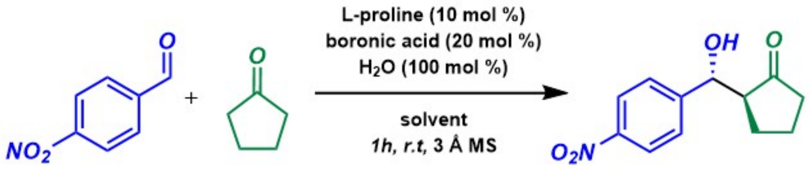

For the subsets in chloroform and hexane, due to the low water solubility in this solvents, water was added to each individual reaction instead of being used as a component in the solvent stock solutions.

A 5 mL vial, containing 5.8 g of Proline (0.05 mmol) and a stirring bar was placed under argon (with a balloon). To this flask, 25 mg of activated molecular sieves (3 Å) and a boroxine derivative were also added (0.0333 mmol). Next, 0.8 mL of a stock solution of internal standard 1,3,5-trimethoxybenzene (25 mg/mL, 0.149 M) in 37.5% V of cyclopentanone and 62.5% V of a given solvent was also added to the solids in the vial. To this mixture, 9 µL of water (0.5 mmol) was added. The mixture was stirred for 15 min at ambient temperature followed by addition of aldehyde (0.5 mmol). The reaction was then let to stir for 1 hour and after that quenched with a saturated solution of ammonium chloride. The organic phase was then reserved, and the water phase was extracted once again with chloroform. The organic phases were then combined and dried over magnesium sulfate. Lastly, solvent was removed under reduced pressure and the reaction crude was then dissolved in $CDCl_3$ and directly analyzed via 1H NMR for yield and diastereoselectivity determination. From this solution, 0.05 mL was sampled into HPLC

vials. The solvent was again removed under high vacuum and the remaining crude was diluted with 1 mL of isopropanol and analyzed via chiral-HPLC for determination of enantioselectivity - Conditions: Chiralpak IC, Eluent: 10% IPA in Hexane; Flow rate: 1 ml/min. 254 nm.

GP3 – Single reactions

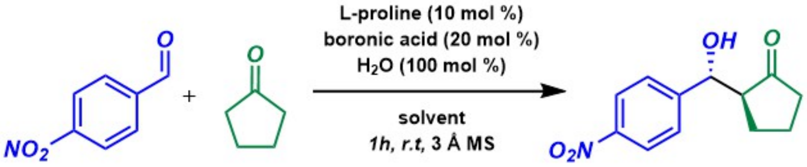

Unlike the reaction in the sets, blank reactions, reaction for studying the stoichiometry of ketone and reactions with methyl prolinate were not performed in batch. They always followed the procedure below. For blank reactions, one of the reaction components are left out, as mentioned accordingly in the Tables:

Proline (0.05 mmol, 5.8 mg), boronic acid (0.0333 mmol), 1,3,5-trimethoxybenzene (20 mg, 0.119 mmol), 25 mg of molecular sieves 3 Å, 0.8 mL cyclopentanone and 9 µL H_2O were placed in a screw capped vial under argon. The mixture was stirred for 15 min at ambient temperature followed by addition of aldehyde (0.5 mmol). After completion of the reaction, the reaction mixture was treated with saturated aqueous ammonium chloride solution and the whole mixture was extracted 3 times with CHCl3. The organic layer was dried over sodium-sulfate. Lastly, solvent was removed under reduced pressure and the reaction crude was then dissolved in $CDCl_3$ and directly analyzed via 1H NMR for yield and diastereoselectivity determination. From this solution, 0.05 mL was sampled into HPLC vials. The solvent was again removed under high vacuum and the remaining crude was diluted with 1 mL of isopropanol and analyzed via chiral-HPLC for determination of enantioselectivity - Conditions: Chiralpak IC, Eluent: 10% IPA in Hexane; Flow rate: 1 ml/min. 254 nm.

GP4 – Synthesis of Boroxine[1]

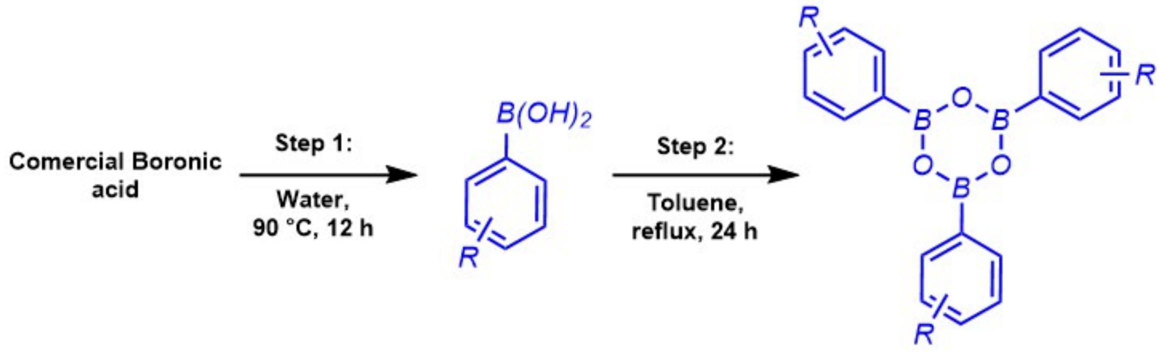

Step 1: Synthesis of Boronic acid

Commercial aryl boroxines are available as a mixture of boronic acid, its dimer and boroxine. To better control the addition of boronic acid to our reaction, we converted the purchased mixture completely to the respective boroxine. This was accomplished by first heating the mixture in water (distilled) for 12 h, at 90 ºC, (heat source: oil bath). This yielded the boronic acid as a solid after filtration. The solid was then brought into a sealed box (or a desiccator) containing Drierite and left to dry for 12 h. Next, the boronic acid was converted to boroxine, following the procedure presented in step 2.

Step 2: Boroxine synthesis

In a dry Dean-Stark apparatus equipped with a magnetic stir bar and under argon, the aryl boronic acid prepared in step 1 (16.4 mmol) and anhydrous toluene (70 mL) were added. The mixture was refluxed for 24 h and was allowed to cool to room temperature, followed by evaporation under reduced pressure to furnish boroxine.

3. Building the data set: outcomes of reactions in different solvents.

Table S1. Boronic acid screening and blanks in acetonitrile

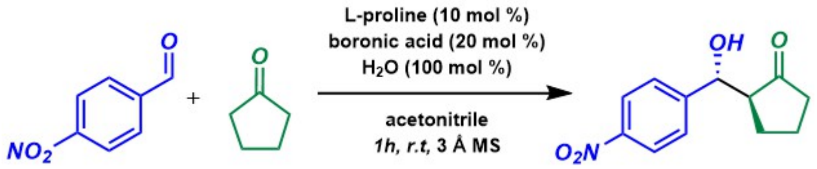

Phenylboronic acid (BA)	d.r. 1 [b]	d.r. 2 [b]	Av dr	Yield 1 % [c]	Yield 2 % [c]	Yield Av.	ee 1 % [d]	ee 2 % [d]	ee Av.	e.r Av.
**3-F-BA[a]**	11.9	12.5	12.2	76	87	81.5	94	96	95	97.5:2.5
**3,5-F-BA[a]**	9.6	9.8	9.7	40	41	40.5	96	96	96	98:2
**4-*t*-Bu-BA[a]**	6.6	6.1	6.35	93	98	95.5	96	94	95	97.5:2.5
**2,4-Me-BA[a]**	5.9	6	5.95	92	92	92	88	89	88.5	94.25:5.75
**3,5-OMe-BA[a]**	9.2	9.2	9.2	88	84	86	94	94	94	97:3
**4-Me-BA[a]**	6.5	6.7	6.6	98	98	98	90	90	90	95:5
**3-CF_3-BA[a]**	10.8	10	10.4	63	60	61.5	95	95	95	97.5:2.5
**2-Naph-BA[a]**	8	8.2	8.1	35	42	38.5	94	94	94	97:3
**4-CF_3-BA[a]**	12.6	11.9	12.25	50	57	53.5	97	96	96.5	98.25:1.75
**4-F-BA[a]**	9.4	9.9	9.65	87	92	89.5	92	93	92.5	96.25:3.75
**4-OMe-BA[a]**	5.7	6	5.85	96	97	96.5	86	88	87	93.5:6.5
**3-Me-BA[a]**	6.9	8	7.45	96	99	97.5	88	90	89	94.5:5.5
**2-Me-BA[a]**	7.2	7.5	7.35	90	96	93	88	90	89	94.5:5.5
**Ph-BA[a]**	8.5	9.2	8.85	89	90	89.5	92	92	92	96:4
**2-F-BA[a]**	19.2	19.2	19.2	18.3	18.3	18.3	92	94	93	96.5:3.5
**No BA [f]**	1	0.9	0.95	39	49	44	77	78	77.5	88.75:11.25
**No H_2O (2-F-BA) 20 hrs[g]**	4	4.1	4.05	15	17	16	86	86	86	93.7
**HFIP instead of water (2-F-BA) [h]**	N.D[h]	N.D[h]	N.D[h]	18	20	19	57	34	46	73:27
**MeOH instead of water (2-F-BA)[h]**	N.D[h]	N.D[h]	N.D[h]	No Conv	No Conv.	No Conv.	N.D[h]	N.D[h]	N.D[h]	N.D[h]

[a] Reactions were performed according to GP1; [b]D.R determined by 1H NMR by comparison of reference peak of anti (4.7-4.8ppm) and syn (4.3-4.4 ppm) products; [c]Yield determined by ^1H NMR using trimethoxybenzene (reference peak: ~6.1 ppm) as internal standard; [d]ee determined via chiral HPLC: IC, 10% IPA in Hexane, flowrate: 1 ml.min^{-1}; [e]not determined, another signal overlaps with the reference peak; [f]Reactions were performed according to GP 3, without addition of boronic acid [g] GP3, without the addition of water and for 20 hours [h] N.D = Not determined – due to low yield, determination of the d.r. using NMR became untrustworthy.

Table S2. Boronic acid screening and blanks in chloroform

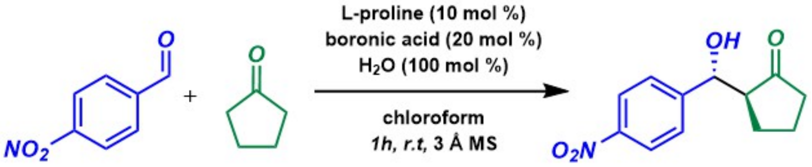

Phenylboronic acid (BA)	d.r. 1 [b]	d.r. 2 [b]	Av dr	Yield 1 % [c]	Yield 2 % [c]	Yield Av.	ee 1 % [d]	ee 2 % [d]	ee Av.	e.r Av.
**3-F-BA**	10.5	13.1	11.8	90	93	91.5	93	93	93	96.5:3.5
**3,5-F-BA**	28.3	29.5	28.9	53	49	51	96	96	96	98:2
**4-*t*-Bu-BA**	6.8	7.1	6.95	92	91	91.5	86	86	86	93:7
**2,4-Me-BA**	6.2	5.7	5.95	89	89	89	86	86	86	93:7
**3,5-OMe-BA**	8.8	8.9	8.85	88	90	89	90	90	90	95:5
**4-Me-BA**	6.48	6.6	6.54	92	85	88.5	84	84	84	92:8
**3-CF_3-BA**	11.2	11.2	11.2	88	91	89.5	93	94	93.5	96.75:3.25
**2-Naph-BA**	7.1	7.2	7.15	93	89	91	88	89	88.5	94.25:5.75
**4-CF_3-BA**	12.2	13	12.6	70	71	70.5	94	94	94	97:3
**4-F-BA**	7.4	7.6	7.5	84	88	86	90	91	90.5	95.25:4.75
**4-OMe-BA**	5.4	5.8	5.6	90	90	90	85	85	85	92.5:7.5
**3-Me-BA**	6.2	6.7	6.45	86	90	88	84	86	85	92.5:7.5
**2-Me-BA**	6.8	6.7	6.75	92	92	92	86	87	86.5	93.25:6.75
**Ph-BA**	7.3	7.6	7.45	93	92	92.5	87	88	87.5	93.75:6.25
**2-F-BA**	16.2	16.3	16.3	25	32	29	89	90	89.5	94.75:5.25
**No BA [f]**	3.7	3.7	3.7	43	55	49	78	79	78.5	89.25:10.75
**No H_2O (2-F-BA) 20 hrs[g]**	N.D[h]	N.D[h]	N.D[h]	13	13	13	84	85	84.5	92.25:7.75

[a]Reactions were performed according to GP2; [b]D.R determined by 1H NMR by comparison of reference peak of anti (4.7-4.8ppm) and syn (4.3-4.4 ppm) products; [c]Yield determined by ^{1}H NMR using trimethoxybenzene (reference peak: ~6.1 ppm) as internal standard. [d]ee determined via chiral HPLC: IC, 10% IPA in Hexane, flowrate: 1 ml.min^{-1}; [e]not determined, another signal overlaps with the reference peak; [f]Reactions were performed according to GP 3, without addition of boronic acid; [g]GP3, without the addition of water and for 20 hour; [h] N.D = Not determined – due to low yield, determination of the d.r. using NMR became untrustworthy.

Table S3. Boronic acid screening and blanks in neat cyclopentanone

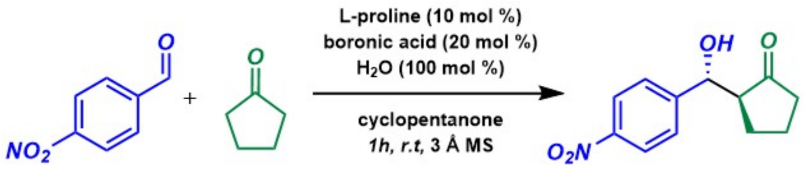

Phenylboronic acid (BA)	d.r. 1 [b]	d.r. 2 [b]	Av dr	Yield 1 % [c]	Yield 2 % [c]	Yield Av.	ee 1 % [d]	ee 2 % [d]	ee Av.	e.r Av.
**3-F-BA**	10.1	10.8	11.5	78	81	80	96	96	96	98:2
**3,5-F-BA**	11.3	9.9	10.6	89	88	89	98	97	98	99:1
**4-*t*-Bu-BA**	3.6	3.4	3.5	93	89	91	90	90	90	95:5
**2,4-Me-BA**	3.3	3.4	3.4	90	91	91	92	90	91	95.5:4.5
**3,5-OMe-BA**	6.4	6.3	6.4	90	95	93	92	92	92	96:4
**4-Me-BA**	3.6	3.9	3.8	76	76	76	90	90	90	95:5
**3-CF_3-BA**	9.9	10.4	10.2	73	78	76	96	96	96	98:2
**2-Naph-BA**	6.1	6.2	6.2	94	98	96	92	92	92	96:4
**4-CF_3-BA**	3.3	3.8	3.6	70	76	73	92	92	92	96:4
**4-F-BA**	6.6	6.9	6.8	76	78	77	94	94	94	97:3
**4-OMe-BA**	3.2	3.3	3.3	71	72	72	90	90	90	95:5
**3-Me-BA**	4.7	5.6	5.2	88	87	87	90	90	90	95:5
**2-Me-BA**	4.4	4.6	4.5	89	90	90	98	98	98	99:1
**Ph-BA**	5.5	5.7	5.6	73	73	73	92	92	92	96:4
**2F-BA**	10.6	12.8	11.7	98	99	99	96	96	96	98:2
**No BA [f]**	0.7	0.6	0.65	36	39	37.5	35	37	36	68:32
**No H_2O (2-F-BA) 20 hrs[g]**	5.1	5.3	5.2	29	32	30.5	89	90	90	95:5

[a]Reactions were performed according to GP1; [b]D.R determined by 1H NMR by comparison of reference peak of anti (4.7-4.8ppm) and syn (4.3-4.4 ppm) products; [c]Yield determined by ^{1}H NMR using trimethoxybenzene (reference peak: ~6.1 ppm) as internal standard. [d]ee determined via chiral HPLC: IC, 10% IPA in Hexane, flowrate: 1 ml.min^{-1}; [e]not determined, another signal overlaps with the reference peak; [f]Reactions were performed according to GP 3, without addition of boronic acid; [g]GP3, without the addition of water and for 20 hour.

Table S4. Boronic acid screening and blanks in MeOH

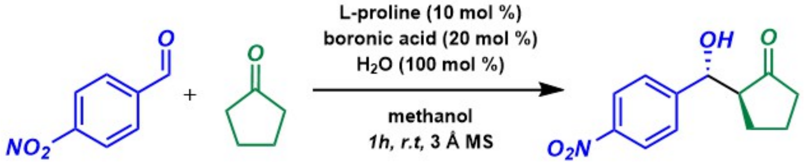

Phenylboronic acid (BA)	d.r.1 [b]	d.r.2 [b]	Av dr	Yield 1 % [c]	Yield 2 % [c]	Yield Av.	ee 1 % [d]	ee 2 % [d]	ee Av.	e.r Av.
**3-F-BA**	6	6.1	6.05	59	62	60.5	22	22	22	61:39
**3,5-F-BA**	5.2	5.1	5.15	40	42	41	48	50	49	74.5:25.5
**4-*t*-Bu-BA**	4.5	5.7	5.1	94	86	90	9	11	10	55:45
**2,4-Me-BA**	3.3	3.2	3.25	64	69	66.5	34	36	35	67.5:32.5
**3,5-OMe-BA**	6.9	7	6.95	80	82	81	11	11	11	55.5:42.5
**4-Me-BA**	6.6	6.9	6.75	79	82	80.5	8	16	12	56:44
**3-CF_3-BA**	4.3	4.9	4.6	72	71	71.5	32	32	32	66:34
**2-Naph-BA**	7.6	6.5	7.05	55	68	61.5	12	12	12	56:44
**4-CF_3-BA**	2.1	2.2	2.15	73	90	81.5	32	33	32.5	66.25:33.75
**4-F-BA**	6.7	6.7	6.7	69	70	69.5	12	13	12.5	56.25:43.75
**4-OMe-BA**	4.6	5	4.8	89	100	94.5	8	11	9.5	54.75:45.25
**3-Me-BA**	6.9	7	6.95	73	78	75.5	8	10	9	54.5:45.5
**2-Me-BA**	3.2	3	3.1	87	100	93.5	34	35	34.5	67.25:32.75
**Ph-BA**	6.7	7	6.85	75	78	76.5	10	10	10	55:45
**2-F-BA**	5.5	5.5	5.5	57	70	63.5	20	20	20	60:40
**No BA [f]**	3.1	3.4	3.25	99	98	98.5	79	77	78	89:11
**No H_2O (2-F-BA) 20 hrs[g]**	4	4.1	4.05	80	82	81	26	26	26	63:37
**No Proline, No BA**	1.3	1.5	1.4	35	39	37	--	--	--	--

[a]Reactions were performed according to GP1; [b]D.R determined by 1H NMR by comparison of reference peak of anti (4.7-4.8ppm) and syn (4.3-4.4 ppm) products; [c]Yield determined by ^{1}H NMR using trimethoxybenzene (reference peak: ~6.1 ppm) as internal standard. [d]ee determined via chiral HPLC: IC, 10% IPA in Hexane, flowrate: 1 ml.min^{-1}; [e]not determined, another signal overlaps with the reference peak; [f]Reactions were performed according to GP 3, without addition of boronic acid; [g]GP3, without the addition of water and for 20 hour.

Table S5. Boronic acid screening and blanks in hexane

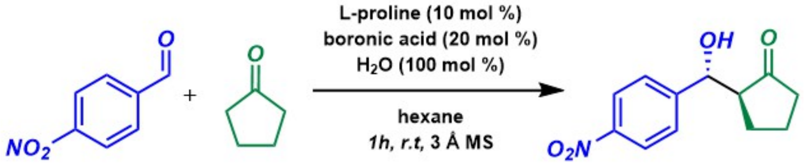

Phenylboronic acid (BA)	d.r.1 [b]	d.r.2 [b]	Av dr	Yield 1 % [c]	Yield 2 % [c]	Yield Av.	ee 1 % [d]	ee 2 % [d]	ee Av.	e.r Av.
**3-F-BA**	4.7	5.1	4.9	73.7	81.1	77.4	63	67	65	82.5:17.5
**3,5-F-BA**	11.62	12.5	12.06	29.3	27.6	28.45	90	91	90.5	95.25:4.75
**4-*t*-Bu-BA**	3.1	3.4	3.25	75.6	75.9	75.75	48	48	48	74:26
**2,4-Me-BA**	3.7	3.7	3.7	74.7	77.1	75.9	56	56	56	78:22
**3,5-OMe-BA**	3.8	4.1	3.95	76.8	80.1	78.45	48	52	50	75:25
**4-Me-BA**	3	2.5	2.75	74.2	77	76	41	40	40.5	60.25:37.75
**3-CF_3-BA**	6.2	6.3	6.25	76.8	80.6	78.7	74	75	74.5	87.25:12.75
**2-Naph-BA**	4.1	4.4	4.25	79	75.9	77.45	49	56	52.5	76.25:23.75
**4-CF_3-BA**	1.9	1.9	1.9	77.3	78	77.65	52	76	64	82:18
**4-F-BA**	3.9	4.4	4.15	77.3	79.9	78.6	59	61	60	80:20
**4-OMe-BA**	3	3.2	3.1	79.2	78.5	78.85	44	40	42	71:29
**3-Me-BA**	3.1	3.2	3.15	73.5	74.4	73.95	40	40	40	70:30
**2-Me-BA**	3.2	3.8	3.5	76.1	77.5	76.8	58	58	58	79:21
**Ph-BA**	3.4	3.4	3.4	71.8	78	74.9	45	46	45.5	72.75:27.25
**2-F-BA**	3.72	3.9	3.81	67.1	78.2	72.65	65	67	66	83:17
**No boronic acid [f]**	0.5	0.5	0.5	79	80	79.5	28	30	29	64.5:35.5
**No H_2O (2-F-BA) 20 hrs[g]**	N.D[h]	N.D[h]	N.D[h]	13	13	13	88	88	88	94:6

[a]Reactions were performed according to GP2; [b]D.R determined by 1H NMR by comparison of reference peak of anti (4.7-4.8ppm) and syn (4.3-4.4 ppm) products; [c]Yield determined by ^{1}H NMR using trimethoxybenzene (reference peak: ~6.1 ppm) as internal standard. [d]ee determined via chiral HPLC: IC, 10% IPA in Hexane, flowrate: 1 ml.min^{-1}; [e]not determined, another signal overlaps with the reference peak; [f]Reactions were performed according to GP 3, without addition of boronic acid; [g]GP3, without the addition of water and for 20 hour; [h] N.D = Not determined - – due to low yield, determination of the d.r. using NMR became untrustworthy.

4. Reaction optimization: Reducing the amount of ketone

Table S6. Reducing the amount of ketone in chloroform with 3,5-F-phenylboronic acid

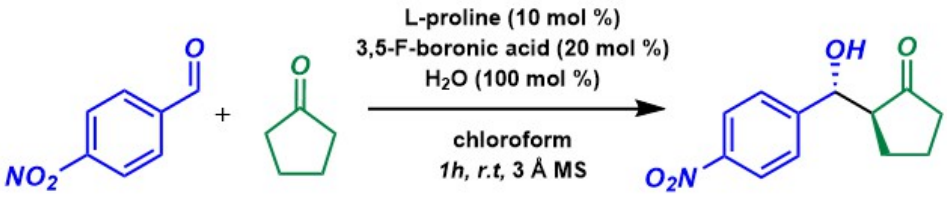

Ketone (eq)	Solvent (mL)	Time (h)	d.r. 1[b]	d.r. 2[b]	Av dr	Yield 1 [c] %	Yield 2 [c] %	Yield Av.	ee 1 [d] %	ee 2 [d] %	ee Av.	e.r. Av.
**6.8**	0.5	3	28	28	28	70	76	73	98	98	98	99:1
**6.8**	0.5	6	22	15	19	90	93	92	98	98	98	99:1
**5**	0.58	6	10	12	11	94	97	96	96	96	96	98:2
**3**	0.67	6	14	14	14	90	91	91	96	96	96	98:2
**2**	0.71	6	16	15	16	67	77	72	96	97	96.5	98.3:1.7
**1**	0.76	6	14	14	14	45	51	48	96	96	96	98:2
**5**	0.58	9	13	14	14	92	93	93	96	96	96	98:2
**3**	0.67	9	12	15	14	85	86	86	95	95	95	97.5:2.5
**2**	0.71	9	14	15	15	84	84	84	95	95	95	97.5:2.5
**1.5**	0.73	9	18	19	19	69	64	67	96	96	96	98:2
**1.2**	0.75	9	16	18	17	64	63	64	96	96	96	98:2
**1**	0.76	9	14	15	15	59	60	60	96	94	95	97.5:2.5

[a]Reactions were performed according to GP3; [b]D.R determined by 1H NMR by comparison of reference peak of anti (4.7-4.8ppm) and syn (4.3-4.4 ppm) products; [c]Yield determined by ^{1}H NMR using trimethoxybenzene (reference peak: ~6.1 ppm) as internal standard. [d]ee determined via chiral HPLC: IC, 10% IPA in Hexane, flowrate: 1 ml.min^{-1};

Table S7. Reducing the amount of ketone in chloroform with 3-CF_3-phenylboronic acid

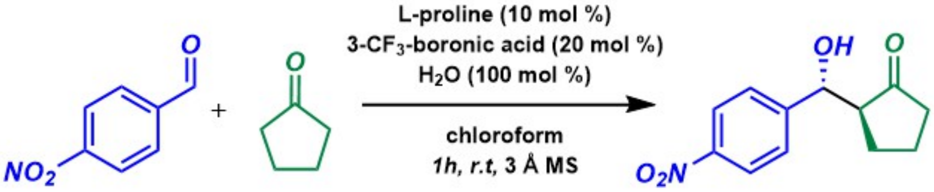

Ketone (eq)	Solvent (mL)	Time (h)	d.r. 1 [b]	d.r. 2 [b]	Av dr	Yield 1 [c] %	Yield 2 [c] %	Yield Av.	ee 1 [d] %	ee 2 [d] %	ee Av.	e.r.Av.
**2**	0.71	1	15	16	16	26	16	21	95	96	95.5	92.8:2.2
**2**	0.71	3	16	17	17	60	66	63	94	94	94	97:3
**2**	0.71	6	14	12	13	88	82	85	94	92	93	96.5:3.5
**2**	0.71	Over night	1.7	1.7	1.7	80	82	81	90	88	89	94.5:5.5

[a]Reactions were performed according to GP3; [b]D.R determined by 1H NMR by comparison of reference peak of anti (4.7-4.8ppm) and syn (4.3-4.4 ppm) products; [c]Yield determined by ^{1}H NMR using trimethoxybenzene (reference peak: ~6.1 ppm) as internal standard. [d]ee determined via chiral HPLC: IC, 10% IPA in Hexane, flowrate: 1 ml.min^{-1};

5. Probing boronic acid influence with methyl prolinate

Table S8. The effect of different boronic acids in the methyl prolinate catalyzed aldol reaction.

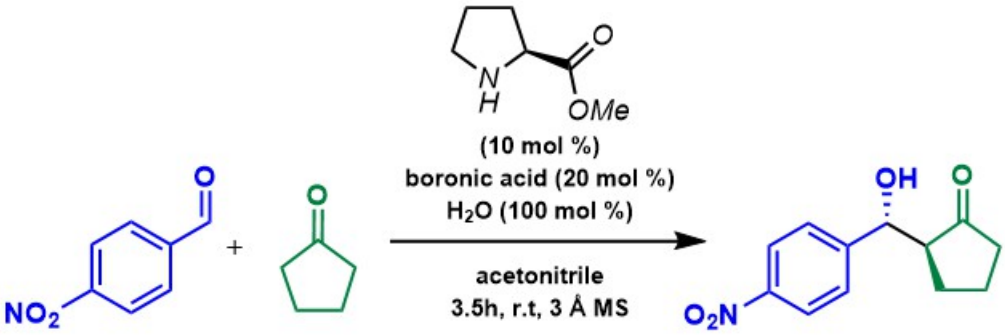

Phenylboronic acid (BA)	d.r. 1 [b]	d.r. 2 [b]	Av dr	Yield 1 [c] %	Yield 2 [c] %	Yield Av.	ee 1 [d] %	ee 2 [d] %	ee Av.	e.r. Av.
**Non**	1:2 (Syn)	1:2 (Syn)	1:2 (Syn)	70	69	70	69	70	69.5	84.8:15.2
**2-F-BA**	1:3 (anti)	1:3 (anti)	1:3 (anti)	72	87	80	46	46	46	73:27
**2,4-Me-BA**	1:5 (anti)	1:5 (anti)	1:5 (anti)	79	82	81	76	76	76	88:12
**3,5-OMe-BA**	1:4 (anti)	1:4 (anti)	1:4 (anti)	79	81	80	58	58	58	79:21

[a]Reactions were performed according to GP3; [b]D.R determined by 1H NMR by comparison of reference peak of anti (4.7-4.8ppm) and syn (4.3-4.4 ppm) products; [c]Yield determined by ^{1}H NMR using trimethoxybenzene (reference peak: ~6.1 ppm) as internal standard. [d]ee determined via chiral HPLC: IC, 10% IPA in Hexane, flowrate: 1 ml.min^{-1};

6. Characterization of Boroxines:

2,4-Me-phenylboroxine[11]

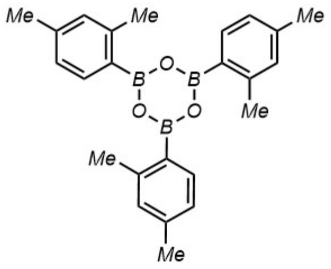

2,4-Me-phenylboroxine was synthesized according to GP4, using 2,4-Me-phenyboronic acid affording a gray powder with 89% yield;

^{1}H NMR (400 MHz, DMSO) δ 7.78 (d, *J* = 7.9 Hz, 1H), 6.94 (dd, *J* = 17.6, 6.7 Hz, 2H), 2.61 (s, 3H), 2.26 (s, 3H); Analytical data matches the reported data in literature.[11]

2-Naphtylboroxine[12]

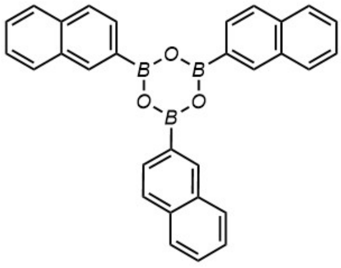

2-Naphtylboroxine was synthesized according to GP4, using 2-Naphtylboronic acid affording a white powder with 93% yield

^{1}H NMR (400 MHz, DMSO) δ 8.56 (s, 1H), 8.12 (d, *J* = 8.2 Hz, 1H), 8.08 – 8.02 (m, 1H), 7.94 (t, *J* = 8.4 Hz, 2H), 7.53 (dd, *J* = 5.8, 3.5 Hz, 2H). Analytical data matches the reported data in literature.[12]

3,5-OMe-phenylboroxine[13]

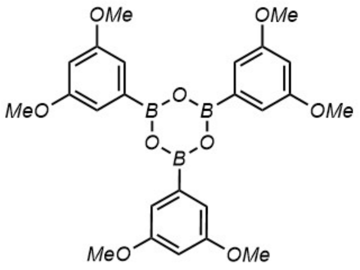

3,5-OMe-phenylboroxine was synthesized according to GP4, using 3,5-OMe-phenylboronic acid affording a gray crystalline with 90% yield

^{1}H NMR (400 MHz, DMSO) δ 7.00 (d, *J* = 2.1 Hz, 2H), 6.53 (s, 1H), 3.89 – 3.72 (m, 6H). Analytical data matches the reported data in literature[13].

3,5-F-phenylboroxine[14]

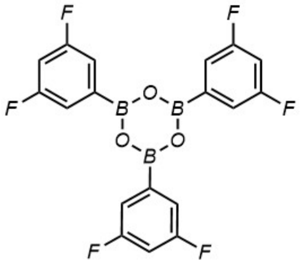

3,5-F-phenylboroxine was synthesized according to GP4, using 3,5-phenyboronic acid affording a white powder with 83% yield

^{1}H NMR (400 MHz, DMSO) δ 7.46 (d, *J* = 6.1 Hz, 2H), 7.25 – 7.11 (m, 1H). Analytical data matches the reported data in literature[14]

4-Me-phenylboroxine[15]

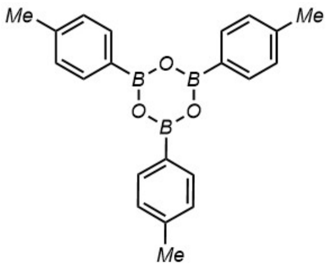

4-Me-phenylboroxine was synthesized according to GP4, using 4-Me-phenylboronic acid affording a white powder with 96% yield

^{1}H NMR (400 MHz, DMSO) δ 7.77 (d, *J* = 7.2 Hz, 2H), 7.18 (d, *J* = 7.3 Hz, 2H), 2.32 (s, 3H); Analytical data matches the reported data in literature[15]

4-OMe-phenylboroxine[15]

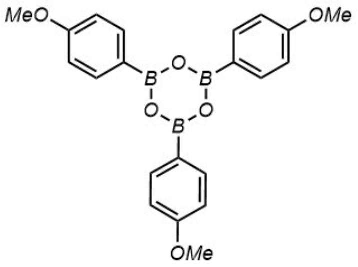

4-OMe-phenylboroxine was synthesized according to GP4, using 4-OMe-phenylboronic acid affording a gray powder with 86% yield

^{1}H NMR (400 MHz, DMSO) δ 7.81 (d, *J* = 8.5 Hz, 2H), 6.93 (d, *J* = 8.6 Hz, 2H), 3.77 (s, 3H). Analytical data matches the reported data in literature[15]

4-^{t}Bu-phenylboroxine[16]

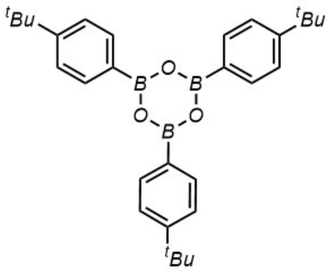

4-^{t}Bu-phenylboroxine was synthesized according to GP4, using 4-^{t}Bu-phenylboronic acid affording a pale gray powder with 92% yield

(400 MHz, DMSO) δ 7.79 (d, *J* = 6.9 Hz, 2H), 7.38 (d, *J* = 7.9 Hz, 2H), 1.29 (s, 9H); Analytical data matches the reported data in literature[16]

2-Me-phenylboroxine[15]

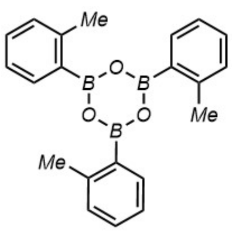

2-Me-phenylboroxine was synthesized according to GP4, using 2-Me-phenyl boronic acid affording a white powder with 86% yield

^{1}H NMR (500 MHz, DMSO) δ 7.88 (d, *J* = 6.6 Hz, 1H), 7.23 (d, *J* = 7.0 Hz, 1H), 7.14 (t, *J* = 7.6 Hz, 2H), 2.64 (s, 3H). Analytical data matches the reported data in literature[15]

2-F-phenylboroxine[17]

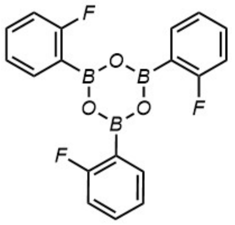

2-F-phenylboroxine was synthesized according to GP4, using 2-F-phenylboronic acid affording a white powder with 91% yield

^{1}H NMR (500 MHz, DMSO) δ 7.80 (s, 1H), 7.42 (d, *J* = 5.8 Hz, 1H), 7.17 (t, *J* = 6.9 Hz, 1H), 7.06 (t, *J* = 8.7 Hz, 1H). Analytical data matches the reported data in literature[17]

3-CF_3-phenylboroxine[18]

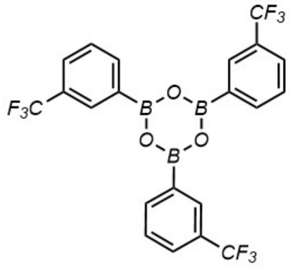

3-CF_3-phenylboroxine was synthesized according to GP4, using 3-CF_3-phenylboronic acid affording a white powder with 86% yield

^{1}H NMR (500 MHz, DMSO) δ 8.15 (d, J = 7.3 Hz, 1H), 8.10 (s, 1H), 7.76 (d, J = 7.6 Hz, 1H), 7.63 (t, J = 7.5 Hz, 1H). Analytical data matches the reported data in literature.[18]

3-F-phenylboroxine[19]

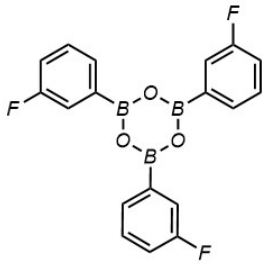

3-F-phenylboroxine was synthesized according to GP4, using 3-F-phenylboronic acid affording a white powder with 81% yield

^{1}H NMR (500 MHz, DMSO) δ 7.69 (d, J = 7.2 Hz, 1H), 7.55 (d, J = 9.5 Hz, 1H), 7.42 (dd, J = 13.6, 6.6 Hz, 1H), 7.20 (t, J = 8.6 Hz, 1H); Analytical data matches the reported data in literature.[19]

3-Me-phenylboroxine[12]

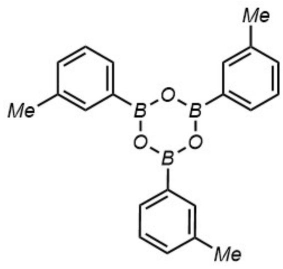

3-Me-phenylboroxine was synthesized according to GP4, using 3-Me-phenylboronic acid affording a white powder with 92% yield

^{1}H NMR (500 MHz, DMSO) δ 7.66 (d, J = 6.0 Hz, 2H), 7.25 (t, J = 7.5 Hz, 1H), 7.19 (d, J = 7.4 Hz, 1H), 2.33 (s, 3H). Analytical data matches the reported data in literature.[12]

4-CF_3-phenylboroxine[20]

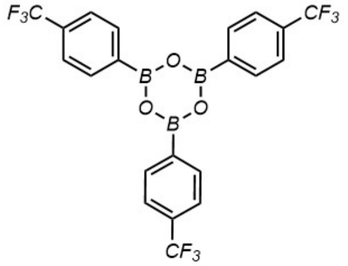

4-CF_3-phenylboroxine was synthesized according to GP4, using 4-CF_3-phenylboronic acid affording a white powder with 88% yield

^{1}H NMR (400 MHz, DMSO) δ 8.20 (d, J = 7.7 Hz, 2H), 7.85 (d, J = 7.9 Hz, 2H). Analytical data matches the reported data in literature.[20]

4-F-phenylboroxine[15]

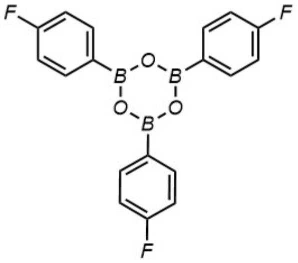

4-F-phenylboroxine was synthesized according to GP4, using 4-F-phenylboronic acid affording a white crystalline solid with 89% yield

^{1}H NMR (400 MHz, DMSO) δ 8.02 (t, J = 7.3 Hz, 2H), 7.29 (t, J = 9.0 Hz, 2H). Analytical data matches the reported data in literature[15]

Phenylboroxine[15]

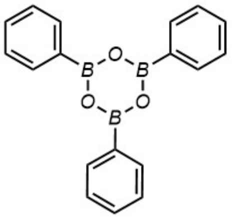

Phenylboroxine was synthesized according to GP4, using phenylboronic acid affording a white powder with 97% yield

^{1}H NMR (500 MHz, DMSO) δ 7.88 (d, J = 6.8 Hz, 2H), 7.37 (d, J = 6.5 Hz, 3H). Analytical data matches the reported data in literature[15]

7. Characterization of (S)-2-((R)-hydroxy(4-nitrophenyl)methyl)cyclopentan-1-one:

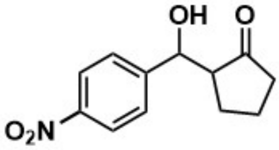

(S)-2-((R)-hydroxy(4-nitrophenyl)methyl)cyclopentan-1-one was synthesized according to GP3, using cyclopentanone as solvent and 2-F-phenylboroxine as boronic acid. Purification was accomplished by flash column chromatography (Silica gel, Hexane: EtOAc (70:30)) affording a colorless oil with 98% yield (117 mg, 0.5 mmol); 1H NMR (400 MHz, $CDCl_3$) δ 8.15 (d, J = 8.6 Hz, 2H), 7.47 (d, J = 8.7 Hz, 2H), 4.78 (d, J = 9.2 Hz, 1H), 4.67 (s, 1H), 2.47 – 2.15 (m, 2H), 2.01 – 1.90 (m, 1H), 1.72 – 1.60 (m, 2H), 1.48 (q, J = 6.7 Hz, 2H). Absolute configuration was determined by comparison to correspondent analytical data reported in literature.[1-3]

HPLC Traces

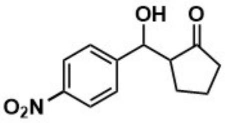

HPLC traces for of Isolated (S)-2-((R)-hydroxy(4-nitrophenyl)methyl)cyclopentan-1-one: Conditions: Chiralpak IC, Eluent: 10% IPA in Hexane; Flow rate: 1 ml/min. 254 nm.

A – Racemic

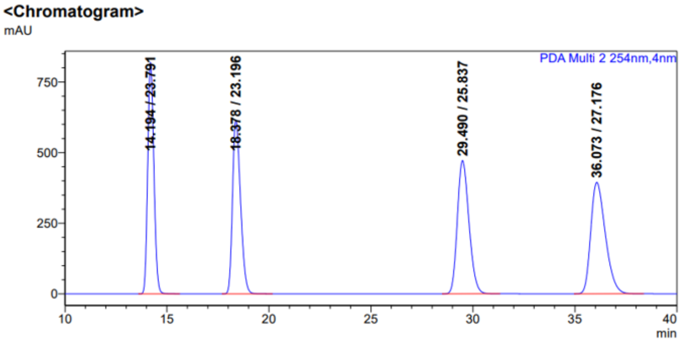

PDA Ch2 254nm

Peak#	Name	Ret. Time	Area	Area%
1		14.194	17211885	23.791
2		18.378	16781439	23.196
3		29.490	18691880	25.837
4		36.073	19660400	27.176
Total			72345604	100.000

B – Chiral

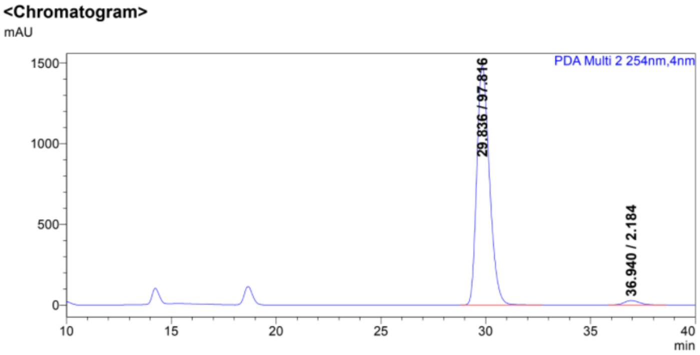

PDA Ch2 254nm

Peak#	Name	Ret. Time	Area	Area%
1		29.836	62096717	97.816
2		36.940	1386767	2.184
Total			63483484	100.000

8. HPLC Traces for Table S1. Boronic acid screening and blanks in acetonitrile

All of the reactions were performed as duplicate following GP1 with different boronic acids. A racemic sample was first analyzed in order to determine the retention time of both enantiomers (see above - page 14). For the chiral sample, enantiomeric excess was determined by comparing the integrated area of these two peaks. All the HPLC traces shown below are for of (S)-2-((R)-hydroxy(4-nitrophenyl)methyl)cyclopentan-1-one, yielded by different reactions as described in table S1.

HPLC traces for reaction with: **3-F-phenylboronic acid**

<Chromatogram>

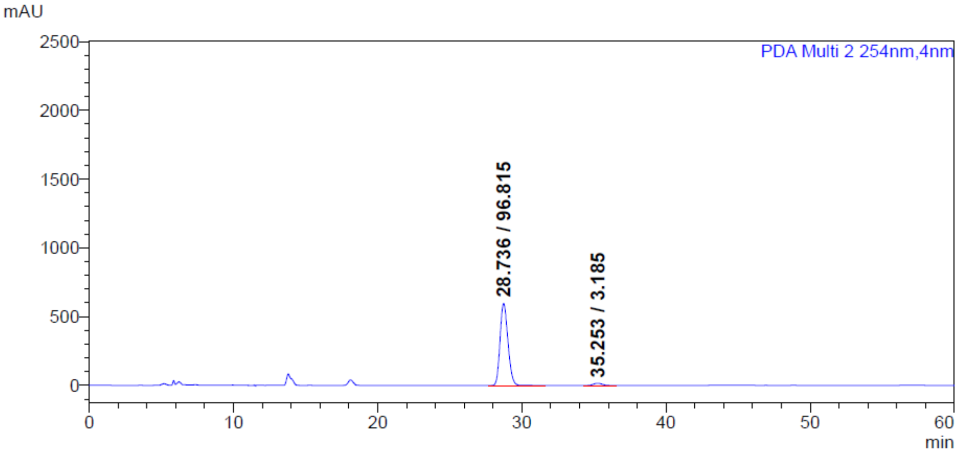

PDA Ch2 254nm

Peak#	Name	Ret. Time	Area	Area%
1		28.736	22793567	96.815
2		35.253	749809	3.185
Total			23543376	100.000

HPLC traces for reaction with: **3-F-phenylboronic acid (duplicate)**

<Chromatogram>

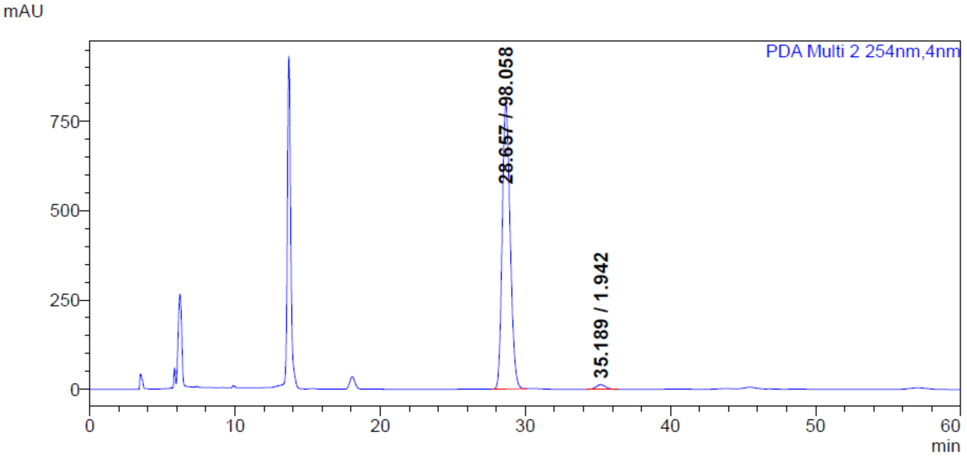

PDA Ch2 254nm

Peak#	Name	Ret. Time	Area	Area%
1		28.657	30387851	98.058
2		35.189	601832	1.942
Total			30989683	100.000

HPLC traces for reaction with: **3,5-F-phenylboronic acid**

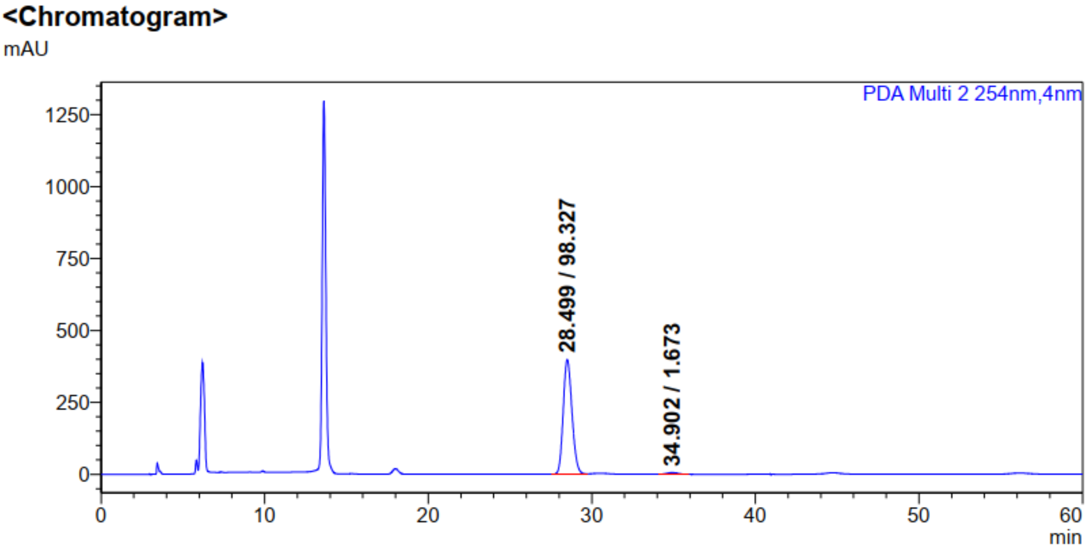

PDA Ch2 254nm

Peak#	Name	Ret. Time	Area	Area%
1		28.499	14885966	98.327
2		34.902	253298	1.673
Total			15139263	100.000

HPLC traces for reaction with: **3,5-F-phenylboronic acid (duplicate)**

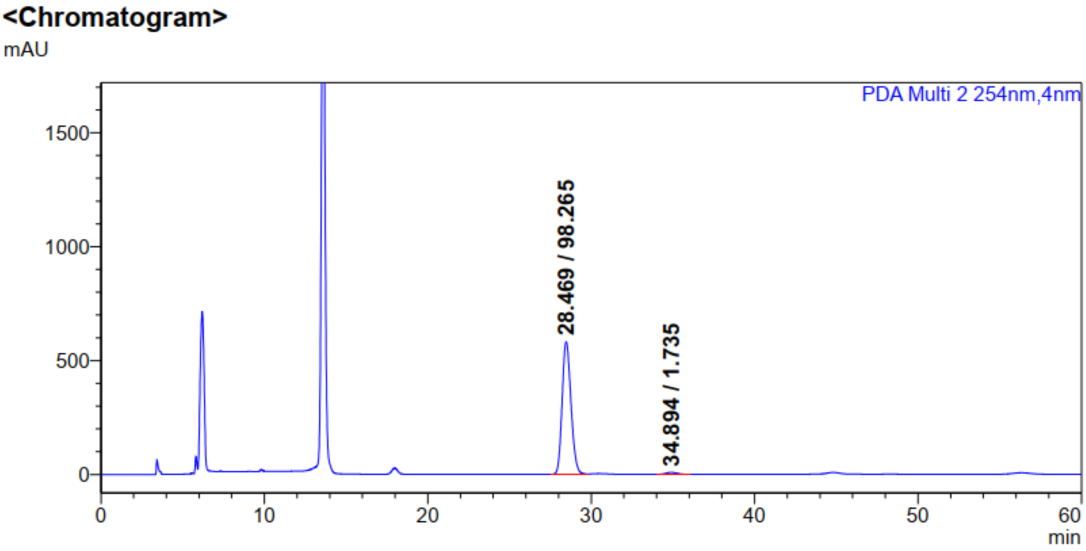

PDA Ch2 254nm

Peak#	Name	Ret. Time	Area	Area%
1		28.469	21909599	98.265
2		34.894	386841	1.735
Total			22296440	100.000

HPLC traces for reaction with: **4-tBu-phenylboronic acid**

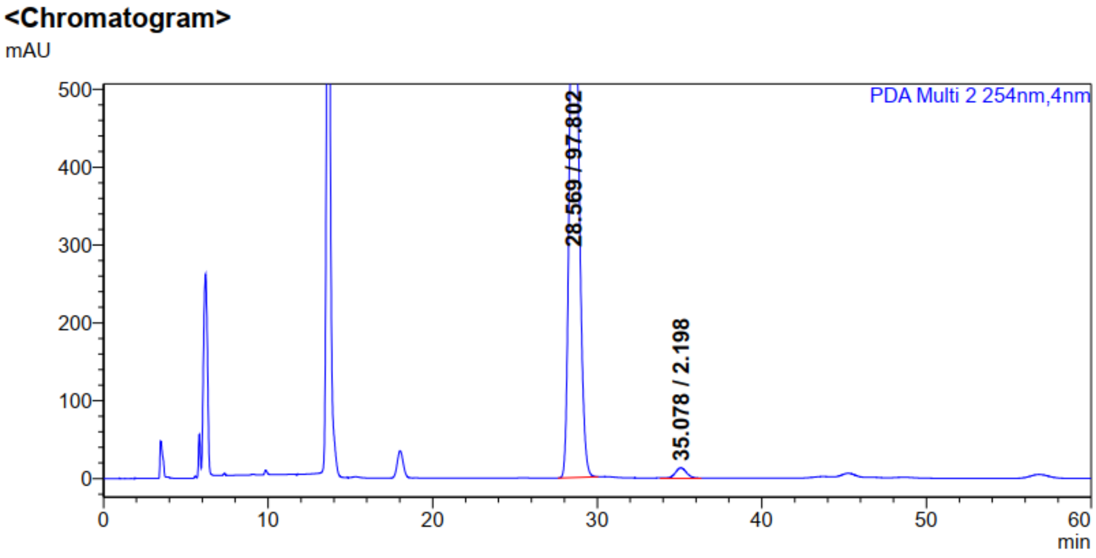

PDA Ch2 254nm

Peak#	Name	Ret. Time	Area	Area%
1		28.569	30405448	97.802
2		35.078	683439	2.198
Total			31088887	100.000

HPLC traces for reaction with: **4-tBu-phenylboronic acid (duplicate)**

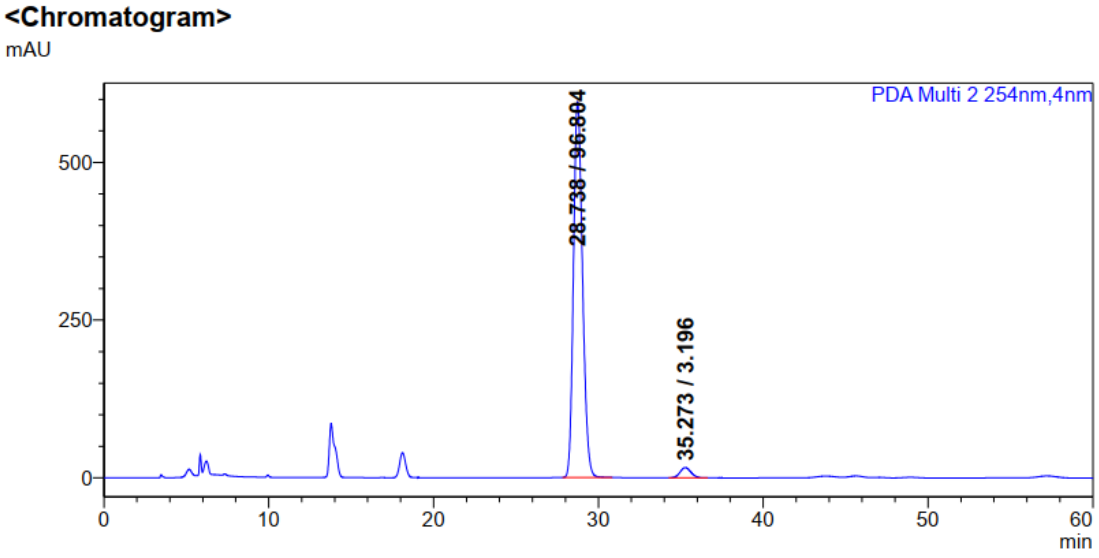

PDA Ch2 254nm

Peak#	Name	Ret. Time	Area	Area%
1		28.738	22692374	96.804
2		35.273	749278	3.196
Total			23441652	100.000

HPLC traces for reaction with: **2,4-Me-phenylboronic acid**

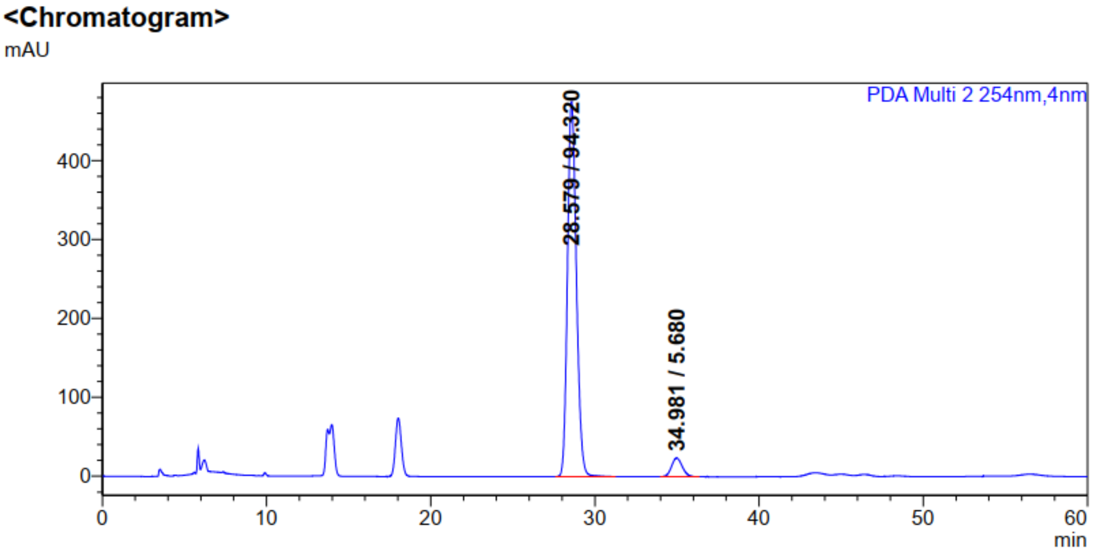

PDA Ch2 254nm

Peak#	Name	Ret. Time	Area	Area%
1		28.579	17884186	94.320
2		34.981	1076996	5.680
Total			18961181	100.000

HPLC traces for reaction with: **2,4–Me-phenylboronic acid (duplicate)**

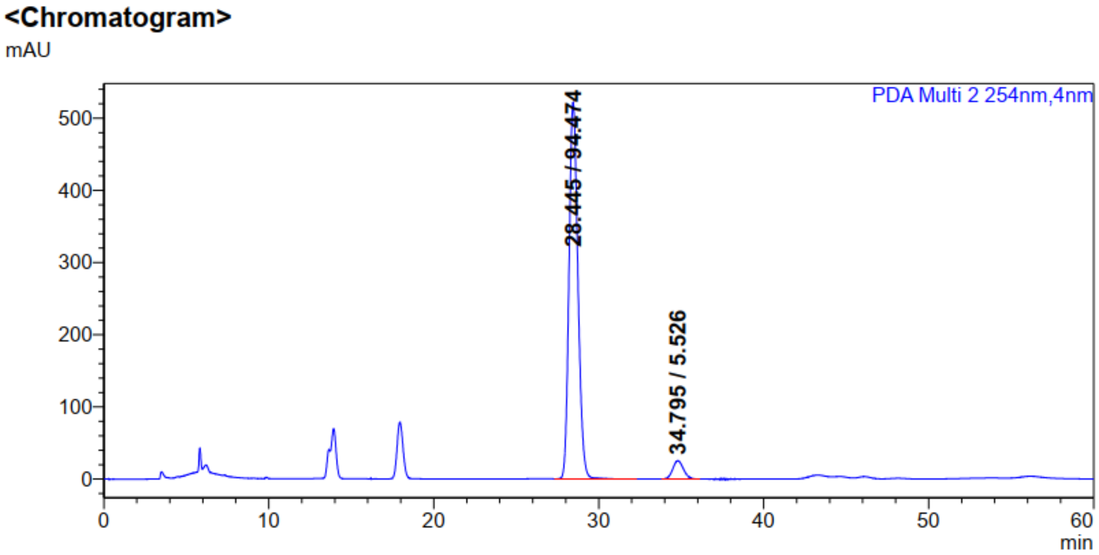

PDA Ch2 254nm

Peak#	Name	Ret. Time	Area	Area%
1		28.445	19609083	94.474
2		34.795	1147032	5.526
Total			20756115	100.000

HPLC traces for reaction with: **3,5-OMe-phenylboronic acid**

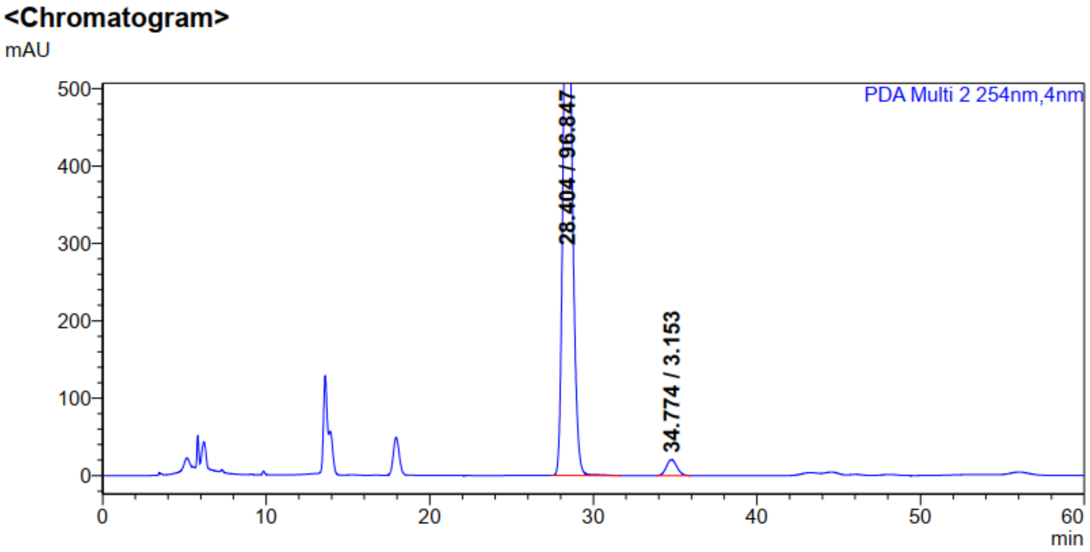

PDA Ch2 254nm

Peak#	Name	Ret. Time	Area	Area%
1		28.404	28359016	96.847
2		34.774	923308	3.153
Total			29282324	100.000

HPLC traces for reaction with: **3,5-OMe-phenylboronic acid (duplicate)**

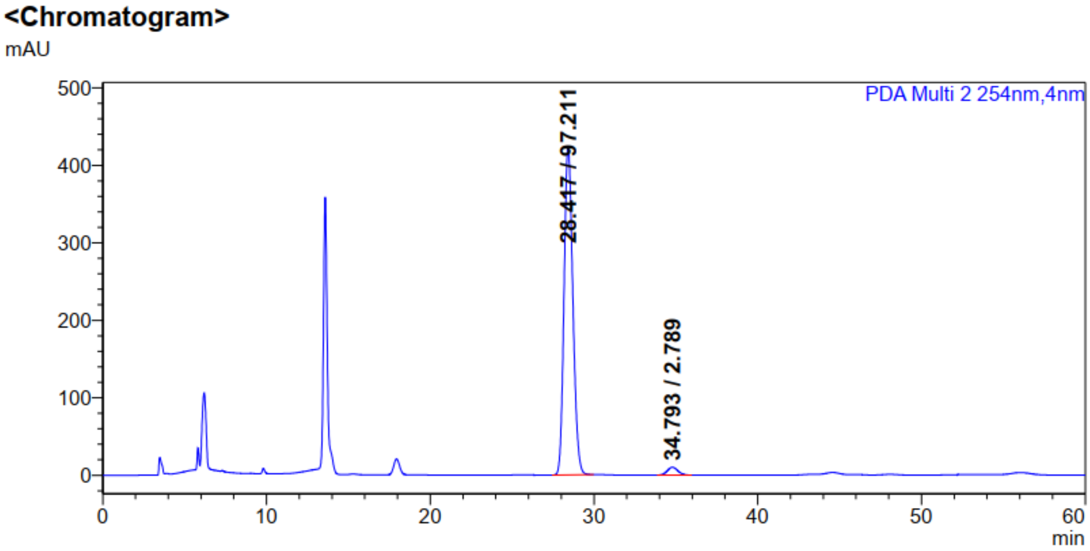

PDA Ch2 254nm

Peak#	Name	Ret. Time	Area	Area%
1		28.417	15781043	97.211
2		34.793	452820	2.789
Total			16233863	100.000

HPLC traces for reaction with: **4-Me-phenylboronic acid**

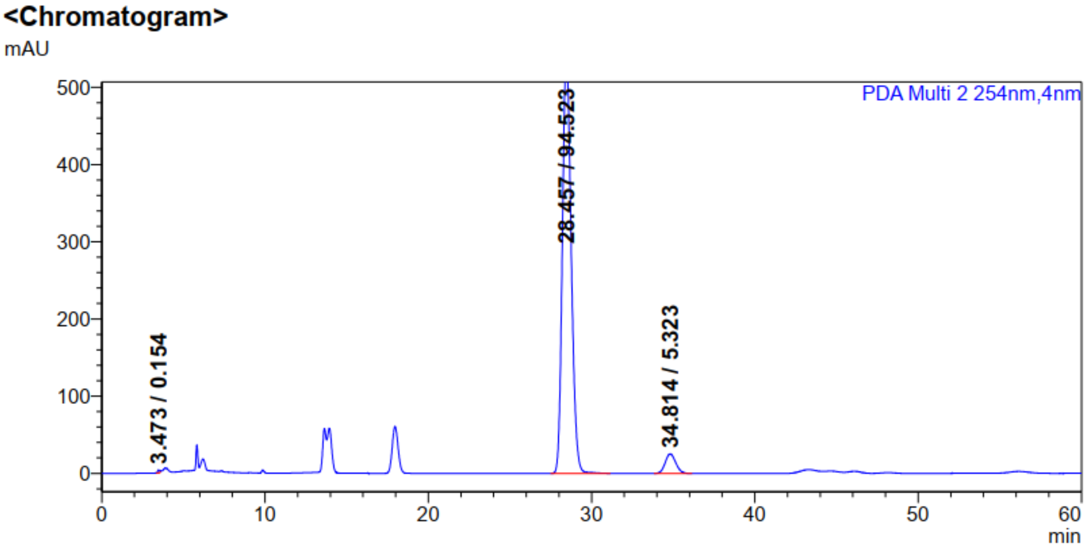

PDA Ch2 254nm

Peak#	Name	Ret. Time	Area	Area%
1		3.473	33267	0.154
2		28.457	20368923	94.523
3		34.814	1147025	5.323
Total			21549215	100.000

HPLC traces for reaction with: **4-Me-phenylboronic acid (duplicate)**

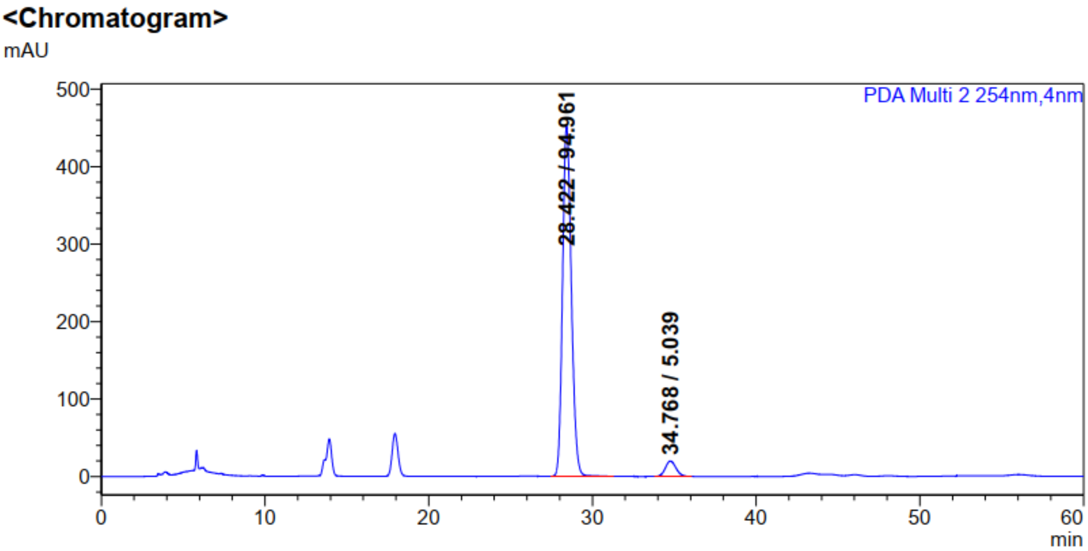

PDA Ch2 254nm

Peak#	Name	Ret. Time	Area	Area%
1		28.422	16873479	94.961
2		34.768	895447	5.039
Total			17768926	100.000

HPLC traces for reaction with: **3-CF_3-phenylboronic acid**

<Chromatogram>

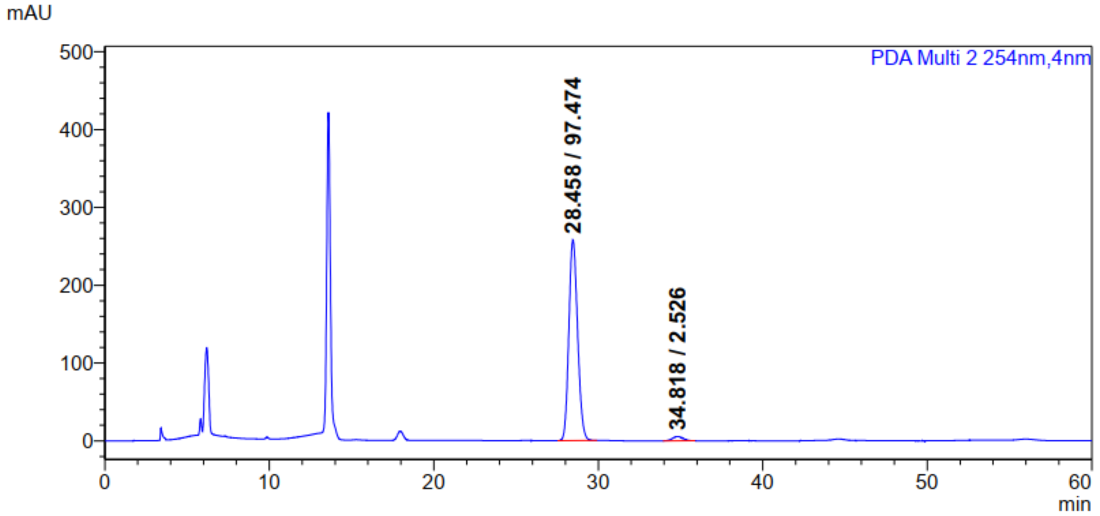

PDA Ch2 254nm

Peak#	Name	Ret. Time	Area	Area%
1		28.458	9540208	97.474
2		34.818	247239	2.526
Total			9787447	100.000

HPLC traces for reaction with: **3-CF_3-phenylboronic acid (duplicate)**

<Chromatogram>

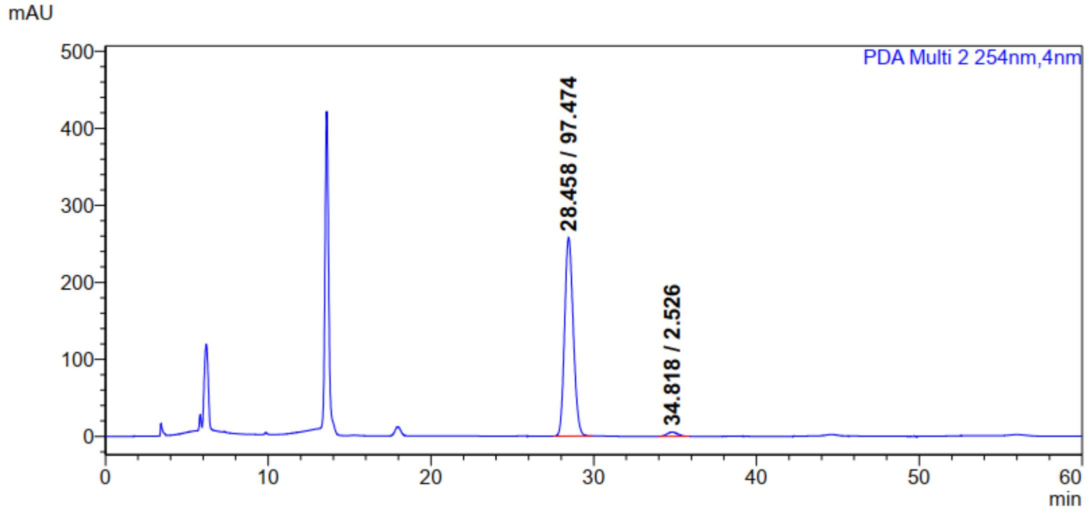

PDA Ch2 254nm

Peak#	Name	Ret. Time	Area	Area%
1		28.458	9540208	97.474
2		34.818	247239	2.526
Total			9787447	100.000

HPLC traces for reaction with: **2-Naphtalene-phenylboronic acid**

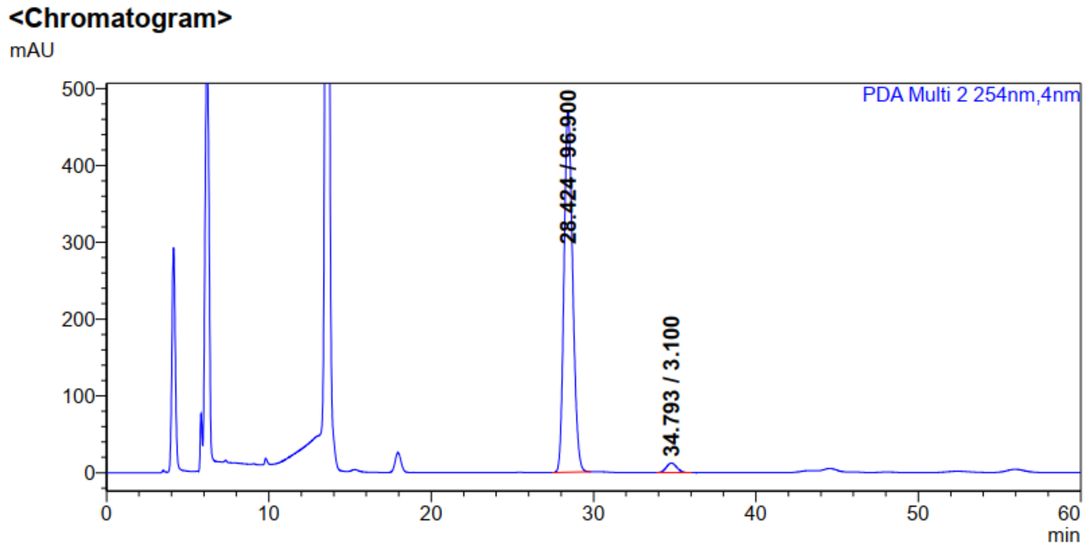

PDA Ch2 254nm

Peak#	Name	Ret. Time	Area	Area%
1		28.424	17495459	96.900
2		34.793	559694	3.100
Total			18055154	100.000

HPLC traces for reaction with: **2-Naphtalene-phenylboronic acid (duplicate)**

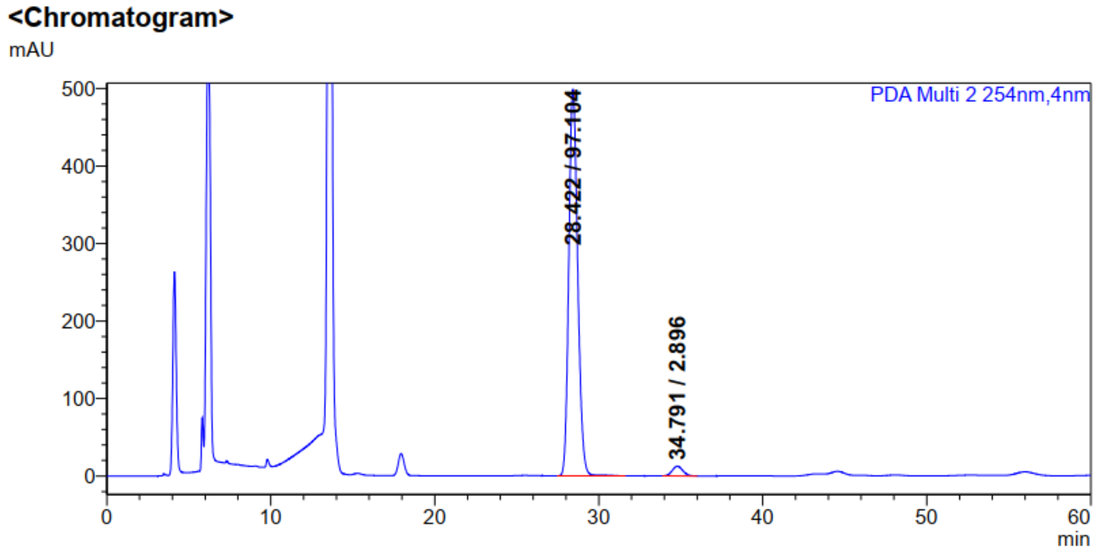

PDA Ch2 254nm

Peak#	Name	Ret. Time	Area	Area%
1		28.422	18759778	97.104
2		34.791	559483	2.896
Total			19319262	100.000

HPLC traces for reaction with: **4CF$_3$-phenylboronic acid**

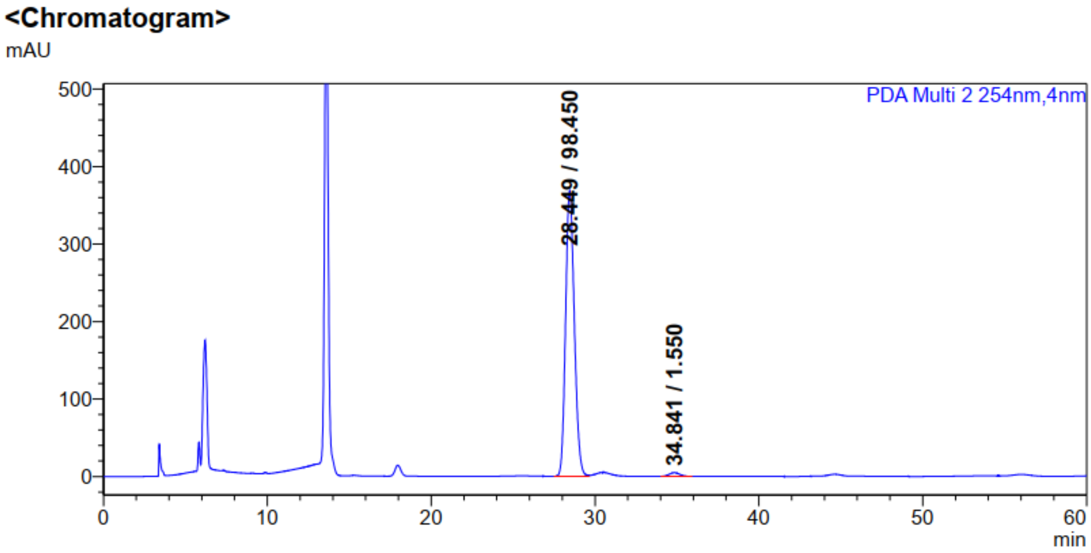

PDA Ch2 254nm

Peak#	Name	Ret. Time	Area	Area%
1		28.449	13692853	98.450
2		34.841	215532	1.550
Total			13908385	100.000

HPLC traces for reaction with: **4CF$_3$-phenylboronic acid (duplicate)**

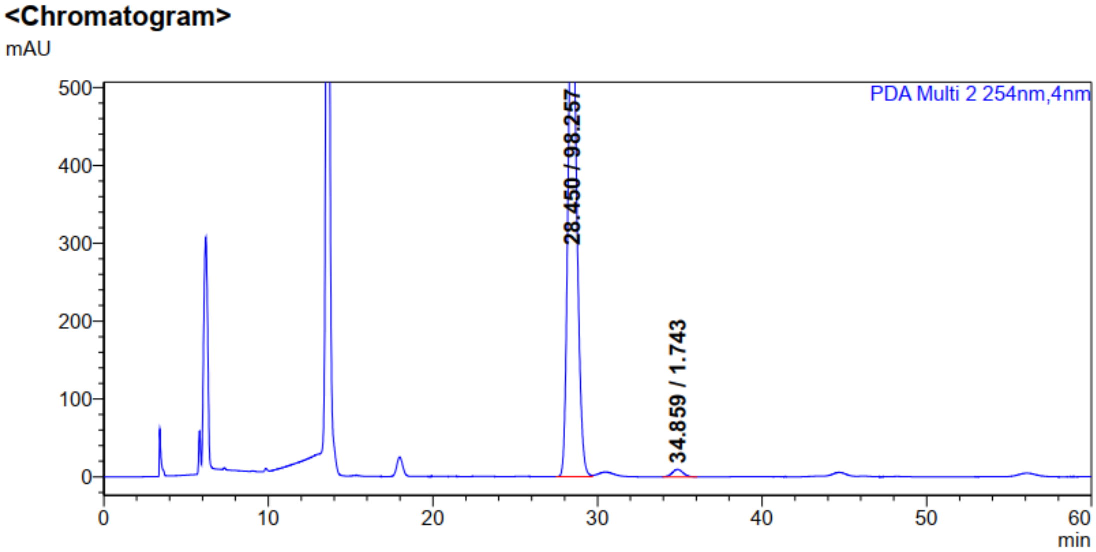

PDA Ch2 254nm

Peak#	Name	Ret. Time	Area	Area%
1		28.450	24149203	98.257
2		34.859	428348	1.743
Total			24577550	100.000

HPLC traces for reaction with: **4-F-phenylboronic acid**

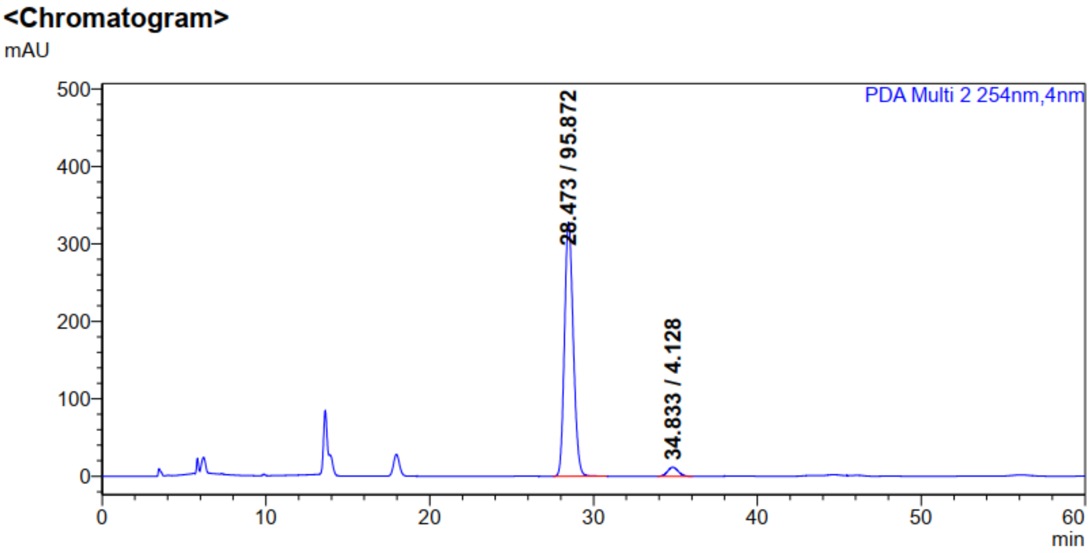

PDA Ch2 254nm

Peak#	Name	Ret. Time	Area	Area%
1		28.473	12221523	95.872
2		34.833	526186	4.128
Total			12747709	100.000

HPLC traces for reaction with: **4-F-phenylboronic acid (duplicate)**

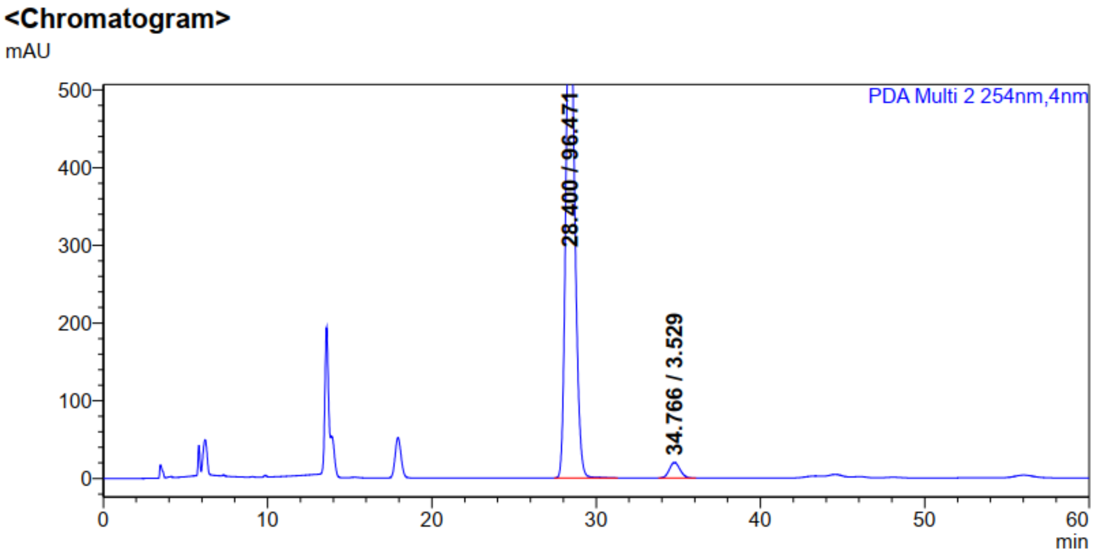

PDA Ch2 254nm

Peak#	Name	Ret. Time	Area	Area%
1		28.400	24653947	96.471
2		34.766	901971	3.529
Total			25555918	100.000

HPLC traces for reaction with: **4-OMe-phenylboronic acid**

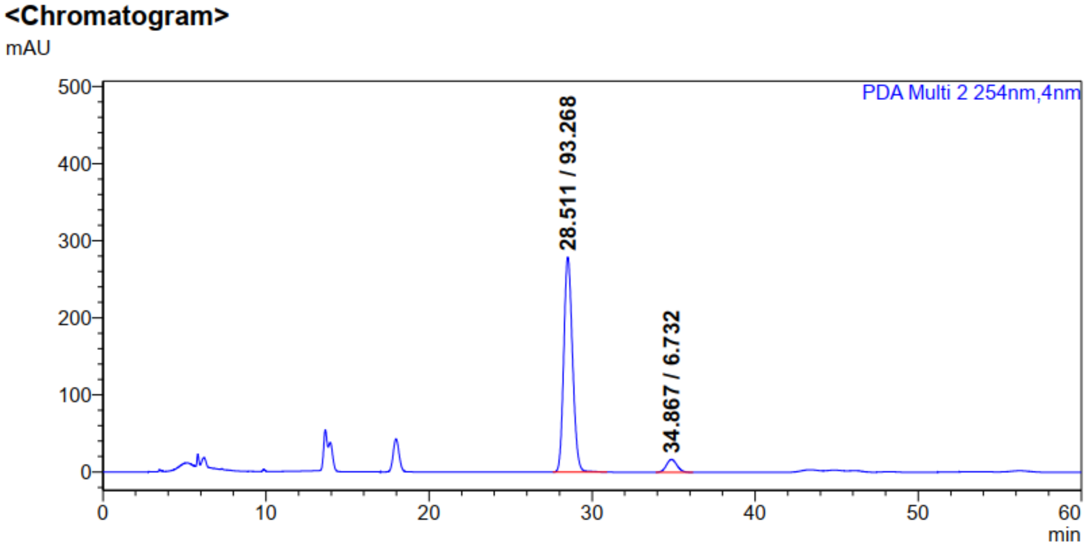

PDA Ch2 254nm

Peak#	Name	Ret. Time	Area	Area%
1		28.511	10424621	93.268
2		34.867	752407	6.732
Total			11177028	100.000

HPLC traces for reaction with: **4-OMe-phenylboronic acid (duplicate)**

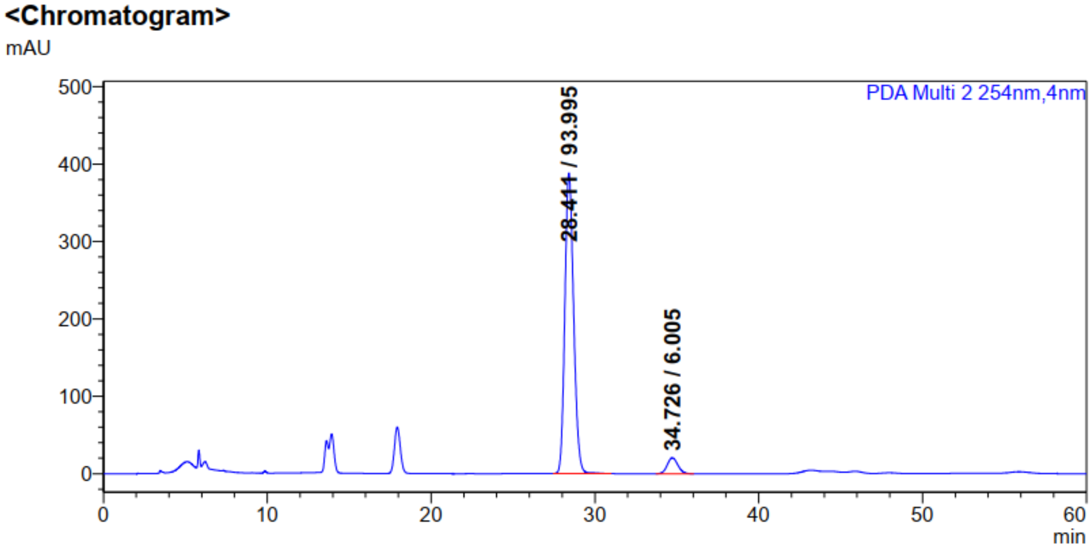

PDA Ch2 254nm

Peak#	Name	Ret. Time	Area	Area%
1		28.411	14448105	93.995
2		34.726	922994	6.005
Total			15371099	100.000

HPLC traces for reaction with: **3-Me-phenylboronic acid**

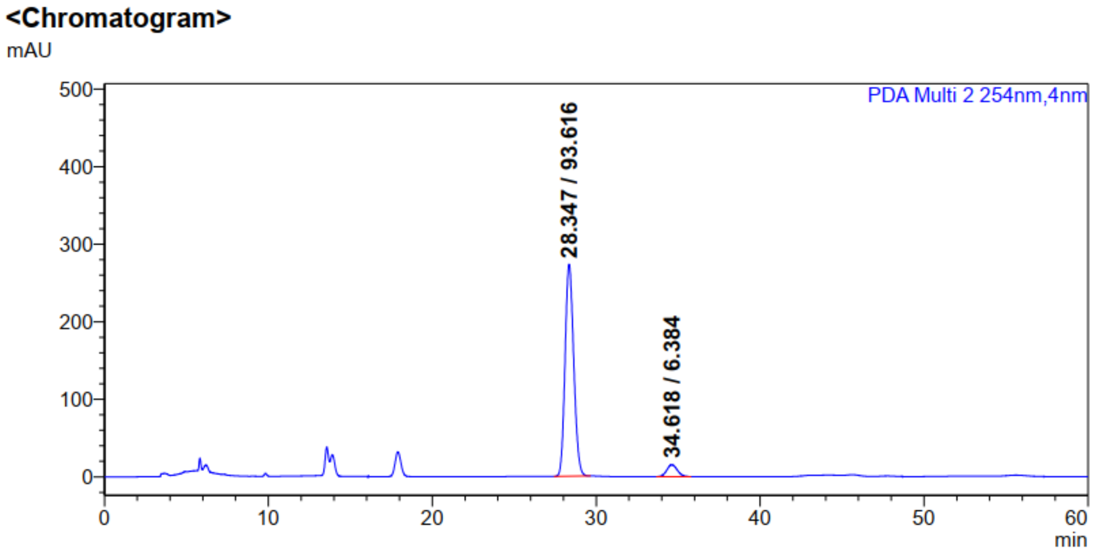

PDA Ch2 254nm

Peak#	Name	Ret. Time	Area	Area%
1		28.347	10029069	93.616
2		34.618	683886	6.384
Total			10712955	100.000

HPLC traces for reaction with: **3-Me-phenylboronic acid (duplicate)**

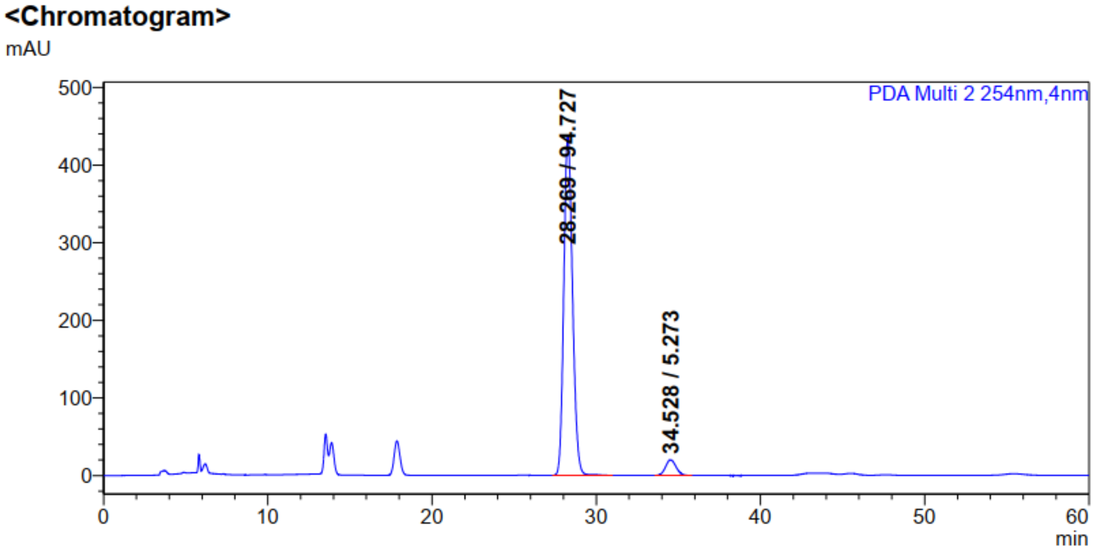

PDA Ch2 254nm

Peak#	Name	Ret. Time	Area	Area%
1		28.269	16166585	94.727
2		34.528	899838	5.273
Total			17066423	100.000

HPLC traces for reaction with: **2-Me-phenylboronic acid**

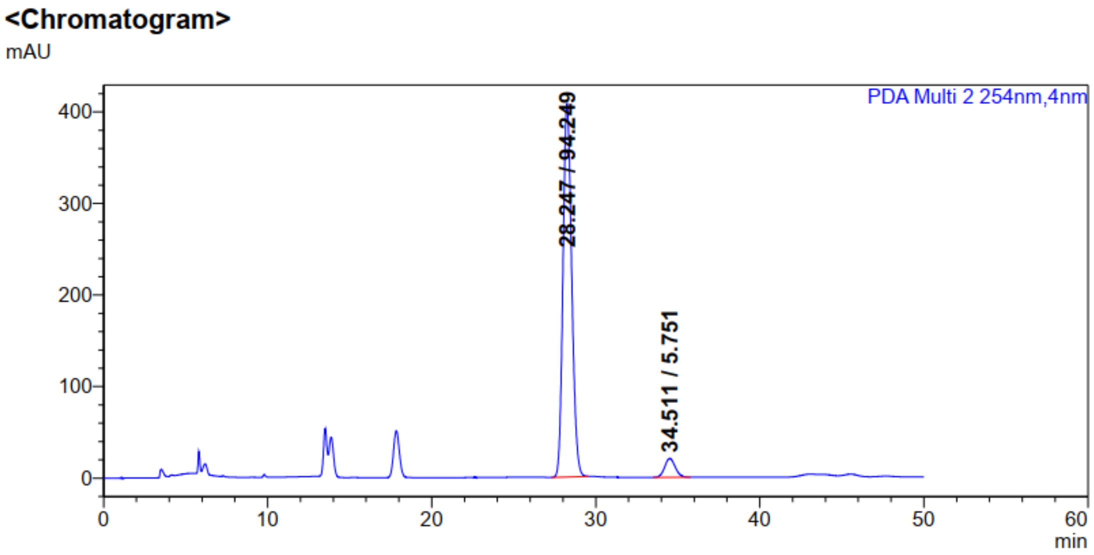

PDA Ch2 254nm

Peak#	Name	Ret. Time	Area	Area%
1		28.247	14982081	94.249
2		34.511	914123	5.751
Total			15896203	100.000

HPLC traces for reaction with: **2-Me-phenylboronic acid (duplicate)**

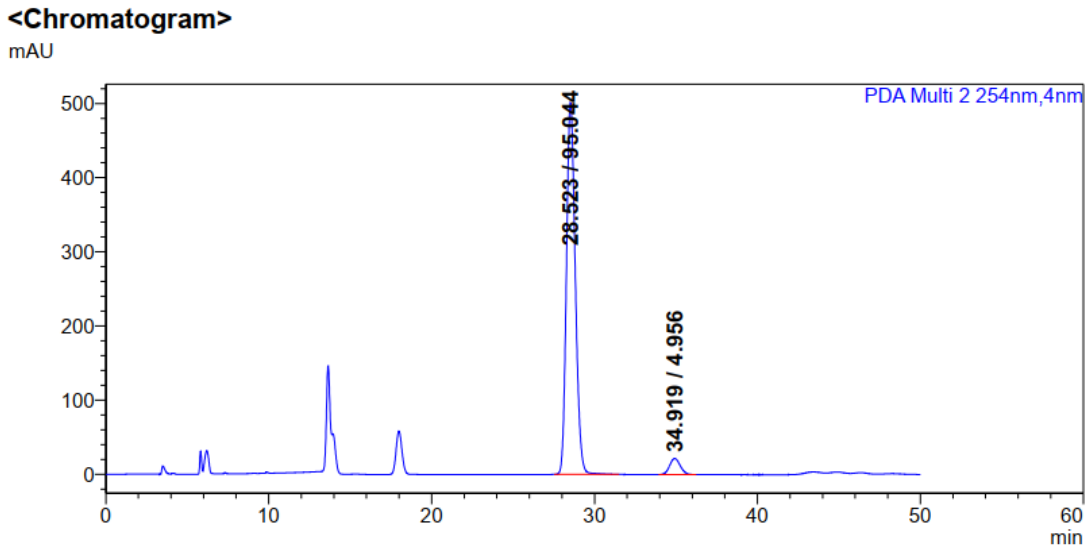

PDA Ch2 254nm

Peak#	Name	Ret. Time	Area	Area%
1		28.523	18911401	95.044
2		34.919	986135	4.956
Total			19897536	100.000

HPLC traces for reaction with: **Ph-phenylboronic acid**

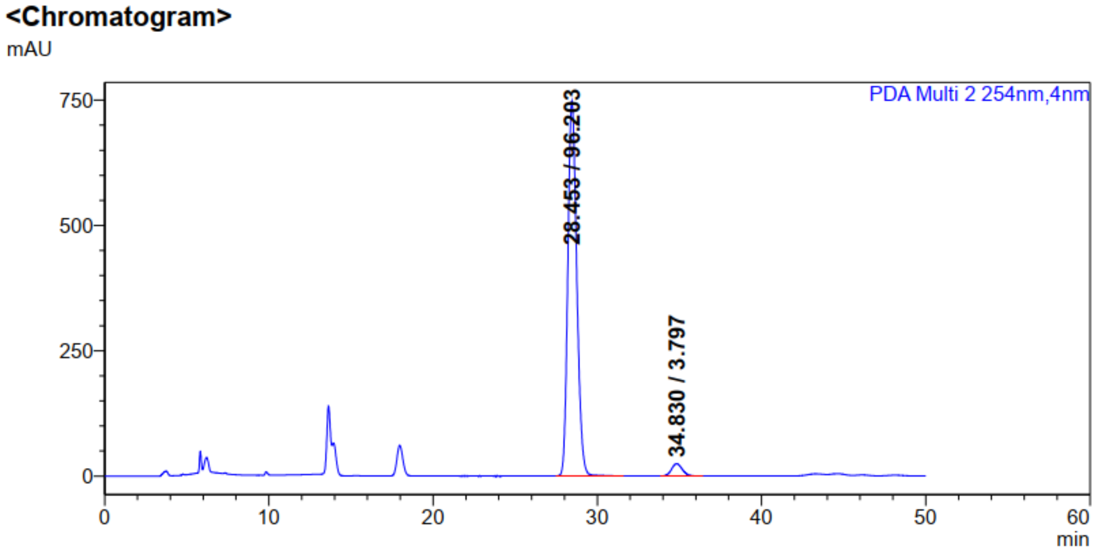

PDA Ch2 254nm

Peak#	Name	Ret. Time	Area	Area%
1		28.453	28231629	96.203
2		34.830	1114132	3.797
Total			29345762	100.000

HPLC traces for reaction with: **Ph-phenylboronic acid (duplicate)**

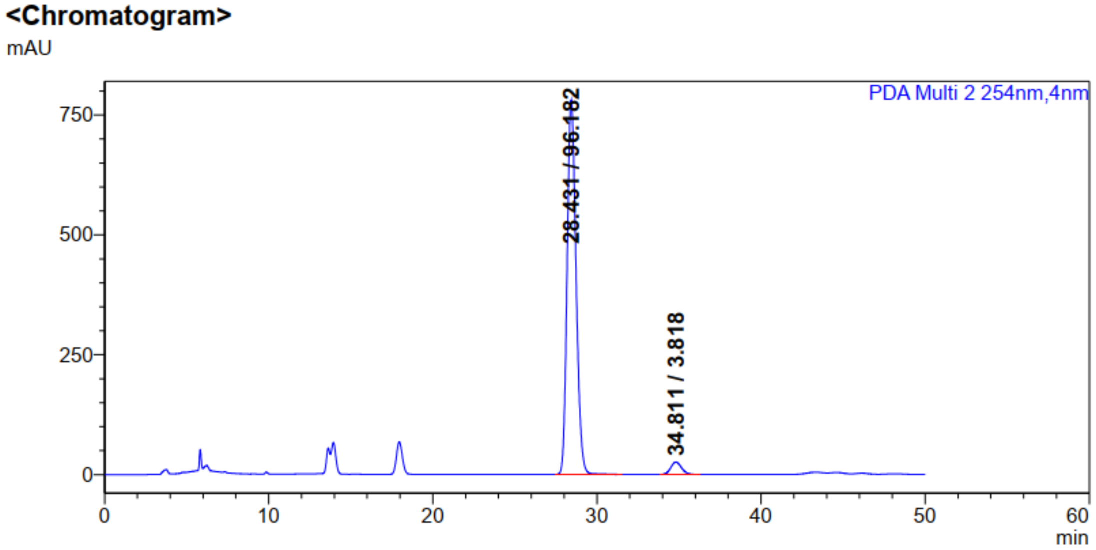

PDA Ch2 254nm

Peak#	Name	Ret. Time	Area	Area%
1		28.431	29461965	96.182
2		34.811	1169635	3.818
Total			30631600	100.000

HPLC traces for reaction with: **2-F-phenylboronic acid**

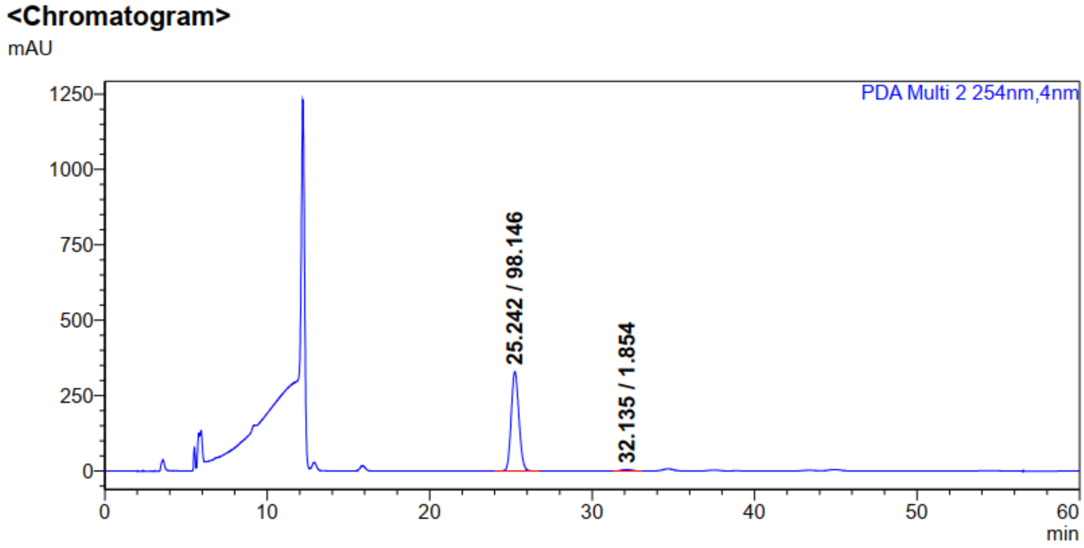

PDA Ch2 254nm

Peak#	Name	Ret. Time	Area	Area%
1		25.242	10857773	98.146
2		32.135	205149	1.854
Total			11062922	100.000

HPLC traces for reaction with: **2-F-phenylboronic acid (duplicate)**

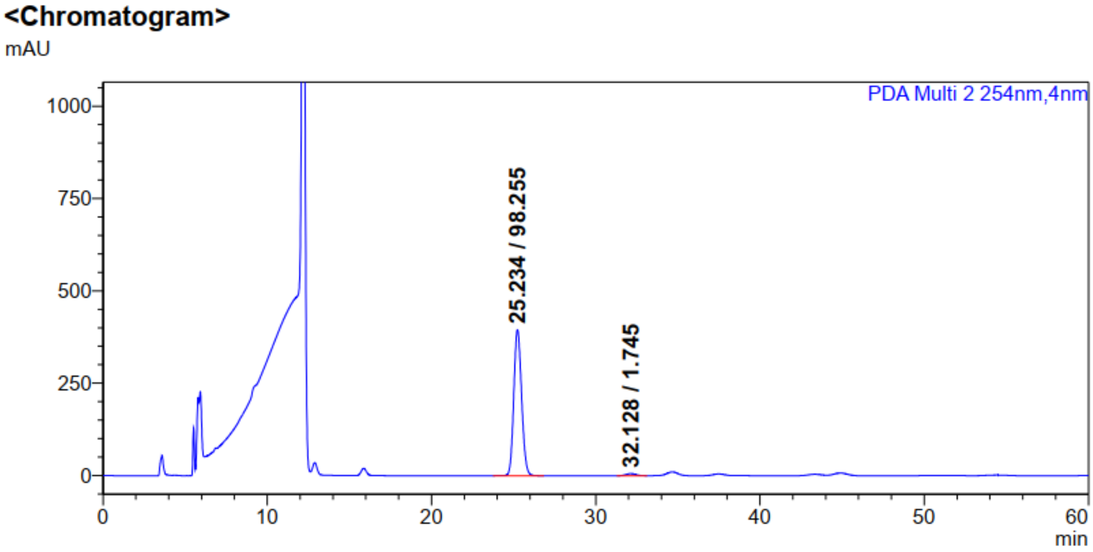

PDA Ch2 254nm

Peak#	Name	Ret. Time	Area	Area%
1		25.234	13061774	98.255
2		32.128	231942	1.745
Total			13293716	100.000

HPLC traces for **reaction without boronic acid**

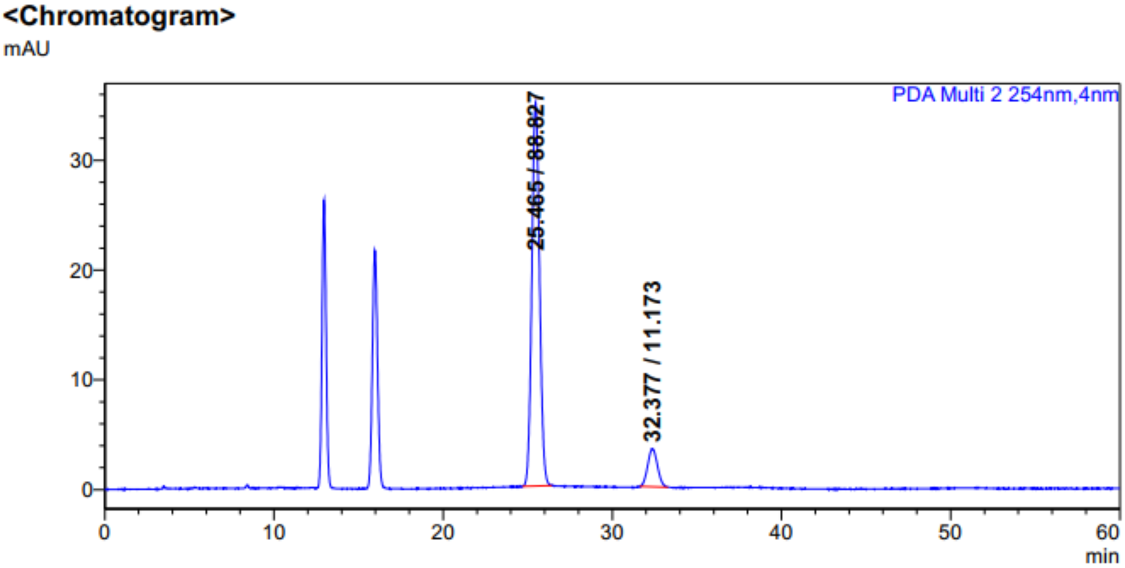

PDA Ch2 254nm

Peak#	Name	Ret. Time	Area	Area%
1		25.465	1105187	88.827
2		32.377	139011	11.173
Total			1244199	100.000

HPLC traces for **reaction without boronic acid (duplicate)**

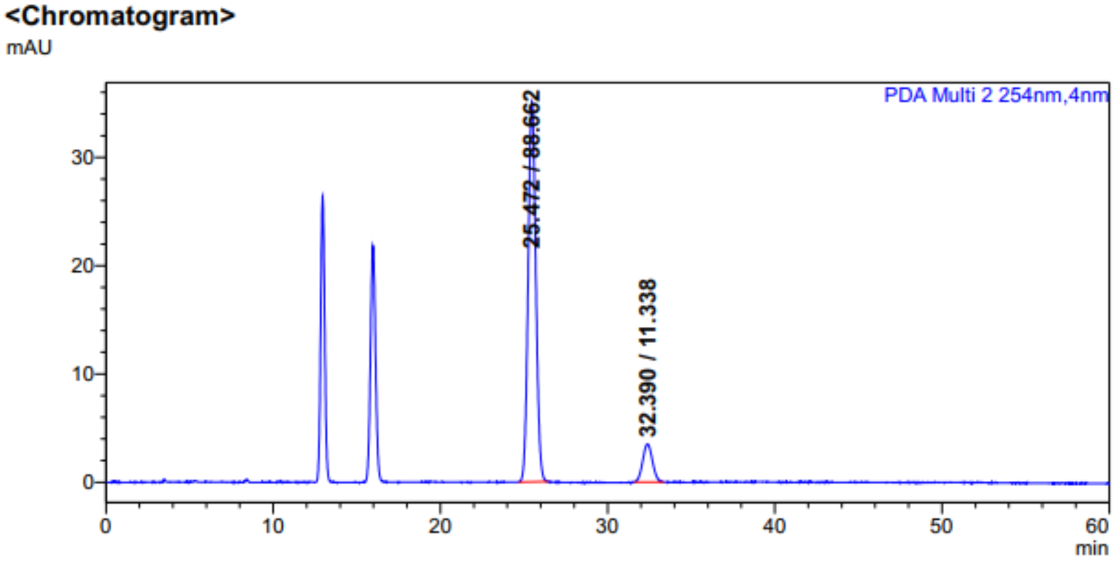

PDA Ch2 254nm

Peak#	Name	Ret. Time	Area	Area%
1		25.472	1113417	88.662
2		32.390	142379	11.338
Total			1255796	100.000

HPLC traces for **reaction without water, with 2-F-phenylboronic acid**

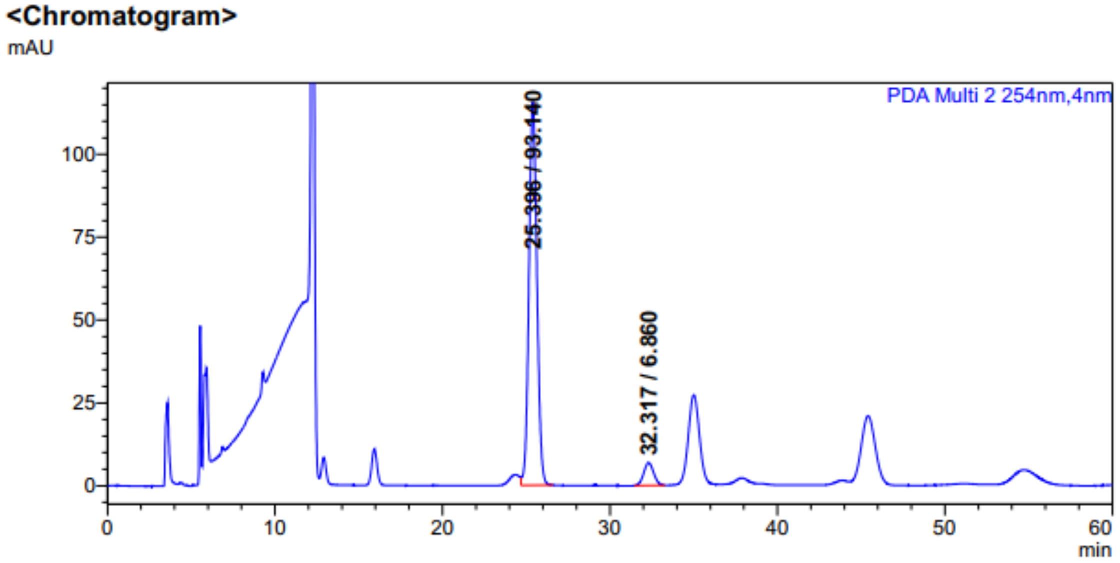

PDA Ch2 254nm

Peak#	Name	Ret. Time	Area	Area%
1		25.396	3825369	93.140
2		32.317	281758	6.860
Total			4107126	100.000

HPLC traces for **reaction without water, with 2-F-phenylboronic acid**

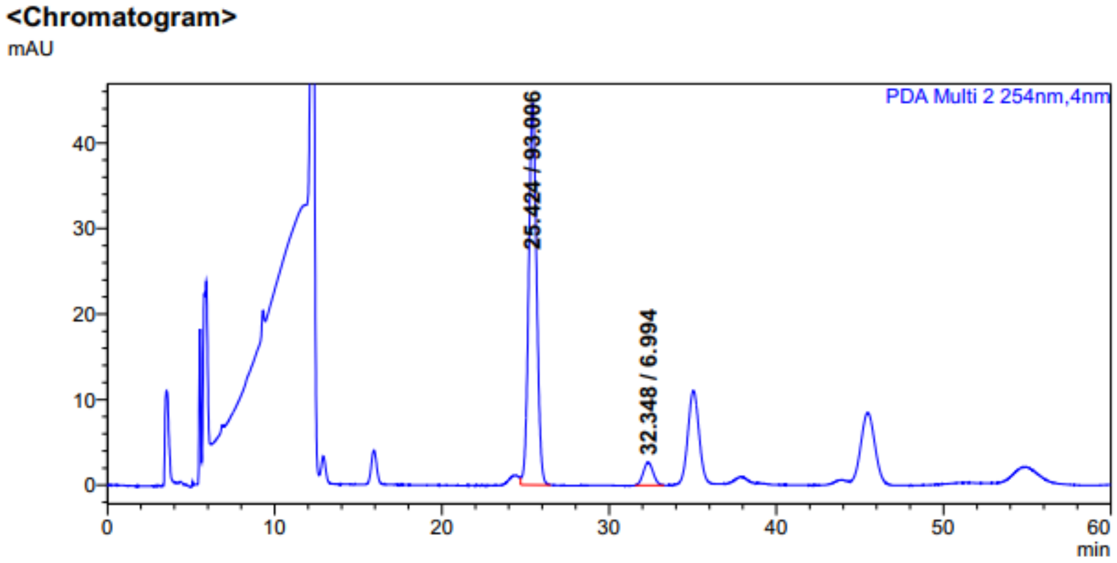

PDA Ch2 254nm

Peak#	Name	Ret. Time	Area	Area%
1		25.424	1471893	93.006
2		32.348	110689	6.994
Total			1582582	100.000

9. HPLC Traces for Table S2. Boronic acid screening and blanks in chloroform

All of the reactions were performed as duplicate following GP2 with different boronic acids. A racemic sample was first analyzed in order to determine the retention time of both enantiomers (See above – page 14). For the chiral sample, enantiomeric excess was determined by comparing the integrated area of these two peaks. All the HPLC traces shown below are for of (S)-2-((R)-hydroxy(4-nitrophenyl)methyl)cyclopentan-1-one, yielded by different reactions as described in table S2.

HPLC traces for reaction with: **3-F-phenylboronic acid**

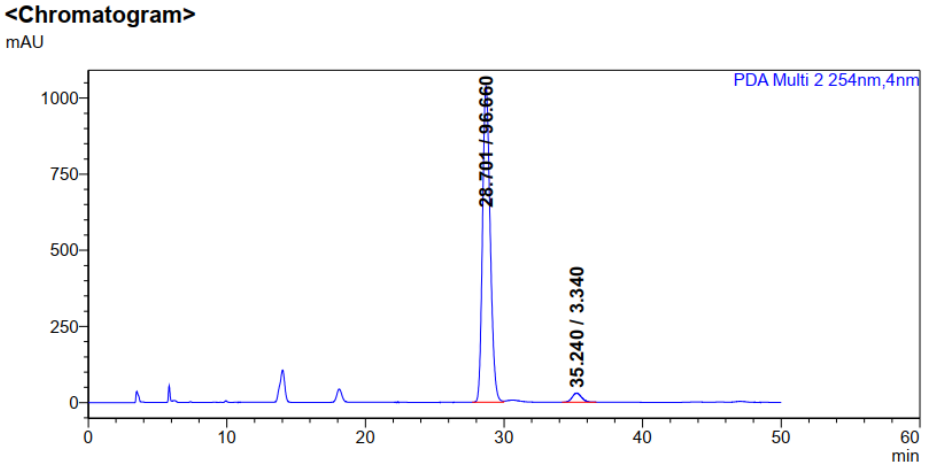

PDA Ch2 254nm

Peak#	Name	Ret. Time	Area	Area%
1		28.701	39916418	96.660
2		35.240	1379103	3.340
Total			41295521	100.000

HPLC traces for reaction with: **3-F-phenylboronic acid (duplicate)**

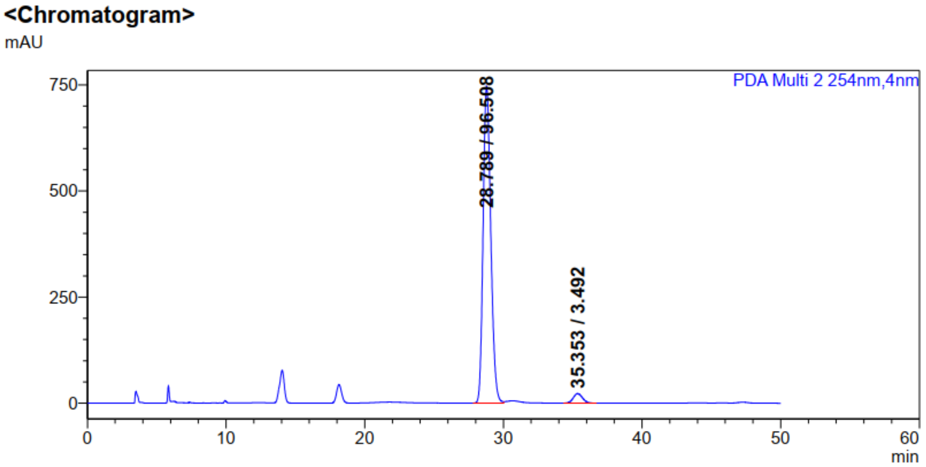

PDA Ch2 254nm

Peak#	Name	Ret. Time	Area	Area%
1		28.789	28678483	96.508
2		35.353	1037745	3.492
Total			29716228	100.000

HPLC traces for reaction with: **3-5-F-phenylboronic acid**

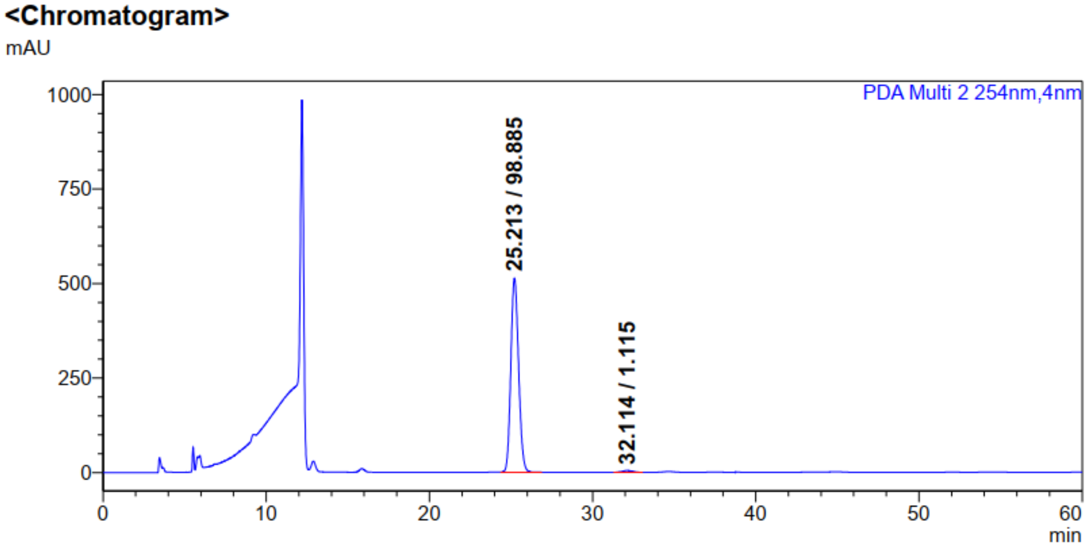

PDA Ch2 254nm

Peak#	Name	Ret. Time	Area	Area%
1		25.213	16946733	98.885
2		32.114	191140	1.115
Total			17137872	100.000

HPLC traces for reaction with: **3,5-F-phenylboronic acid (duplicate)**

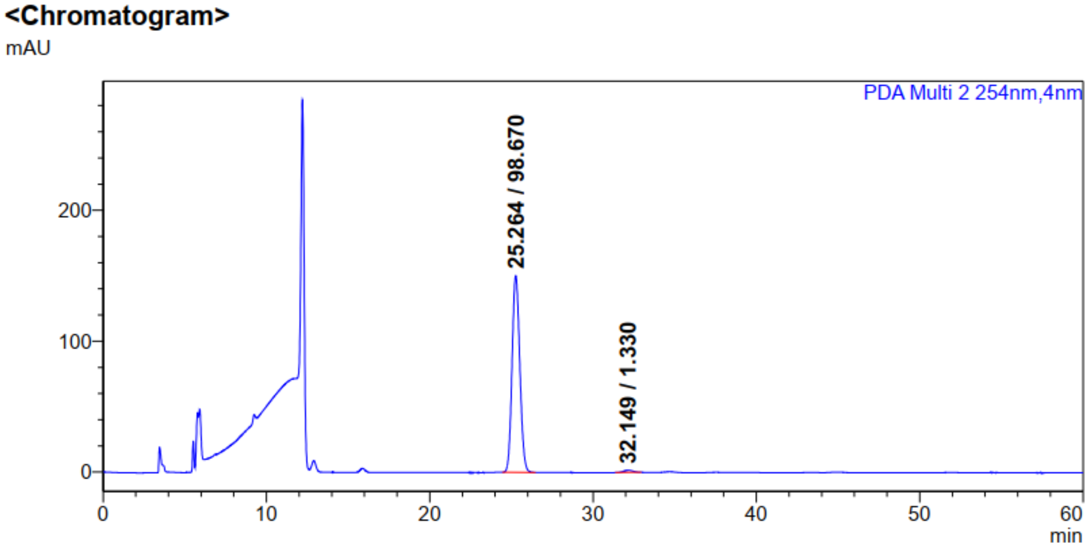

PDA Ch2 254nm

Peak#	Name	Ret. Time	Area	Area%
1		25.264	4949308	98.670
2		32.149	66711	1.330
Total			5016019	100.000

HPLC traces for reaction with: **4-tBu-phenylboronic acid**

<Chromatogram>

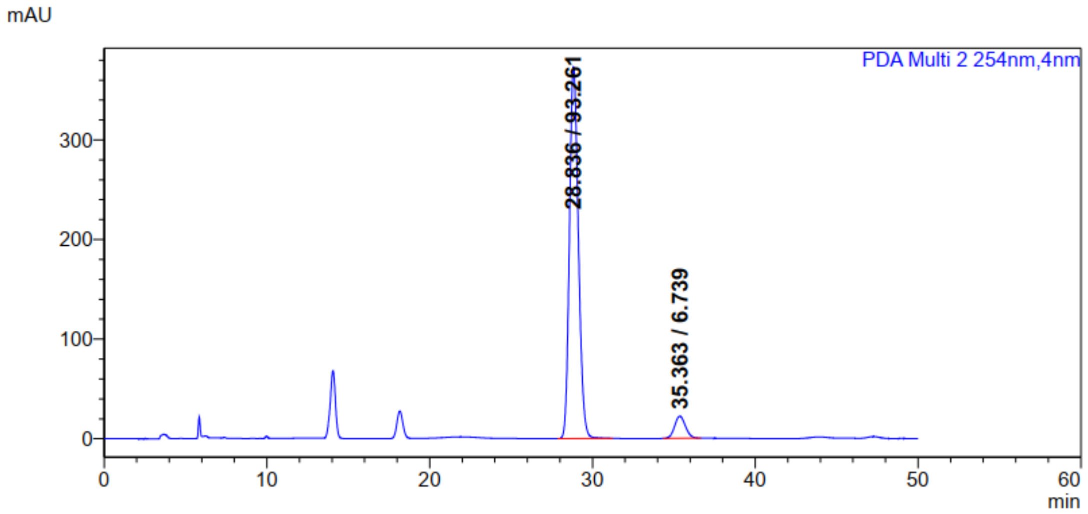

PDA Ch2 254nm

Peak#	Name	Ret. Time	Area	Area%
1		28.836	14236554	93.261
2		35.363	1028772	6.739
Total			15265326	100.000

HPLC traces for reaction with: **4-tBu-phenylboronic acid (duplicate)**

<Chromatogram>

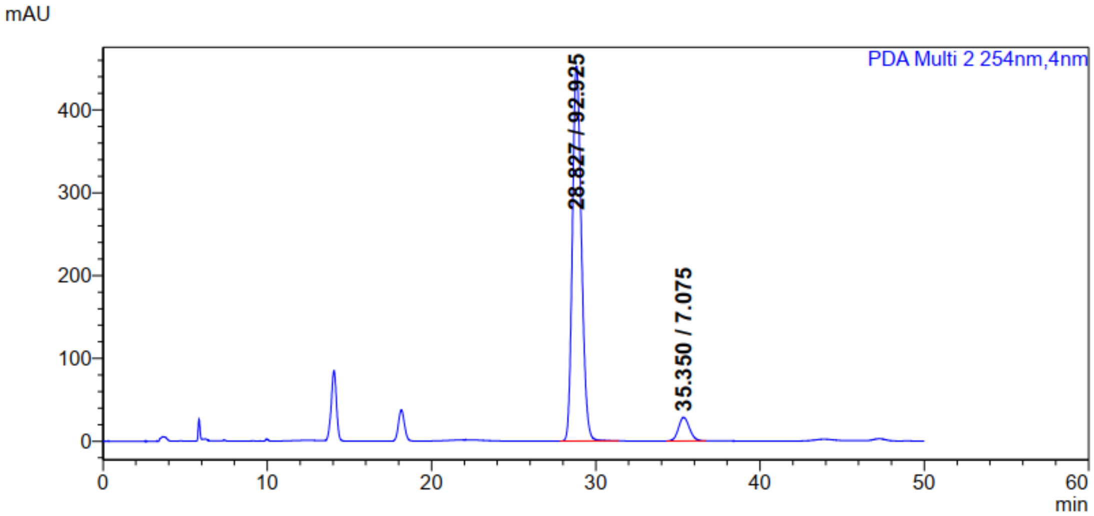

PDA Ch2 254nm

Peak#	Name	Ret. Time	Area	Area%
1		28.827	17311536	92.925
2		35.350	1318059	7.075
Total			18629595	100.000

HPLC traces for reaction with: **2,4-Me-phenylboronic acid.**

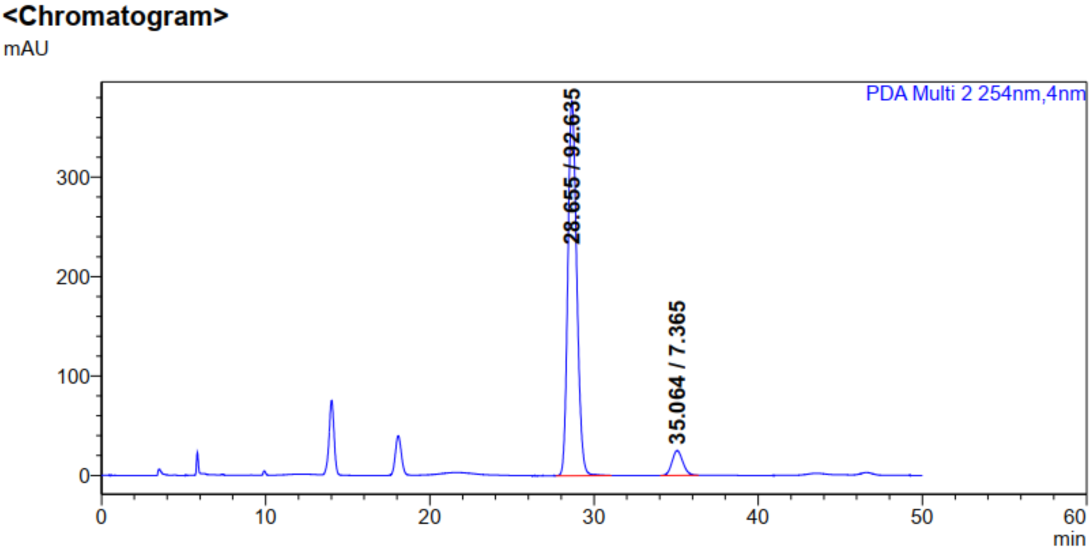

PDA Ch2 254nm

Peak#	Name	Ret. Time	Area	Area%
1		28.655	14222889	92.635
2		35.064	1130862	7.365
Total			15353751	100.000

HPLC traces for reaction with: **2,4-Me-phenylboronic acid (duplicate)**

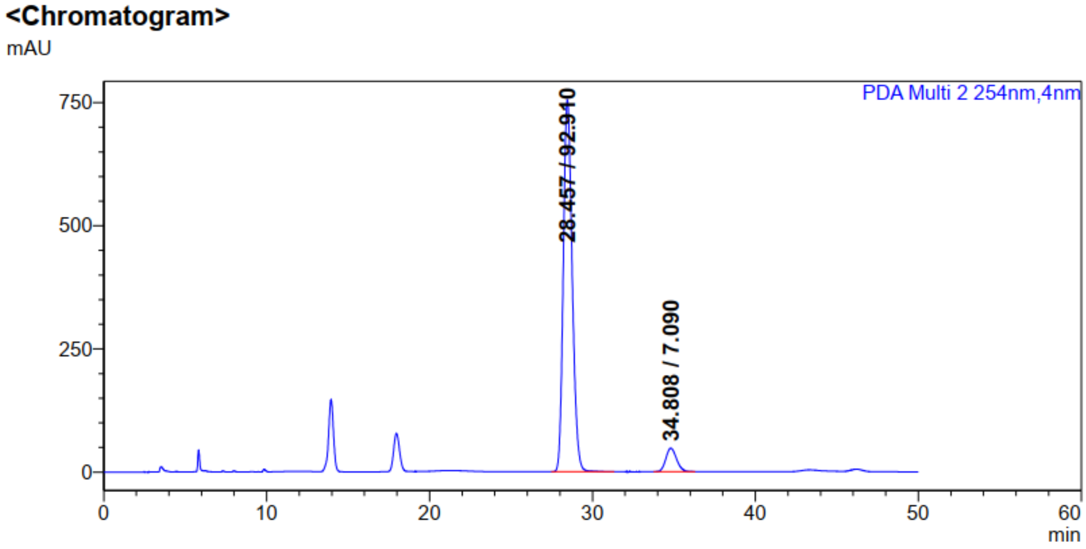

PDA Ch2 254nm

Peak#	Name	Ret. Time	Area	Area%
1		28.457	28495967	92.910
2		34.808	2174702	7.090
Total			30670670	100.000

HPLC traces for reaction with: **3,5-OMe-phenylboronic acid**

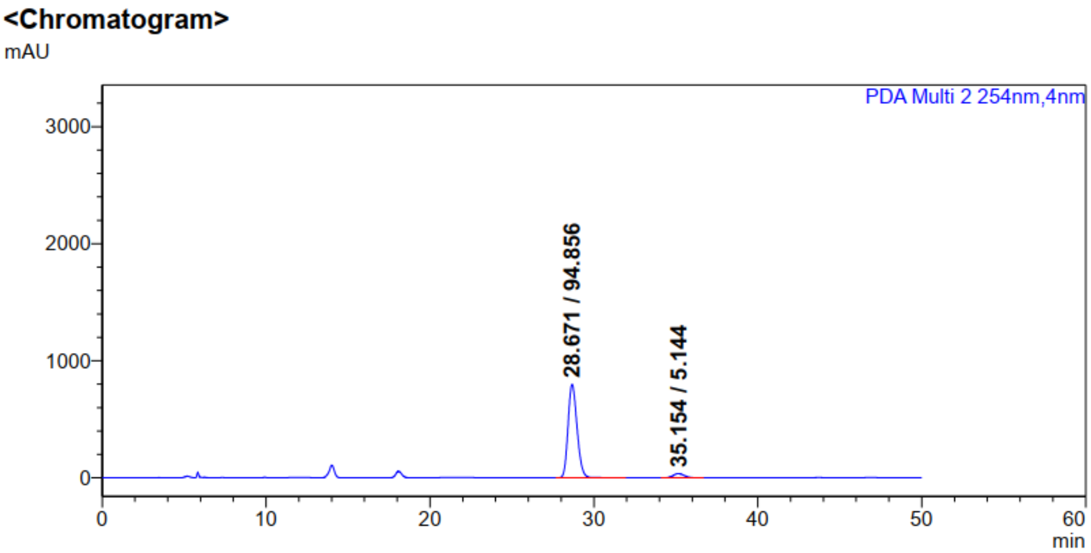

PDA Ch2 254nm

Peak#	Name	Ret. Time	Area	Area%
1		28.671	30624527	94.856
2		35.154	1660924	5.144
Total			32285452	100.000

HPLC traces for reaction with: **3,5-OMe-phenylboronic acid (duplicate)**

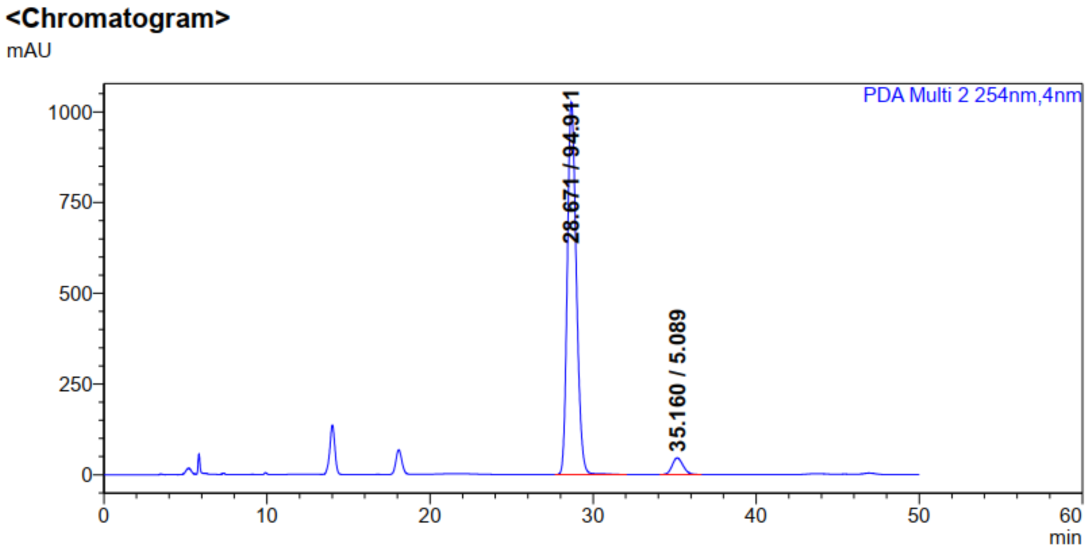

PDA Ch2 254nm

Peak#	Name	Ret. Time	Area	Area%
1		28.671	39499420	94.911
2		35.160	2117783	5.089
Total			41617203	100.000

HPLC traces for reaction with: **4-Me-phenylboronic acid**

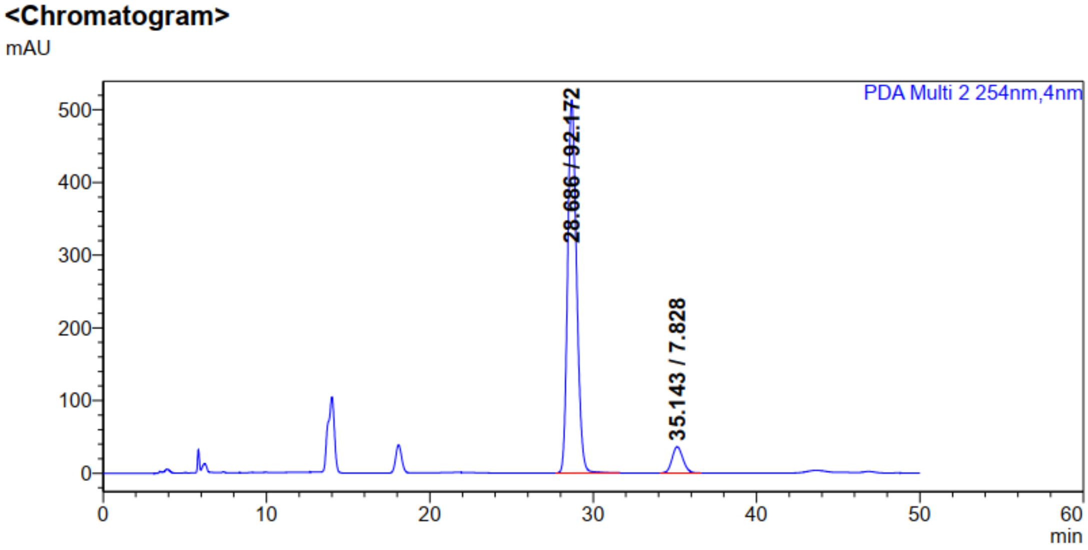

PDA Ch2 254nm

Peak#	Name	Ret. Time	Area	Area%
1		28.686	19551609	92.172
2		35.143	1660432	7.828
Total			21212042	100.000

HPLC traces for reaction with: **4-Me-phenylboronic acid (duplicate)**

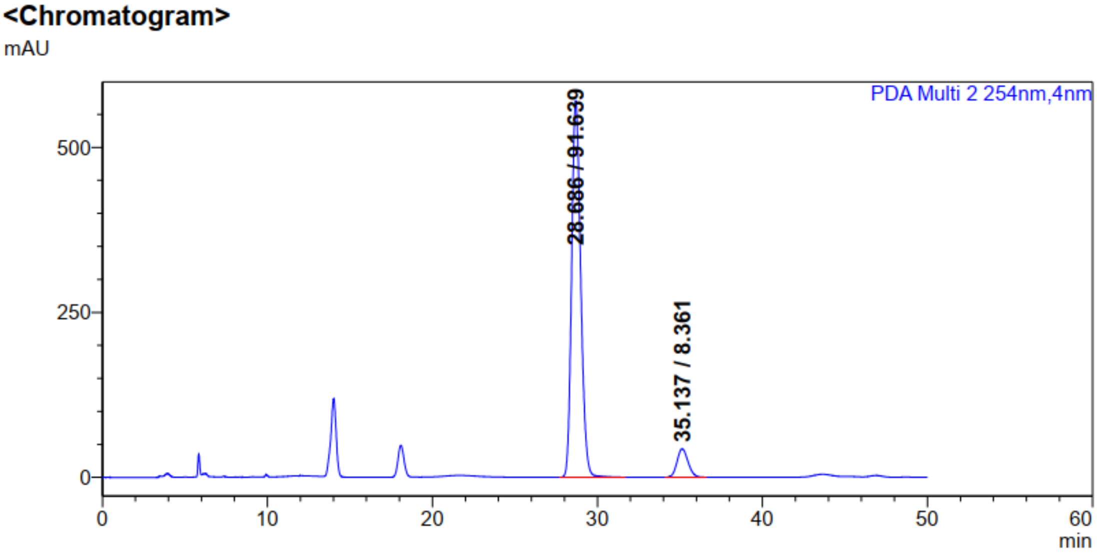

PDA Ch2 254nm

Peak#	Name	Ret. Time	Area	Area%
1		28.686	21759946	91.639
2		35.137	1985259	8.361
Total			23745205	100.000

HPLC traces for reaction with: **3-CF_3-phenylboronic acid**

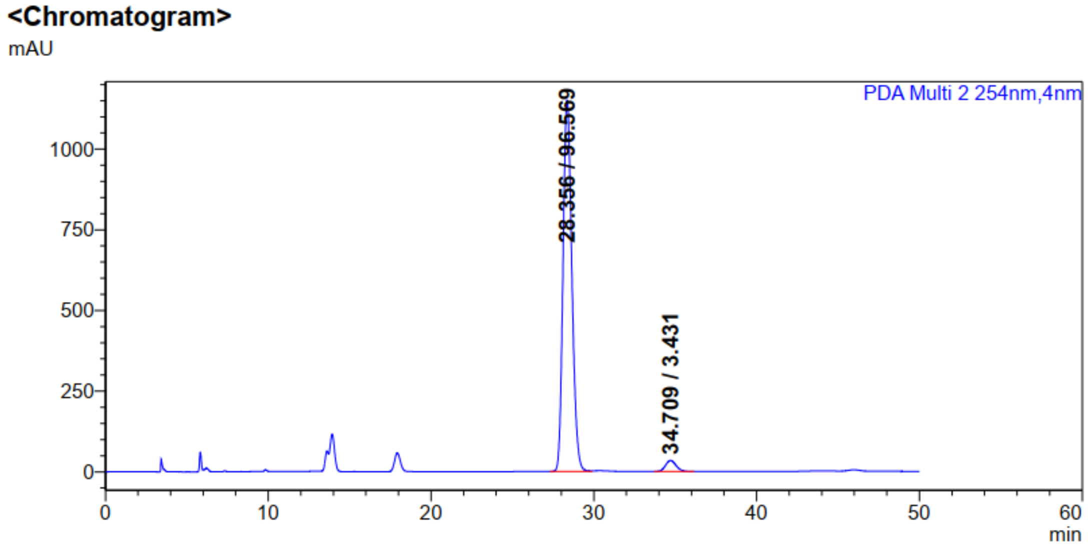

PDA Ch2 254nm

Peak#	Name	Ret. Time	Area	Area%
1		28.356	43315317	96.569
2		34.709	1538802	3.431
Total			44854119	100.000

HPLC traces for reaction with: **3-CF_3-phenylboronic acid (duplicate)**

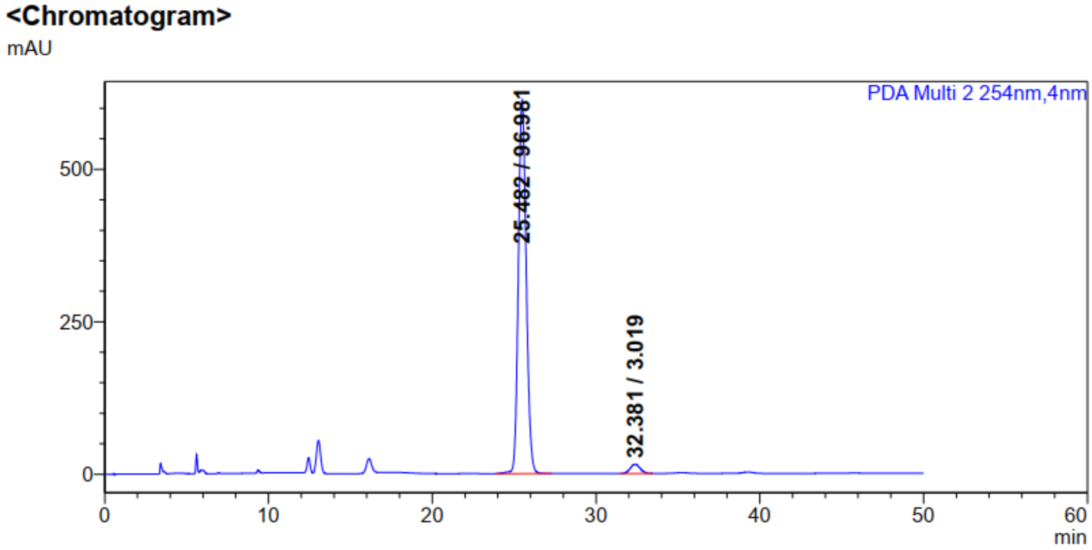

PDA Ch2 254nm

Peak#	Name	Ret. Time	Area	Area%
1		25.482	20400838	96.981
2		32.381	635161	3.019
Total			21035999	100.000

HPLC traces for reaction with: **2-Naphtalene-phenylboronic acid**

<Chromatogram>

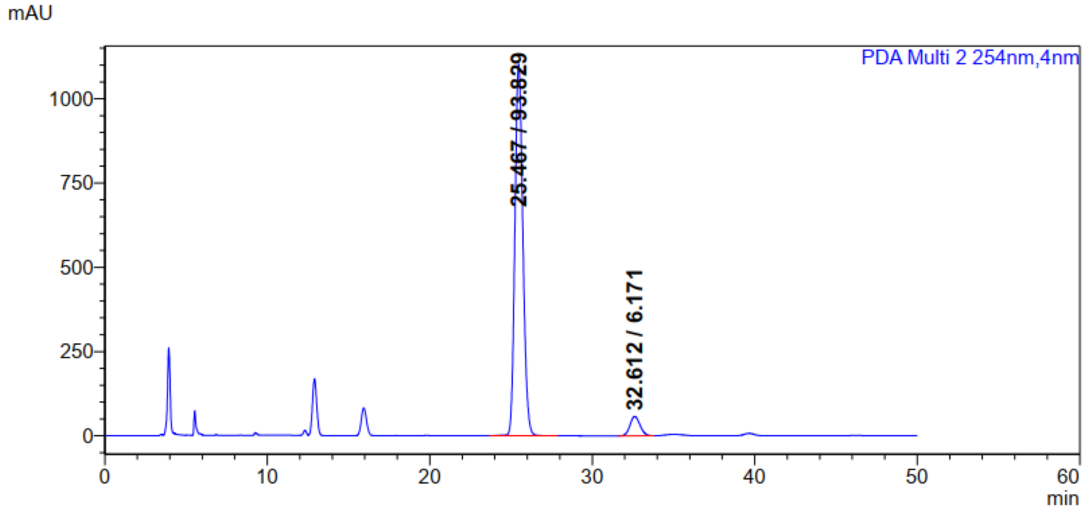

PDA Ch2 254nm

Peak#	Name	Ret. Time	Area	Area%
1		25.467	37576864	93.829
2		32.612	2471321	6.171
Total			40048184	100.000

HPLC traces for reaction with: **2-Naphtalene-phenylboronic acid (duplicate)**

<Chromatogram>

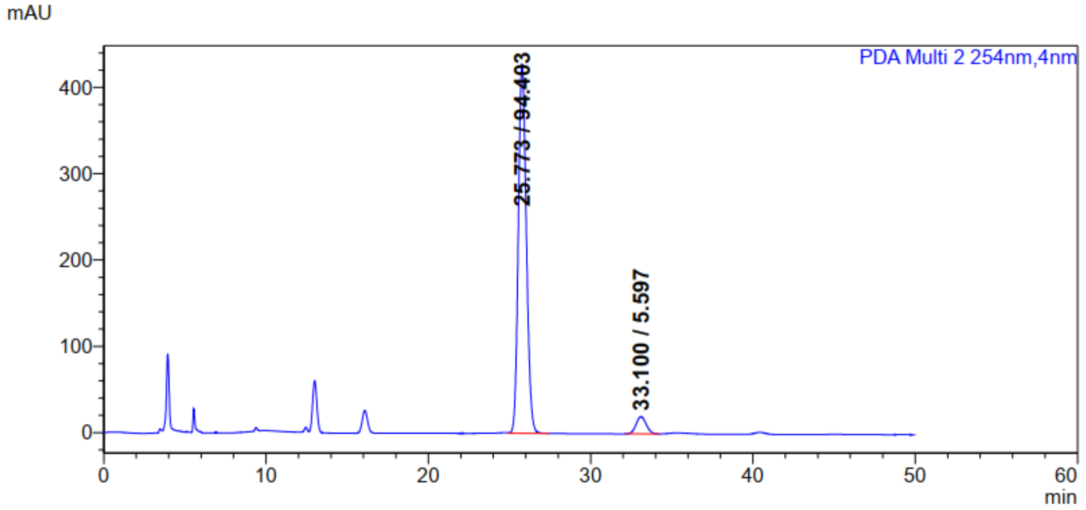

PDA Ch2 254nm

Peak#	Name	Ret. Time	Area	Area%
1		25.773	14635539	94.403
2		33.100	867677	5.597
Total			15503216	100.000

HPLC traces for reaction with: **4-CF$_3$-phenylboronic acid**

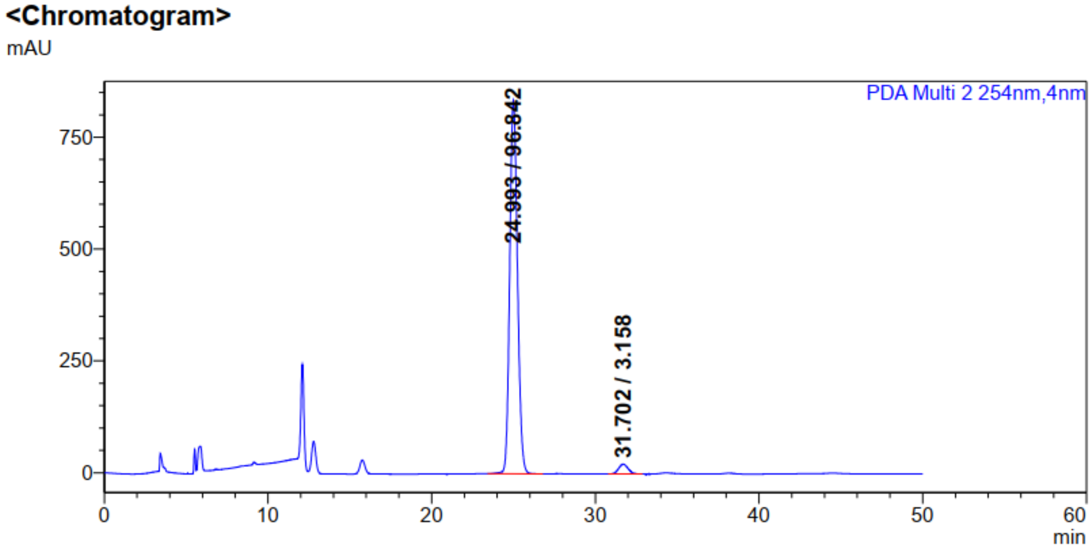

PDA Ch2 254nm

Peak#	Name	Ret. Time	Area	Area%
1		24.993	27454206	96.842
2		31.702	895309	3.158
Total			28349515	100.000

HPLC traces for reaction with: **4-CF$_3$-phenylboronic acid (duplicate)**

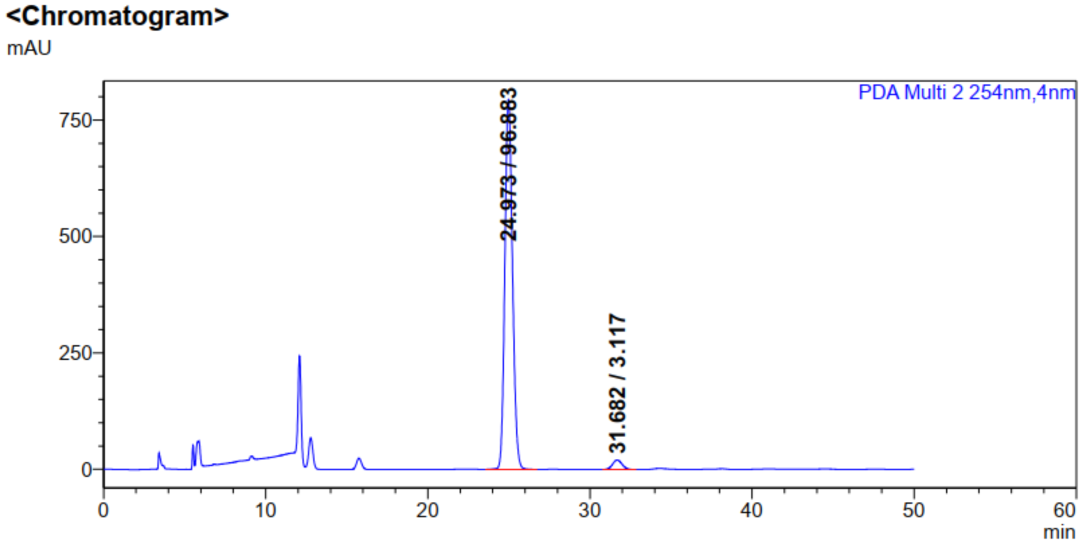

PDA Ch2 254nm

Peak#	Name	Ret. Time	Area	Area%
1		24.973	26038920	96.883
2		31.682	837854	3.117
Total			26876774	100.000

HPLC traces for reaction with: **4-F-phenylboronic acid**

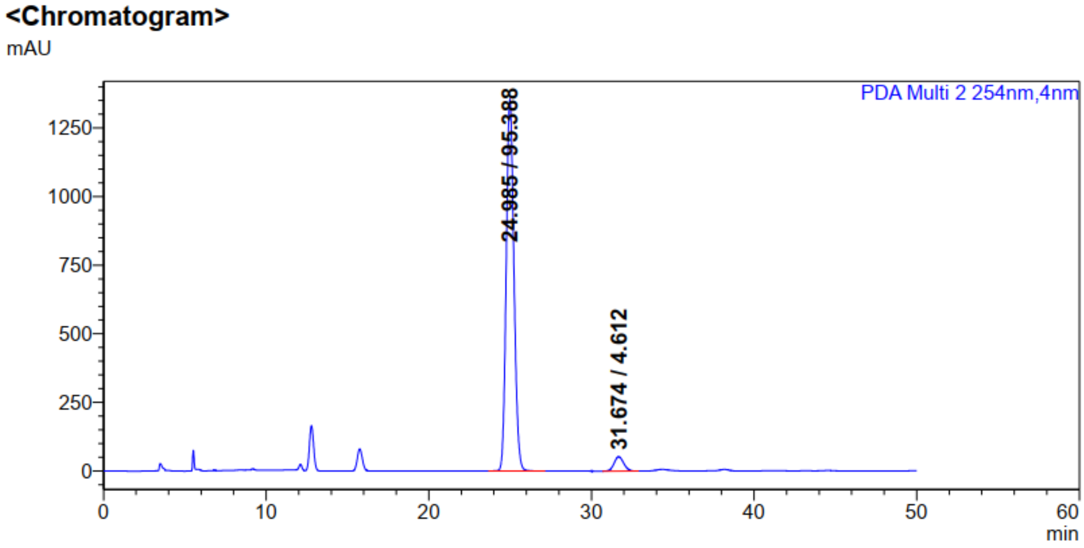

PDA Ch2 254nm

Peak#	Name	Ret. Time	Area	Area%
1		24.985	44387963	95.388
2		31.674	2146135	4.612
Total			46534098	100.000

HPLC traces for reaction with: **4-F-phenylboronic acid (duplicate)**

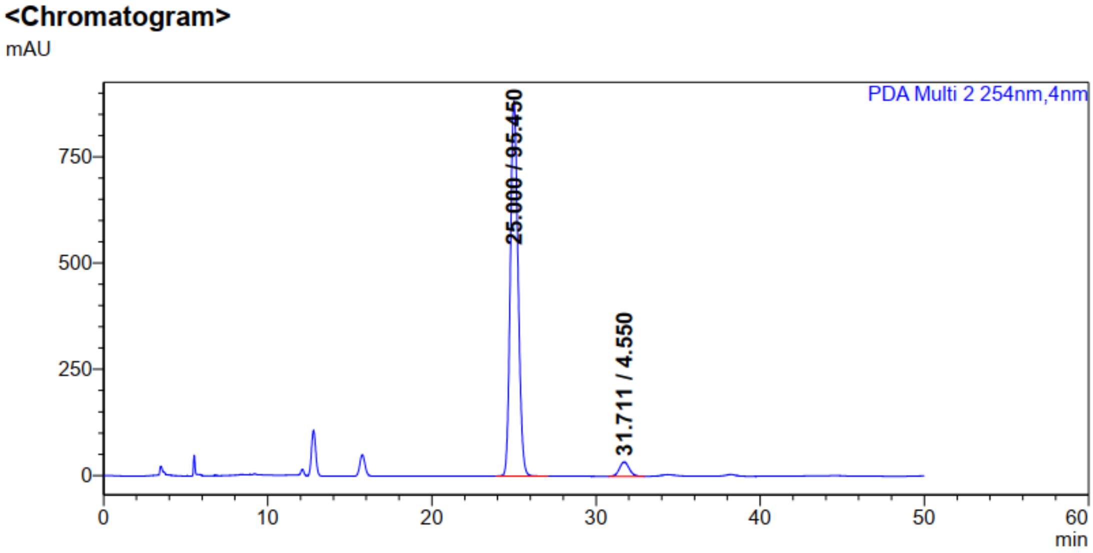

PDA Ch2 254nm

Peak#	Name	Ret. Time	Area	Area%
1		25.000	28836534	95.450
2		31.711	1374553	4.550
Total			30211087	100.000

HPLC traces for reaction with: **4-OMe-phenylboronic acid**

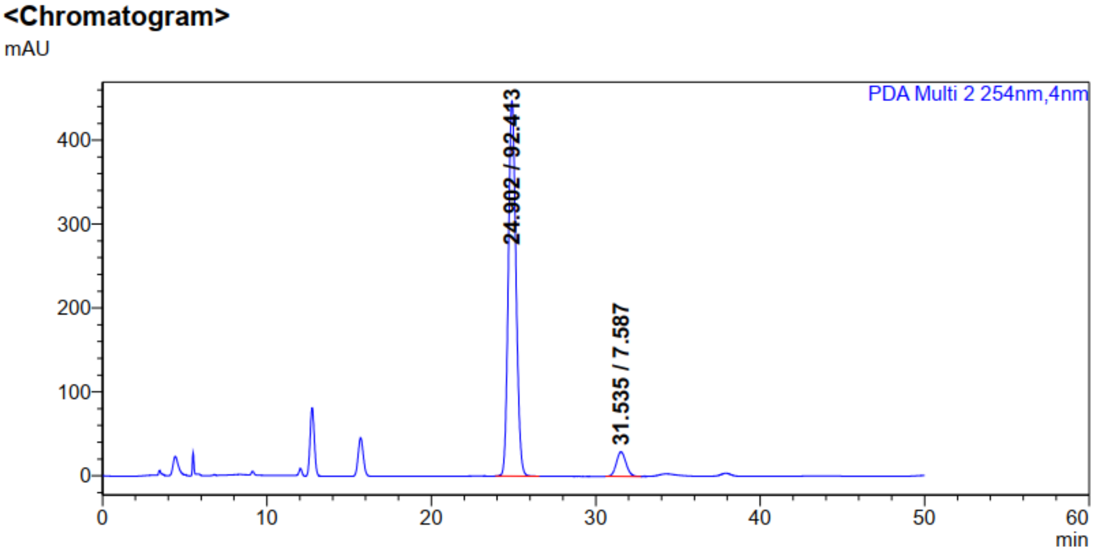

PDA Ch2 254nm

Peak#	Name	Ret. Time	Area	Area%
1		24.902	14453829	92.413
2		31.535	1186629	7.587
Total			15640459	100.000

HPLC traces for reaction with: **4-OMe-phenylboronic acid (duplicate)**

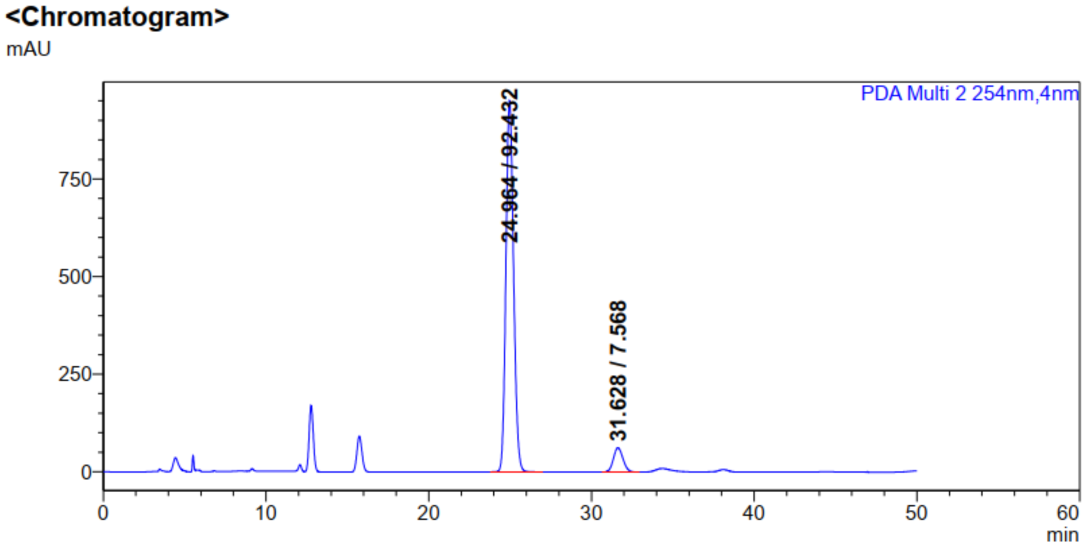

PDA Ch2 254nm

Peak#	Name	Ret. Time	Area	Area%
1		24.964	31121473	92.432
2		31.628	2548224	7.568
Total			33669697	100.000

HPLC traces for reaction with: **3-Me-phenylboronic acid**

<Chromatogram>

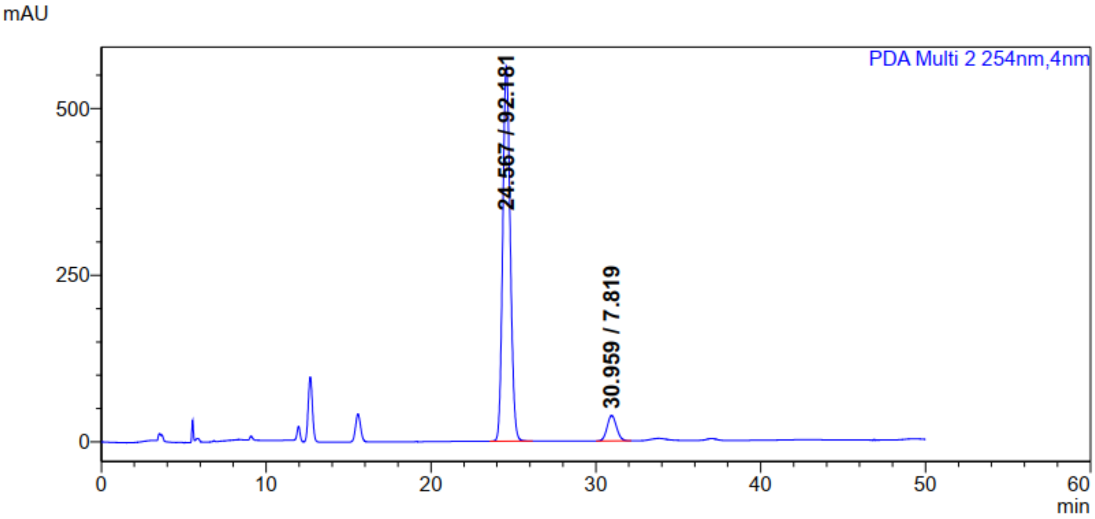

PDA Ch2 254nm

Peak#	Name	Ret. Time	Area	Area%
1		24.567	17752123	92.181
2		30.959	1505866	7.819
Total			19257989	100.000

HPLC traces for reaction with: **3-Me-phenylboronic acid (duplicate)**

<Chromatogram>

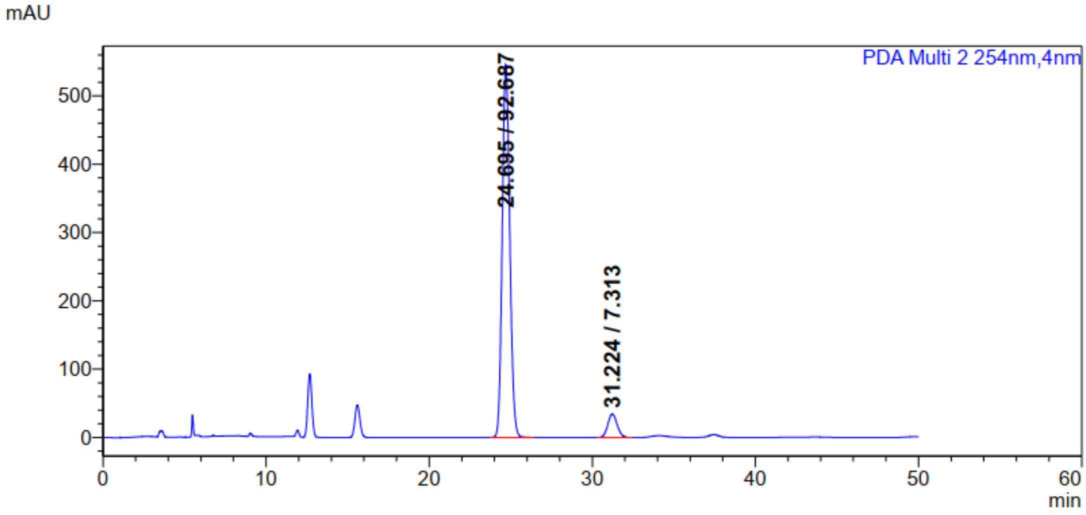

PDA Ch2 254nm

Peak#	Name	Ret. Time	Area	Area%
1		24.695	17491033	92.687
2		31.224	1380024	7.313
Total			18871057	100.000

HPLC traces for reaction with: **2-Me-phenylboronic acid**

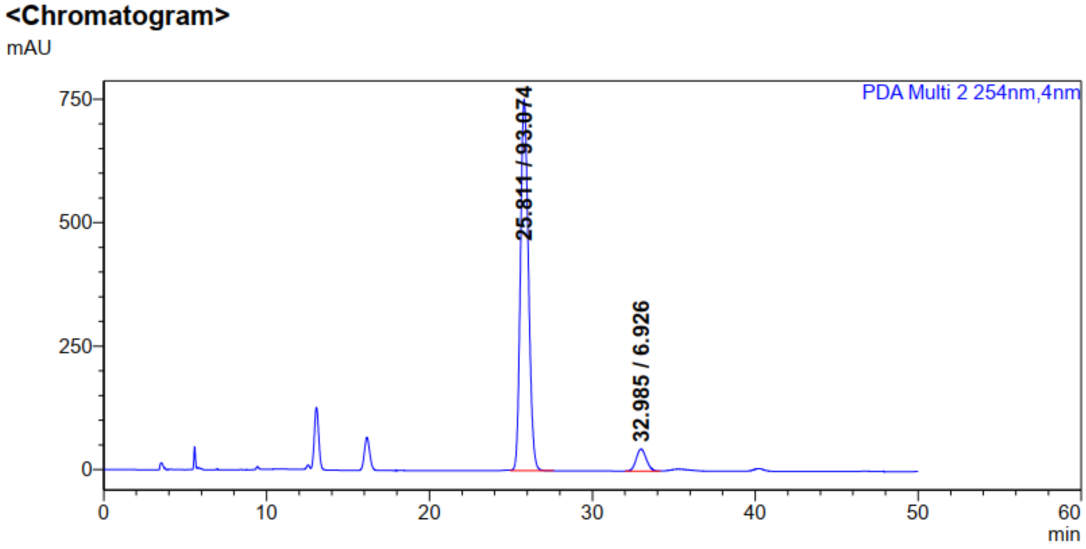

PDA Ch2 254nm

Peak#	Name	Ret. Time	Area	Area%
1		25.811	25718211	93.074
2		32.985	1913833	6.926
Total			27632043	100.000

HPLC traces for reaction with: **2-Me-phenylboronic acid (duplicate)**

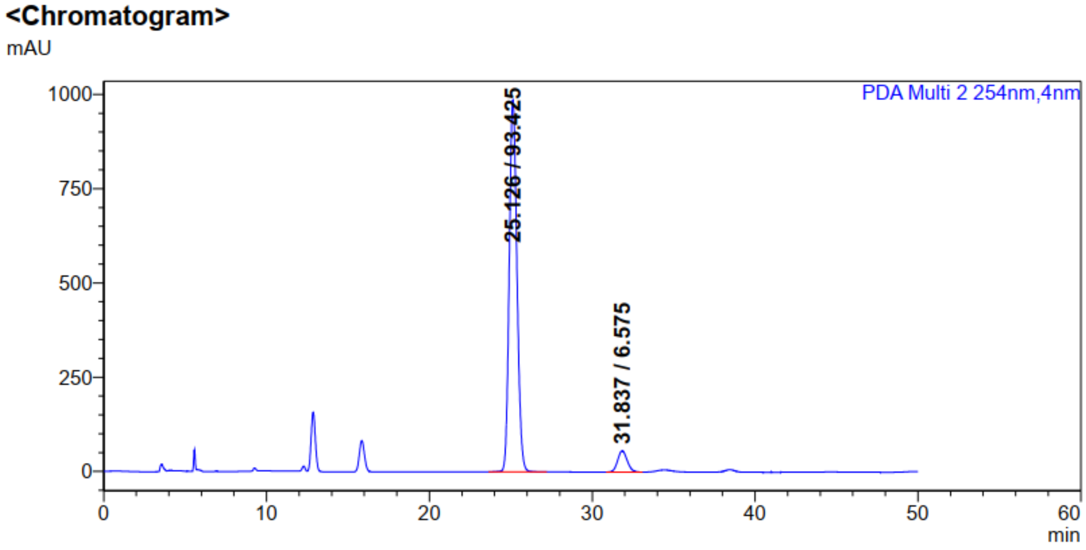

PDA Ch2 254nm

Peak#	Name	Ret. Time	Area	Area%
1		25.126	32502820	93.425
2		31.837	2287574	6.575
Total			34790394	100.000

HPLC traces for reaction with: **Ph-phenylboronic acid**

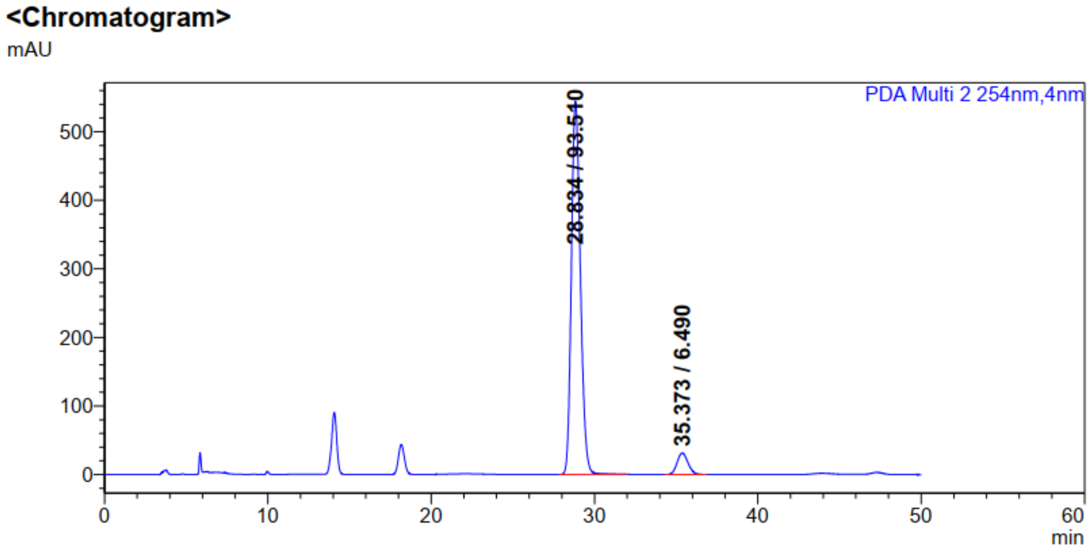

PDA Ch2 254nm

Peak#	Name	Ret. Time	Area	Area%
1		28.834	20886731	93.510
2		35.373	1449713	6.490
Total			22336444	100.000

HPLC traces for reaction with: **Ph-phenylboronic acid (duplicate)**

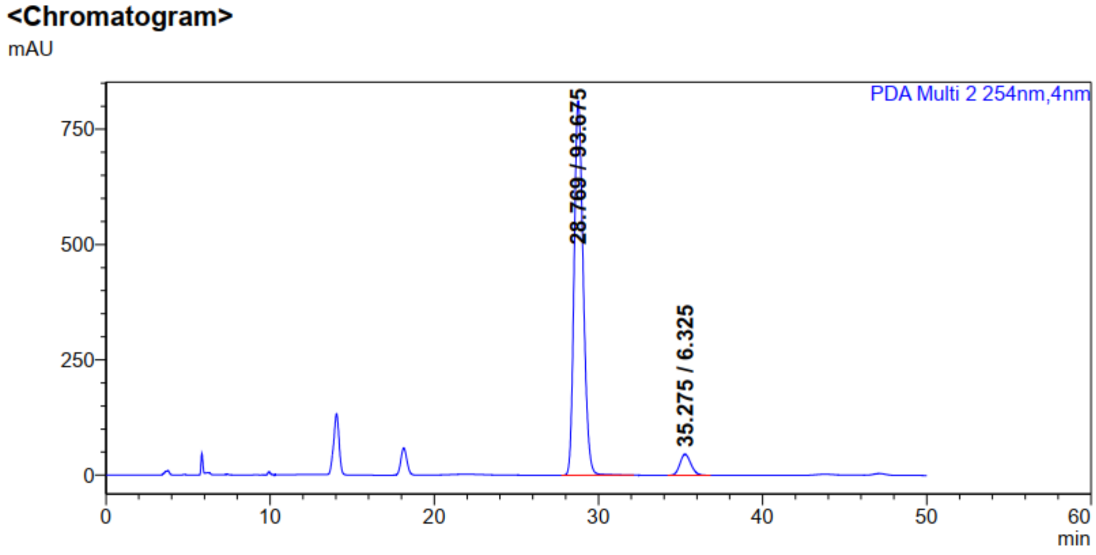

PDA Ch2 254nm

Peak#	Name	Ret. Time	Area	Area%
1		28.769	31282315	93.675
2		35.275	2112283	6.325
Total			33394598	100.000

HPLC traces for reaction with: **2-F-phenylboronic acid**

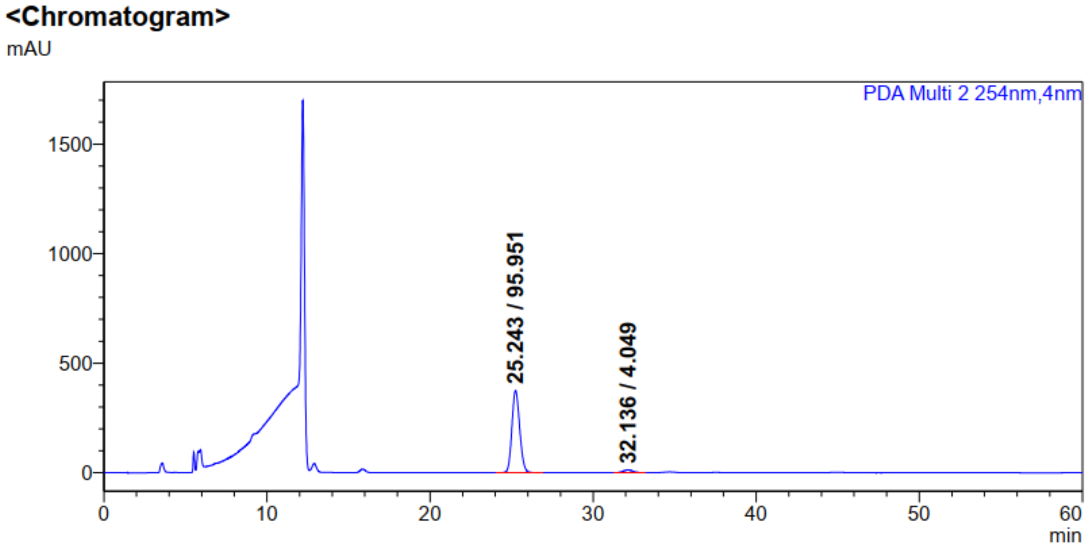

PDA Ch2 254nm

Peak#	Name	Ret. Time	Area	Area%
1		25.243	12416156	95.951
2		32.136	524003	4.049
Total			12940159	100.000

HPLC traces for reaction with: **2-F-phenylboronic acid (duplicate)**

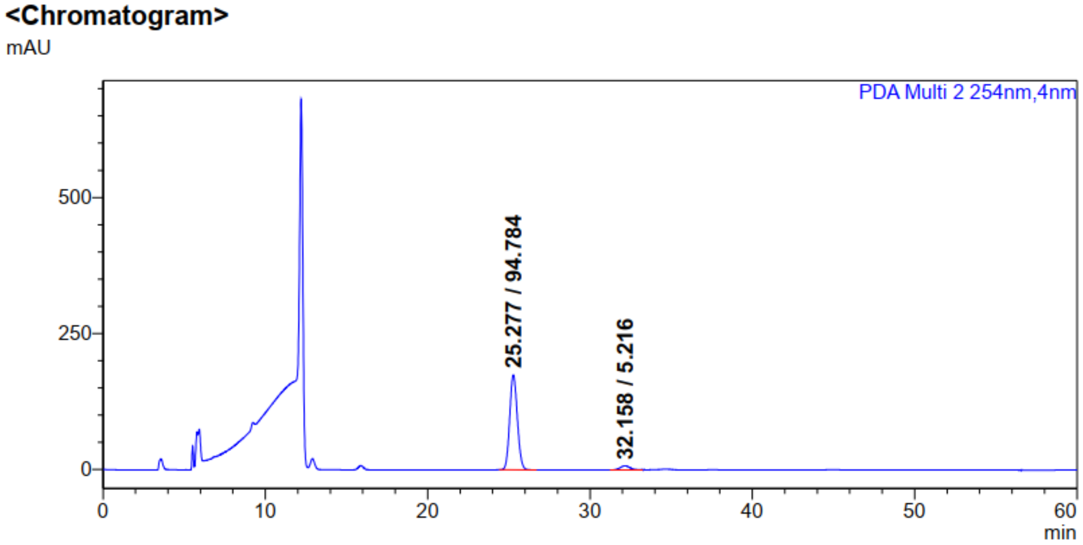

PDA Ch2 254nm

Peak#	Name	Ret. Time	Area	Area%
1		25.277	5713439	94.784
2		32.158	314438	5.216
Total			6027877	100.000

HPLC traces for reaction **without boronic acid**

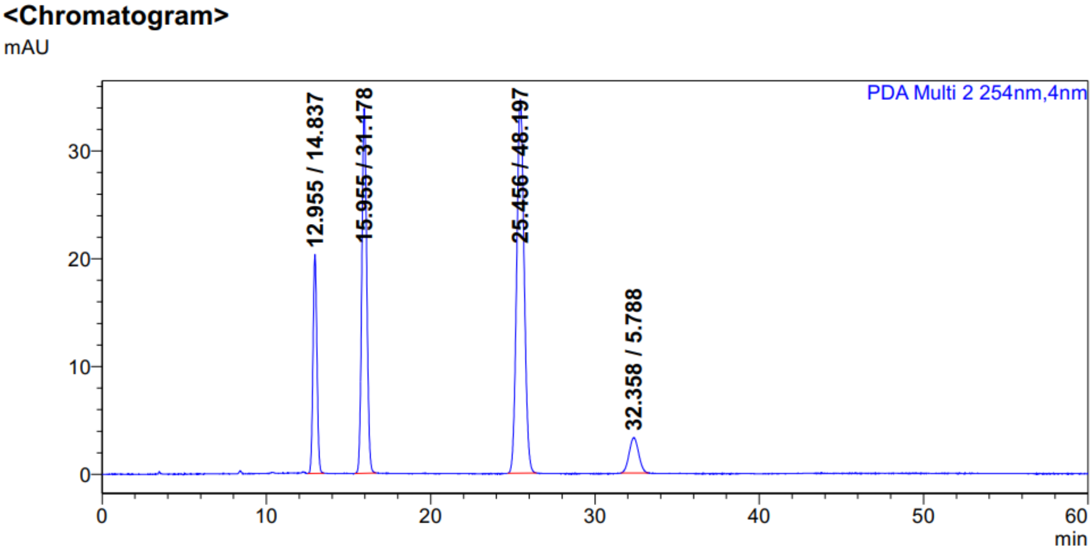

PDA Ch2 254nm

Peak#	Name	Ret. Time	Area	Area%
1		12.955	337937	14.837
2		15.955	710107	31.178
3		25.456	1097732	48.197
4		32.358	131831	5.788
Total			2277607	100.000

HPLC traces for reaction **without boronic acid (duplicate)**

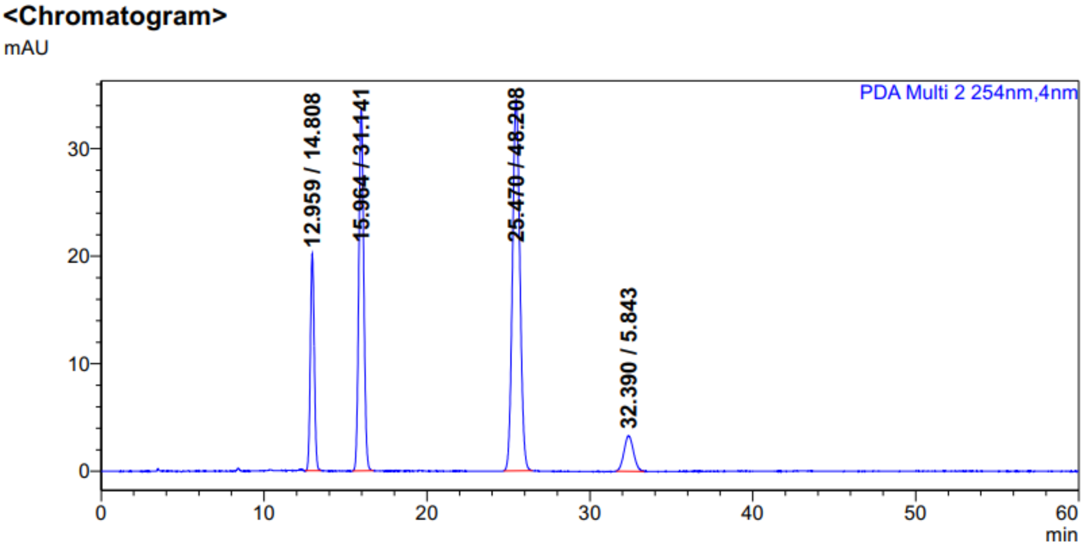

PDA Ch2 254nm

Peak#	Name	Ret. Time	Area	Area%
1		12.959	336258	14.808
2		15.964	707167	31.141
3		25.470	1094719	48.208
4		32.390	132682	5.843
Total			2270827	100.000

HPLC traces for reaction **without water, with 2-F-phenylboronic acid**

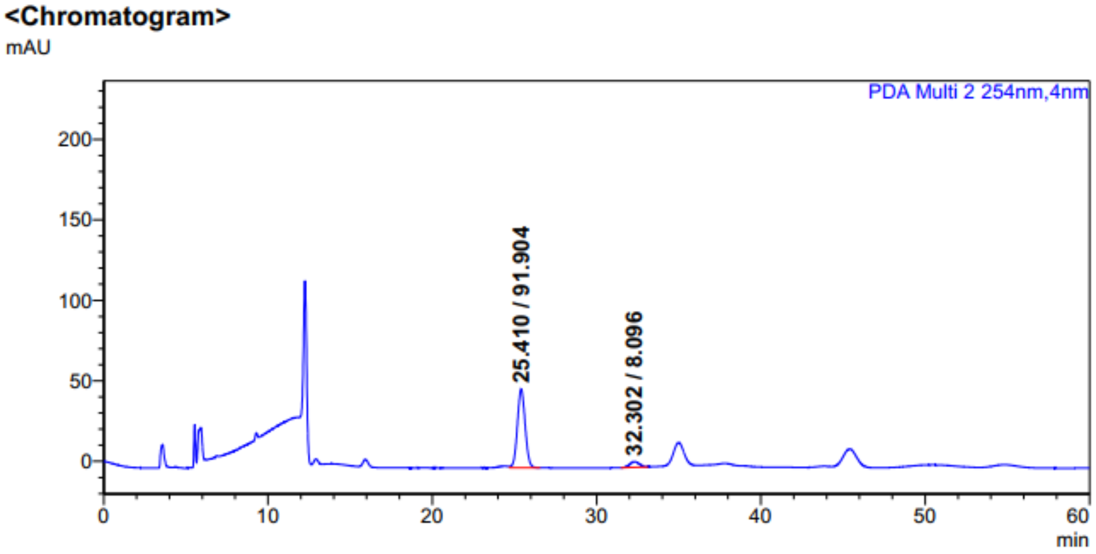

PDA Ch2 254nm

Peak#	Name	Ret. Time	Area	Area%
1		25.410	1603801	91.904
2		32.302	141286	8.096
Total			1745087	100.000

HPLC traces for reaction **without water, with 2-F-phenylboronic acid (duplicate)**

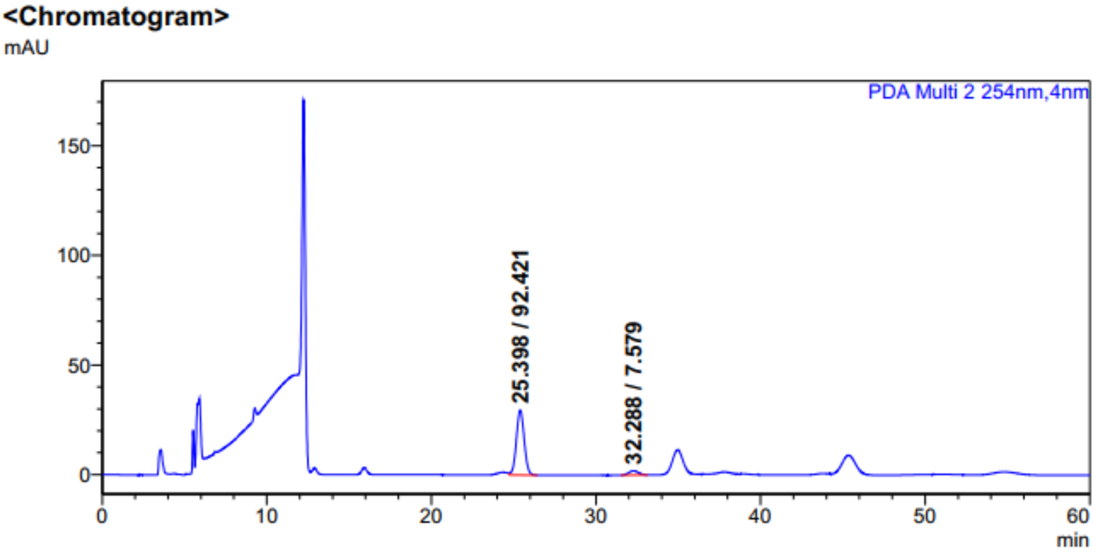

PDA Ch2 254nm

Peak#	Name	Ret. Time	Area	Area%
1		25.398	979710	92.421
2		32.288	80344	7.579
Total			1060054	100.000

10. HPLC Traces for Table S3. Boronic acid screening and blanks in neat cyclopentanone

All of the reactions were performed as duplicate following GP1 with different boronic acids. A racemic sample was first analyzed in order to determine the retention time of both enantiomers. For the chiral sample, enantiomeric excess was determined by comparing the integrated area of these two peaks. All the HPLC traces shown below are for of (S)-2-((R)-hydroxy(4-nitrophenyl)methyl)cyclopentan-1-one, yielded by different reactions as described in table S3.

HPLC traces for reaction with: **3-F-phenylboronic acid**

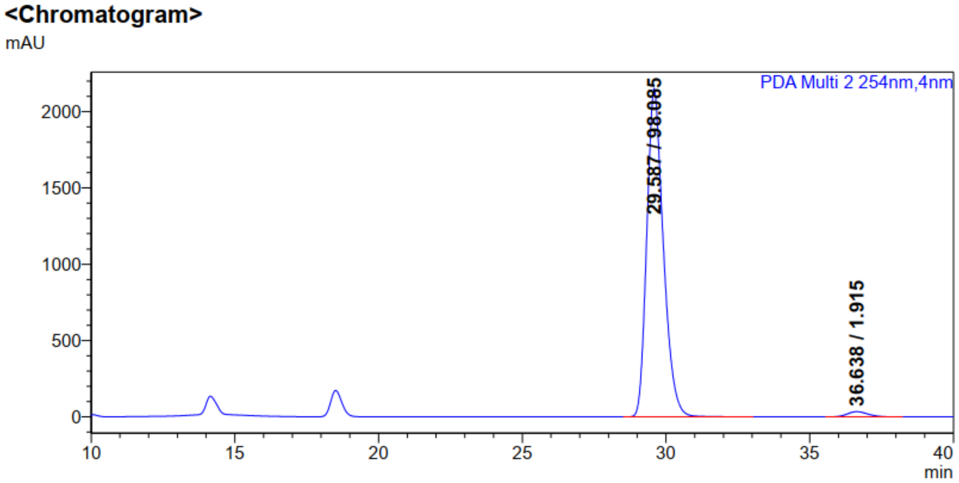

PDA Ch2 254nm

Peak#	Name	Ret. Time	Area	Area%
1		29.587	85953307	98.085
2		36.638	1678005	1.915
Total			87631312	100.000

HPLC traces for reaction with: **3-F-phenylboronic acid (duplicate)**

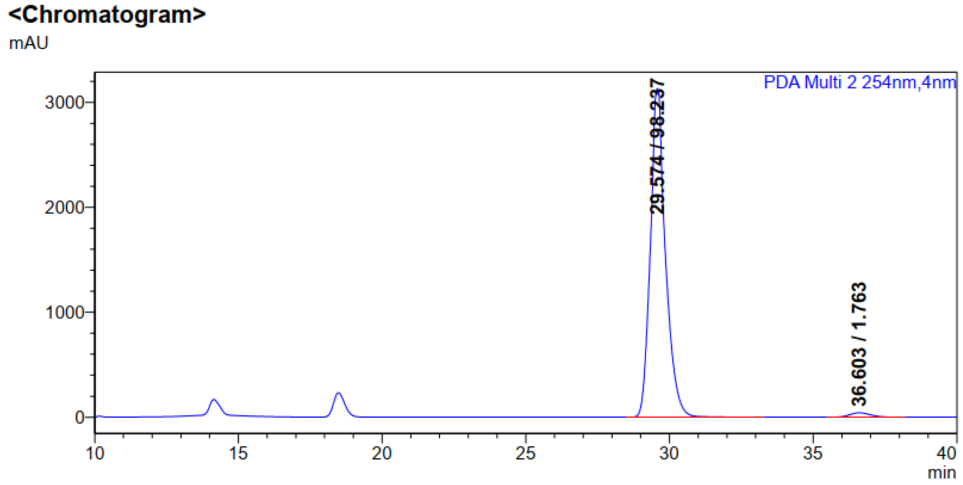

PDA Ch2 254nm

Peak#	Name	Ret. Time	Area	Area%
1		29.574	114589226	98.237
2		36.603	2055925	1.763
Total			116645150	100.000

HPLC traces for reaction with: **4-tBu-phenylboronic acid**

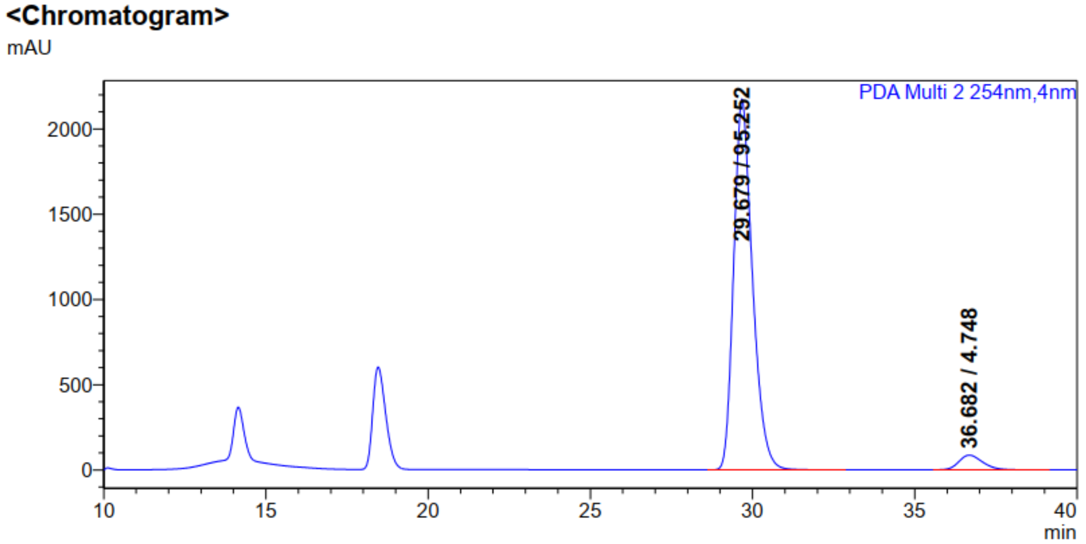

PDA Ch2 254nm

Peak#	Name	Ret. Time	Area	Area%
1		29.679	87282414	95.252
2		36.682	4351065	4.748
Total			91633479	100.000

HPLC traces for reaction with: **4-tBu-phenylboronic acid (duplicate)**

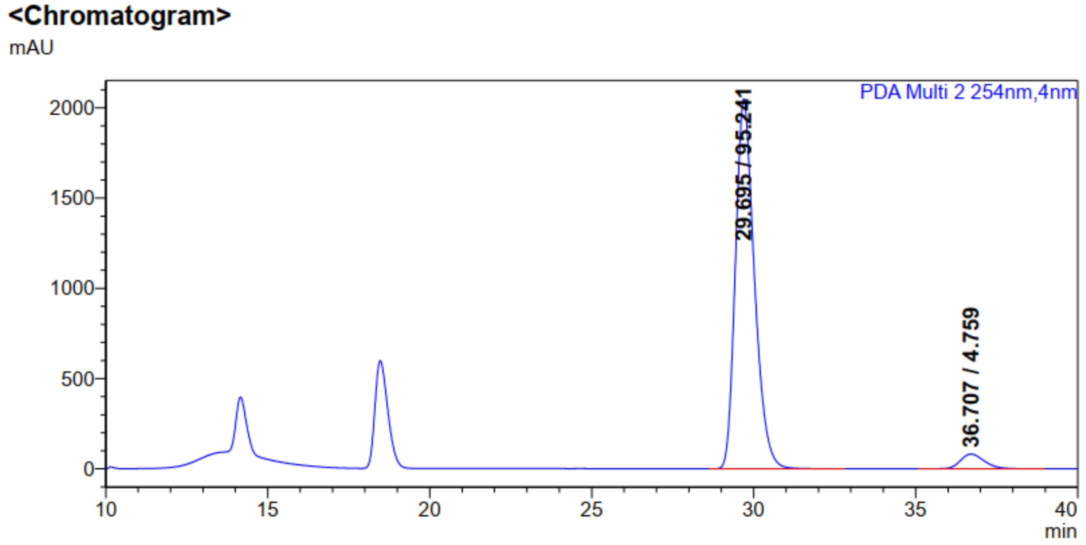

PDA Ch2 254nm

Peak#	Name	Ret. Time	Area	Area%
1		29.695	83139513	95.241
2		36.707	4154334	4.759
Total			87293847	100.000

HPLC traces for reaction with: **2,4-Me-phenylboronic acid**

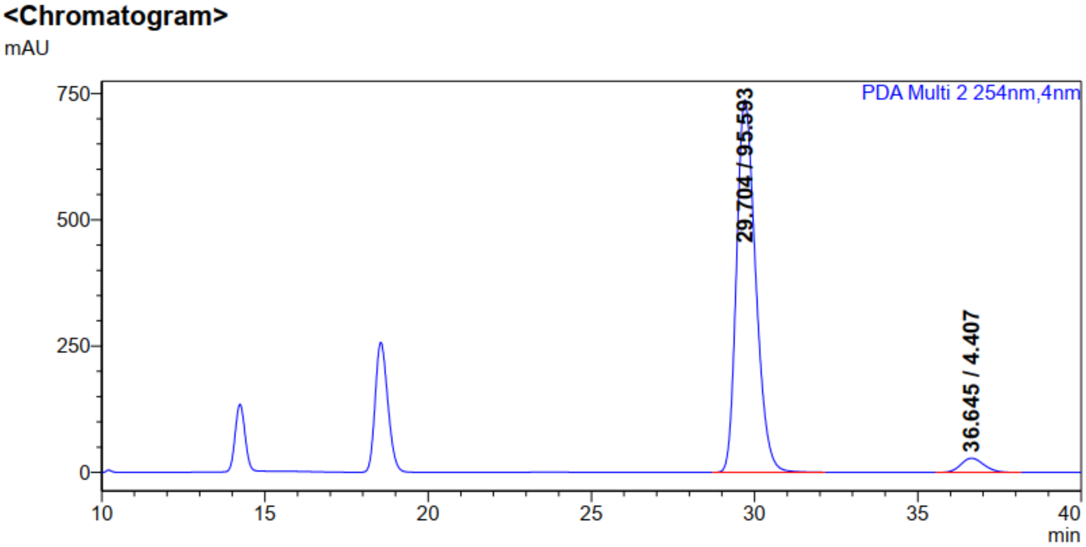

PDA Ch2 254nm

Peak#	Name	Ret. Time	Area	Area%
1		29.704	29914075	95.593
2		36.645	1378987	4.407
Total			31293062	100.000

HPLC traces for reaction with: **2,4-Me-phenylboronic acid (duplicate)**

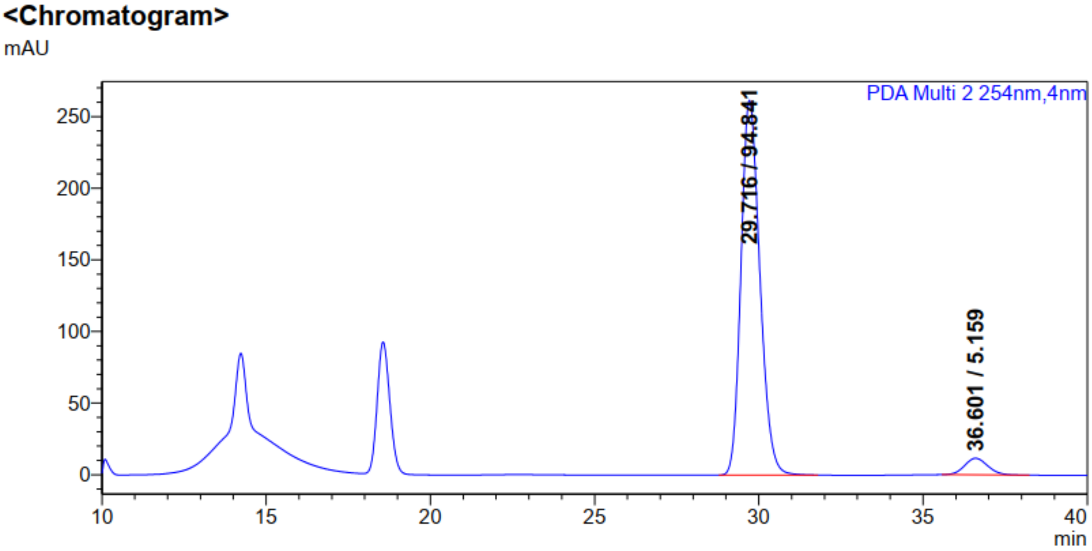

PDA Ch2 254nm

Peak#	Name	Ret. Time	Area	Area%
1		29.716	10448584	94.841
2		36.601	568392	5.159
Total			11016976	100.000

HPLC traces for reaction with: **3,5-OMe-phenylboronic acid**

<Chromatogram>

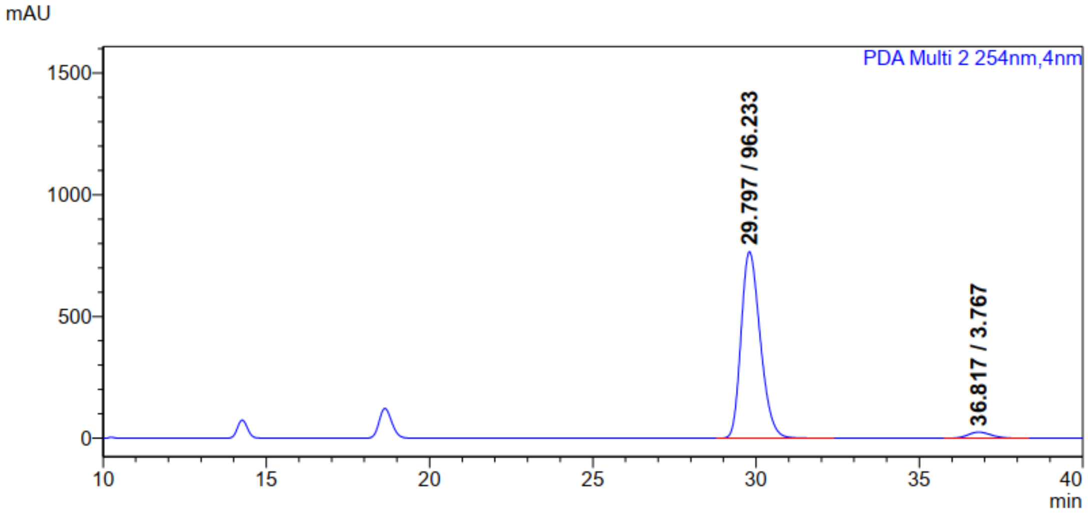

PDA Ch2 254nm

Peak#	Name	Ret. Time	Area	Area%
1		29.797	31324840	96.233
2		36.817	1226281	3.767
Total			32551121	100.000

HPLC traces for reaction with: **3,5-OMe-phenylboronic acid (duplicate)**

<Chromatogram>

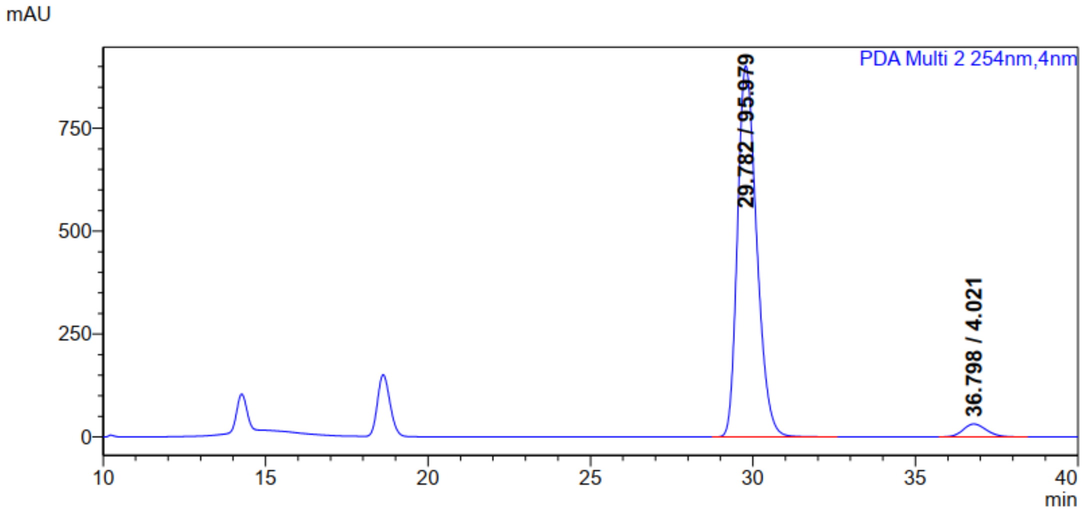

PDA Ch2 254nm

Peak#	Name	Ret. Time	Area	Area%
1		29.782	36934082	95.979
2		36.798	1547496	4.021
Total			38481578	100.000

HPLC traces for reaction with: **4-Me-phenylboronic acid**

<Chromatogram>

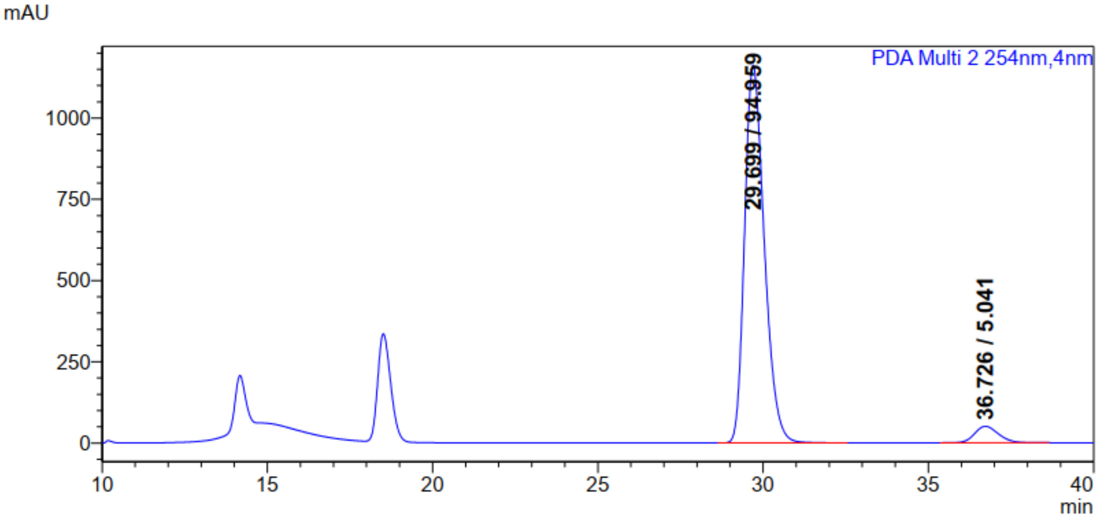

PDA Ch2 254nm

Peak#	Name	Ret. Time	Area	Area%
1		29.699	48077068	94.959
2		36.726	2552277	5.041
Total			50629345	100.000

HPLC traces for reaction with: **4-Me-phenylboronic acid (duplicate)**

<Chromatogram>

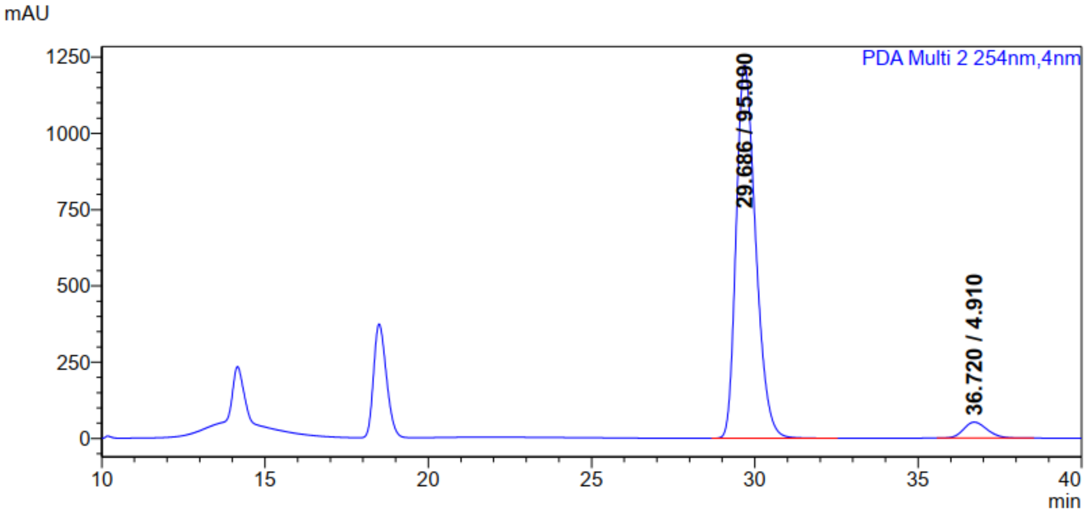

PDA Ch2 254nm

Peak#	Name	Ret. Time	Area	Area%
1		29.686	50750333	95.090
2		36.720	2620494	4.910
Total			53370826	100.000

HPLC traces for reaction with: **3-CF_3-phenylboronic acid**

<Chromatogram>

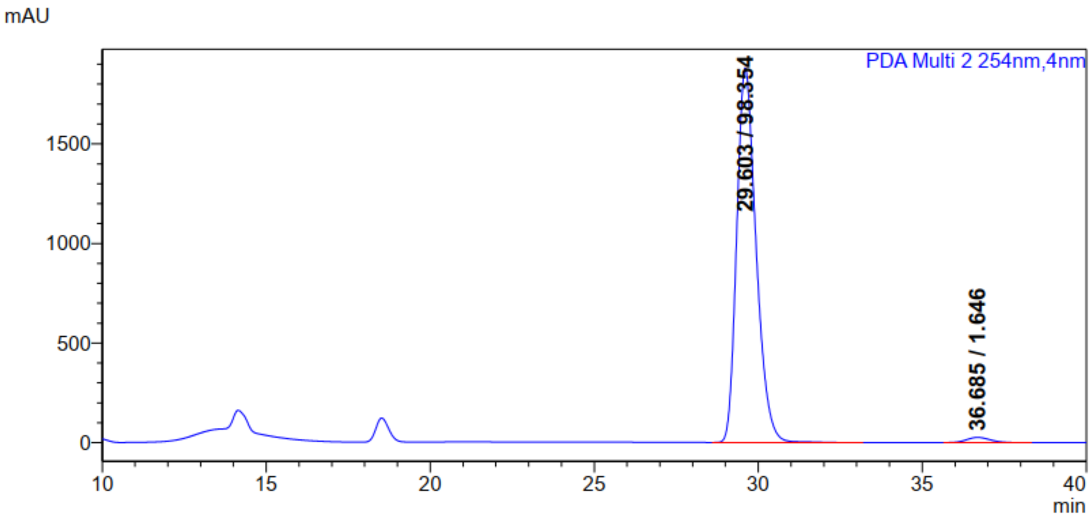

PDA Ch2 254nm

Peak#	Name	Ret. Time	Area	Area%
1		29.603	77037262	98.354
2		36.685	1289482	1.646
Total			78326744	100.000

HPLC traces for reaction with: **3-CF_3-phenylboronic acid (duplicate)**

<Chromatogram>

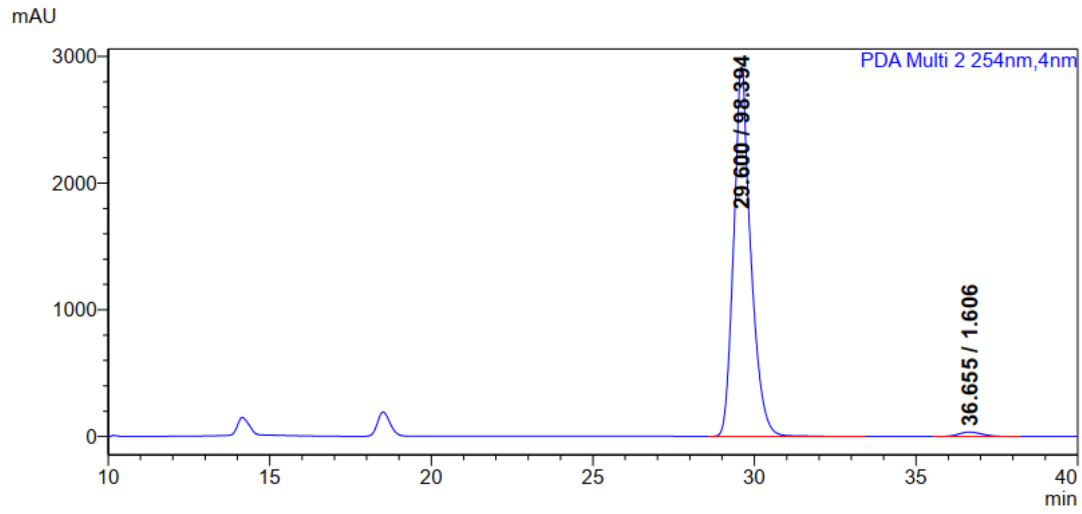

PDA Ch2 254nm

Peak#	Name	Ret. Time	Area	Area%
1		29.600	106872736	98.394
2		36.655	1744636	1.606
Total			108617372	100.000

HPLC traces for reaction with: **2-Naphthalene-phenylboronic acid**

<Chromatogram>

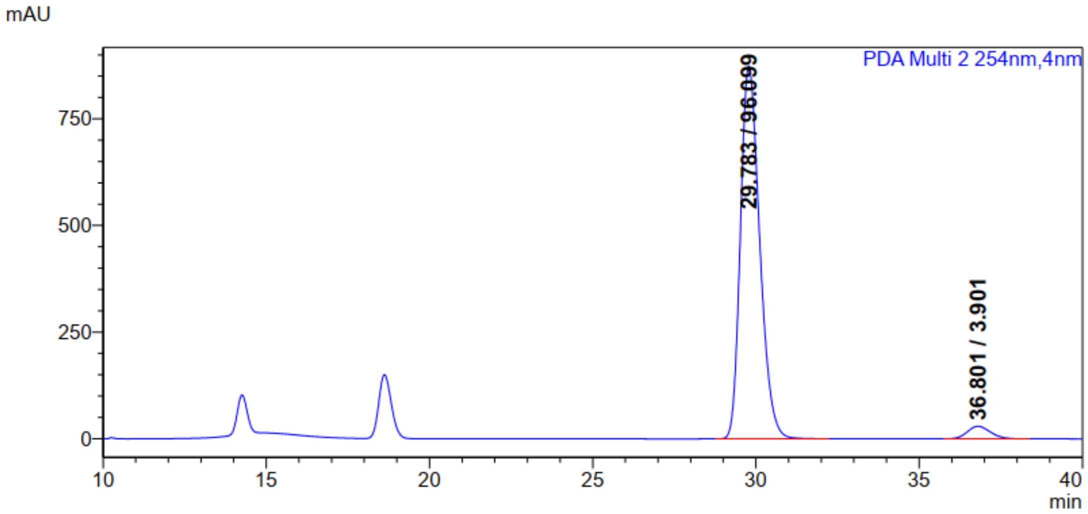

PDA Ch2 254nm

Peak#	Name	Ret. Time	Area	Area%
1		29.783	35739364	96.099
2		36.801	1450717	3.901
Total			37190081	100.000

HPLC traces for reaction with: **2-Naphthalene-phenylboronic acid (duplicate)**

<Chromatogram>

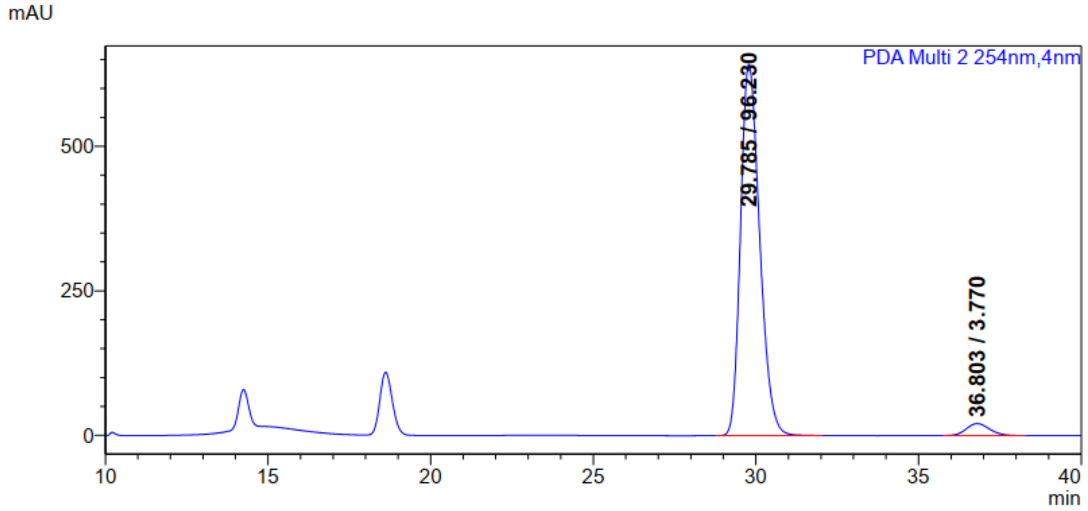

PDA Ch2 254nm

Peak#	Name	Ret. Time	Area	Area%
1		29.785	26198375	96.230
2		36.803	1026261	3.770
Total			27224637	100.000

HPLC traces for reaction with: **4-CF_3-phenylboronic acid**

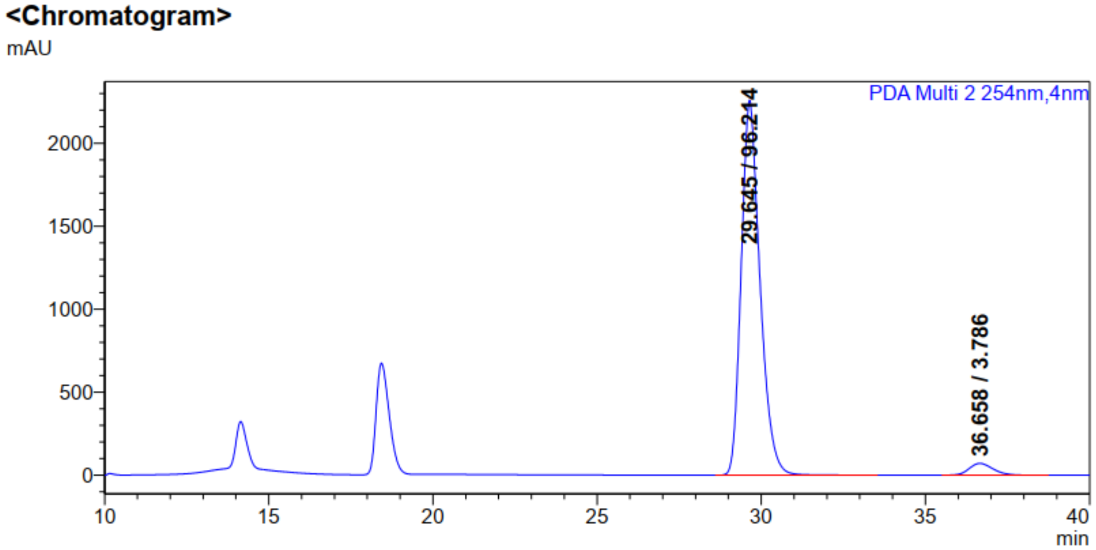

PDA Ch2 254nm

Peak#	Name	Ret. Time	Area	Area%
1		29.645	89710948	96.214
2		36.658	3529693	3.786
Total			93240641	100.000

HPLC traces for reaction with: **4-CF_3-phenylboronic acid (duplicate)**

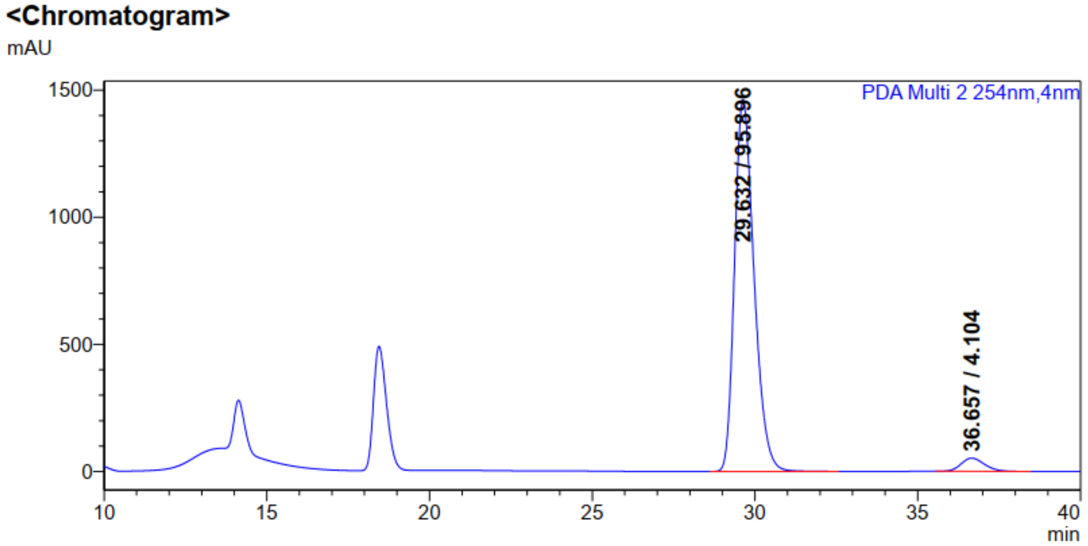

PDA Ch2 254nm

Peak#	Name	Ret. Time	Area	Area%
1		29.632	60672321	95.896
2		36.657	2596640	4.104
Total			63268960	100.000

HPLC traces for reaction with: **4-F-phenylboronic acid**

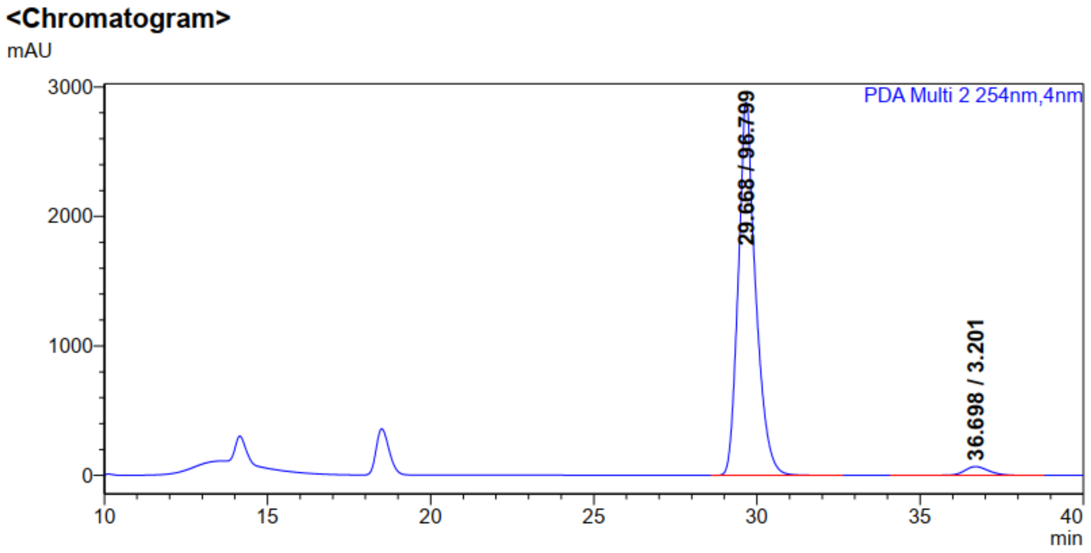

PDA Ch2 254nm

Peak#	Name	Ret. Time	Area	Area%
1		29.668	106455889	96.799
2		36.698	3520684	3.201
Total			109976573	100.000

HPLC traces for reaction with: **4-F-phenylboronic acid (duplicate)**

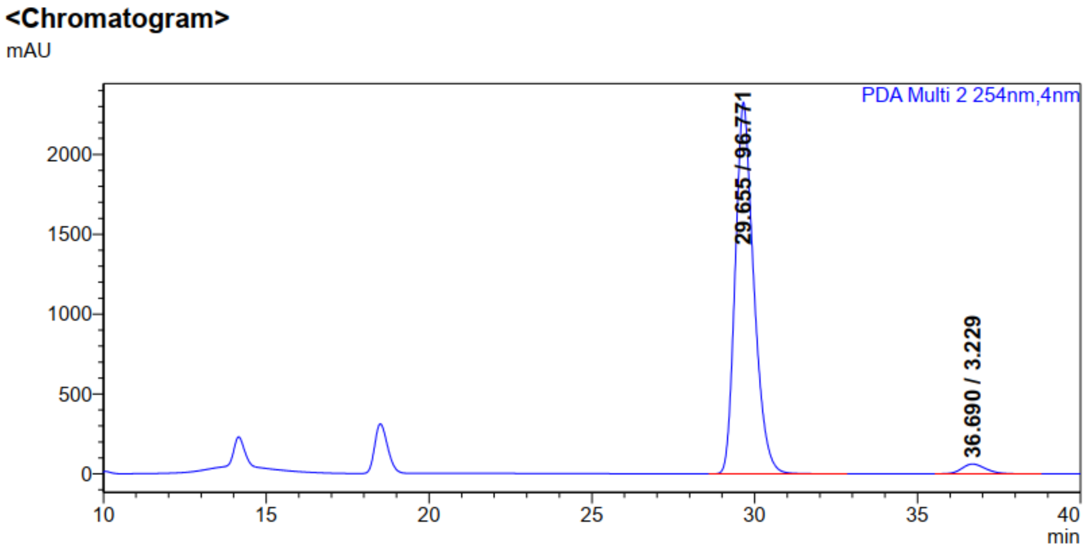

PDA Ch2 254nm

Peak#	Name	Ret. Time	Area	Area%
1		29.655	91811091	96.771
2		36.690	3063664	3.229
Total			94874754	100.000

HPLC traces for reaction with: **4-OMe-phenylboronic acid**

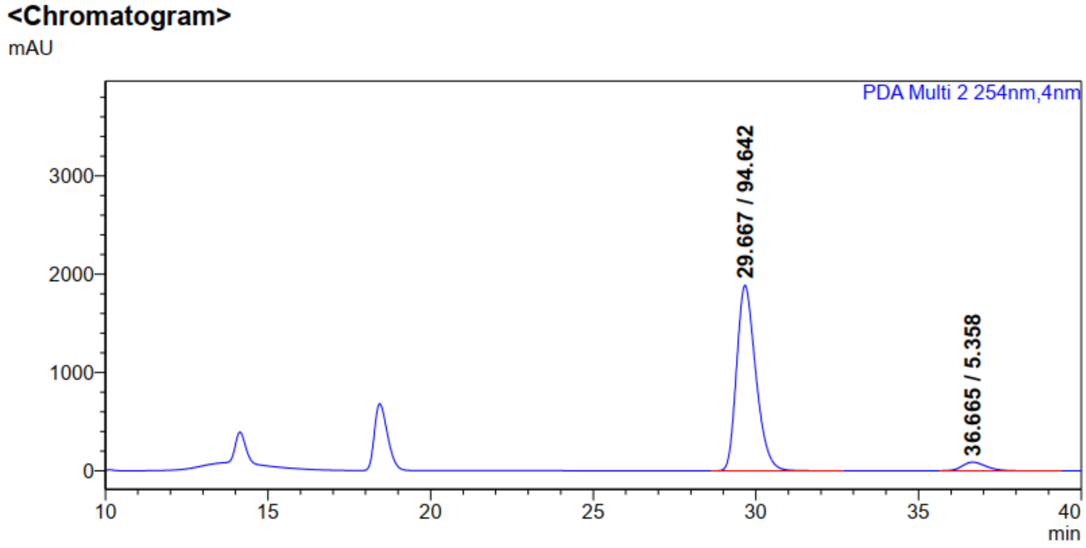

PDA Ch2 254nm

Peak#	Name	Ret. Time	Area	Area%
1		29.667	77540341	94.642
2		36.665	4389705	5.358
Total			81930045	100.000

HPLC traces for reaction with: **4-OMe-phenylboronic acid (duplicate)**

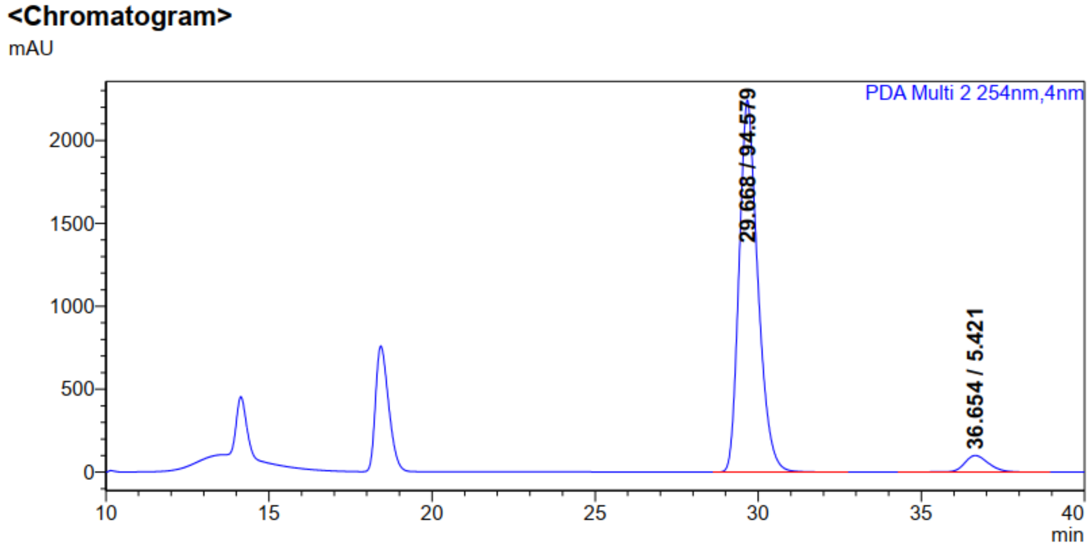

PDA Ch2 254nm

Peak#	Name	Ret. Time	Area	Area%
1		29.668	89432300	94.579
2		36.654	5126467	5.421
Total			94558767	100.000

HPLC traces for reaction with: **3-Me-phenylboronic acid**

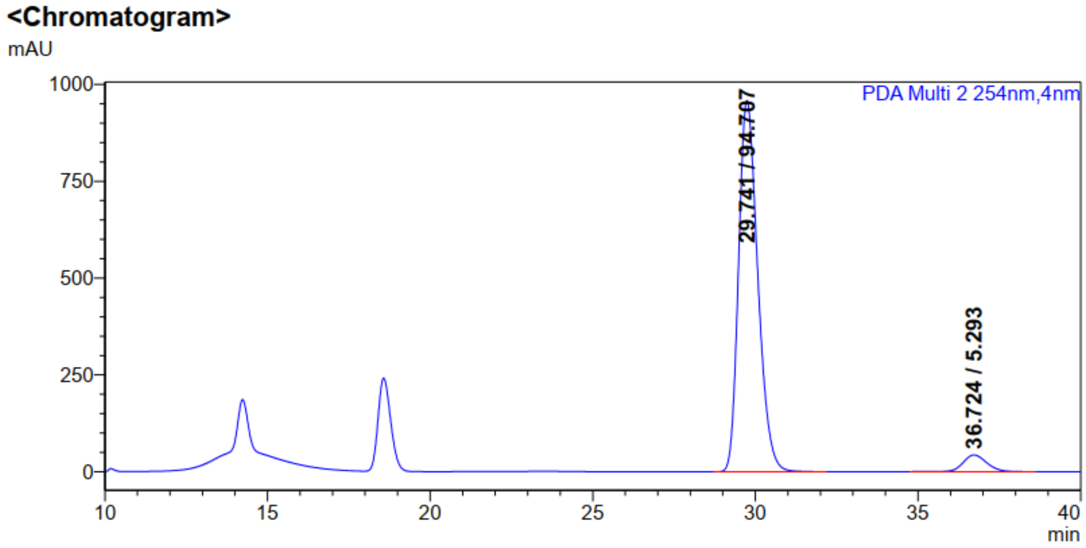

PDA Ch2 254nm

Peak#	Name	Ret. Time	Area	Area%
1		29.741	39221797	94.707
2		36.724	2192016	5.293
Total			41413814	100.000

HPLC traces for reaction with: **3-Me-phenylboronic acid (duplicate)**

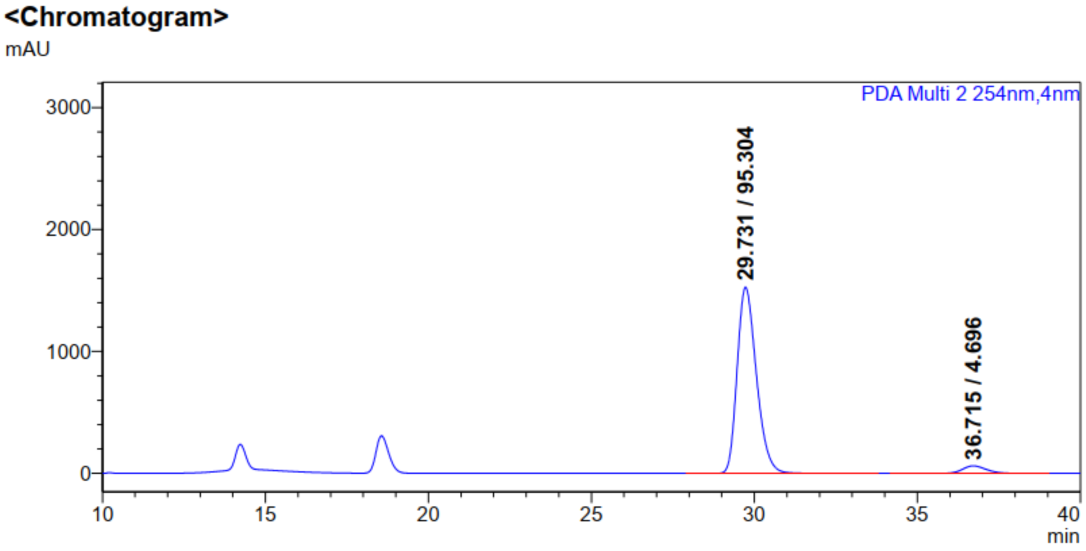

PDA Ch2 254nm

Peak#	Name	Ret. Time	Area	Area%
1		29.731	62696494	95.304
2		36.715	3089419	4.696
Total			65785912	100.000

HPLC traces for reaction with: **2-Me-phenylboronic acid**

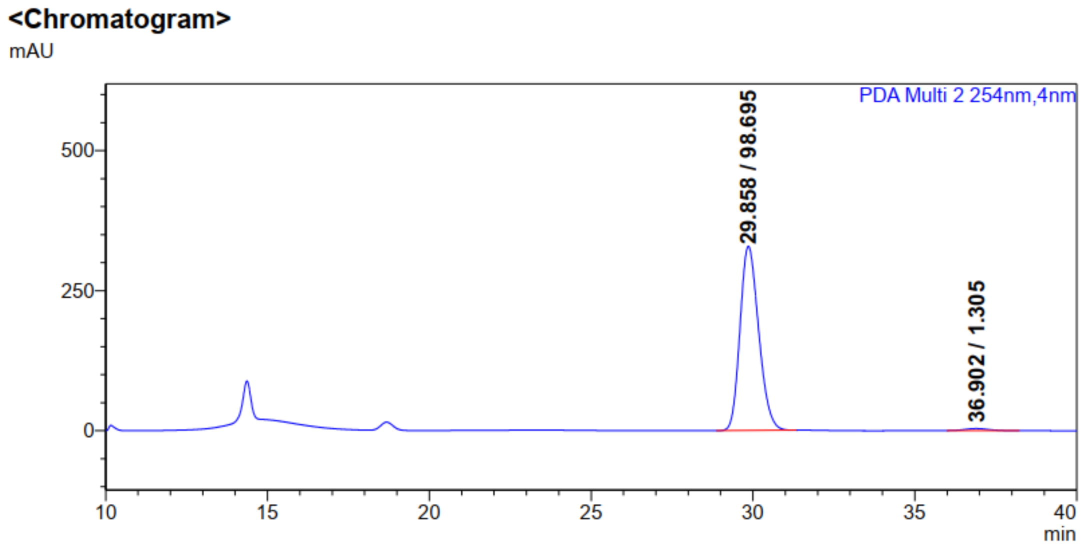

PDA Ch2 254nm

Peak#	Name	Ret. Time	Area	Area%
1		29.858	13194141	98.695
2		36.902	174511	1.305
Total			13368652	100.000

HPLC traces for reaction with: **2-Me-phenylboronic acid (duplicate)**

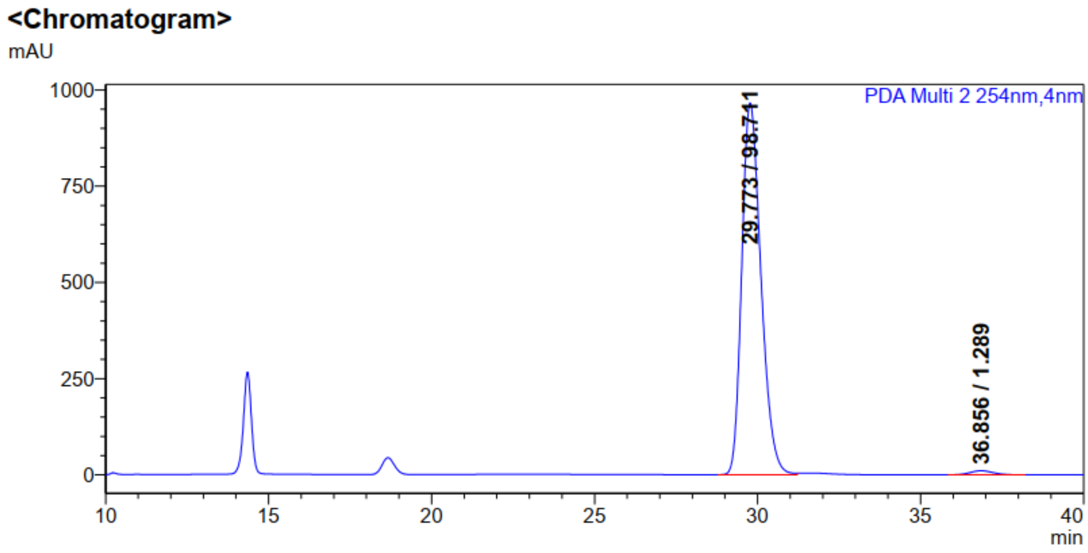

PDA Ch2 254nm

Peak#	Name	Ret. Time	Area	Area%
1		29.773	39463928	98.711
2		36.856	515165	1.289
Total			39979093	100.000

HPLC traces for reaction with: **Ph-phenylboronic acid**

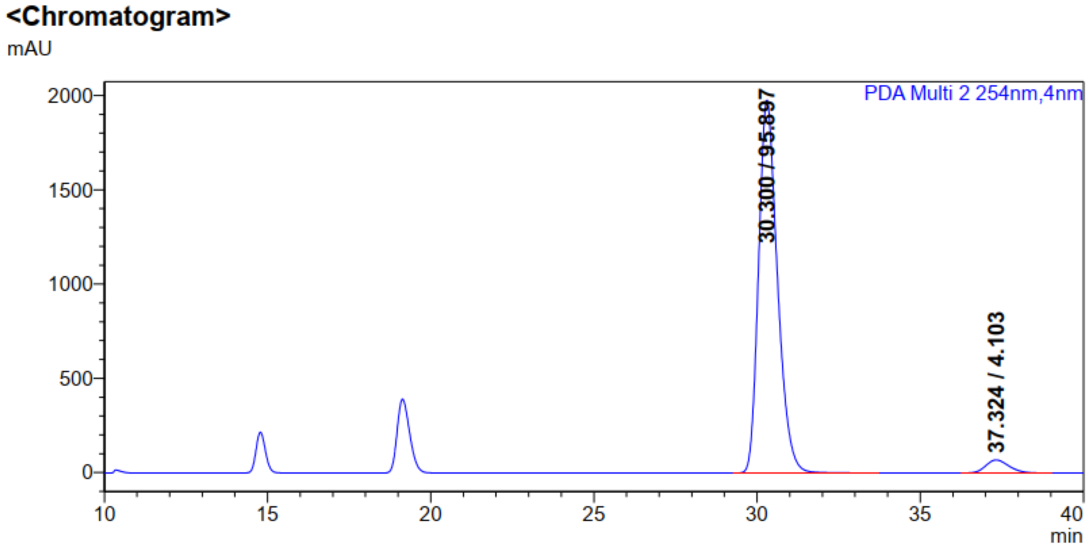

PDA Ch2 254nm

Peak#	Name	Ret. Time	Area	Area%
1		30.300	79258608	95.897
2		37.324	3390787	4.103
Total			82649396	100.000

HPLC traces for reaction with: **Ph-phenylboronic acid (duplicate)**

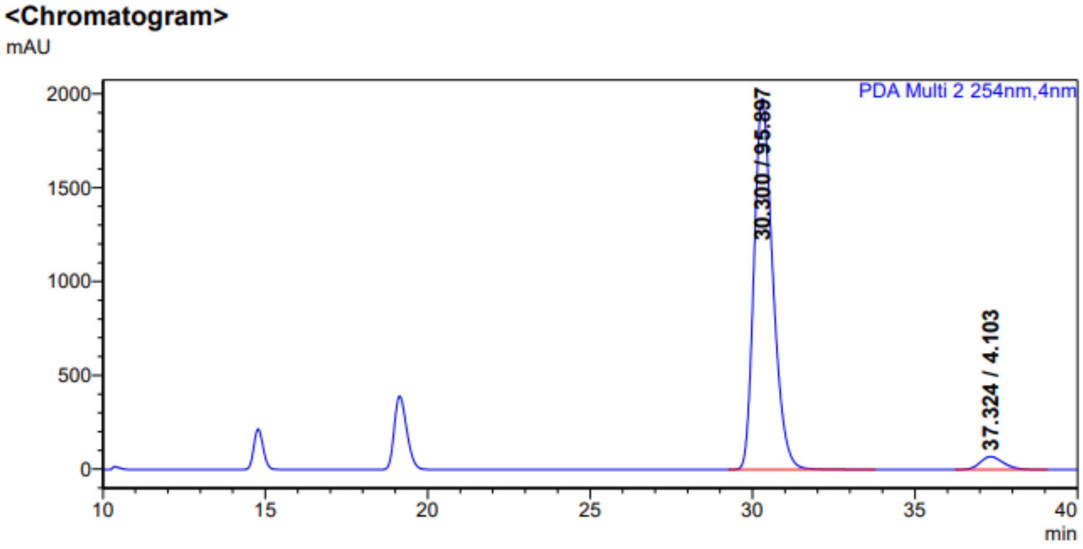

PDA Ch2 254nm

Peak#	Name	Ret. Time	Area	Area%
1		30.300	79258608	95.897
2		37.324	3390787	4.103
Total			82649396	100.000

HPLC traces for reaction with: **2-F-phenylboronic acid**

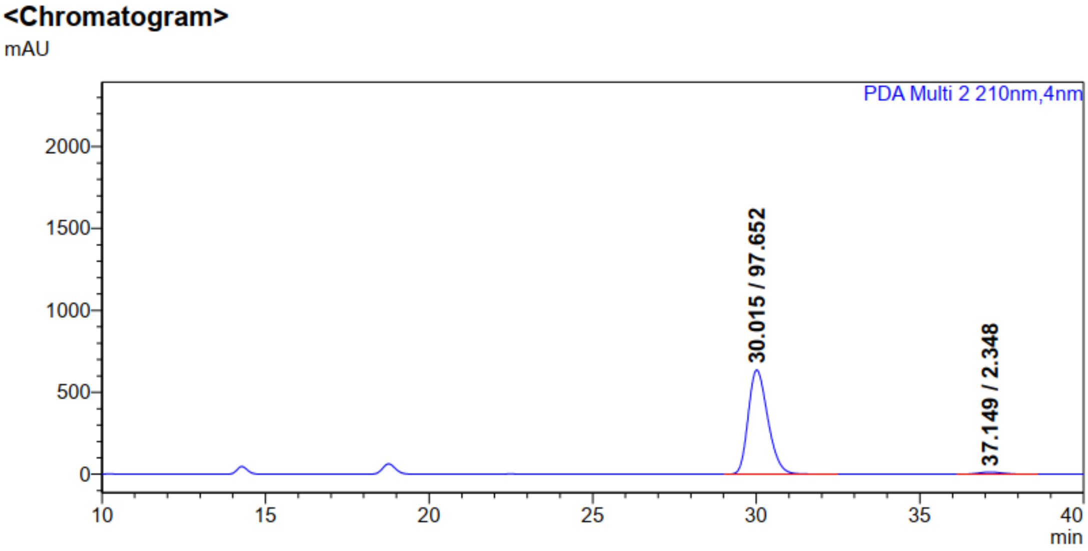

PDA Ch2 210nm

Peak#	Name	Ret. Time	Area	Area%
1		30.015	26489424	97.652
2		37.149	636956	2.348
Total			27126380	100.000

HPLC traces for reaction with**: 2-F-phenylboronic acid (duplicate)**

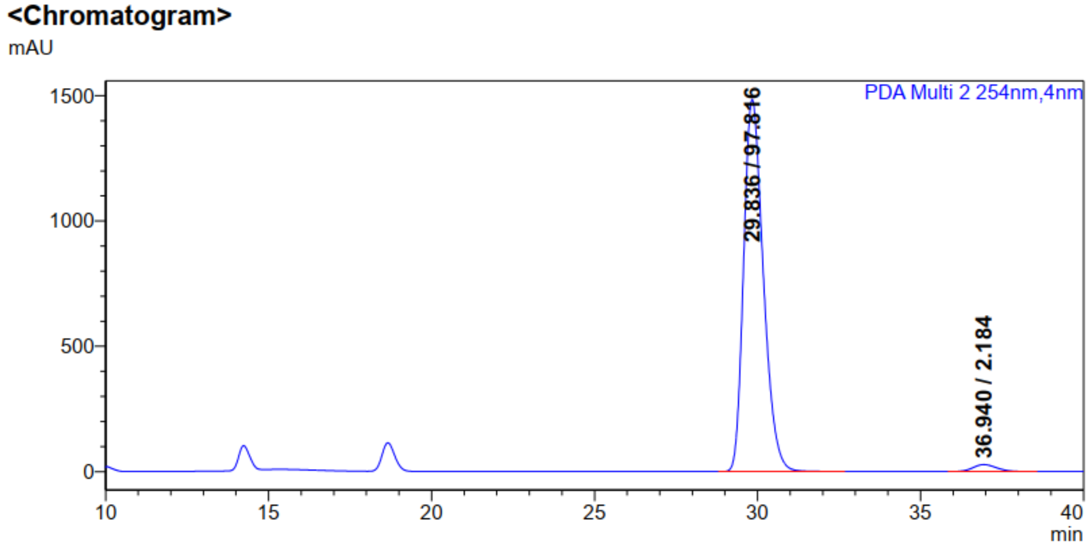

PDA Ch2 254nm

Peak#	Name	Ret. Time	Area	Area%
1		29.836	62096717	97.816
2		36.940	1386767	2.184
Total			63483484	100.000

HPLC traces for reaction **without boronic acid**

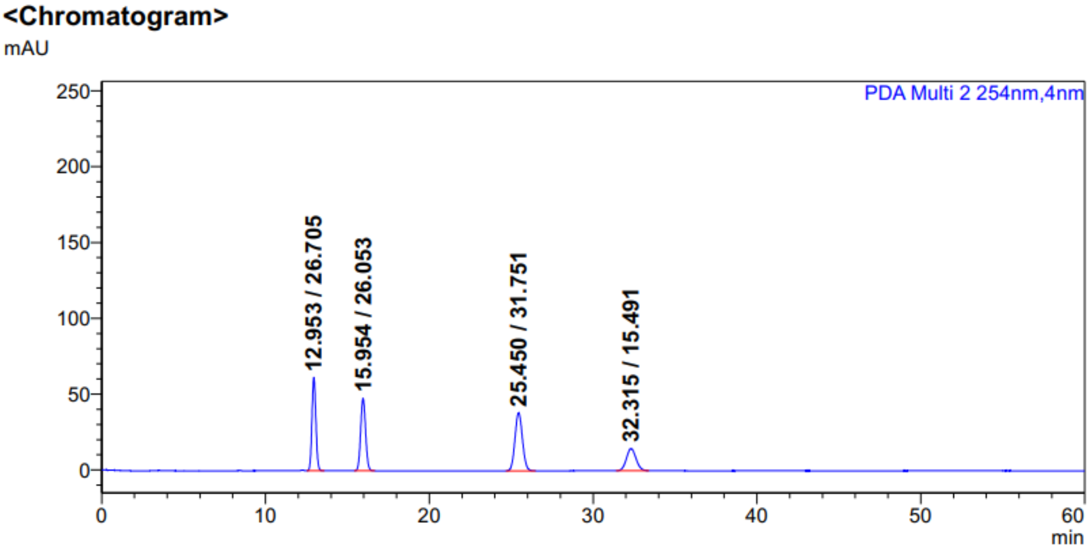

PDA Ch1 230nm

Peak#	Name	Ret. Time	Area	Area%
1		12.953	362912	27.703
2		15.953	349884	26.708
3		25.450	422631	32.261
4		32.313	174601	13.328
Total			1310027	100.000

HPLC traces for reaction **without boronic acid (duplicate)**

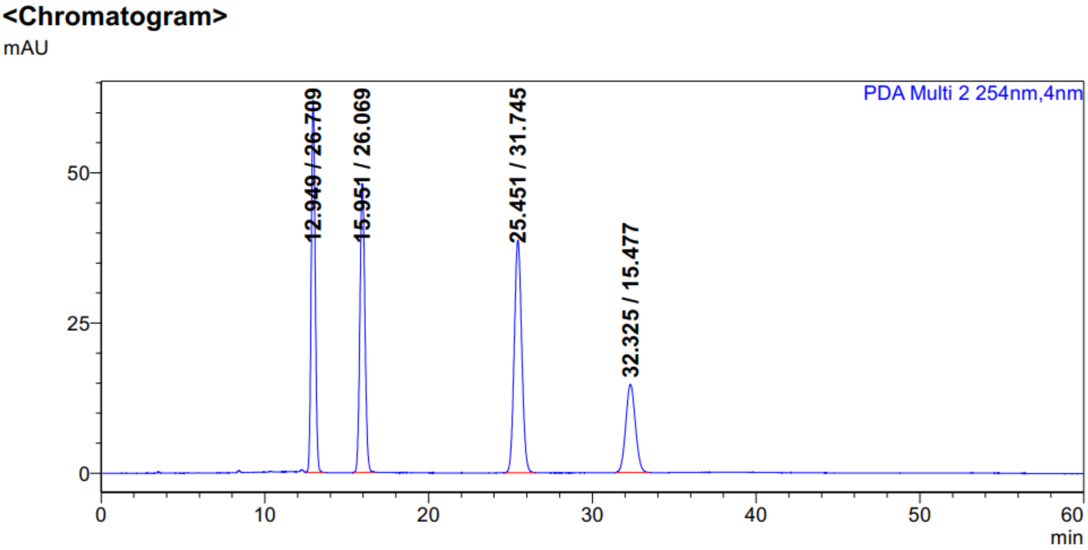

PDA Ch2 254nm

Peak#	Name	Ret. Time	Area	Area%
1		12.949	1033131	26.709
2		15.951	1008341	26.069
3		25.451	1227893	31.745
4		32.325	598667	15.477
Total			3868032	100.000

HPLC traces for reaction **without water, with 2-F-phenylboronic acid**

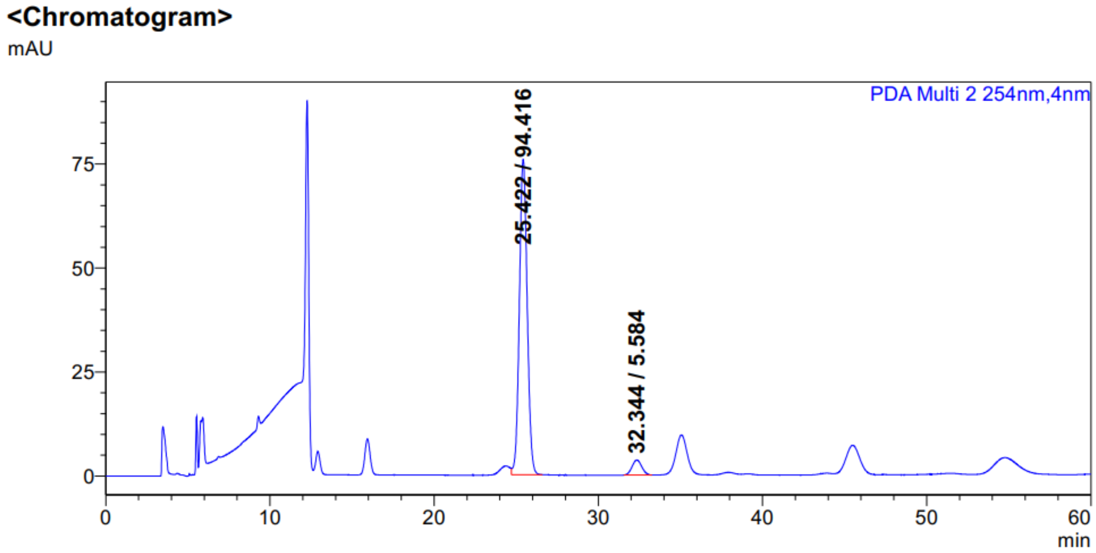

PDA Ch2 254nm

Peak#	Name	Ret. Time	Area	Area%
1		25.422	2507013	94.416
2		32.344	148278	5.584
Total			2655290	100.000

HPLC traces for reaction **without water, with 2-F-phenylboronic acid**

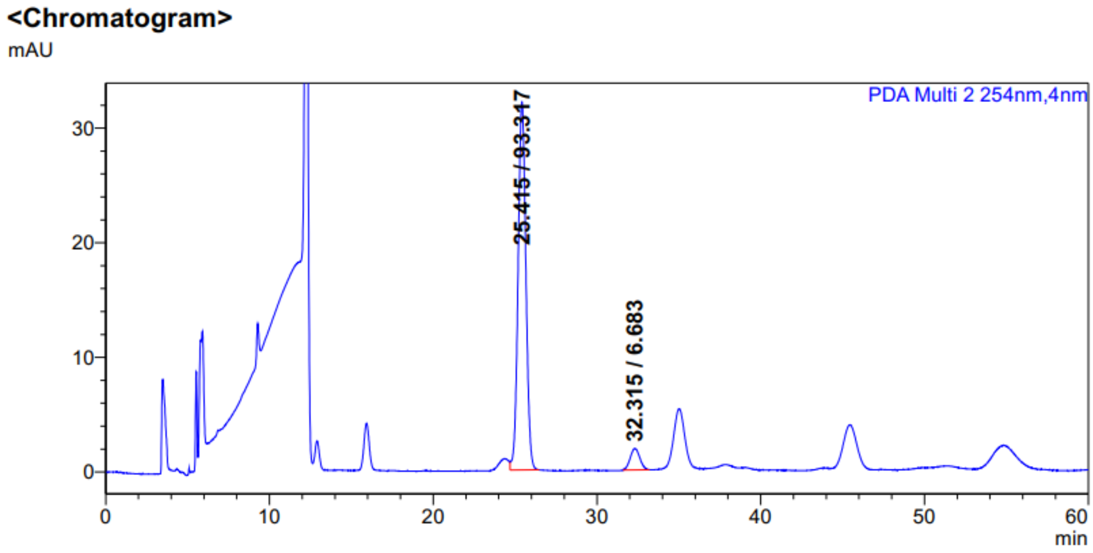

PDA Ch2 254nm

Peak#	Name	Ret. Time	Area	Area%
1		25.415	1059350	93.317
2		32.315	75872	6.683
Total			1135222	100.000

11. HPLC Traces for Table S4. Boronic acid screening and blanks in MeOH

All of the reactions were performed as duplicate following GP1 with different boronic acids. A racemic sample was first analyzed in order to determine the retention time of both enantiomers. For the chiral sample, enantiomeric excess was determined by comparing the integrated area of these two peaks. All the HPLC traces shown below are for of (S)-2-((R)-hydroxy(4-nitrophenyl)methyl)cyclopentan-1-one, yielded by different reactions as described in table S4.

HPLC traces for reaction with: **3-F-phenylboronic acid**

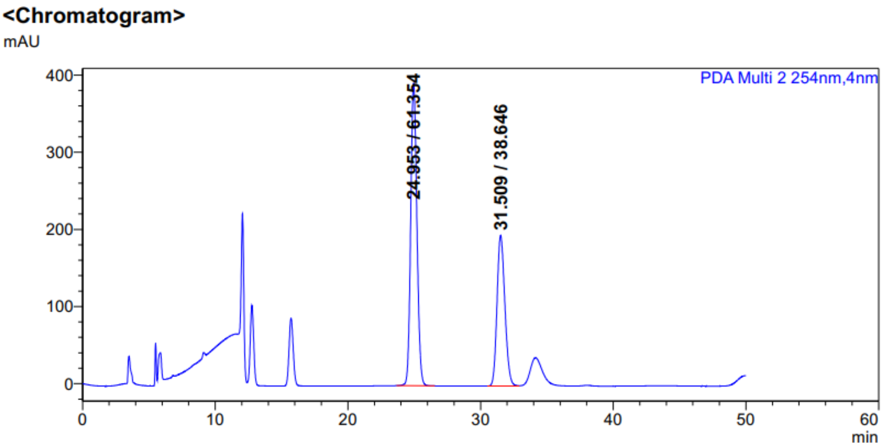

PDA Ch2 254nm

Peak#	Name	Ret. Time	Area	Area%
1		24.953	12760507	61.354
2		31.509	8037630	38.646
Total			20798138	100.000

HPLC traces for reaction with: **3-F-phenylboronic acid (duplicate)**

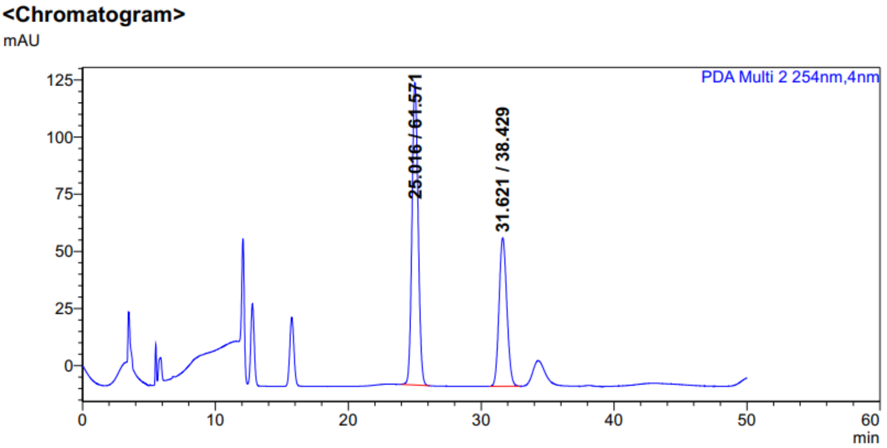

PDA Ch2 254nm

Peak#	Name	Ret. Time	Area	Area%
1		25.016	4253702	61.571
2		31.621	2654953	38.429
Total			6908655	100.000

HPLC traces for reaction with: **3,5-F-phenylboronic acid**

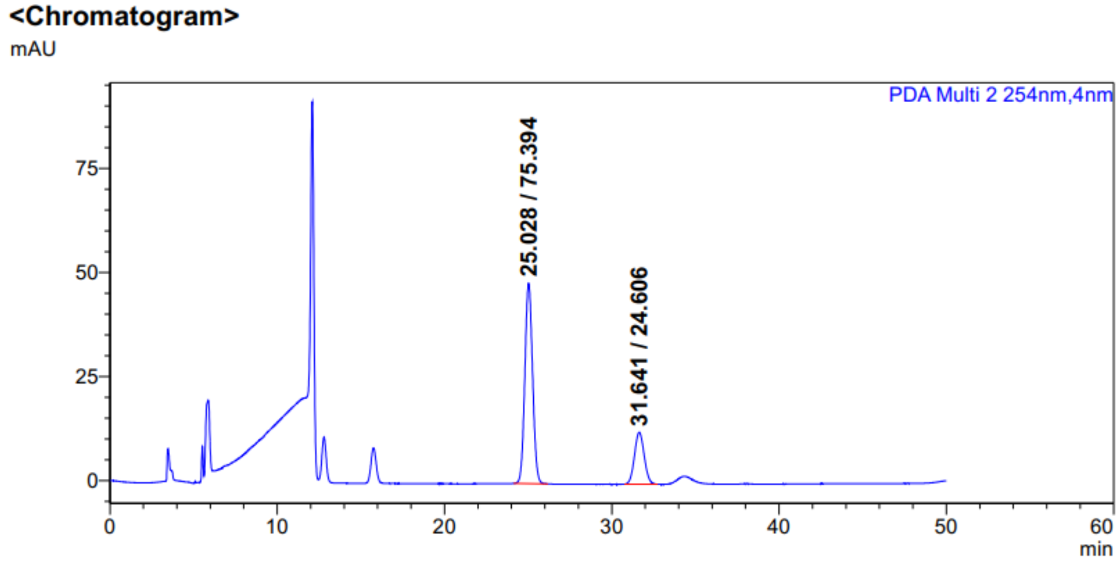

PDA Ch2 254nm

Peak#	Name	Ret. Time	Area	Area%
1		25.028	1544401	75.394
2		31.641	504030	24.606
Total			2048431	100.000

HPLC traces for reaction with: **3,5-F-phenylboronic acid (duplicate)**

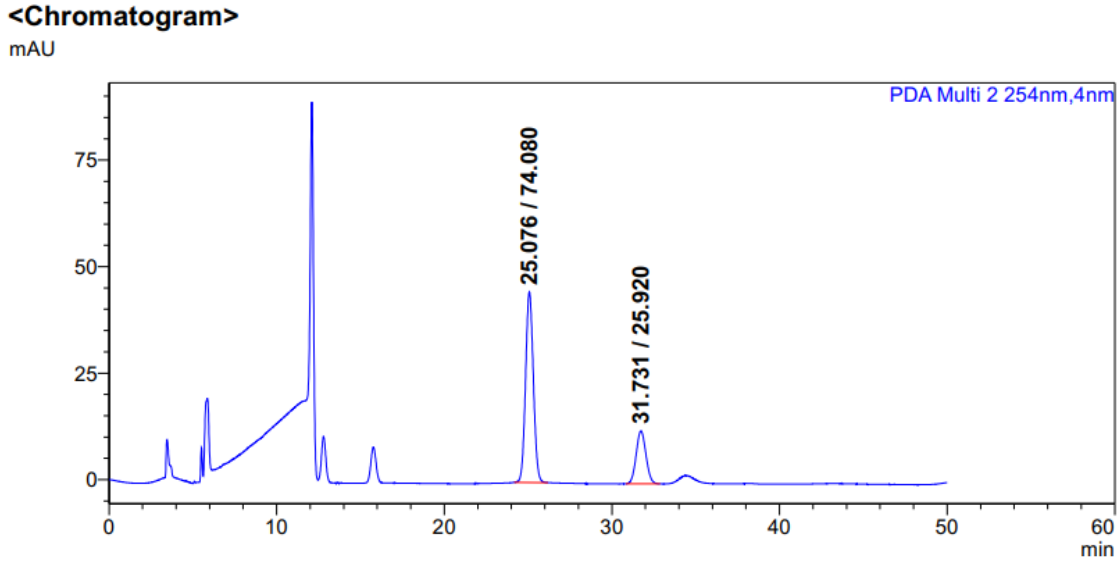

PDA Ch2 254nm

Peak#	Name	Ret. Time	Area	Area%
1		25.076	1439824	74.080
2		31.731	503774	25.920
Total			1943598	100.000

HPLC traces for reaction with: **4-tBu-phenylboronic acid**

<Chromatogram>

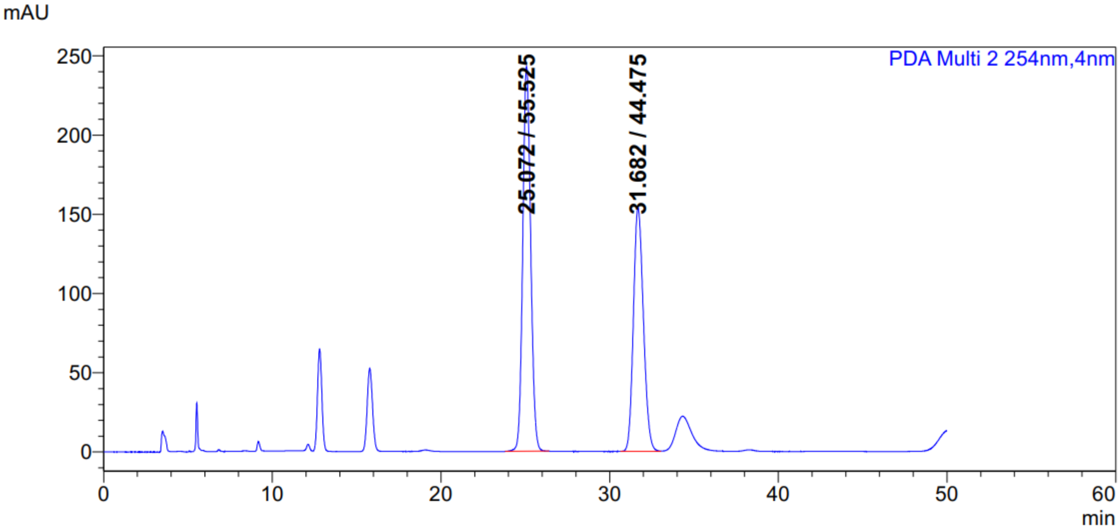

PDA Ch2 254nm

Peak#	Name	Ret. Time	Area	Area%
1		25.072	7920355	55.525
2		31.682	6344201	44.475
Total			14264556	100.000

HPLC traces for reaction with: **4-tBu-phenylboronic acid (duplicate)**

<Chromatogram>

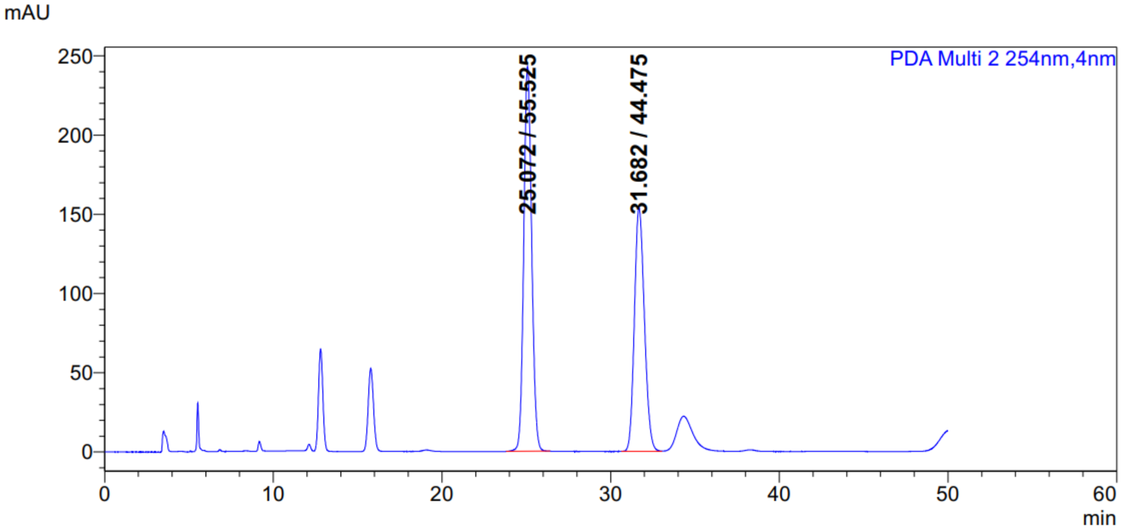

PDA Ch2 254nm

Peak#	Name	Ret. Time	Area	Area%
1		25.072	7920355	55.525
2		31.682	6344201	44.475
Total			14264556	100.000

HPLC traces for reaction with: **2,4-Me-phenylboronic acid**

<Chromatogram>

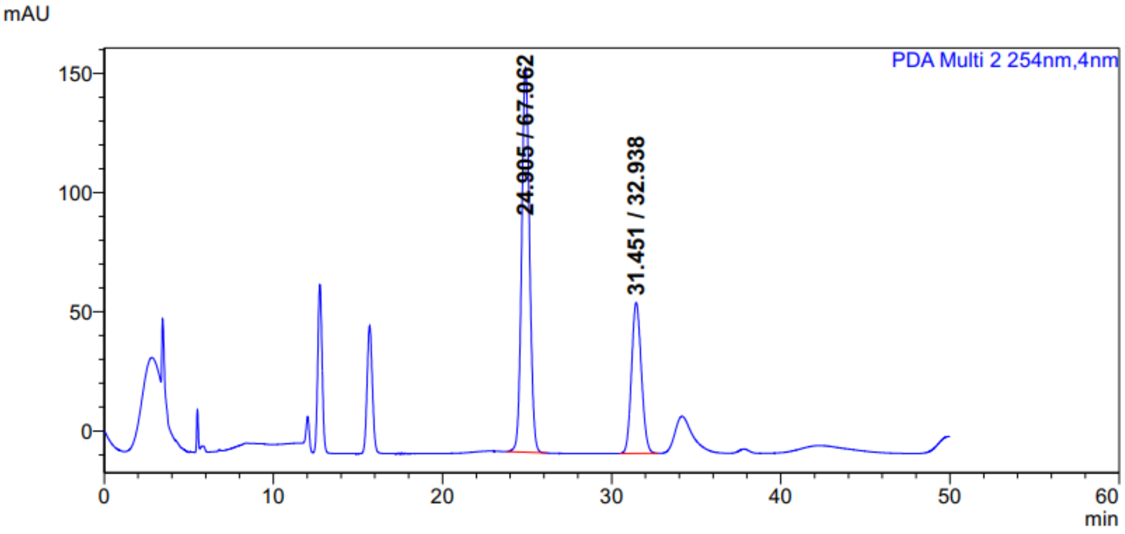

PDA Ch2 254nm

Peak#	Name	Ret. Time	Area	Area%
1		24.905	5220492	67.062
2		31.451	2564085	32.938
Total			7784577	100.000

HPLC traces for reaction with: **2,4-Me-phenylboronic acid (duplicate)**

<Chromatogram>

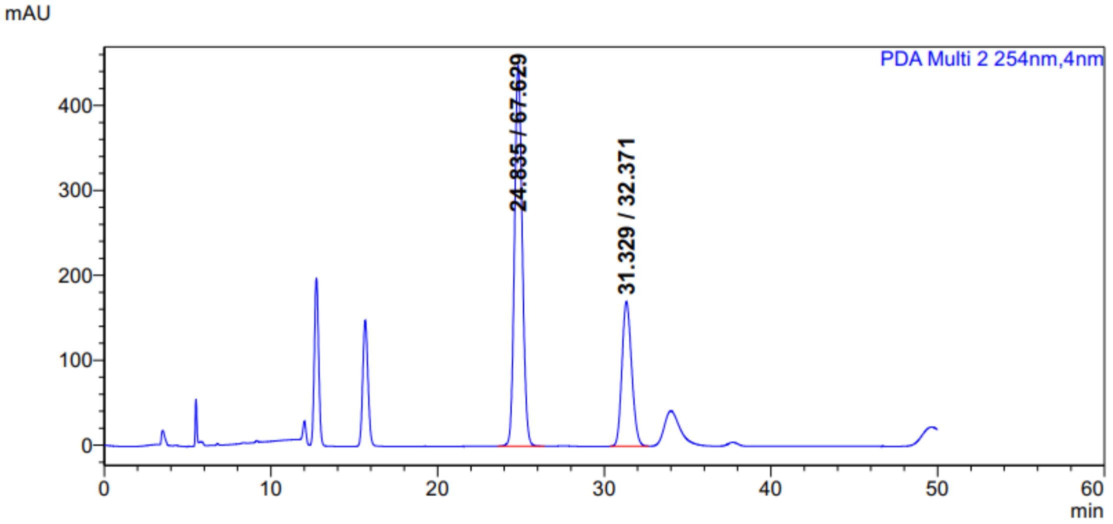

PDA Ch2 254nm

Peak#	Name	Ret. Time	Area	Area%
1		24.835	14480989	67.629
2		31.329	6931379	32.371
Total			21412368	100.000

HPLC traces for reaction with: **3,5-OMe-phenylboronic acid**

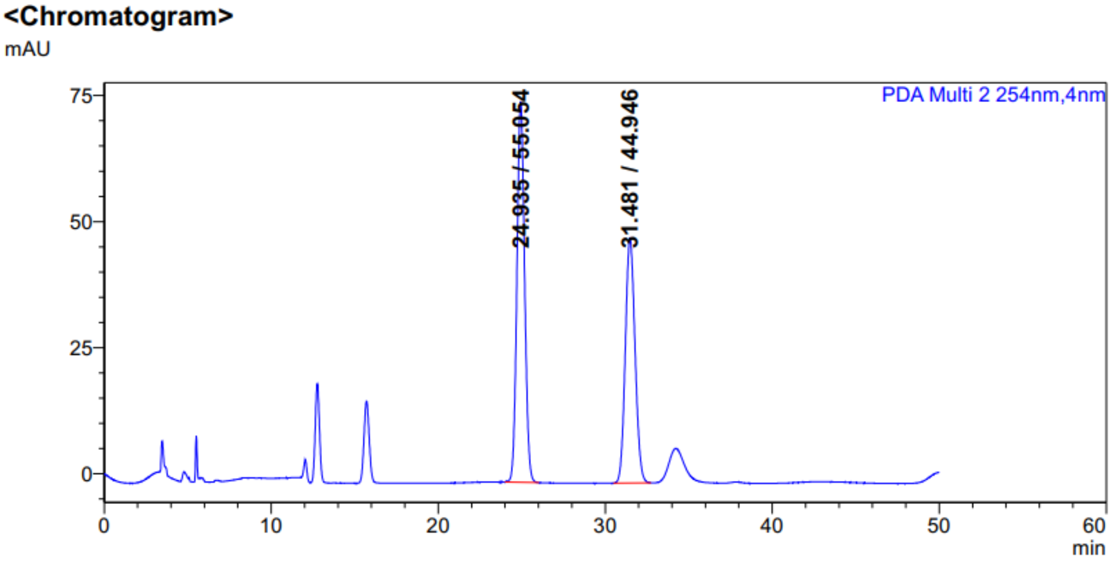

PDA Ch2 254nm

Peak#	Name	Ret. Time	Area	Area%
1		24.935	2406273	55.054
2		31.481	1964495	44.946
Total			4370768	100.000

HPLC traces for reaction with: **3,5-OMe-phenylboronic acid (duplicate)**

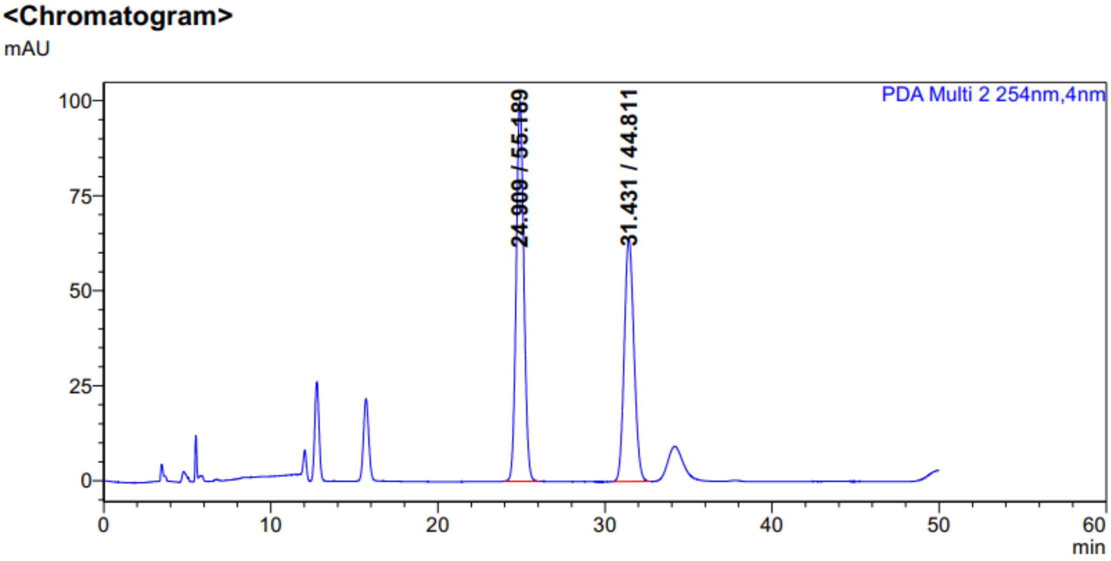

PDA Ch2 254nm

Peak#	Name	Ret. Time	Area	Area%
1		24.909	3185509	55.189
2		31.431	2586457	44.811
Total			5771966	100.000

HPLC traces for reaction with: **4-Me-phenylboronic acid**

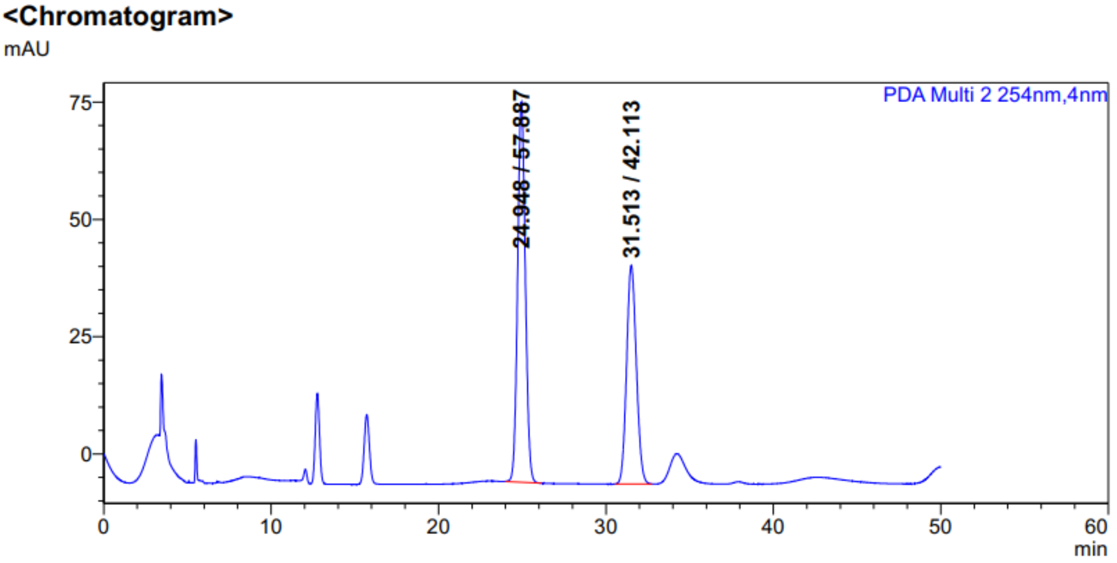

PDA Ch2 254nm

Peak#	Name	Ret. Time	Area	Area%
1		24.948	2592779	57.887
2		31.513	1886238	42.113
Total			4479017	100.000

HPLC traces for reaction with: **4-Me-phenylboronic acid (duplicate)**

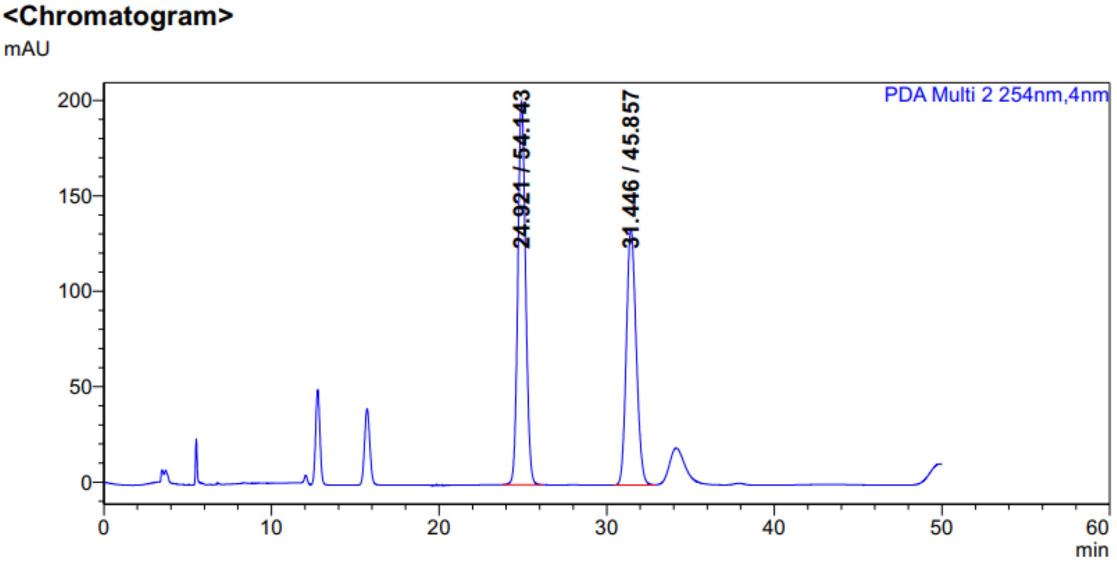

PDA Ch2 254nm

Peak#	Name	Ret. Time	Area	Area%
1		24.921	6456011	54.143
2		31.446	5468036	45.857
Total			11924047	100.000

HPLC traces for reaction with: **3-CF_3-phenylboronic acid**

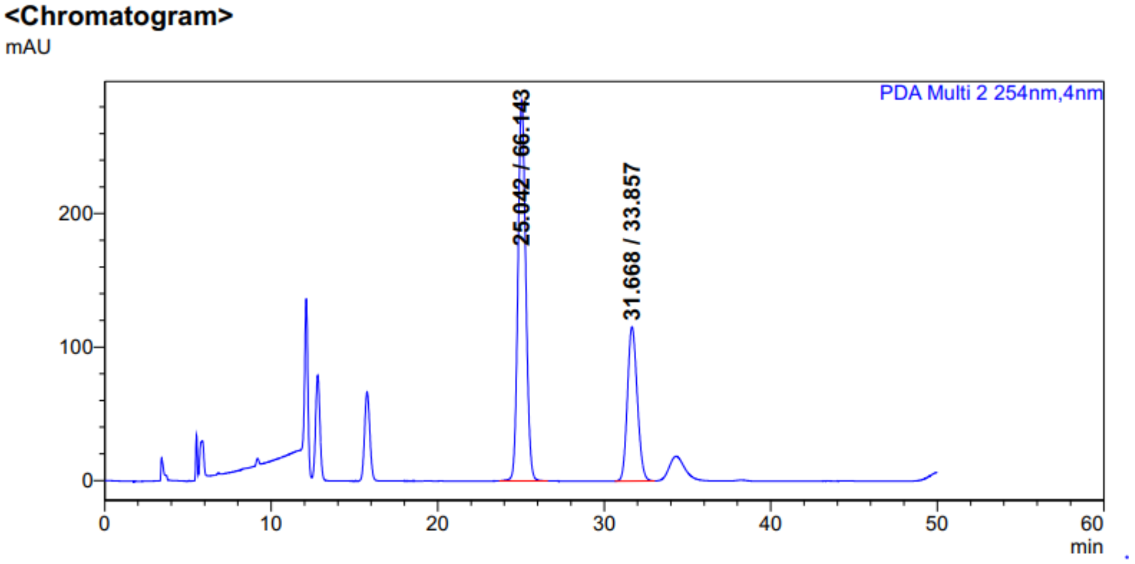

PDA Ch2 254nm

Peak#	Name	Ret. Time	Area	Area%
1		25.042	9297496	66.143
2		31.668	4759206	33.857
Total			14056702	100.000

HPLC traces for reaction with: **3-CF_3-phenylboronic acid (duplicate)**

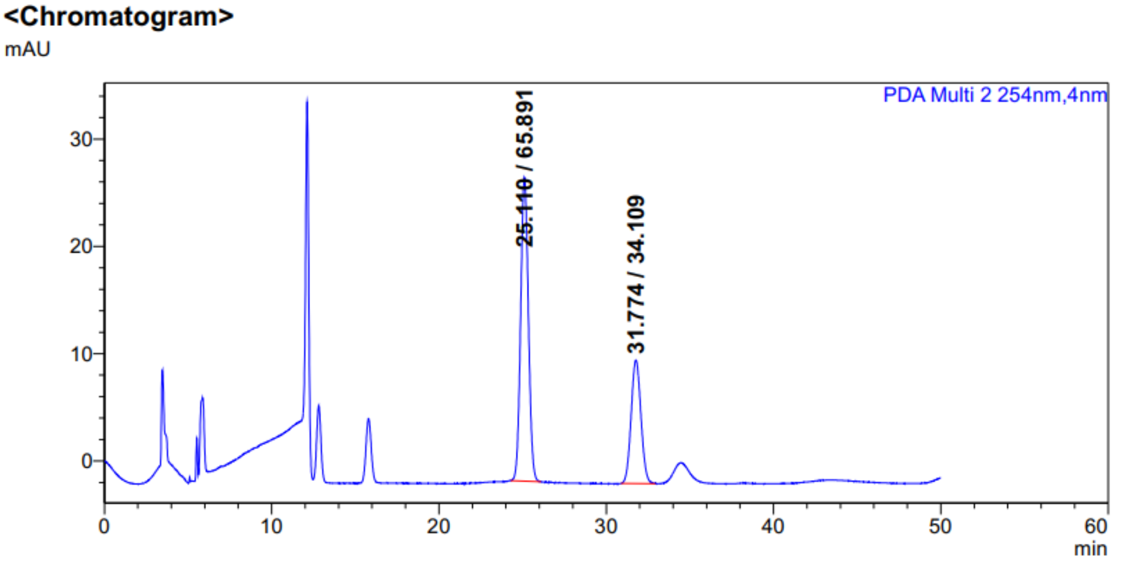

PDA Ch2 254nm

Peak#	Name	Ret. Time	Area	Area%
1		25.110	906787	65.891
2		31.774	469409	34.109
Total			1376196	100.000

HPLC traces for reaction with: **2-naphtalene-phenylboronic acid**

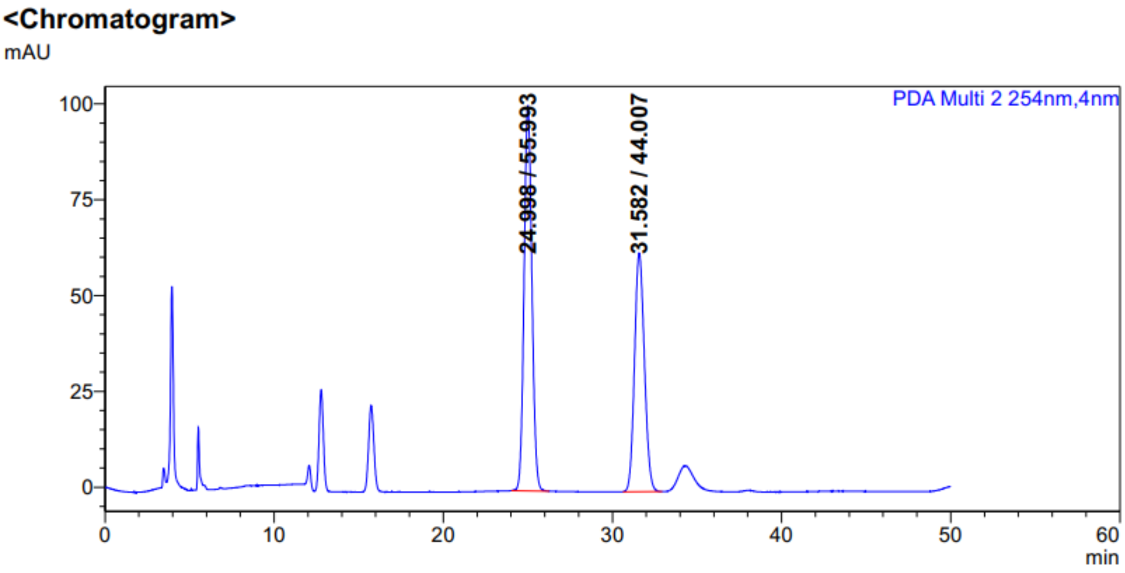

PDA Ch2 254nm

Peak#	Name	Ret. Time	Area	Area%
1		24.998	3223859	55.993
2		31.582	2533798	44.007
Total			5757657	100.000

HPLC traces for reaction with: **2-naphtalene-phenylboronic acid (duplicate)**

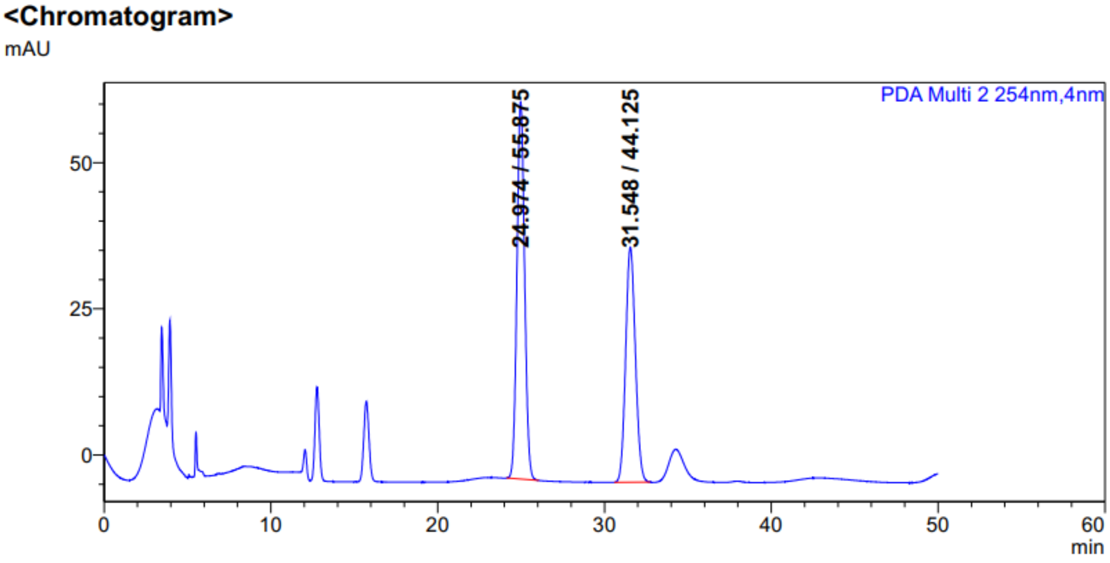

PDA Ch2 254nm

Peak#	Name	Ret. Time	Area	Area%
1		24.974	2060585	55.875
2		31.548	1627273	44.125
Total			3687858	100.000

HPLC traces for reaction with: **4-CF_3-phenylboronic acid**

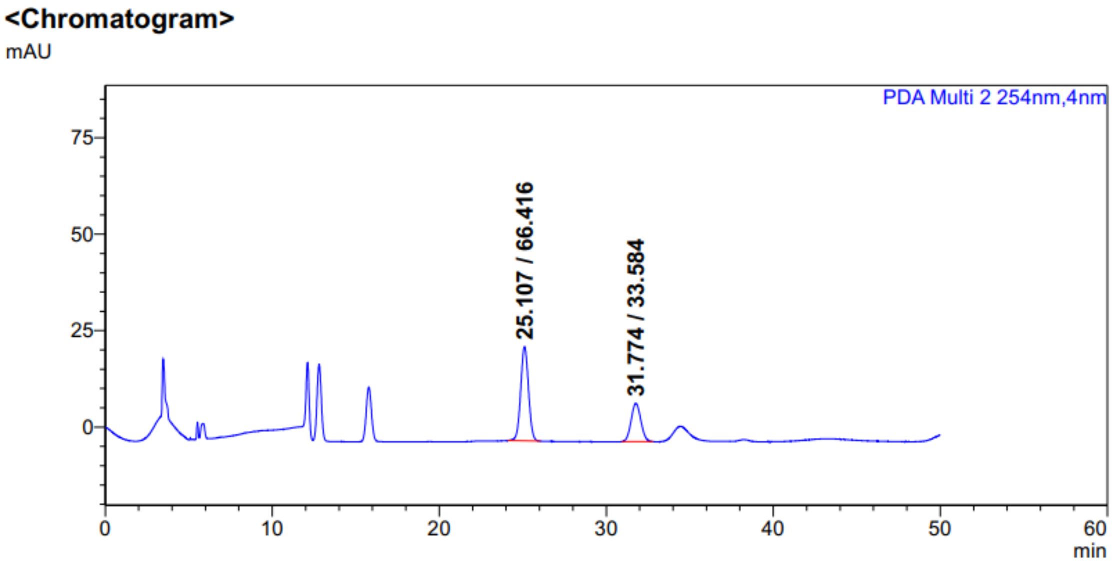

PDA Ch2 254nm

Peak#	Name	Ret. Time	Area	Area%
1		25.107	795551	66.416
2		31.774	402287	33.584
Total			1197838	100.000

HPLC traces for reaction with:**4CF_3-phenylboronic acid (duplicate)**

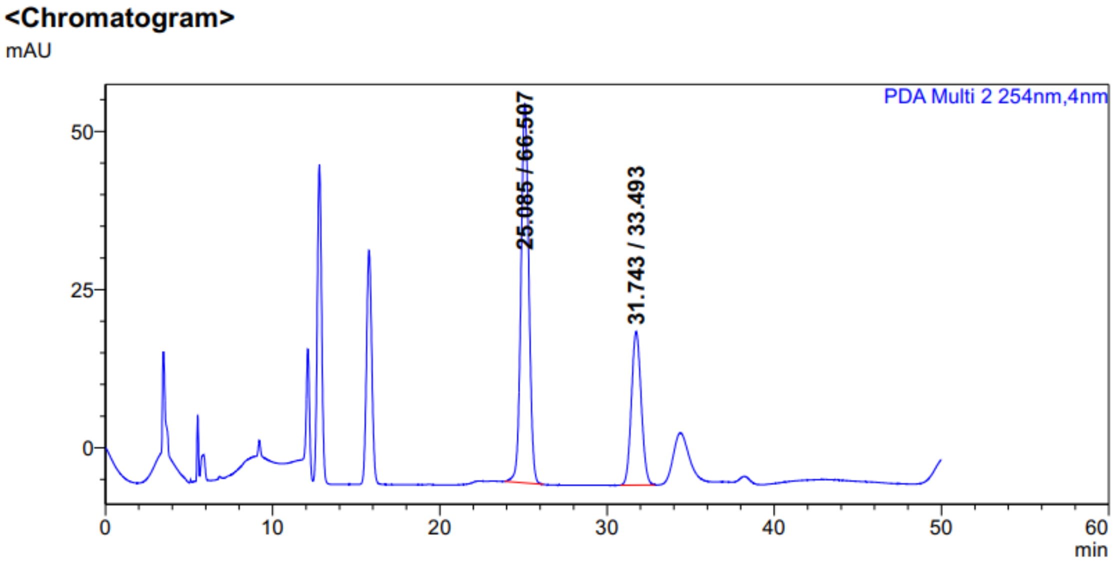

PDA Ch2 254nm

Peak#	Name	Ret. Time	Area	Area%
1		25.085	1961439	66.507
2		31.743	987764	33.493
Total			2949202	100.000

HPLC traces for reaction with: **4-F-phenylboronic acid**

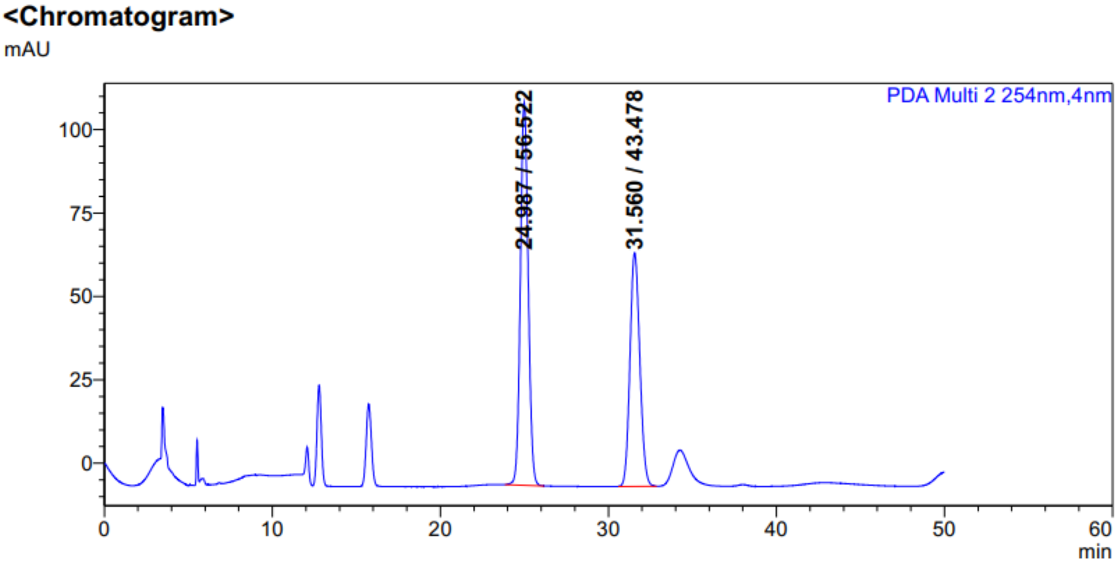

PDA Ch2 254nm

Peak#	Name	Ret. Time	Area	Area%
1		24.987	3705290	56.522
2		31.560	2850176	43.478
Total			6555466	100.000

HPLC traces for reaction with: **4-F-phenylboronic acid (duplicate)**

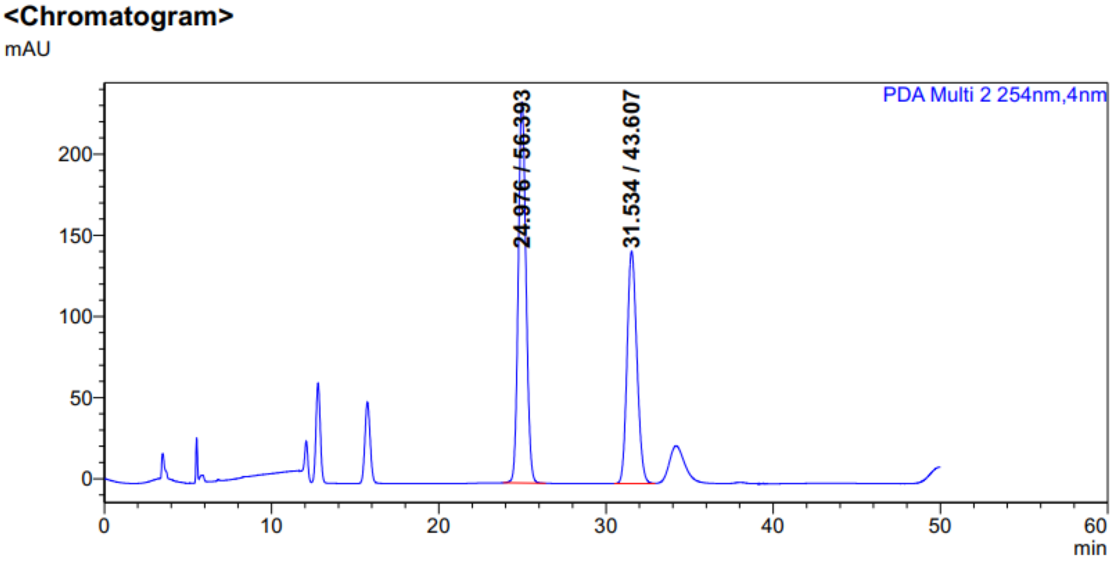

PDA Ch2 254nm

Peak#	Name	Ret. Time	Area	Area%
1		24.976	7584040	56.393
2		31.534	5864606	43.607
Total			13448646	100.000

HPLC traces for reaction with: **4-OMe-phenylboronic acid**

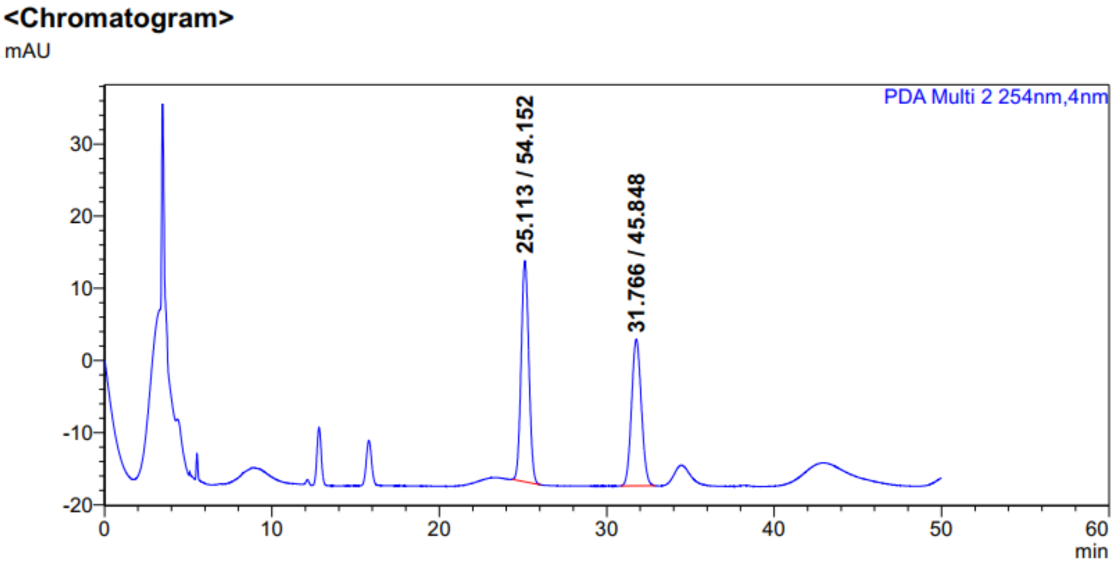

PDA Ch2 254nm

Peak#	Name	Ret. Time	Area	Area%
1		25.113	977027	54.152
2		31.766	827202	45.848
Total			1804228	100.000

HPLC traces for reaction with: **4-OMe-phenylboronic acid (duplicate)**

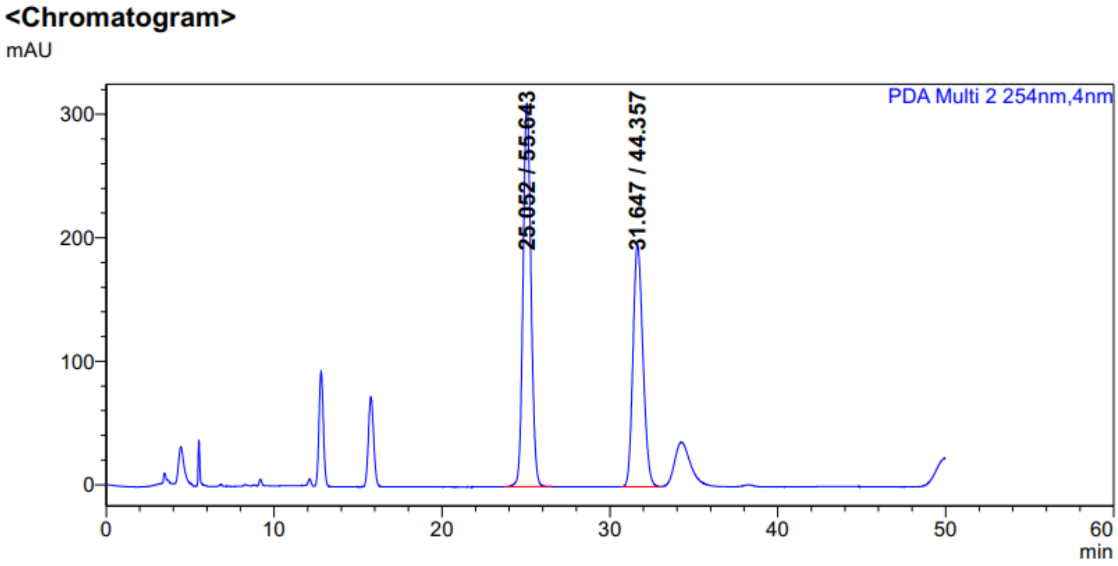

PDA Ch2 254nm

Peak#	Name	Ret. Time	Area	Area%
1		25.052	10168703	55.643
2		31.647	8106319	44.357
Total			18275022	100.000

HPLC traces for reaction with: **3-Me-phenylboronic acid**

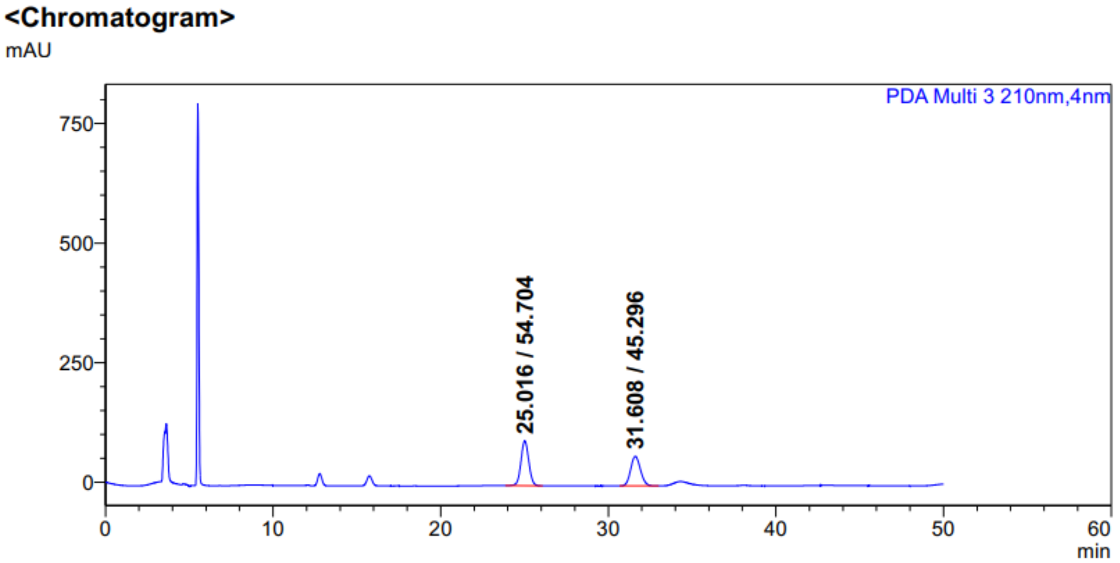

PDA Ch2 254nm

Peak#	Name	Ret. Time	Area	Area%
1		25.015	3017801	54.640
2		31.609	2505221	45.360
Total			5523021	100.000

HPLC traces for reaction with: **3-Me-phenylboronic acid (duplicate)**

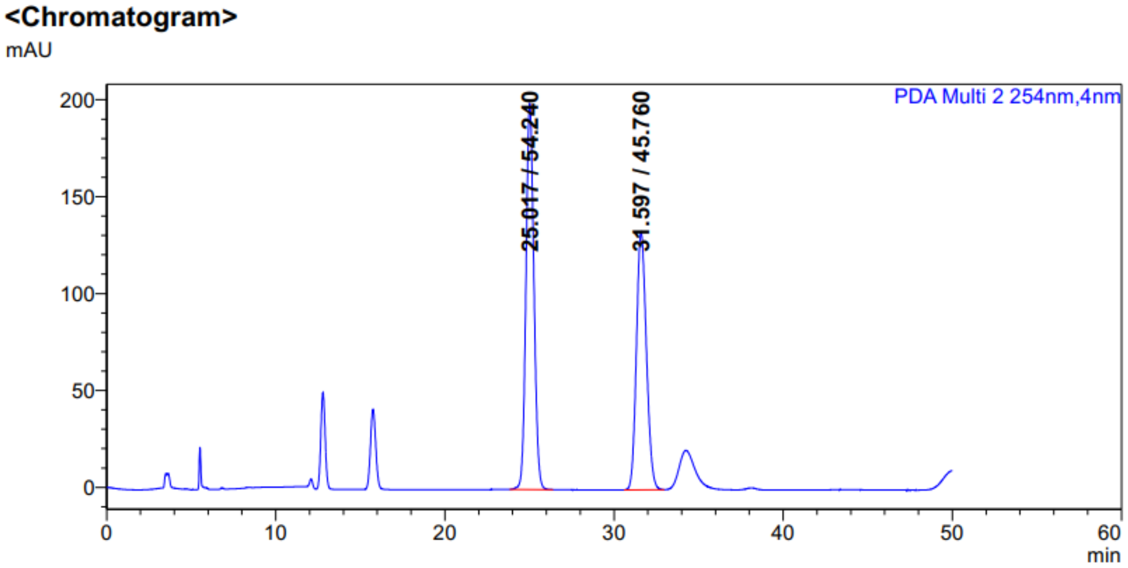

PDA Ch2 254nm

Peak#	Name	Ret. Time	Area	Area%
1		25.017	6465050	54.240
2		31.597	5454187	45.760
Total			11919236	100.000

HPLC traces for reaction with: **2-Me-phenylboronic acid**

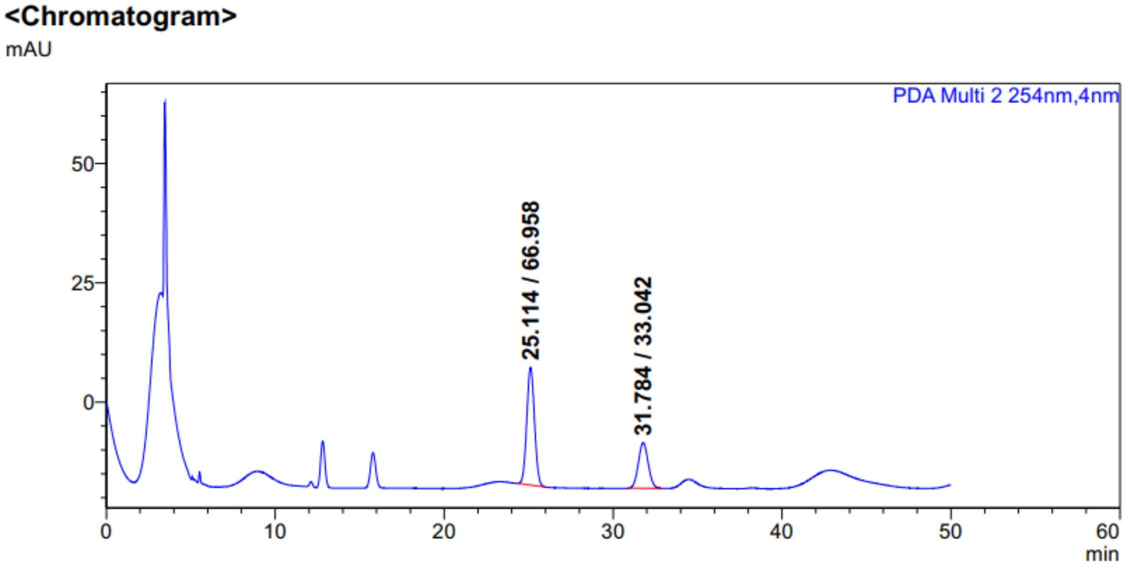

PDA Ch2 254nm

Peak#	Name	Ret. Time	Area	Area%
1		25.114	785654	66.958
2		31.784	387700	33.042
Total			1173354	100.000

HPLC traces for reaction with: **2-Me-phenylboronic acid (duplicate)**

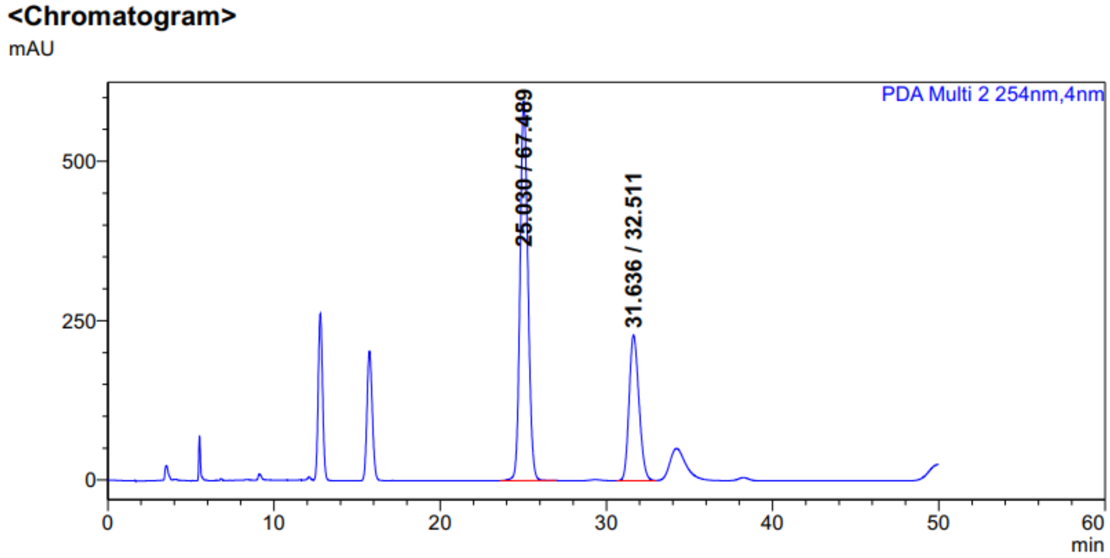

PDA Ch2 254nm

Peak#	Name	Ret. Time	Area	Area%
1		25.030	19644600	67.489
2		31.636	9463238	32.511
Total			29107838	100.000

HPLC traces for reaction with: **Ph-phenylboronic acid**

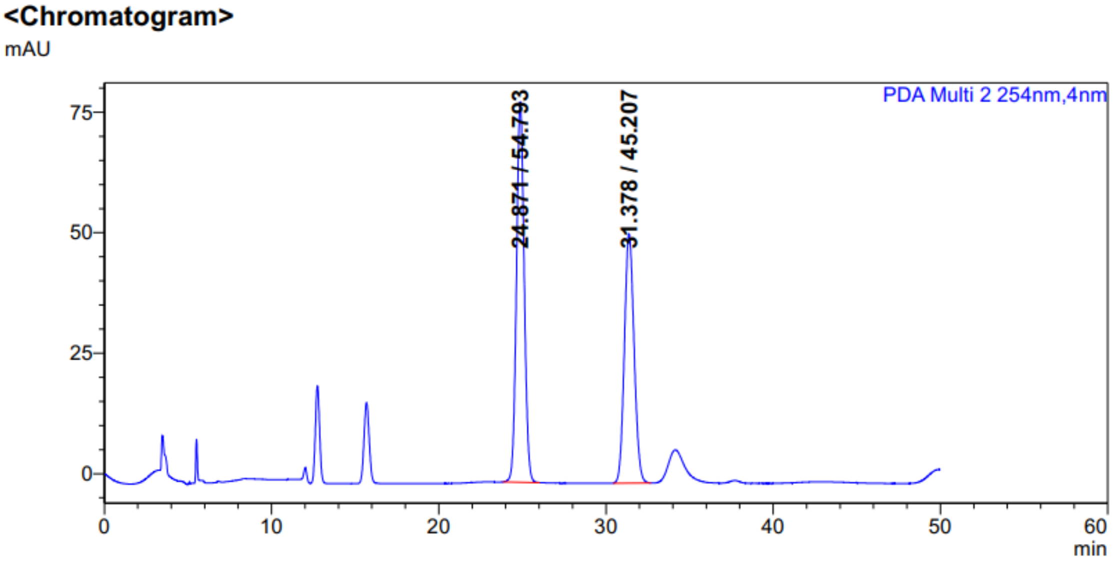

PDA Ch2 254nm

Peak#	Name	Ret. Time	Area	Area%
1		24.871	2526016	54.793
2		31.378	2084051	45.207
Total			4610067	100.000

HPLC traces for reaction with: **Ph-phenylboronic acid (duplicate)**

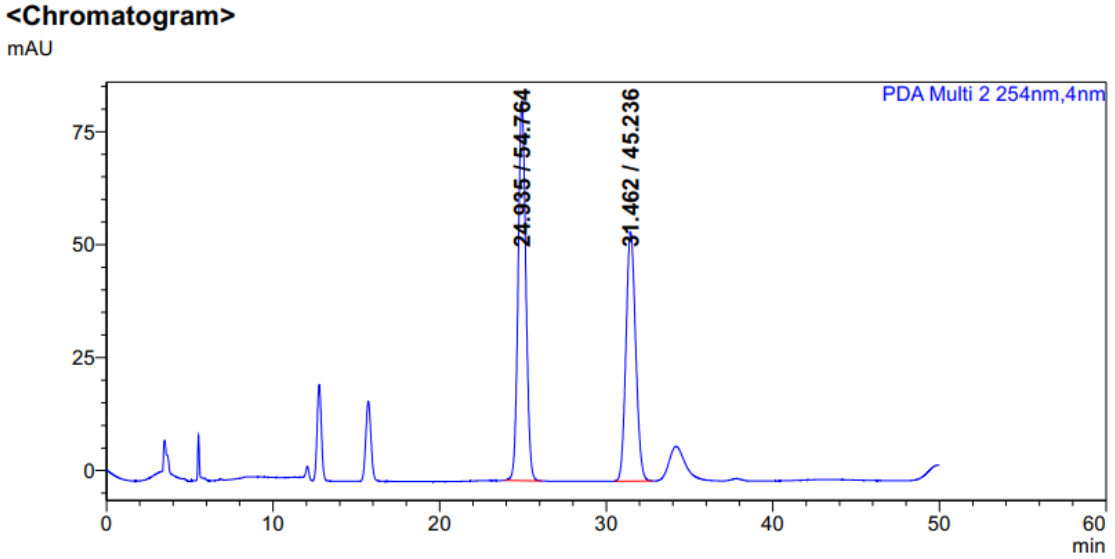

PDA Ch2 254nm

Peak#	Name	Ret. Time	Area	Area%
1		24.935	2694601	54.764
2		31.462	2225749	45.236
Total			4920351	100.000

HPLC traces for reaction with: **2-F-phenylboronic acid**

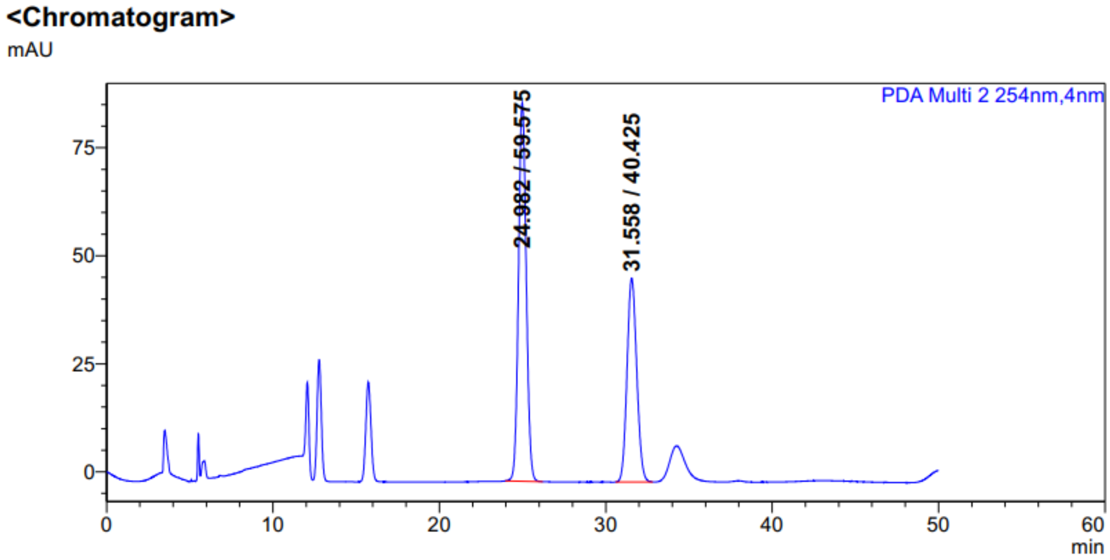

PDA Ch2 254nm

Peak#	Name	Ret. Time	Area	Area%
1		24.982	2819298	59.575
2		31.558	1913041	40.425
Total			4732339	100.000

HPLC traces for reaction with: **2-F-phenylboronic acid (duplicate)**

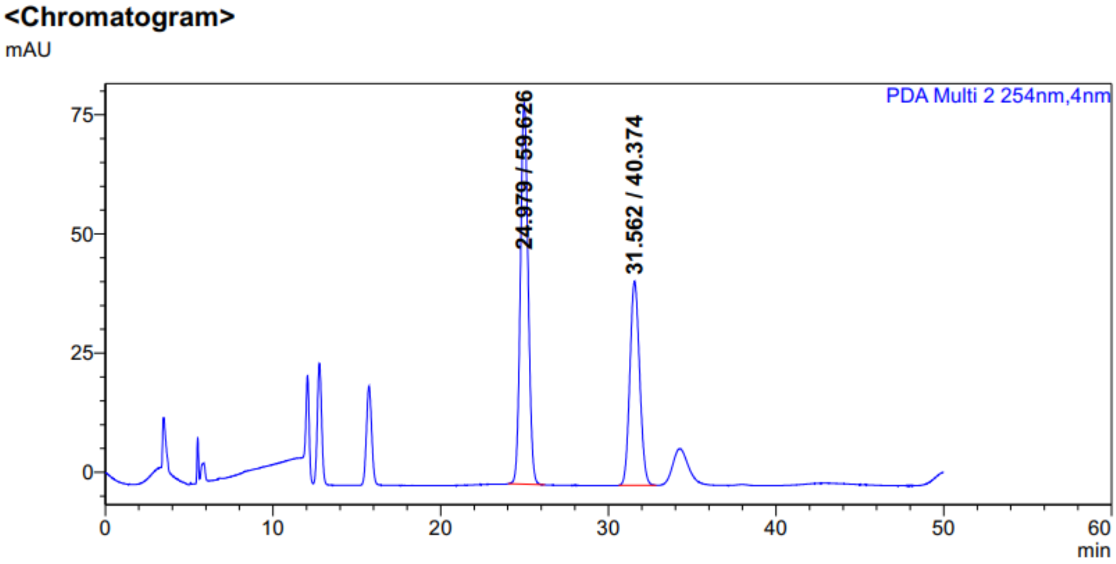

PDA Ch2 254nm

Peak#	Name	Ret. Time	Area	Area%
1		24.979	2566961	59.626
2		31.562	1738167	40.374
Total			4305128	100.000

HPLC traces for reaction **without boronic acid**:

<Chromatogram>

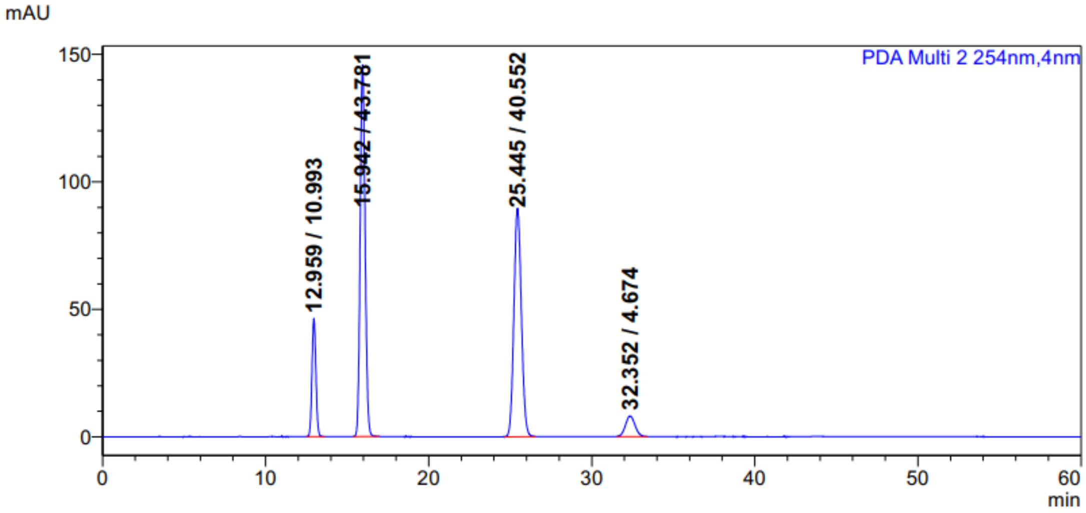

PDA Ch2 254nm

Peak#	Name	Ret. Time	Area	Area%
1		12.959	771437	10.993
2		15.942	3072412	43.781
3		25.445	2845825	40.552
4		32.352	328025	4.674
Total			7017699	100.000

HPLC traces for reaction **without boronic acid (duplicate)**:

<Chromatogram>

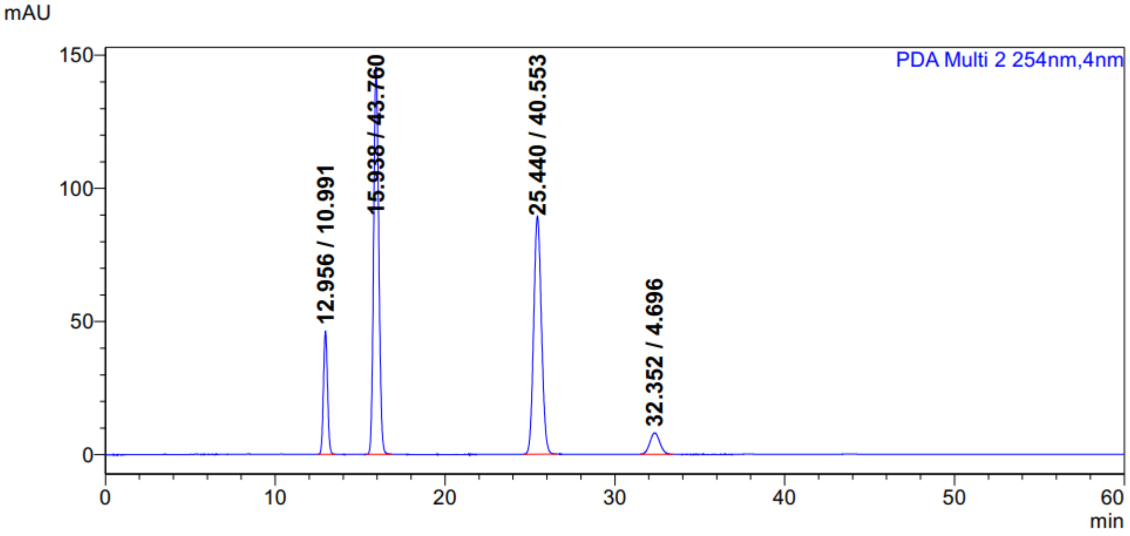

PDA Ch2 254nm

Peak#	Name	Ret. Time	Area	Area%
1		12.956	769218	10.991
2		15.938	3062766	43.760
3		25.440	2838277	40.553
4		32.352	328668	4.696
Total			6998930	100.000

HPLC traces for reaction **without water and with 2-F-phenylboronic acid**:

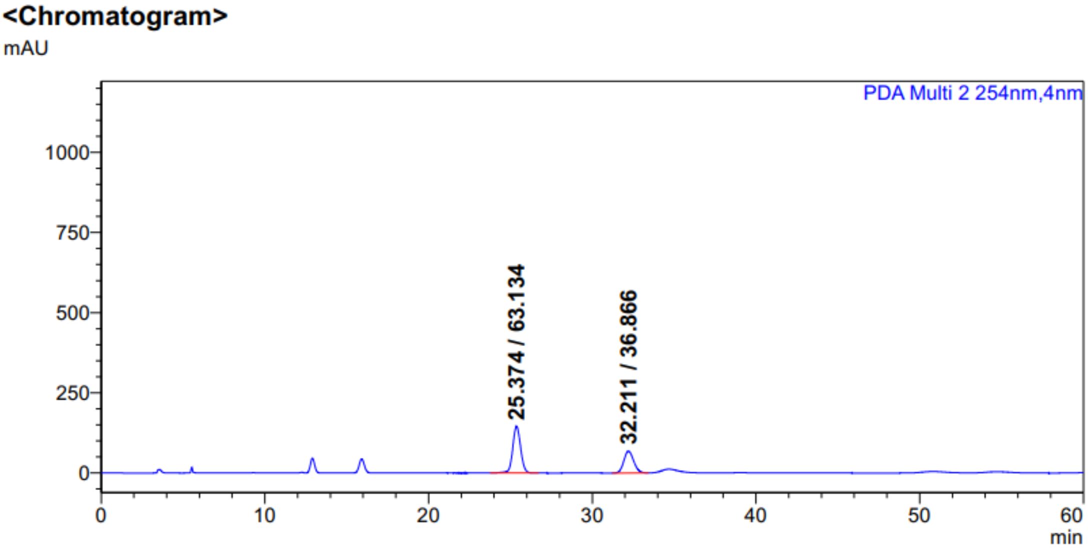

PDA Ch2 254nm

Peak#	Name	Ret. Time	Area	Area%
1		25.374	4900890	63.134
2		32.211	2861831	36.866
Total			7762721	100.000

HPLC traces for reaction **without water and with 2-F-phenylboronic acid (duplicate)**:

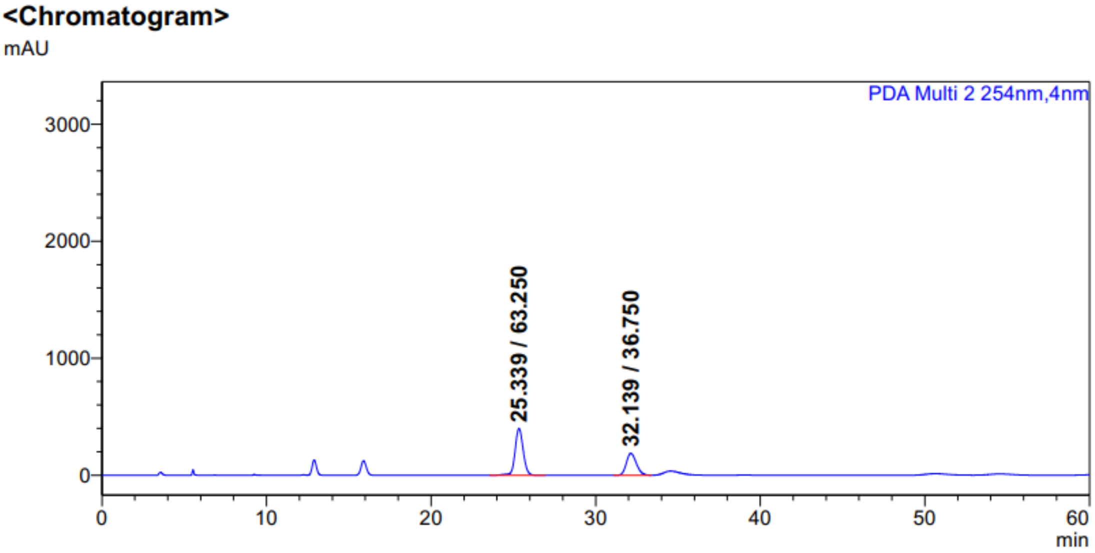

PDA Ch2 254nm

Peak#	Name	Ret. Time	Area	Area%
1		25.339	13680796	63.250
2		32.139	7949041	36.750
Total			21629838	100.000

12. HPLC Traces for Table S5. Boronic acid screening and blanks in hexane

All of the reactions were performed as duplicate following GP2 with different boronic acids. A racemic sample was first analyzed in order to determine the retention time of both enantiomers. For the chiral sample, enantiomeric excess was determined by comparing the integrated area of these two peaks. All the HPLC traces shown below are for of (S)-2-((R)-hydroxy(4-nitrophenyl)methyl)cyclopentan-1-one, yielded by different reactions as described in table S5.

HPLC traces for reaction with: **3-F-phenylboronic acid**

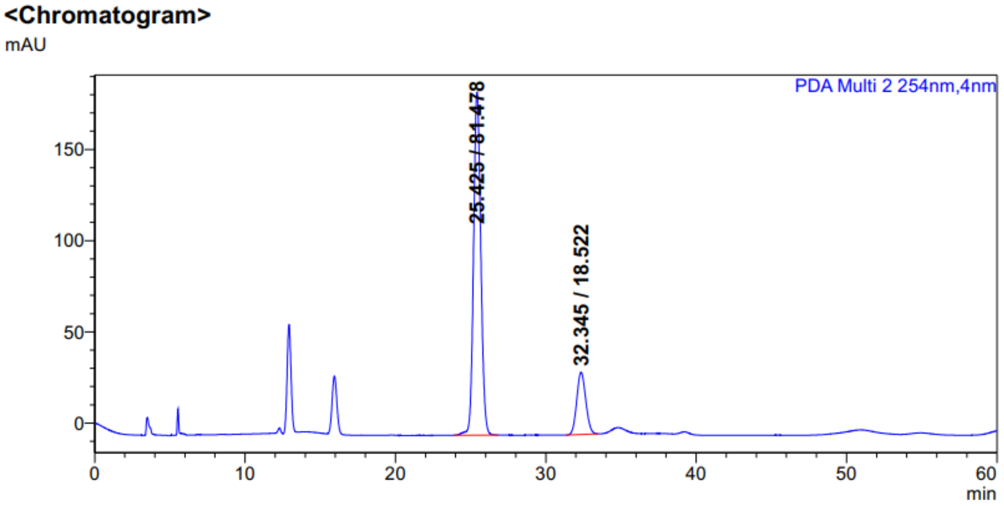

PDA Ch2 254nm

Peak#	Name	Ret. Time	Area	Area%
1		25.425	6274897	81.478
2		32.345	1426406	18.522
Total			7701304	100.000

HPLC traces for reaction with: **3-F-phenylboronic acid (duplicate)**

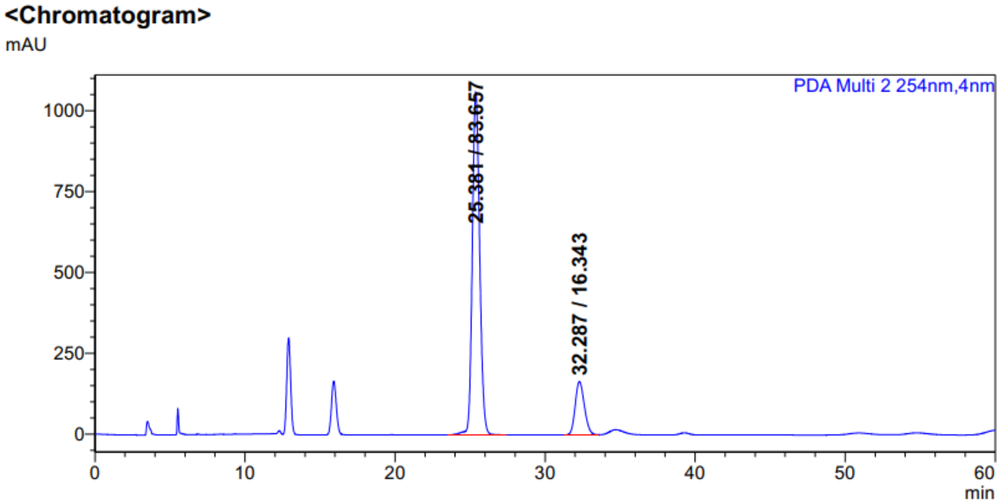

PDA Ch2 254nm

Peak#	Name	Ret. Time	Area	Area%
1		25.381	35989202	83.657
2		32.287	7030730	16.343
Total			43019932	100.000

HPLC traces for reaction with: **3,5-F -phenylboronic acid**

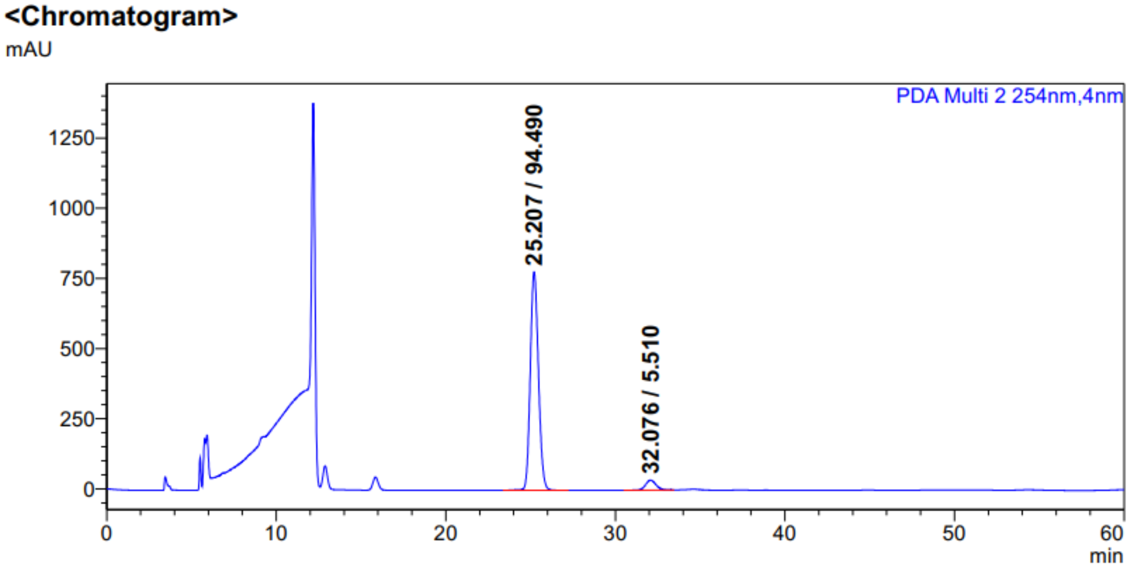

PDA Ch2 254nm

Peak#	Name	Ret. Time	Area	Area%
1		25.207	25967636	94.490
2		32.076	1514182	5.510
Total			27481818	100.000

HPLC traces for reaction with: **3,5-F-phenylboronic acid (duplicate)**

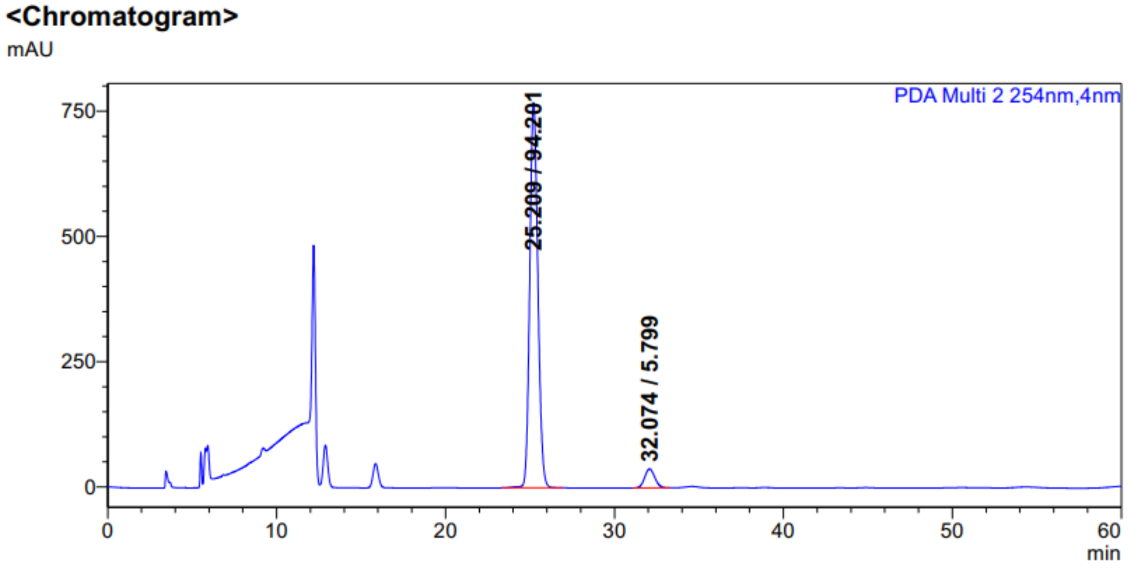

PDA Ch2 254nm

Peak#	Name	Ret. Time	Area	Area%
1		25.209	25650891	94.201
2		32.074	1579032	5.799
Total			27229923	100.000

HPLC traces for reaction with: **4-tBu-phenylboronic acid**

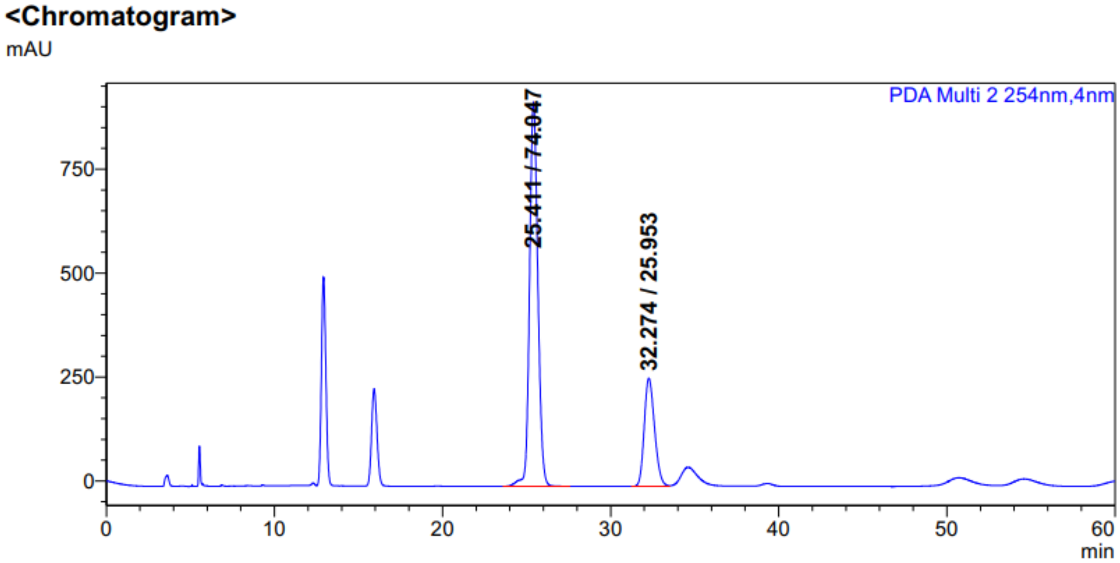

PDA Ch2 254nm

Peak#	Name	Ret. Time	Area	Area%
1		25.411	31690416	74.047
2		32.274	11107171	25.953
Total			42797586	100.000

HPLC traces for reaction with: **4-tBu-phenylboronic acid (duplicate)**

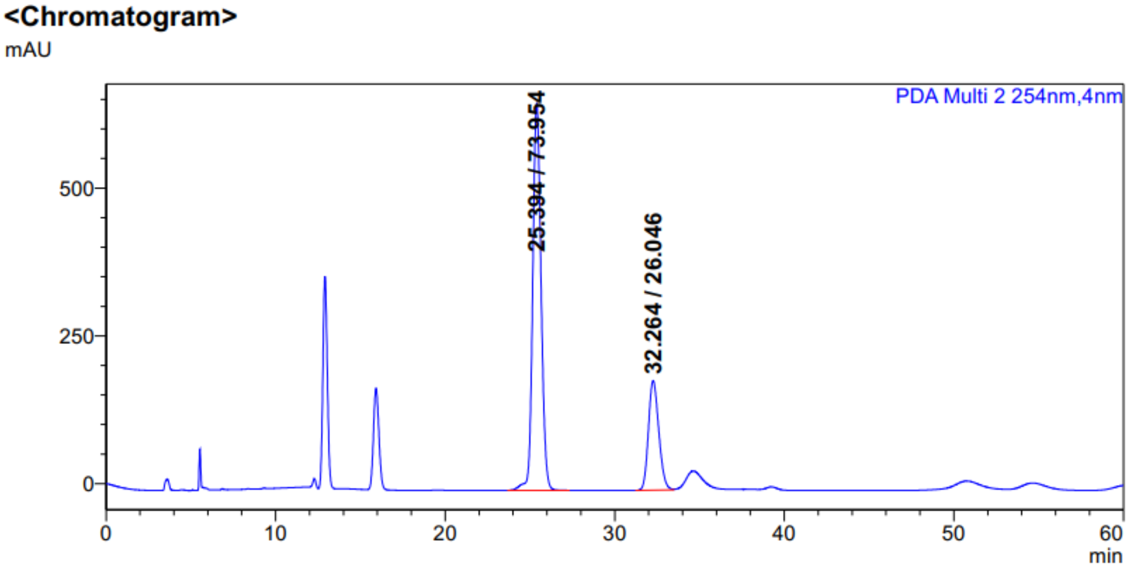

PDA Ch2 254nm

Peak#	Name	Ret. Time	Area	Area%
1		25.394	22362300	73.954
2		32.264	7875716	26.046
Total			30238016	100.000

HPLC traces for reaction with: **2,4-Me-phenylboronic acid**

<Chromatogram>

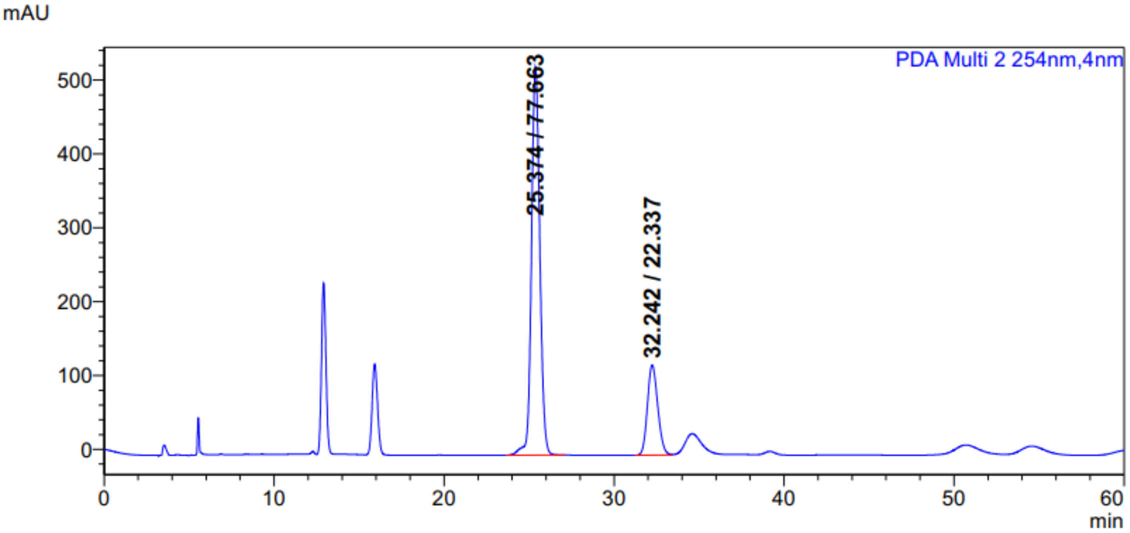

PDA Ch2 254nm

Peak#	Name	Ret. Time	Area	Area%
1		25.374	17934169	77.663
2		32.242	5158060	22.337
Total			23092229	100.000

HPLC traces for reaction with: **2,4-Me-phenylboronic acid (duplicate)**

<Chromatogram>

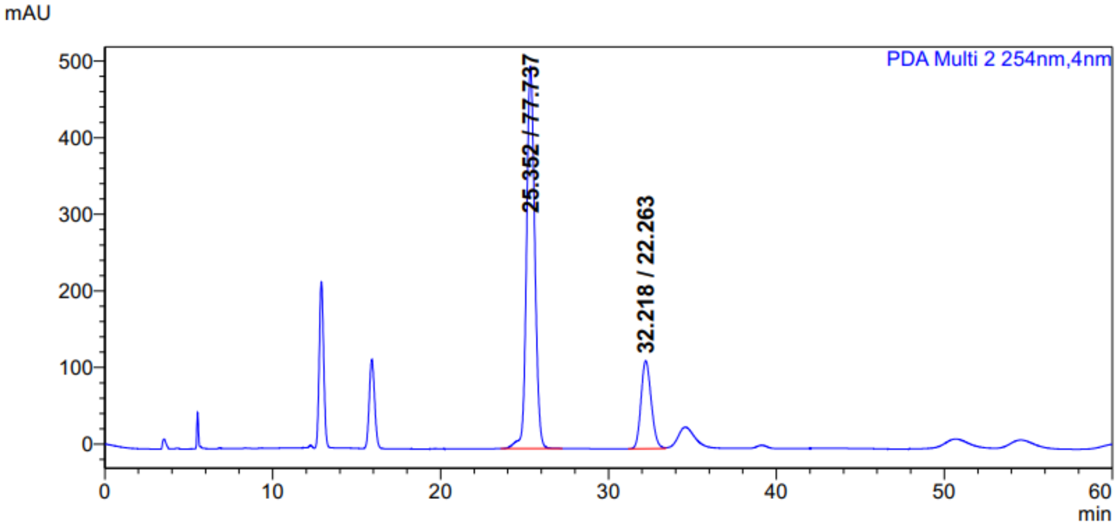

PDA Ch2 254nm

Peak#	Name	Ret. Time	Area	Area%
1		25.352	17027057	77.737
2		32.218	4876331	22.263
Total			21903388	100.000

HPLC traces for reaction with: **3,5-OMe-phenylboronic acid**

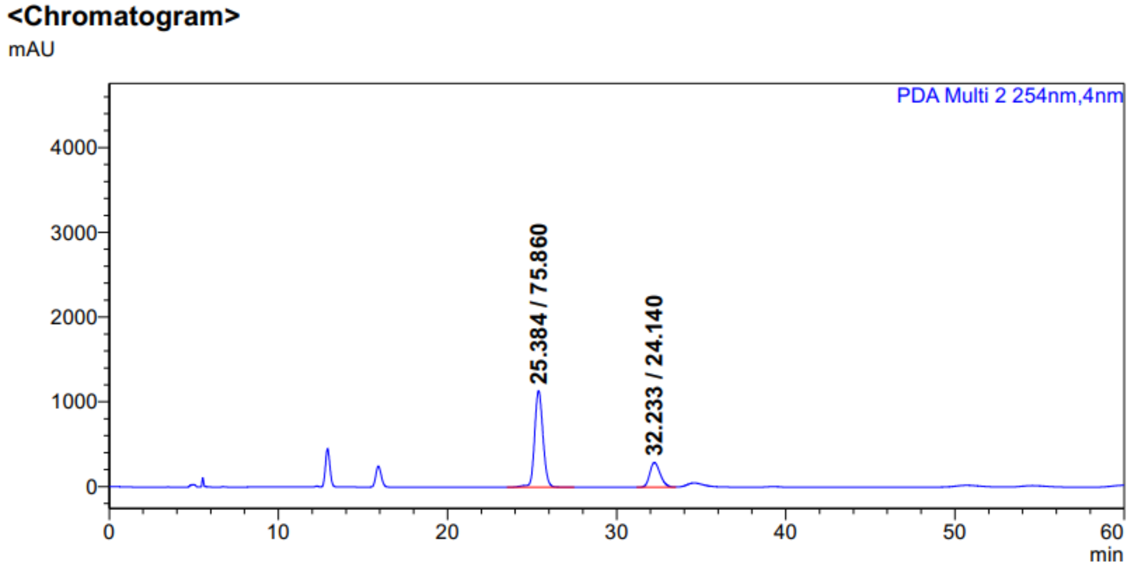

PDA Ch2 254nm

Peak#	Name	Ret. Time	Area	Area%
1		25.384	39174689	75.860
2		32.233	12465839	24.140
Total			51640529	100.000

HPLC traces for reaction with: **3,5-OMe-phenylboronic acid (duplicate)**

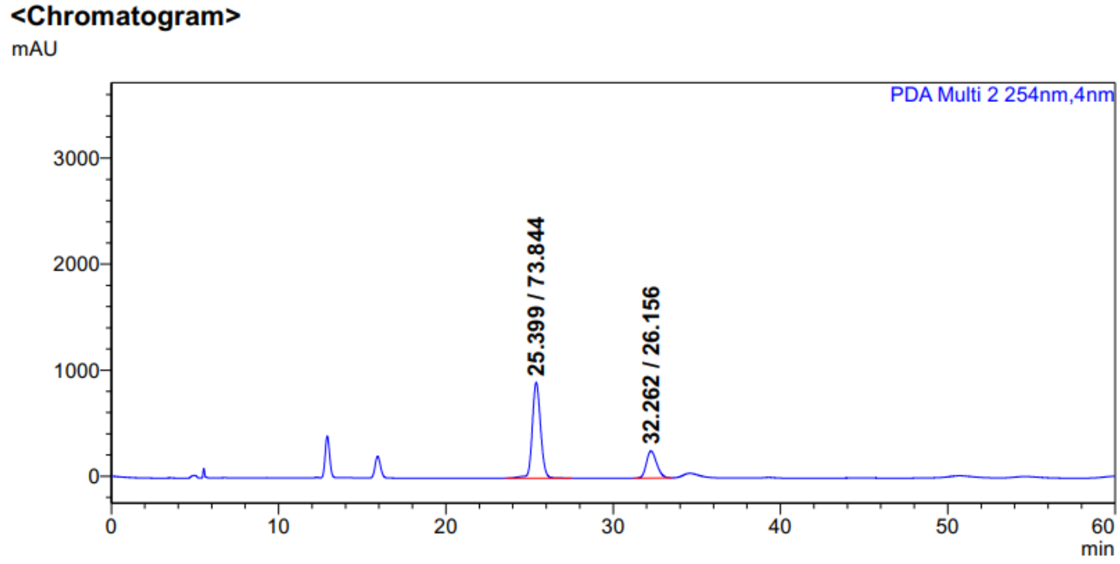

PDA Ch2 254nm

Peak#	Name	Ret. Time	Area	Area%
1		25.399	30989467	73.844
2		32.262	10976633	26.156
Total			41966100	100.000

HPLC traces for reaction with: **4-Me-phenylboronic acid**

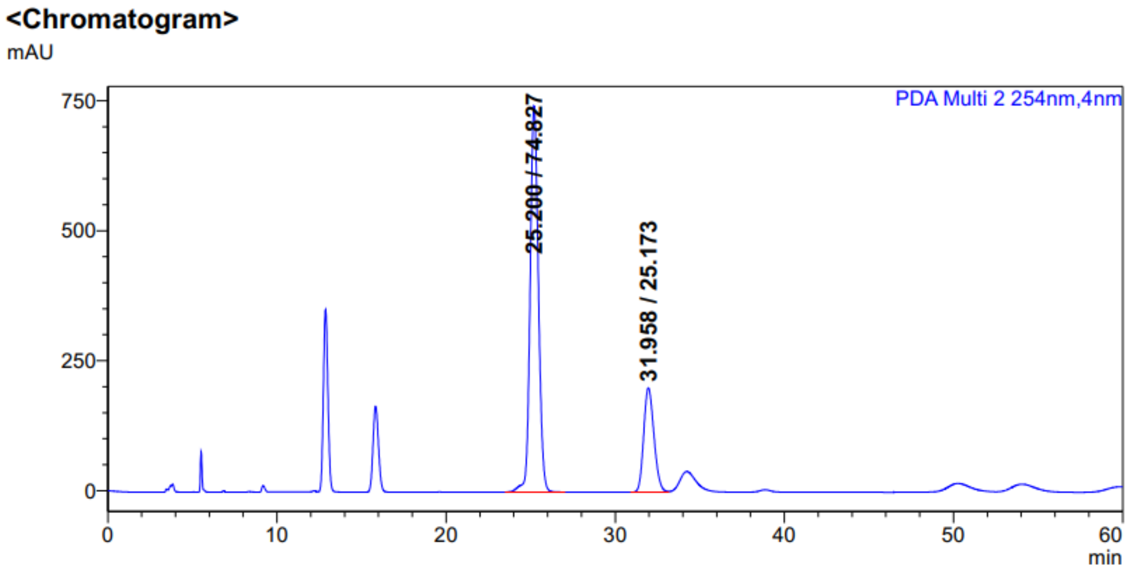

PDA Ch2 254nm

Peak#	Name	Ret. Time	Area	Area%
1		25.200	25204976	74.827
2		31.958	8479230	25.173
Total			33684206	100.000

HPLC traces for reaction with: **4-Me-phenylboronic acid (duplicate)**

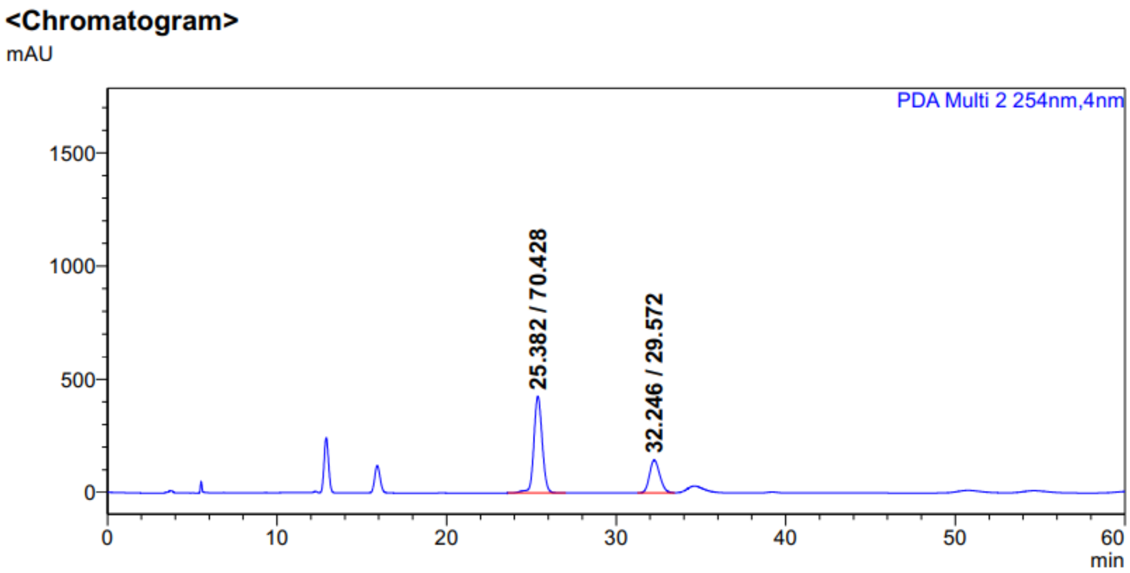

PDA Ch2 254nm

Peak#	Name	Ret. Time	Area	Area%
1		25.382	14657988	70.428
2		32.246	6154864	29.572
Total			20812852	100.000

HPLC traces for reaction with: **3-CF_3-phenylboronic acid**

<Chromatogram>

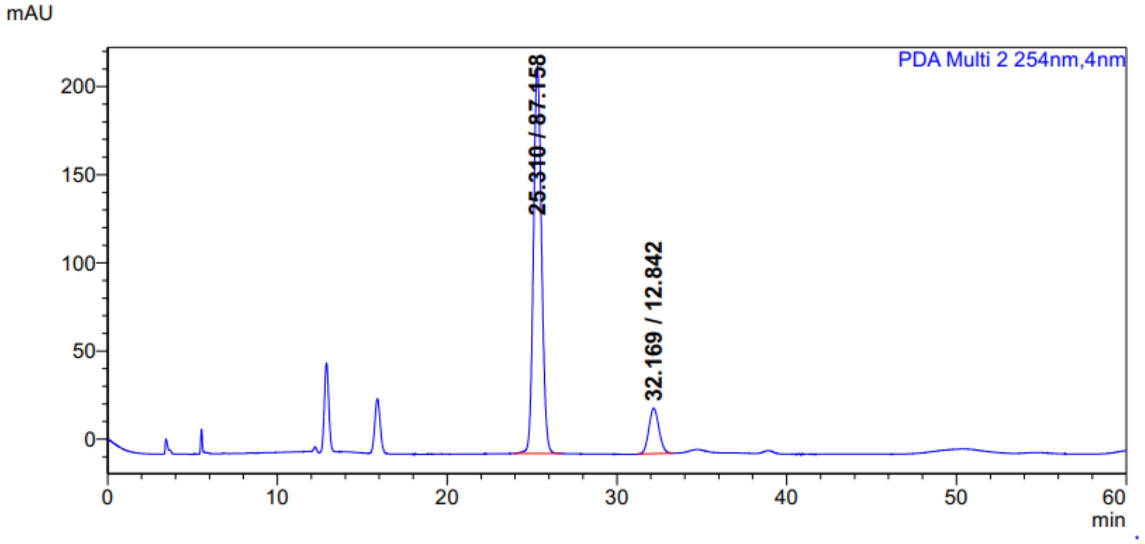

PDA Ch2 254nm

Peak#	Name	Ret. Time	Area	Area%
1		25.310	7248804	87.158
2		32.169	1068019	12.842
Total			8316823	100.000

HPLC traces for reaction with: **3-CF_3-phenylboronic acid (duplicate)**

<Chromatogram>

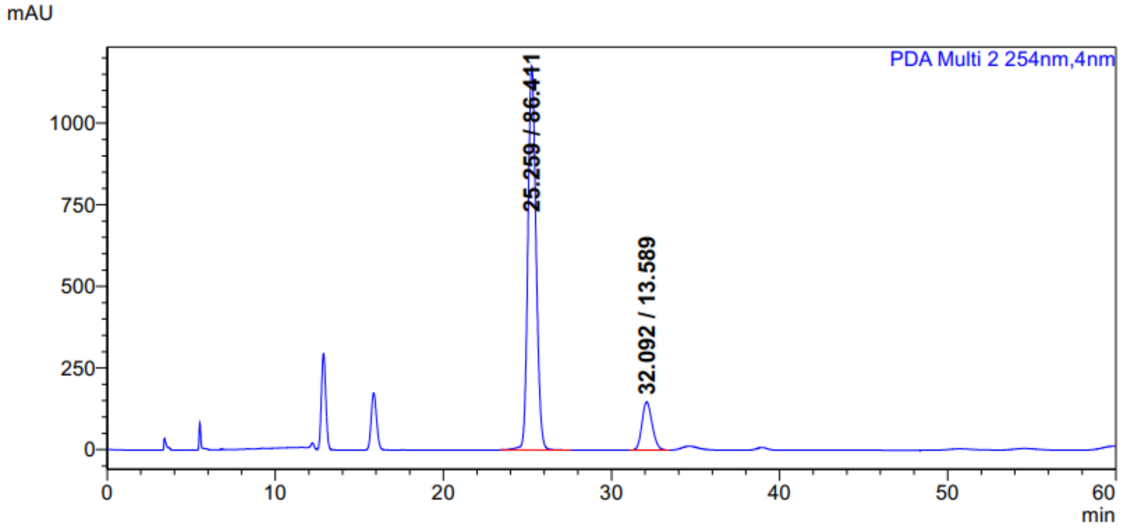

PDA Ch2 254nm

Peak#	Name	Ret. Time	Area	Area%
1		25.259	39552716	86.411
2		32.092	6220141	13.589
Total			45772856	100.000

HPLC traces for reaction with: **2-naphtalene-phenylboronic acid**

<Chromatogram>

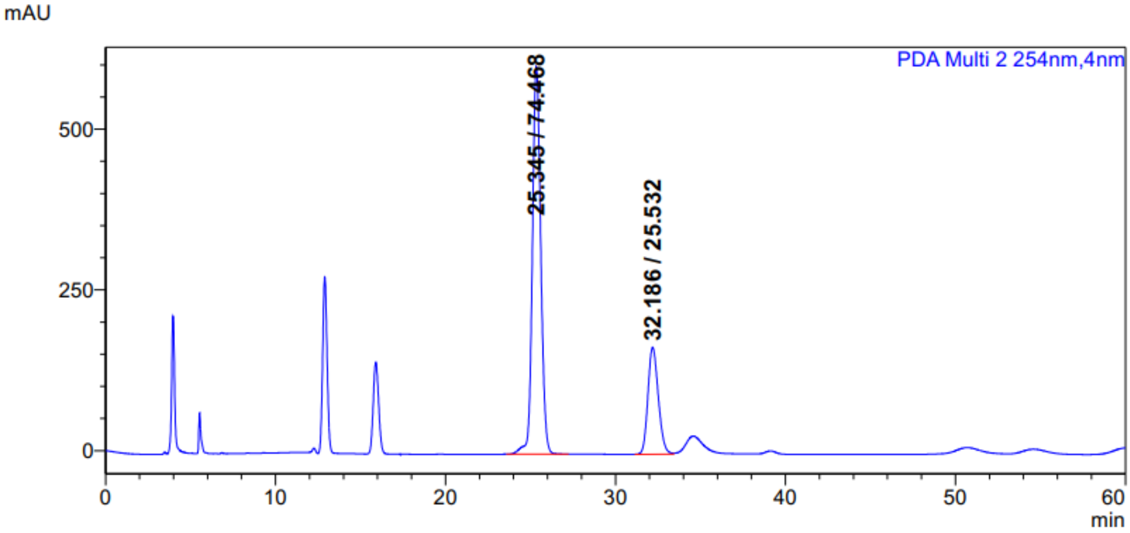

PDA Ch2 254nm

Peak#	Name	Ret. Time	Area	Area%
1		25.345	20590967	74.468
2		32.186	7059918	25.532
Total			27650885	100.000

HPLC traces for reaction with: **2-naphtalene-phenylboronic acid (duplicate)**

<Chromatogram>

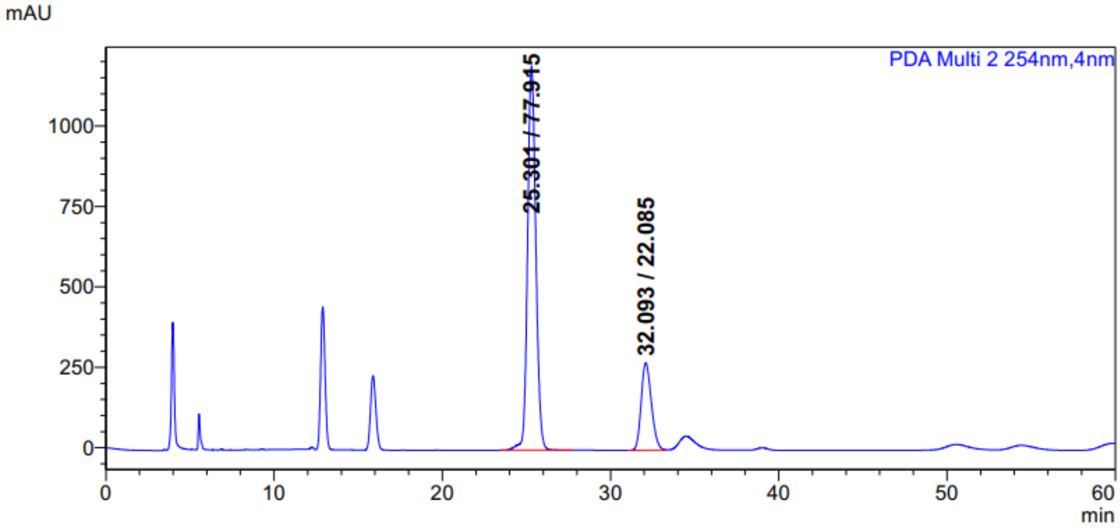

PDA Ch2 254nm

Peak#	Name	Ret. Time	Area	Area%
1		25.301	40670103	77.915
2		32.093	11527818	22.085
Total			52197921	100.000

HPLC traces for reaction with: **4-CF_3-phenylboronic acid**

<Chromatogram>

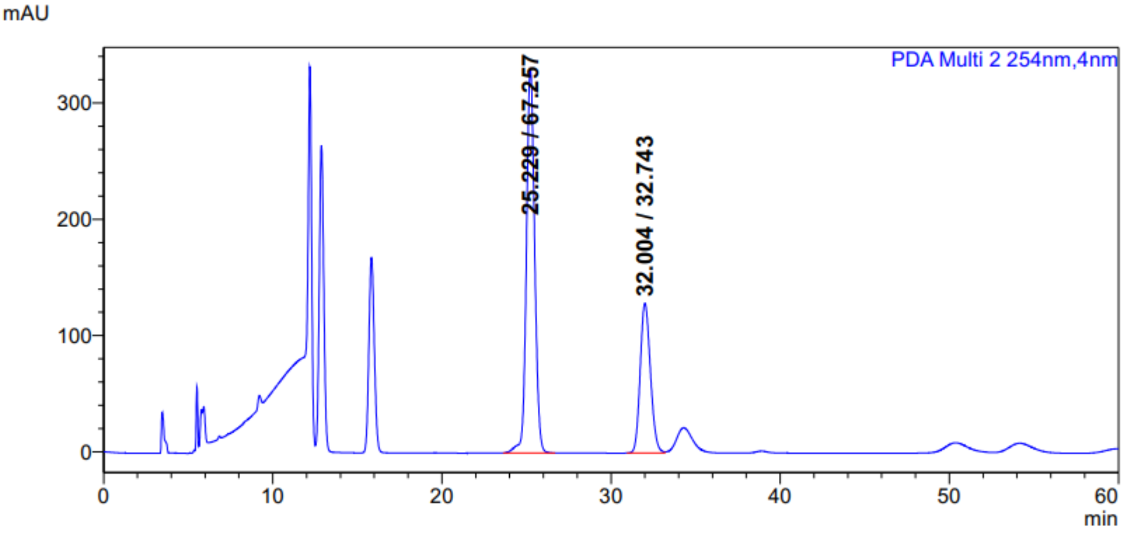

PDA Ch2 254nm

Peak#	Name	Ret. Time	Area	Area%
1		25.229	11157702	67.257
2		32.004	5432007	32.743
Total			16589709	100.000

HPLC traces for reaction with: **4-CF_3-phenylboronic acid (duplicate)**

<Chromatogram>

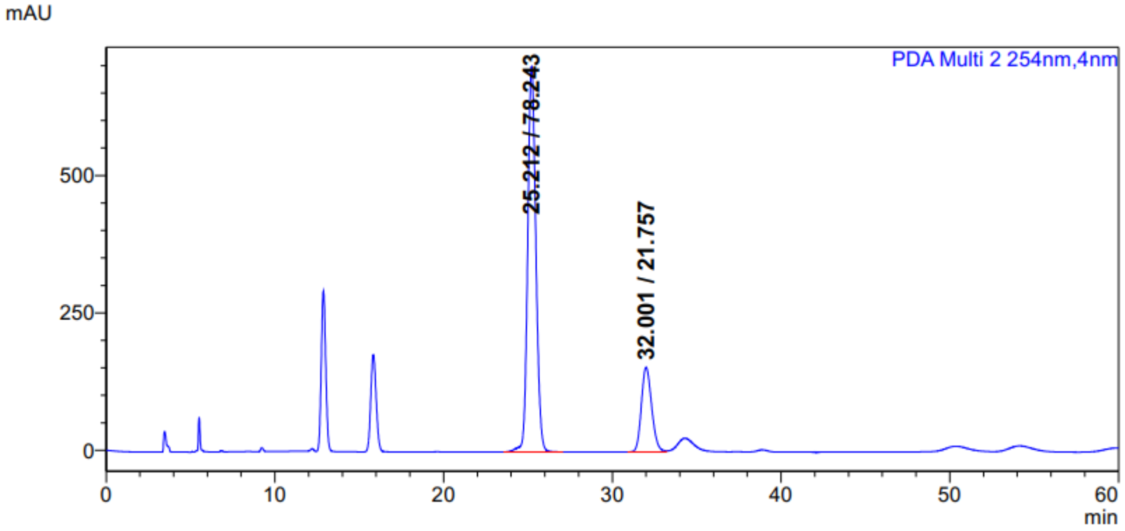

PDA Ch2 254nm

Peak#	Name	Ret. Time	Area	Area%
1		25.212	23565690	78.243
2		32.001	6552969	21.757
Total			30118660	100.000

HPLC traces for reaction with: **4-F-phenylboronic acid**

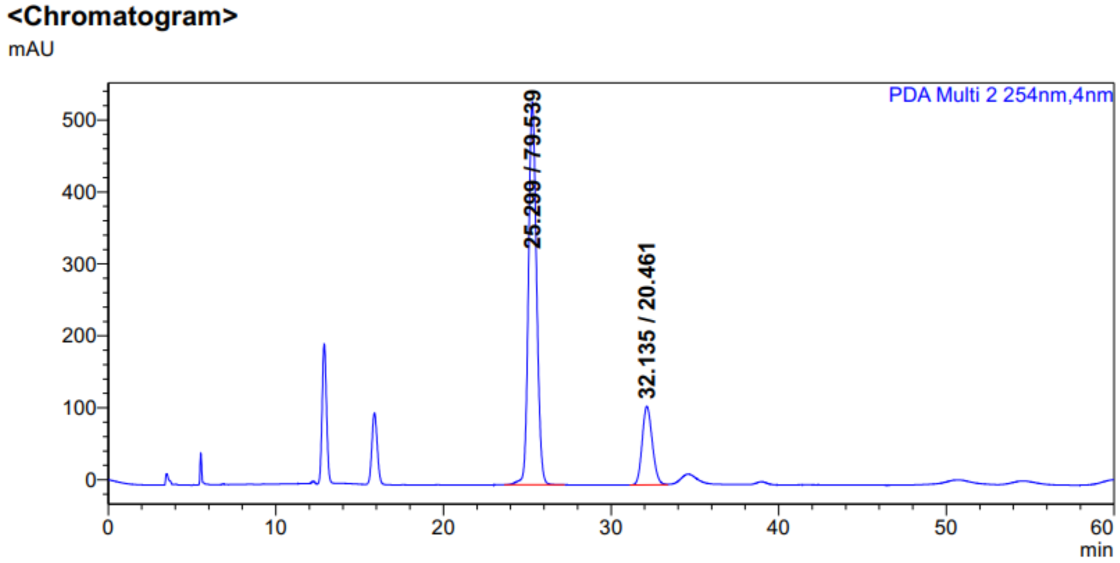

PDA Ch2 254nm

Peak#	Name	Ret. Time	Area	Area%
1		25.299	17876179	79.539
2		32.135	4598611	20.461
Total			22474790	100.000

HPLC traces for reaction with: **4-F-phenylboronic acid (duplicate)**

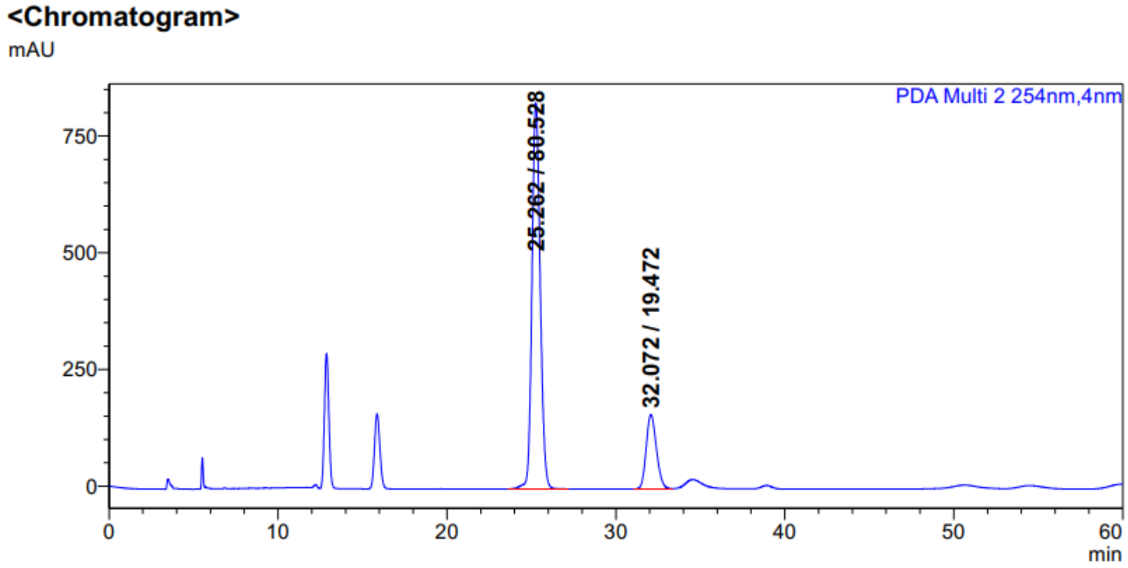

PDA Ch2 254nm

Peak#	Name	Ret. Time	Area	Area%
1		25.262	27802777	80.528
2		32.072	6722705	19.472
Total			34525482	100.000

HPLC traces for reaction with: **4-OMe-phenylboronic acid**

<Chromatogram>

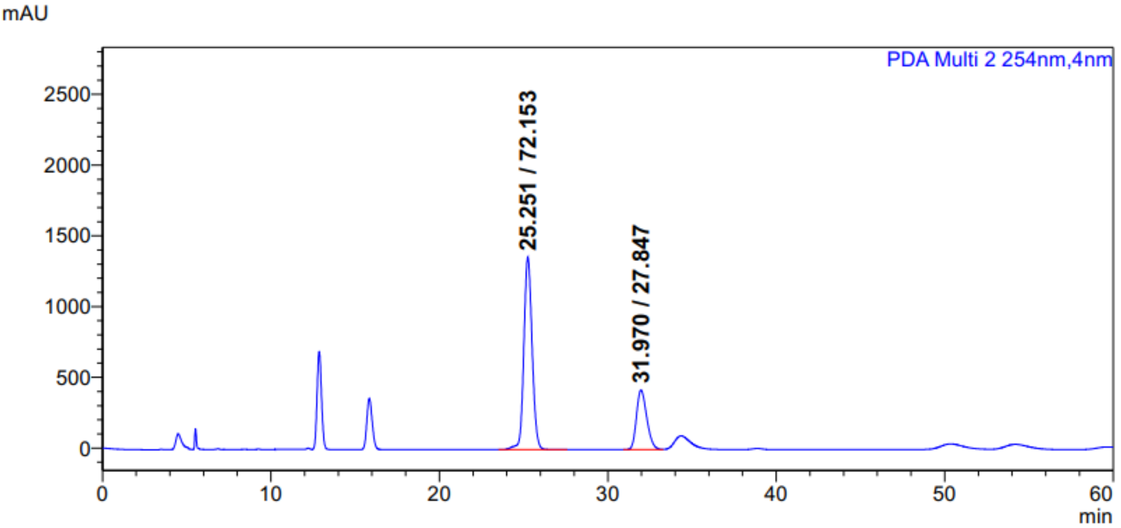

PDA Ch2 254nm

Peak#	Name	Ret. Time	Area	Area%
1		25.251	46487072	72.153
2		31.970	17941134	27.847
Total			64428206	100.000

HPLC traces for reaction with: **4-OMe-phenylboronic acid (duplicate)**

<Chromatogram>

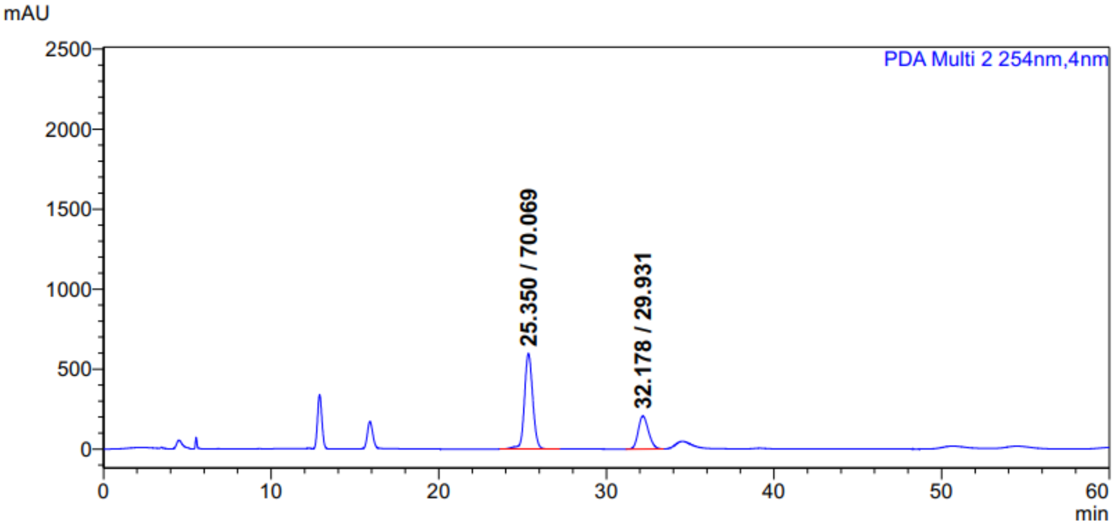

PDA Ch2 254nm

Peak#	Name	Ret. Time	Area	Area%
1		25.350	20548967	70.069
2		32.178	8777816	29.931
Total			29326783	100.000

HPLC traces for reaction with: **3-Me-phenylboronic acid**

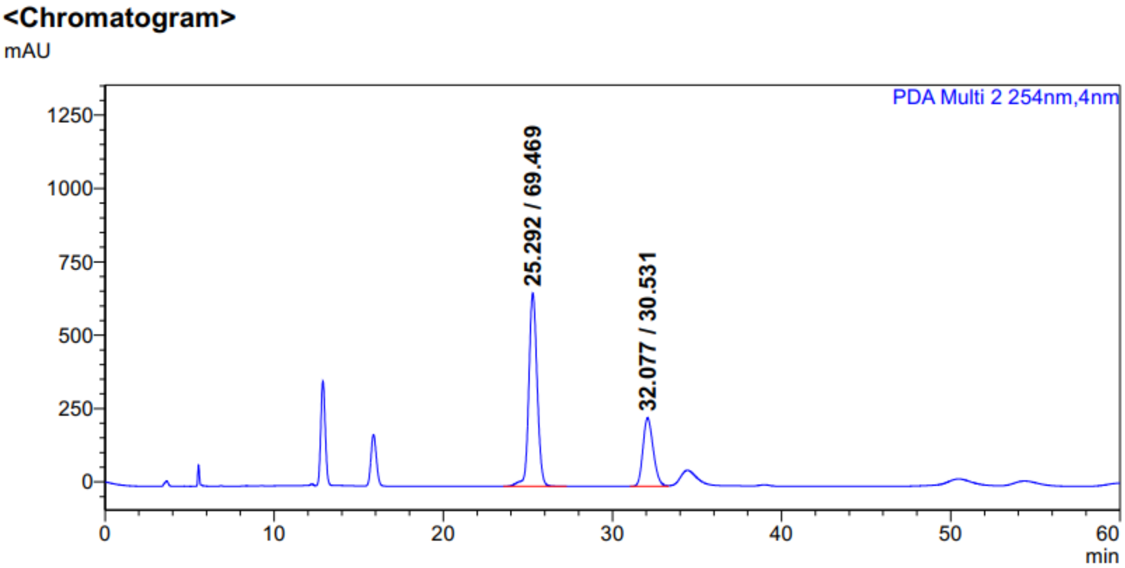

PDA Ch2 254nm

Peak#	Name	Ret. Time	Area	Area%
1		25.292	22535818	69.469
2		32.077	9904373	30.531
Total			32440191	100.000

HPLC traces for reaction with: **3-Me-phenylboronic acid (duplicate)**

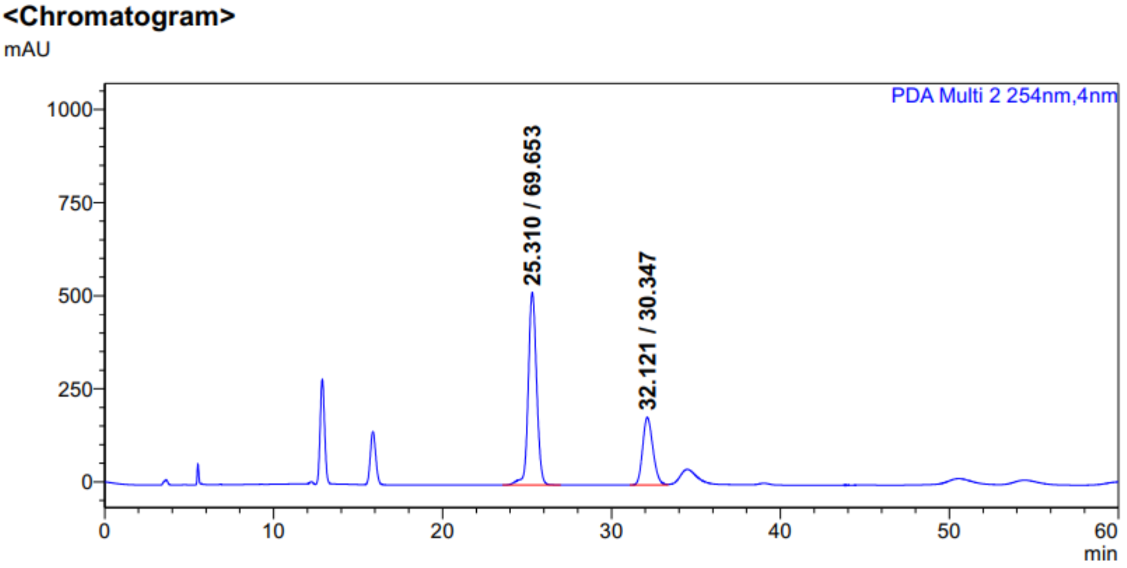

PDA Ch2 254nm

Peak#	Name	Ret. Time	Area	Area%
1		25.310	17764817	69.653
2		32.121	7739871	30.347
Total			25504688	100.000

HPLC traces for reaction with: **2-Me-phenylboronic acid**

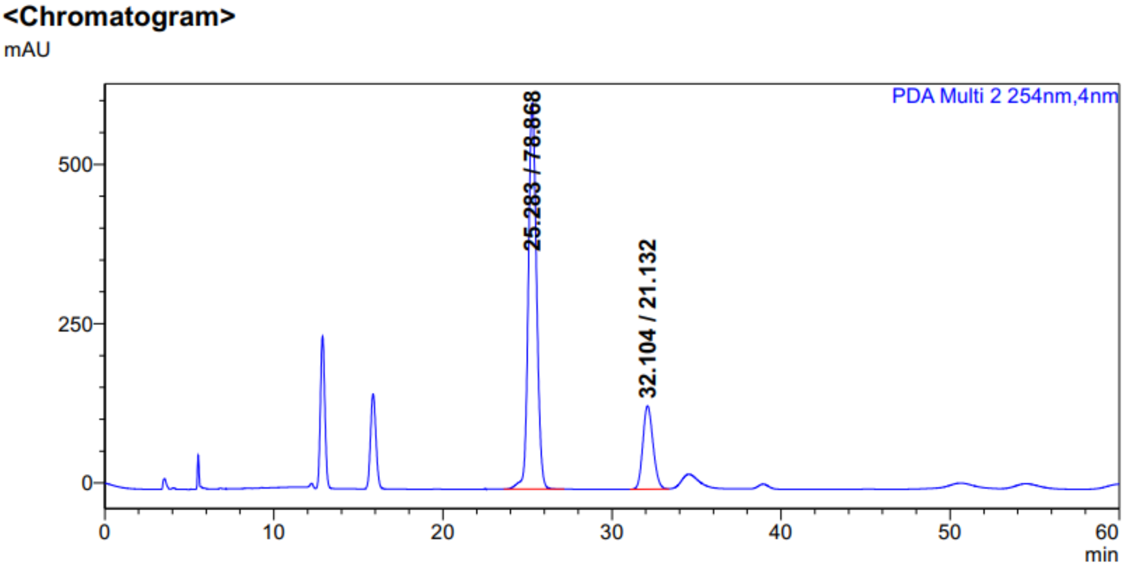

PDA Ch2 254nm

Peak#	Name	Ret. Time	Area	Area%
1		25.283	20527094	78.868
2		32.104	5500095	21.132
Total			26027190	100.000

HPLC traces for reaction with: **2-Me-phenylboronic acid (duplicate)**

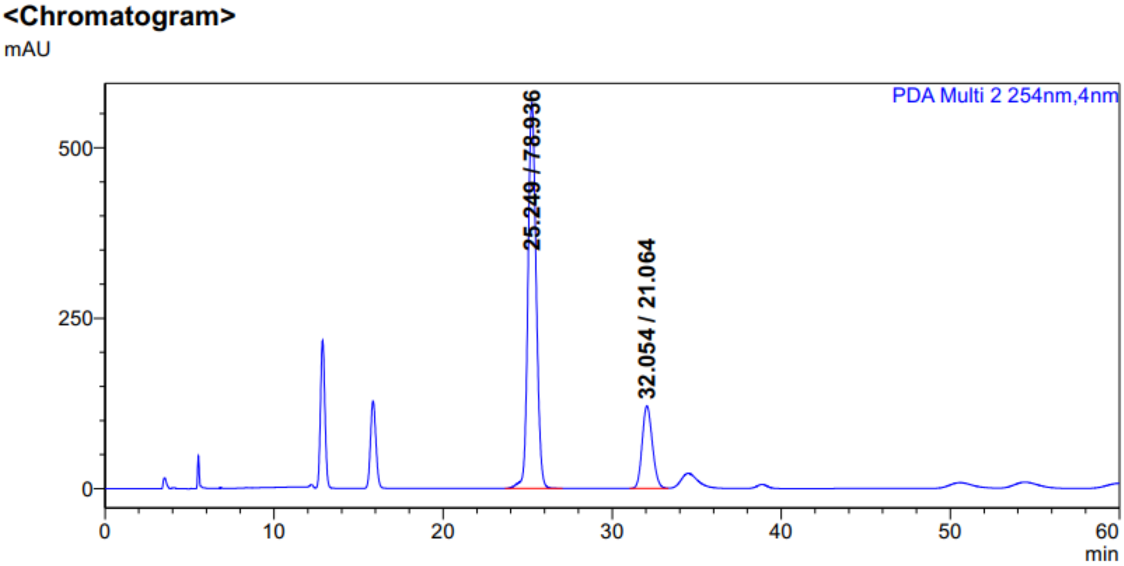

PDA Ch2 254nm

Peak#	Name	Ret. Time	Area	Area%
1		25.249	19026838	78.936
2		32.054	5077413	21.064
Total			24104251	100.000

HPLC traces for reaction with: **Ph-phenylboronic acid**

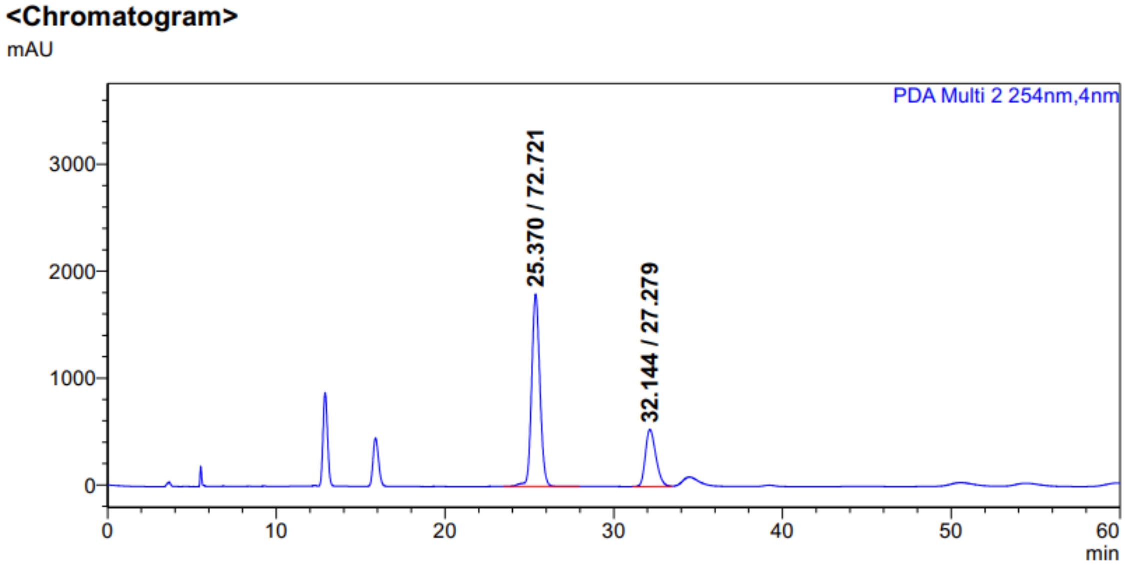

PDA Ch2 254nm

Peak#	Name	Ret. Time	Area	Area%
1		25.370	61565179	72.721
2		32.144	23094655	27.279
Total			84659834	100.000

HPLC traces for reaction with: **Ph-phenylboronic acid (duplicate)**

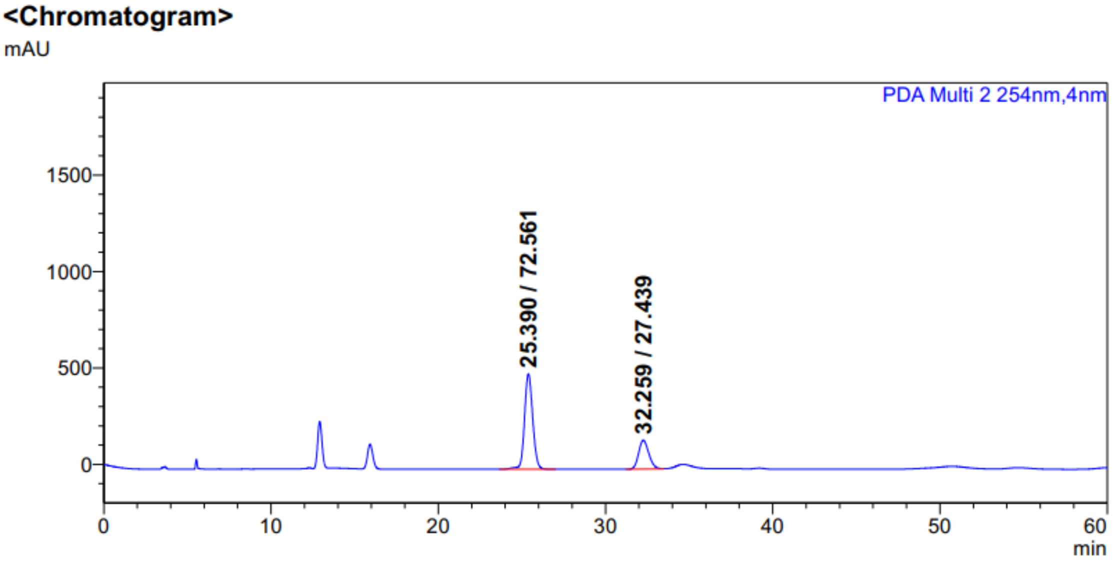

PDA Ch2 254nm

Peak#	Name	Ret. Time	Area	Area%
1		25.390	16830821	72.561
2		32.259	6364596	27.439
Total			23195416	100.000

HPLC traces for reaction with: **2-F-phenylboronic acid**

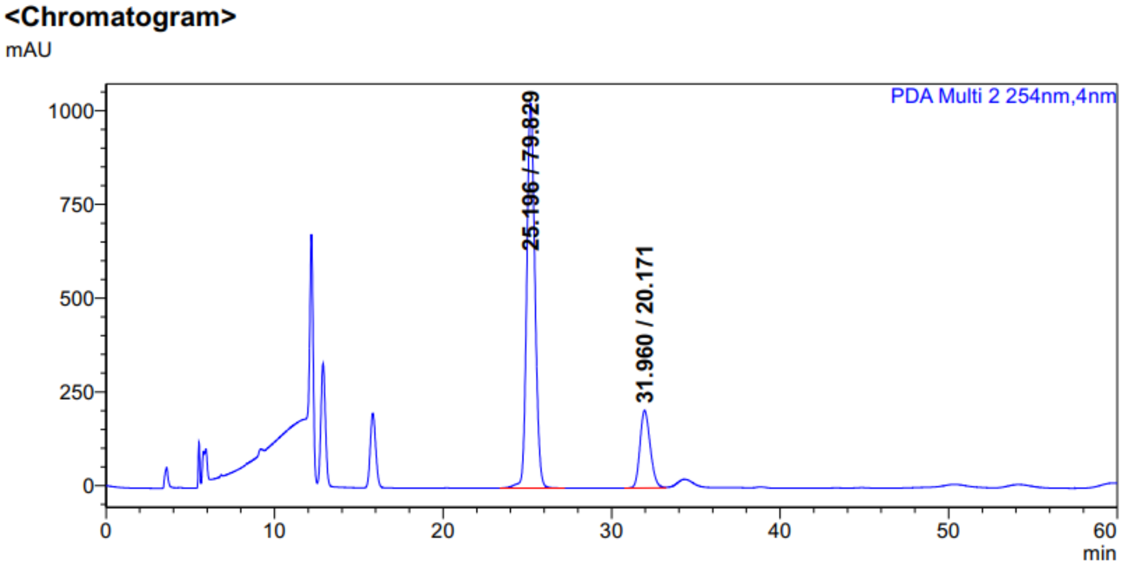

PDA Ch2 254nm

Peak#	Name	Ret. Time	Area	Area%
1		25.196	34688635	79.829
2		31.960	8765272	20.171
Total			43453907	100.000

HPLC traces for reaction with: **2-F-phenylboronic acid (duplicate)**

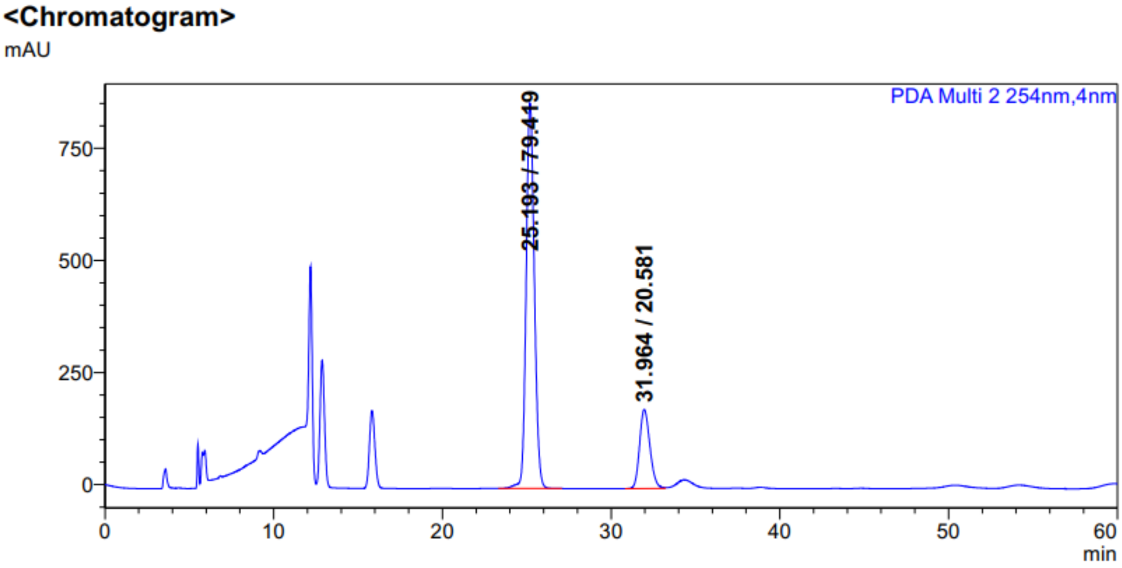

PDA Ch2 254nm

Peak#	Name	Ret. Time	Area	Area%
1		25.193	29035760	79.419
2		31.964	7524545	20.581
Total			36560305	100.000

HPLC traces for reaction **without boronic acid**:

<Chromatogram>

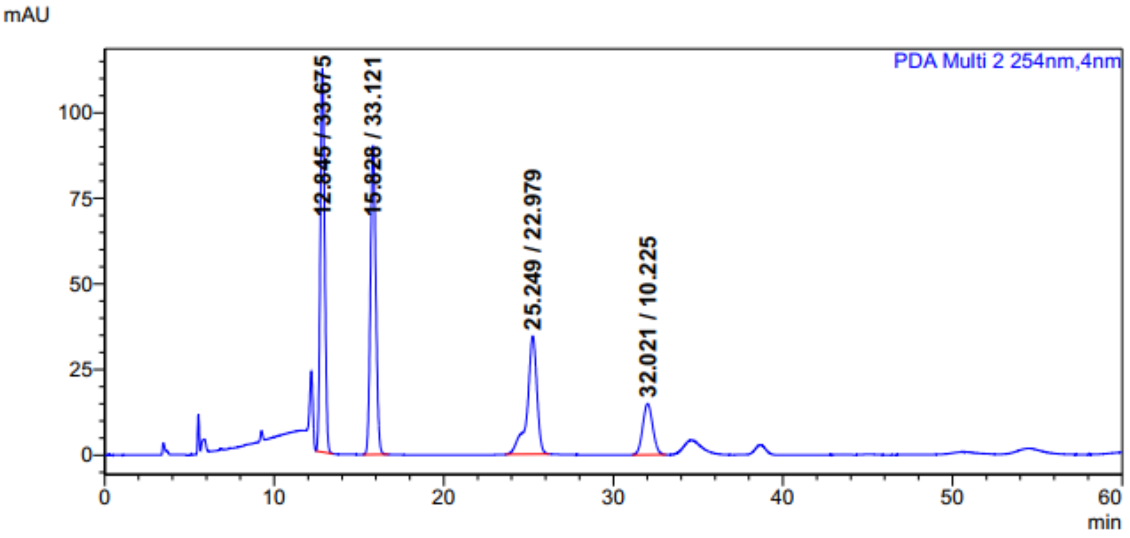

PDA Ch2 254nm

Peak#	Name	Ret. Time	Area	Area%
1		12.845	2021823	33.675
2		15.828	1988546	33.121
3		25.249	1379602	22.979
4		32.021	613872	10.225
Total			6003844	100.000

HPLC traces for reaction **without boronic acid (duplicate)**:

<Chromatogram>

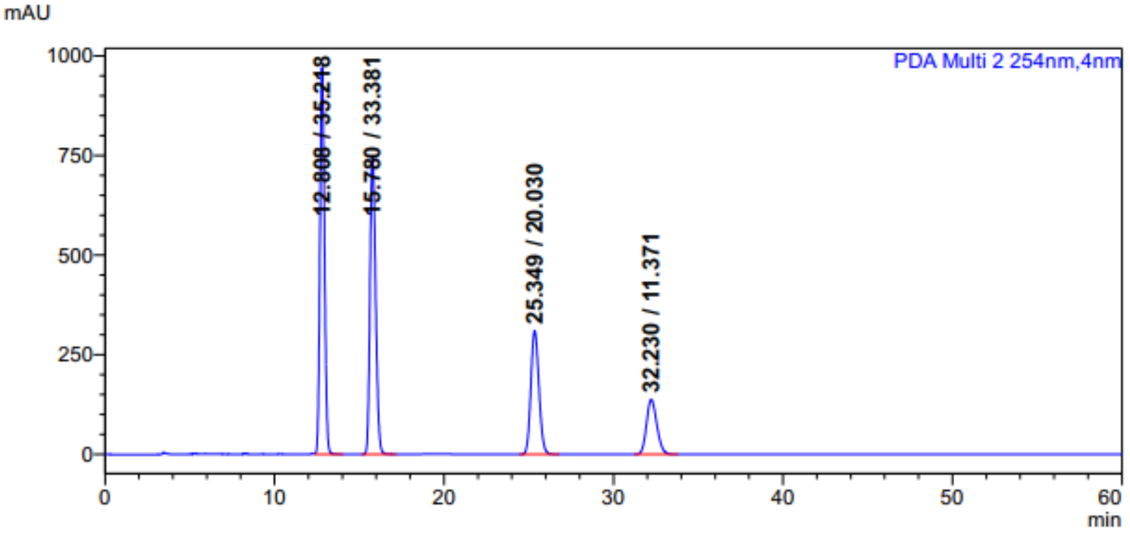

PDA Ch2 254nm

Peak#	Name	Ret. Time	Area	Area%
1		12.808	18093540	35.218
2		15.780	17149977	33.381
3		25.349	10290539	20.030
4		32.230	5841915	11.371
Total			51375971	100.000

HPLC traces for reaction **without water, with 2-F-phenylboronic acid (duplicate)**:

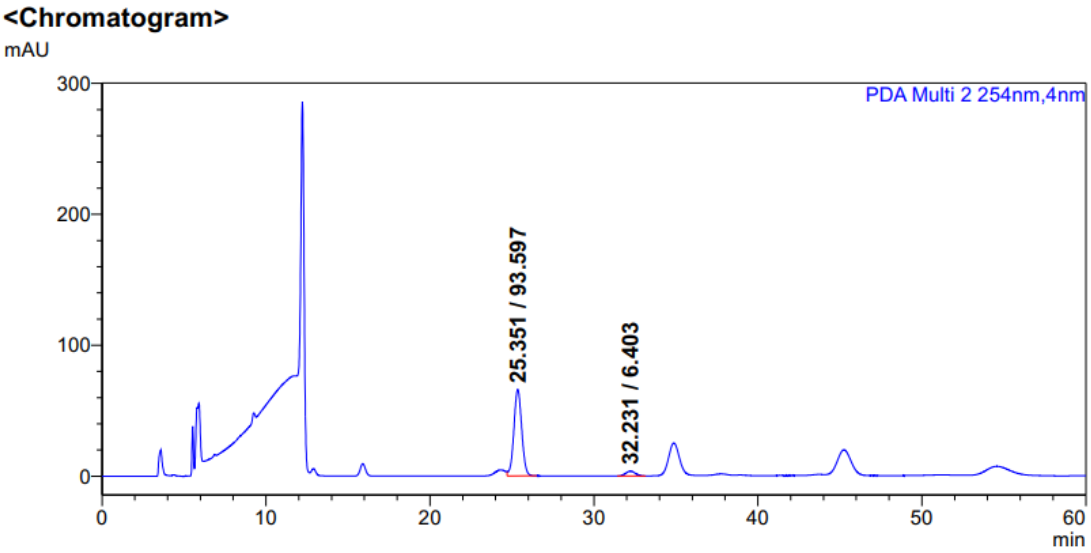

PDA Ch2 254nm

Peak#	Name	Ret. Time	Area	Area%
1		25.351	2195091	93.597
2		32.231	150166	6.403
Total			2345257	100.000

HPLC traces for reaction **without water, with 2-F-phenylboronic acid (duplicate)**:

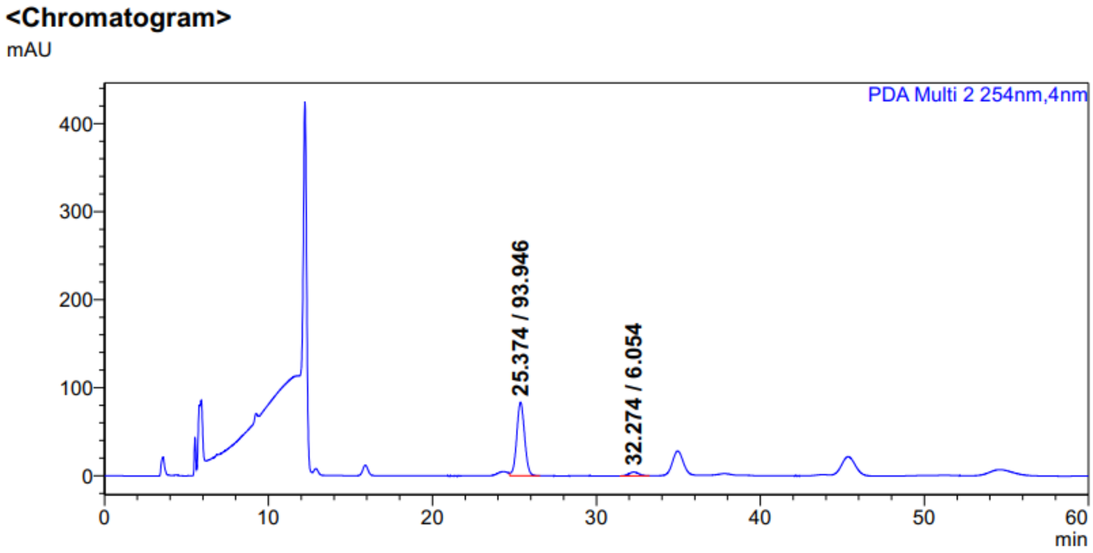

PDA Ch2 254nm

Peak#	Name	Ret. Time	Area	Area%
1		25.374	2780307	93.946
2		32.274	179159	6.054
Total			2959466	100.000

13. HPLC Traces for Table S6. Reducing amount of ketone with 3,5-F-phenyl boronic acid

All of the reactions were performed as duplicate following GP3 with 3,5-F-phenylboronic acid. A racemic sample was first analyzed in order to determine the retention time of both enantiomers. For the chiral sample, enantiomeric excess was determined by comparing the integrated area of these two peaks. All the HPLC traces shown below are of (S)-2-((R)-hydroxy(4-nitrophenyl)methyl)cyclopentan-1-one, yielded by different reactions as described in table S6.

HPLC traces for reaction with: **3,5-F-phenylboronic acid**

3,5-F-phenylboronic acid (6.8 eq cyclopentanone, 3 hours reaction)

<Chromatogram>

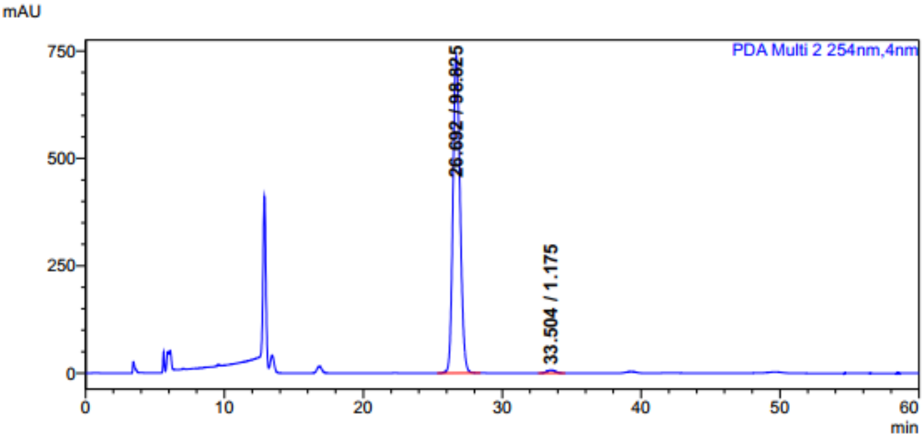

PDA Ch2 254nm

Peak#	Name	Ret. Time	Area	Area%
1		26.692	26350807	98.825
2		33.504	313261	1.175
Total			26664068	100.000

3,5-F-phenylboronic acid (6.8 eq cyclopentanone, 3 hours reaction) - duplicate

<Chromatogram>

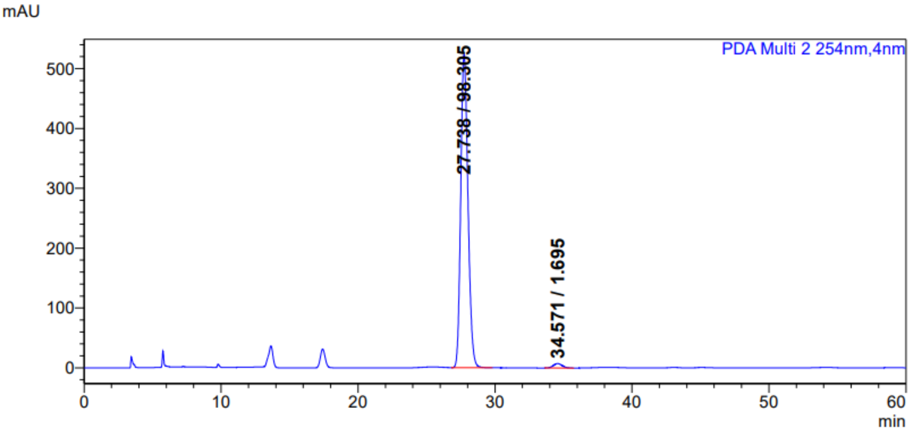

PDA Ch2 254nm

Peak#	Name	Ret. Time	Area	Area%
1		27.738	19239750	98.305
2		34.571	331706	1.695
Total			19571456	100.000

3,5-F-phenylboronic acid (6.8 eq cyclopentanone, 6 hours reaction)

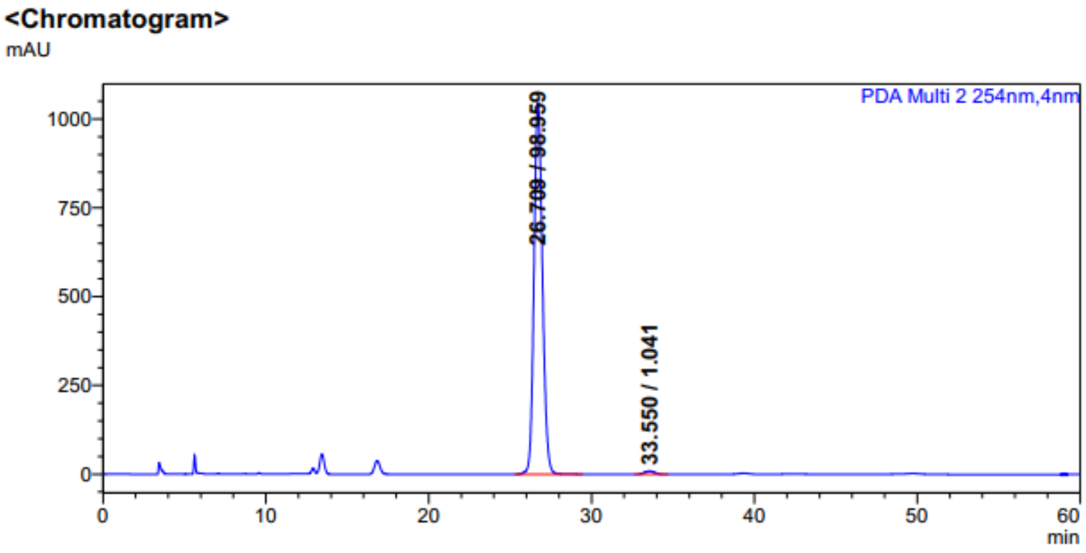

PDA Ch2 254nm

Peak#	Name	Ret. Time	Area	Area%
1		26.709	37702507	98.959
2		33.550	396749	1.041
Total			38099255	100.000

3,5-F-phenylboronic acid (6.8 eq cyclopentanone, 6 hours reaction) - duplicate

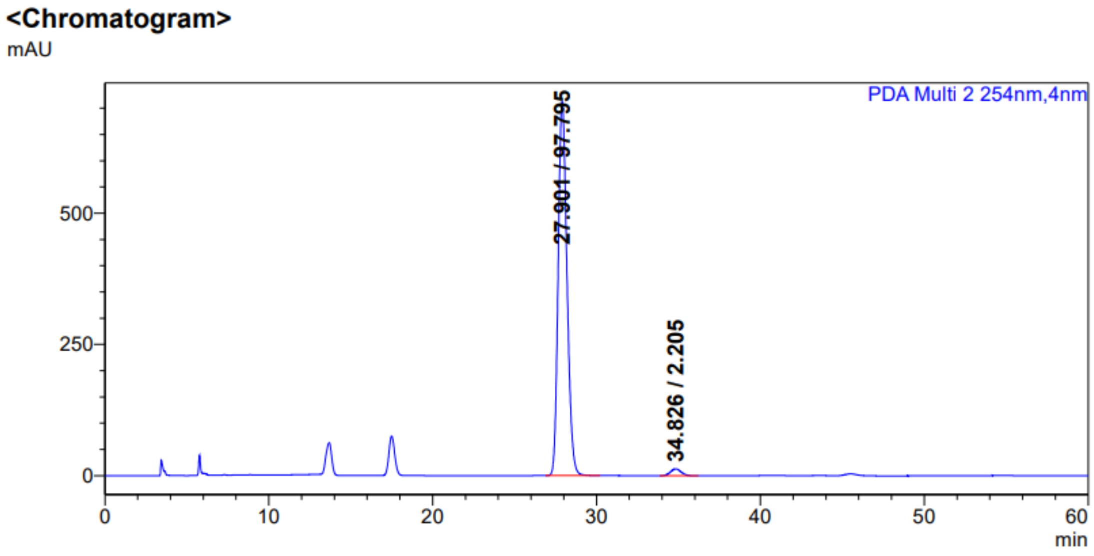

PDA Ch2 254nm

Peak#	Name	Ret. Time	Area	Area%
1		27.901	26596665	97.795
2		34.826	599771	2.205
Total			27196436	100.000

3,5-F-phenylboronic acid (5 eq cyclopentanone, 9 hours reaction)

<Chromatogram>

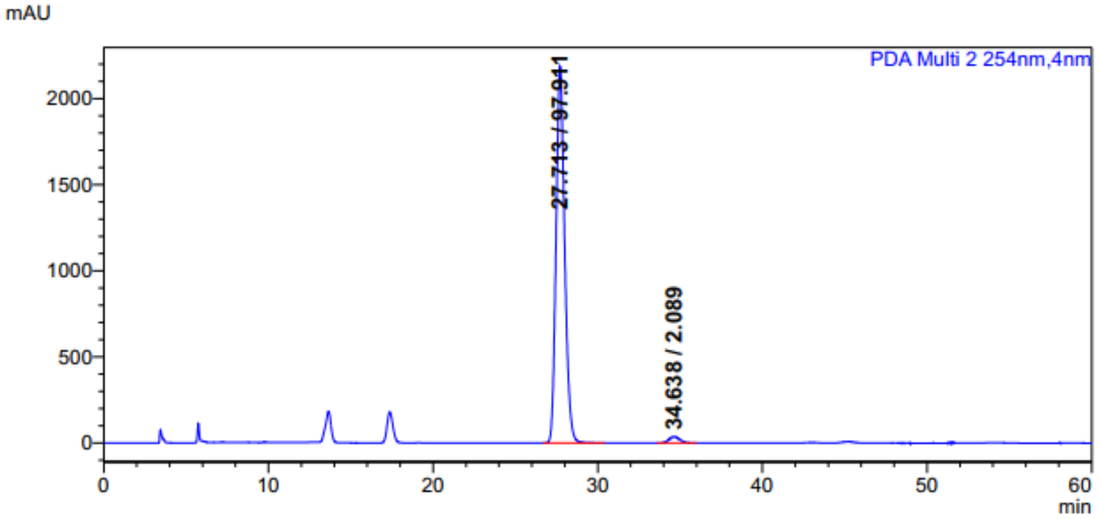

PDA Ch2 254nm

Peak#	Name	Ret. Time	Area	Area%
1		27.713	79107538	97.911
2		34.638	1688070	2.089
Total			80795608	100.000

3,5-F-phenylboronic acid (5 eq cyclopentanone, 9 hours reaction) – duplicate

<Chromatogram>

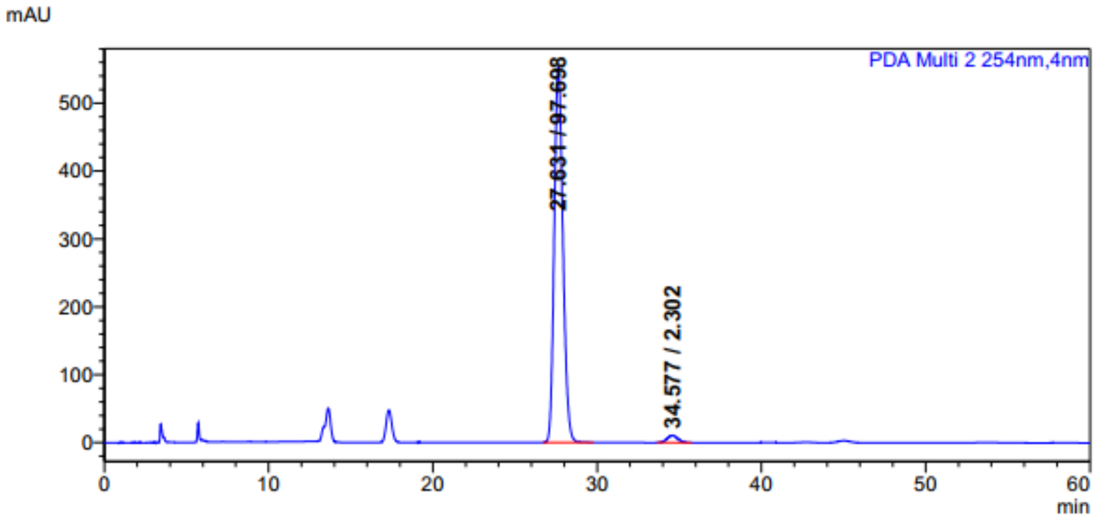

PDA Ch2 254nm

Peak#	Name	Ret. Time	Area	Area%
1		27.631	20385739	97.698
2		34.577	480275	2.302
Total			20866014	100.000

3,5-F-phenylboronic acid (3 eq cyclopentanone, 9 hours reaction)

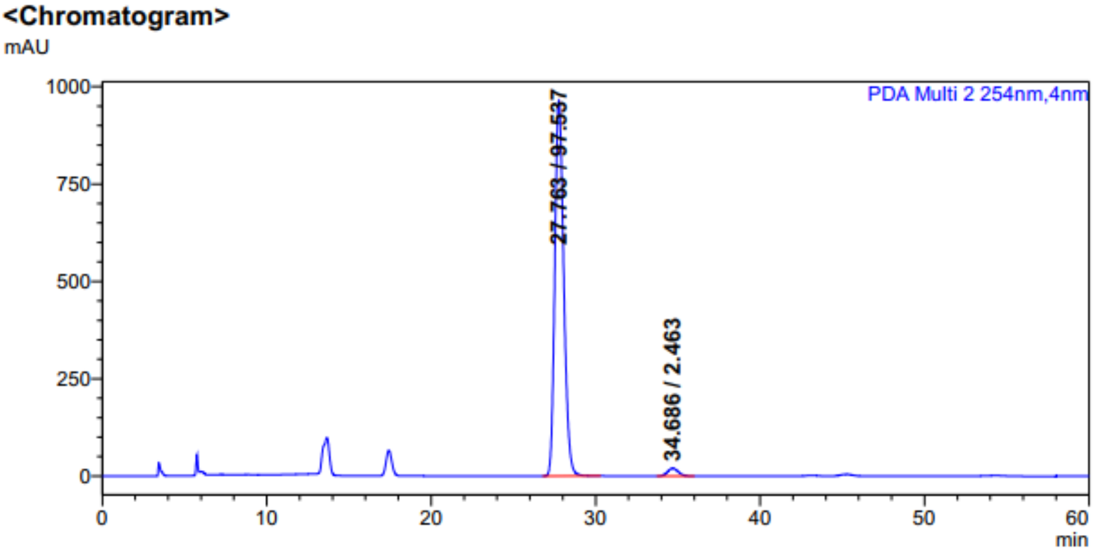

PDA Ch2 254nm

Peak#	Name	Ret. Time	Area	Area%
1		27.763	36168226	97.537
2		34.686	913212	2.463
Total			37081438	100.000

3,5-F-phenylboronic acid (3 eq cyclopentanone, 9 hours reaction) - duplicate

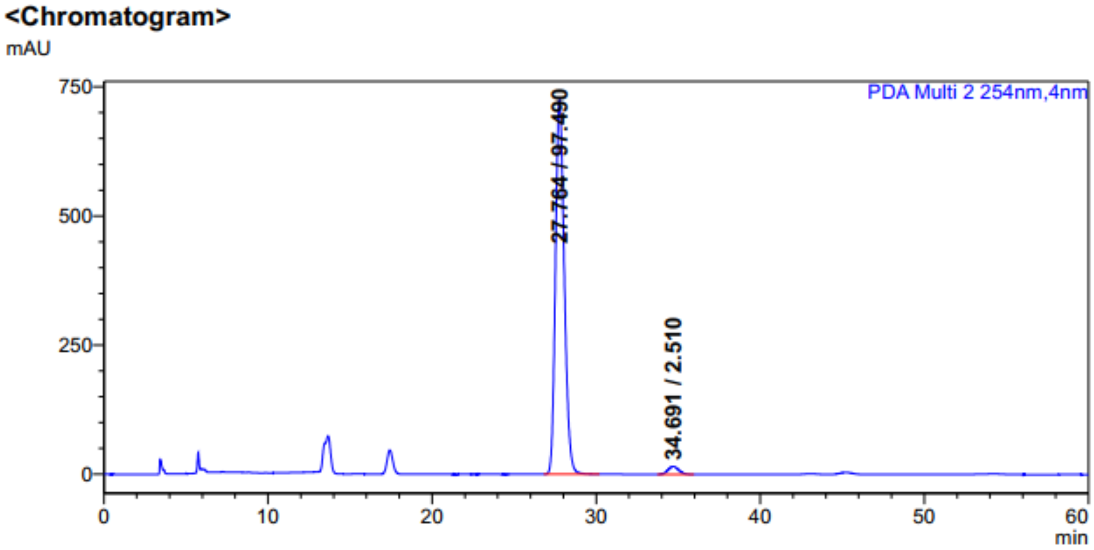

PDA Ch2 254nm

Peak#	Name	Ret. Time	Area	Area%
1		27.764	27046200	97.490
2		34.691	696468	2.510
Total			27742668	100.000

3,5-F-phenylboronic acid (2 eq cyclopentanone, 9 hours reaction)

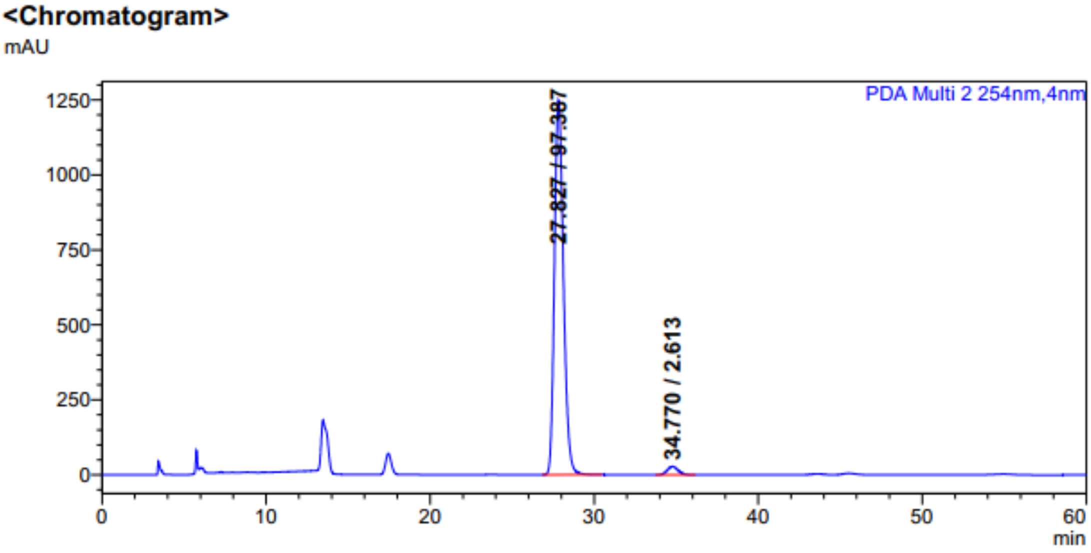

PDA Ch2 254nm

Peak#	Name	Ret. Time	Area	Area%
1		27.827	47504110	97.387
2		34.770	1274597	2.613
Total			48778707	100.000

3,5-F-phenylboronic acid (2 eq cyclopentanone, 9 hours reaction) – duplicate

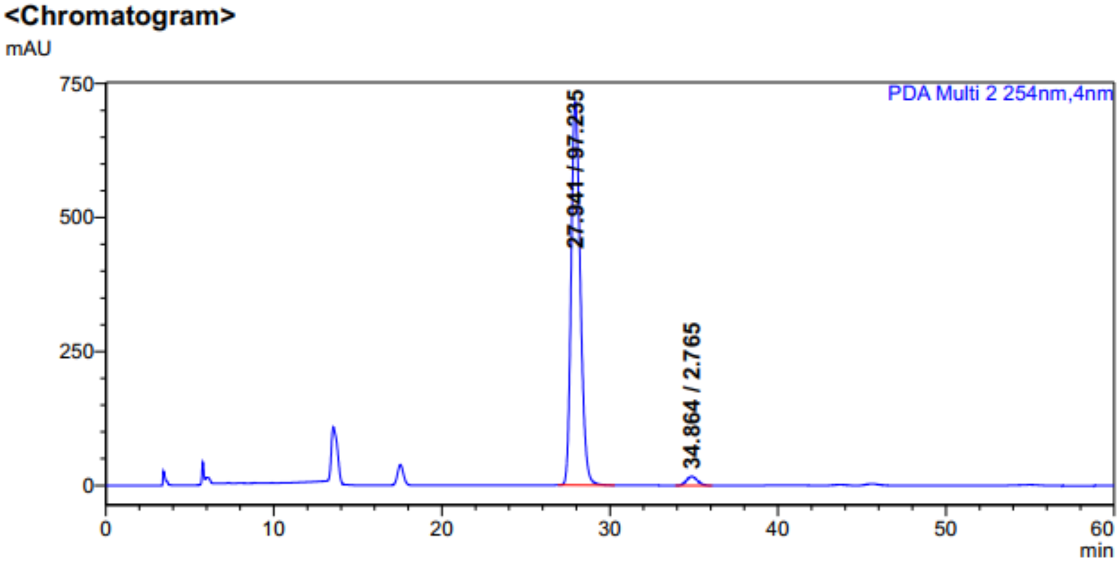

PDA Ch2 254nm

Peak#	Name	Ret. Time	Area	Area%
1		27.941	27074213	97.235
2		34.864	769795	2.765
Total			27844008	100.000

3,5-F-phenylboronic acid (1.5 eq cyclopentanone, 9 hours reaction)

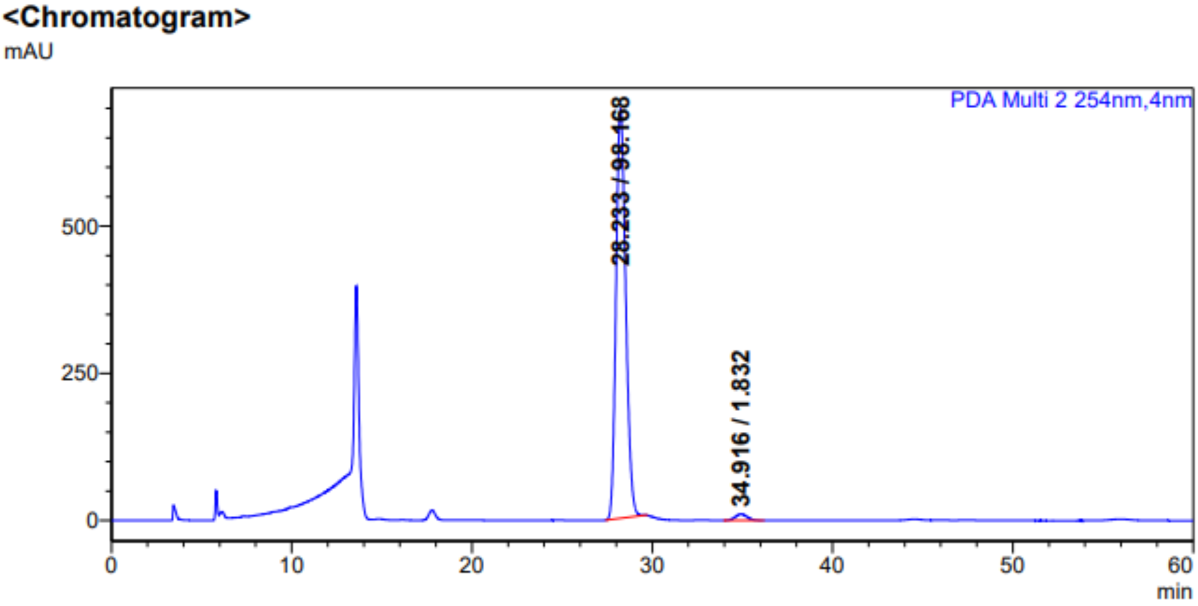

PDA Ch2 254nm

Peak#	Name	Ret. Time	Area	Area%
1		28.233	25933387	98.168
2		34.916	483917	1.832
Total			26417305	100.000

3,5-F-phenylboronic acid (1.5 eq cyclopentanone, 9 hours reaction) – duplicate

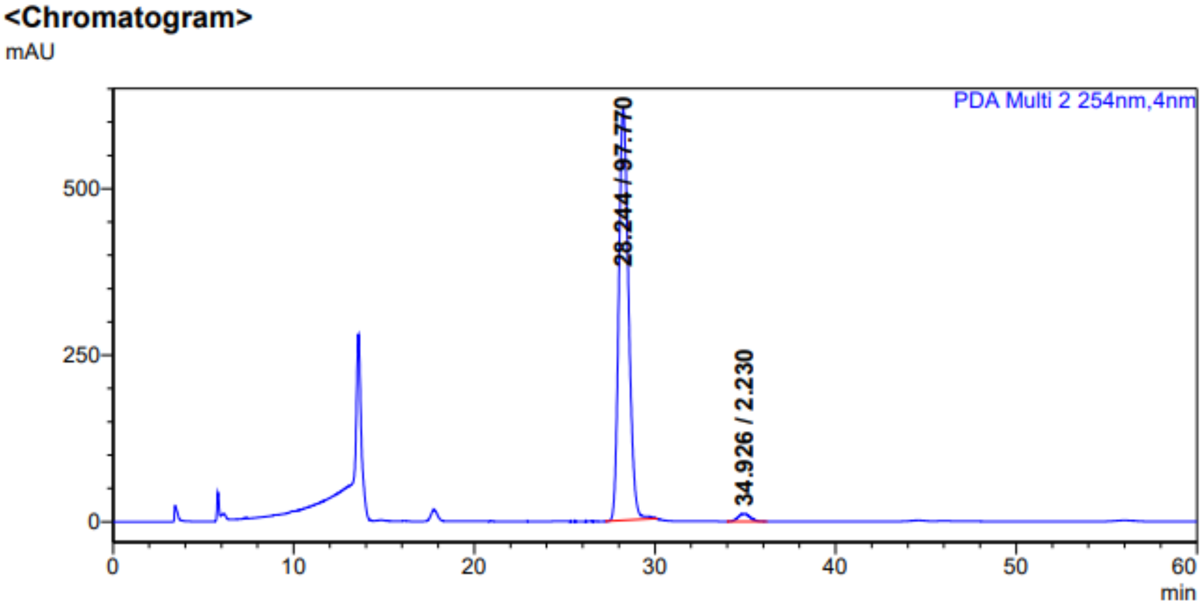

PDA Ch2 254nm

Peak#	Name	Ret. Time	Area	Area%
1		28.244	23212017	97.770
2		34.926	529504	2.230
Total			23741521	100.000

3,5-F-phenylboronic acid (1.2 eq cyclopentanone, 9 hours reaction)

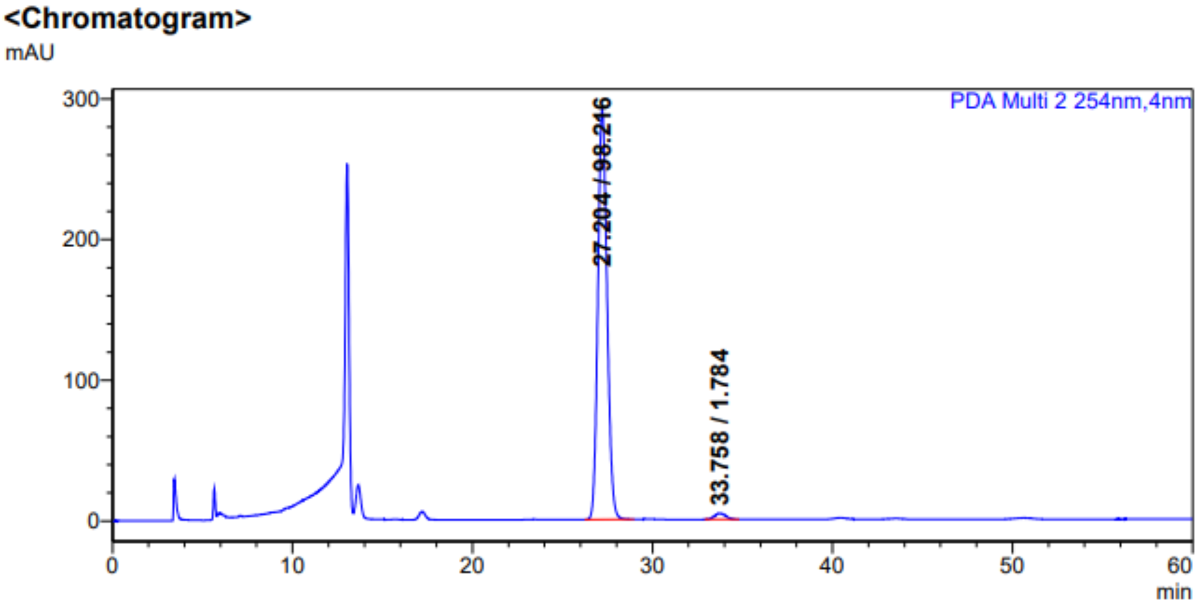

PDA Ch2 254nm

Peak#	Name	Ret. Time	Area	Area%
1		27.204	10358520	98.216
2		33.758	188177	1.784
Total			10546697	100.000

3,5-F-phenylboronic acid (1.2 eq cyclopentanone, 9 hours reaction) – duplicate

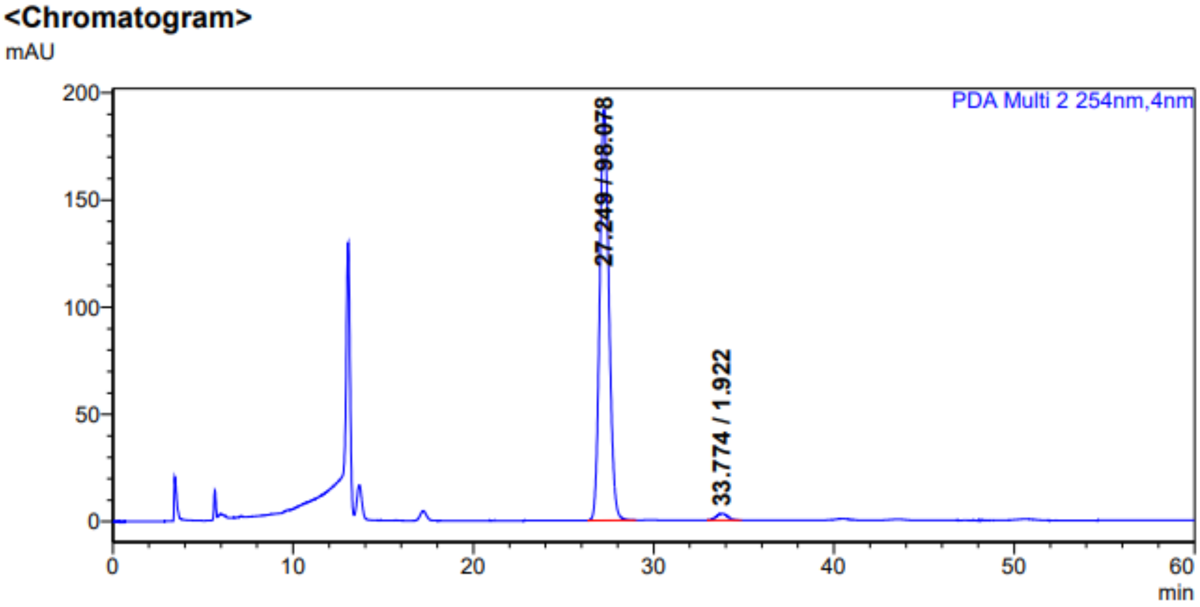

PDA Ch2 254nm

Peak#	Name	Ret. Time	Area	Area%
1		27.249	6832812	98.078
2		33.774	133879	1.922
Total			6966691	100.000

3,5-F-phenylboronic acid (1 eq cyclopentanone, 9 hours reaction)

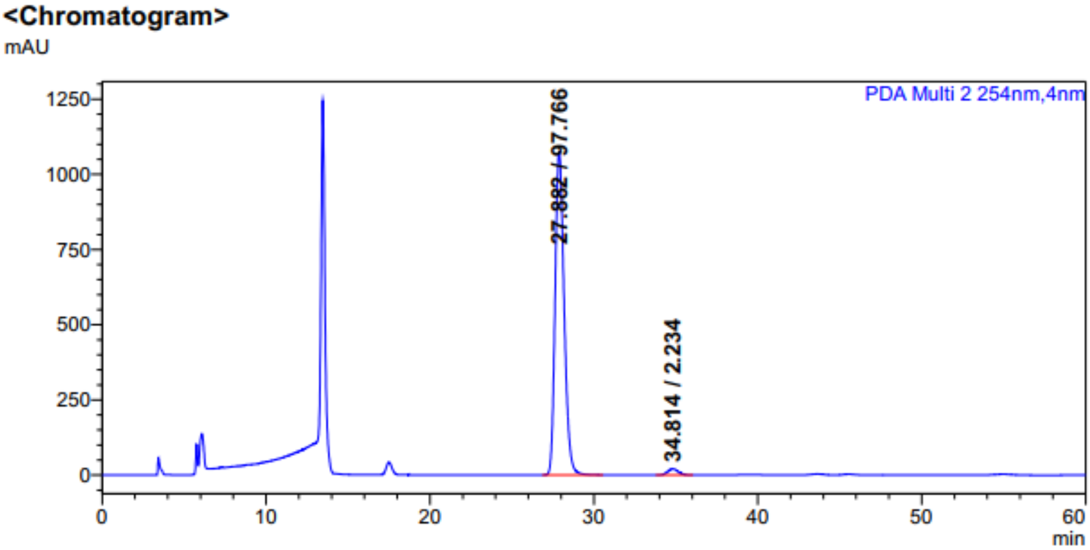

PDA Ch2 254nm

Peak#	Name	Ret. Time	Area	Area%
1		27.882	40902702	97.766
2		34.814	934531	2.234
Total			41837233	100.000

3,5-F-phenylboronic acid (1 eq cyclopentanone, 9 hours reaction) – duplicate

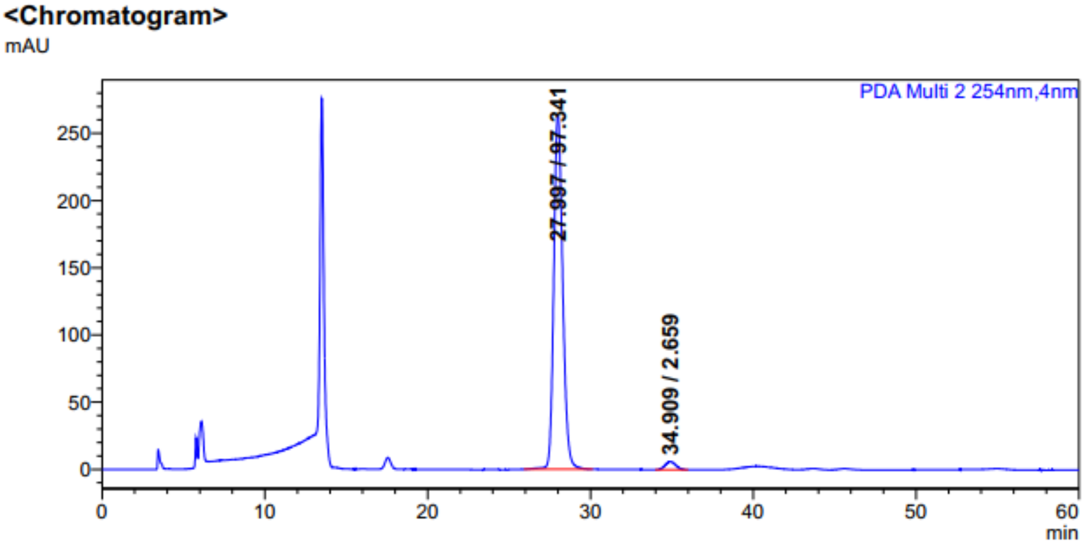

PDA Ch2 254nm

Peak#	Name	Ret. Time	Area	Area%
1		27.997	10016498	97.341
2		34.909	273610	2.659
Total			10290108	100.000

3,5-F-phenylboronic acid (5 eq cyclopentanone, 6 hours reaction)

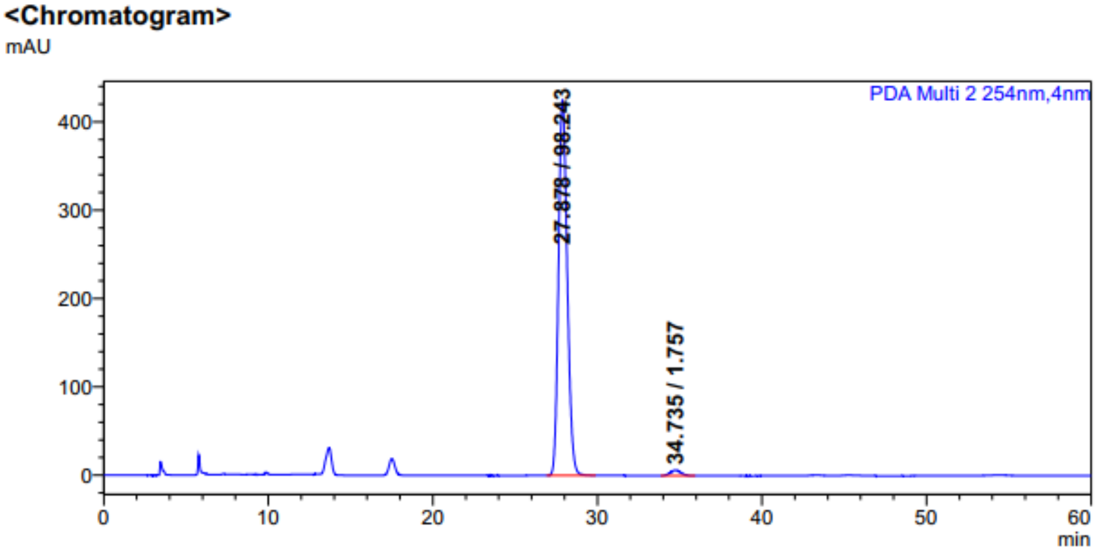

PDA Ch2 254nm

Peak#	Name	Ret. Time	Area	Area%
1		27.878	15674112	98.243
2		34.735	280298	1.757
Total			15954410	100.000

3,5-F-phenylboronic acid (5 eq cyclopentanone, 6 hours reaction) – duplicate

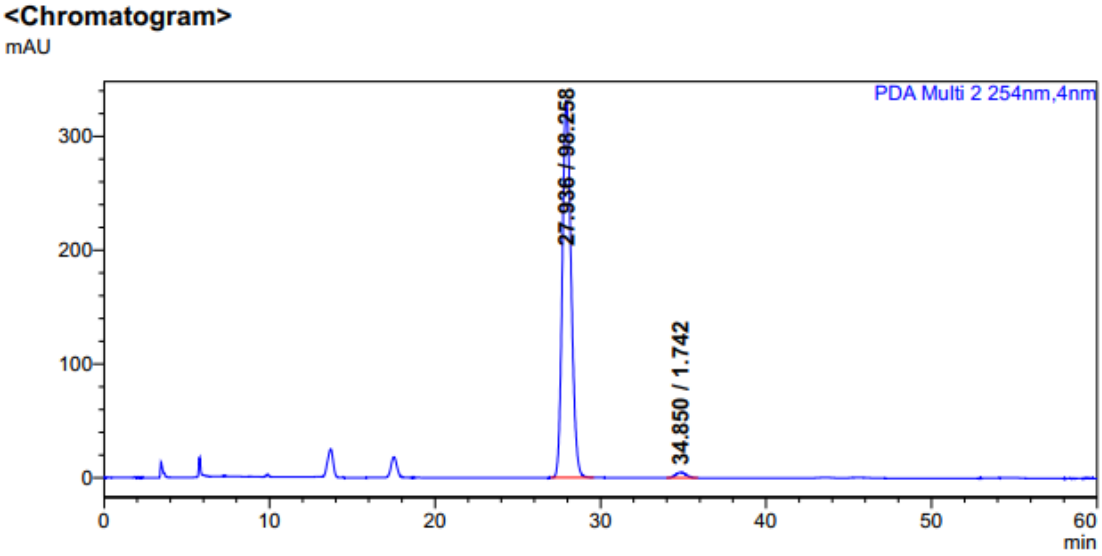

PDA Ch2 254nm

Peak#	Name	Ret. Time	Area	Area%
1		27.936	12237358	98.258
2		34.850	216998	1.742
Total			12454356	100.000

3,5-F-phenylboronic acid (3 eq cyclopentanone, 6 hours reaction)

<Chromatogram>

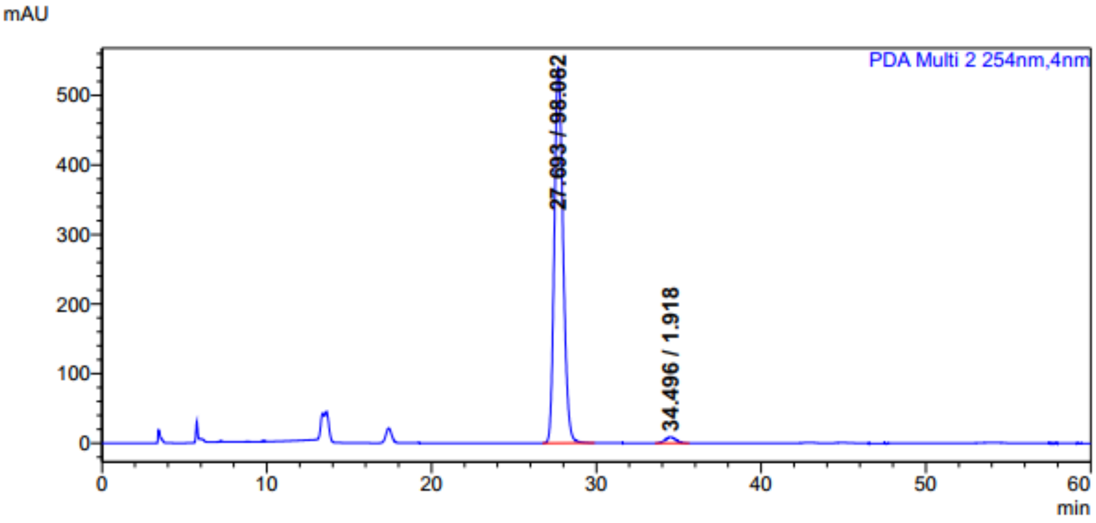

PDA Ch2 254nm

Peak#	Name	Ret. Time	Area	Area%
1		27.693	19918453	98.082
2		34.496	389501	1.918
Total			20307954	100.000

3,5-F-phenylboronic acid (3 eq cyclopentanone, 6 hours reaction) – duplicate

<Chromatogram>

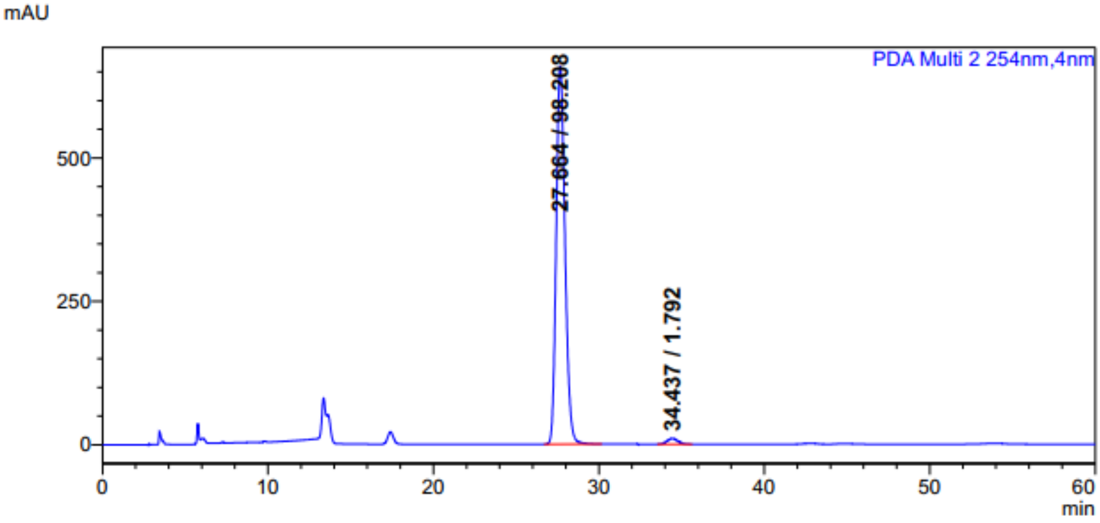

PDA Ch2 254nm

Peak#	Name	Ret. Time	Area	Area%
1		27.664	24305558	98.208
2		34.437	443484	1.792
Total			24749041	100.000

3,5-F-phenylboronic acid (2 eq cyclopentanone, 6 hours reaction)

<Chromatogram>

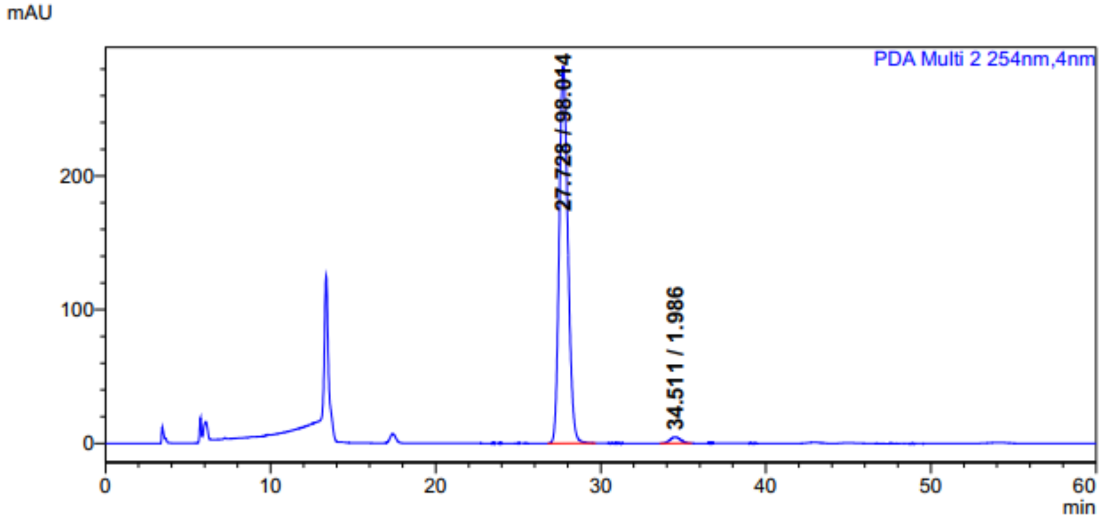

PDA Ch2 254nm

Peak#	Name	Ret. Time	Area	Area%
1		27.728	10342557	98.014
2		34.511	209515	1.986
Total			10552071	100.000

3,5-F-phenylboronic acid (2 eq cyclopentanone, 6 hours reaction) – duplicate

<Chromatogram>

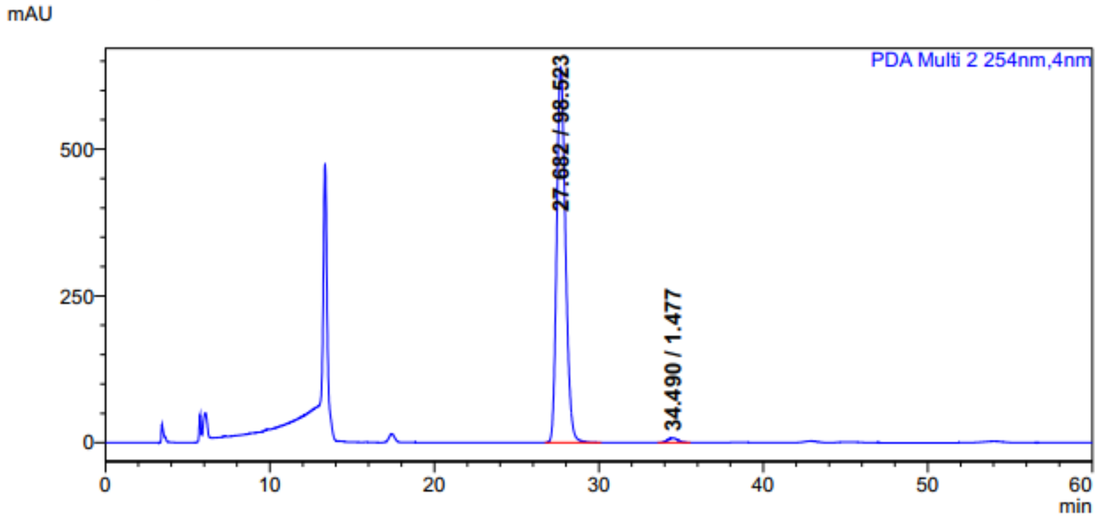

PDA Ch2 254nm

Peak#	Name	Ret. Time	Area	Area%
1		27.682	23732584	98.523
2		34.490	355892	1.477
Total			24088476	100.000

3,5-F-phenylboronic acid (1 eq cyclopentanone, 6 hours reaction)

<Chromatogram>

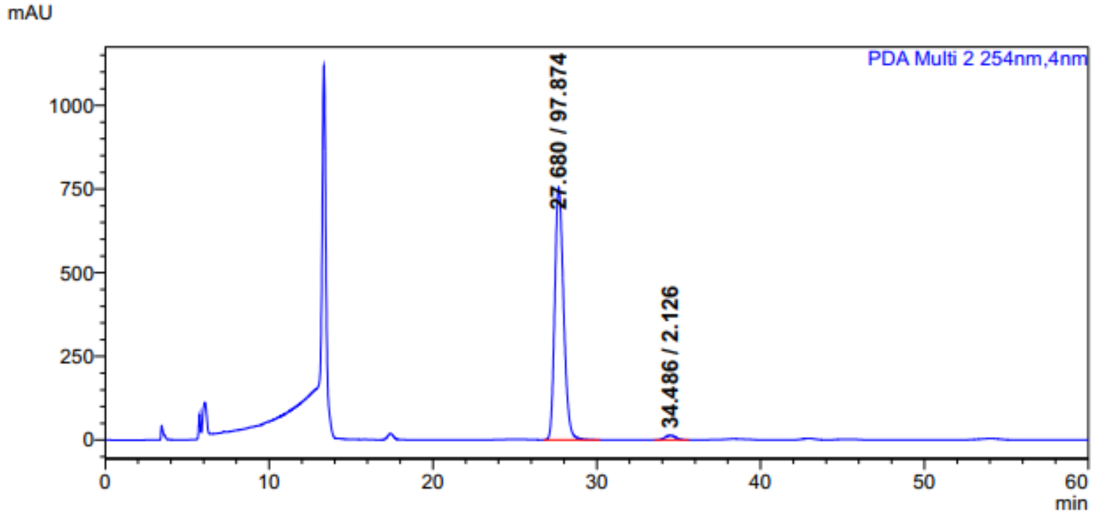

PDA Ch2 254nm

Peak#	Name	Ret. Time	Area	Area%
1		27.680	28225685	97.874
2		34.486	612978	2.126
Total			28838663	100.000

3,5-F-phenylboronic acid (1 eq cyclopentanone, 6 hours reaction) - duplicate

<Chromatogram>

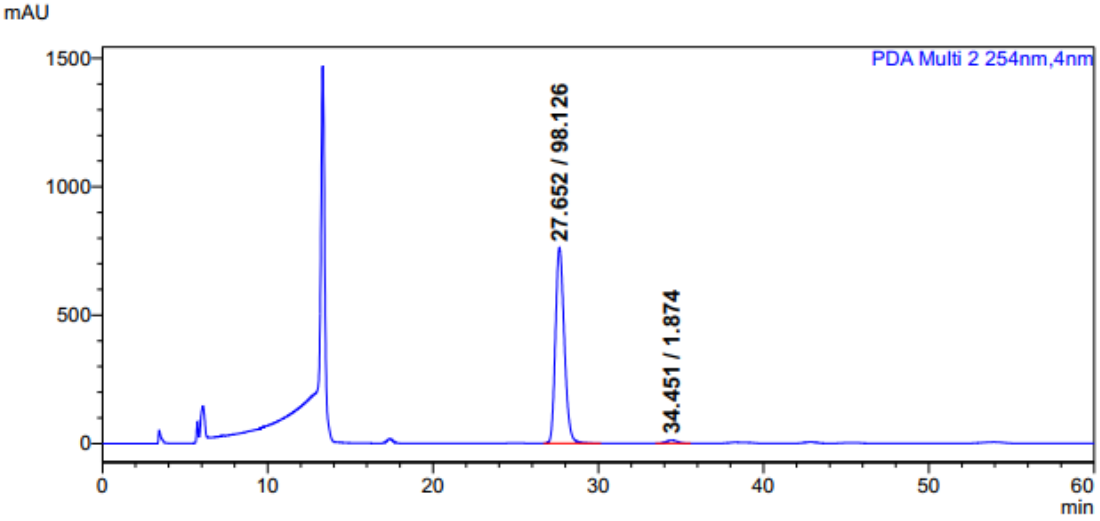

PDA Ch2 254nm

Peak#	Name	Ret. Time	Area	Area%
1		27.652	28447163	98.126
2		34.451	543231	1.874
Total			28990393	100.000

14. HPLC Traces for Table S7. Reducing amount of ketone with 3-CF_3-phenyl boronic acid

All of the reactions were performed as duplicate following GP3 with 3-CF_3-phenylboronic acid. A racemic sample was first analyzed in order to determine the retention time of both enantiomers. For the chiral sample, enantiomeric excess was determined by comparing the integrated area of these two peaks. All the HPLC traces shown below are of (S)-2-((R)-hydroxy(4-nitrophenyl)methyl)cyclopentan-1-one, yielded by different reactions as described in table S7.

HPLC traces for reaction with: **3-CF_3-phenylboronic acid**

3-CF_3-phenylboronic acid (2 eq cyclopentanone, 1 hours reaction)

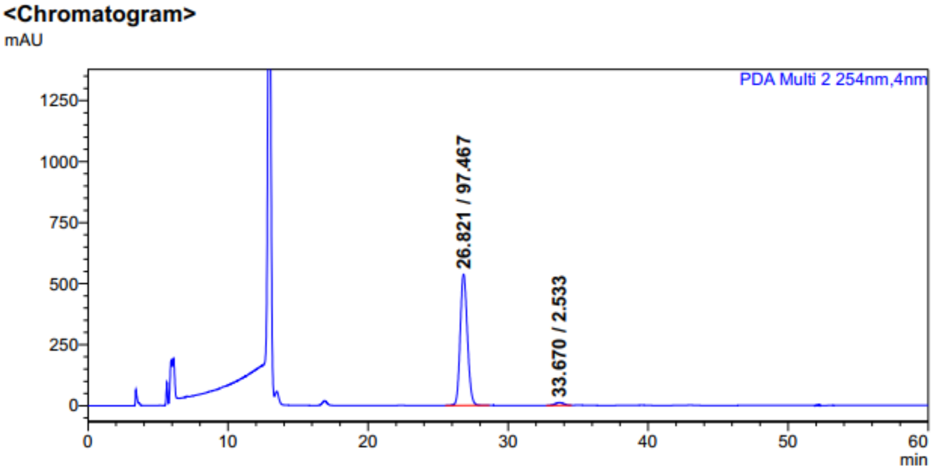

PDA Ch2 254nm

Peak#	Name	Ret. Time	Area	Area%
1		26.821	19117218	97.467
2		33.670	496875	2.533
Total			19614093	100.000

3-CF_3-phenylboronic acid (2 eq cyclopentanone, 1 hours reaction) – duplicate

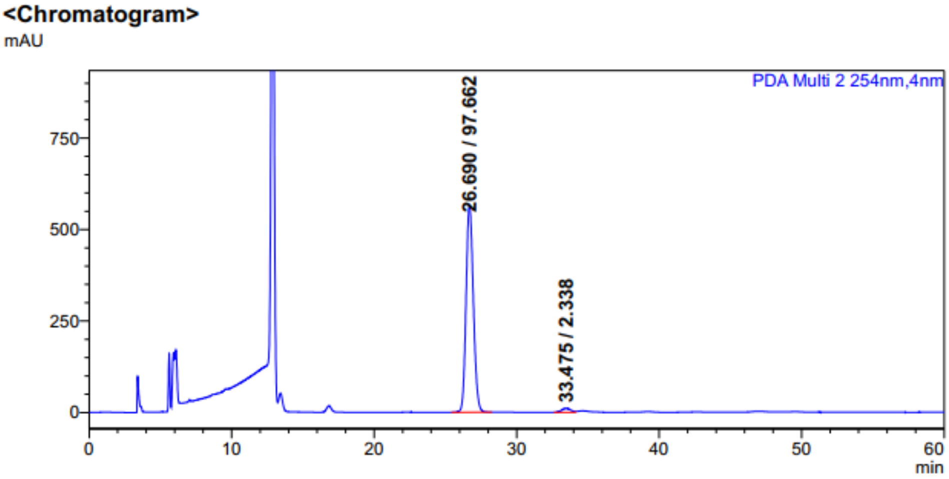

PDA Ch2 254nm

Peak#	Name	Ret. Time	Area	Area%
1		26.690	20026720	97.662
2		33.475	479400	2.338
Total			20506121	100.000

3-CF_3-phenylboronic acid (2 eq cyclopentanone, 3 hours reaction)

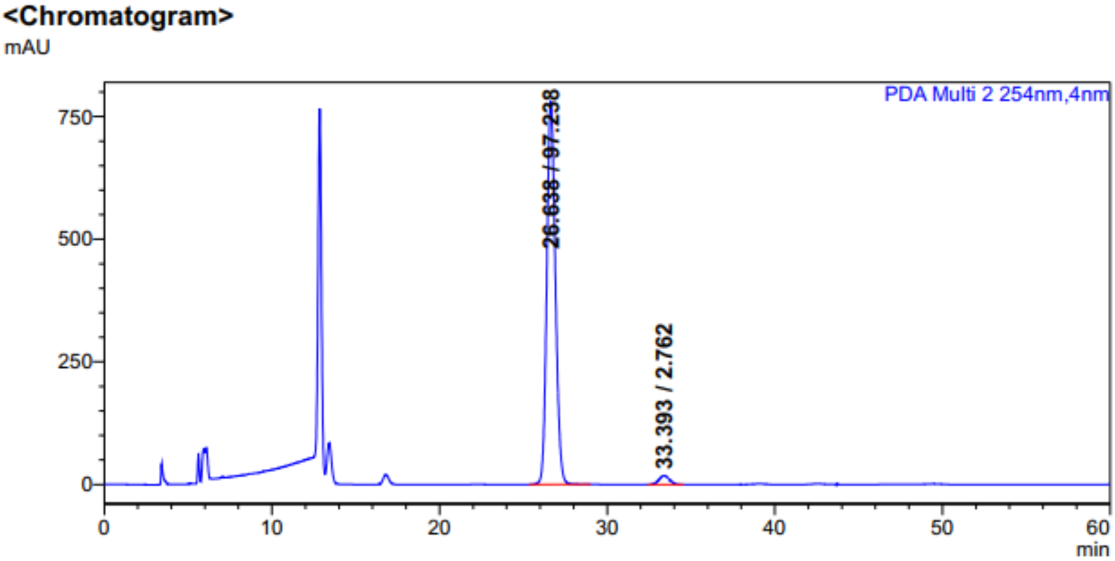

PDA Ch2 254nm

Peak#	Name	Ret. Time	Area	Area%
1		26.638	27711011	97.238
2		33.393	787166	2.762
Total			28498177	100.000

3-CF_3-phenylboronic acid (2 eq cyclopentanone, 3 hours reaction) – duplicate

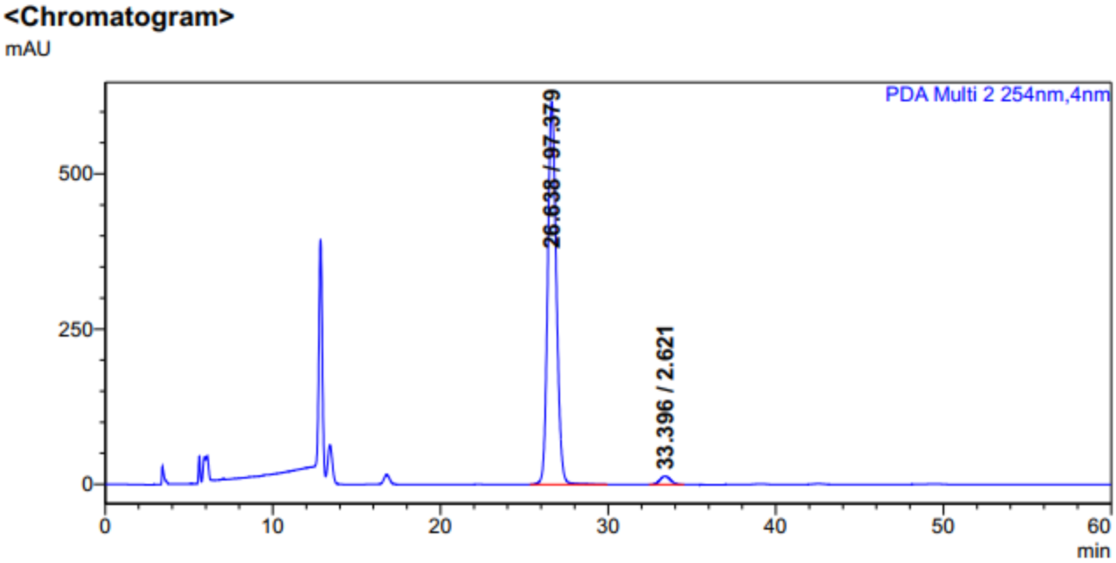

PDA Ch2 254nm

Peak#	Name	Ret. Time	Area	Area%
1		26.638	22050617	97.379
2		33.396	593604	2.621
Total			22644220	100.000

3-CF_3-phenylboronic acid (2 eq cyclopentanone, 6 hours reaction)

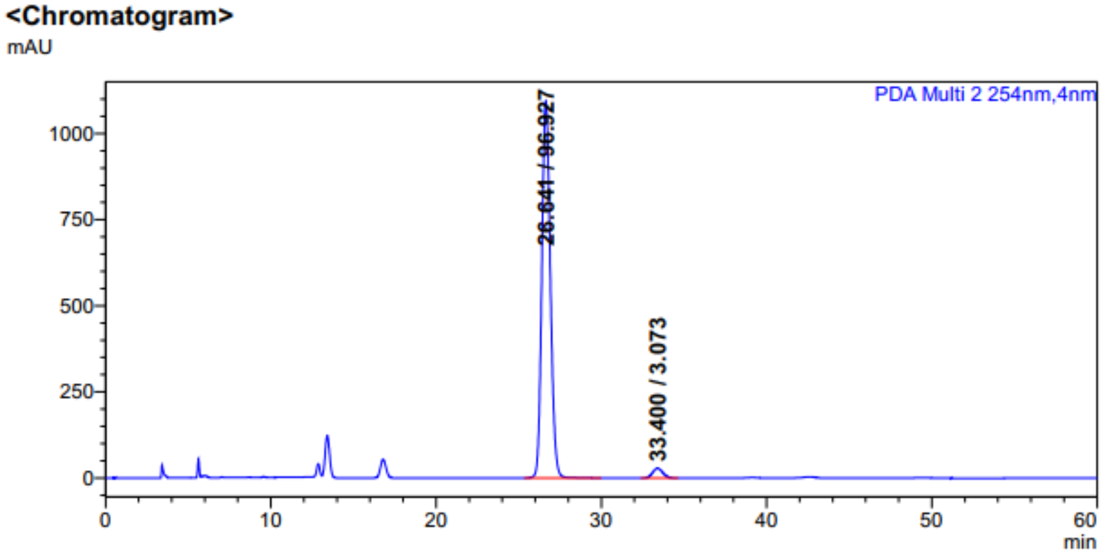

PDA Ch2 254nm

Peak#	Name	Ret. Time	Area	Area%
1		26.641	39113526	96.927
2		33.400	1239942	3.073
Total			40353468	100.000

3-CF_3-phenylboronic acid (2 eq cyclopentanone, 6 hours reaction) – duplicate

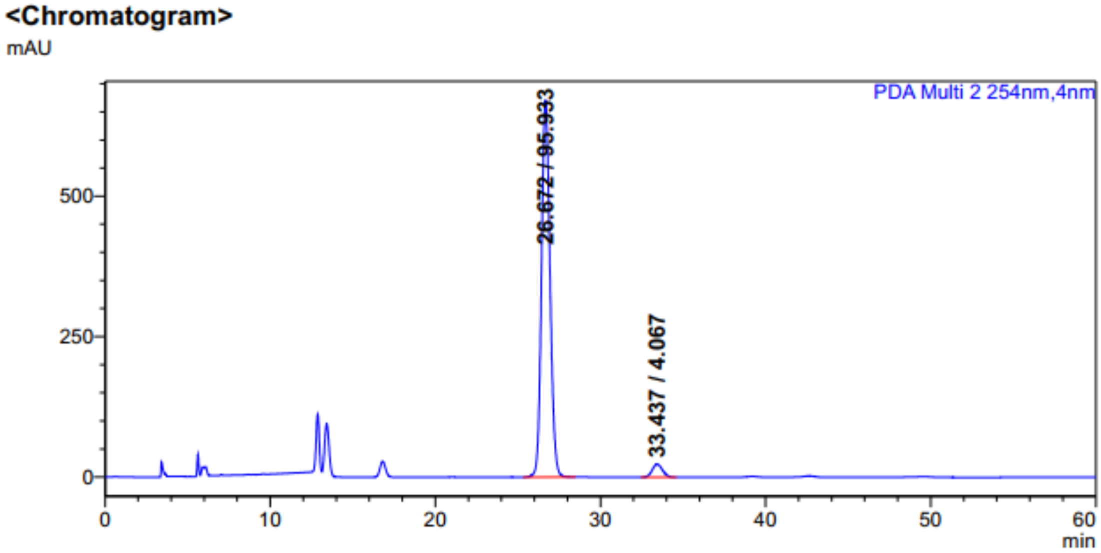

PDA Ch2 254nm

Peak#	Name	Ret. Time	Area	Area%
1		26.672	23961940	95.933
2		33.437	1015743	4.067
Total			24977684	100.000

3-CF_3-phenylboronic acid (2 eq cyclopentanone, overnight reaction)

<Chromatogram>

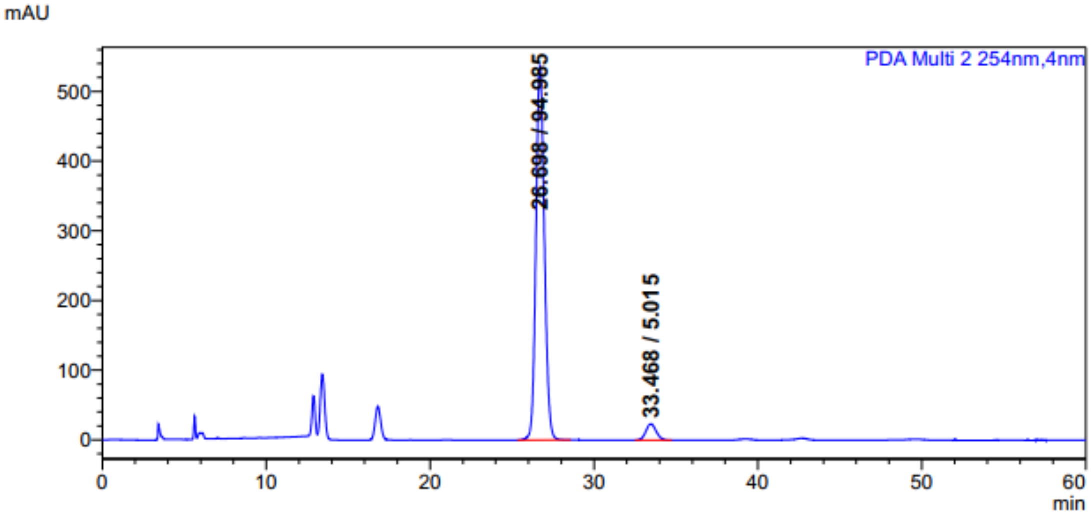

PDA Ch2 254nm

Peak#	Name	Ret. Time	Area	Area%
1		26.698	19174817	94.985
2		33.468	1012420	5.015
Total			20187237	100.000

3-CF_3-phenylboronic acid (2 eq cyclopentanone, overnight reaction) – duplicate

<Chromatogram>

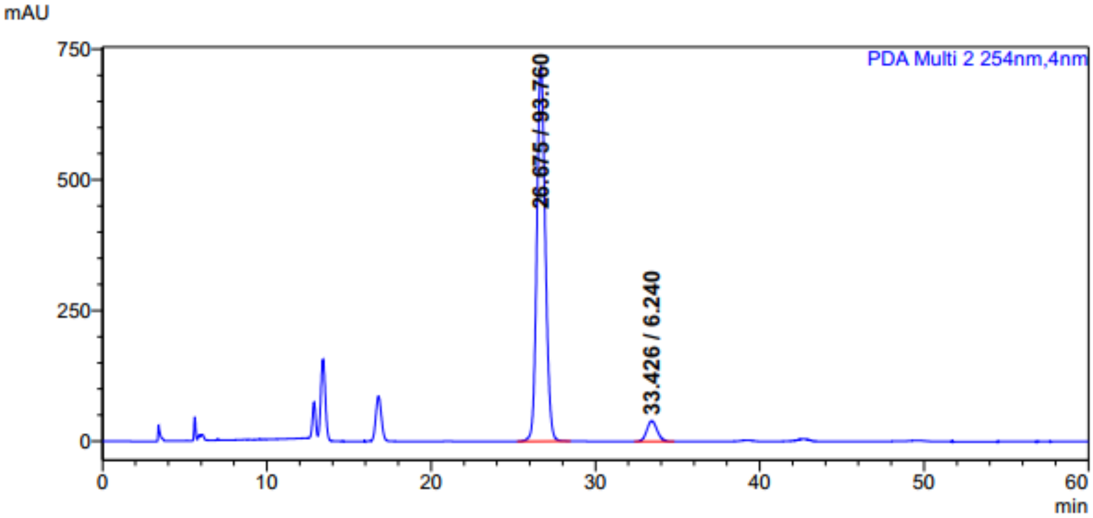

PDA Ch2 254nm

Peak#	Name	Ret. Time	Area	Area%
1		26.675	25764055	93.760
2		33.426	1714609	6.240
Total			27478664	100.000

15. HPLC Traces for Table S8. Evaluating the influence of boronic acid in methyl prolinate catalyzed aldol reaction

All of the reactions were performed as duplicate following GP3 with different phenylboronic acid. A racemic sample was first analyzed in order to determine the retention time of both enantiomers. For the chiral sample, enantiomeric excess was determined by comparing the integrated area of these two peaks. All the HPLC traces shown below are of (S)-2-((R)-hydroxy(4-nitrophenyl)methyl)cyclopentan-1-one, yielded by different reactions as described in table S7.

HPLC traces for reaction with: **No phenylboronic acid**

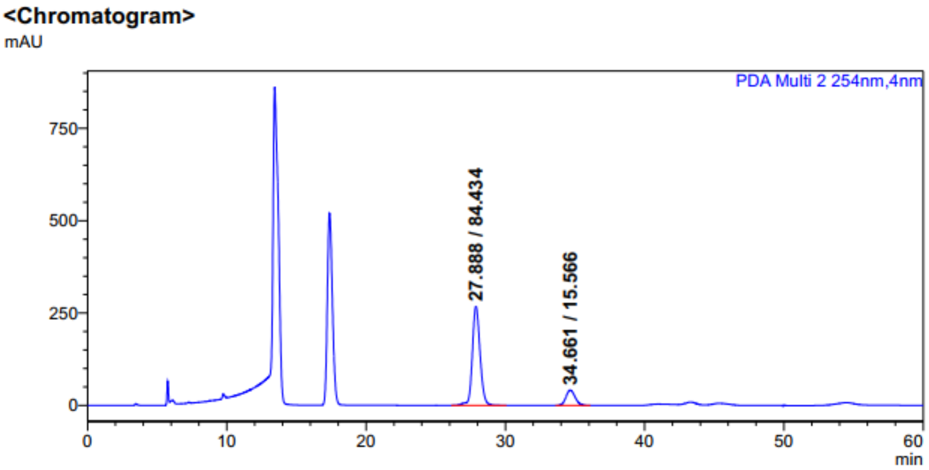

PDA Ch2 254nm

Peak#	Name	Ret. Time	Area	Area%
1		27.888	10292514	84.434
2		34.661	1897535	15.566
Total			12190049	100.000

HPLC traces for reaction with: **No phenylboronic acid – duplicate**

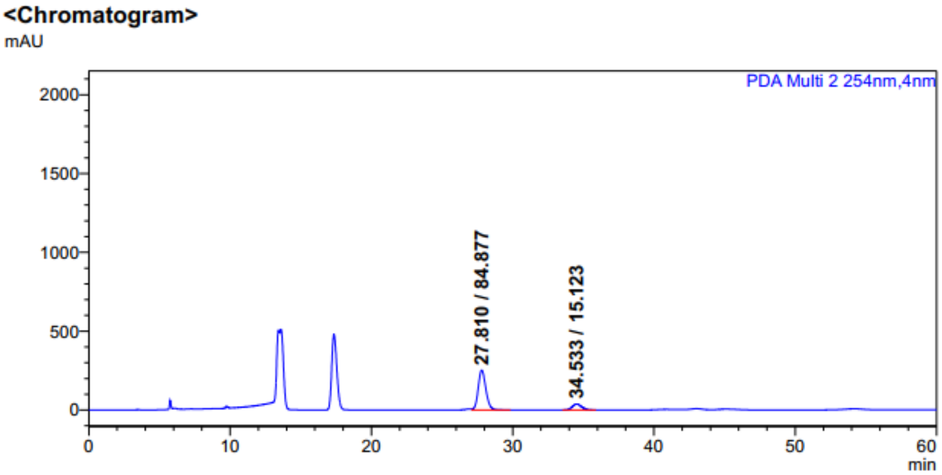

PDA Ch2 254nm

Peak#	Name	Ret. Time	Area	Area%
1		27.810	9473555	84.877
2		34.533	1687904	15.123
Total			11161459	100.000

HPLC traces for reaction with: **2-F-phenylboronic acid**

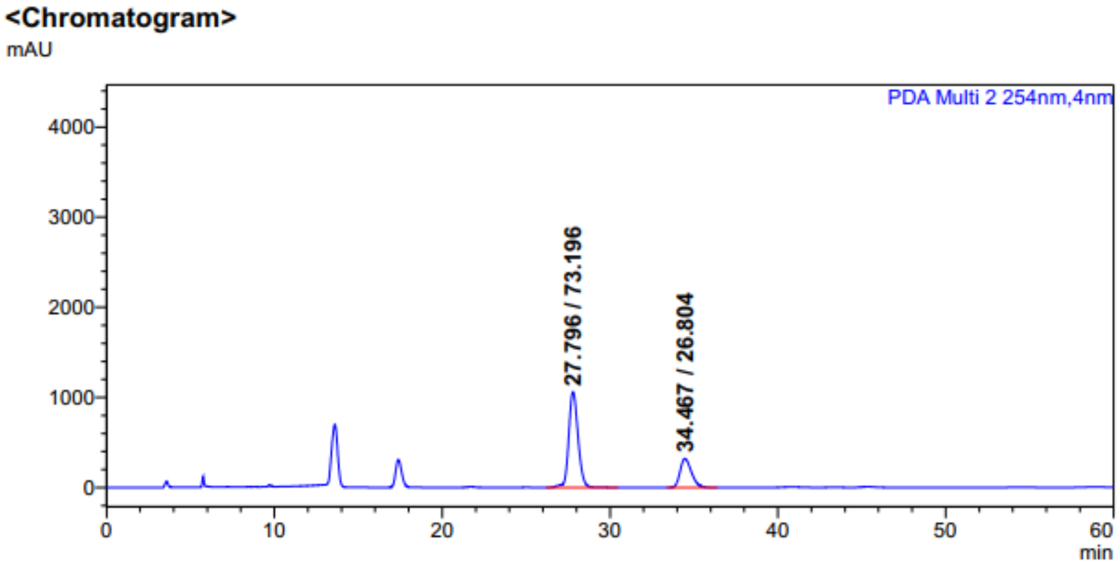

PDA Ch2 254nm

Peak#	Name	Ret. Time	Area	Area%
1		27.796	40986576	73.196
2		34.467	15008938	26.804
Total			55995513	100.000

HPLC traces for reaction with: **2-F-phenylboronic acid – duplicate**

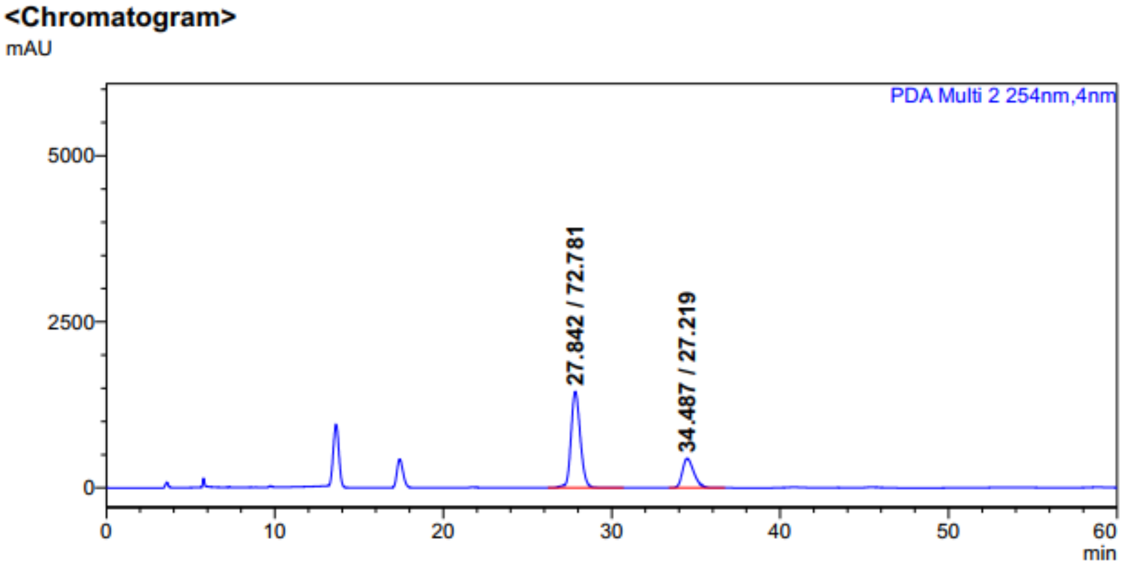

PDA Ch2 254nm

Peak#	Name	Ret. Time	Area	Area%
1		27.842	55634356	72.781
2		34.487	20806618	27.219
Total			76440974	100.000

HPLC traces for reaction with: **2,4-Me-phenylboronic acid**

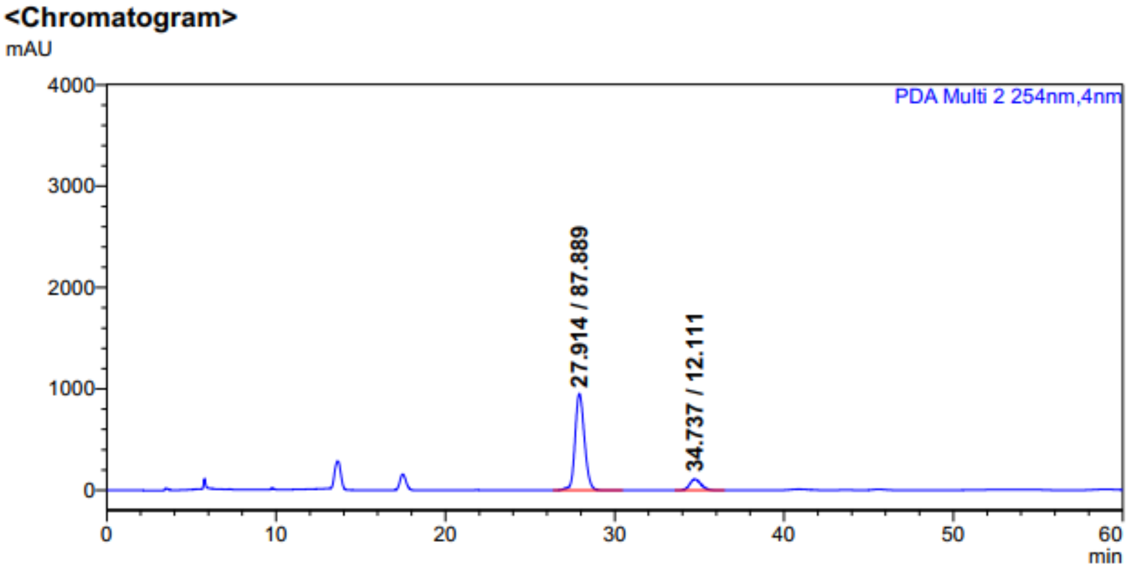

PDA Ch2 254nm

Peak#	Name	Ret. Time	Area	Area%
1		27.914	36750791	87.889
2		34.737	5064176	12.111
Total			41814967	100.000

HPLC traces for reaction with: **2,4-Me-phenylboronic acid - duplicate**

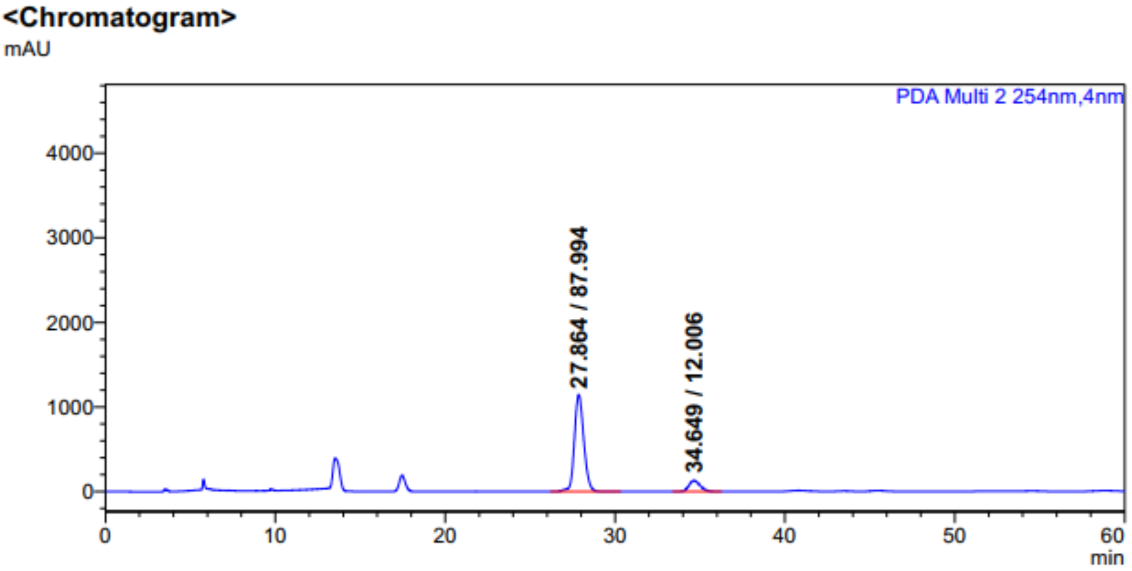

PDA Ch2 254nm

Peak#	Name	Ret. Time	Area	Area%
1		27.864	44157355	87.994
2		34.649	6024732	12.006
Total			50182088	100.000

HPLC traces for reaction with: **3,5-OMe-phenylboronic acid**

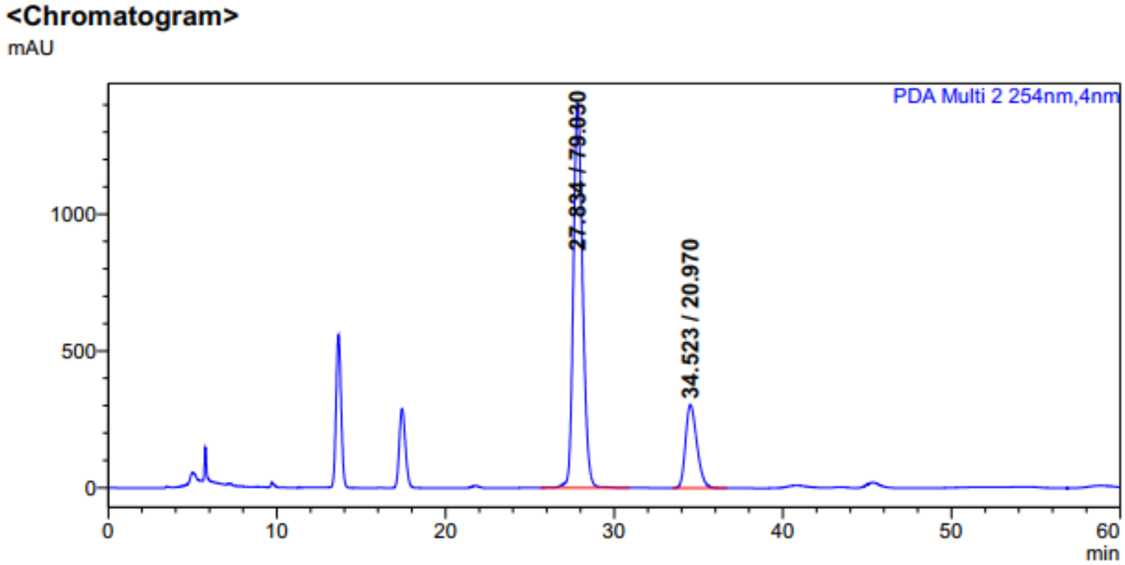

PDA Ch2 254nm

Peak#	Name	Ret. Time	Area	Area%
1		27.834	53695778	79.030
2		34.523	14247637	20.970
Total			67943415	100.000

HPLC traces for reaction with: **3,5-OMe-phenylboronic acid - duplicate**

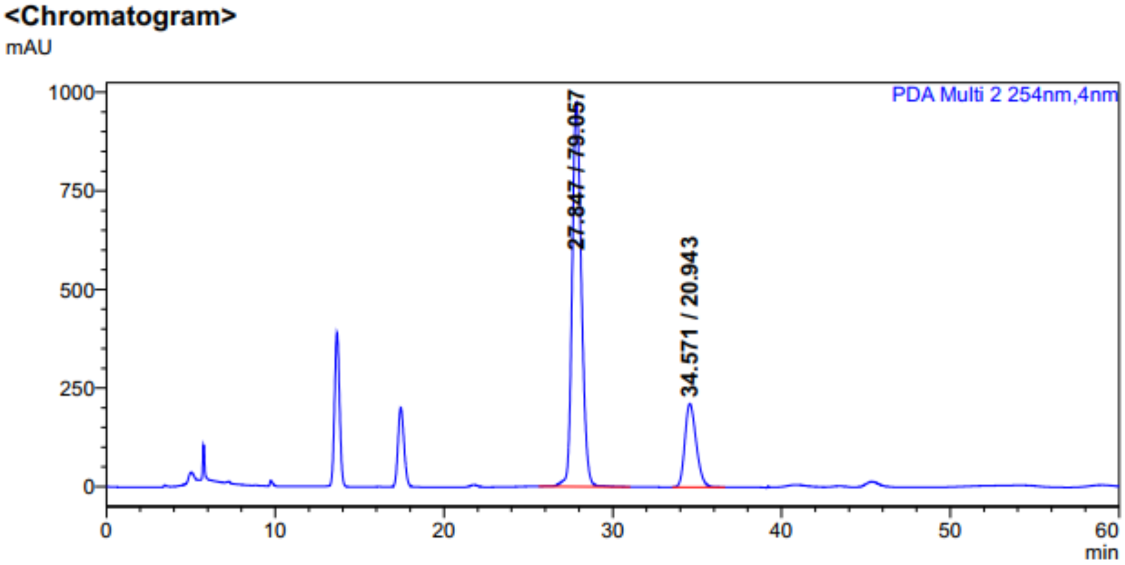

PDA Ch2 254nm

Peak#	Name	Ret. Time	Area	Area%
1		27.847	37221184	79.057
2		34.571	9860353	20.943
Total			47081537	100.000

16. NMR for Boroxines

2,4-Me-phenylboroxine (400 MHz, DMSO)

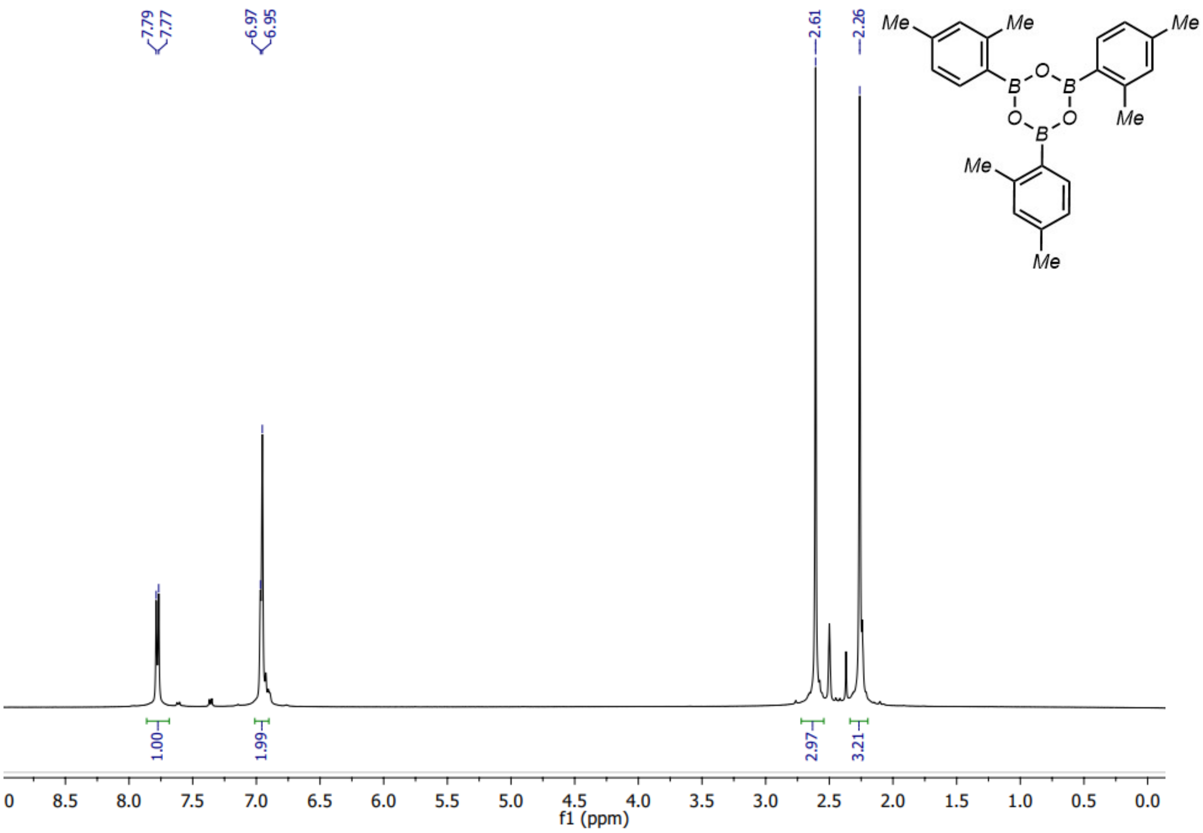

2-naphtyl-phenylboroxine (400 MHz, DMSO)

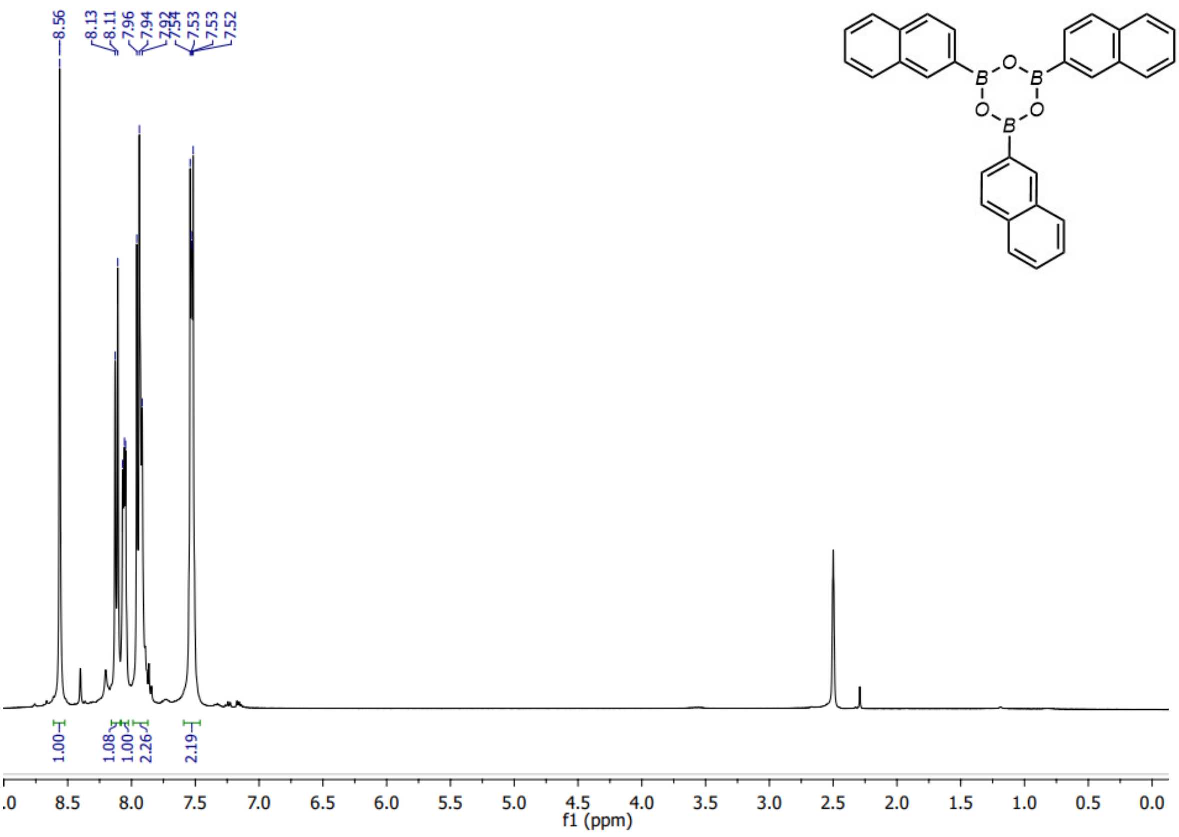

3,5-OMe-phenylboroxine (400 MHz, DMSO)

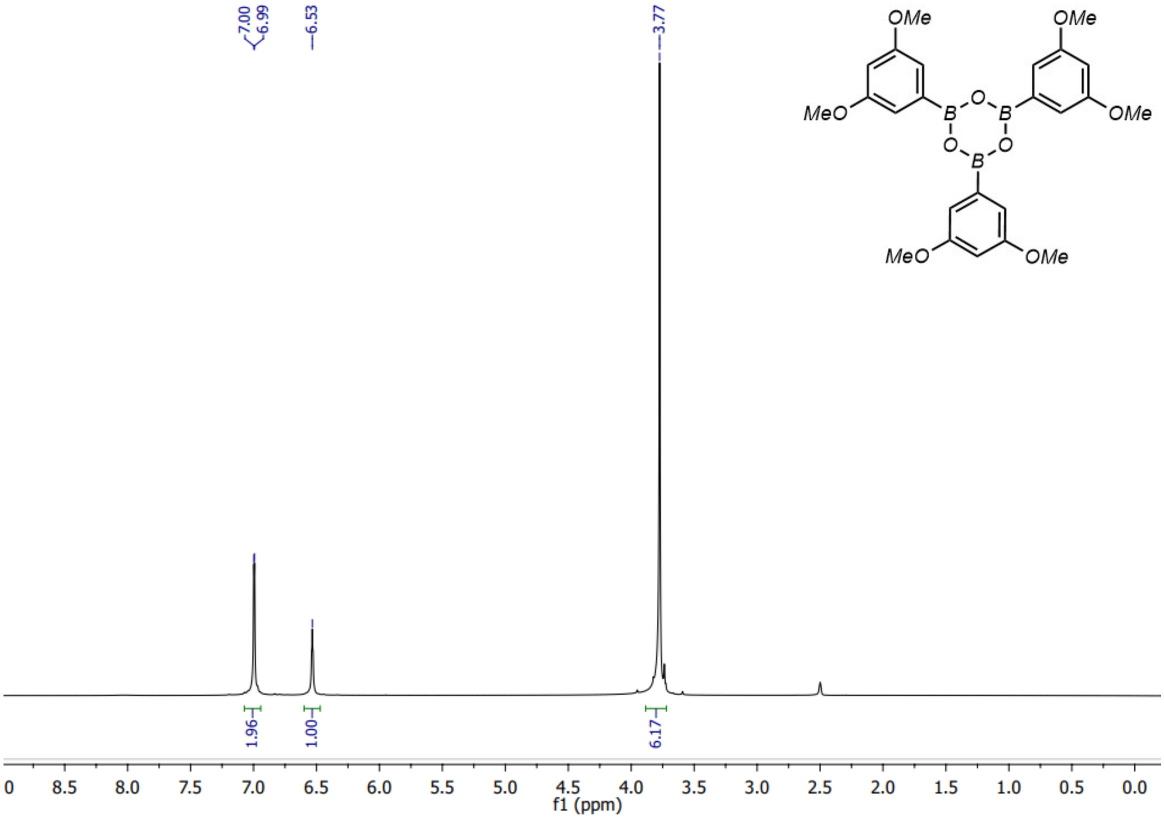

3,5-F-phenylboroxine (400 MHz, DMSO)

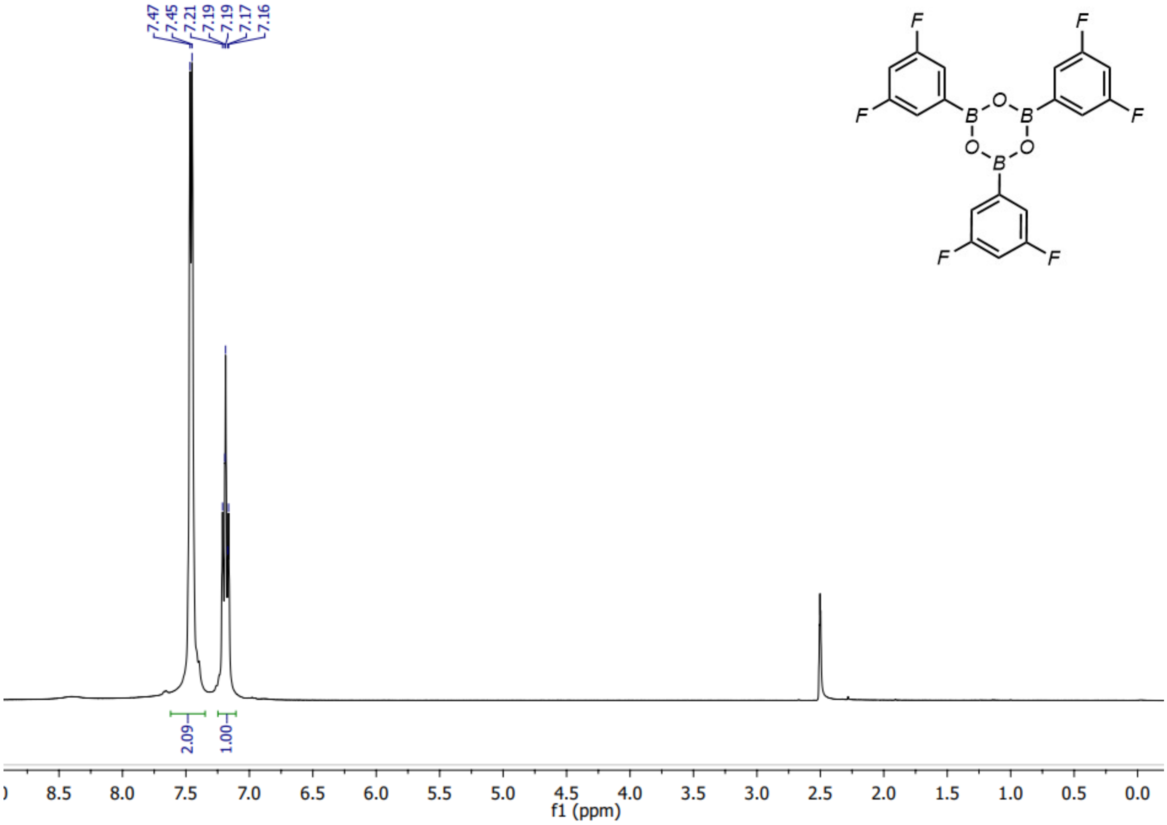

4-Me-phenylboroxine (400 MHz, DMSO)

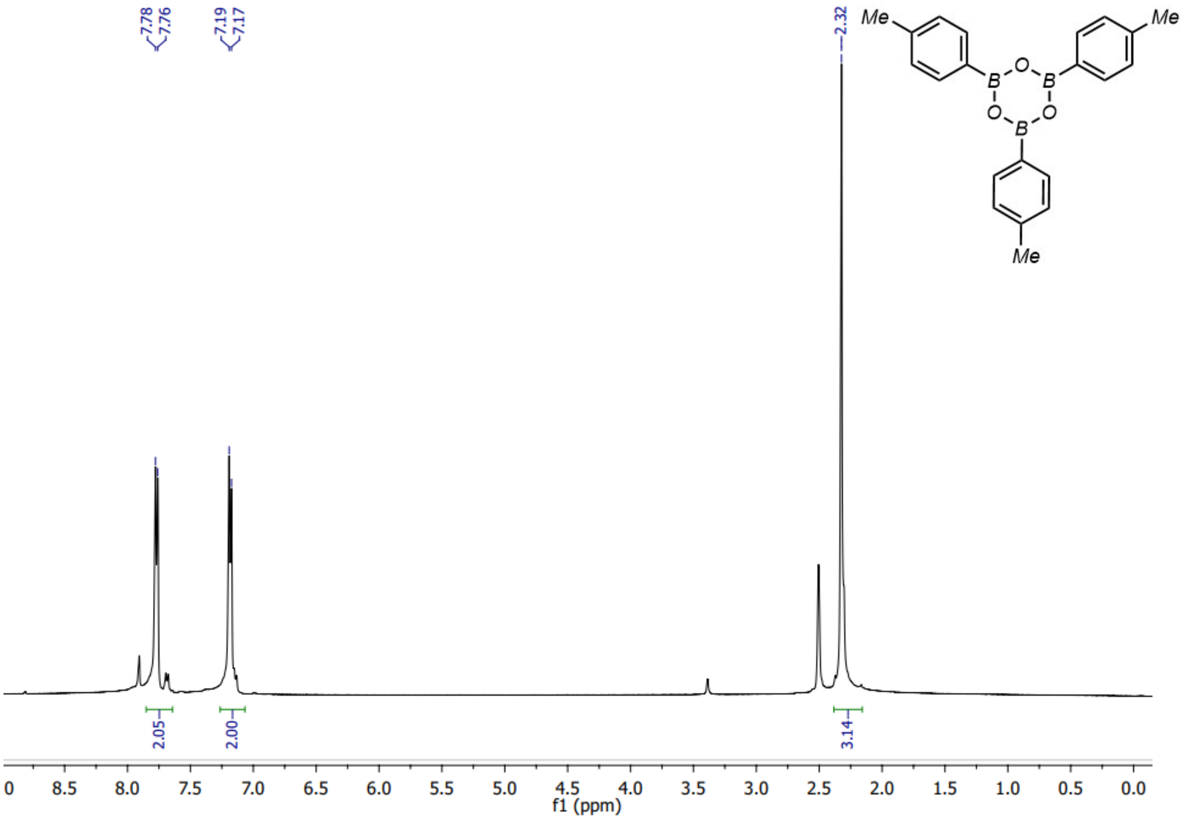

4-OMe-phenylboroxine (400 MHz, DMSO)

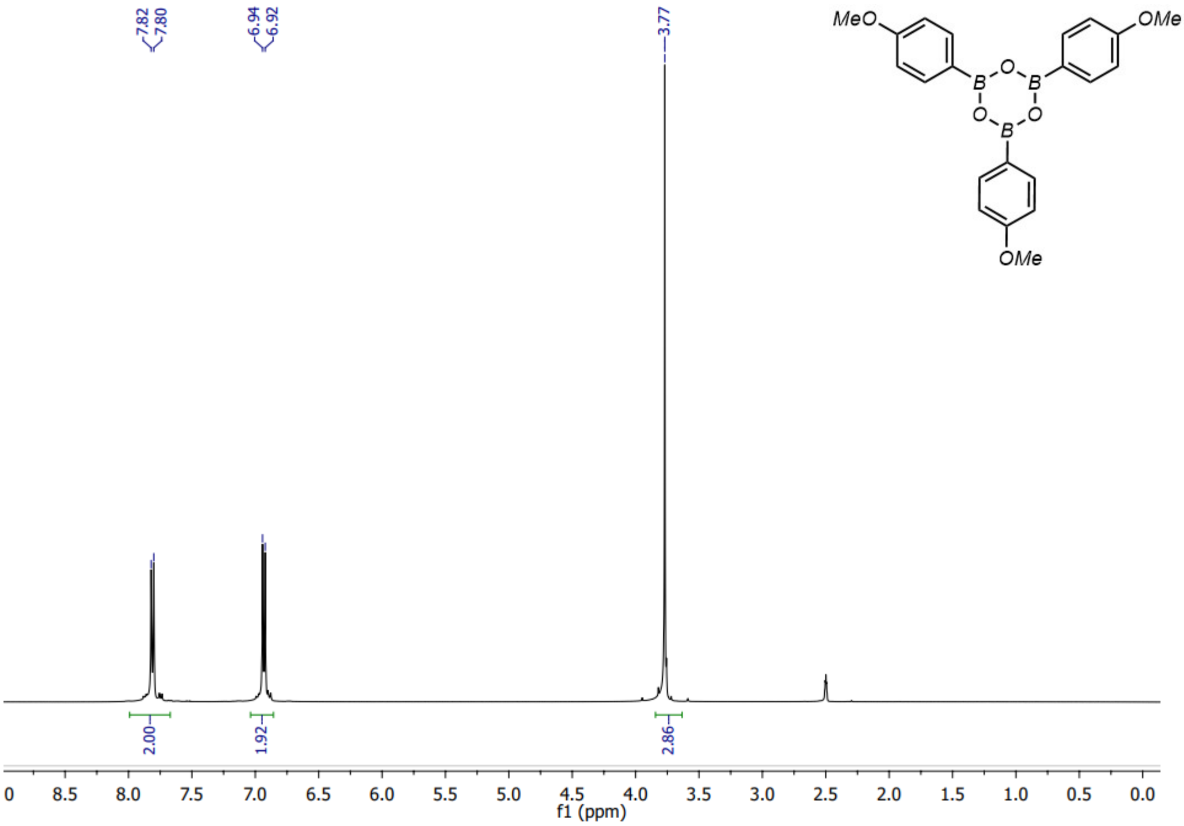

4-tBu-phenylboroxine (400 MHz, DMSO)

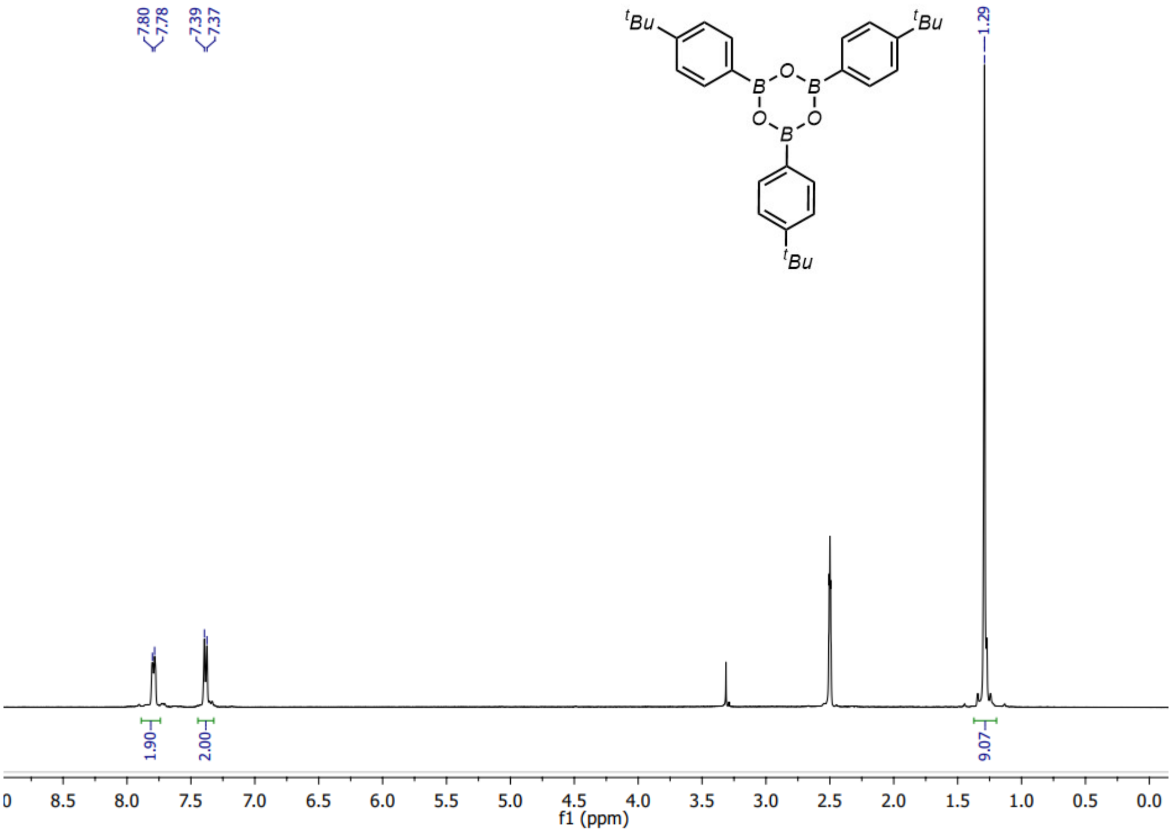

2-Me-phenylboroxine (400 MHz, DMSO)

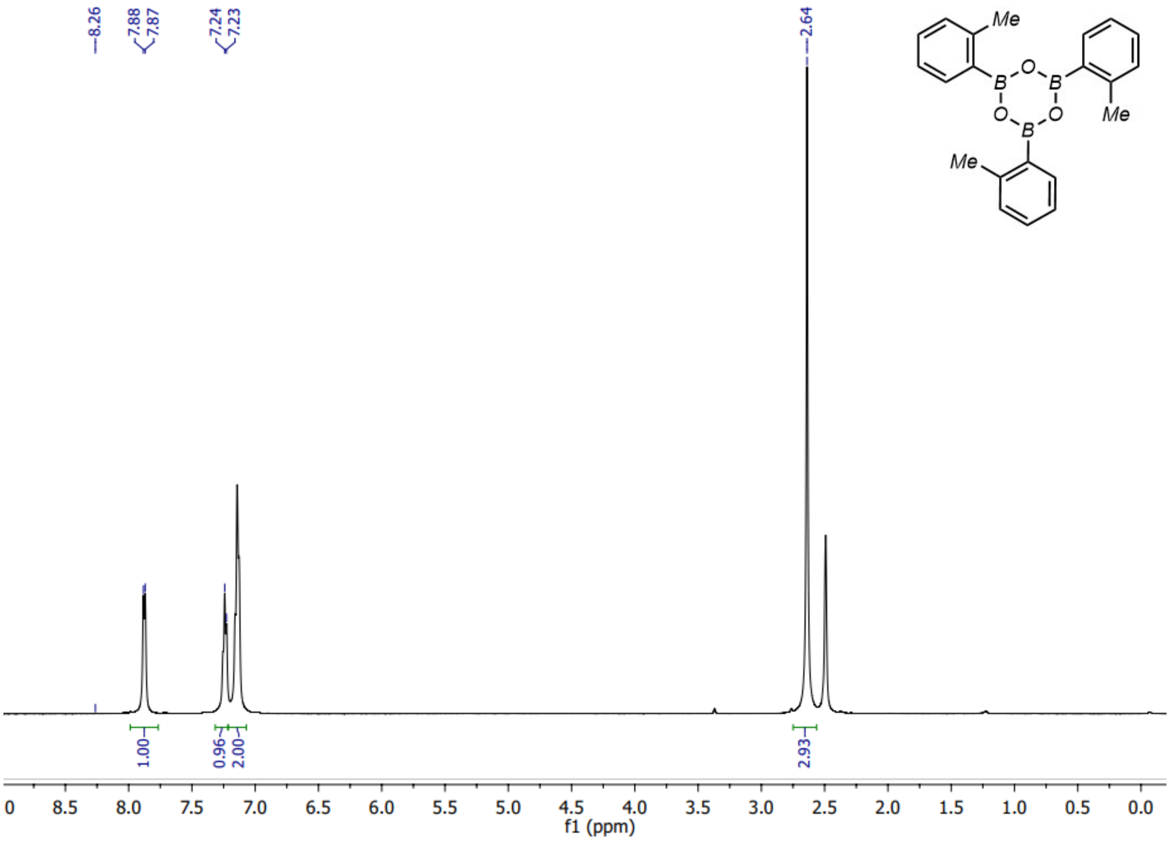

2-F-phenylboroxine (400 MHz, DMSO)

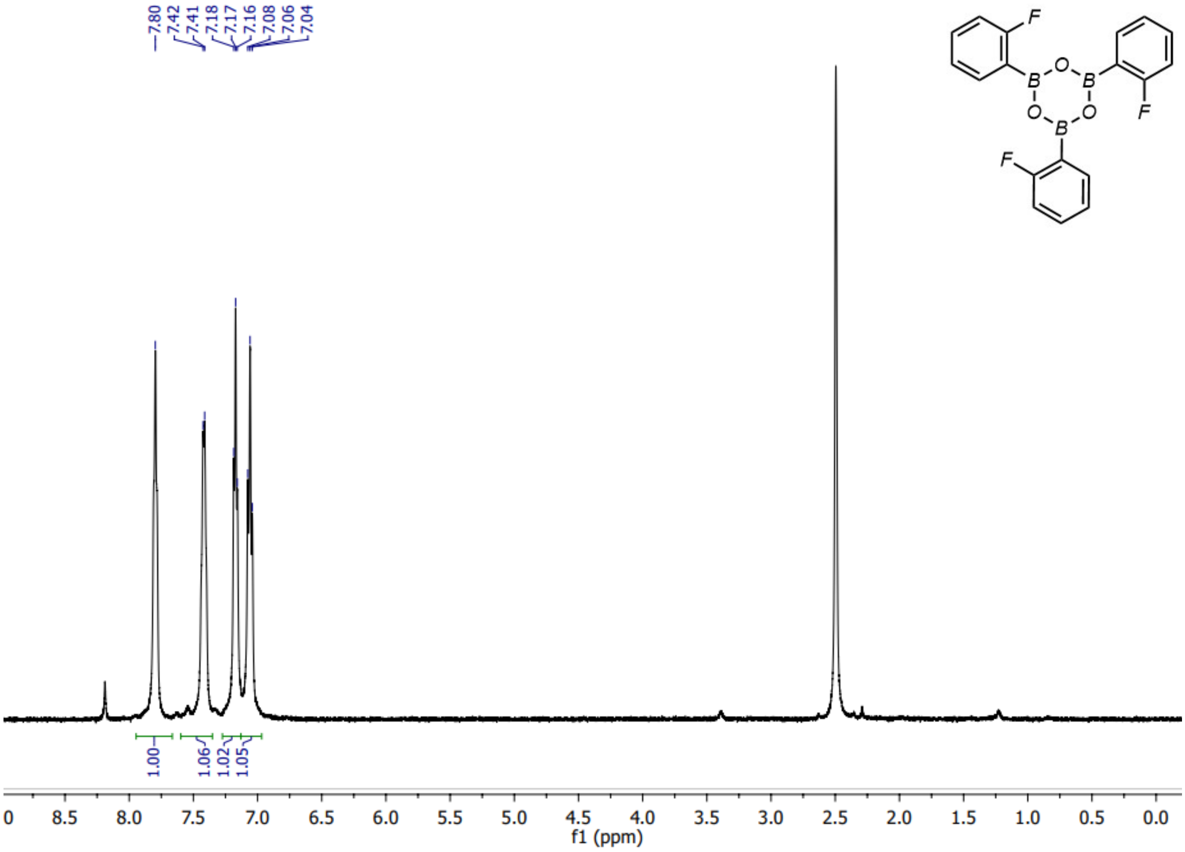

3-CF3-phenylboroxine (400 MHz, DMSO)

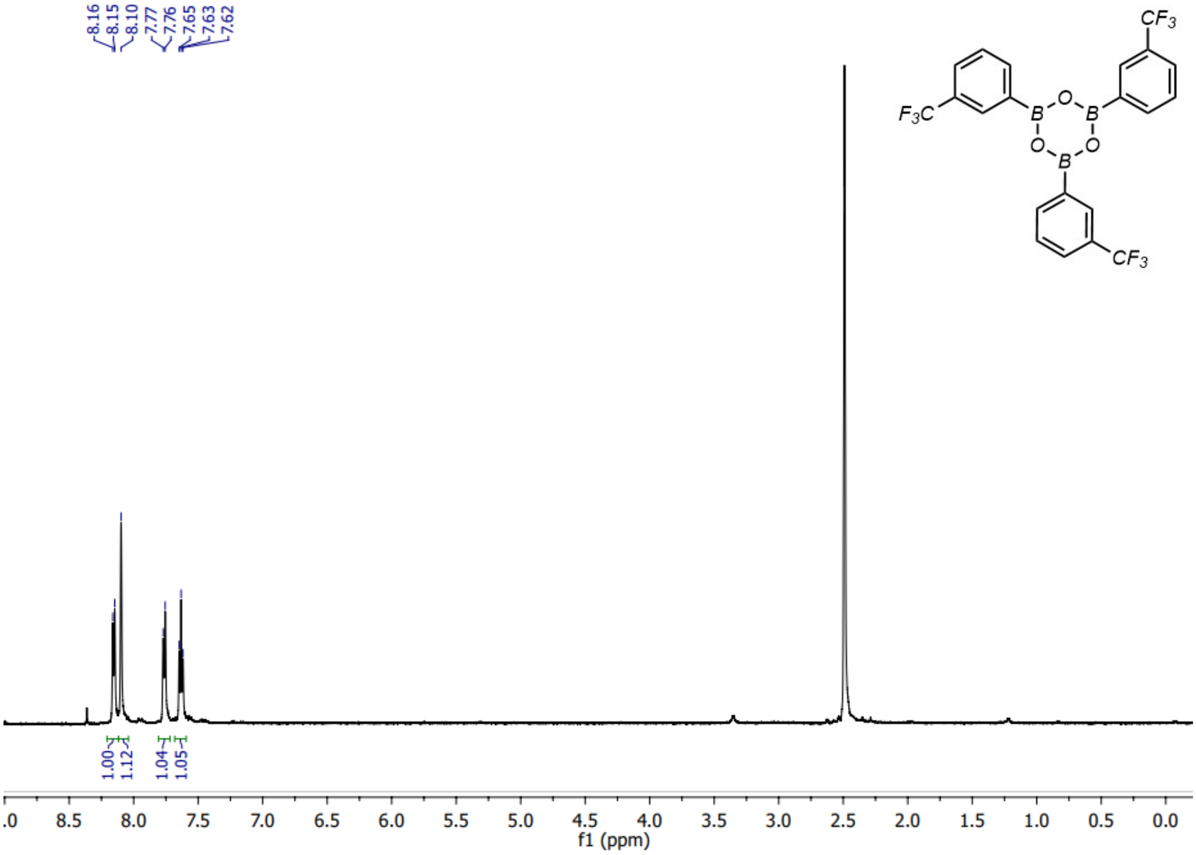

3-F-phenylboroxine (400 MHz, DMSO)

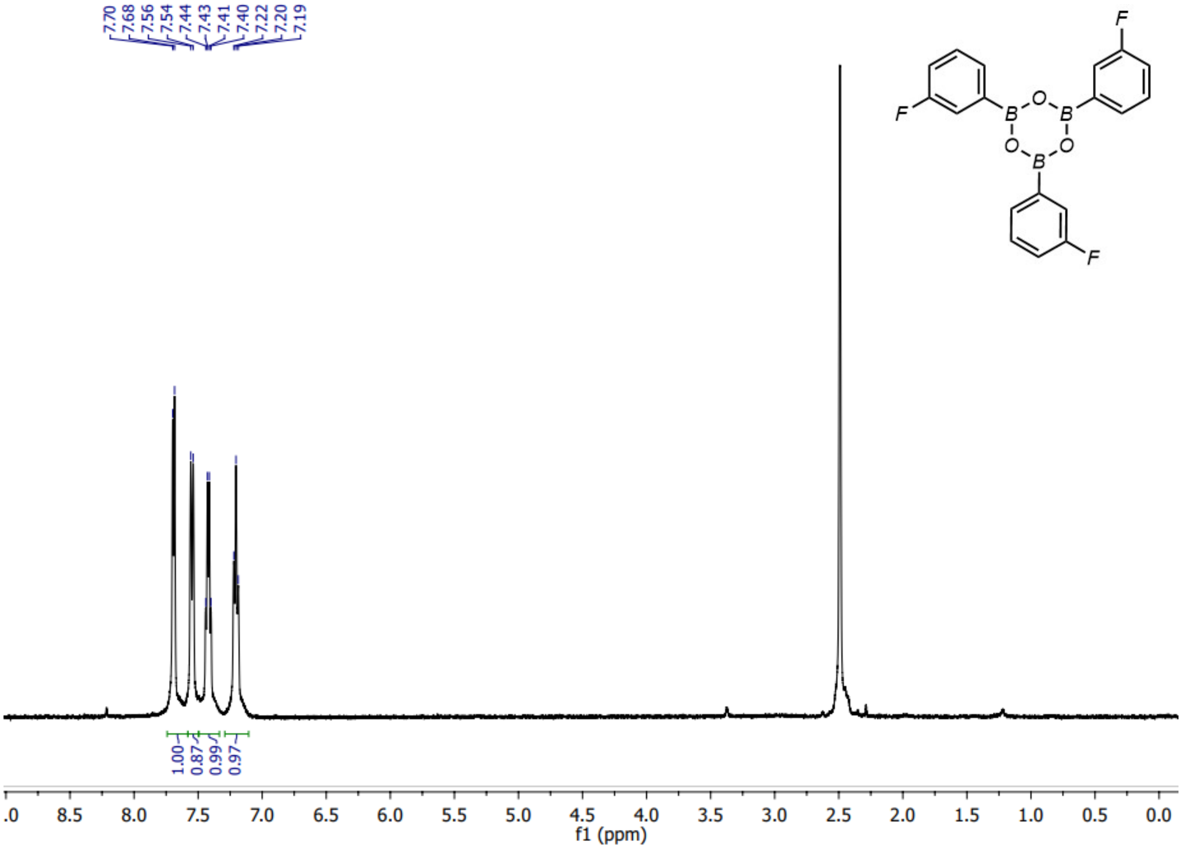

3-Me-phenylboroxine (400 MHz, DMSO)

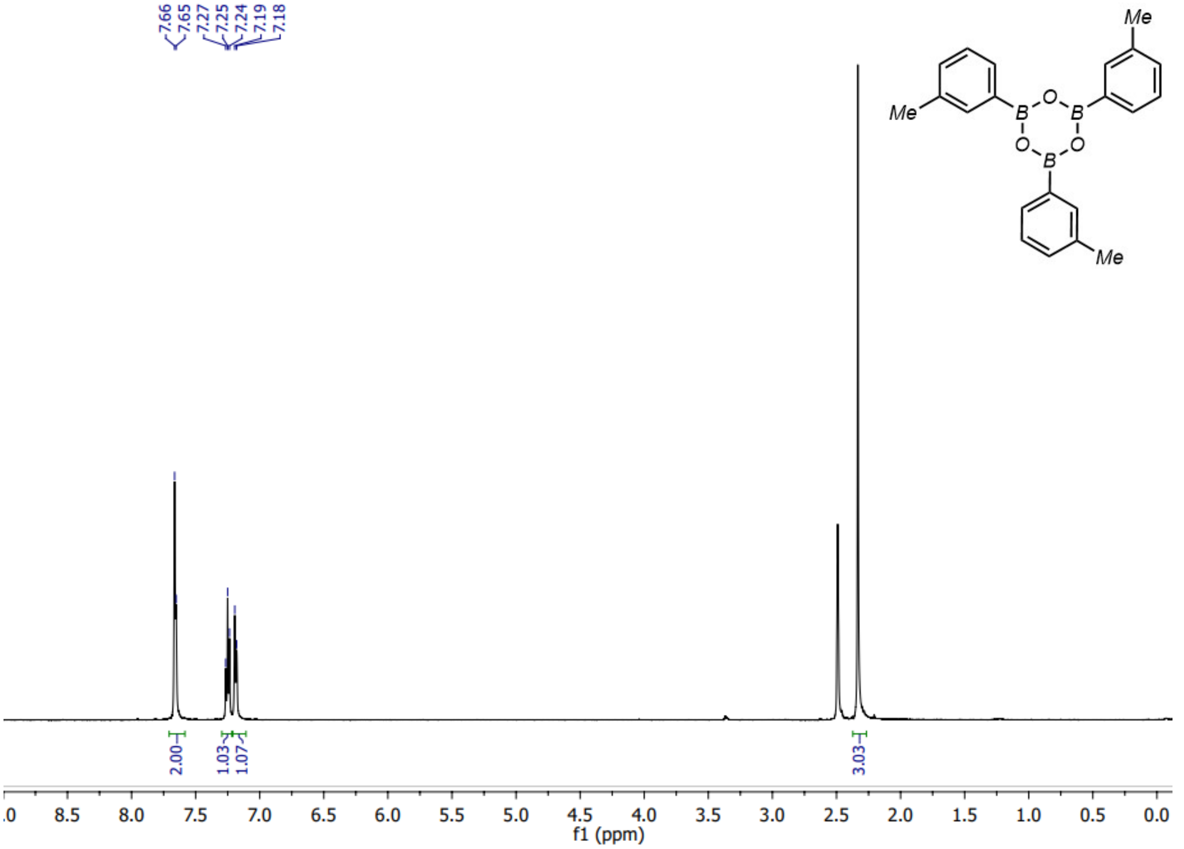

4-CF3-phenylboroxine (400 MHz, DMSO)

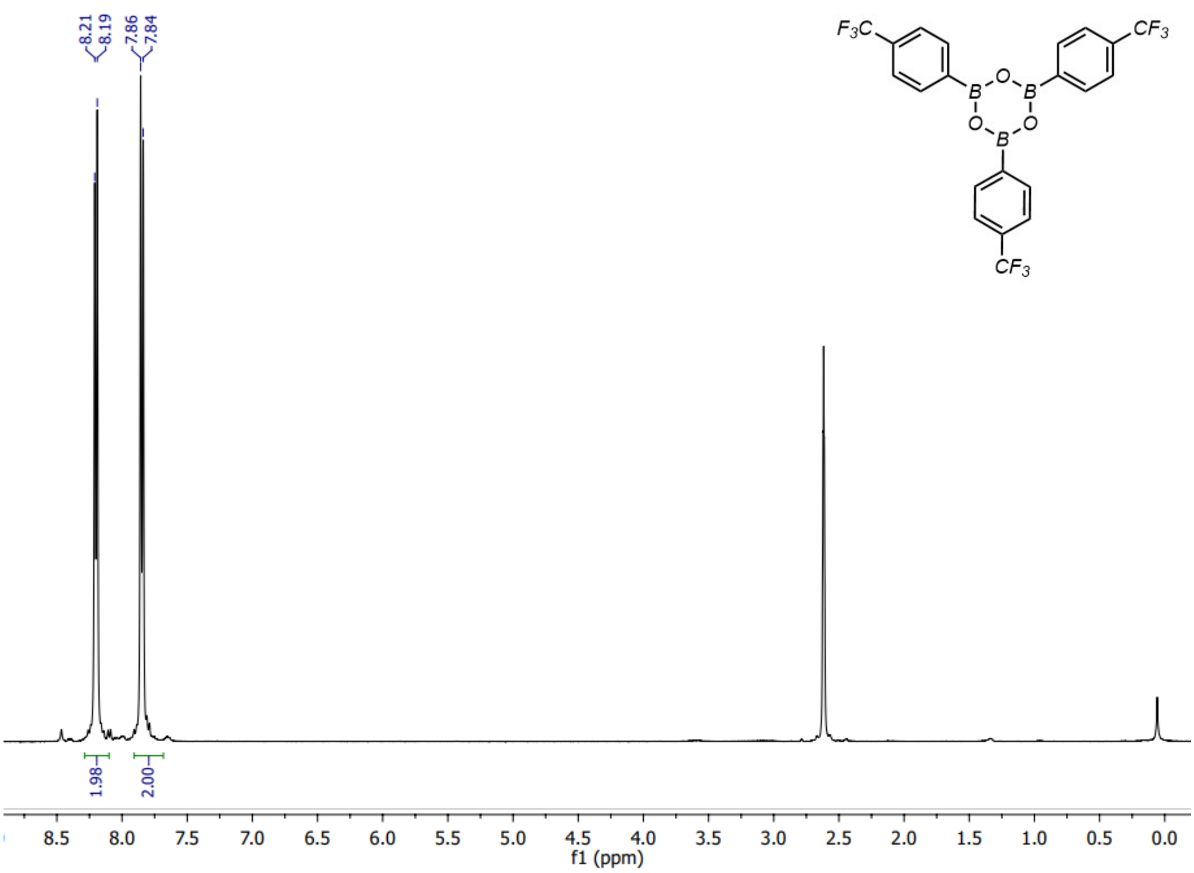

4-F-phenylboroxine (400 MHz, DMSO)

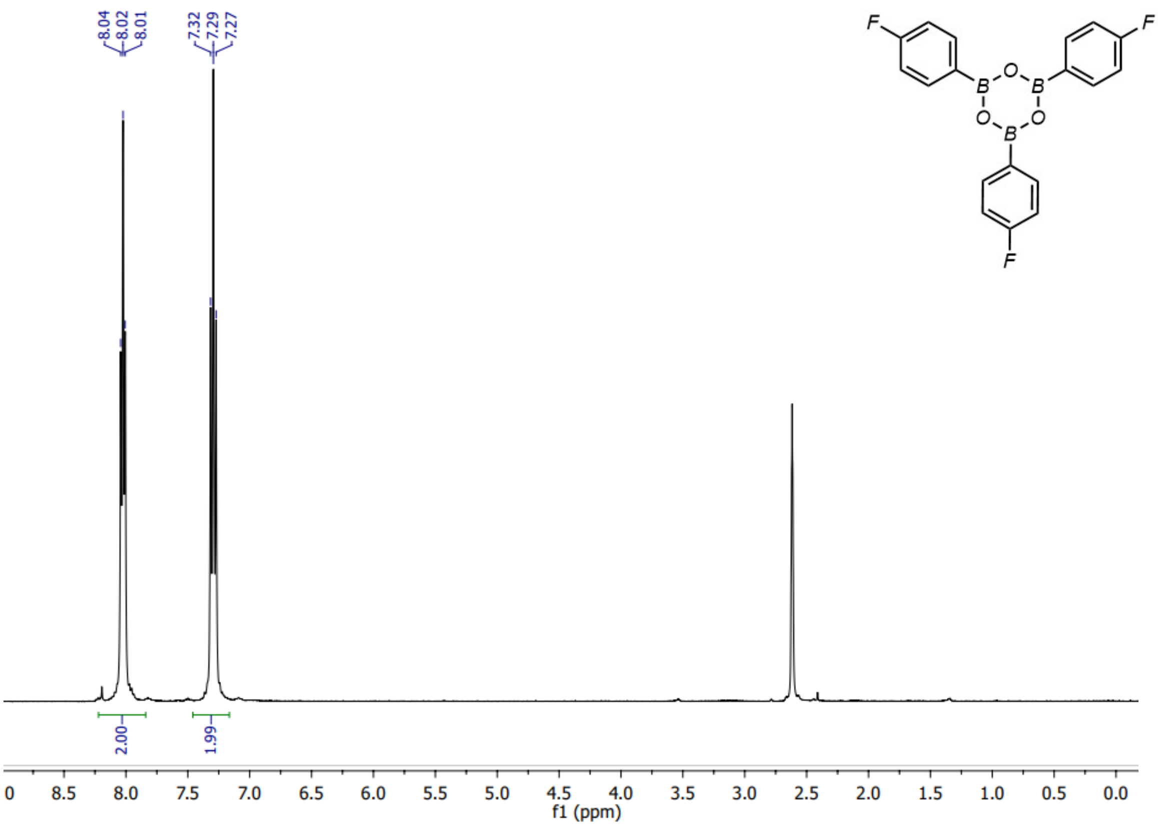

4-Me-phenylboroxine (400 MHz, DMSO)

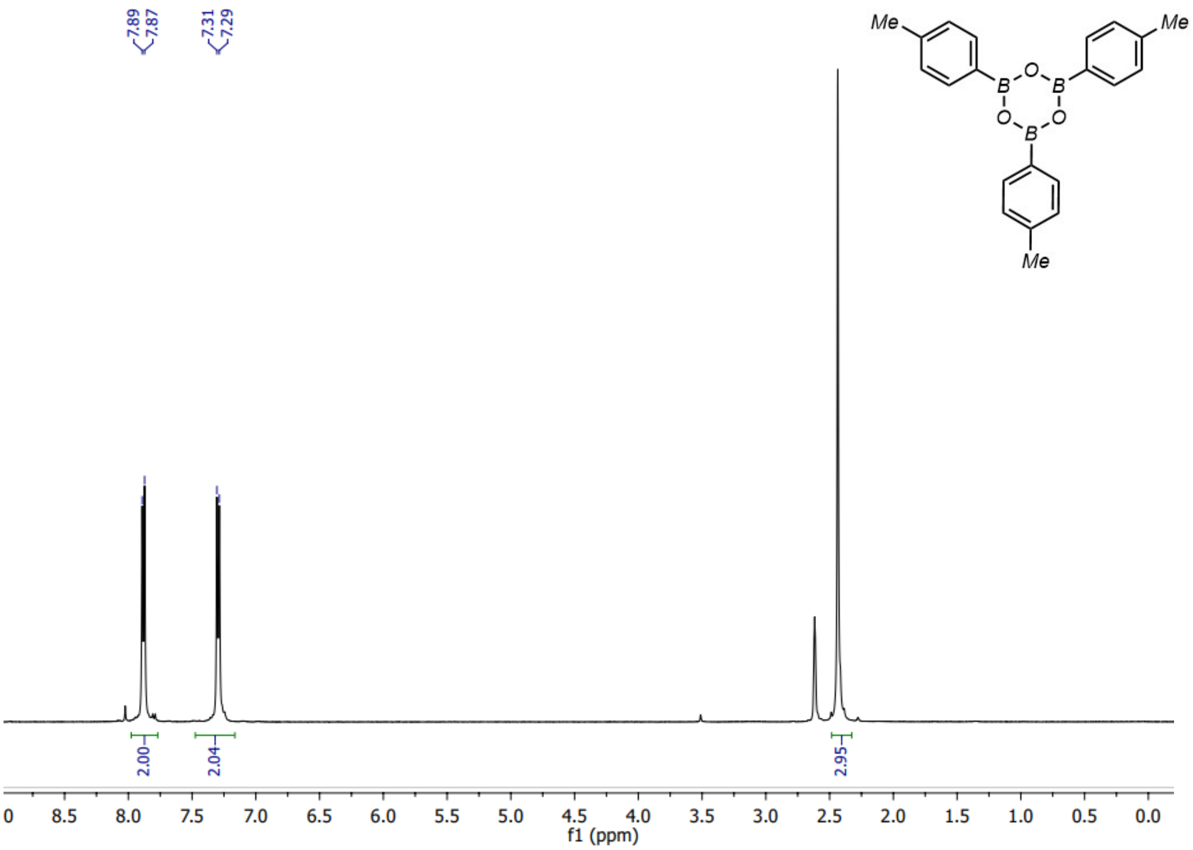

Phenylboroxine (400 MHz, DMSO)

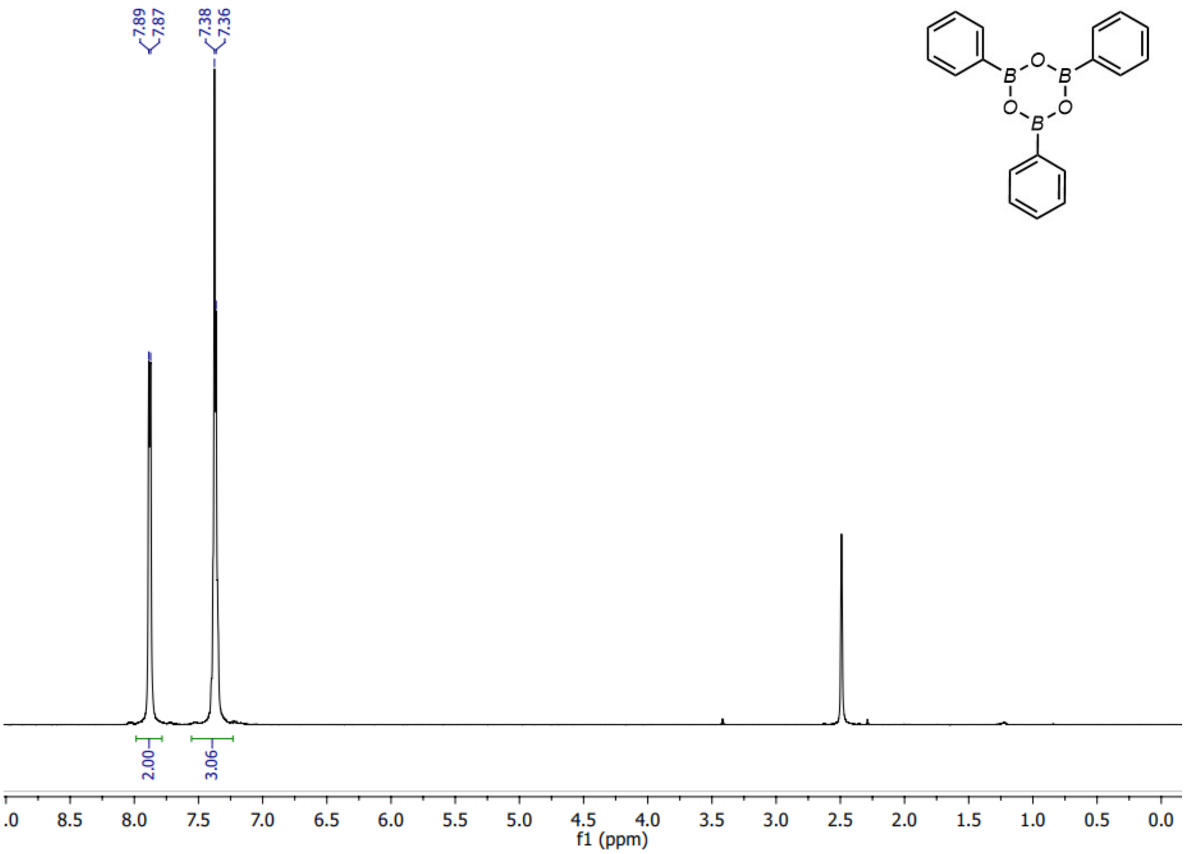

17. NMR of isolated (S)-2-((R)-hydroxy(4-nitrophenyl)methyl)cyclopentan-1-one

1H NMR of (S)-2-((R)-hydroxy(4-nitrophenyl)methyl)cyclopentan-1-one (400 MHz, $CDCl_3$**)**

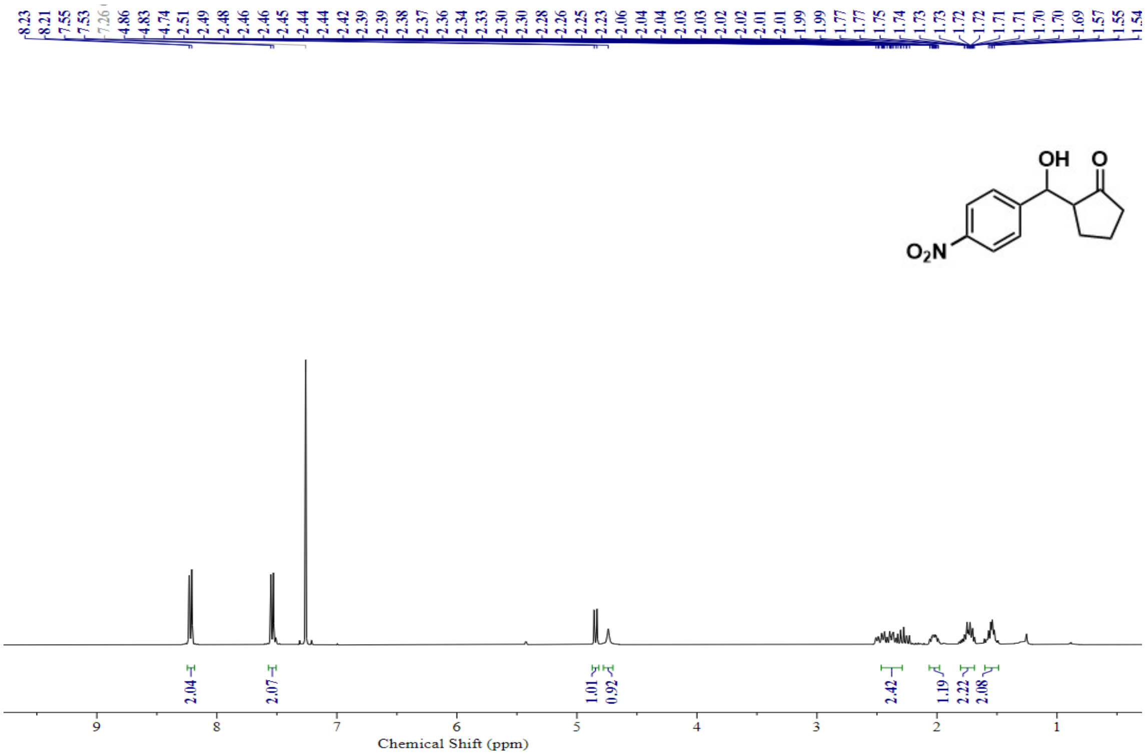

13C{1H} NMR of (S)-2-((R)-hydroxy(4-nitrophenyl)methyl)cyclopentan-1-one (100 MHz, $CDCl_3$**)**

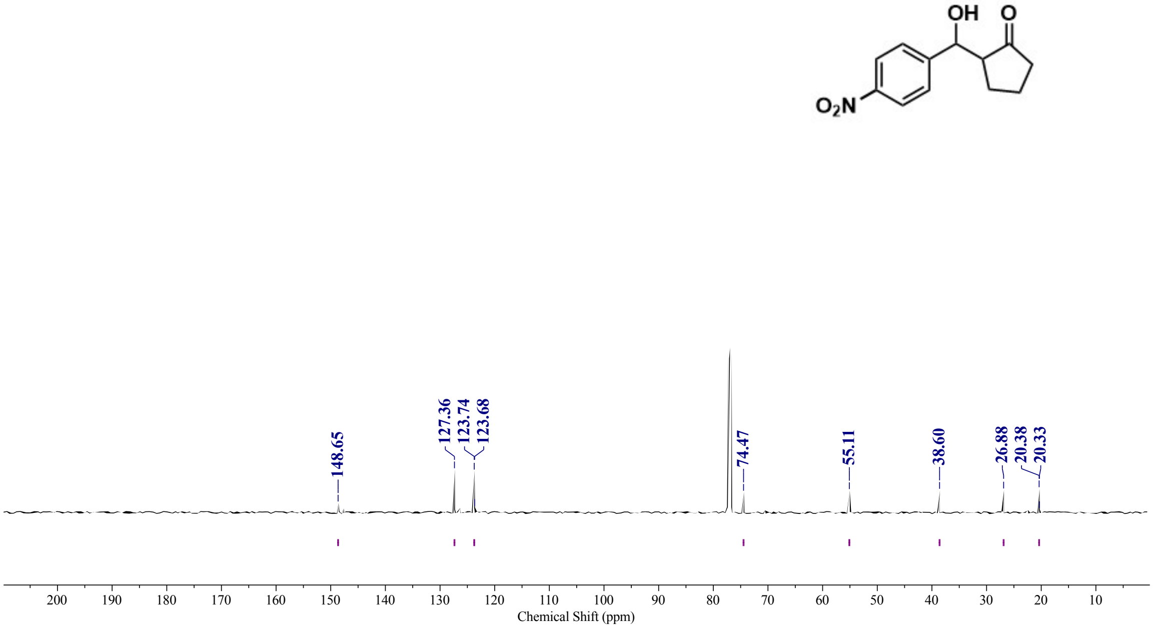

18. NMR traces for diastereoselectivity determination

All NMR samples of crudes were prepared as stated in GP1 and GP2: "after the reactions were finished, solvent was removed under reduced pressure and the reaction crude was then dissolved in $CDCl_3$ and directly analyzed via 1H NMR for yield and diastereoselectivity determination."

All diastereomeric ratios were determined by comparing the integrated area of syn and anti product peaks in 1H-NMR.

The NMRs in section 18 are of the crude reaction mixture and are presented with the aim of showing the reported diastereoselectivity. We have added a zoom on the peak area of the syn (5.30-5.45) and anti (4.75 - 4.85) products. The isolated NMR with a full peak assignment appears in the previous page (section 17).

18.1. NMR Traces for Table S1. Boronic acid Screening and blanks in acetonitrile

NMR traces for reaction with: **3-F-phenylboronic acid (400 MHz,** $CDCl_3$**)**

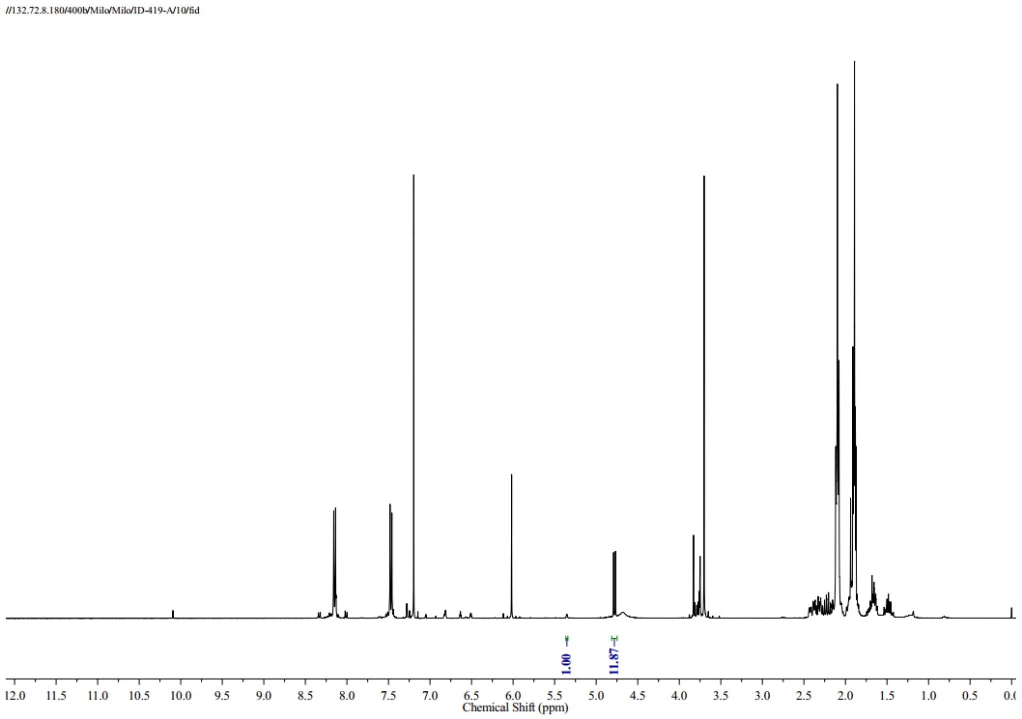

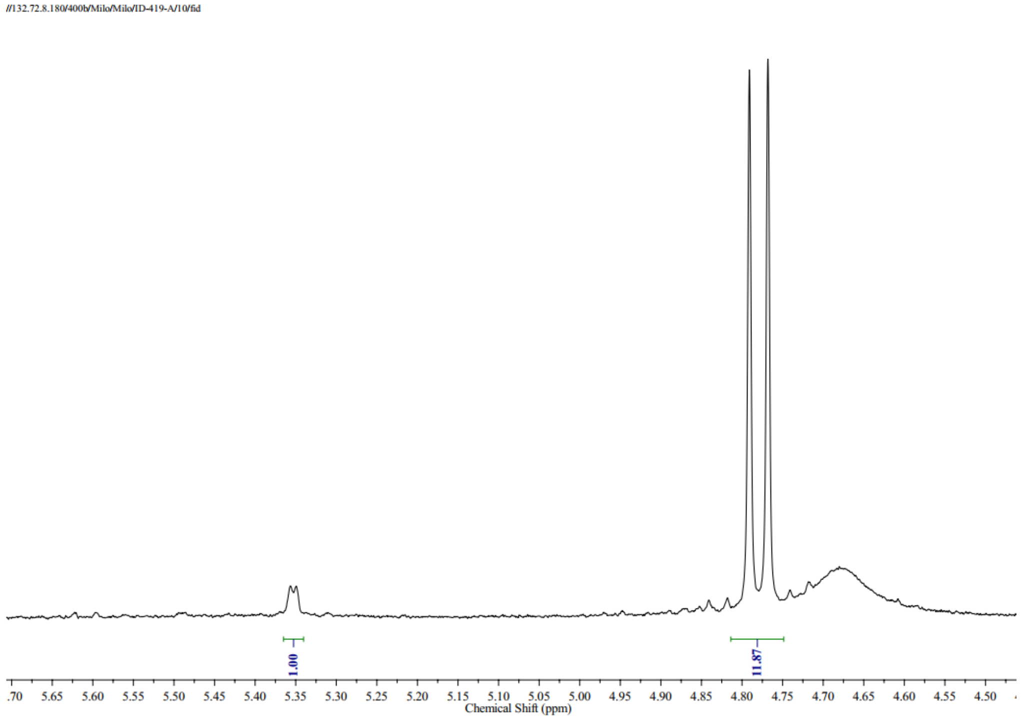

NMR traces for reaction with: **3-F-phenylboronic acid (duplicate) (400 MHz,** CDCl$_3$**)**

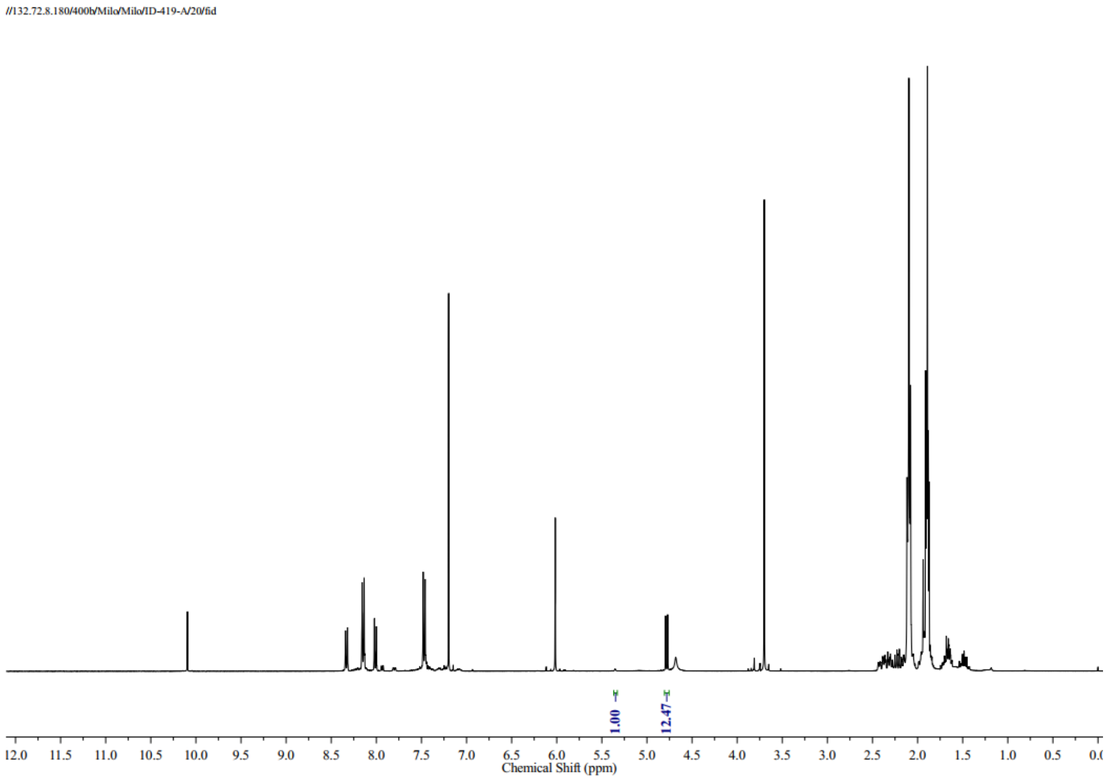

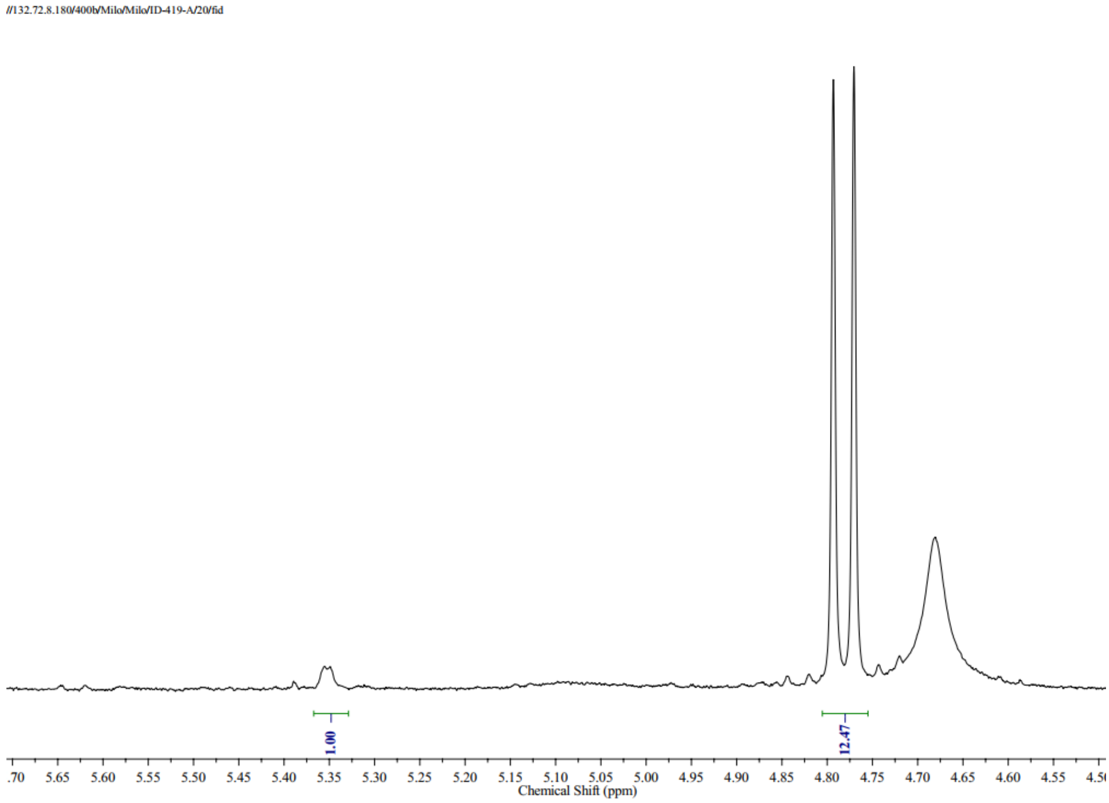

NMR traces for reaction with: **3,5-F-phenylboronic acid (400 MHz,** $CDCl_3$**)**

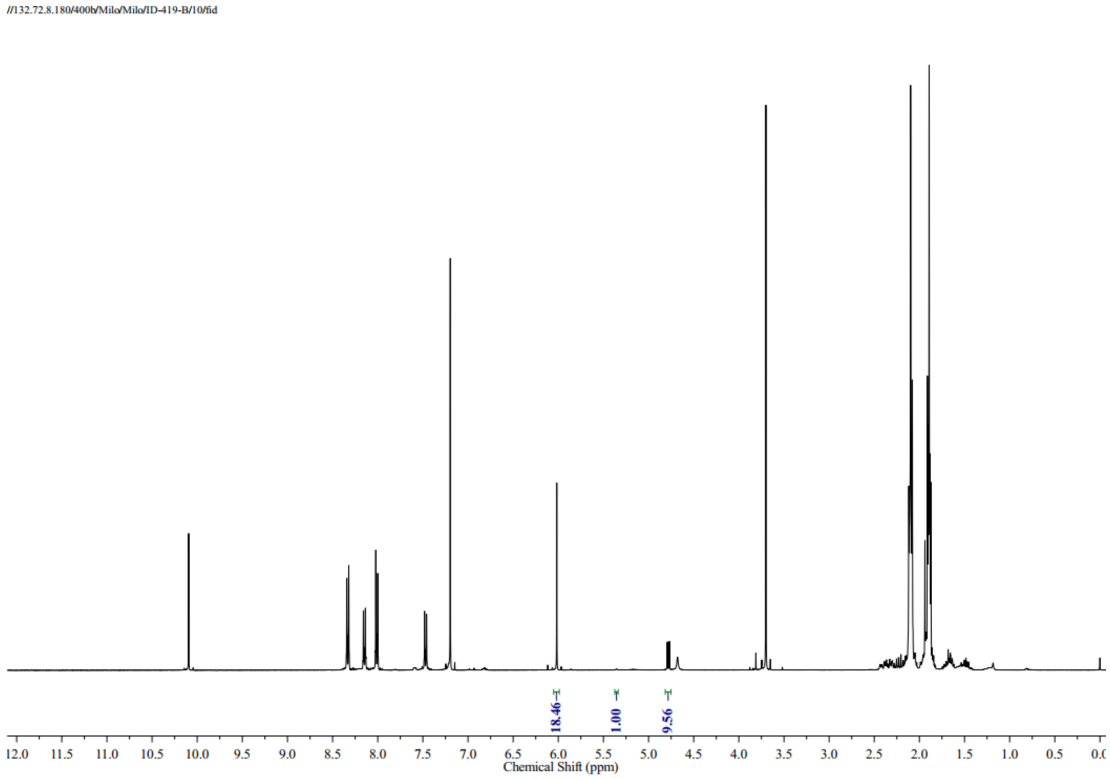

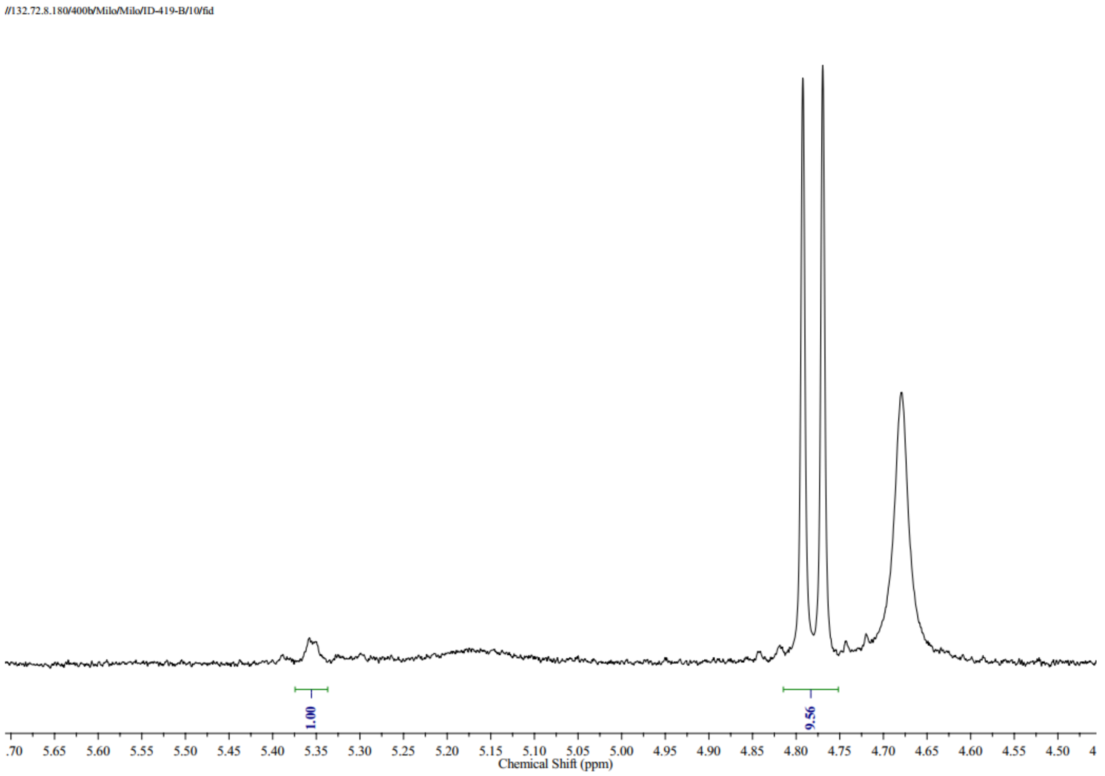

NMR traces for reaction with: **3,5-F-phenylboronic acid (duplicate) (400 MHz,** $CDCl_3$**)**

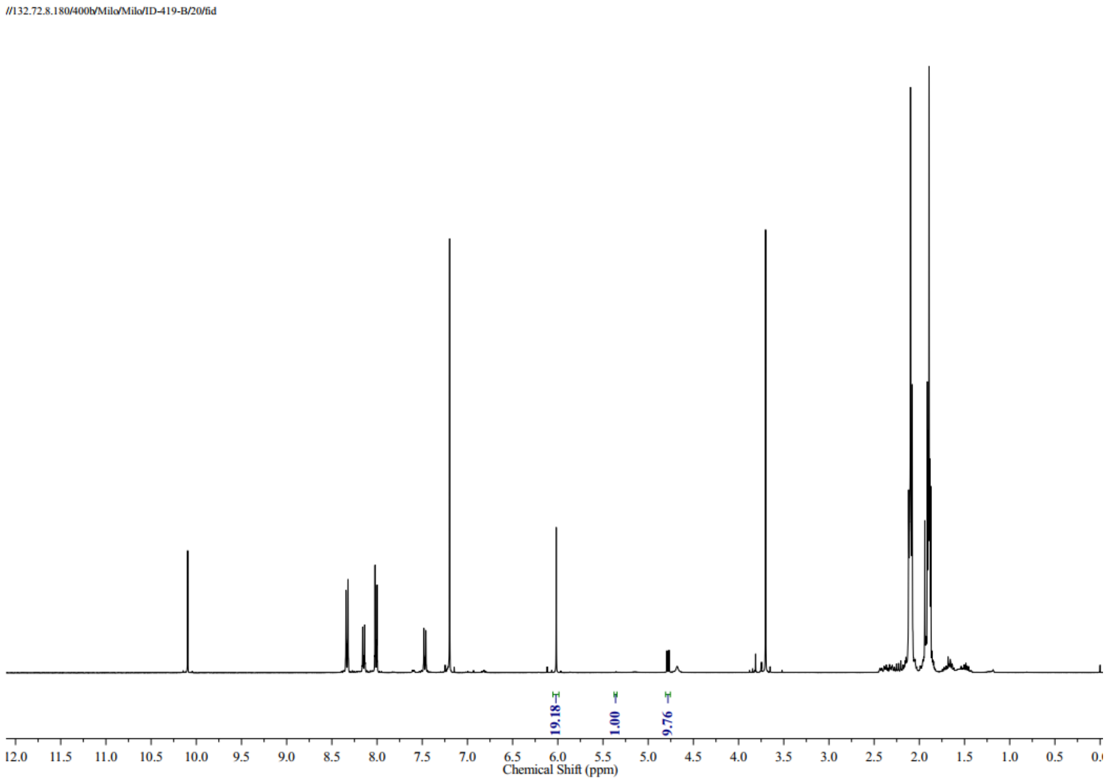

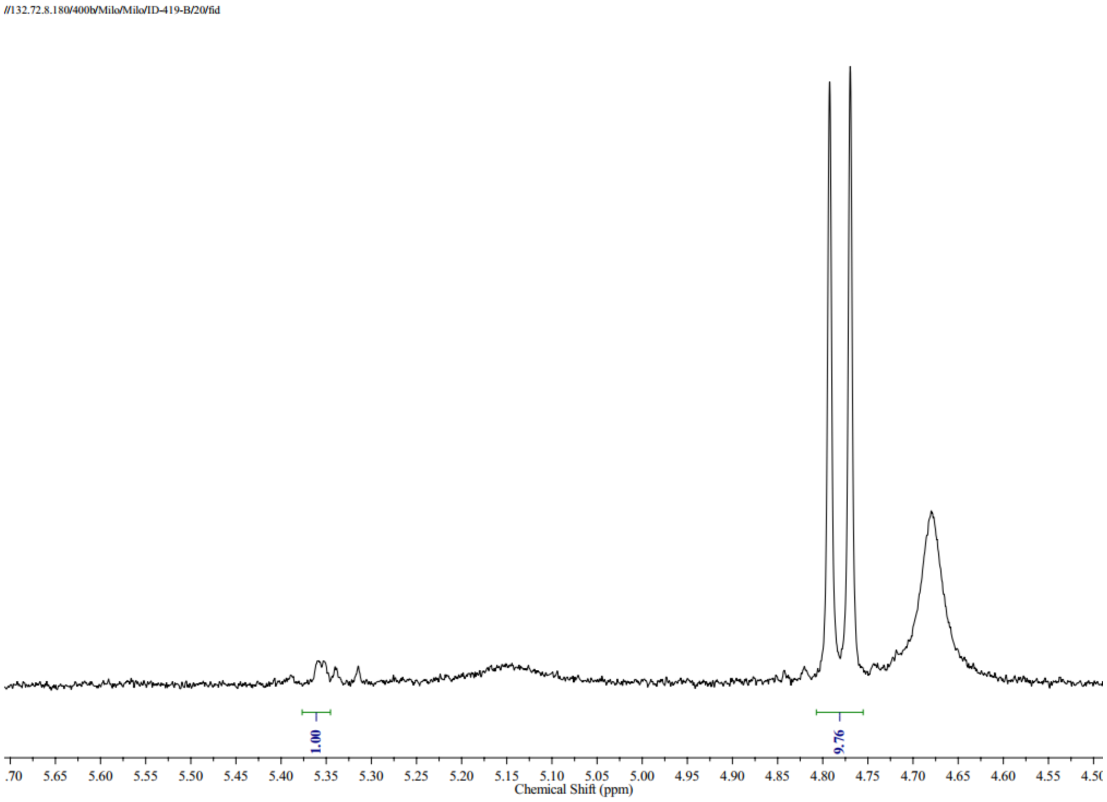

NMR traces for reaction with: **4-tBu-phenylboronic acid (400 MHz,** $CDCl_3$**)**

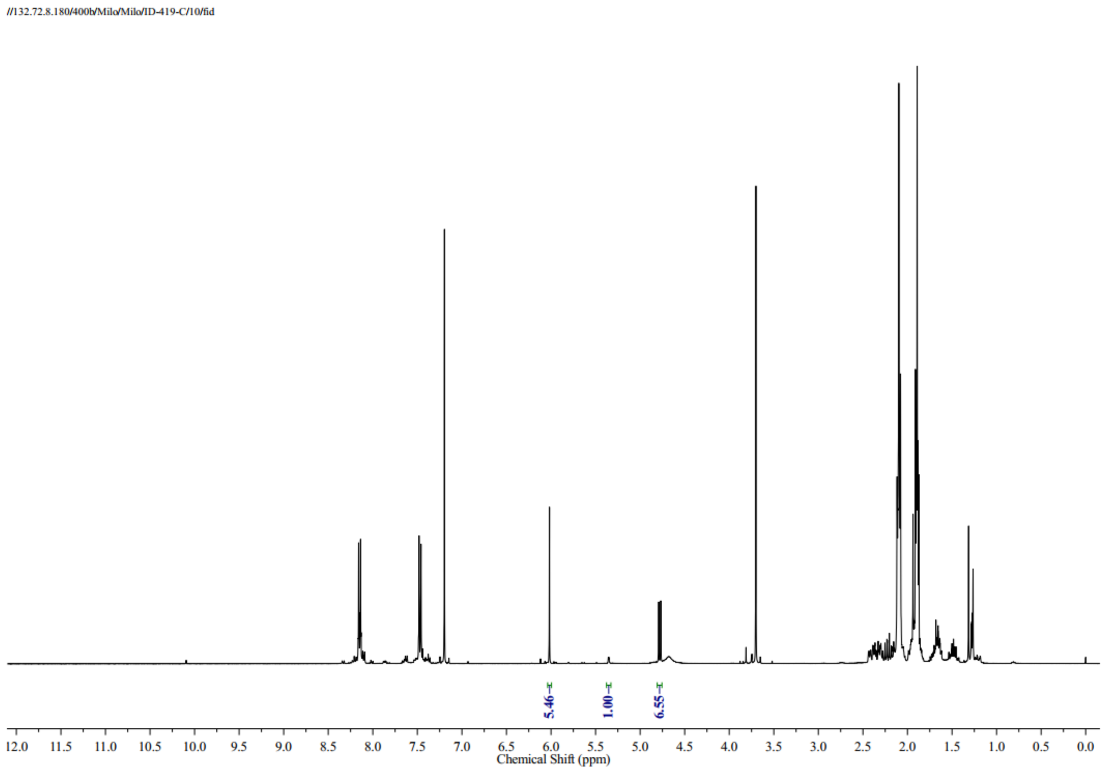

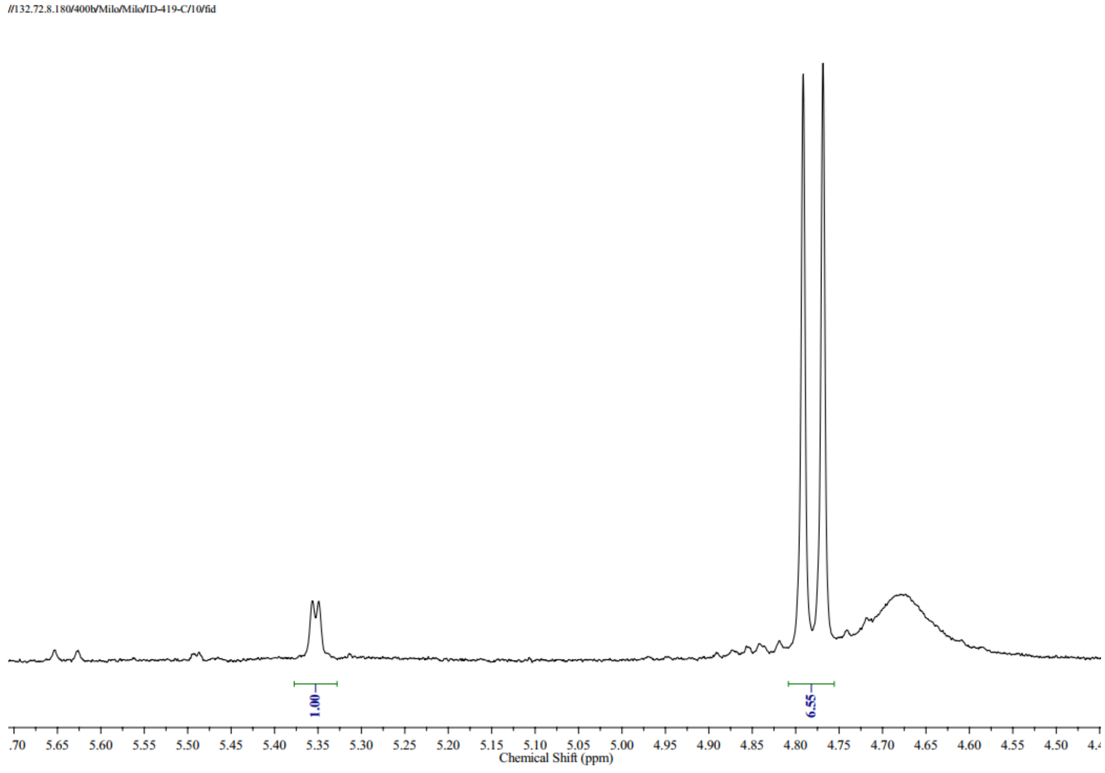

NMR traces for reaction with: **4-tBu-phenylboronic acid (duplicate) (400 MHz,** CDCl$_3$**)**

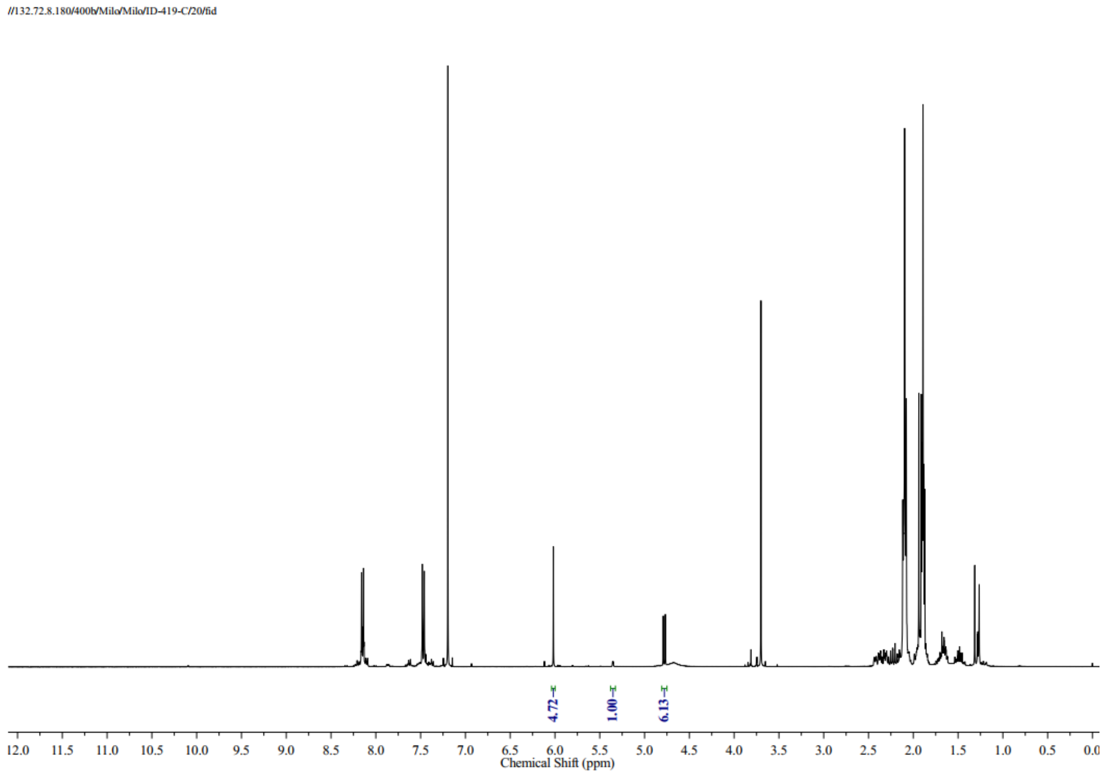

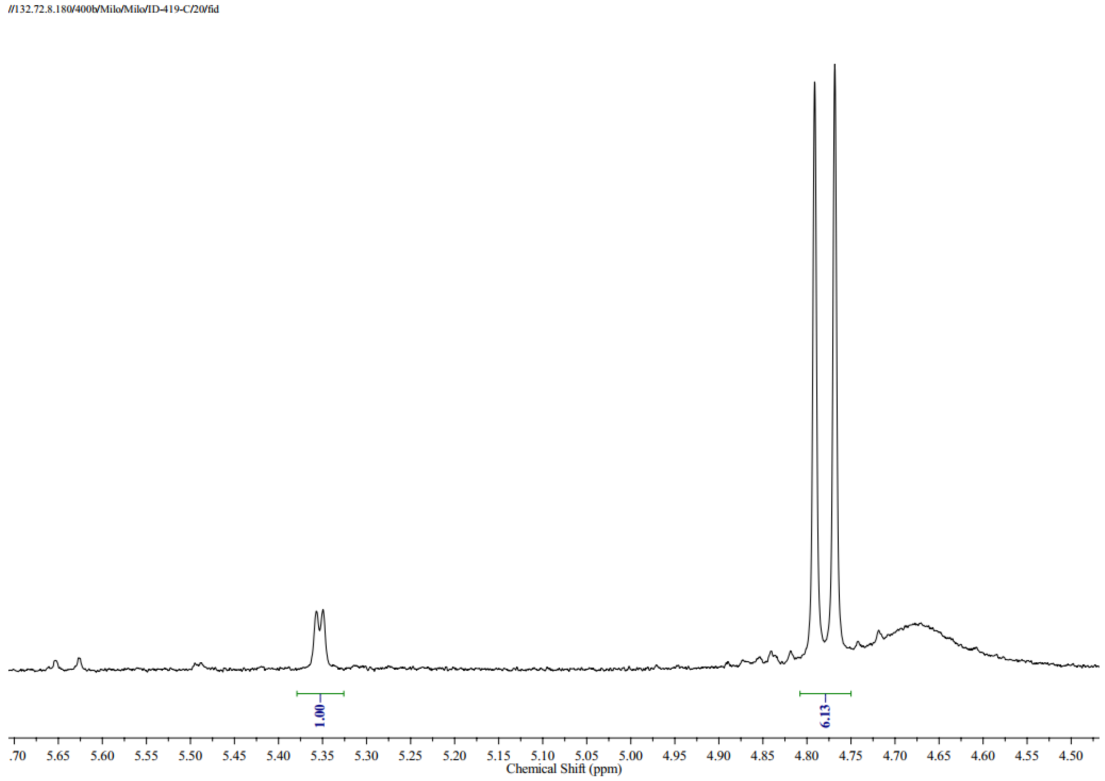

NMR traces for reaction with: **2,4-Me-phenylboronic acid (400 MHz,** $CDCl_3$**)**

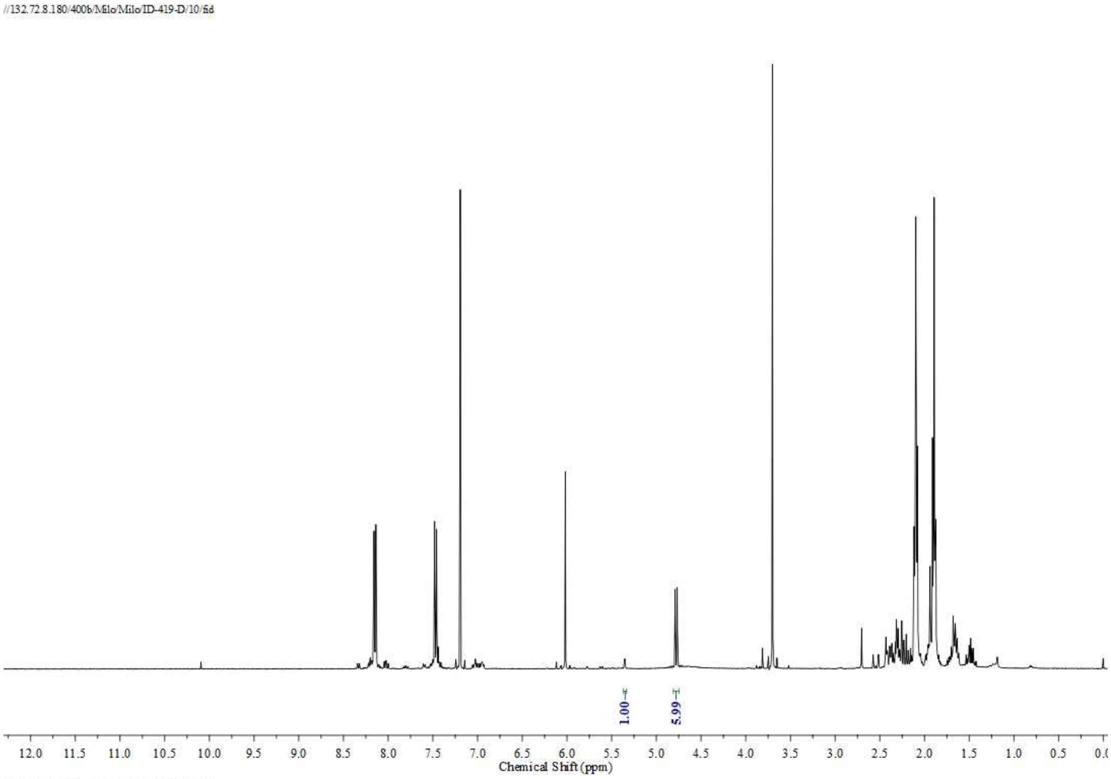

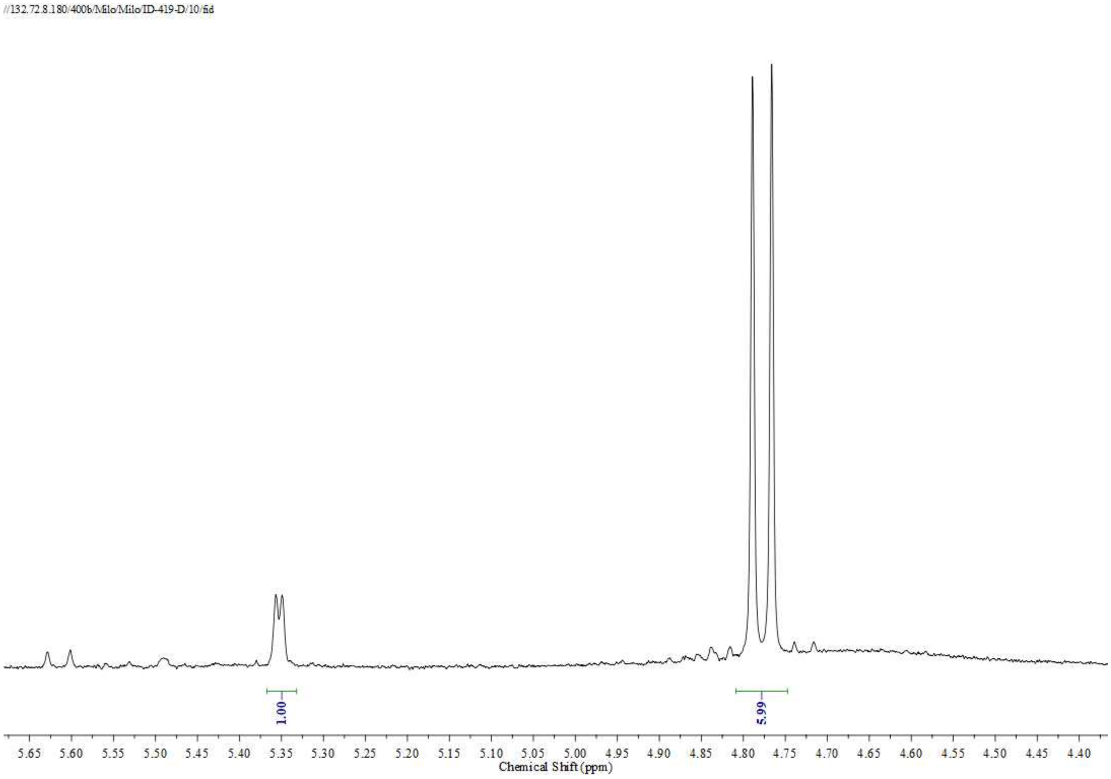

NMR traces for reaction with: **2,4-Me-phenylboronic acid (duplicate) (400 MHz,** $CDCl_3$**)**

//132.72.8.180/400b/Milo/Milo/ID-419-D/10/fid

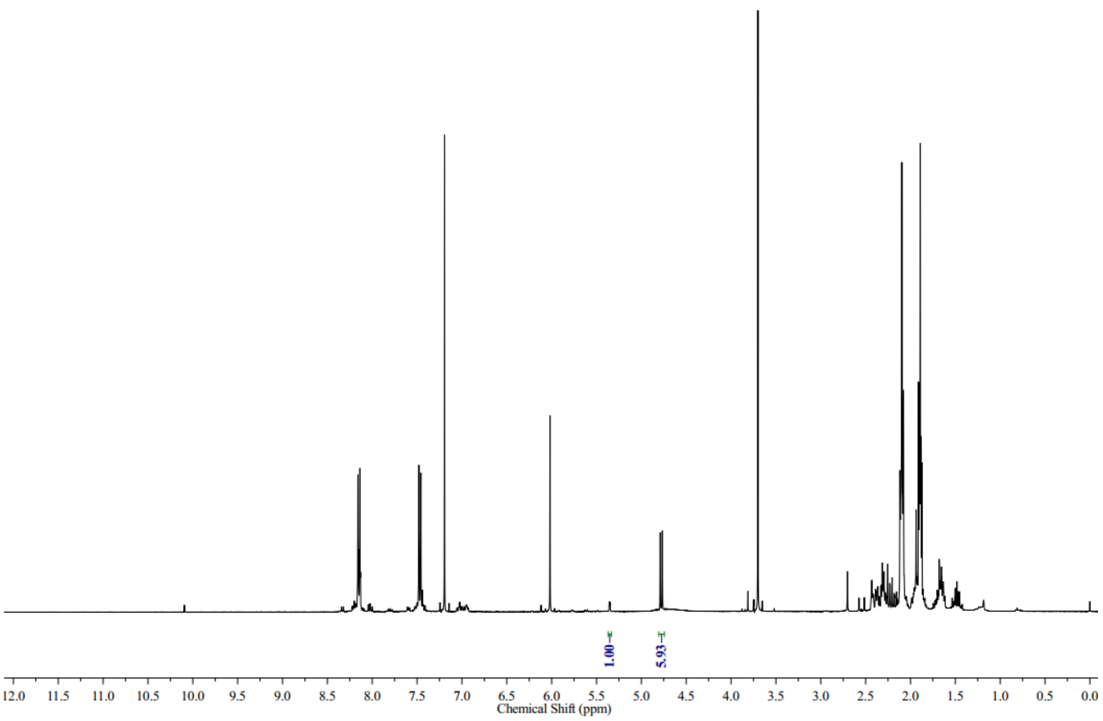

//132.72.8.180/400b/Milo/Milo/ID-419-D/10/fid

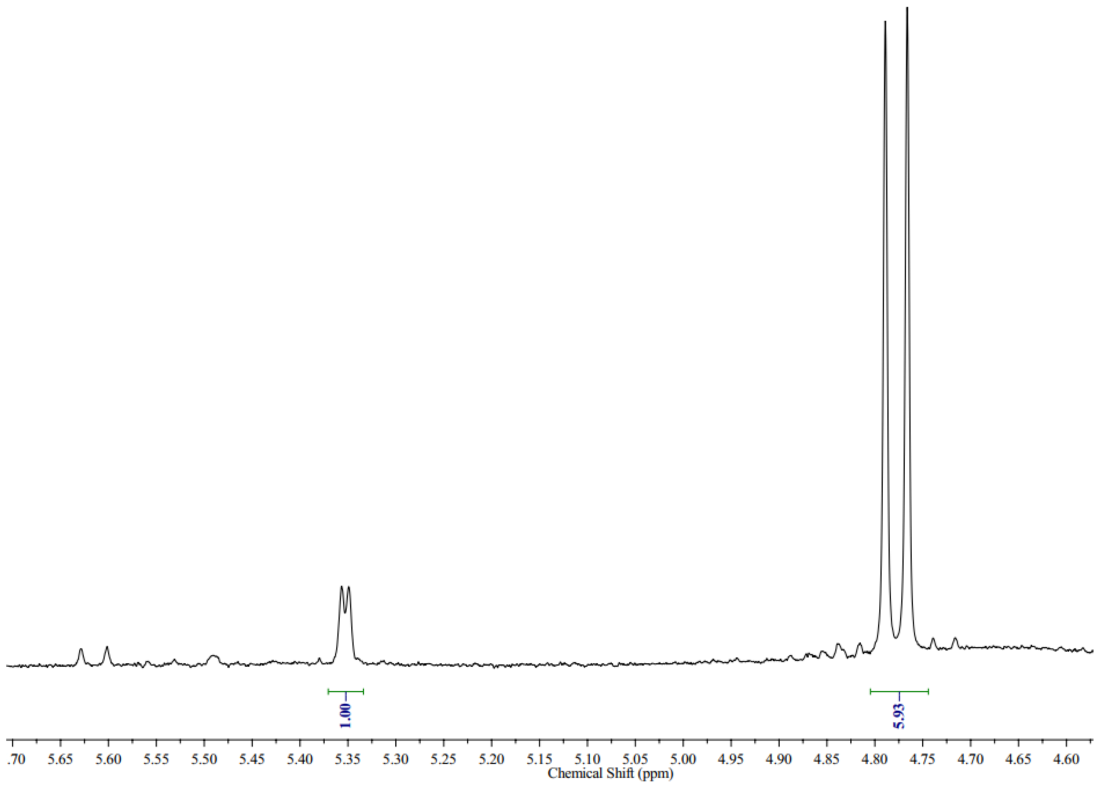

NMR traces for reaction with: **3,5-OMe-phenylboronic acid (400 MHz,** CDCl$_3$**)**

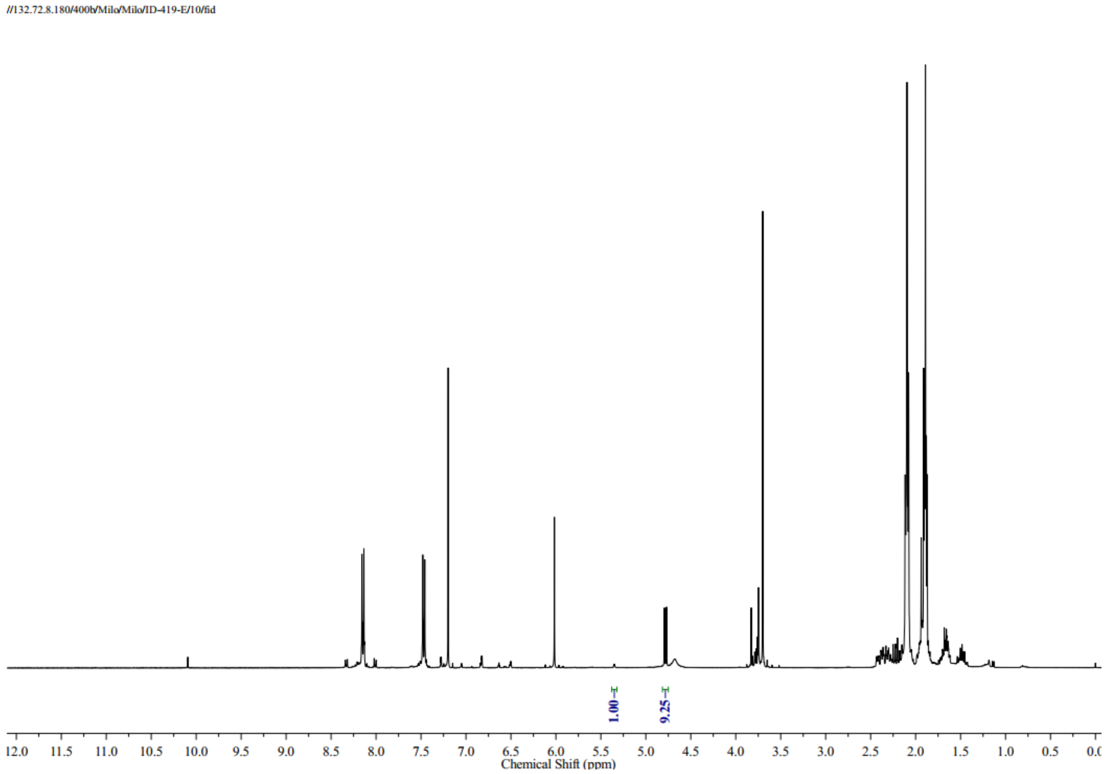

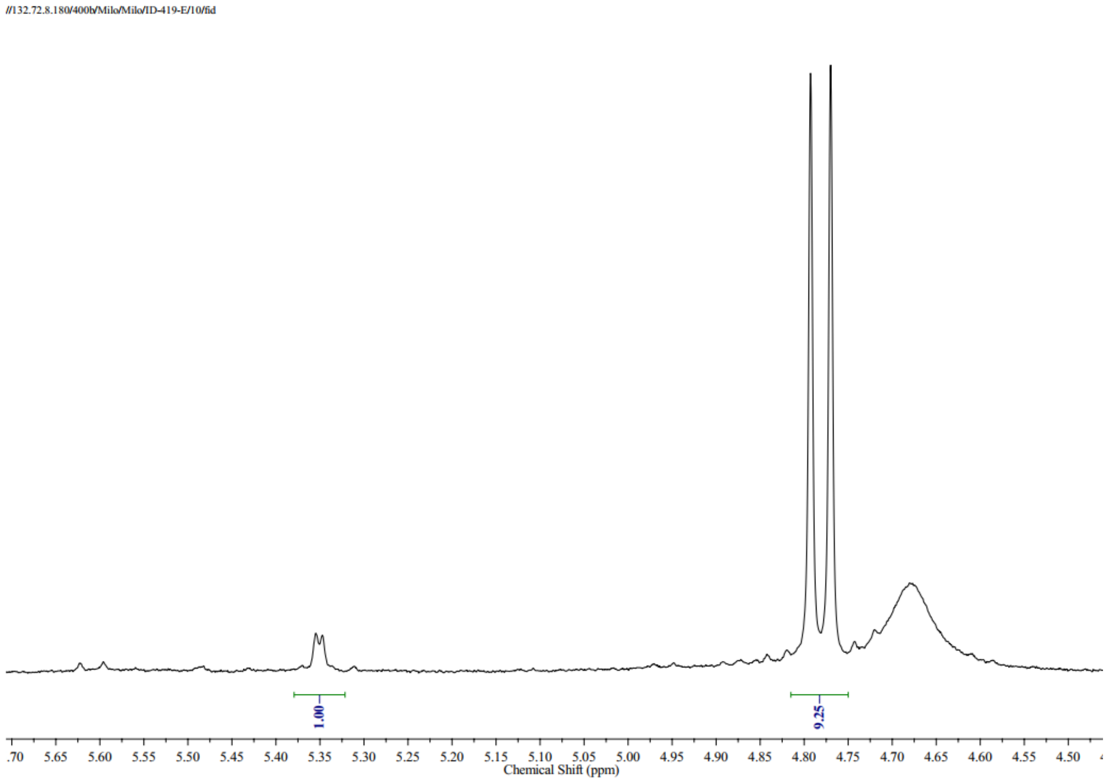

NMR traces for reaction with: **3,5-OMe-phenylboronic acid (400 MHz,** $CDCl_3$**)**

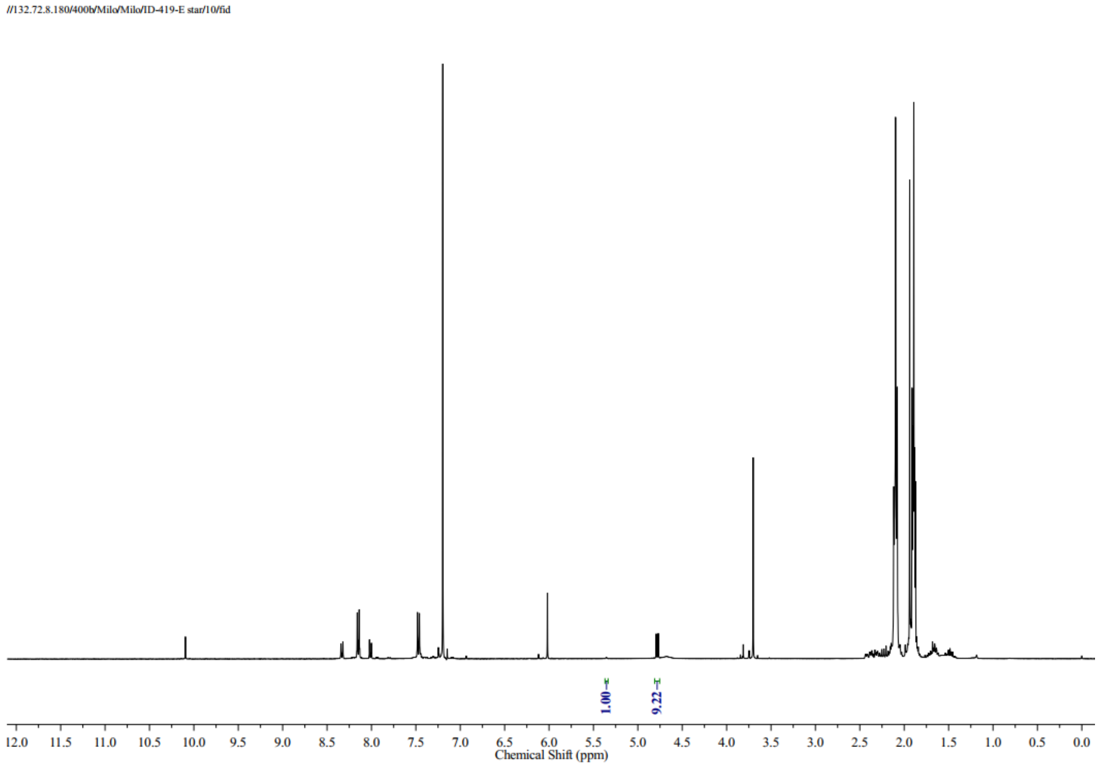

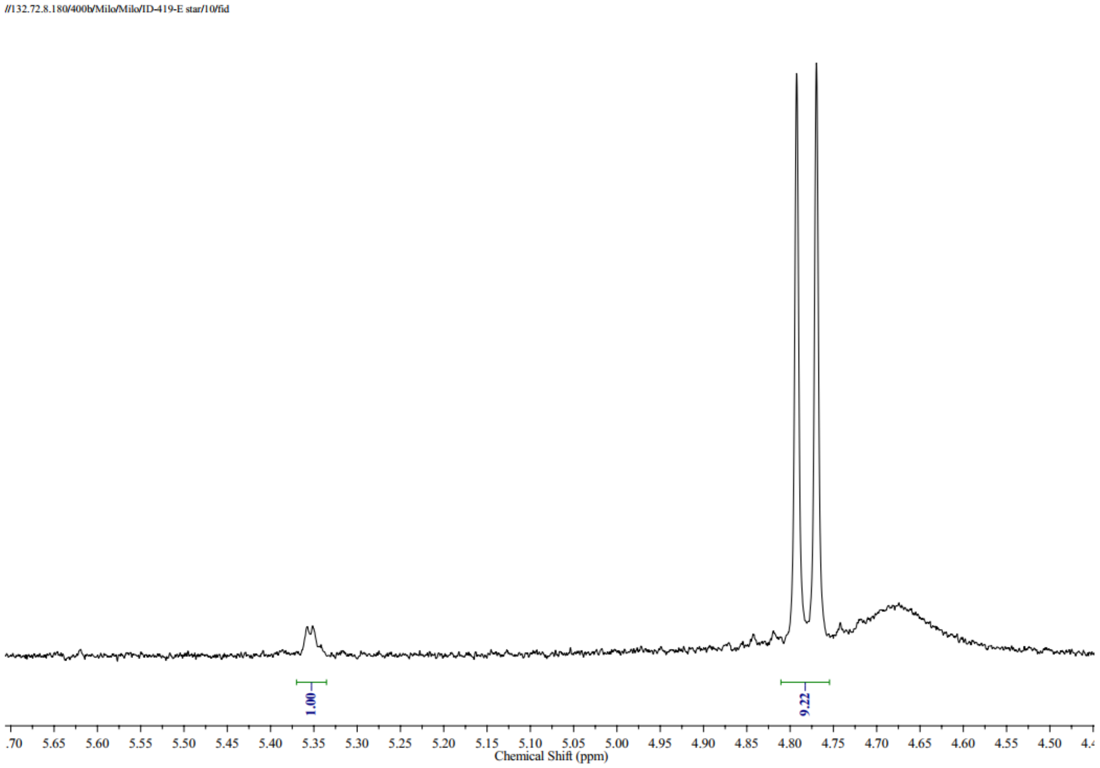

NMR traces for reaction with: **4-Me-phenylboronic acid (400 MHz,** CDCl$_3$**)**

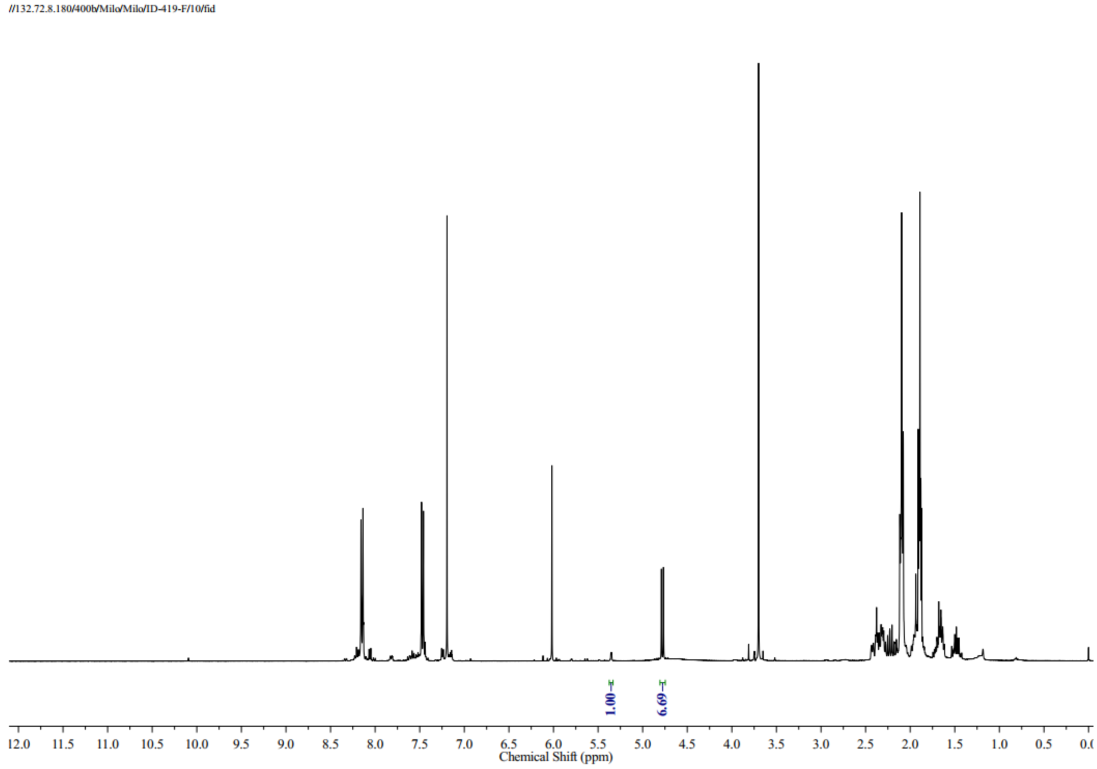

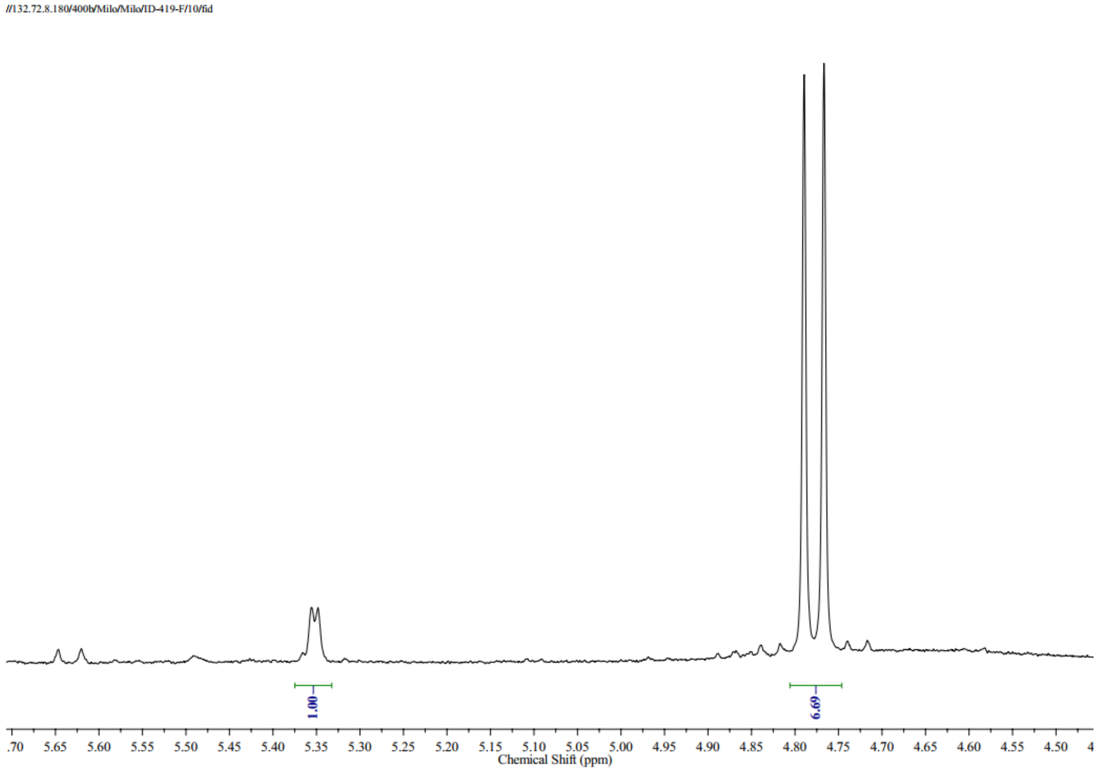

NMR traces for reaction with: **4-Me-phenylboronic acid (duplicate) (400 MHz,** CDCl$_3$**)**

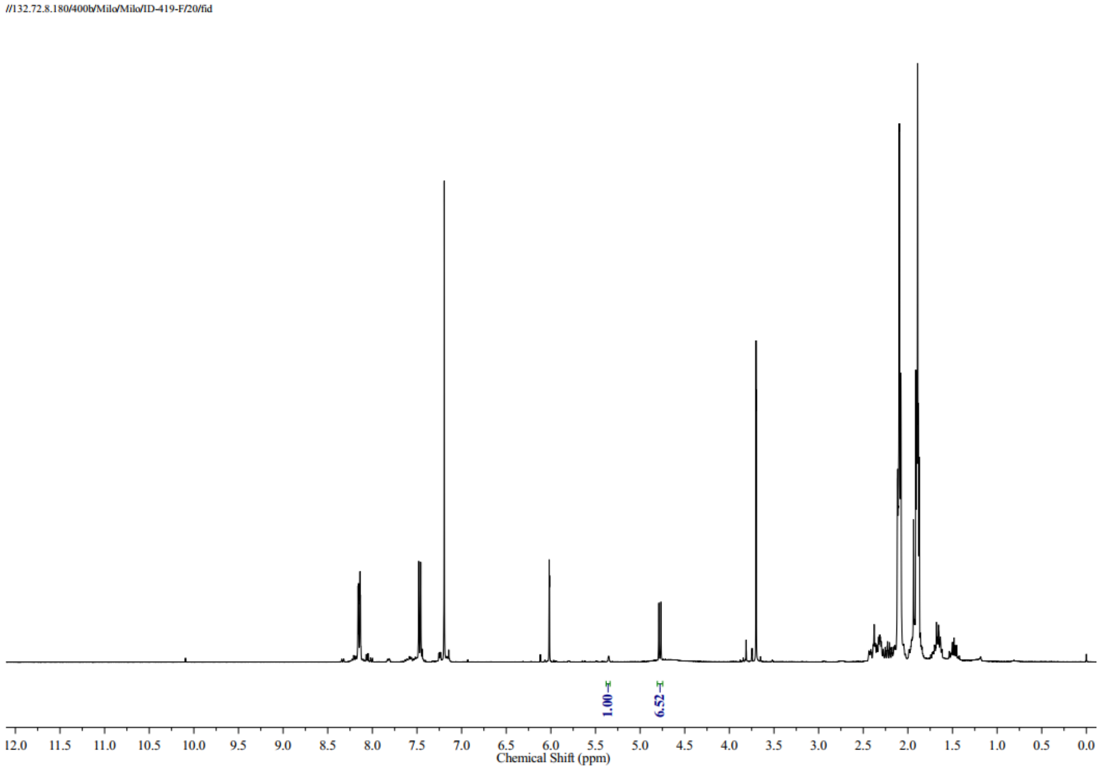

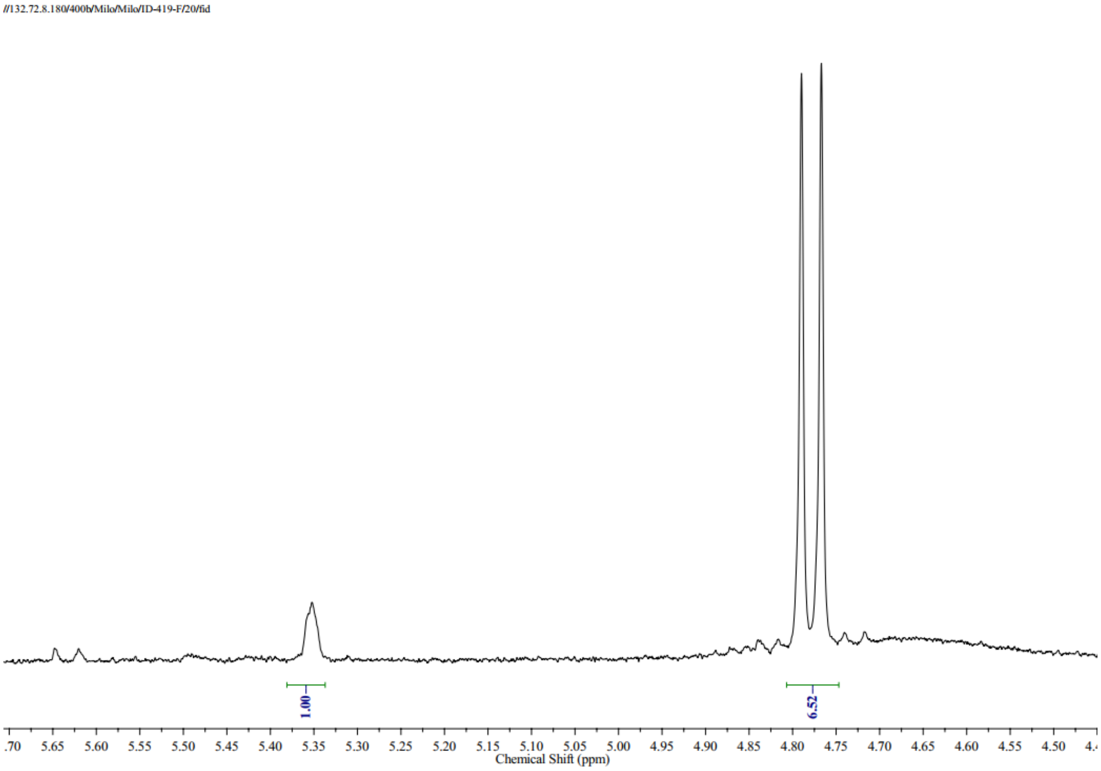

NMR traces for reaction with: **3-CF_3-phenylboronic acid (400 MHz,** $CDCl_3$**)**

//132.72.8.180/400b/Milo/Milo/ID-419-G/10/fid

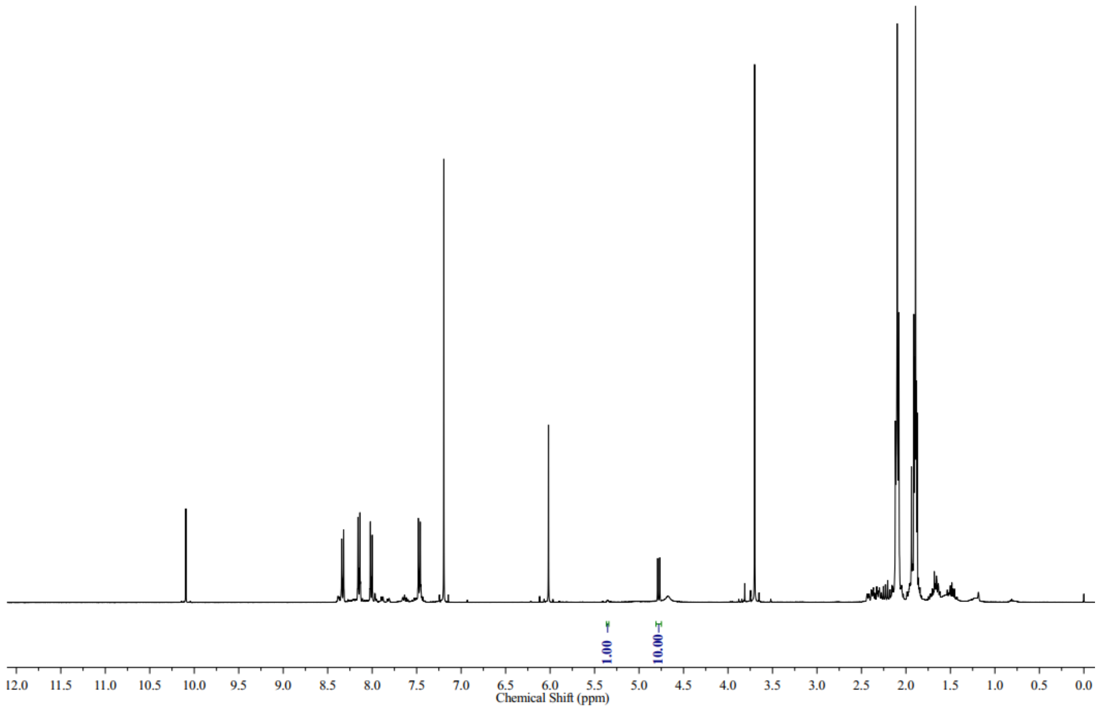

//132.72.8.180/400b/Milo/Milo/ID-419-G/10/fid

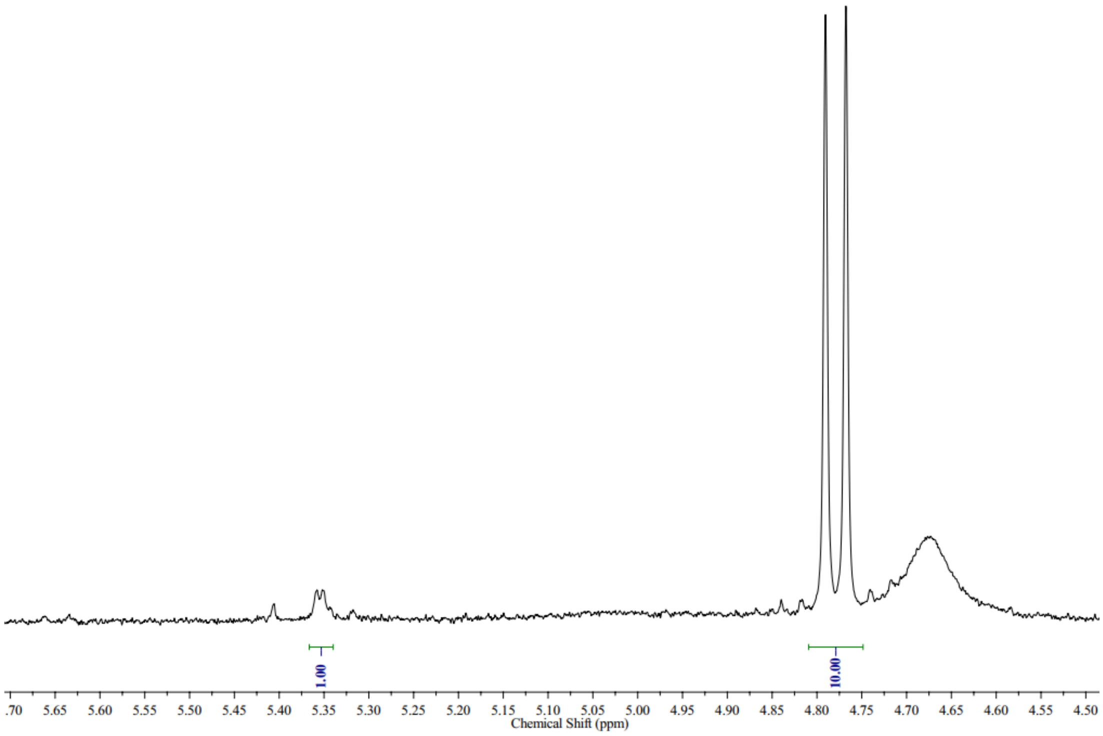

NMR traces for reaction with: **3-CF_3-phenylboronic acid (duplicate) (400 MHz,** $CDCl_3$**)**

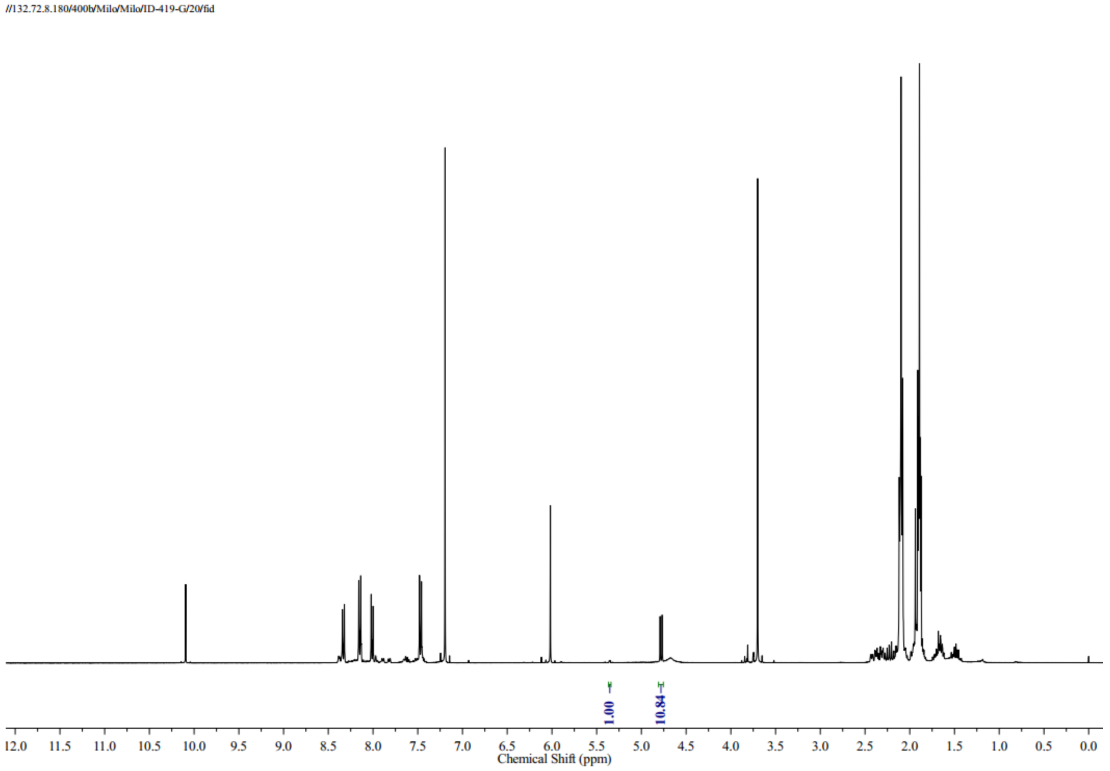

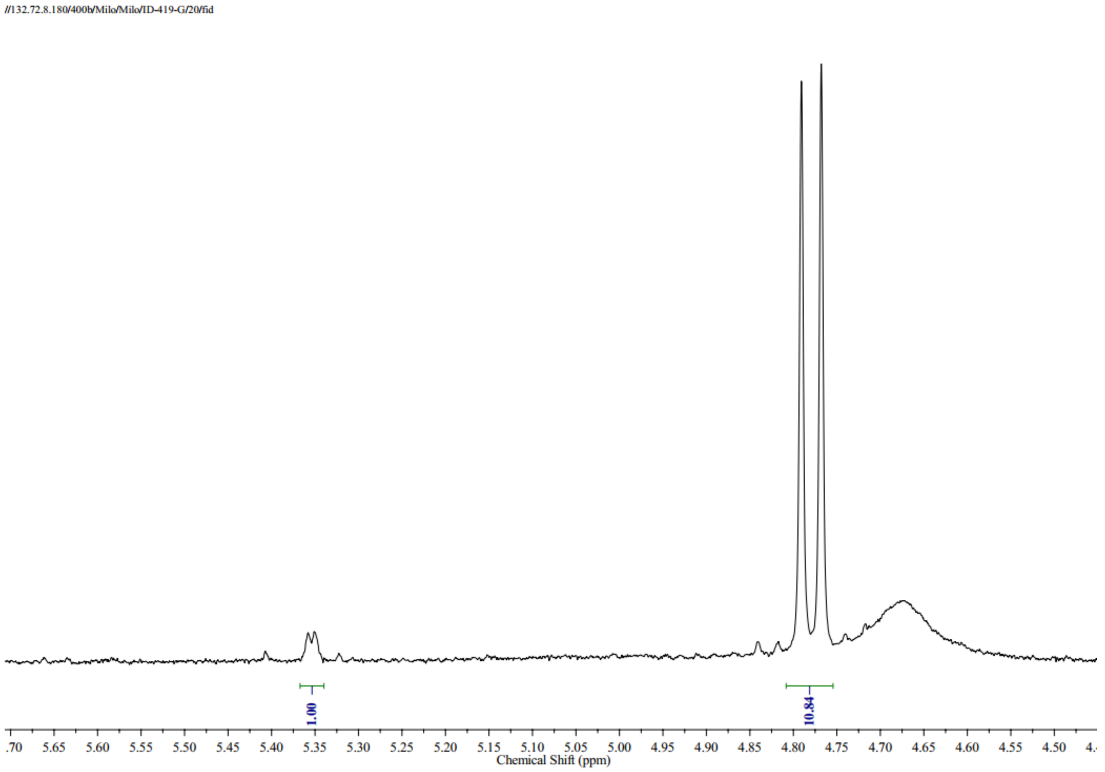

NMR traces for reaction with: **2-Naphtalene-phenylboronic acid (400 MHz,** $CDCl_3$**)**

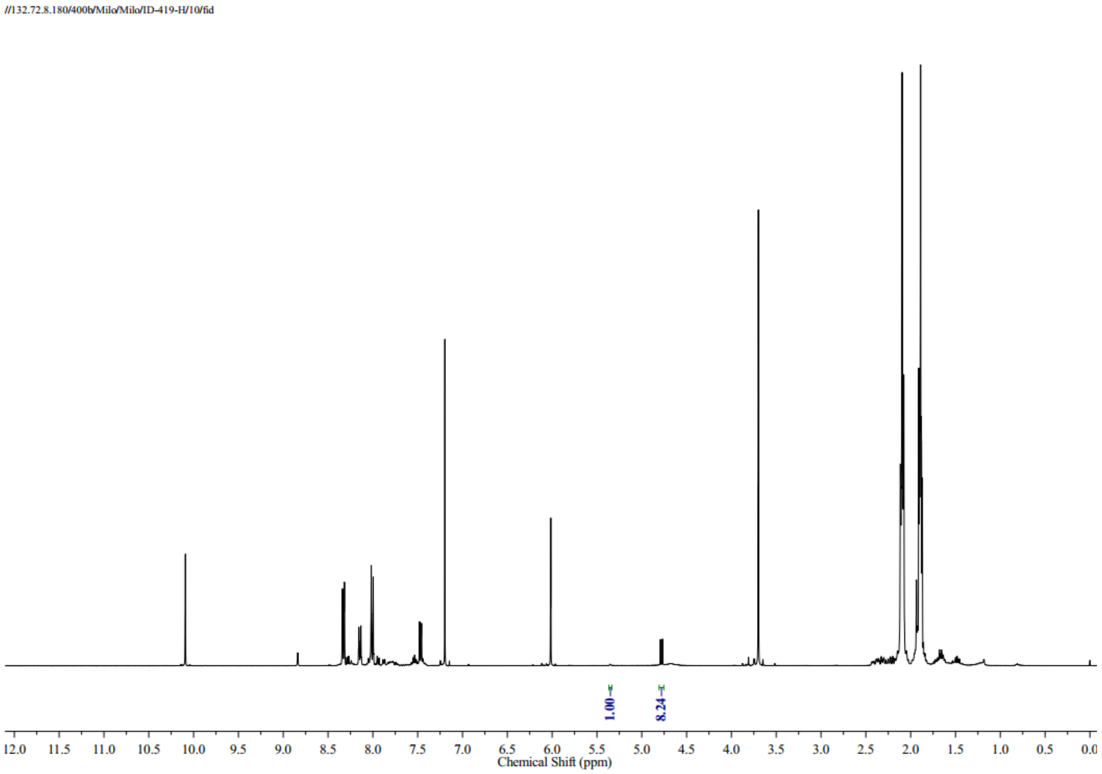

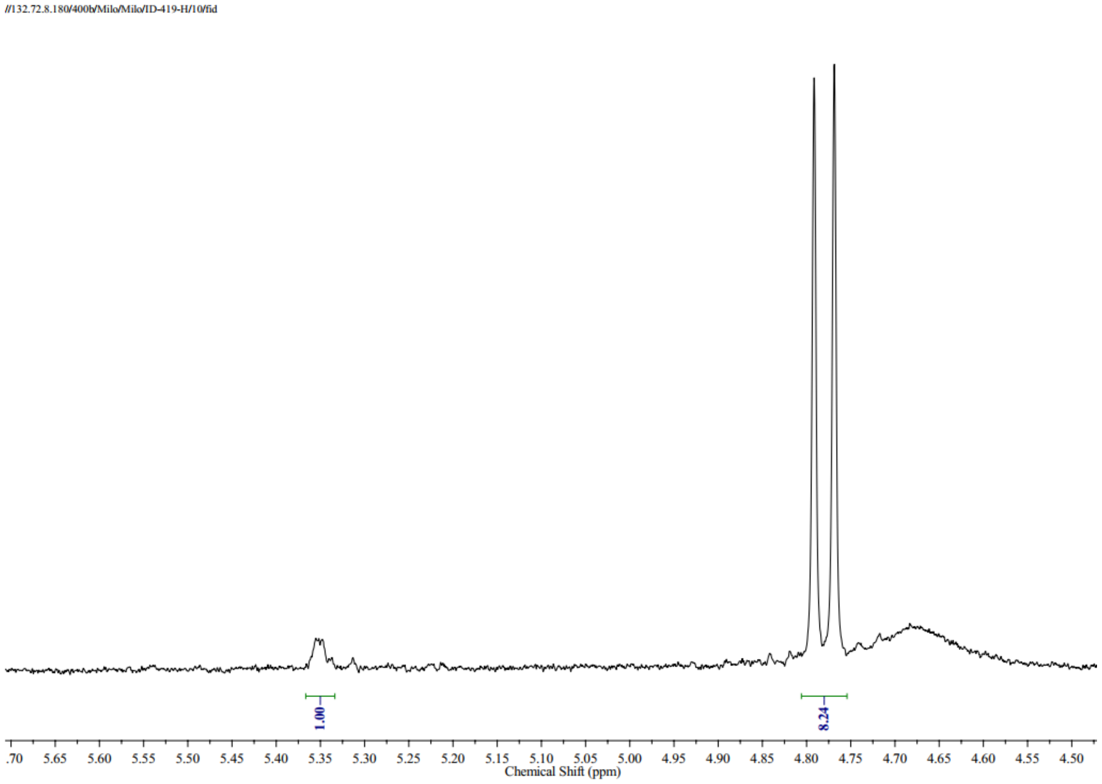

NMR traces for reaction with: **2-Naphtalene-phenylboronic acid (duplicate) (400 MHz,** CDCl$_3$**)**

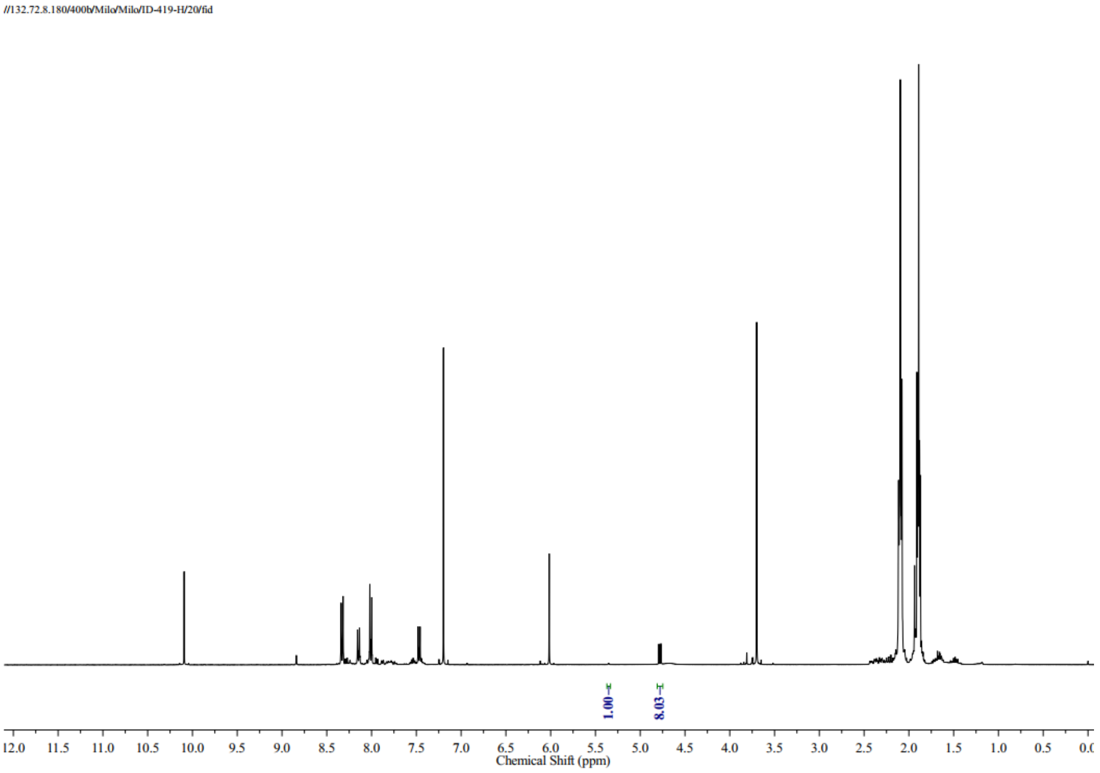

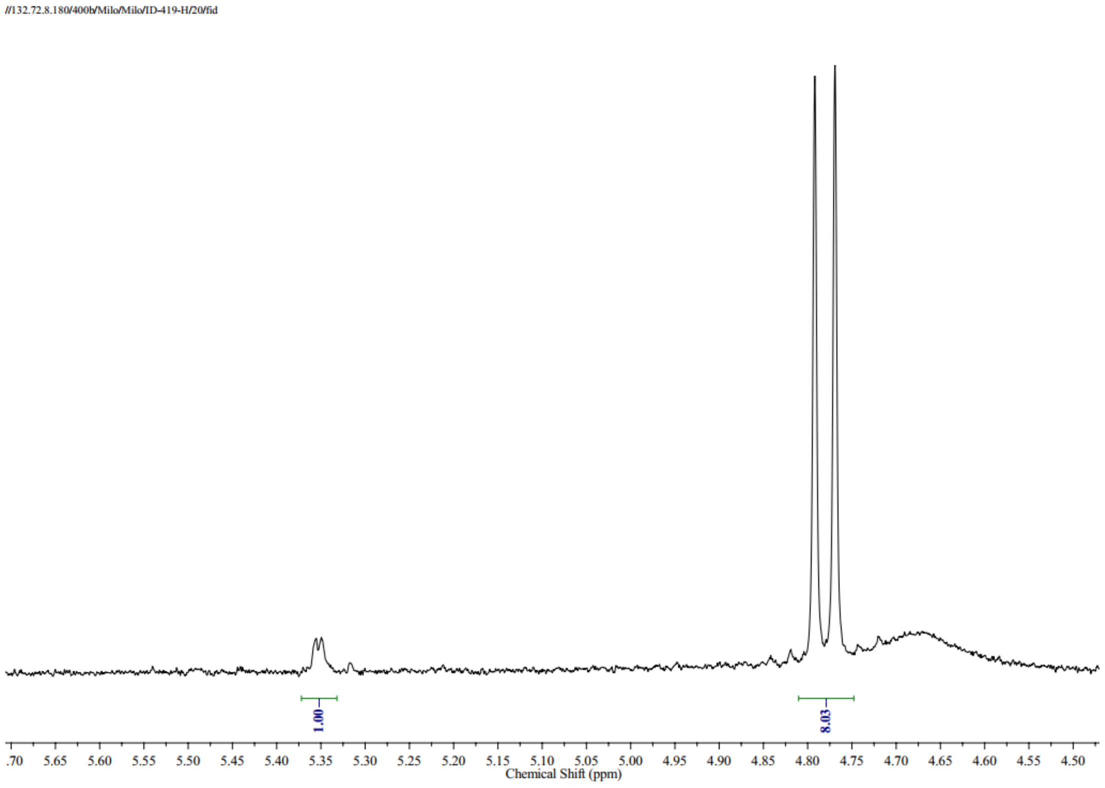

NMR traces for reaction with: **4-CF_3-phenylboronic acid (400 MHz,** $CDCl_3$**)**

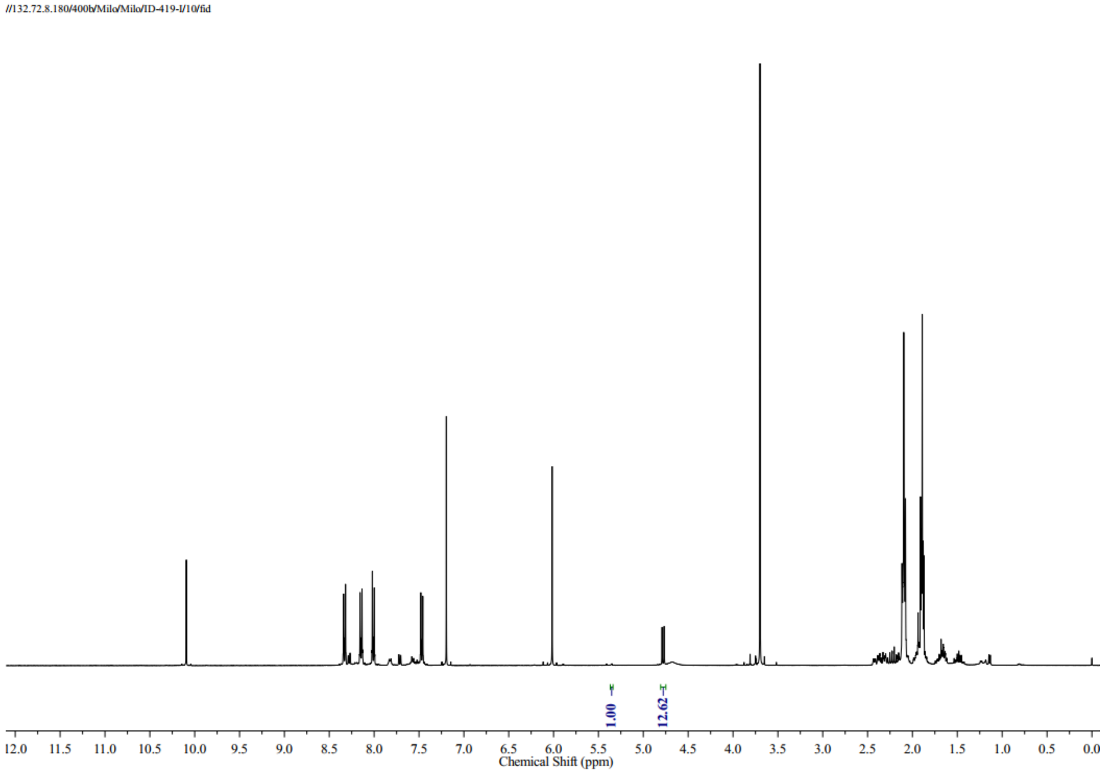

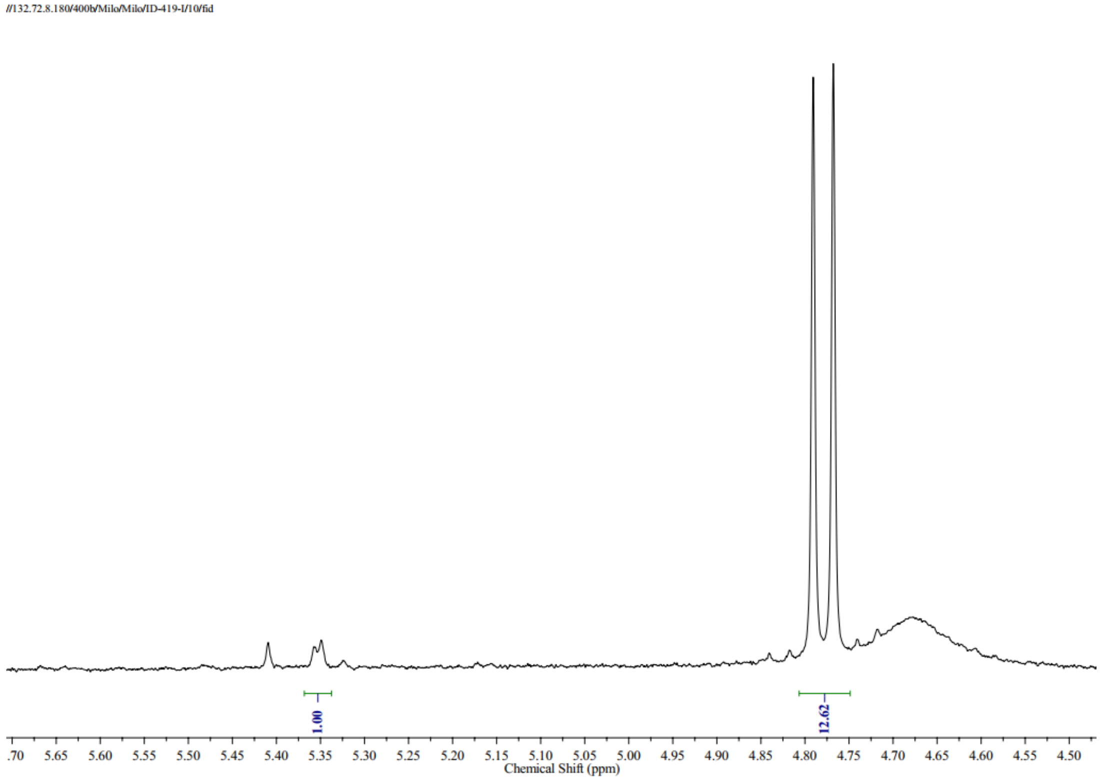

NMR traces for reaction with: **4-CF_3-phenylboronic acid (duplicate) (400 MHz,** $CDCl_3$**)**

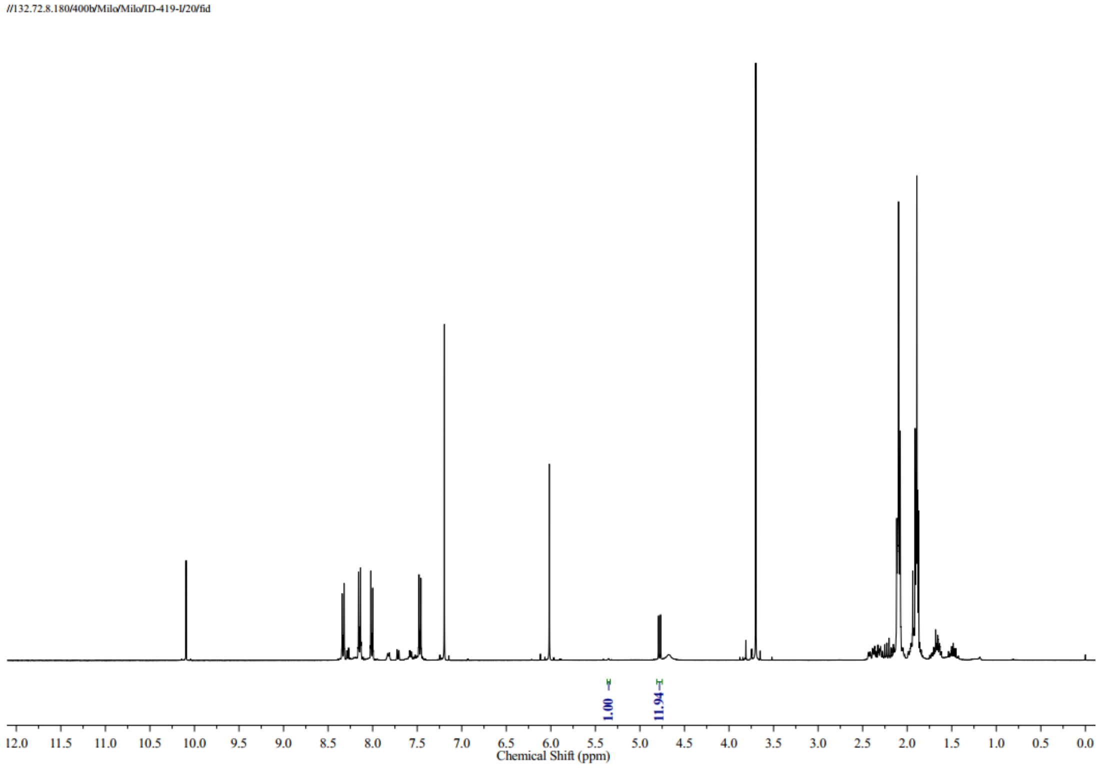

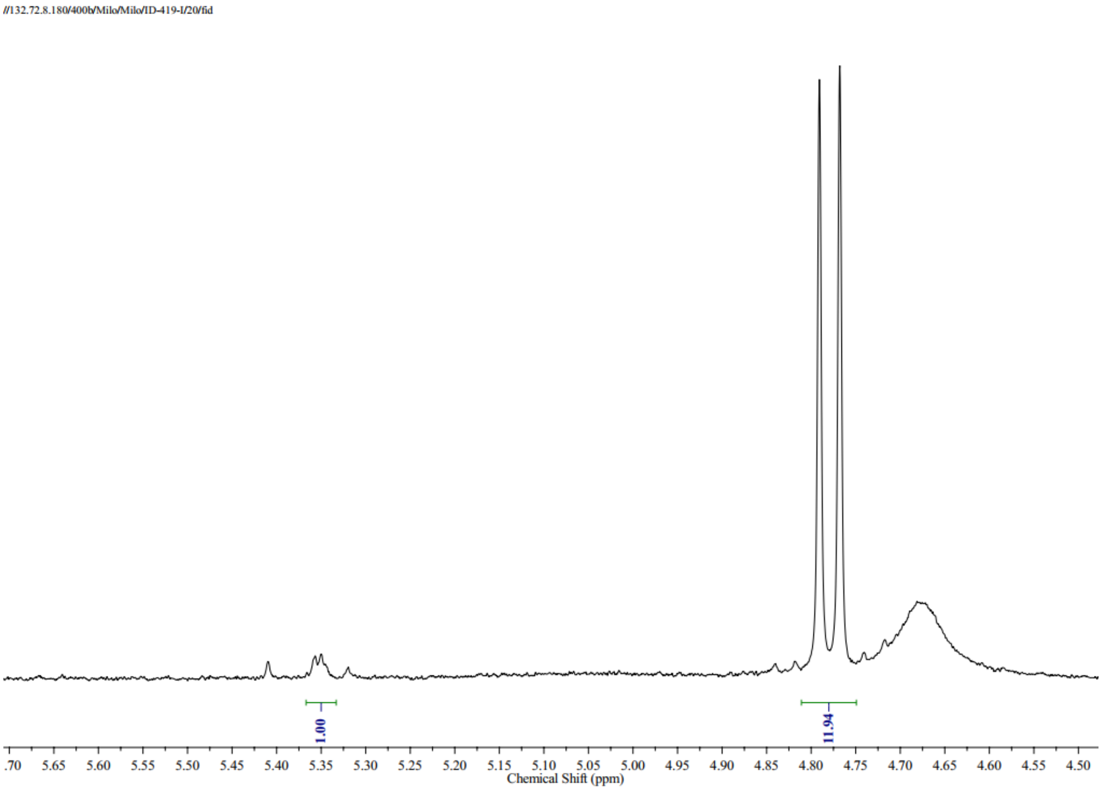

NMR traces for reaction with: **4-F-phenylboronic acid (400 MHz,** $CDCl_3$**)**

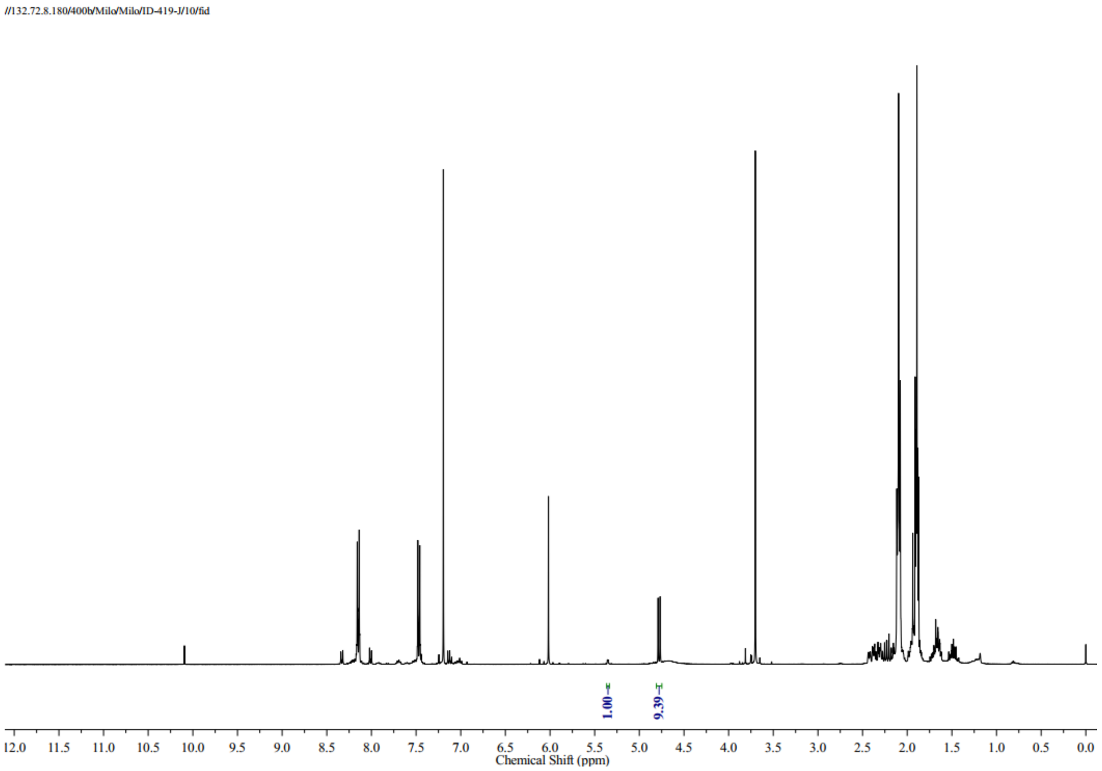

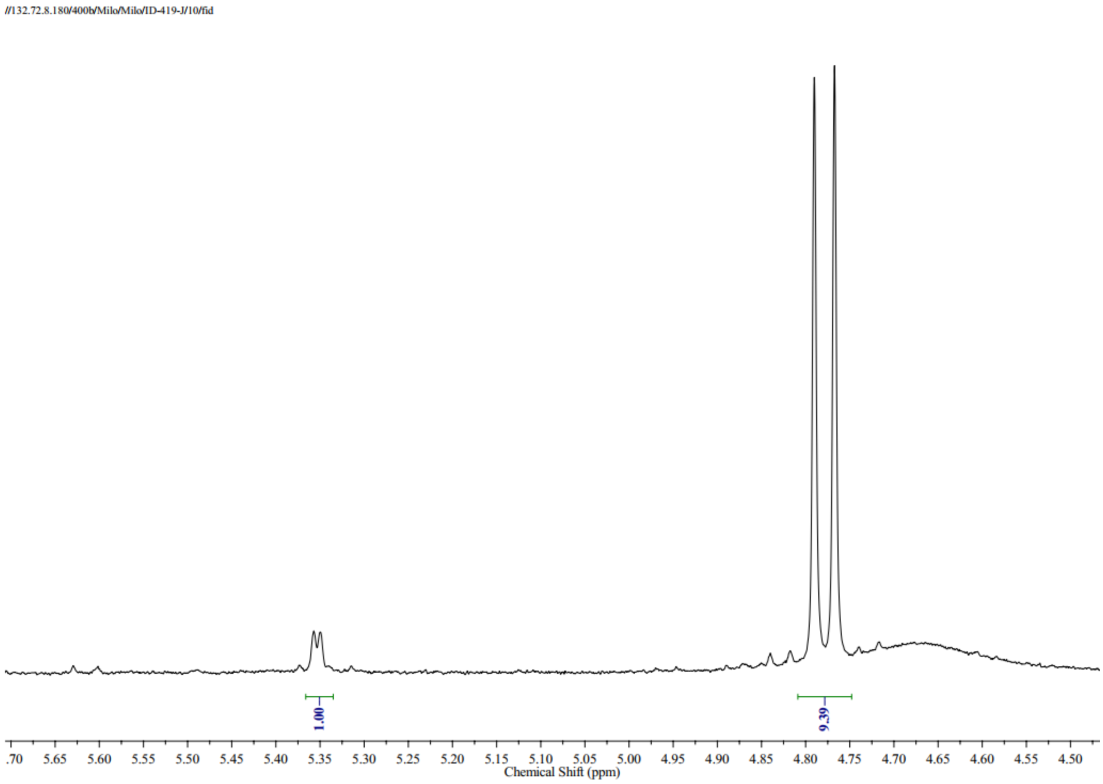

NMR traces for reaction with: **4-F-phenylboronic acid (duplicate) (400 MHz,** CDCl$_3$**)**

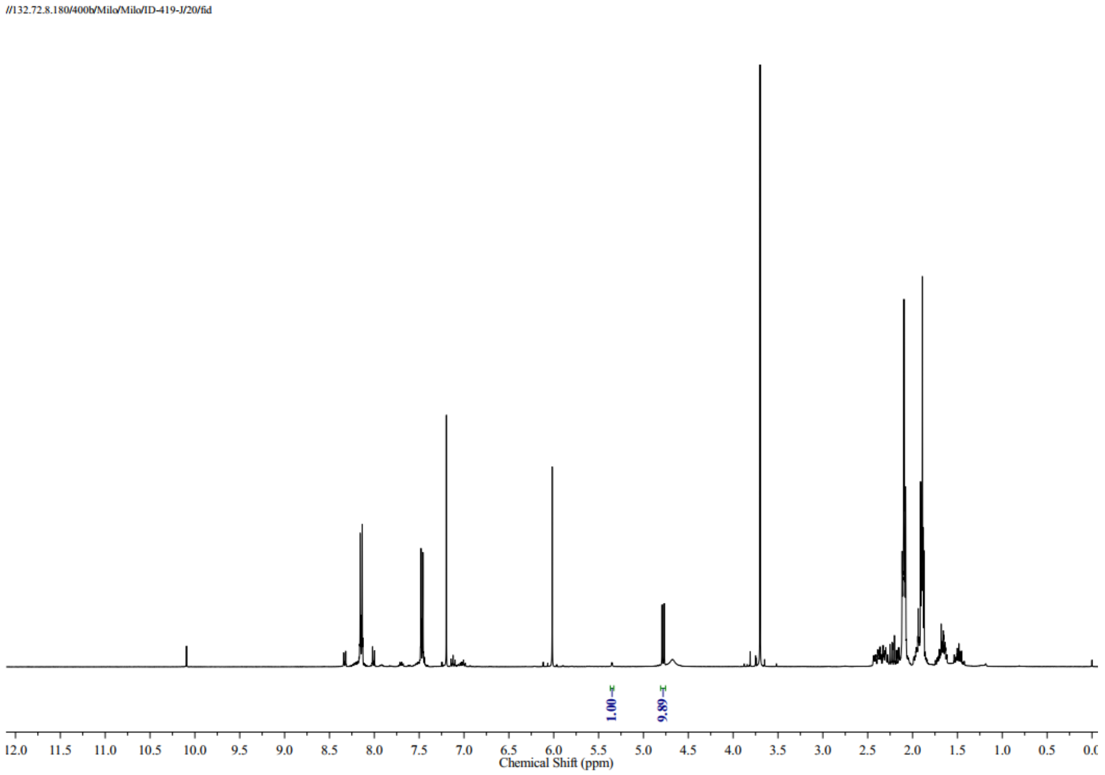

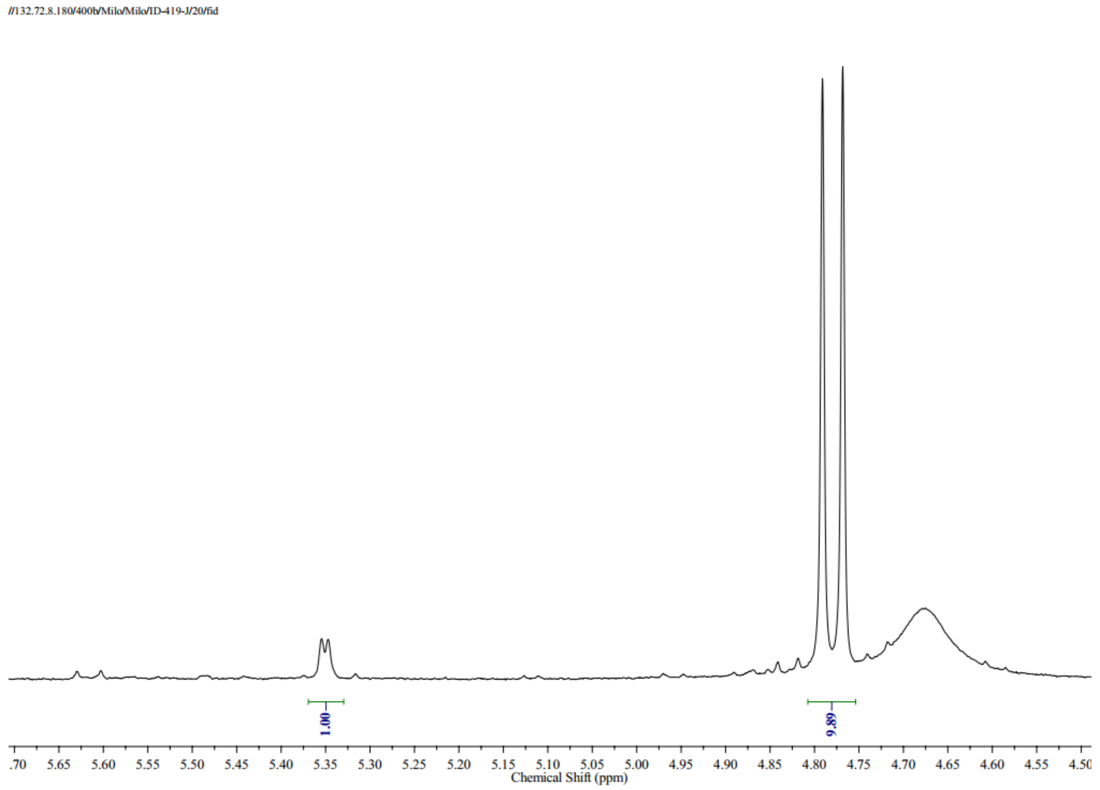

NMR traces for reaction with: **4-OMe-phenylboronic acid (400 MHz,** $CDCl_3$**)**

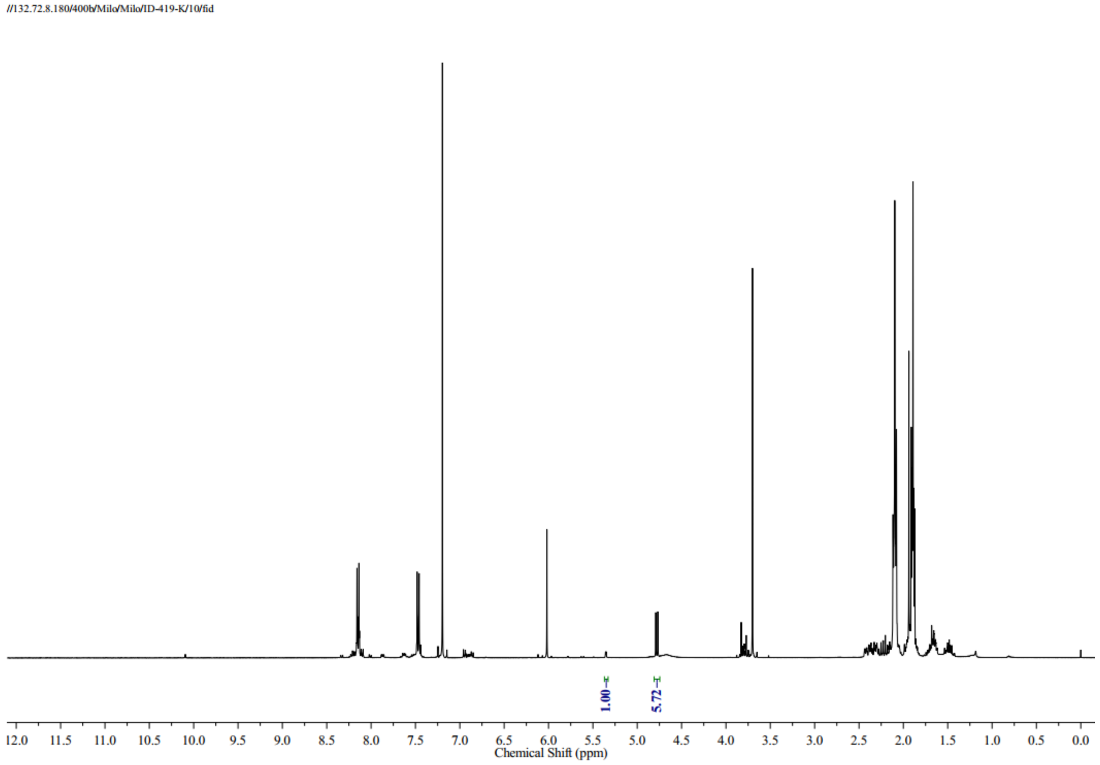

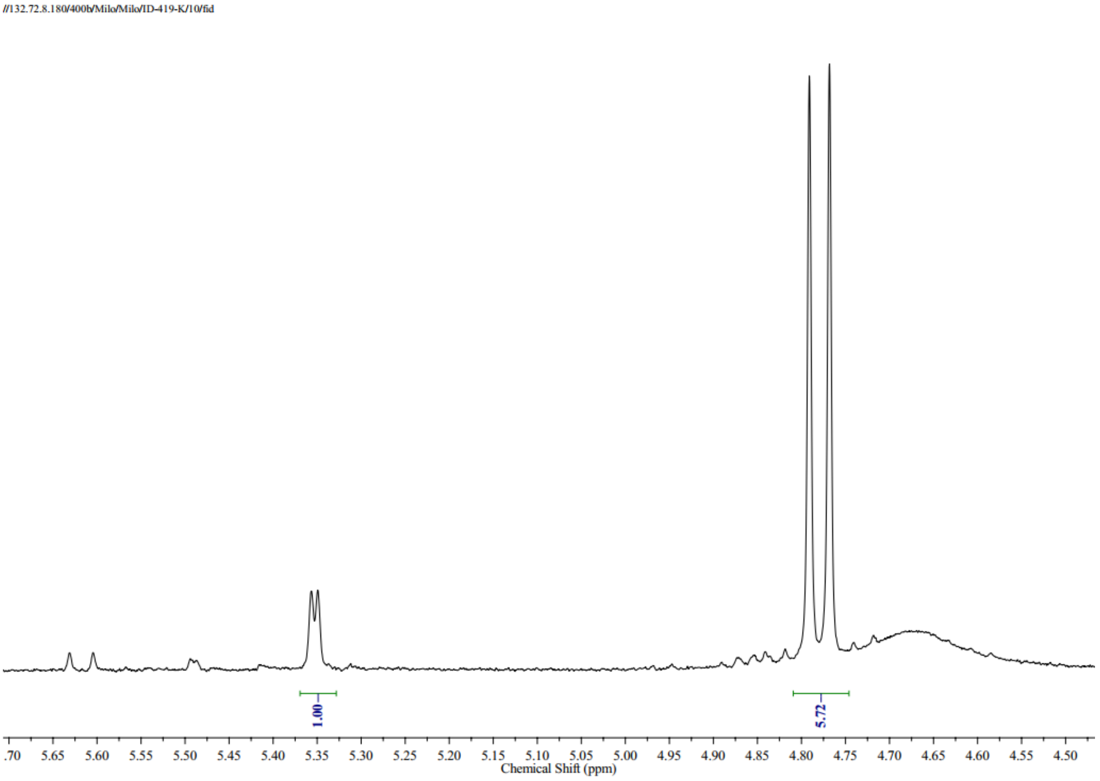

NMR traces for reaction with: **4-OMe-phenylboronic acid (duplicate) (400 MHz,** $CDCl_3$**)**

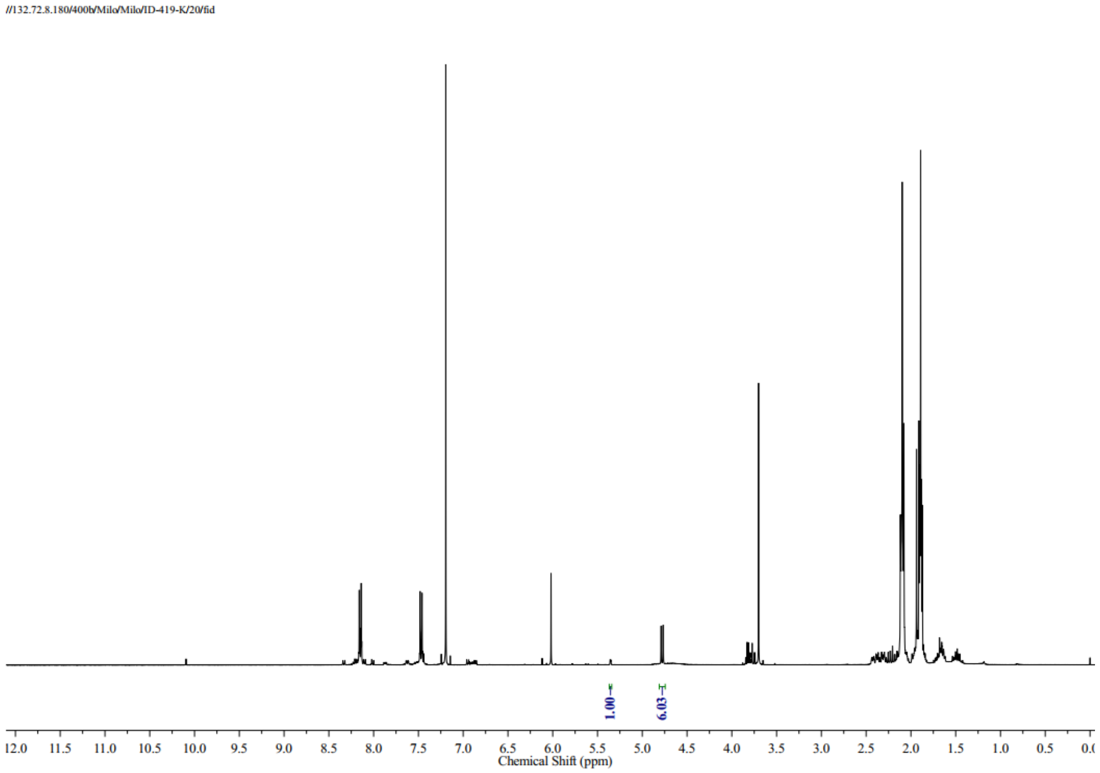

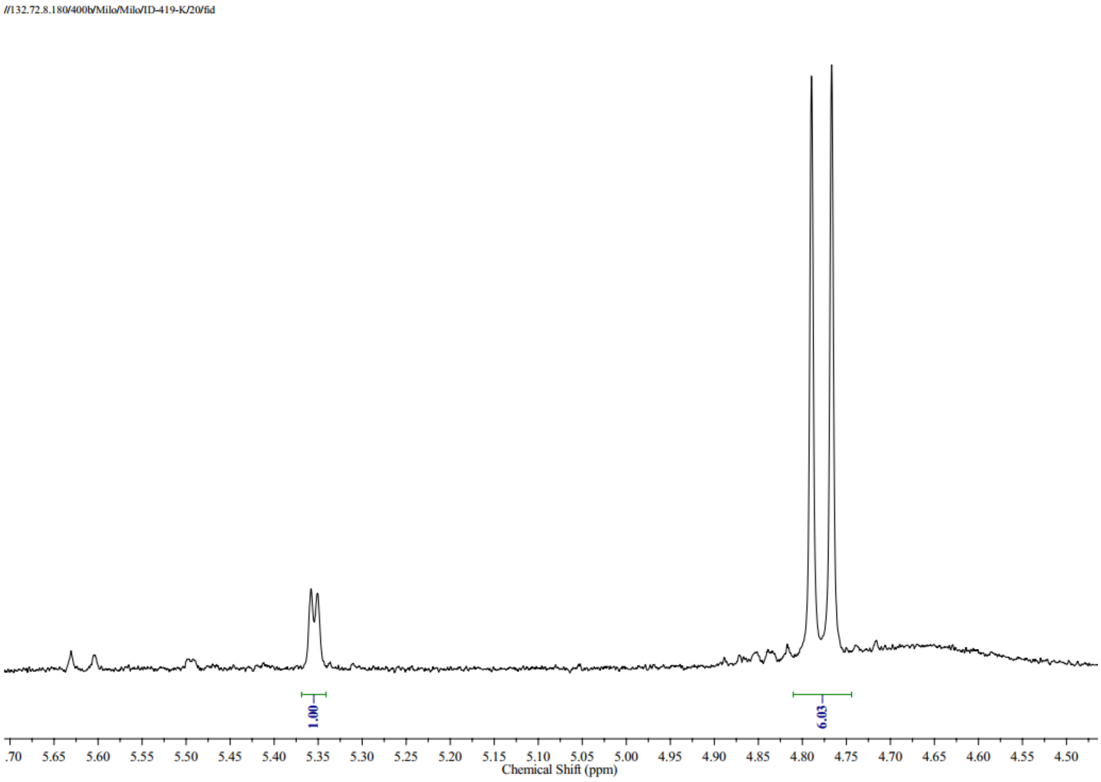

NMR traces for reaction with: **3-Me-phenylboronic acid (400 MHz,** $CDCl_3$**)**

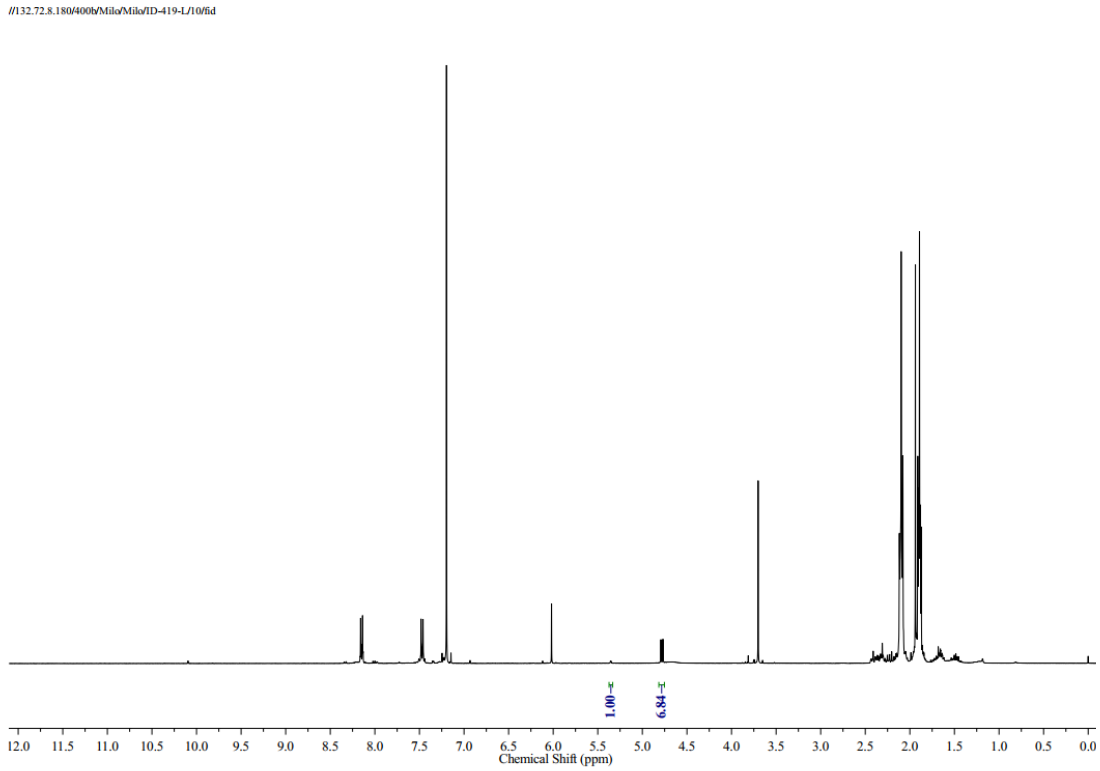

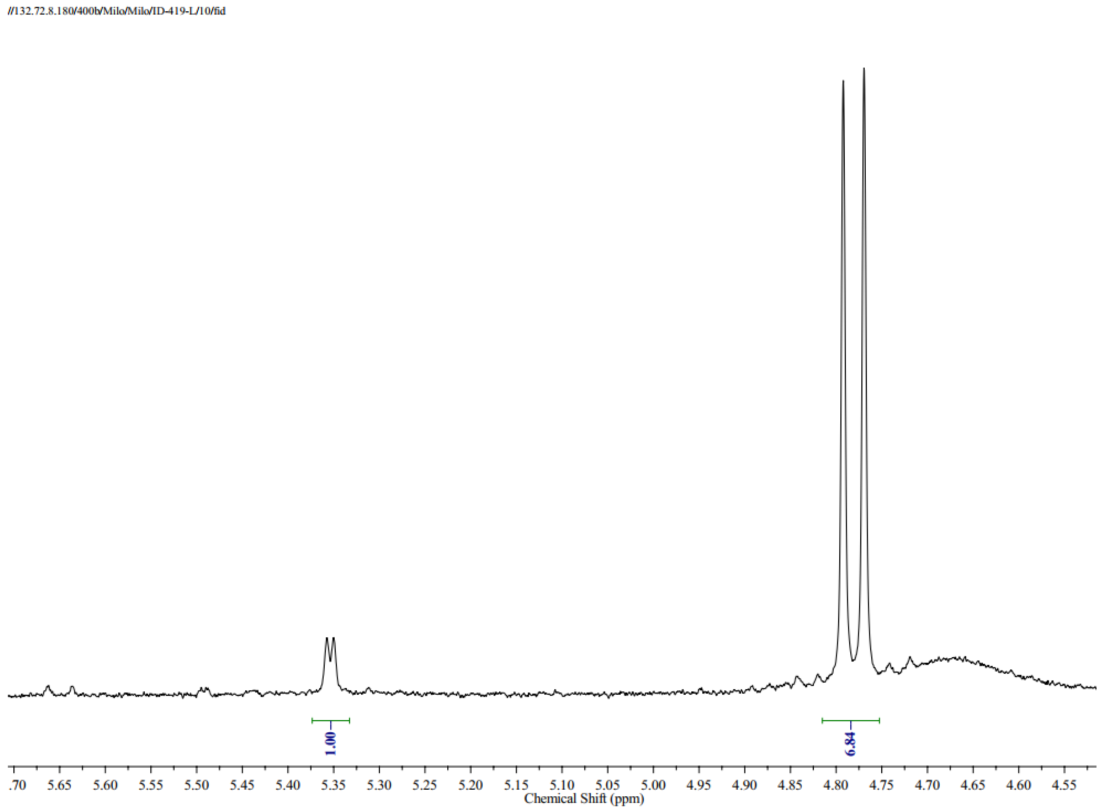

NMR traces for reaction with: **3-Me-phenylboronic acid (400 MHz,** $CDCl_3$**)**

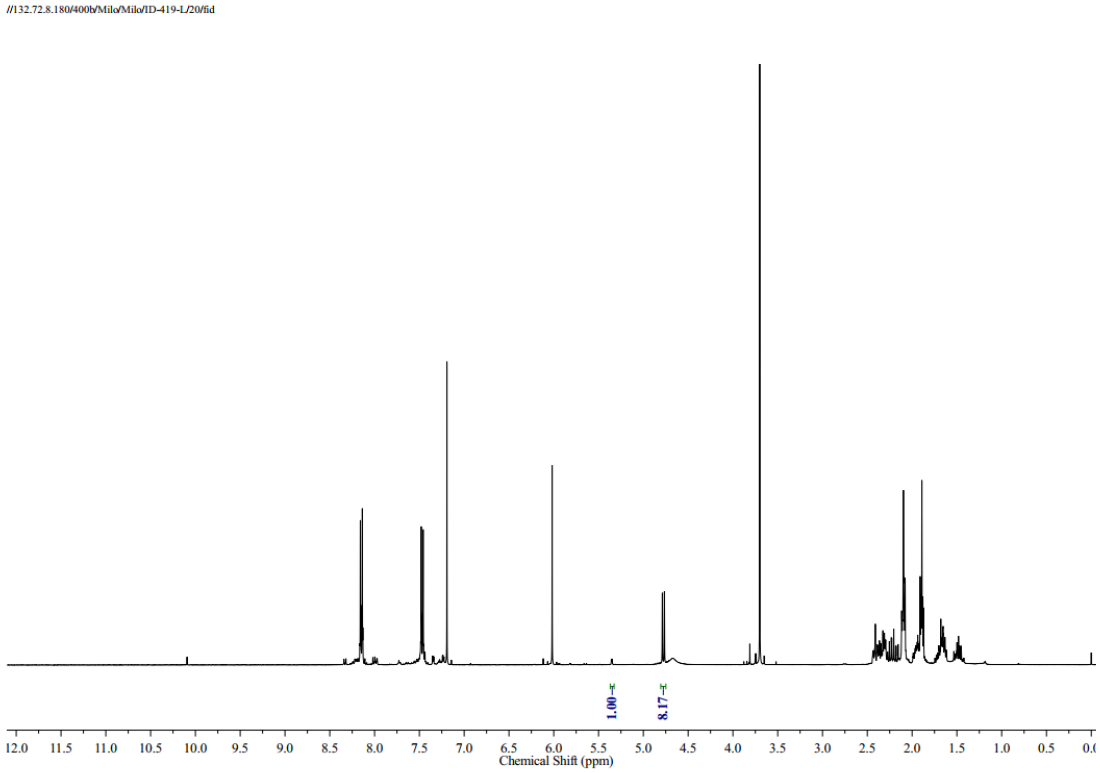

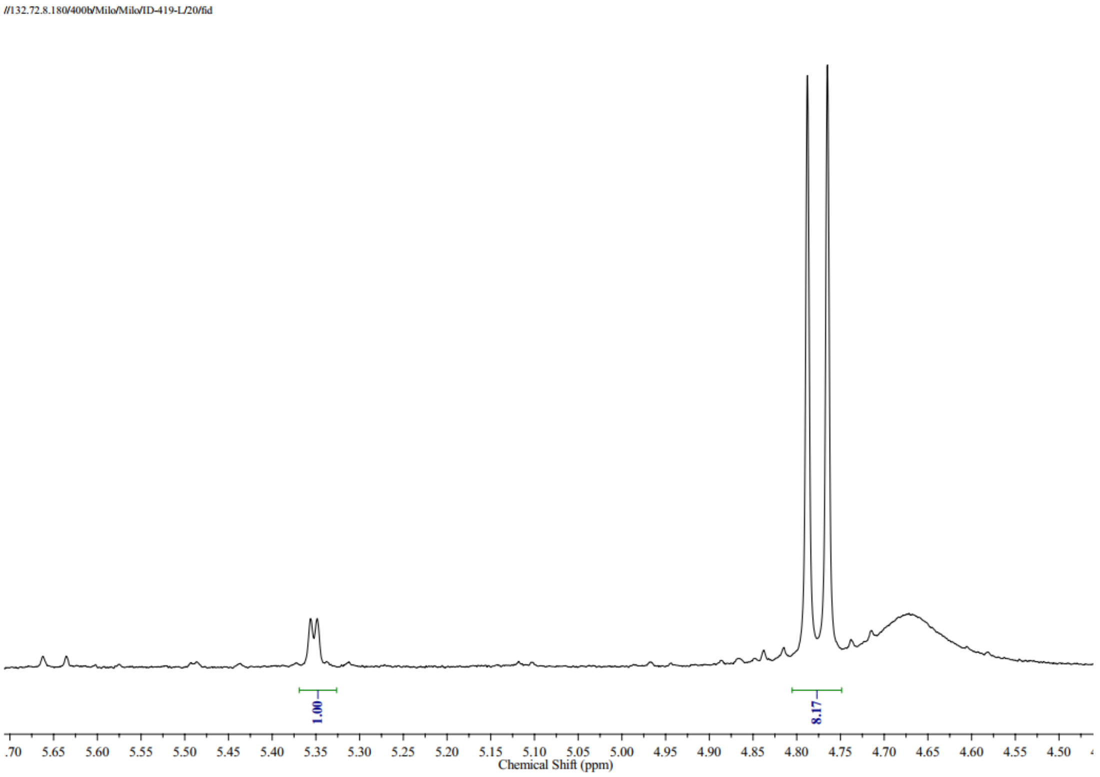

2-Me-phenylboronic acid (400 MHz, CDCl$_3$**)**

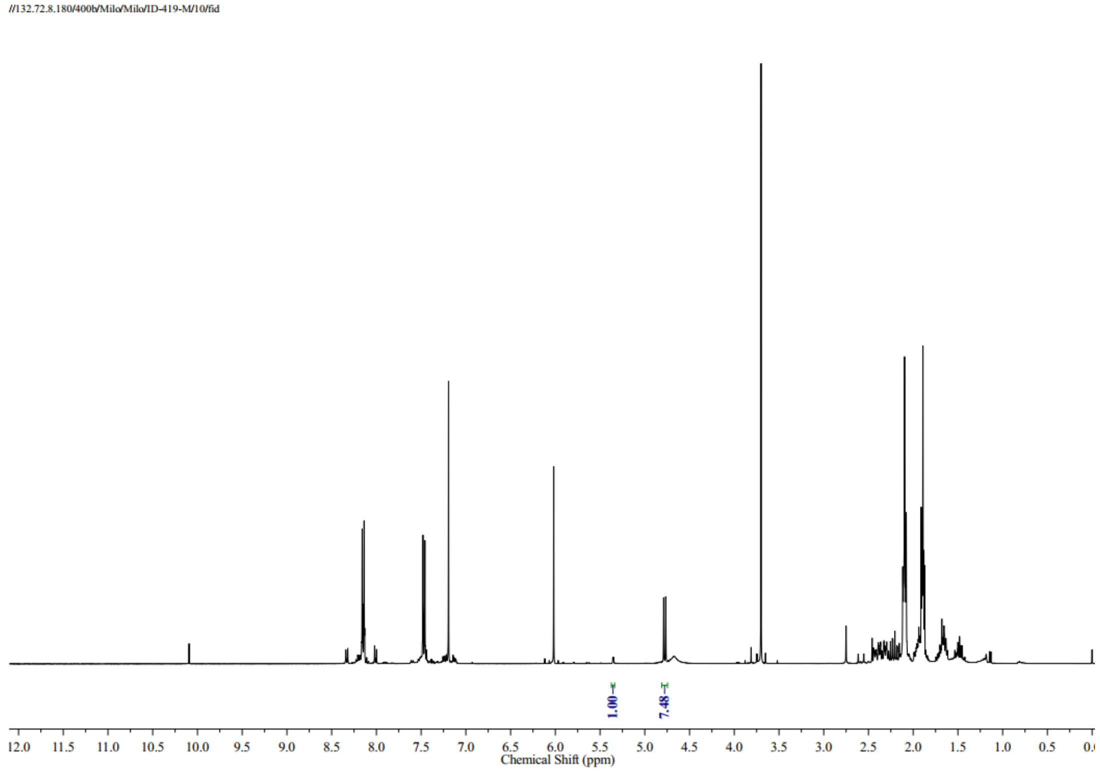

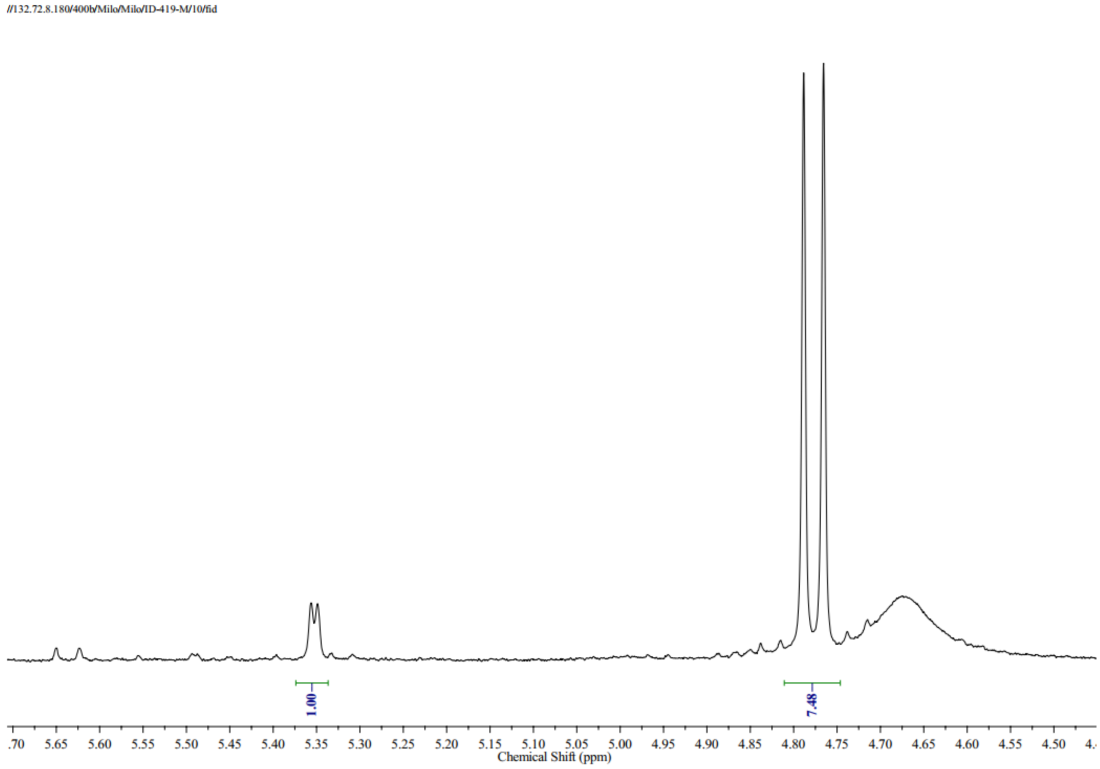

NMR traces for reaction with: **2-Me-phenylboronic acid (duplicate) (400 MHz,** CDCl$_3$**)**

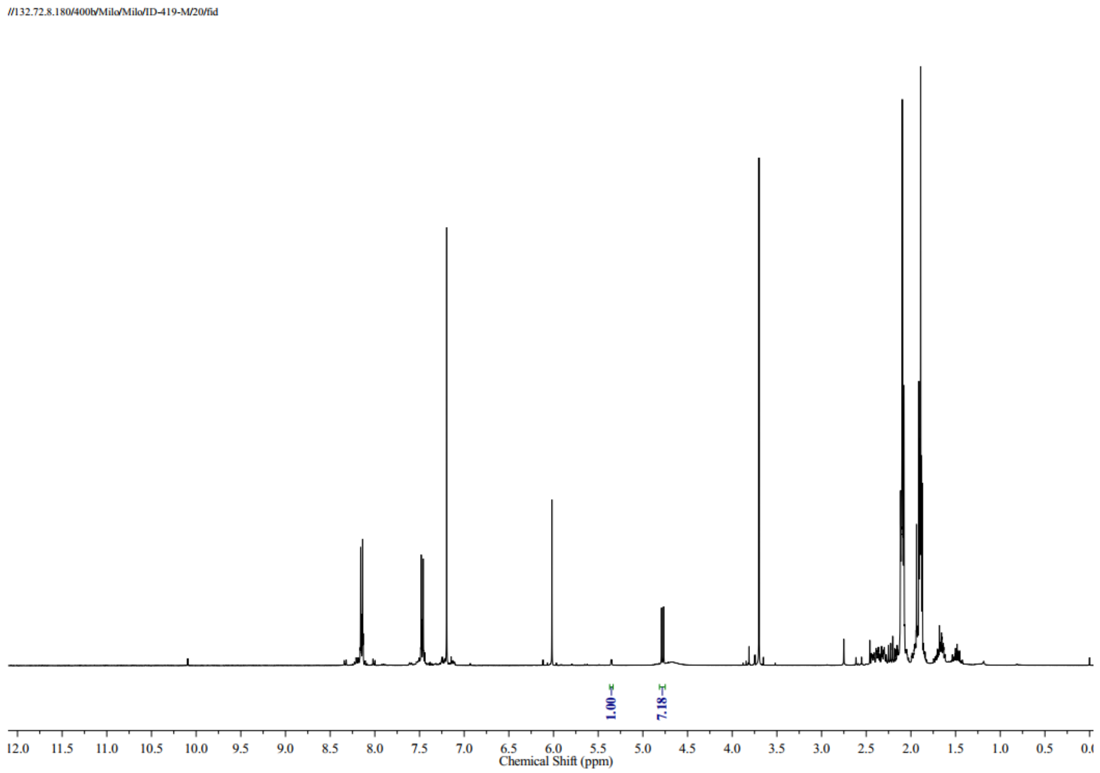

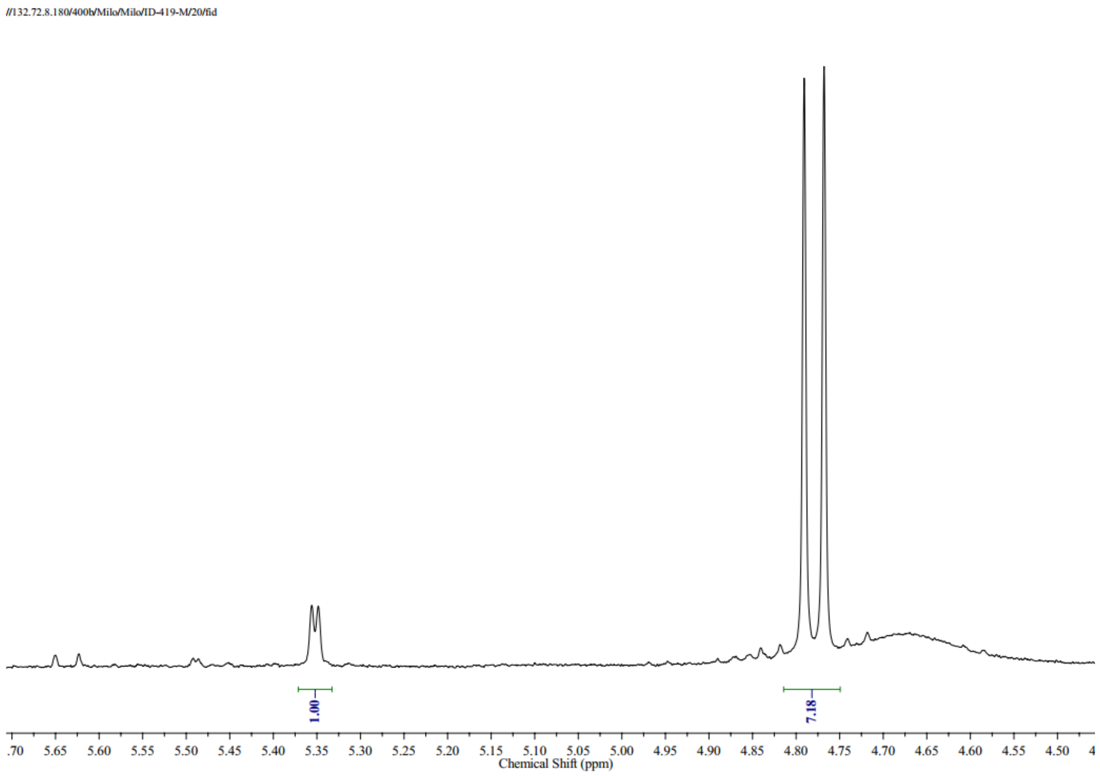

NMR traces for reaction with: **Ph-phenylboronic acid (400 MHz,** CDCl$_3$**)**

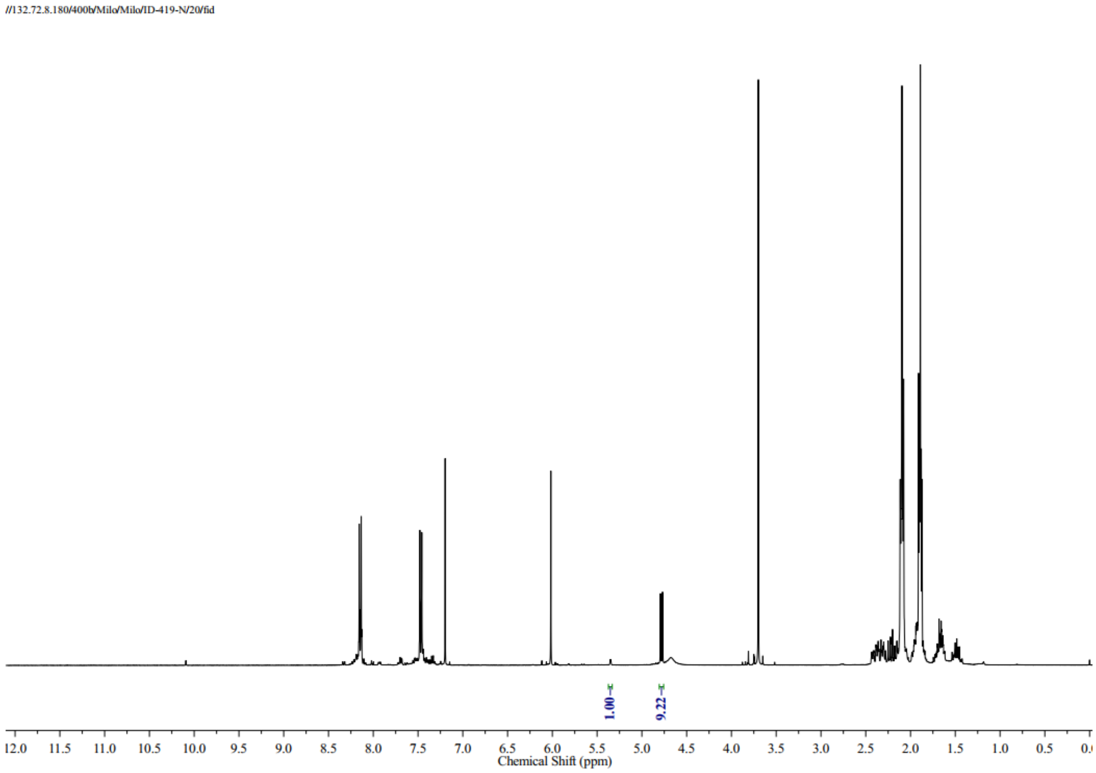

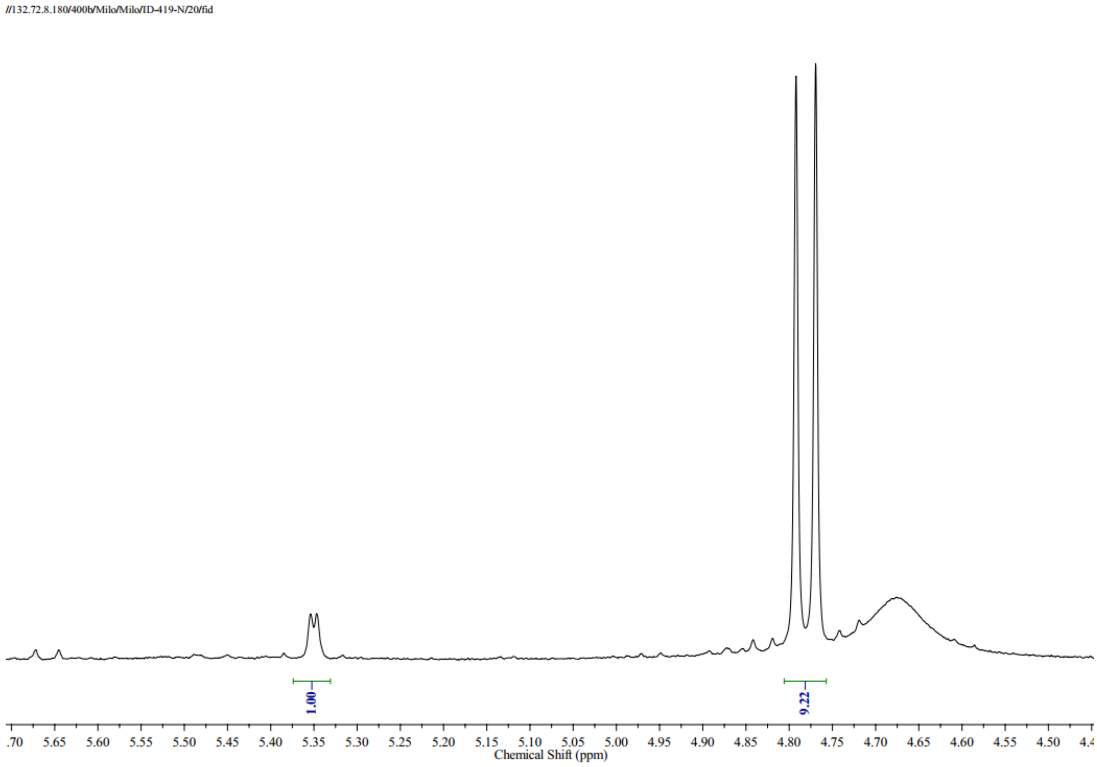

NMR traces for reaction with: **Ph-phenylboronic acid (duplicate) (400 MHz,** $CDCl_3$**)**

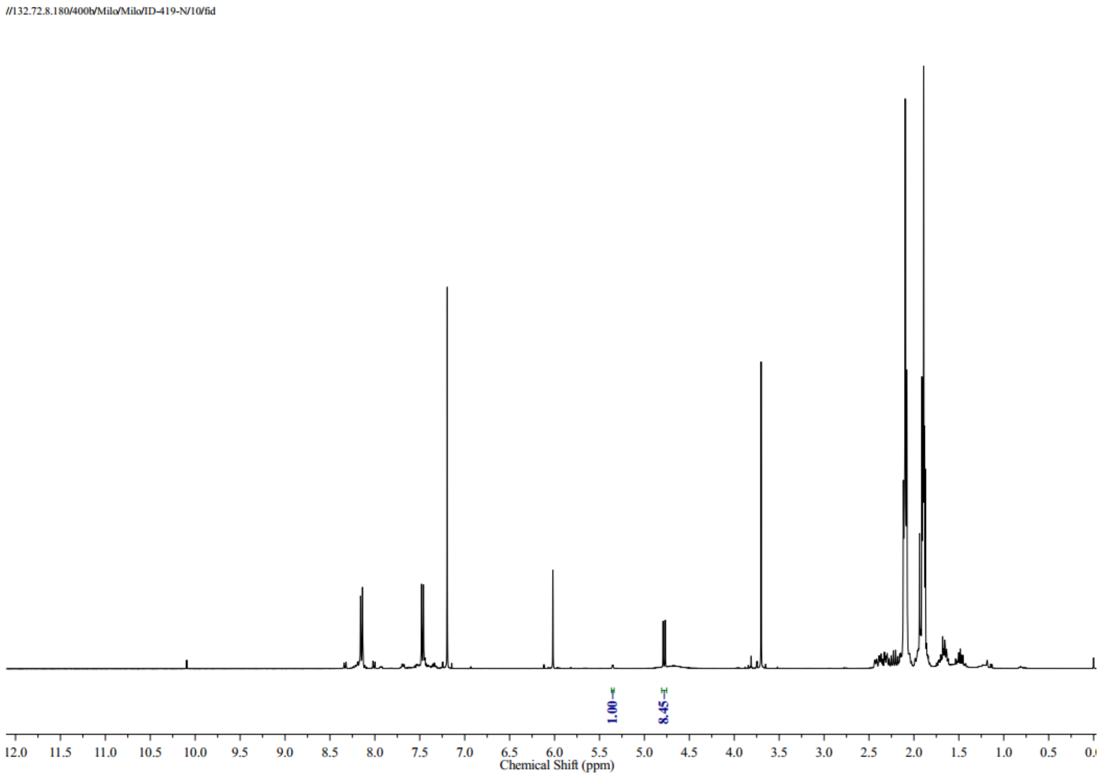

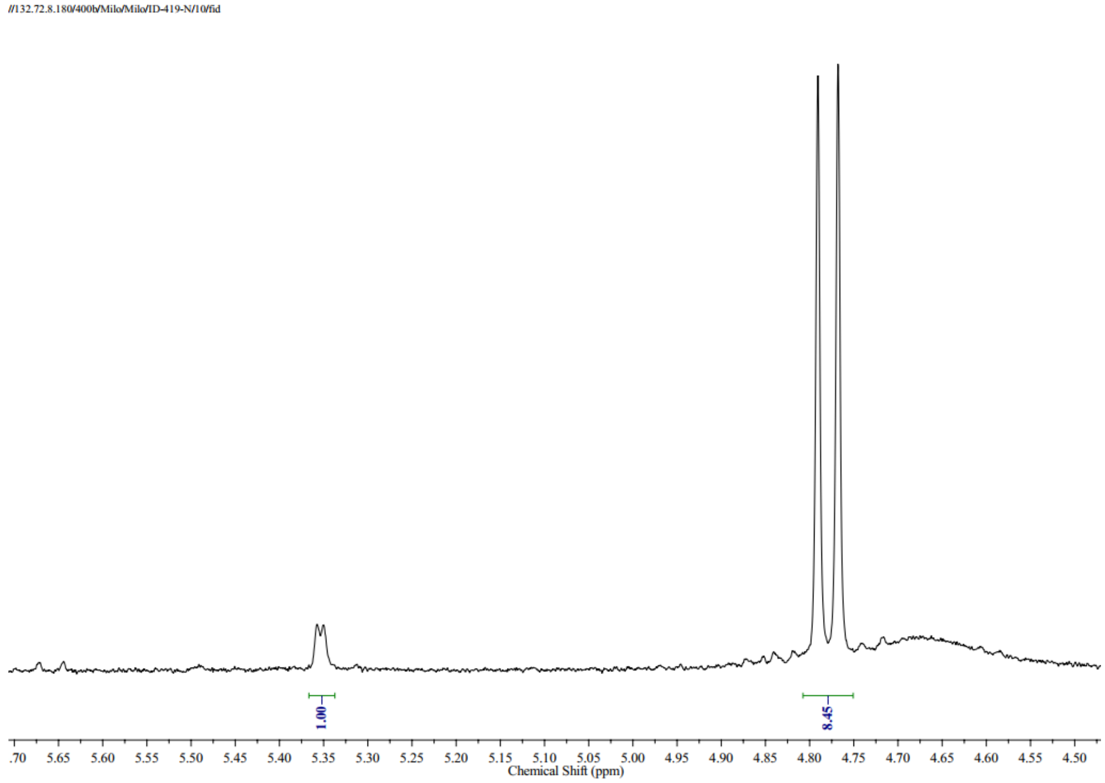

NMR traces for reaction with: **2-F-phenylboronic acid (400 MHz,** $CDCl_3$**)**

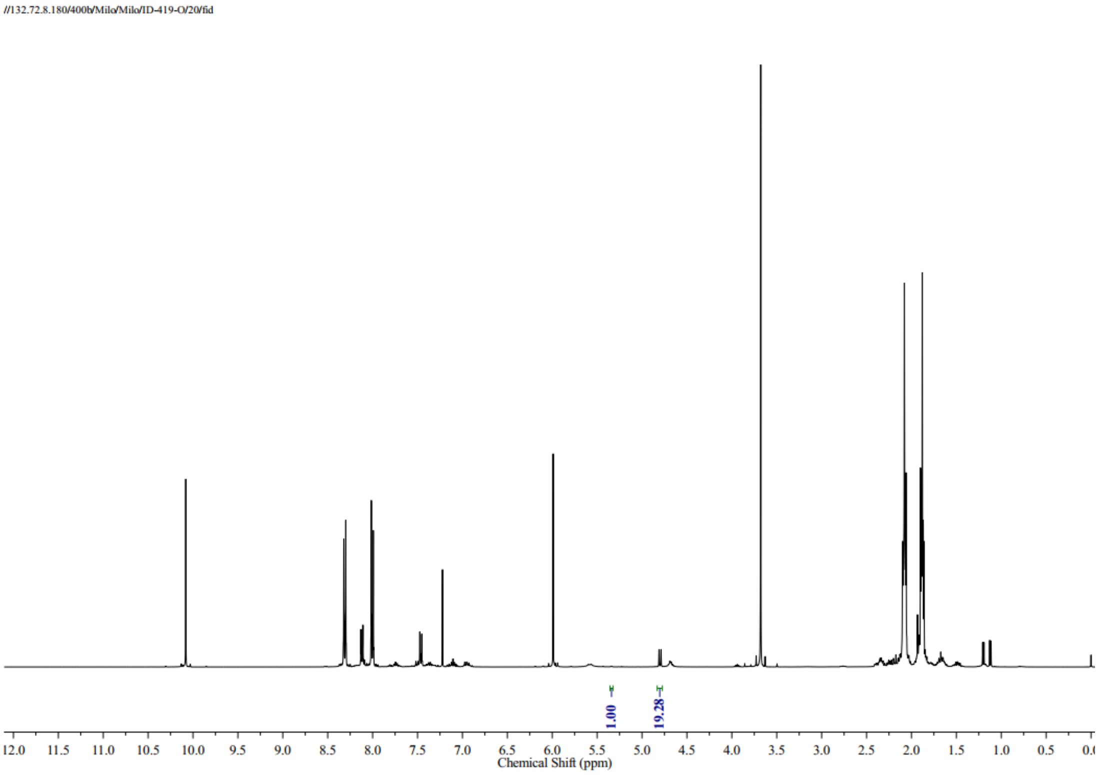

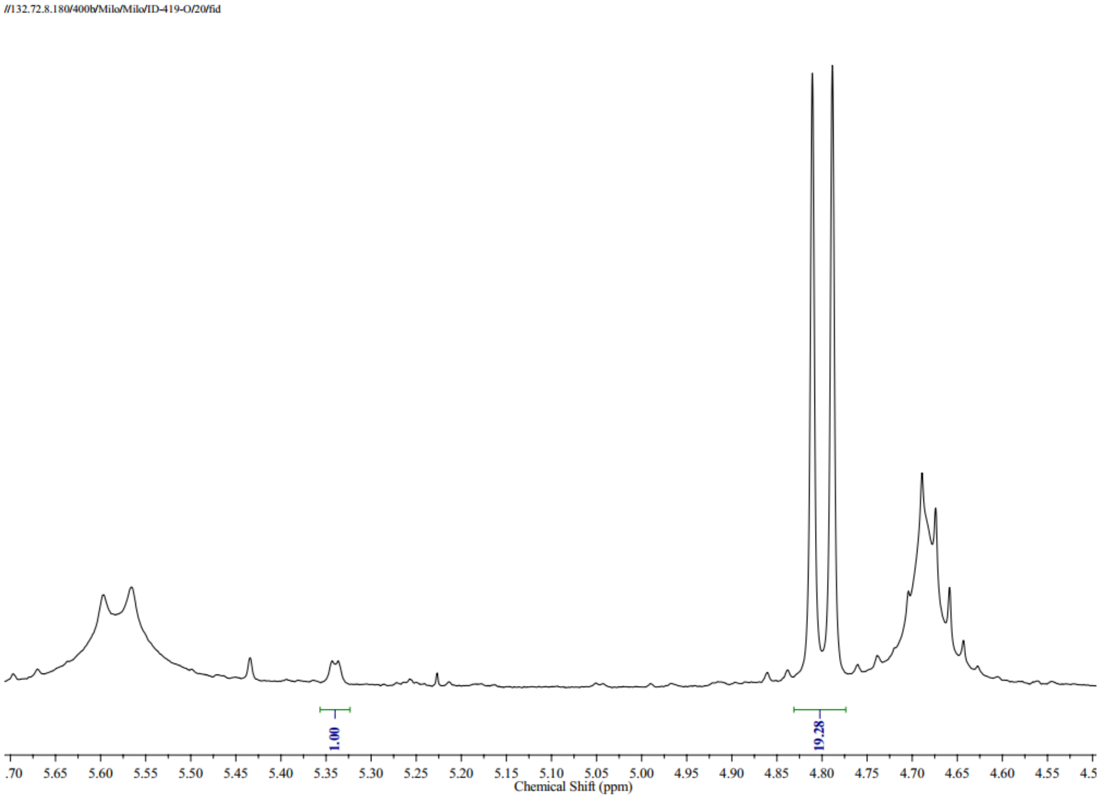

NMR traces for blank reaction **without boronic acid (400 MHz,** $CDCl_3$**)**

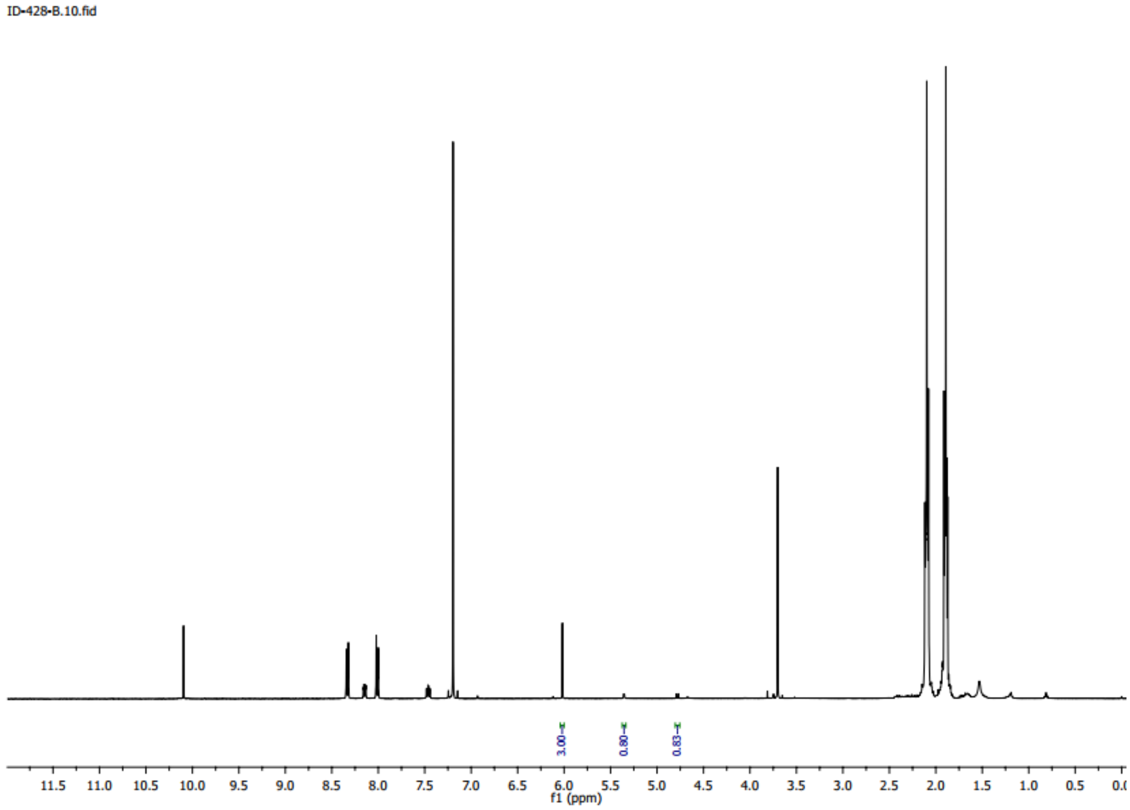

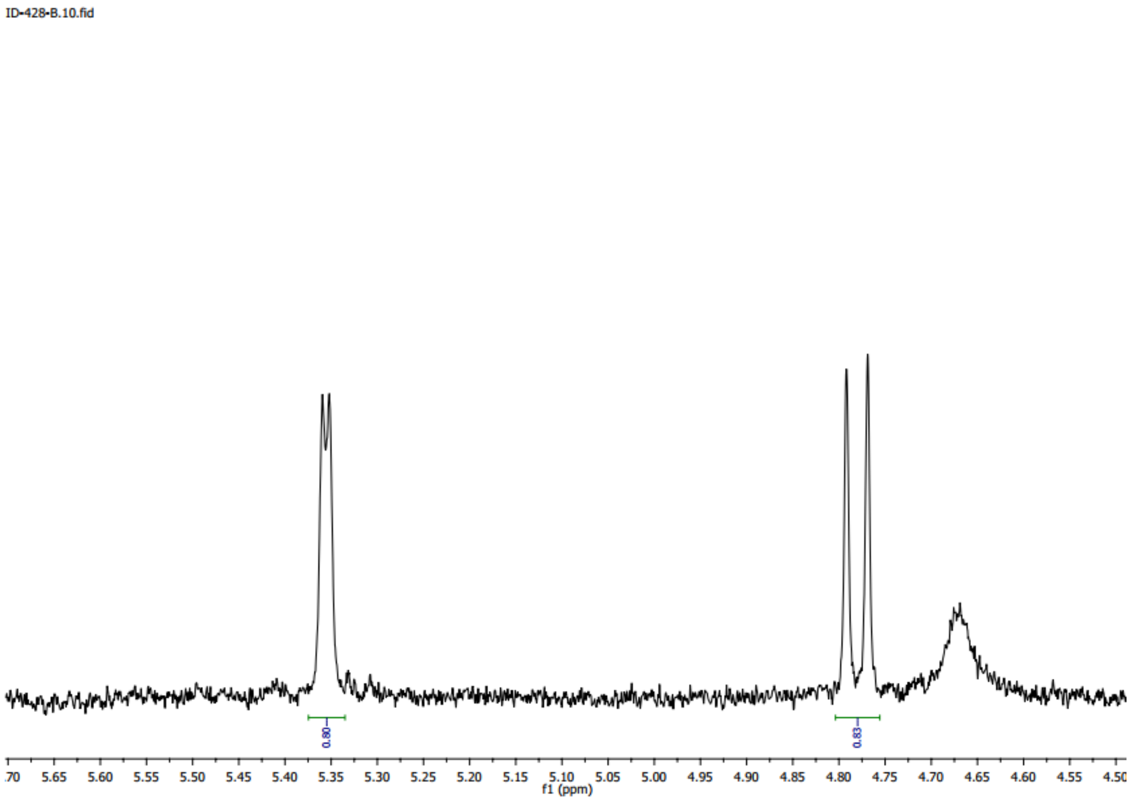

NMR traces for blank reaction **without boronic acid (duplicate) (400 MHz,** CDCl$_3$**)**

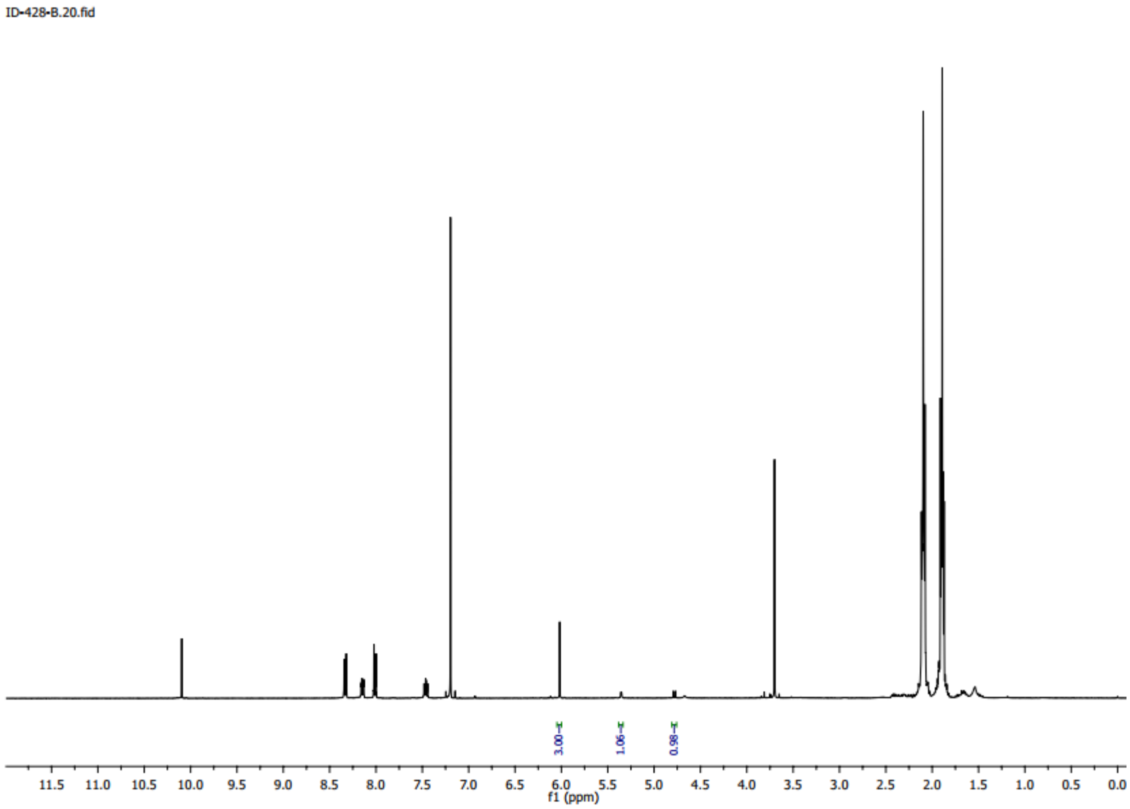

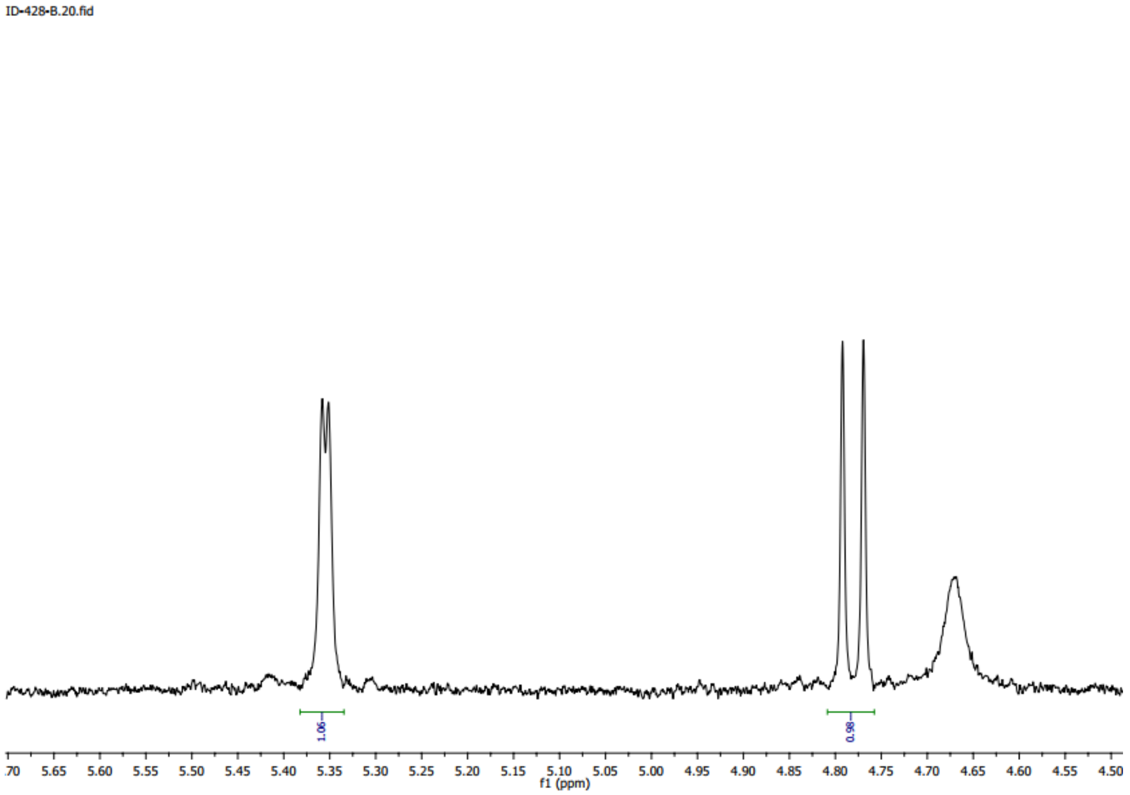

NMR traces for blank reaction **without water with 2-F-phenylboronic acid (400 MHz,** CDCl3**)**

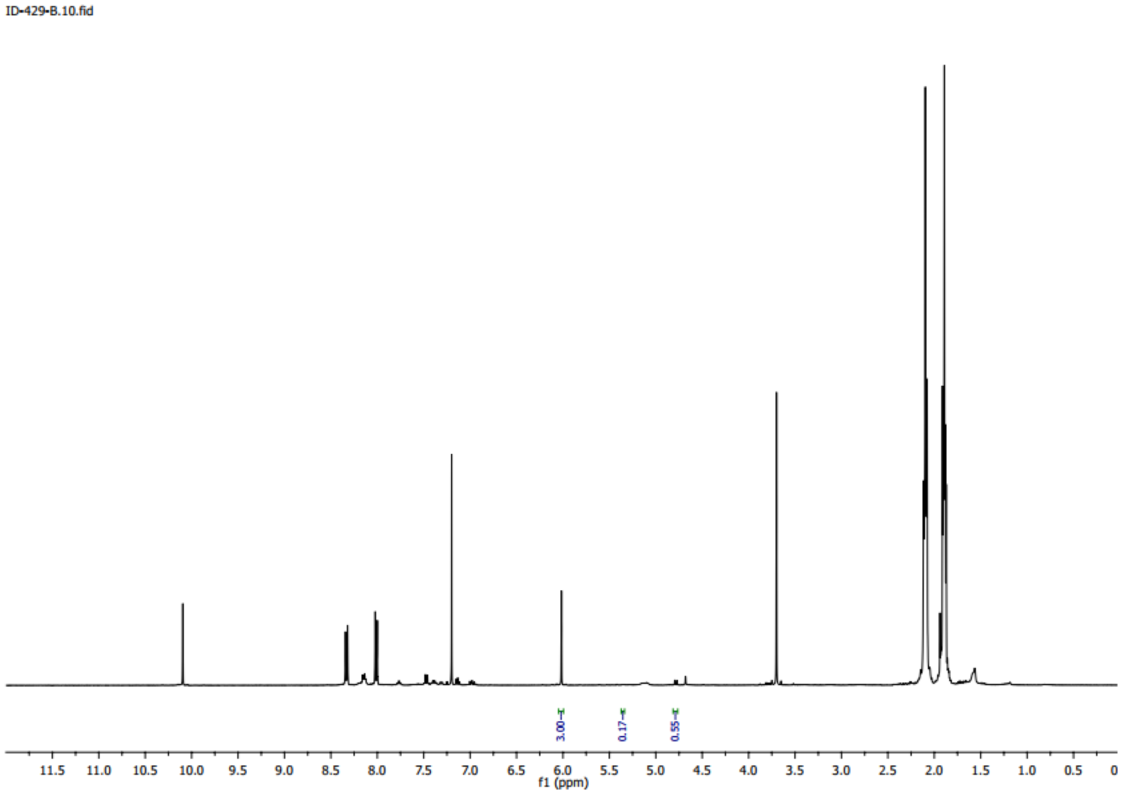

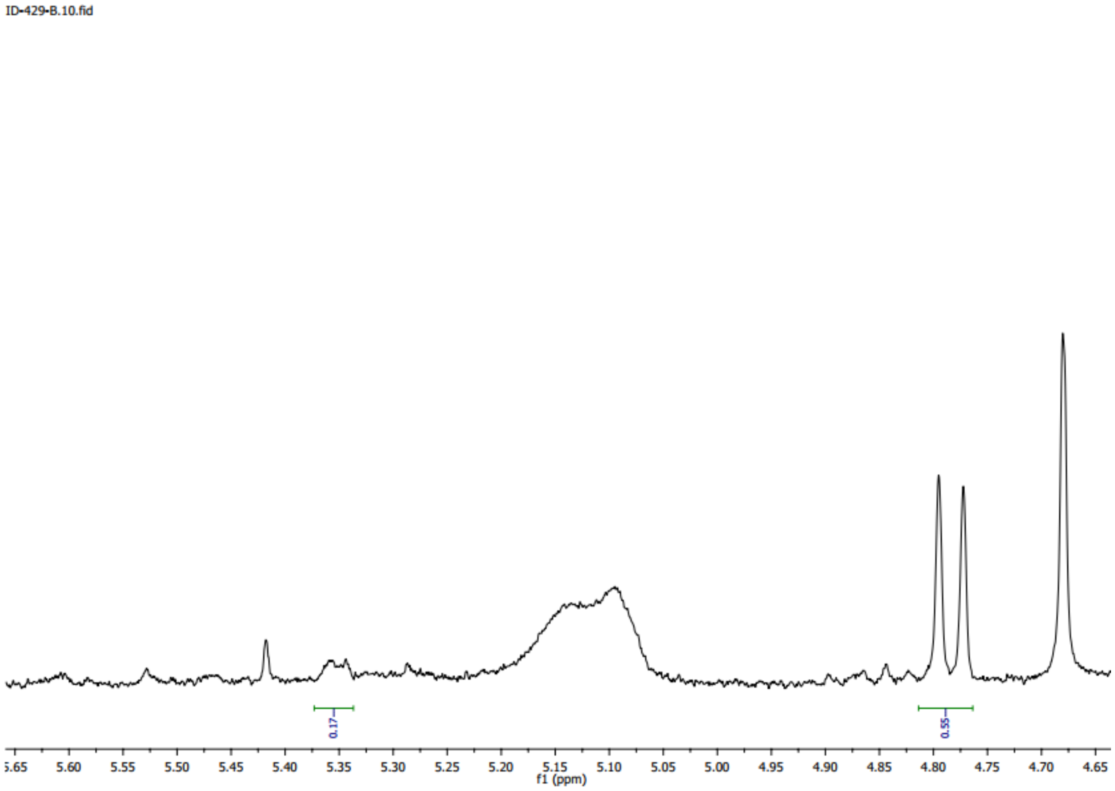

NMR traces for blank reaction **without water with 2-F-phenylboronic acid (duplicate) (400 MHz, $CDCl_3$)**

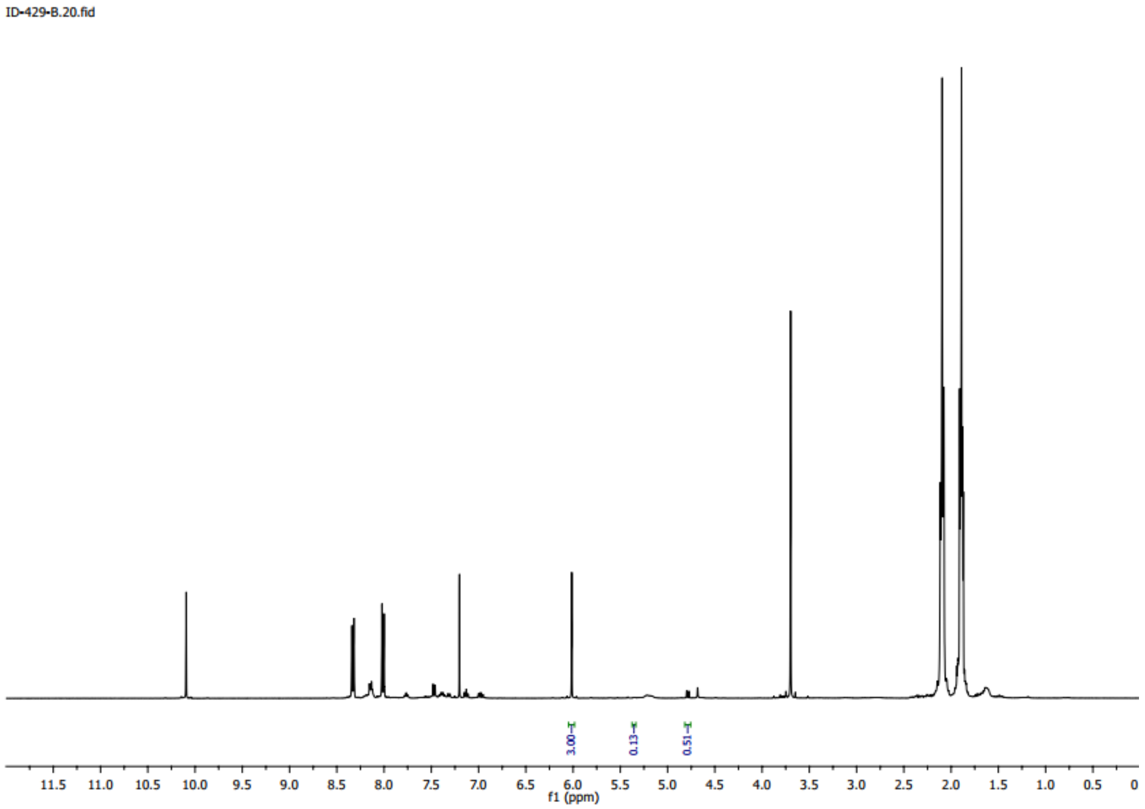

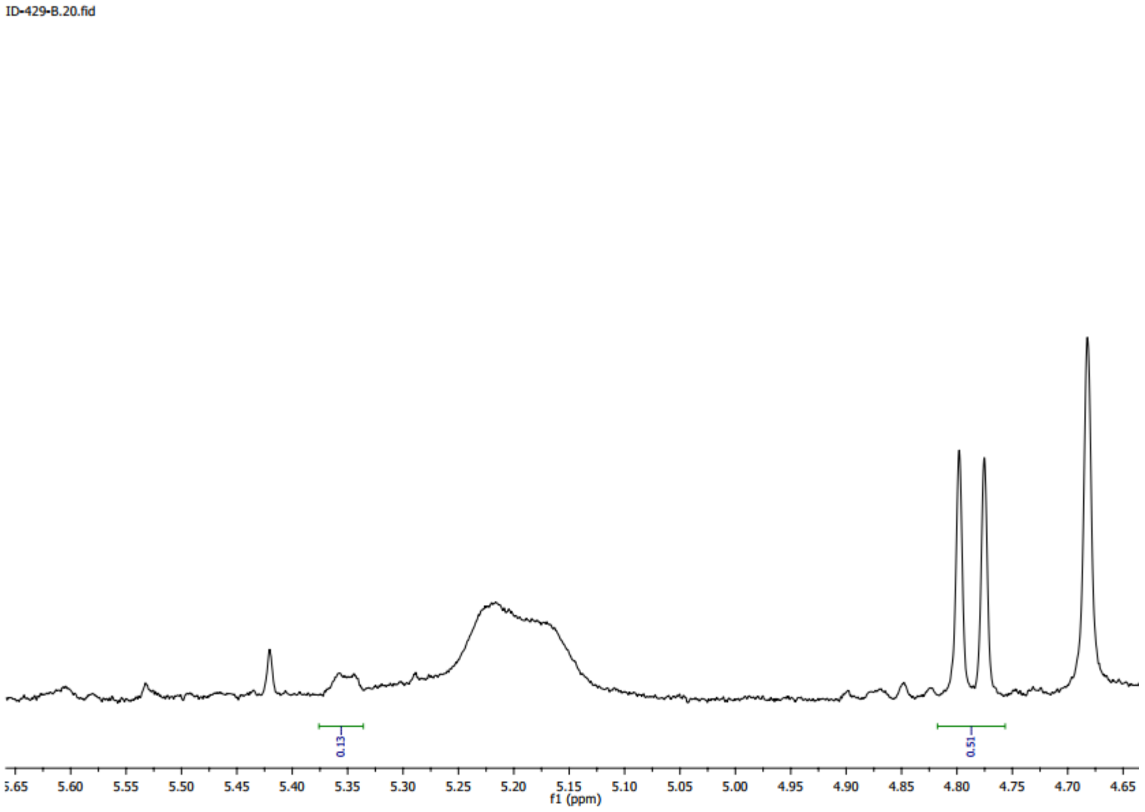

18.2. NMR Traces for Table S2. Boronic acid screening and blanks in chloroform

NMR traces for reaction with: **3-F-phenylboronic acid (400 MHz,** $CDCl_3$**)**

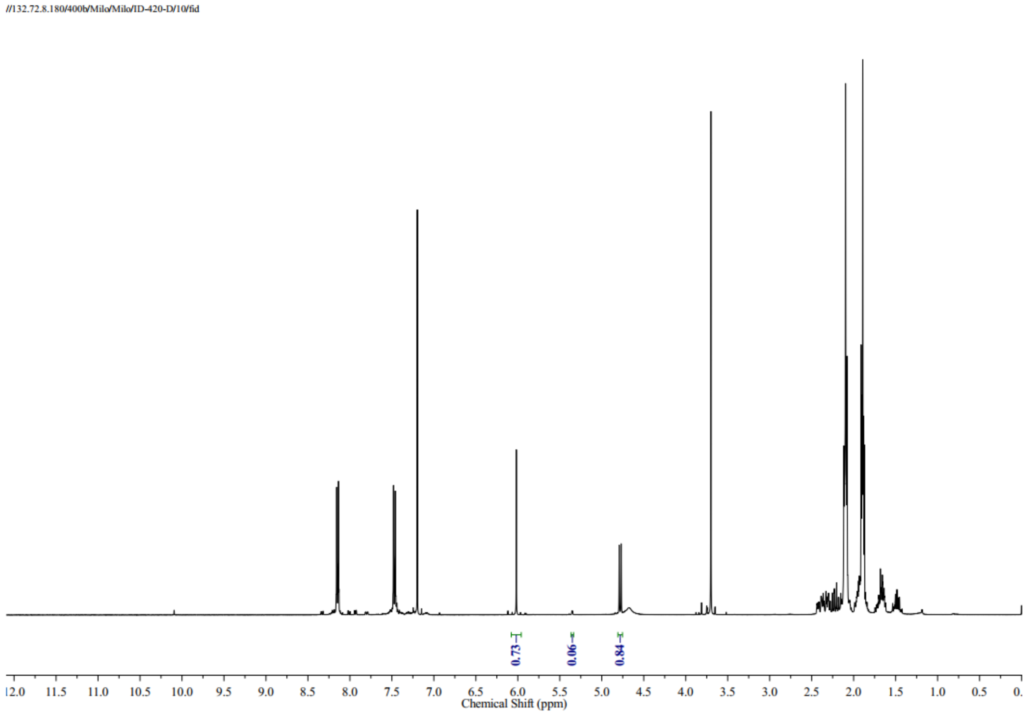

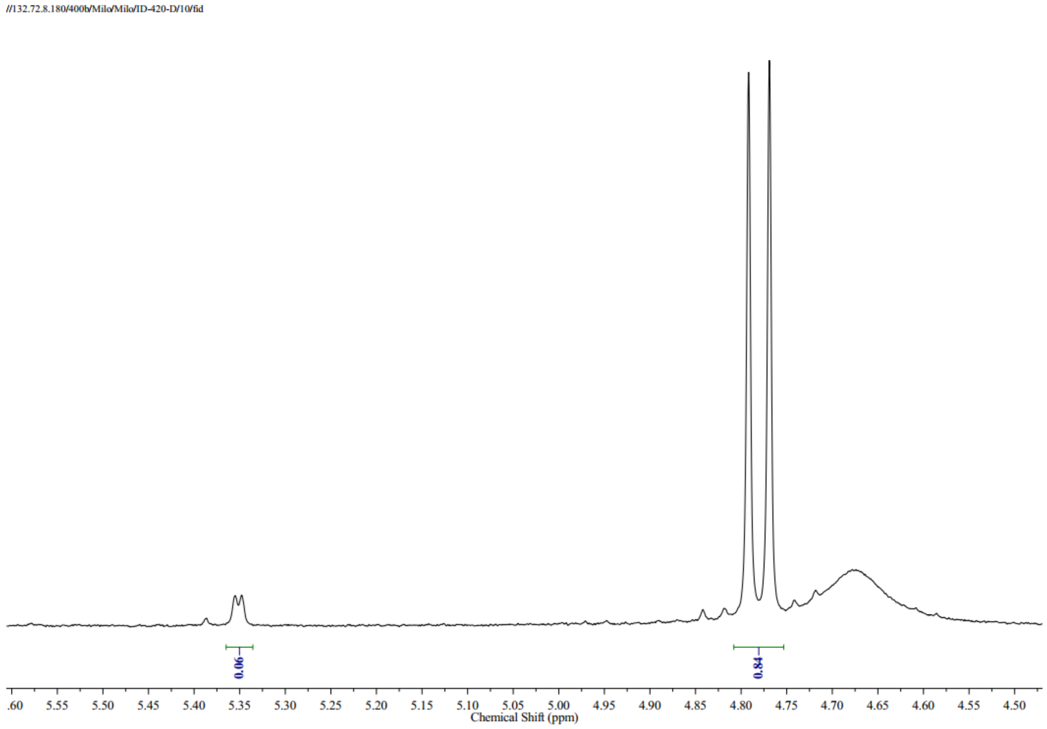

NMR traces for reaction with: **3-F-phenylboronic acid (duplicate) (400 MHz,** $CDCl_3$**)**

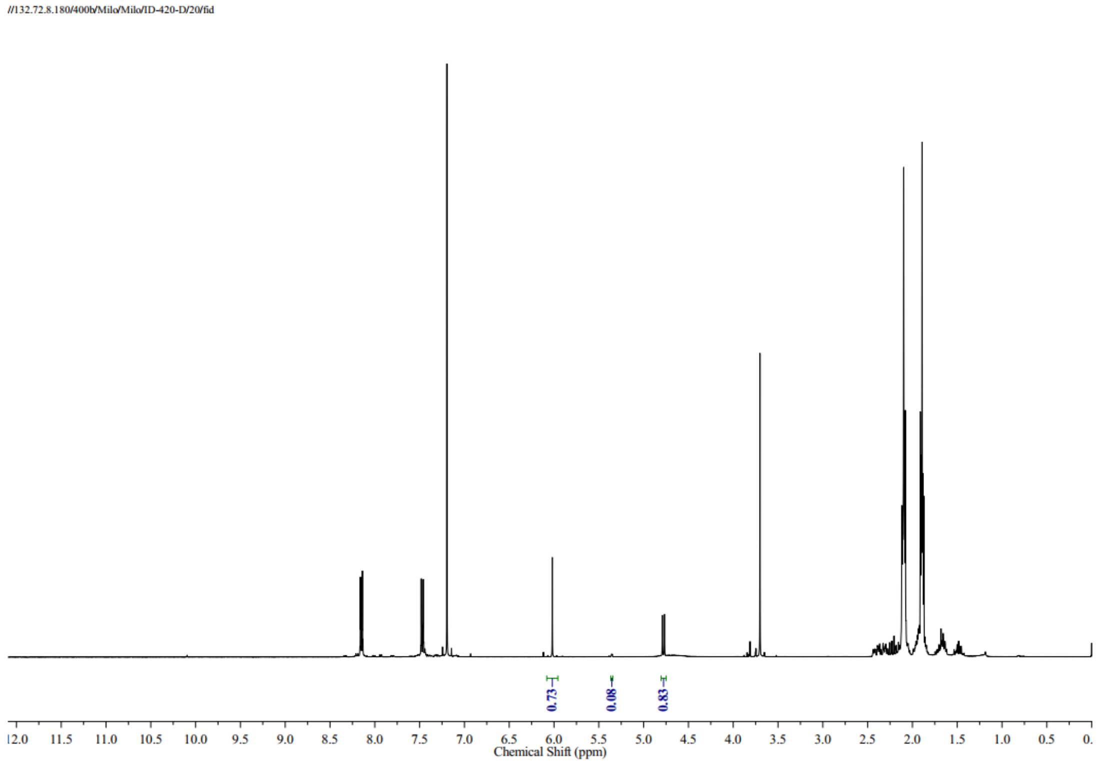

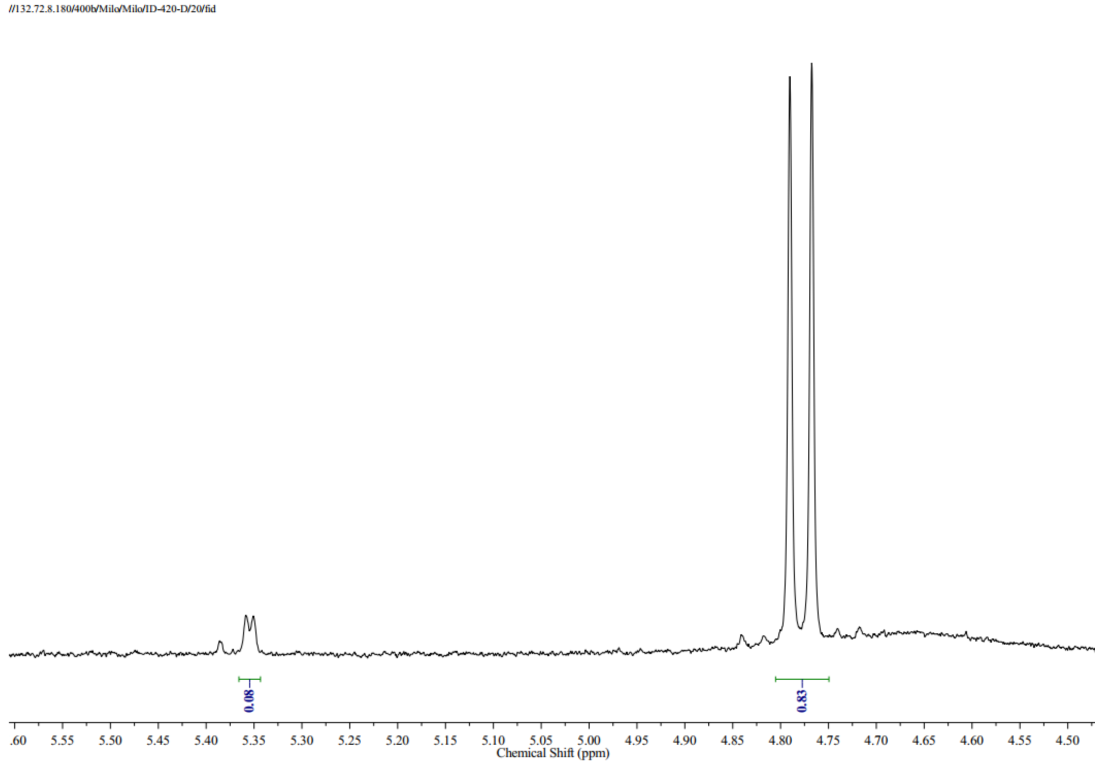

NMR traces for reaction with: **3,5-F-phenylboronic acid (400 MHz,** $CDCl_3$**)**

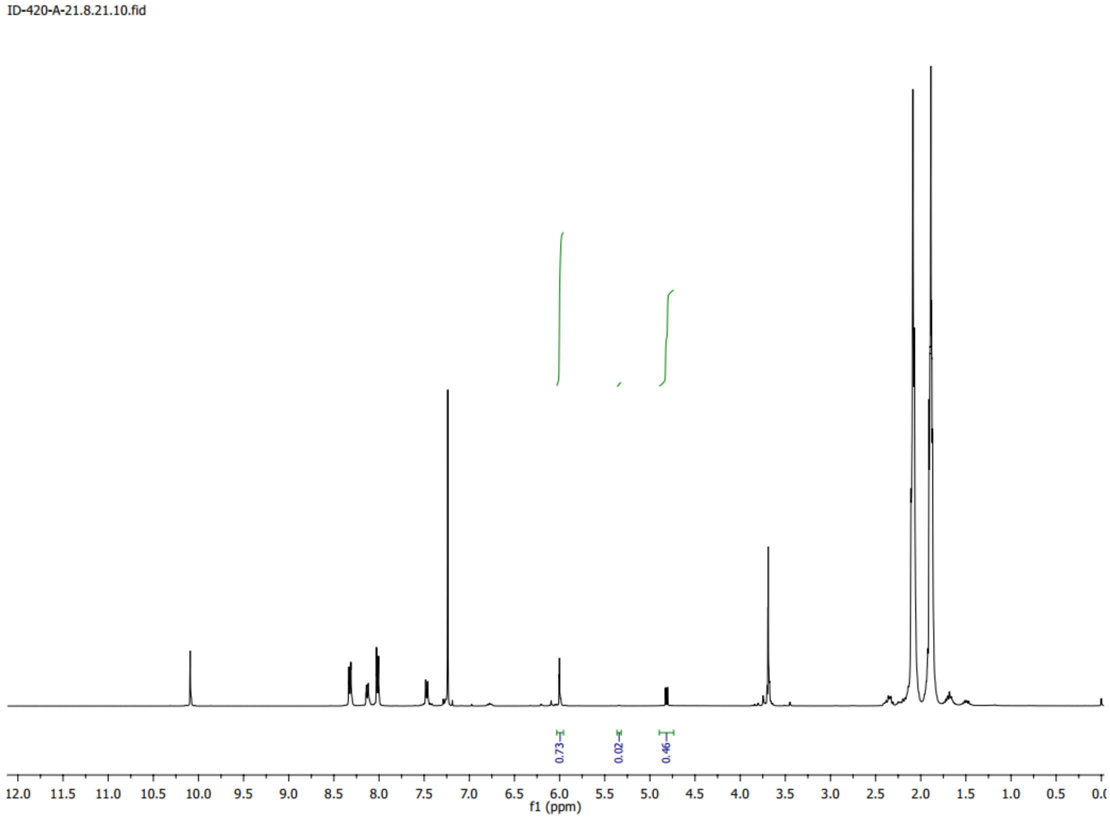

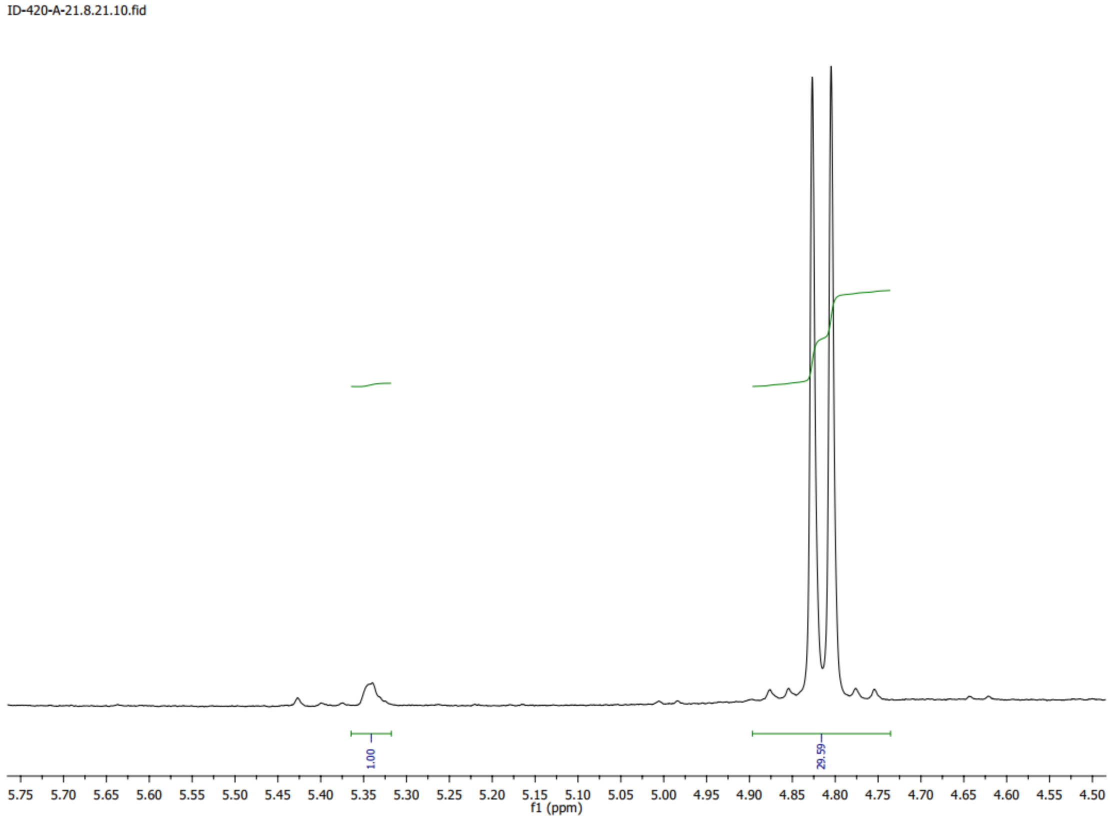

NMR traces for reaction with: **3,5-F-phenylboronic acid (duplicate) (400 MHz,** $CDCl_3$**)**

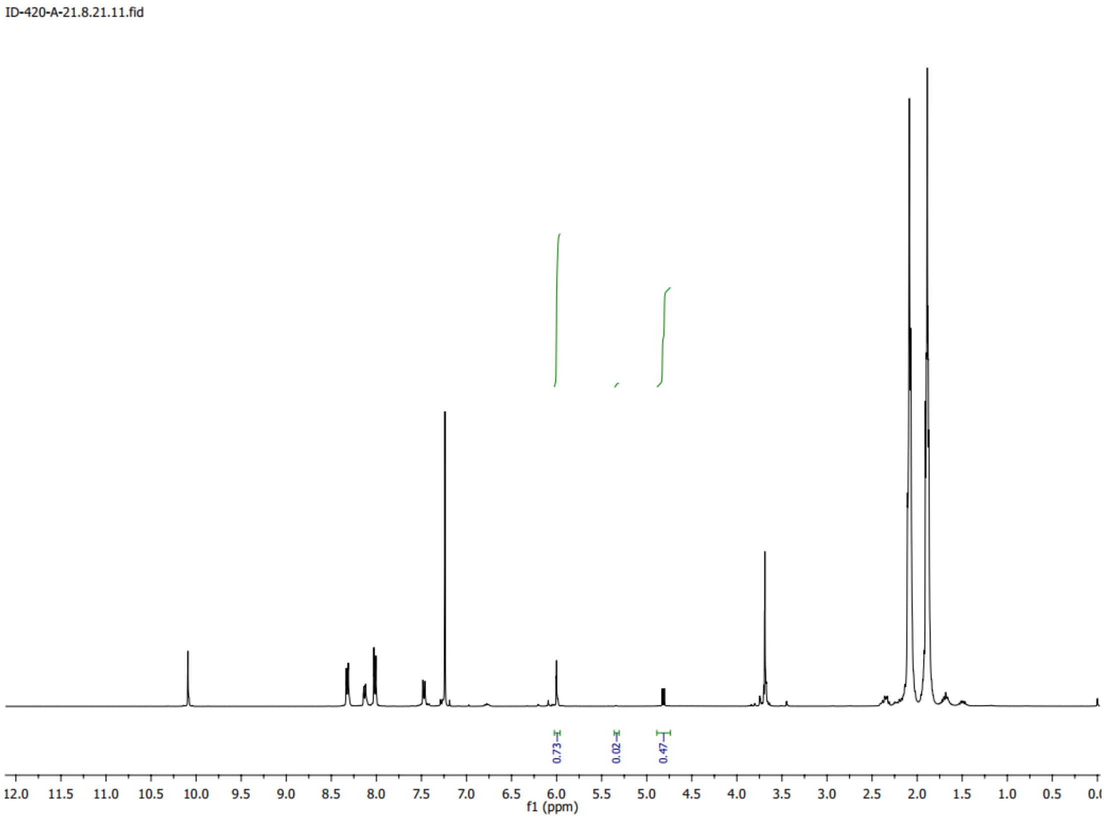

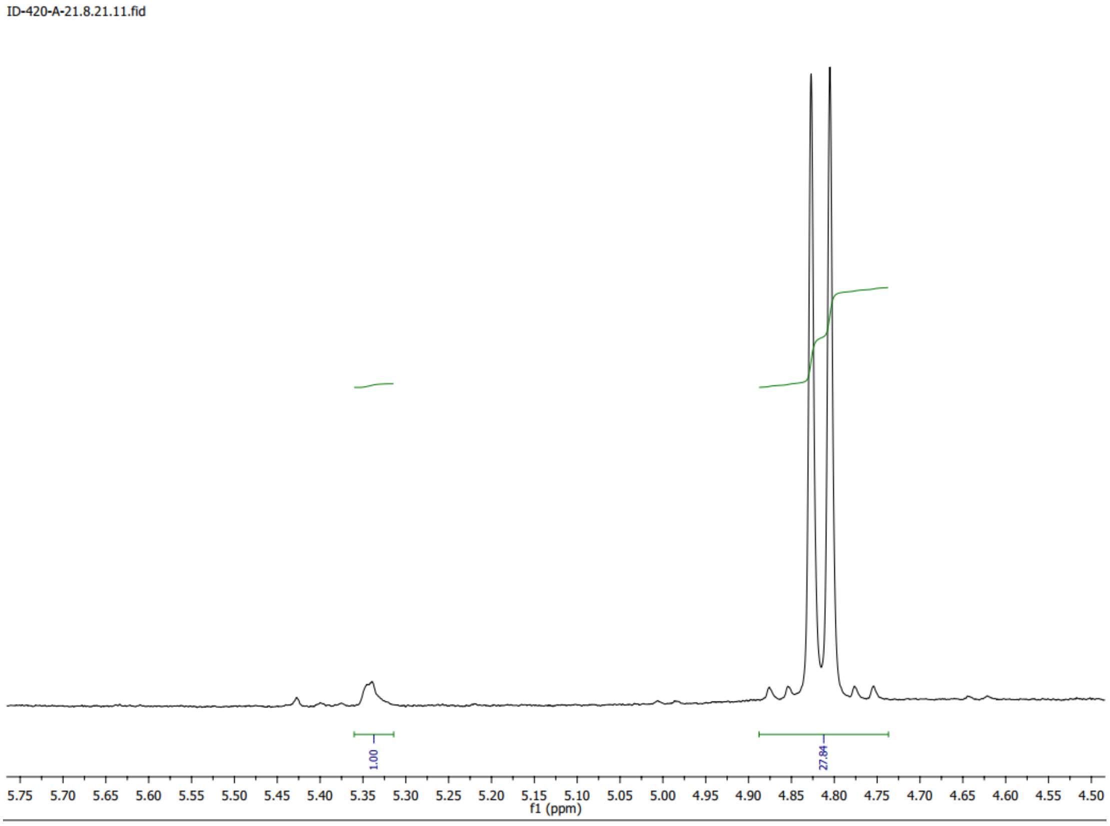

NMR traces for reaction with: **4-tBu-phenylboronic acid (400 MHz,** $CDCl_3$**)**

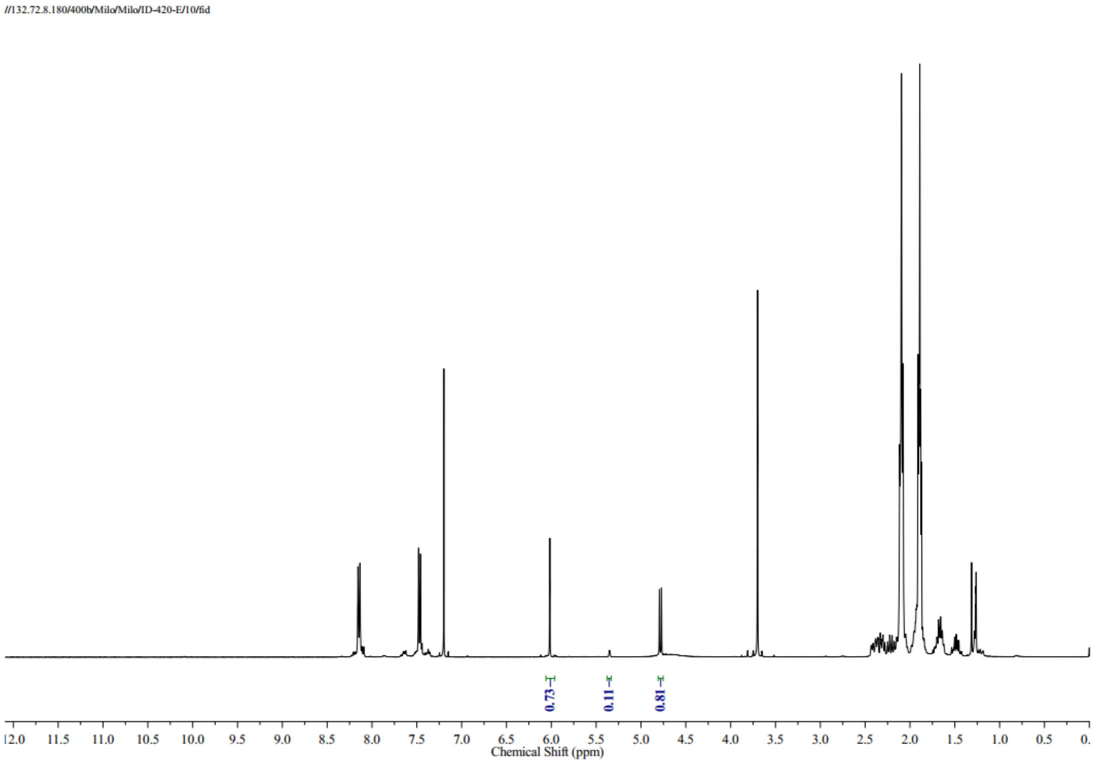

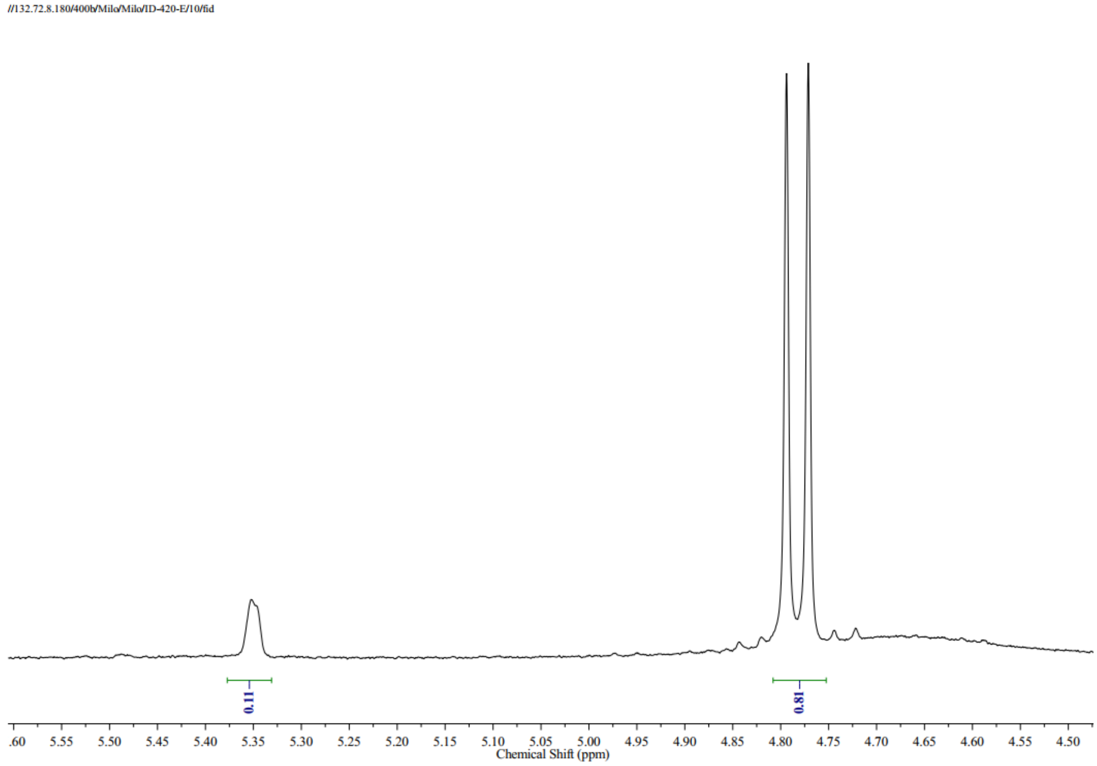

NMR traces for reaction with: **4-tBu-phenylboronic acid (duplicate) (400 MHz,** CDCl$_3$**)**

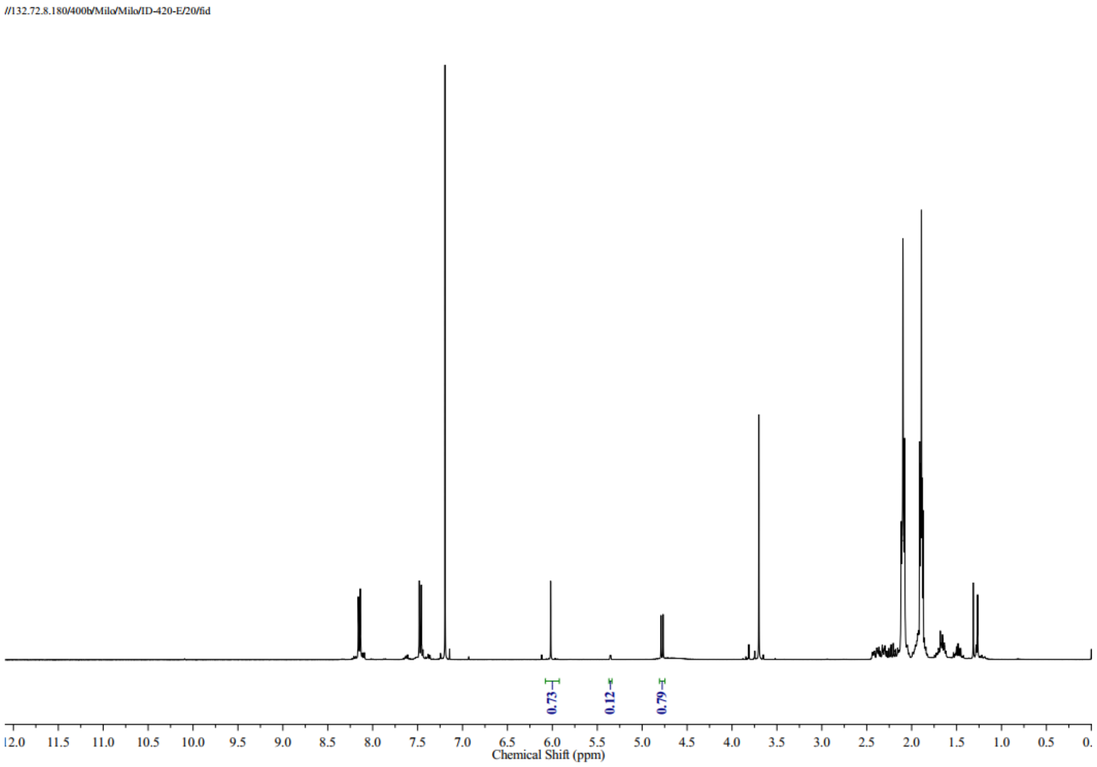

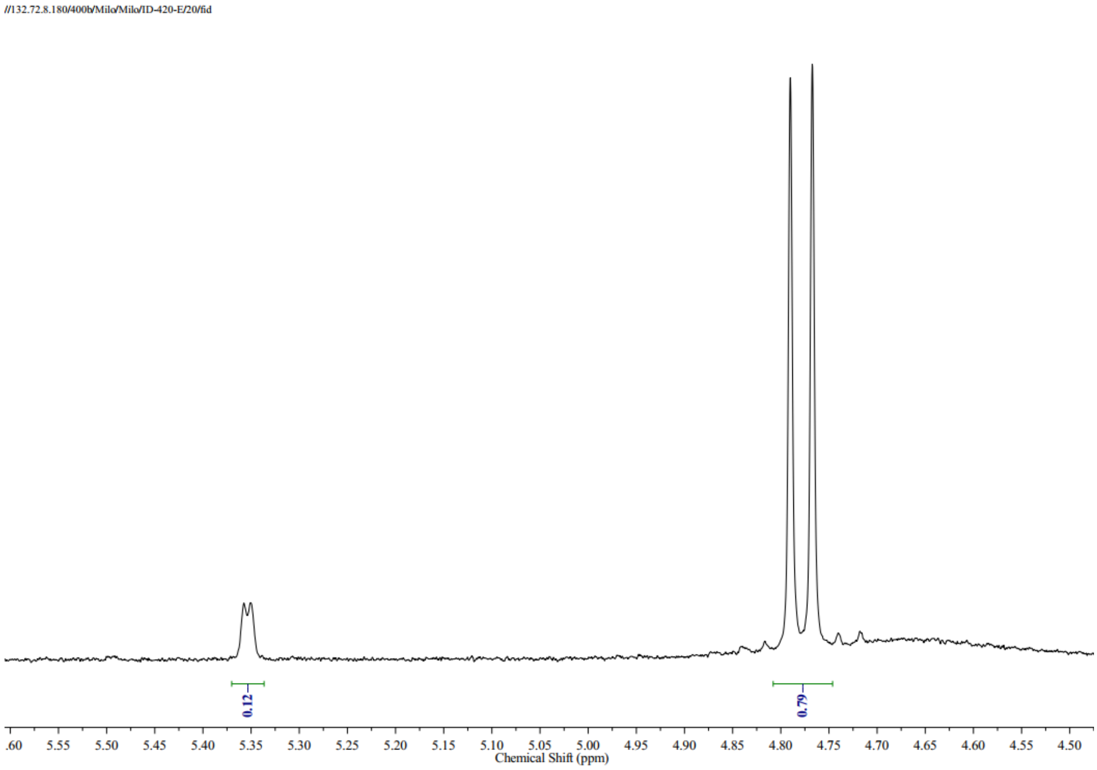

NMR traces for reaction with: **2,4-Me-phenylboronic acid (400 MHz,** $CDCl_3$**)**

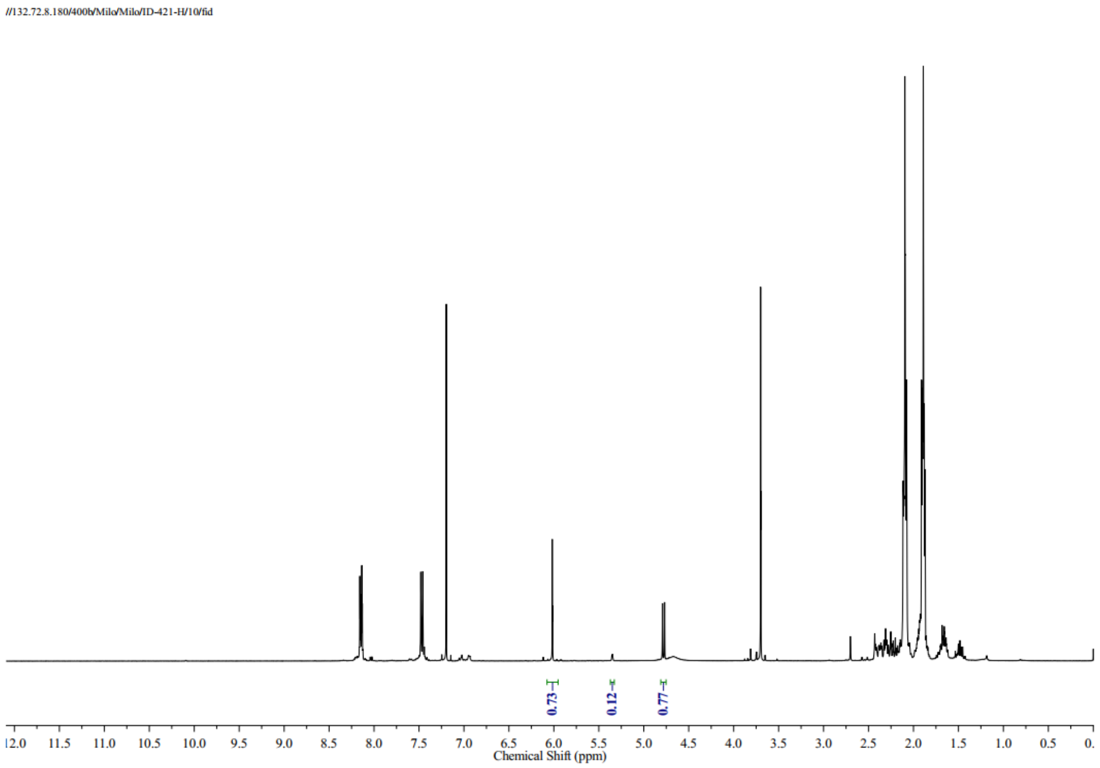

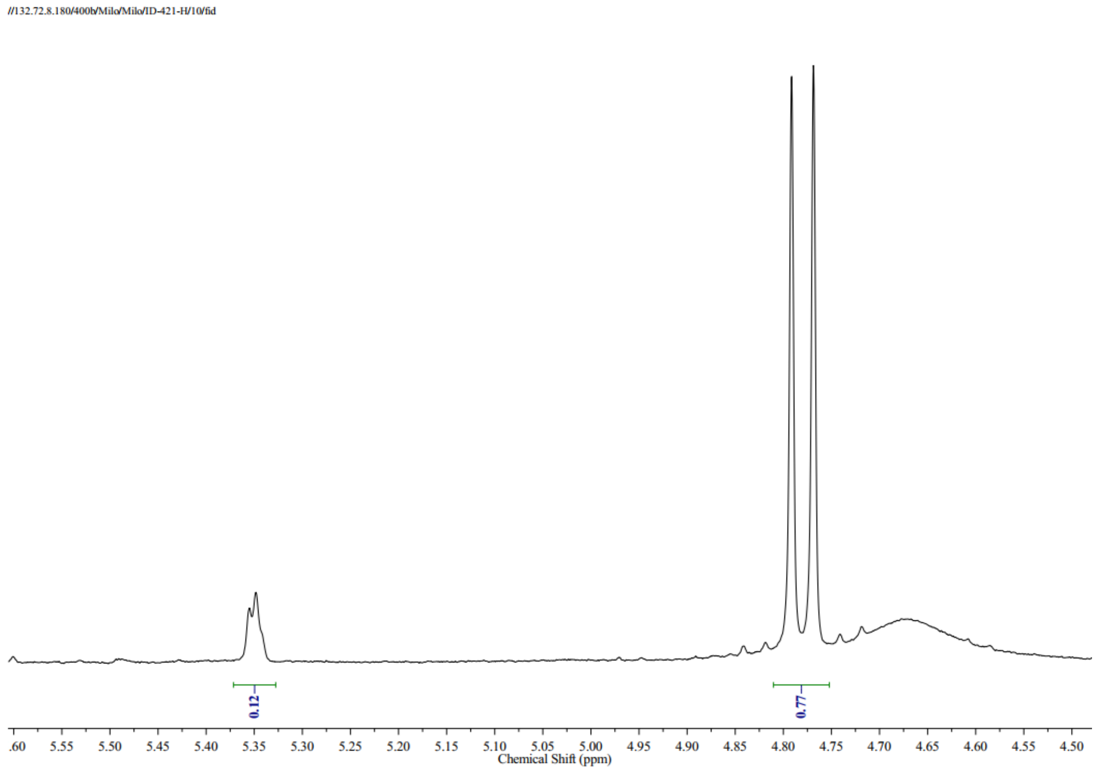

NMR traces for reaction with: **2,4-Me-phenylboronic acid (duplicate) (400 MHz,** CDCl$_3$**)**

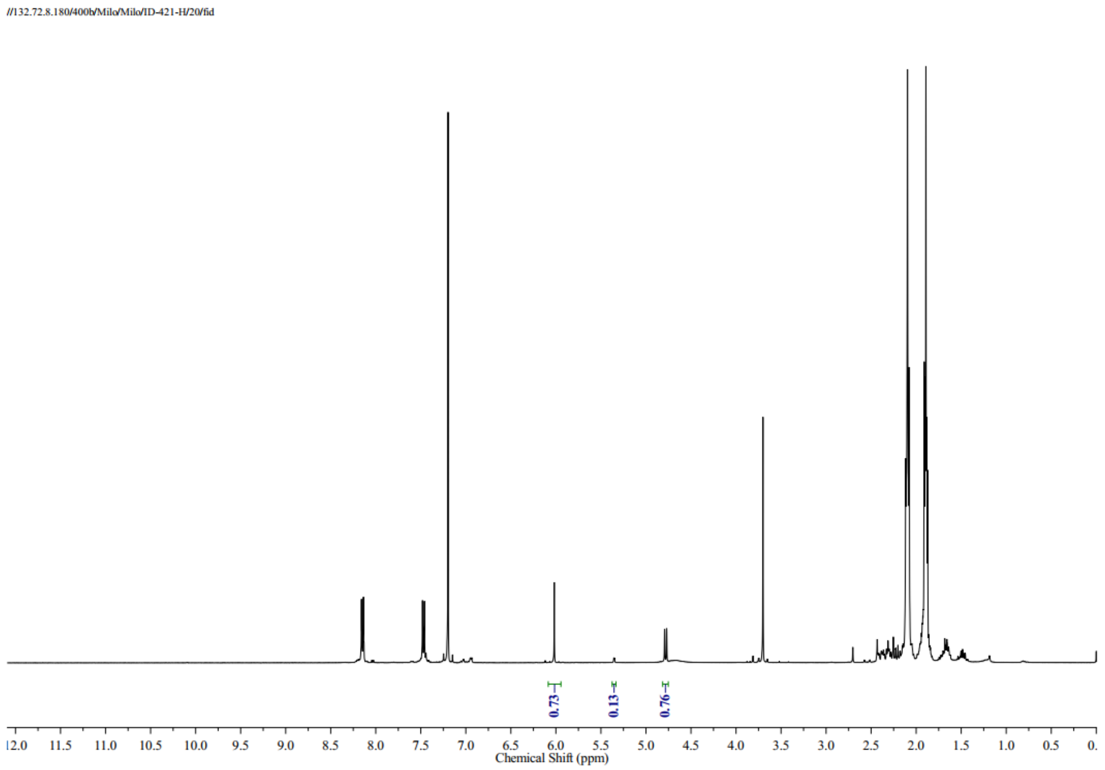

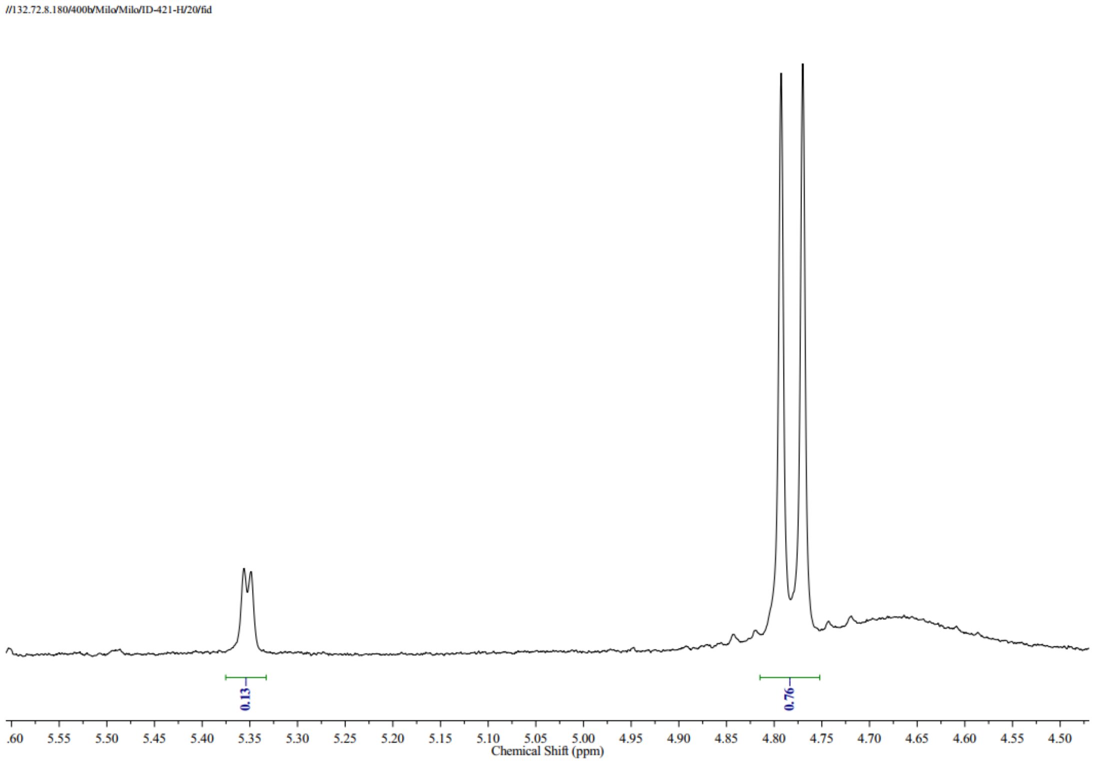

NMR traces for reaction with: **3,5-OMe-phenylboronic acid (400 MHz,** $CDCl_3$**)**

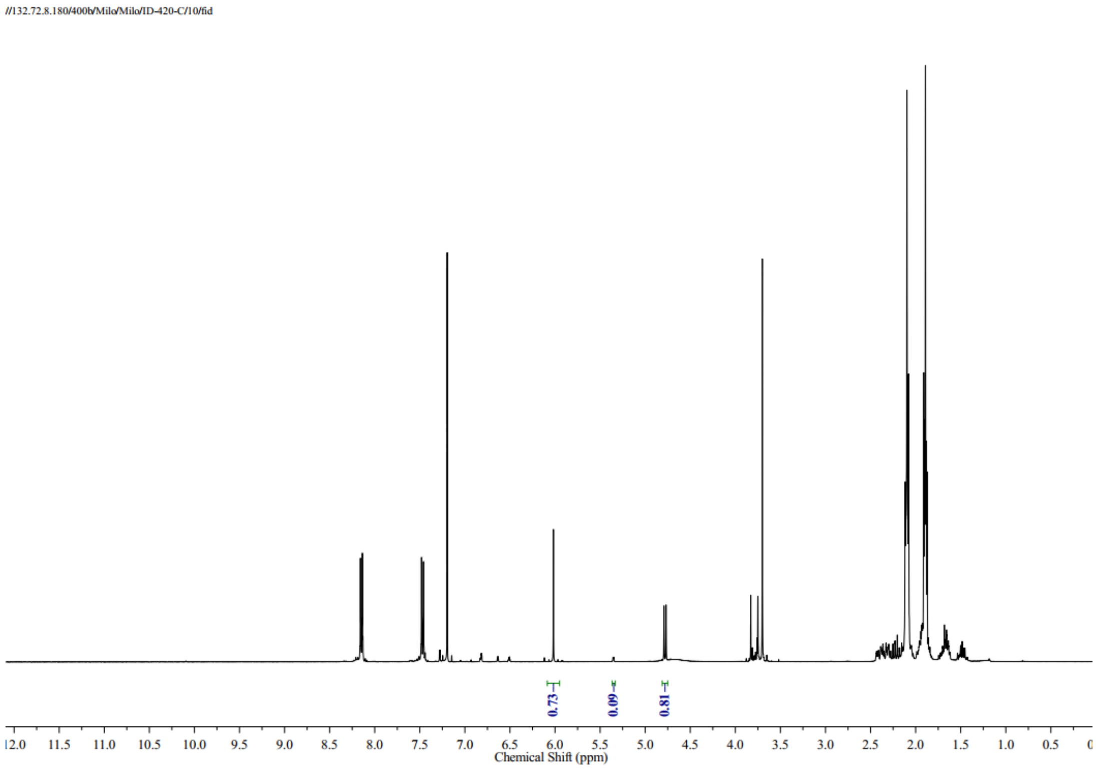

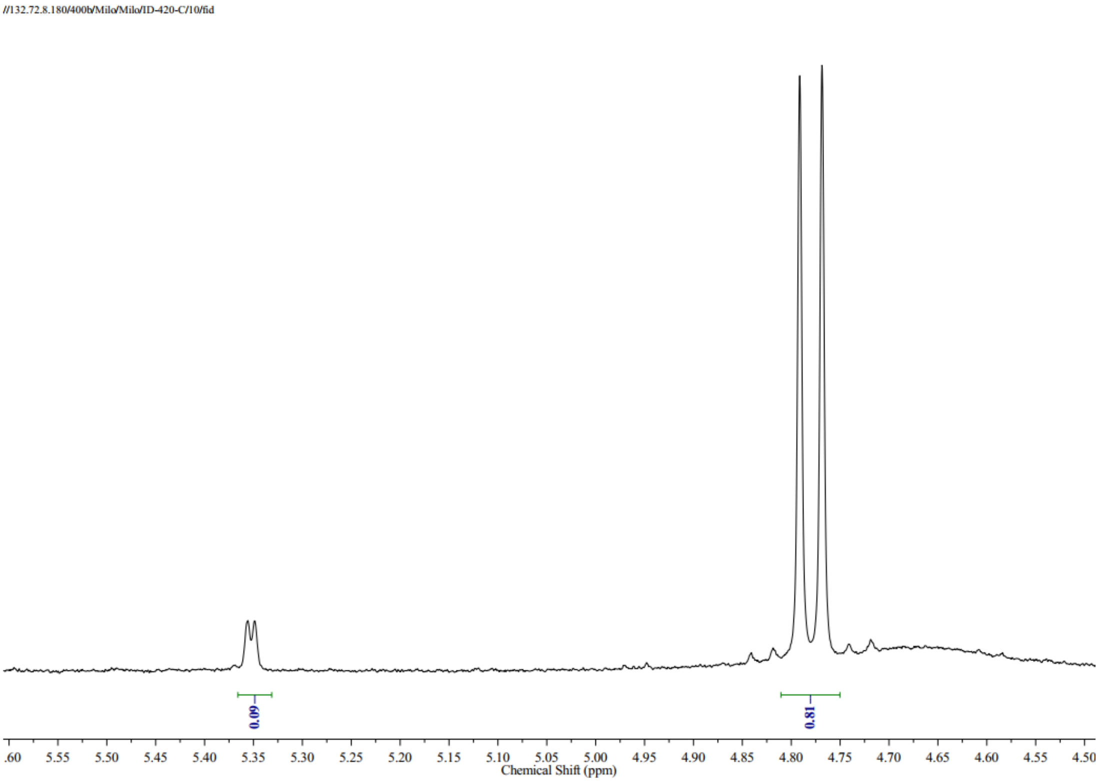

NMR traces for reaction with: **3,5-OMe-phenylboronic acid (duplicate) (400 MHz,** $CDCl_3$**)**

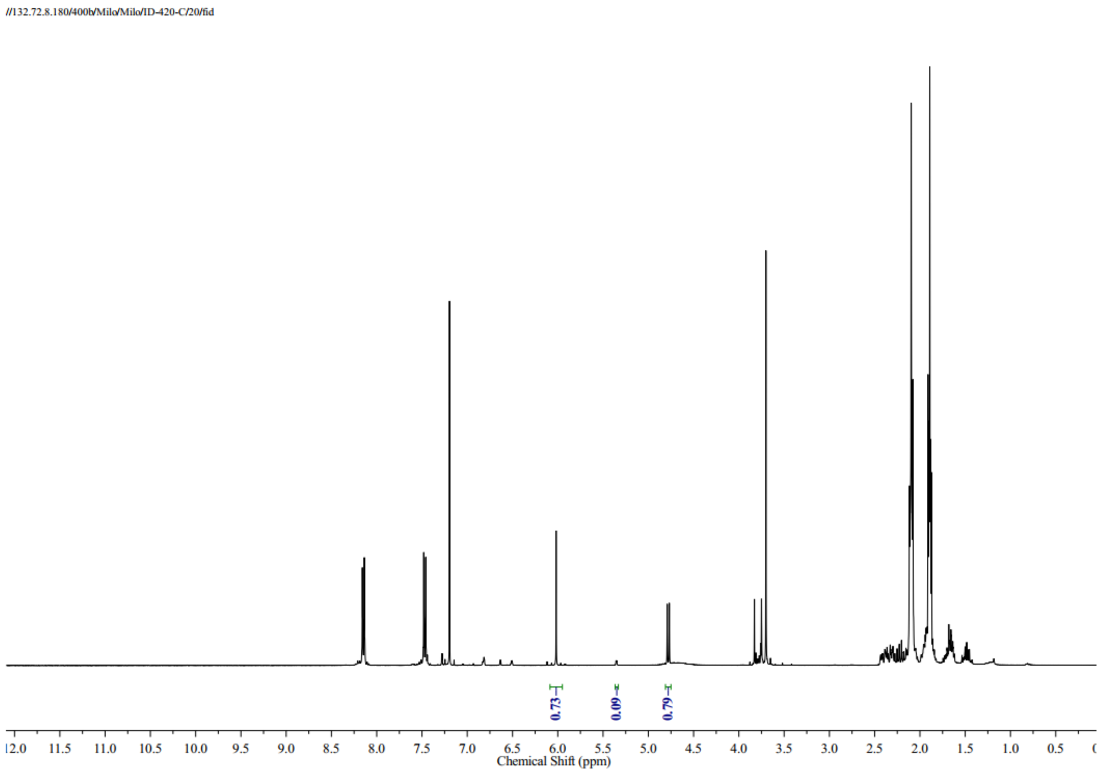

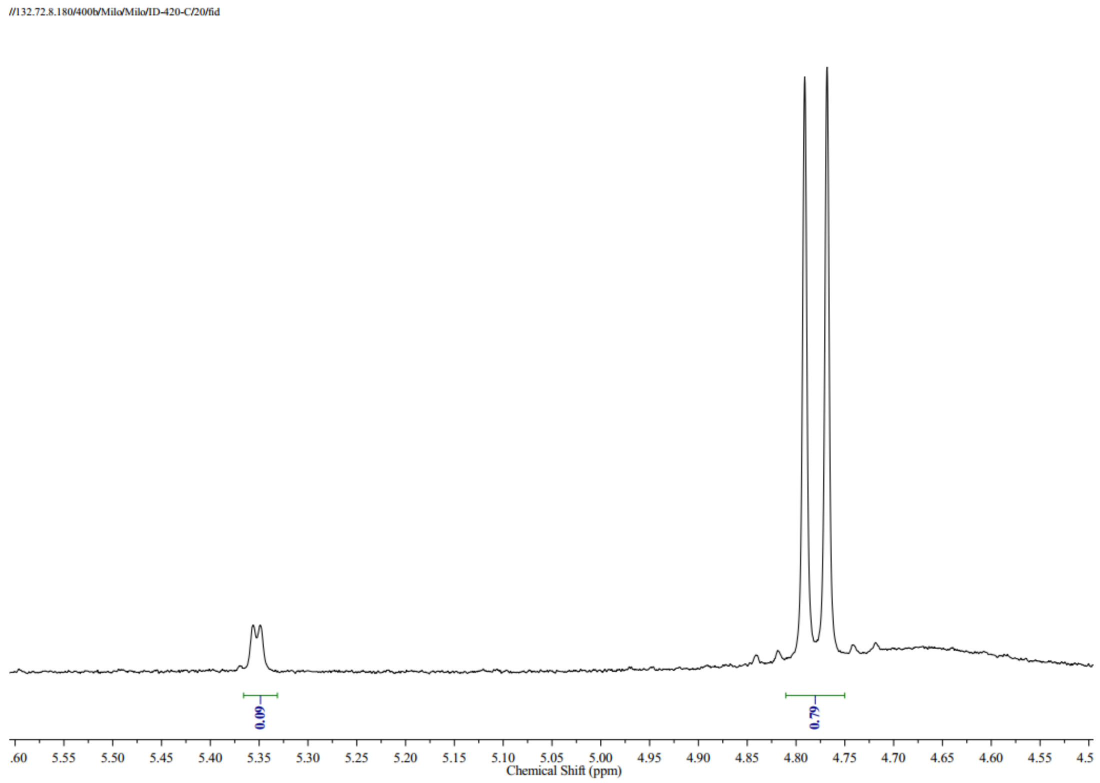

NMR traces for reaction with: **4-Me-phenylboronic acid (400 MHz,** CDCl$_3$**)**

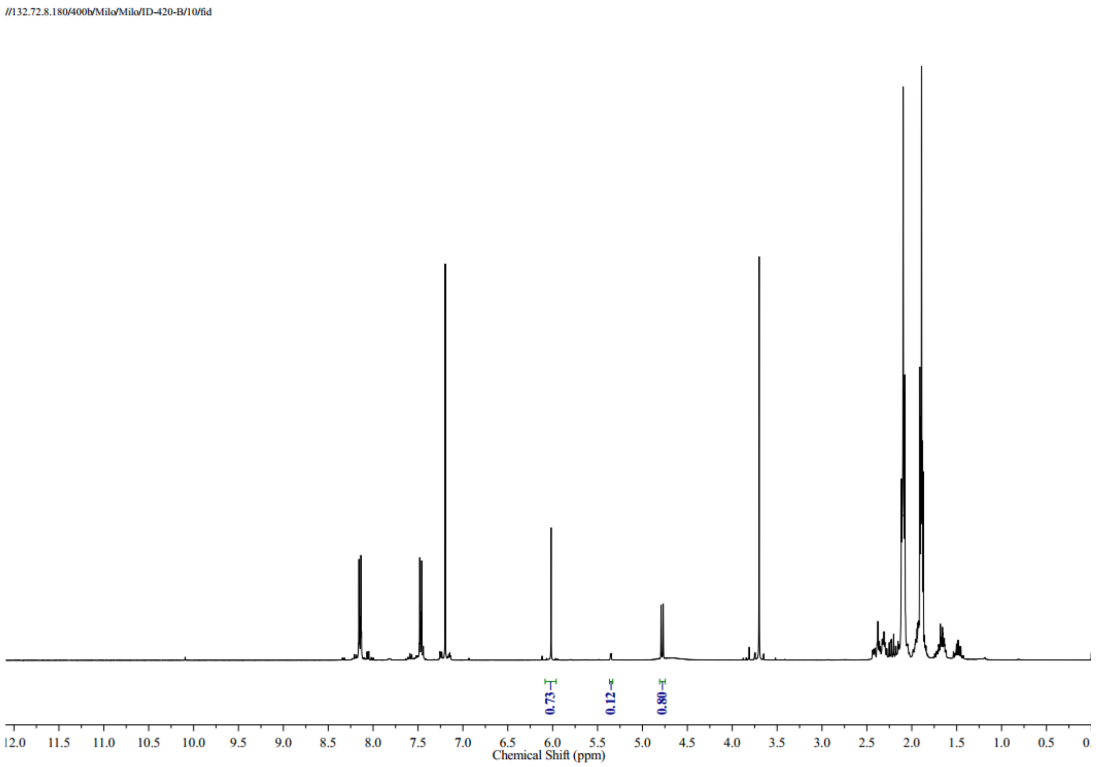

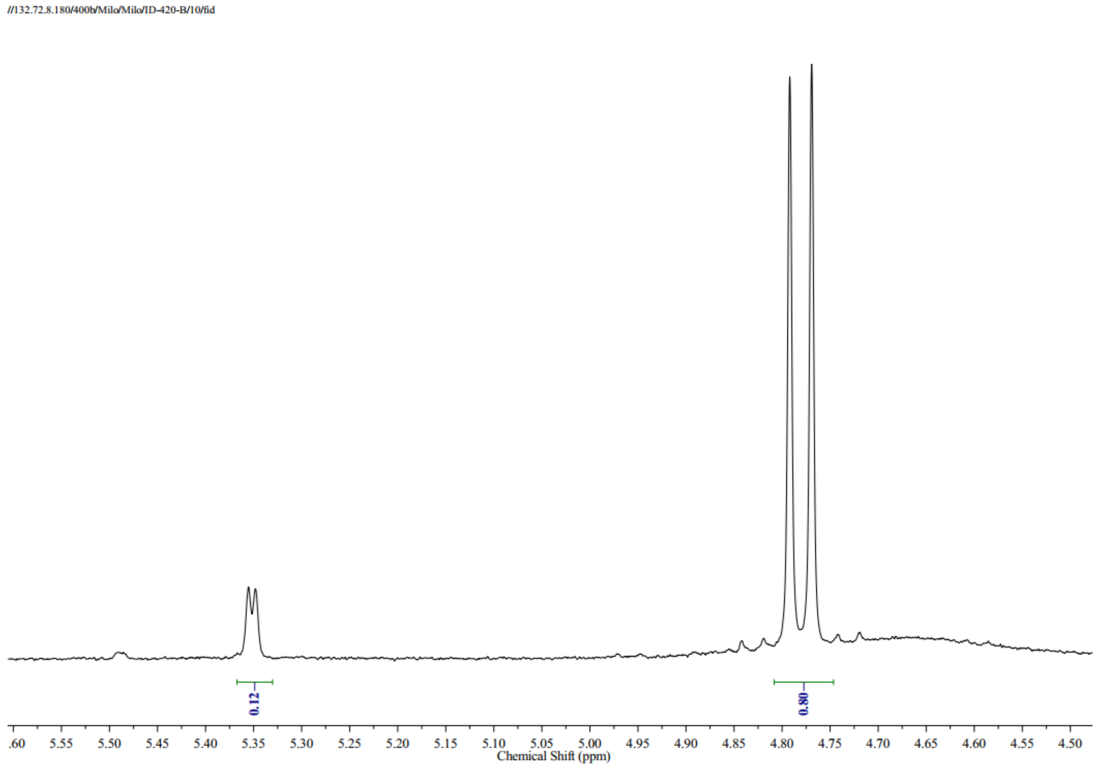

NMR traces for reaction with: **4-Me-phenylboronic acid (duplicate) (400 MHz,** CDCl$_3$**)**

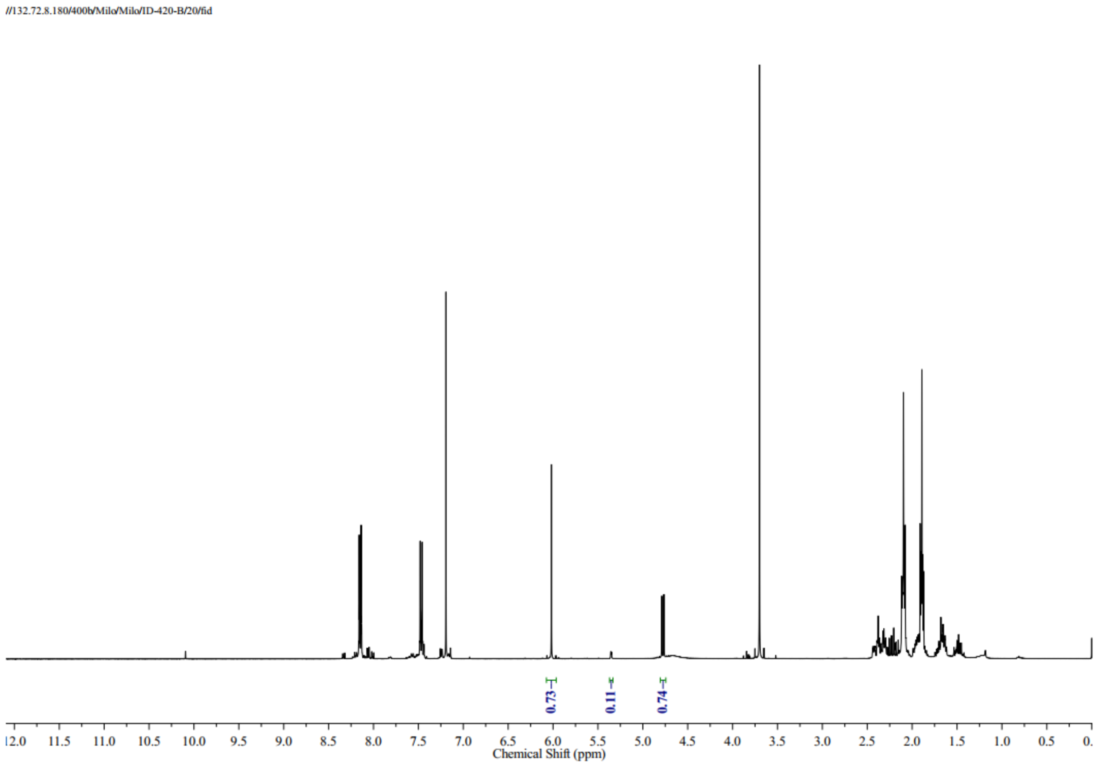

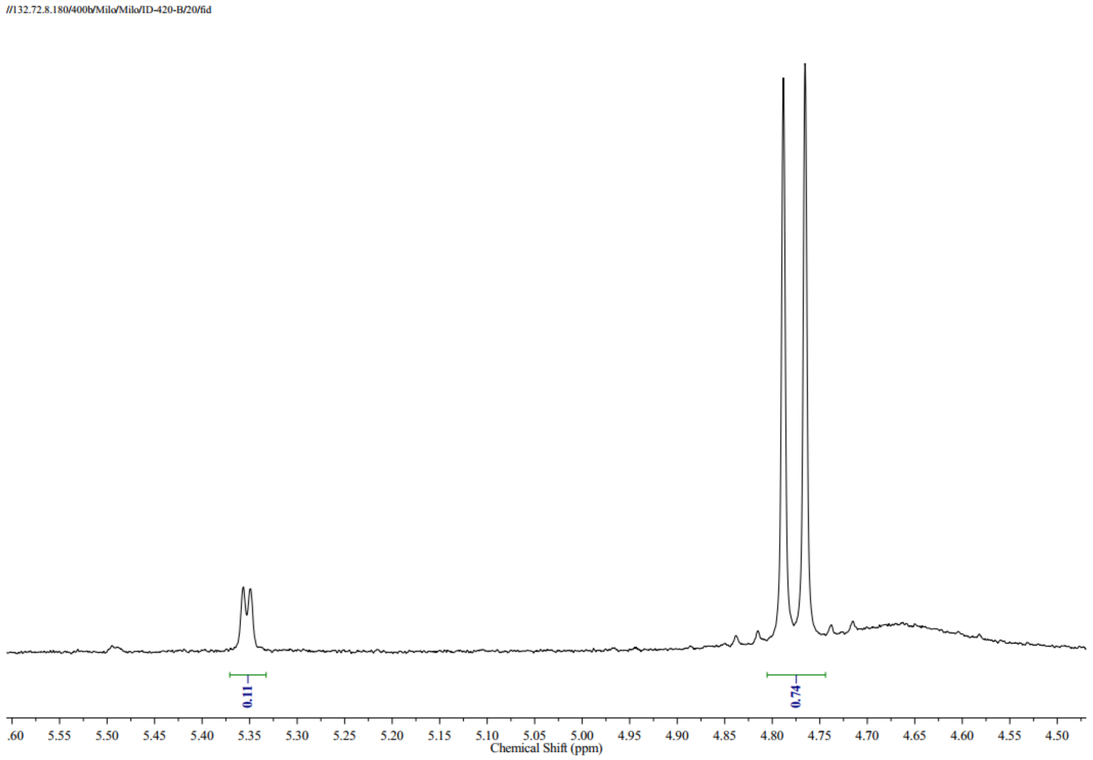

NMR traces for reaction with: **3-CF_3-phenylboronic acid (400 MHz,** $CDCl_3$**)**

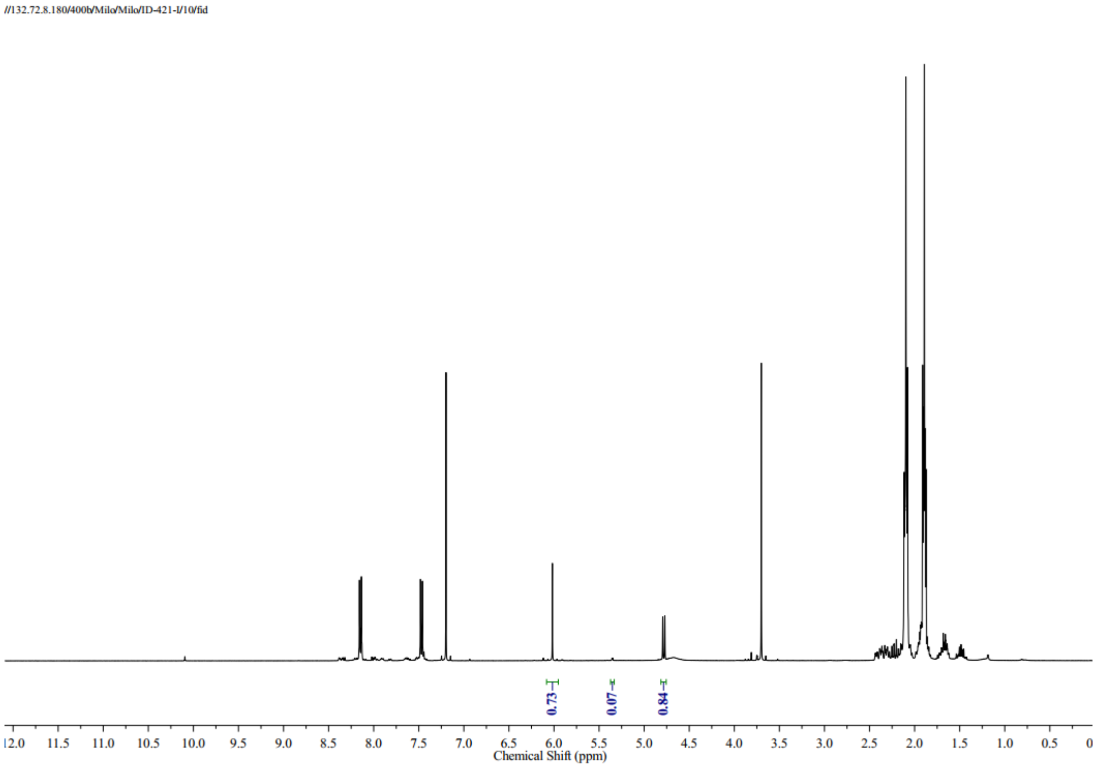

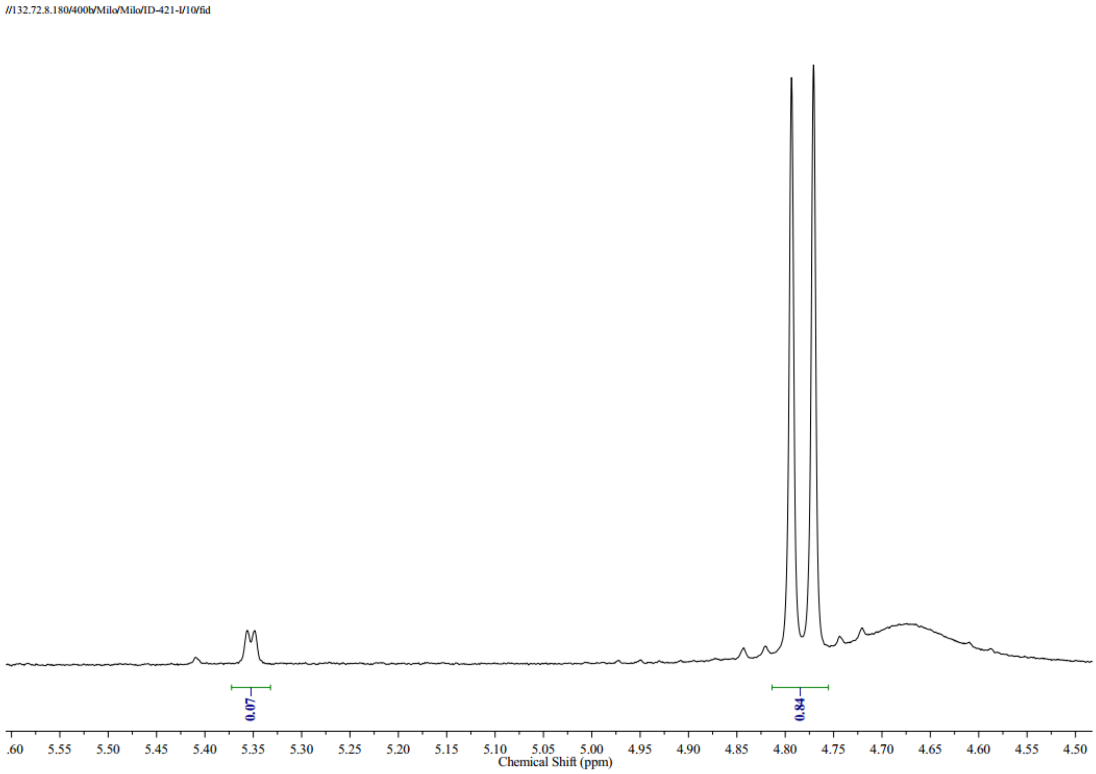

NMR traces for reaction with: **3-CF_3-phenylboronic acid (duplicate) (400 MHz,** $CDCl_3$**)**

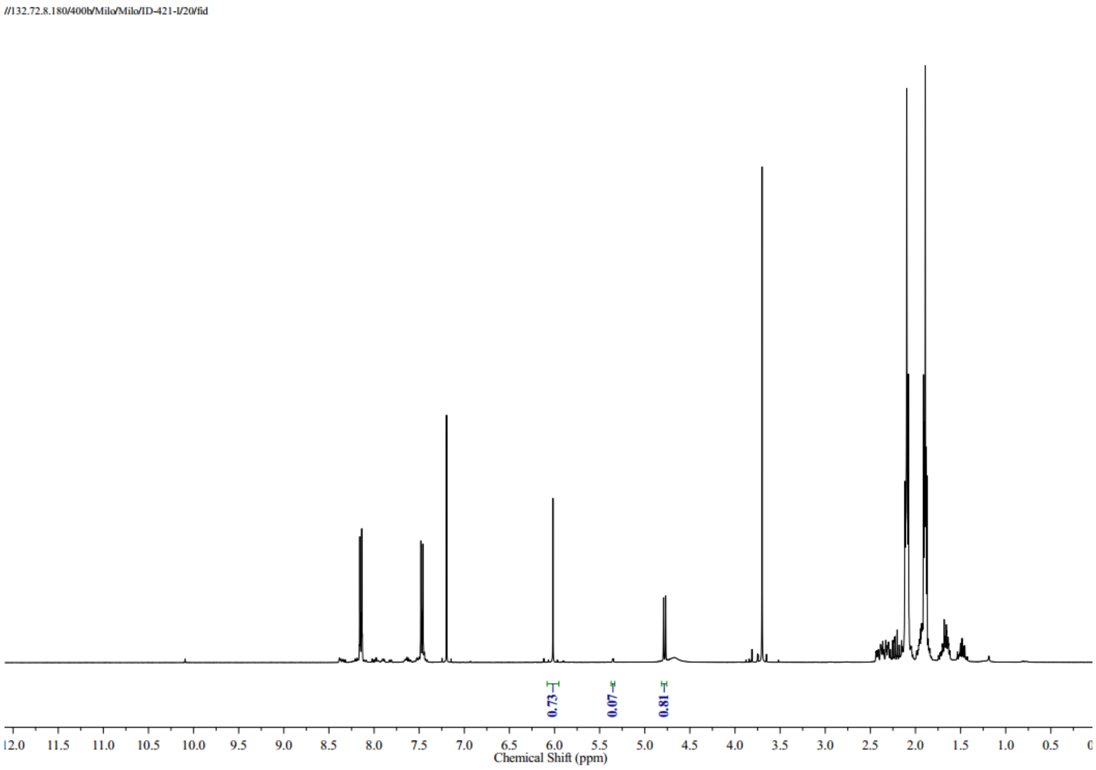

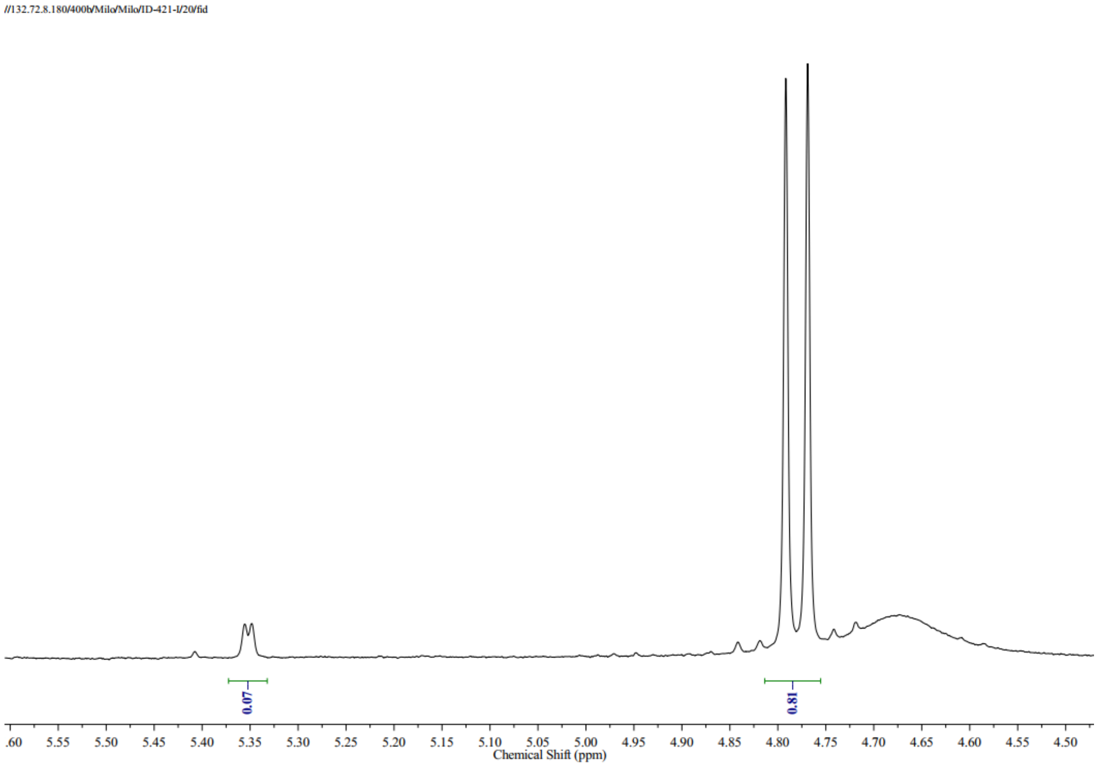

NMR traces for reaction with: **2-naphtalene-phenylboronic acid (400 MHz,** $CDCl_3$**)**

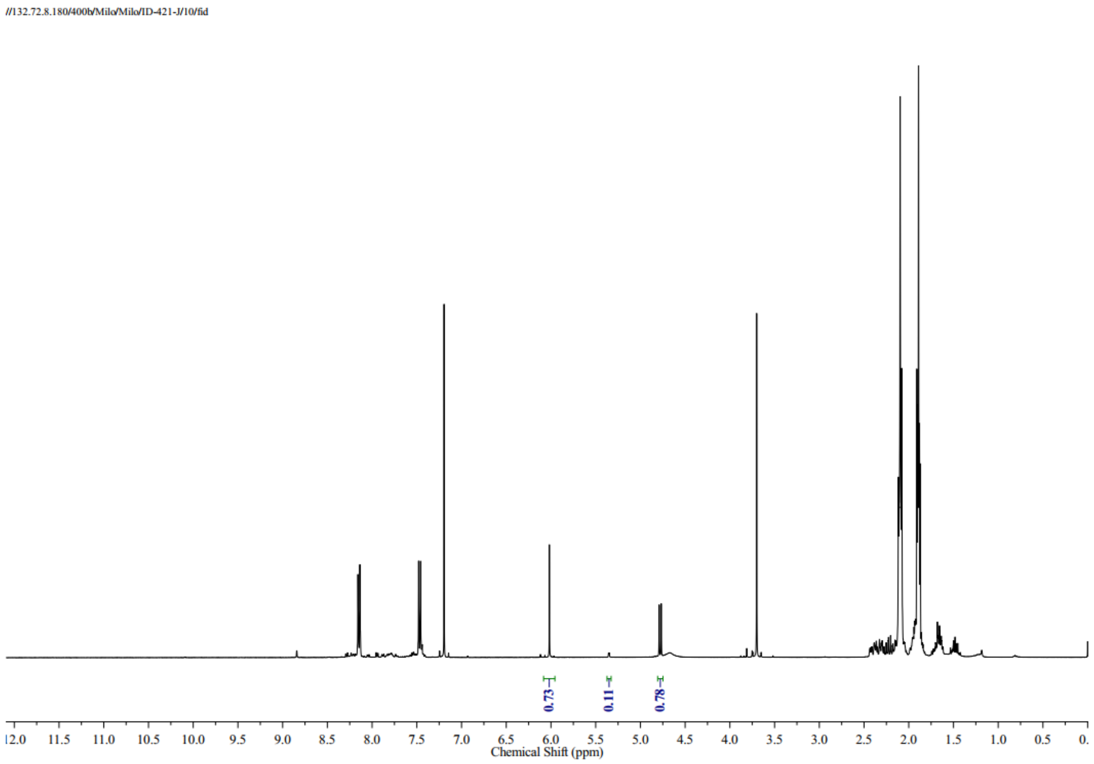

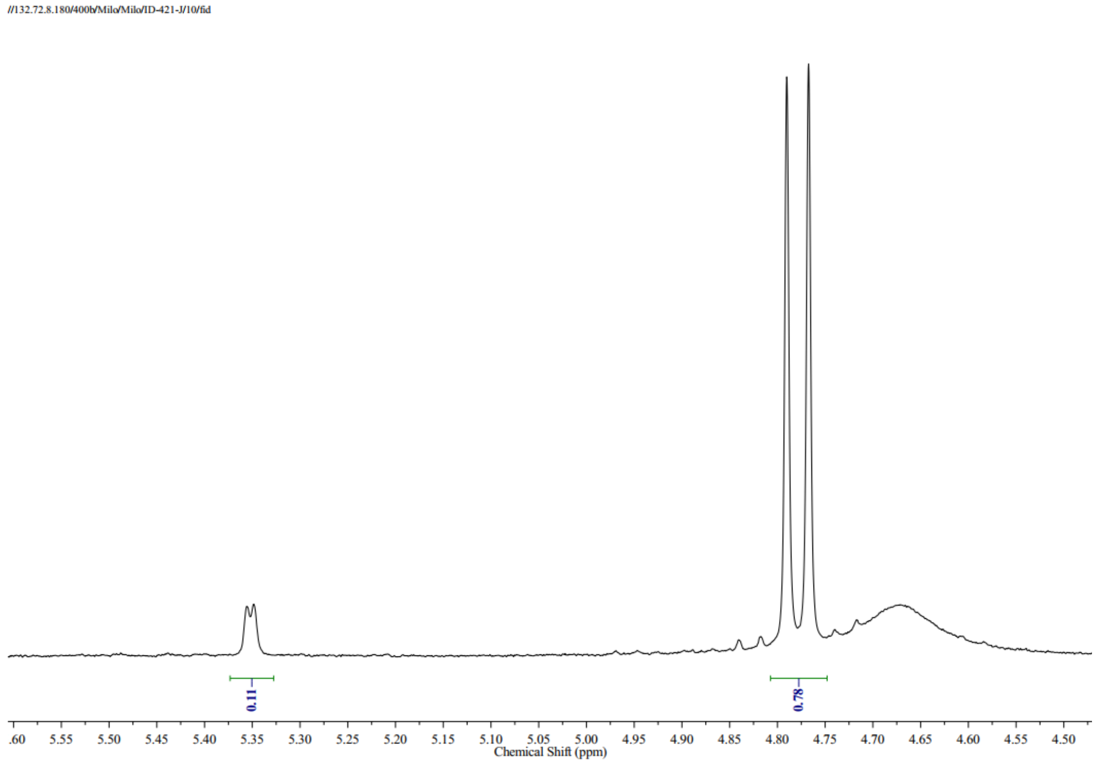

NMR traces for reaction with: **2-naphtalene-phenylboronic acid (duplicate) (400 MHz,** $CDCl_3$**)**

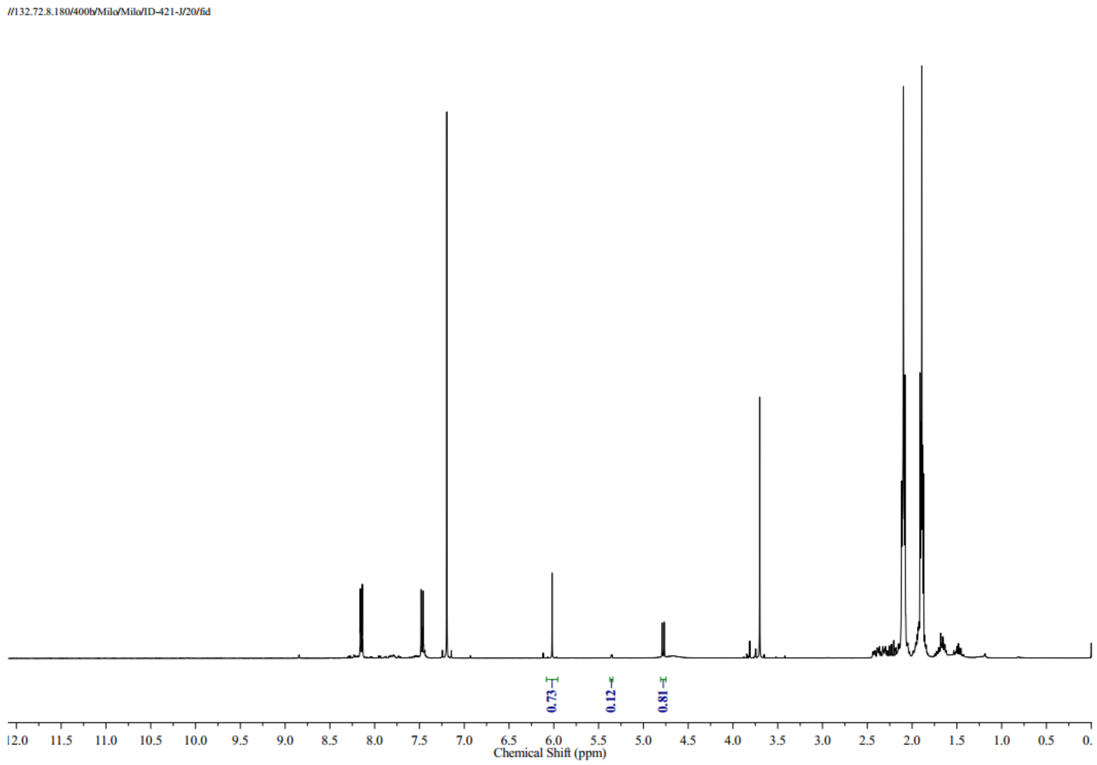

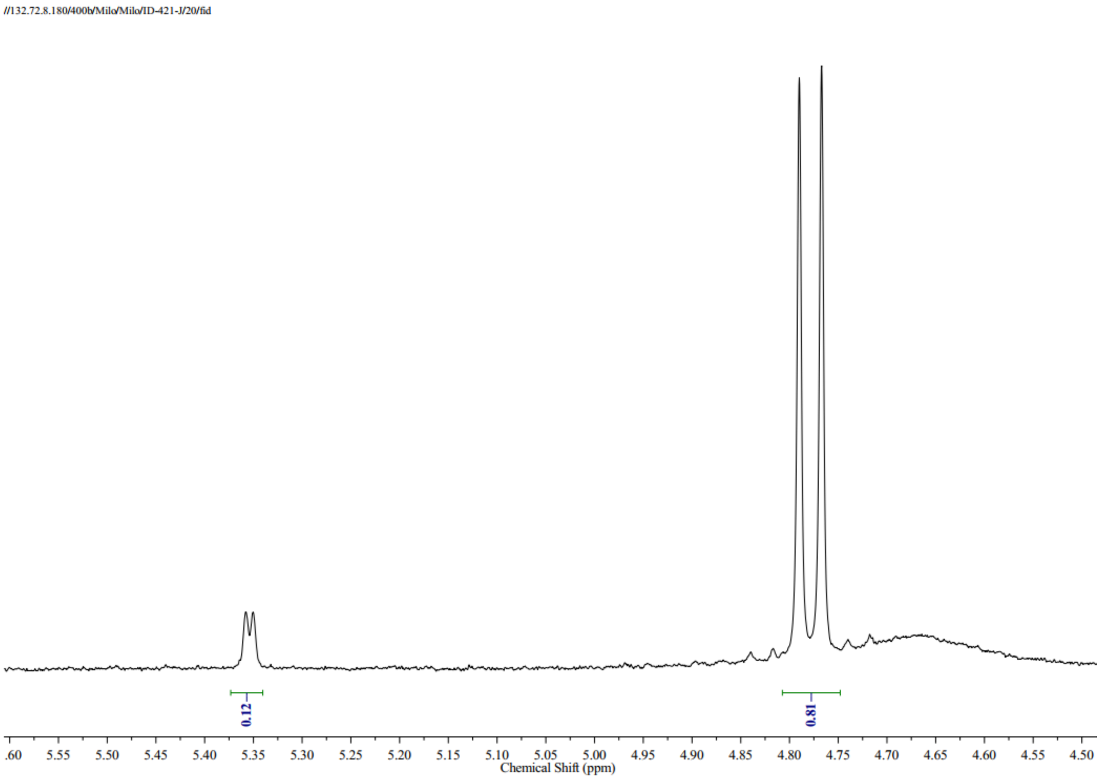

NMR traces for reaction with: **4-CF$_3$-phenylboronic acid (400 MHz,** $CDCl_3$**)**

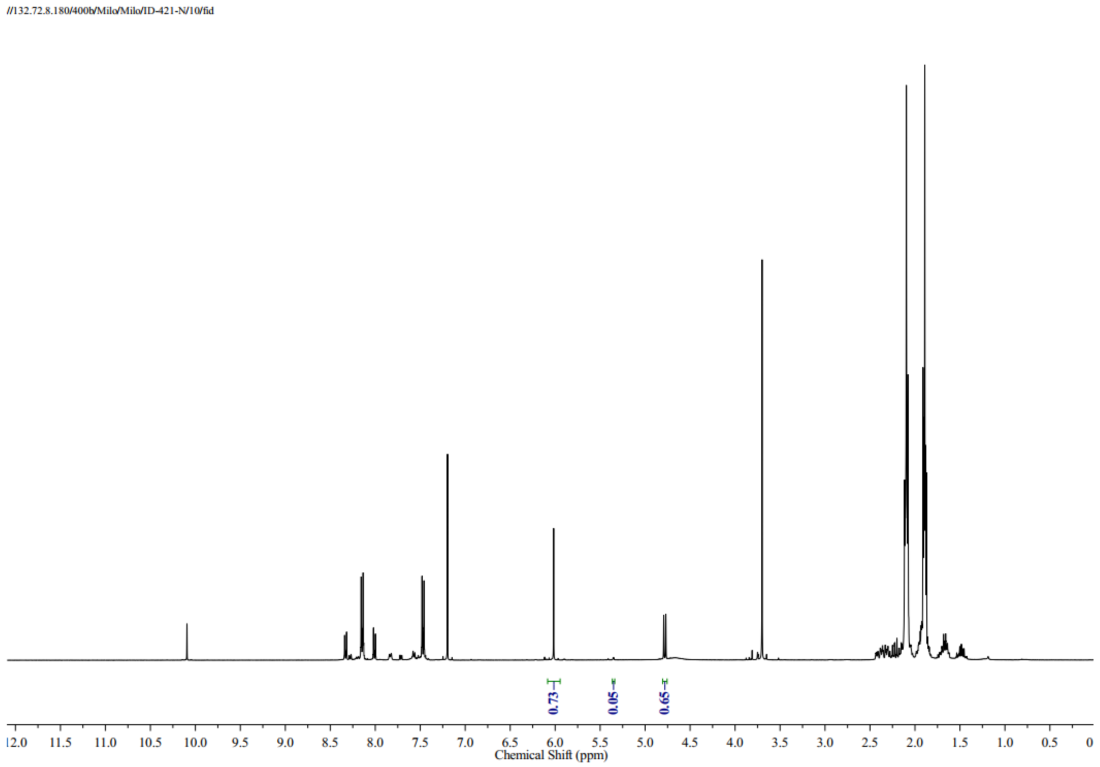

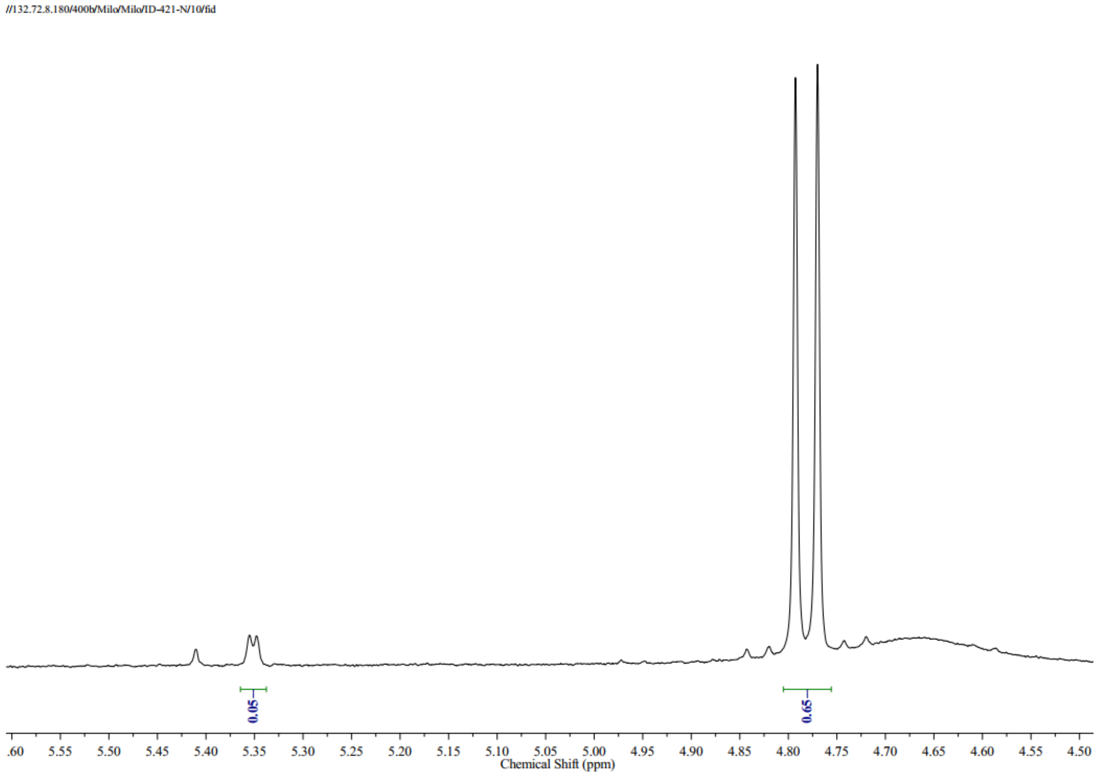

NMR traces for reaction with: **4-CF_3-phenylboronic acid (duplicate) (400 MHz,** $CDCl_3$**)**

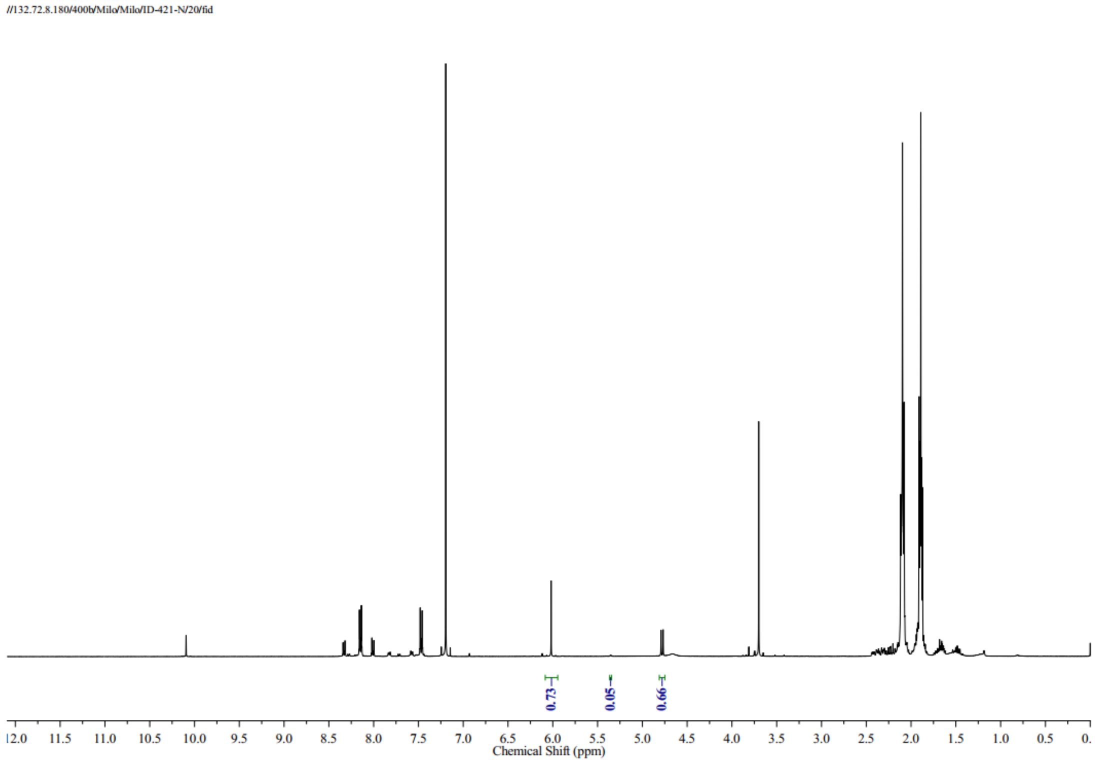

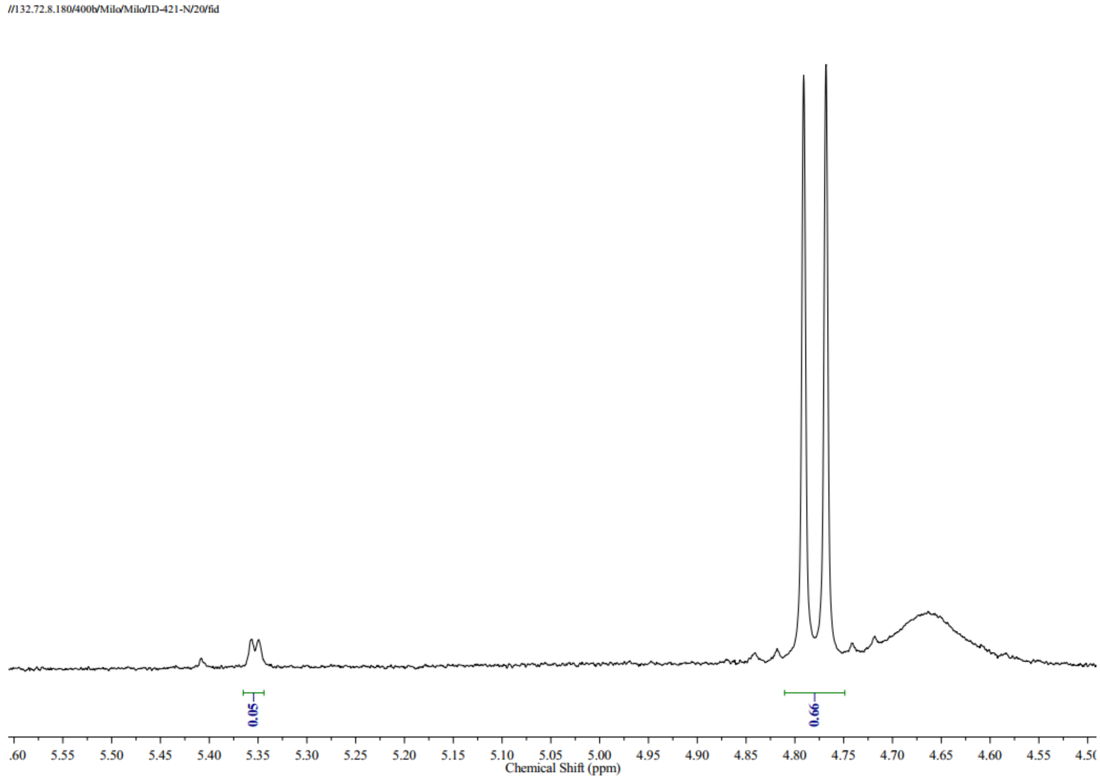

NMR traces for reaction with: **4-F-phenylboronic acid (400 MHz,** $CDCl_3$**)**

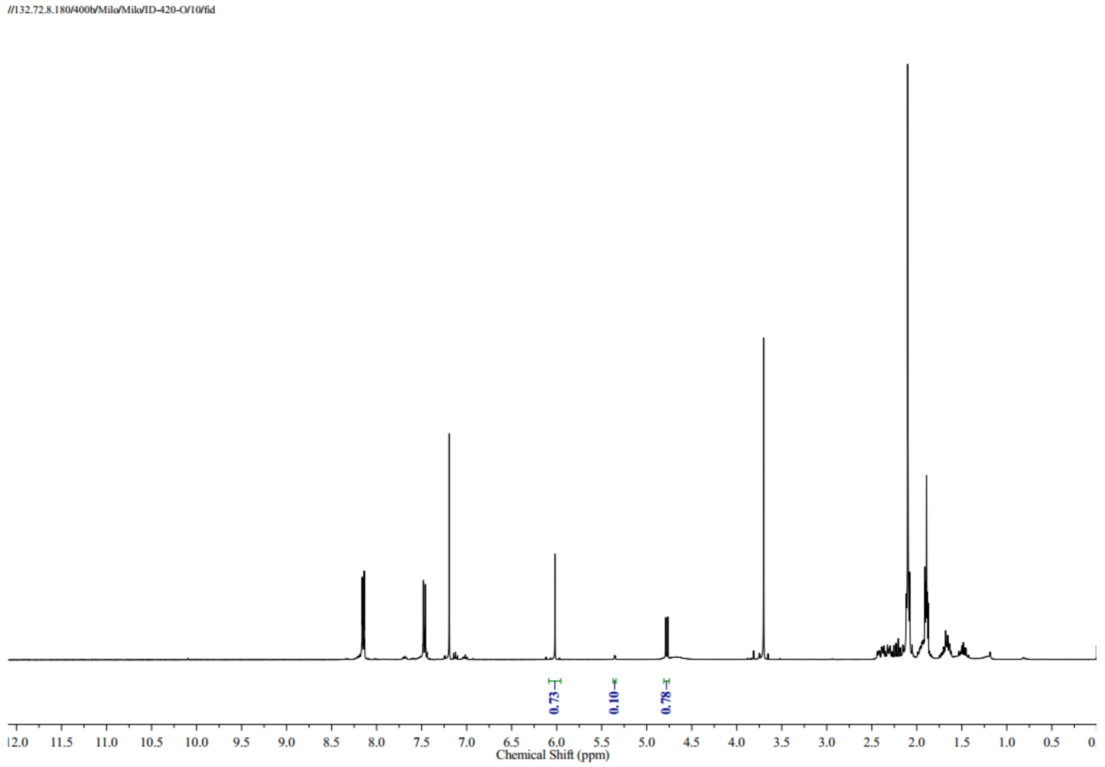

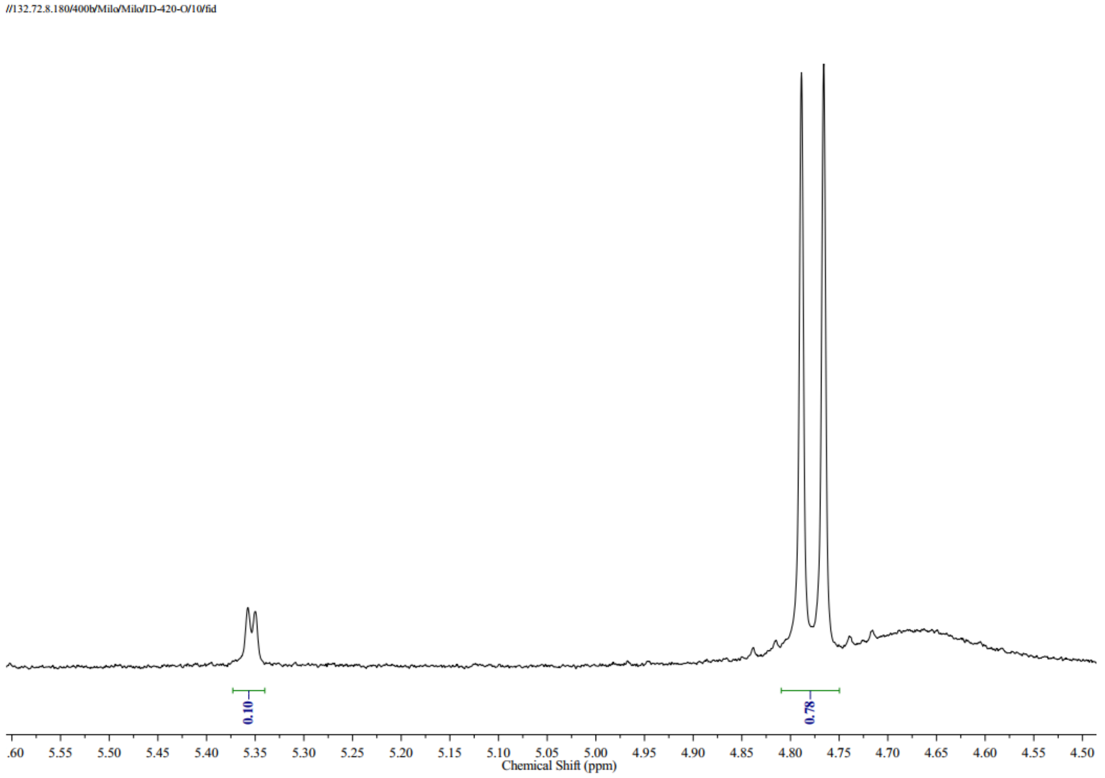

NMR traces for reaction with: **4-F-phenylboronic acid (duplicate) (400 MHz,** $CDCl_3$**)**

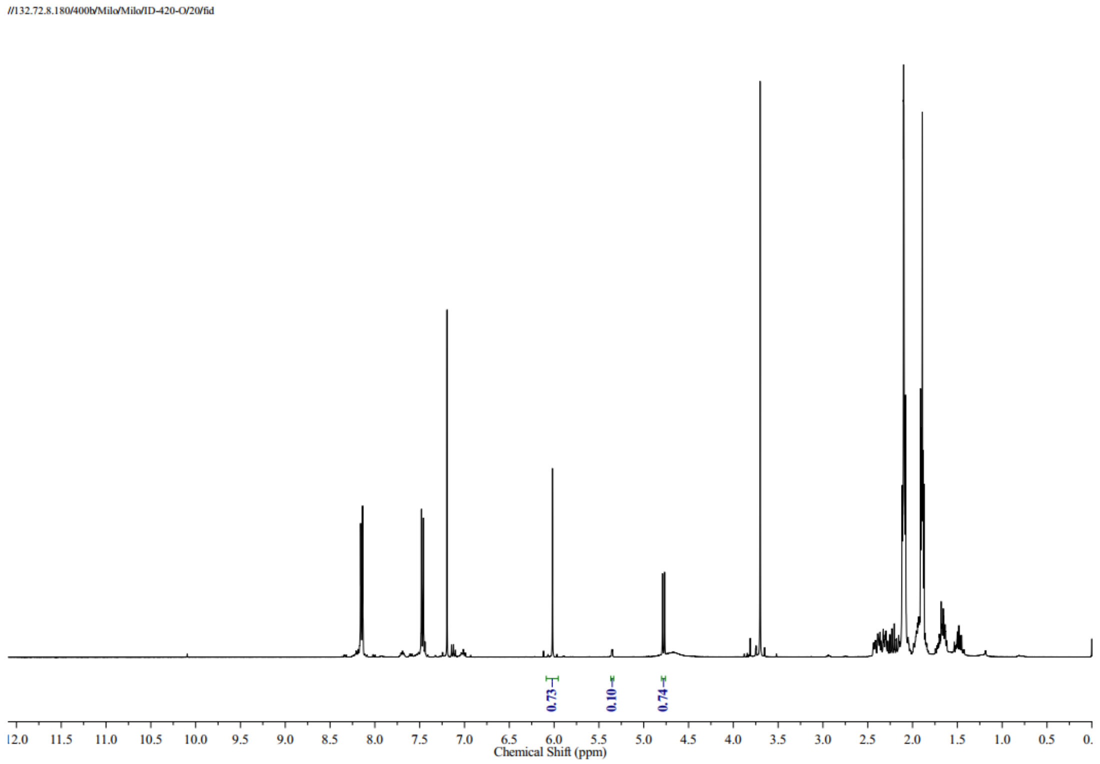

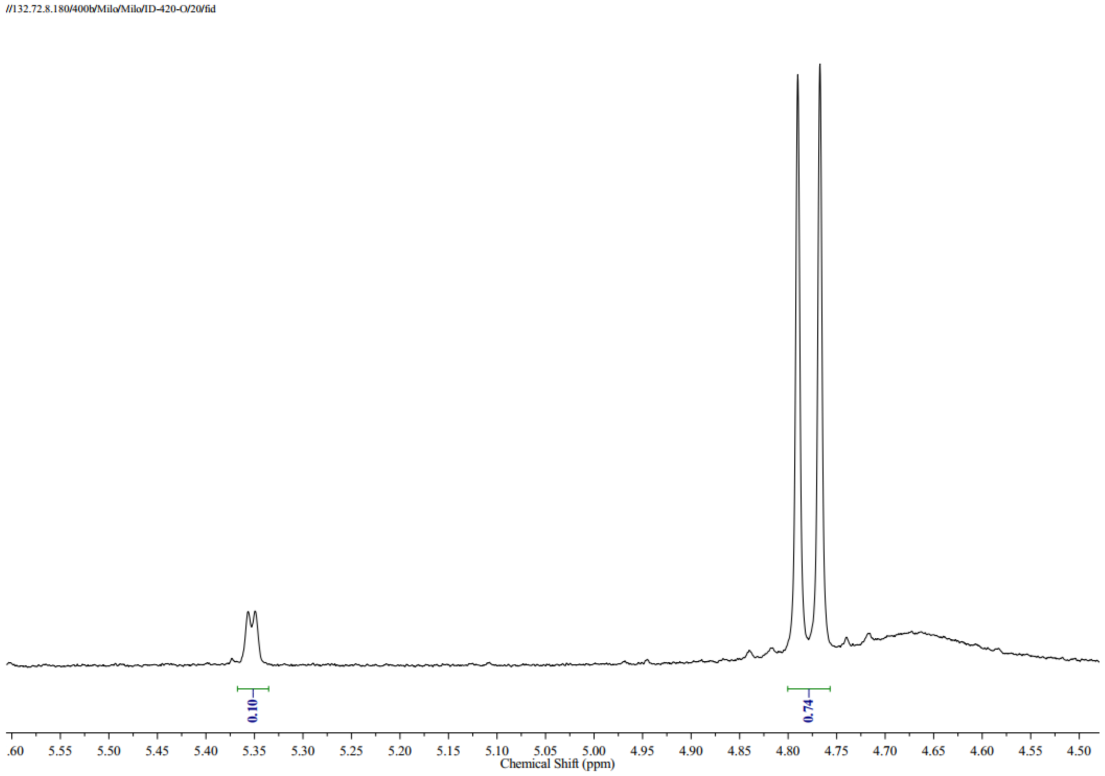

NMR traces for reaction with: **4-OMe-phenylboronic acid (400 MHz,** $CDCl_3$**)**

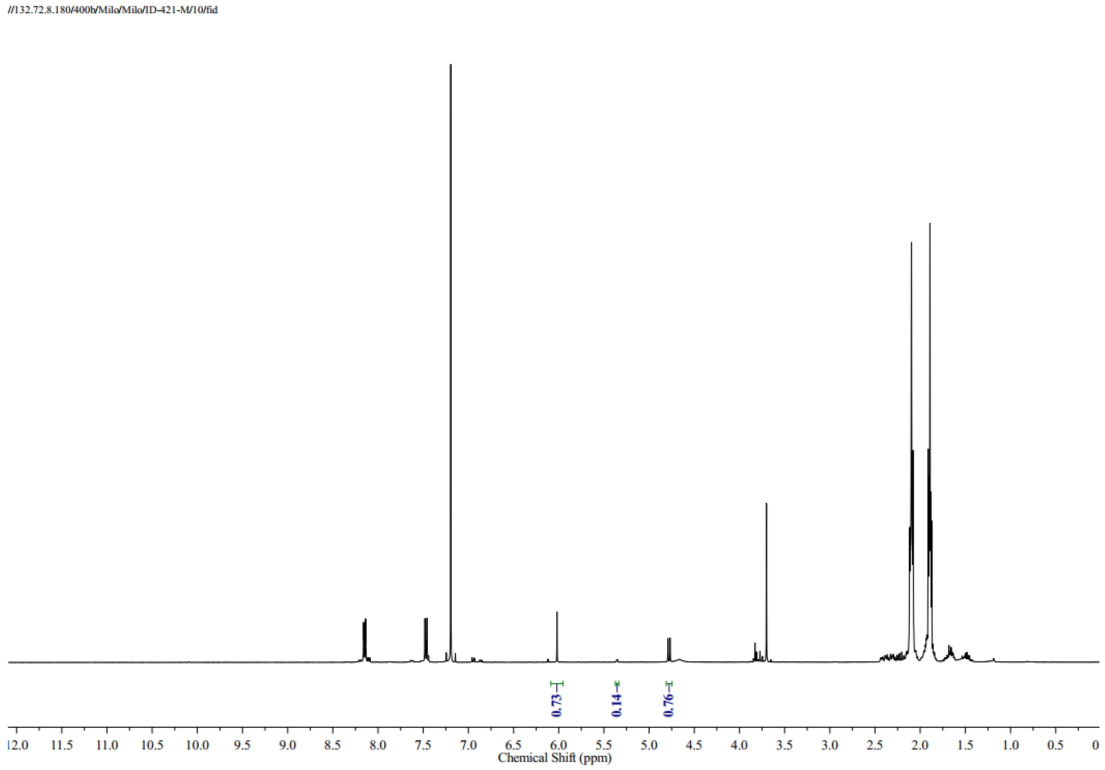

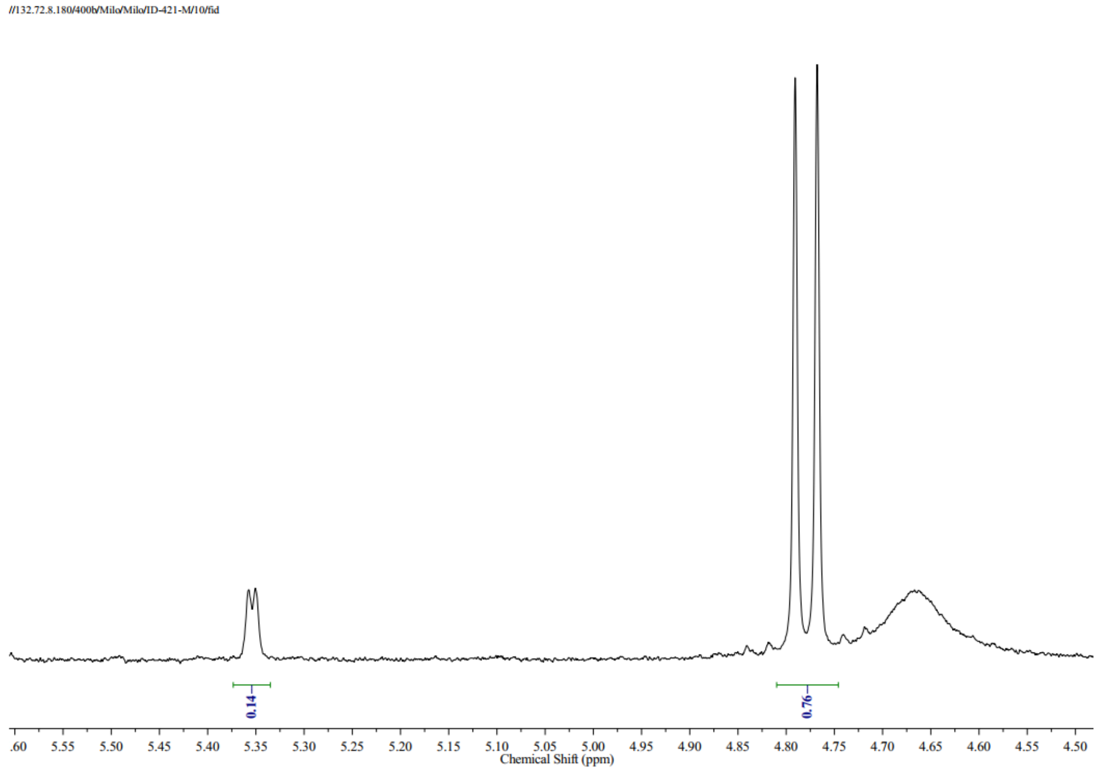

NMR traces for reaction with: **4-OMe-phenylboronic acid (duplicate) (400 MHz,** $CDCl_3$**)**

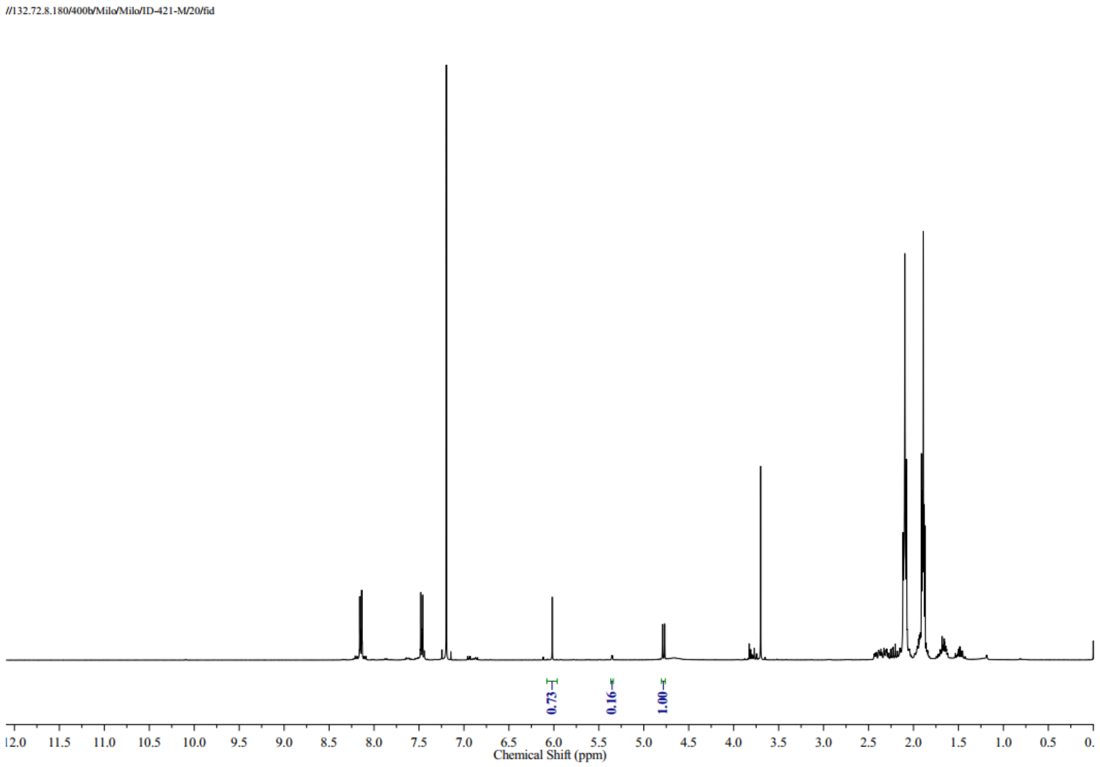

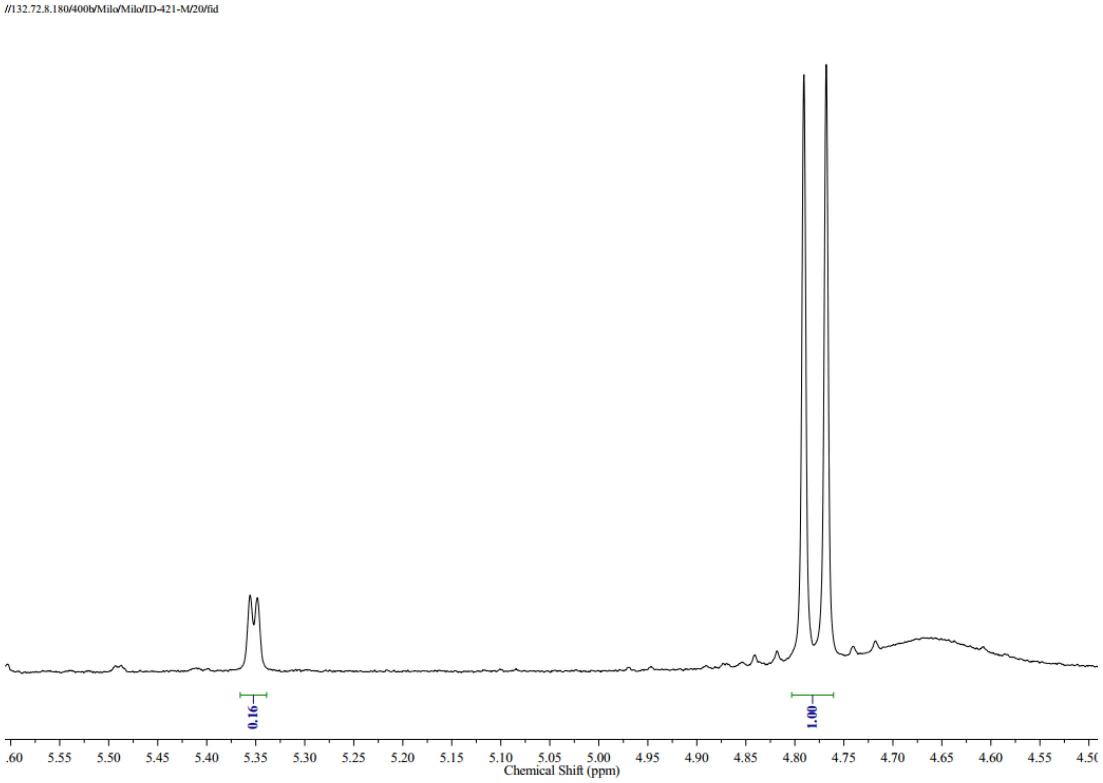

NMR traces for reaction with: **3-Me-phenylboronic acid (400 MHz,** CDCl$_3$**)**

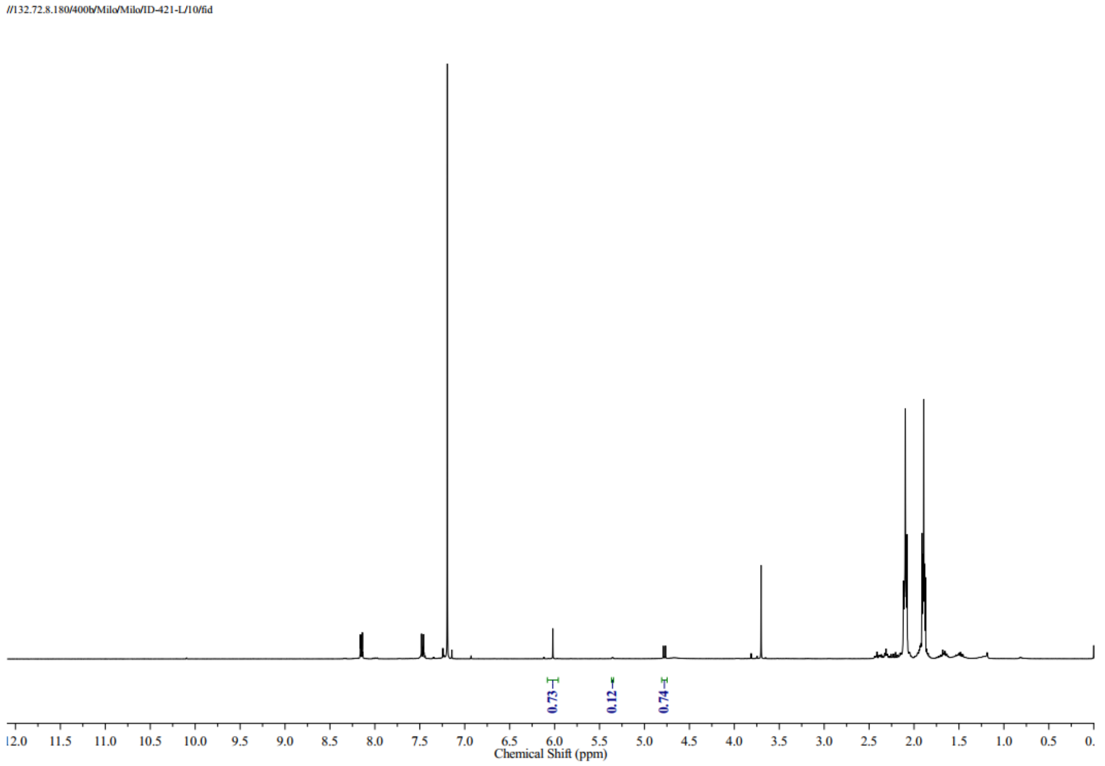

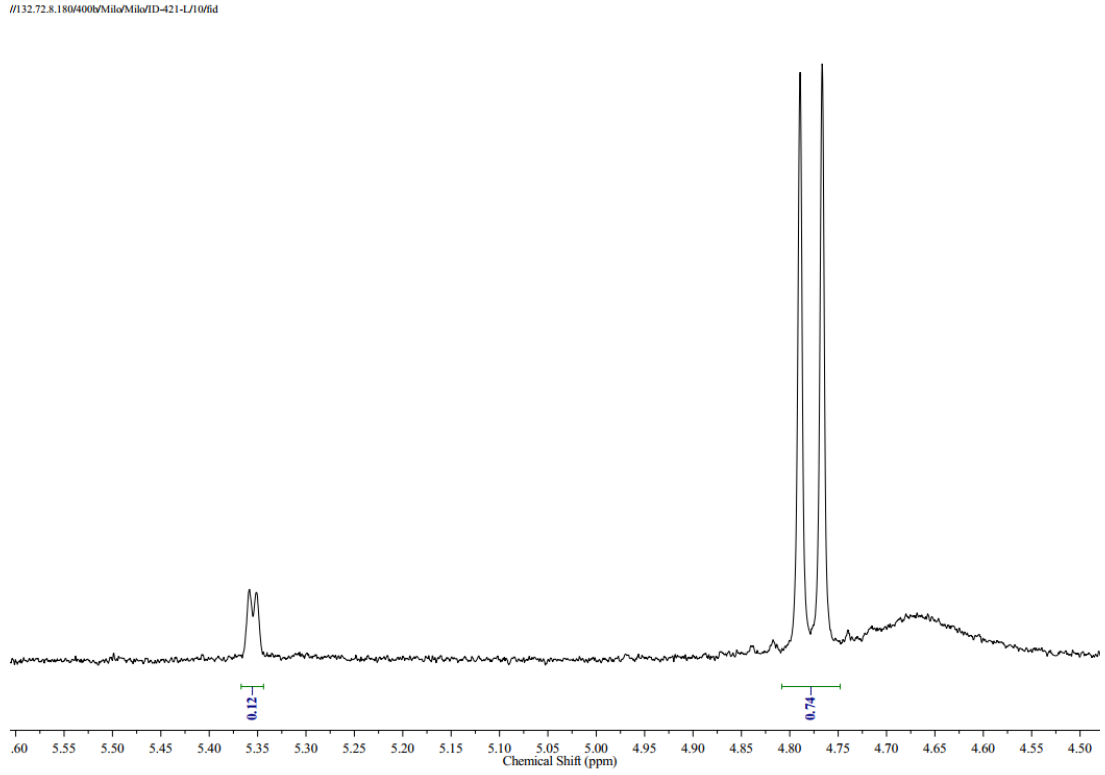

NMR traces for reaction with: **3-Me-phenylboronic acid (duplicate) (400 MHz,** CDCl$_3$**)**

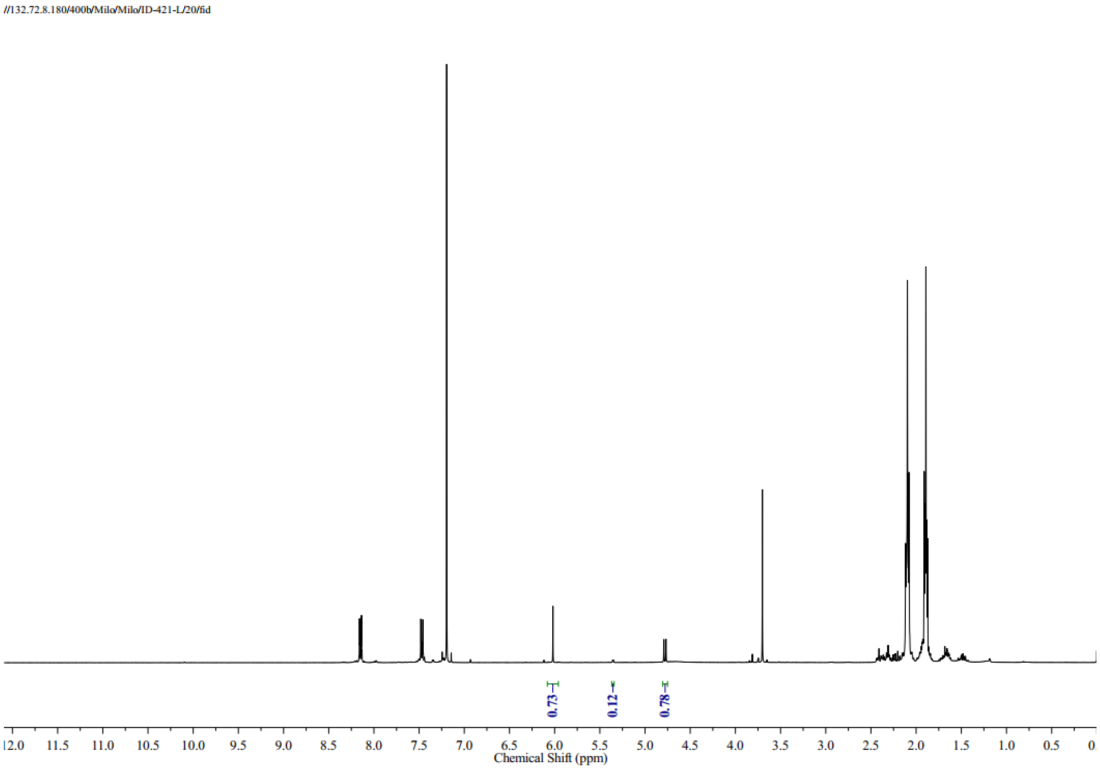

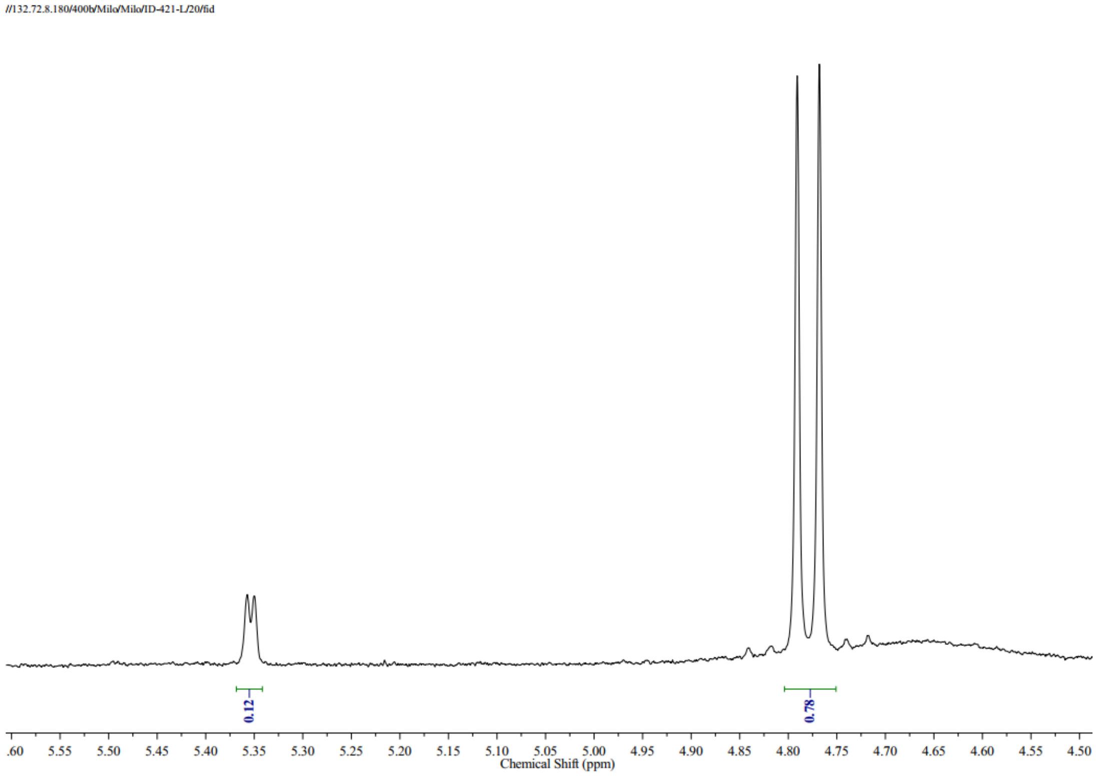

NMR traces for reaction with: **2-Me-phenylboronic acid (400 MHz,** $CDCl_3$**)**

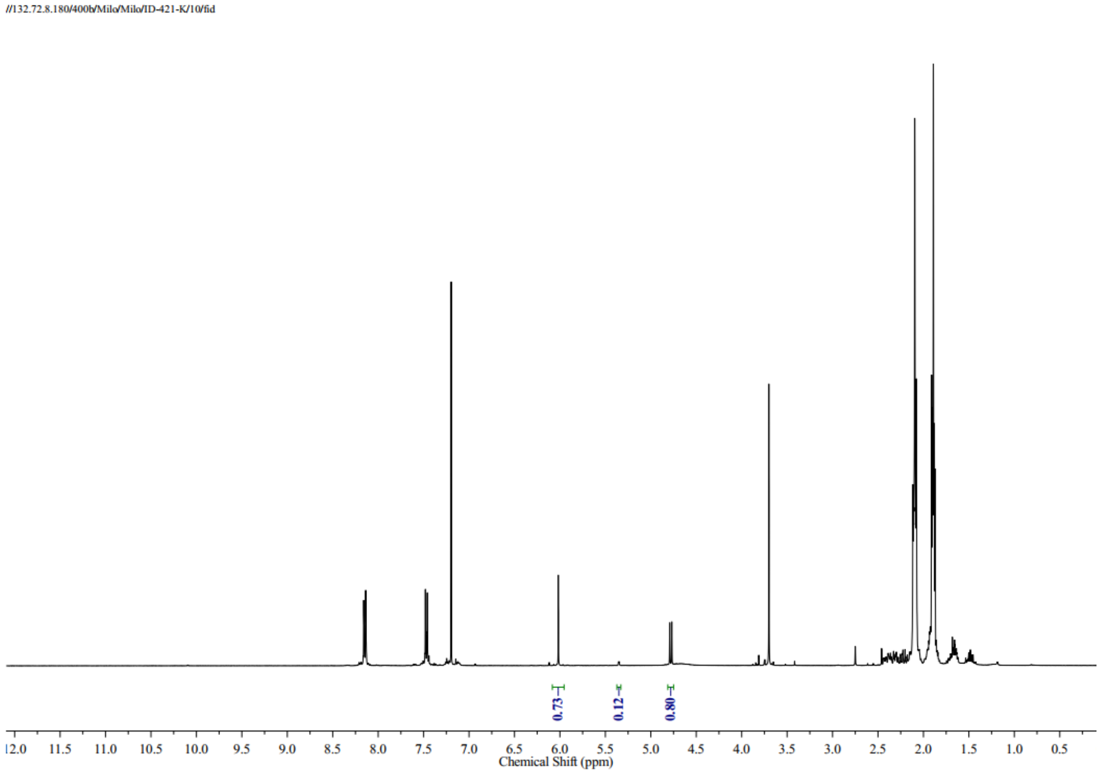

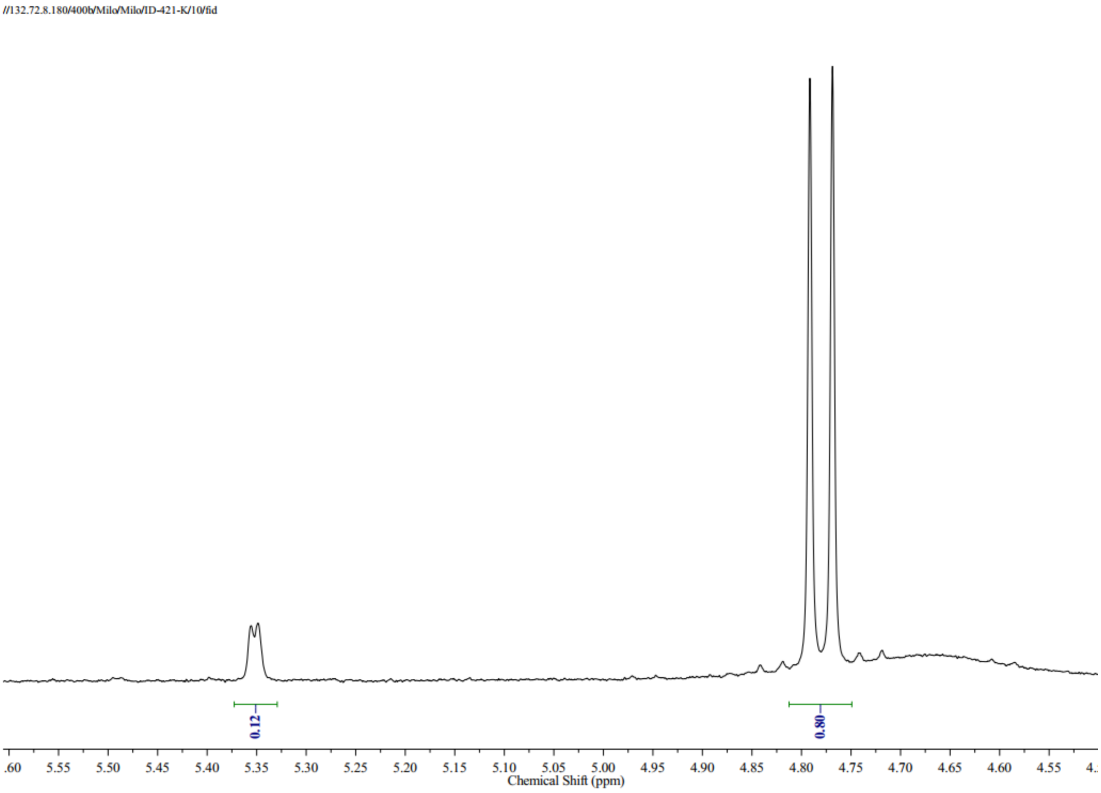

NMR traces for reaction with: **2-Me-phenylboronic acid (duplicate) (400 MHz,** CDCl$_3$**)**

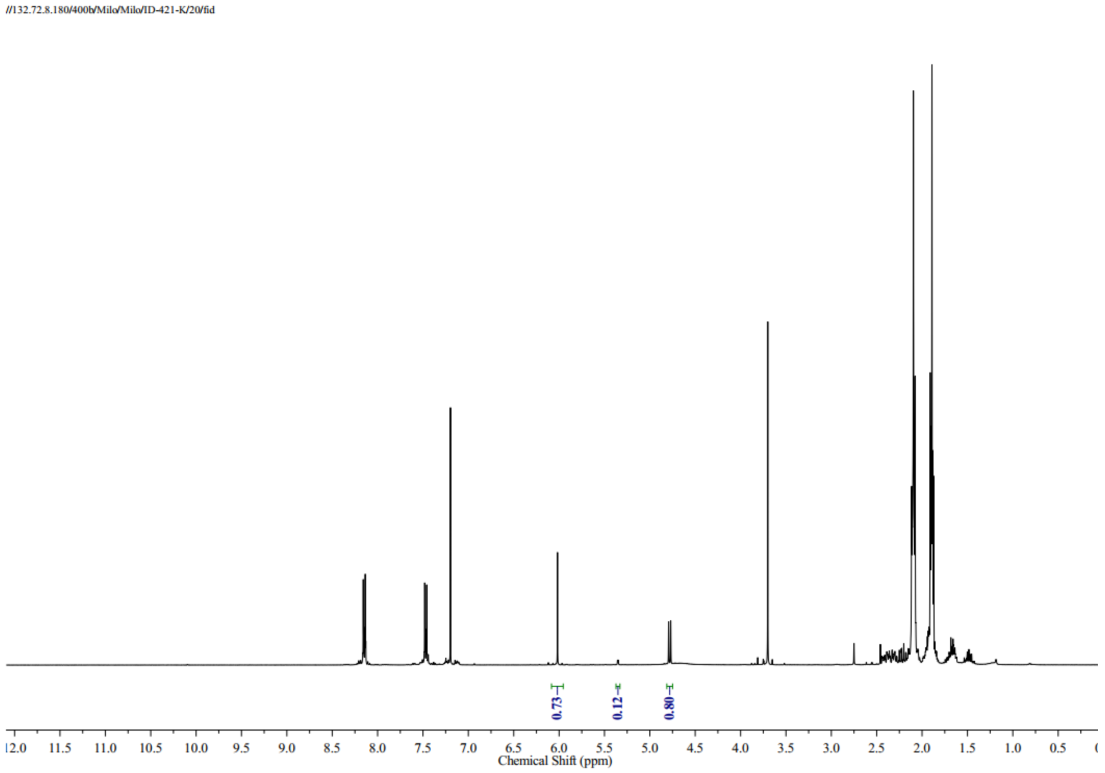

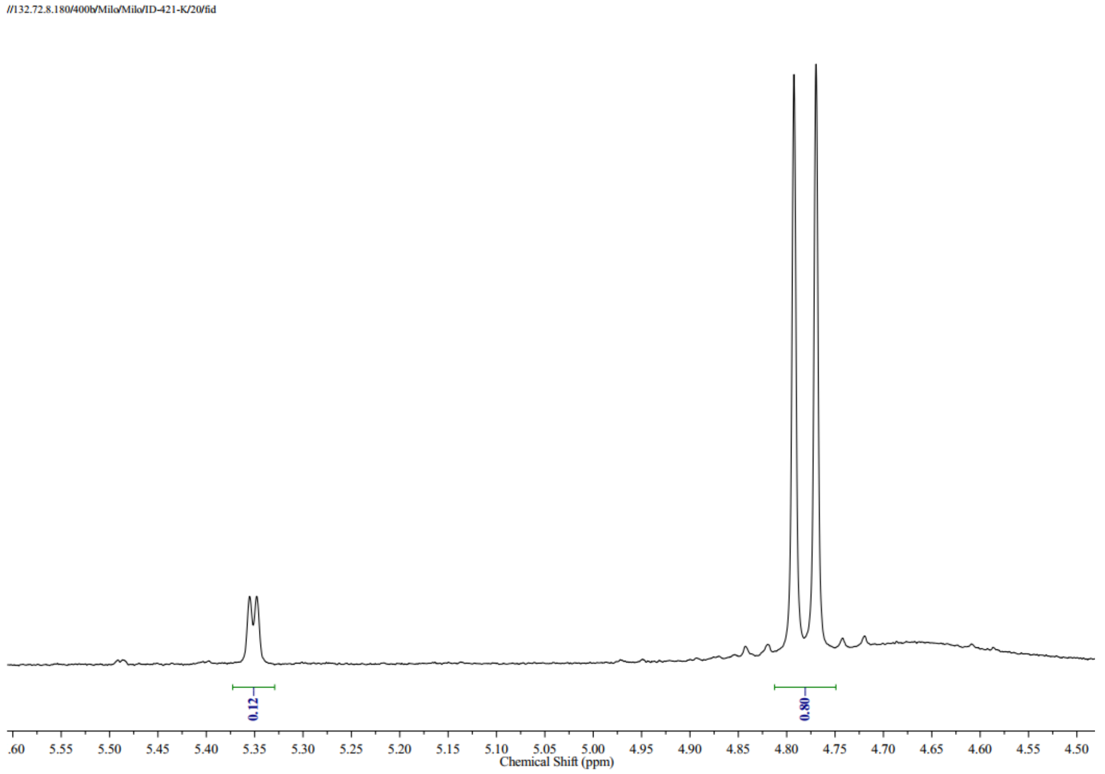

NMR traces for reaction with: **Ph-phenylboronic acid (400 MHz,** $CDCl_3$**)**

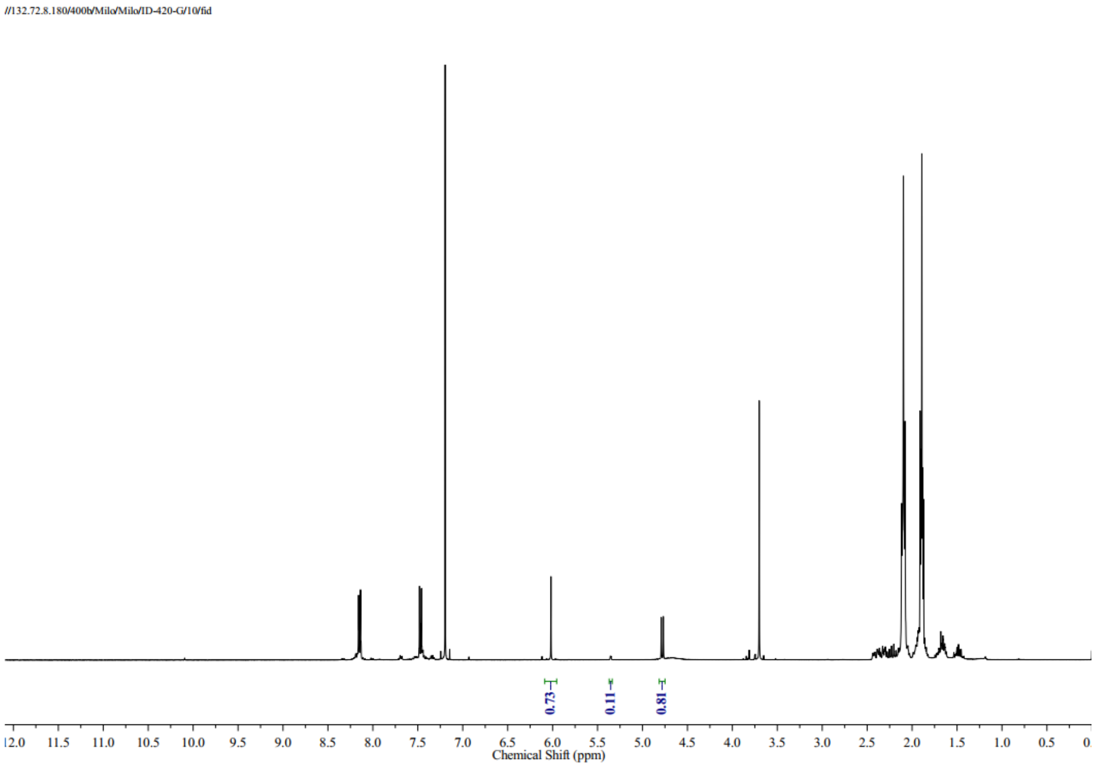

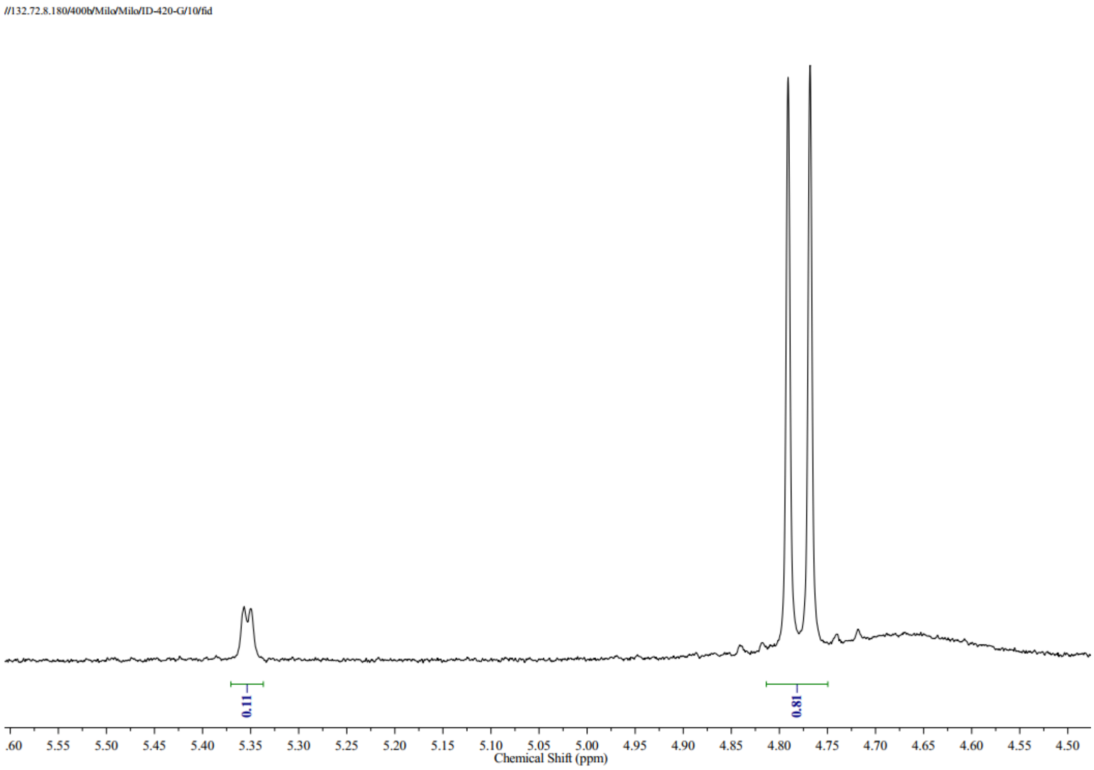

NMR traces for reaction with: **Ph-phenylboronic acid (duplicate) (400 MHz,** $CDCl_3$**)**

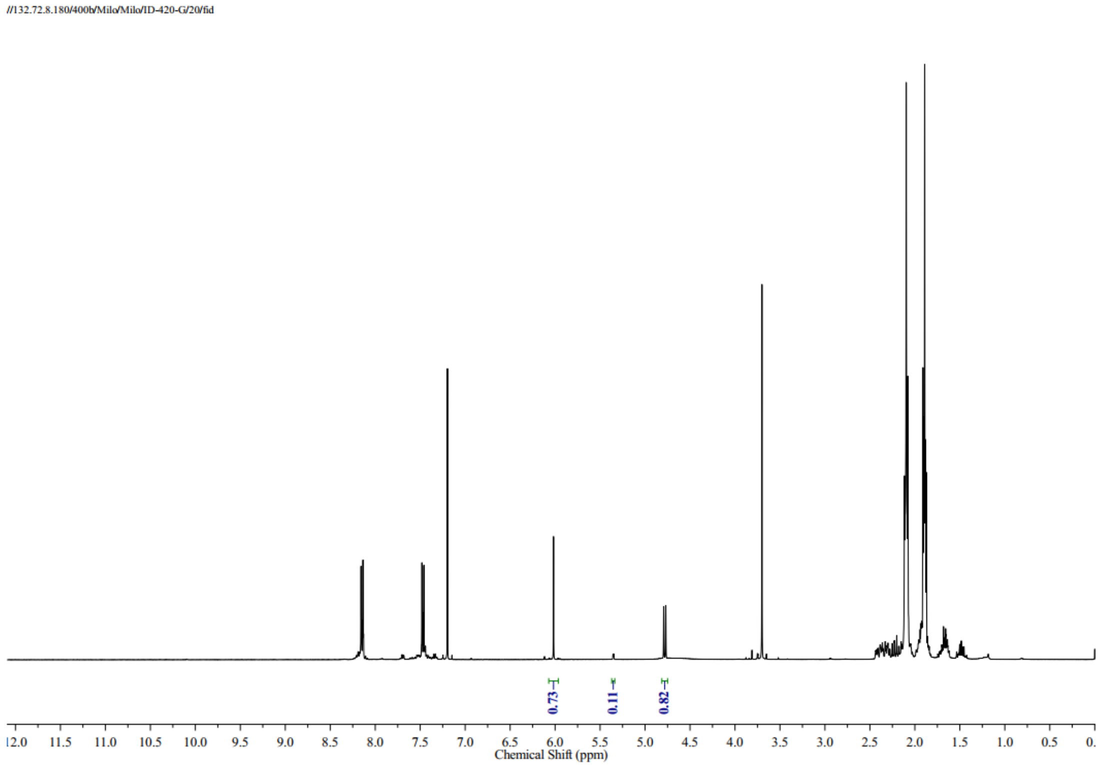

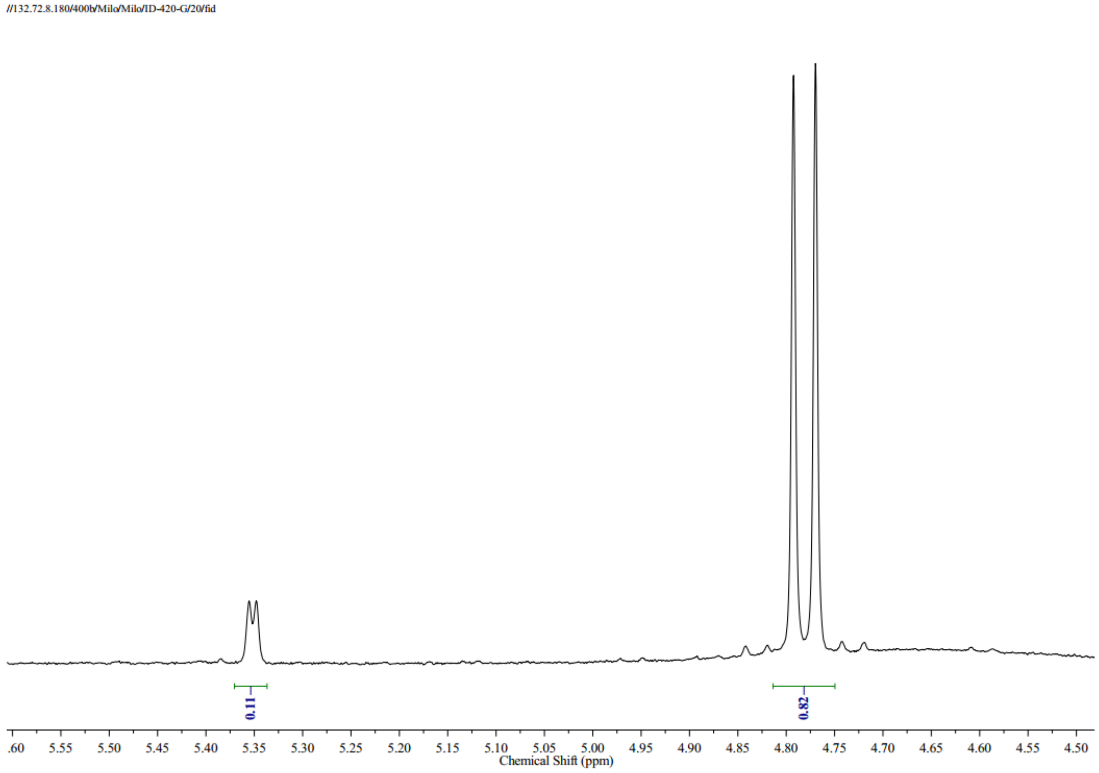

NMR traces for reaction with: **2-F-phenylboronic acid (400 MHz,** $CDCl_3$**)**

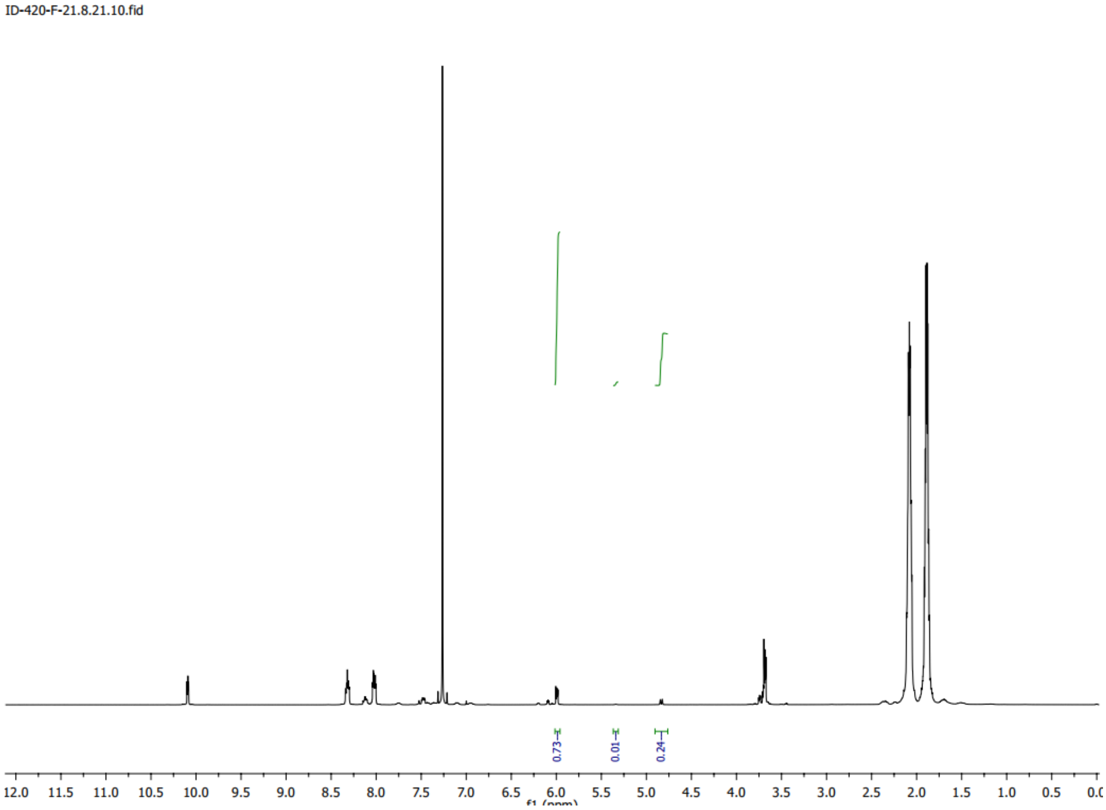

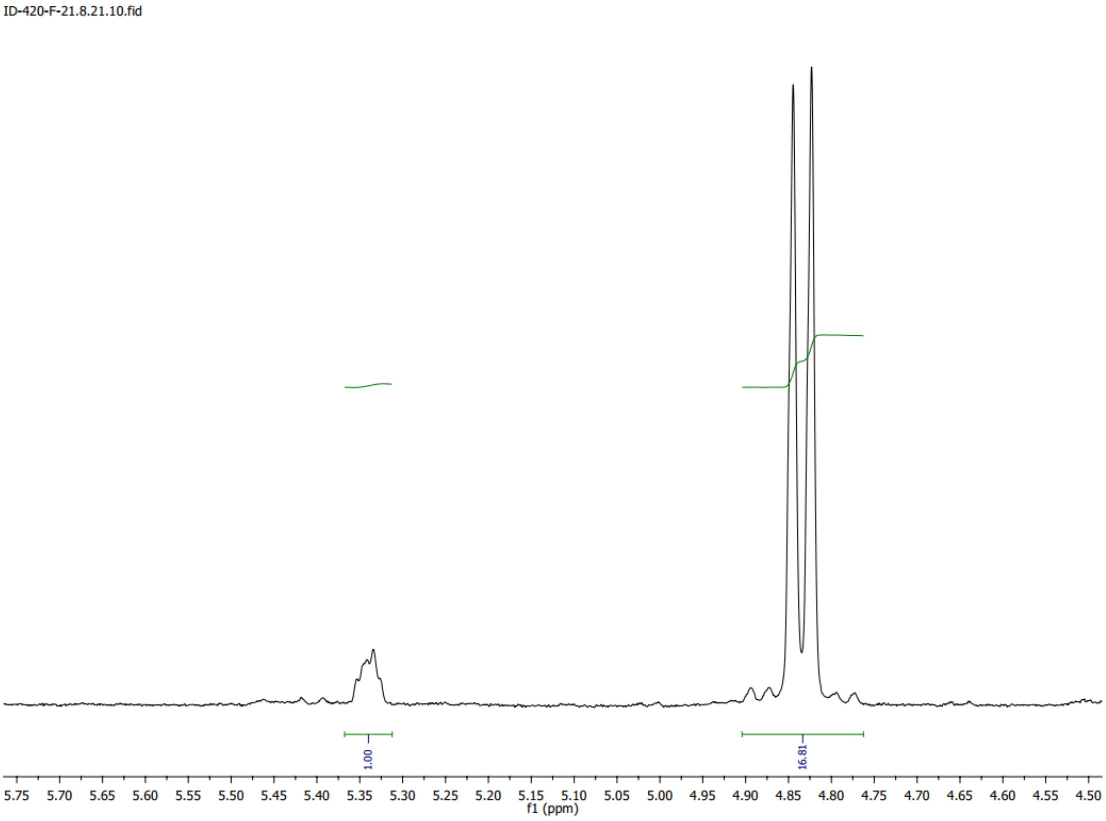

NMR traces for reaction with: **2-F-phenylboronic acid (duplicate) (400 MHz,** CDCl$_3$**)**

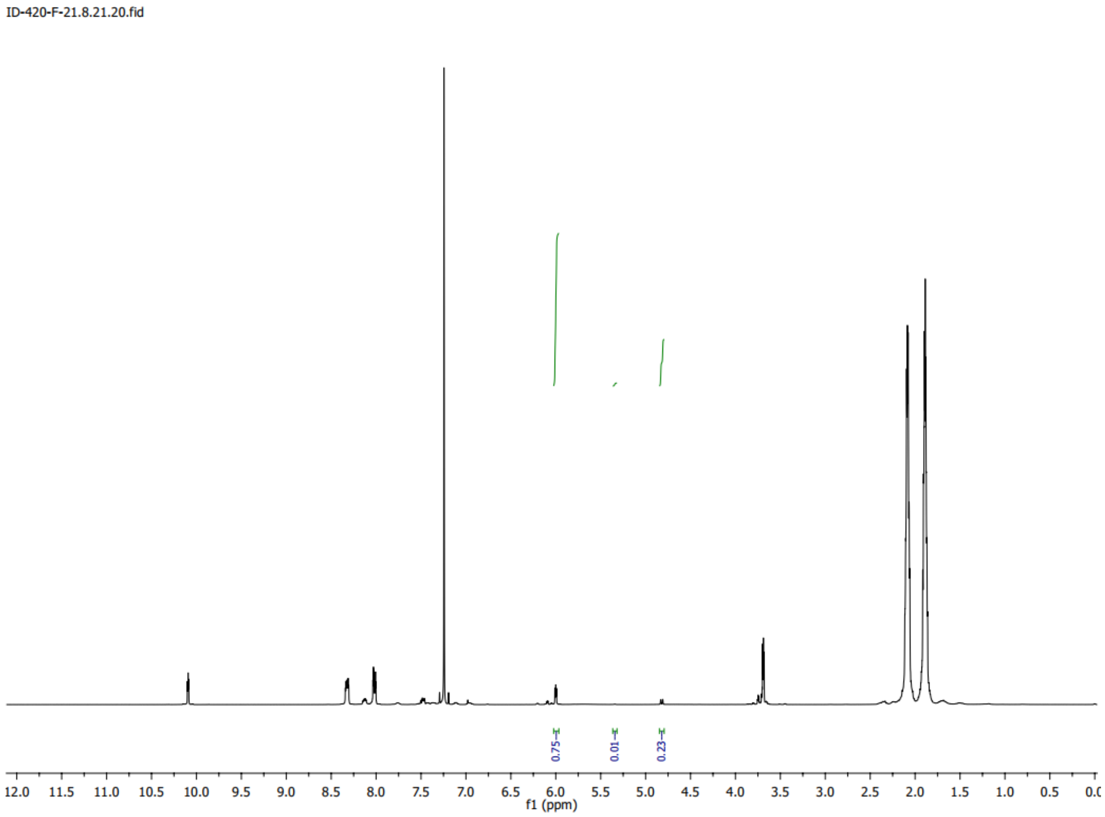

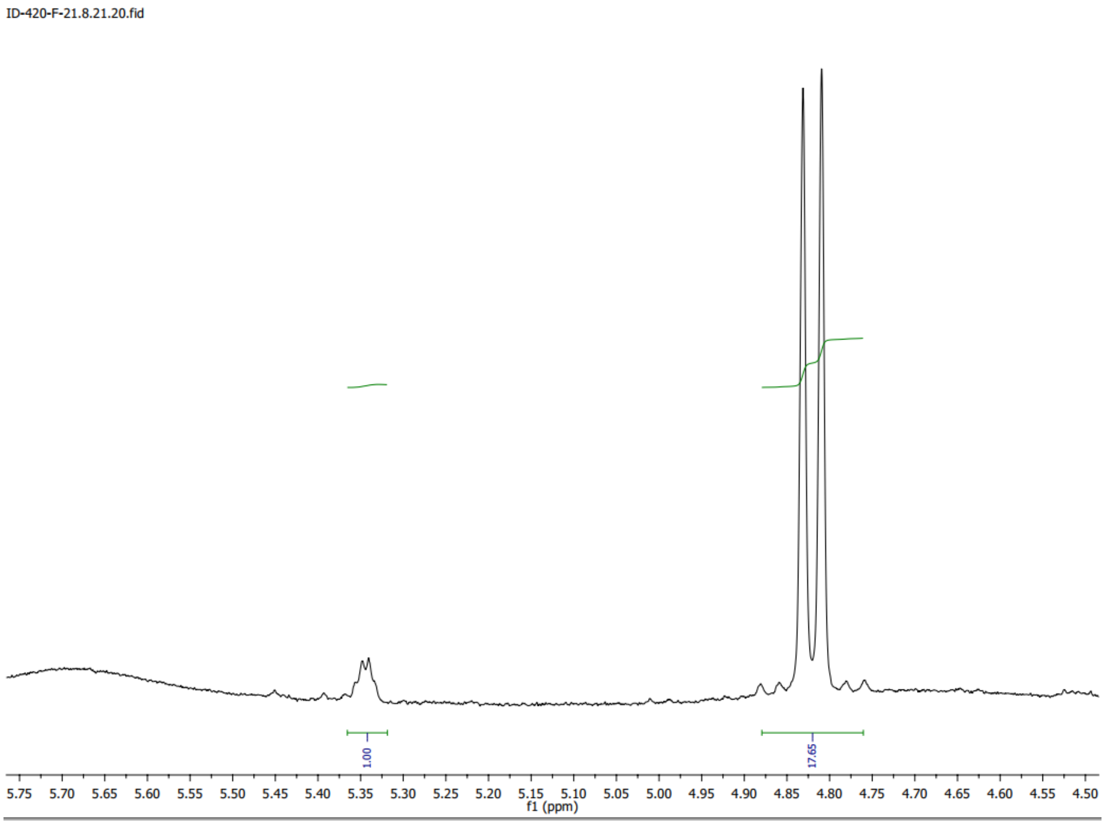

NMR traces for reaction **without boronic acid (400 MHz,** $CDCl_3$**)**:

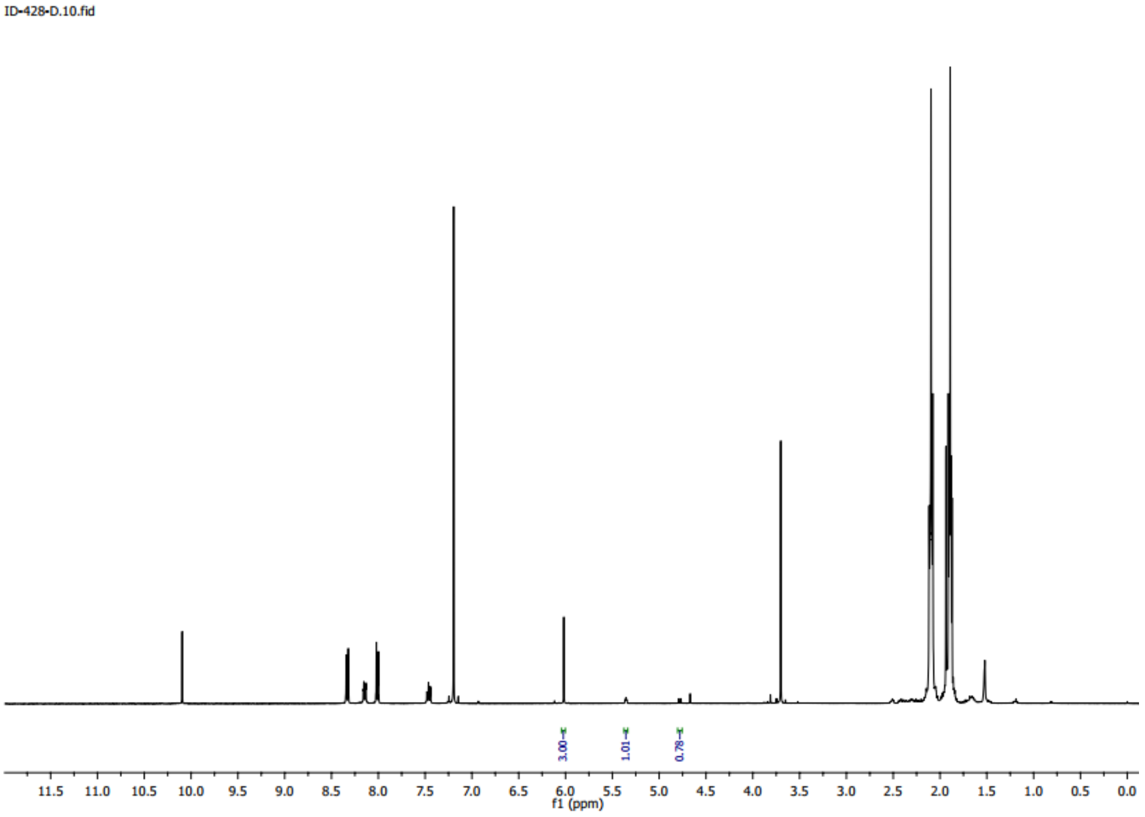

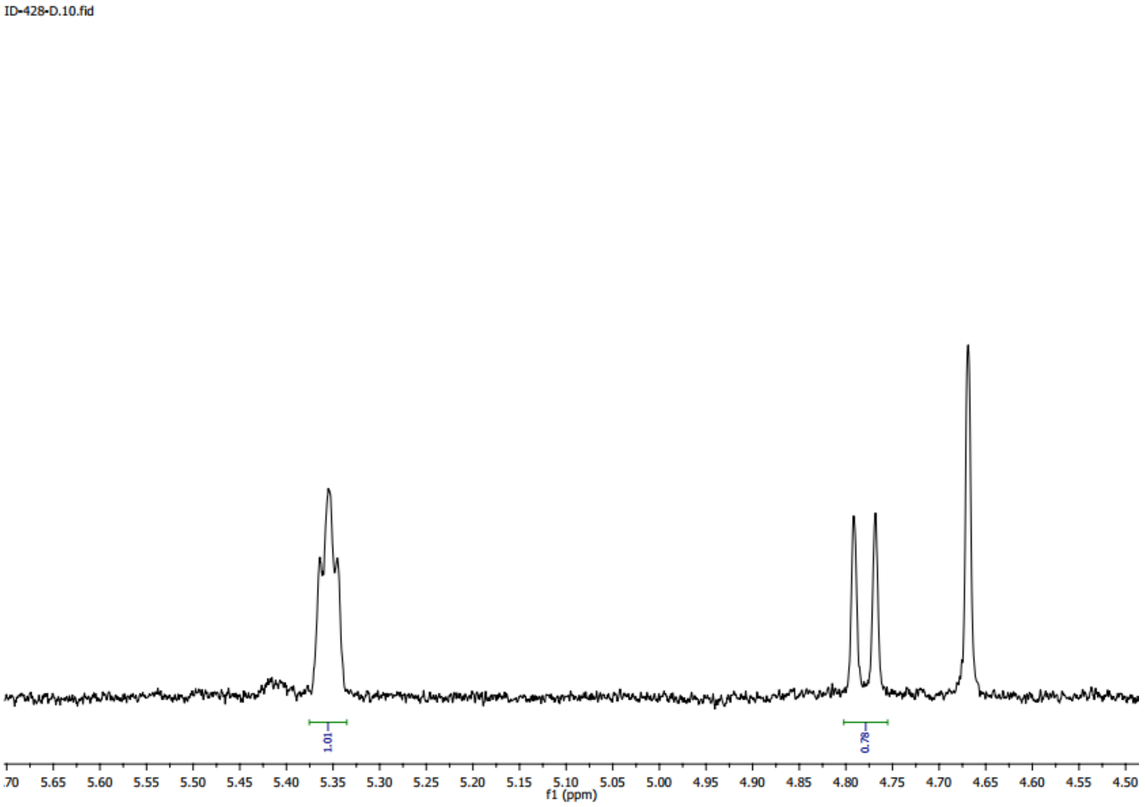

NMR traces for reaction **without boronic acid (duplicate) (400 MHz,** $CDCl_3$**)**:

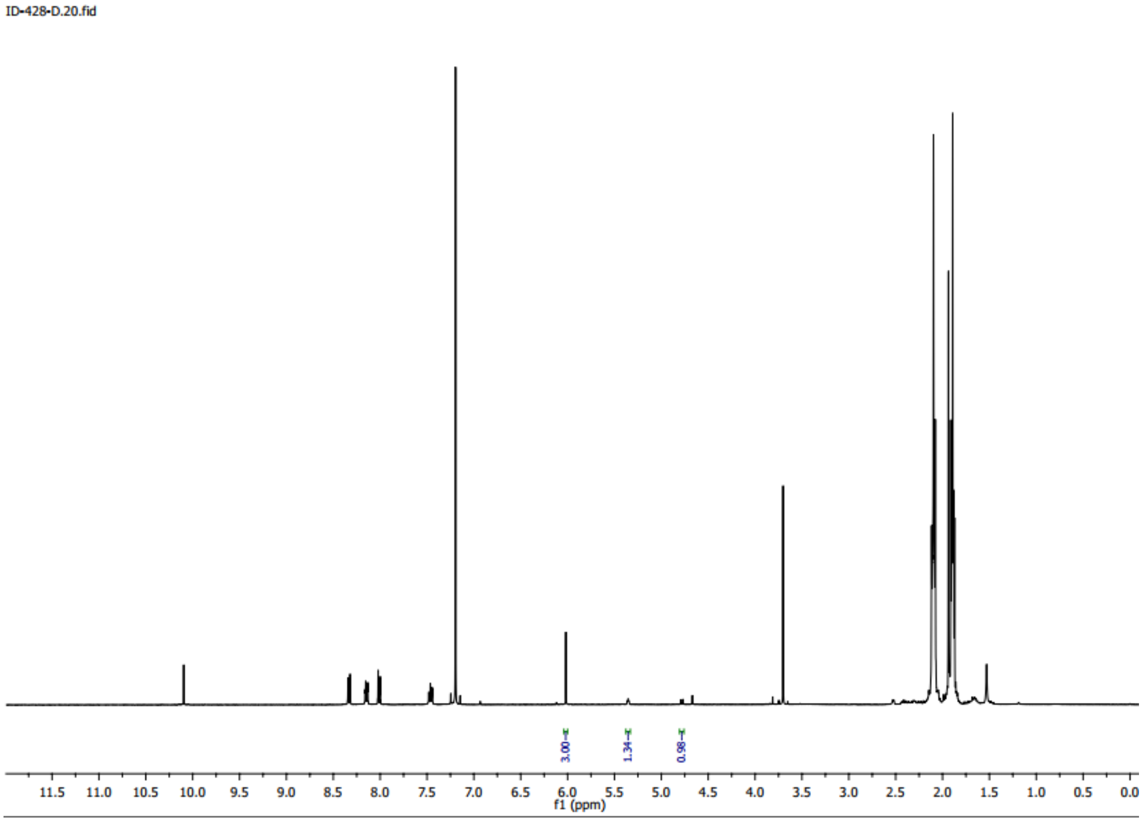

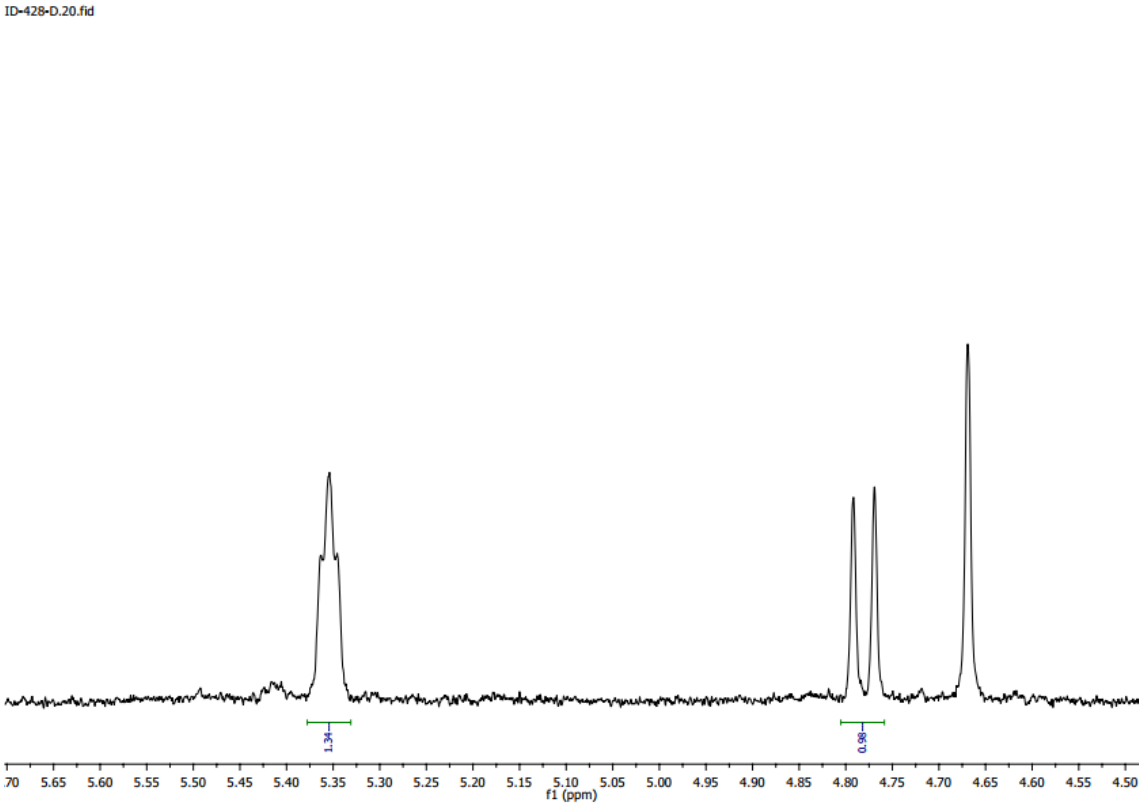

NMR traces for reaction **without water, with 2-F-phenylboronic acid (400 MHz,** $CDCl_3$**)**:

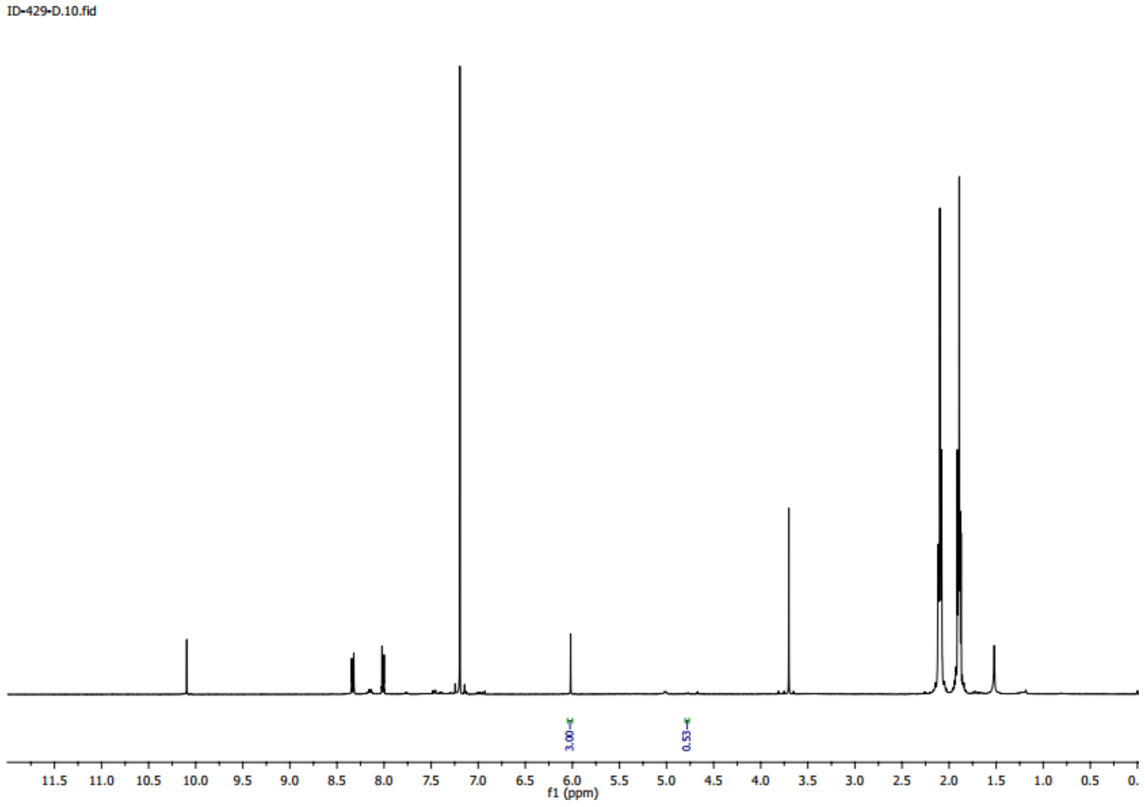

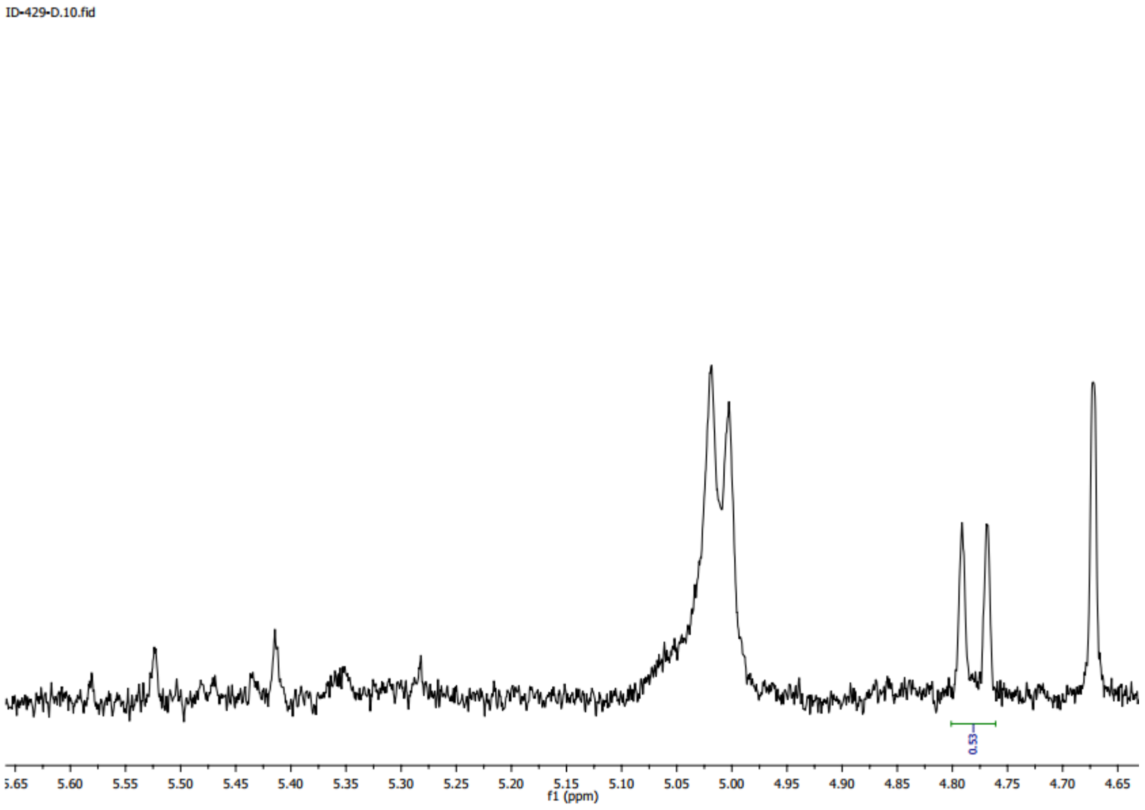

NMR traces for reaction **without water, with 2-F-phenylboronic acid (duplicate) (400 MHz,** CDCl$_3$**)**:

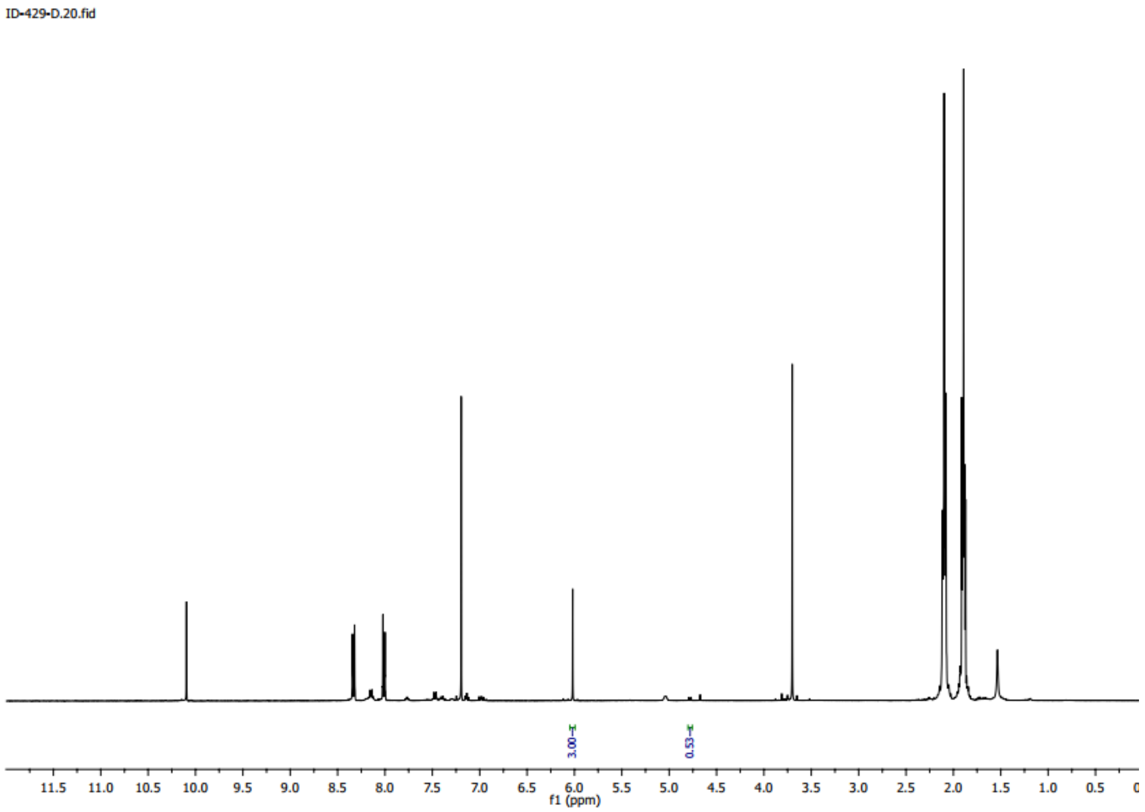

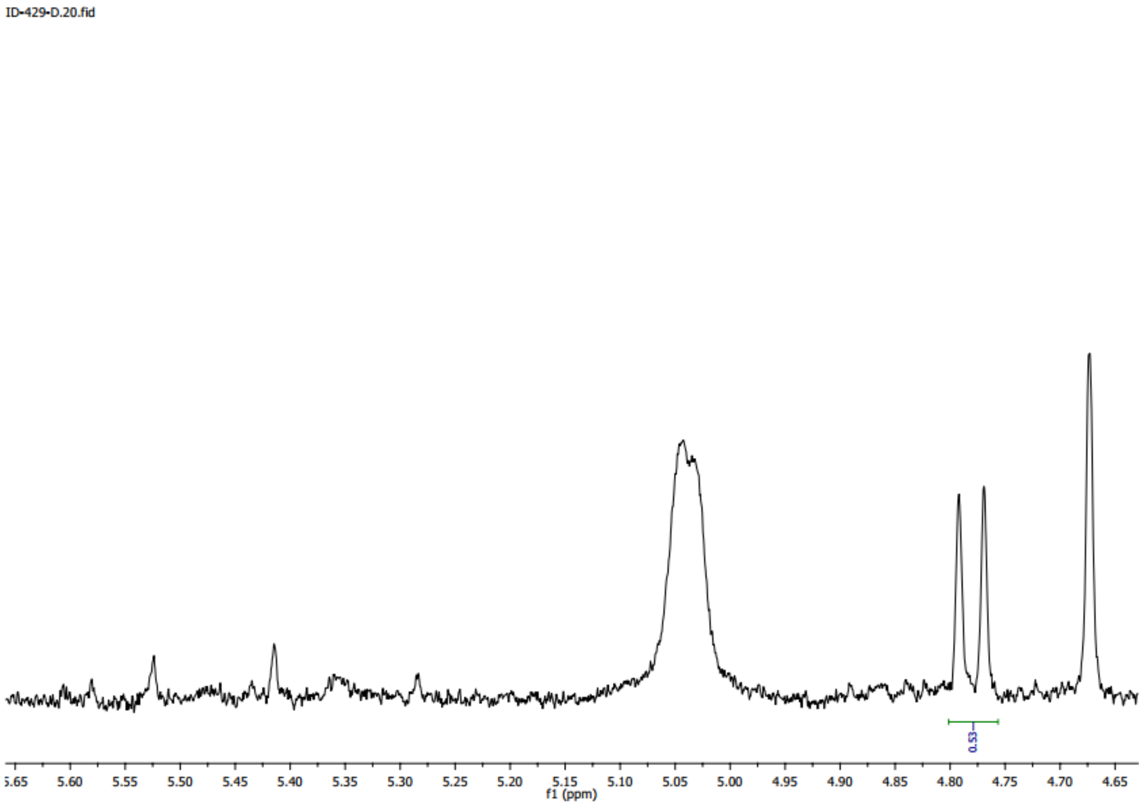

18.3. NMR Traces for Table S3. Boronic acid screening and blanks in neat cyclopentanone

NMR traces for reaction with: **3-F-phenylboronic acid (400 MHz,** $CDCl_3$**)**

//132.72.8.180/400/Bruker/TOPSPIN/data/anat/nmr/ID_345_1/1/fid

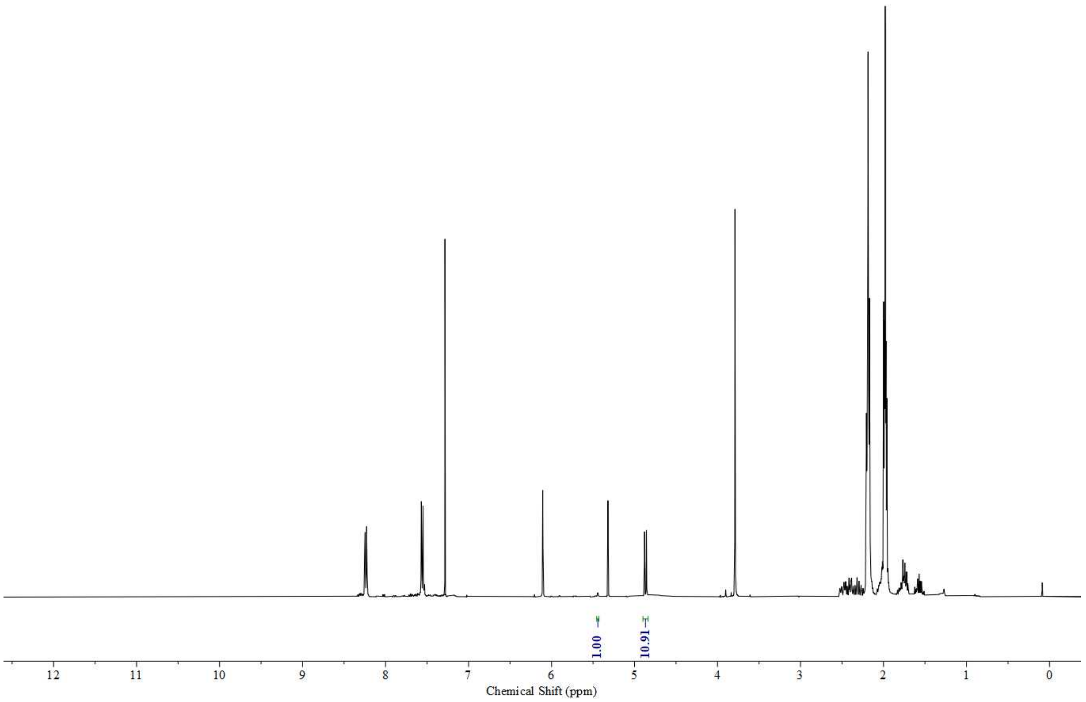

//132.72.8.180/400/Bruker/TOPSPIN/data/anat/nmr/ID_345_1/1/fid

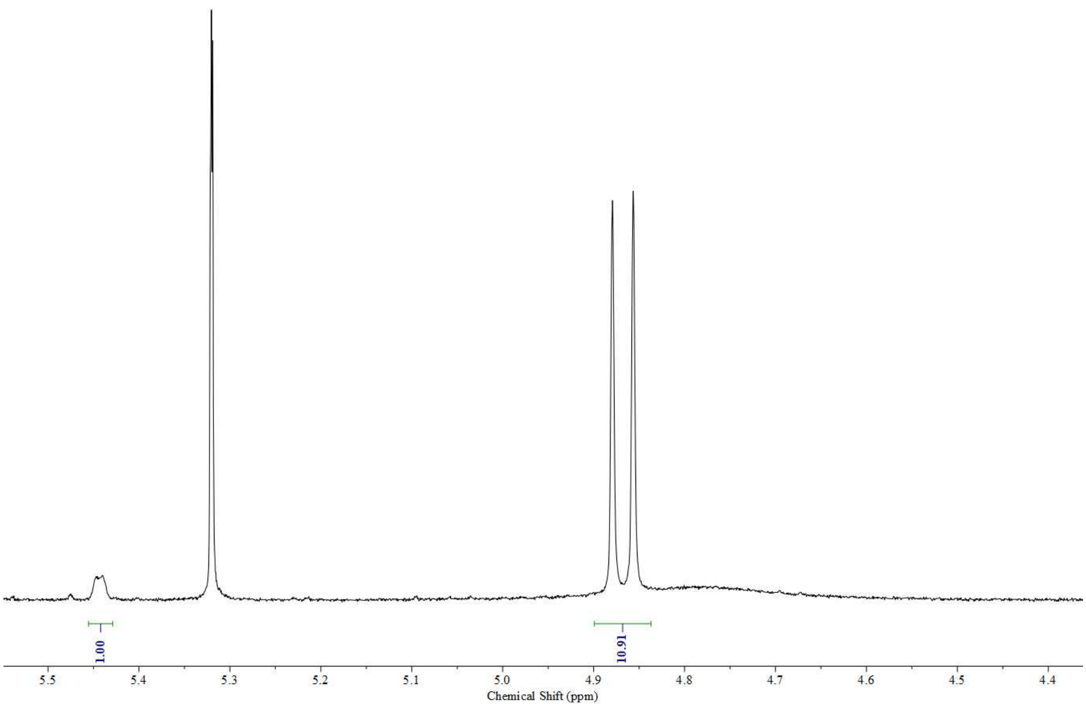

NMR traces for reaction with: **3-F-phenylboronic acid (duplicate) (400 MHz,** $CDCl_3$**)**

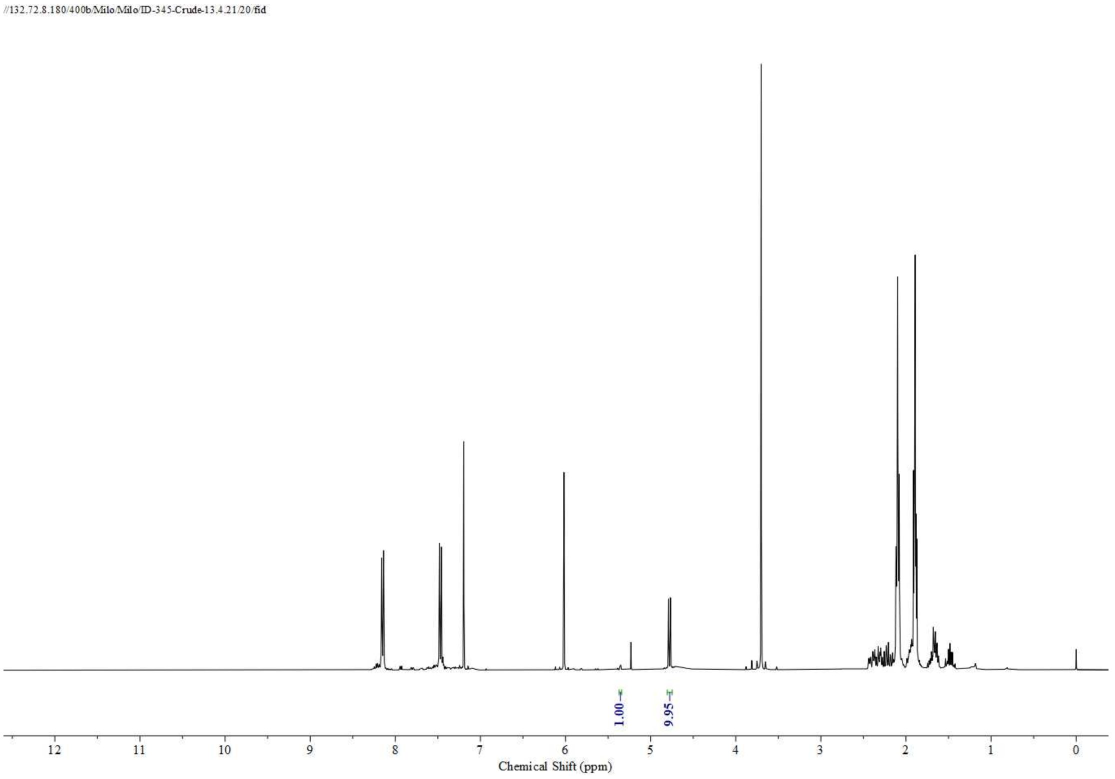

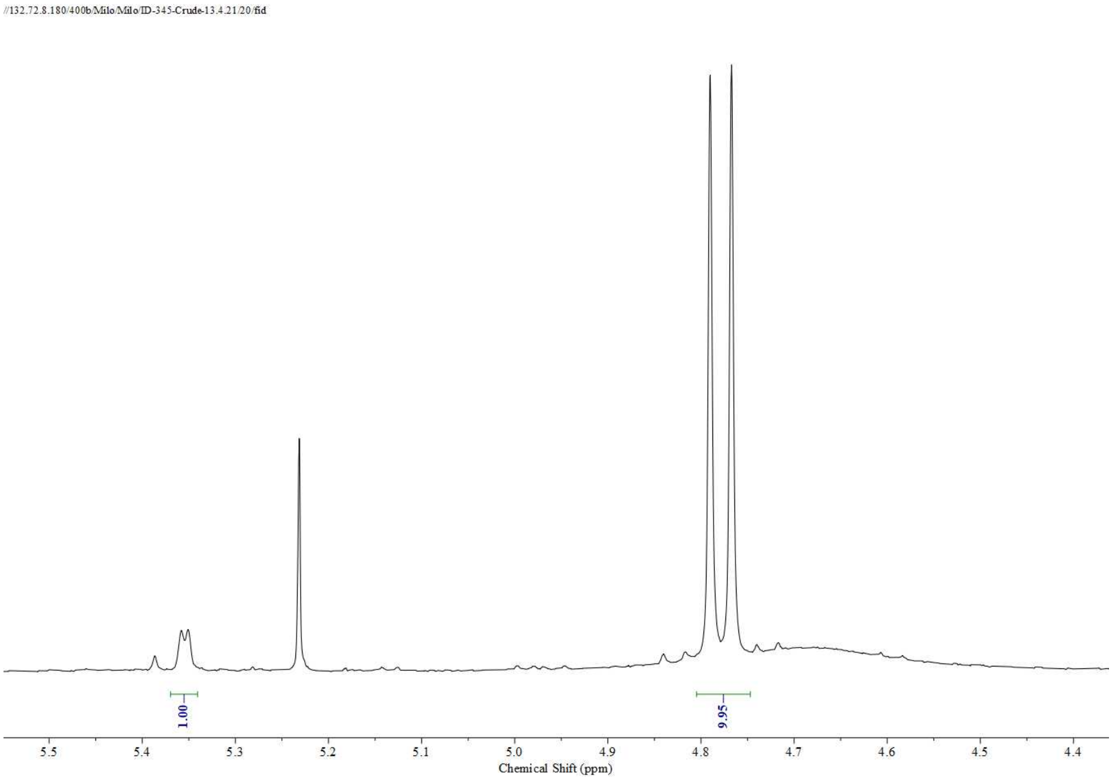

NMR traces for reaction with: **3,5-F-phenylboronic acid (400 MHz,** $CDCl_3$**)**

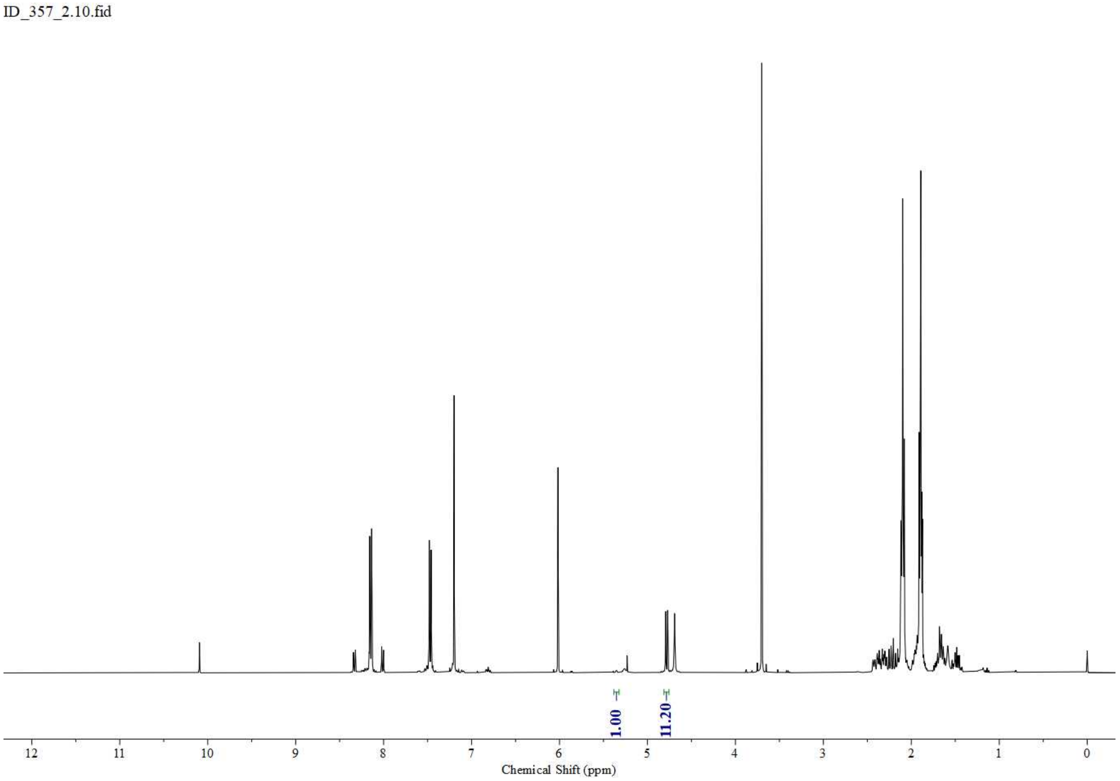

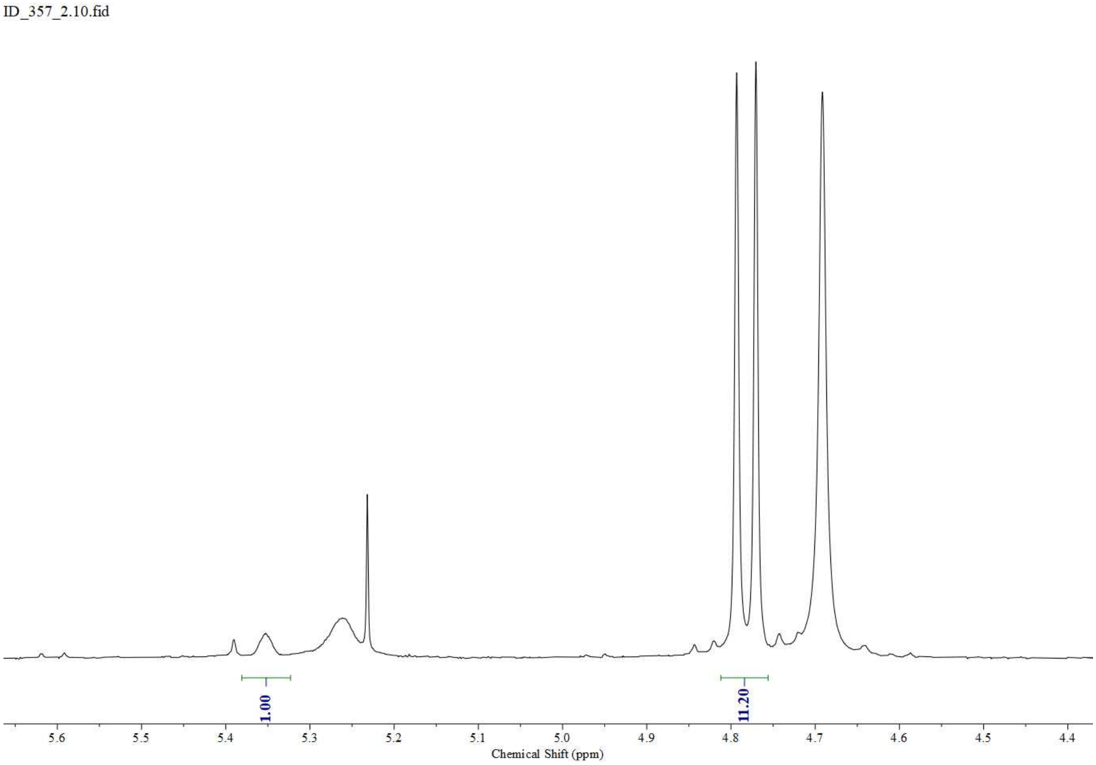

NMR traces for reaction with: **3,5-F-phenylboronic acid (duplicate) (400 MHz,** CDCl$_3$**)**

ID_357_2.10.fid

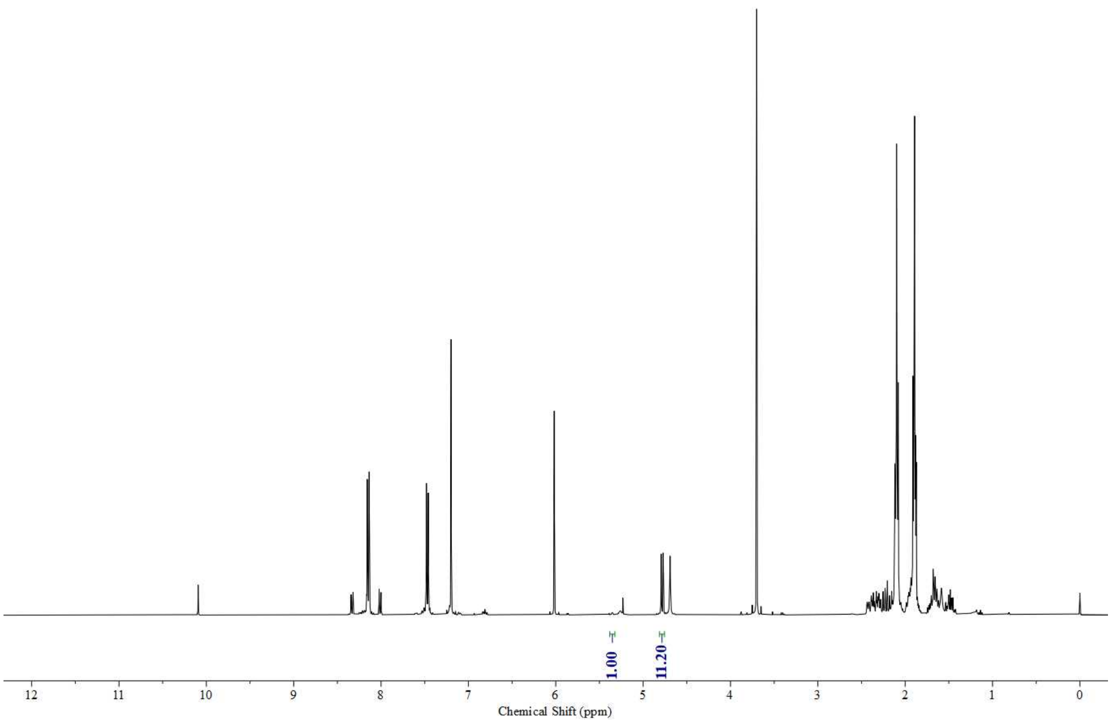

ID_357_1.10.fid

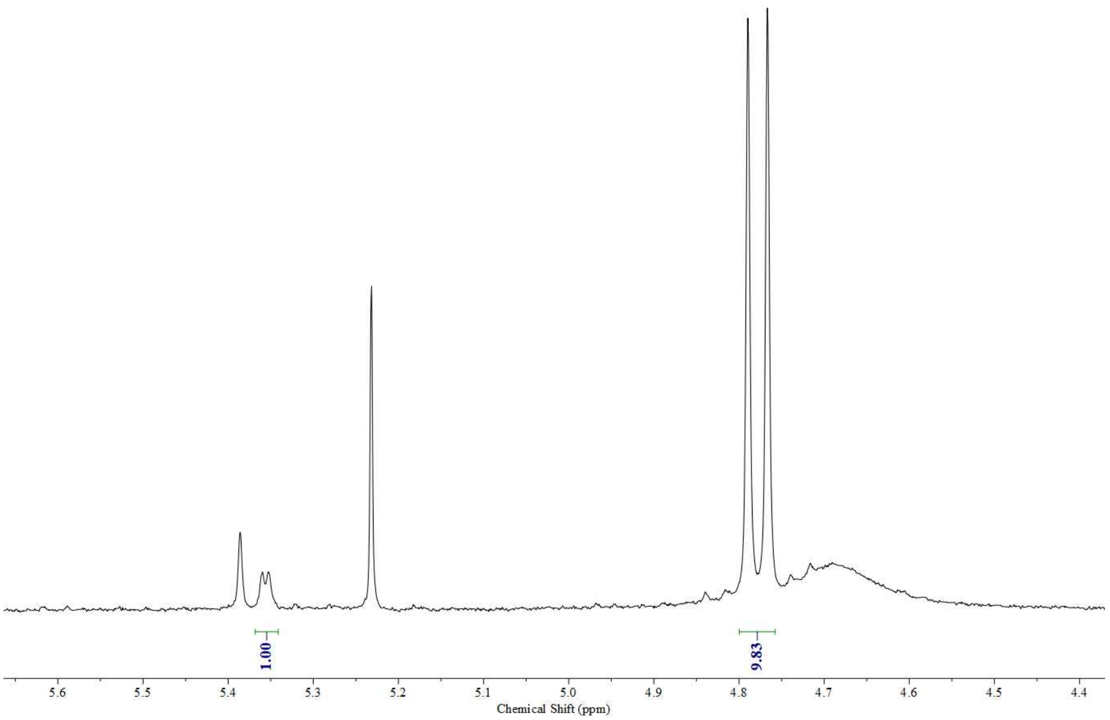

NMR traces for reaction with: **4-tBu-phenylboronic acid (400 MHz,** $CDCl_3$**)**

//132.72.8.180/400b/Milo/Milo/ID-340-Crude-13.4.21/10/fid

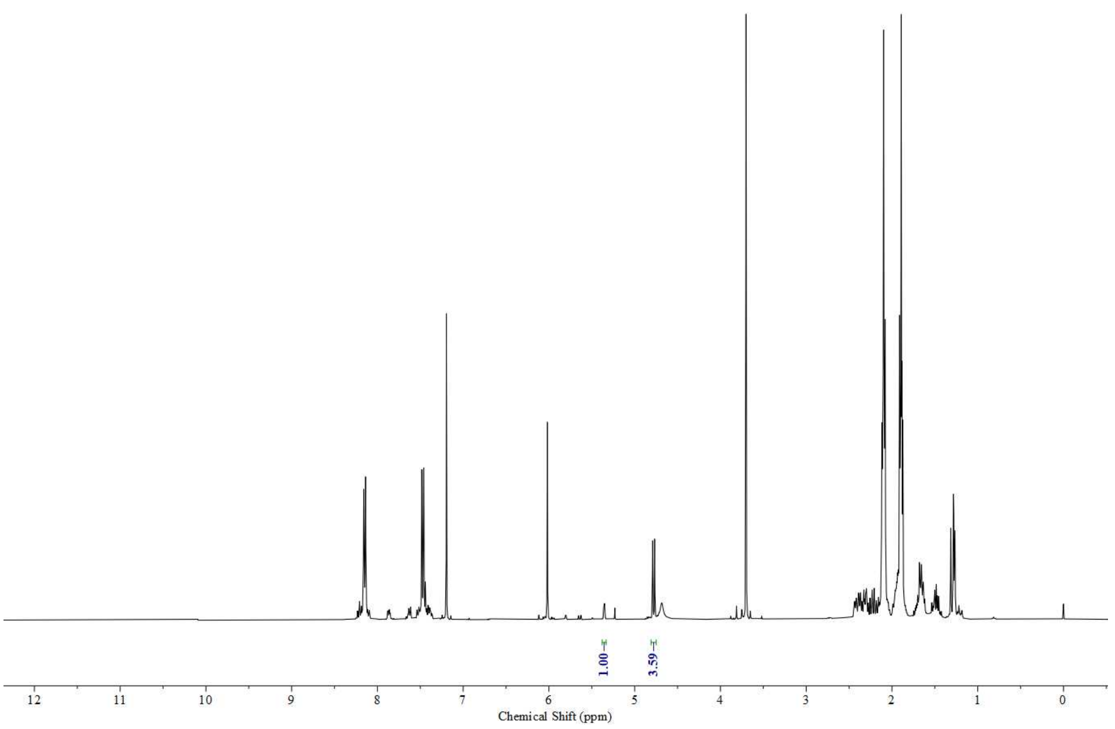

//132.72.8.180/400b/Milo/Milo/ID-340-Crude-13.4.21/10/fid

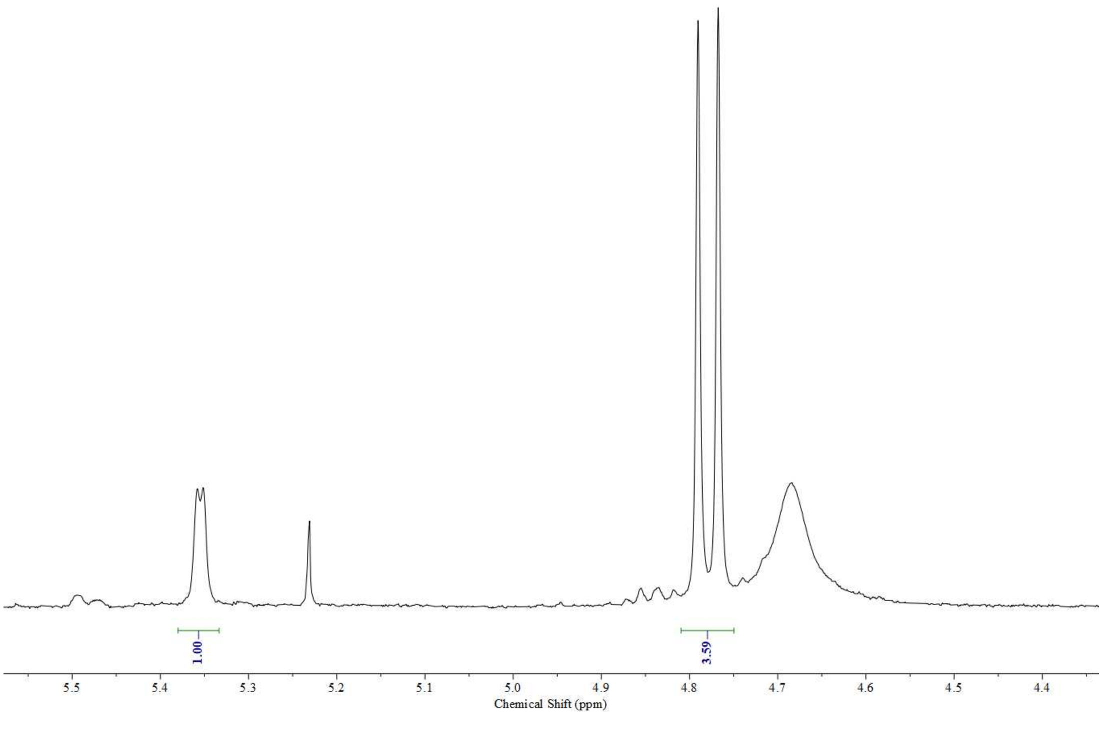

NMR traces for reaction with: **4-tBu-phenylboronic acid (duplicate) (400 MHz,** CDCl$_3$**)**

//132.72.8.180/400b/Milo/Milo/ID-340-Crude-13.4.21/20/fid

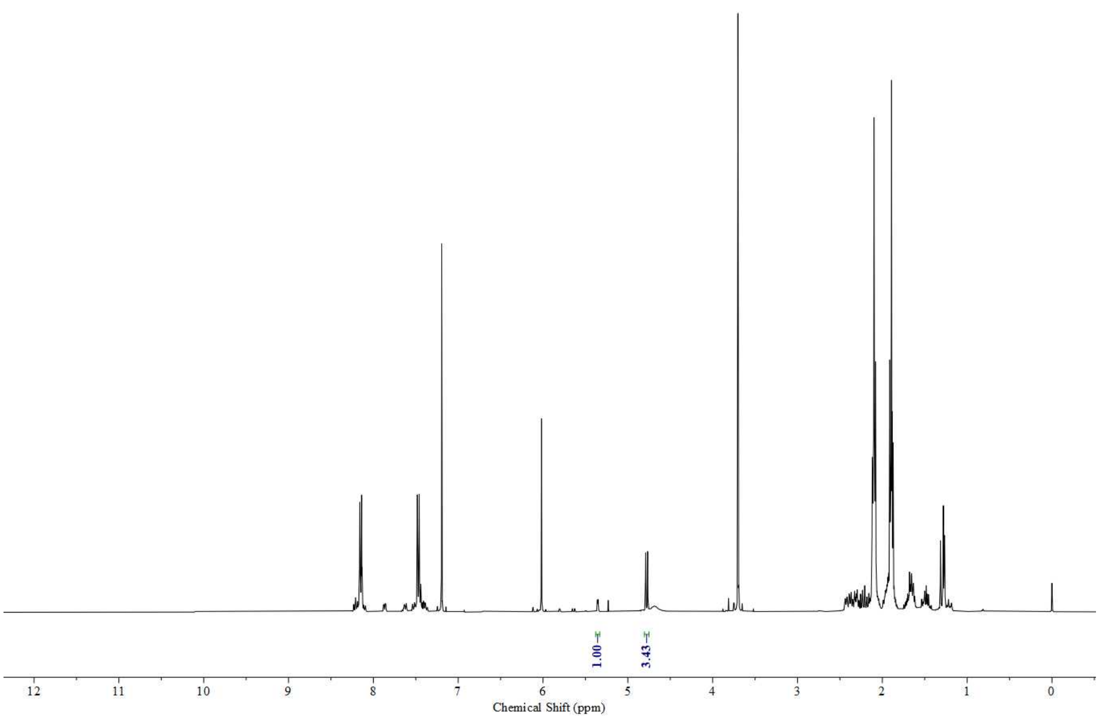

//132.72.8.180/400b/Milo/Milo/ID-340-Crude-13.4.21/20/fid

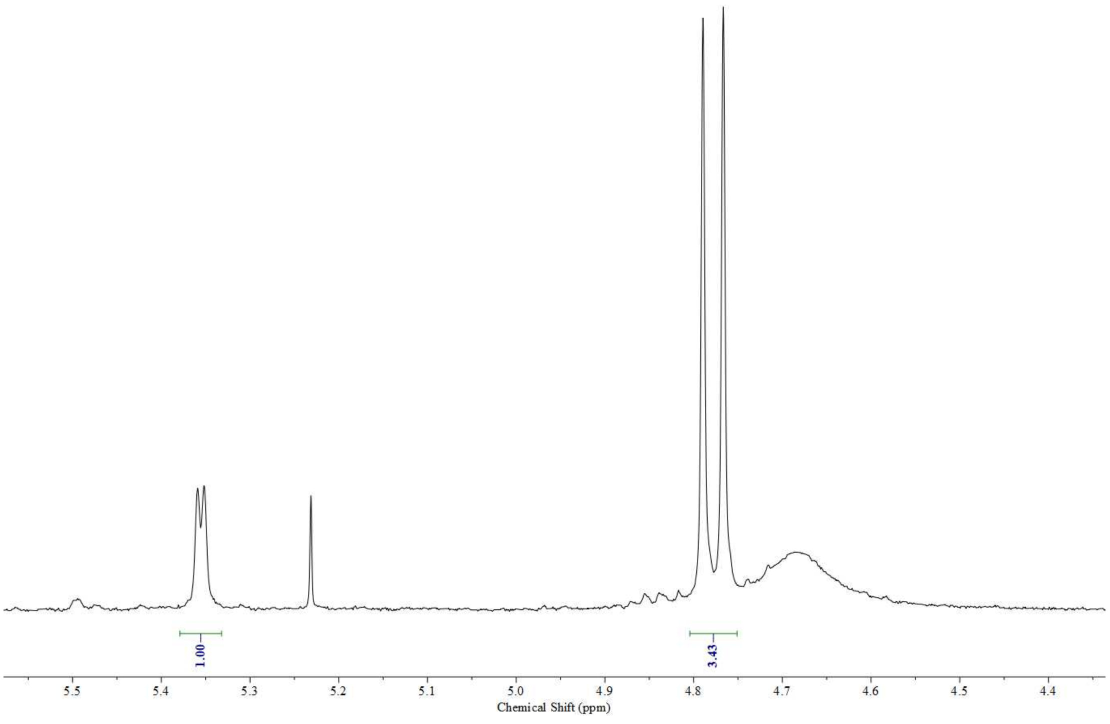

NMR traces for reaction with: **2,4-Me-phenylboronic acid (400 MHz,** $CDCl_3$**)**

ID_358_2.10.fid

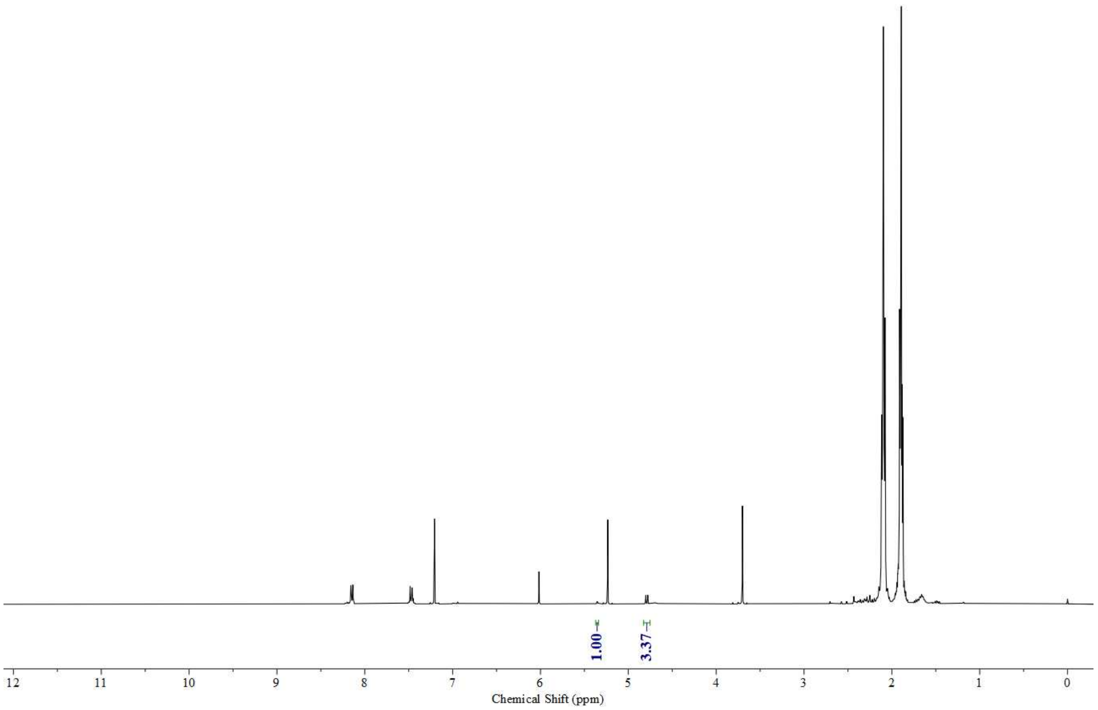

ID_358_2.10.fid

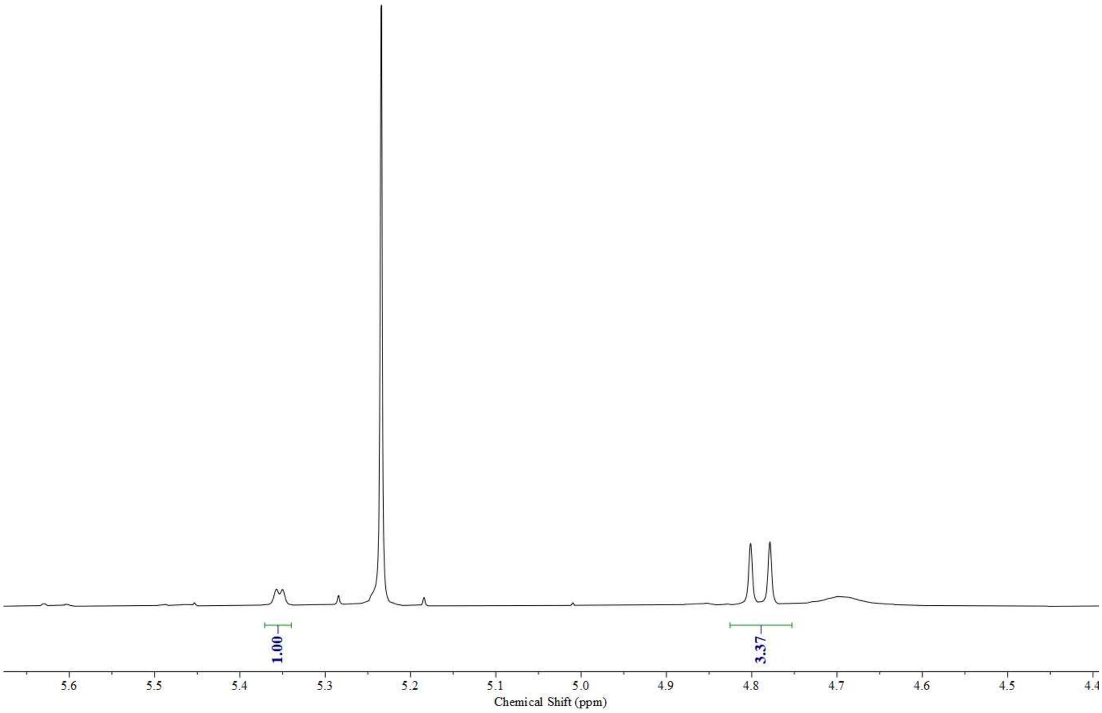

NMR traces for reaction with: **2,4-Me-phenylboronic acid (duplicate) (400 MHz,** $CDCl_3$**)**

ID_358_1.10.fid

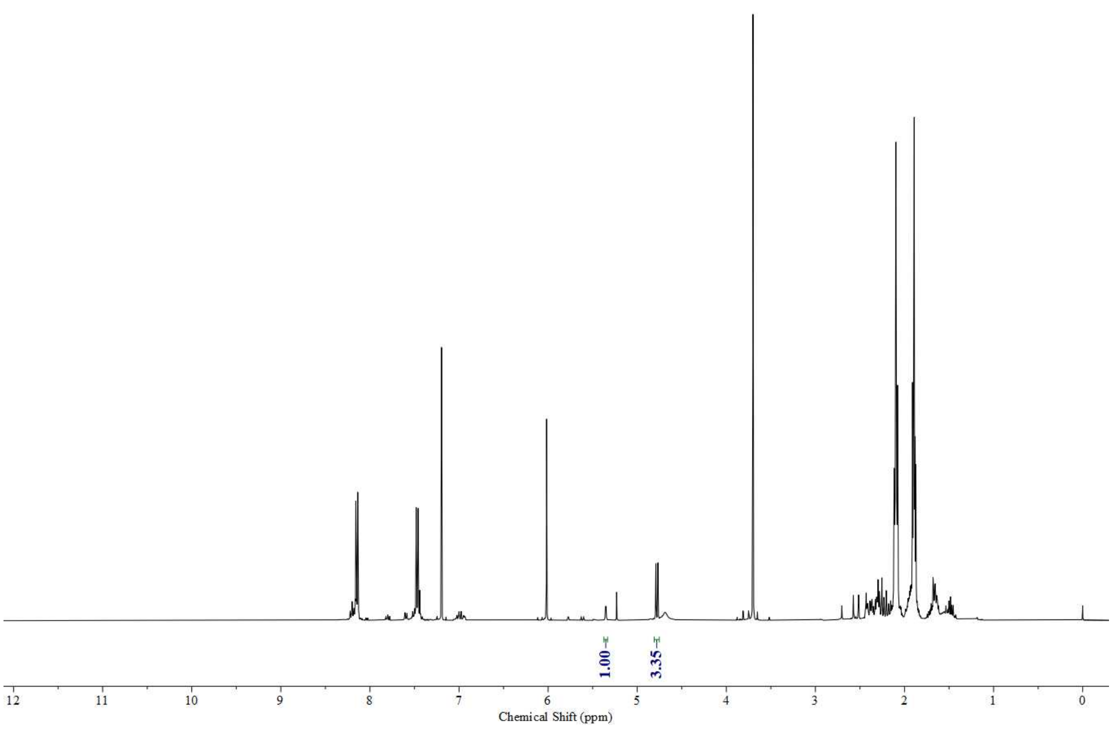

ID_358_1.10.fid

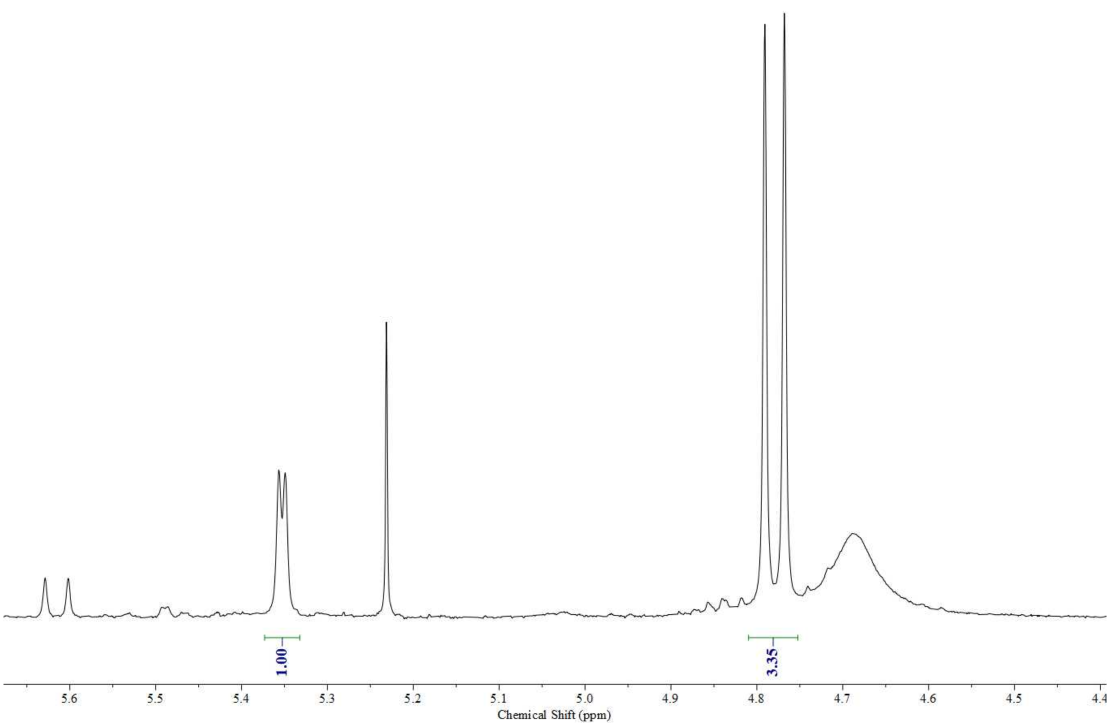

NMR traces for reaction with: **3,5-OMe-phenylboronic acid (400 MHz,** $CDCl_3$**)**

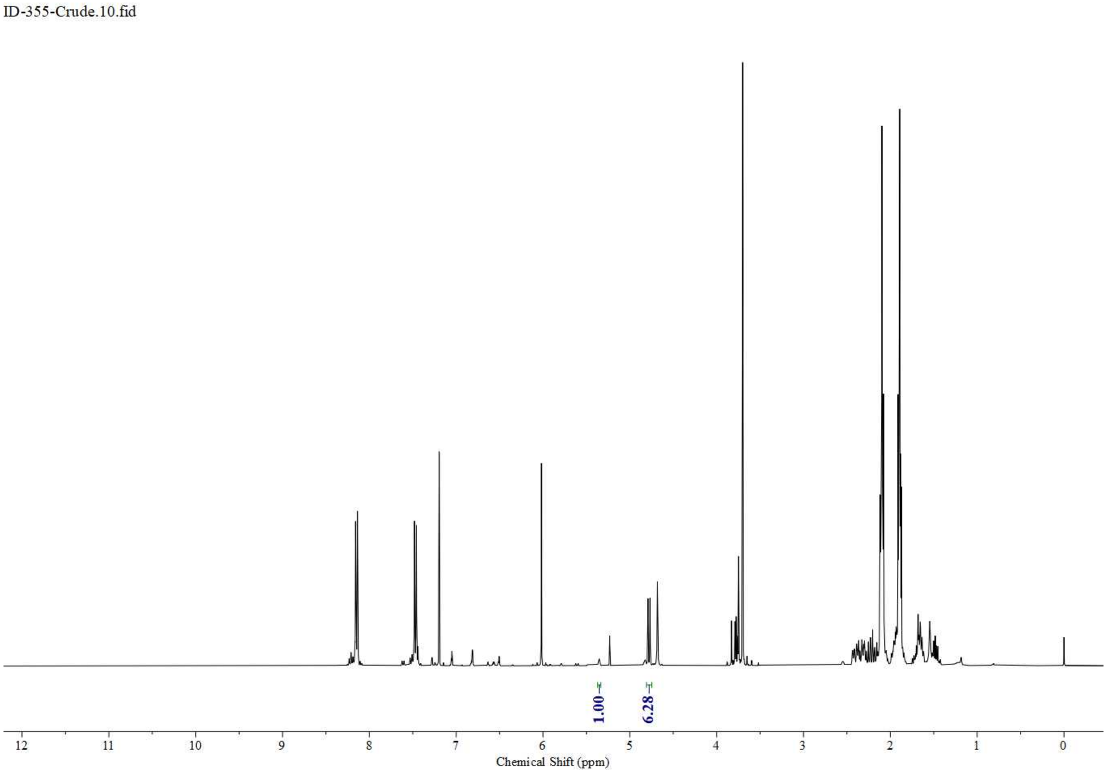

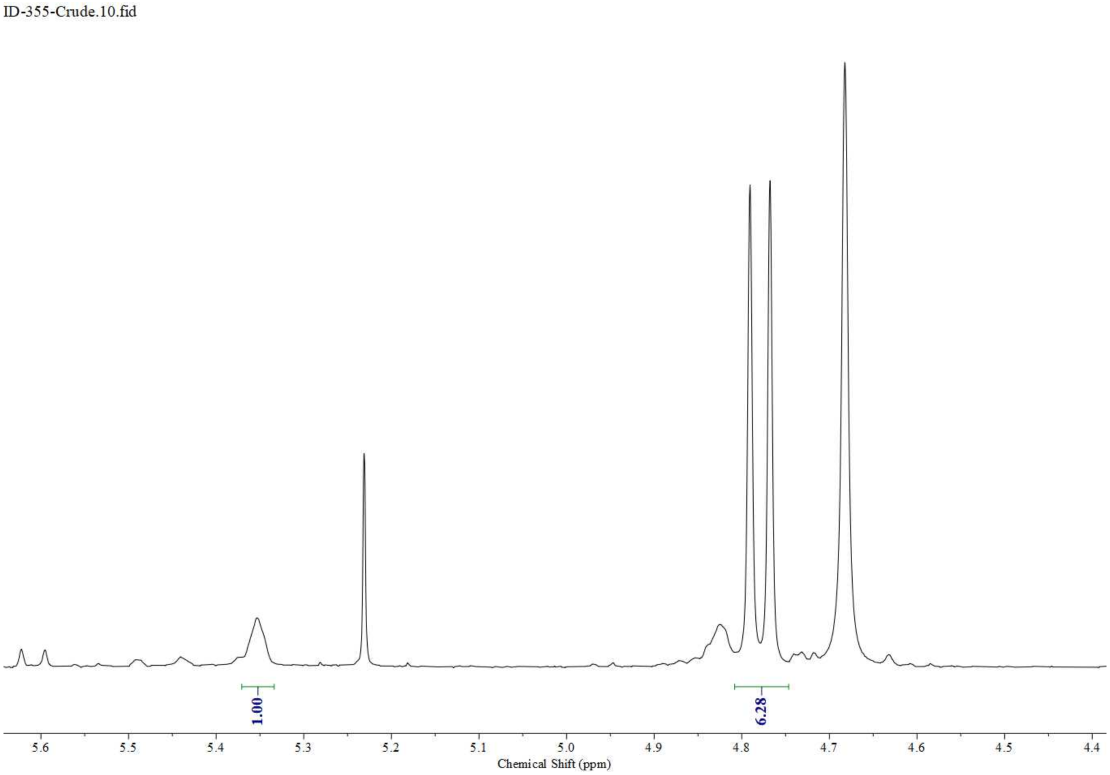

NMR traces for reaction with: **3,5-OMe-phenylboronic acid (duplicate) (400 MHz,** $CDCl_3$**)**

ID-355-Crude.20.fid

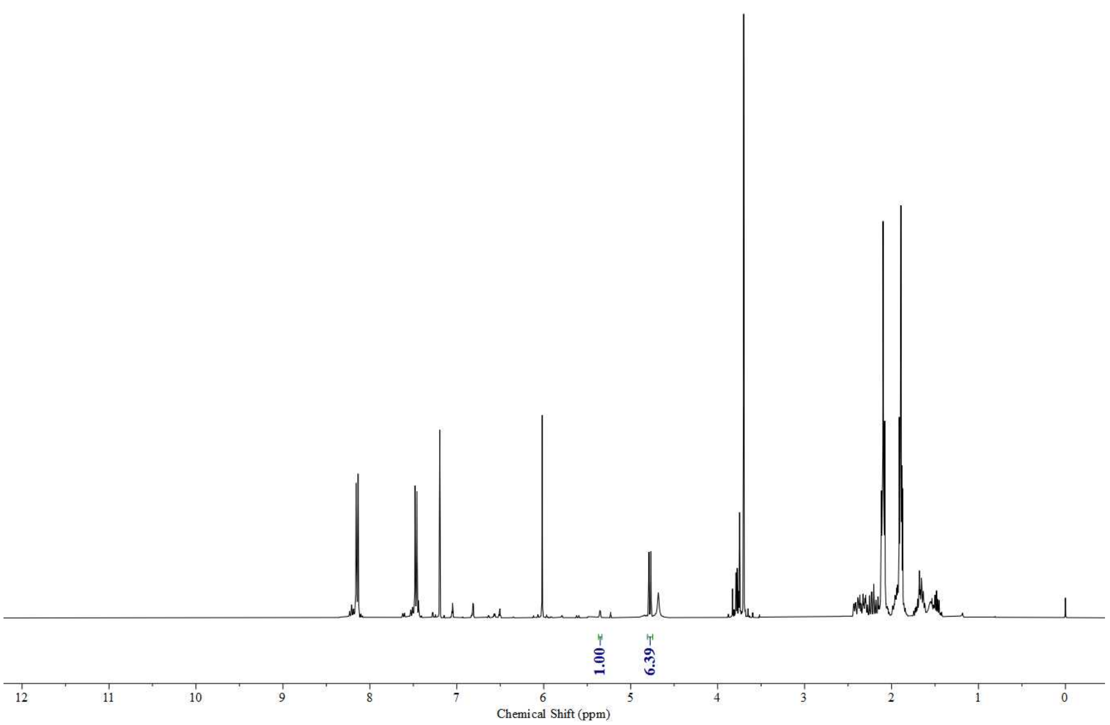

ID-355-Crude.20.fid

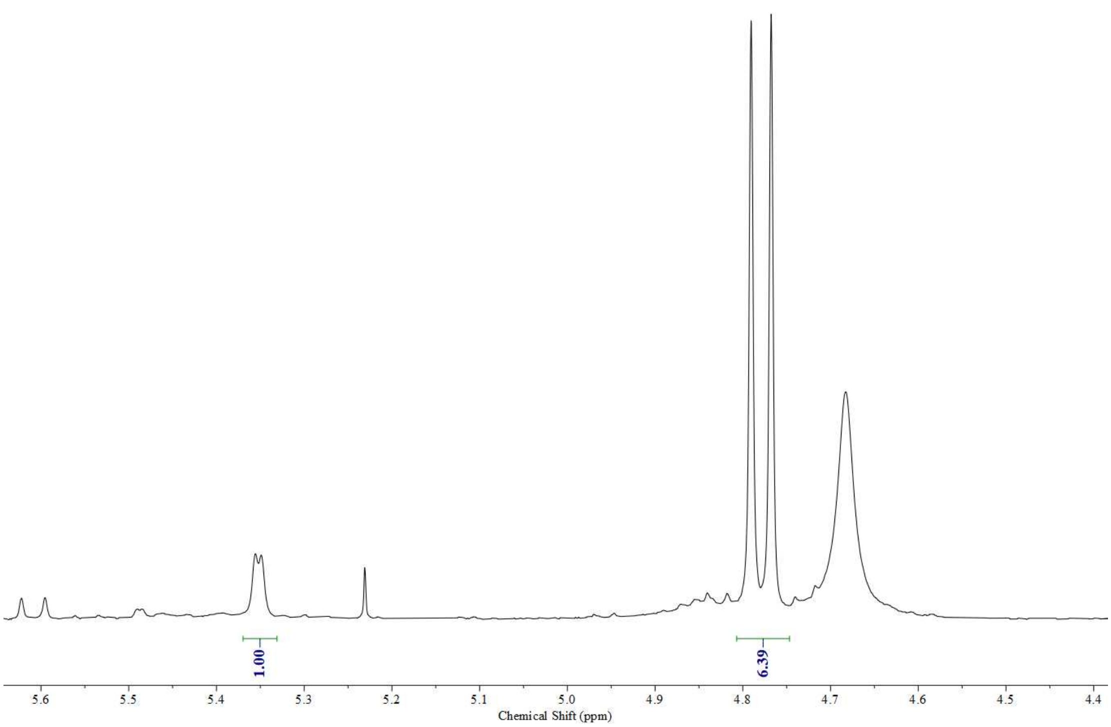

NMR traces for reaction with: **4-Me-phenylboronic acid (400 MHz,** $CDCl_3$**)**

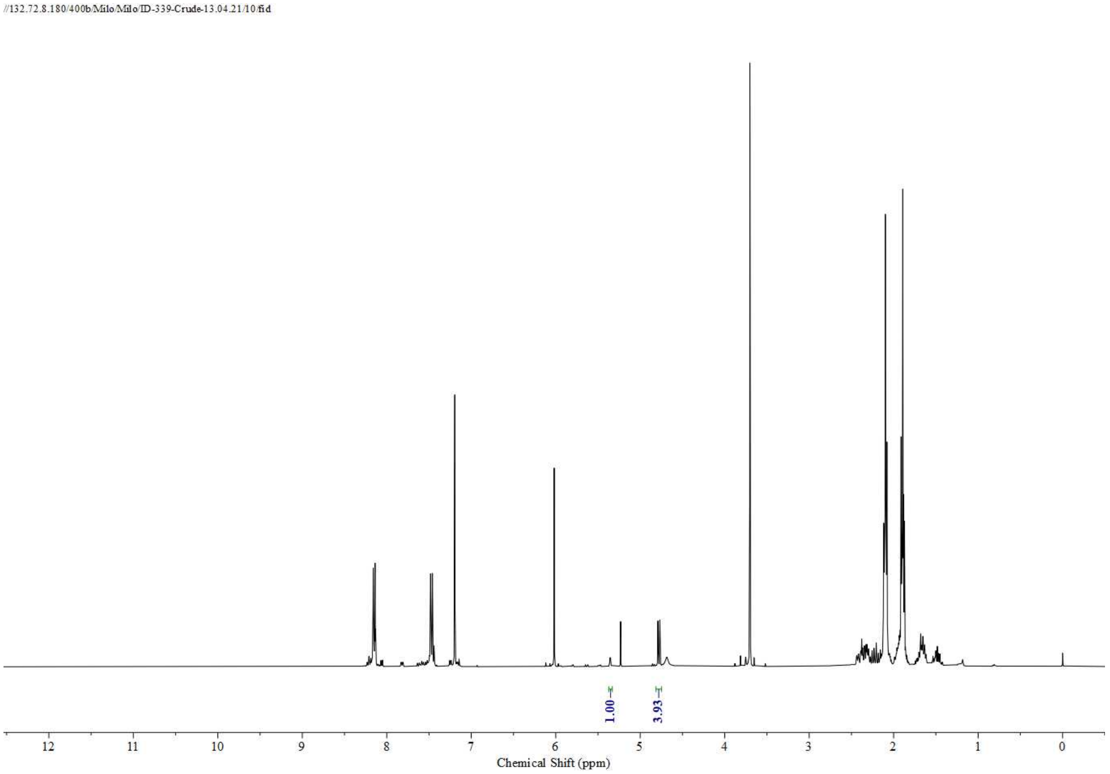

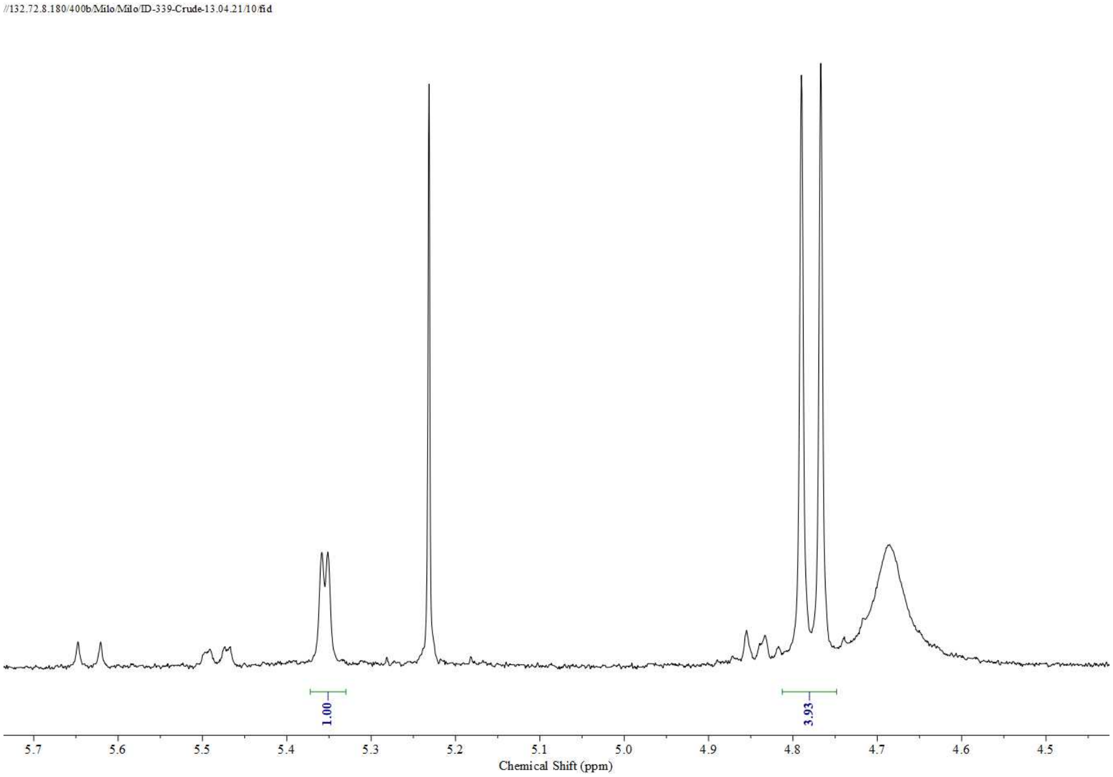

NMR traces for reaction with: **4-Me-phenylboronic acid (duplicate) (400 MHz,** $CDCl_3$**)**

//132.72.8.180/400b/Milo/Milo/ID-339-Crude-13.04.21/20/fid

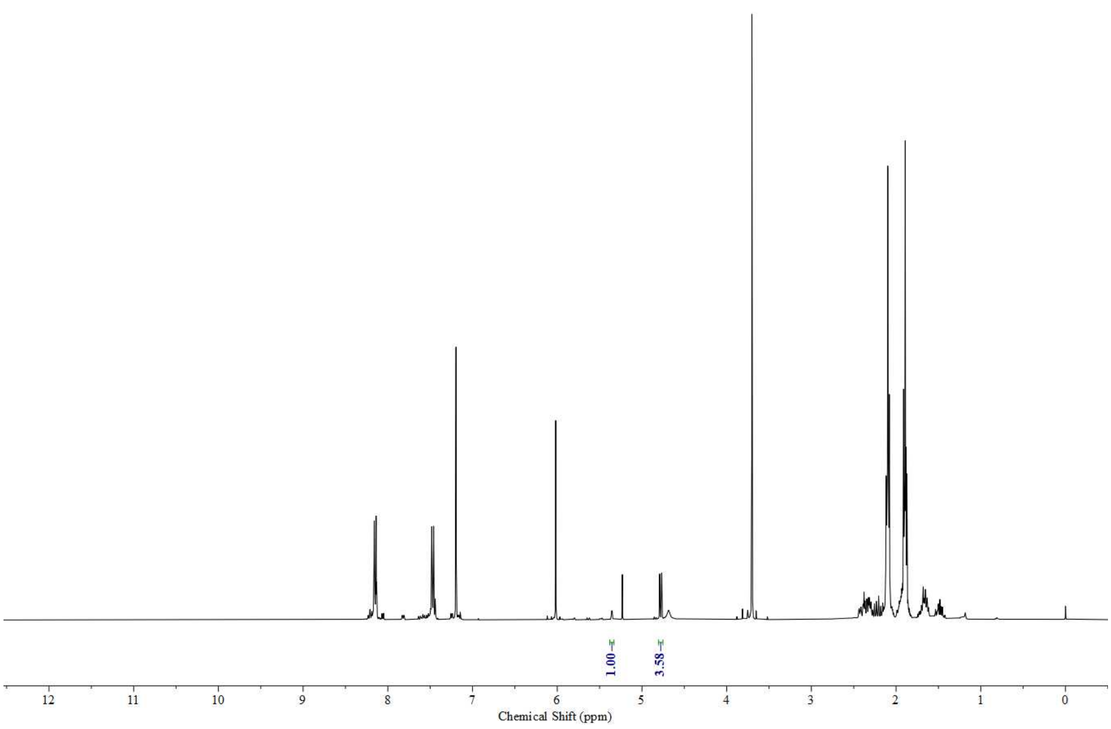

//132.72.8.180/400b/Milo/Milo/ID-339-Crude-13.04.21/10/fid

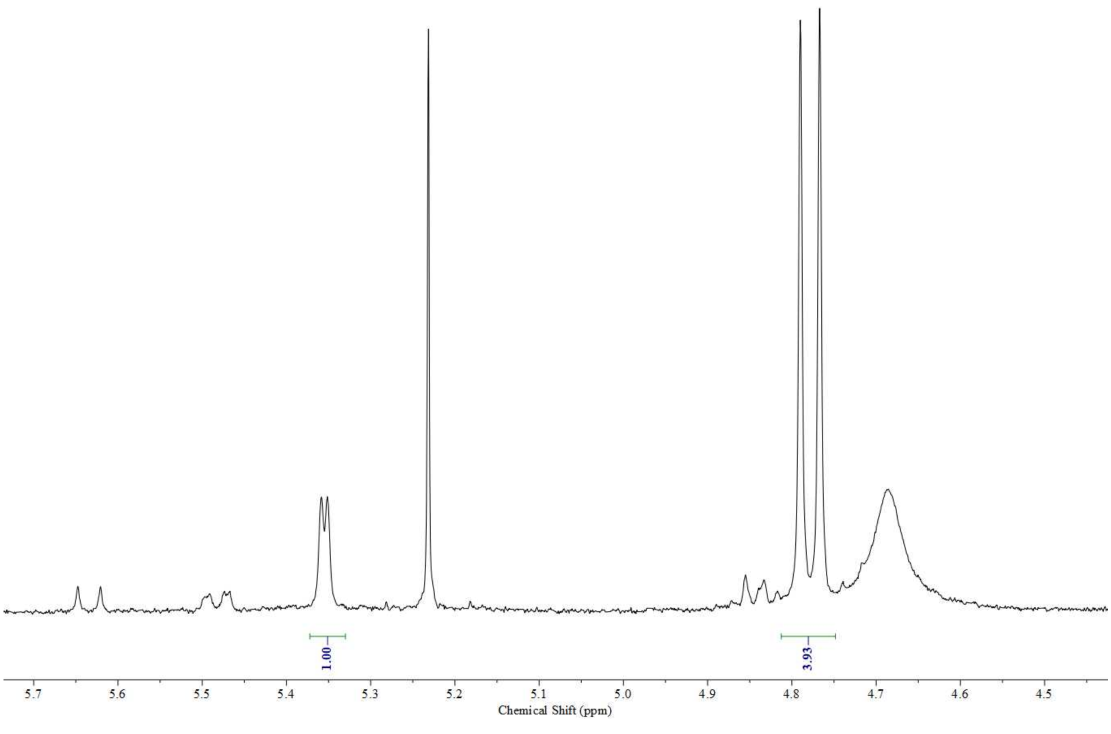

NMR traces for reaction with: **3-CF_3 -phenylboronic acid (400 MHz,** $CDCl_3$**)**

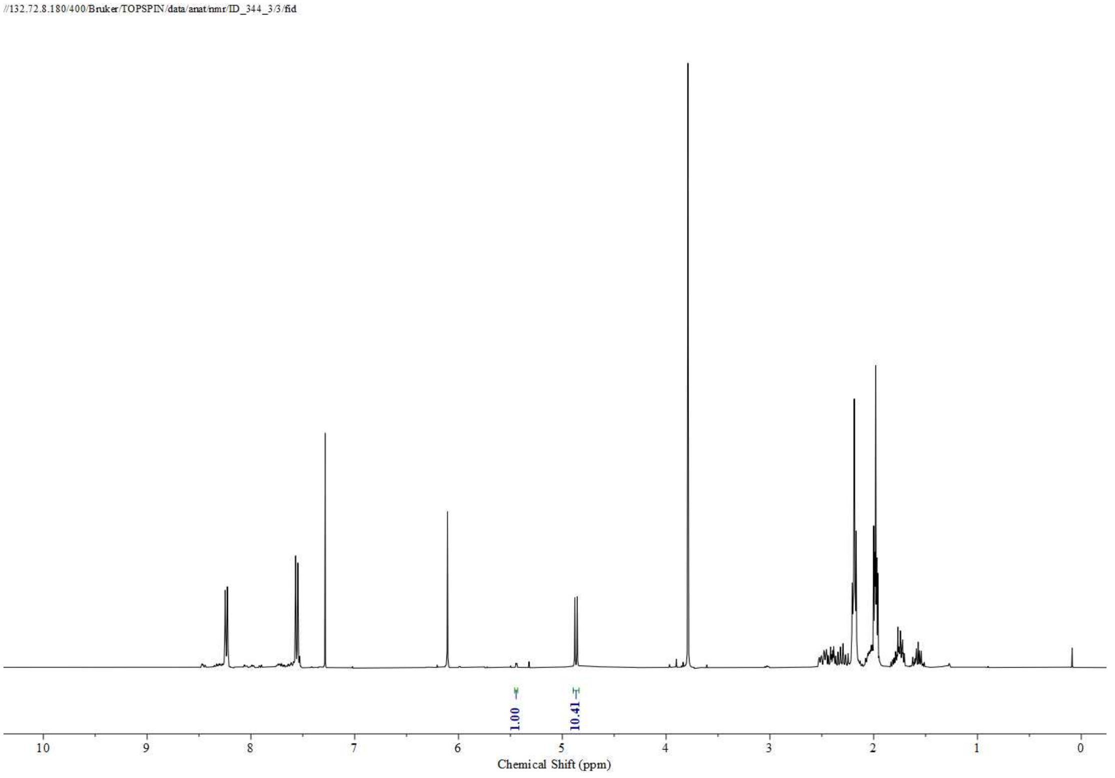

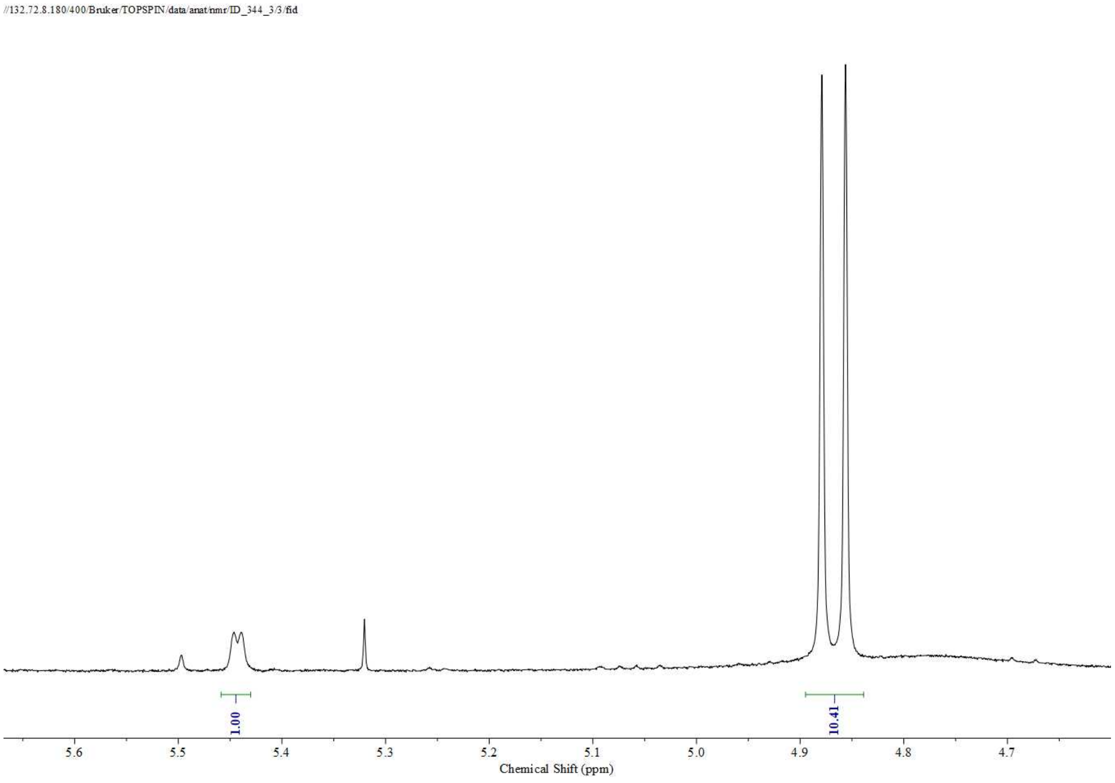

NMR traces for reaction with: **3-CF_3-phenylboronic acid (duplicate) (400 MHz,** $CDCl_3$**)**

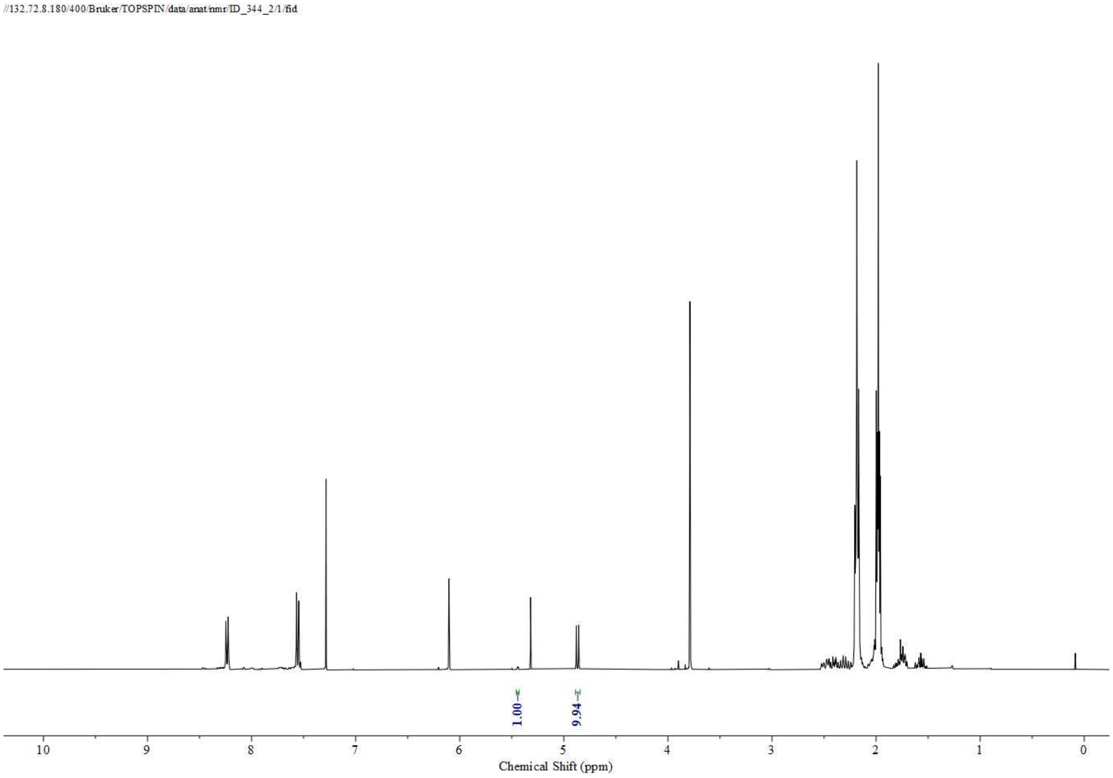

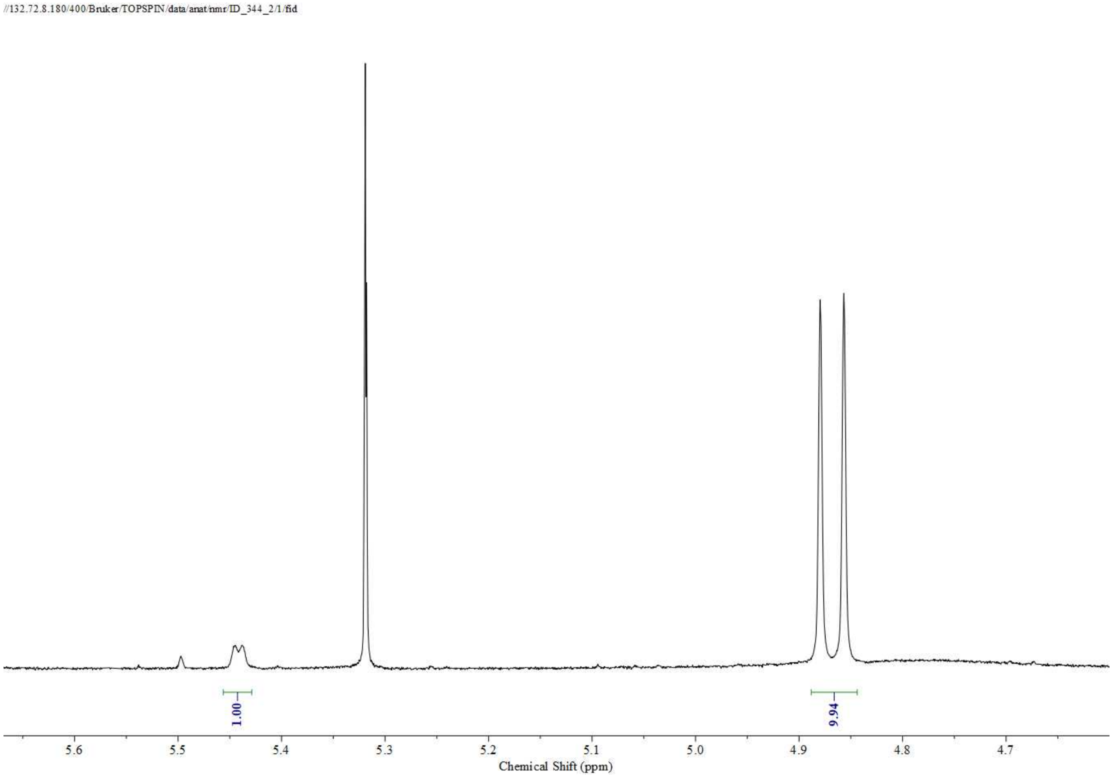

NMR traces for reaction with: **2-Naphthalene-phenylboronic acid (400 MHz,** $CDCl_3$**)**

ID-352-Crude.10.fid

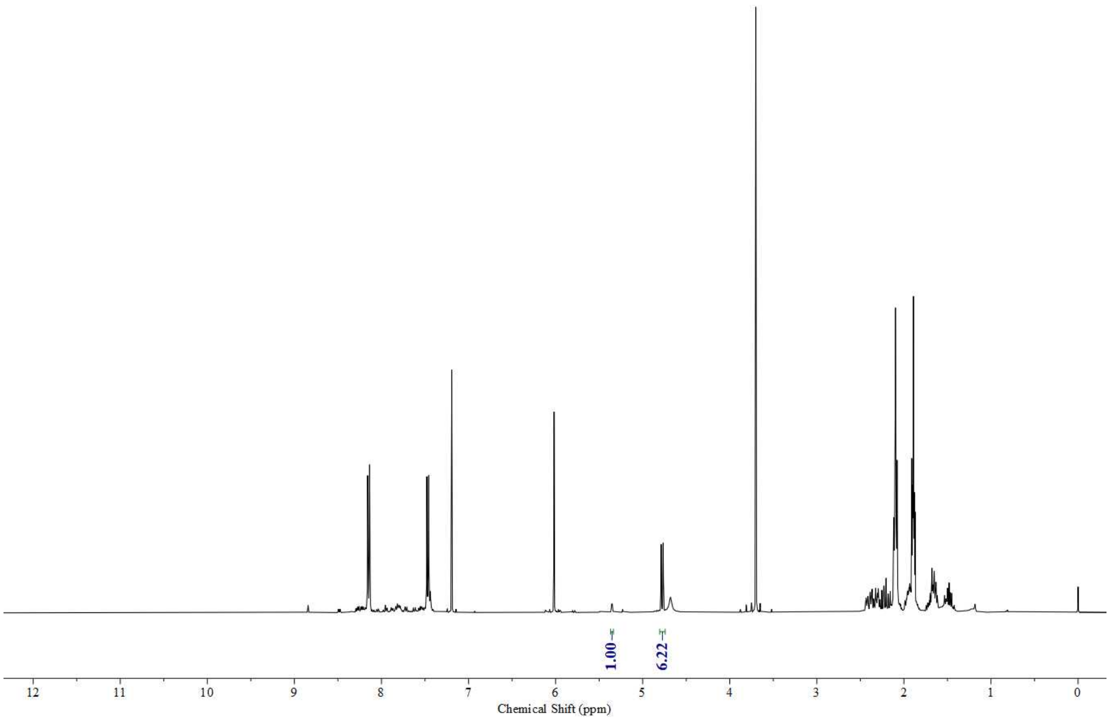

ID-352-Crude.10.fid

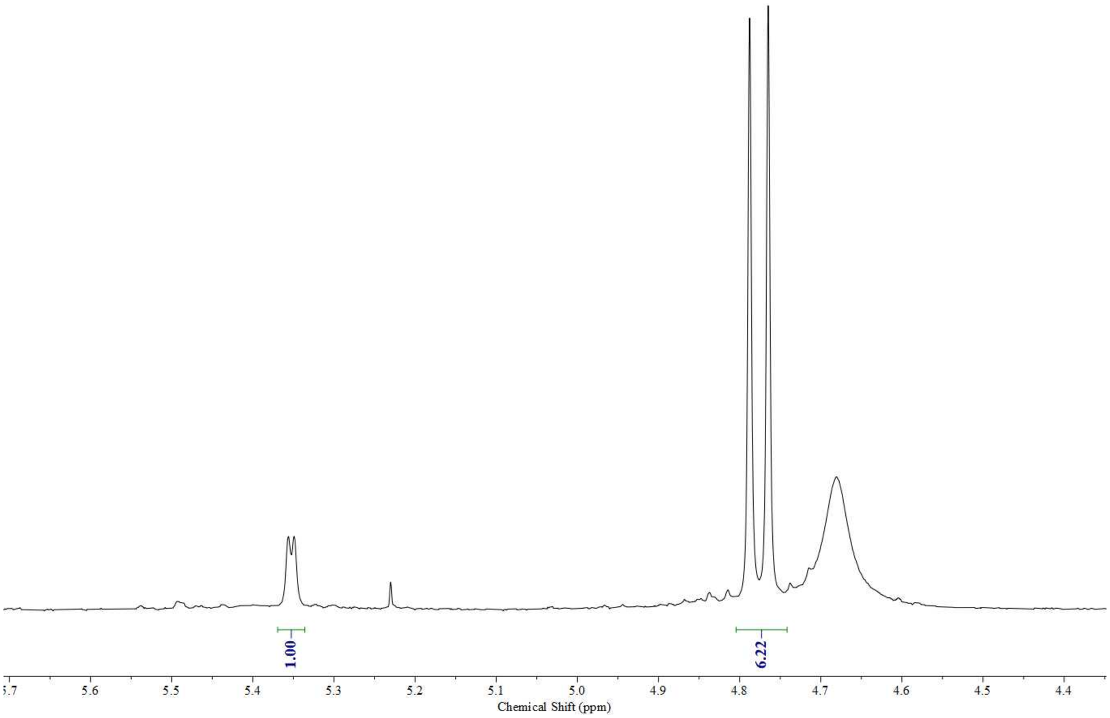

NMR traces for reaction with: **2-Naphthalene-phenylboronic acid** (duplicate) **(400 MHz,** CDCl$_3$**)**

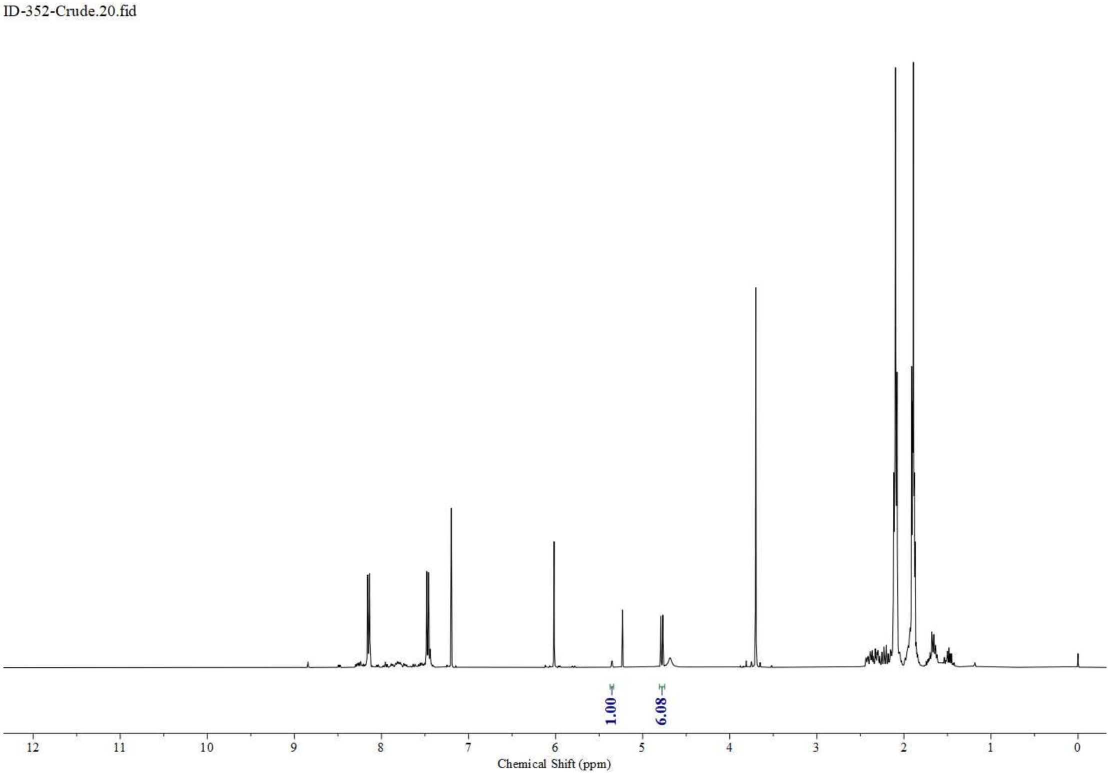

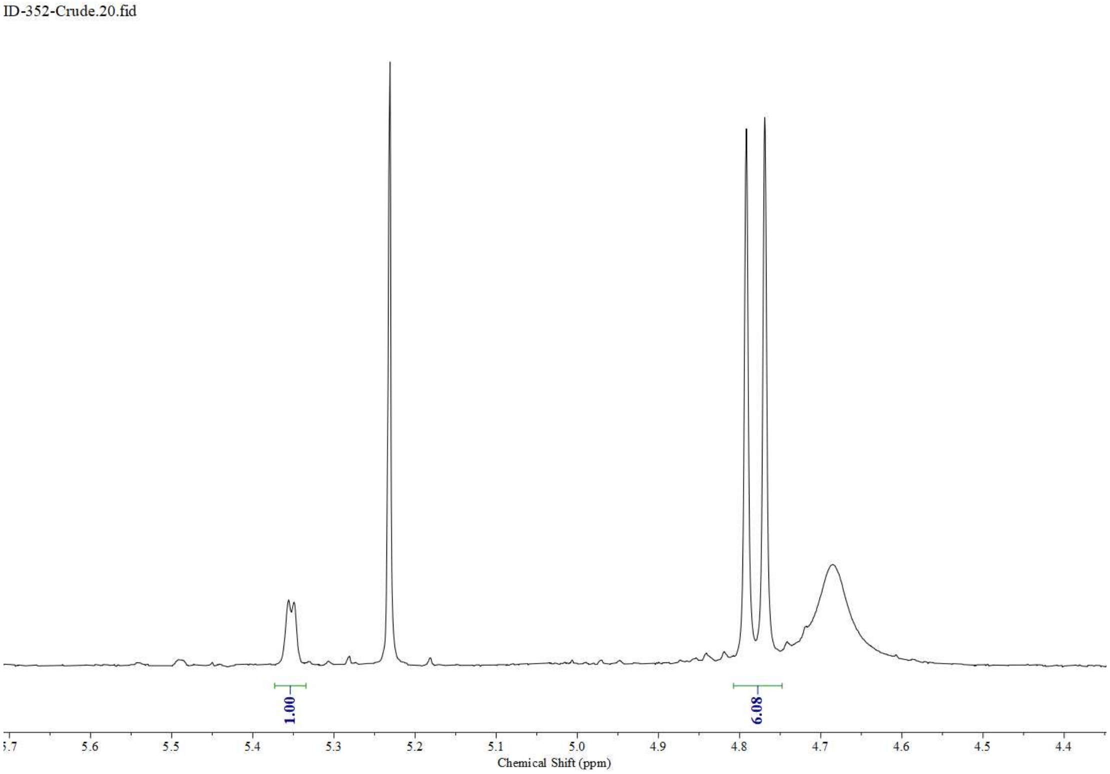

NMR traces for reaction with: **4-CF_3-phenylboronic acid (400 MHz,** $CDCl_3$**)**

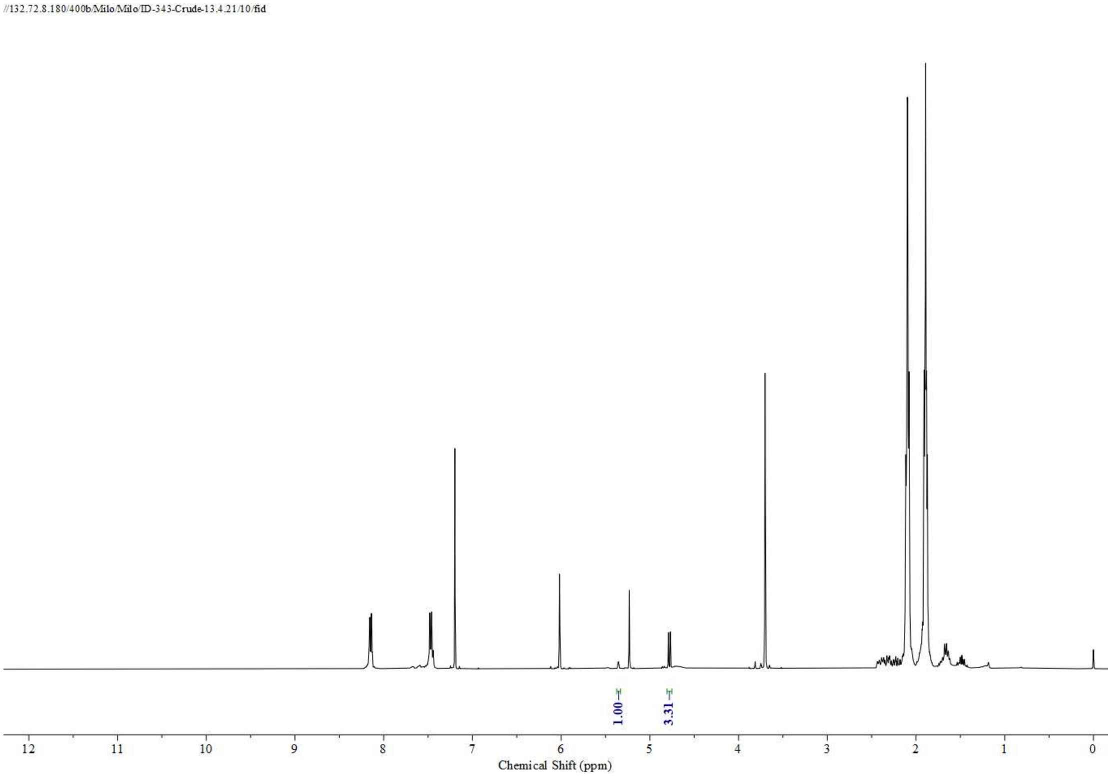

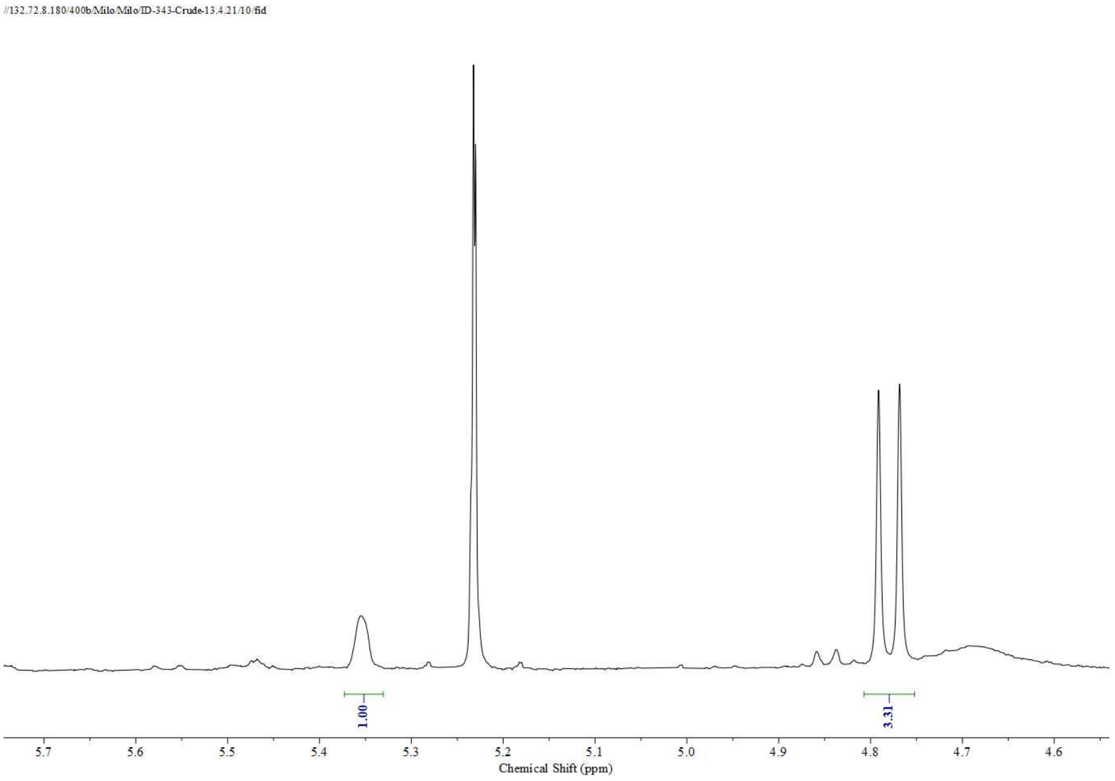

NMR traces for reaction with: **4-CF_3-phenylboronic acid (duplicate) (400 MHz,** $CDCl_3$**)**

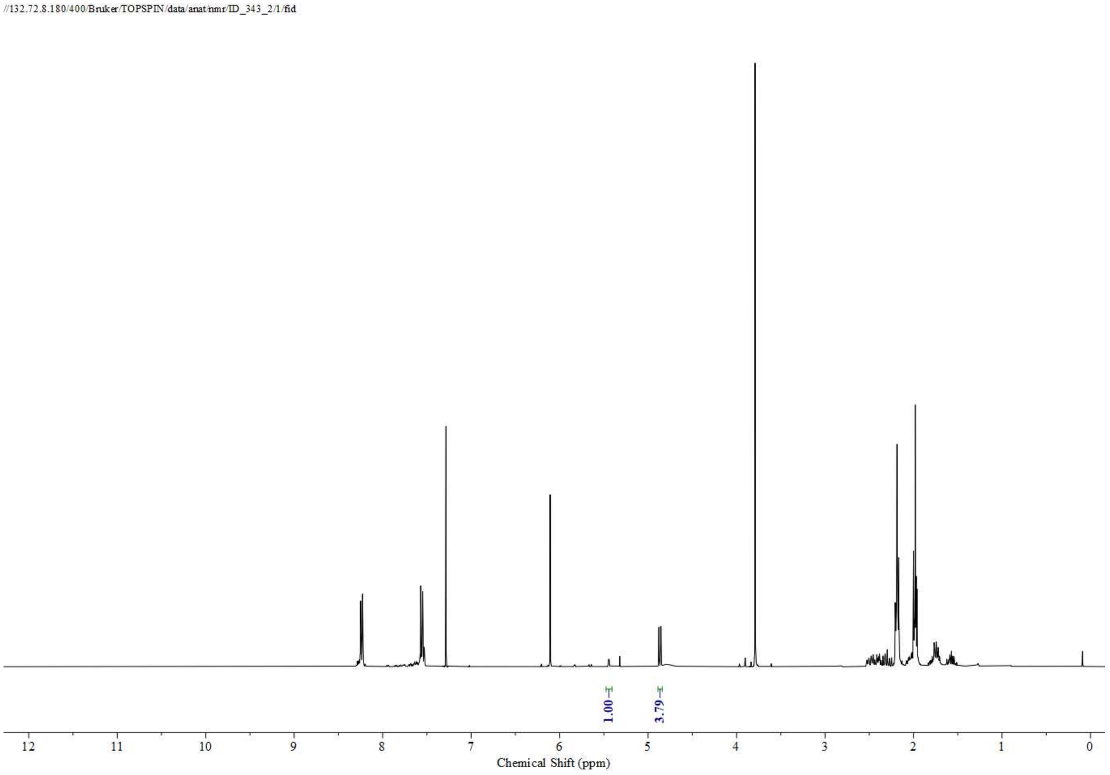

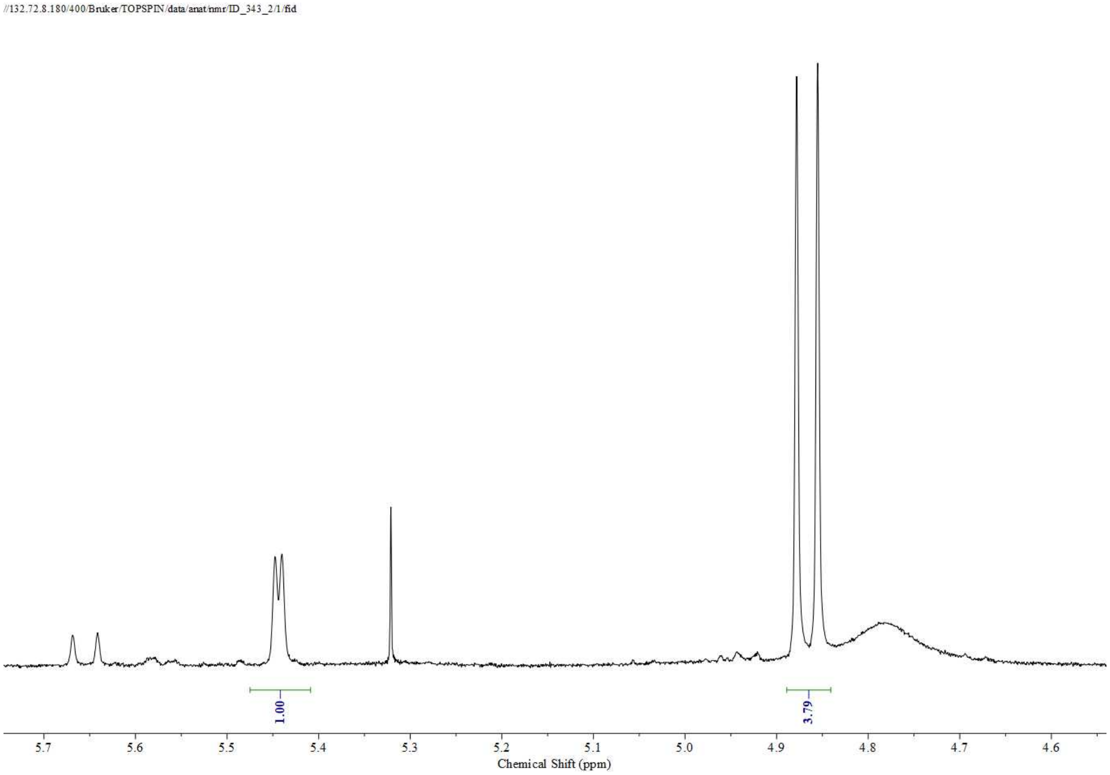

NMR traces for reaction with: **4-F-phenylboronic acid (400 MHz,** $CDCl_3$**)**

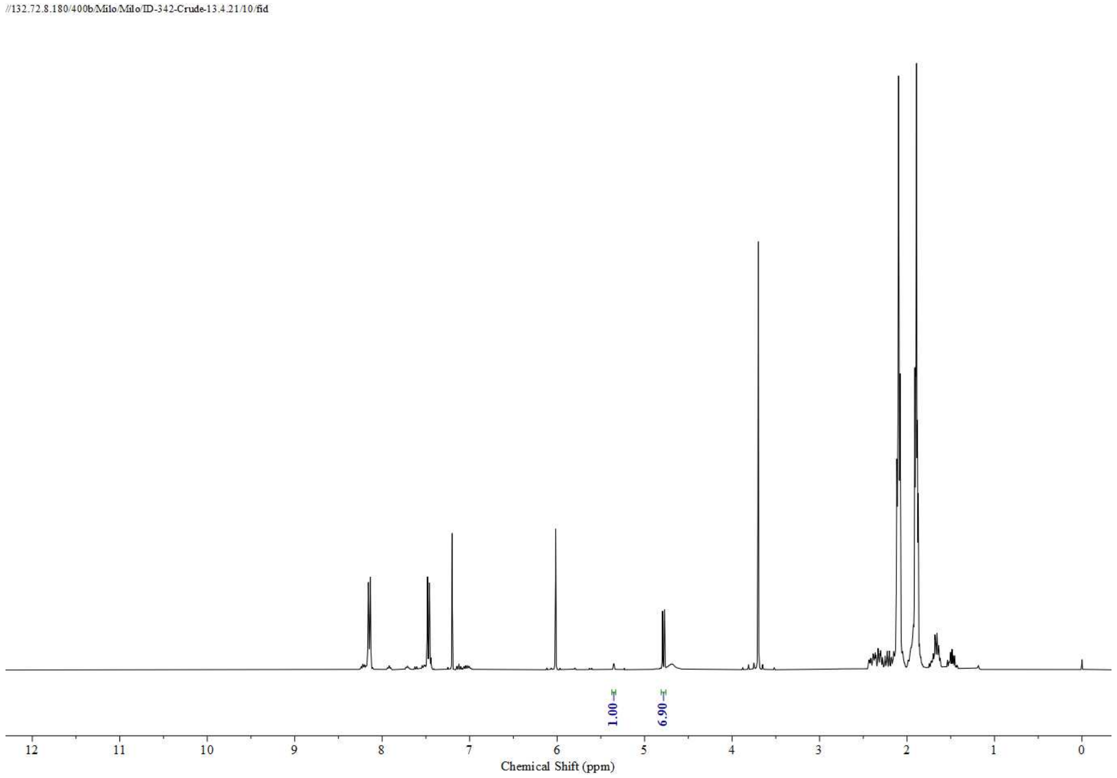

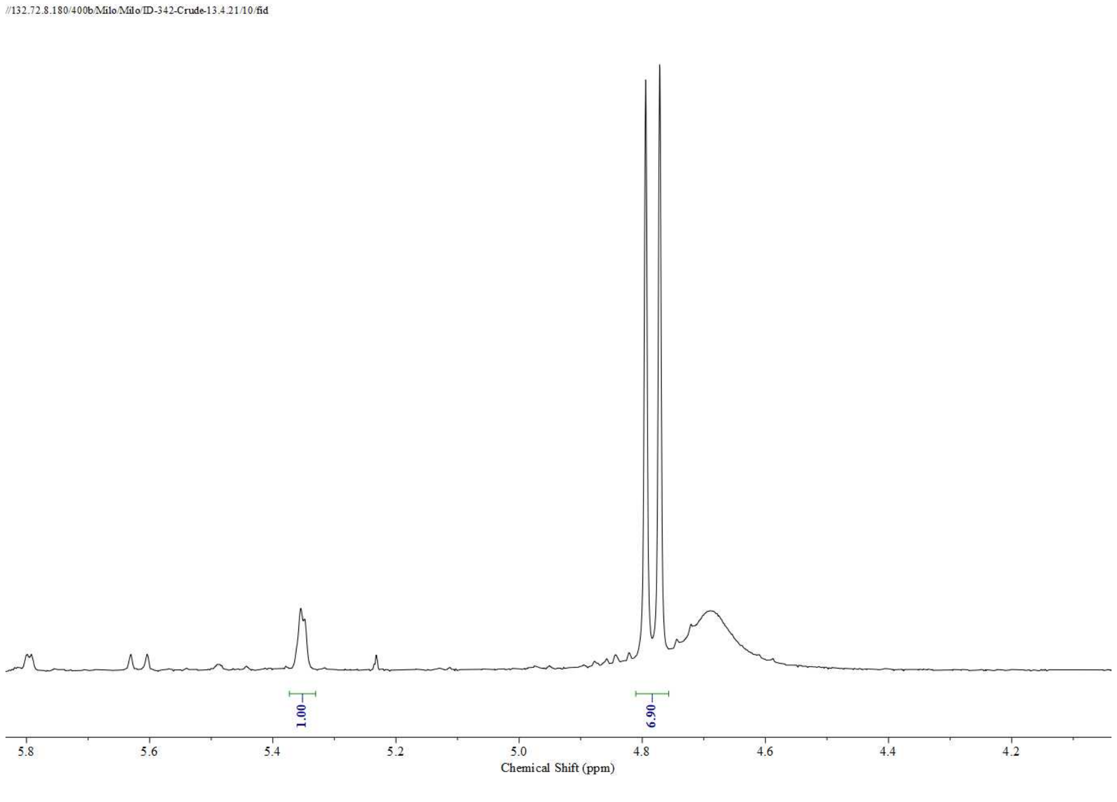

NMR traces for reaction with: **4-F-phenylboronic acid (duplicate) (400 MHz,** $CDCl_3$**)**

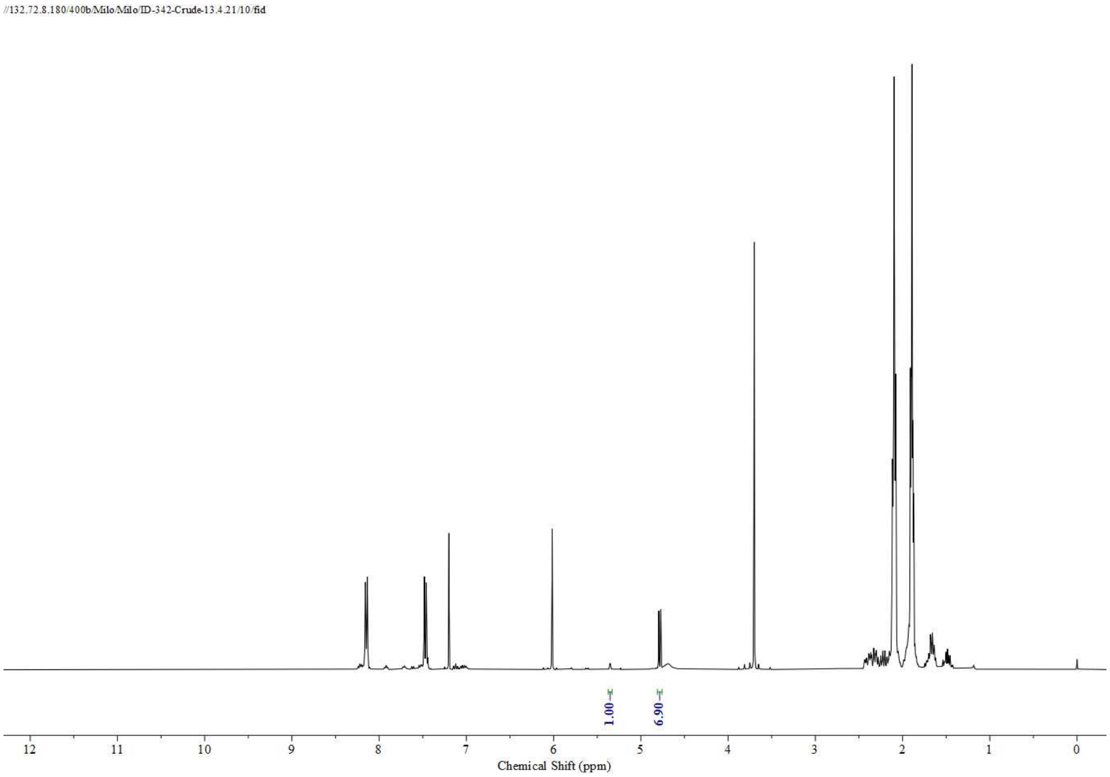

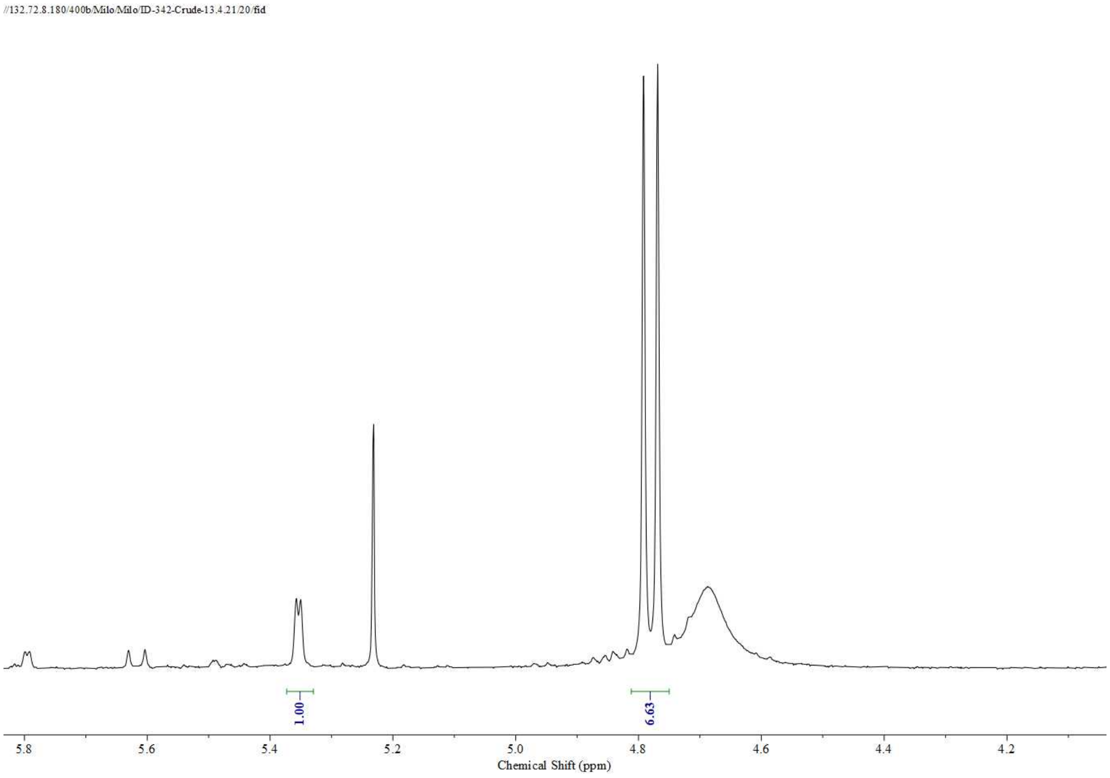

NMR traces for reaction with: **4-OMe-phenylboronic acid (400 MHz,** $CDCl_3$**)**

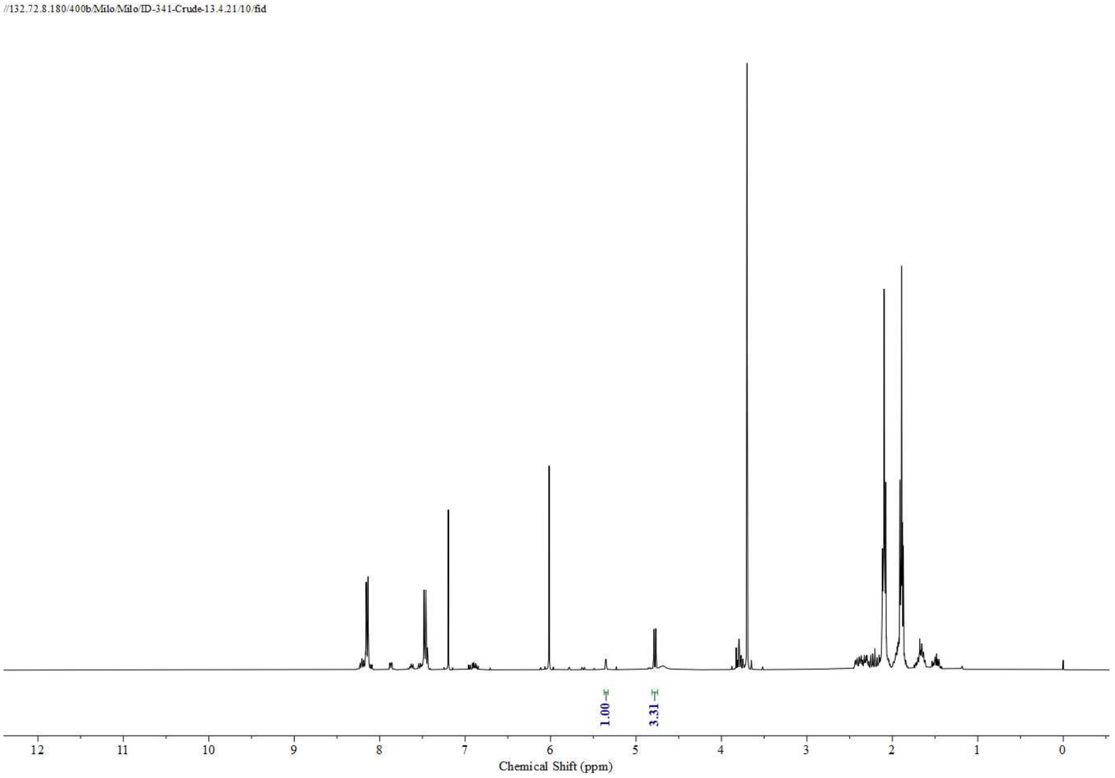

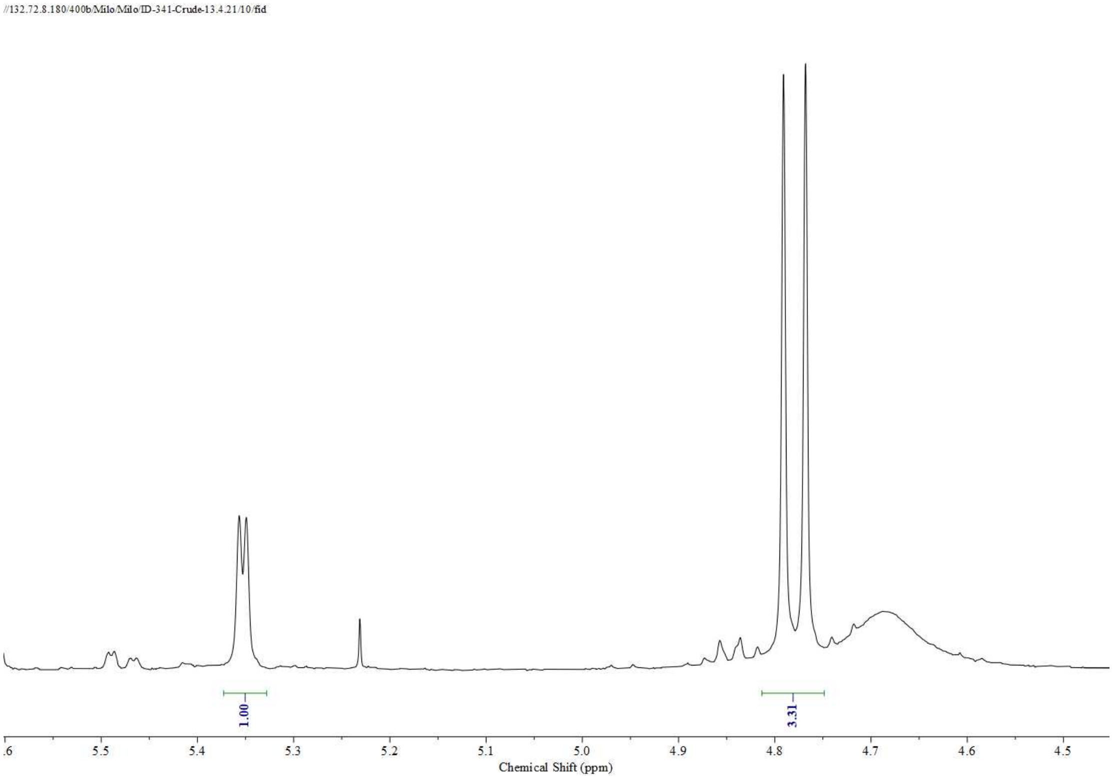

NMR traces for reaction with: **4-OMe-phenylboronic acid (duplicate) (400 MHz,** $CDCl_3$**)**

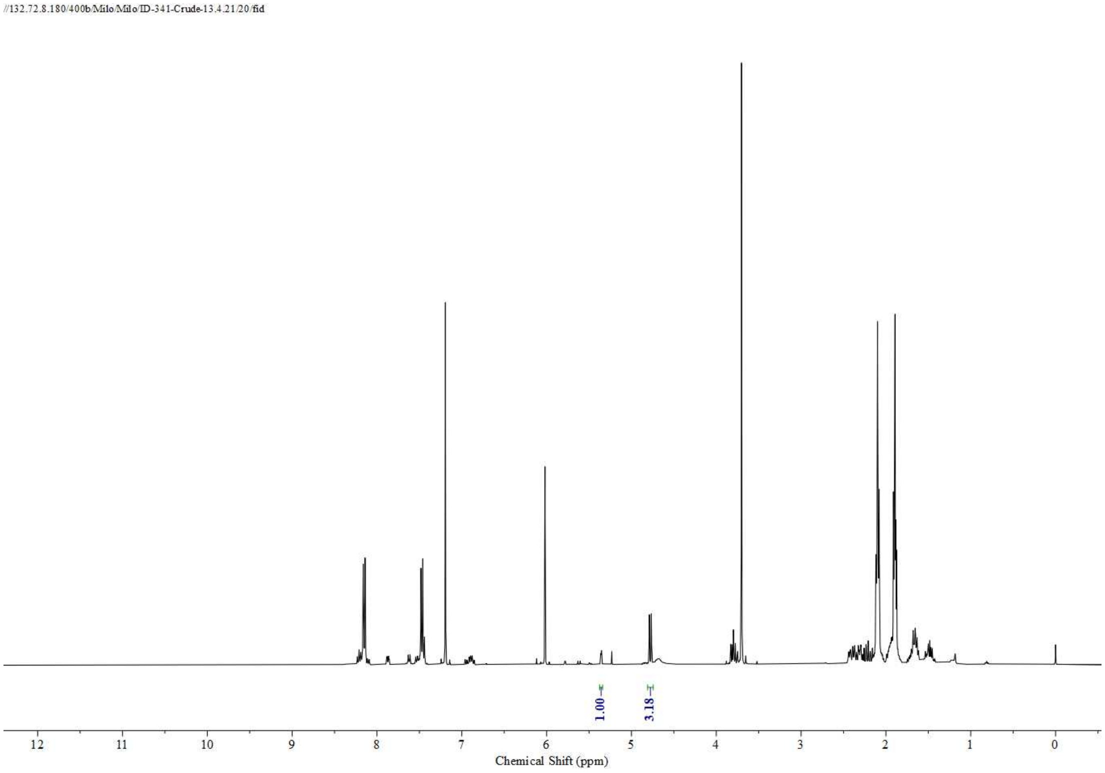

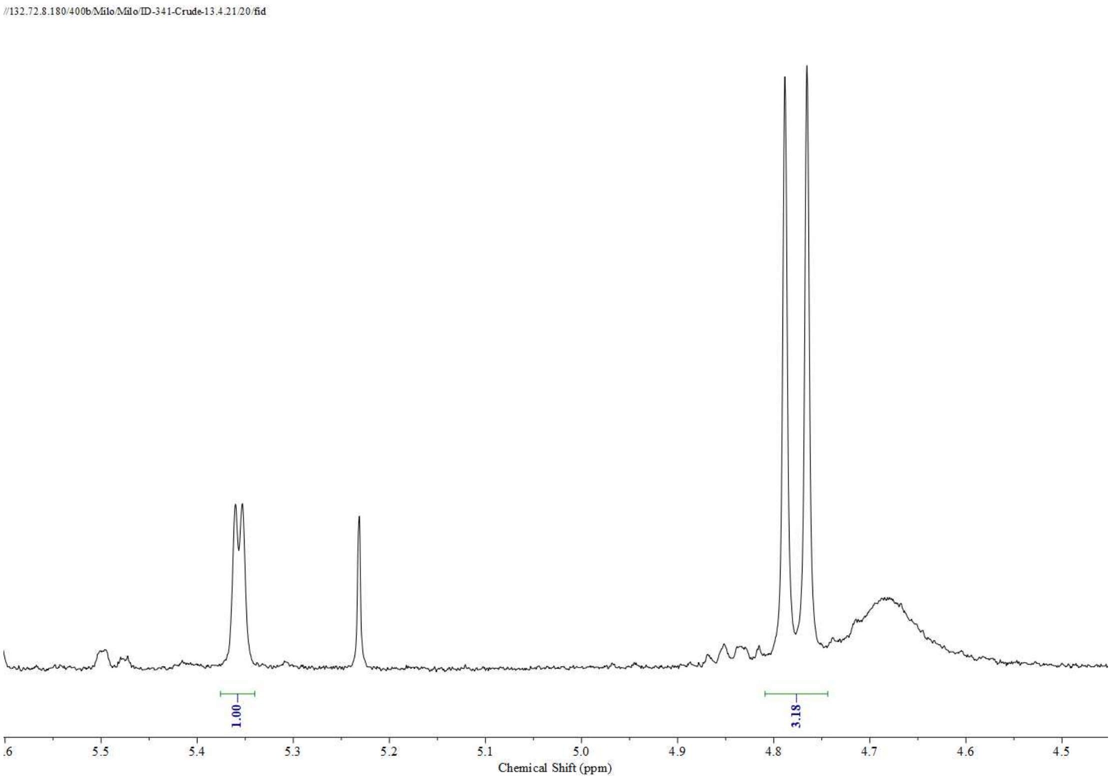

NMR traces for reaction with: **3-Me-phenylboronic acid (400 MHz,** $CDCl_3$**)**

ID-350-Crude.10.fid

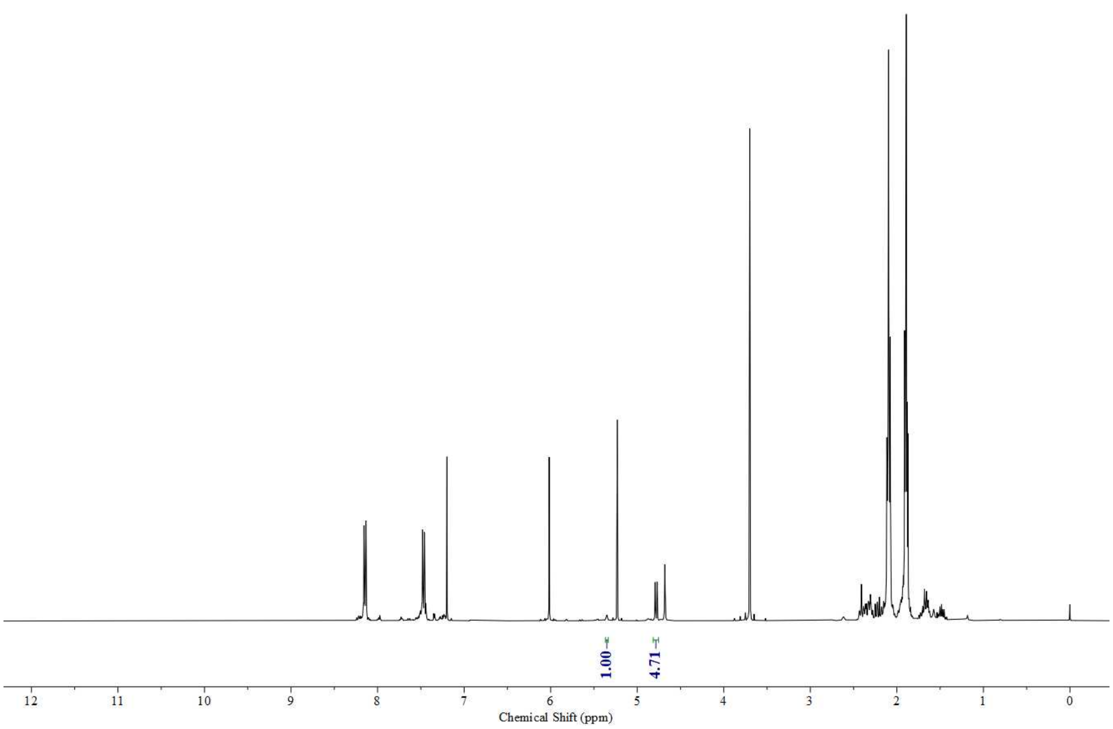

ID-350-Crude.10.fid

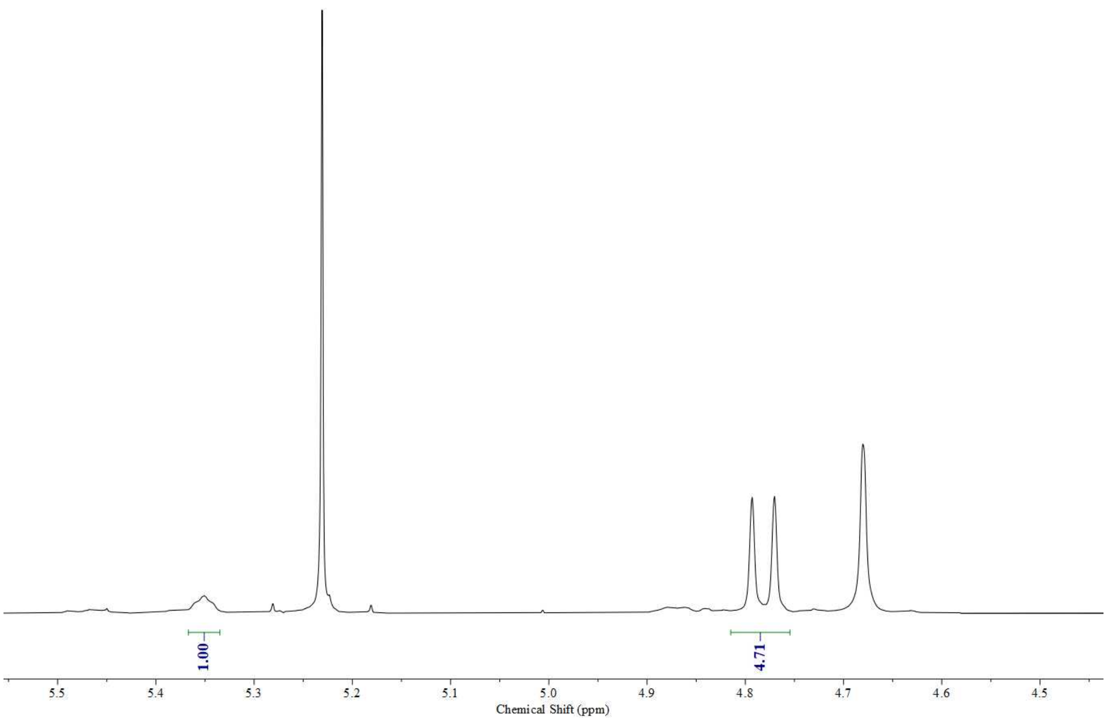

NMR traces for reaction with: **3-Me-phenylboronic acid (duplicate) (400 MHz,** $CDCl_3$**)**

ID-350-Crude.10.fid

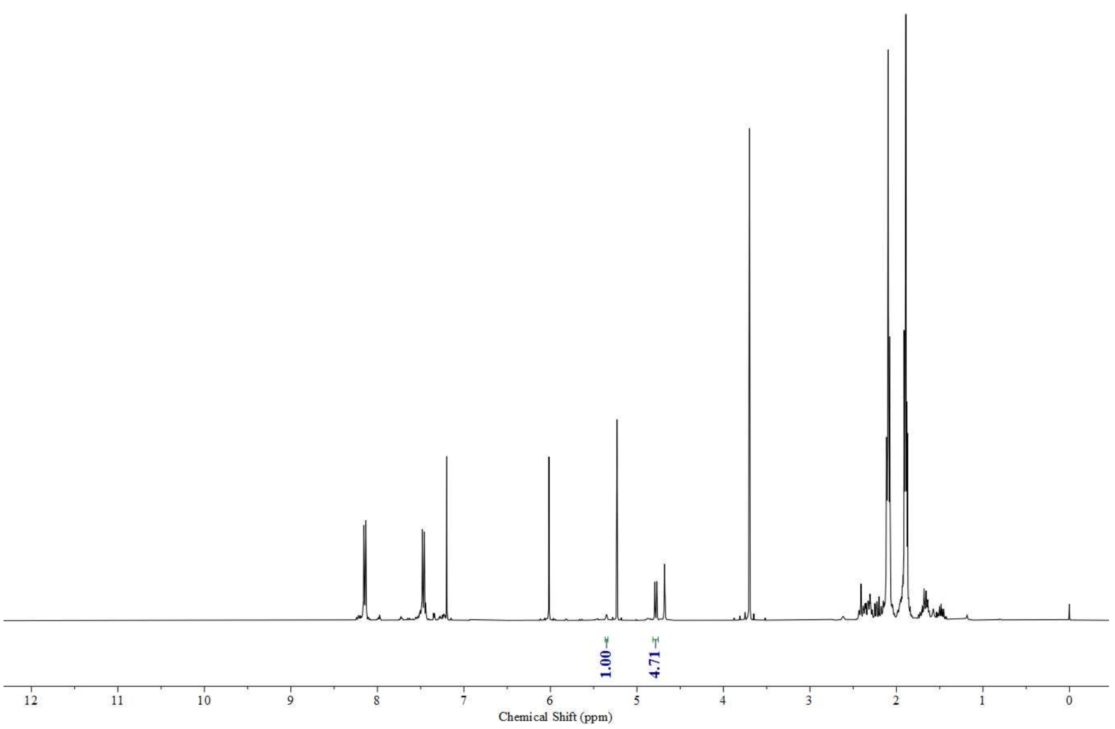

ID-350-Crude.20.fid

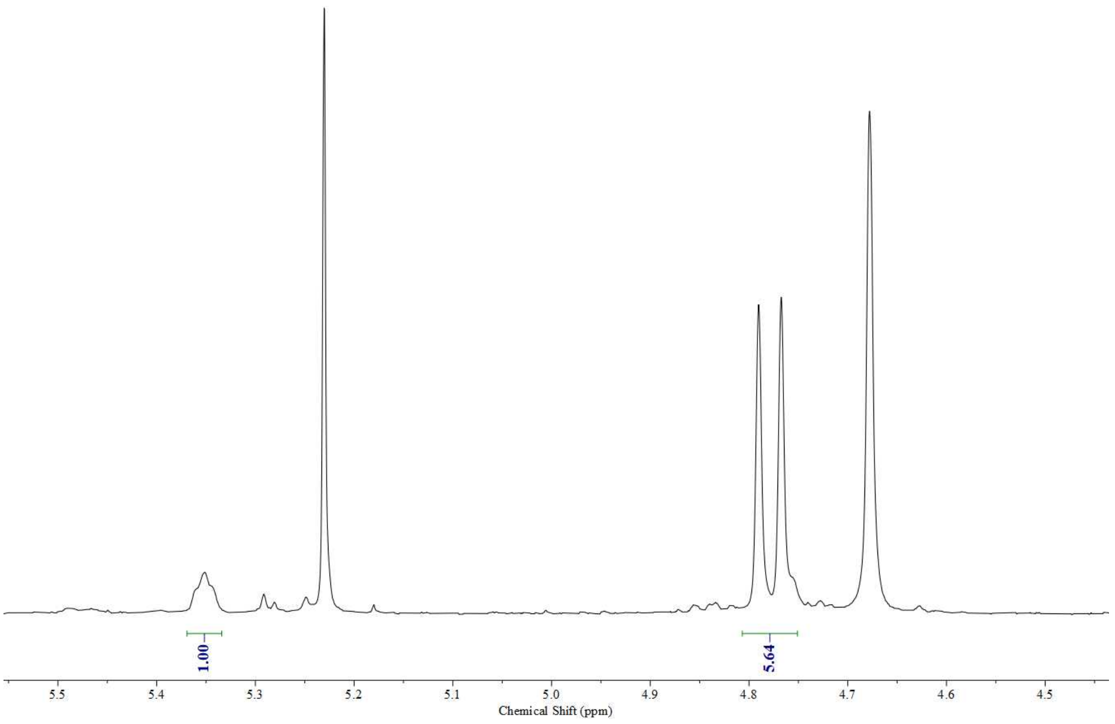

NMR traces for reaction with: **2-Me-phenylboronic acid (400 MHz,** $CDCl_3$**)**

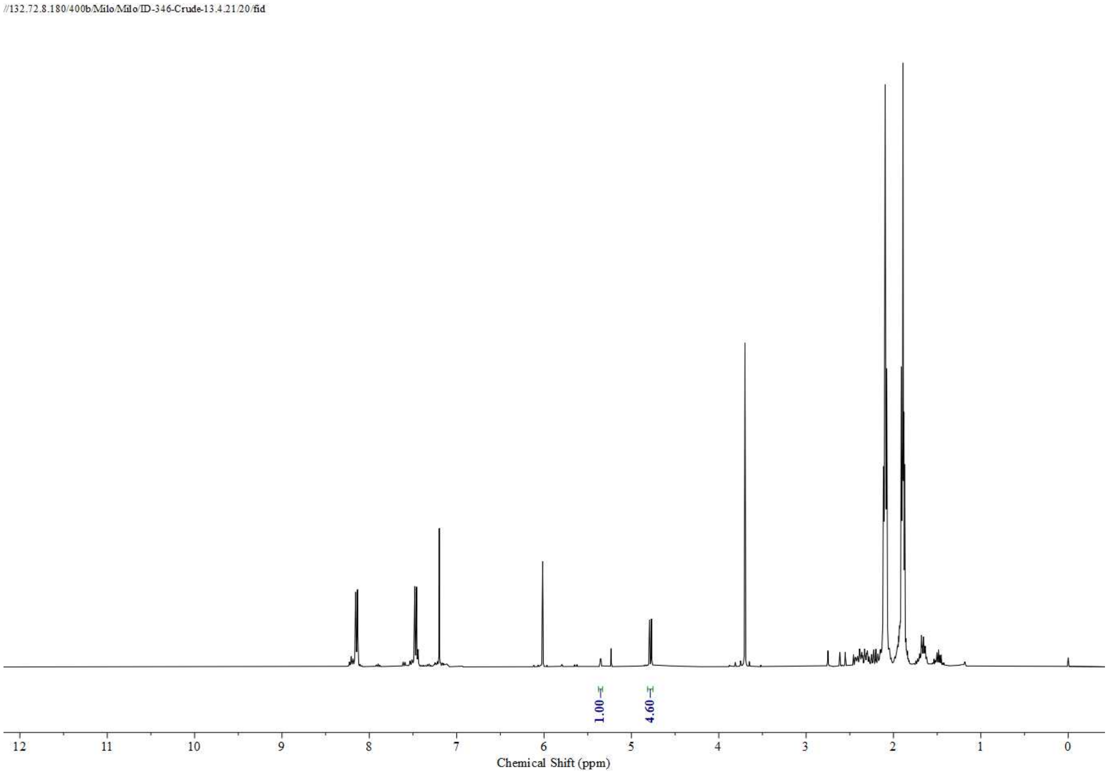

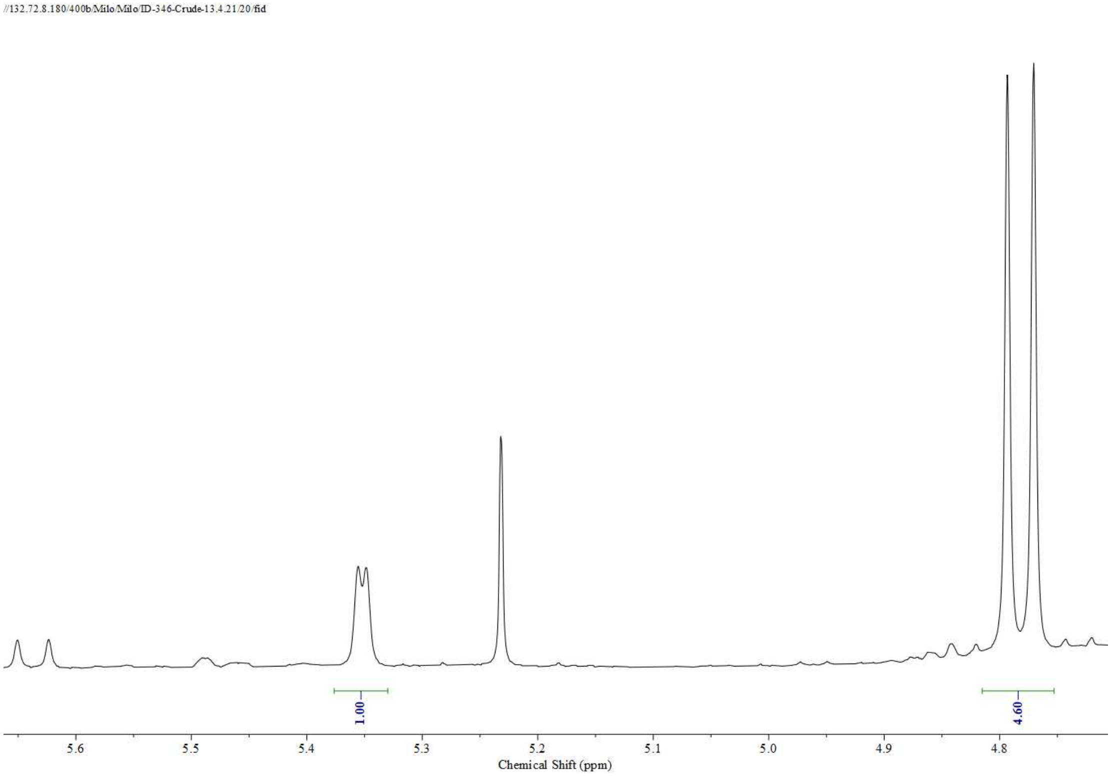

NMR traces for reaction with: **2-Me-phenylboronic acid (duplicate) (400 MHz,** $CDCl_3$**)**

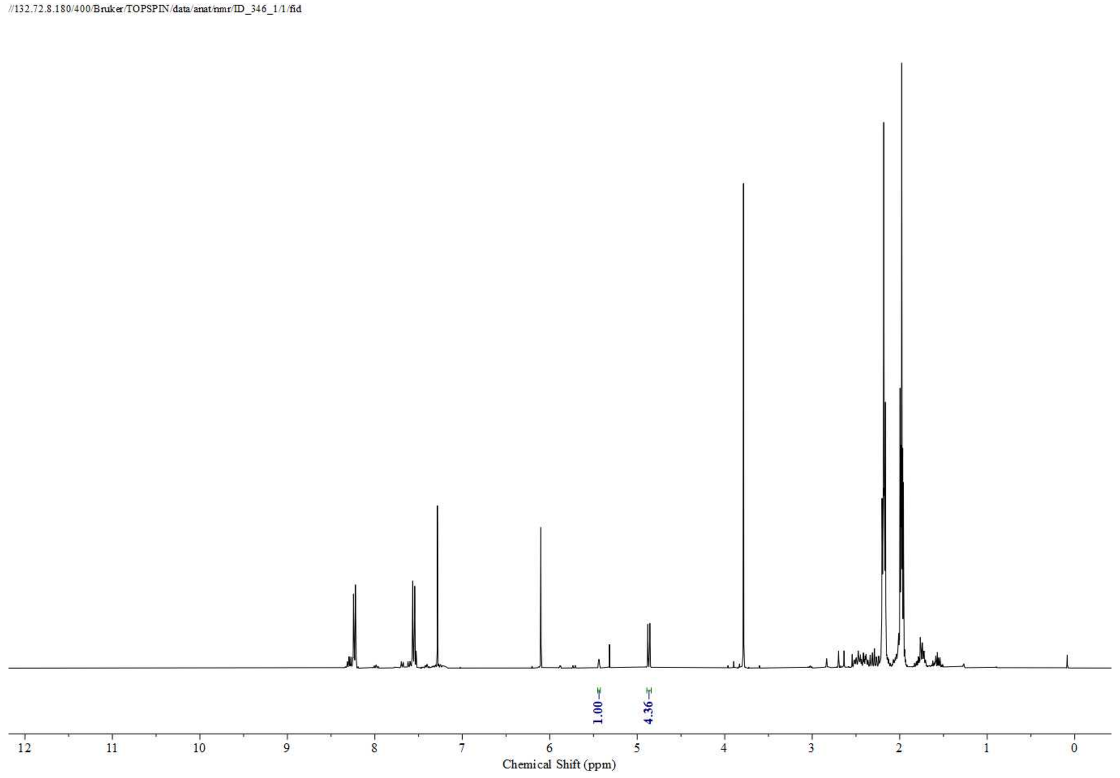

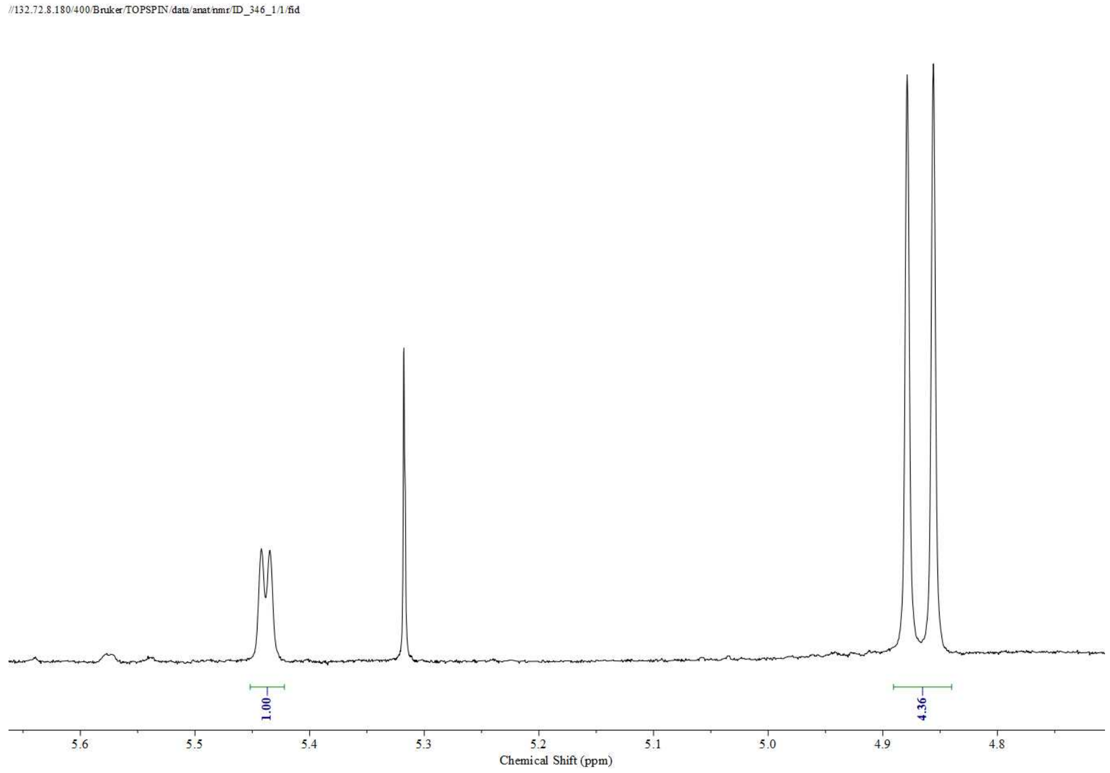

NMR traces for reaction with: **Ph-phenylboronic acid (400 MHz,** $CDCl_3$**)**

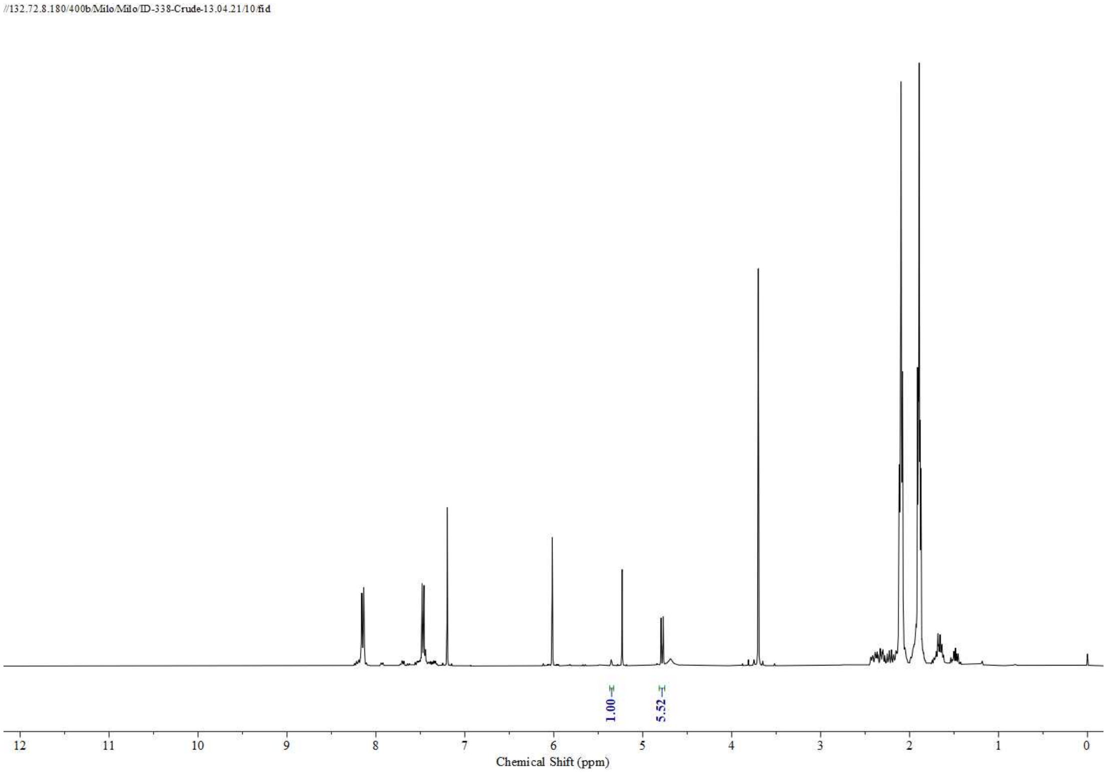

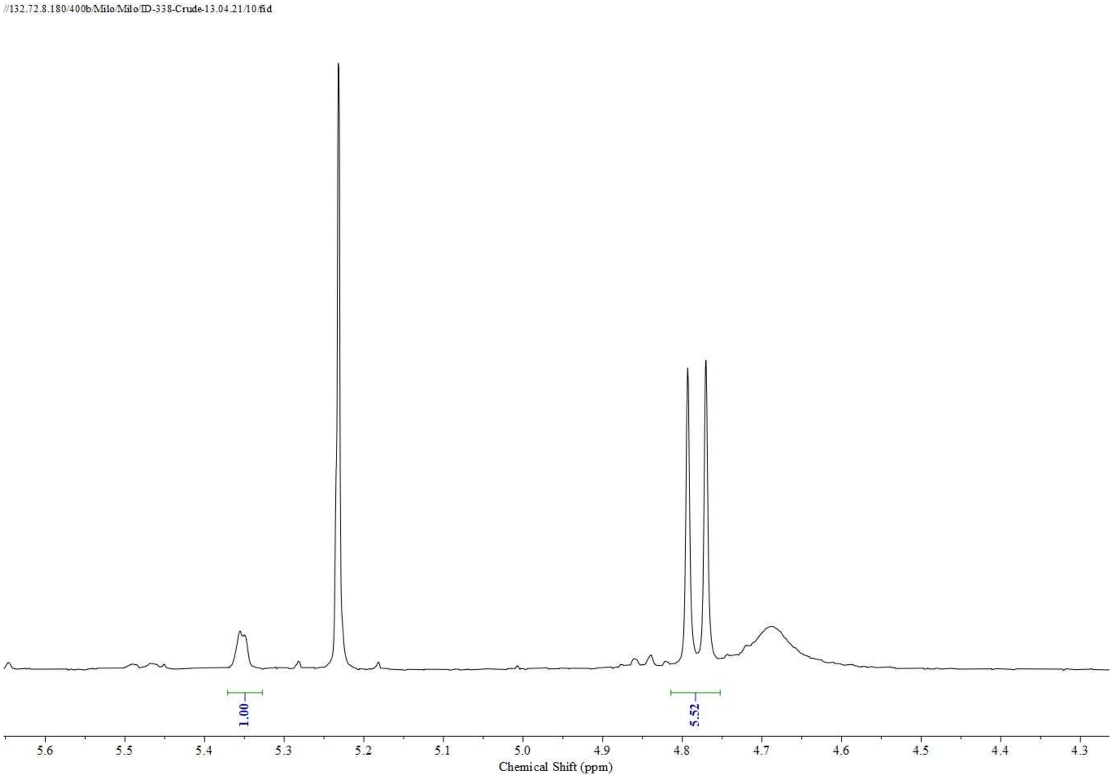

NMR traces for reaction with: **Ph-phenylboronic acid (duplicate) (400 MHz,** $CDCl_3$**)**

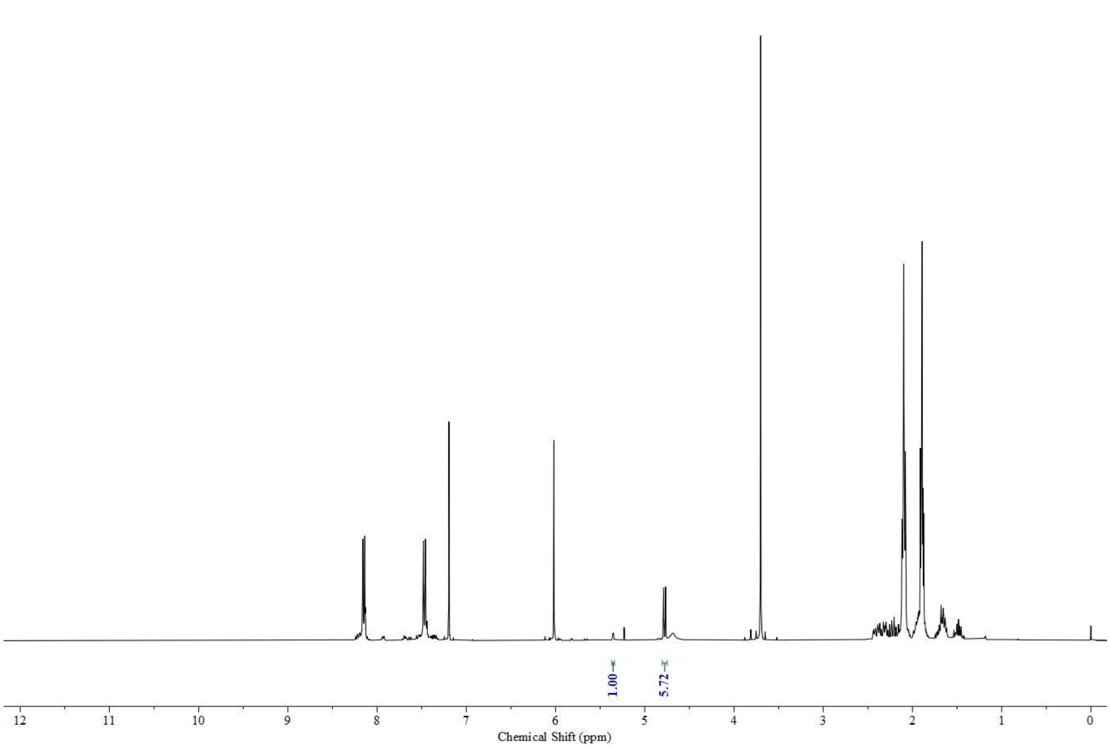

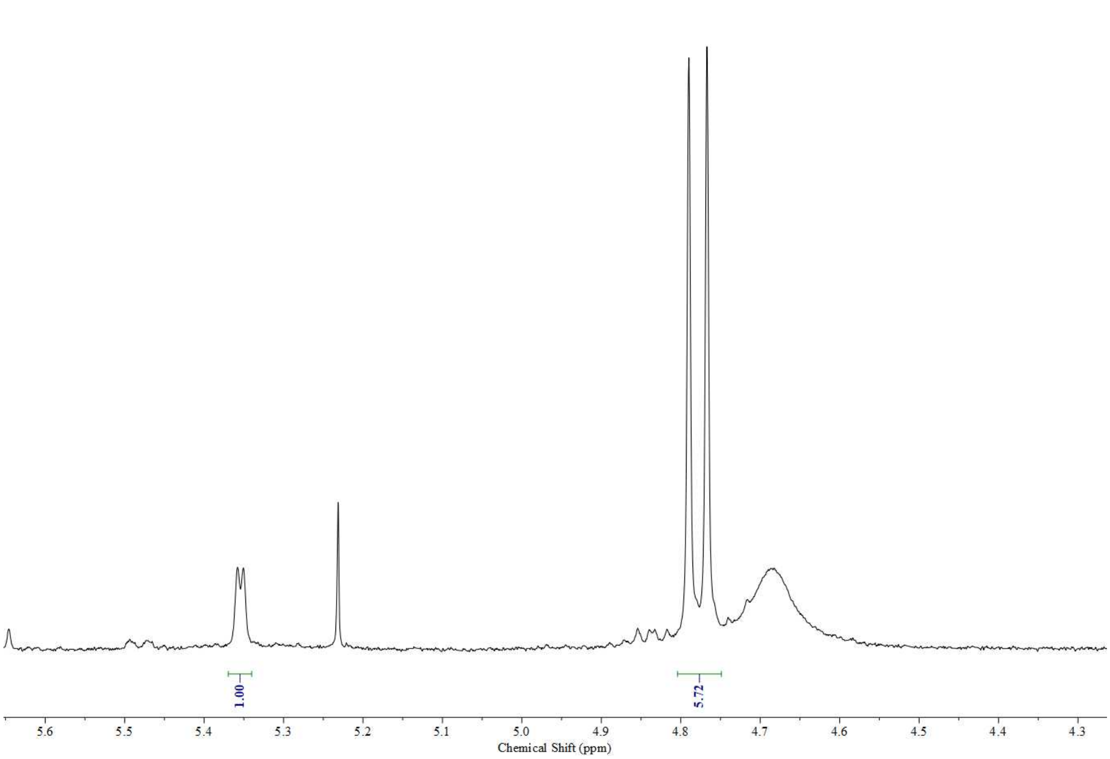

NMR traces for reaction with: **2-F-phenylboronic acid (400 MHz,** $CDCl_3$**)**

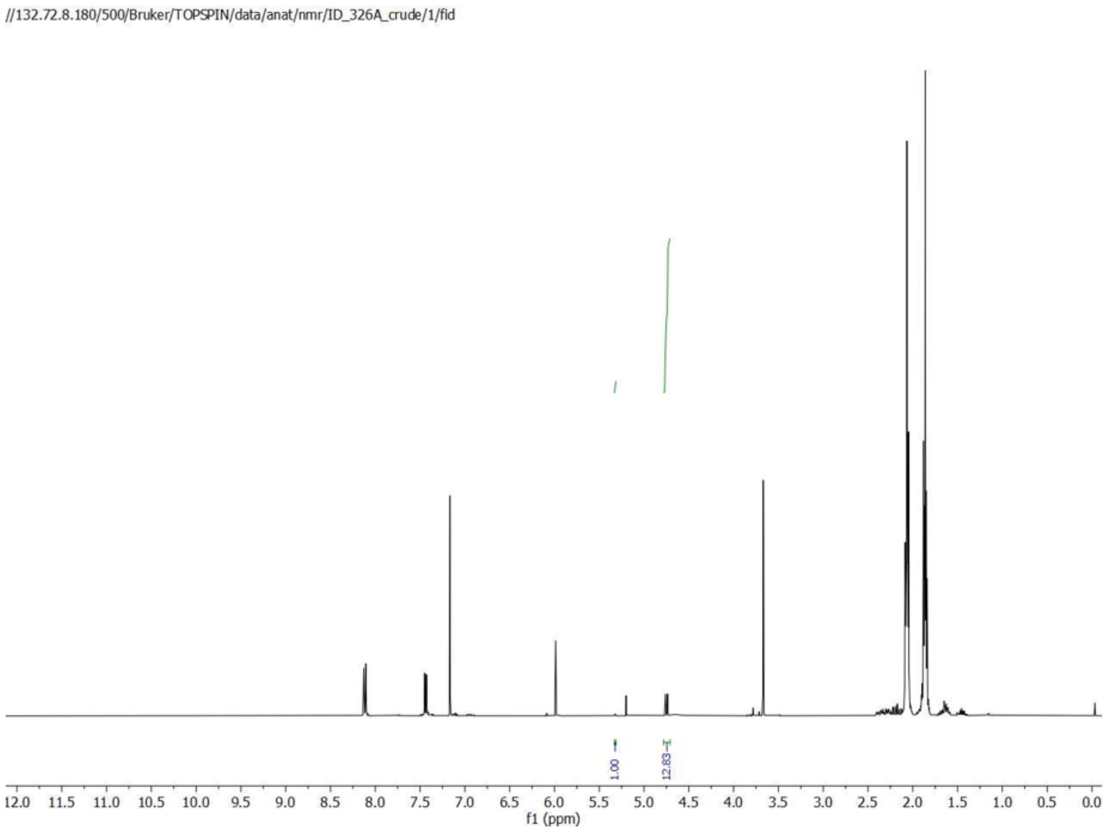

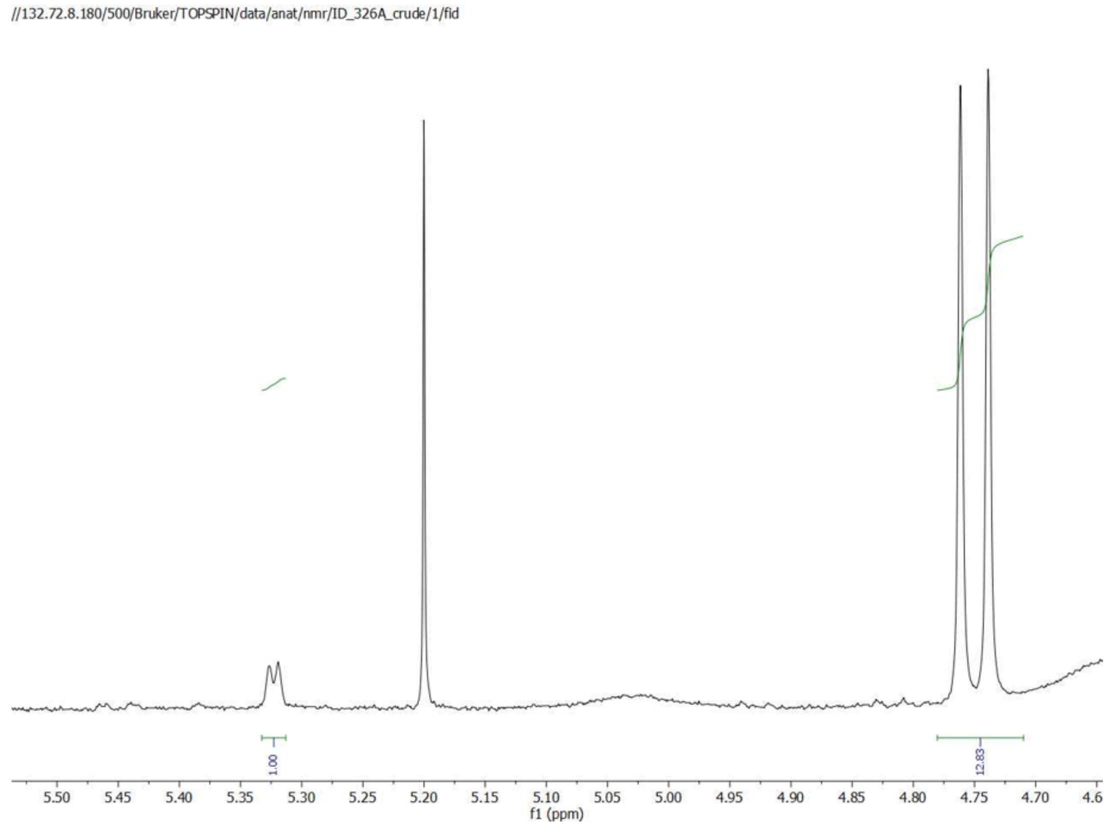

NMR traces for reaction with: **2-F-phenylboronic acid (duplicate) (400 MHz,** $CDCl_3$**)**

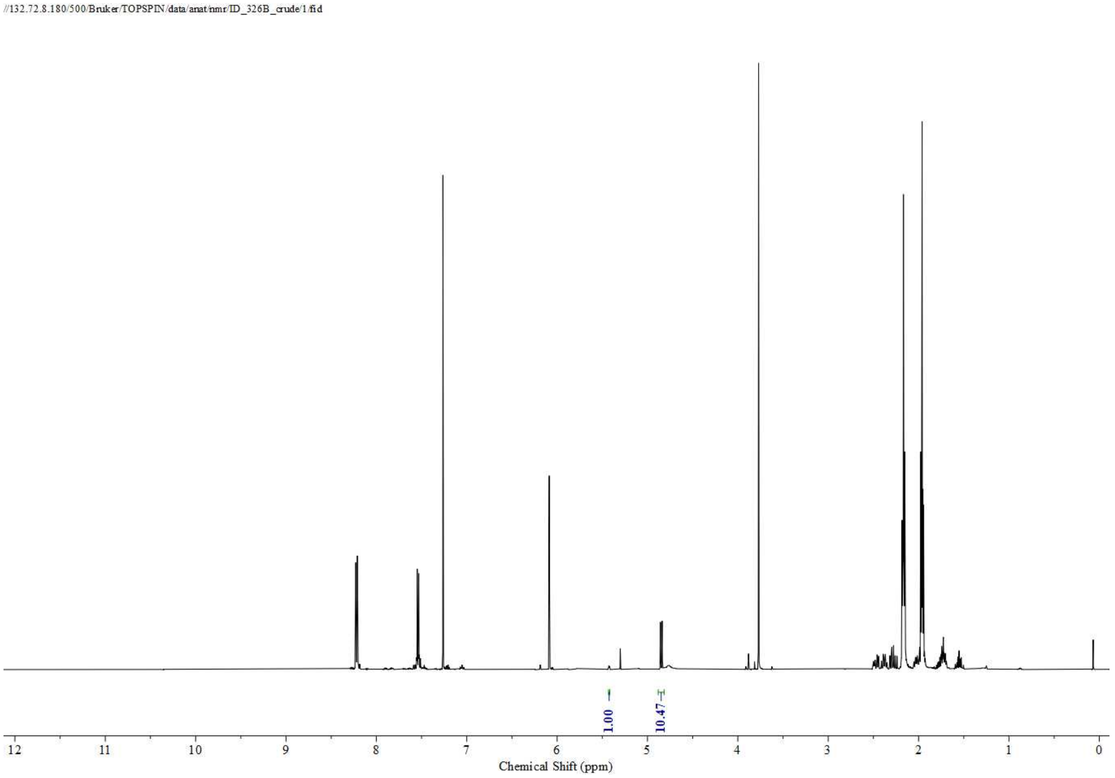

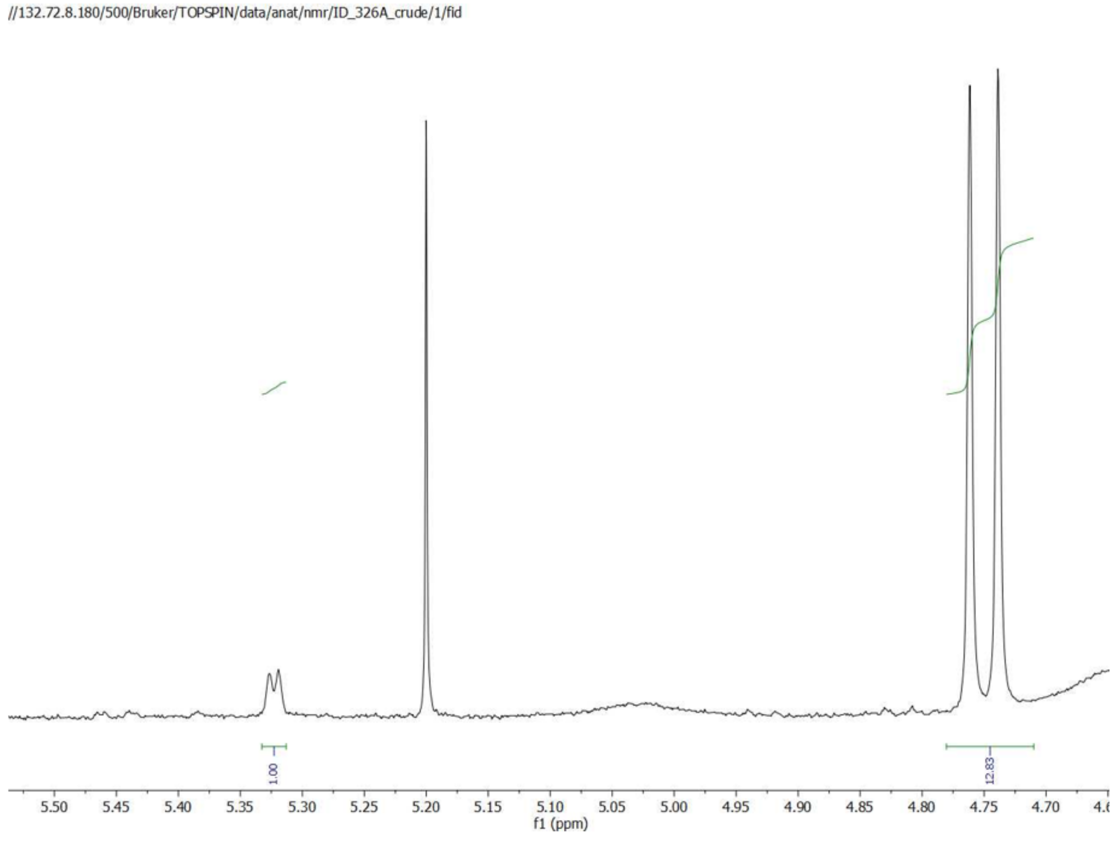

NMR traces for reaction **without boronic acid (400 MHz,** CDCl$_3$**)**

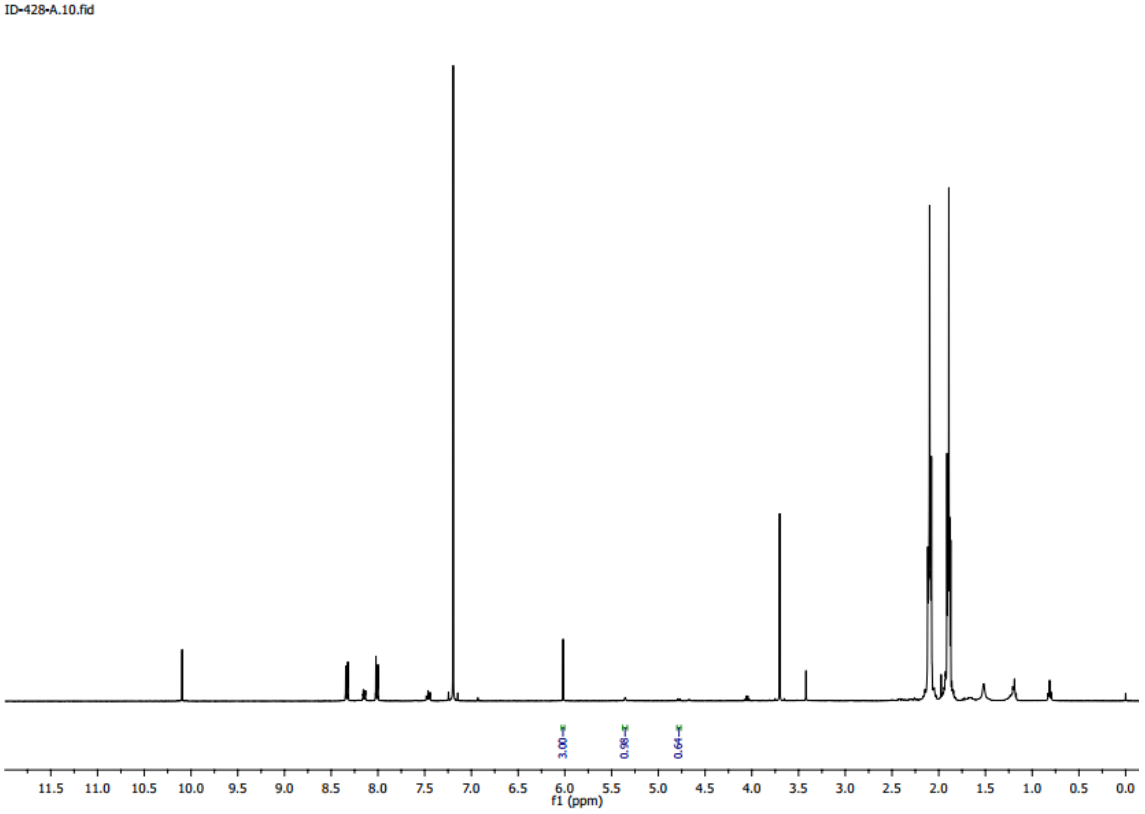

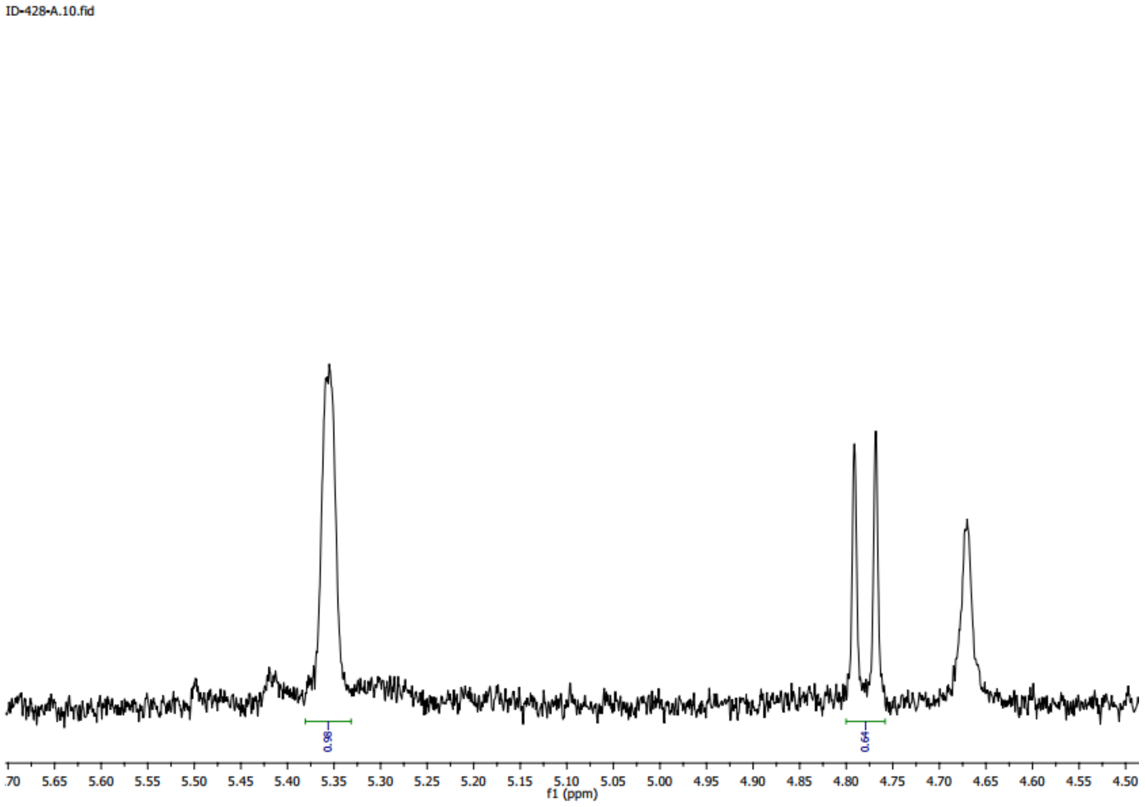

NMR traces for reaction **without boronic acid (duplicate) (400 MHz,** CDCl$_3$**):**

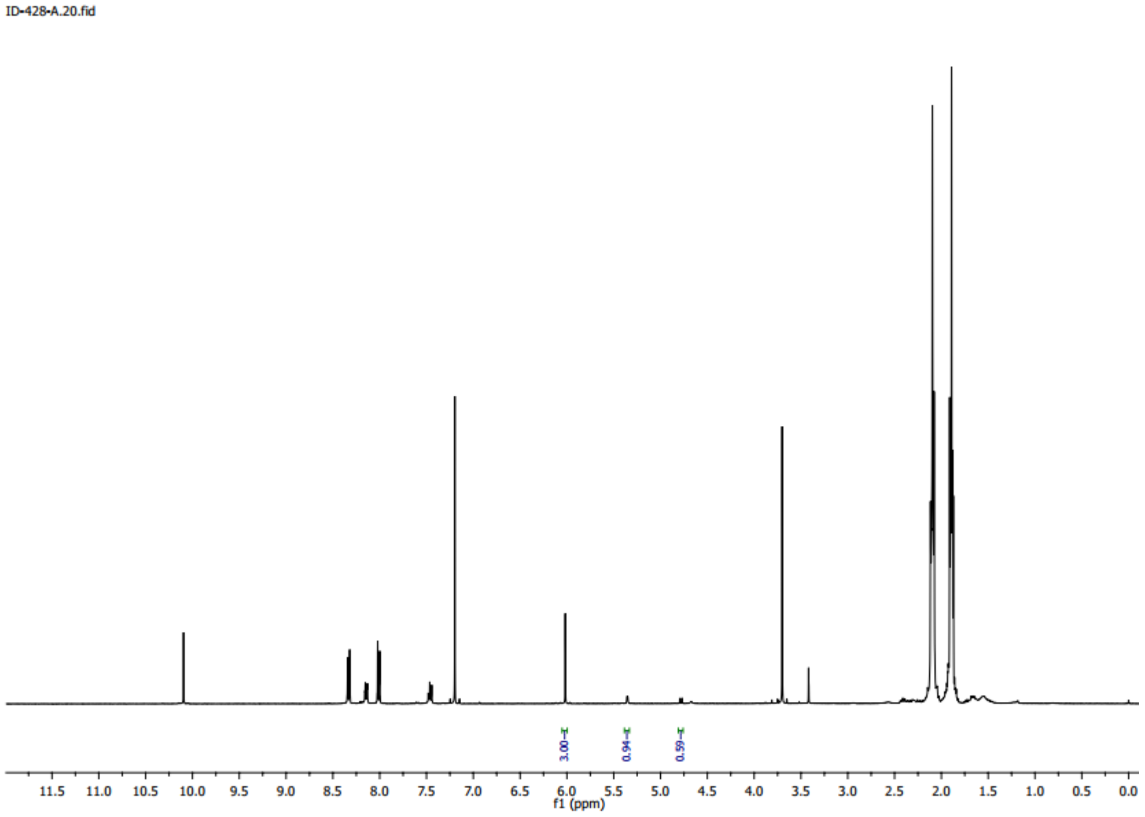

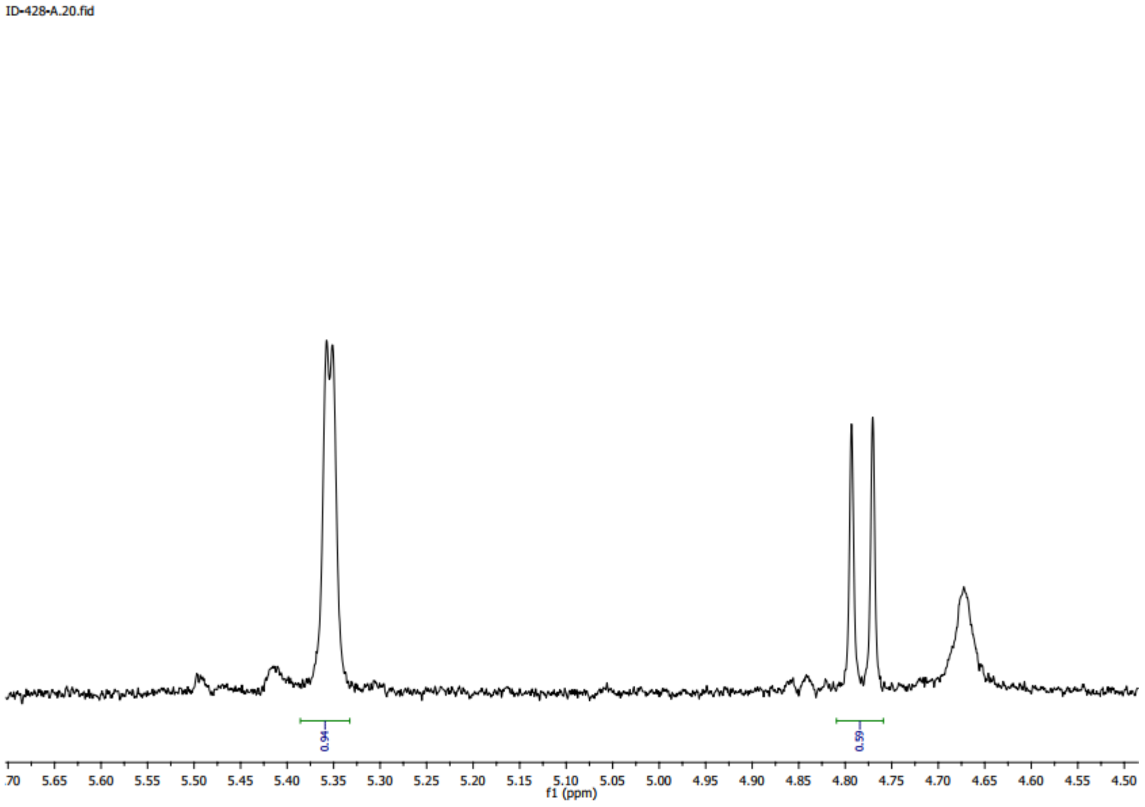

NMR traces for reaction **without water, with 2-F-phenylboronic acid (400 MHz,** $CDCl_3$):

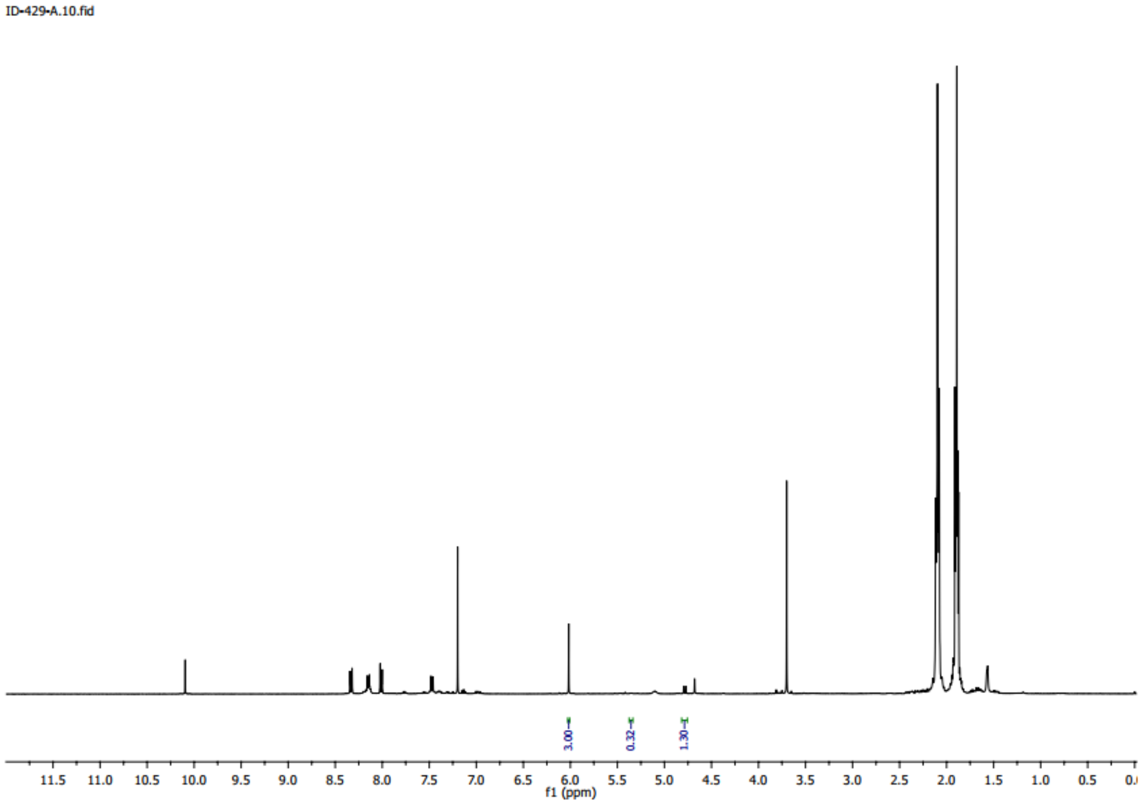

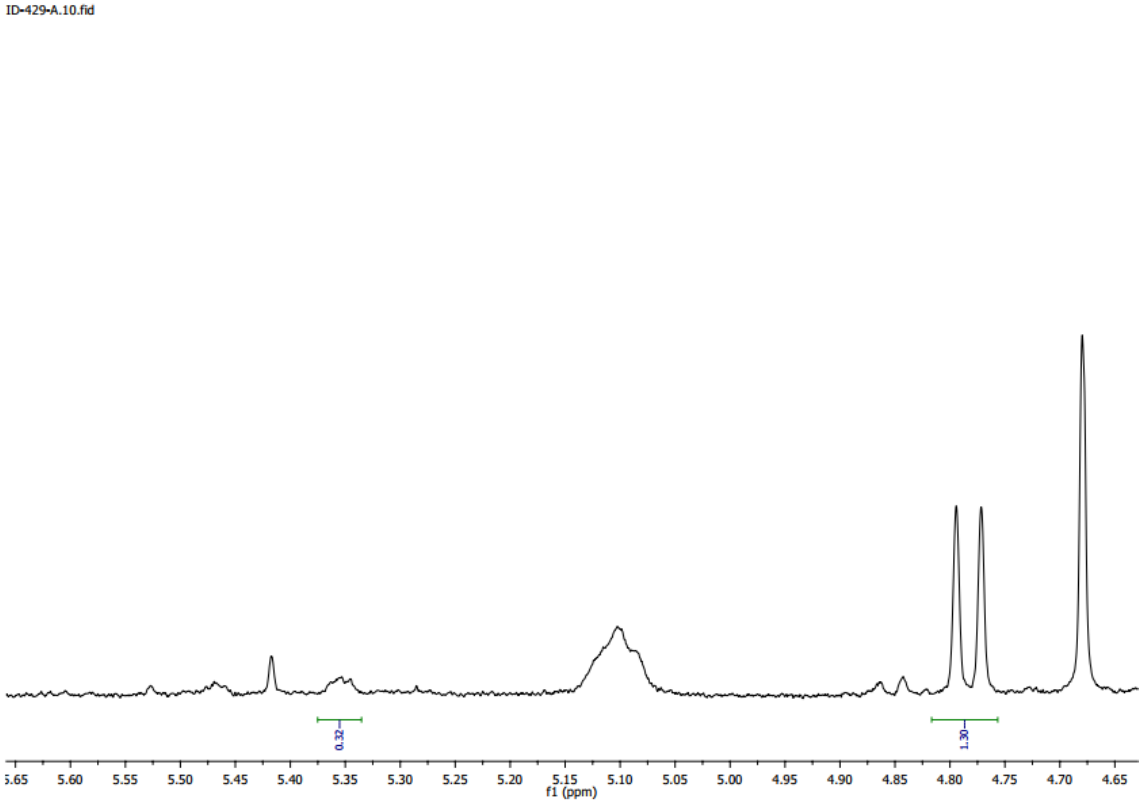

NMR traces for reaction **without water, with 2-F-phenylboronic acid (duplicate) (400 MHz,** CDCl$_3$**)**:

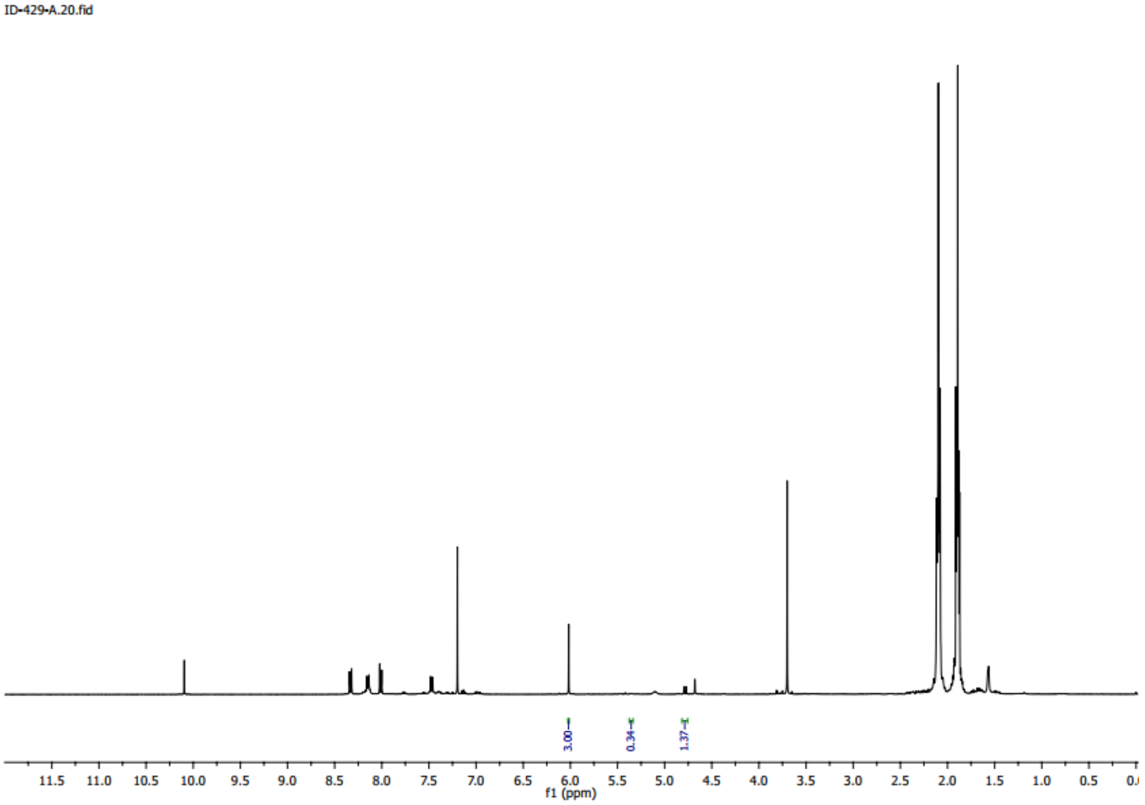

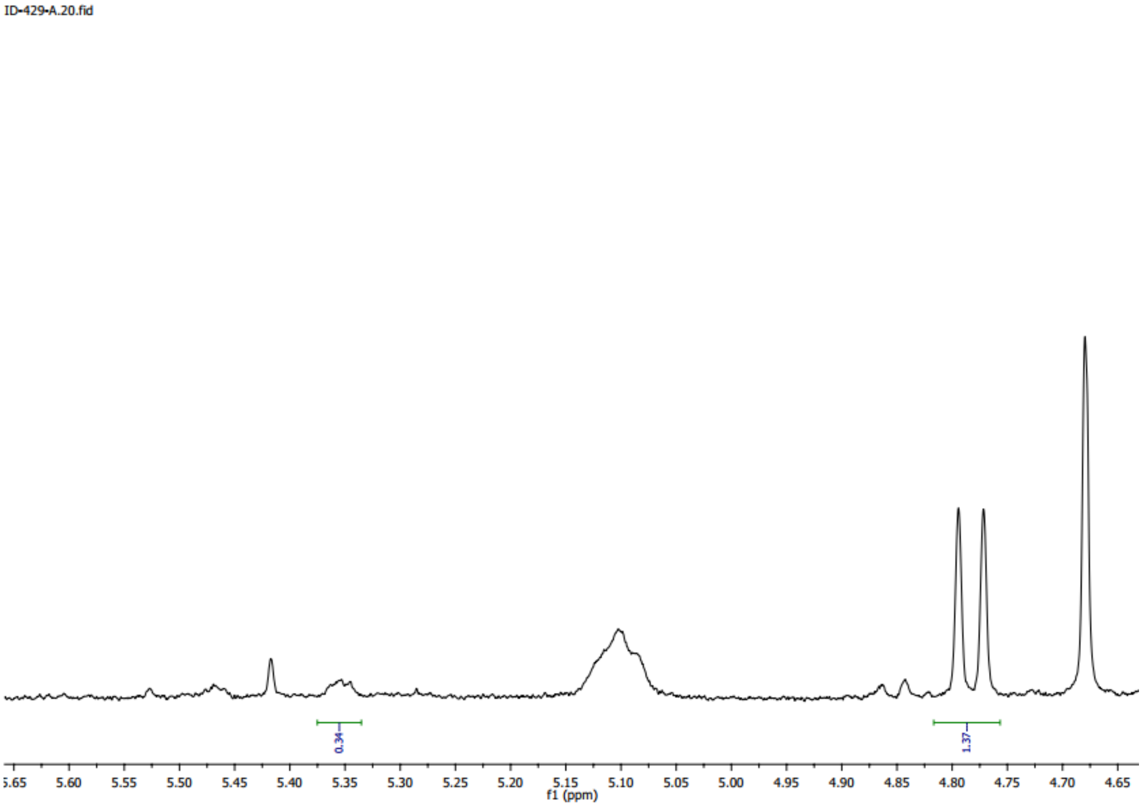

18.4. NMR Traces for Table S4. Boronic acid screening and blanks in MeOH

NMR traces for reaction with: **3-F-phenylboronic acid (400 MHz,** $CDCl_3$**)**

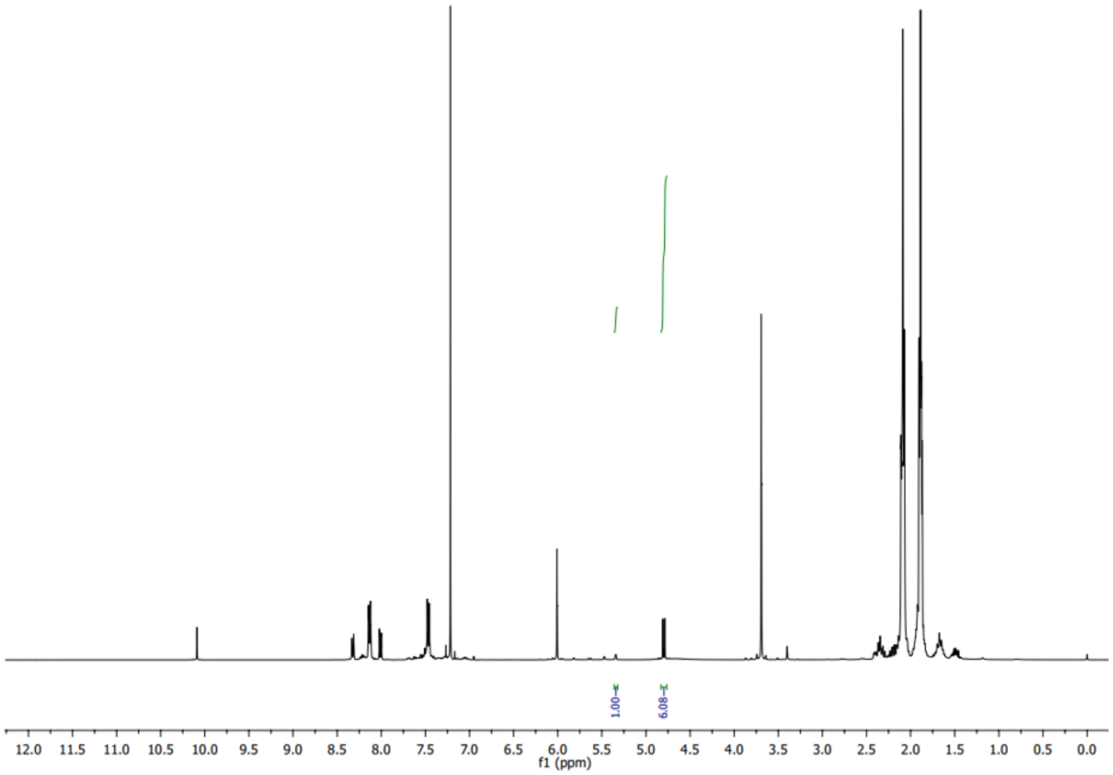

ID_422_O.10.fid

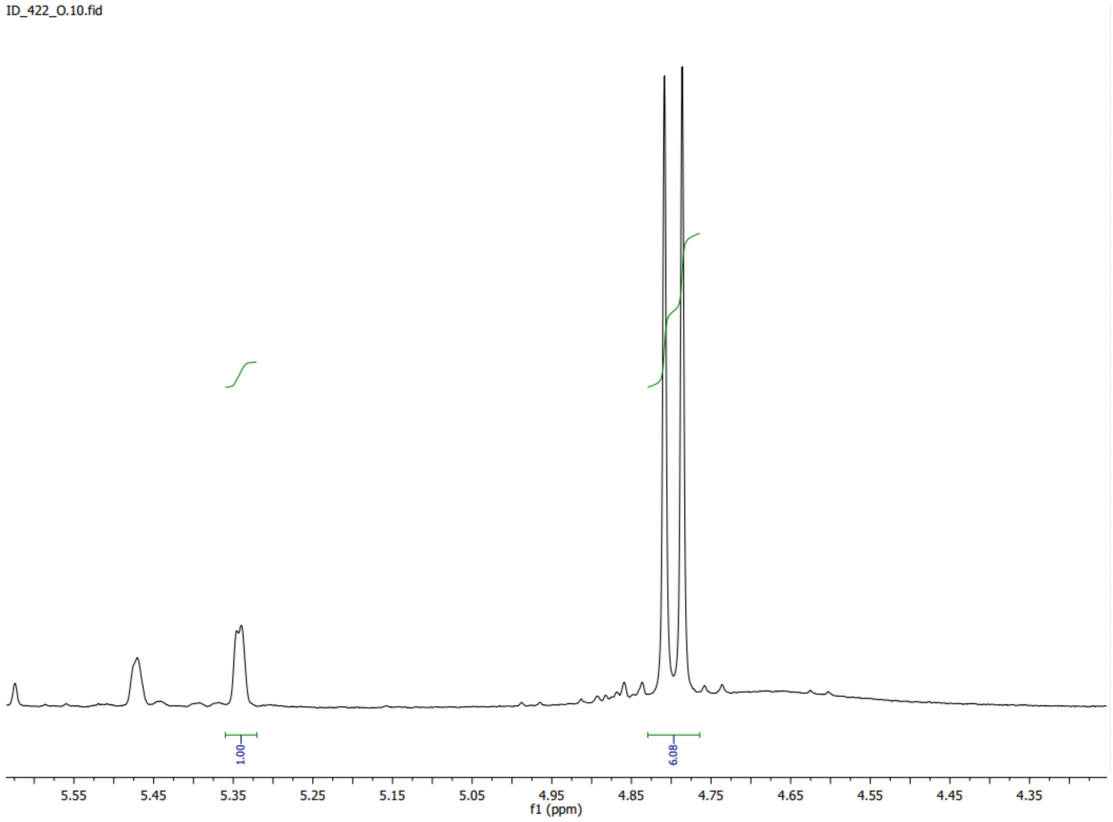

NMR traces for reaction with: **3-F-phenylboronic acid (duplicate) (400 MHz,** CDCl$_3$**)**

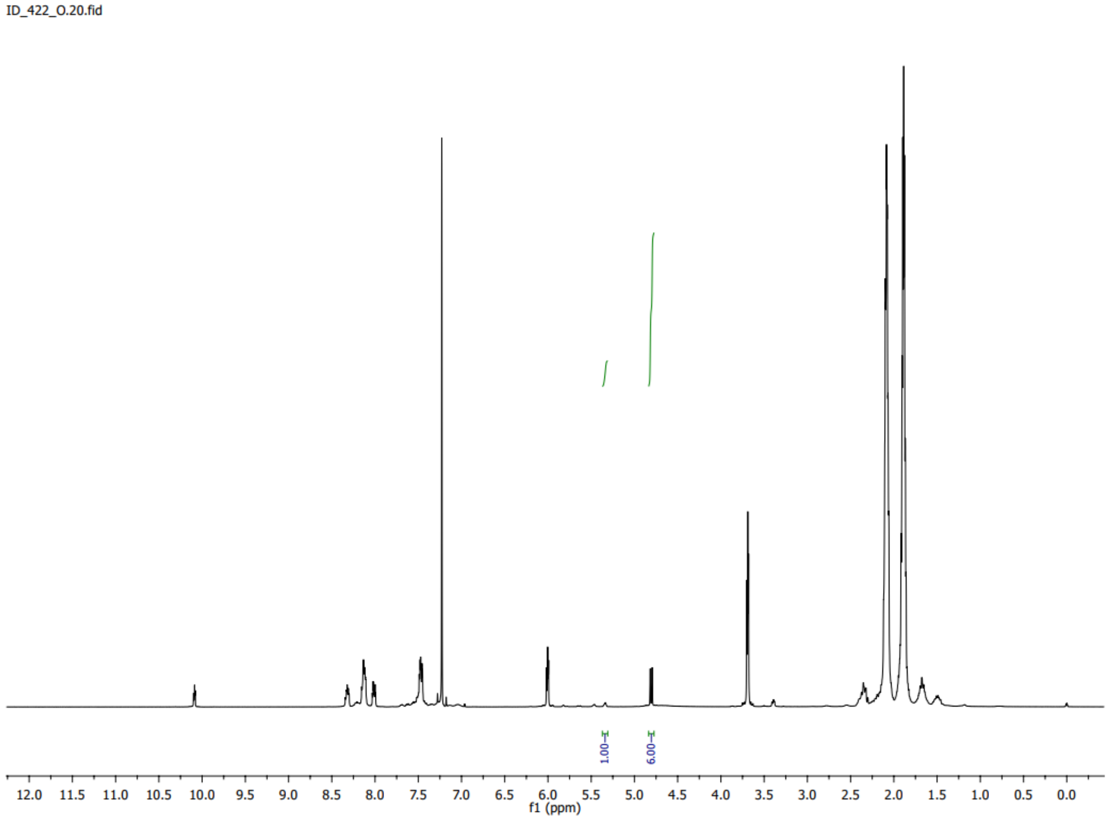

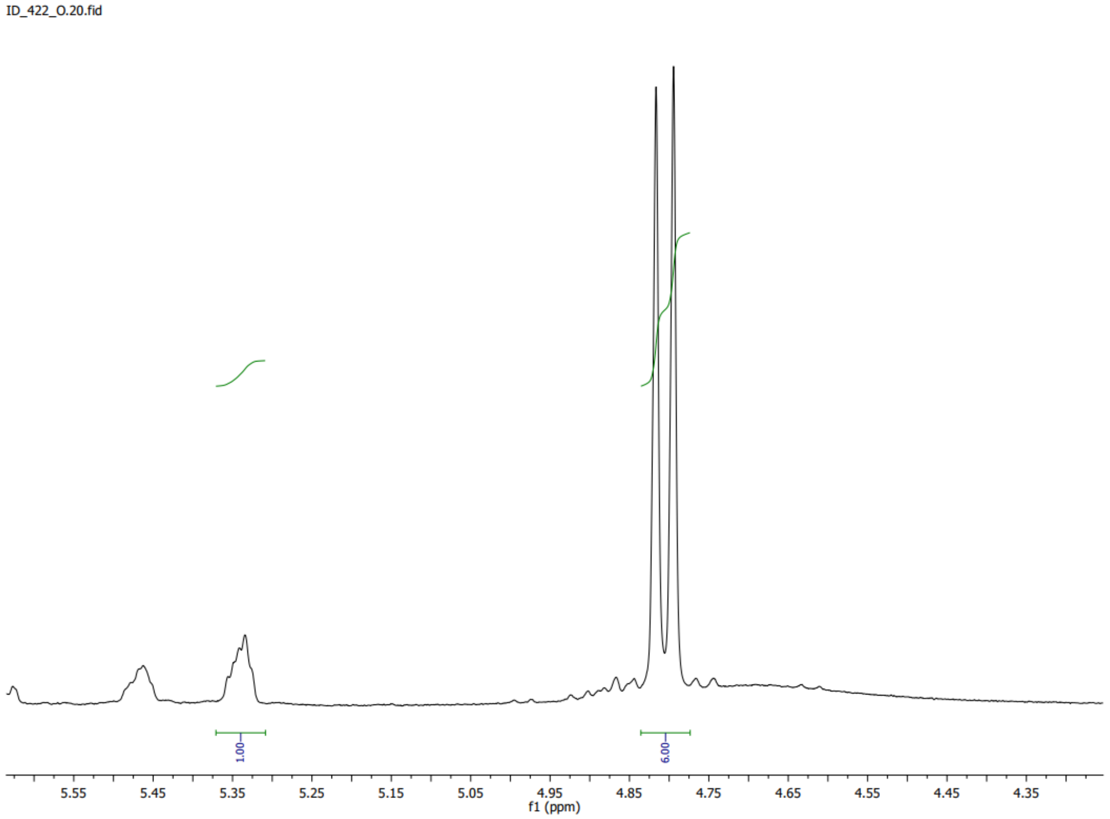

NMR traces for reaction with: **3,5-F-phenylboronic acid (400 MHz,** $CDCl_3$**)**

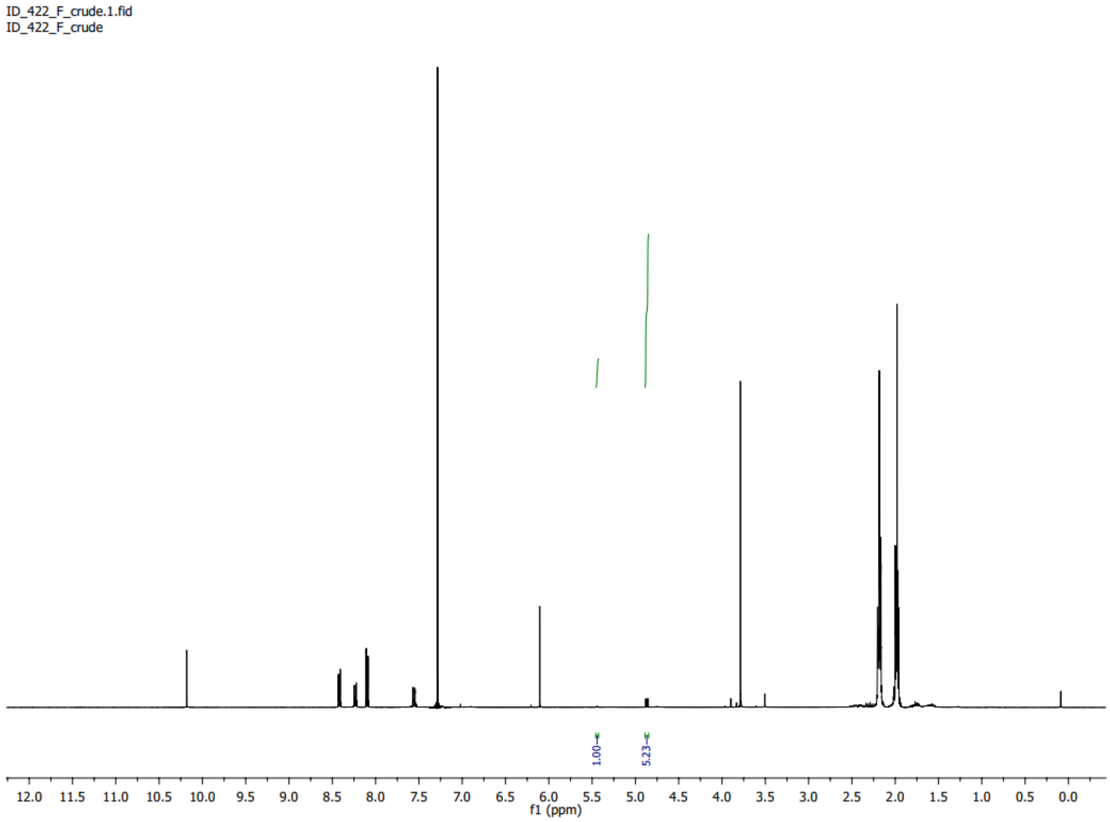

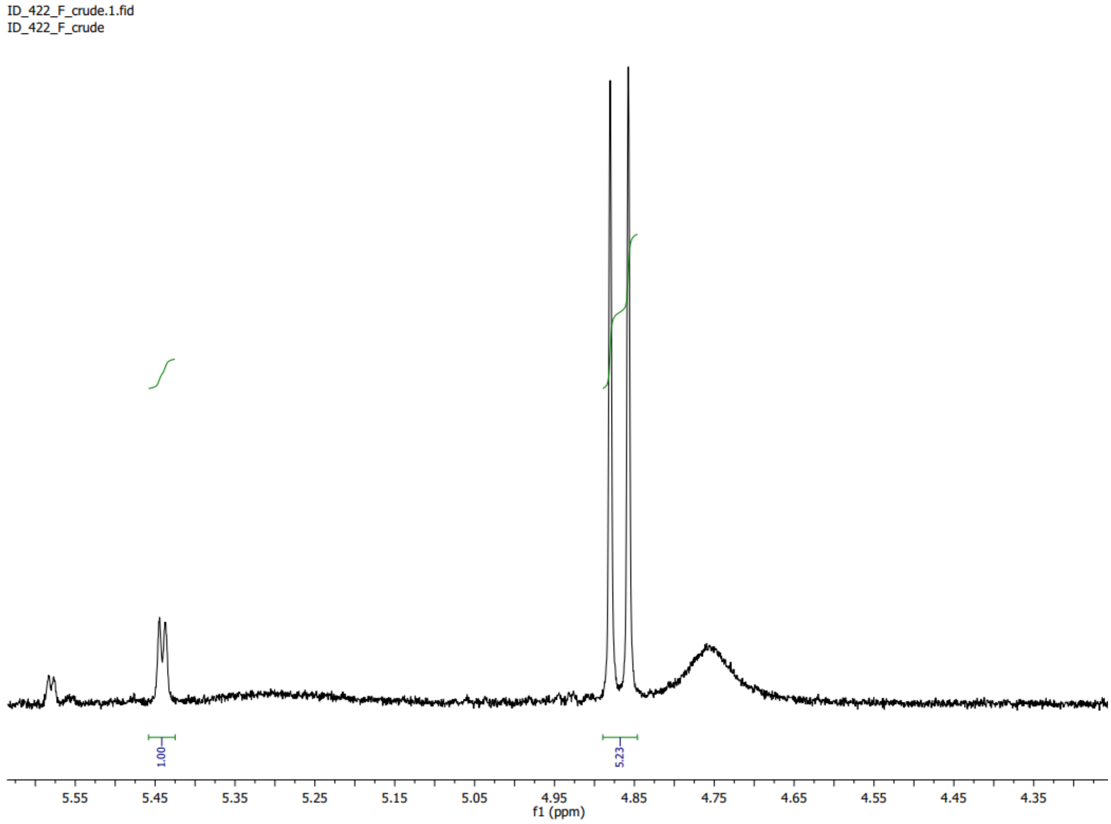

NMR traces for reaction with: **3,5-F-phenylboronic acid (duplicate) (400 MHz,** CDCl$_3$**)**

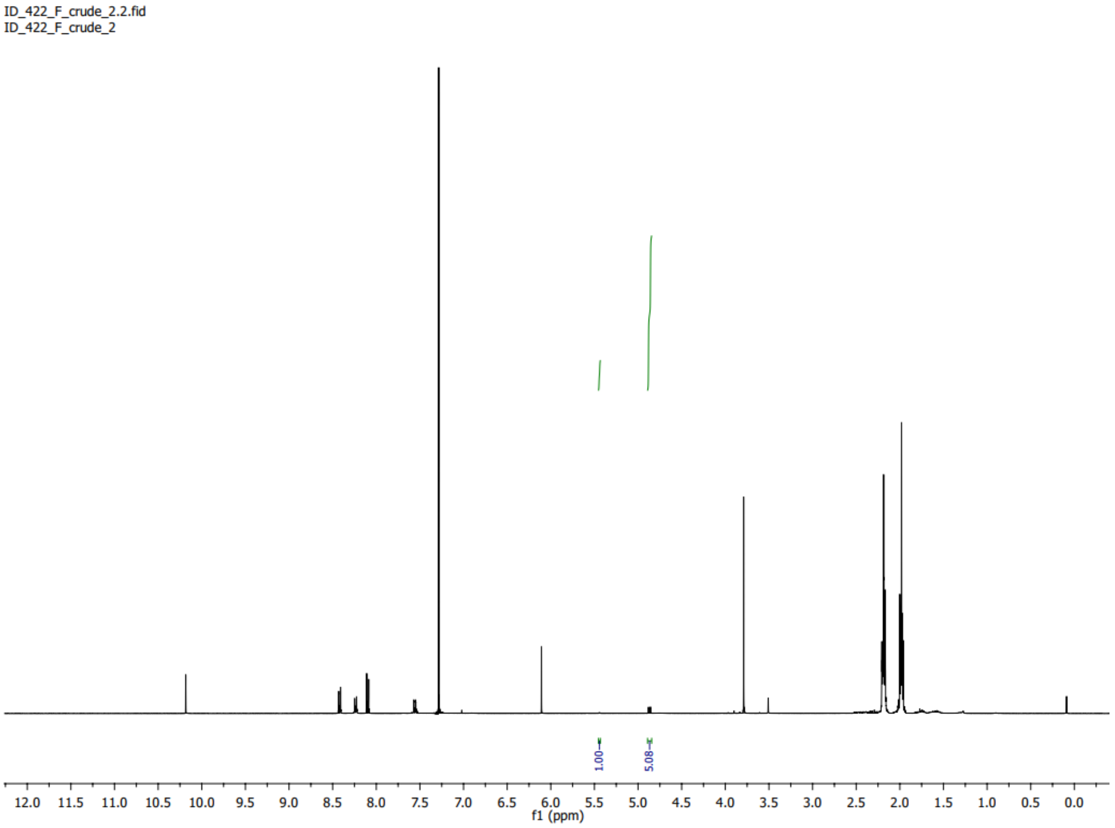

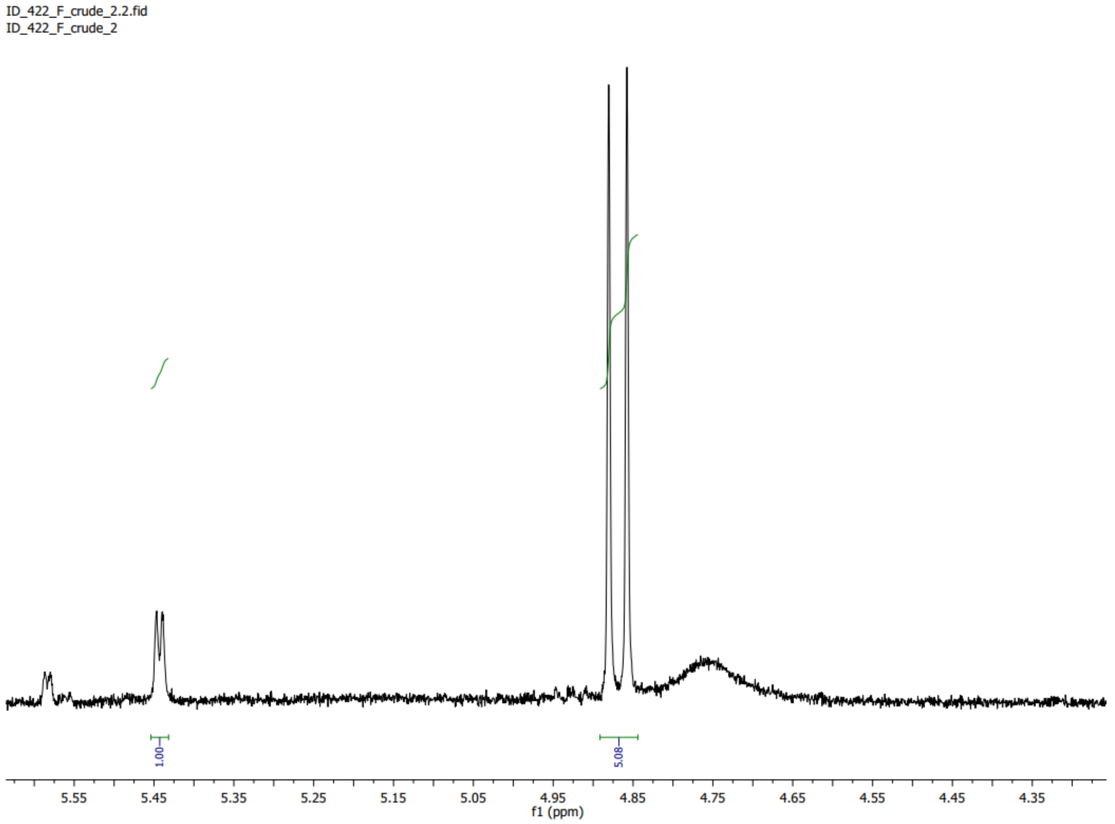

NMR traces for reaction with: **4-tBu-phenylboronic acid (400 MHz,** $CDCl_3$**)**

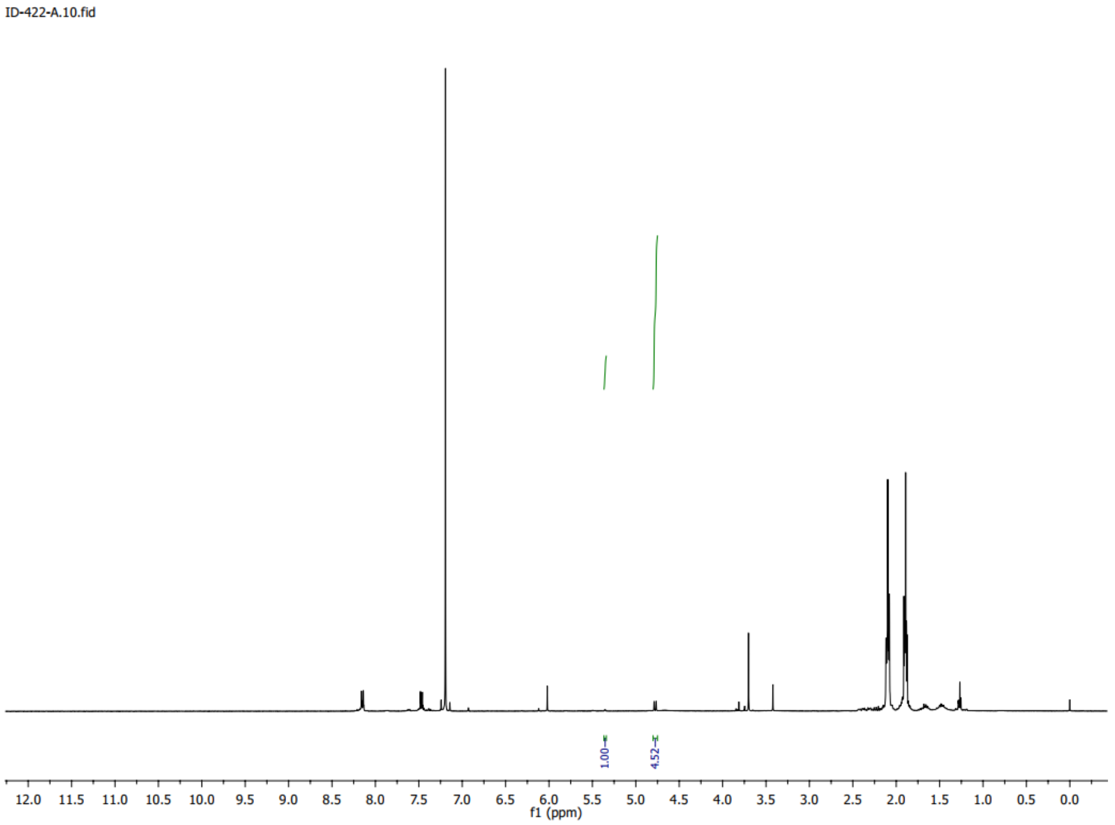

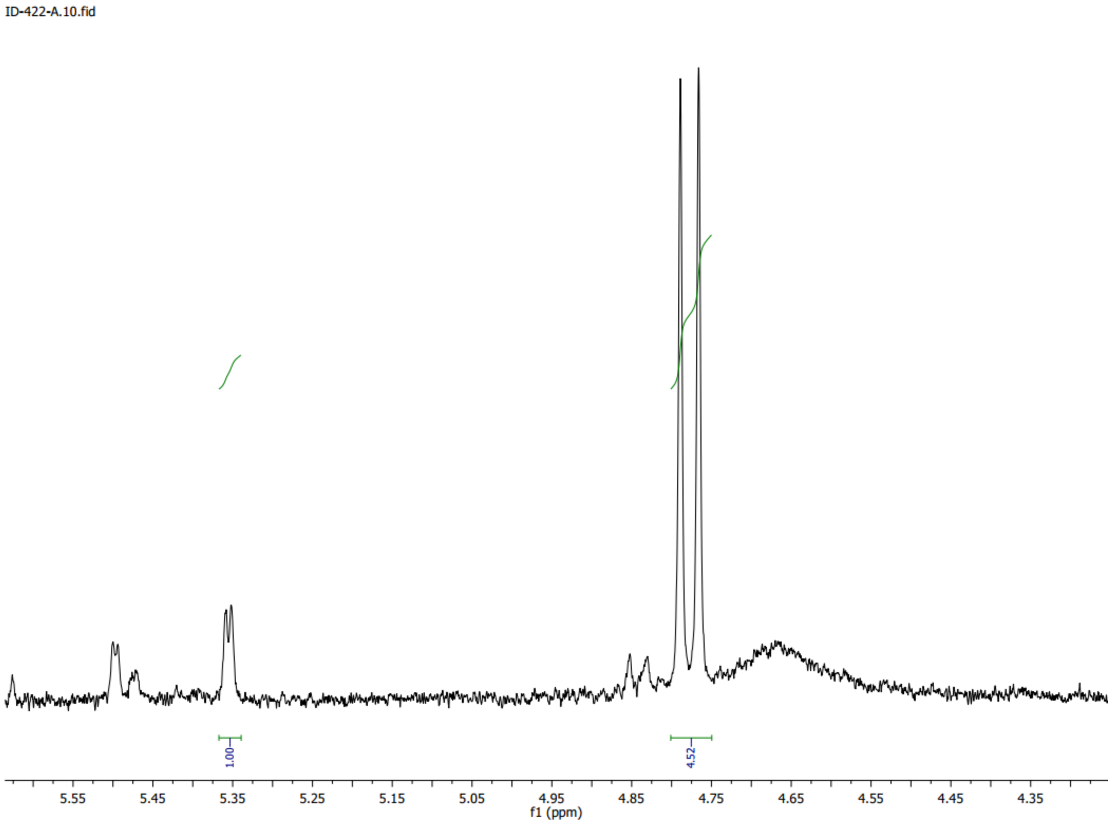

NMR traces for reaction with: **4-tBu-phenylboronic acid(duplicate) (400 MHz,** $CDCl_3$**)**

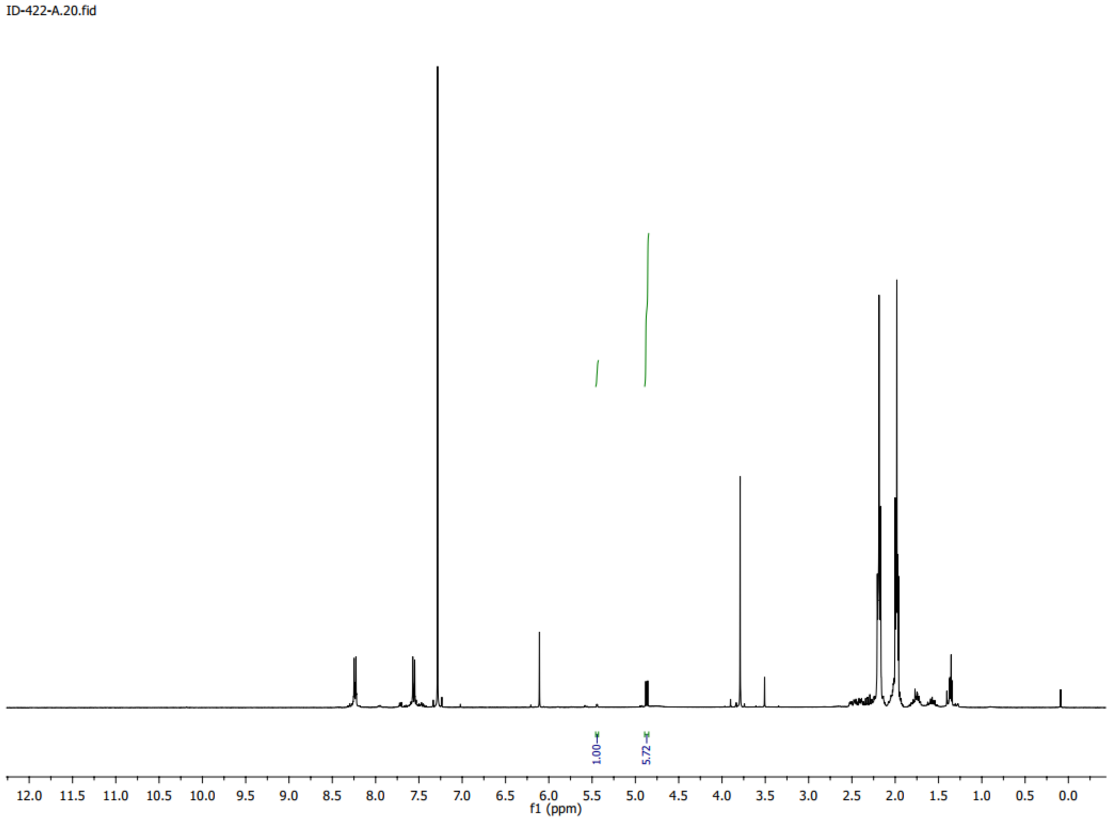

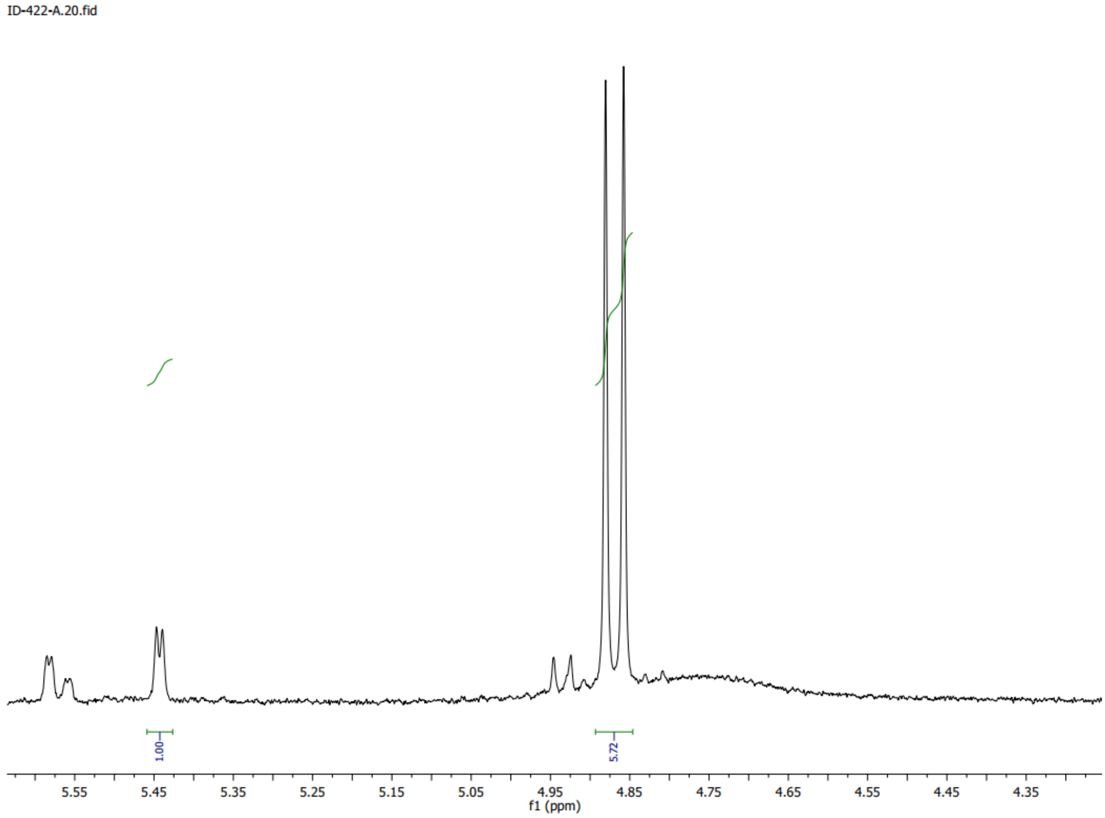

NMR traces for reaction with: **2,4-Me-phenylboronic acid (400 MHz,** $CDCl_3$**)**

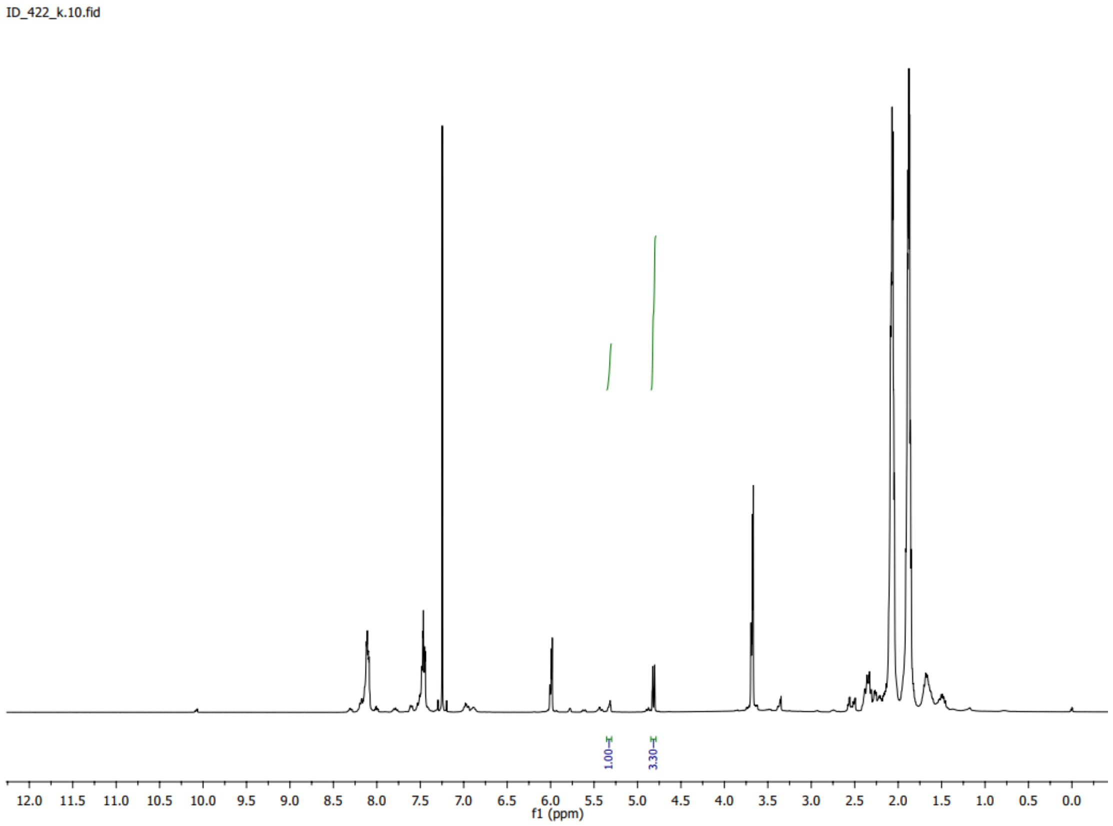

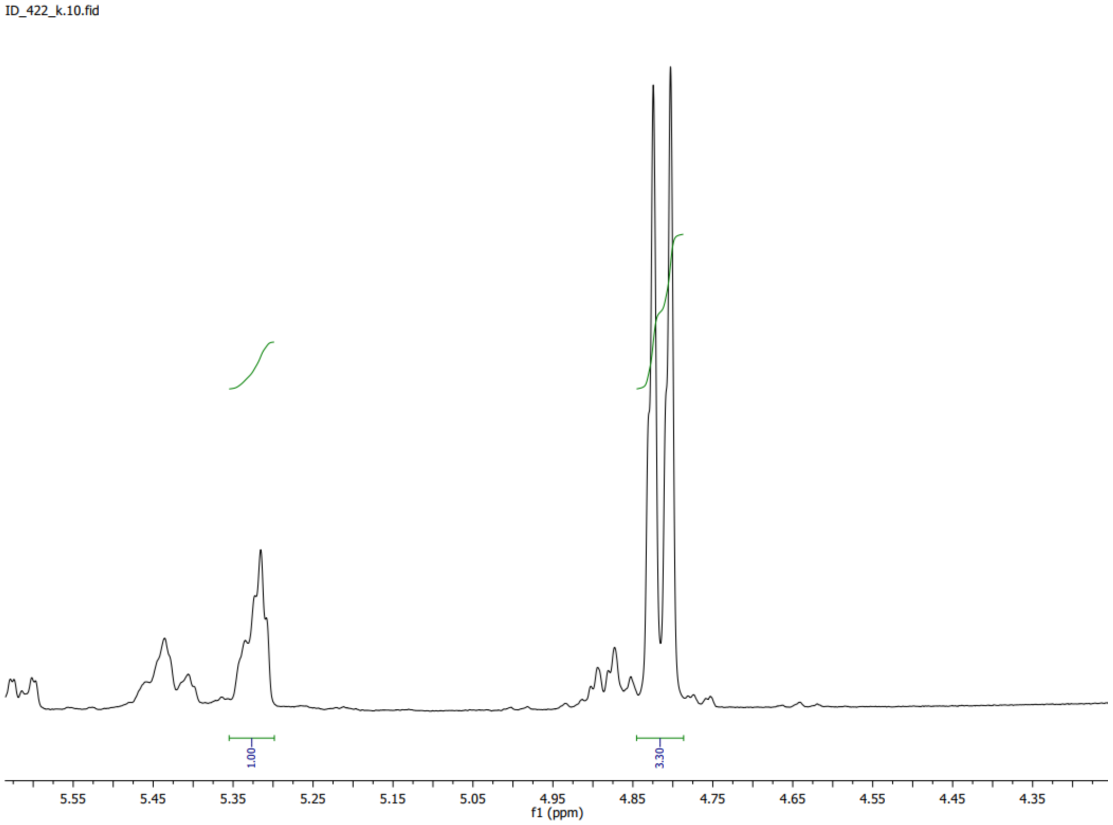

NMR traces for reaction with: **2,4-Me-phenylboronic acid (duplicate) (400 MHz,** $CDCl_3$**)**

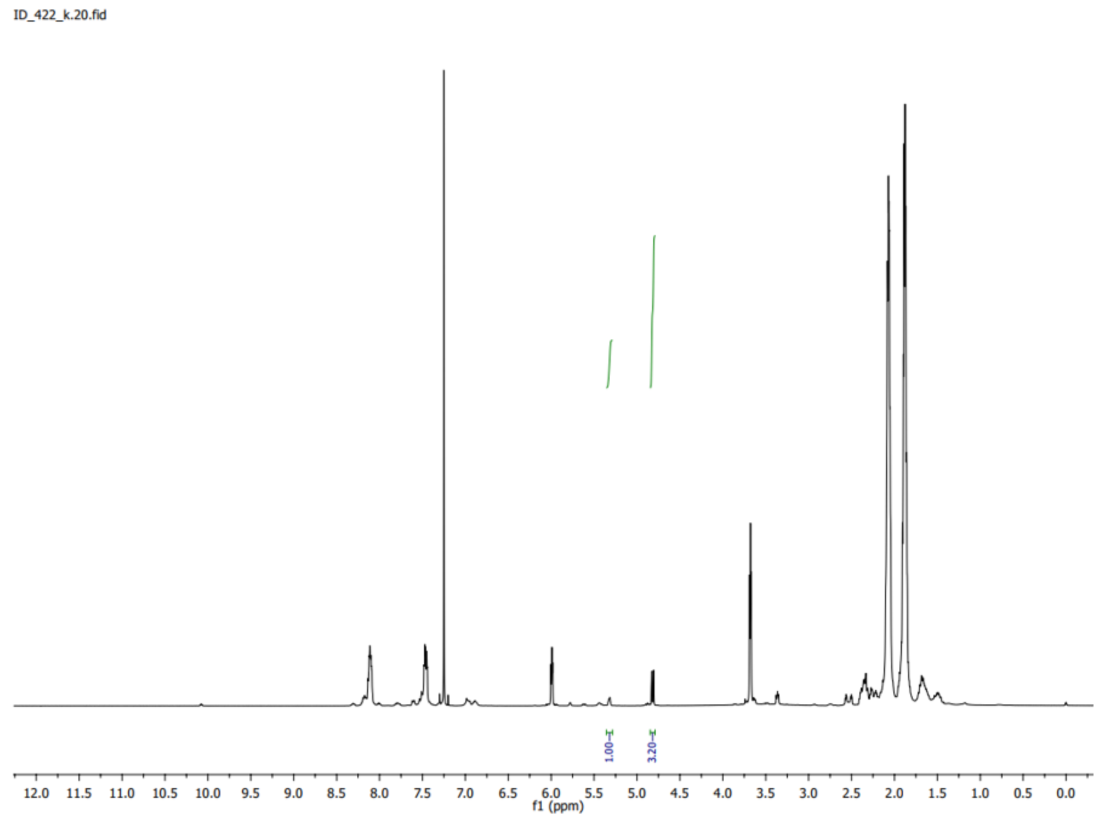

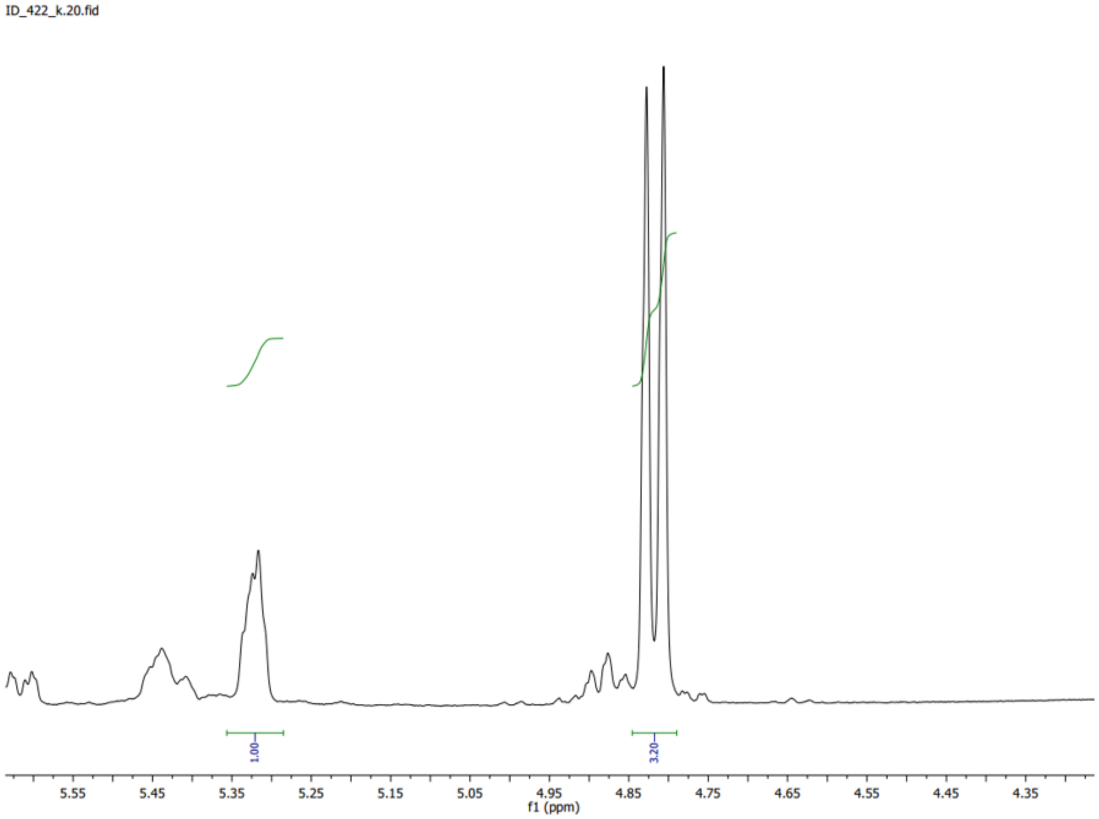

NMR traces for reaction with: **3,5-OMe-phenylboronic acid (400 MHz,** CDCl$_3$**)**

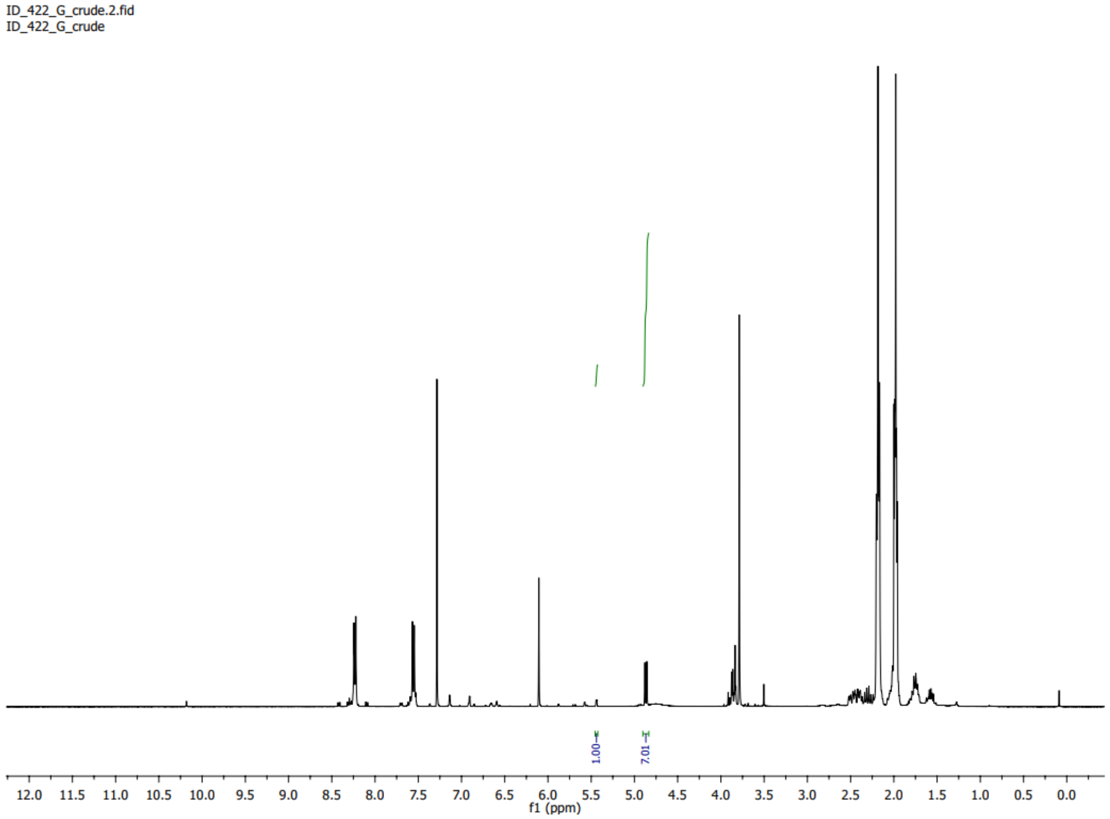

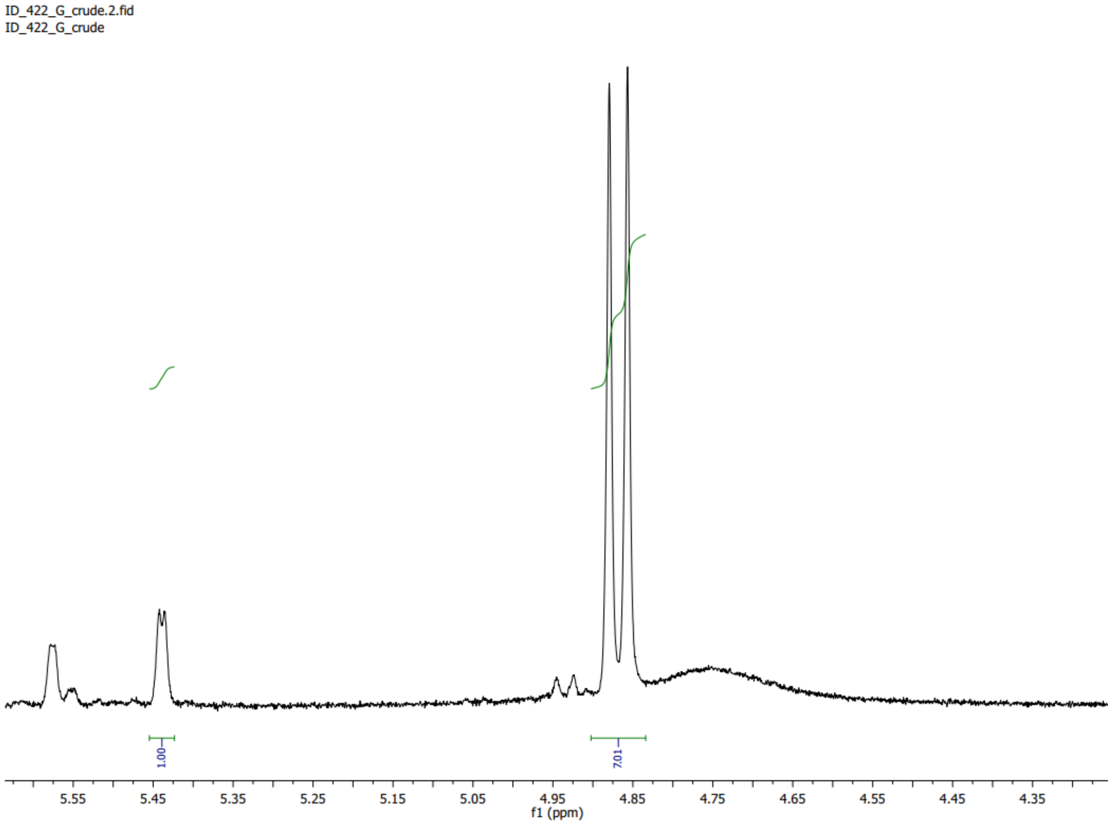

NMR traces for reaction with: **3,5-OMe-phenylboronic acid (duplicate) (400 MHz,** CDCl$_3$**)**

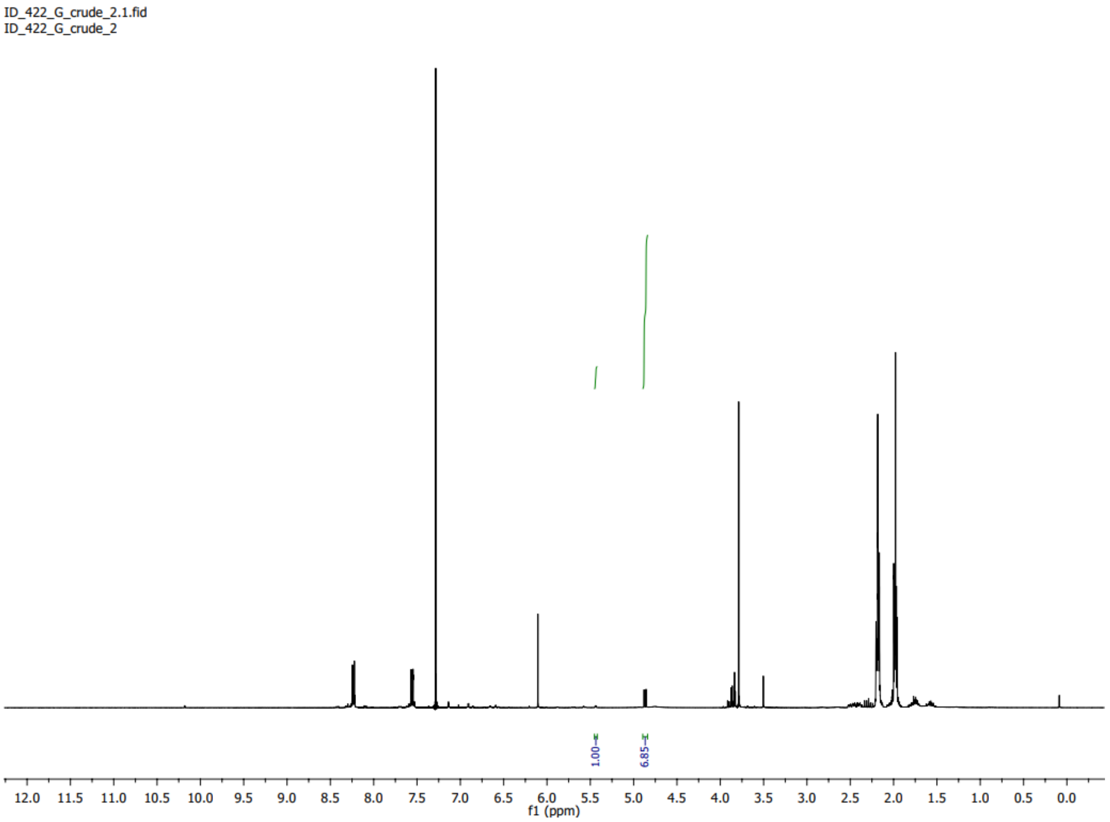

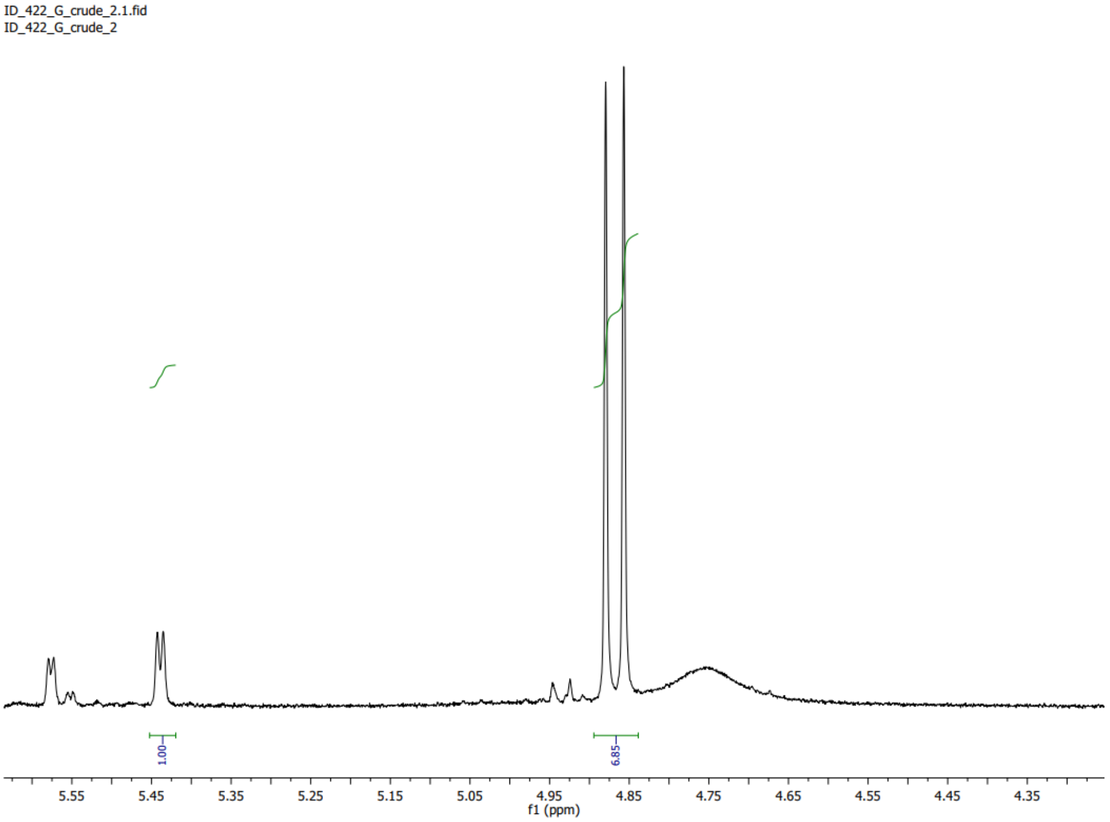

NMR traces for reaction with: **4-Me-phenylboronic acid (400 MHz,** CDCl$_3$**)**

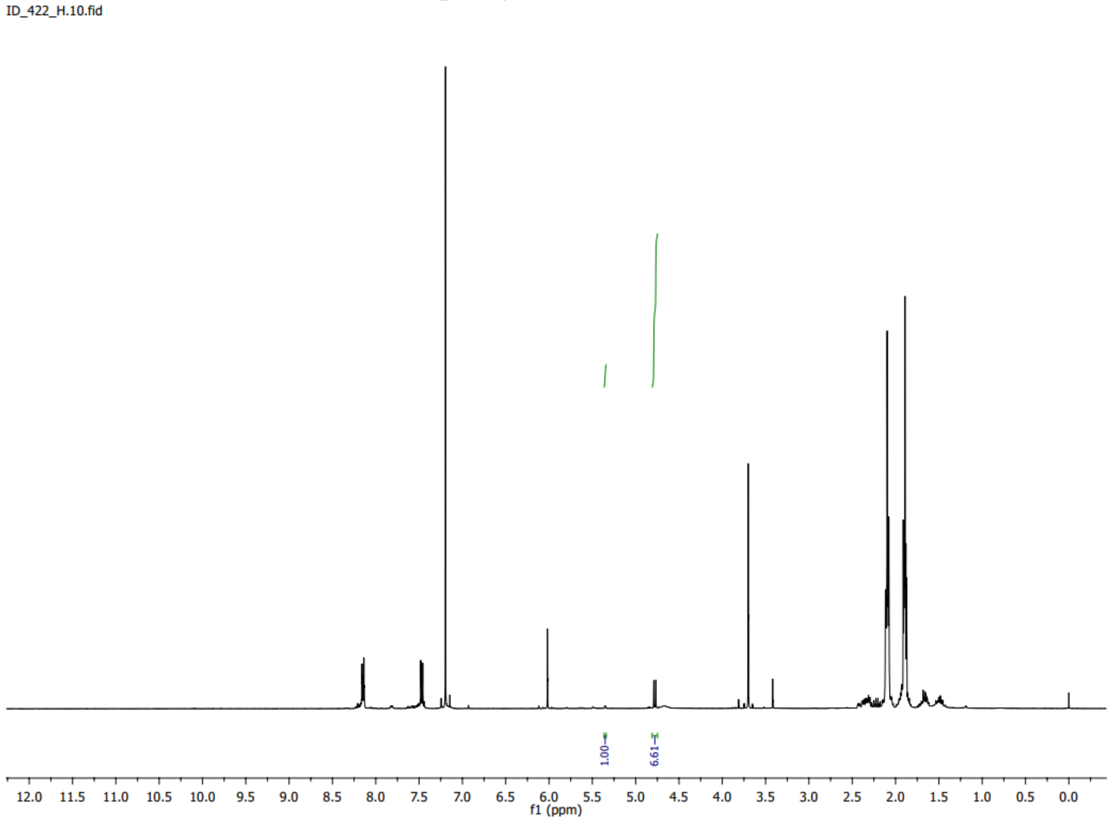

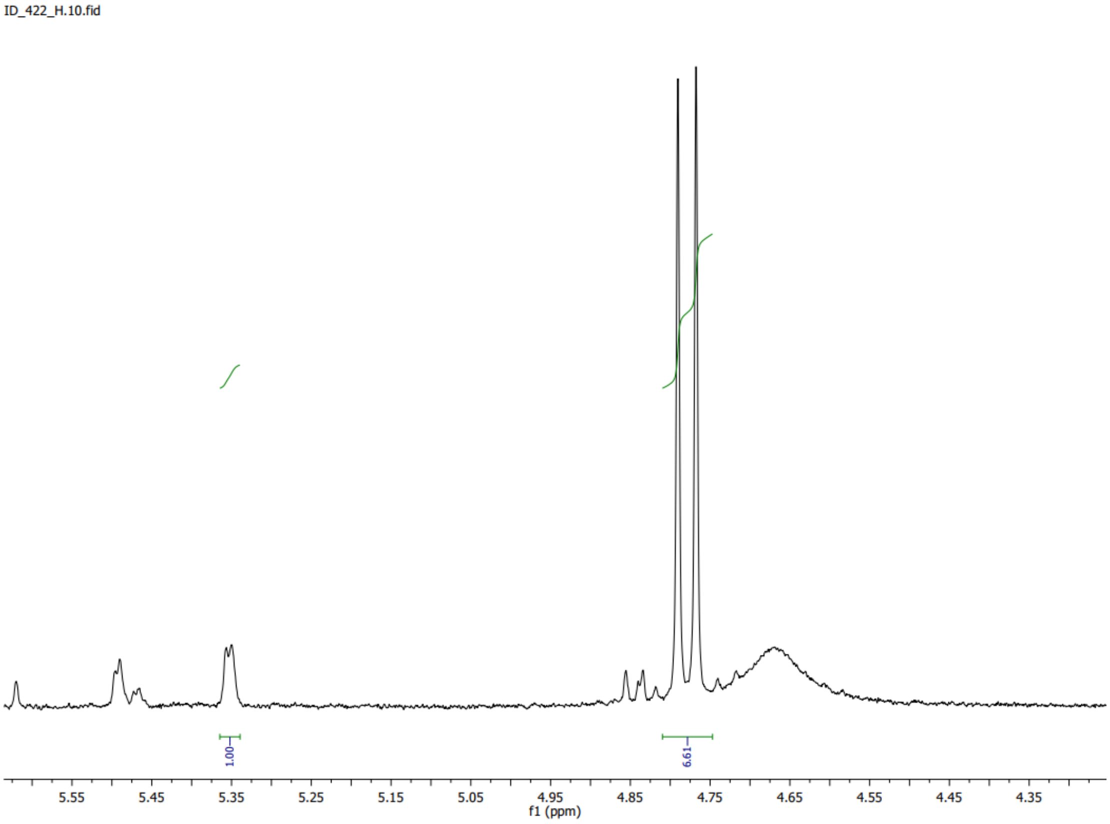

NMR traces for reaction with: **4-Me-phenylboronic acid (duplicate) (400 MHz,** CDCl$_3$**)**

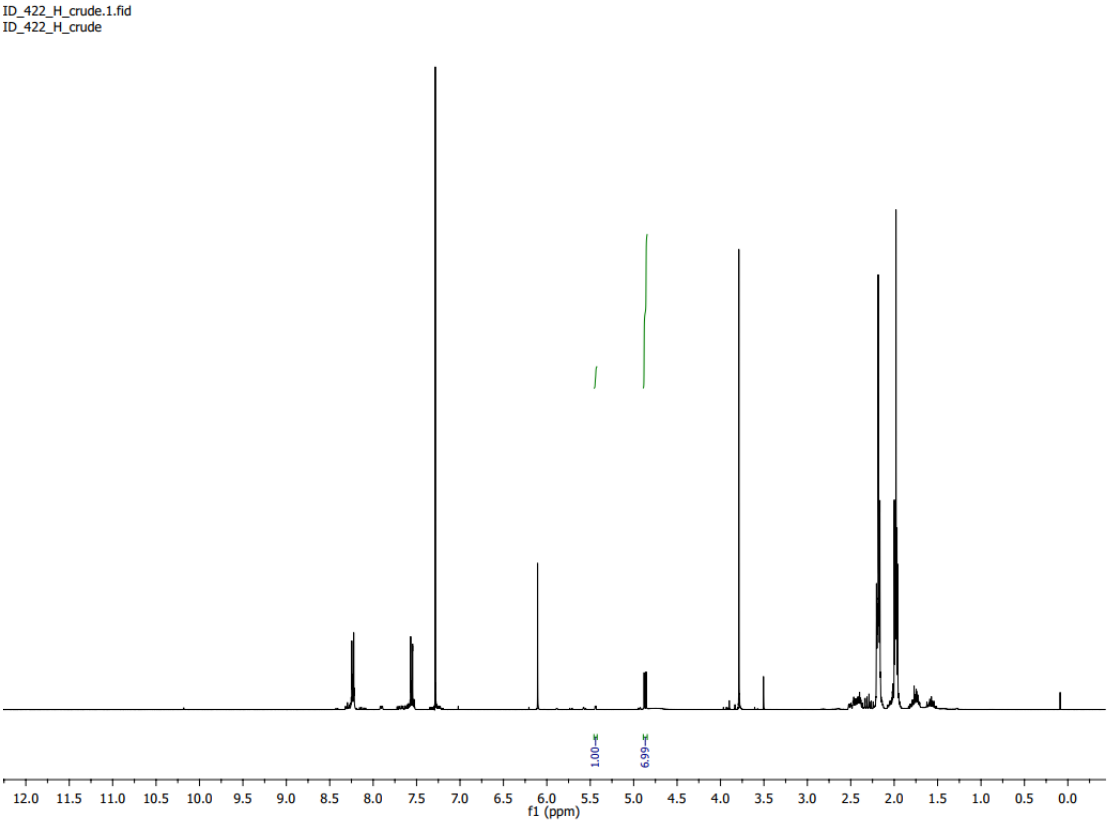

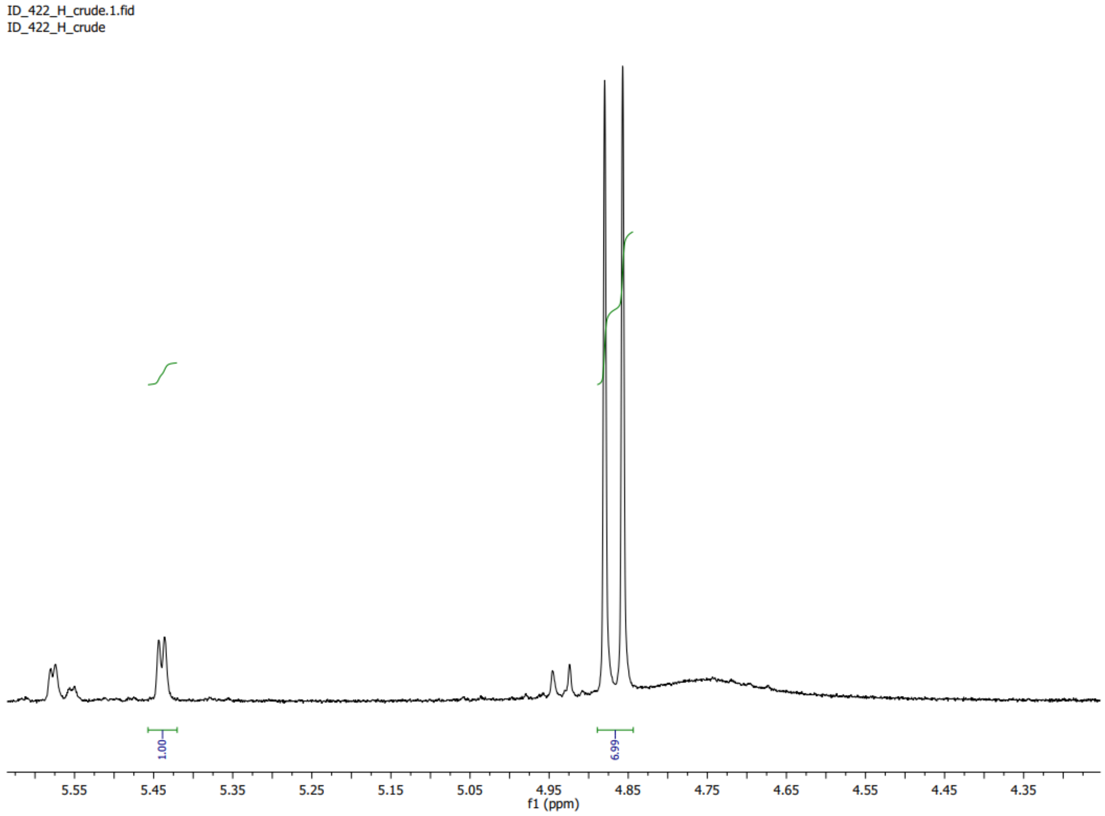

NMR traces for reaction with: **3-CF_3-phenylboronic acid (400 MHz,** $CDCl_3$**)**

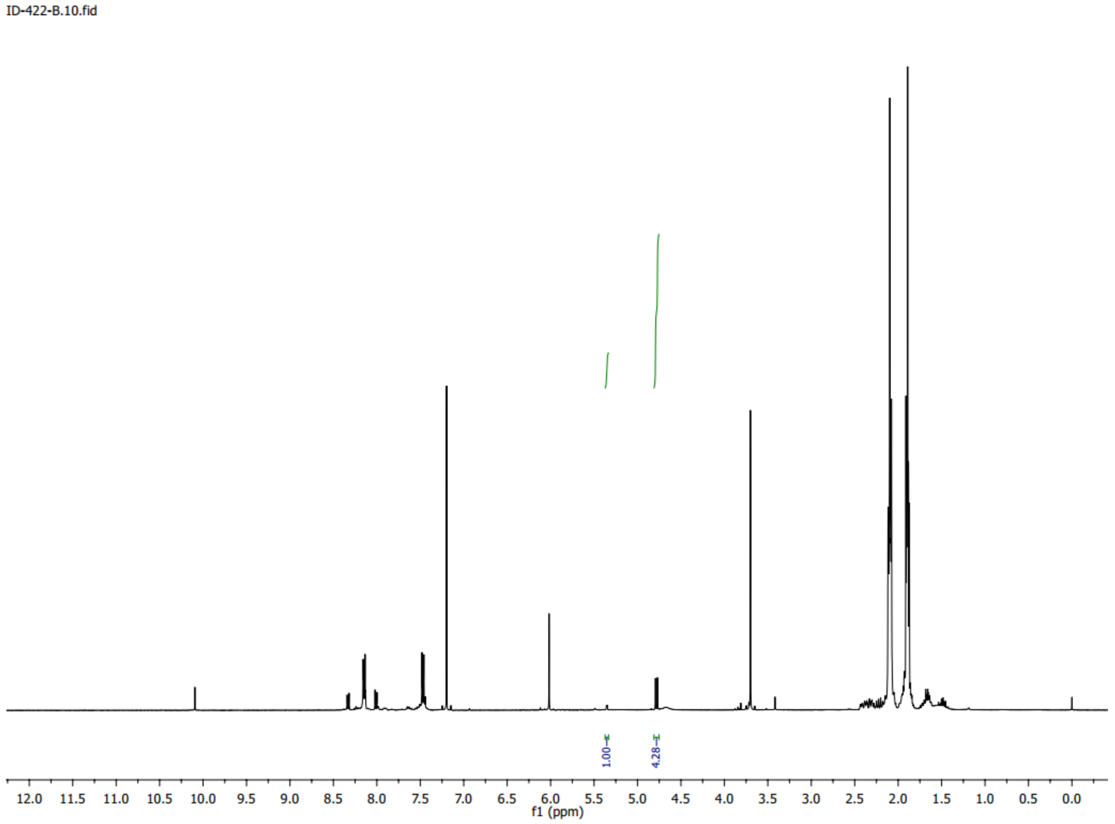

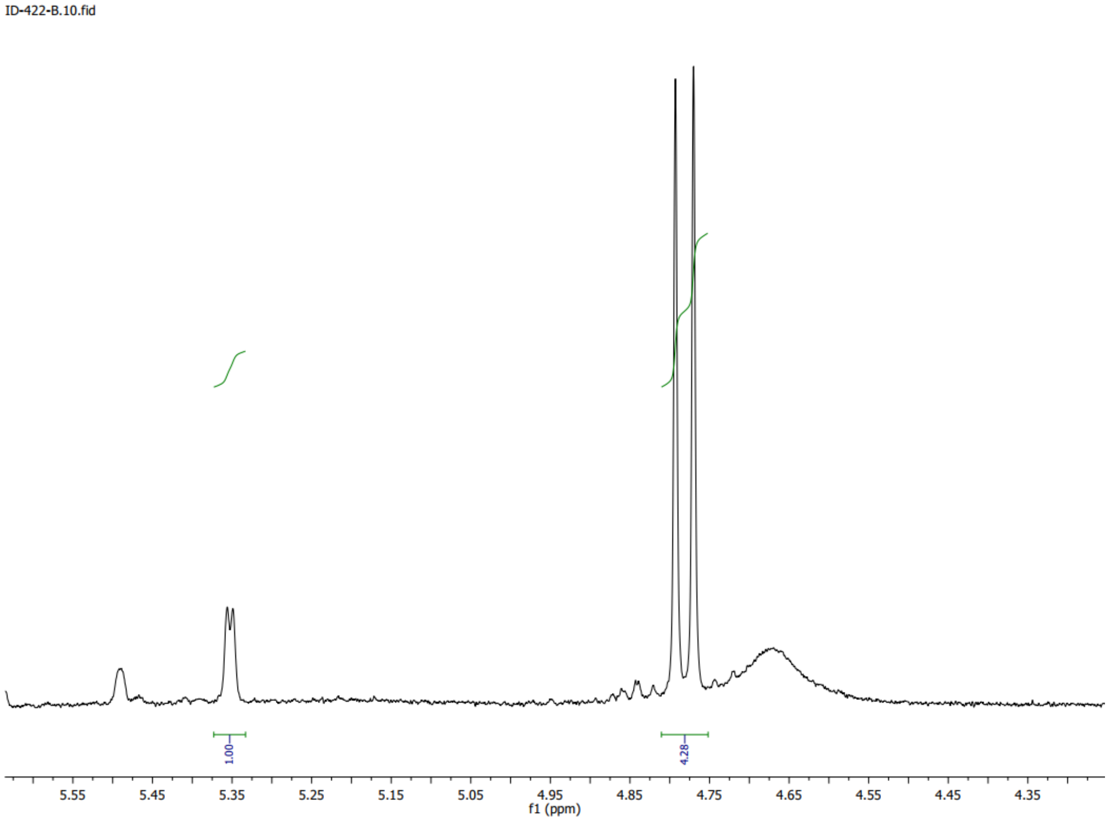

NMR traces for reaction with: **3-CF_3-phenylboronic acid (duplicate) (400 MHz,** $CDCl_3$**)**

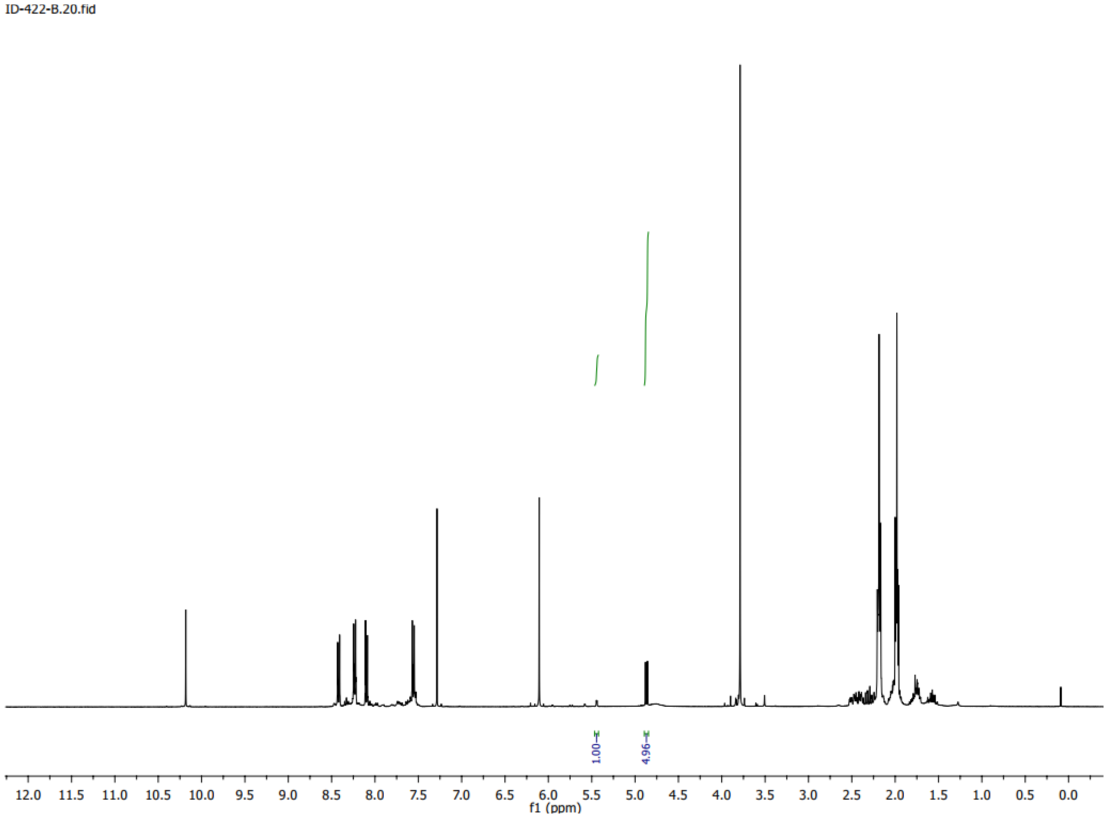

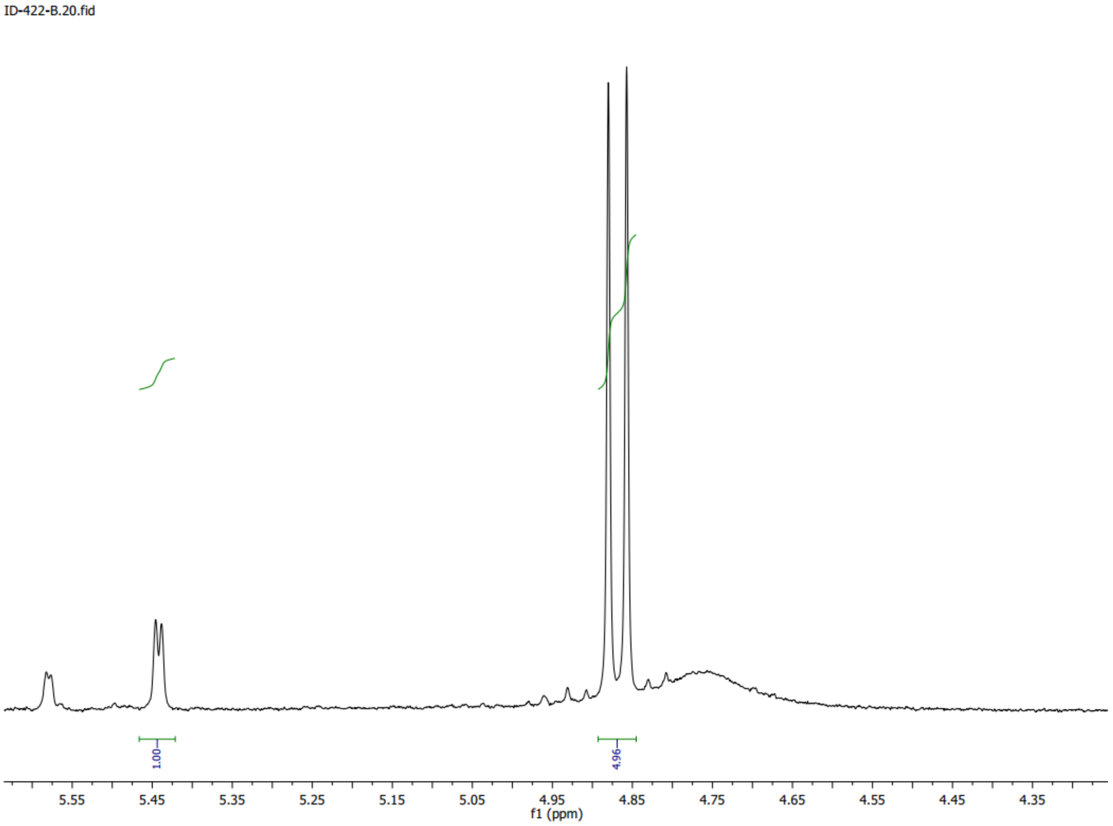

NMR traces for reaction with: **2-naphtalene-phenylboronic acid (400 MHz,** $CDCl_3$**)**

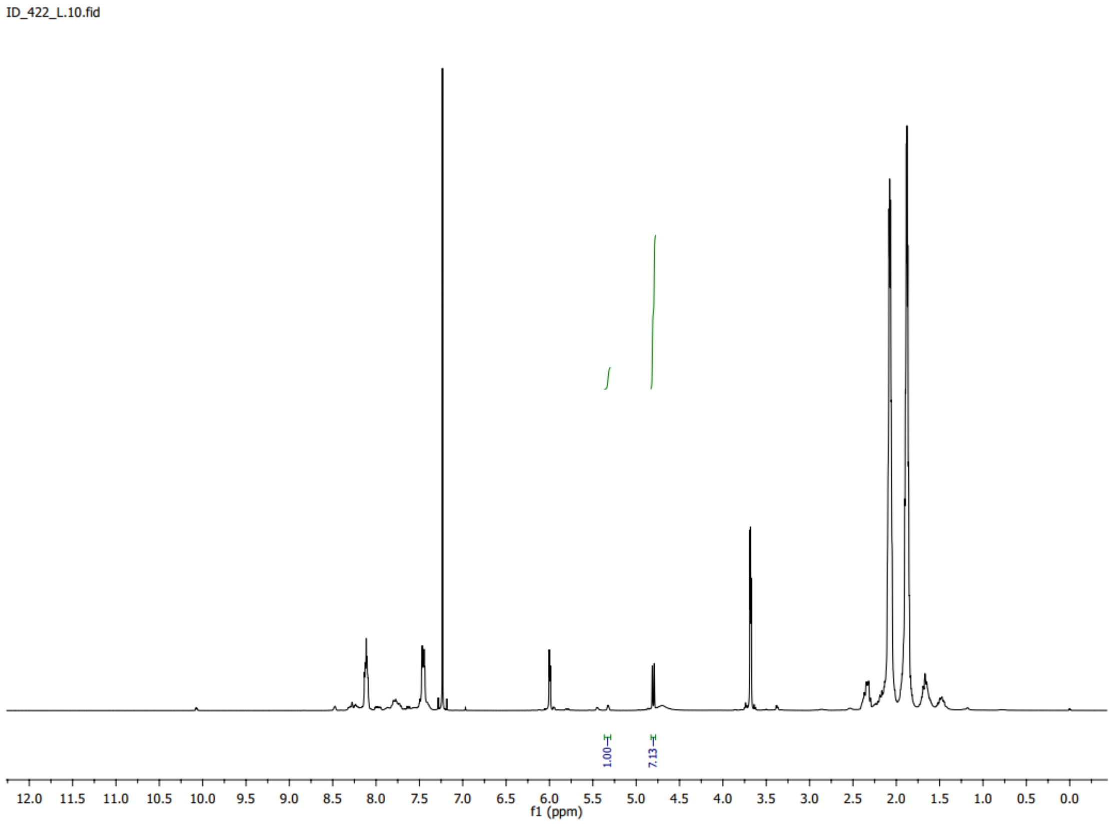

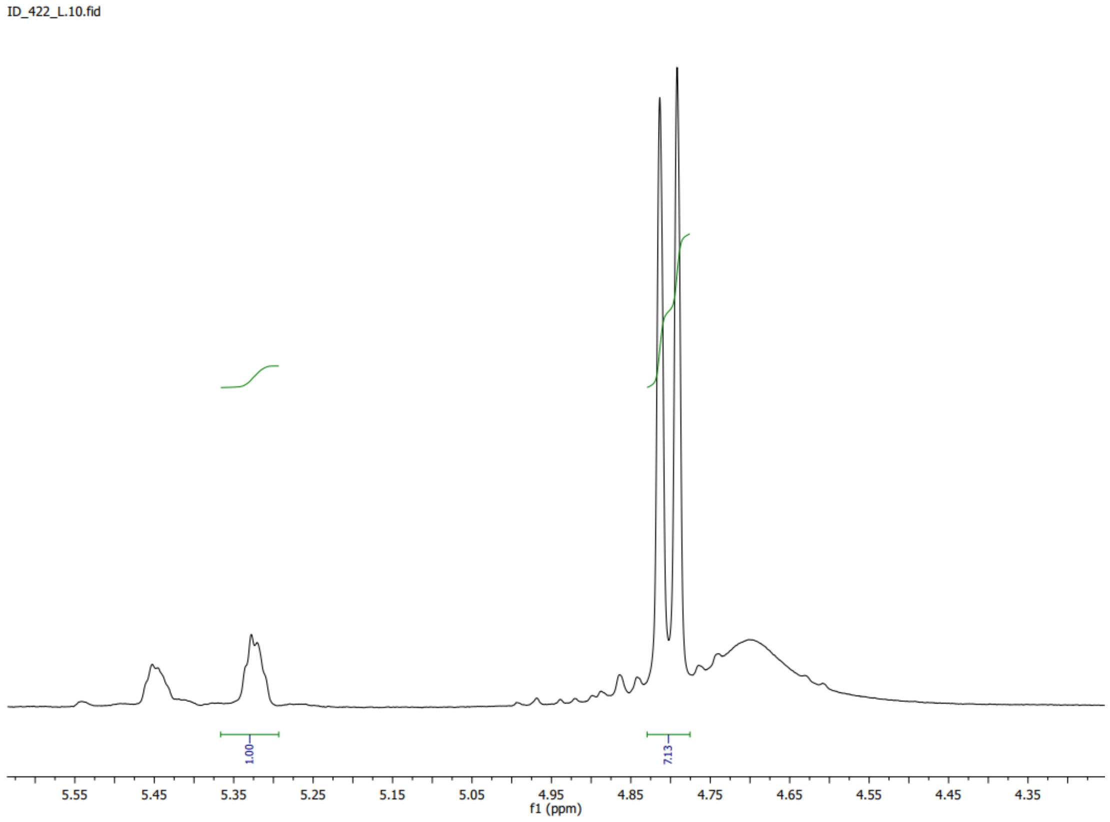

NMR traces for reaction with: **2-naphtalene-phenylboronic acid (duplicate) (400 MHz,** $CDCl_3$**)**

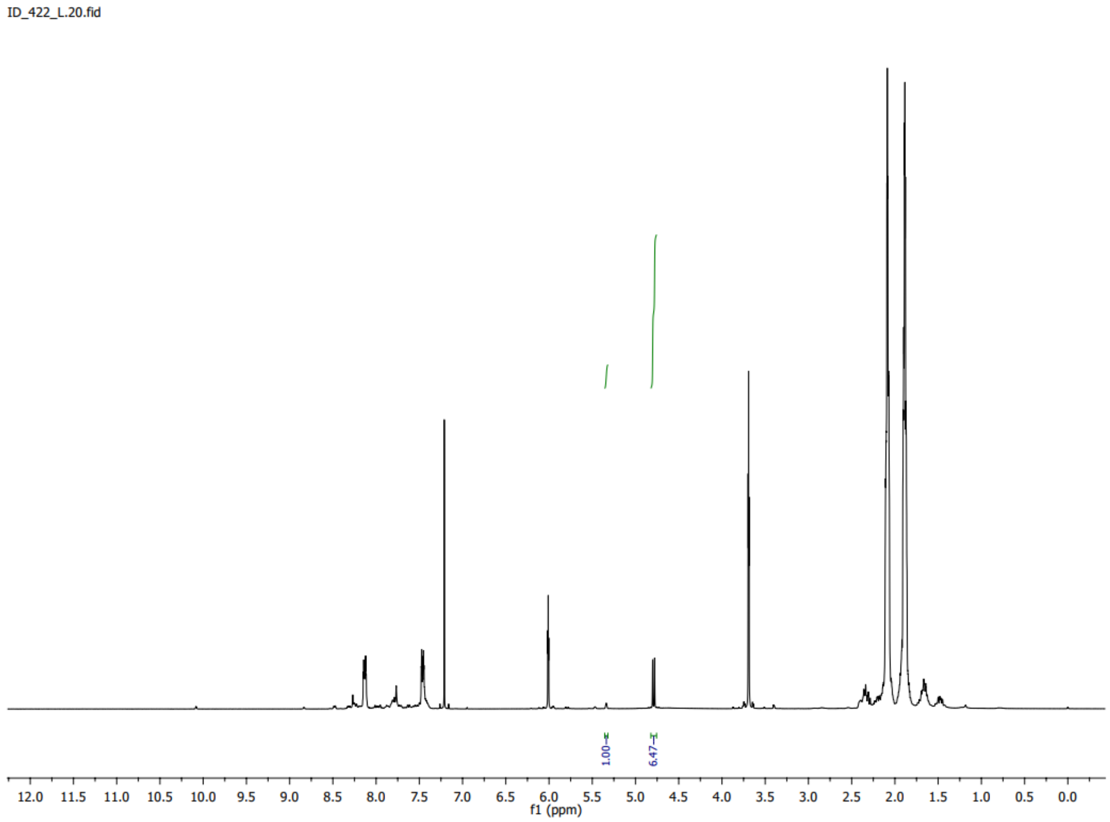

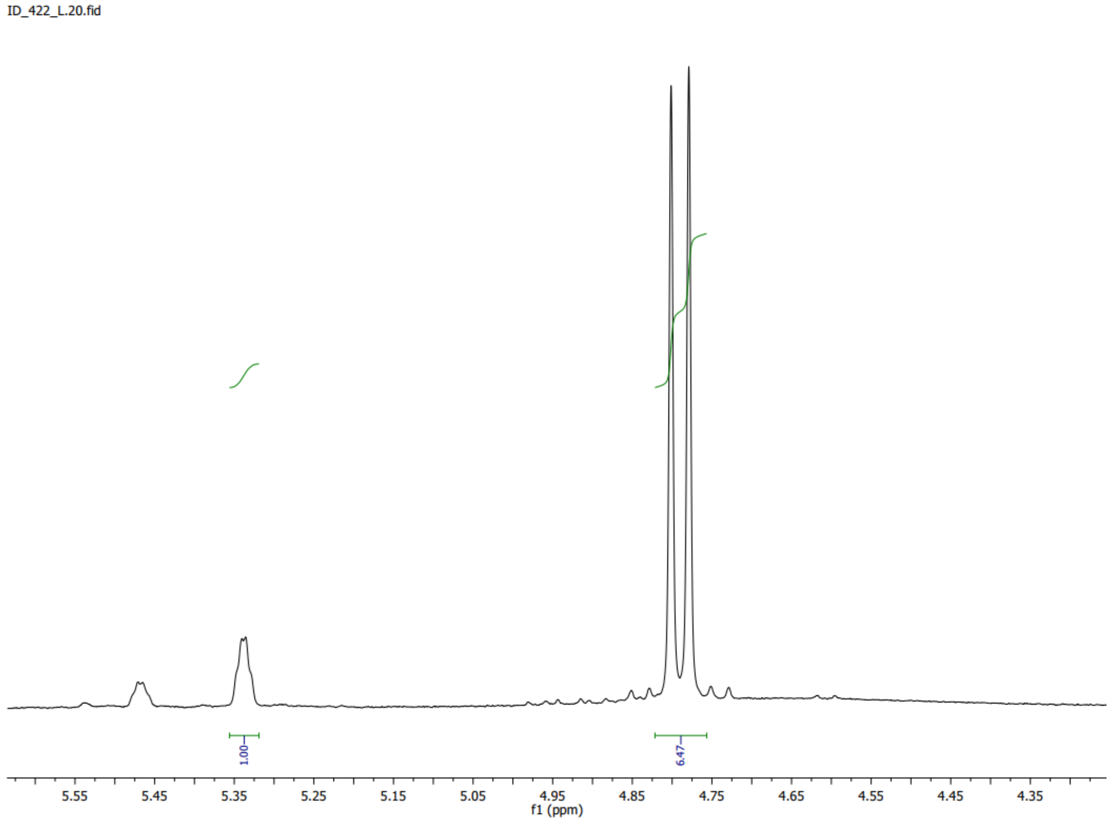

NMR traces for reaction with: **4-CF$_3$-phenylboronic acid (400 MHz,** $CDCl_3$**)**

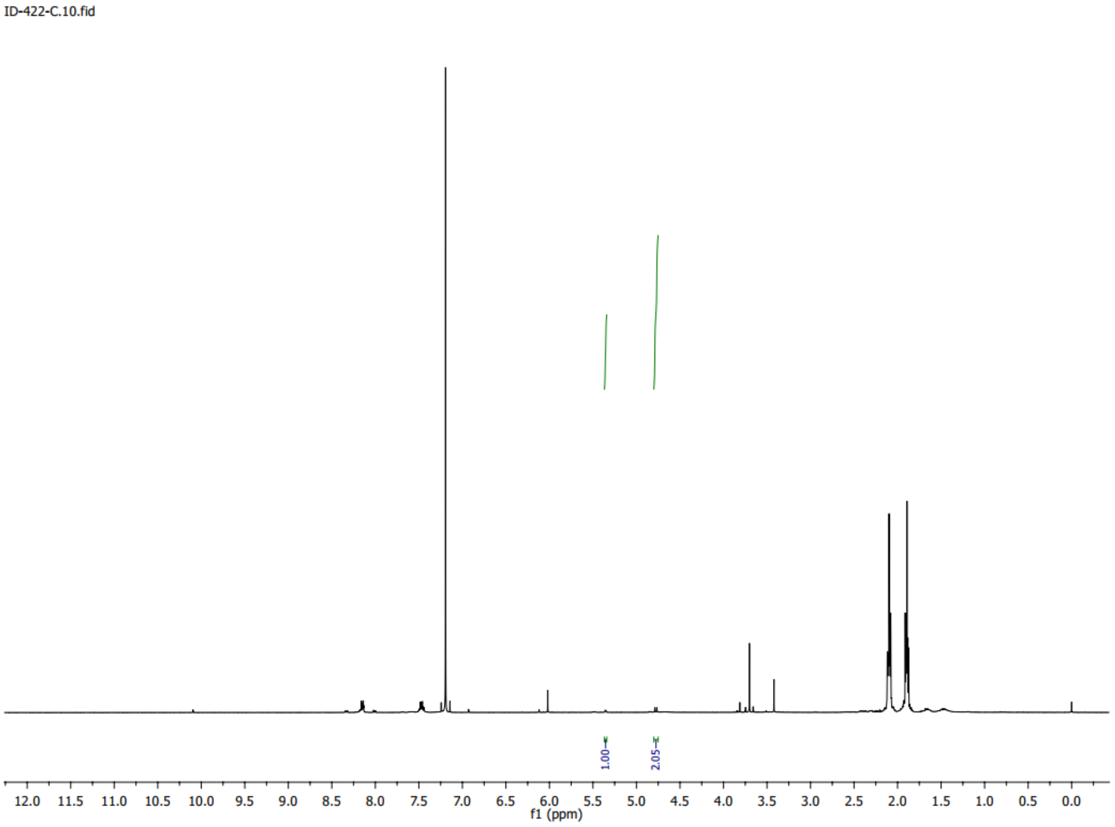

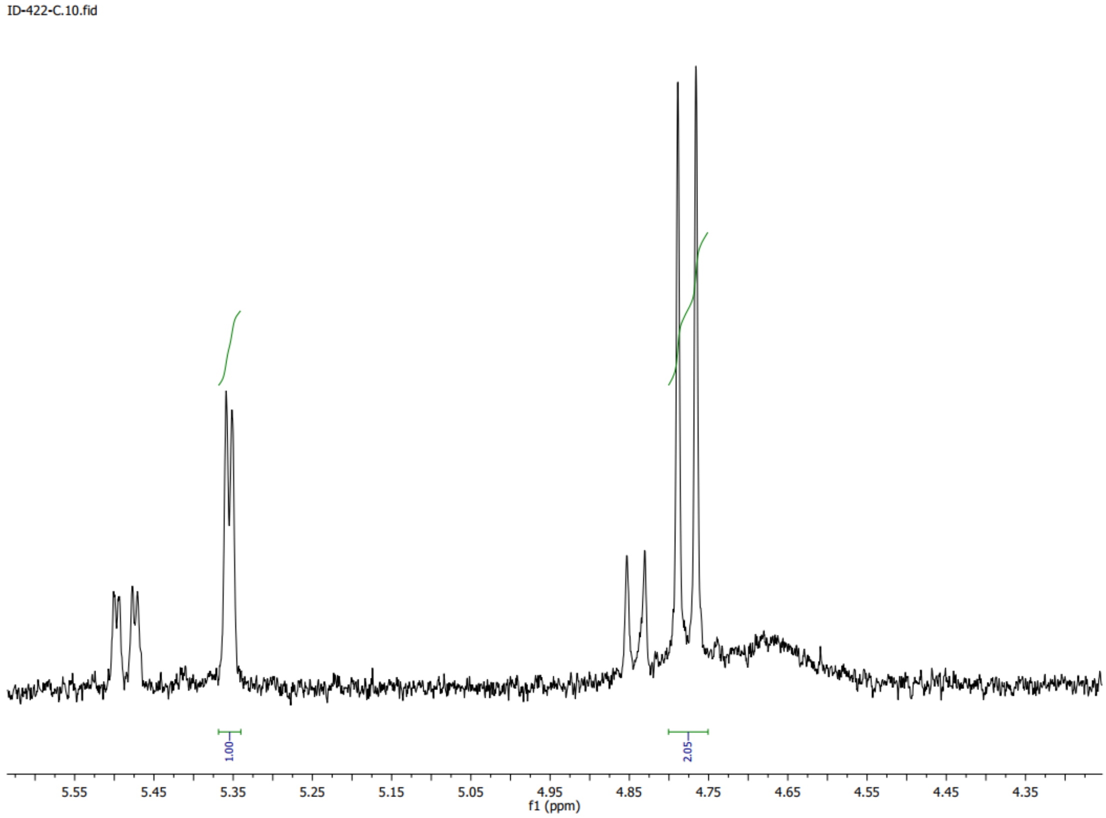

NMR traces for reaction with: **4-CF_3-phenylboronic acid (duplicate) (400 MHz,** $CDCl_3$**)**

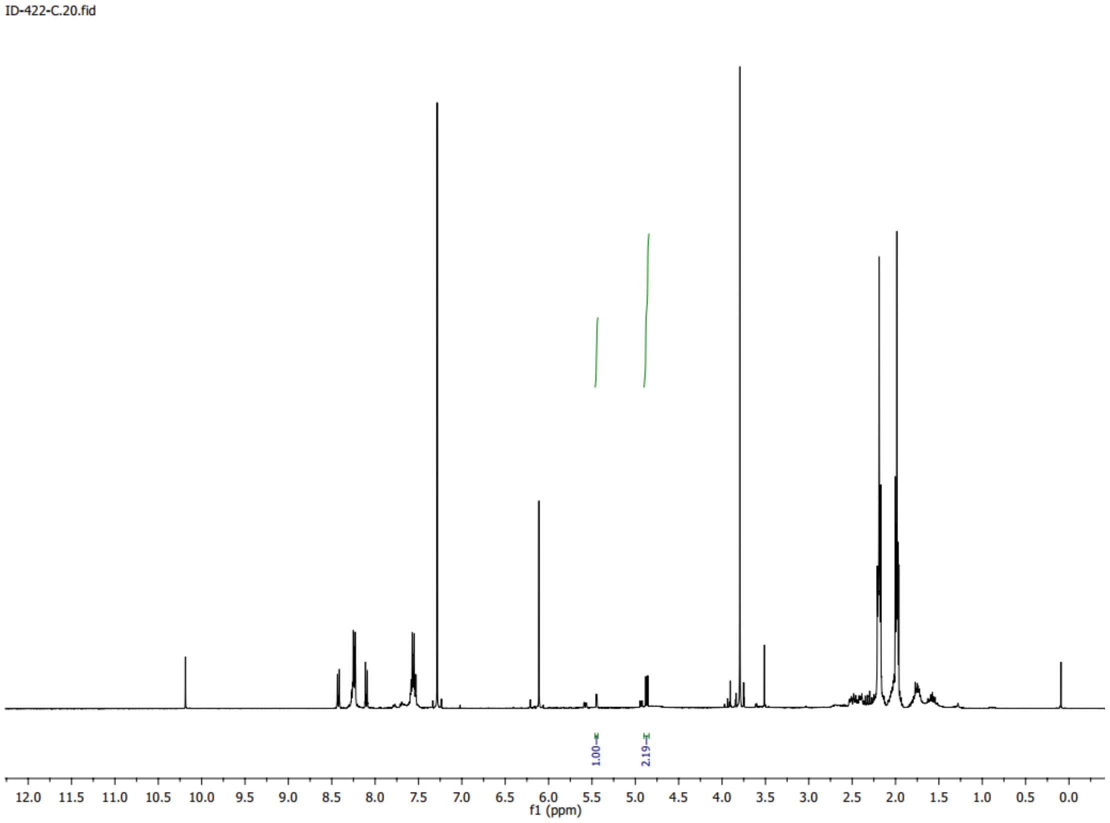

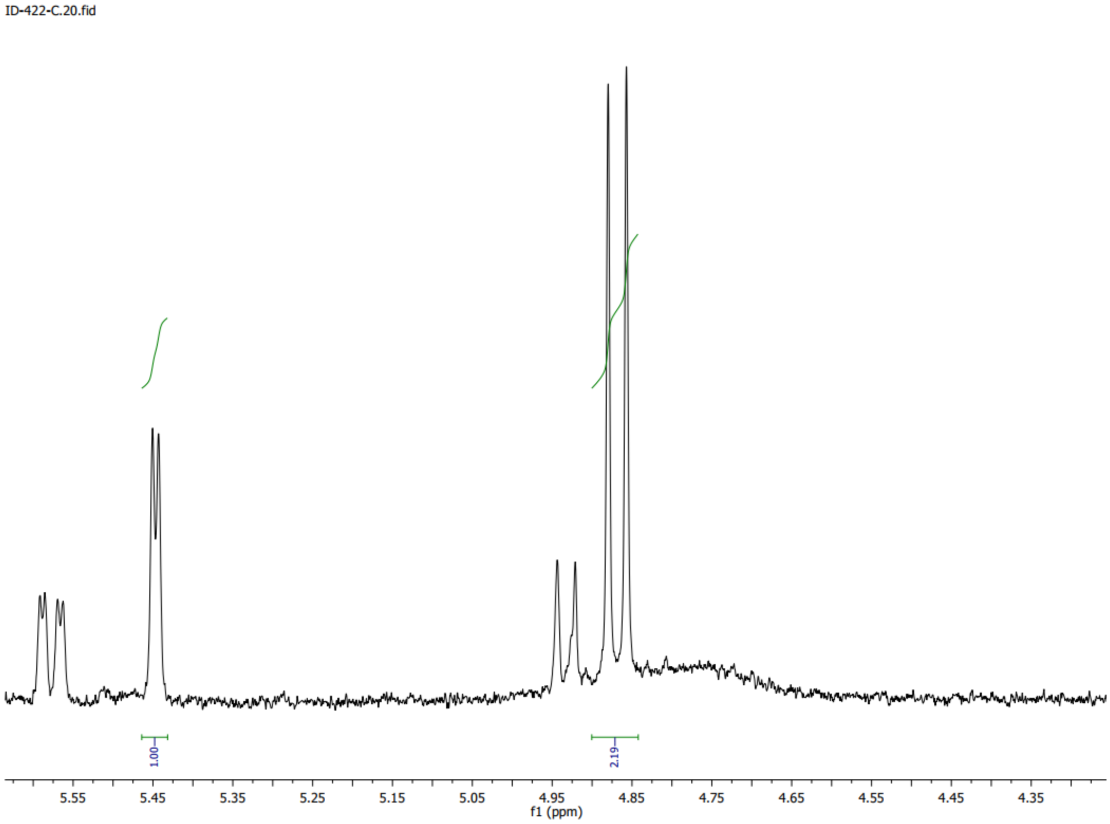

NMR traces for reaction with: **4-F-phenylboronic acid (400 MHz,** $CDCl_3$**)**

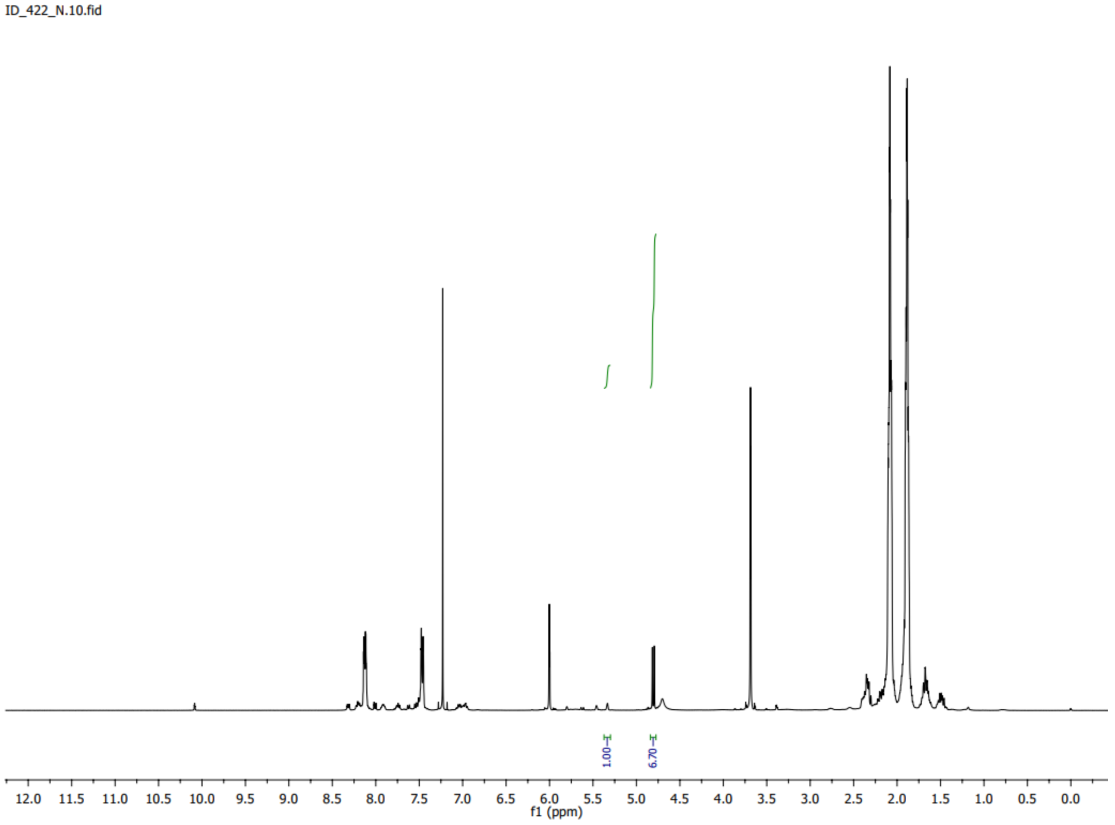

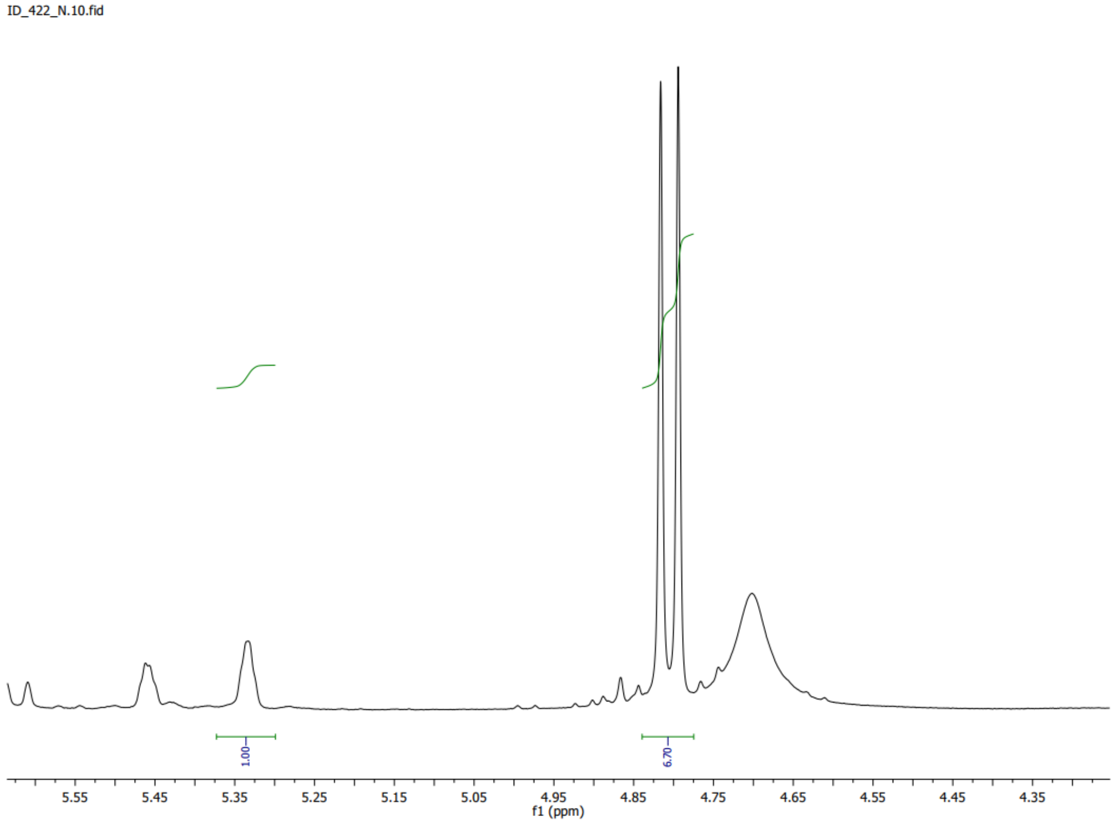

NMR traces for reaction with: **4-F-phenylboronic acid (duplicate) (400 MHz,** $CDCl_3$**)**

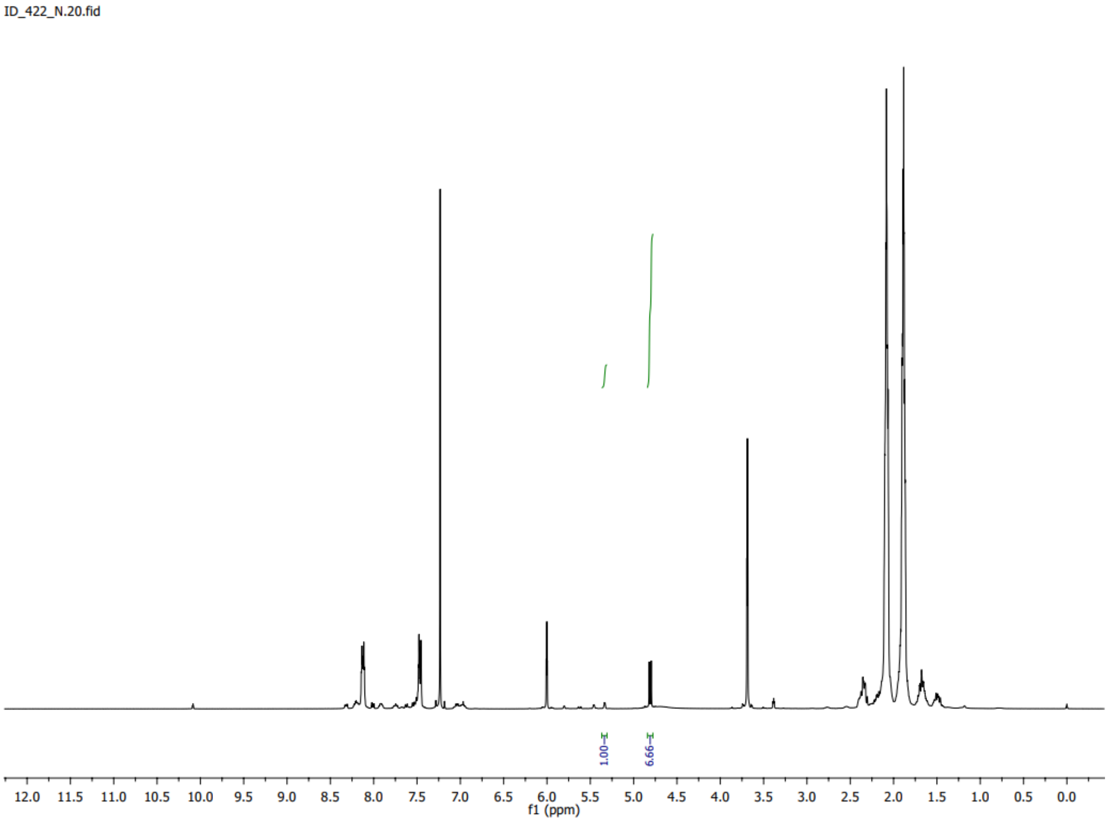

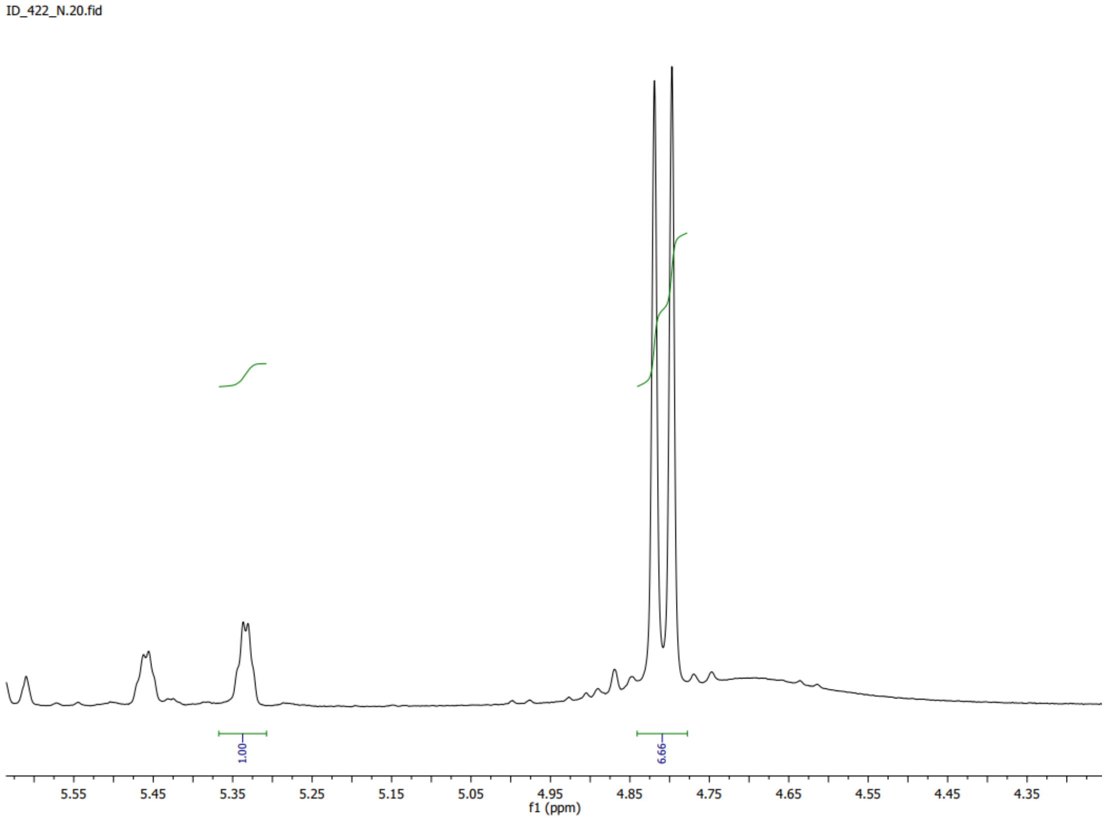

NMR traces for reaction with: **4-OMe-phenylboronic acid (400 MHz,** $CDCl_3$**)**

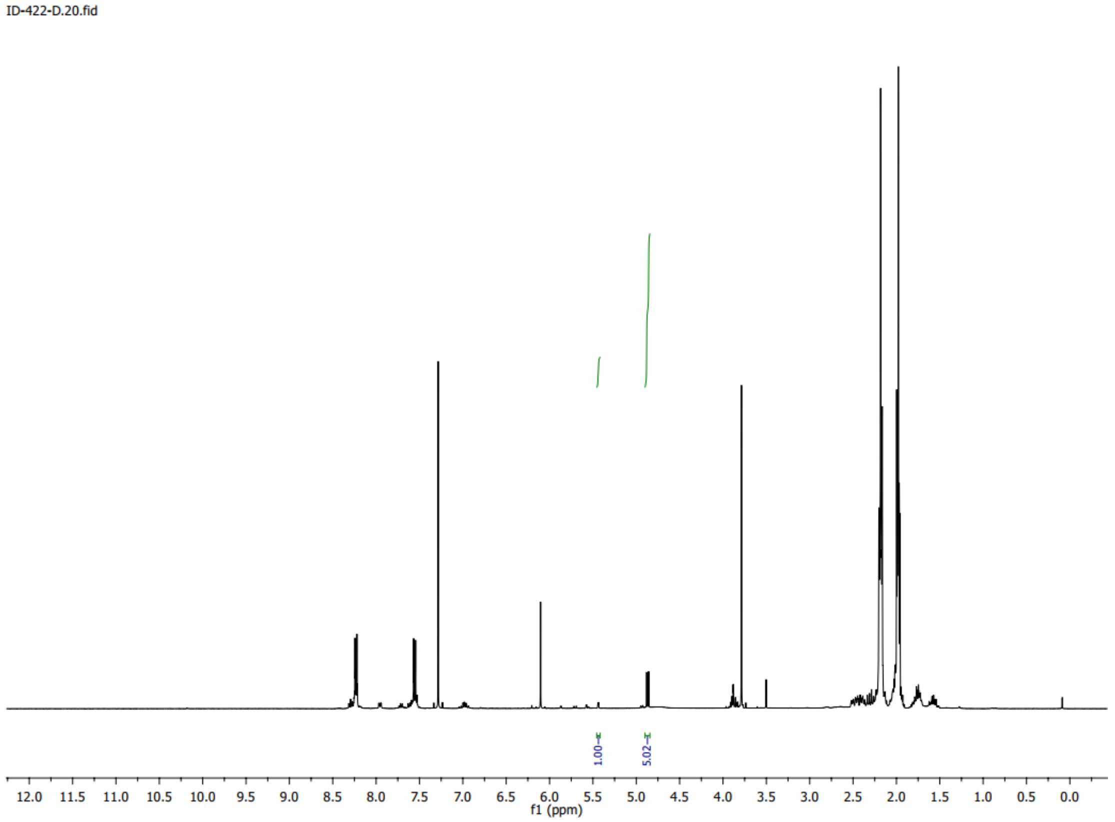

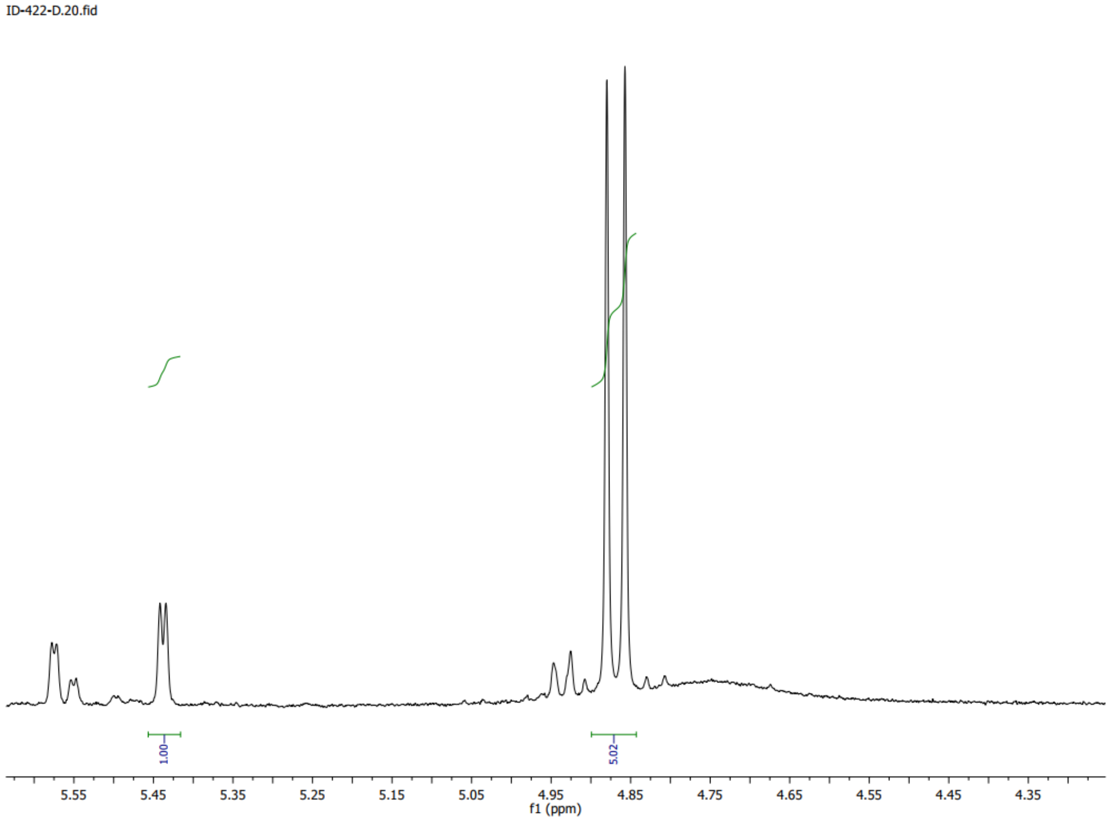

NMR traces for reaction with: **4-OMe-phenylboronic acid (duplicate) (400 MHz,** $CDCl_3$**)**

ID-422-D.30.fid

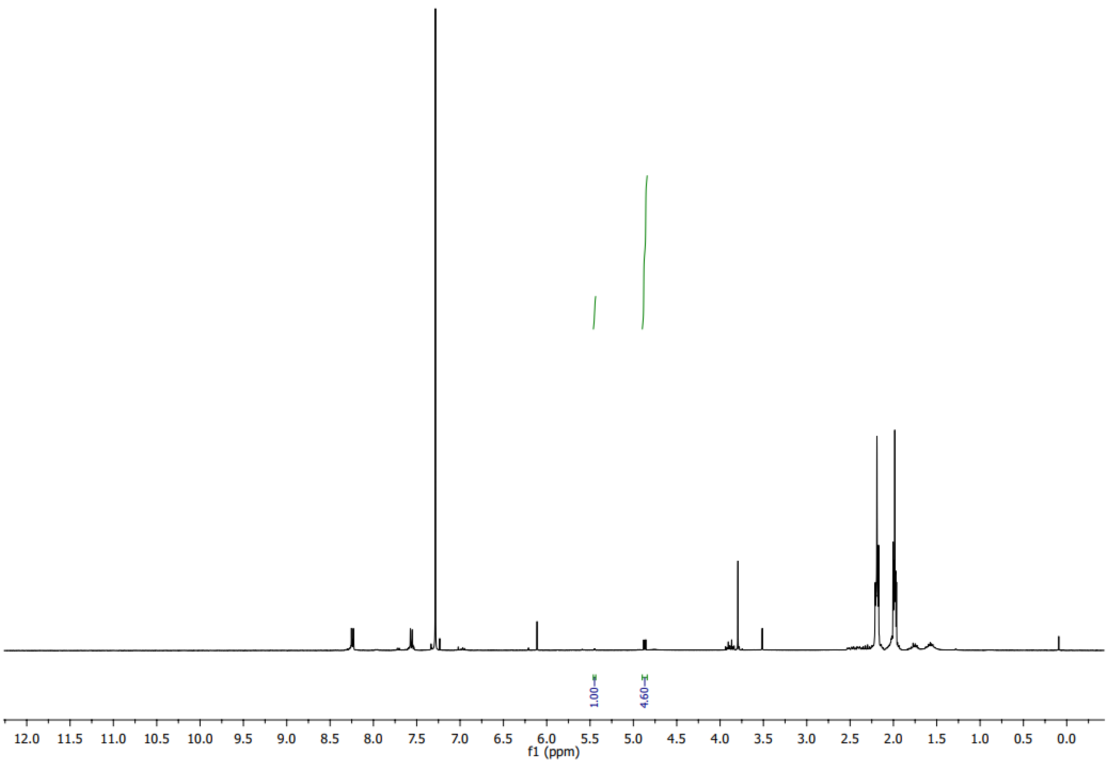

ID-422-D.30.fid

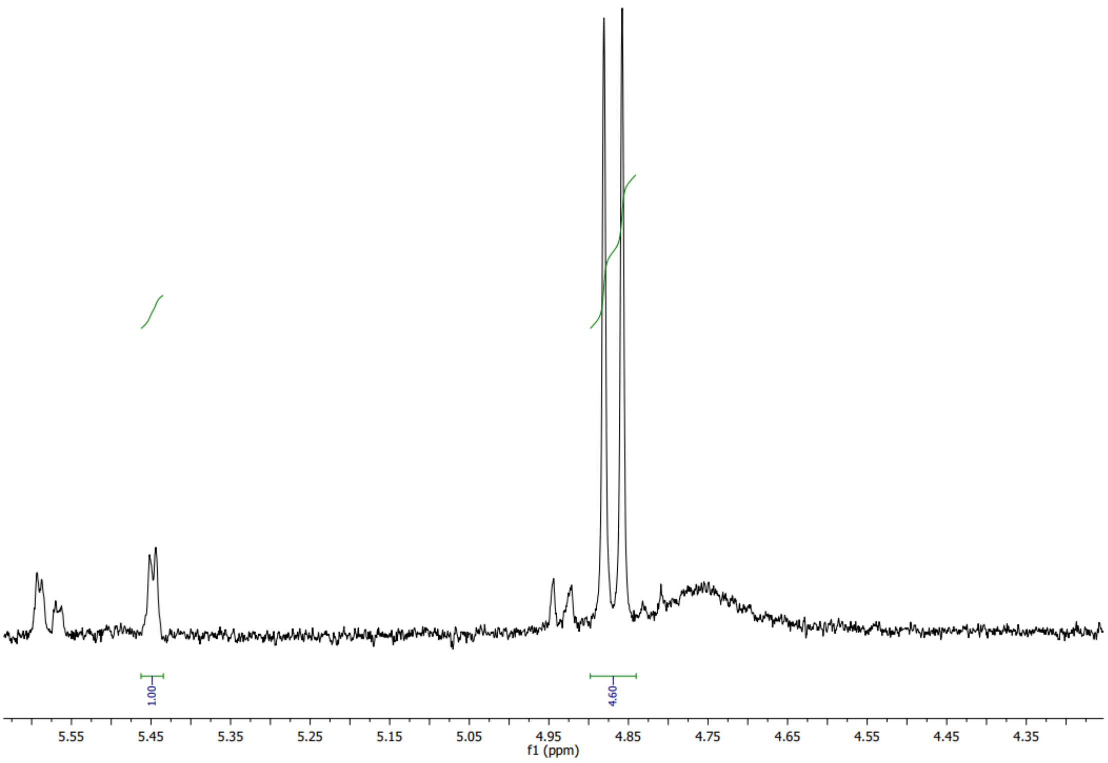

NMR traces for reaction with: **3-Me-phenylboronic acid (400 MHz,** $CDCl_3$**)**

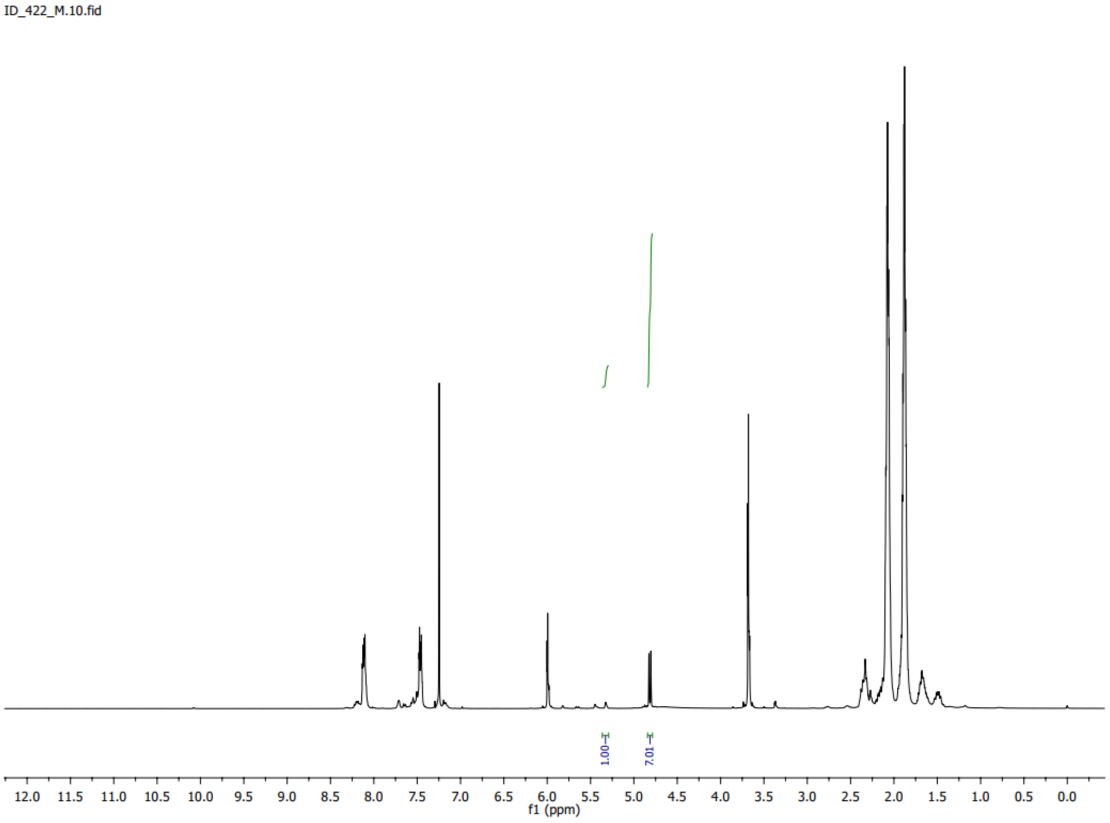

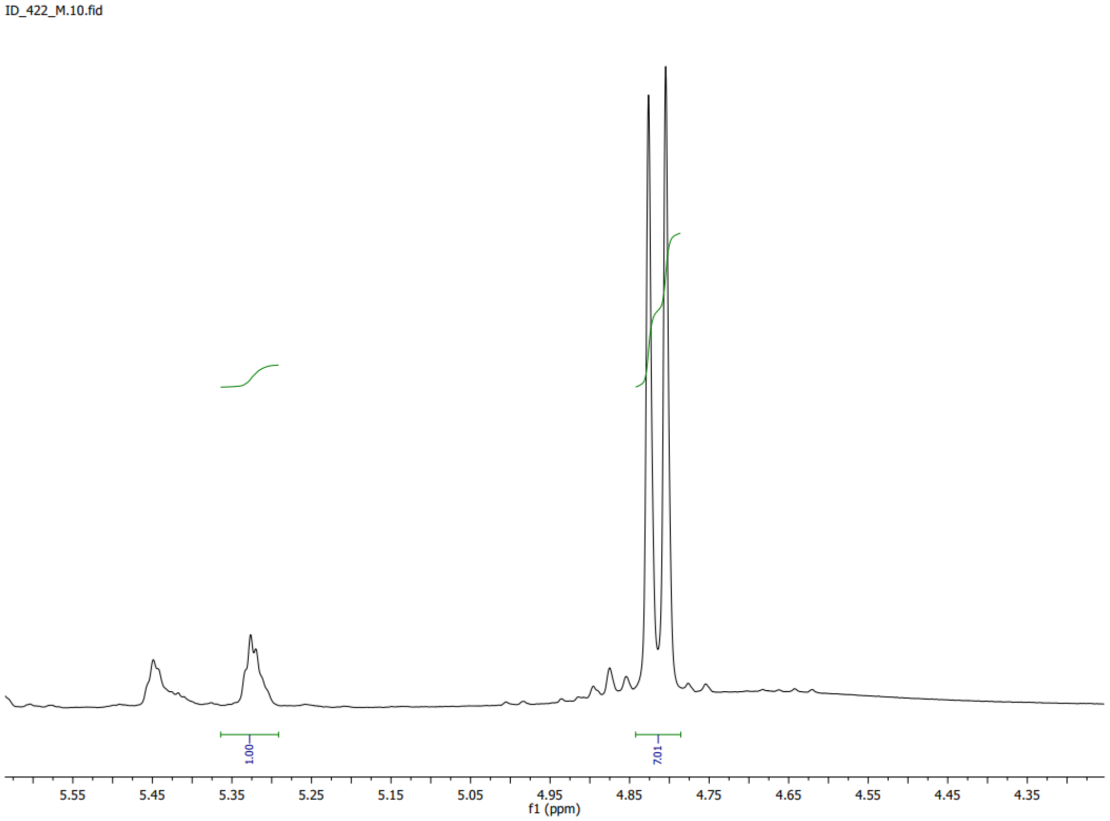

NMR traces for reaction with: **3-Me-phenylboronic acid (duplicate) (400 MHz,** $CDCl_3$**)**

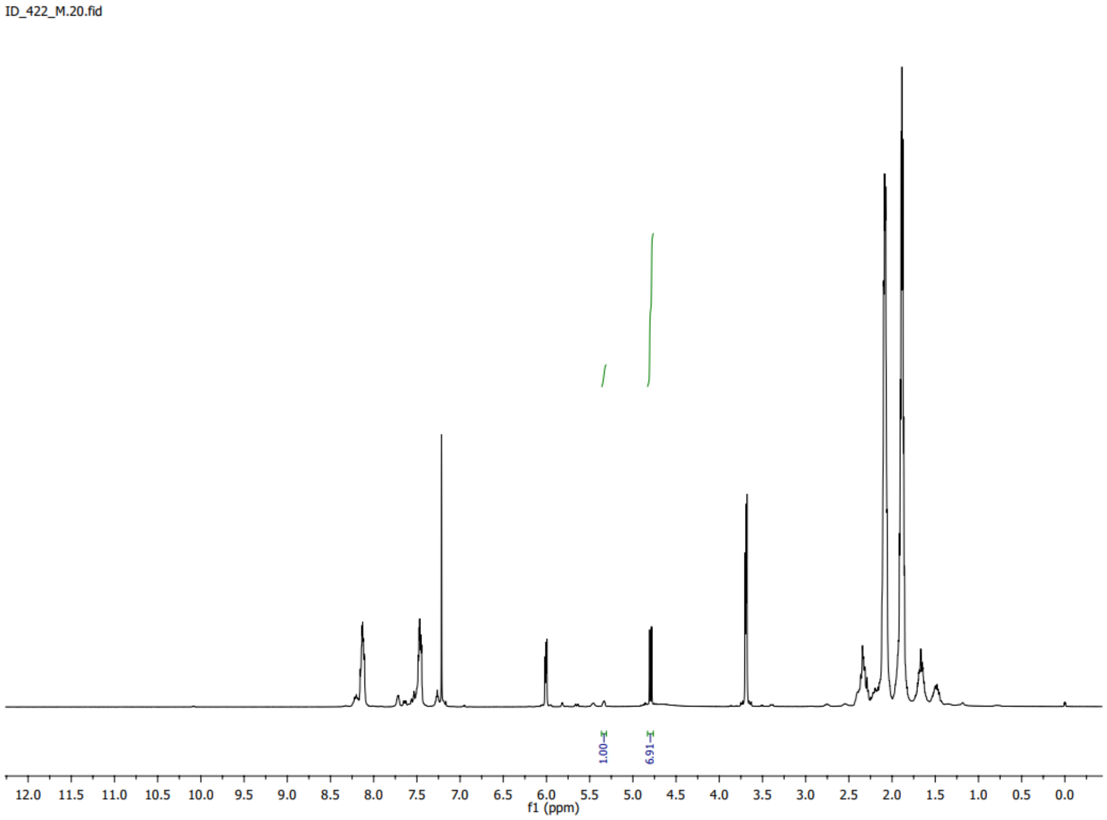

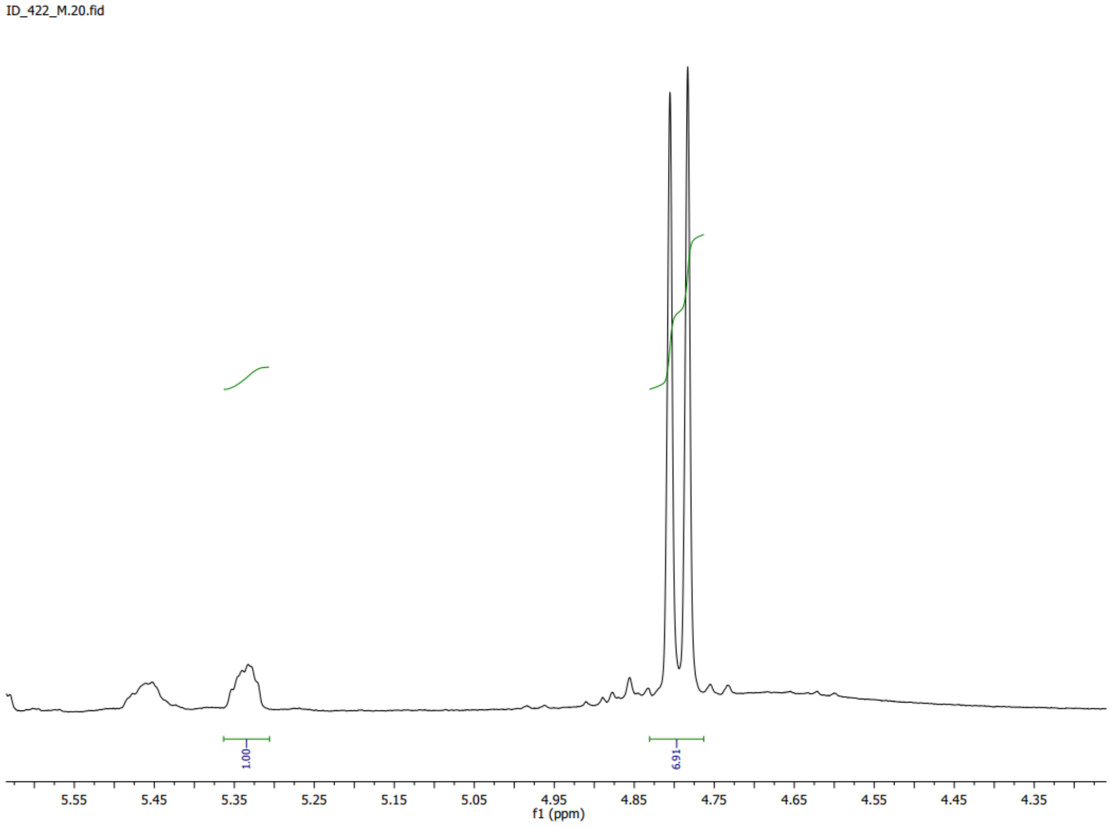

NMR traces for reaction with: **2-Me-phenylboronic acid (400 MHz,** CDCl$_3$**)**

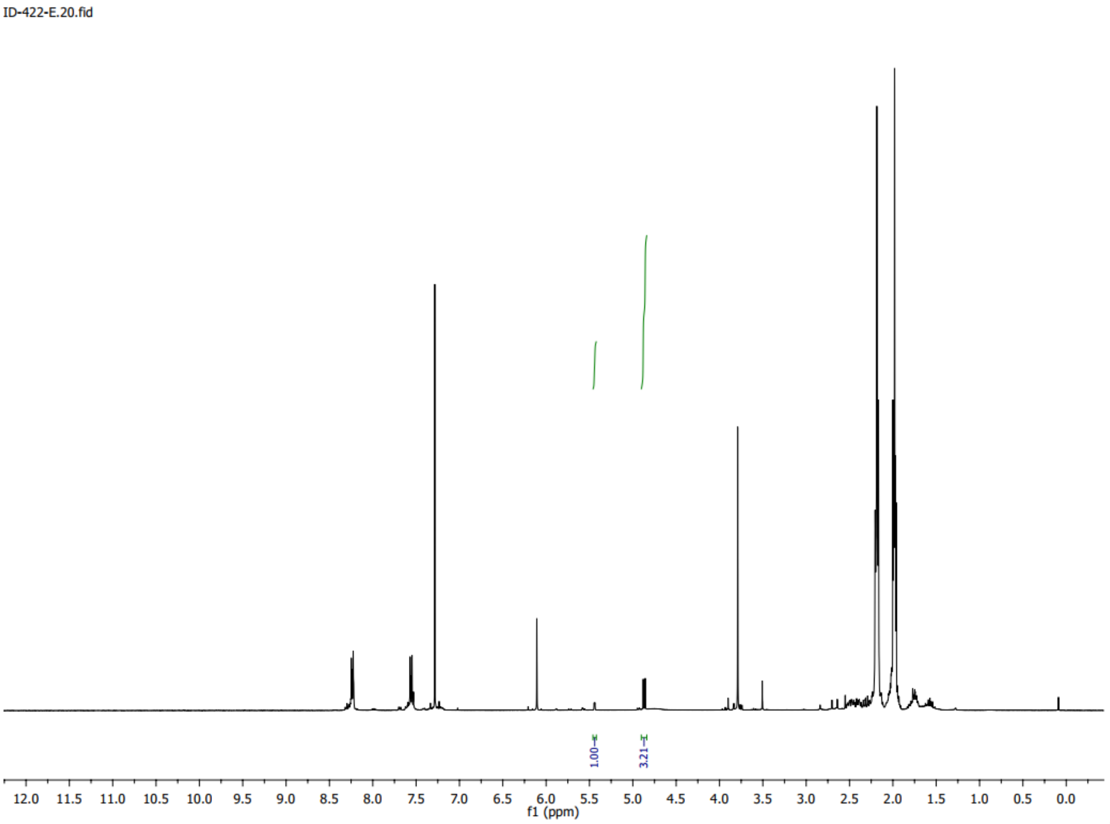

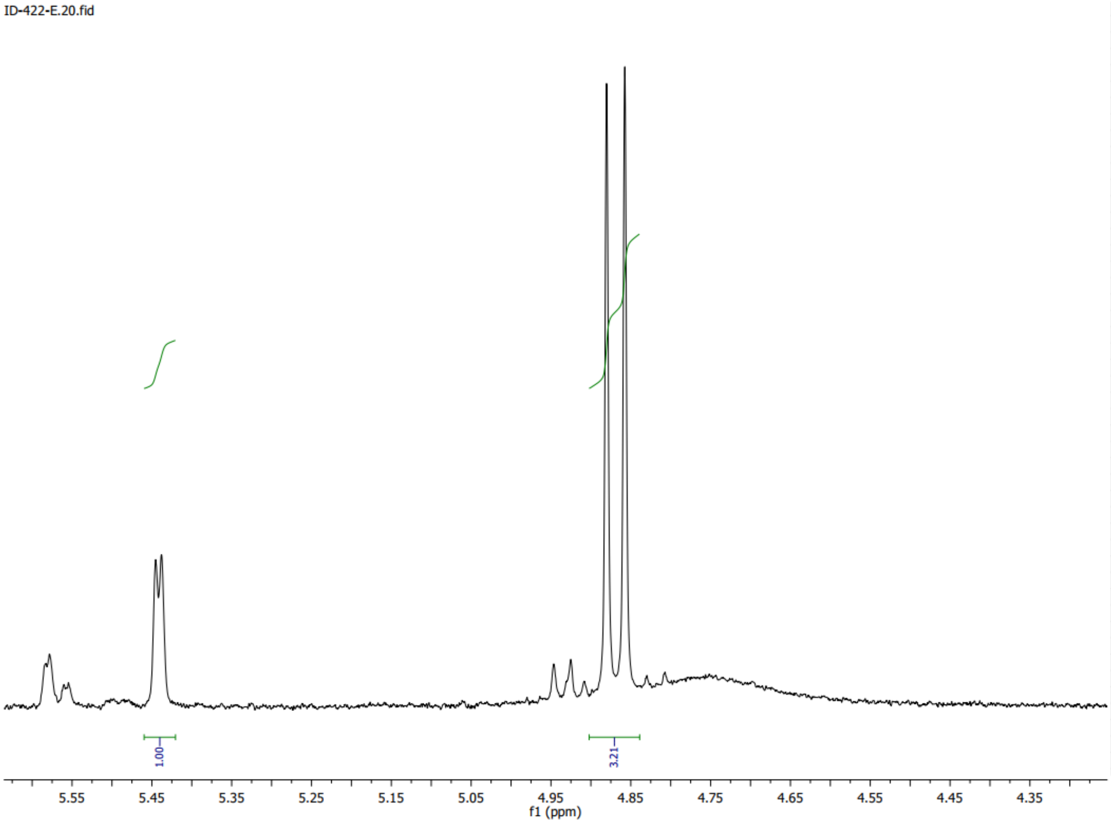

NMR traces for reaction with: **2-Me-phenylboronic acid (duplicate) (400 MHz,** CDCl$_3$**)**

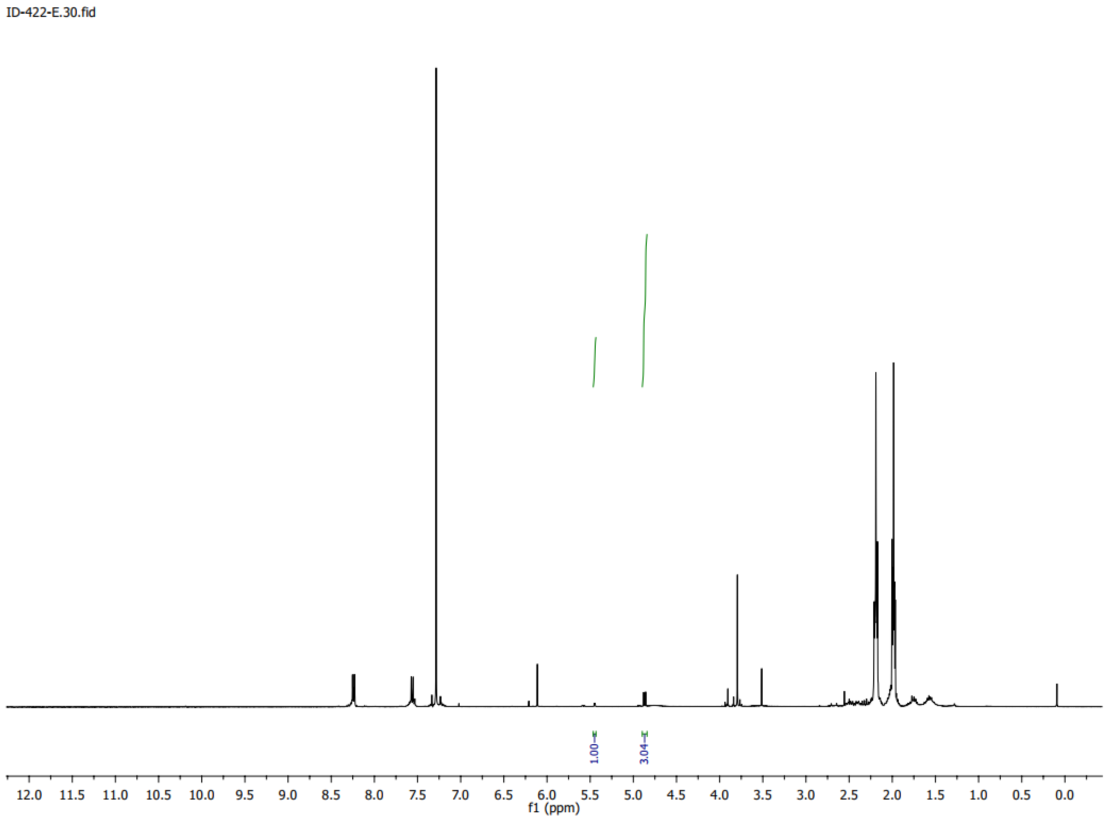

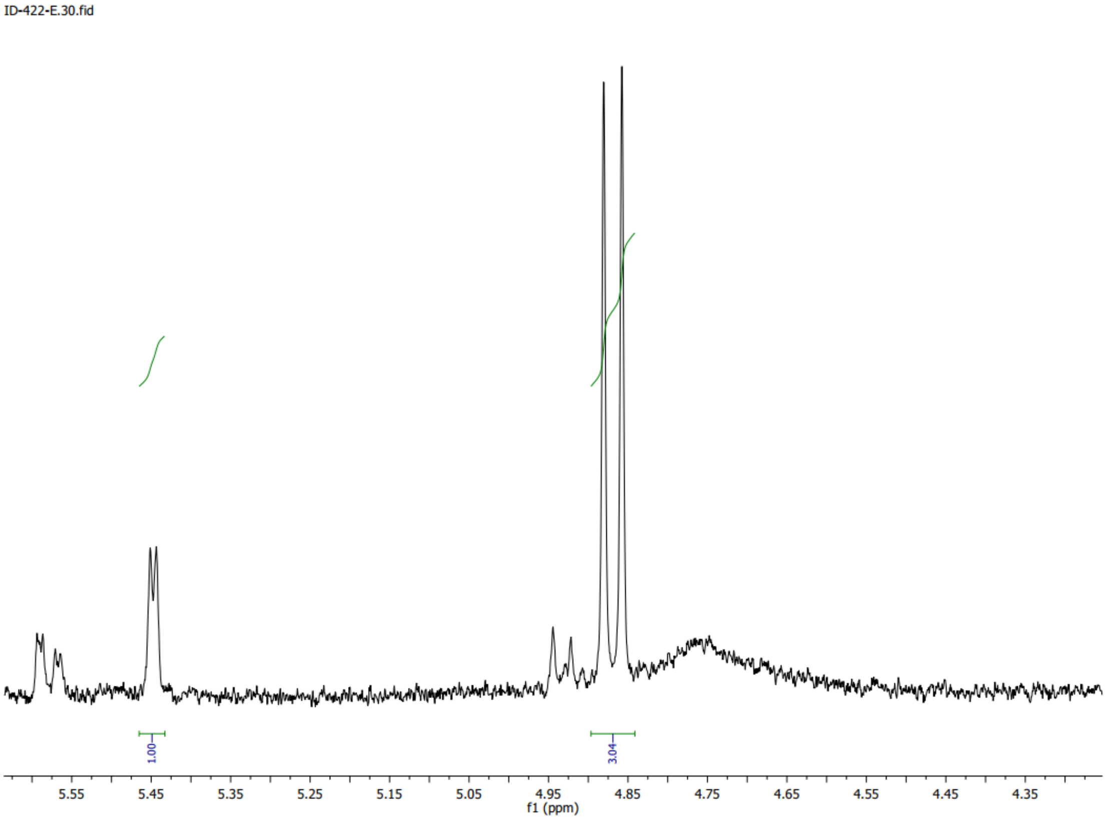

NMR traces for reaction with: **Ph-phenylboronic acid (400 MHz,** $CDCl_3$**)**

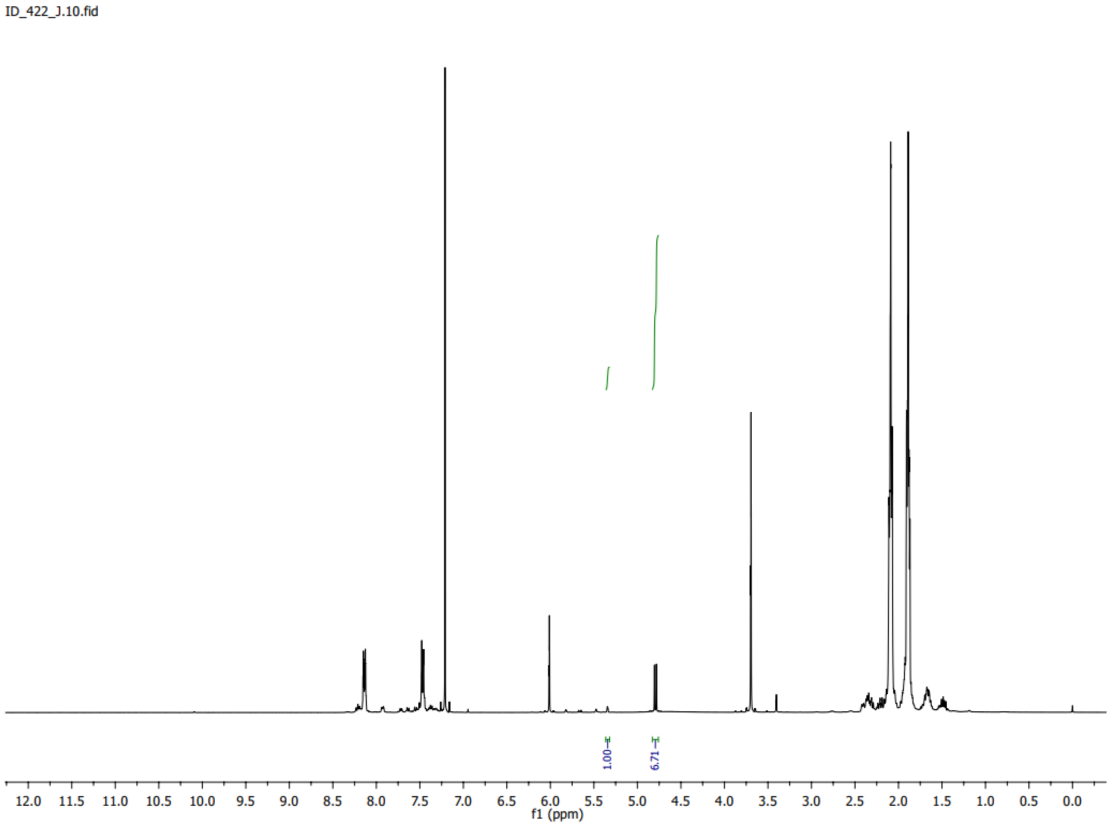

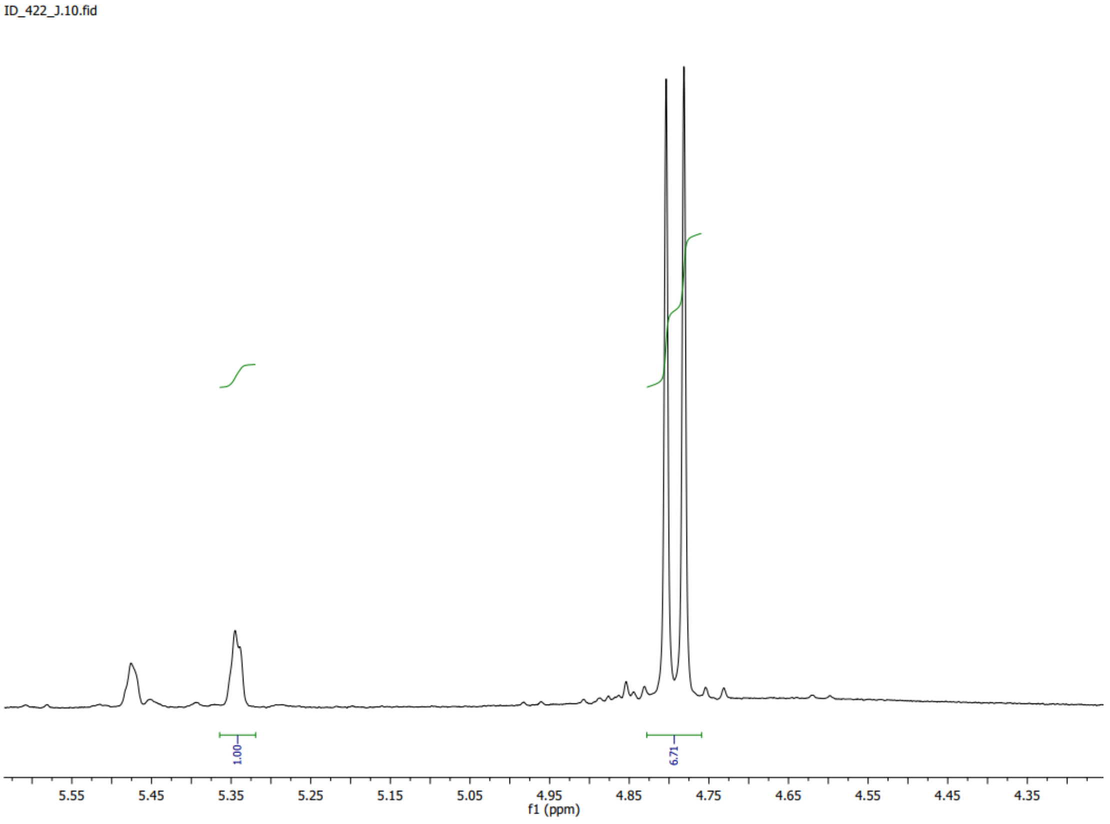

NMR traces for reaction with: **Ph-phenylboronic acid (duplicate) (400 MHz,** $CDCl_3$**)**

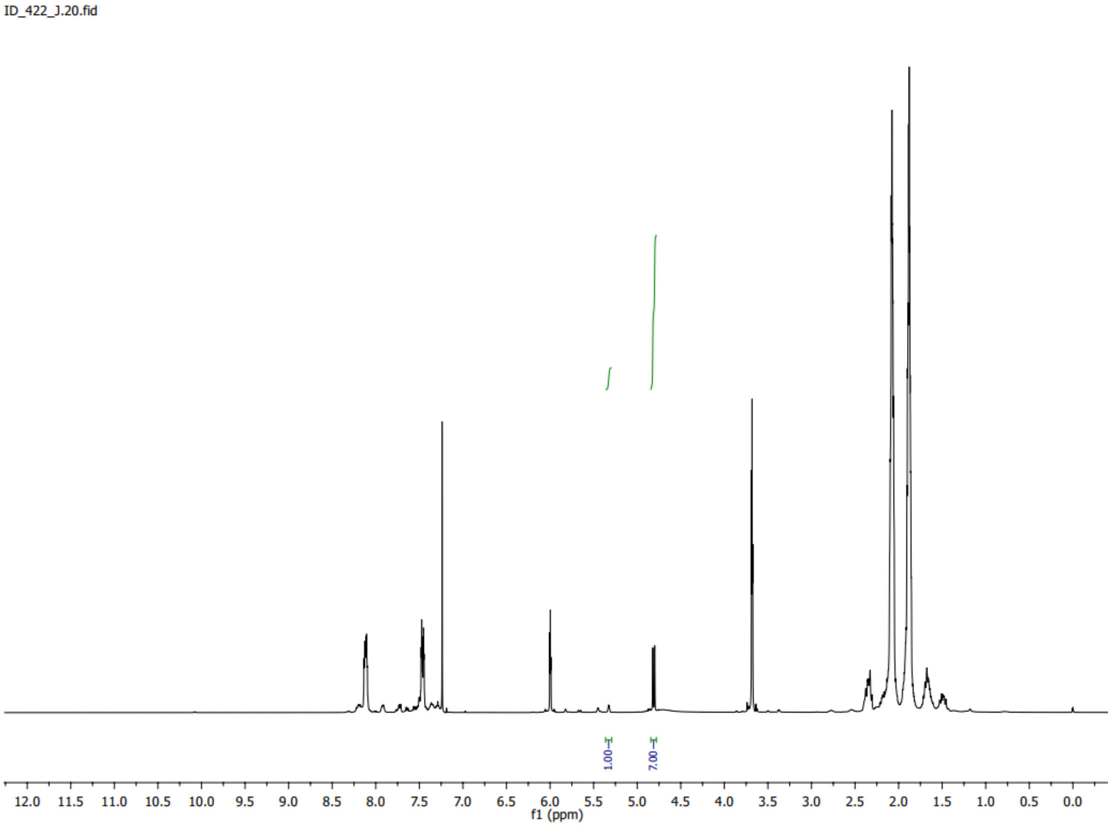

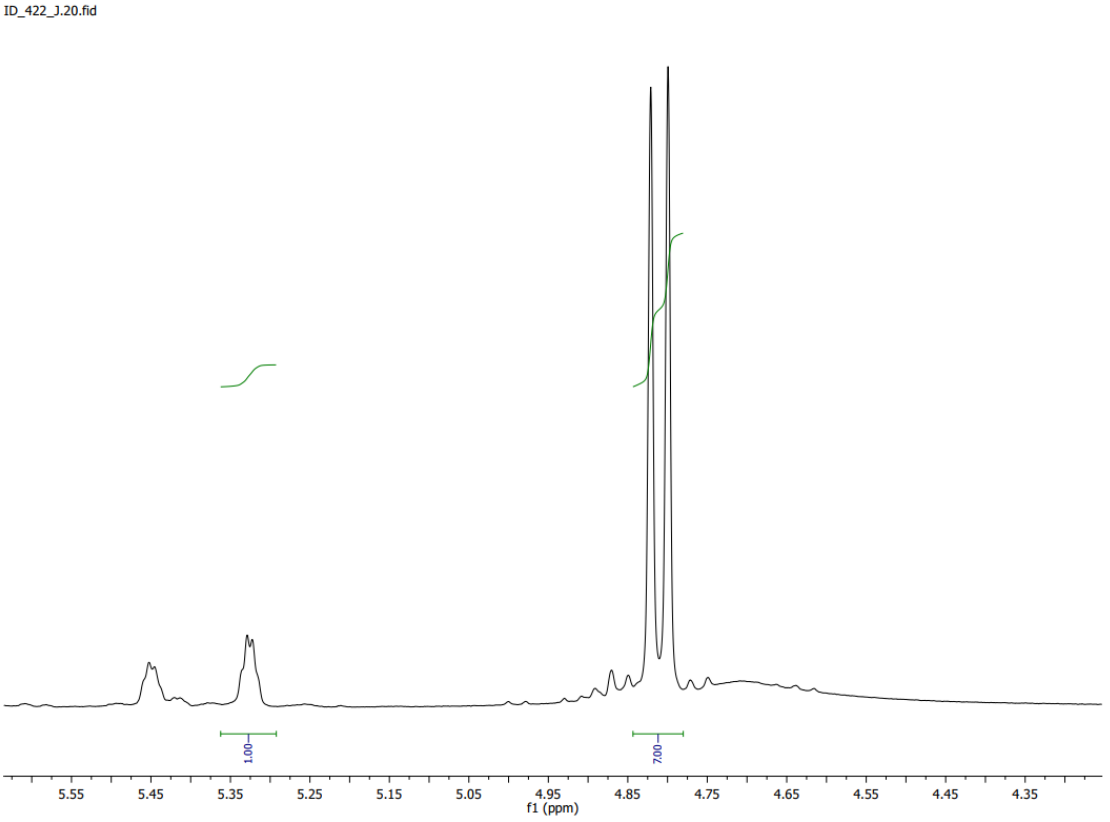

NMR traces for reaction with: **2-F-phenylboronic acid (400 MHz,** $CDCl_3$**)**

ID_422_I.10.fid

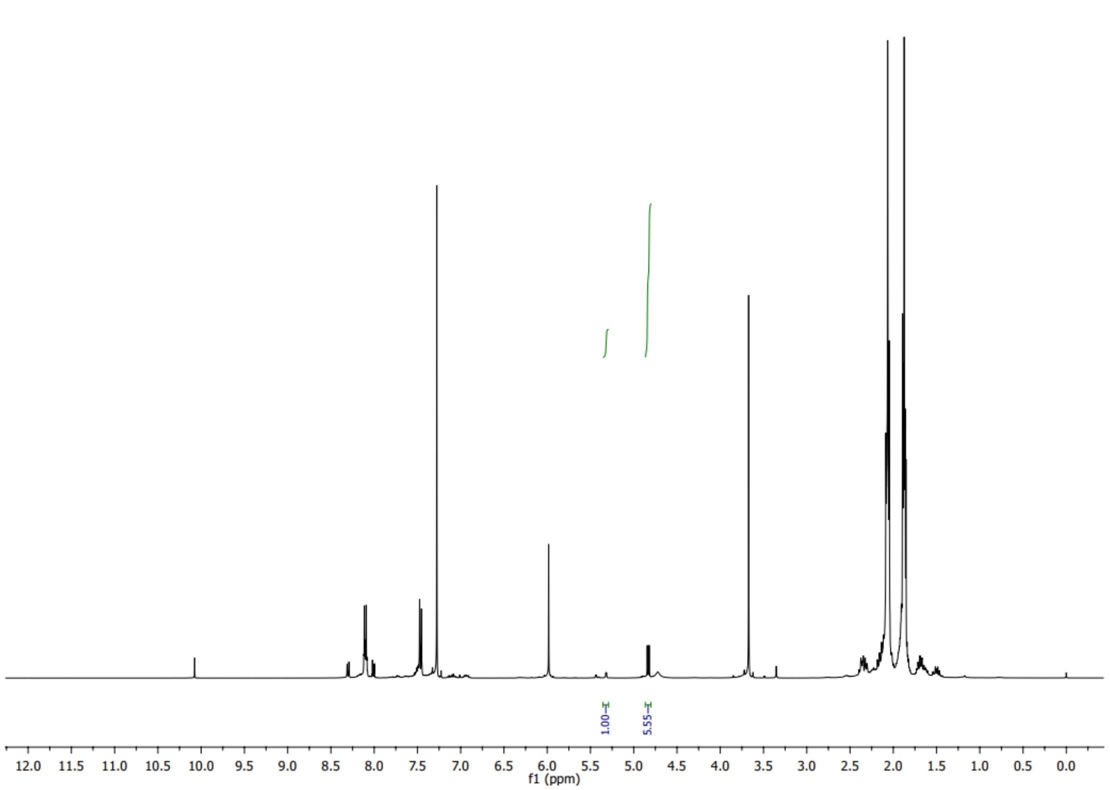

ID_422_I.10.fid

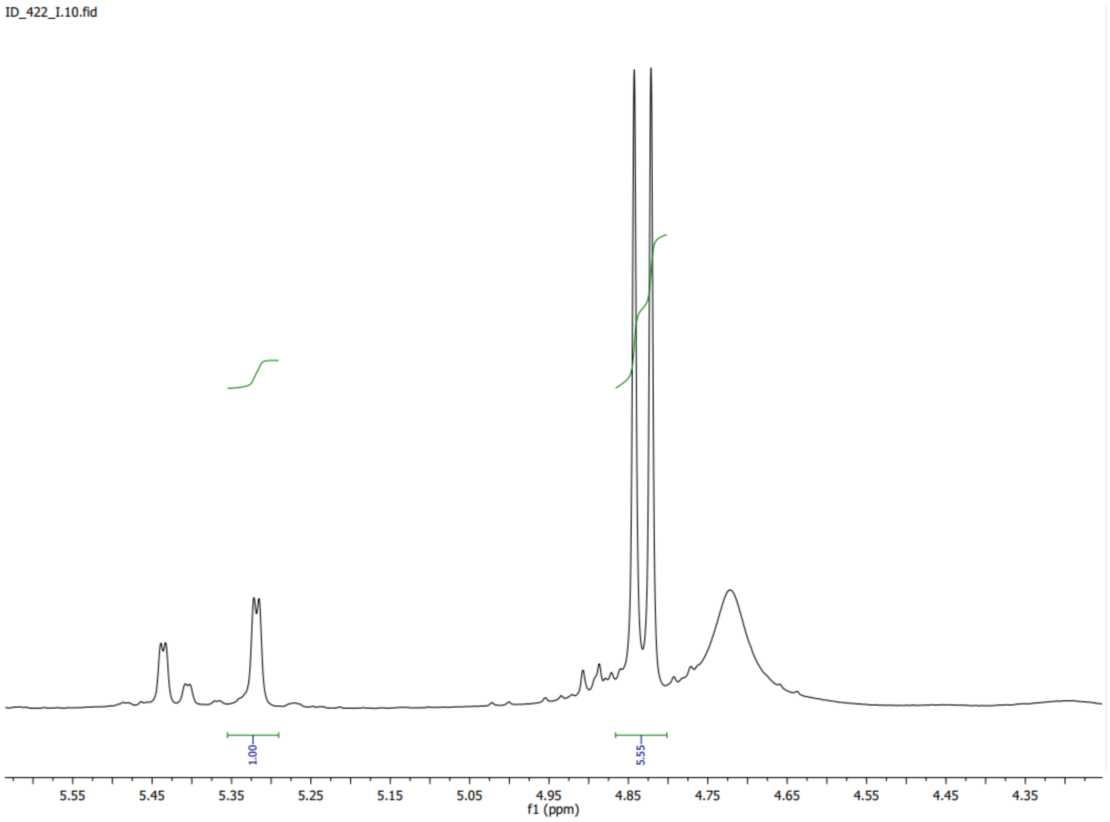

NMR traces for reaction with: **2-F-phenylboronic acid (duplicate) (400 MHz,** $CDCl_3$**)**

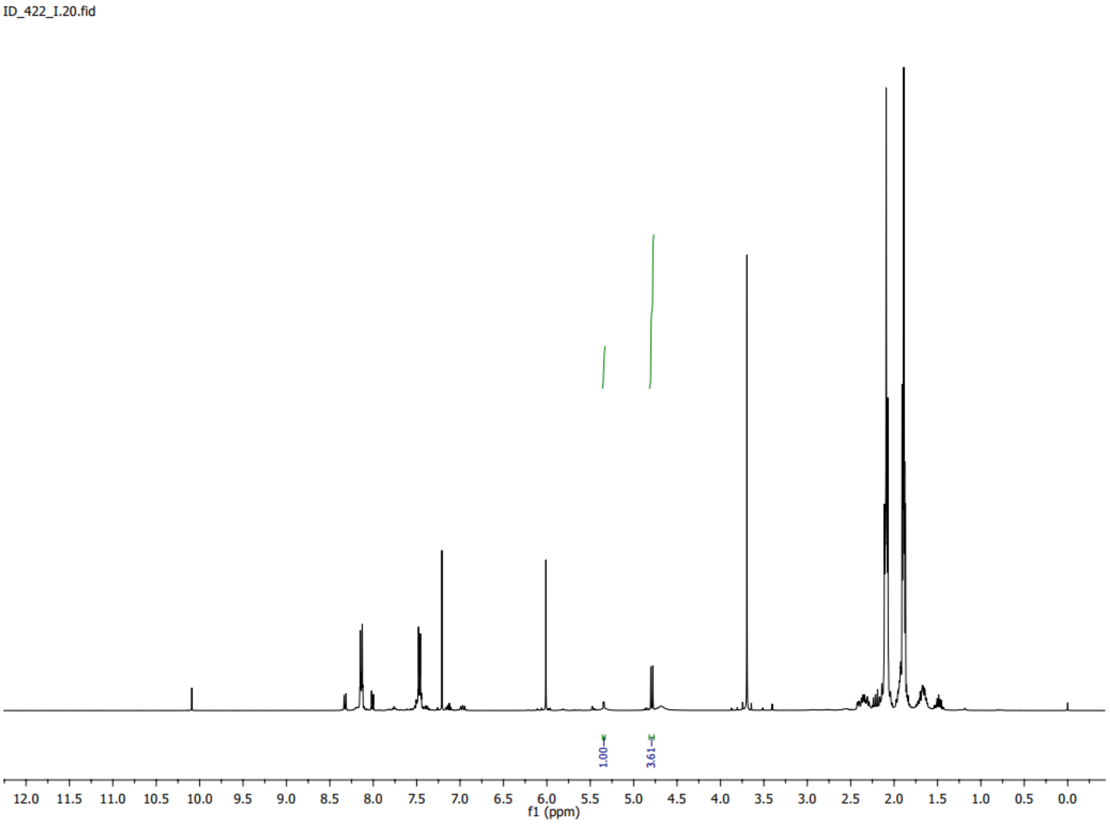

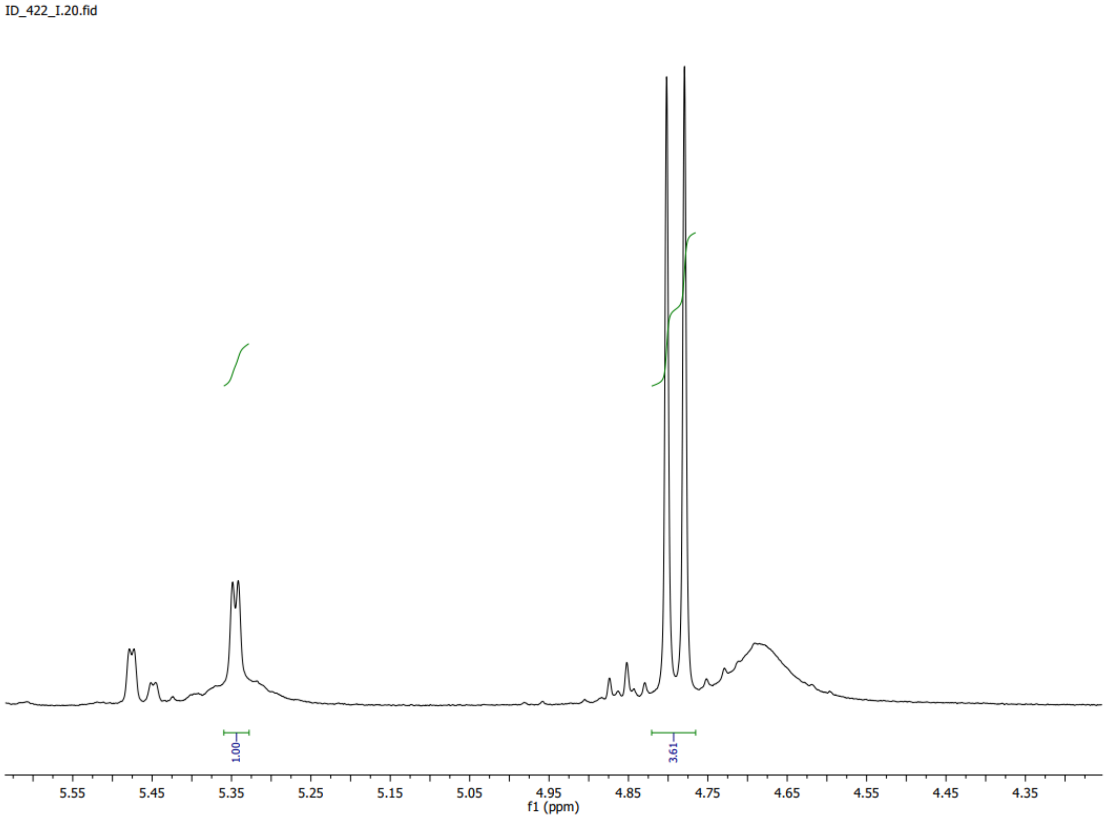

NMR traces for reaction **without boronic acid (400 MHz,** $CDCl_3$**):**

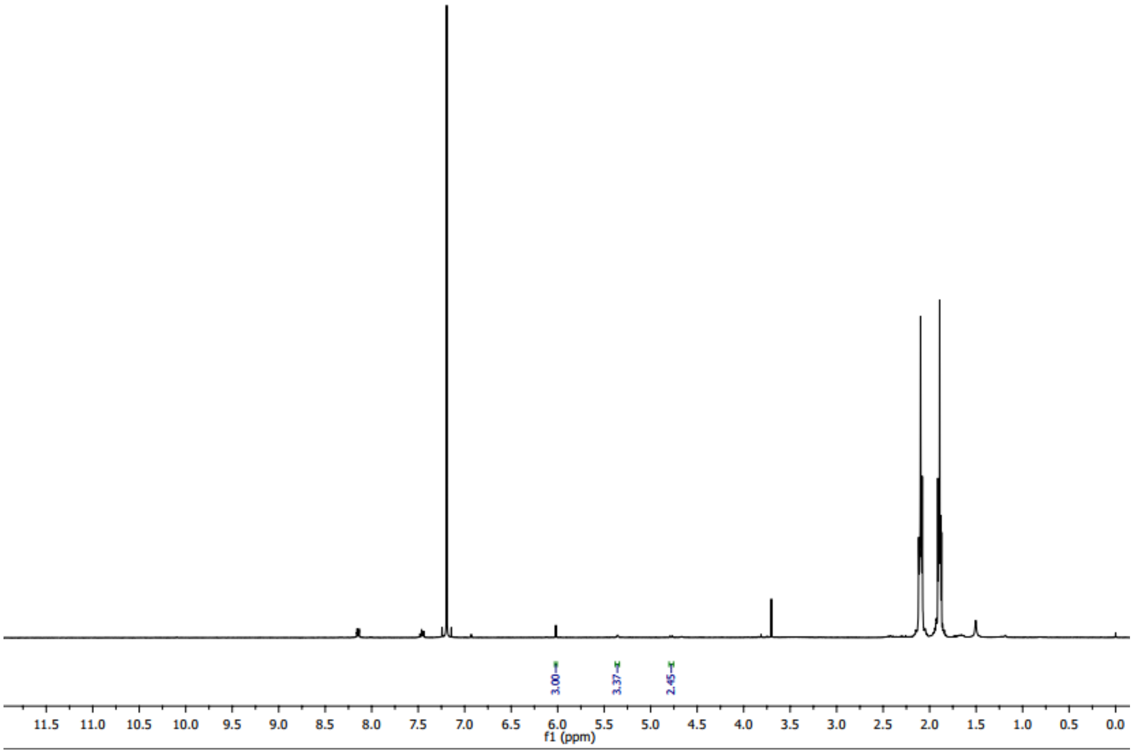

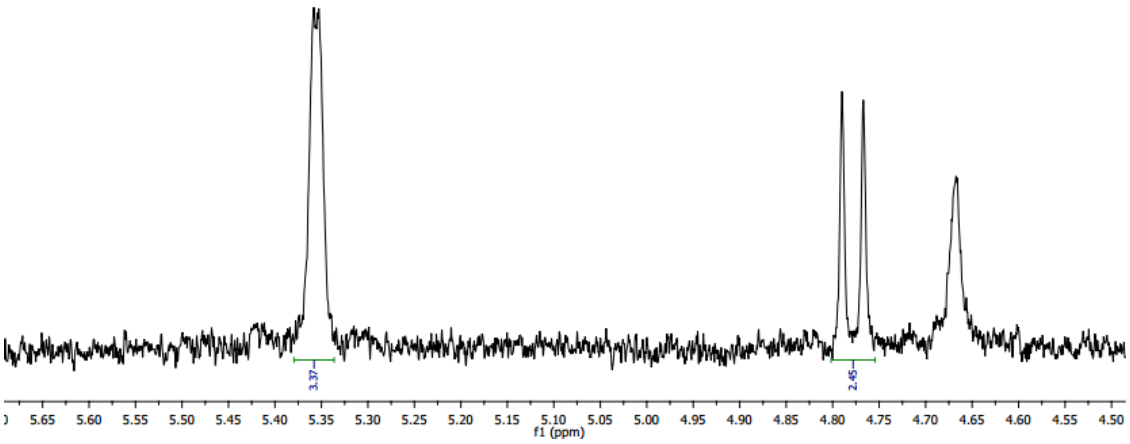

NMR traces for reaction **without boronic acid (duplicate) (400 MHz,** $CDCl_3$**)**

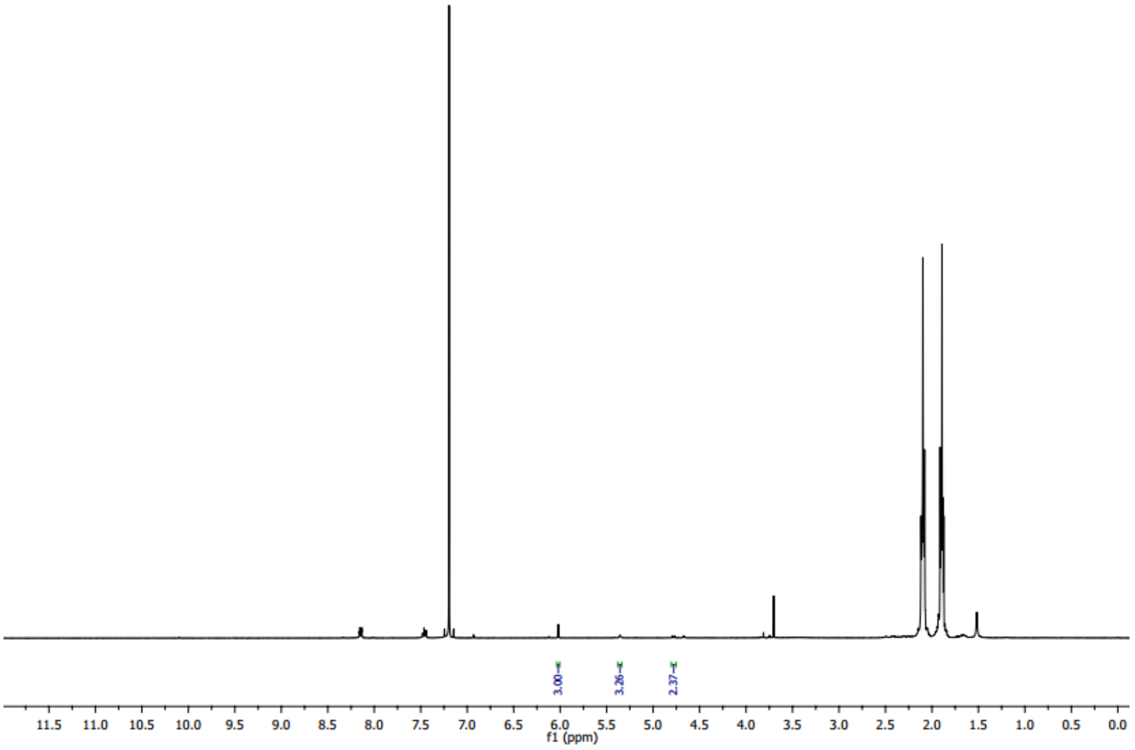

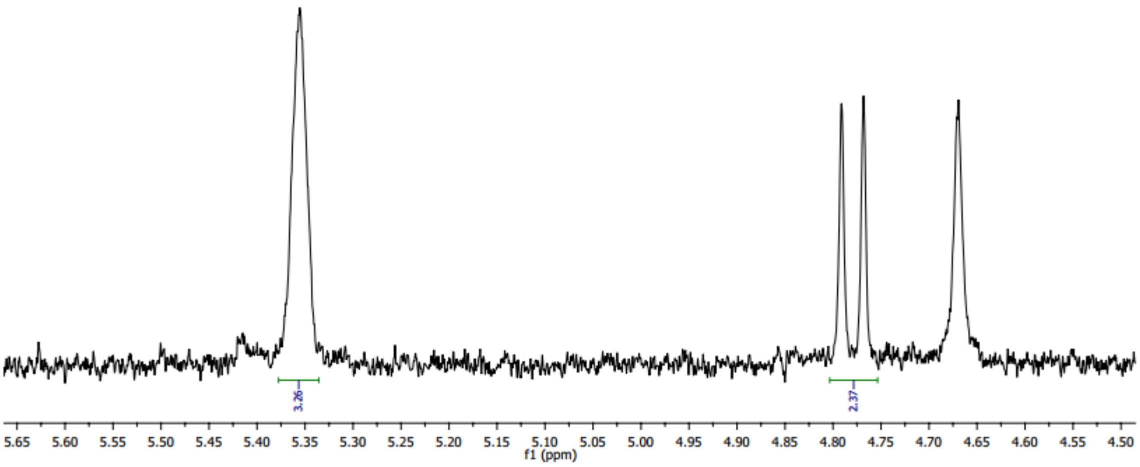

NMR traces for reaction **without water and with 2-F-phenylboronic acid (400 MHz,** $CDCl_3$**):**

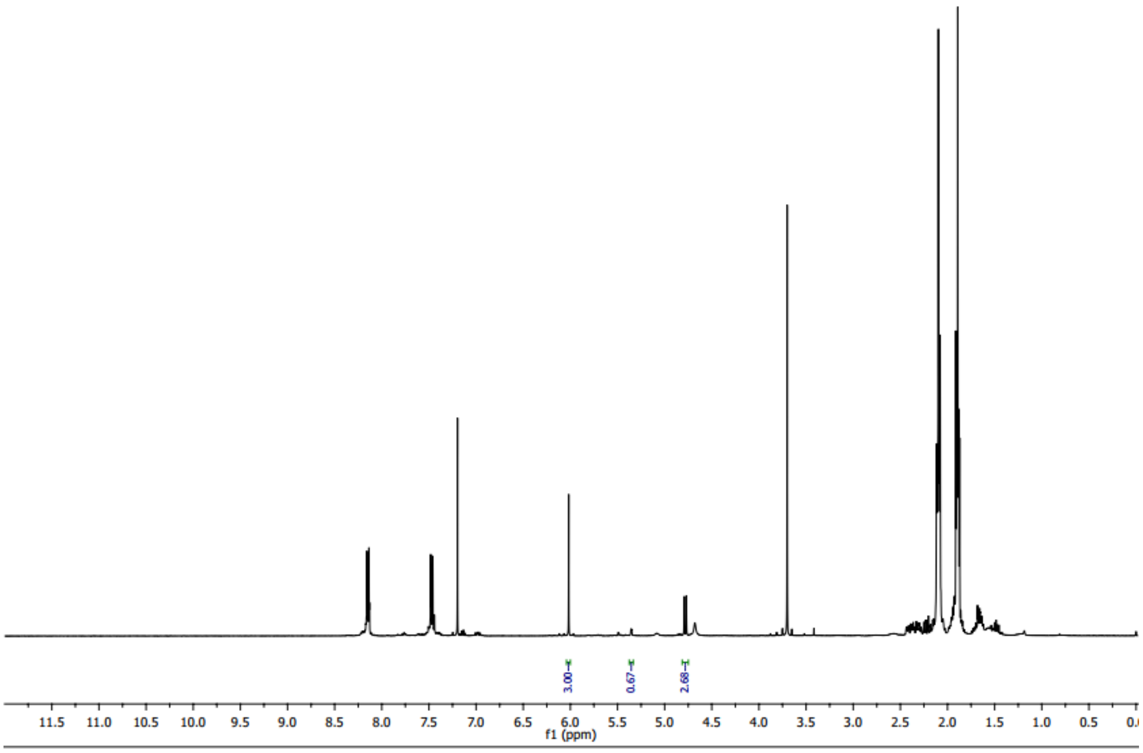

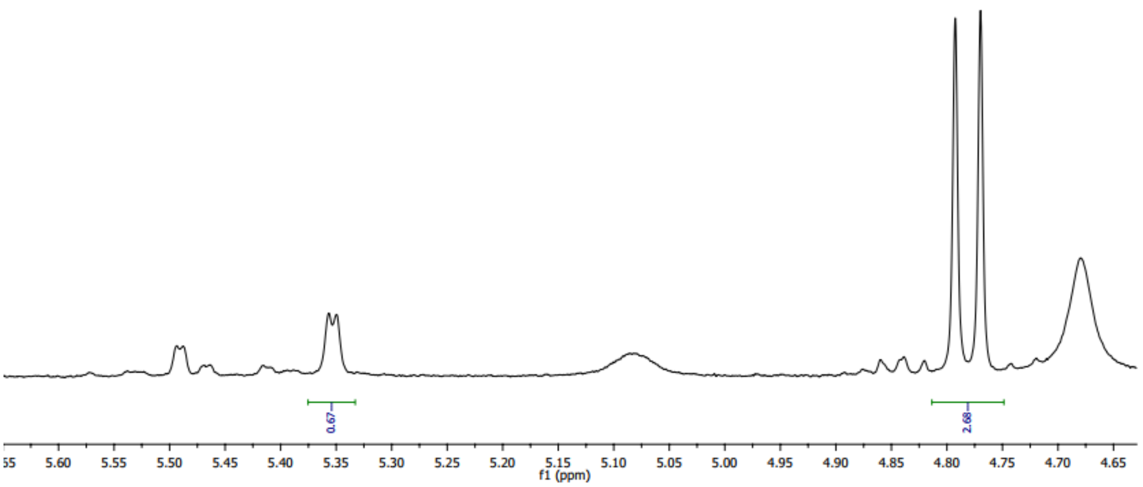

NMR traces for reaction **without water and with 2-F-phenylboronic acid (duplicate) (400 MHz, CDCl$_3$):**

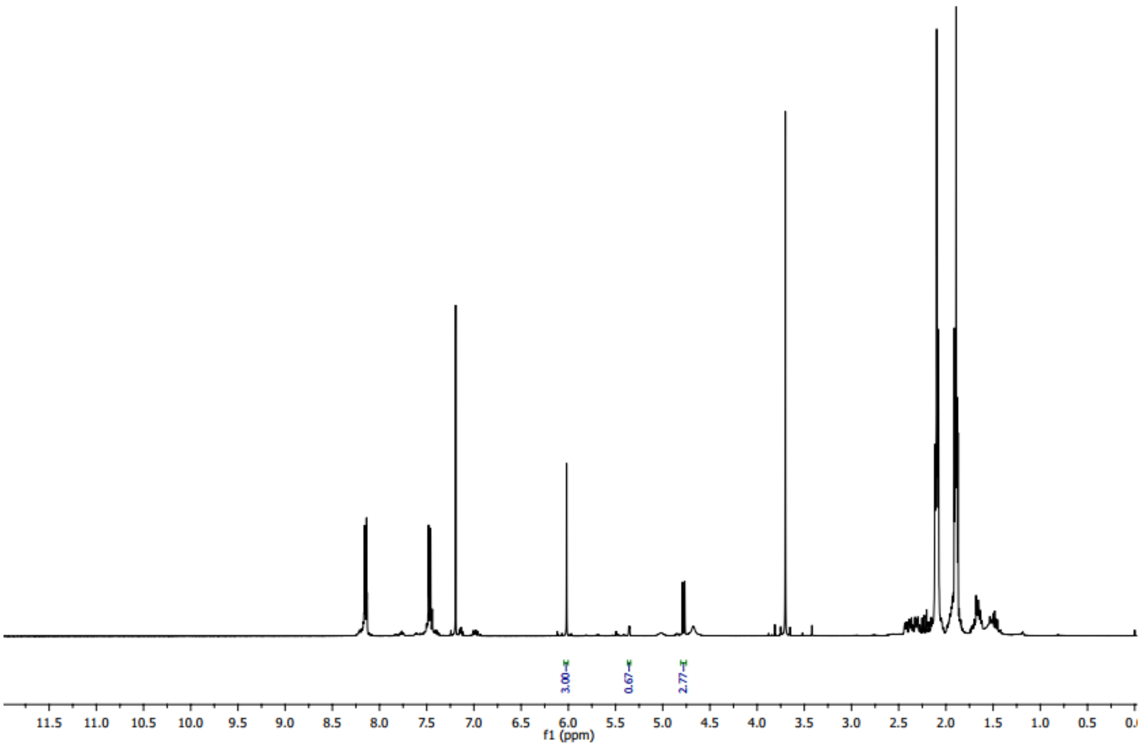

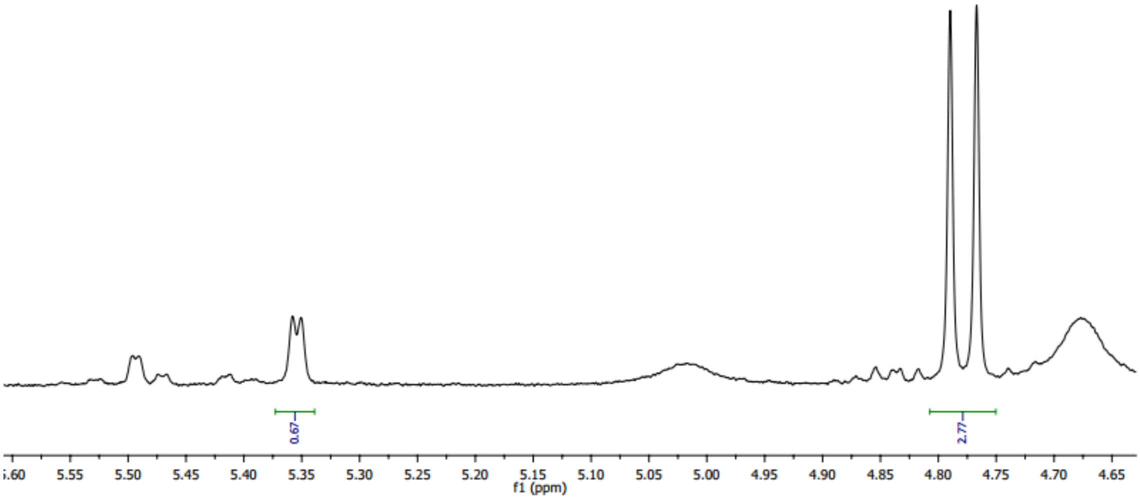

18.5. NMR Traces for Table S5. Boronic acid screening and blanks in hexane

NMR traces for reaction with: **3-F-phenylboronic acid (400 MHz,** $CDCl_3$**)**

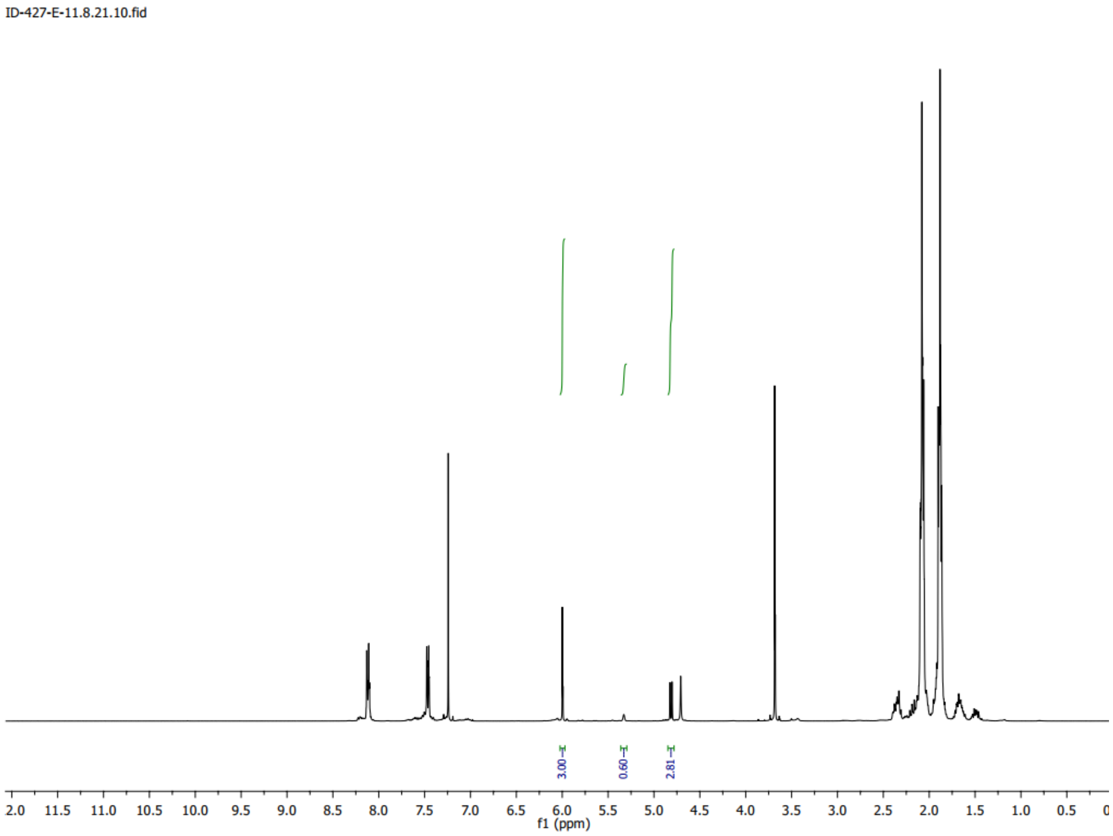

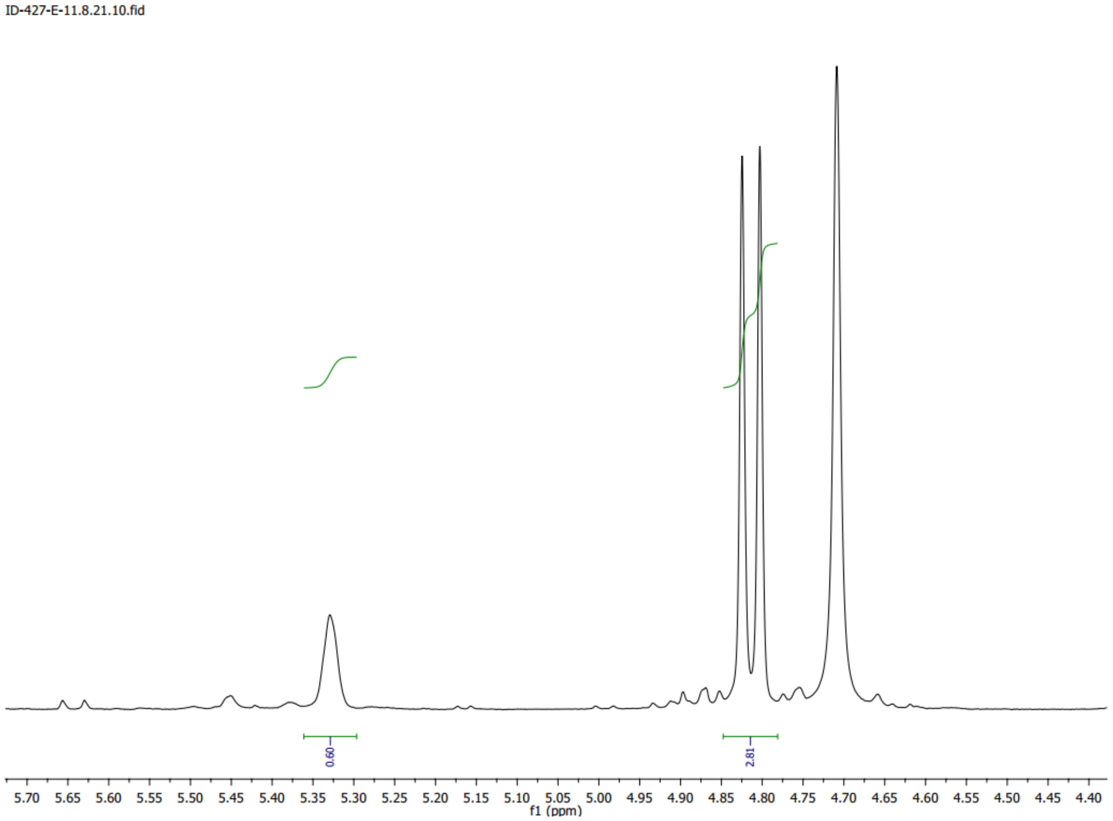

NMR traces for reaction with: **3-F-phenylboronic acid (duplicate) (400 MHz,** $CDCl_3$**)**

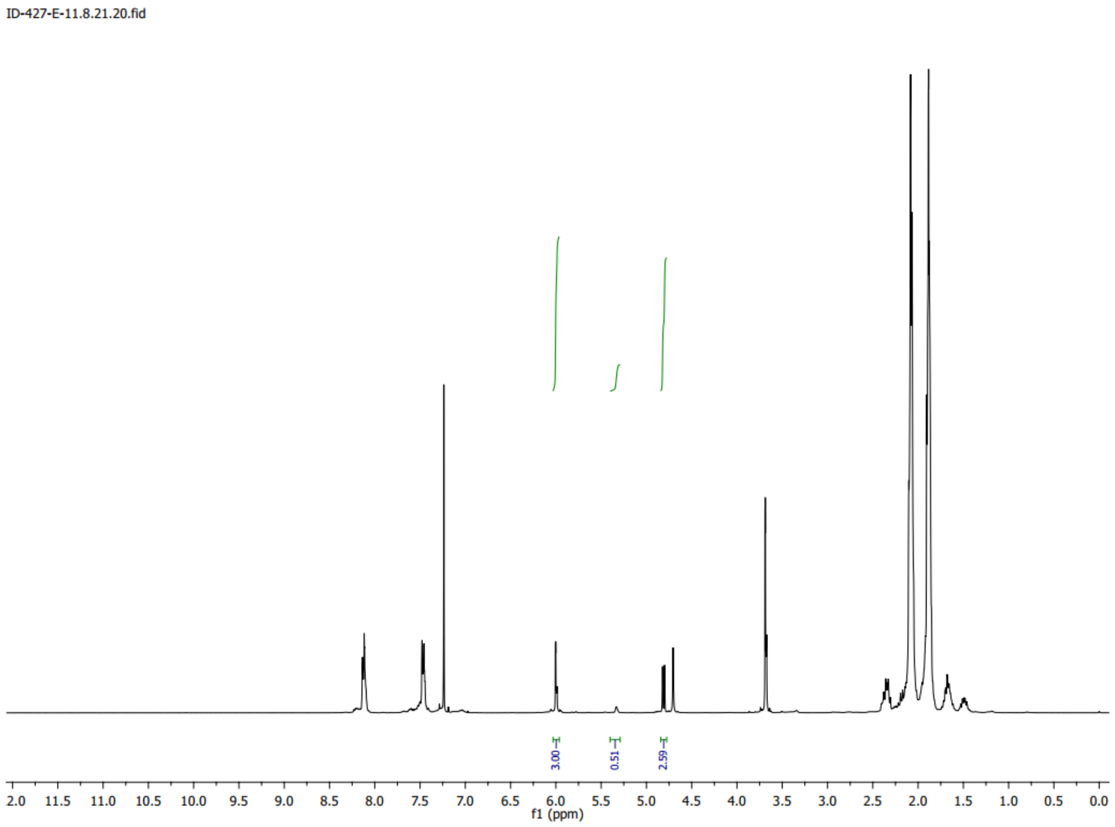

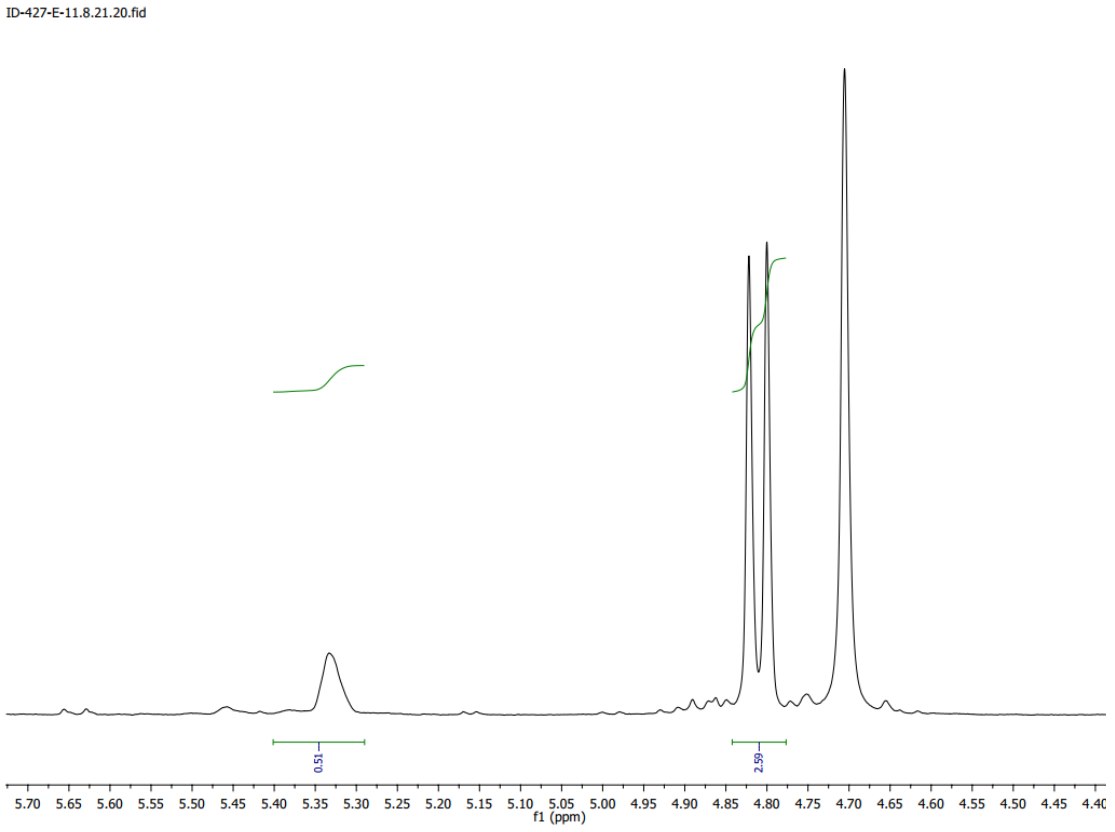

NMR traces for reaction with: **3,5-F-phenylboronic acid (400 MHz,** CDCl$_3$**)**

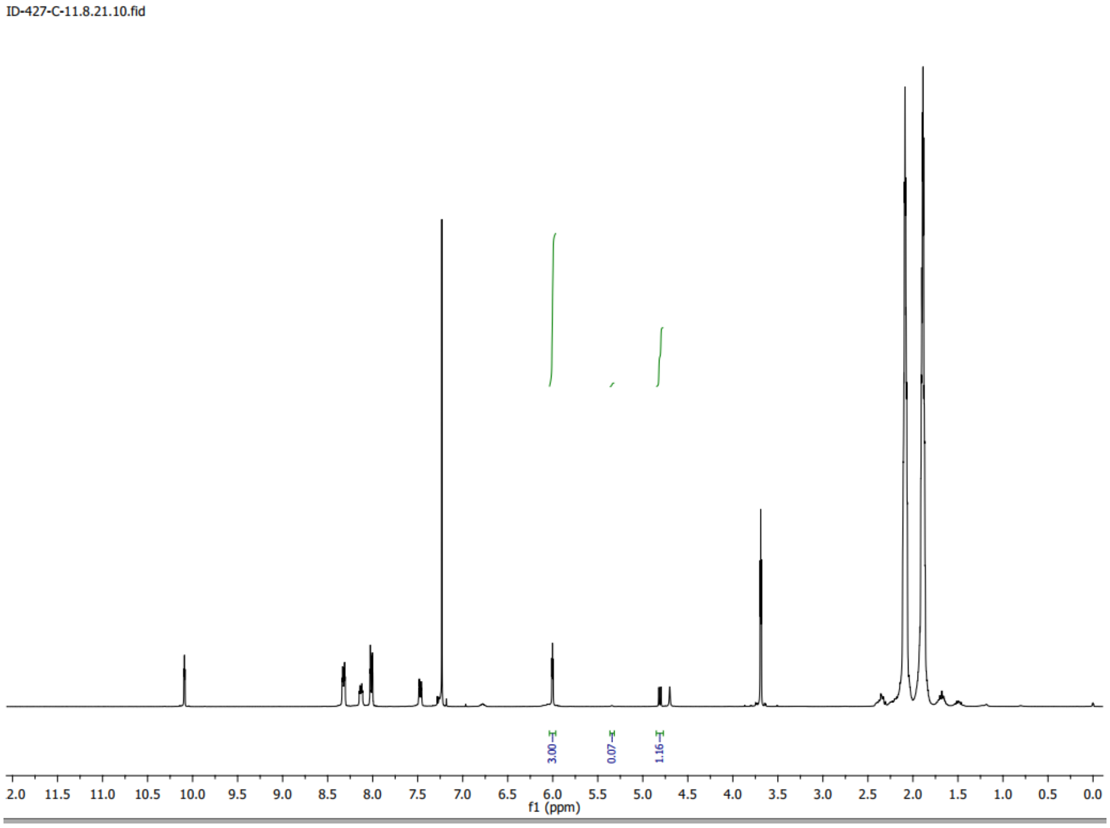

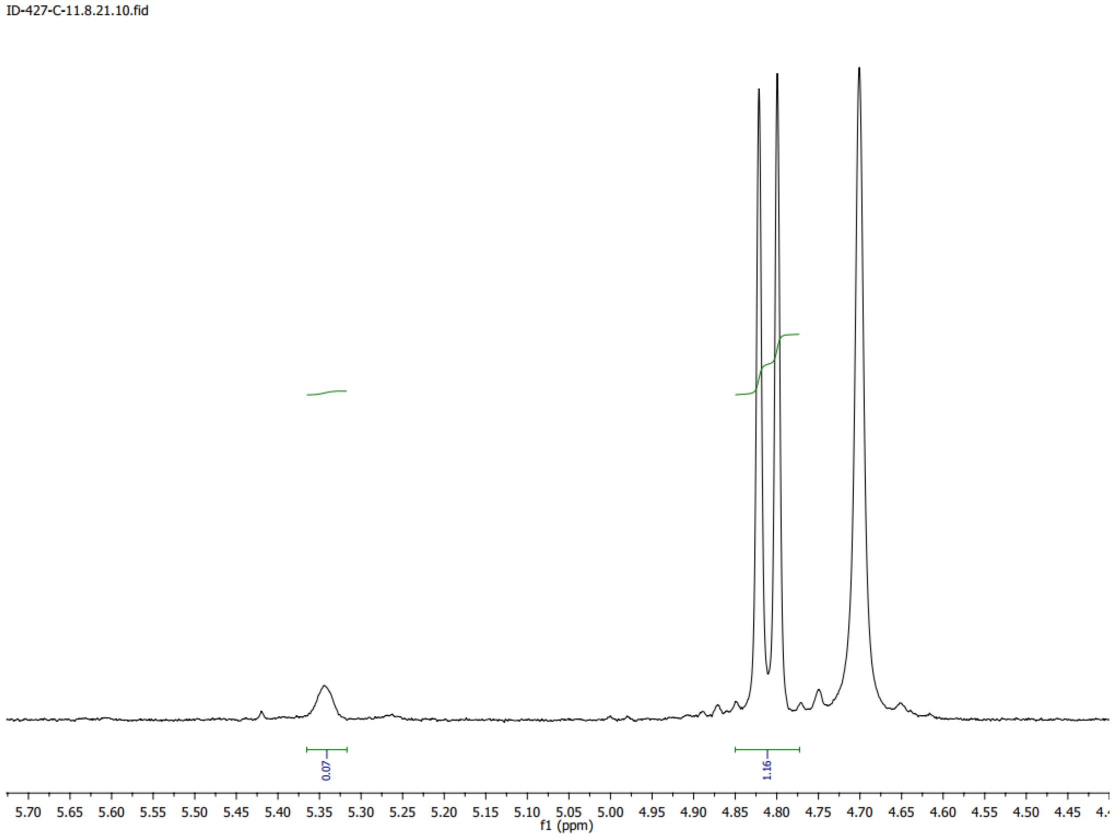

NMR traces for reaction with: **3,5-F-phenylboronic acid (duplicate) (400 MHz,** CDCl3**)**

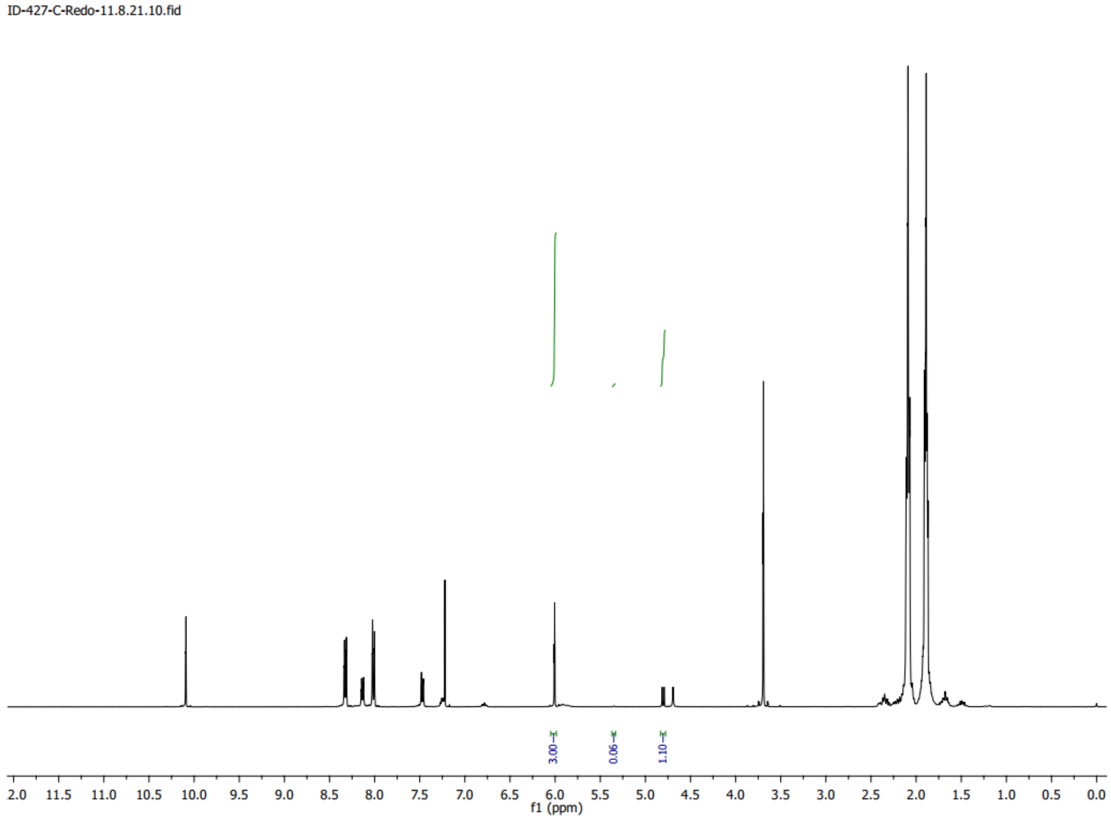

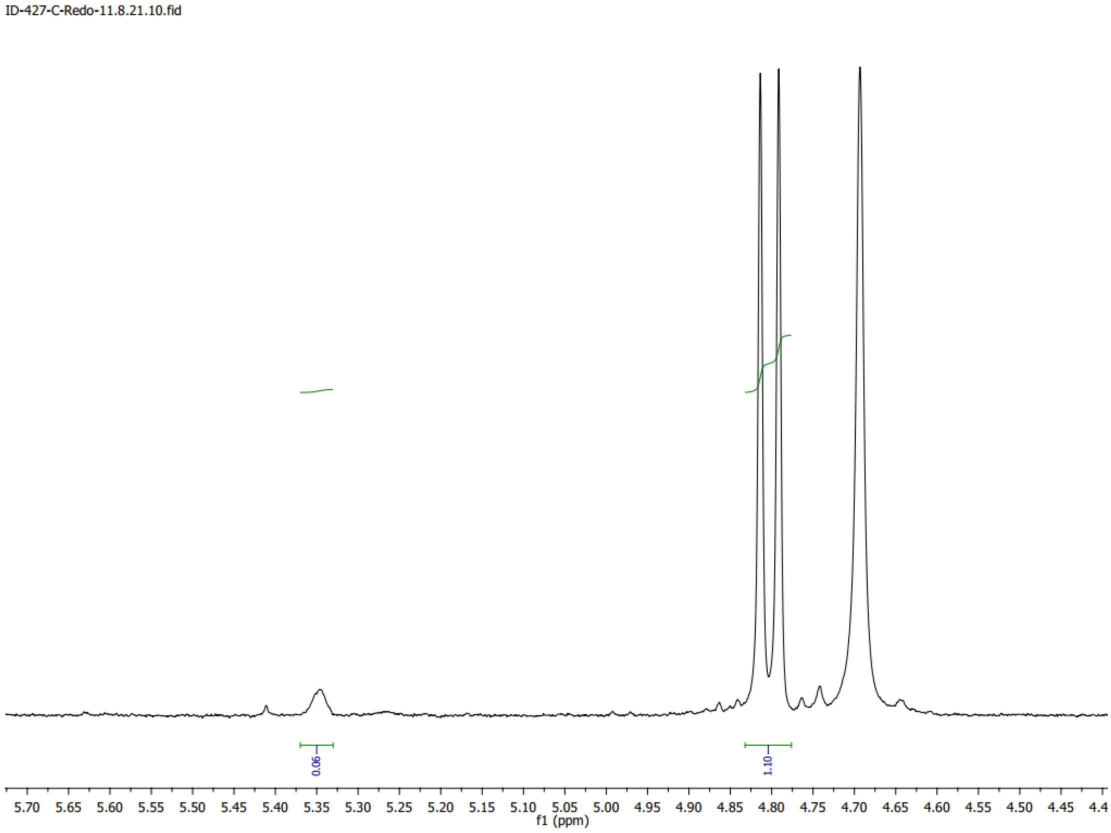

NMR traces for reaction with: **4-tBu-phenylboronic acid (400 MHz,** CDCl$_3$**)**

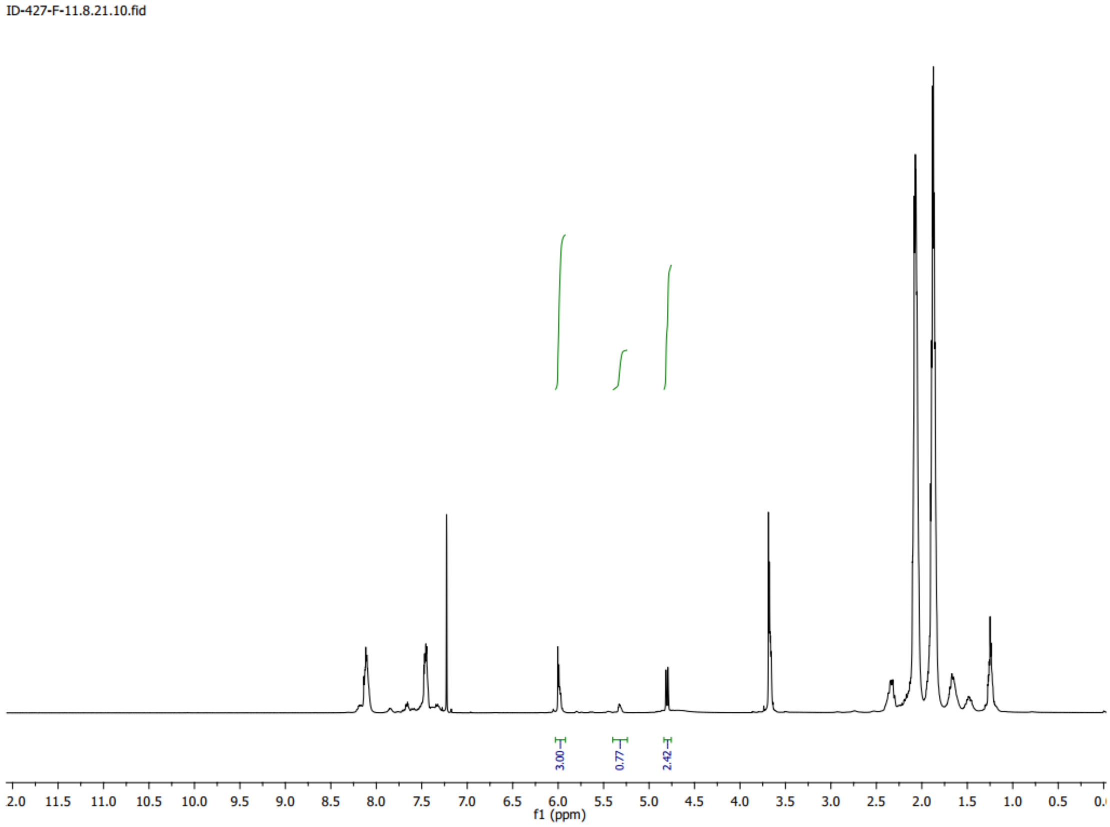

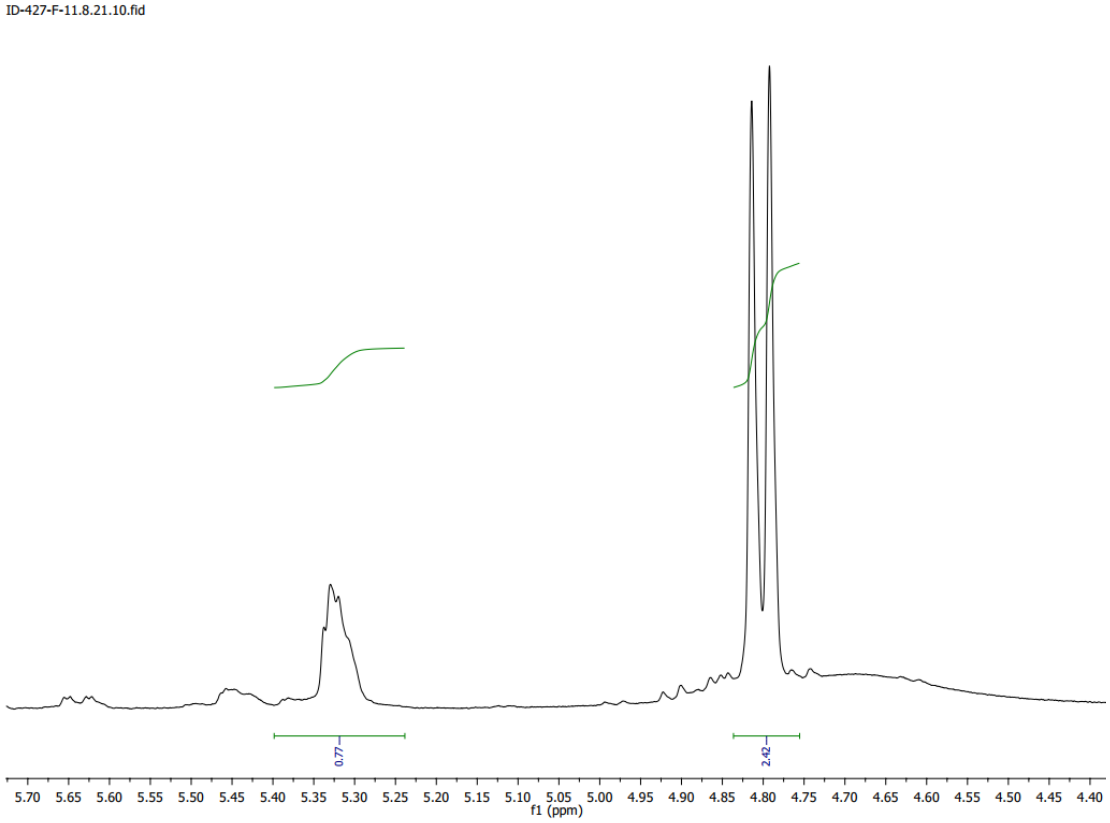

NMR traces for reaction with: **4-tBu-phenylboronic acid (duplicate) (400 MHz,** $CDCl_3$**)**

ID-427-F-11.8.21.20.fid

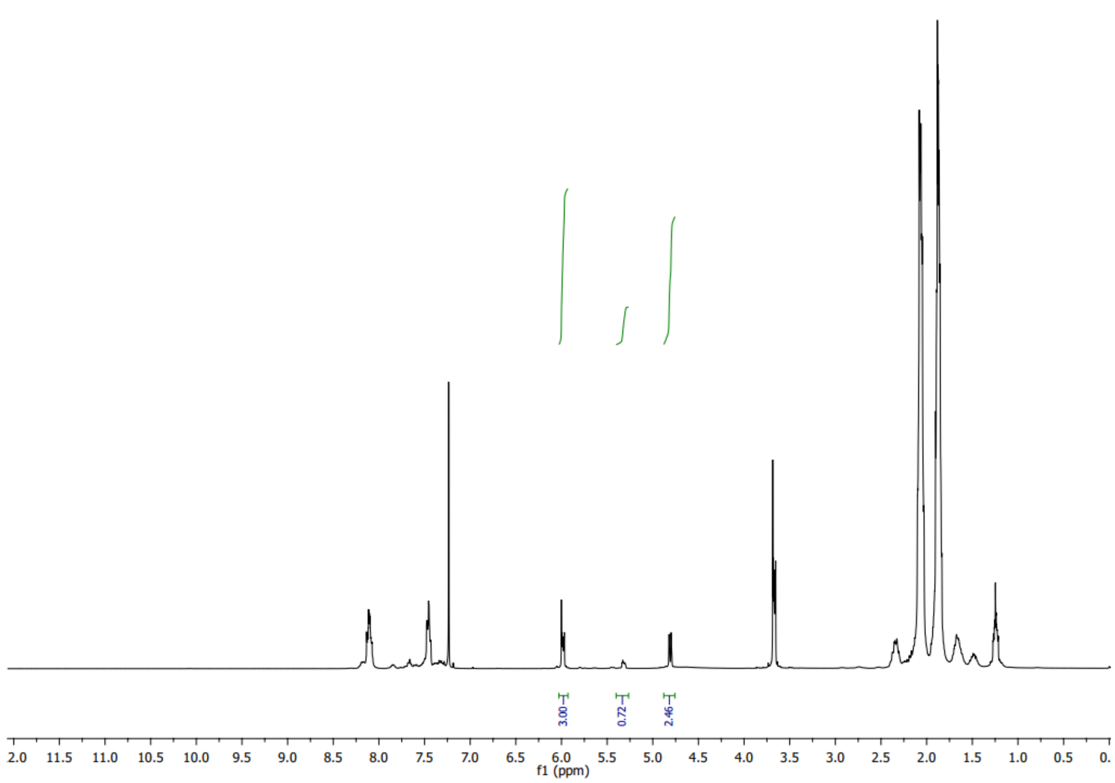

ID-427-F-11.8.21.20.fid

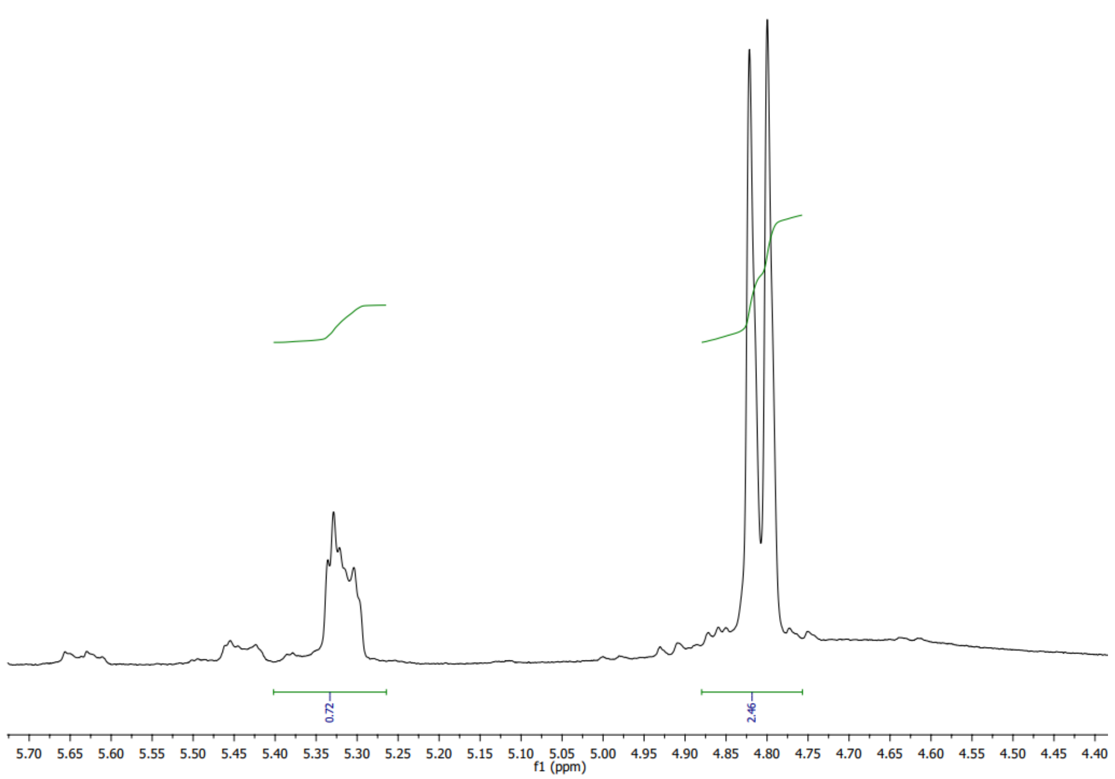

NMR traces for reaction with: **2,4-Me-phenylboronic acid (400 MHz,** CDCl$_3$**)**

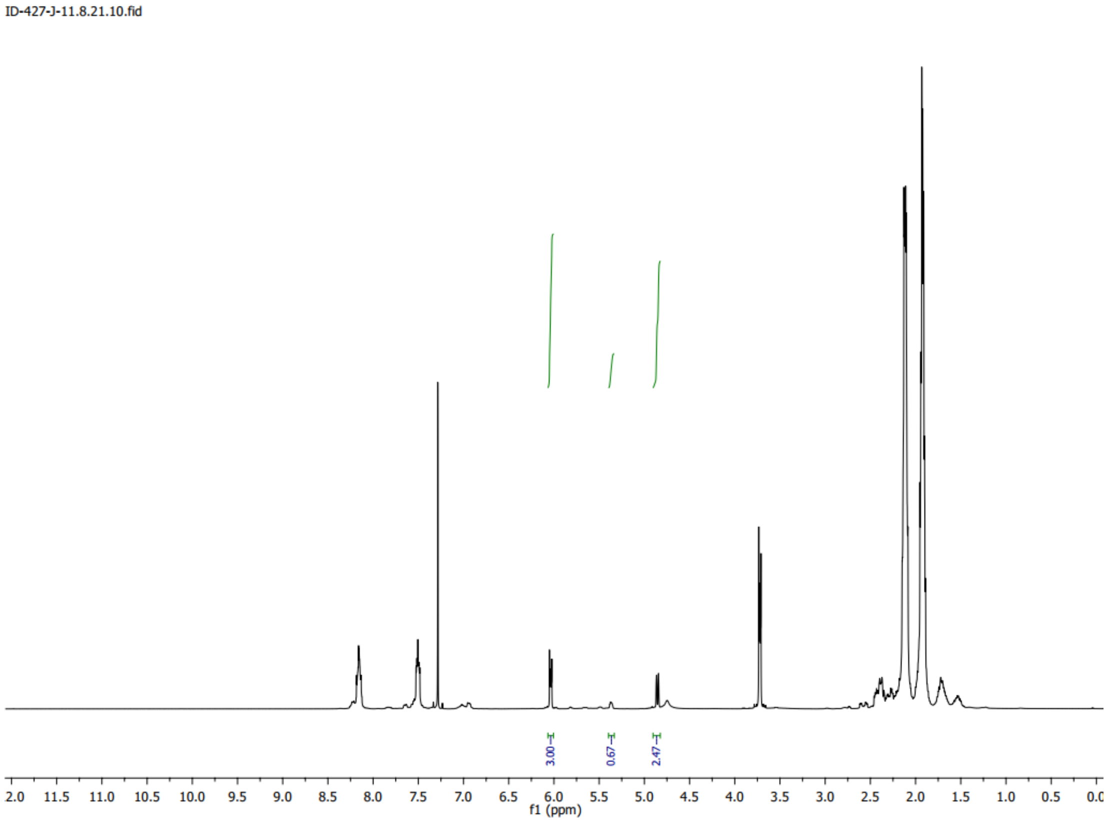

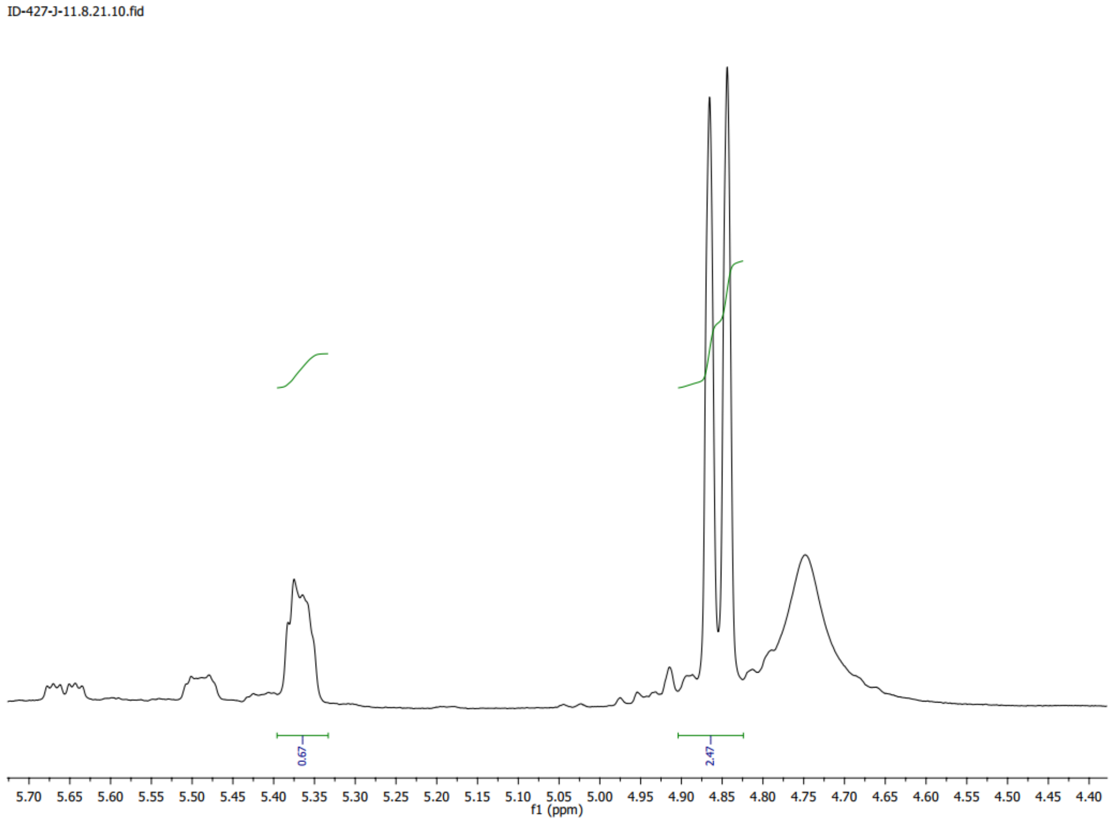

NMR traces for reaction with: **2,4-Me-phenylboronic acid (duplicate) (400 MHz,** $CDCl_3$**)**

ID-427-J-11.8.21.20.fid

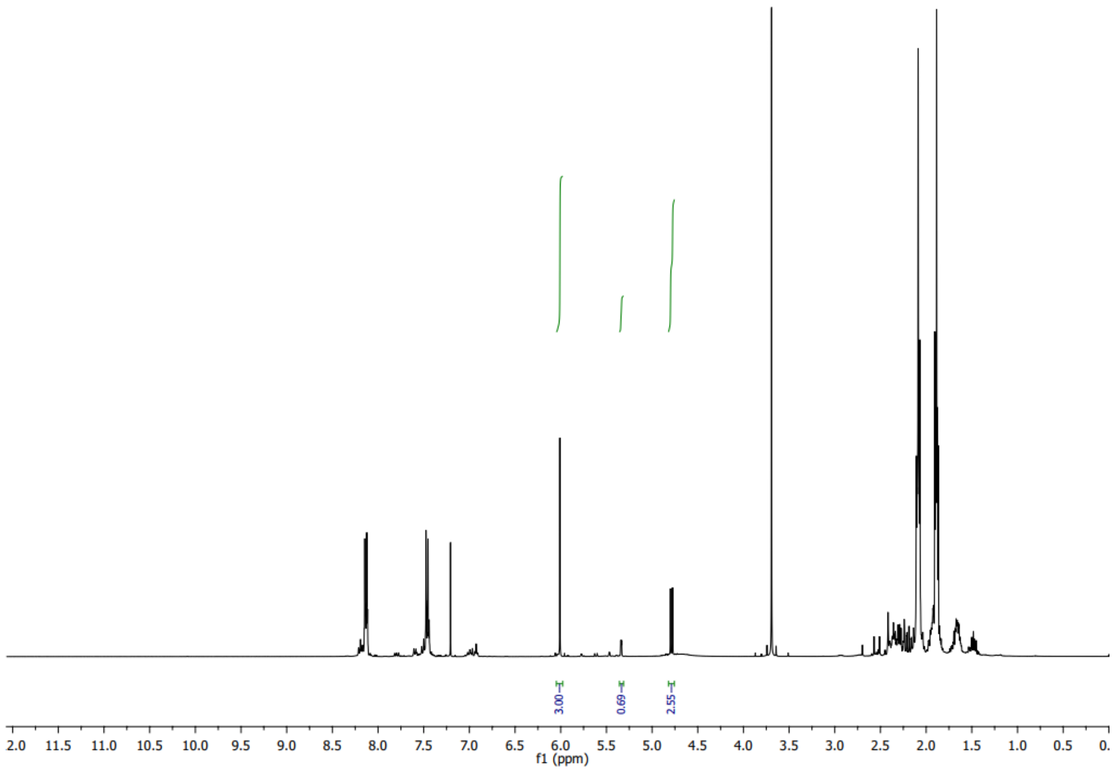

ID-427-J-11.8.21.20.fid

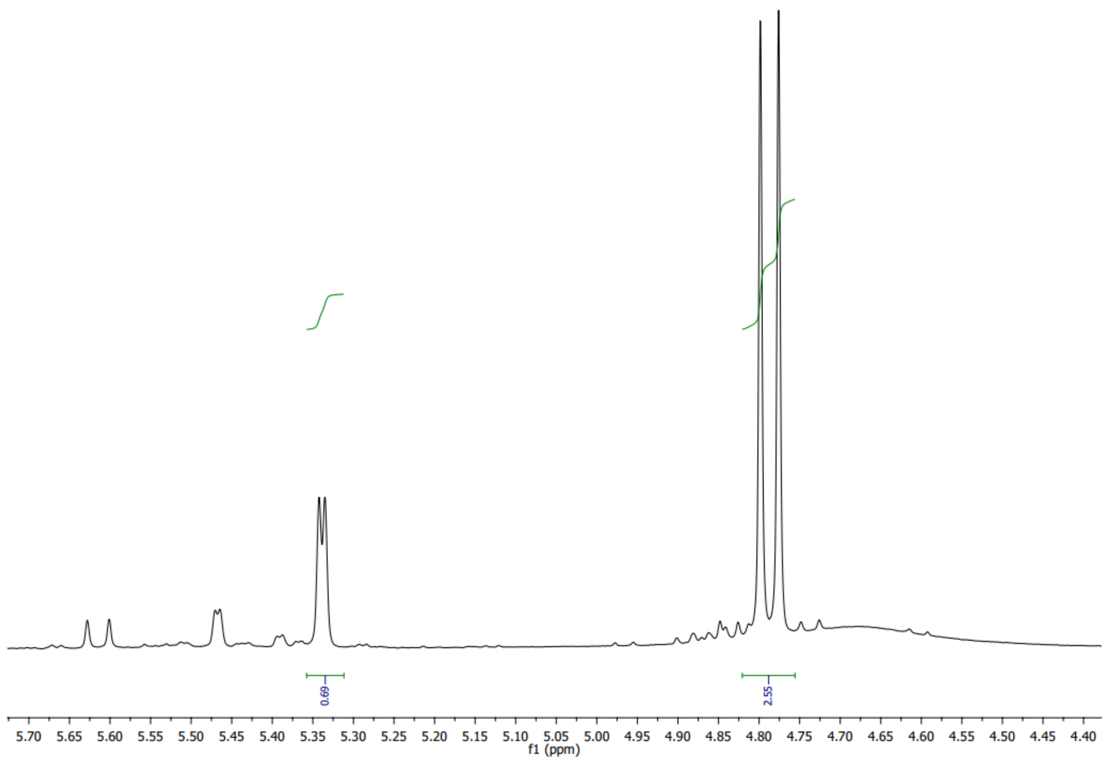

NMR traces for reaction with: **3,5-OMe-phenylboronic acid (duplicate) (400 MHz,** $CDCl_3$**)**

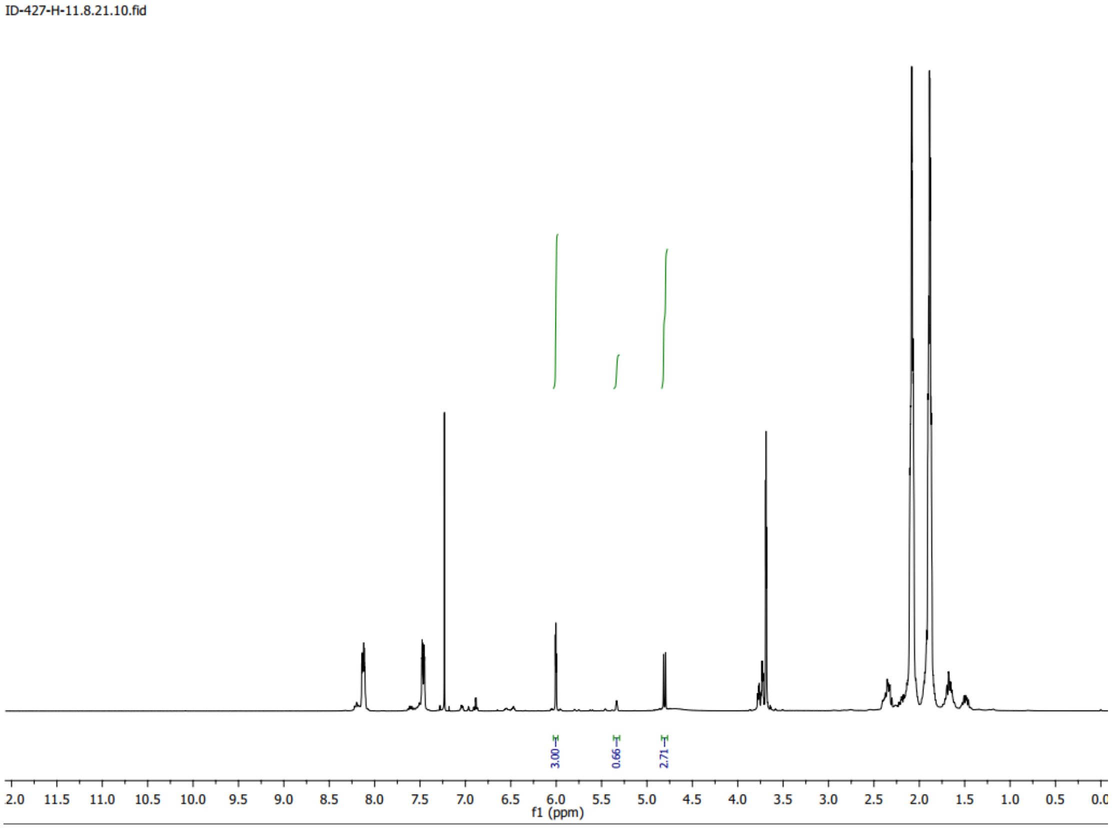

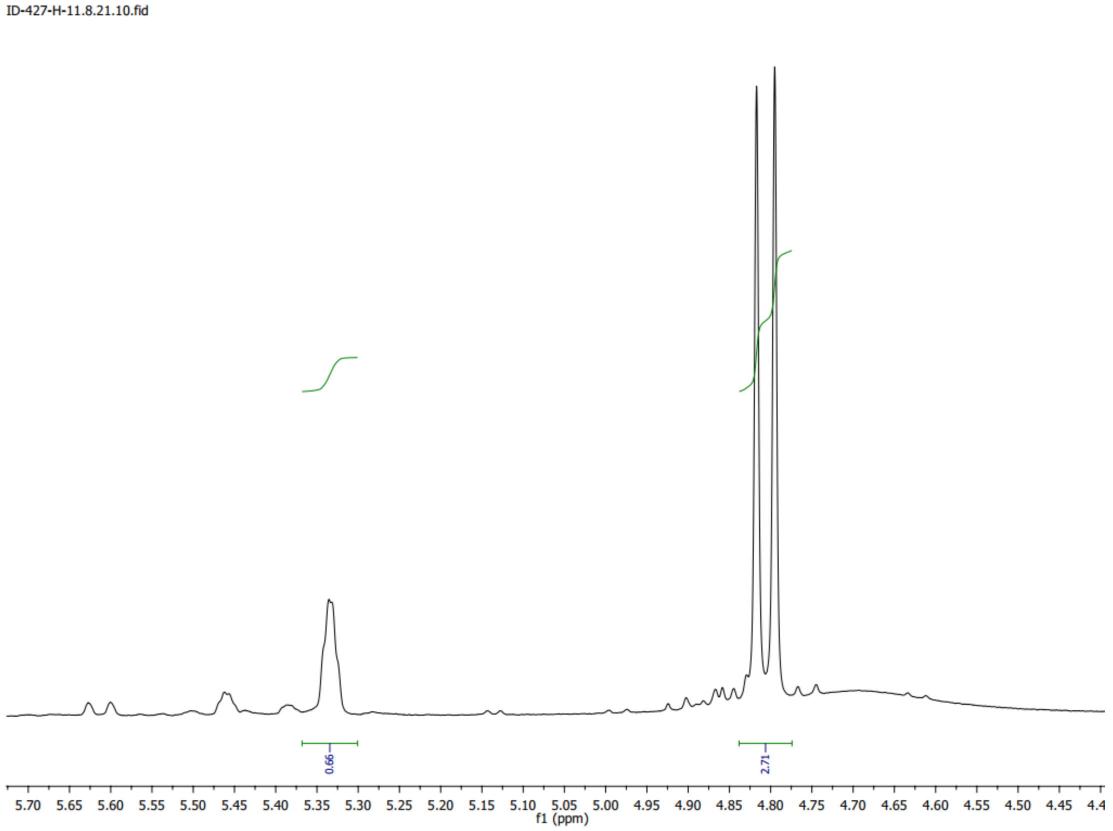

NMR traces for reaction with: **3,5-OMe-phenylboronic acid (duplicate) (400 MHz,** $CDCl_3$**)**

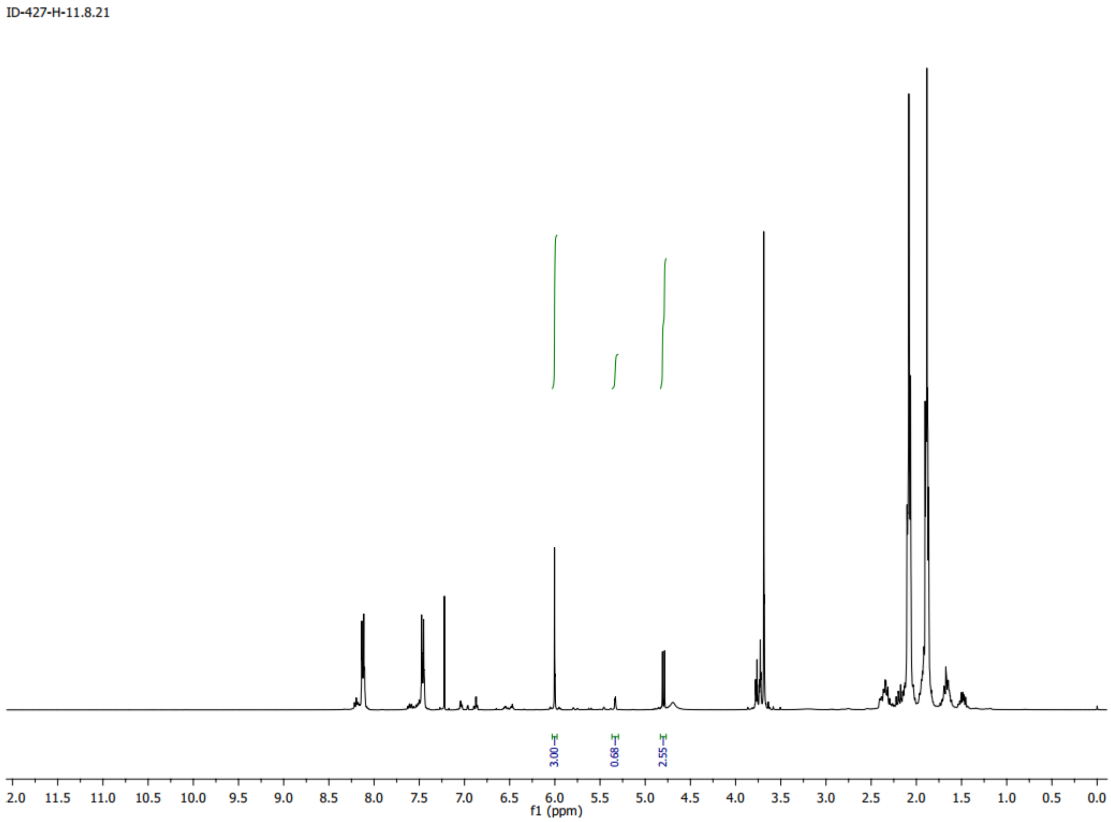

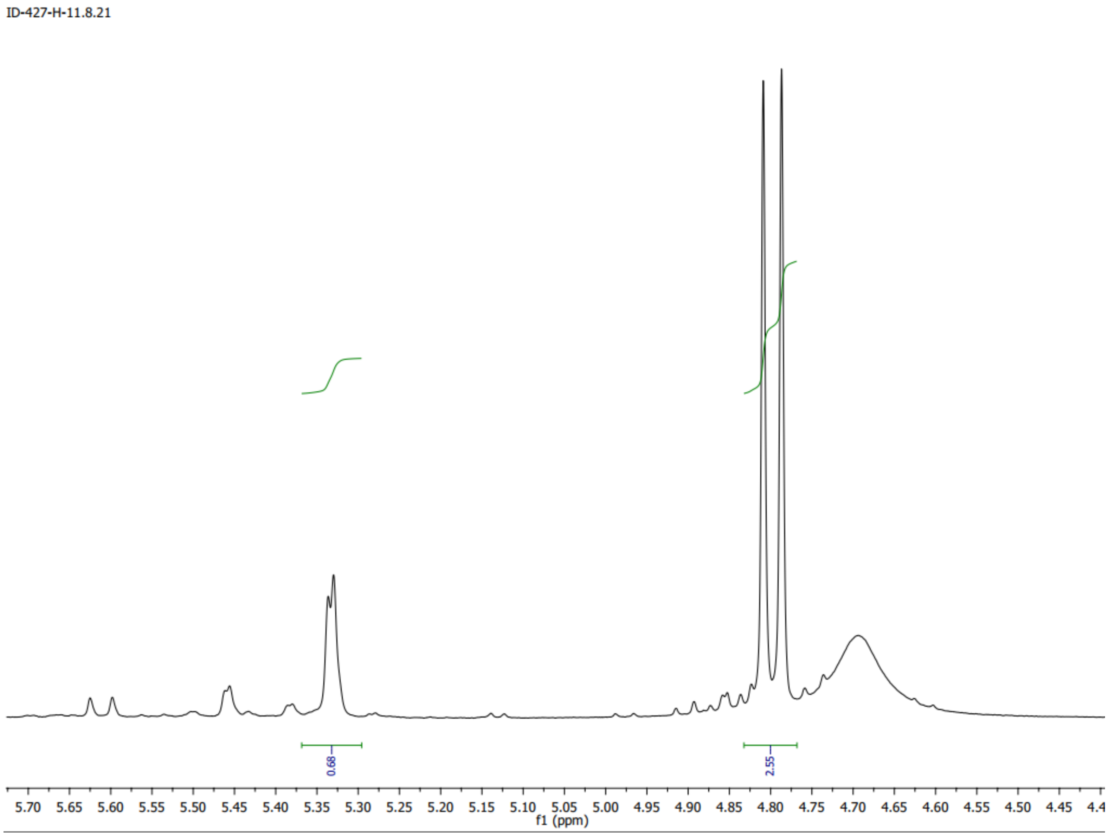

NMR traces for reaction with: **4-Me-phenylboronic acid (400 MHz,** $CDCl_3$**)**

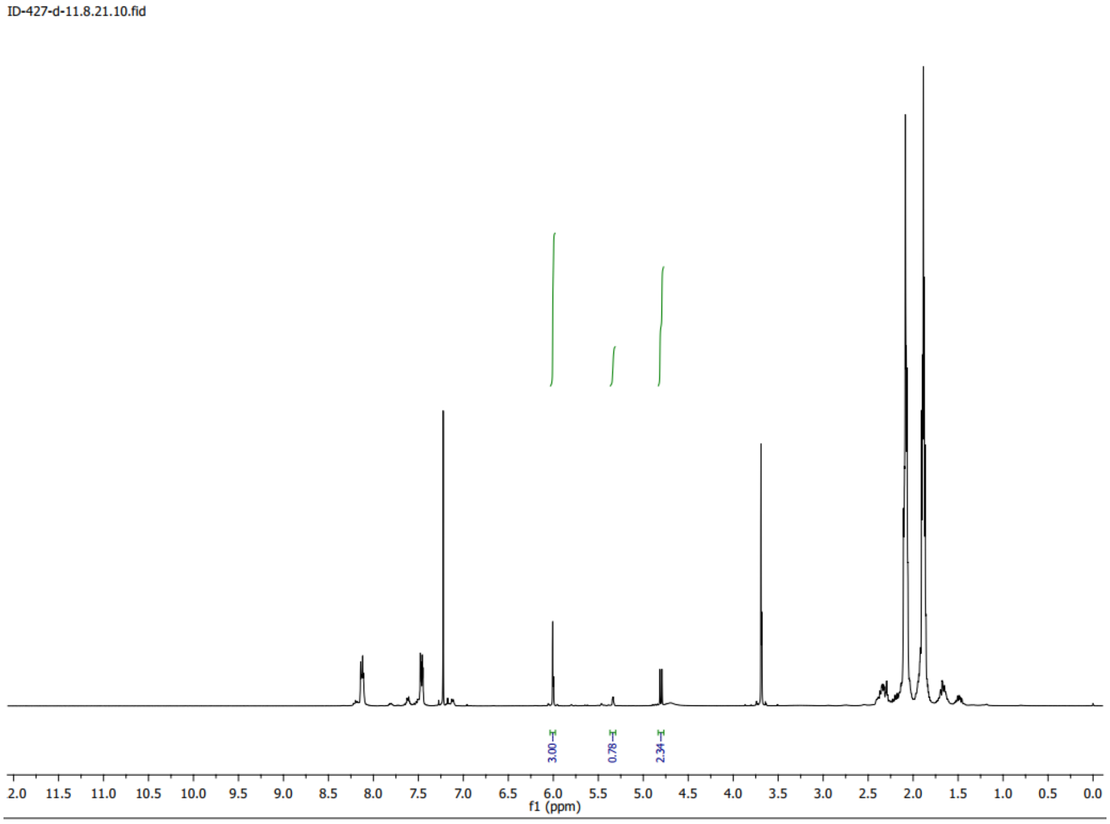

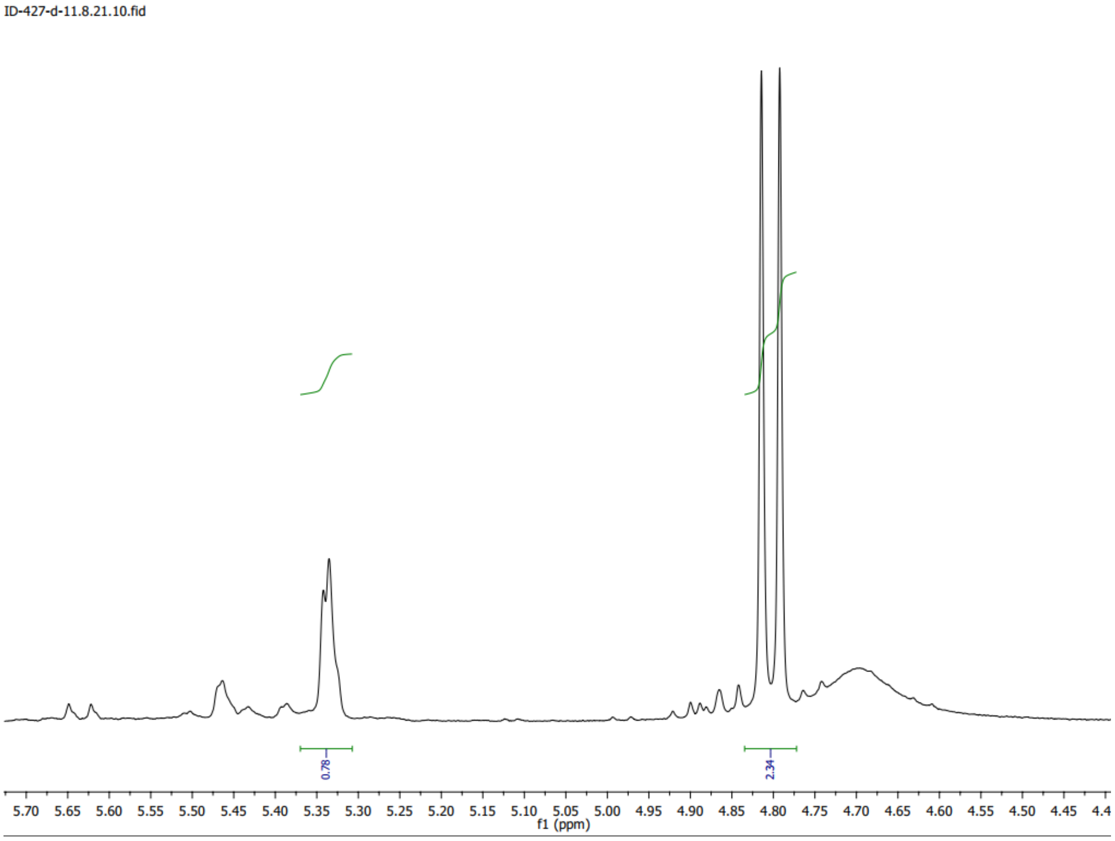

NMR traces for reaction with: **3-CF$_3$-phenylboronic acid (400 MHz,** CDCl$_3$**)**

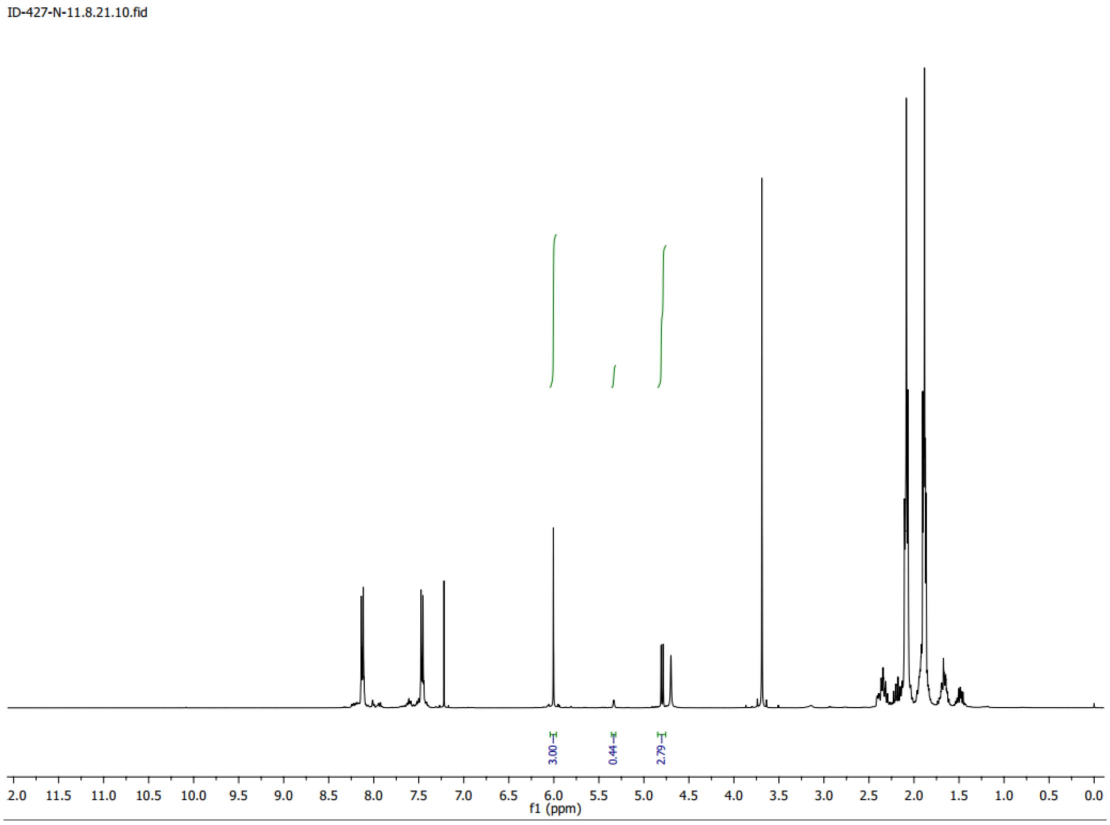

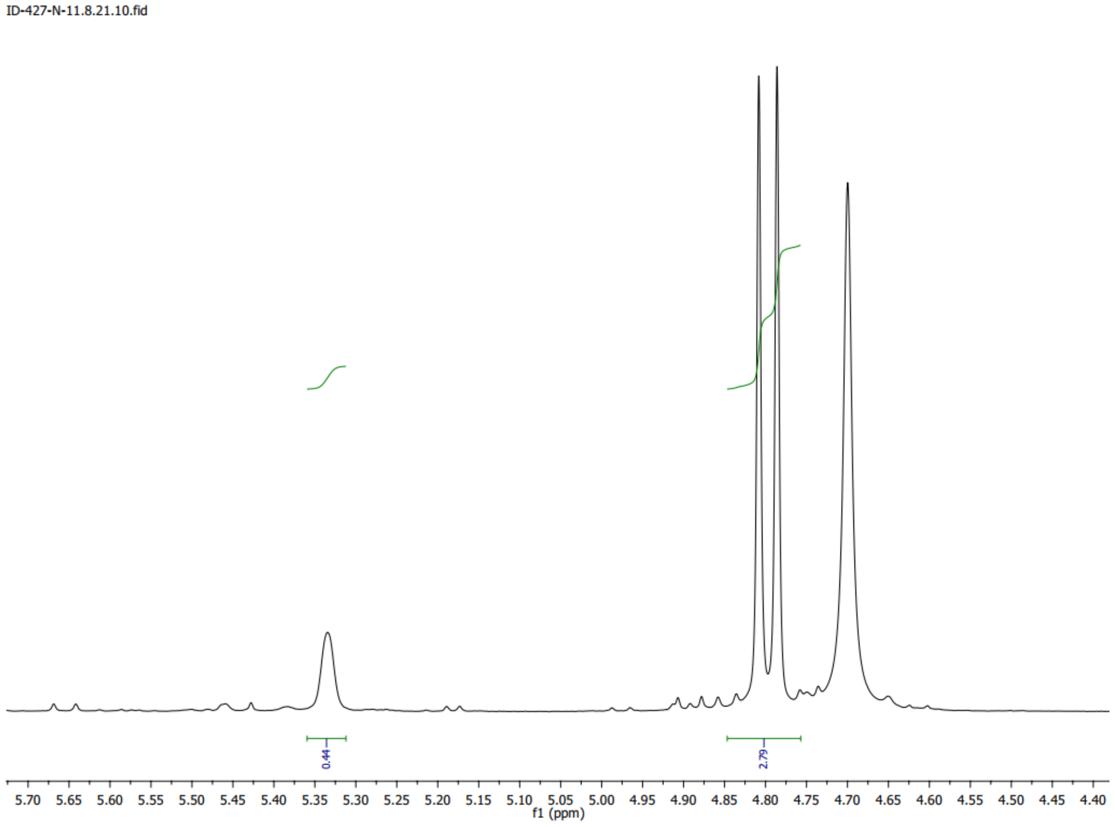

NMR traces for reaction with: **3-CF$_3$-phenylboronic acid (400 MHz,** $CDCl_3$**)**

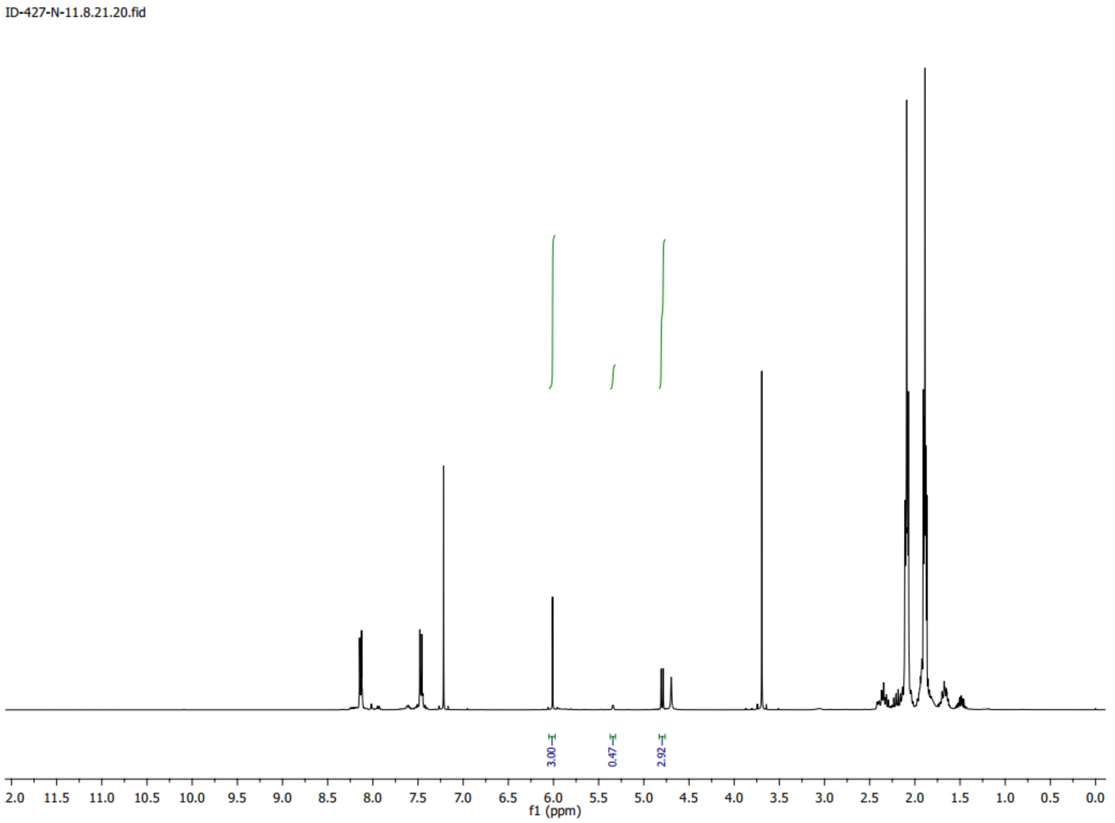

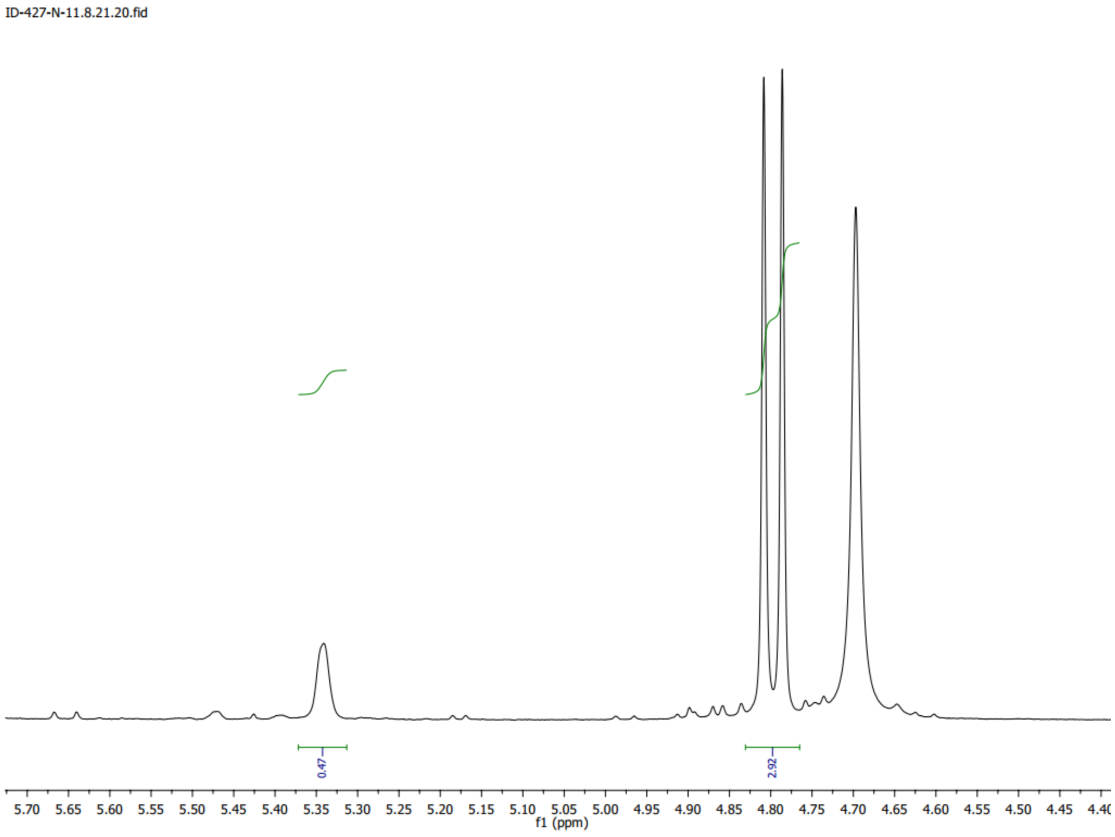

NMR traces for reaction with: **2-naphtalene-phenylboronic acid (400 MHz,** CDCl$_3$**)**

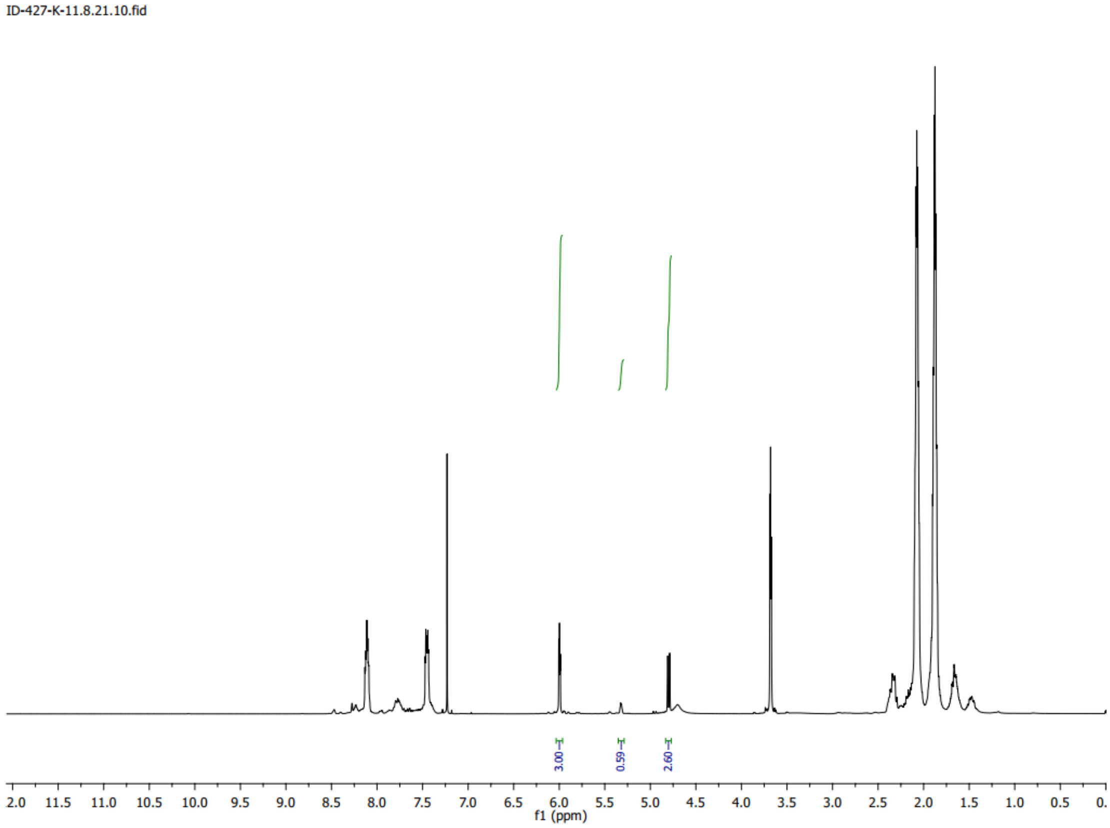

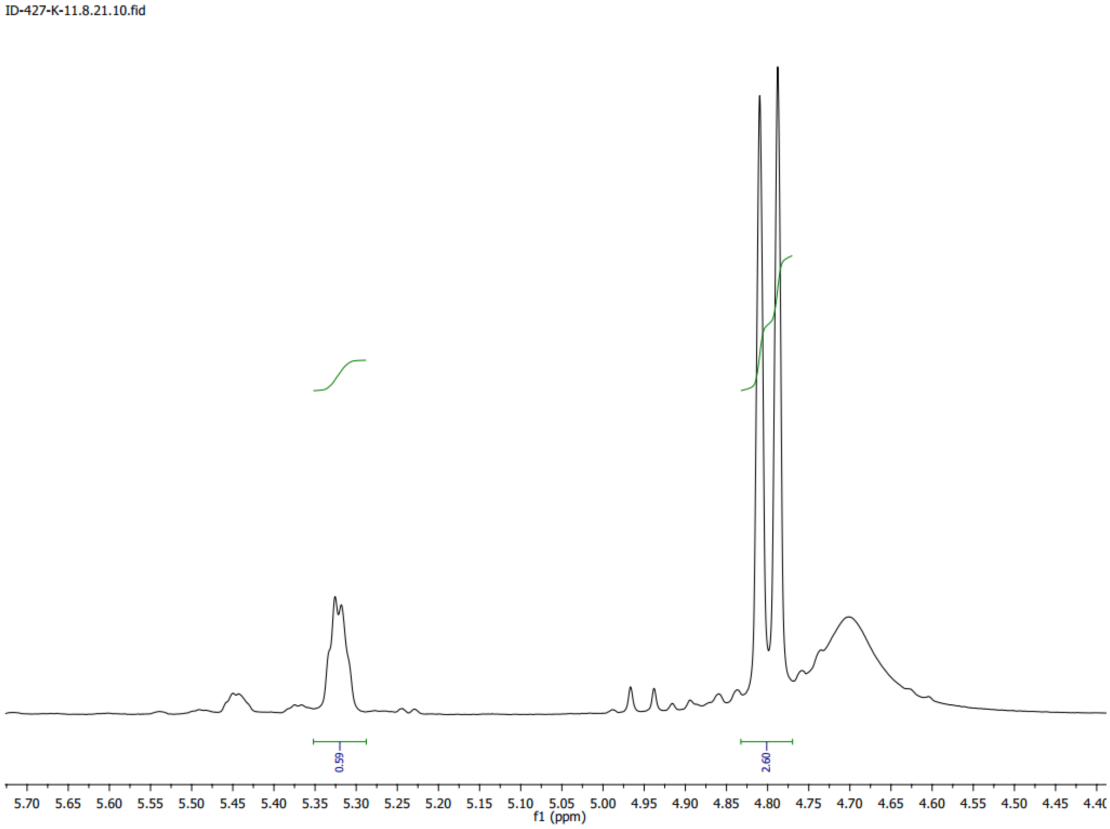

NMR traces for reaction with: **2-naphtalene-phenylboronic acid (duplicate) (400 MHz,** CDCl$_3$**)**

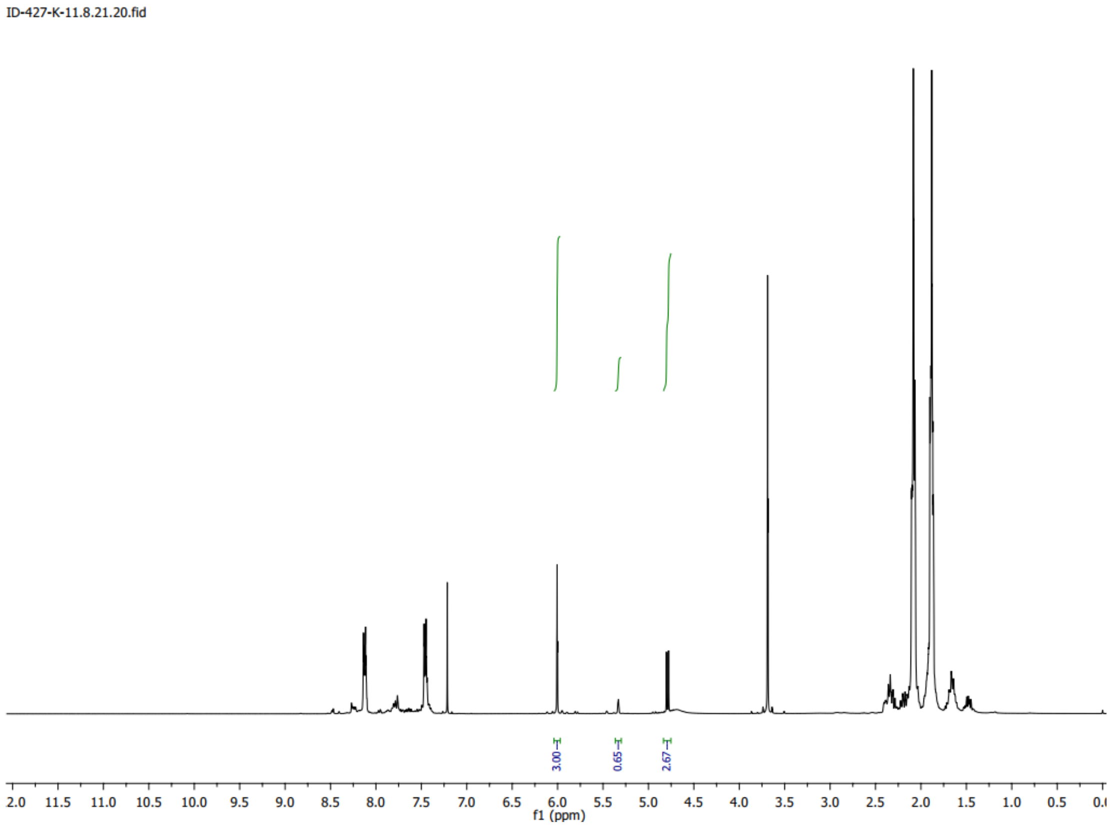

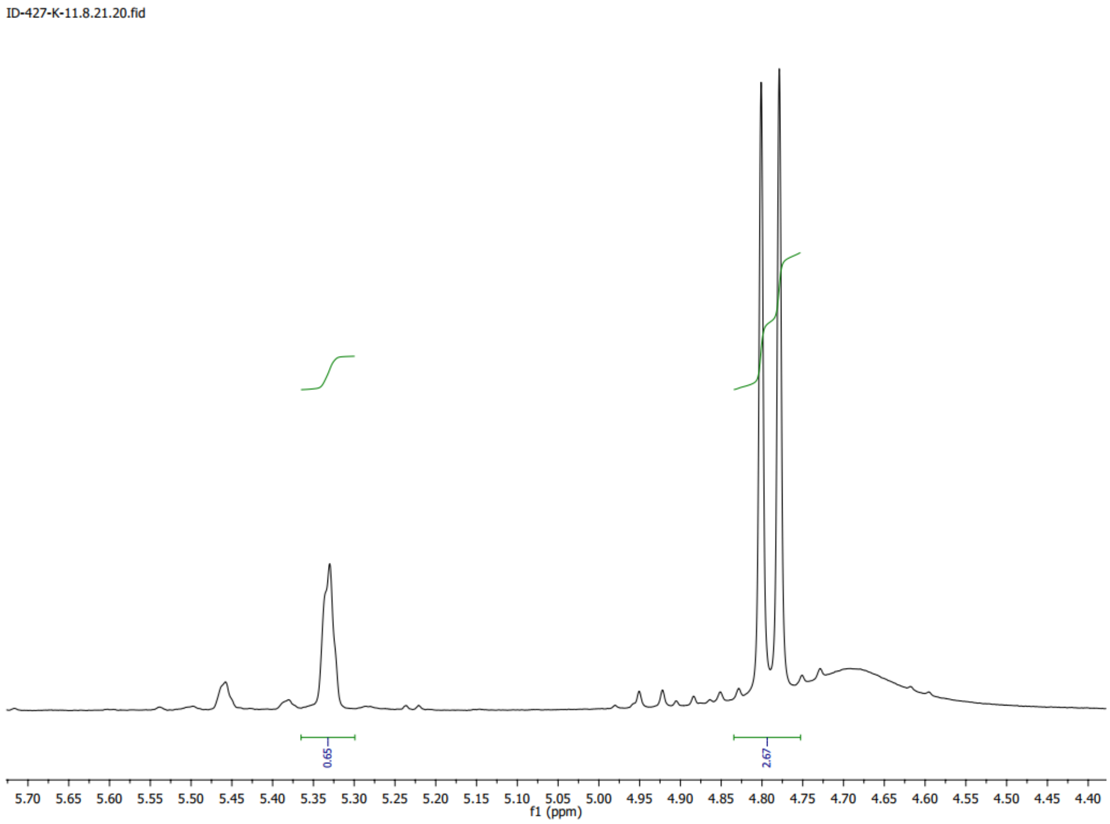

NMR traces for reaction with: **4-CF_3-phenylboronic acid (400 MHz,** $CDCl_3$)

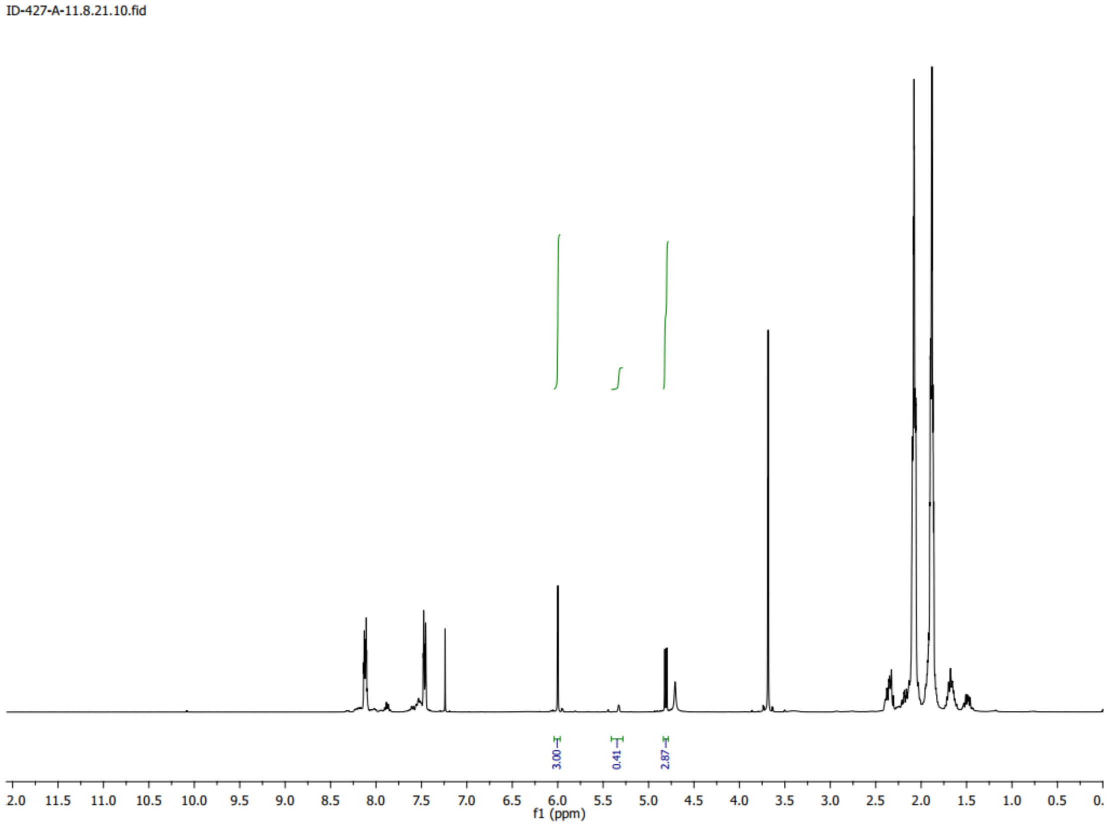

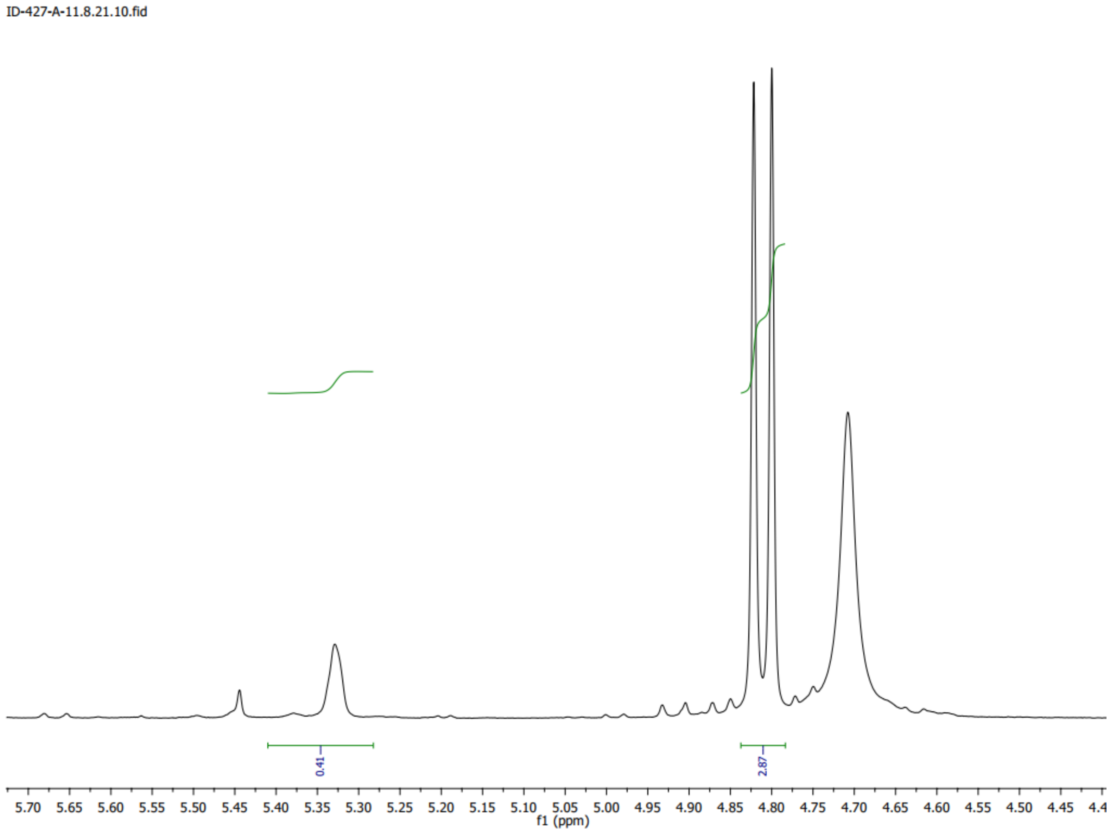

NMR traces for reaction with: **4-CF_3-phenylboronic acid (duplicate) (400 MHz,** $CDCl_3$**)**

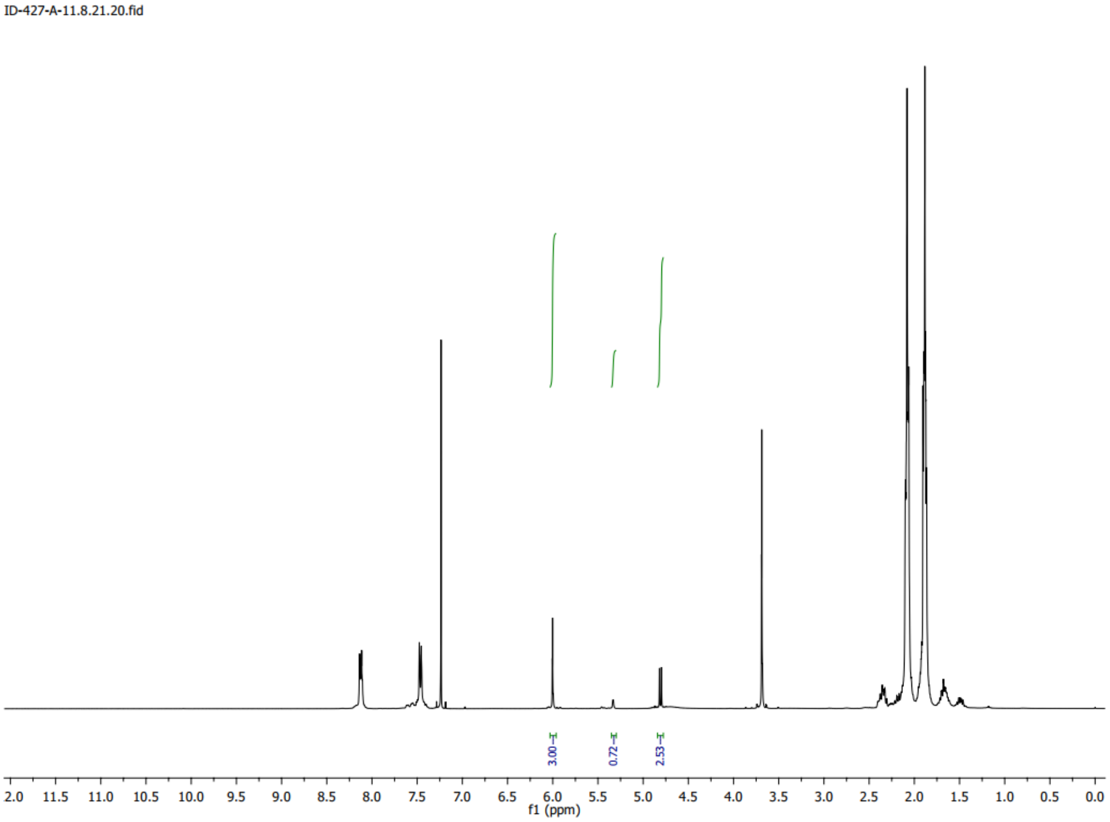

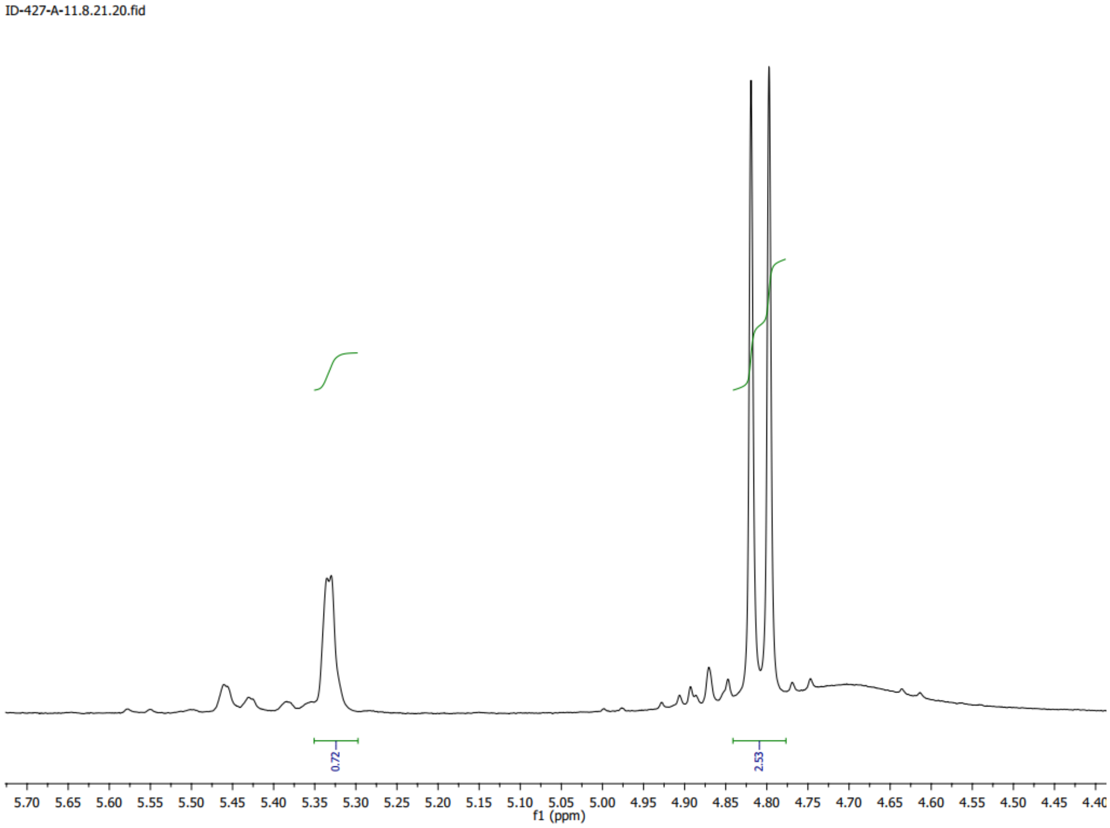

NMR traces for reaction with: **4-F-phenylboronic acid (400 MHz,** CDCl$_3$**)**

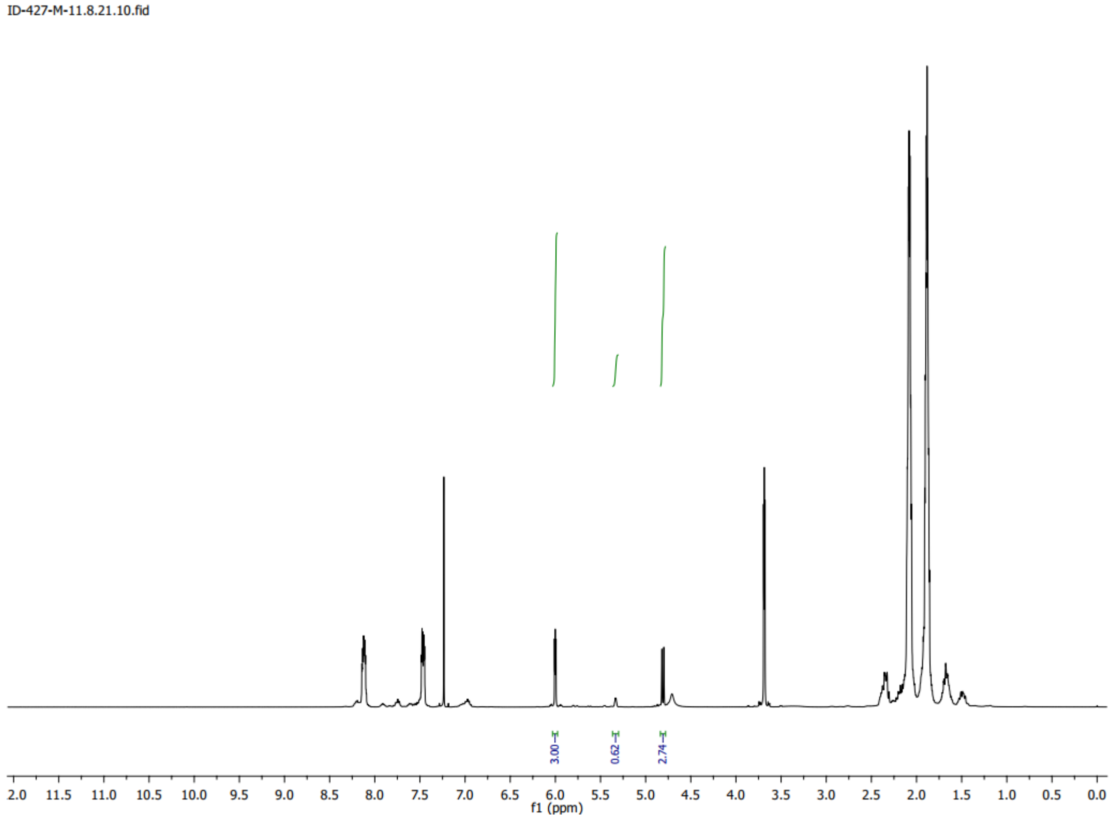

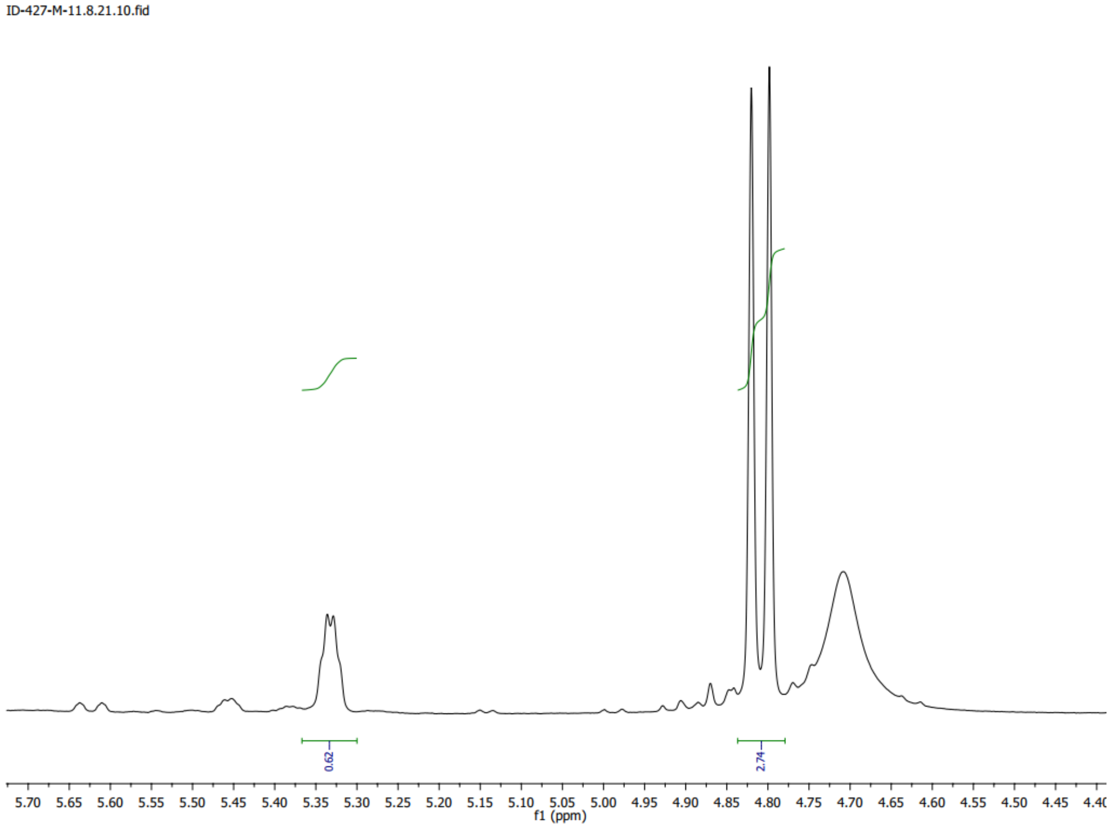

NMR traces for reaction with: **4-F-phenylboronic acid (duplicate) (400 MHz,** $CDCl_3$**)**

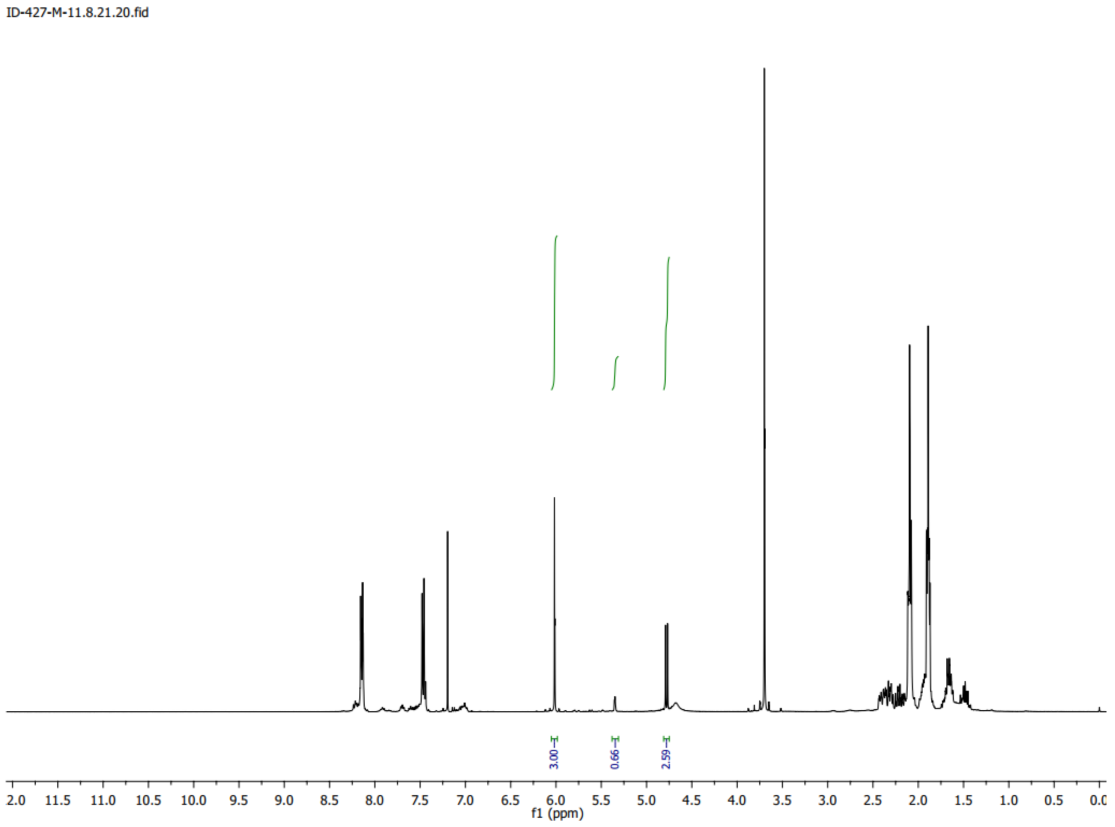

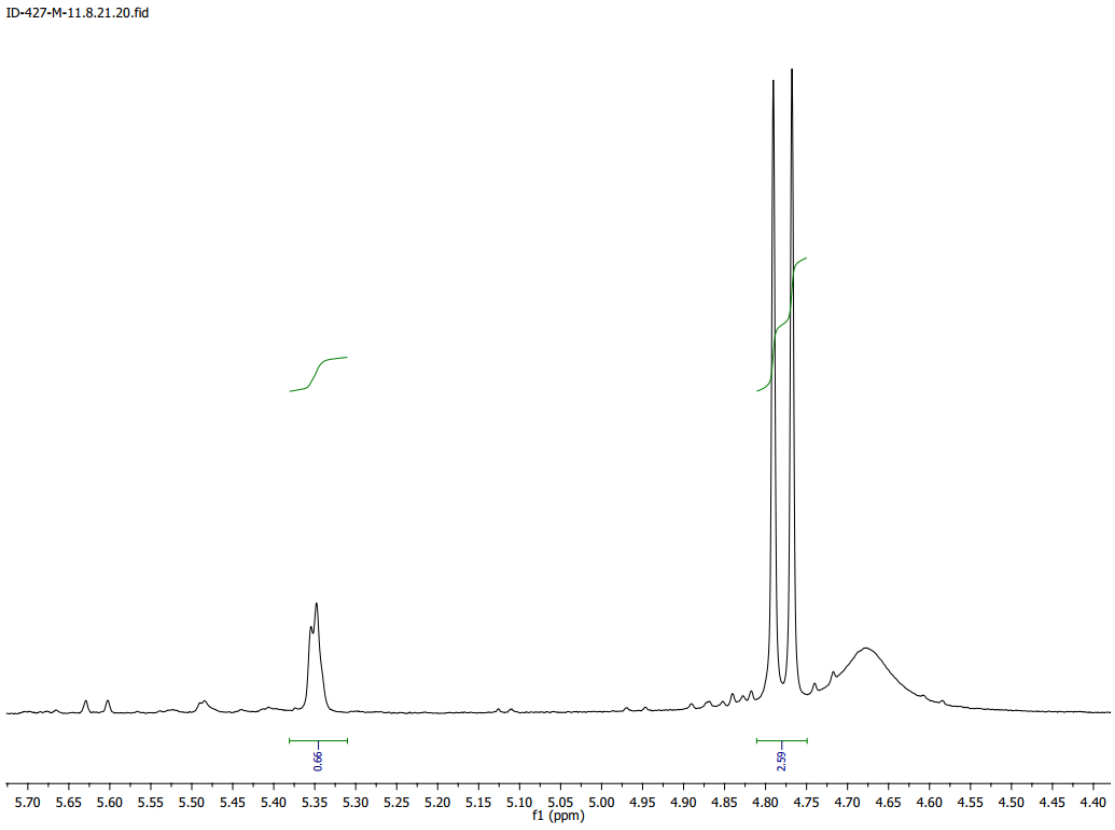

NMR traces for reaction with: **4-OMe-phenylboronic acid (400 MHz,** $CDCl_3$**)**

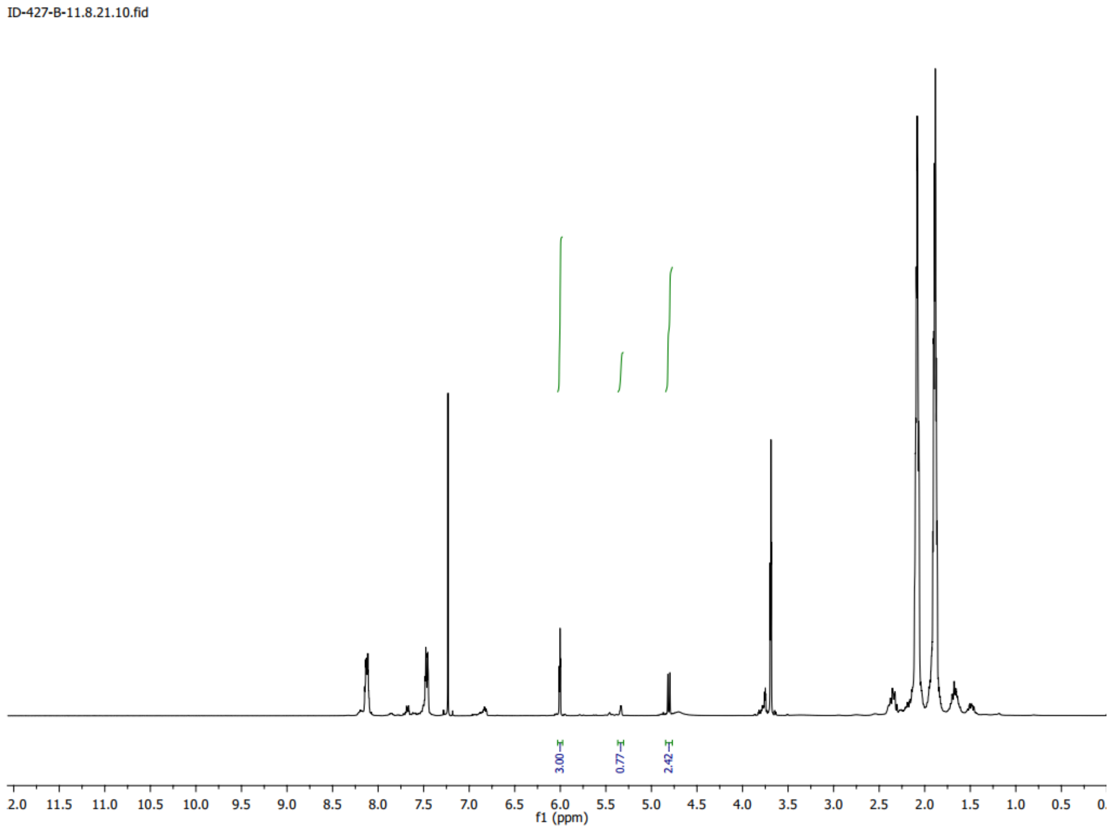

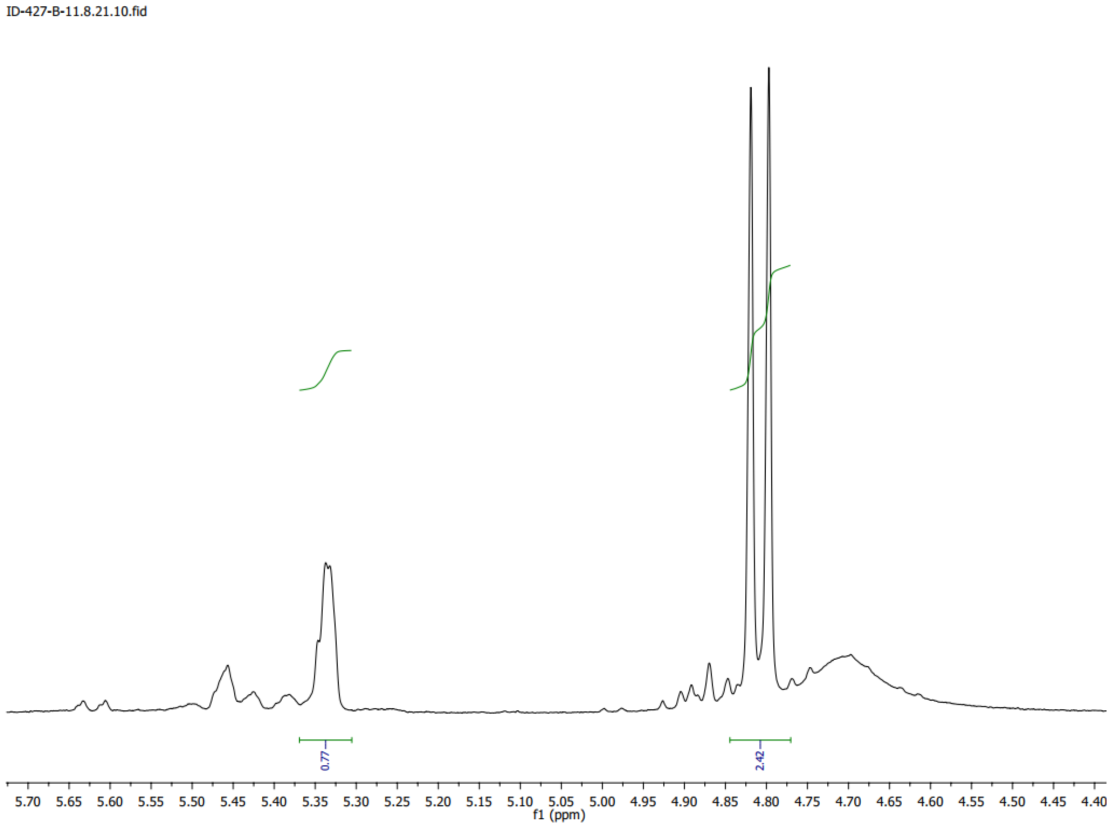

NMR traces for reaction with: **4-OMe-phenylboronic acid (duplicate) (400 MHz,** $CDCl_3$**)**

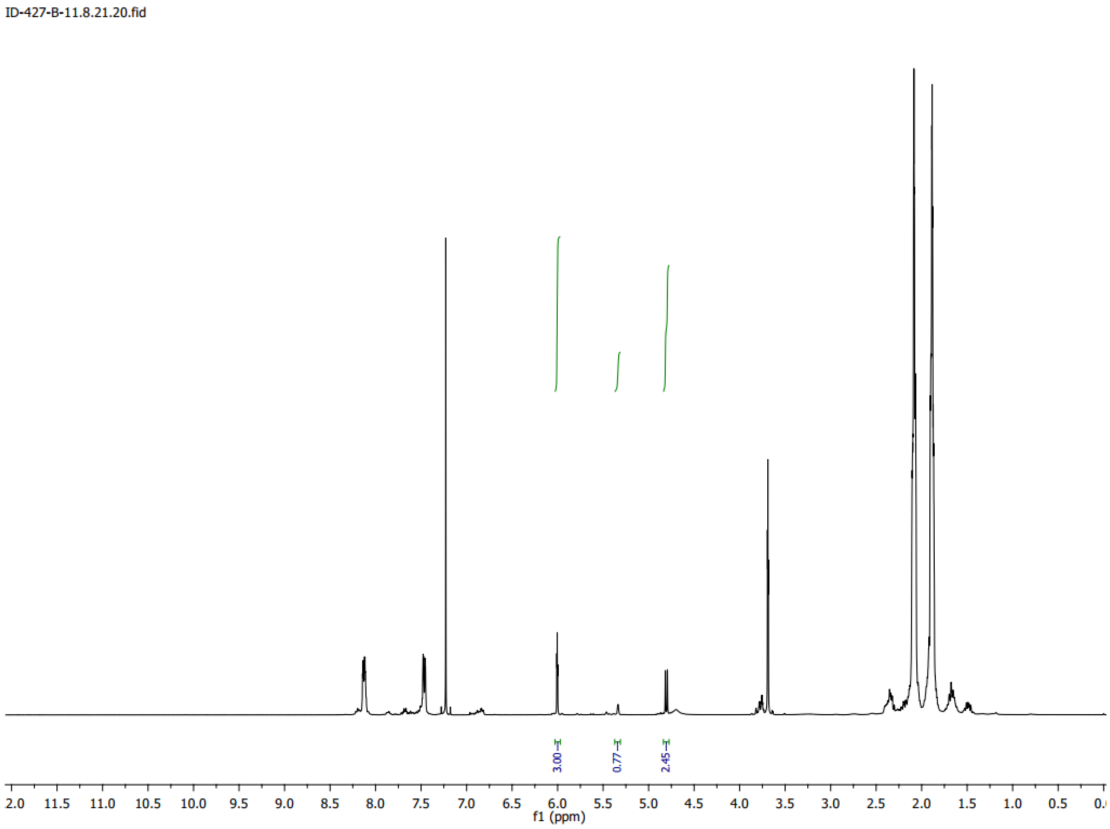

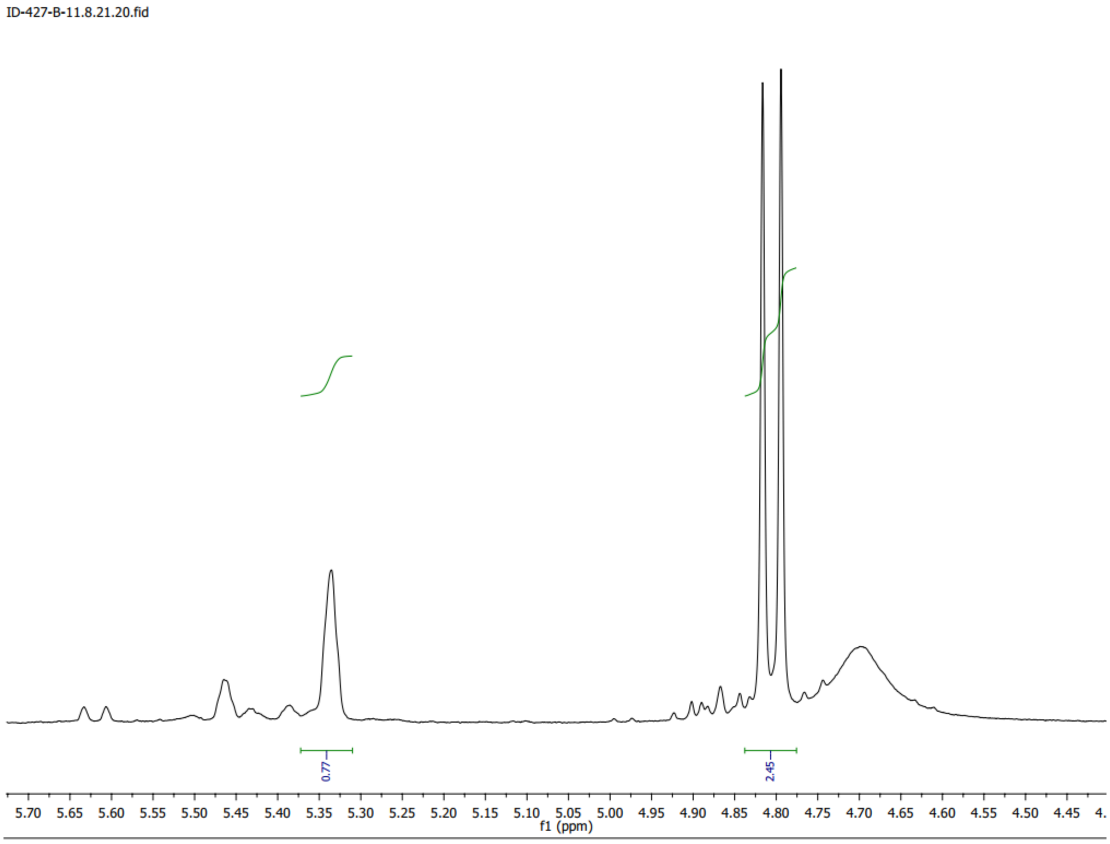

NMR traces for reaction with: **3-Me-phenylboronic acid (400 MHz,** $CDCl_3$**)**

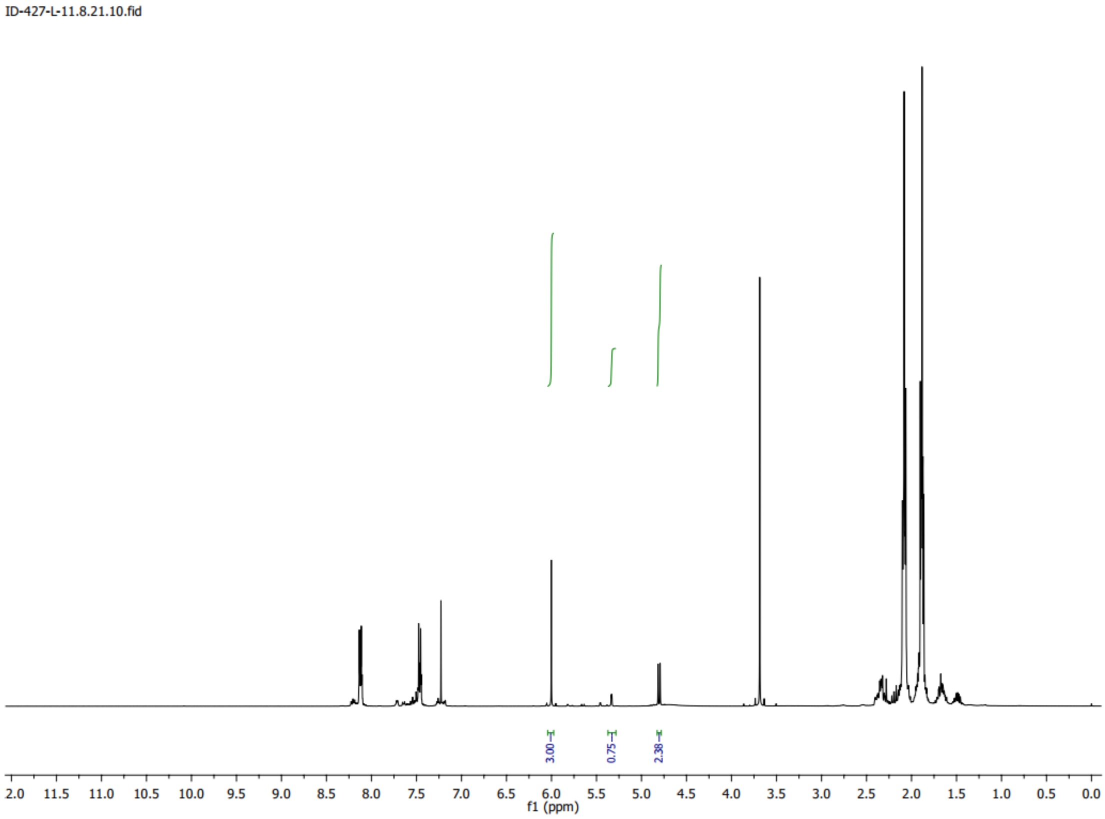

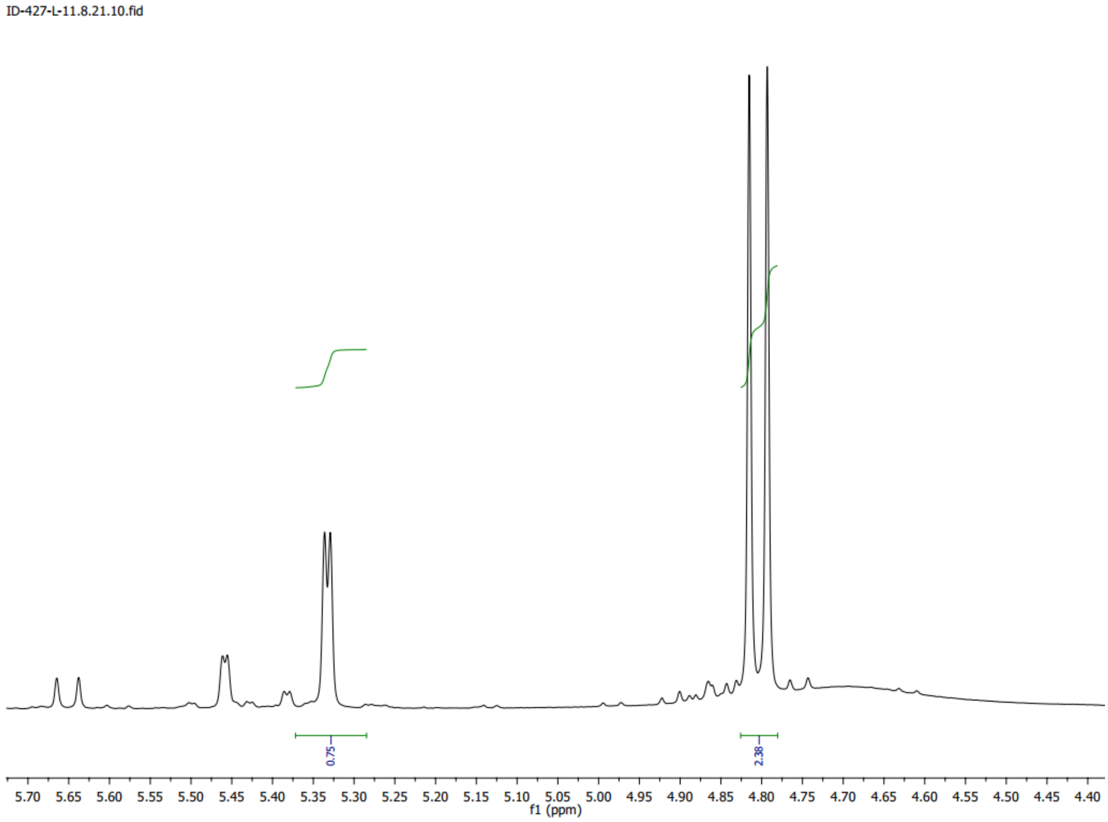

NMR traces for reaction with: **3-Me-phenylboronic acid (duplicate) (400 MHz,** CDCl$_3$**)**

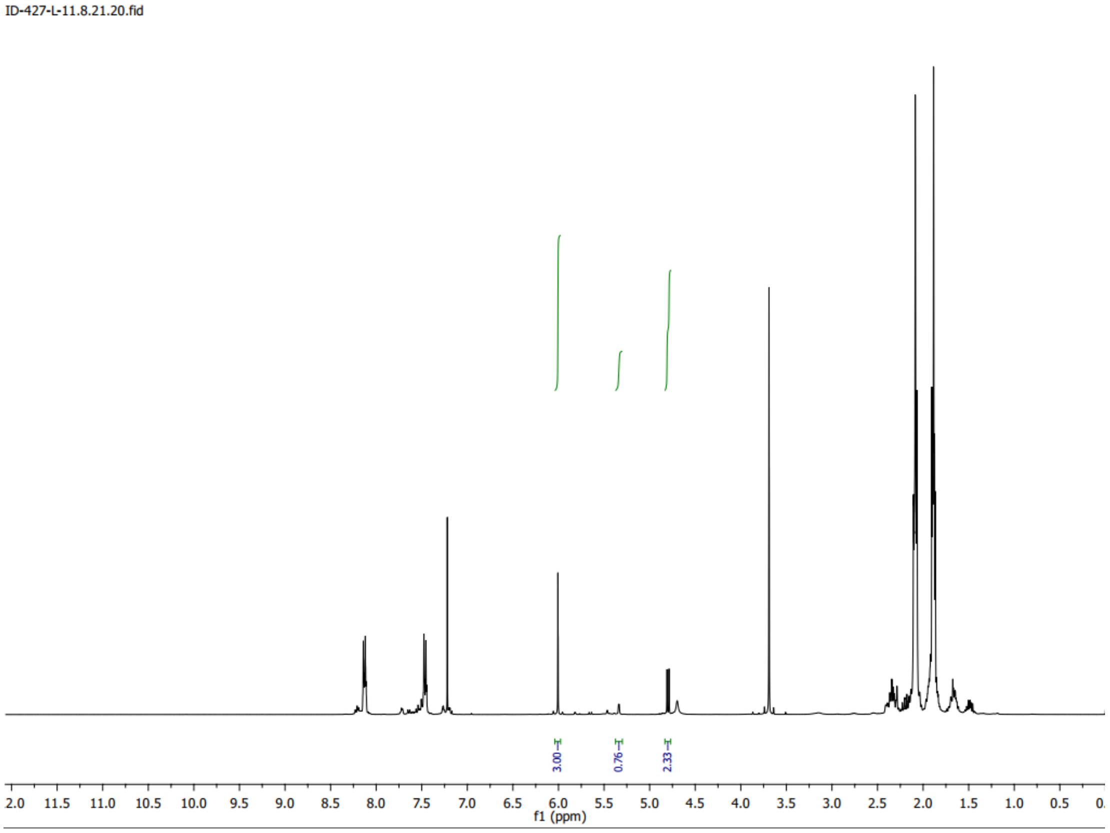

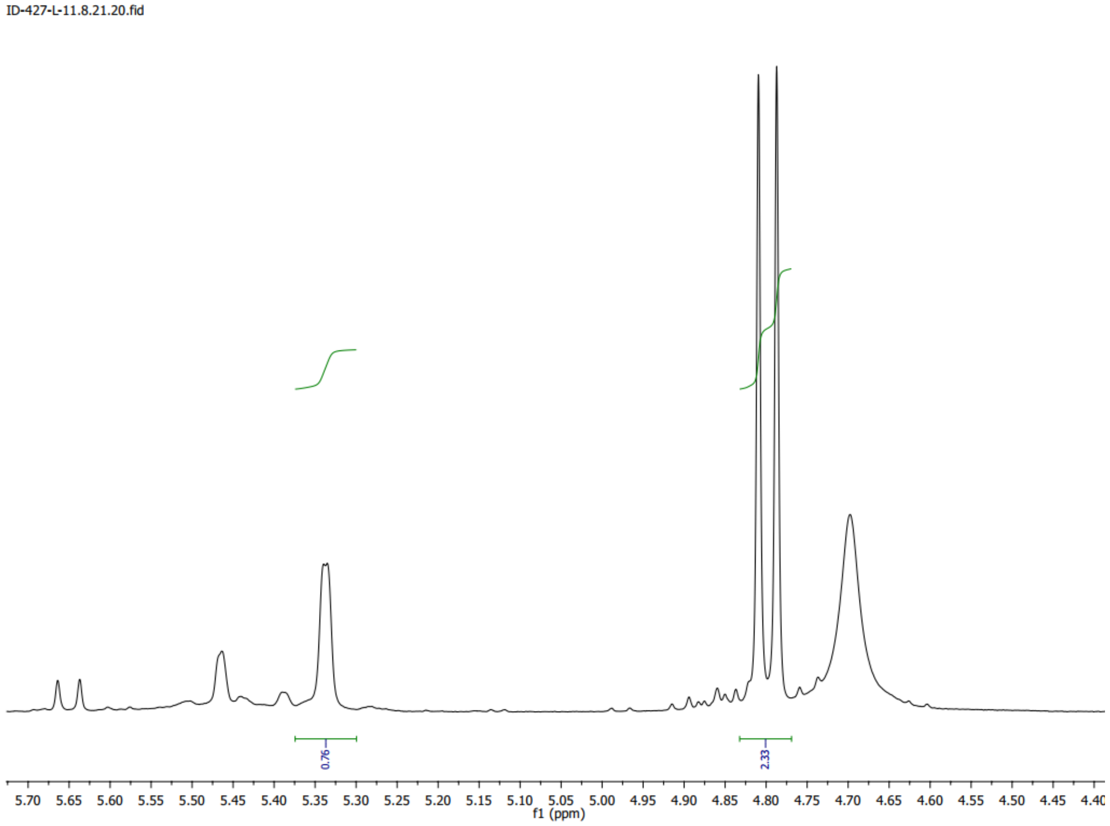

NMR traces for reaction with: **2-Me-phenylboronic acid (400 MHz,** CDCl$_3$**)**

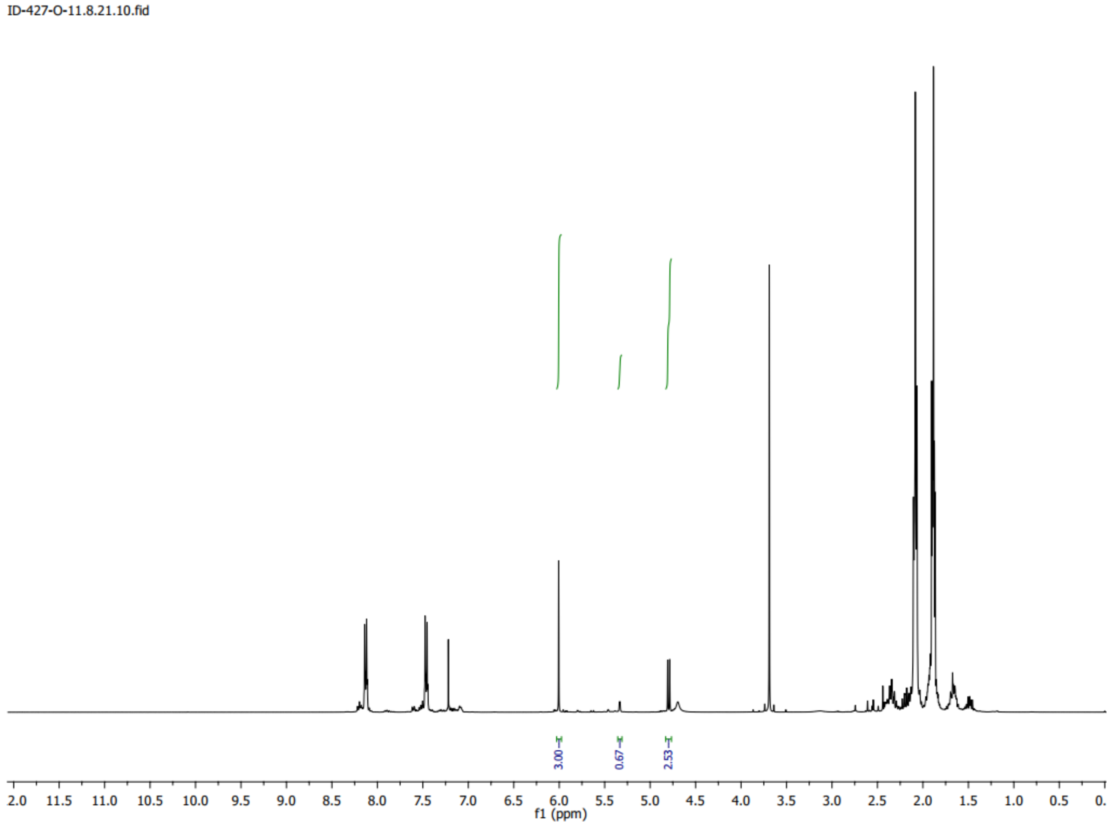

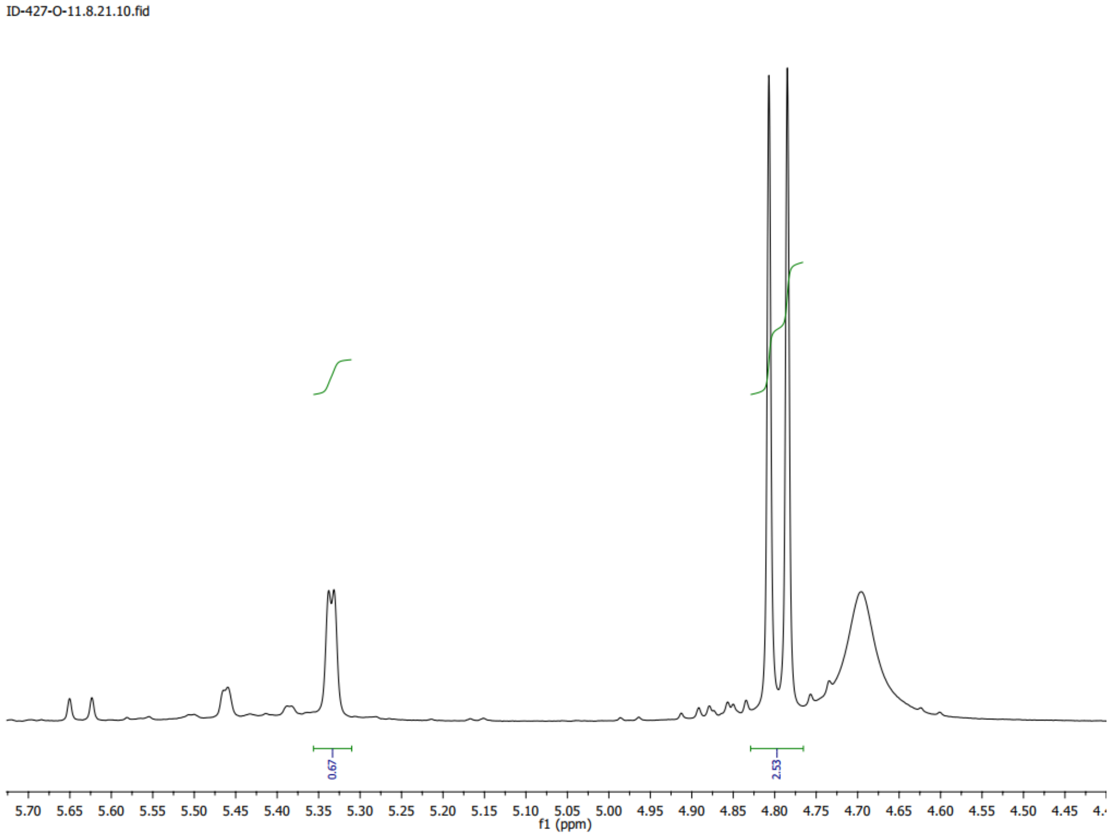

NMR traces for reaction with: **2-Me-phenylboronic acid (duplicate) (400 MHz,** $CDCl_3$**)**

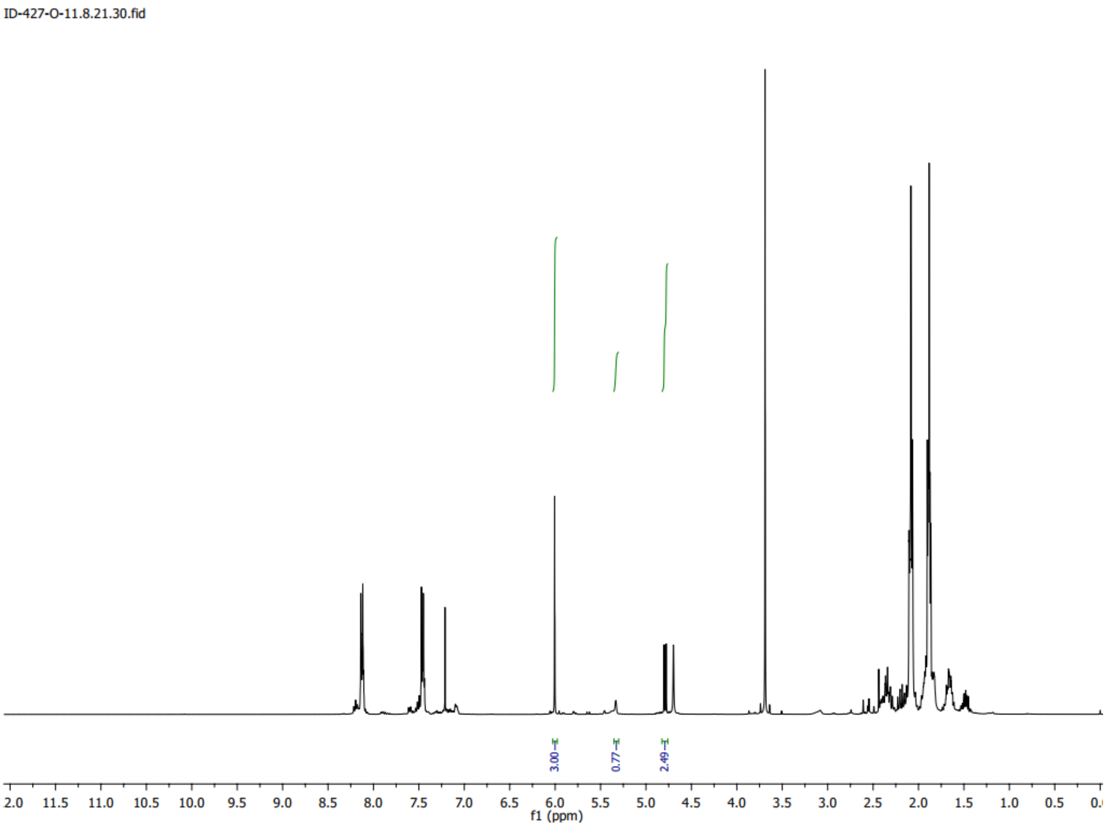

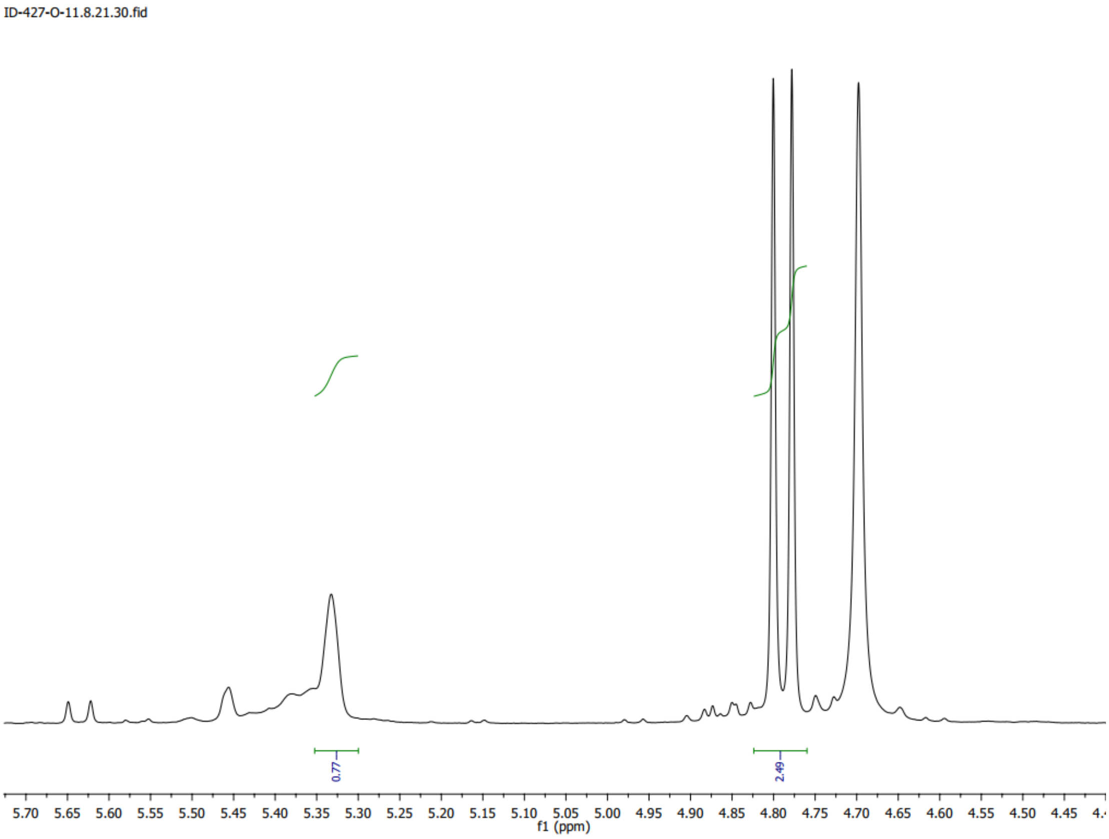

NMR traces for reaction with: **Ph-phenylboronic acid (400 MHz,** $CDCl_3$**)**

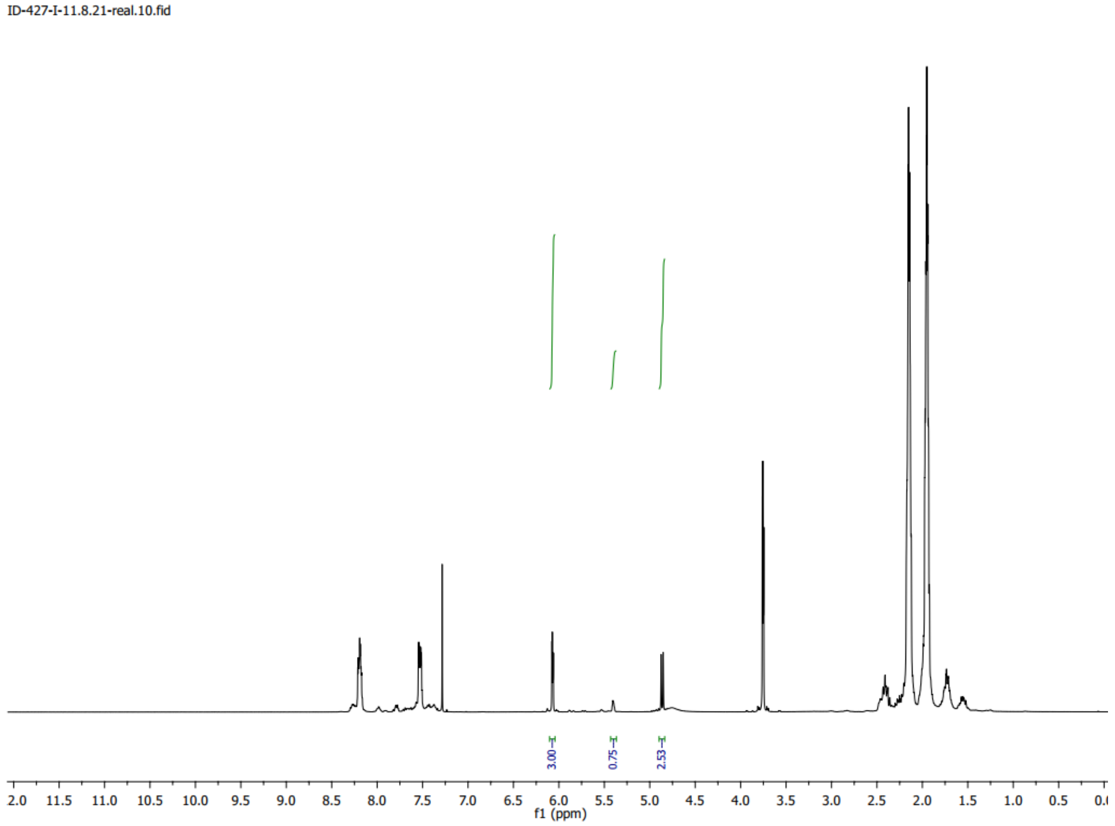

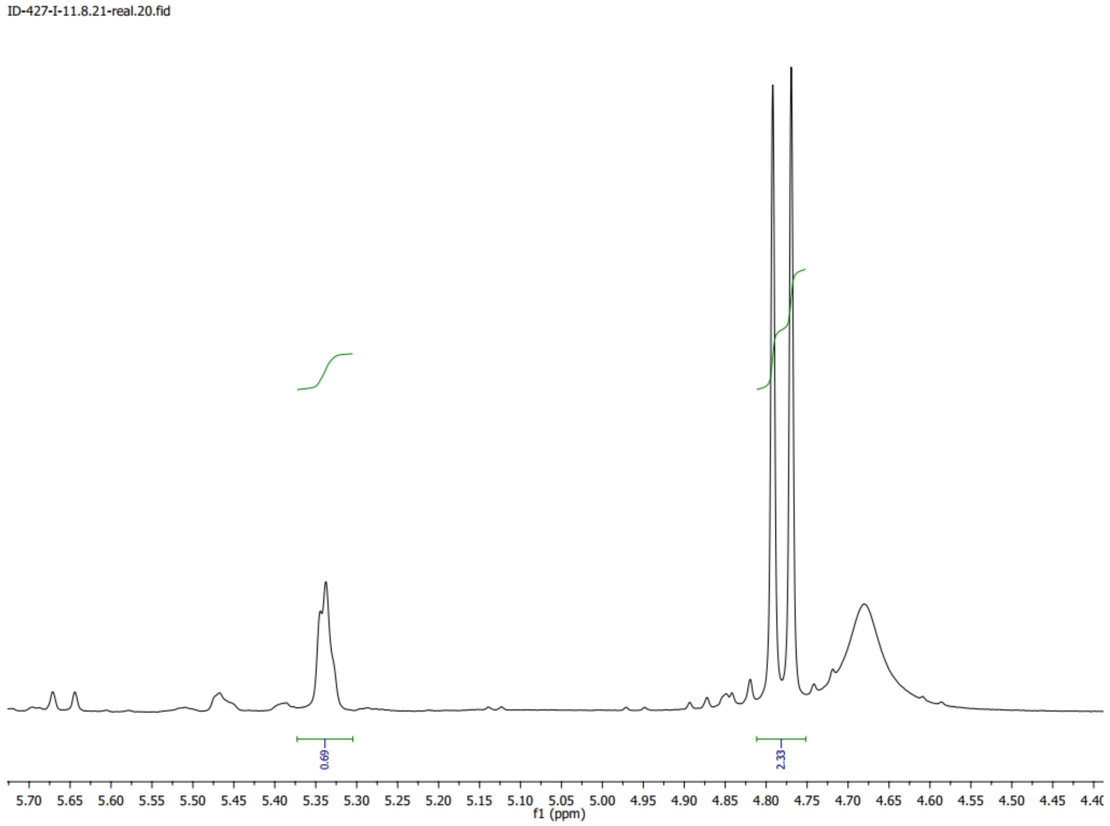

NMR traces for reaction with: **Ph-phenylboronic acid (duplicate) (400 MHz,** CDCl$_3$**)**

ID-427-I-11.8.21-real.20.fid

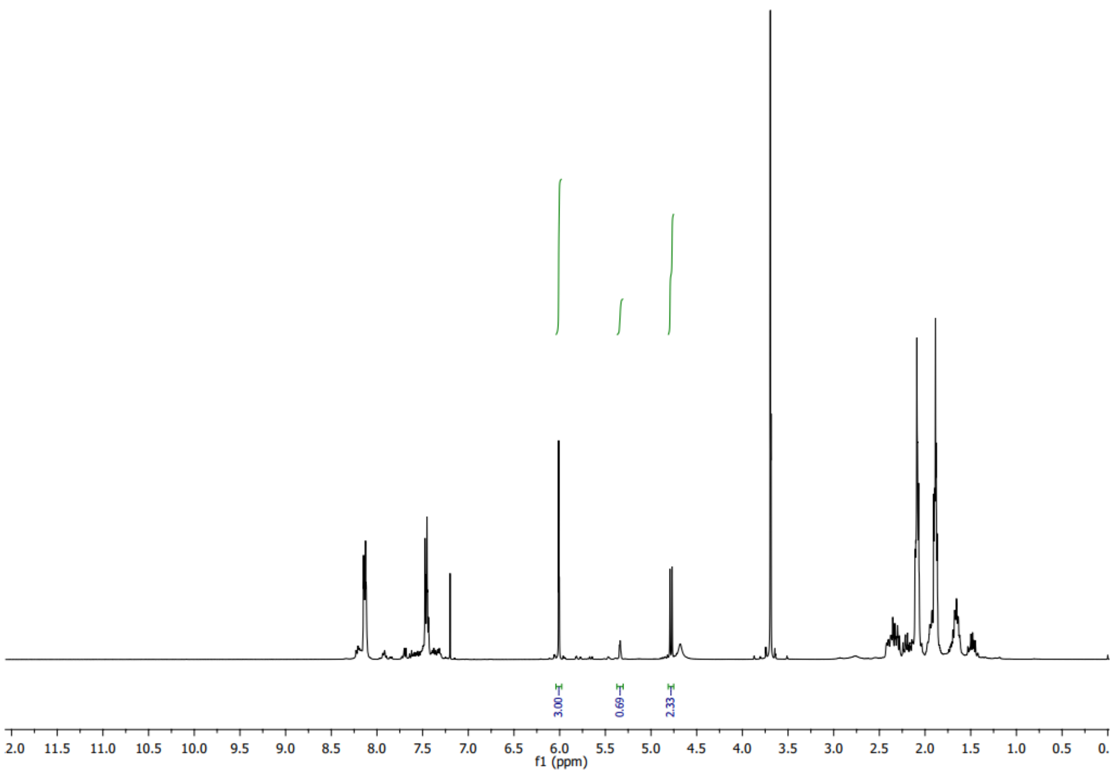

ID-427-I-11.8.21-real.10.fid

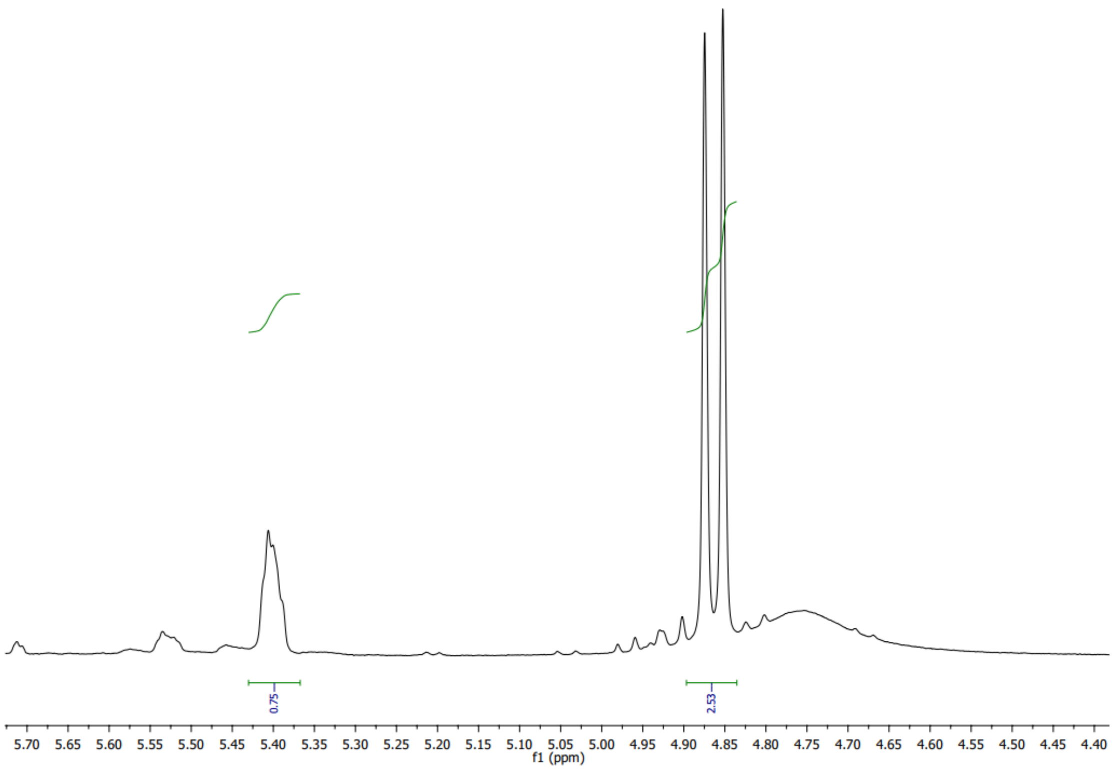

NMR traces for reaction with: **2-F-phenylboronic acid (400 MHz,** CDCl$_3$**)**

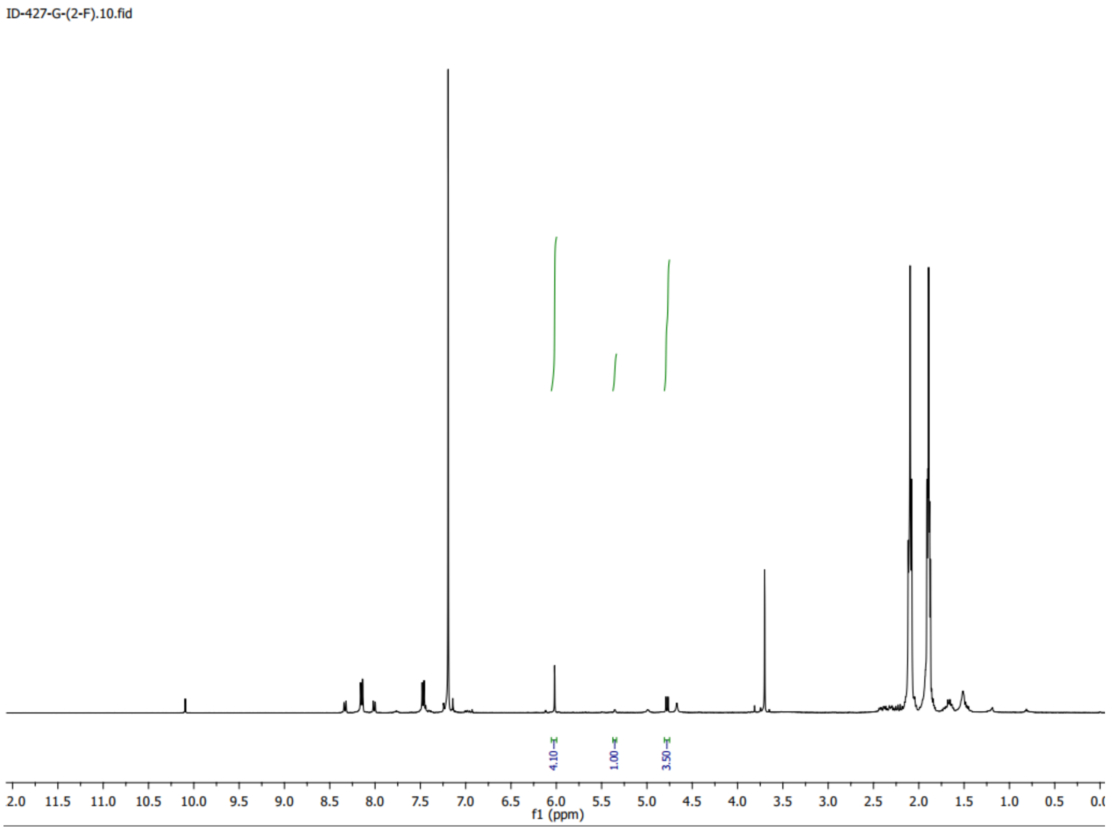

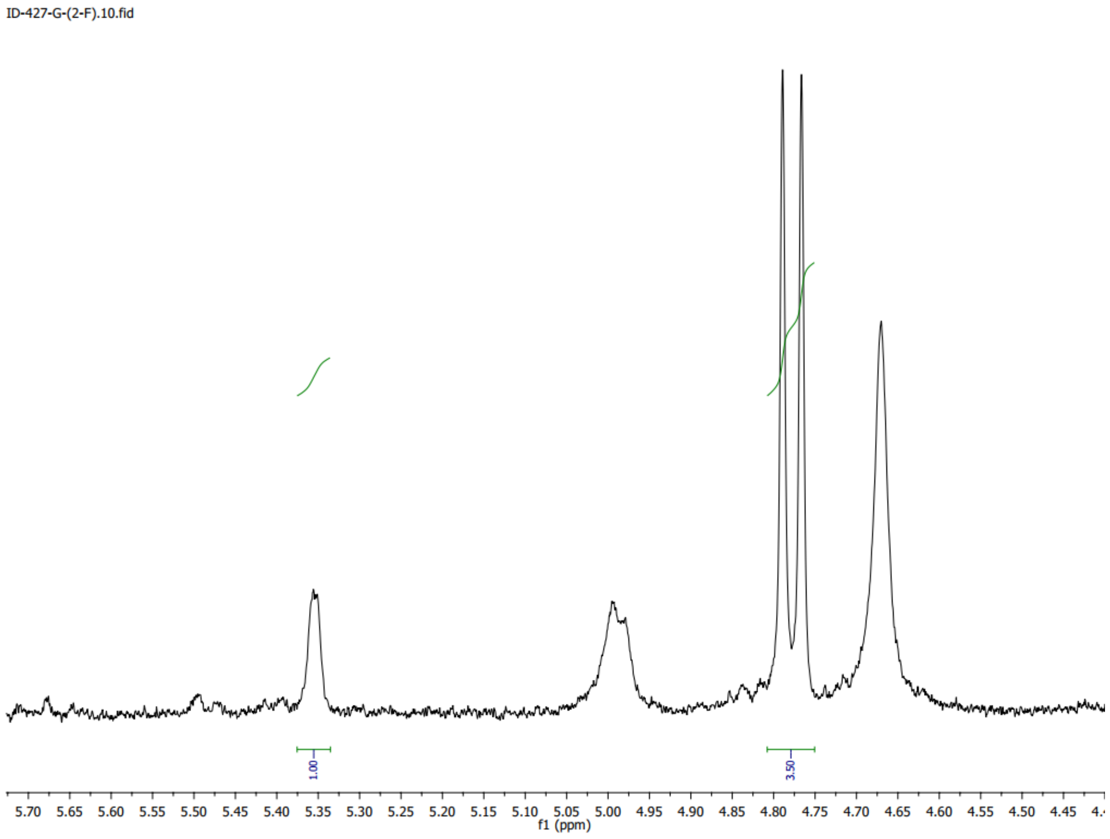

NMR traces for reaction with: **2-F-phenylboronic acid (duplicate) (400 MHz,** CDCl$_3$**)**

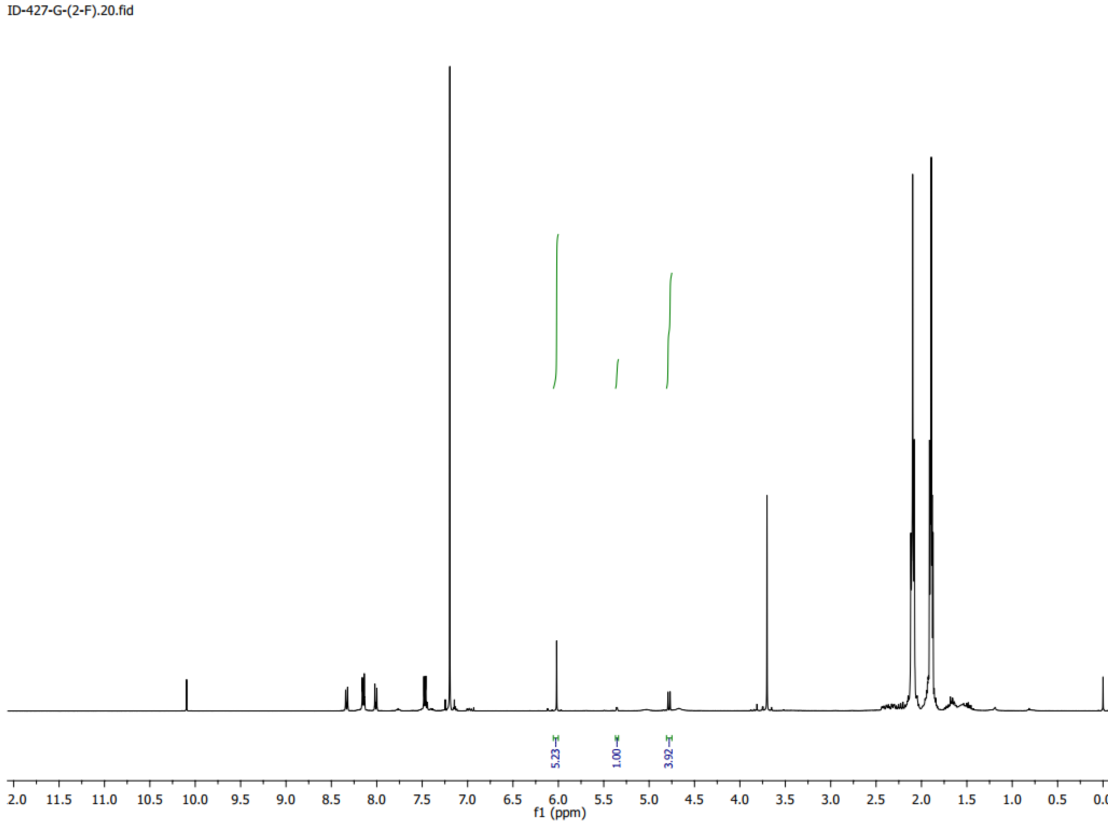

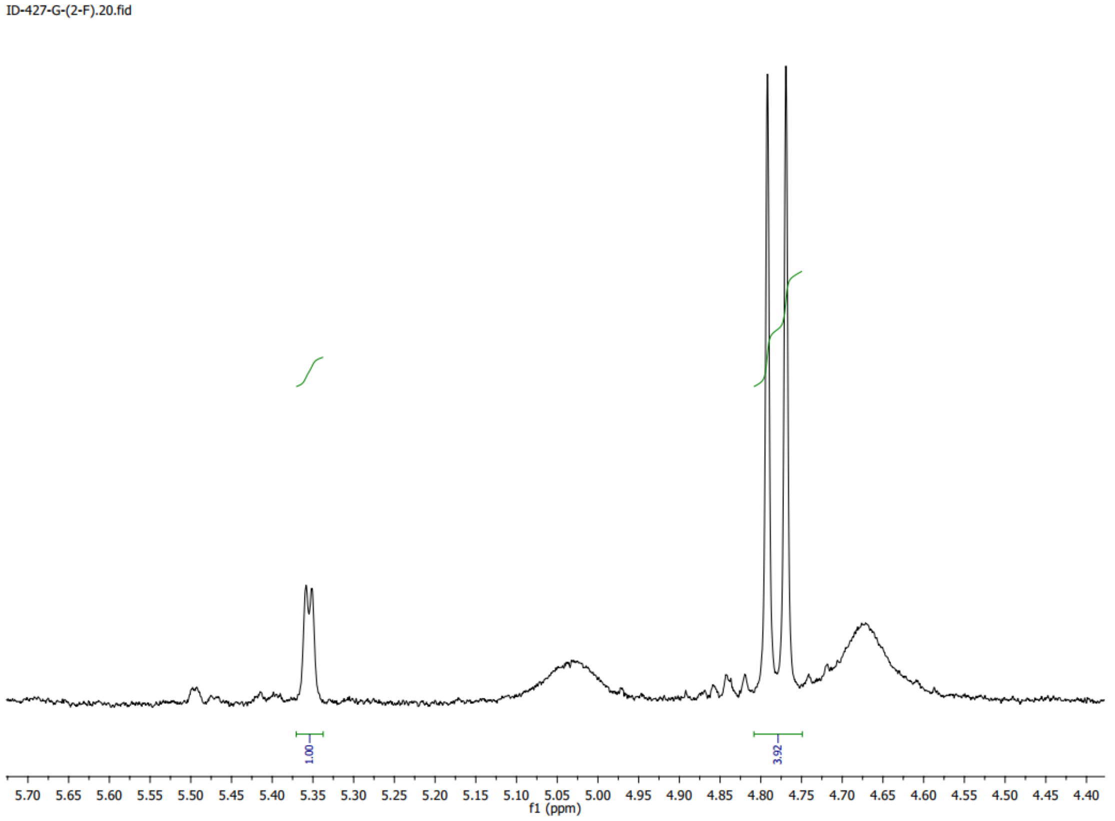

NMR traces for reaction **without boronic acid**: **(400 MHz,** $CDCl_3$**)**

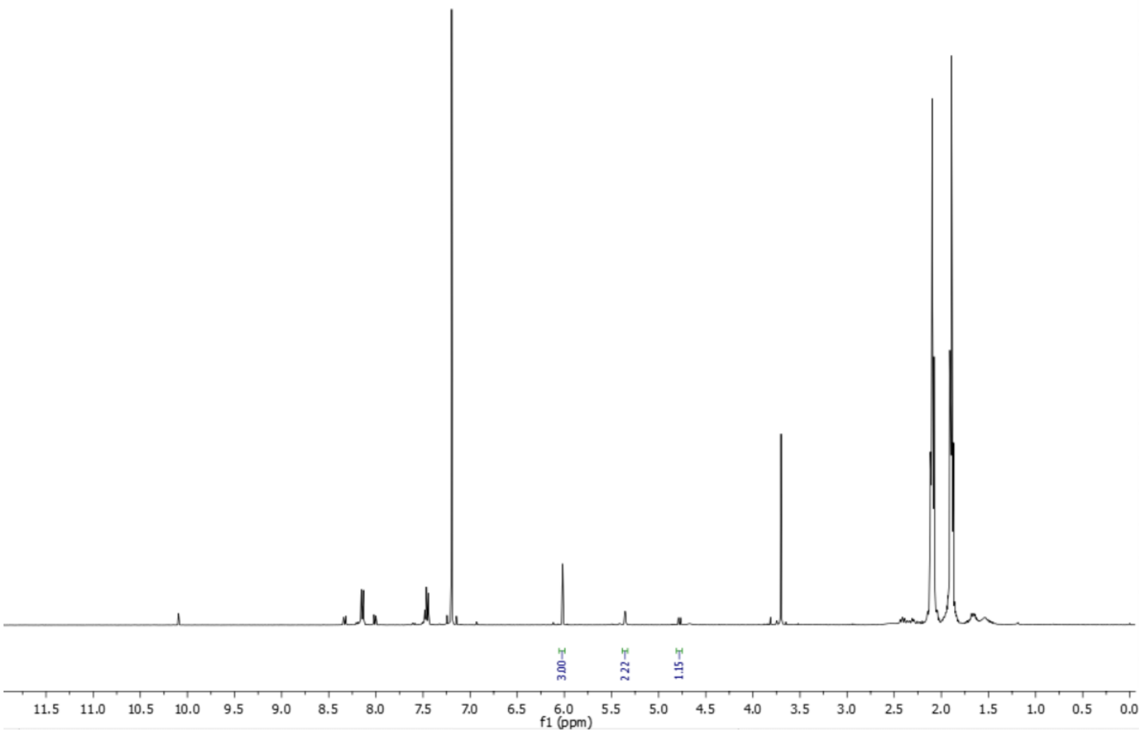

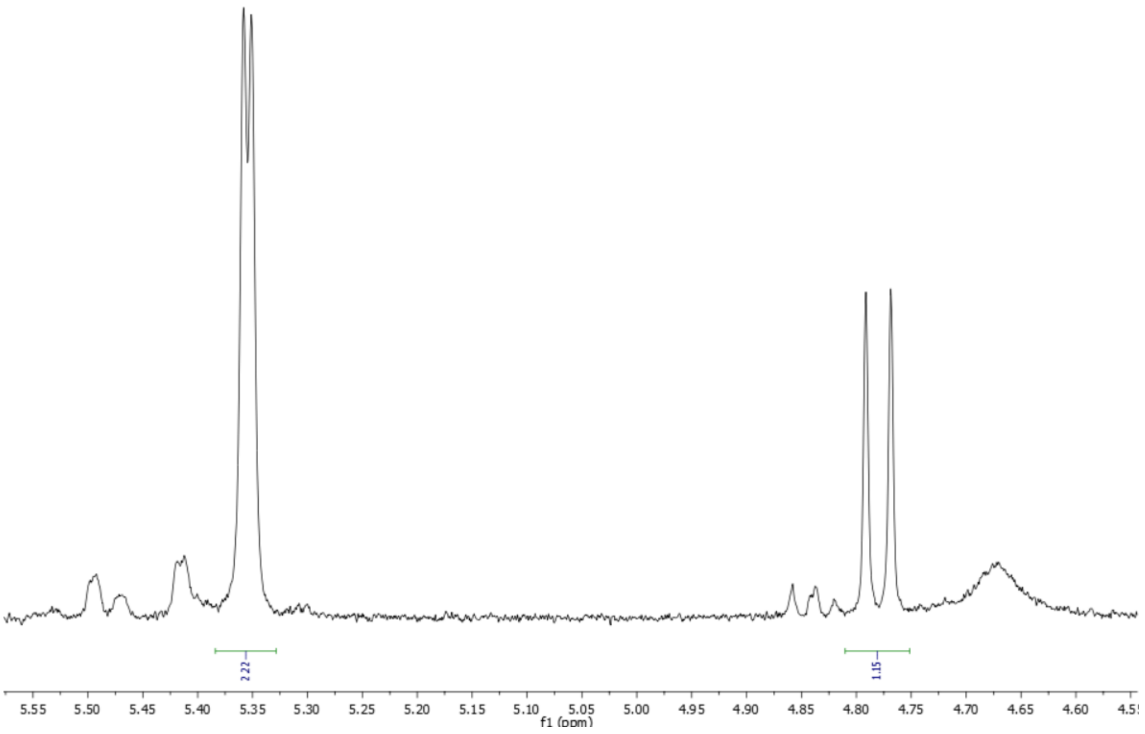

NMR traces for reaction **without boronic acid (duplicate) (400 MHz,** $CDCl_3$**)**

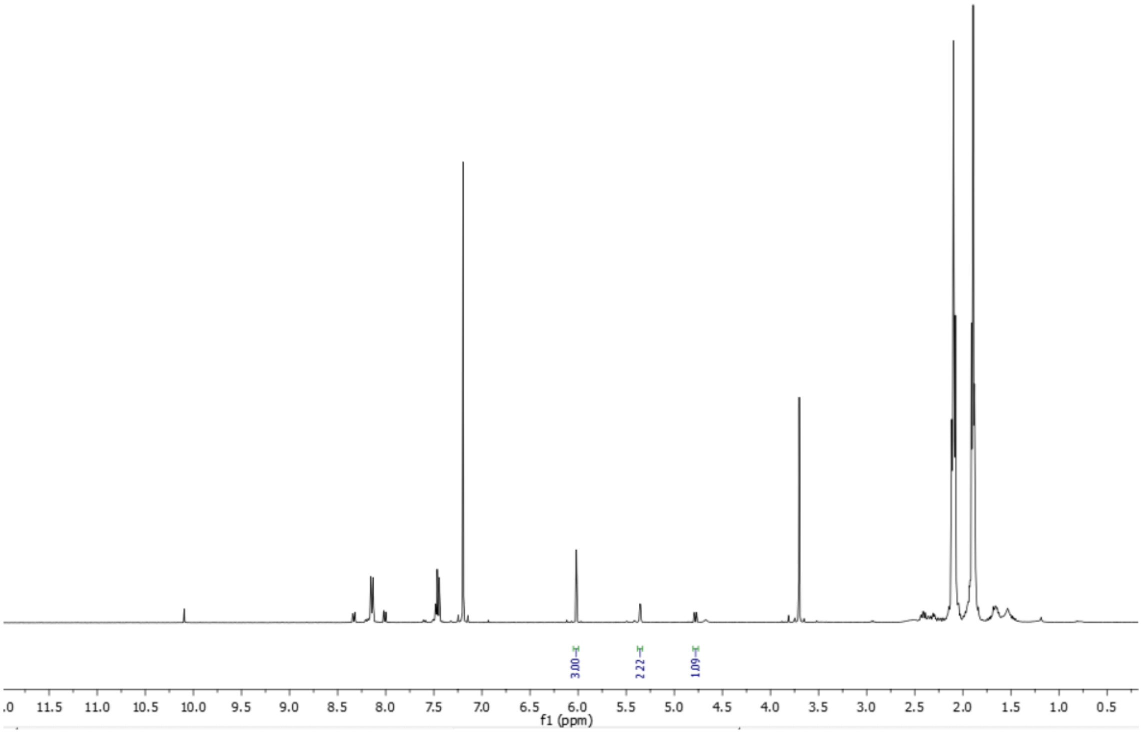

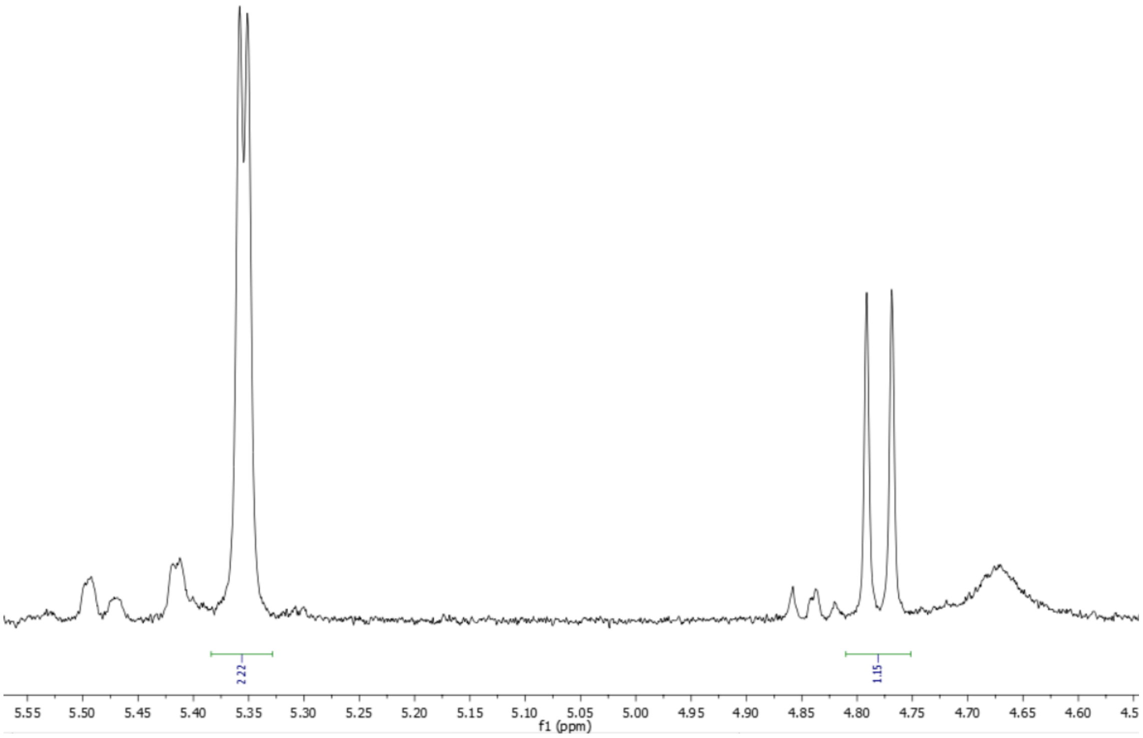

NMR traces for reaction **without water, with 2-F-phenylboronic acid (400 MHz,** $CDCl_3$**)**:

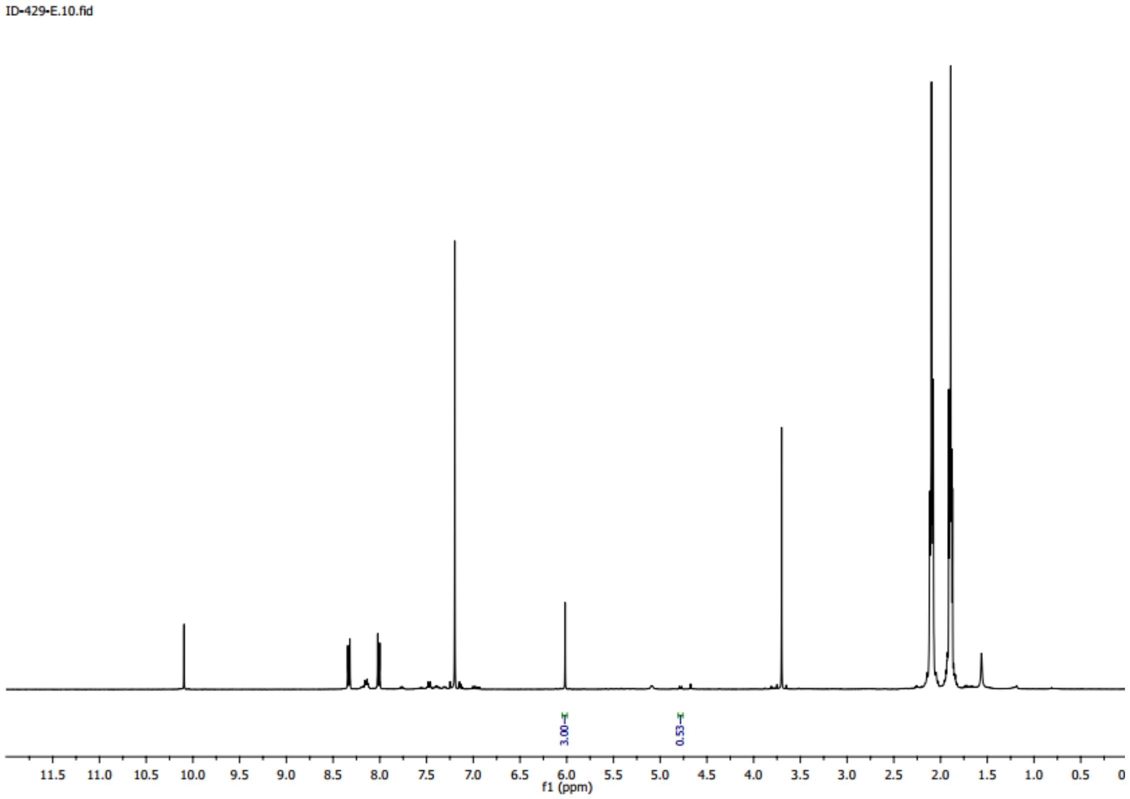

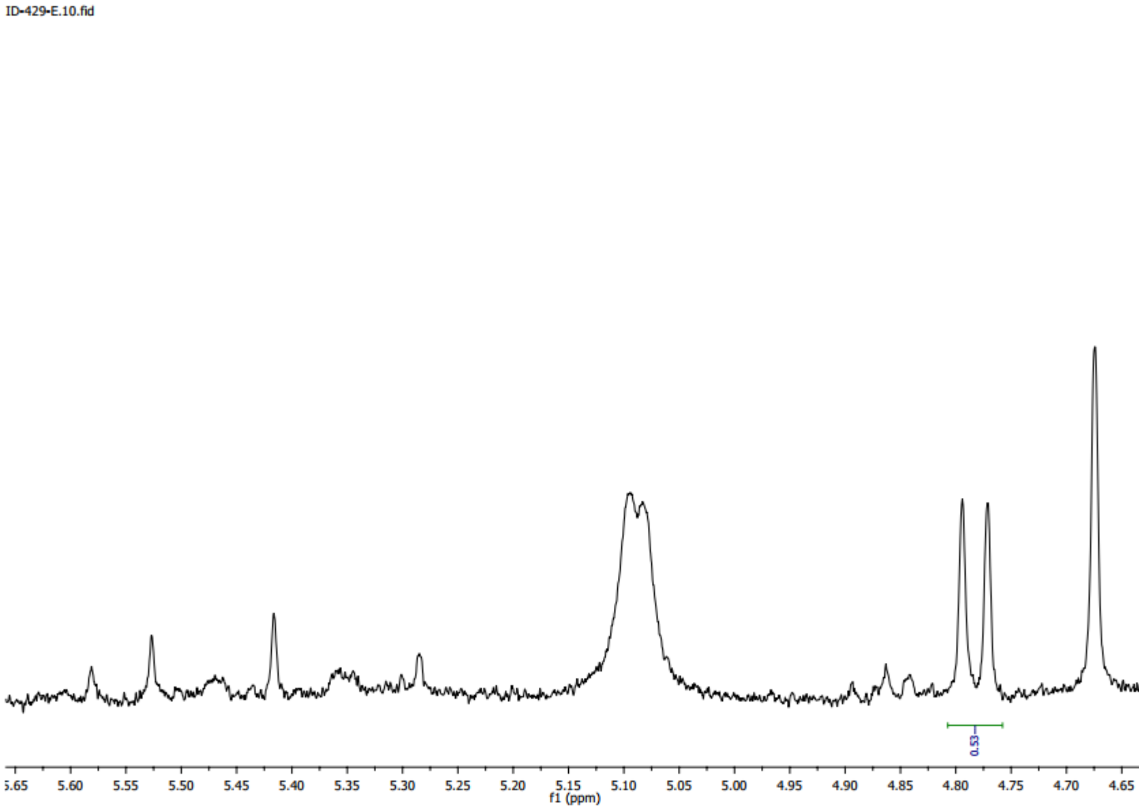

NMR traces for reaction **without water, with 2-F-phenylboronic acid (duplicate) (400 MHz,** CDCl$_3$**)**:

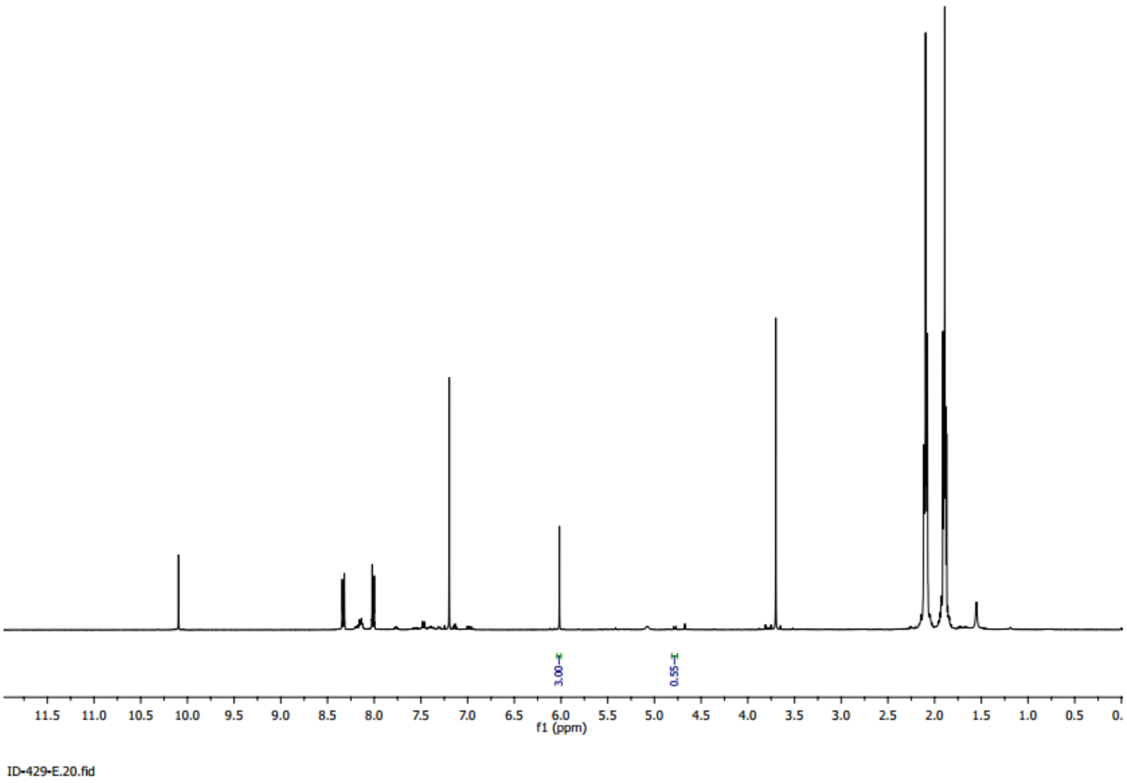

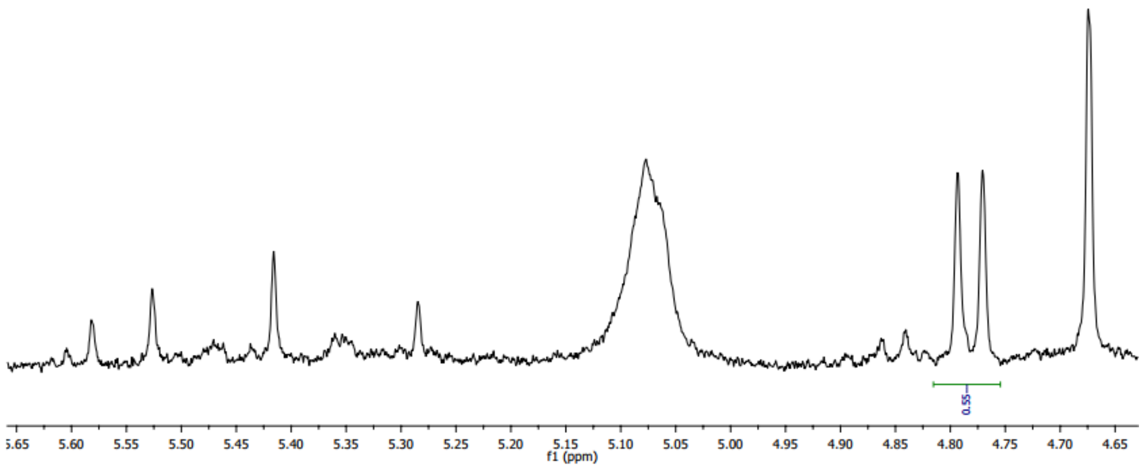

NMR traces for reaction **without boronic acid and without Proline (400 MHz,** $CDCl_3$**)**

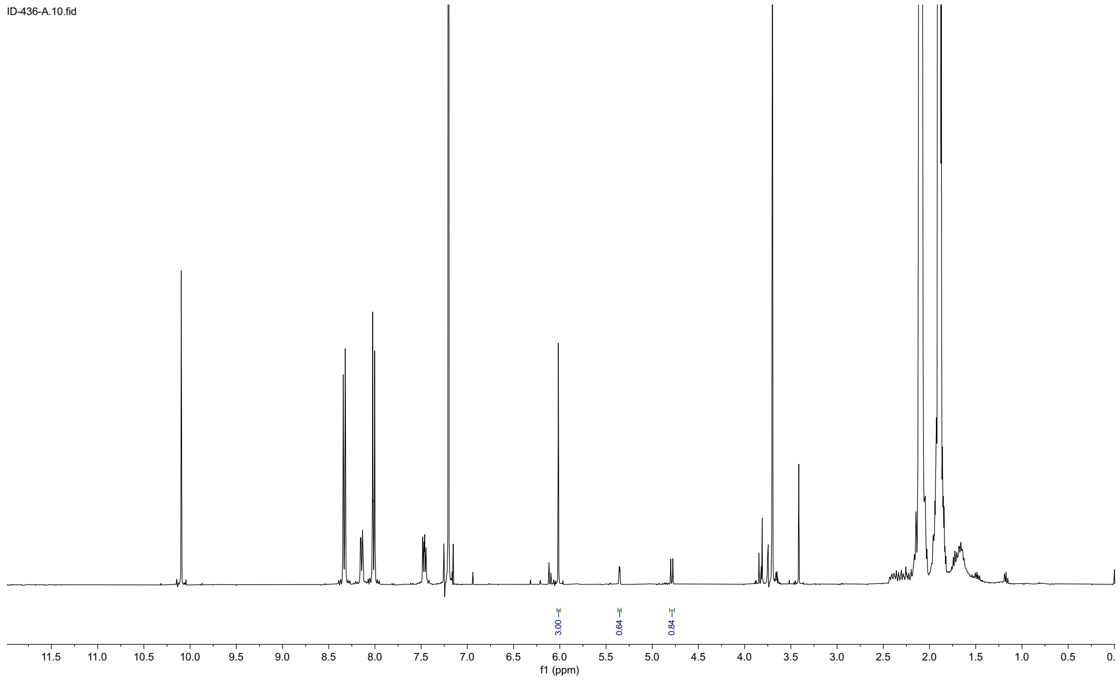

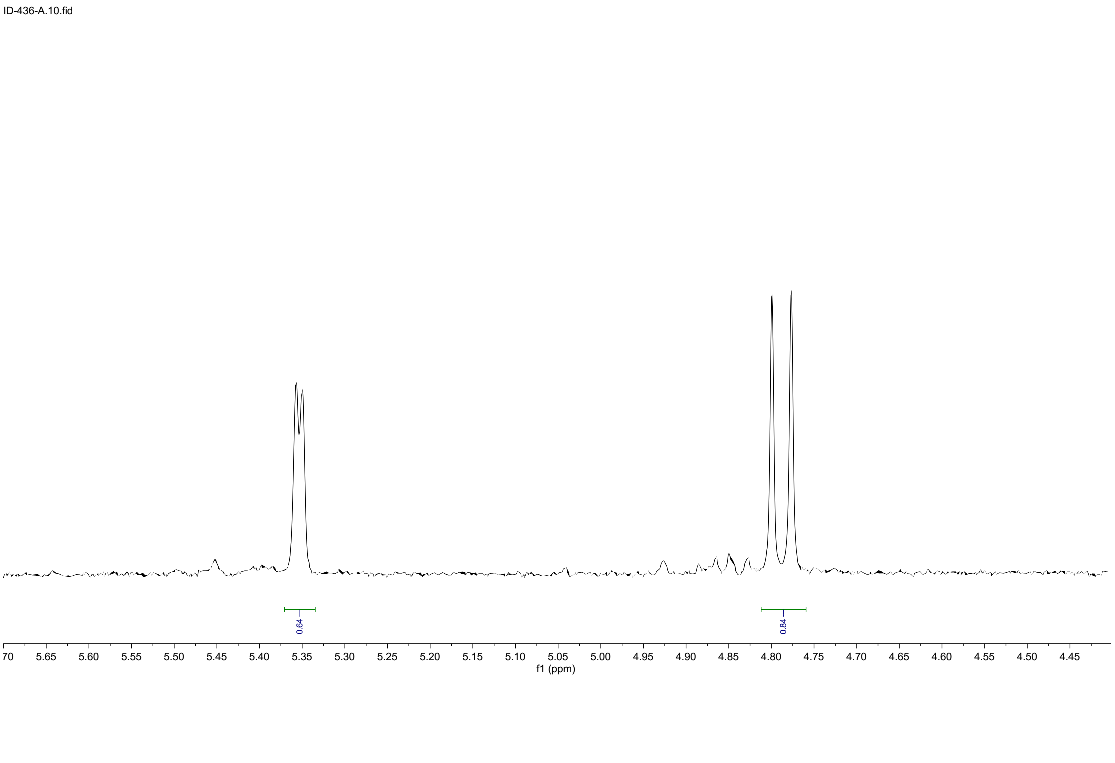

NMR traces for reaction **without boronic acid and without Proline (duplicate) (400 MHz,** $CDCl_3$**):**

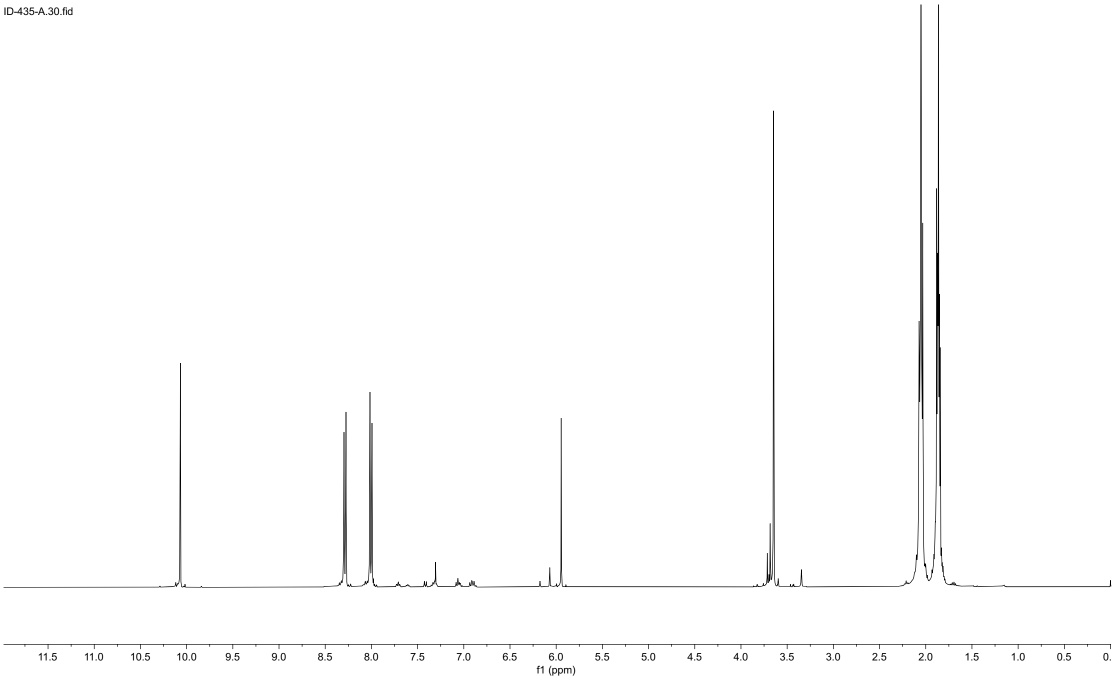

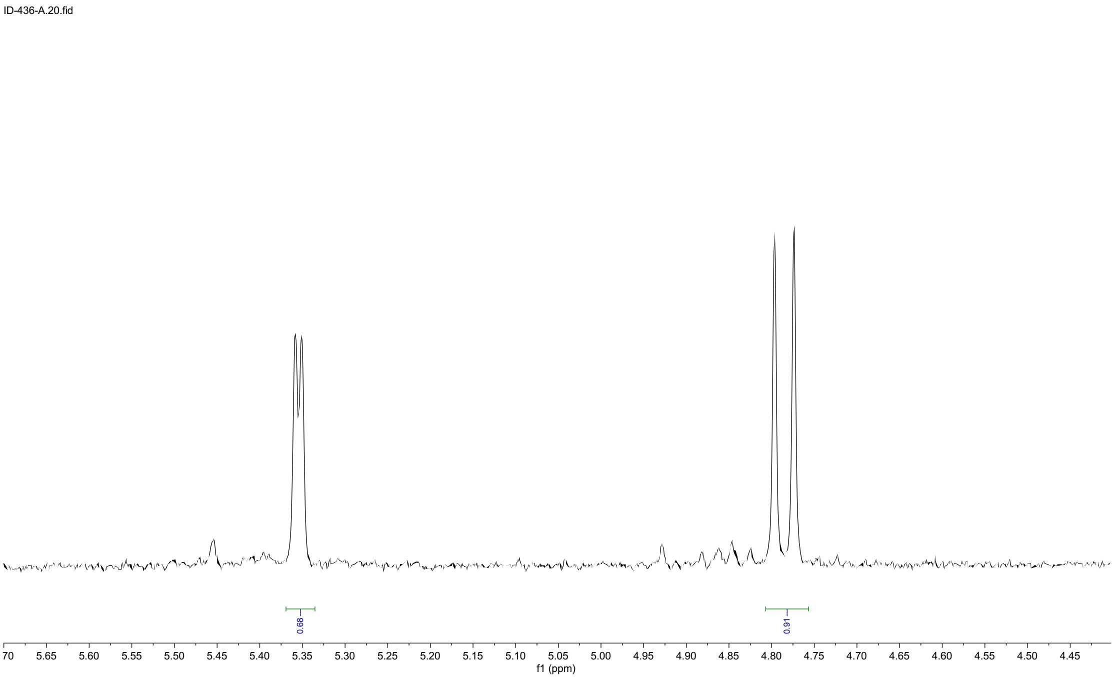

<u>18.6. NMR Traces for Table S6. Reducing amount of ketone with 3,5-F-phenyl boronic acid</u>

3,5-F-phenylboronic acid (6.8 eq cyclopentanone,3 hours reaction) (400 MHz, $CDCl_3$**)**

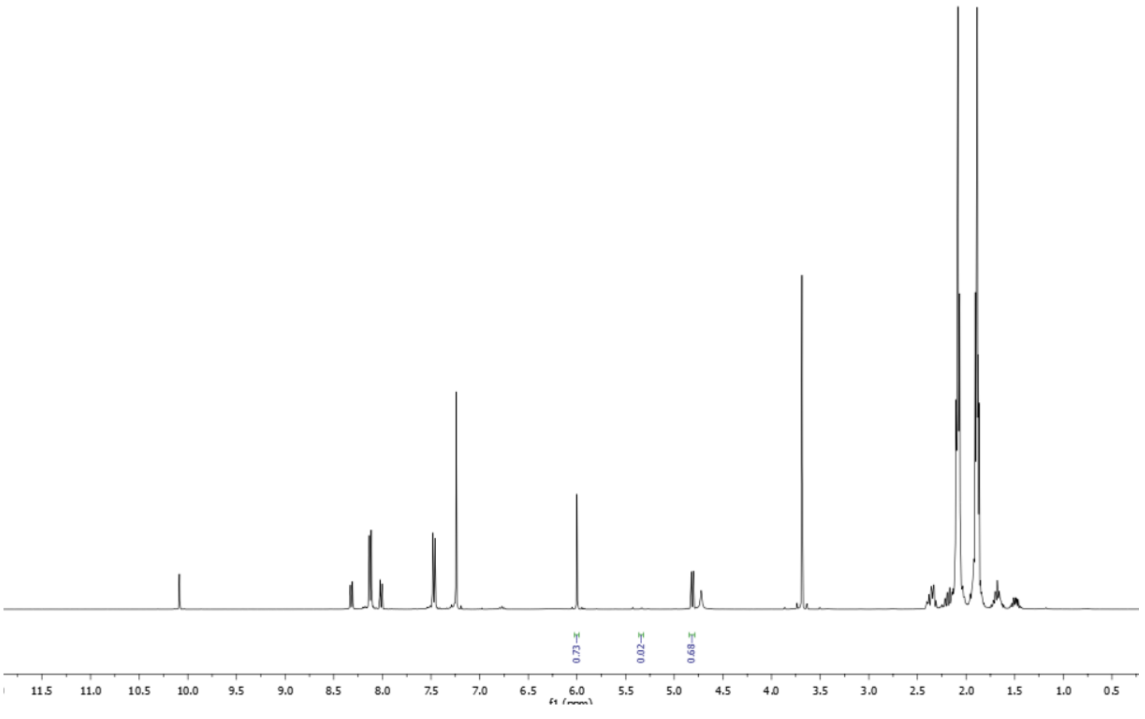

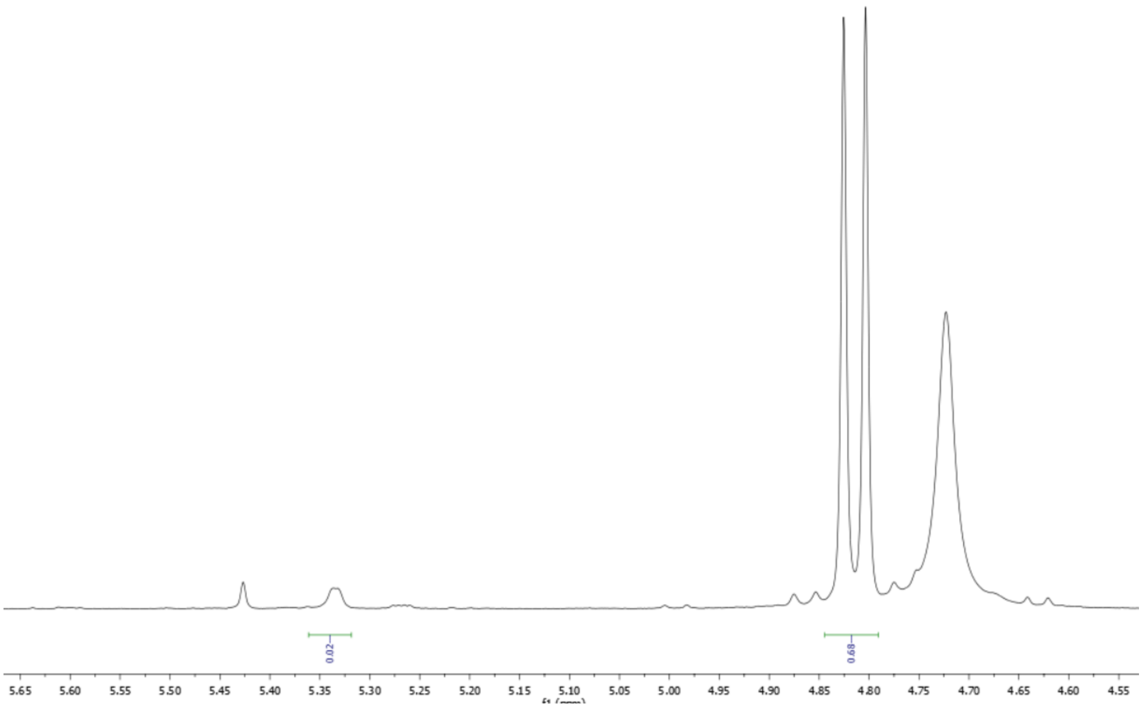

3,5-F-phenylboronic acid (6.8 eq cyclopentanone,3 hours reaction) - duplicate (400 MHz, CDCl$_3$**)**

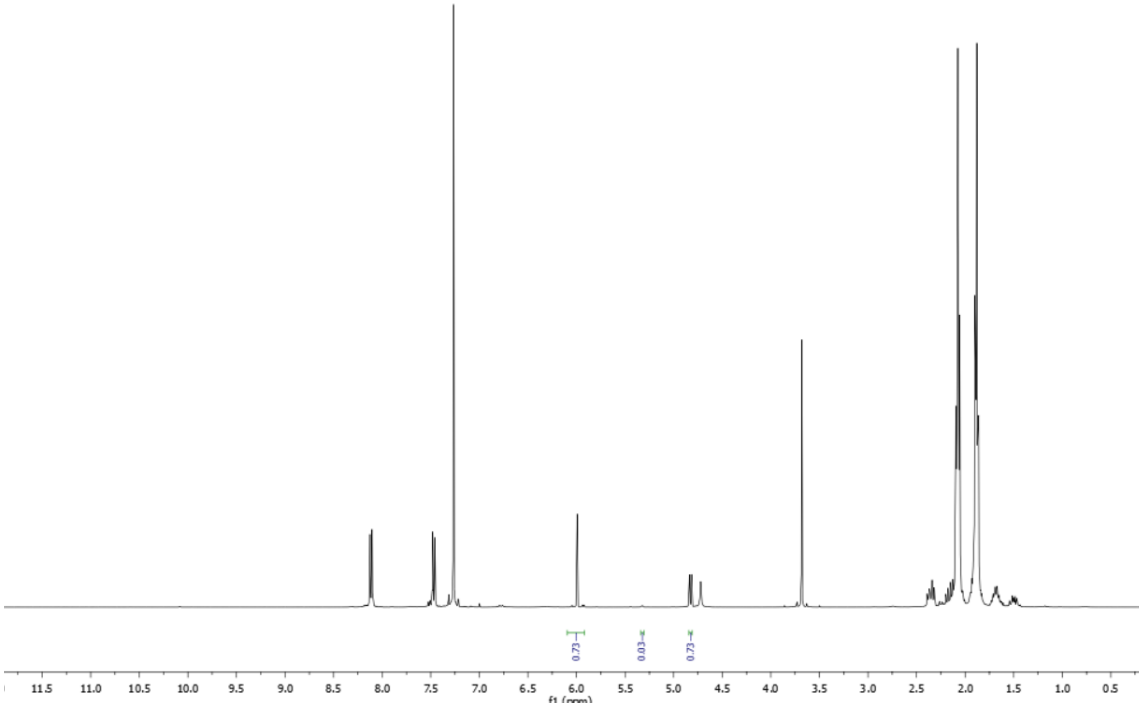

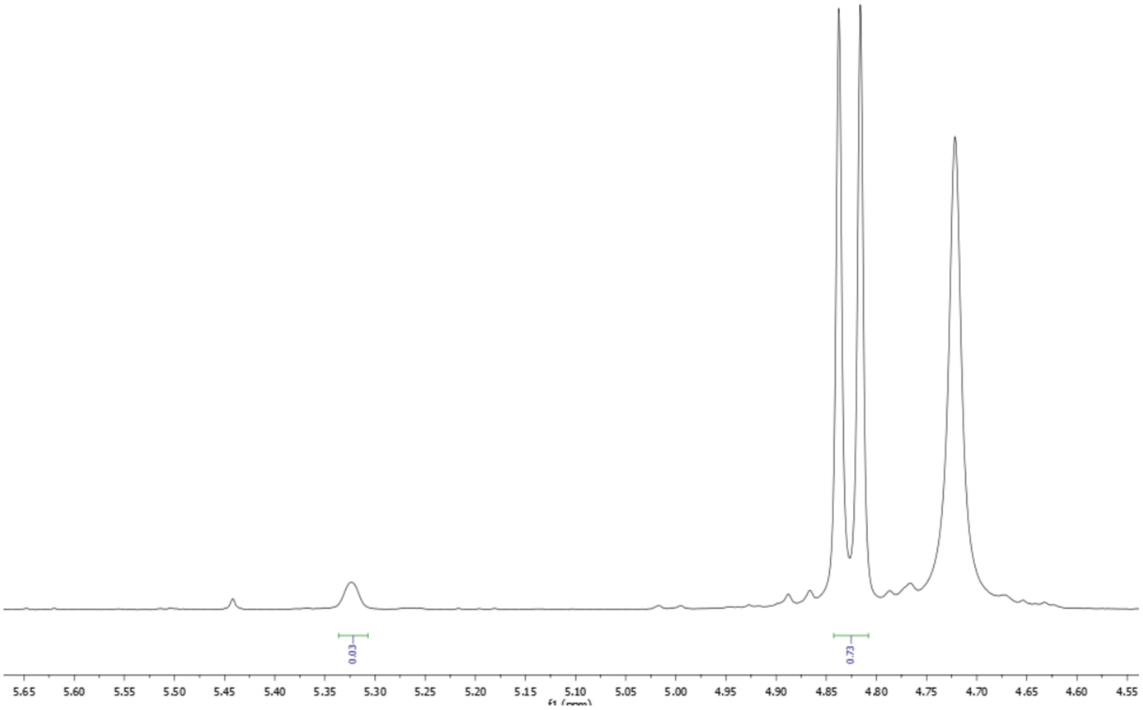

3,5-F-phenylboronic acid (6.8 eq cyclopentanone,6 hours reaction) (400 MHz, CDCl$_3$)

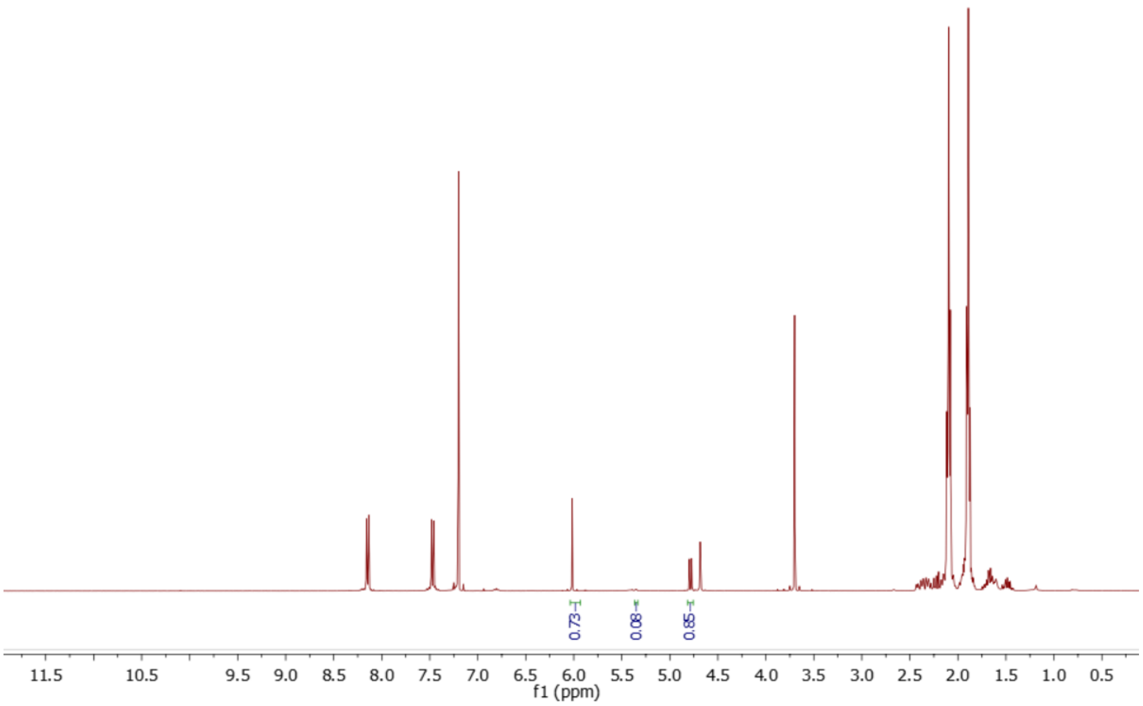

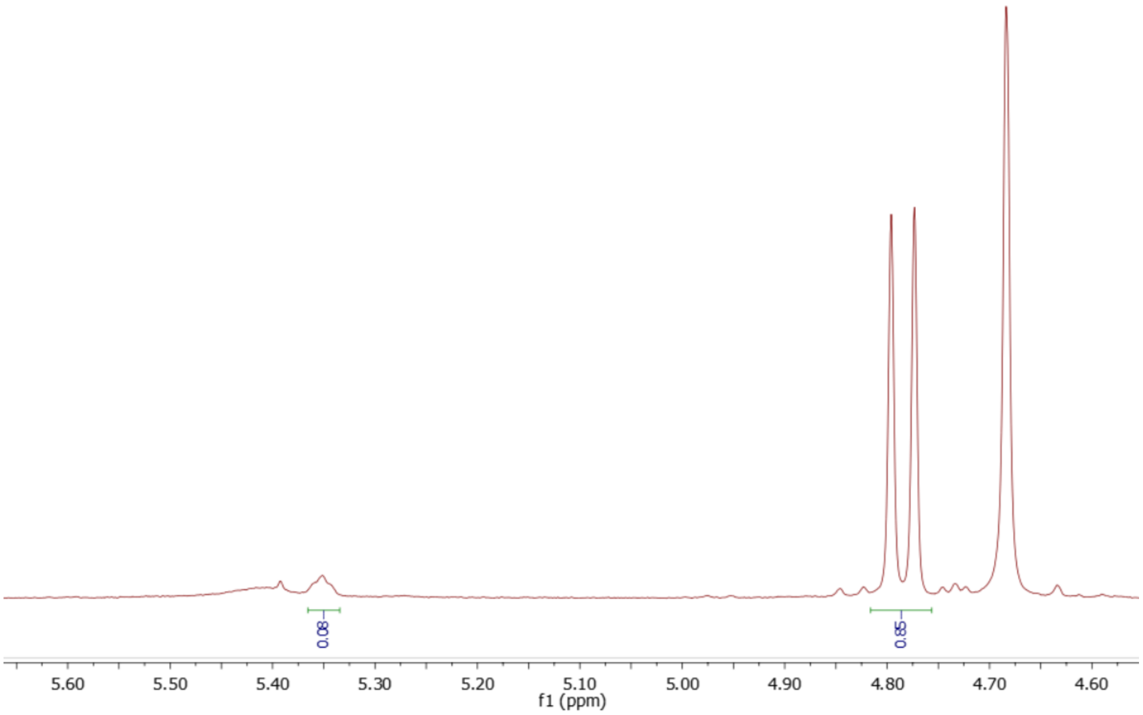

3,5-F-phenylboronic acid (6.8 eq cyclopentanone,6 hours reaction) - duplicate (400 MHz, $CDCl_3$**)**

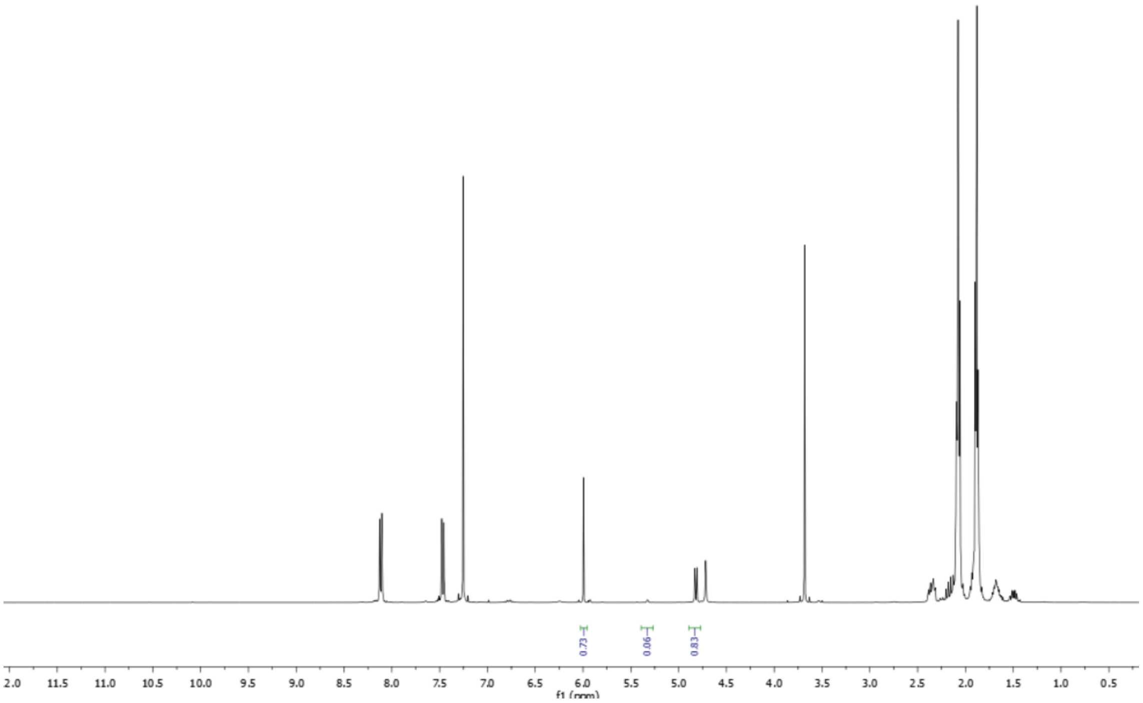

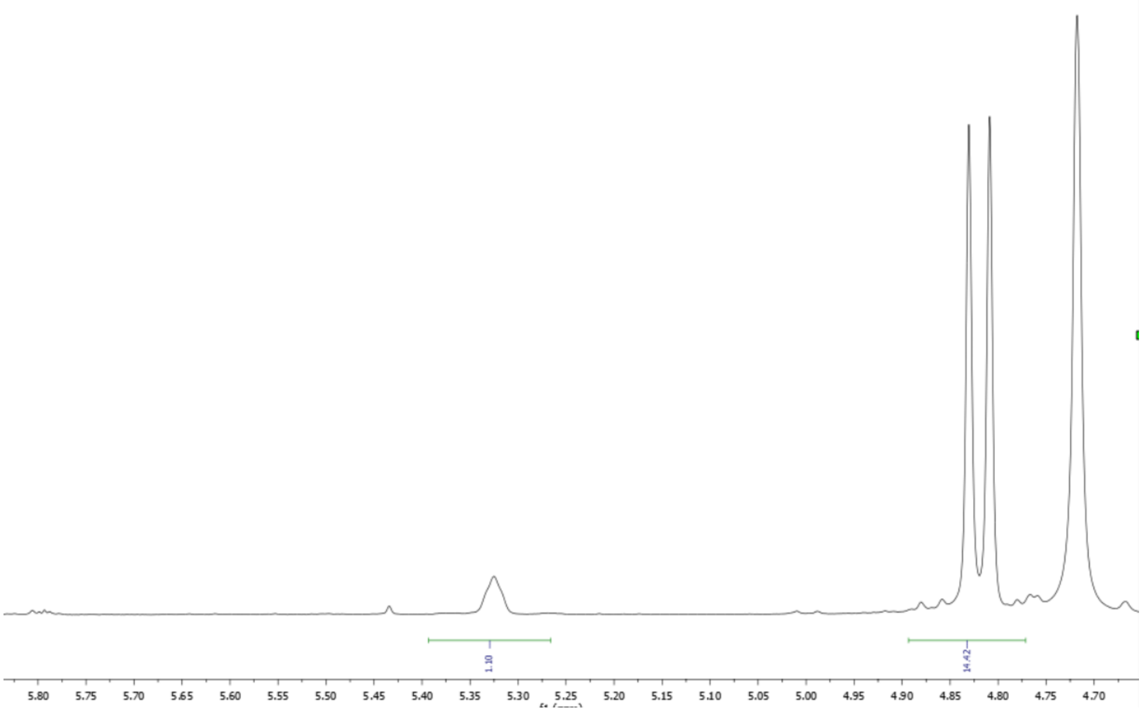

3,5-F-phenylboronic acid (5 eq cyclopentanone,9 hours reaction) (400 MHz, CDCl$_3$**)**

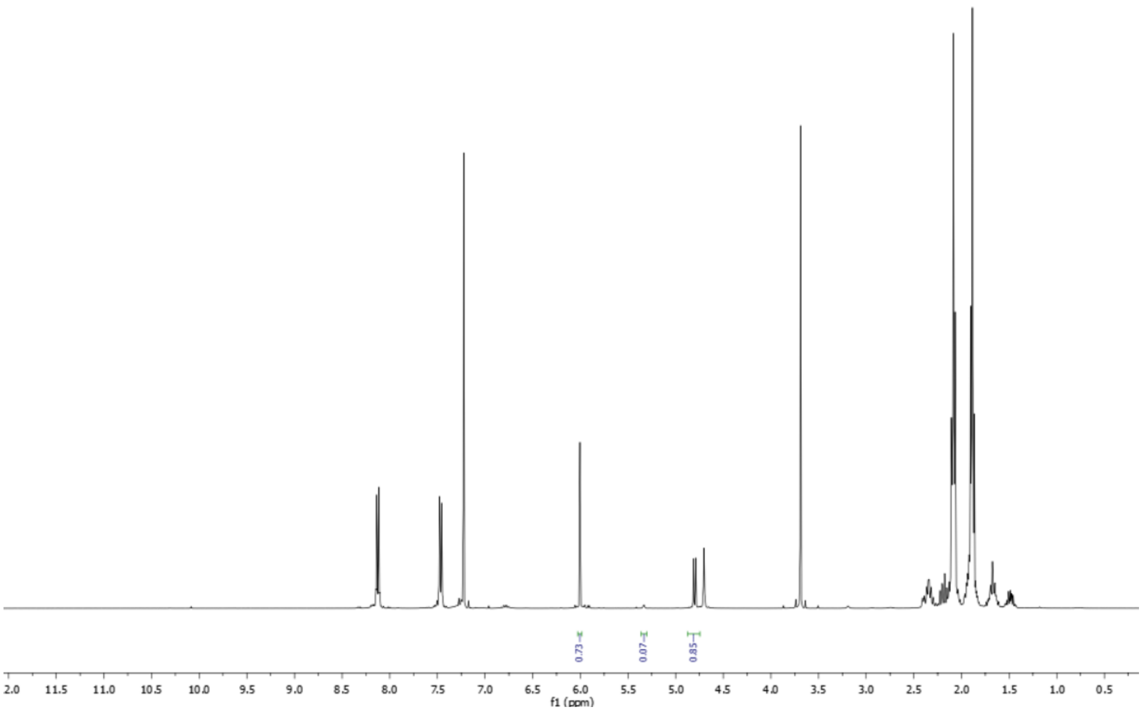

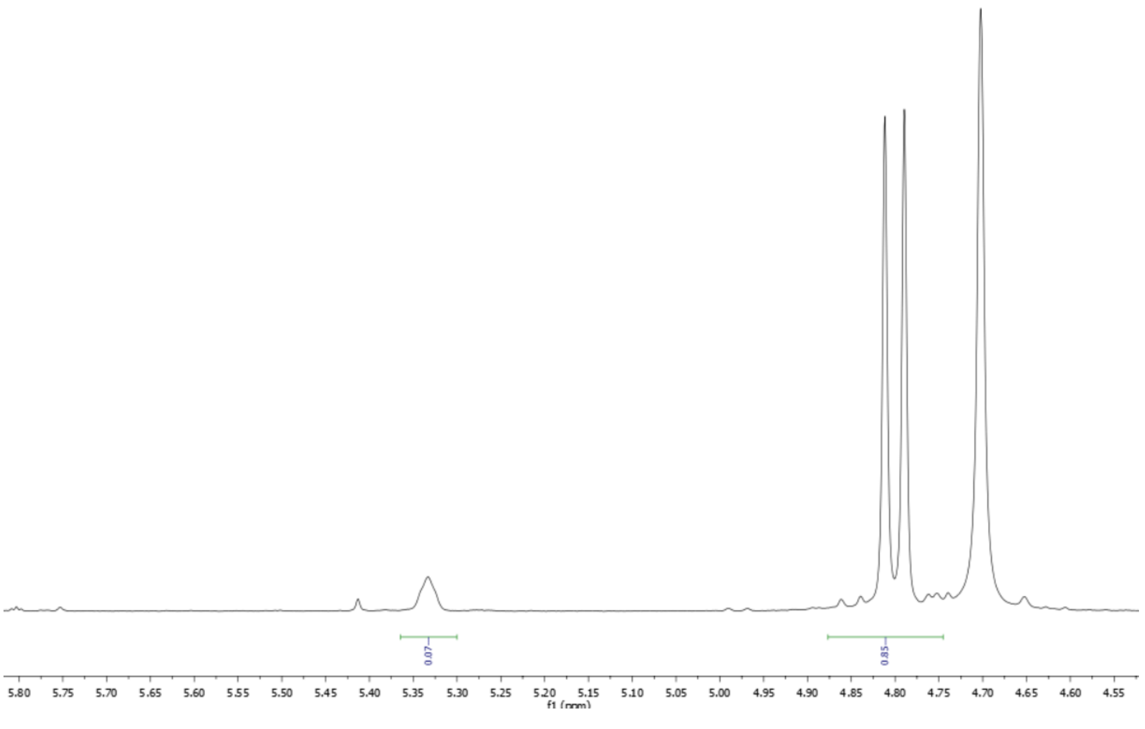

3,5-F-phenylboronic acid (5 eq cyclopentanone,9 hours reaction – duplicate) (400 MHz, $CDCl_3$**)**

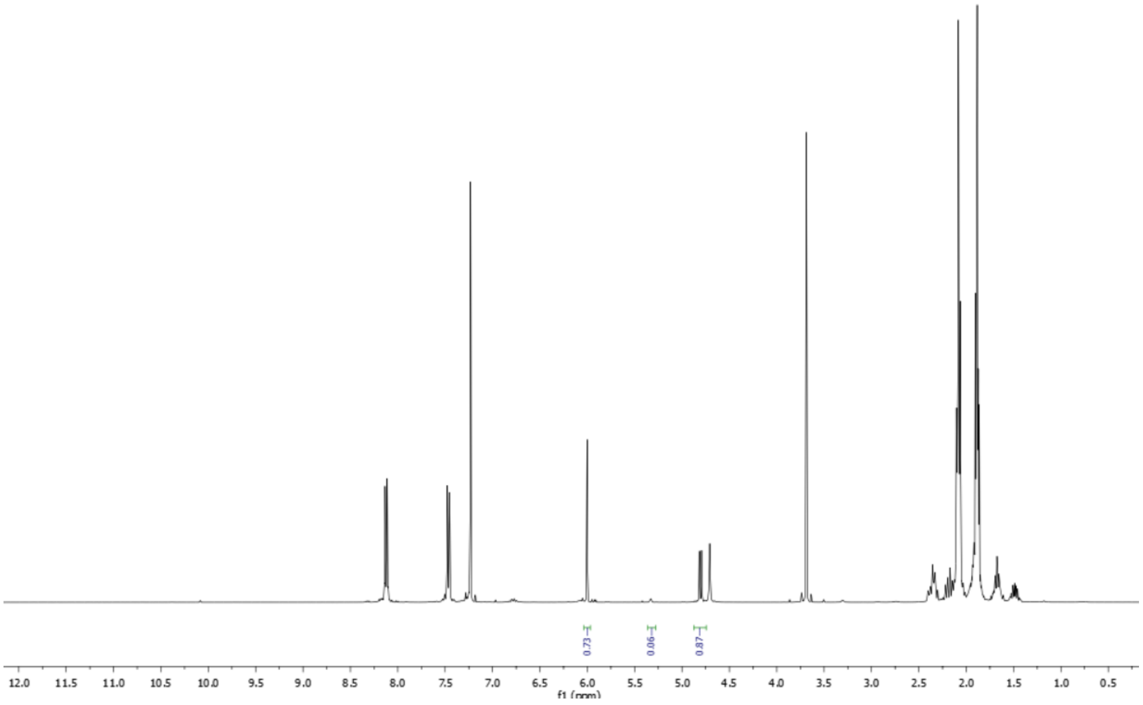

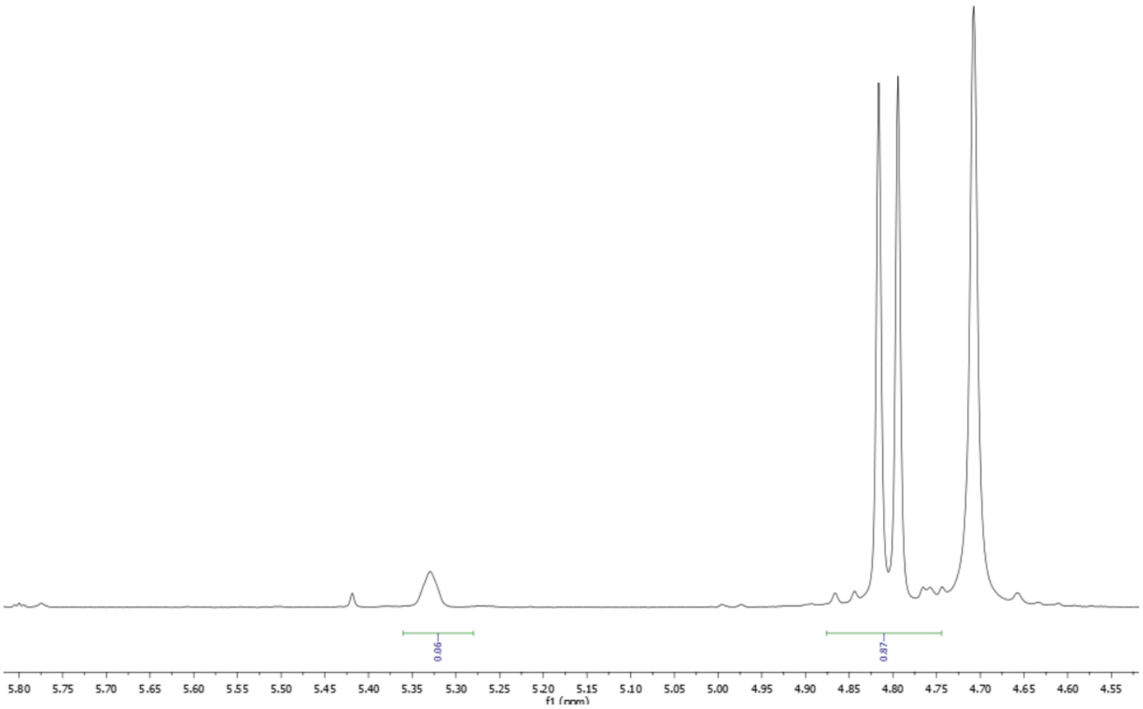

3,5-F-phenylboronic acid (3 eq cyclopentanone,9 hours reaction) (400 MHz, CDCl$_3$**)**

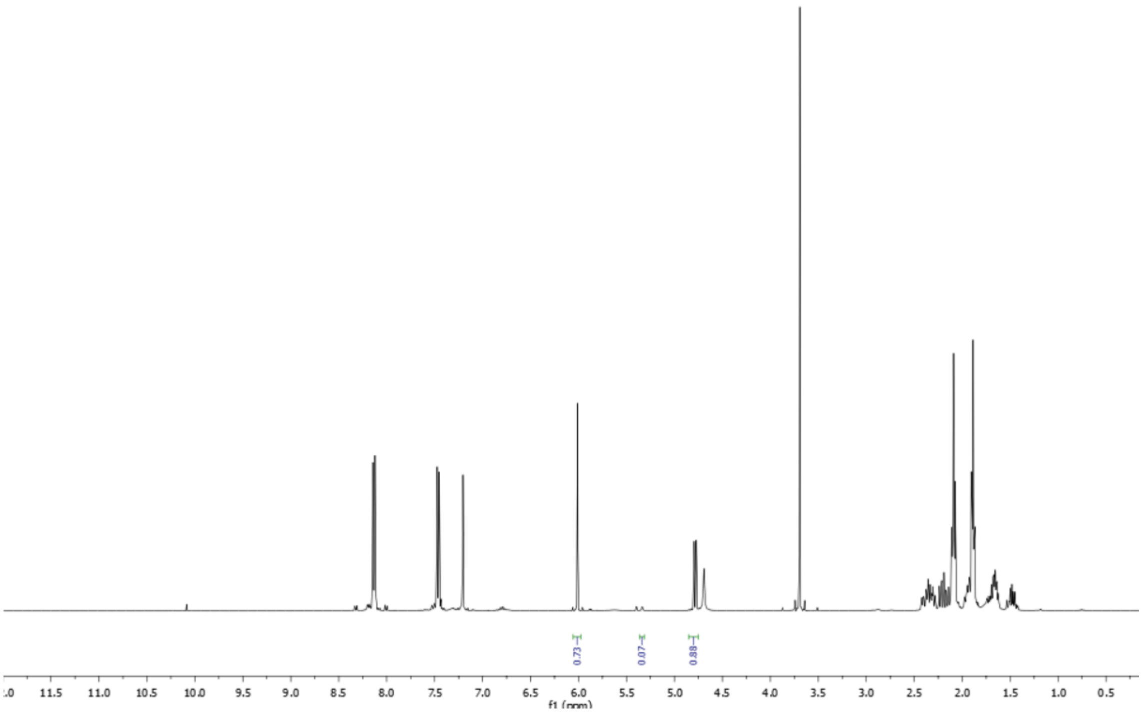

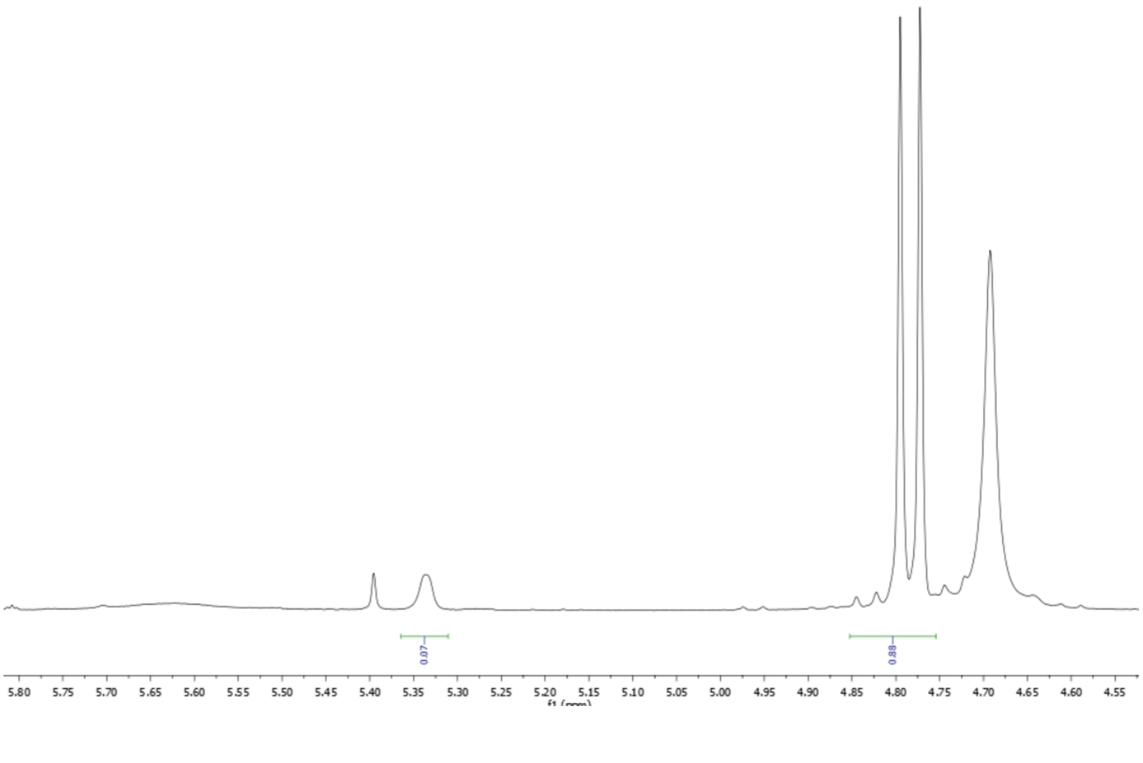

3,5-F-phenylboronic acid (3 eq cyclopentanone,9 hours reaction – duplicate) (400 MHz, $CDCl_3$**)**

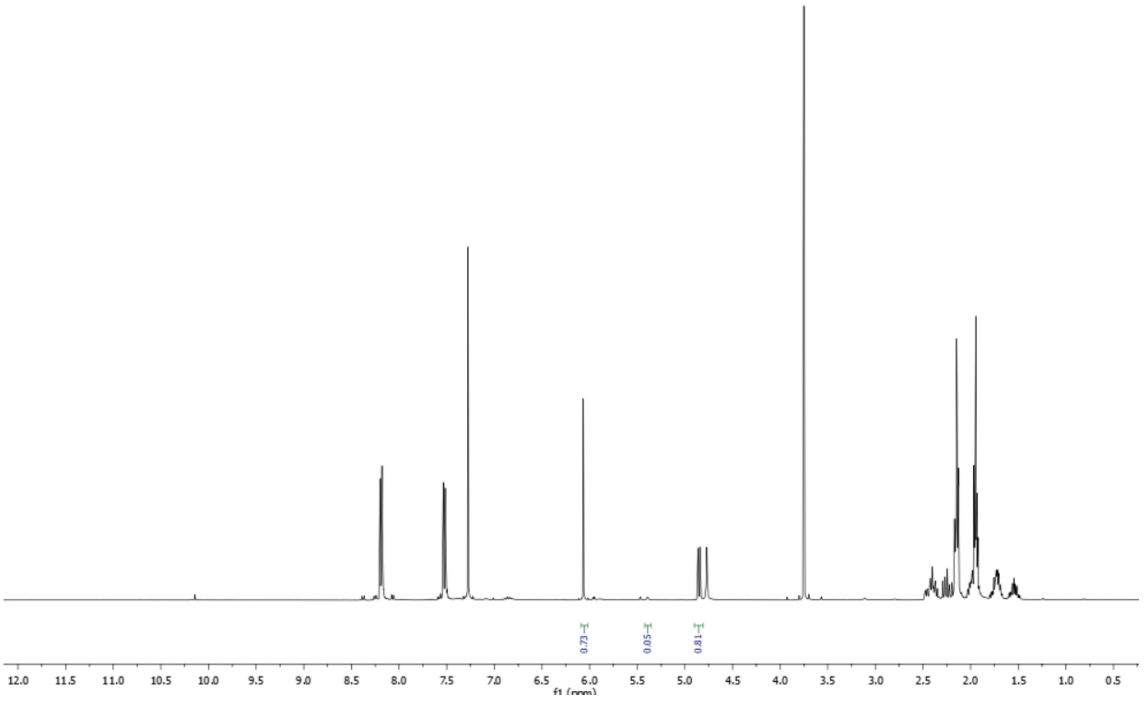

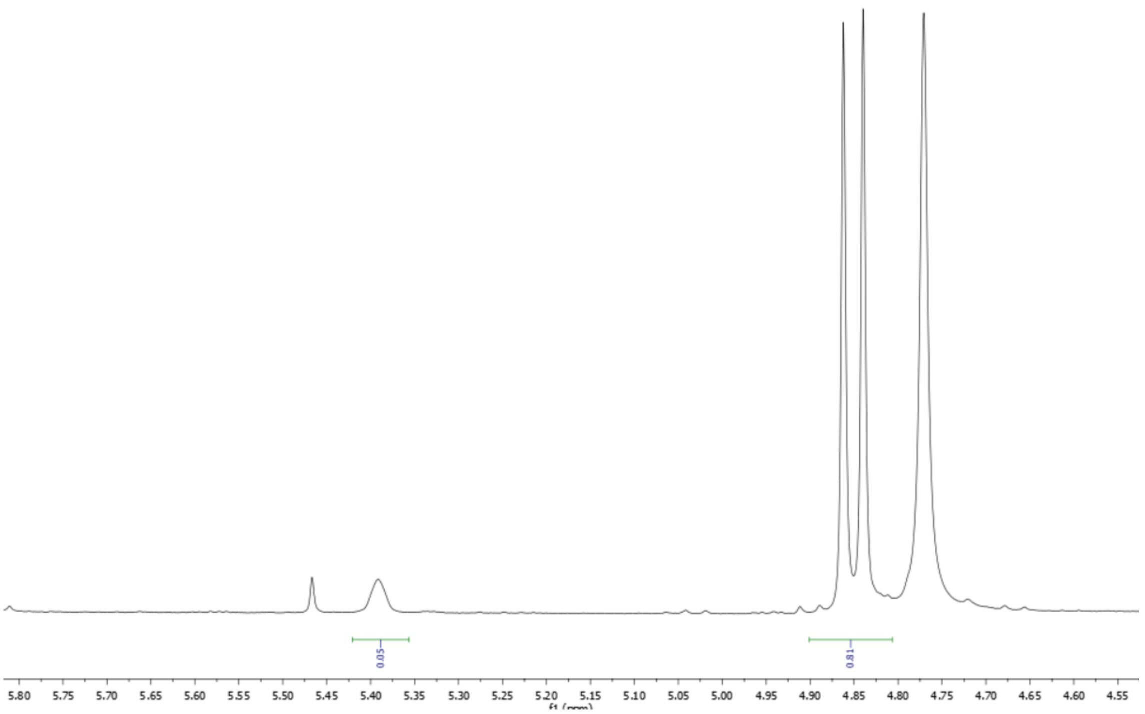

3,5-F-phenylboronic acid (2 eq cyclopentanone,9 hours reaction) (400 MHz, CDCl$_3$**)**

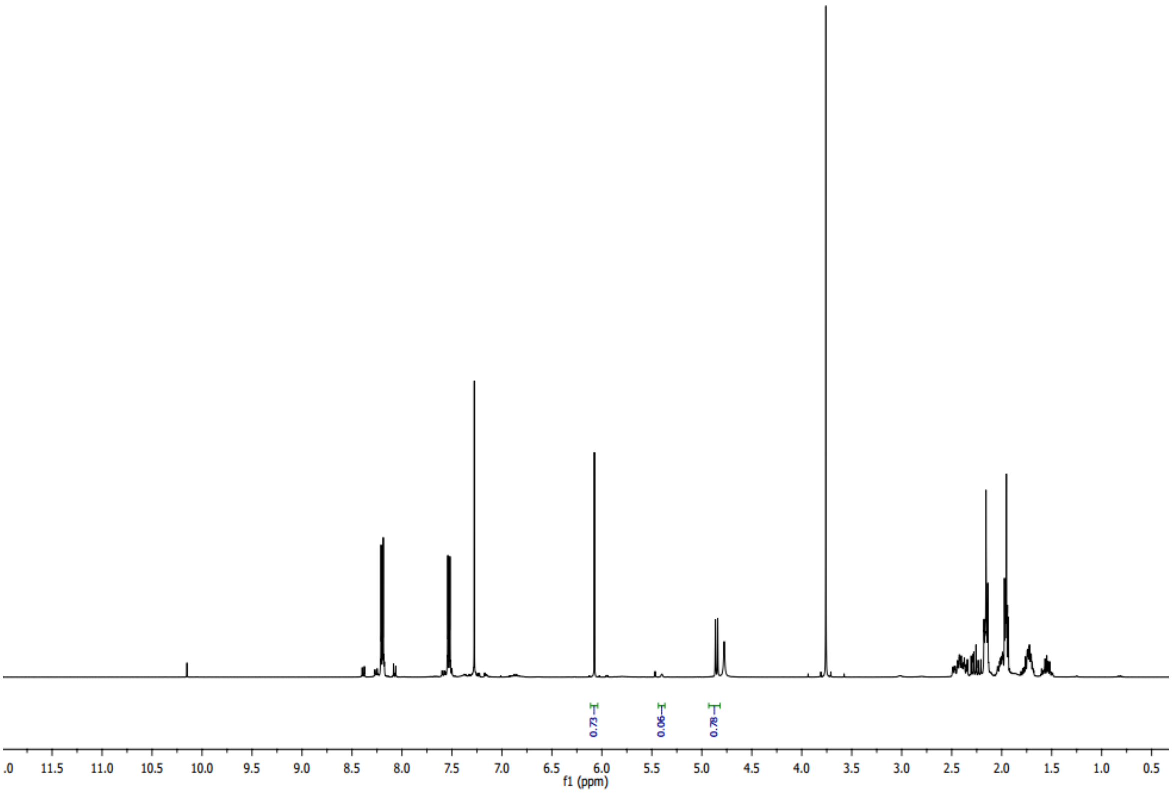

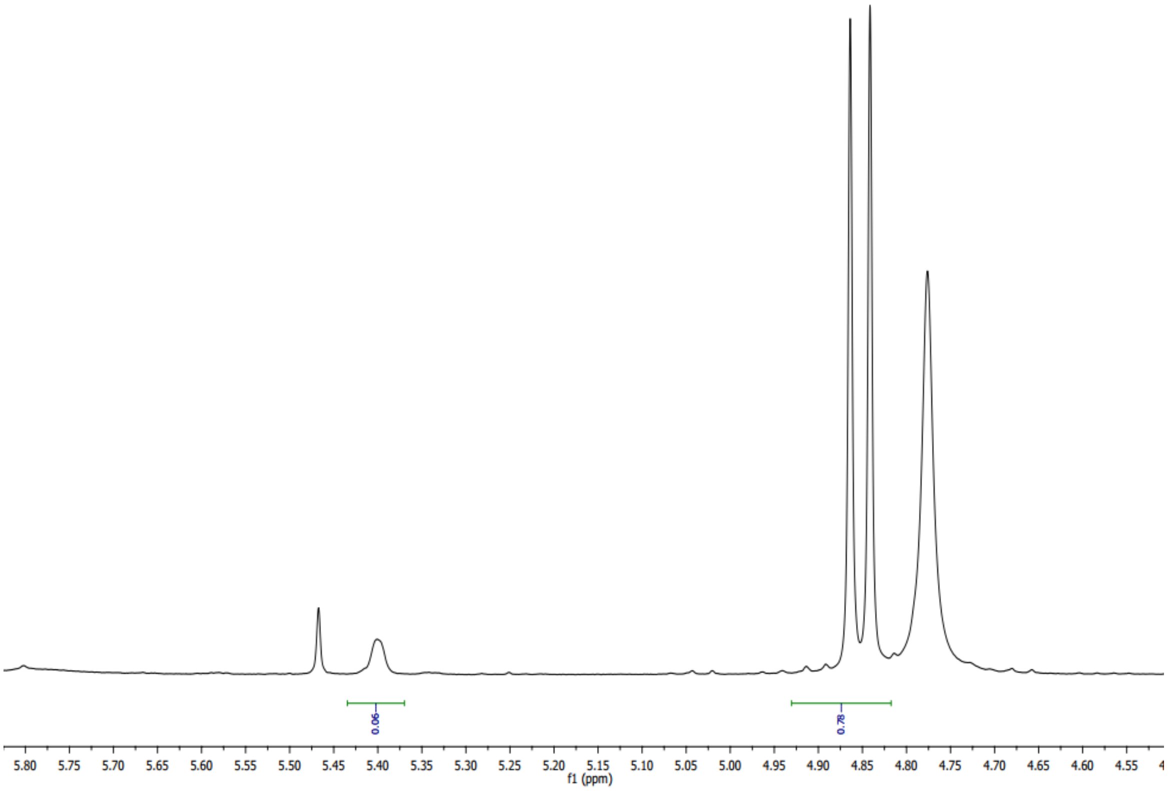

3,5-F-phenylboronic acid (2 eq cyclopentanone,9 hours reaction – duplicate) (400 MHz, $CDCl_3$**)**

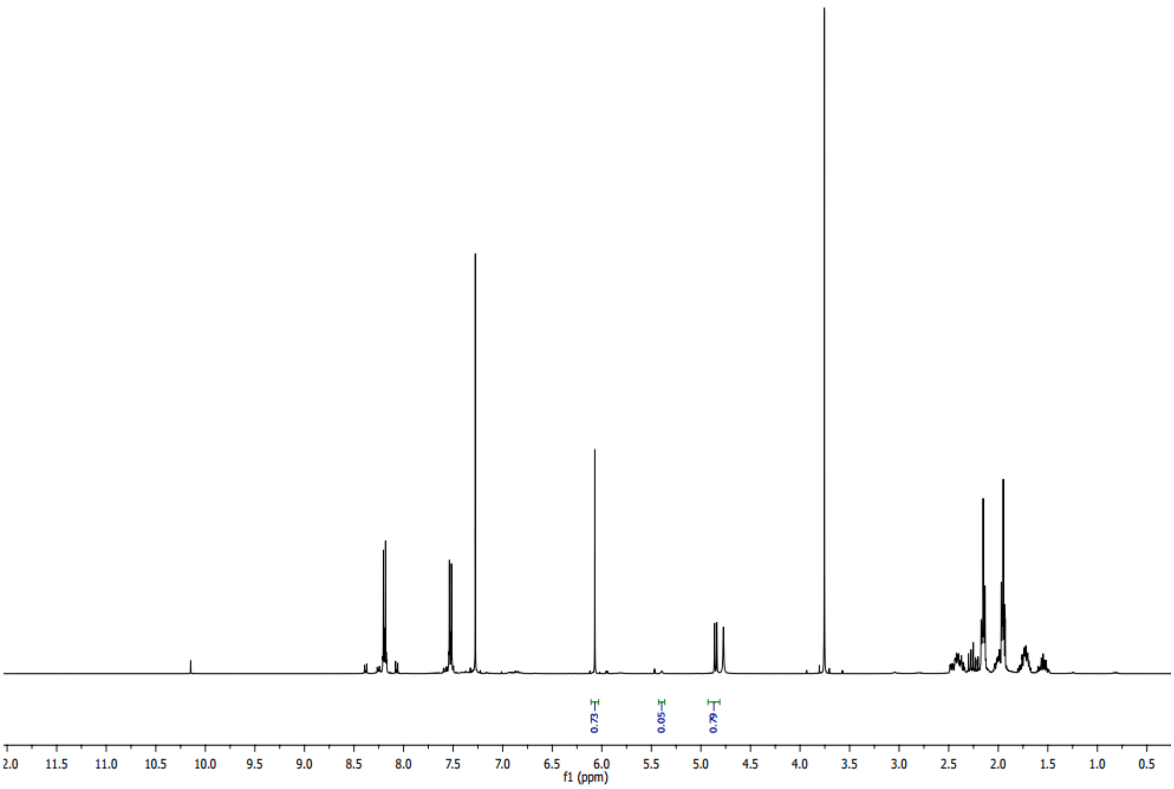

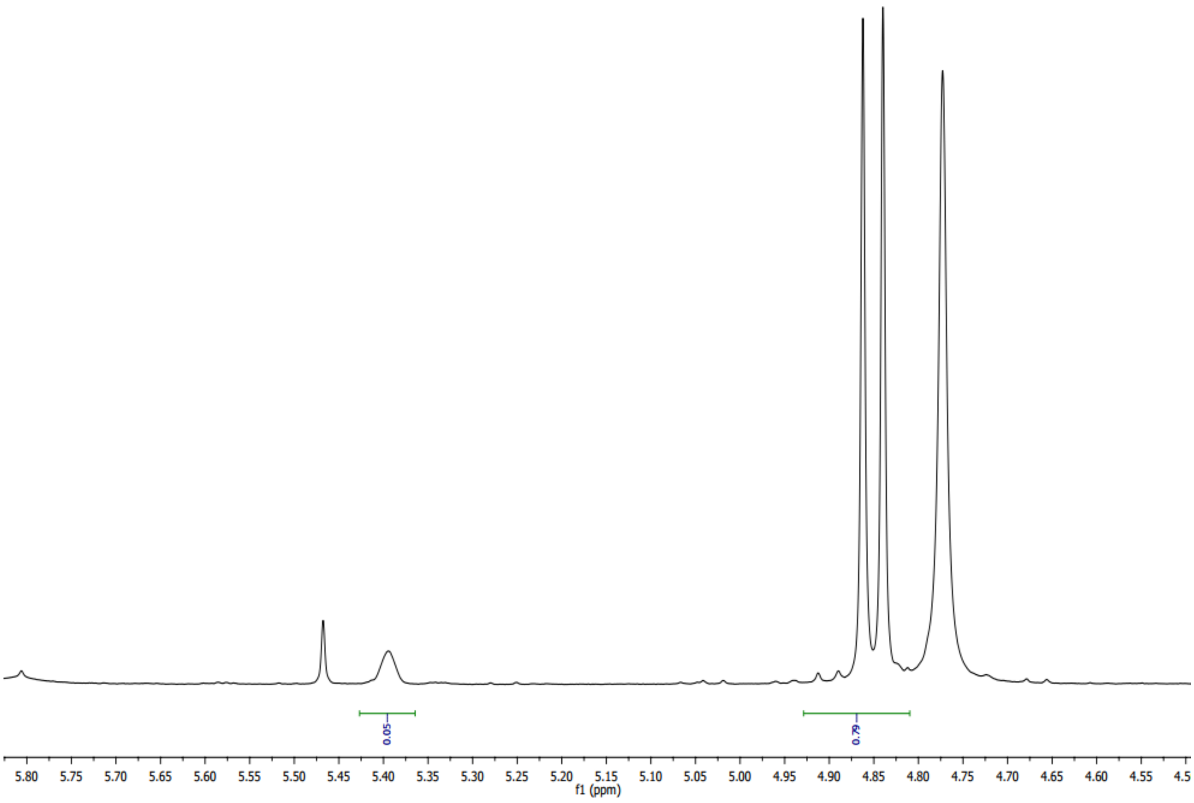

3,5-F-phenylboronic acid (1.5 eq cyclopentanone,9 hours reaction) (400 MHz, CDCl$_3$**)**

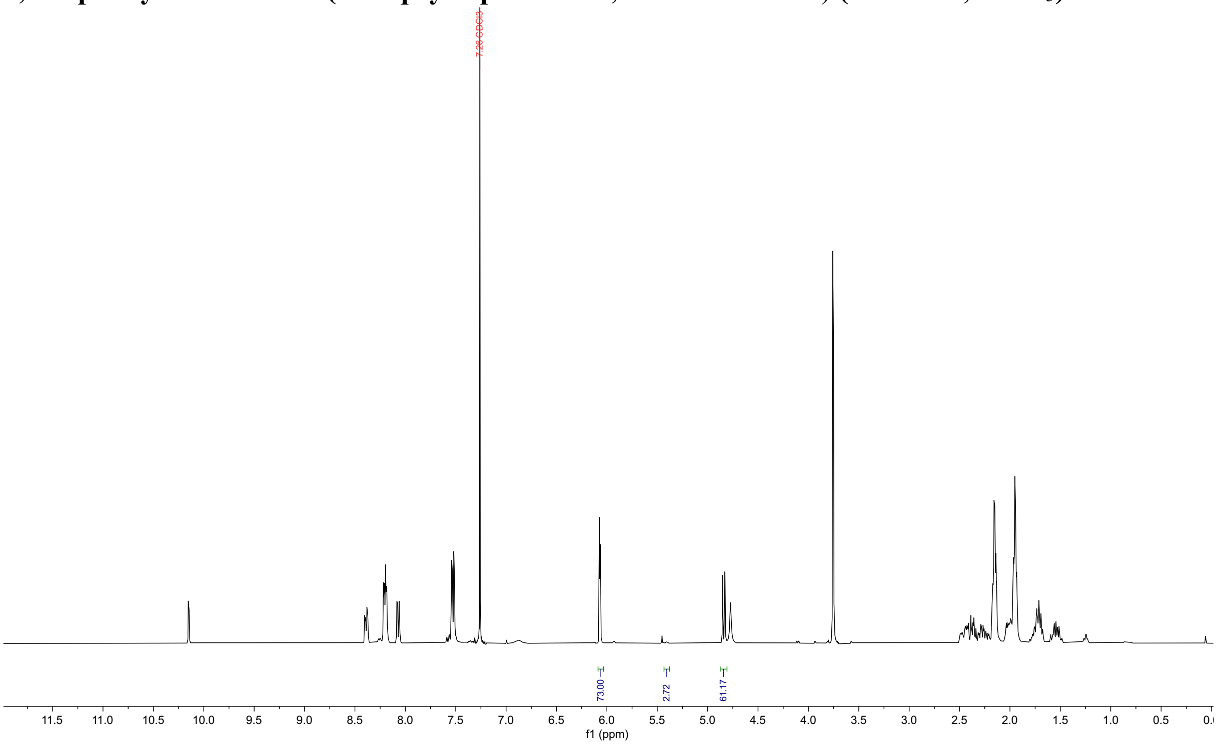

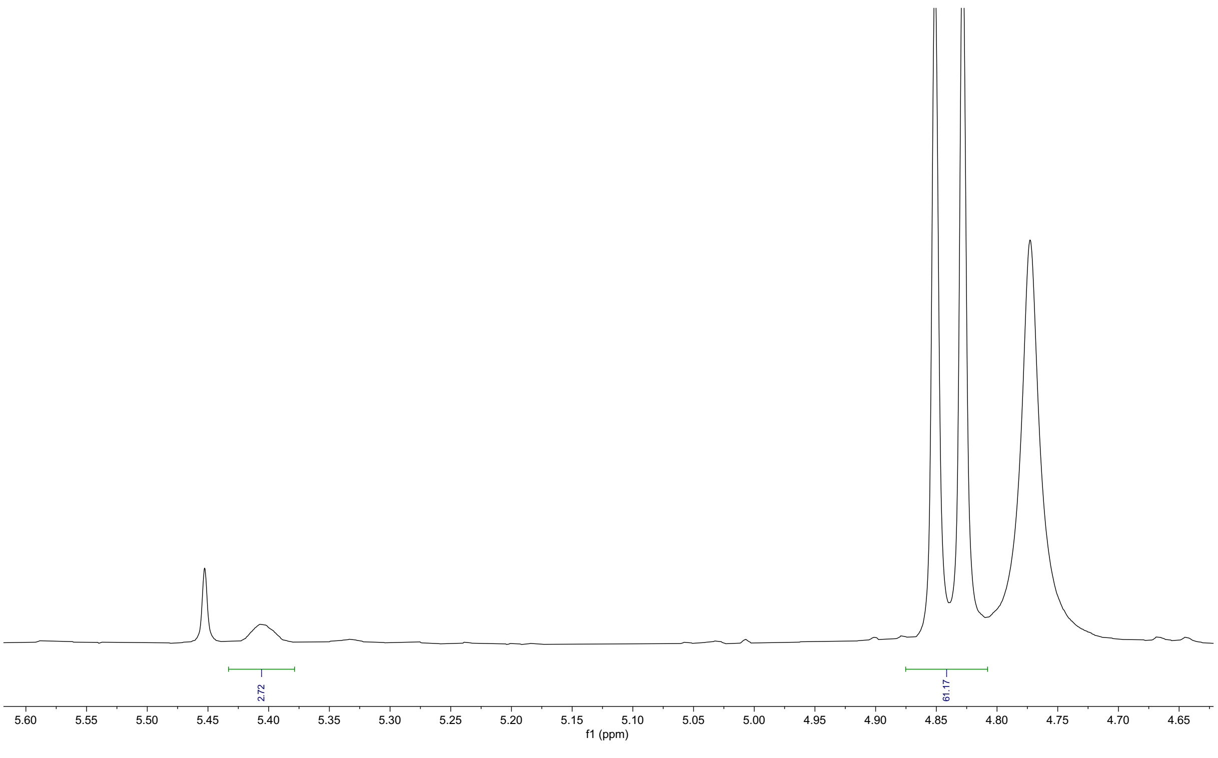

3,5-F-phenylboronic acid (1.5 eq cyclopentanone,9 hours reaction – duplicate) (400 MHz, $CDCl_3$**)**

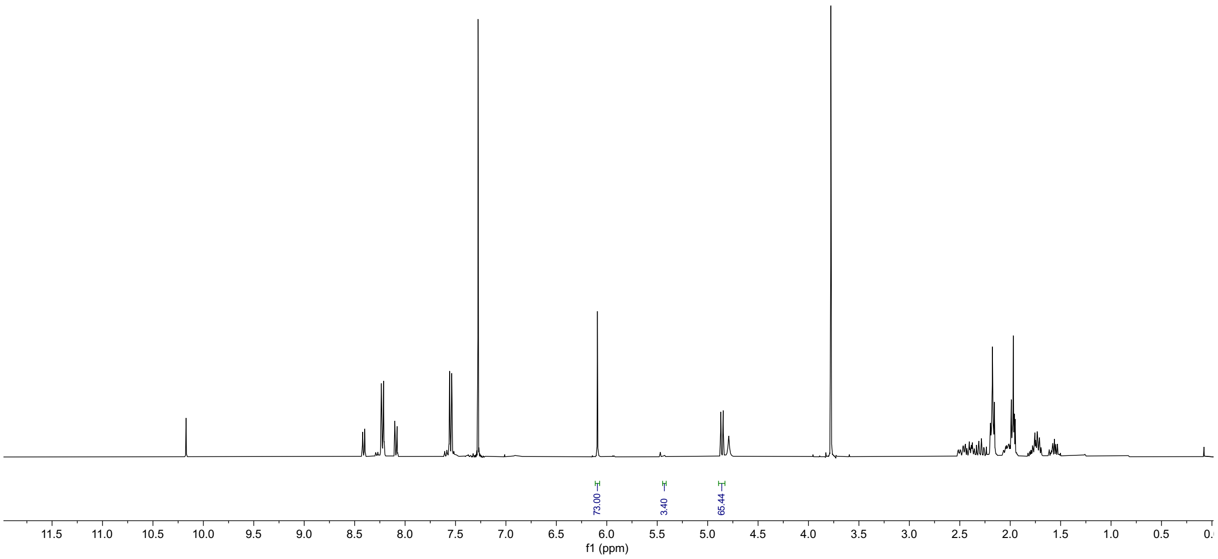

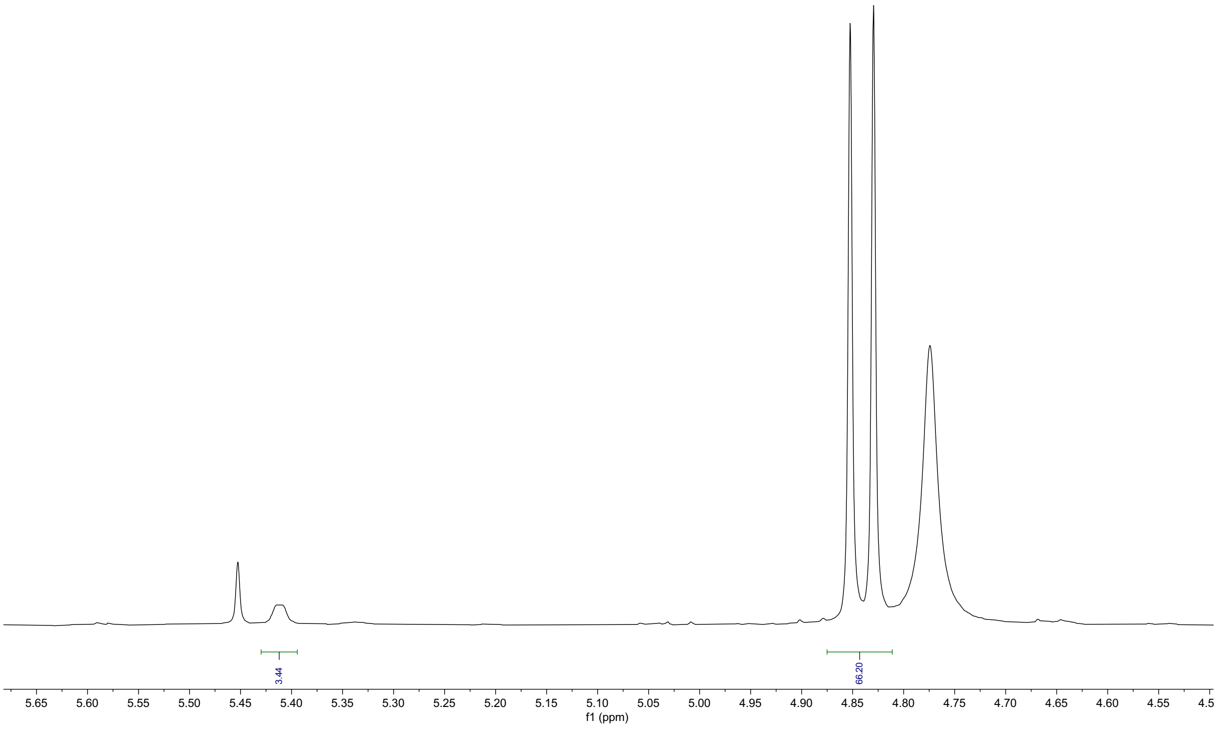

3,5-F-phenylboronic acid (1.2 eq cyclopentanone,9 hours reaction) (400 MHz, $CDCl_3$**)**

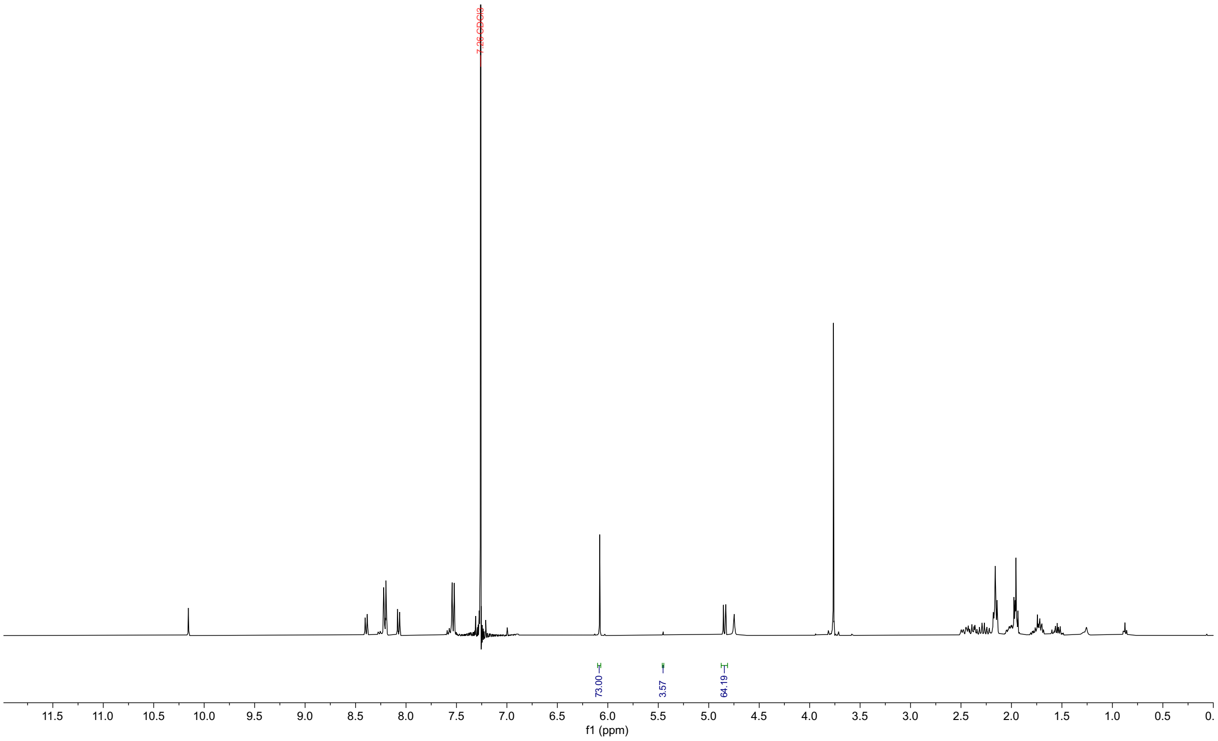

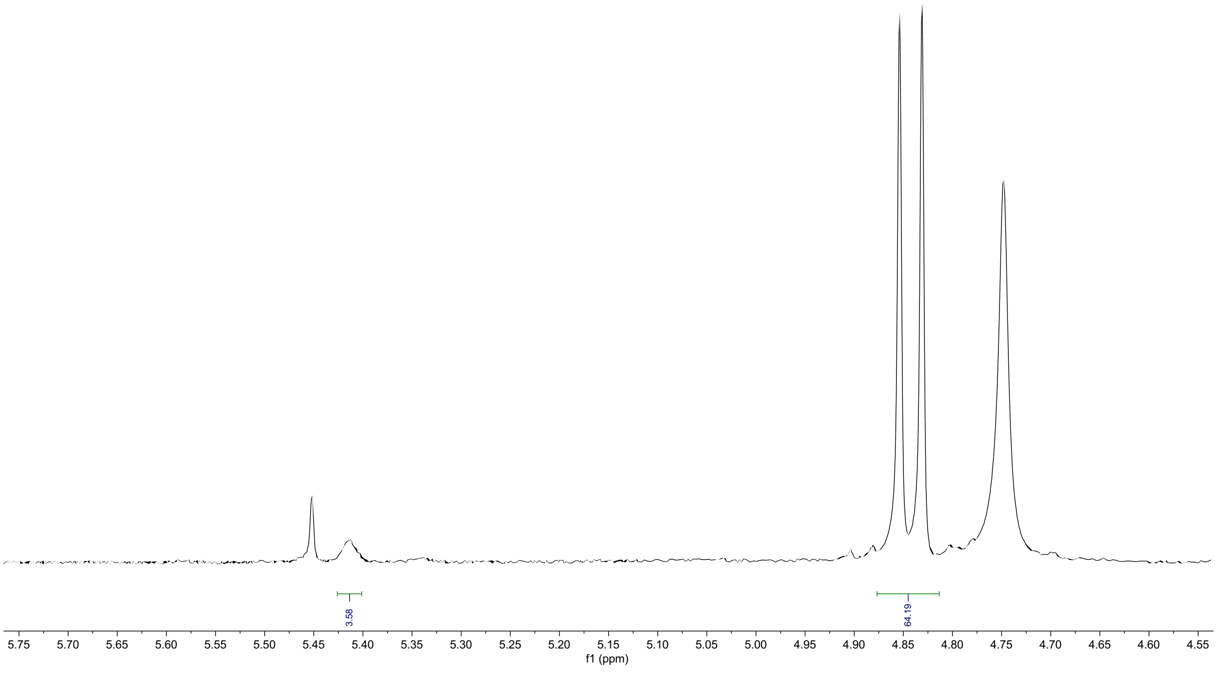

3,5-F-phenylboronic acid (1.2 eq cyclopentanone,9 hours reaction – duplicate) (400 MHz, $CDCl_3$**)**

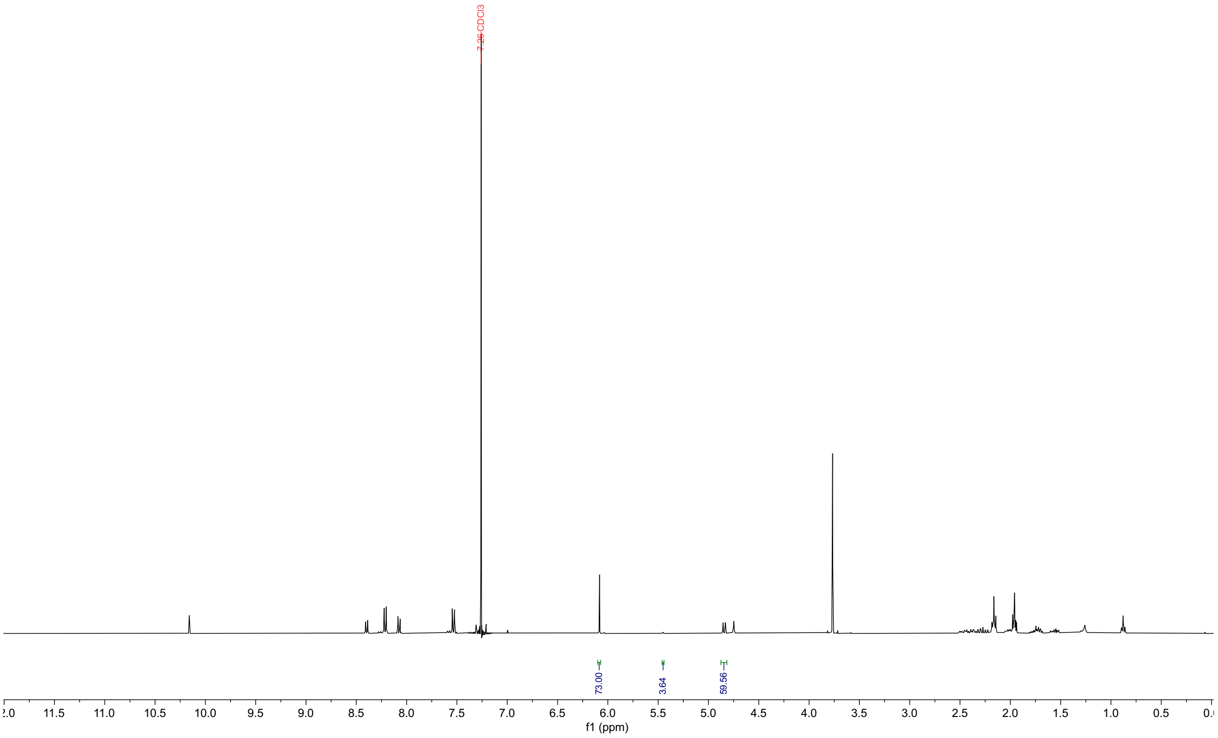

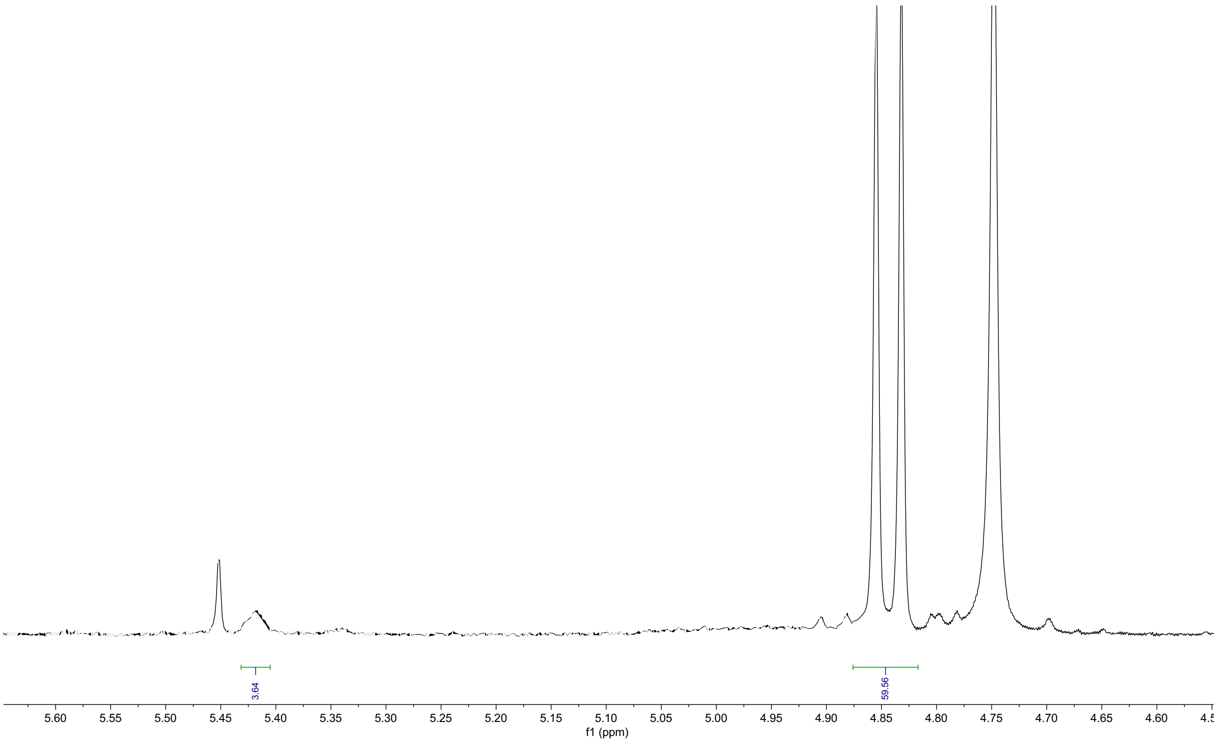

3,5-F-phenylboronic acid (1 eq cyclopentanone,9 hours reaction) (400 MHz, CDCl$_3$**)**

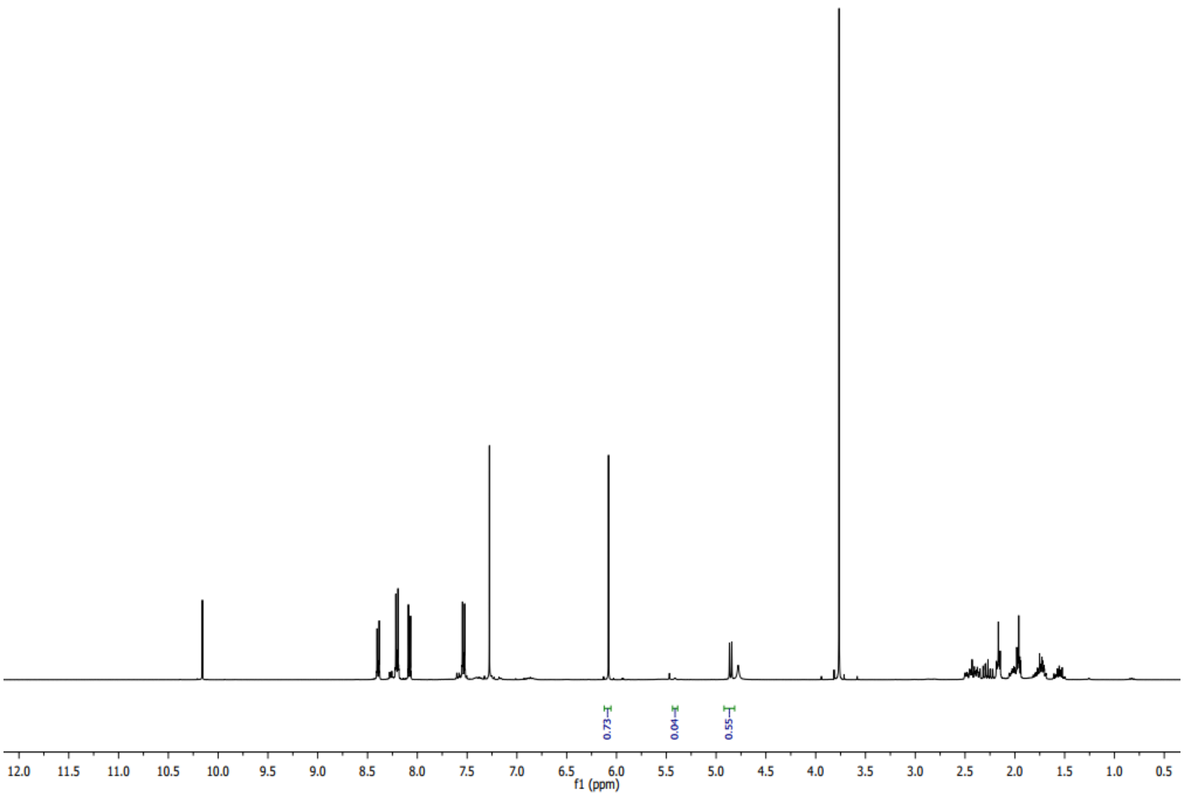

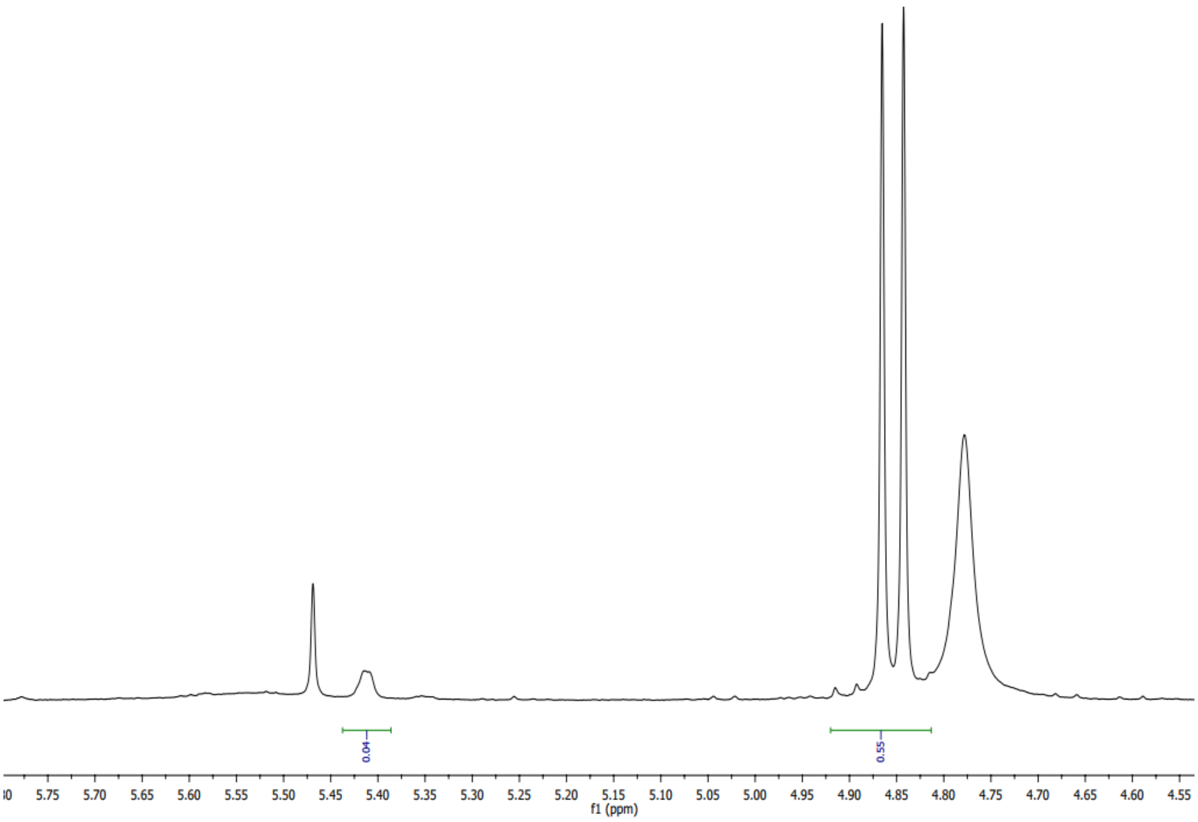

3,5-F-phenylboronic acid (1 eq cyclopentanone,9 hours reaction – duplicate) (400 MHz, $CDCl_3$**)**

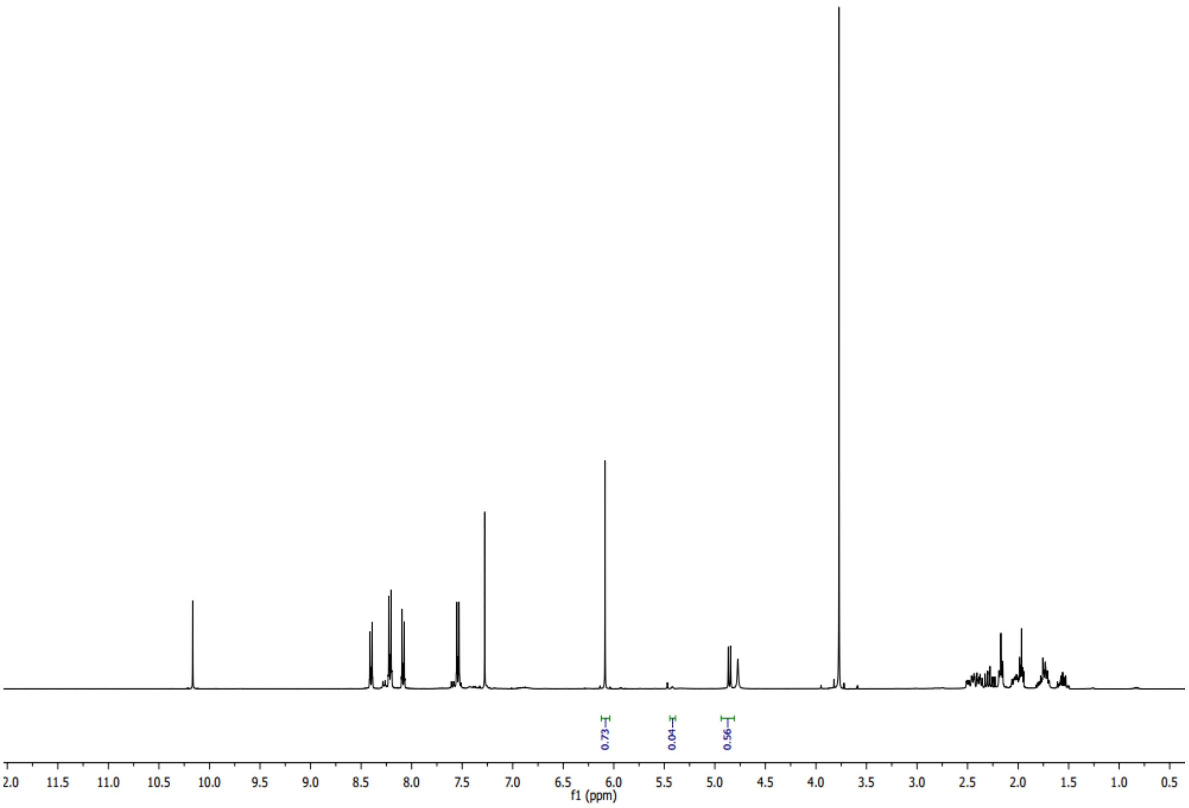

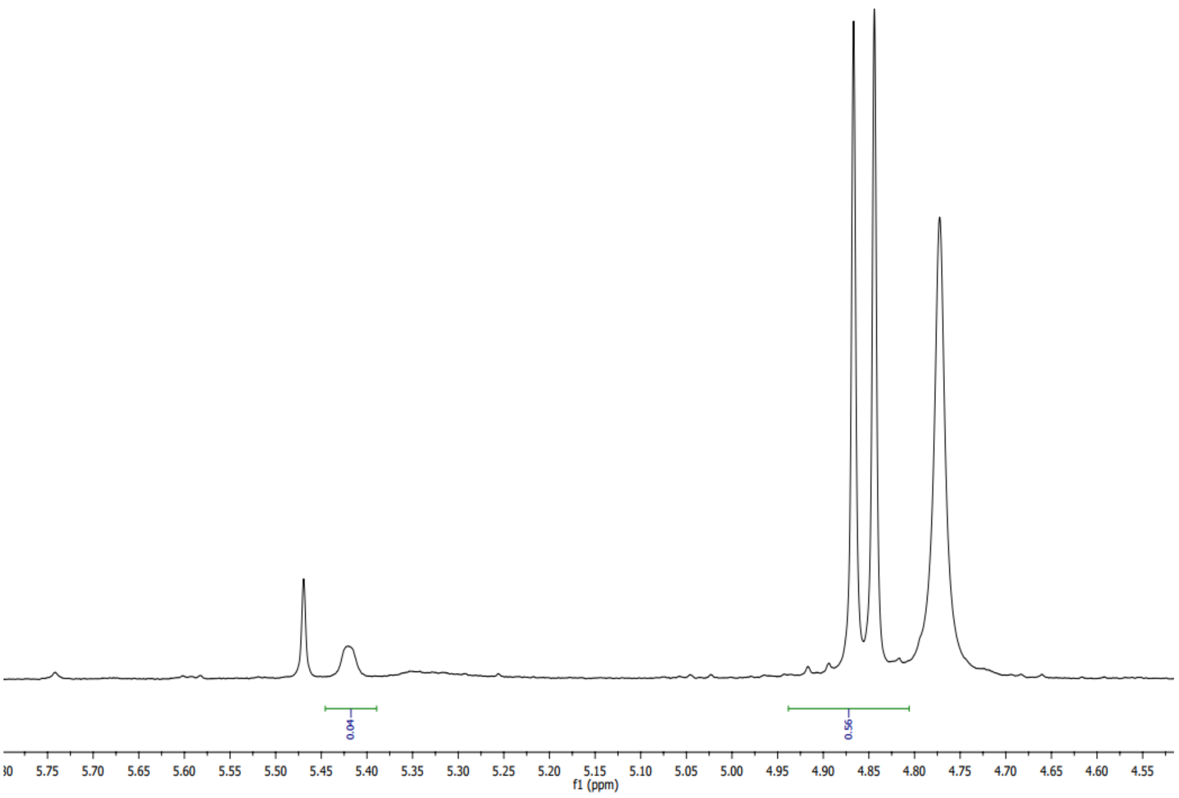

3,5-F-phenylboronic acid (5 eq cyclopentanone, 6 hours reaction) (400 MHz, CDCl$_3$)

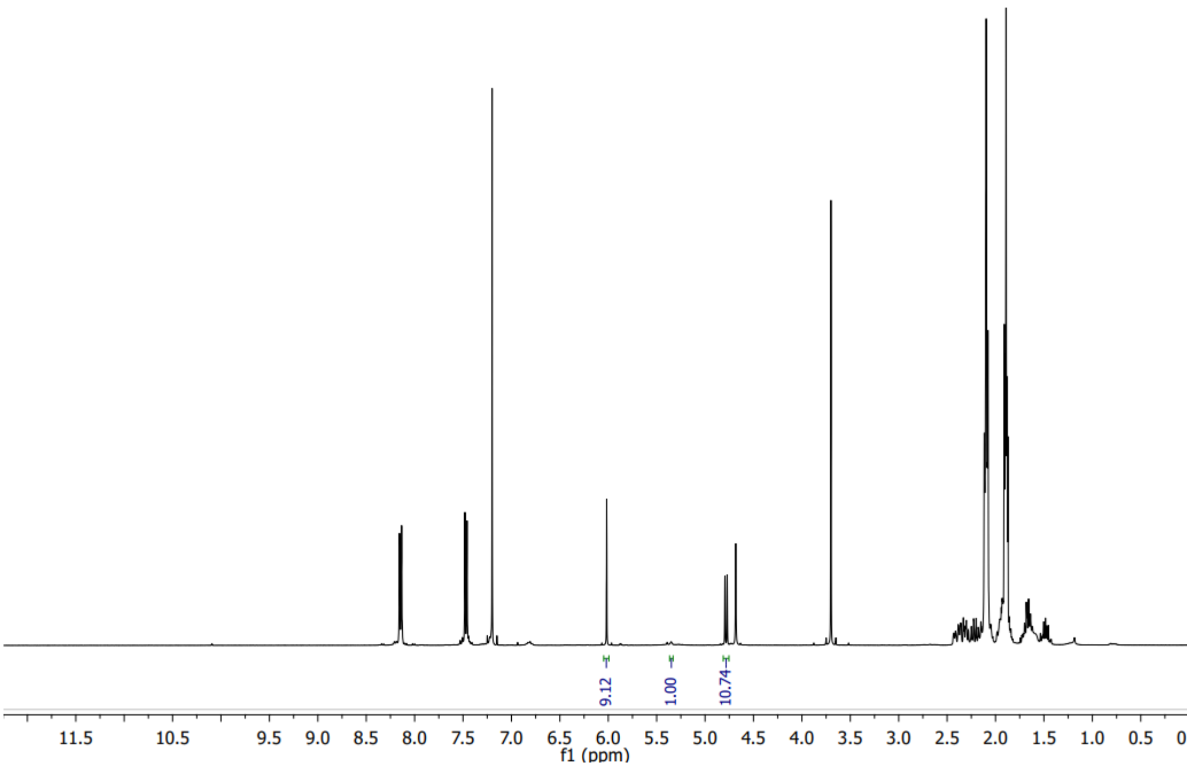

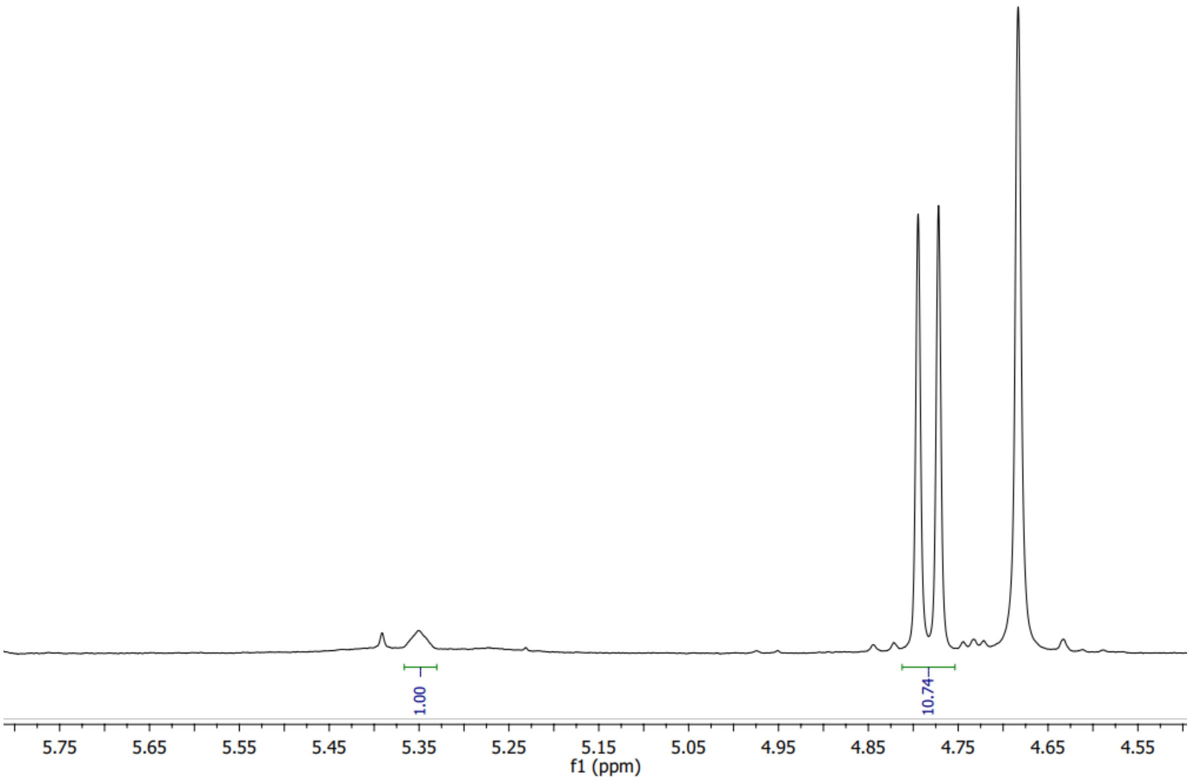

3,5-F-phenylboronic acid (5 eq cyclopentanone, 6 hours reaction – duplicate) (400 MHz, $CDCl_3$**)**

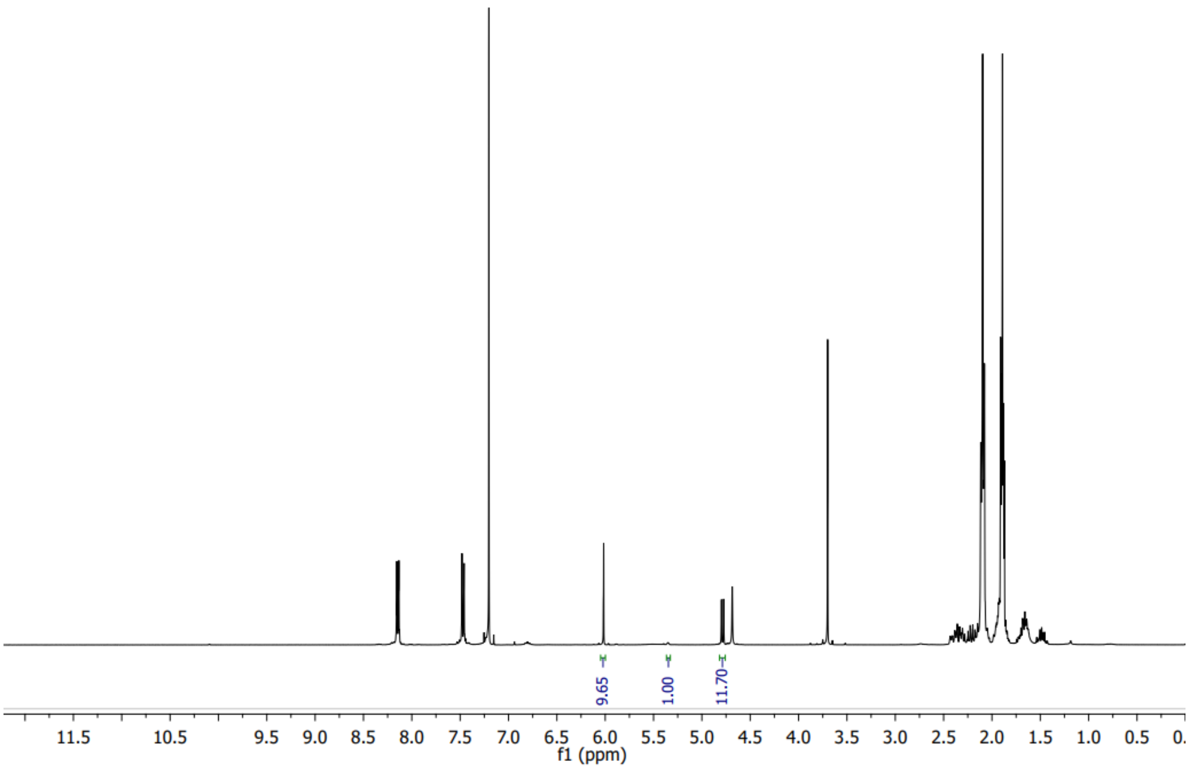

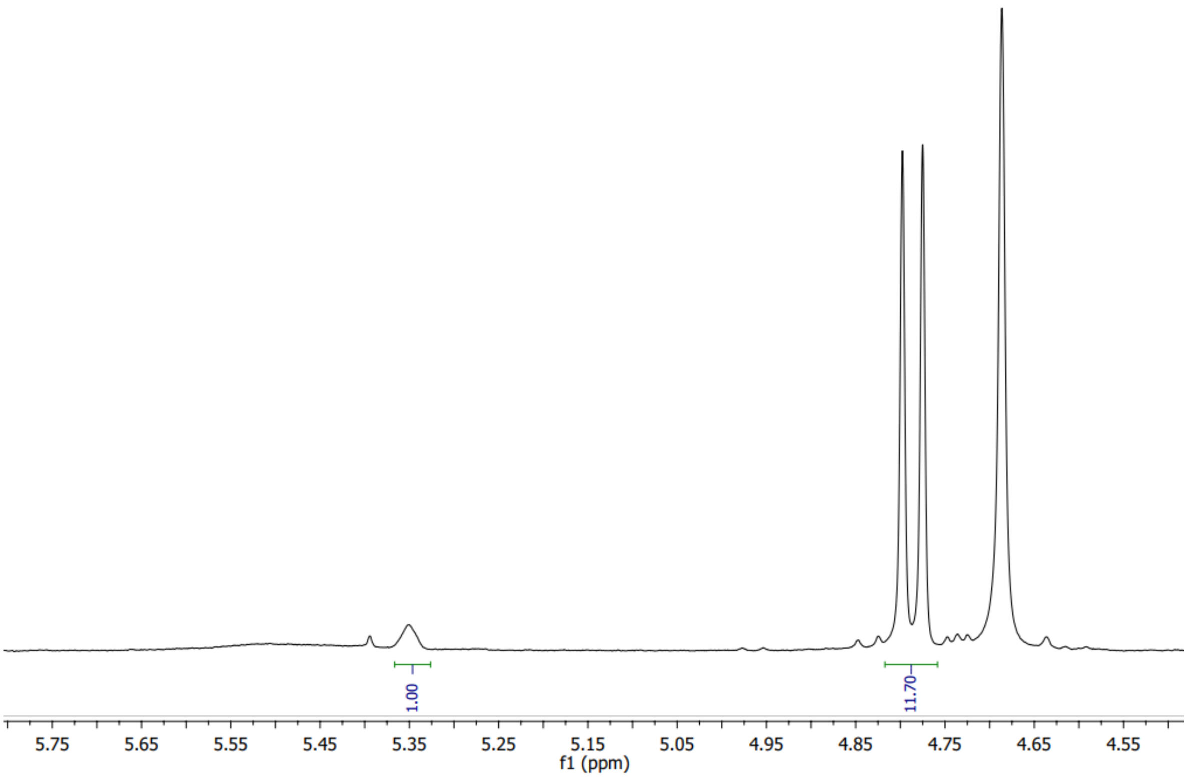

3,5-F-phenylboronic acid (3 eq cyclopentanone, 6 hours reaction) (400 MHz, CDCl$_3$)

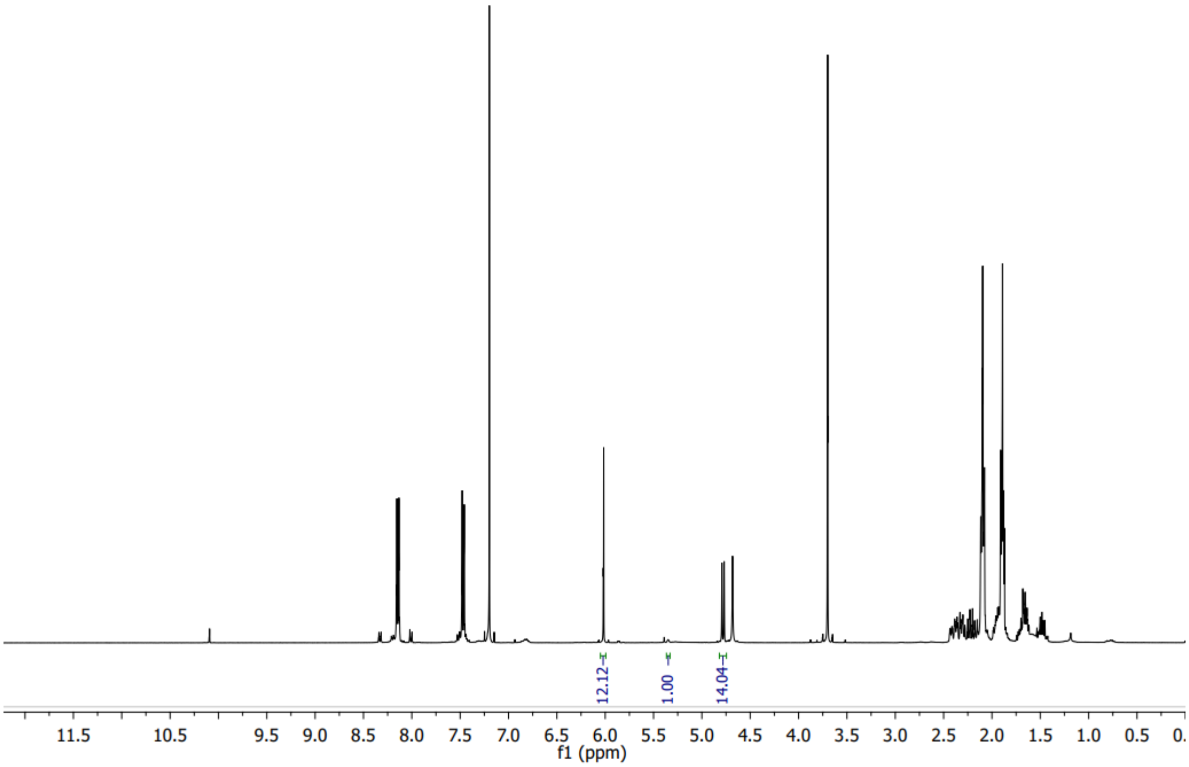

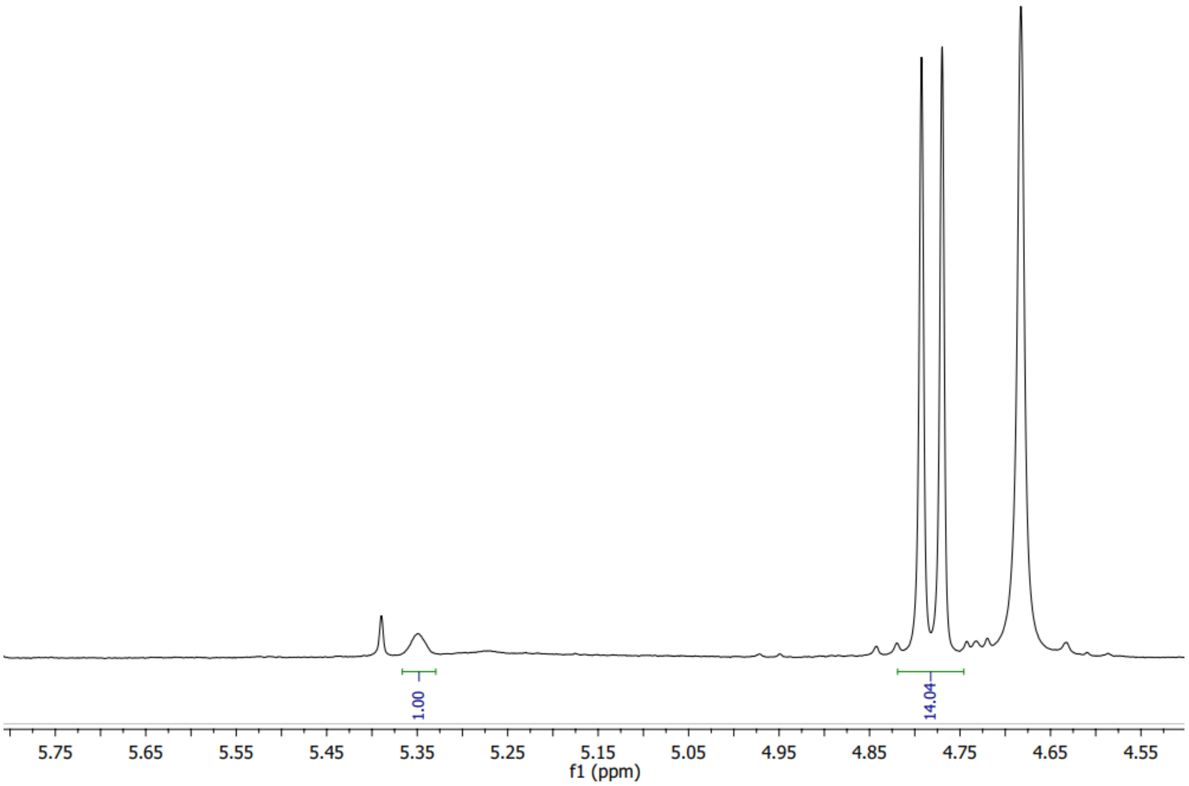

3,5-F-phenylboronic acid (3 eq cyclopentanone, 6 hours reaction - duplicate) (400 MHz, $CDCl_3$)

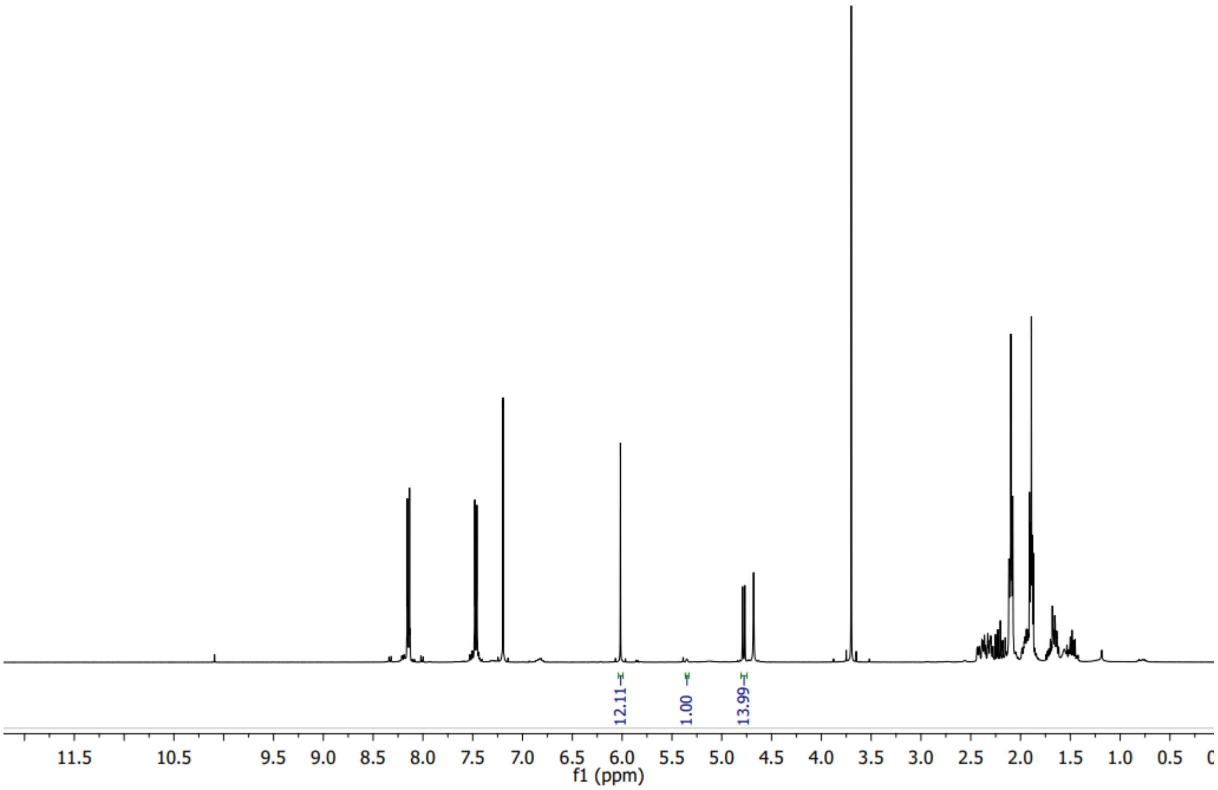

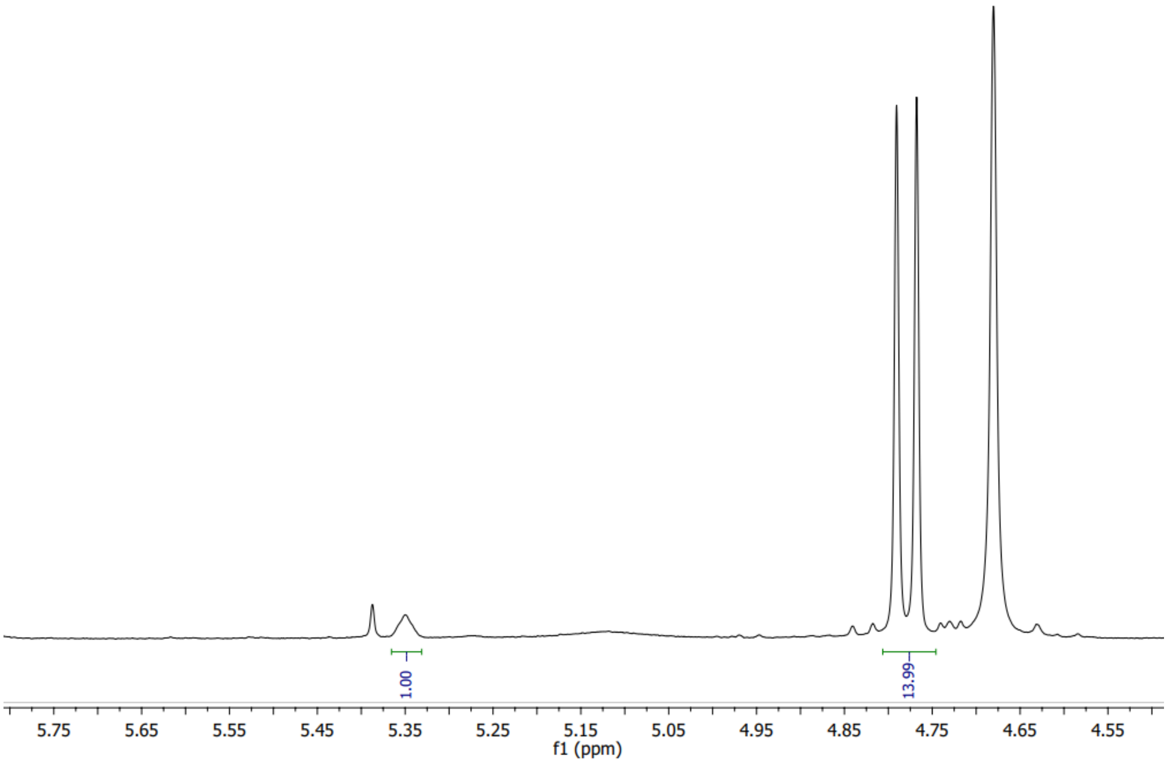

3,5-F-phenylboronic acid (2 eq cyclopentanone, 6 hours reaction) (400 MHz, CDCl$_3$**)**

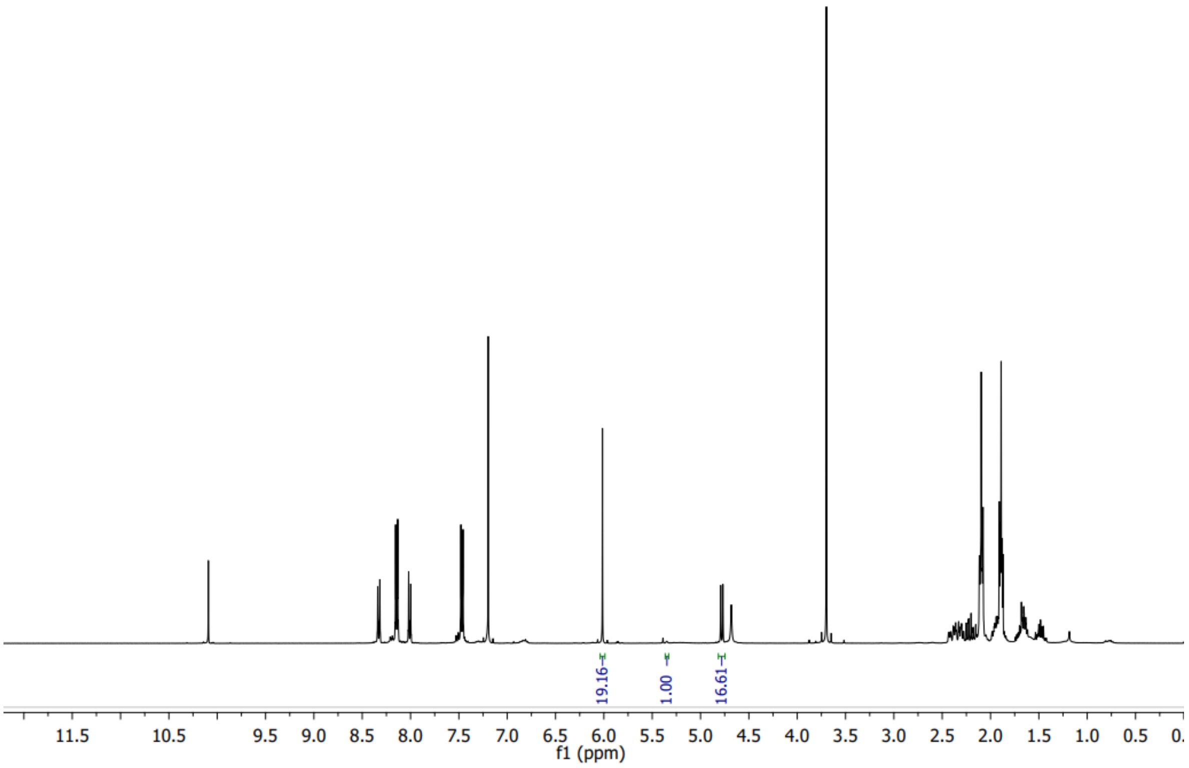

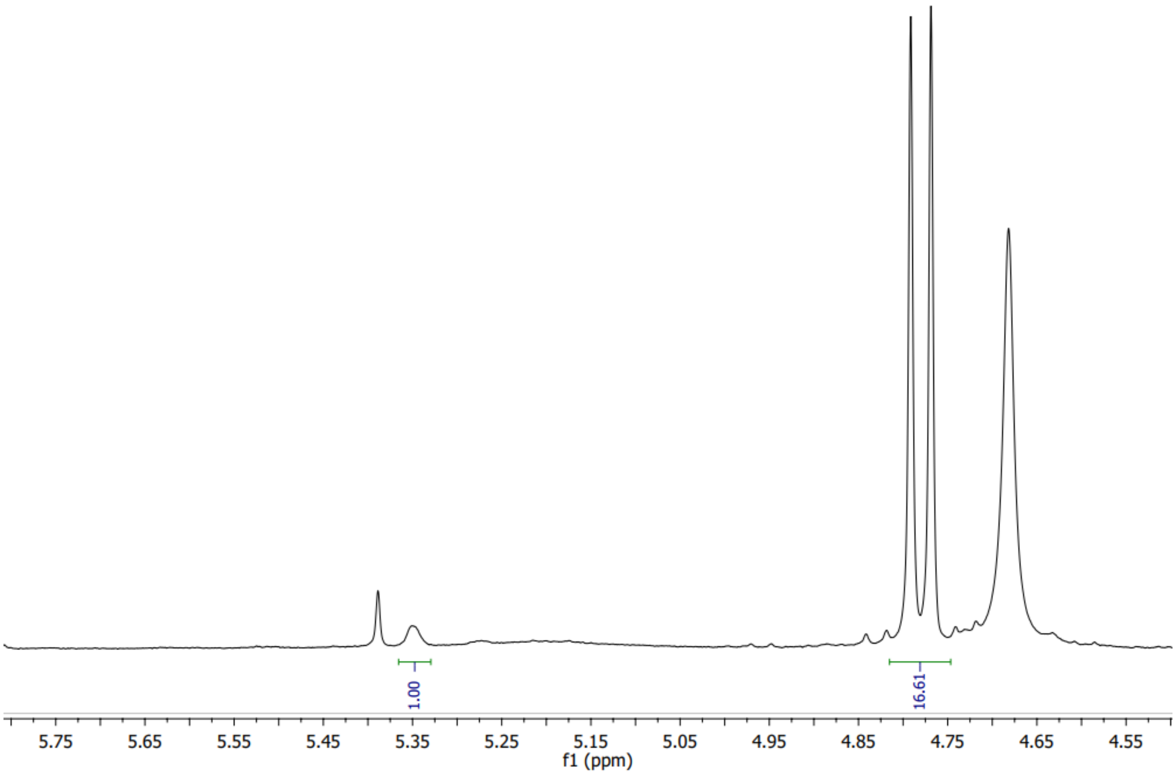

3,5-F-phenylboronic acid (2 eq cyclopentanone, 6 hours reaction - duplicate) (400 MHz, CDCl$_3$**)**

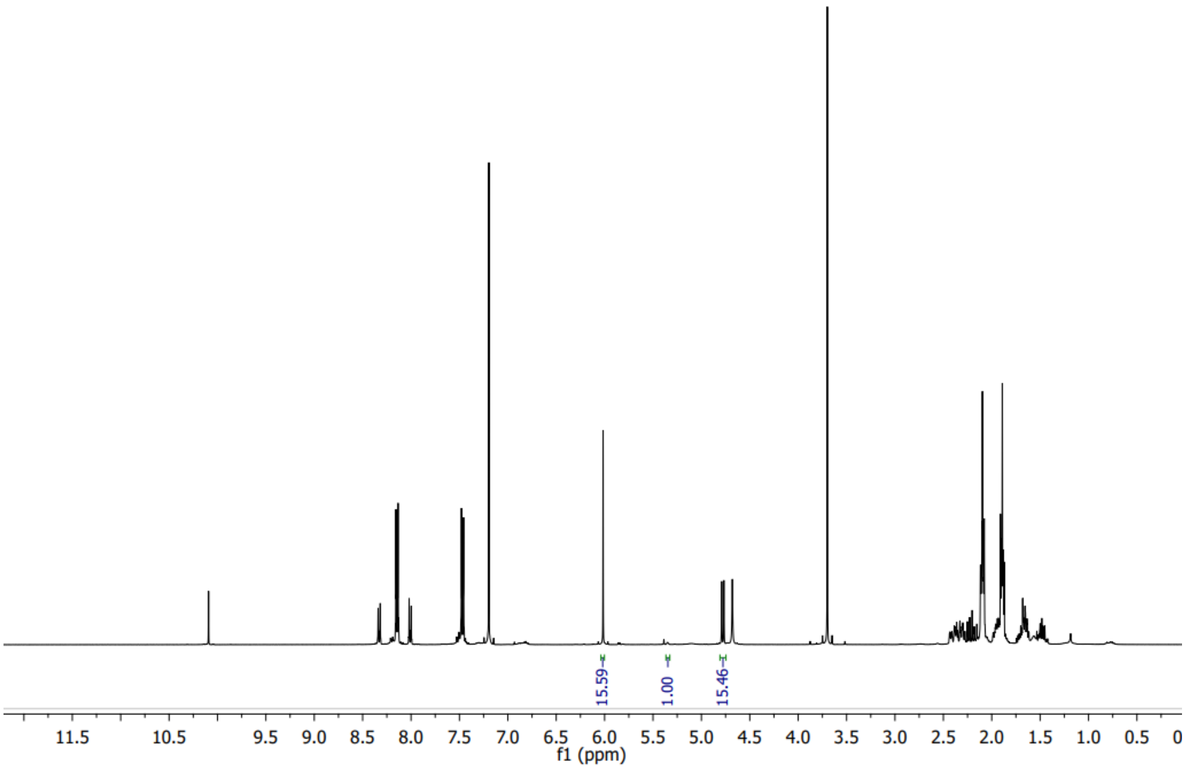

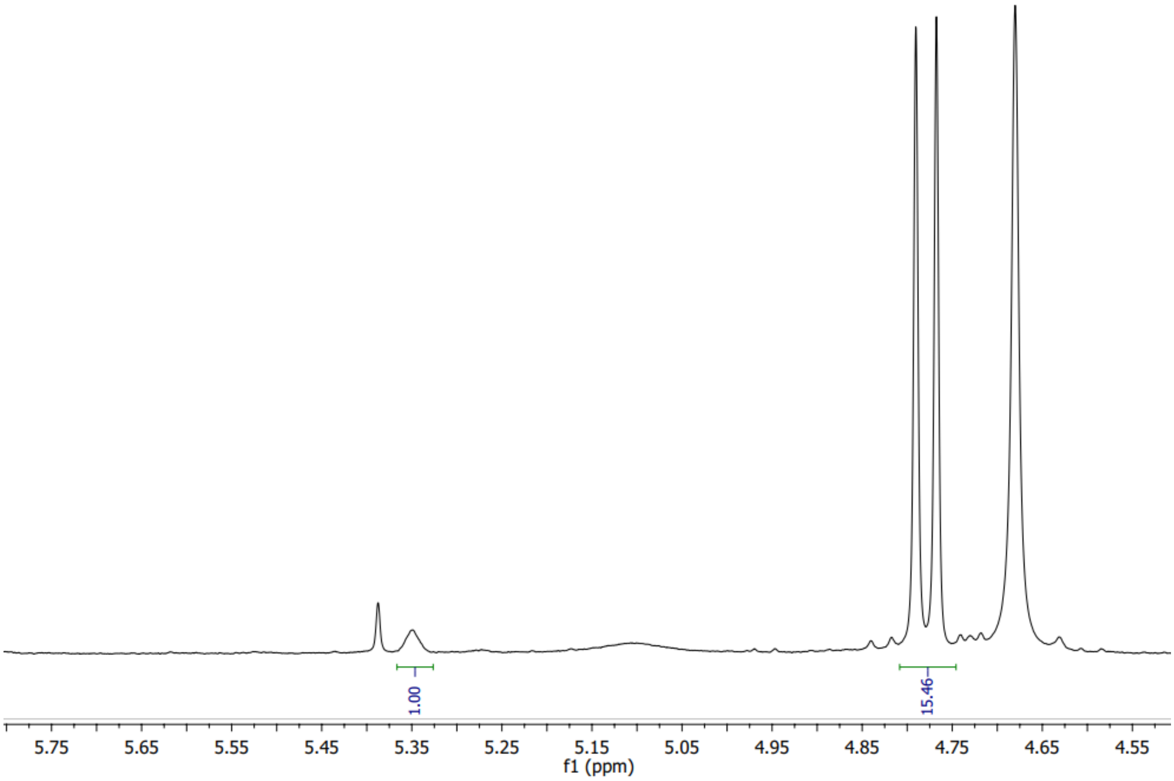

3,5-F-phenylboronic acid (1 eq cyclopentanone, 6 hours reaction) (400 MHz, CDCl$_3$)

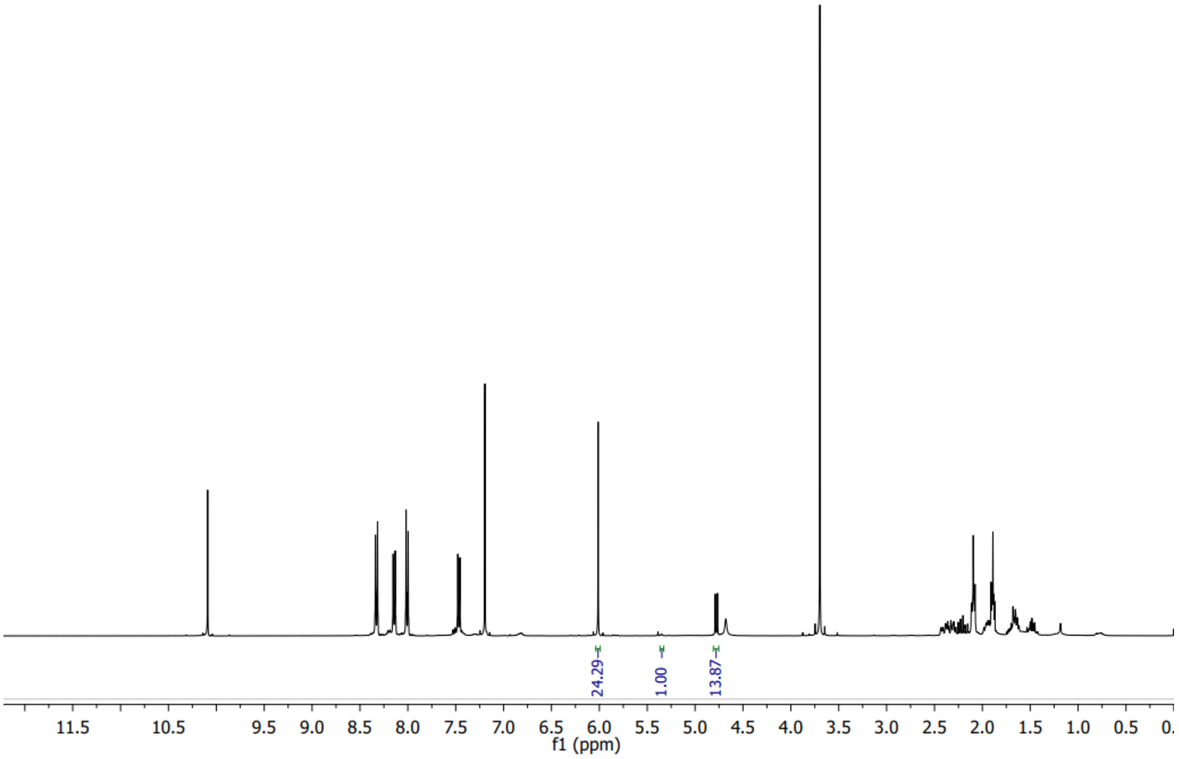

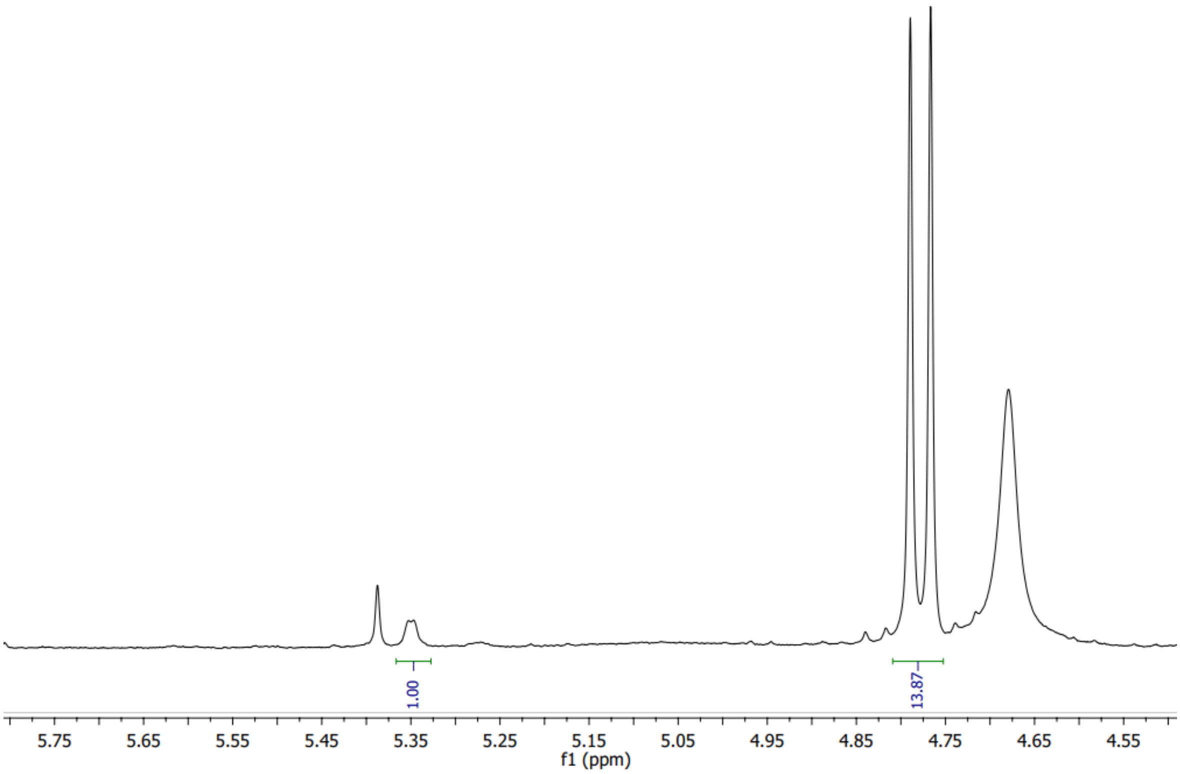

3,5-F-phenylboronic acid (1 eq cyclopentanone, 6 hours reaction - duplicate) (400 MHz, CDCl$_3$)

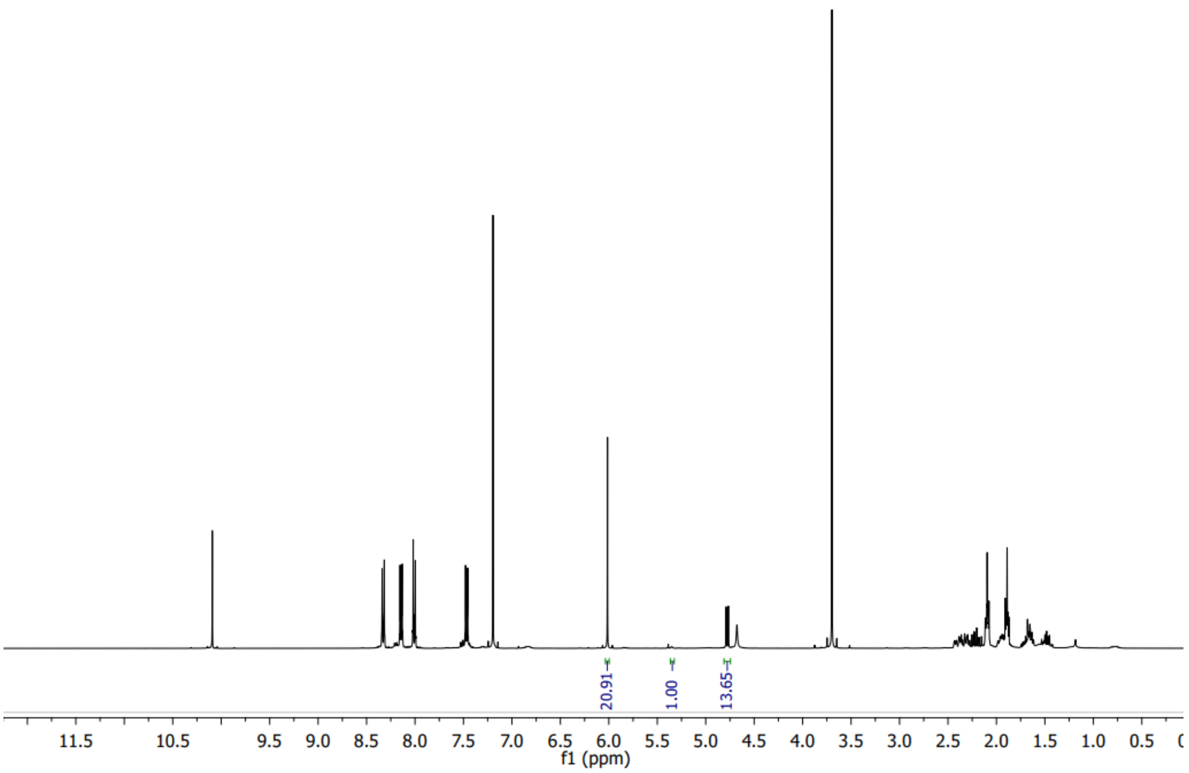

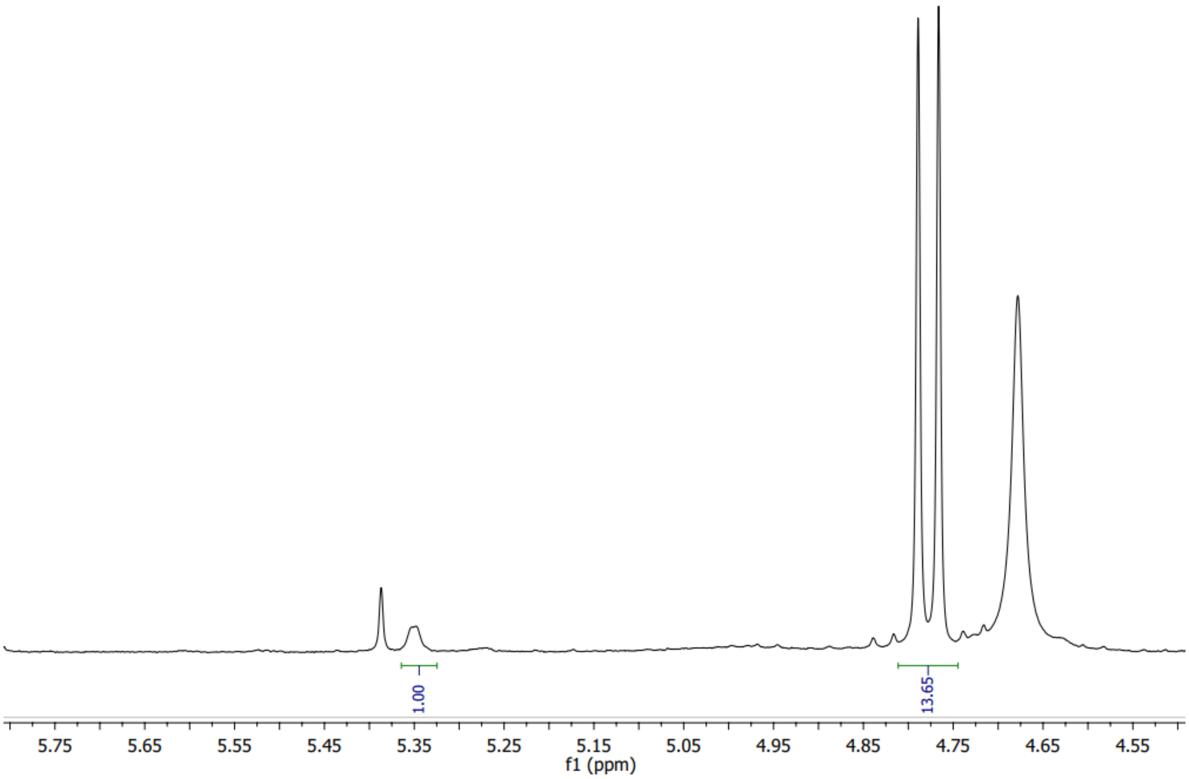

18.7. NMR Traces for Table S7. Reducing amount of ketone with 3-CF$_3$-phenyl boronic acid

3-CF$_3$-phenylboronic acid (2 eq cyclopentanone, 1 hours reaction) (400 MHz, CDCl$_3$**)**

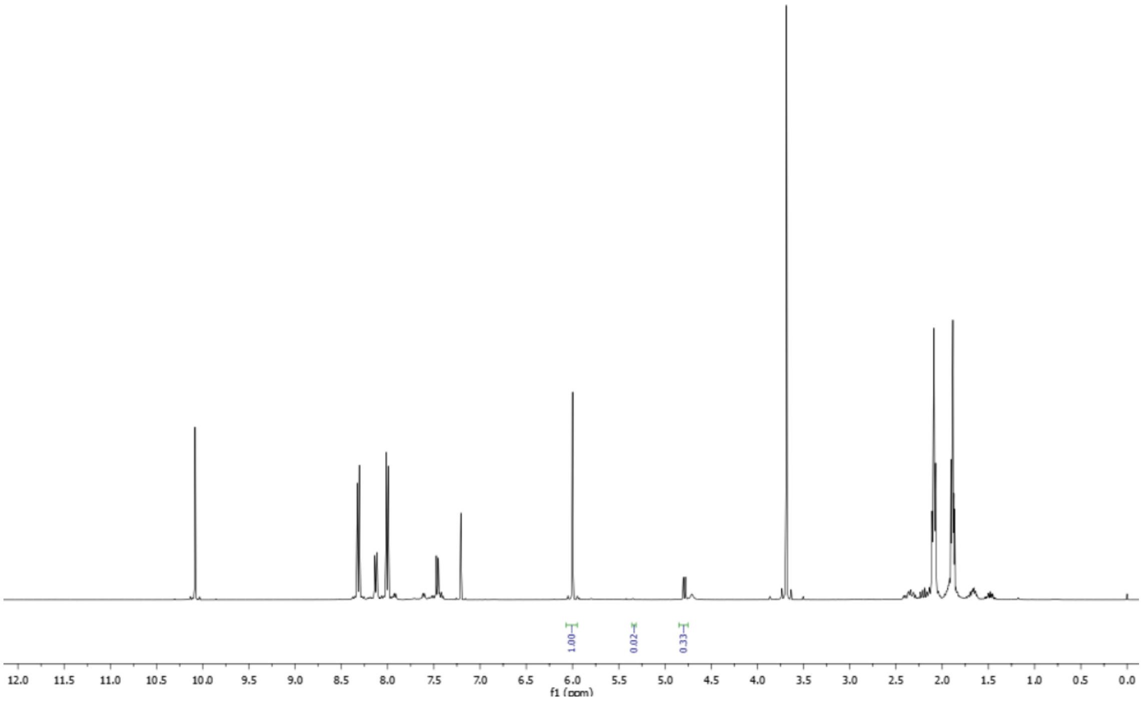

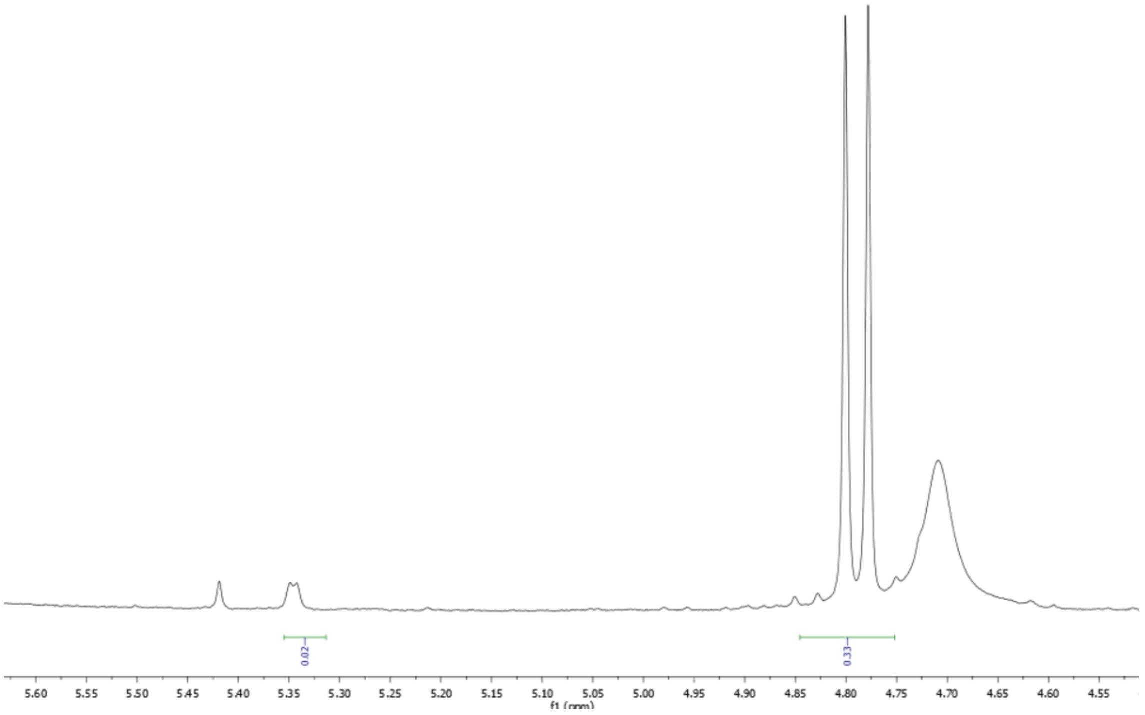

3-CF_3-phenylboronic acid (2 eq cyclopentanone, 1 hours reaction) - duplicate (400 MHz, $CDCl_3$)

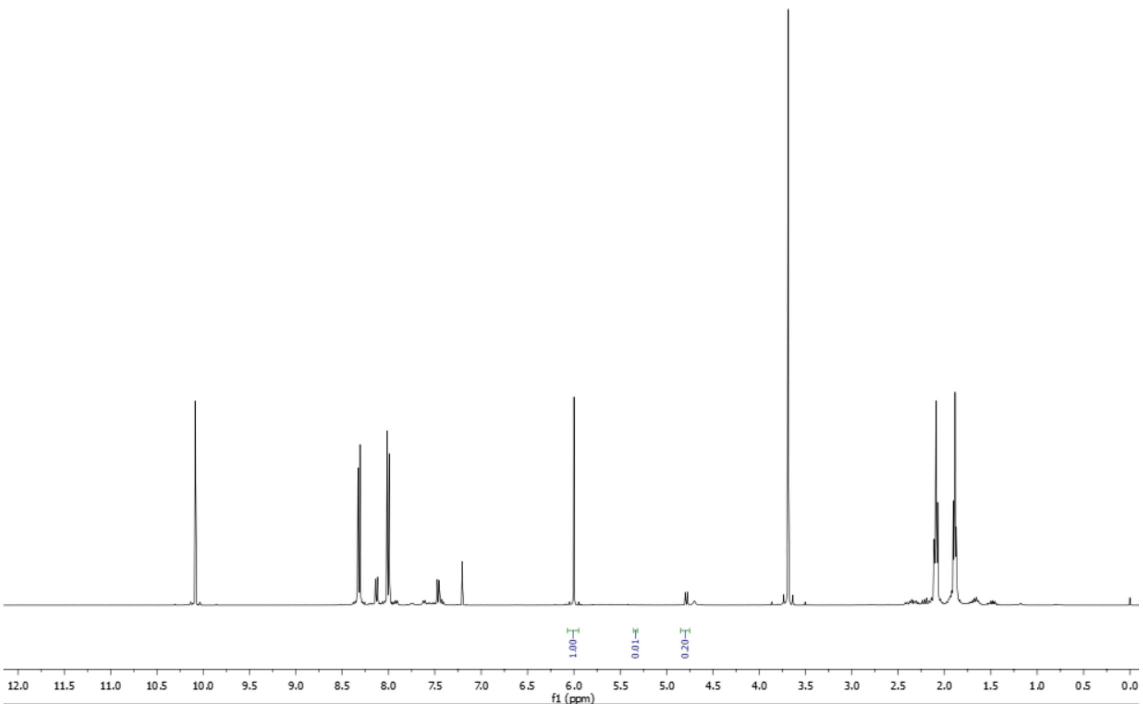

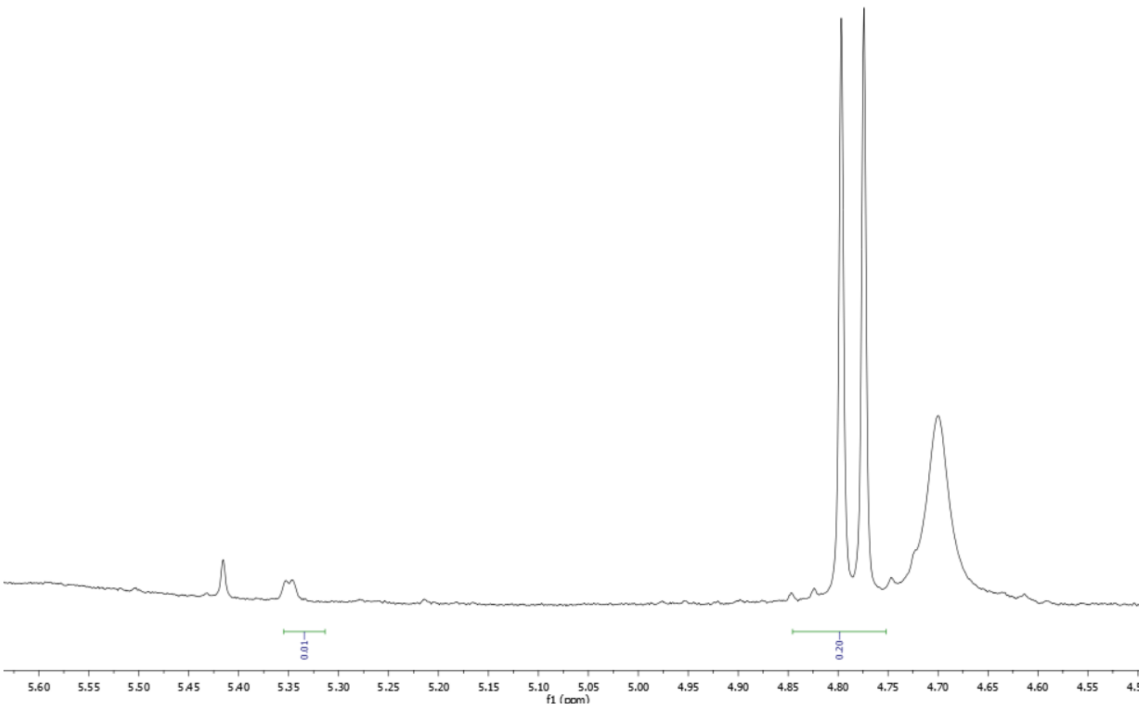

3-CF_3-phenylboronic acid (2 eq cyclopentanone, 3 hours reaction) (400 MHz, $CDCl_3$**)**

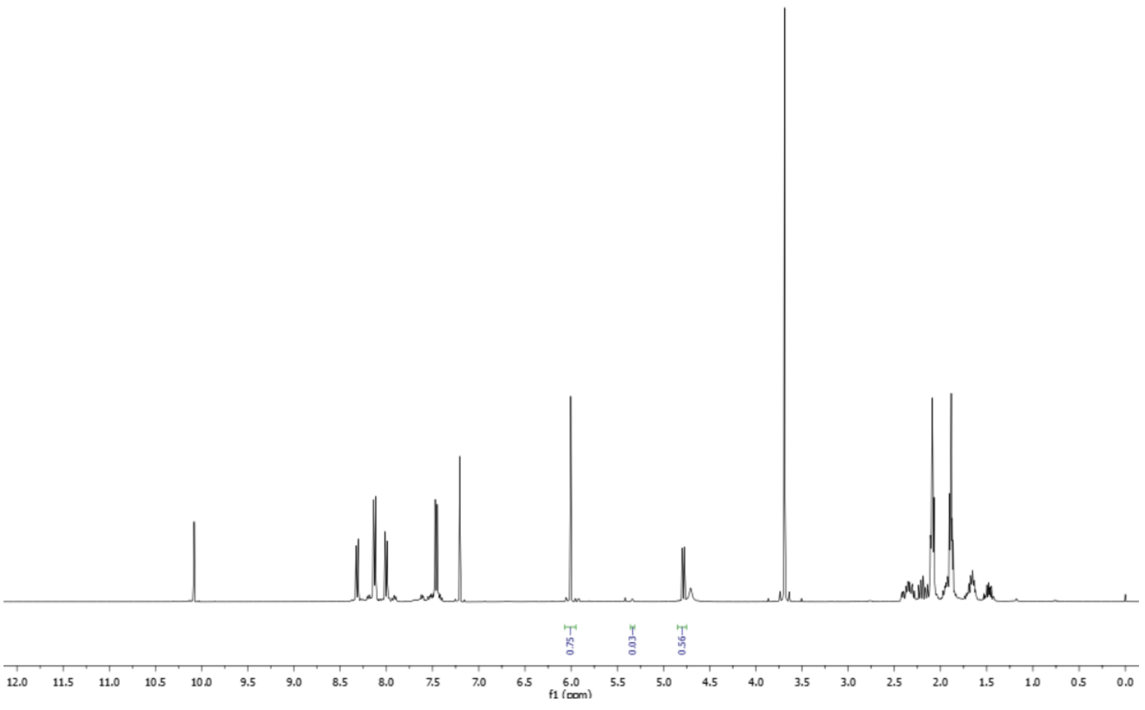

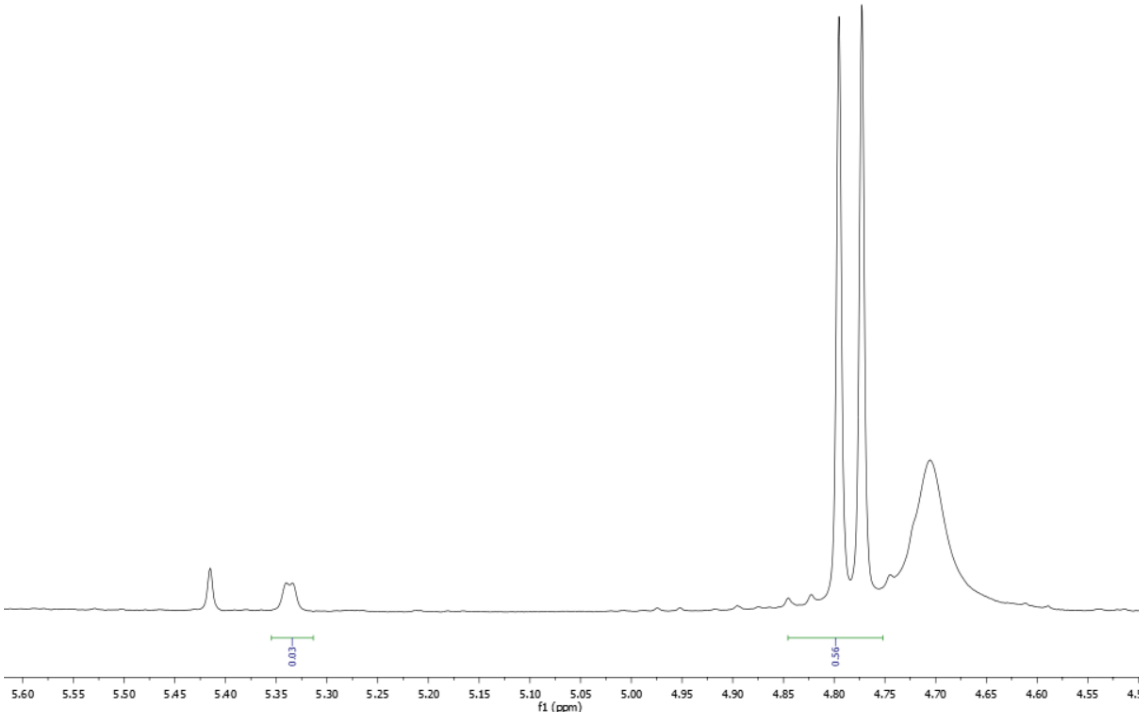

3-CF_3-phenylboronic acid (2 eq cyclopentanone, 3 hours reaction – duplicate) (400 MHz, $CDCl_3$)

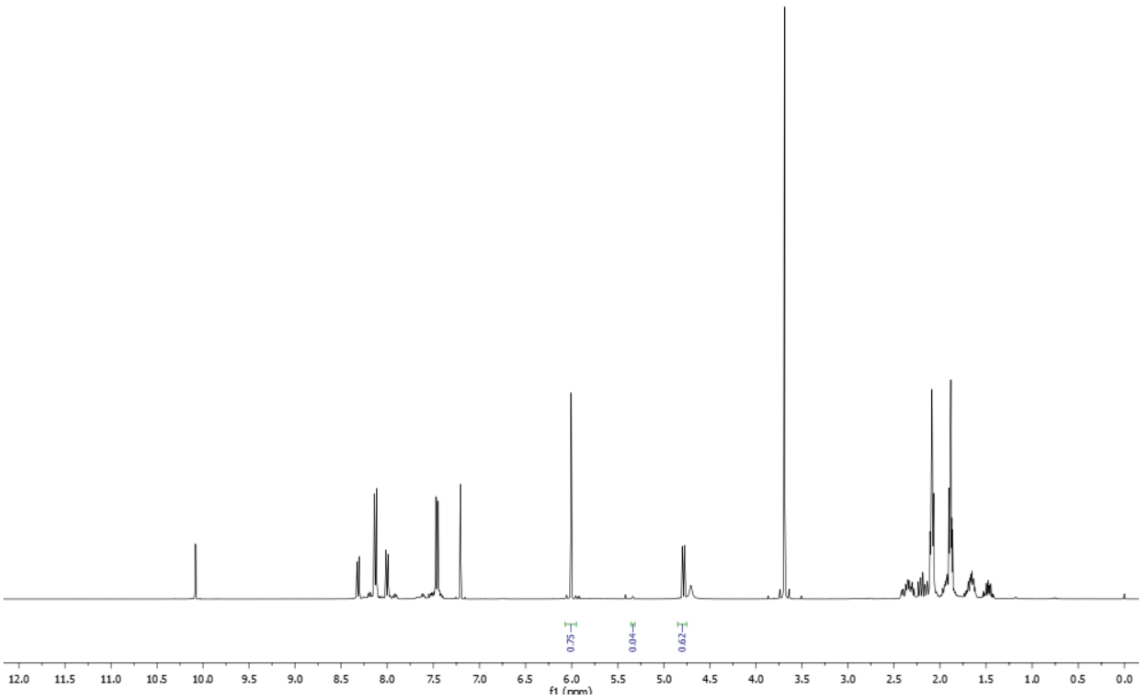

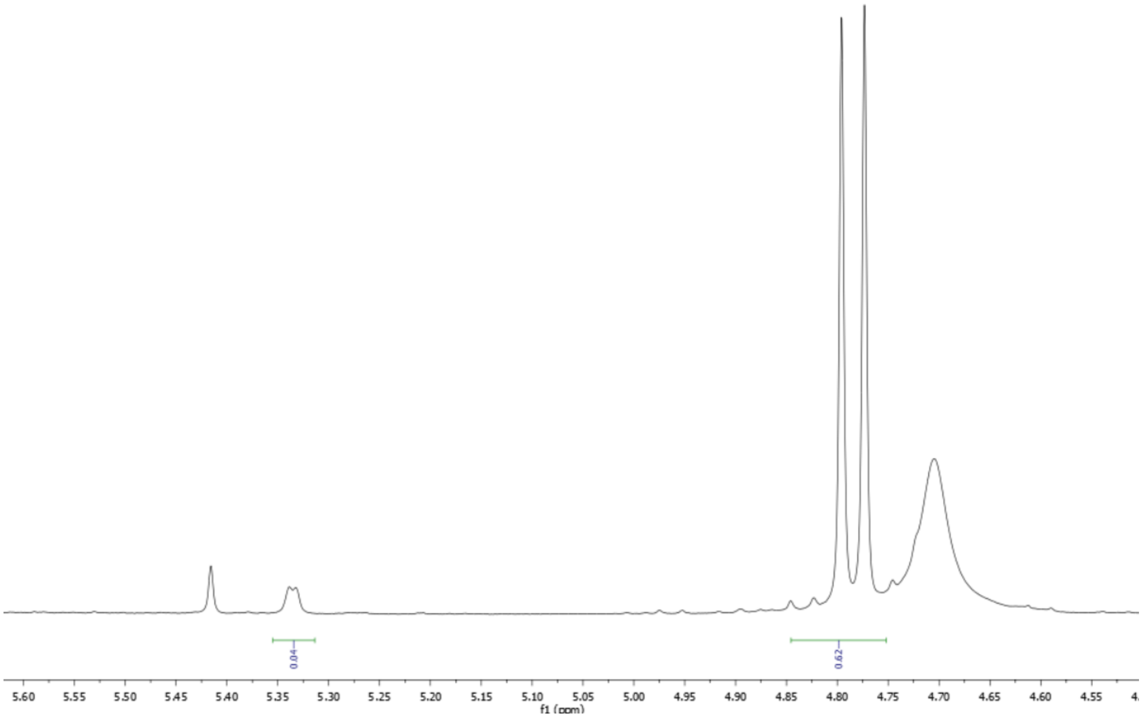

3-CF_3-phenylboronic acid (2 eq cyclopentanone, 6 hours reaction) (400 MHz, $CDCl_3$**)**

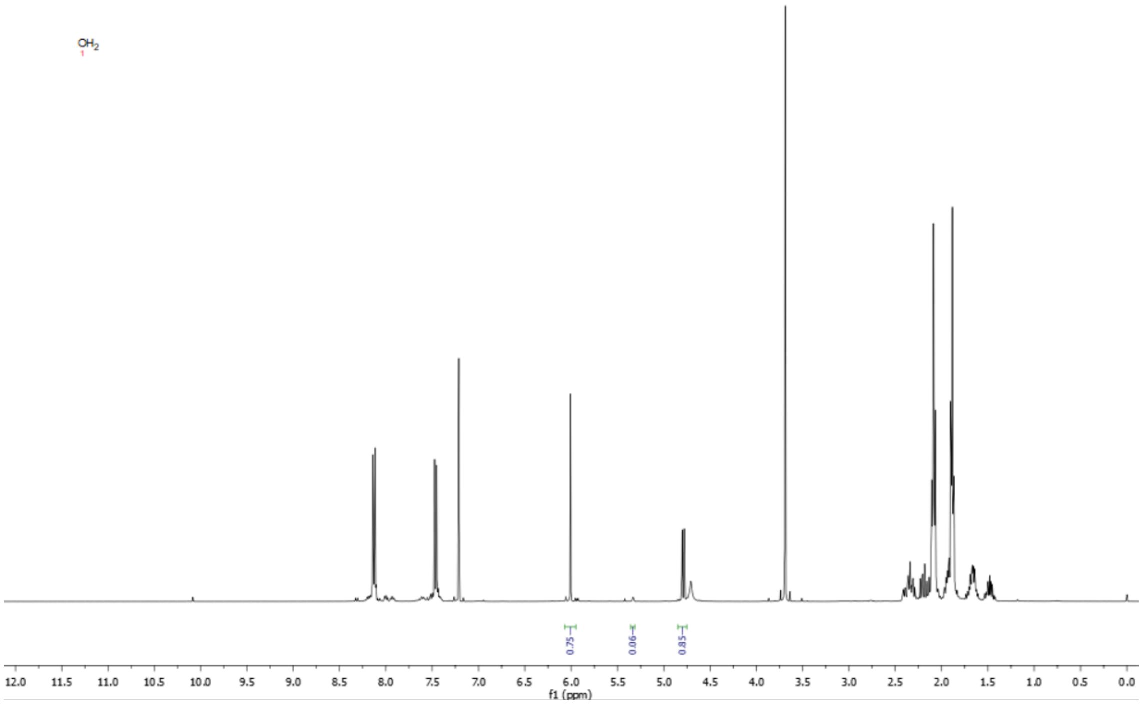

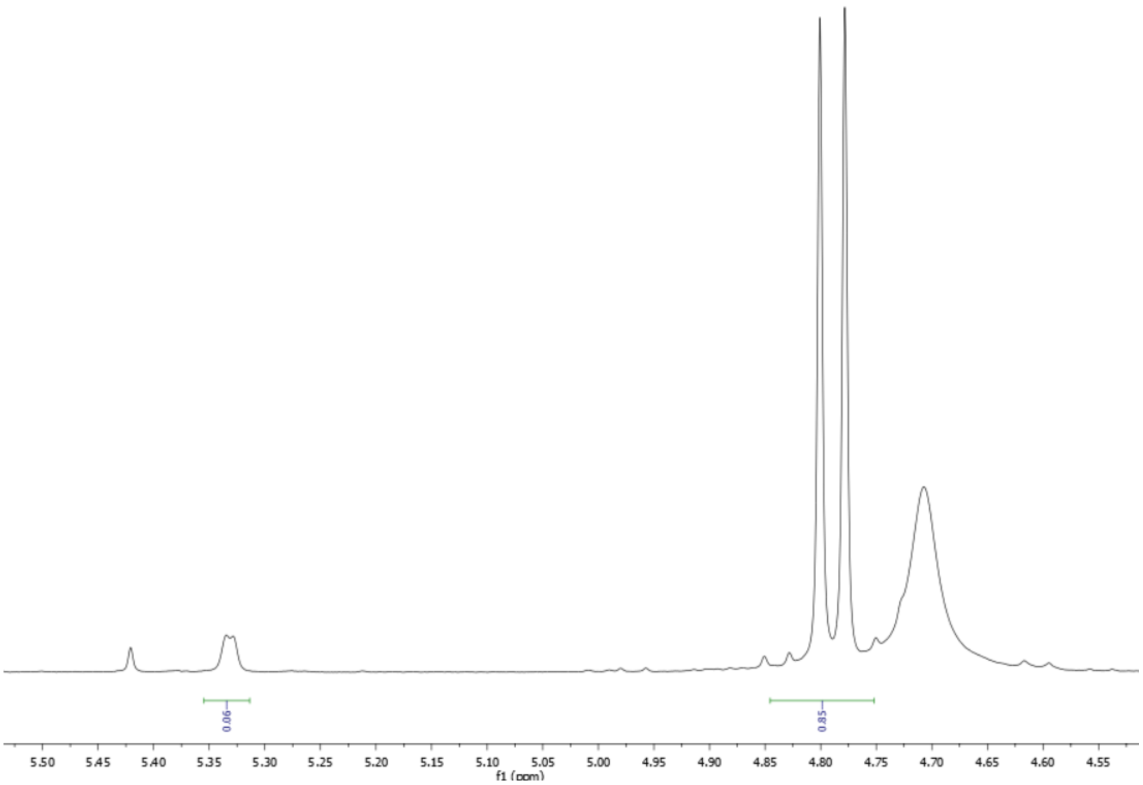

3-CF_3-phenylboronic acid (2 eq cyclopentanone, 6 hours reaction - duplicate) (400 MHz, $CDCl_3$**)**

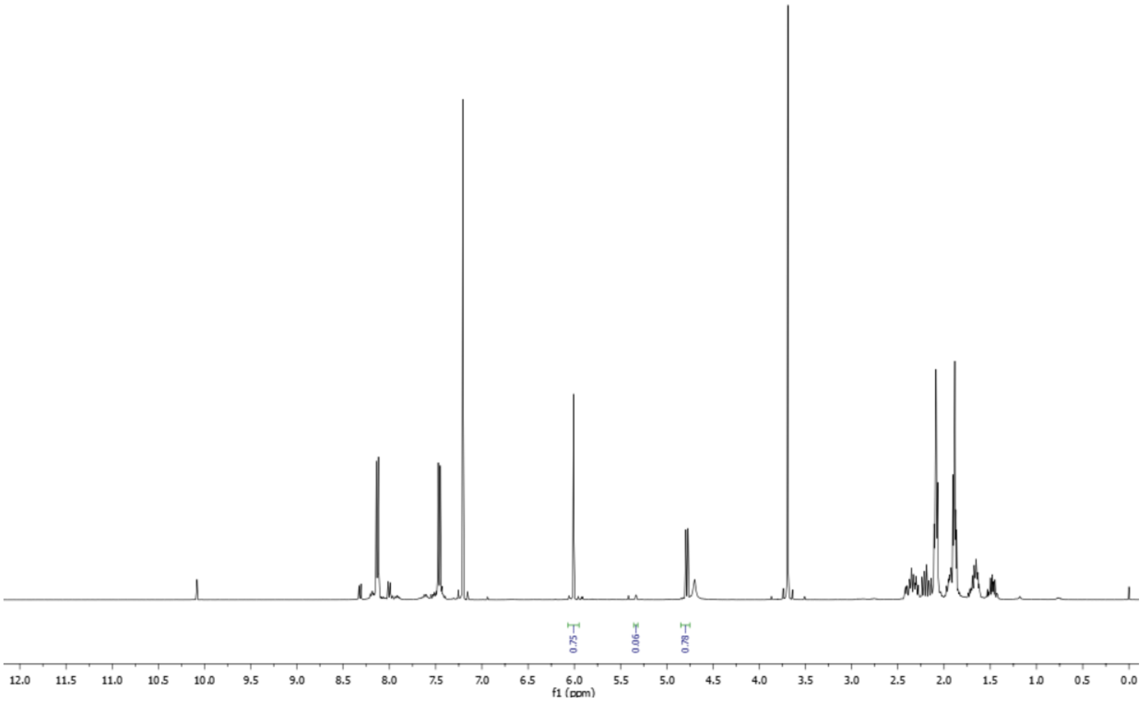

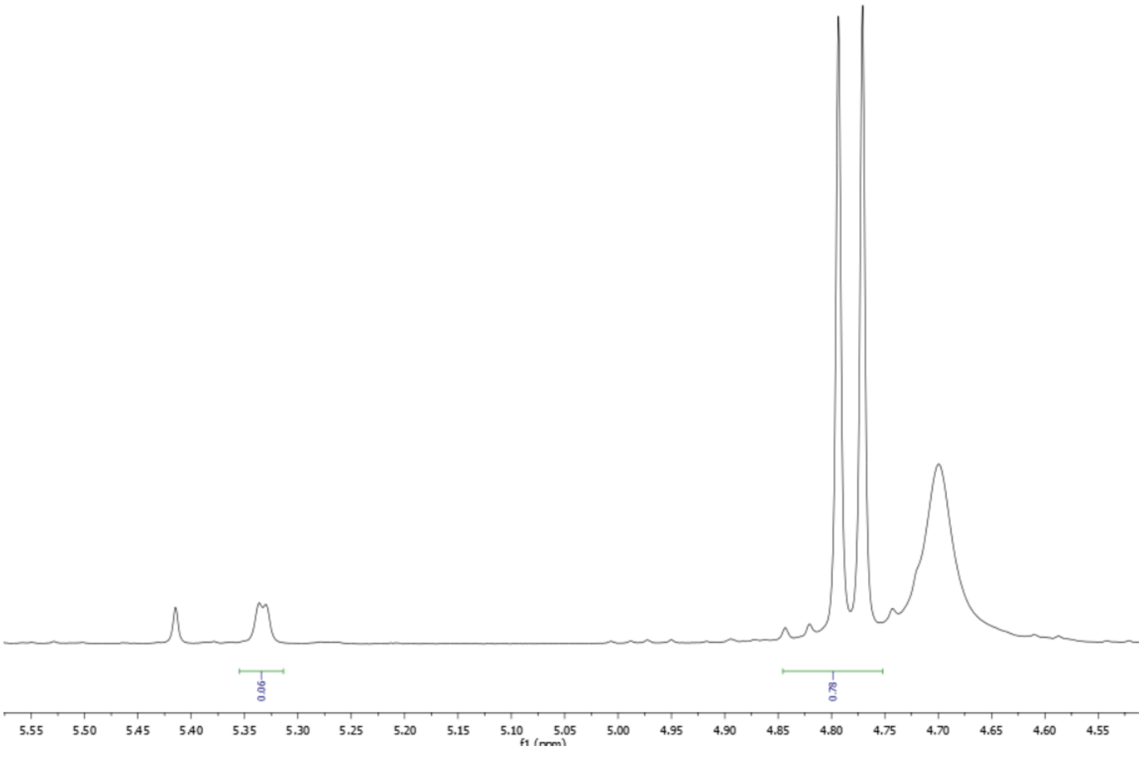

3-CF$_3$-phenylboronic acid (2 eq cyclopentanone, overnight reaction) (400 MHz, CDCl$_3$**)**

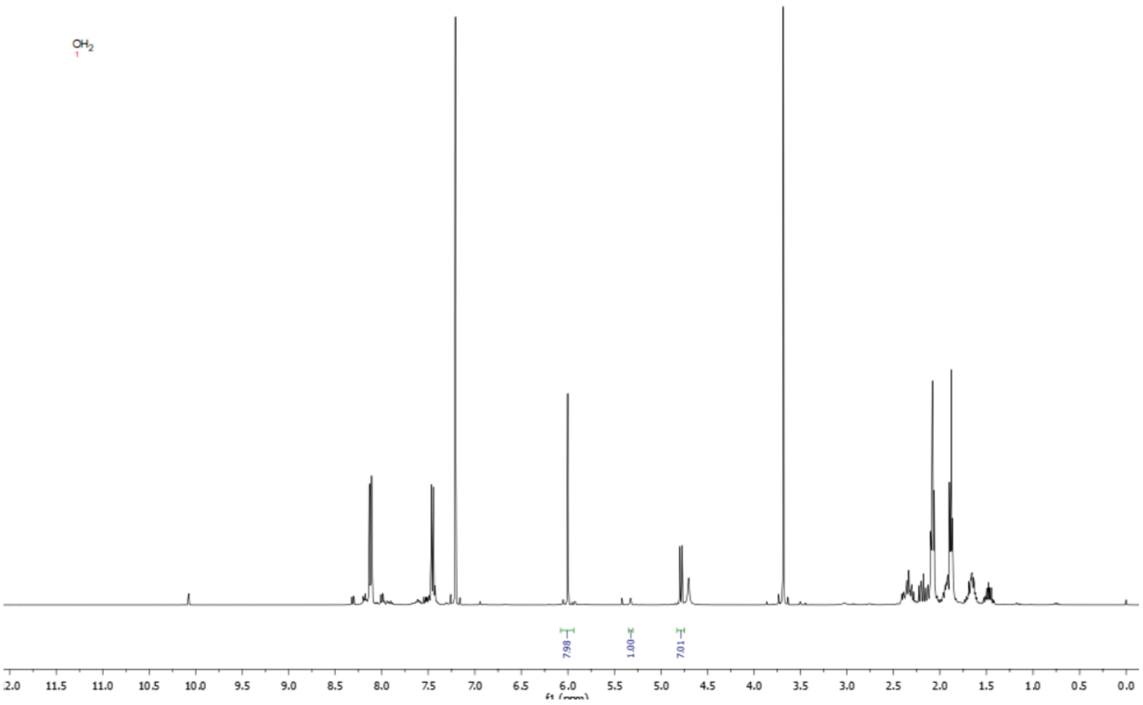

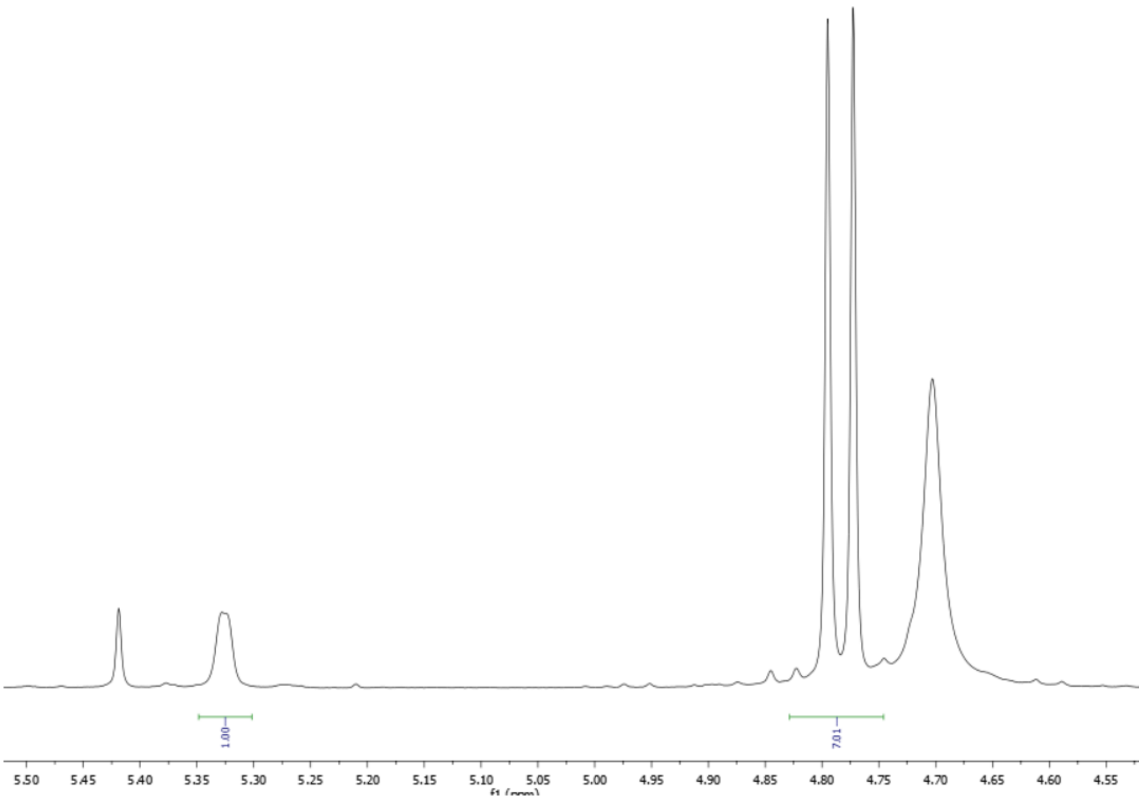

3-CF$_3$-phenylboronic acid (2 eq cyclopentanone, overnight reaction - duplicate) (400 MHz, CDCl$_3$**)**

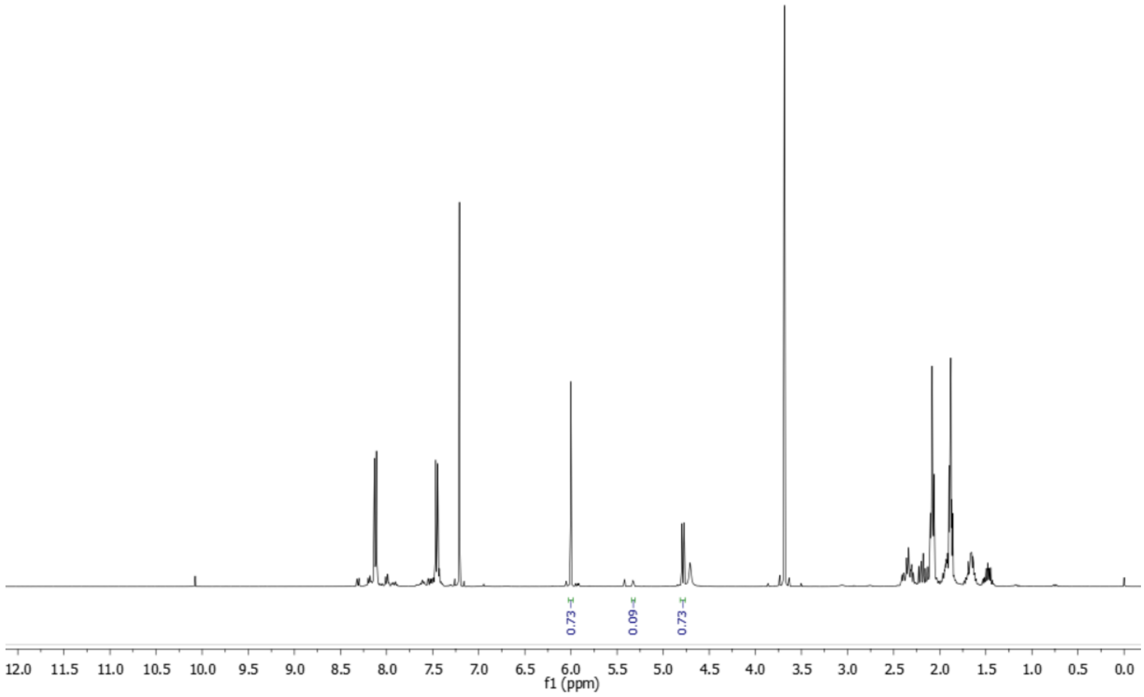

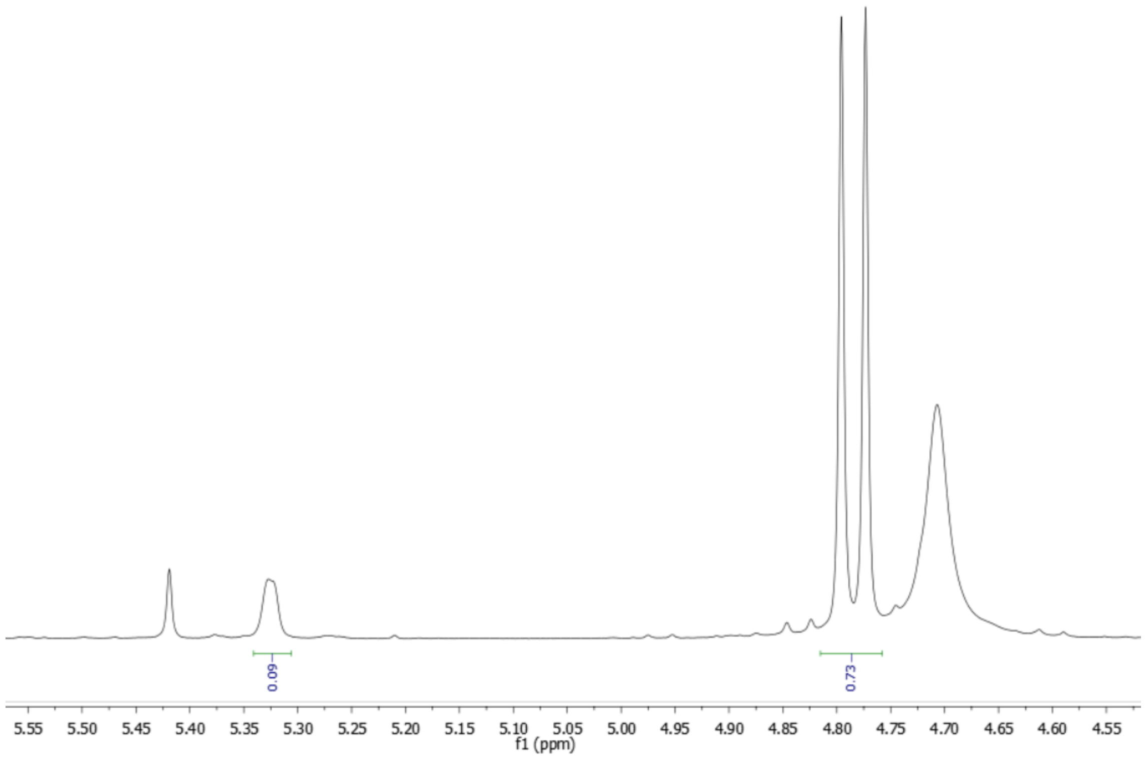

<u>18.8. NMR Traces for Table S8. Evaluating the influence of boronic acid in methyl prolinate catalyzed aldol reaction</u>

No phenylboronic acid

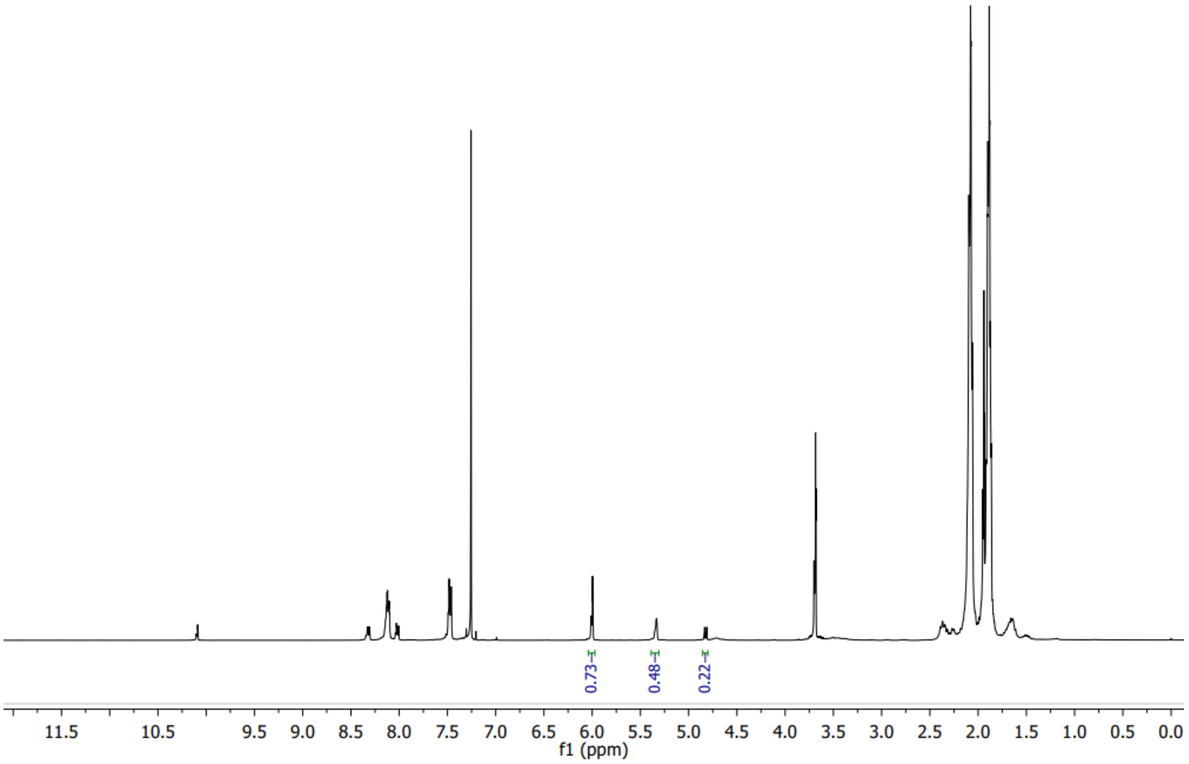

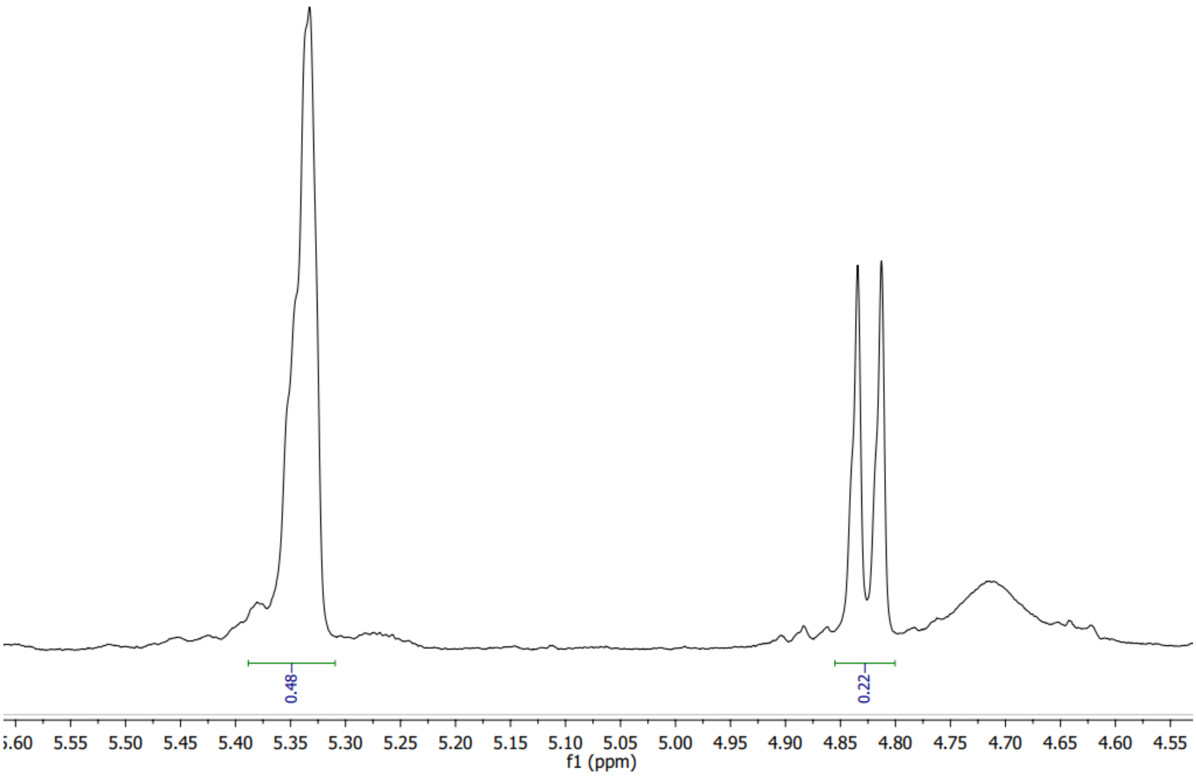

No phenylboronic acid - duplicate

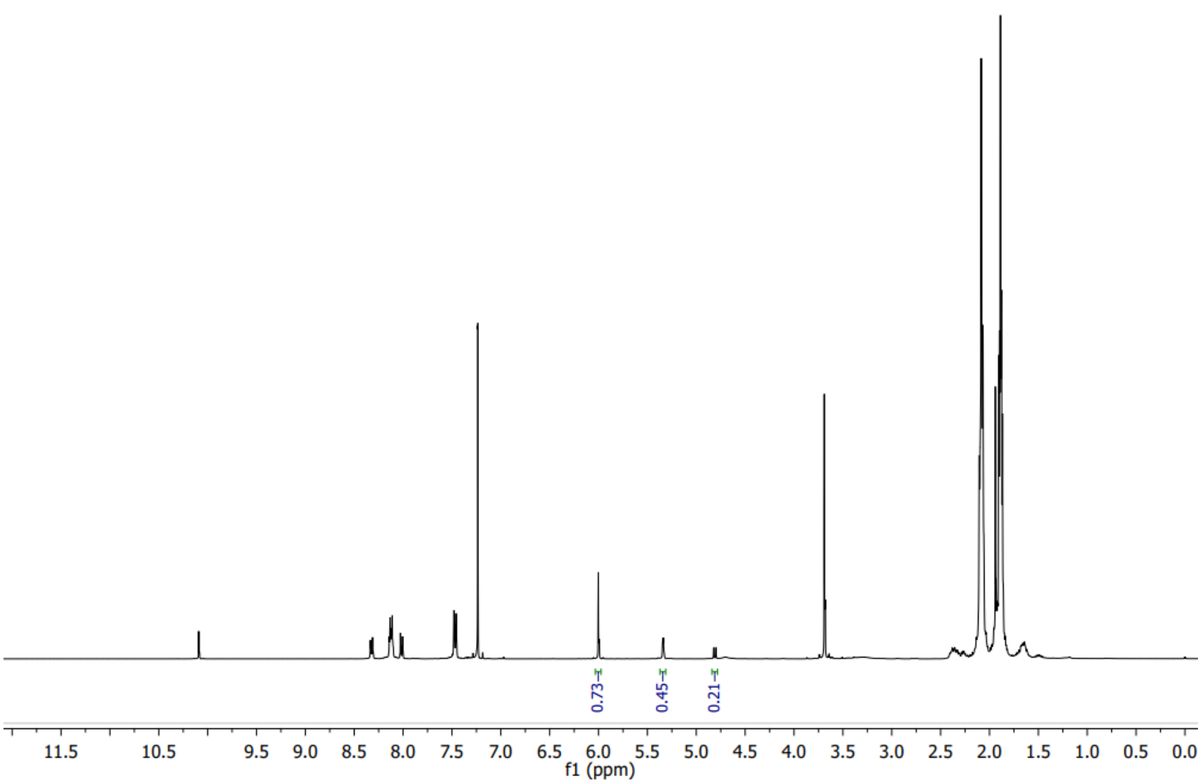

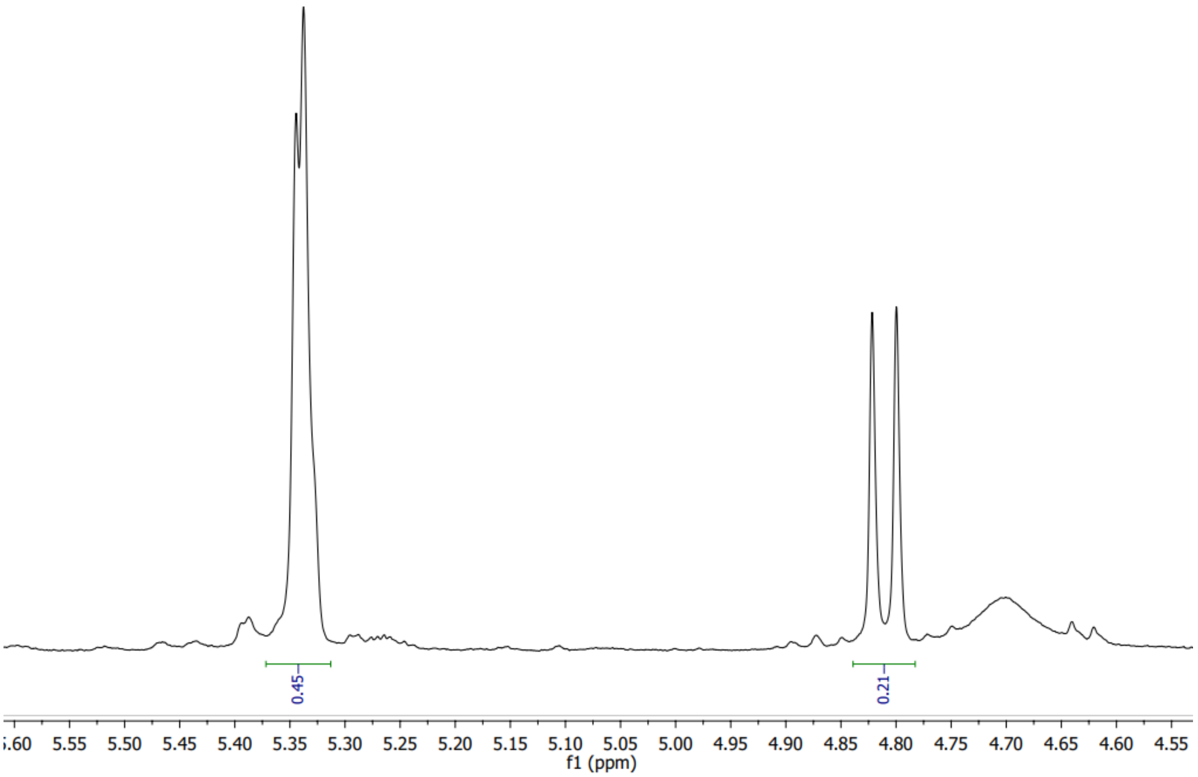

2-F-phenylboronic acid

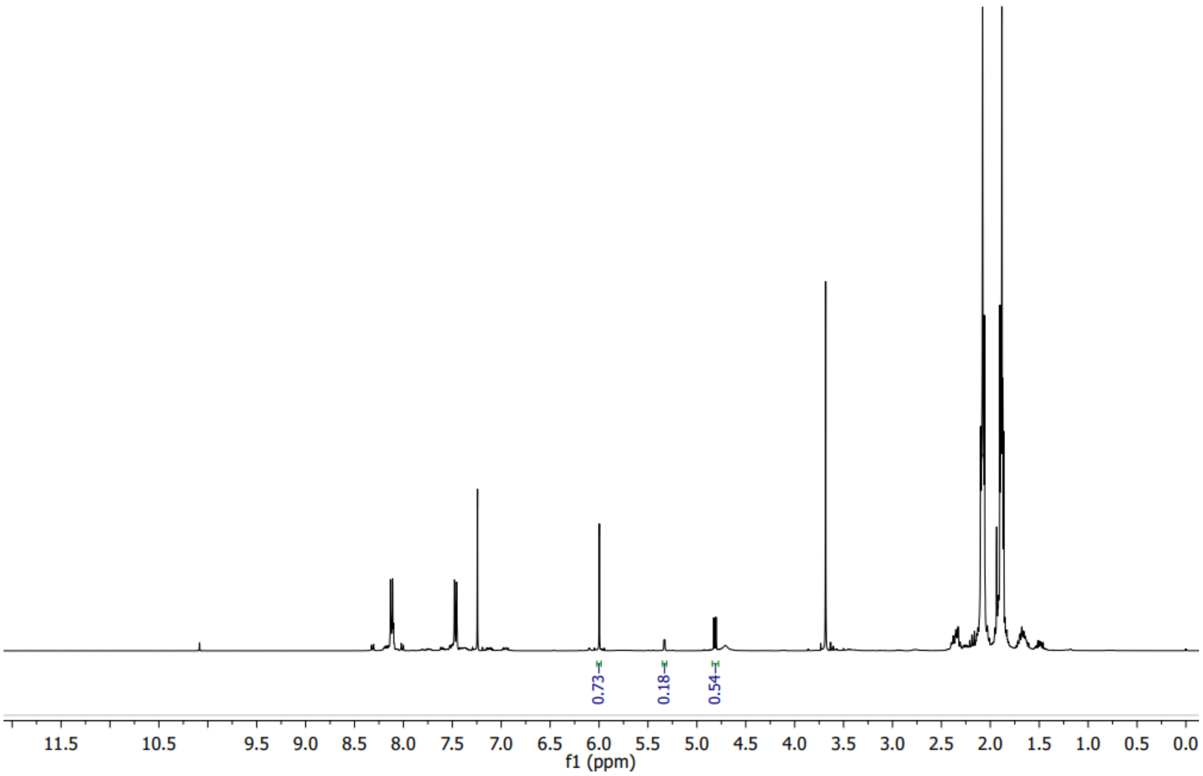

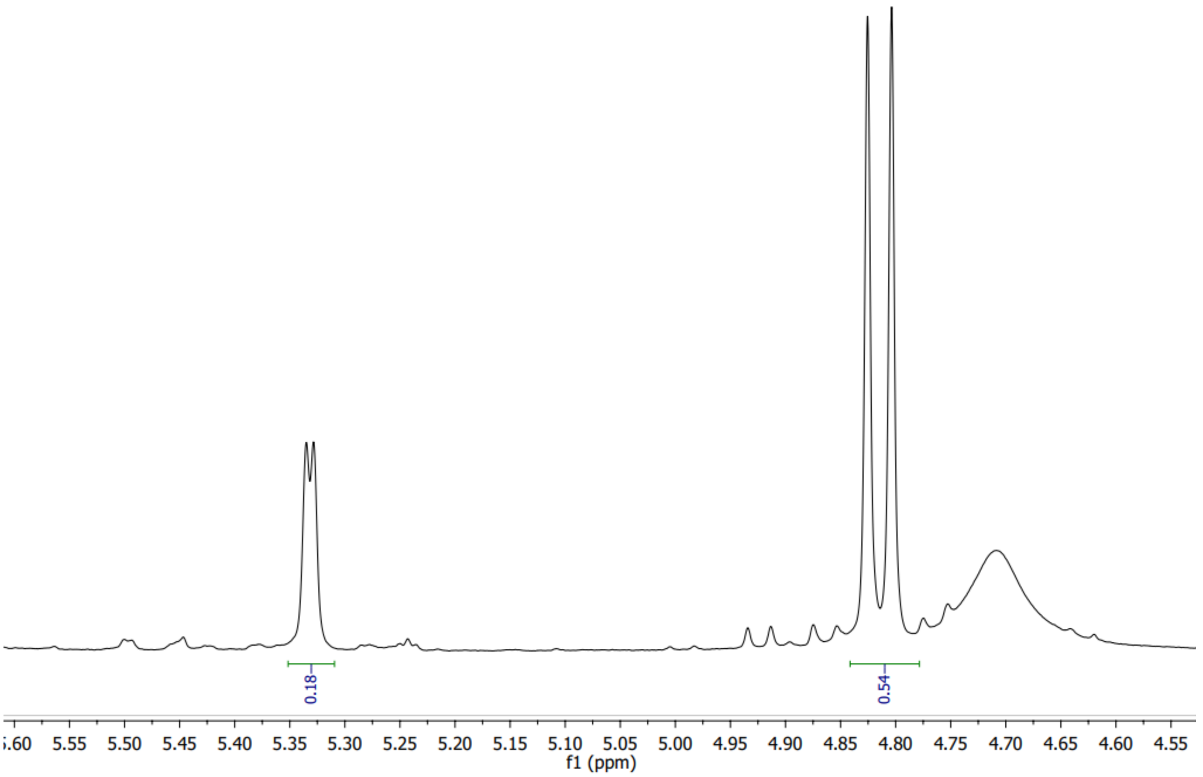

2-F-phenylboronic acid - duplicate

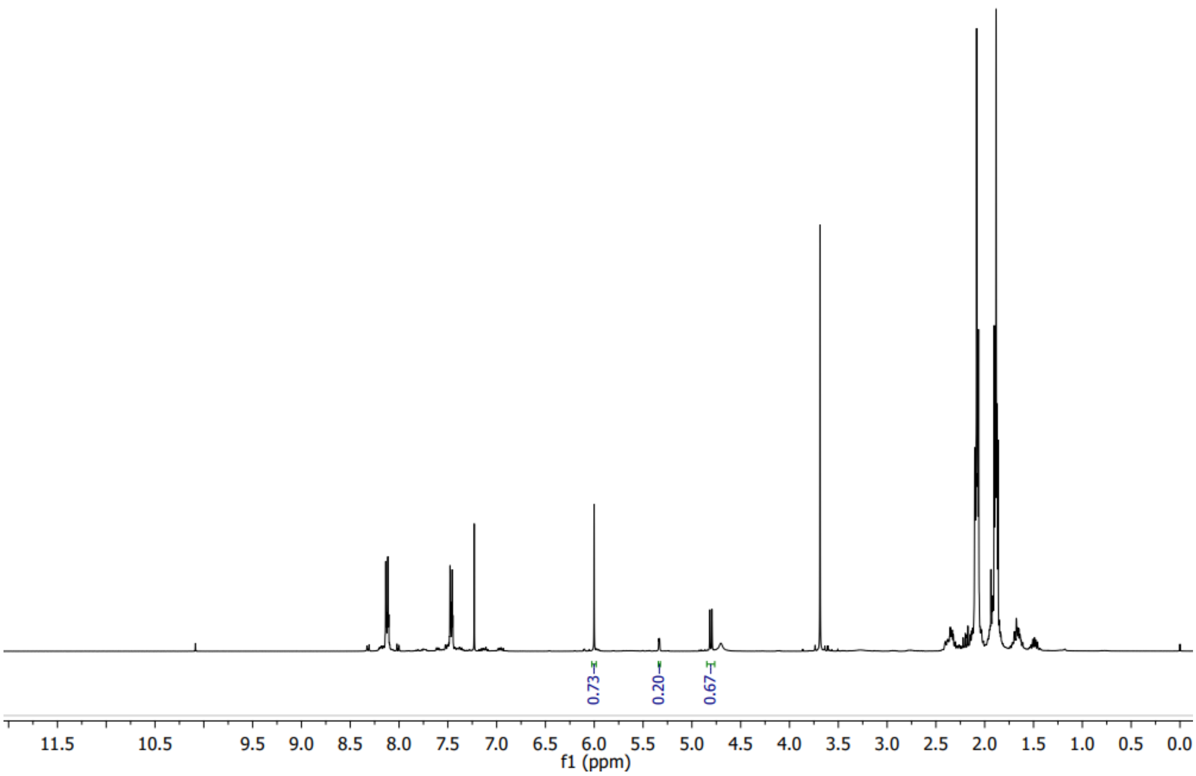

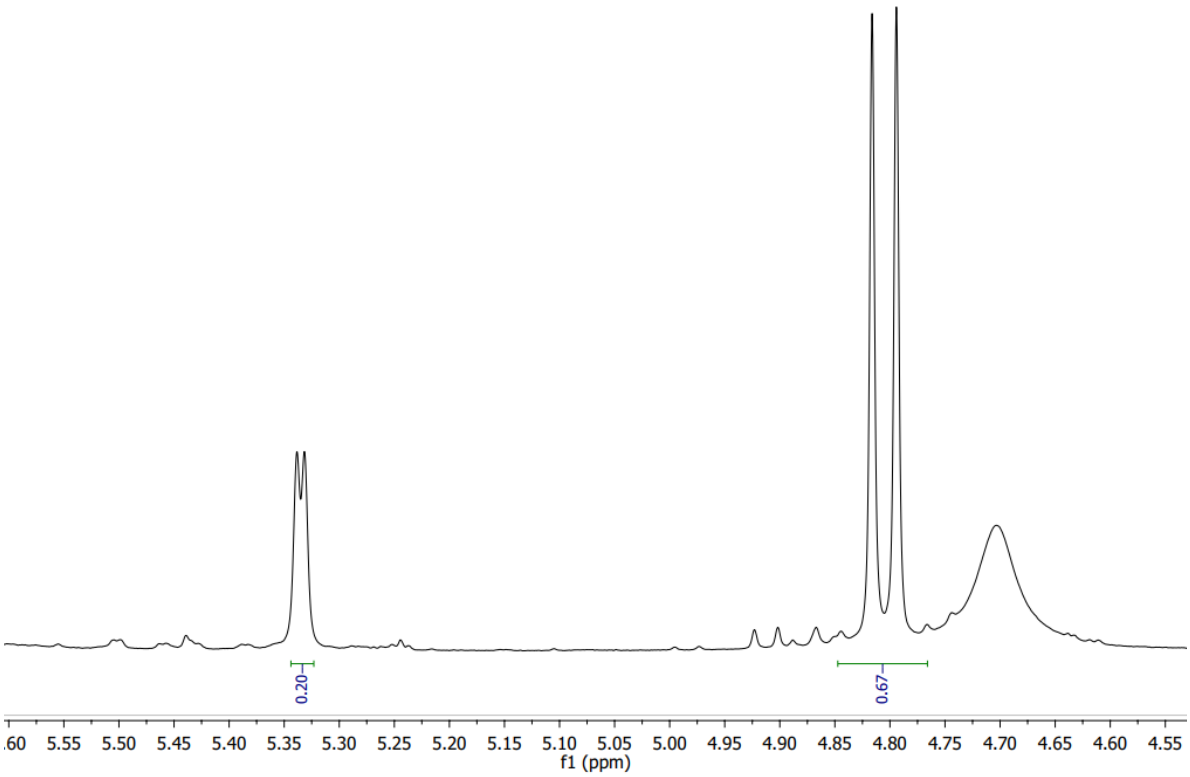

2,4-Me-phenylboronic acid

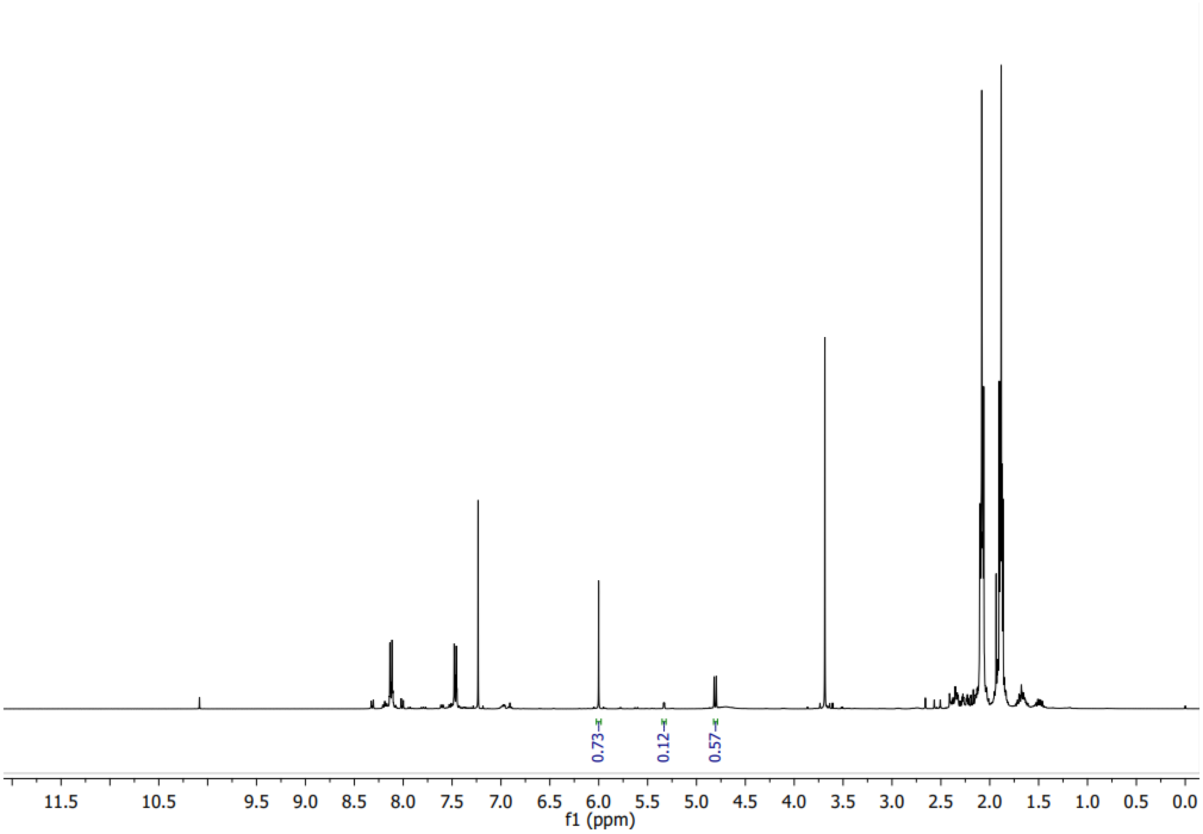

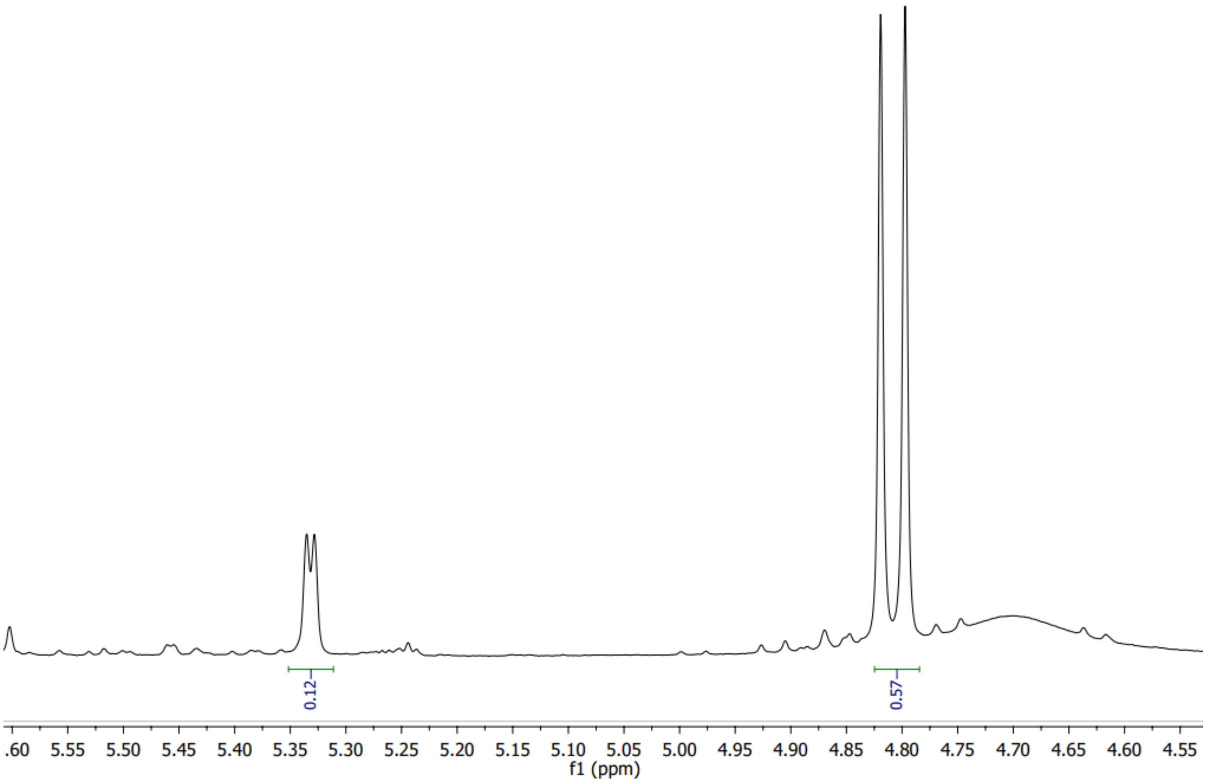

2,4-Me-phenylboronic acid - duplicate

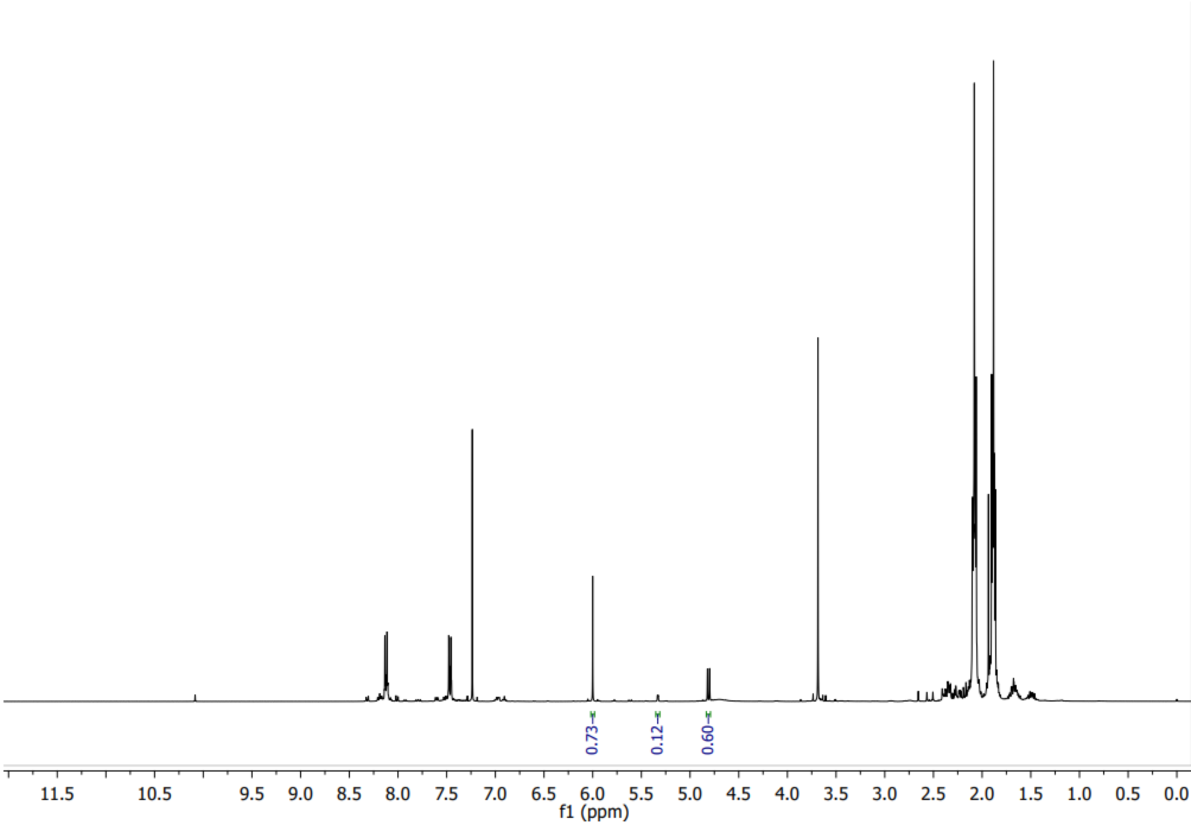

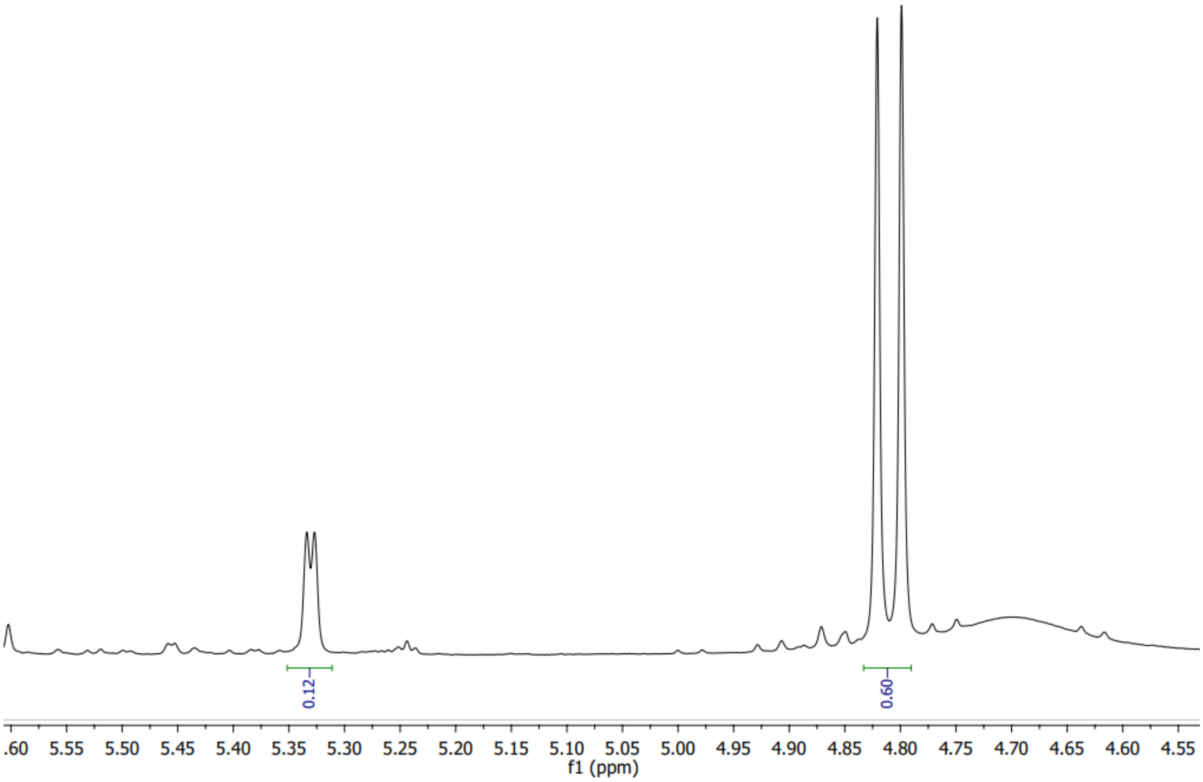

3.5-OMe-phenylboronic acid

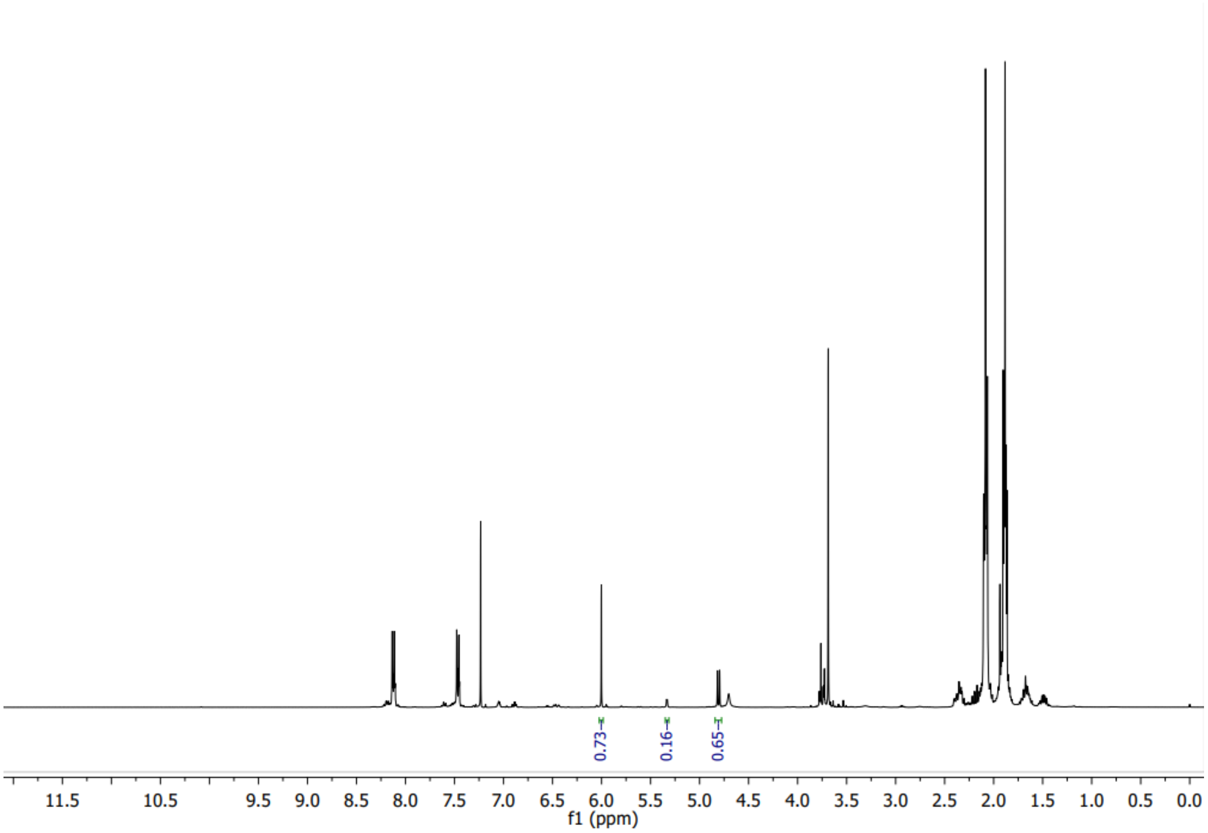

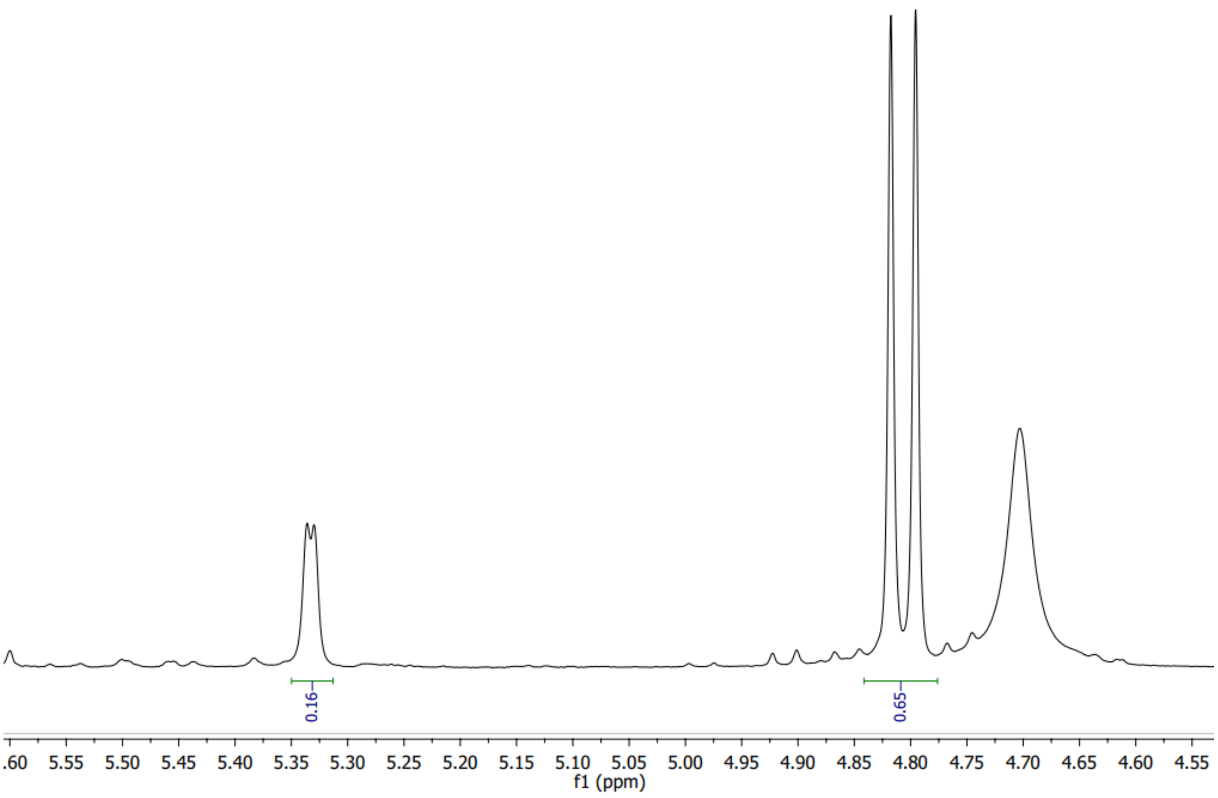

3.5-OMe-phenylboronic acid

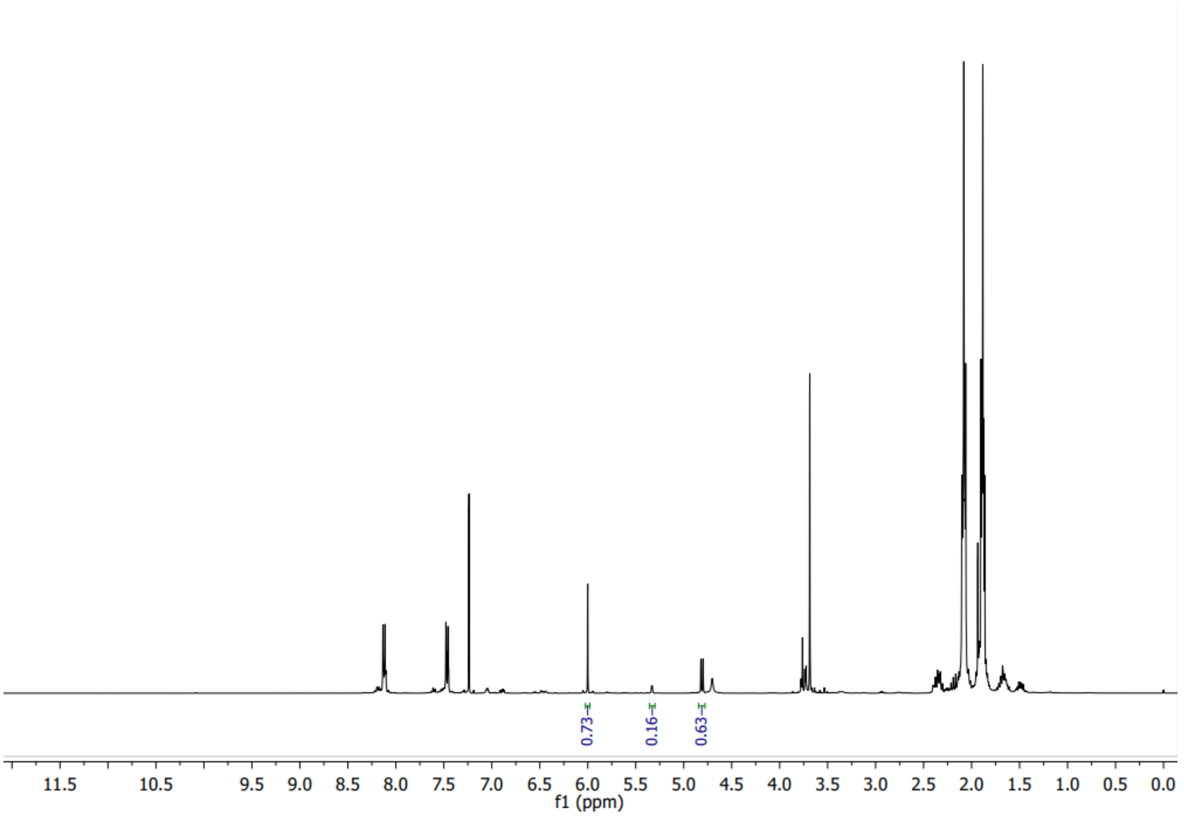

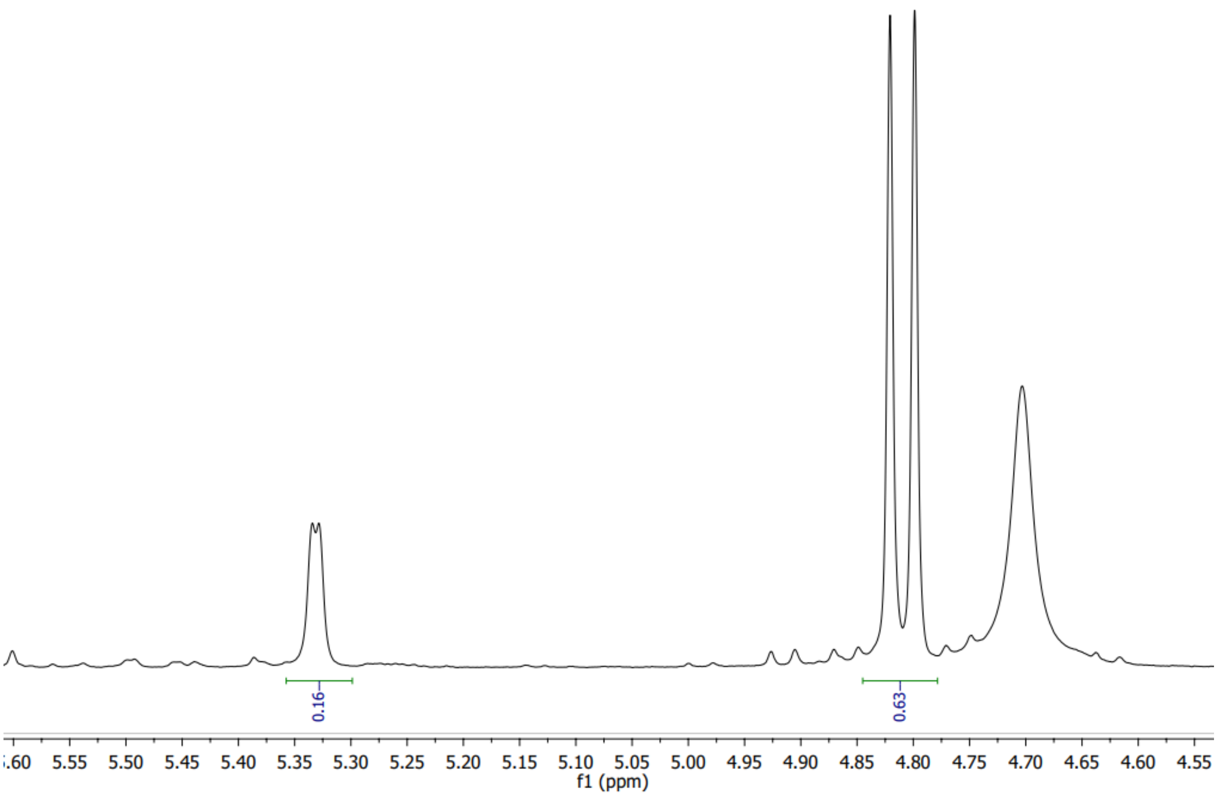

18. High-resolution (HR)-MS experiments

Acetonitrile, Methanol and Chloroform were used as solvent to prepare different samples for HR-MS experiments. Each sample was prepared by mixing 1 equivalent of proline, 1 equivalent of 2-fluorophenyl boronic acid and 0.1 mL of cyclopentanone (2.3 equivalents). Cyclopentanone was used in lower equivalents than the 6.8 equivalents used to build the main reaction sets in order to avoid saturation of this compound on the mass spectrometer. To this mixture, 0.8 mL of one of the three solvents mentioned above and 1 equivalent of water were also added.

The same samples were also prepared without the addition of water in order to evaluate the water influence on the formation of intermediates. Unfortunately, Hexane could not be used as solvent for these analyses once the solubility of the compounds in this solvent was low.

Below you can find an analysis of the results. First, we present the whole mass window, without any specific zoom and assign structures to the main observed peaks. Next, we search among the less intense peaks for specific structures that we hypothesized could be found as intermediates in our reaction medium.

Experiment 1: ACN without water

The most intense peaks to be observed were attributed to the free enamine with addition of a proton (182.1174), the complex [proline+Na], with a mass of 138.1275 and [cyclopentanone+H] (85.0646)

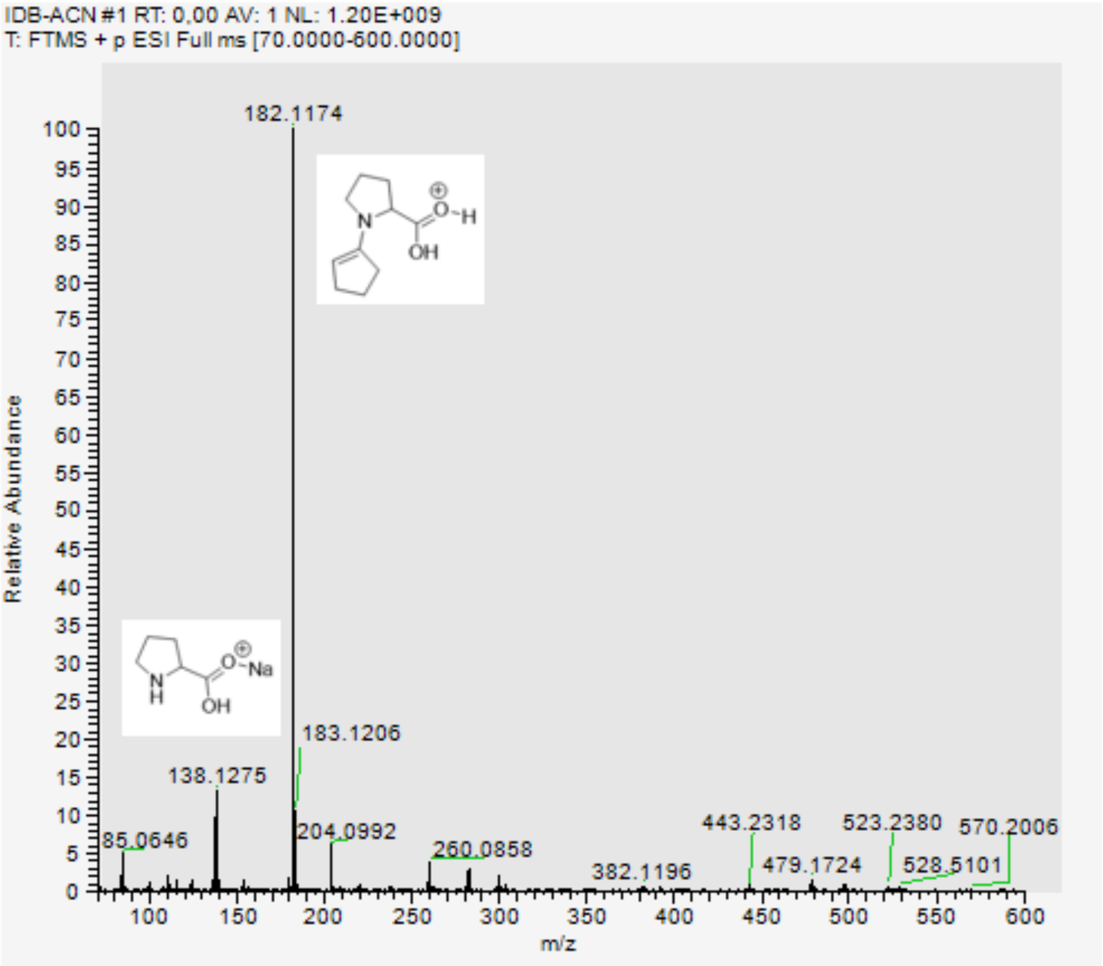

While searching for specific peaks, the following intermediates were suggested to fit the observed mass:

a- *Proline-boronic acid covalently bound*. In addition to the spectrogram shown below, the corresponding mass for sodium and potassium adducts were identified.

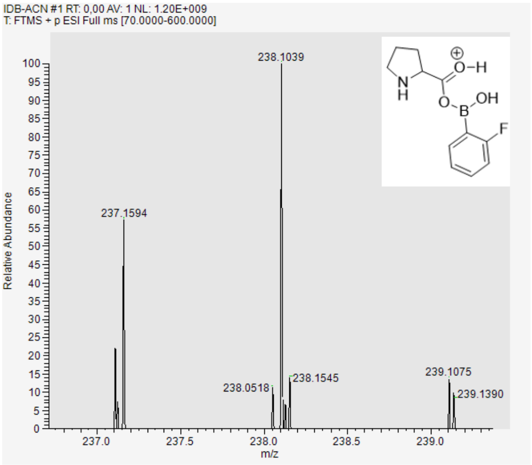

b- *Enamine-boronic acid covalently bound*.

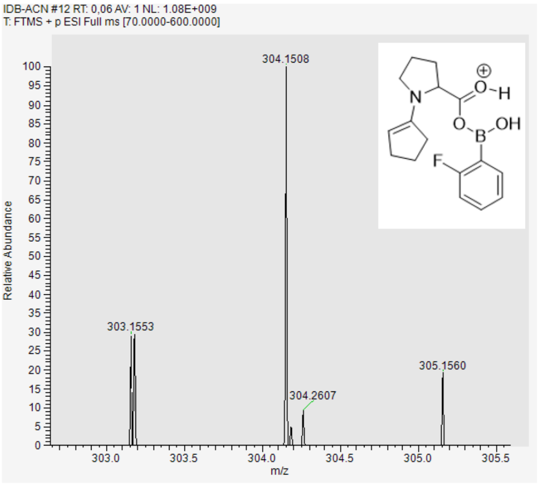

Experiment 2: ACN with water

The addition of water did not seem to have much influence on the observed intermediates. In addition to the species already described in Experiment 1, a species was identified that fits the mass of *1 enamine and 2 boronic acids*

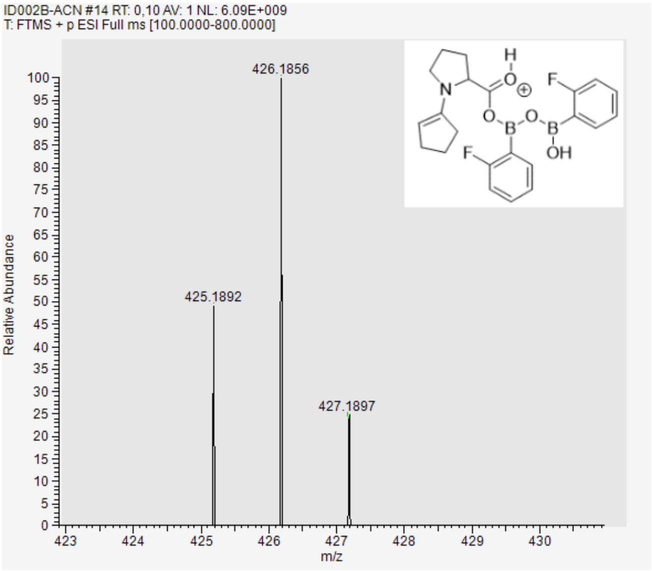

Experiment 3: Methanol without water

The most intense peaks observed were attributed to the complexes [enamine+Na] (204.0993) and [enamine+H] (182.1174).

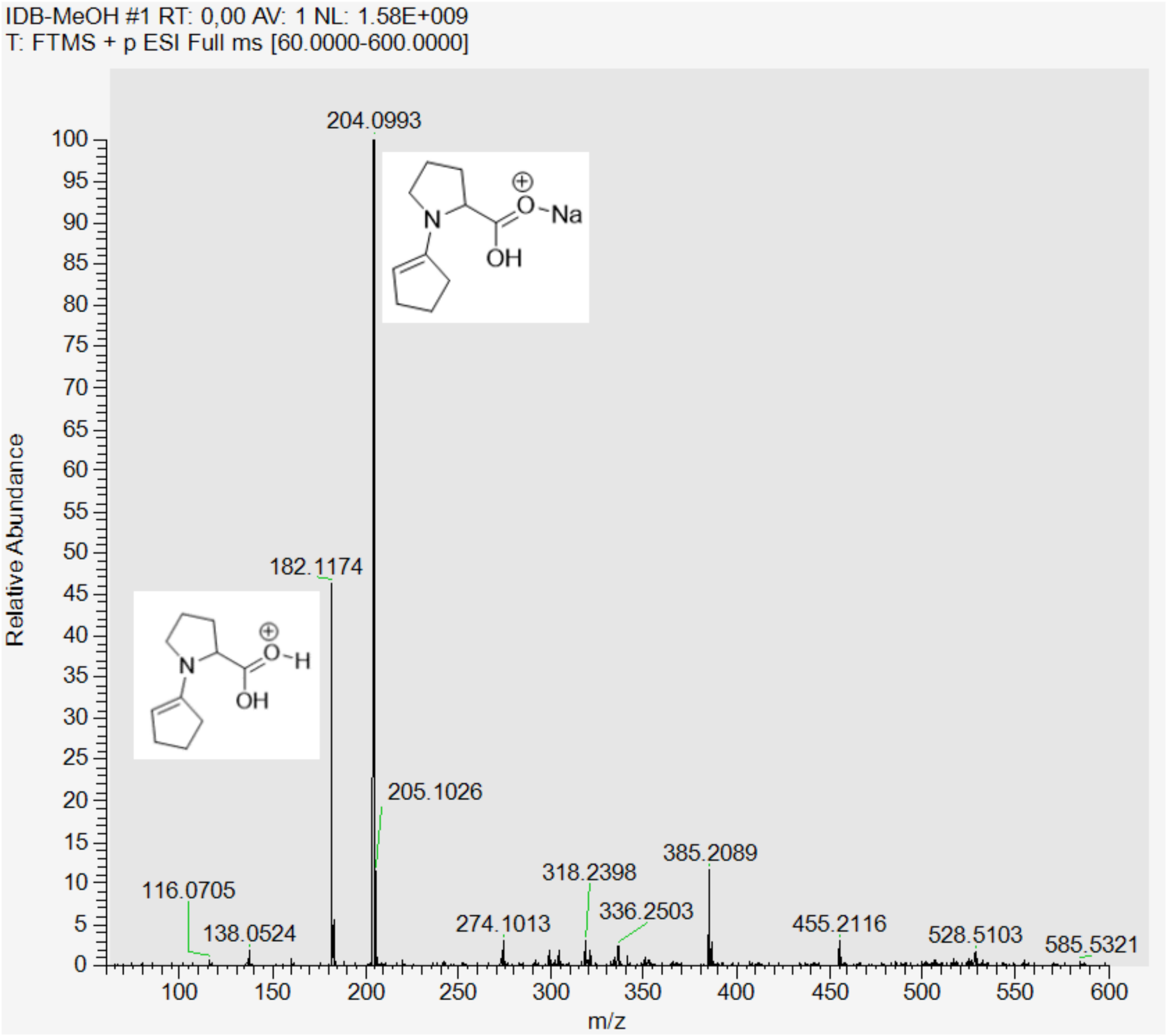

While searching for specific peaks, in the samples prepared in methanol no masses were identified that fit boronic acid adducts without the addition of water.

Experiment 4: Methanol with water

Unlike experiment 3, the addition of water allowed for the detection of several masses that fit boronic acid containing molecules. In this experiment, the most intense peak is attributed to [enamine+H] (182.1174).

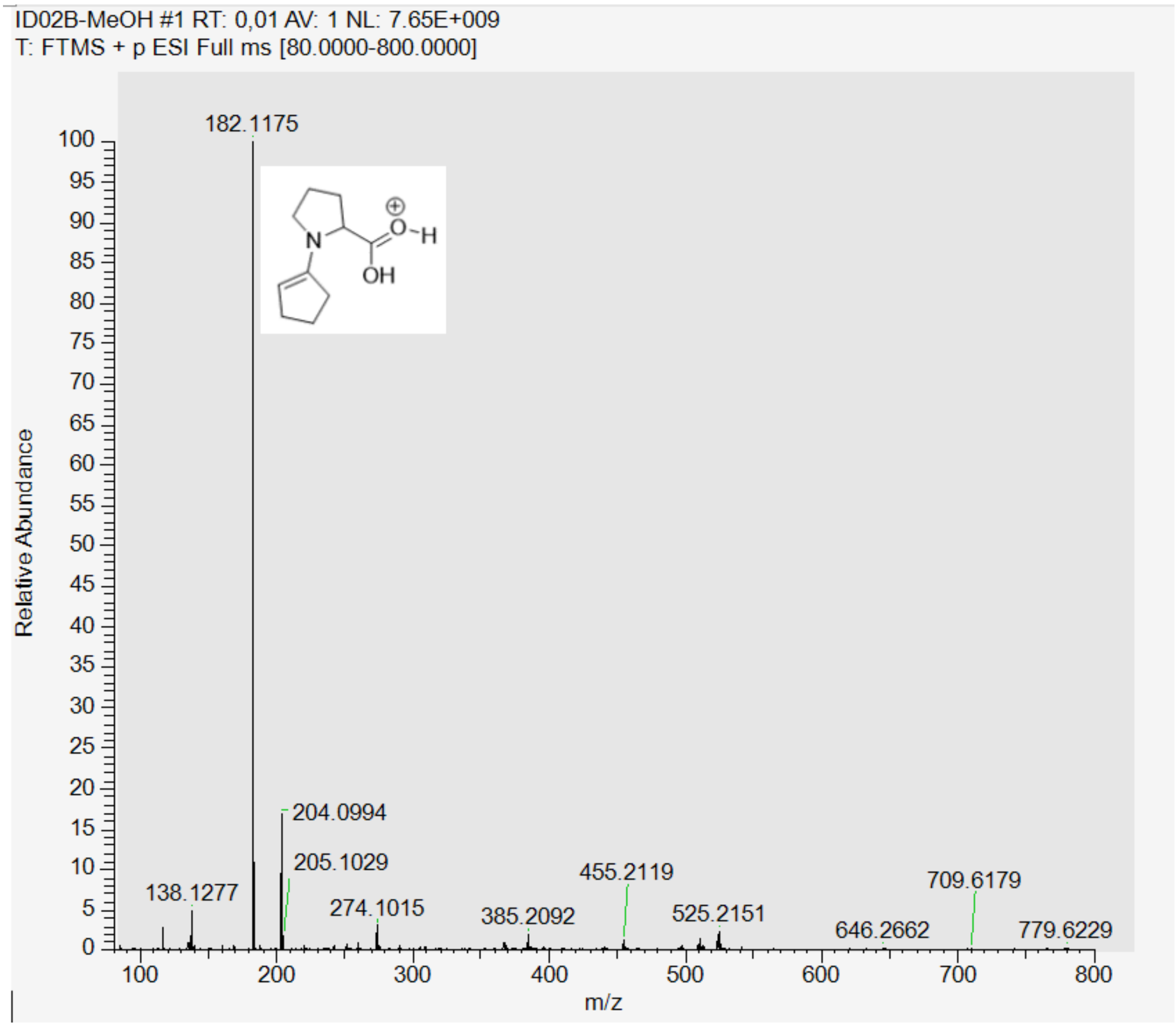

While searching for specific peaks, the following intermediates were suggested to fit the observed mass:

a- *Proline-boronic acid and proline+2 boronic acid* complexes:

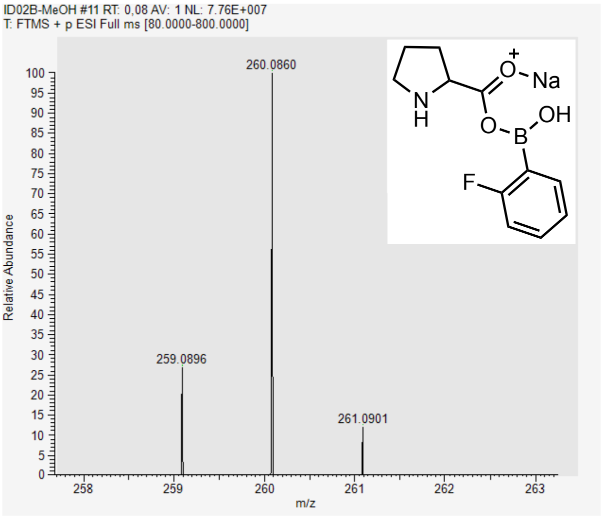

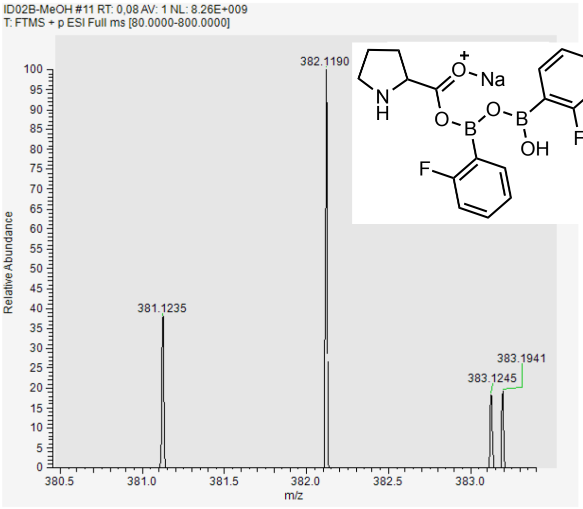

b- *Enamine-boronic acid*:

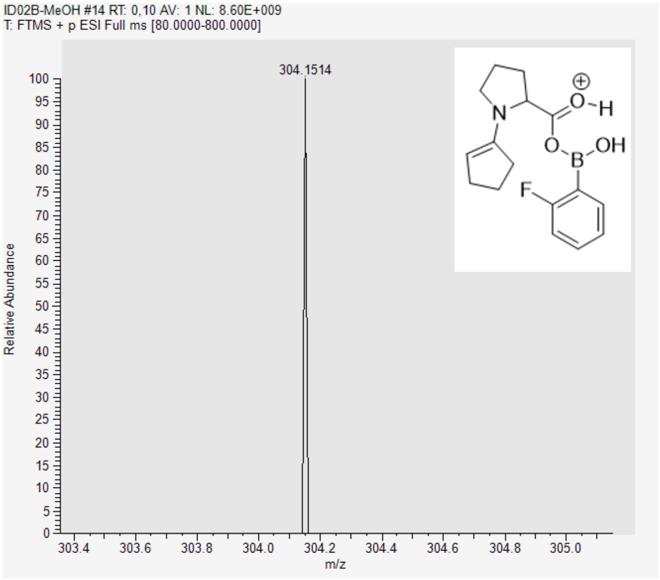

Experiment 5: Chloroform without water

In this experiment, the main peak that could be observed was attributed to the free enamine with 182.1175 and 214 that was attributed to the complex [Enamine+CH3OH+H]

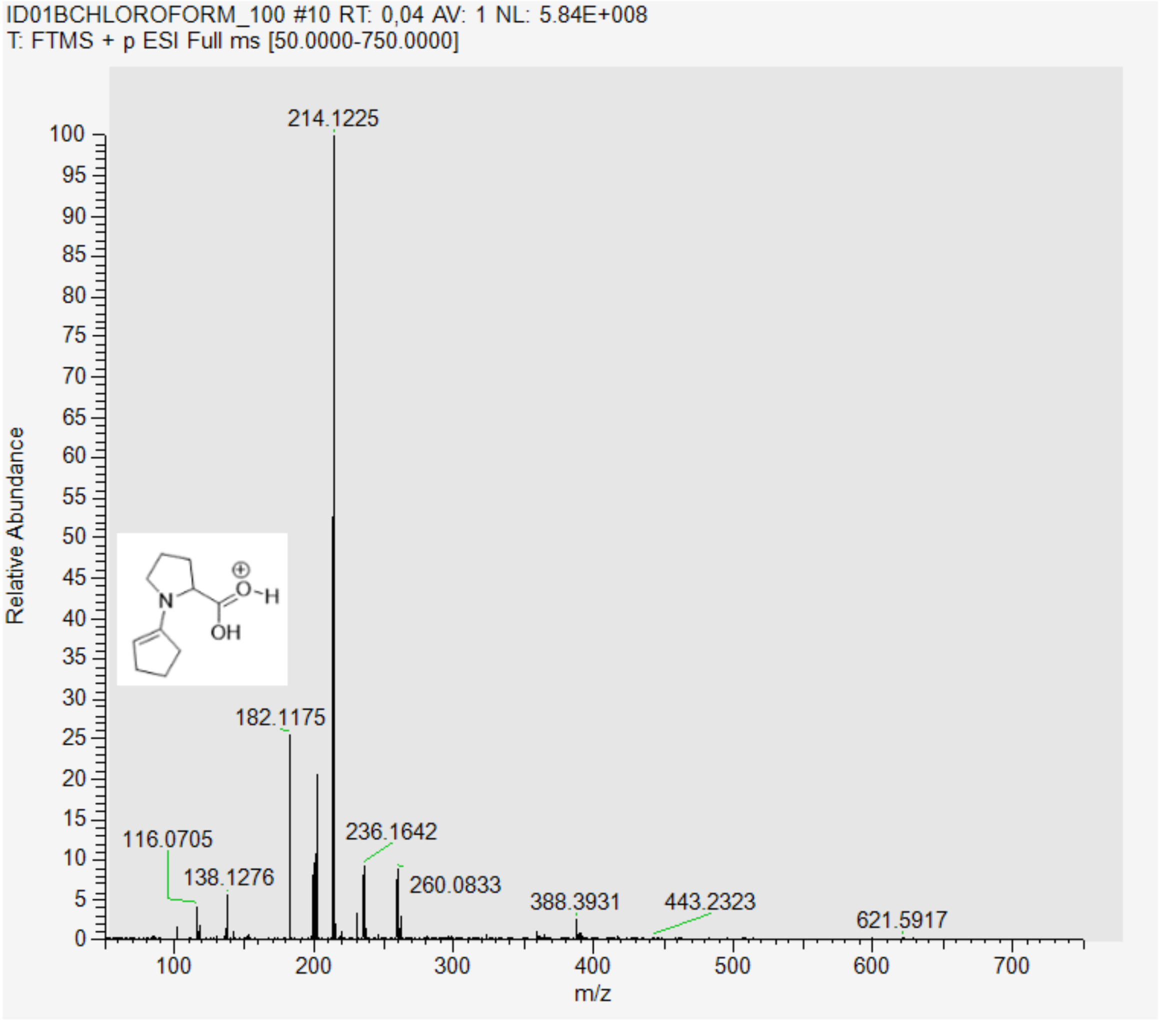

Similarly to the experiments with acetonitrile, even before the addition of water, species containing bound boronic acid could be assigned.

While searching for specific peaks, the following intermediates were suggested to fit the observed mass:

a- *Proline-boronic acid and enamine+boronic acid* complexes:

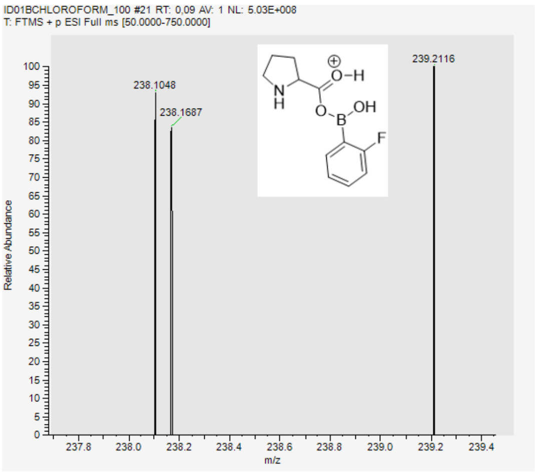

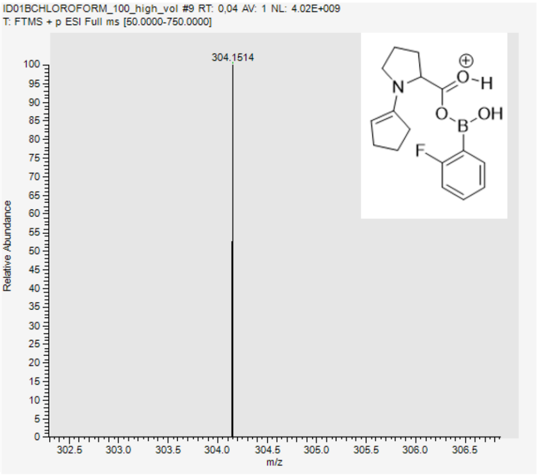

Experiment 6: Chloroform with water

The addition of water did not seem to have much influence on the observed intermediates. In addition to the intermediates already described in Experiment 5, a species was identified that fits the mass of *1 enamine and 2 boronic acids*

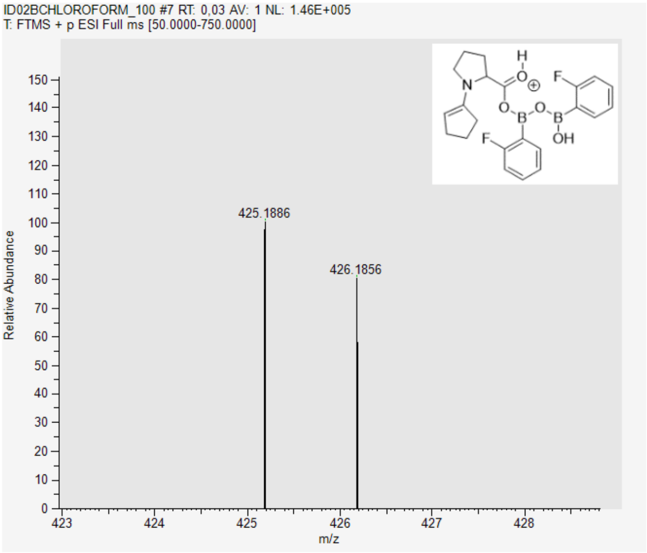

19. NMR of isolated (S)-2-((R)-hydroxy(4-nitrophenyl)methyl)cyclopentan-1-one

1H NMR of (S)-2-((R)-hydroxy(4-nitrophenyl)methyl)cyclopentan-1-one (400 MHz, $CDCl_3$)

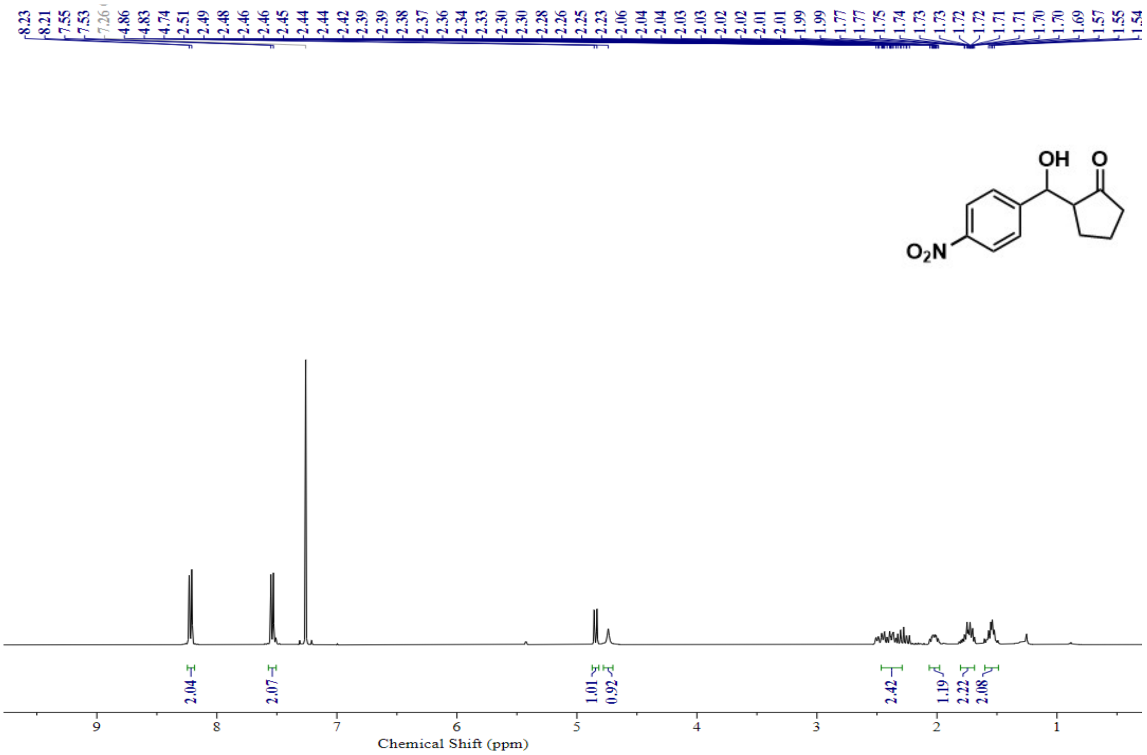

13C{1H} NMR of (S)-2-((R)-hydroxy(4-nitrophenyl)methyl)cyclopentan-1-one (100 MHz, $CDCl_3$)

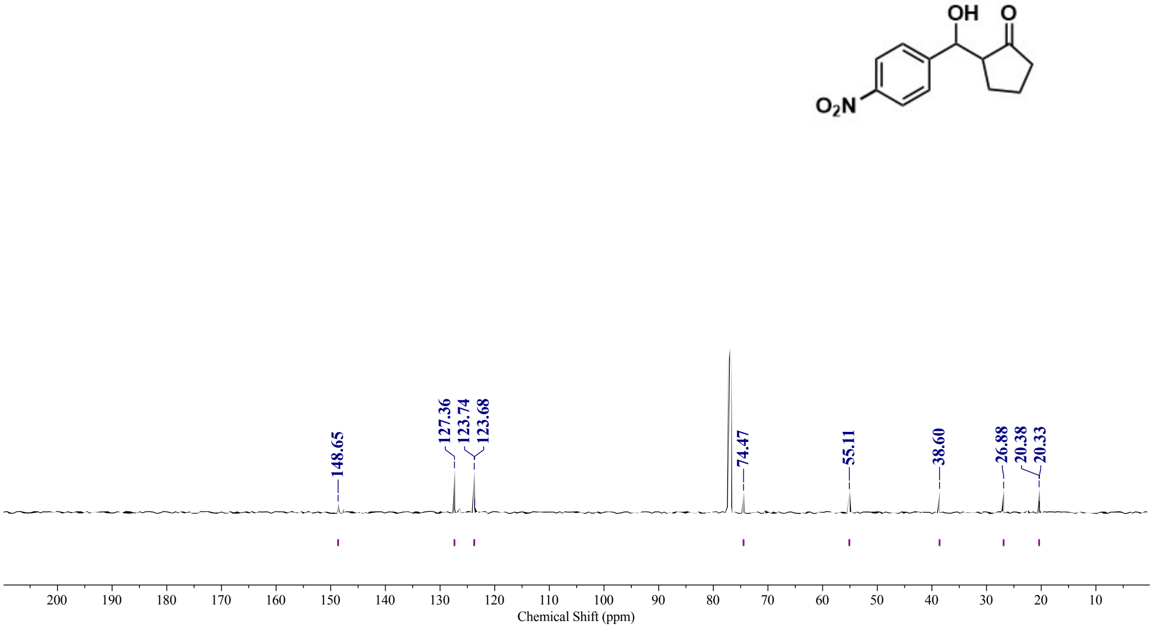

20. References – Experimental Supporting Information

(1) Li, R.; Liu, F.; Dong, G. Redox-Neutral Ortho Functionalization of Aryl Boroxines via Palladium/Norbornene Cooperative Catalysis. *Chem* **2019**, *5* (4), 929–939. https://doi.org/10.1016/j.chempr.2019.02.005.

(2) So, C. M.; Kume, S.; Hayashi, T. Rhodium-Catalyzed Asymmetric Hydroarylation of 3-Pyrrolines Giving 3-Arylpyrrolidines: Protonation as a Key Step. *J. Am. Chem. Soc.* **2013**, *135* (30), 10990–10993. https://doi.org/10.1021/ja406169s.

(3) Guan, B. T.; Wang, Y.; Li, B. J.; Yu, D. G.; Shi, Z. J. Biaryl Construction via Ni-Catalyzed C-O Activation of Phenolic Carboxylates. *J. Am. Chem. Soc.* **2008**, *130* (44), 14468–14470. https://doi.org/10.1021/ja8056503.

(4) Wu, C.; Qin, X.; Moeljadi, A. M. P.; Hirao, H.; Zhou, J. S. Copper-Catalyzed Asymmetric Arylation of N-Heteroaryl Aldimines: Elementary Step of a 1,4-Insertion. *Angew. Chemie - Int. Ed.* **2019**, *58* (9), 2705–2709. https://doi.org/10.1002/anie.201812646.

(5) Crampton, R.; Woodward, S.; Fox, M. Bis-Sulfamyl Imines: Potent Substrates for Asymmetric Additions of Arylboroxines under Rhodium Catalysis. *Adv. Synth. Catal.* **2011**, *353* (6), 903–906. https://doi.org/10.1002/adsc.201000838.

(6) Smith, M. K.; Northrop, B. H. Vibrational Properties of Boroxine Anhydride and Boronate Ester Materials: Model Systems for the Diagnostic Characterization of Covalent Organic Frameworks. *Chem. Mater.* **2014**, *26* (12), 3781–3795. https://doi.org/10.1021/cm5013679.

(7) Knecht, T.; Pinkert, T.; Dalton, T.; Lerchen, A.; Glorius, F. CpRh III -Catalyzed Allyl-Aryl Coupling of Olefins and Arylboron Reagents Enabled by C(Sp 3)-H Activation. *ACS Catal.* **2019**, *9* (2), 1253–1257. https://doi.org/10.1021/acscatal.8b04677.

(8) Sun, C. L.; Yang, W.; Xiao, Z.; Wu, Z. H.; Li, B. J.; Guan, B. T.; Shi, Z. J. Construction of Polysubstituted Olefins through Ni-Catalyzed Direct Activation of Alkenyl C-O of Substituted Alkenyl Acetates. *Chem. - A Eur. J.* **2010**, *16* (20), 5844–5847. https://doi.org/10.1002/chem.200902785.

(9) Zhou, Q.; Srinivas, H. D.; Dasgupta, S.; Watson, M. P. Nickel-Catalyzed Cross-Couplings of Benzylic Pivalates with Arylboroxines: Stereospecific Formation of Diarylalkanes and Triarylmethanes. *J. Am. Chem. Soc.* **2013**, *135* (9), 3307–3310. https://doi.org/10.1021/ja312087x.

(10) Xiao, Q.; Tian, L.; Tan, R.; Xia, Y.; Qiu, D.; Zhang, Y.; Wang, J. Transition-Metal-Free Electrophilic Amination of Arylboroxines. *Org. Lett.* **2012**, *14* (16), 4230–4233. https://doi.org/10.1021/ol301912a.

(11) Li, R.; Liu, F.; Dong, G. Redox-Neutral Ortho Functionalization of Aryl Boroxines via Palladium/Norbornene Cooperative Catalysis. *Chem* **2019**, *5* (4), 929–939. https://doi.org/10.1016/j.chempr.2019.02.005.

(12) So, C. M.; Kume, S.; Hayashi, T. Rhodium-Catalyzed Asymmetric Hydroarylation of 3-Pyrrolines Giving 3-Arylpyrrolidines: Protonation as a Key Step. *J. Am. Chem. Soc.* **2013**, *135* (30), 10990–10993. https://doi.org/10.1021/ja406169s

(13) Guan, B. T.; Wang, Y.; Li, B. J.; Yu, D. G.; Shi, Z. J. Biaryl Construction via Ni-Catalyzed C-O Activation of Phenolic Carboxylates. *J. Am. Chem. Soc.* **2008**, *130* (44), 14468–14470. https://doi.org/10.1021/ja8056503.

(14) Wu, C.; Qin, X.; Moeljadi, A. M. P.; Hirao, H.; Zhou, J. S. Copper-Catalyzed Asymmetric Arylation of N-Heteroaryl Aldimines: Elementary Step of a 1,4-Insertion. *Angew. Chemie - Int. Ed.* **2019**, *58* (9), 2705–2709. https://doi.org/10.1002/anie.201812646.

(15) Crampton, R.; Woodward, S.; Fox, M. Bis-Sulfamyl Imines: Potent Substrates for Asymmetric Additions of Arylboroxines under Rhodium Catalysis. *Adv. Synth. Catal.* **2011**, *353* (6), 903–906. https://doi.org/10.1002/adsc.201000838.

(16) Smith, M. K.; Northrop, B. H. Vibrational Properties of Boroxine Anhydride and Boronate Ester Materials: Model Systems for the Diagnostic Characterization of Covalent Organic Frameworks. *Chem. Mater.* **2014**, *26* (12), 3781–3795. https://doi.org/10.1021/cm5013679.

(17) Knecht, T.; Pinkert, T.; Dalton, T.; Lerchen, A.; Glorius, F. CpRh III -Catalyzed Allyl-Aryl Coupling of Olefins and Arylboron Reagents Enabled by C(Sp 3)-H Activation. *ACS Catal.* **2019**, *9* (2), 1253–1257. https://doi.org/10.1021/acscatal.8b04677.

(18) Sun, C. L.; Yang, W.; Xiao, Z.; Wu, Z. H.; Li, B. J.; Guan, B. T.; Shi, Z. J. Construction of Polysubstituted Olefins through Ni-Catalyzed Direct Activation of Alkenyl C-O of Substituted Alkenyl Acetates. *Chem. - A Eur. J.* **2010**, *16* (20), 5844–5847. https://doi.org/10.1002/chem.200902785.

(19) Zhou, Q.; Srinivas, H. D.; Dasgupta, S.; Watson, M. P. Nickel-Catalyzed Cross-Couplings of Benzylic Pivalates with Arylboroxines: Stereospecific Formation of Diarylalkanes and Triarylmethanes. *J. Am. Chem. Soc.* **2013**, *135* (9), 3307–3310. https://doi.org/10.1021/ja312087x.

(20) Xiao, Q.; Tian, L.; Tan, R.; Xia, Y.; Qiu, D.; Zhang, Y.; Wang, J. Transition-Metal-Free Electrophilic Amination of Arylboroxines. *Org. Lett.* **2012**, *14* (16), 4230–4233. https://doi.org/10.1021/ol301912a.

21. Multivariate Modelling Supporting Information

All .xyz files, code and datasets used in this work are provided online:

https://github.com/Milo-group/SolEffects

Geometry optimizations and frequency calculations were carried out using Gaussian 16 software.[1] The functional used for DFT calculation is M06-2X, which was previously benchmarked for thermodynamic and kinetic accuracy of main group elements, and for non-covalent interactions.[2,3] A triple zeta potential basis-set (def2-TZVP)[4,5] was chosen based on Zhao and Truhlar's evaluation of the M06-2X functional for organic molecules, indicating that a triple zeta quality is generally more quantitative.[5] As this study seeks correlations, we opted not to incorporate scaling factors for vibrational terms.[6] This simplification was justified by the assumption that a constant scaling factor would neither change the descriptive parameters identified, nor the relationship between them. Sterimol values were calculated for the geometry optimized structures with the use of our in-house developed Sterimol program (available at https://github.com/Milo-group/SteRimol), based on Verloop's original definitions.[7] Sterimol values were calculated along the substituent axis such that the principal axis begins on the boronic acid's boron. Thus, L is the added length of the substituted ring and the boron–carbon bond distance, B_1 is the minimal width perpendicular to the principal axis L and B_5 is the maximal width perpendicular to the principal axis L (for most of the herein studied aromatic aldehydes, B_1 and B_5 correspond the thickness and the width of the substituted ring respectively).

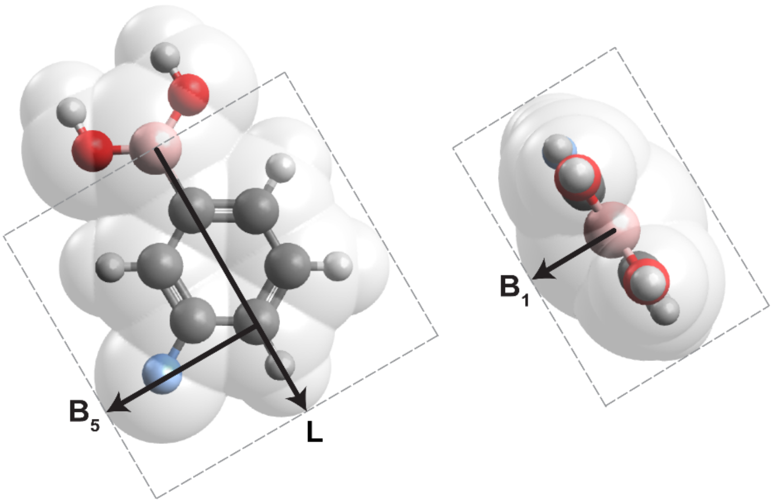

Figure S1. Verloop's Sterimol parameters L, B_1 and B_5, illustrated with 3-fluorophenylboronic acid.

NBO charges were calculated using the NBO 3.1 extension incorporated in Gaussian.[8] Dipole moments were calculated for the geometry optimized structures based on Gaussian's default dipole computation with respect to a consistent Cartesian origin located on the center of the phenyl moiety of the boronic acid, with an orientation such that the Boron atom acts as the y axis and the x axis shares a plane with the substituted ring (see figure S3.). This allows for the use of the dipole cartesian components as parameters. Bond and ring vibrational frequencies, bond lengths, angles and distances between atoms are extracted from Gaussian output.

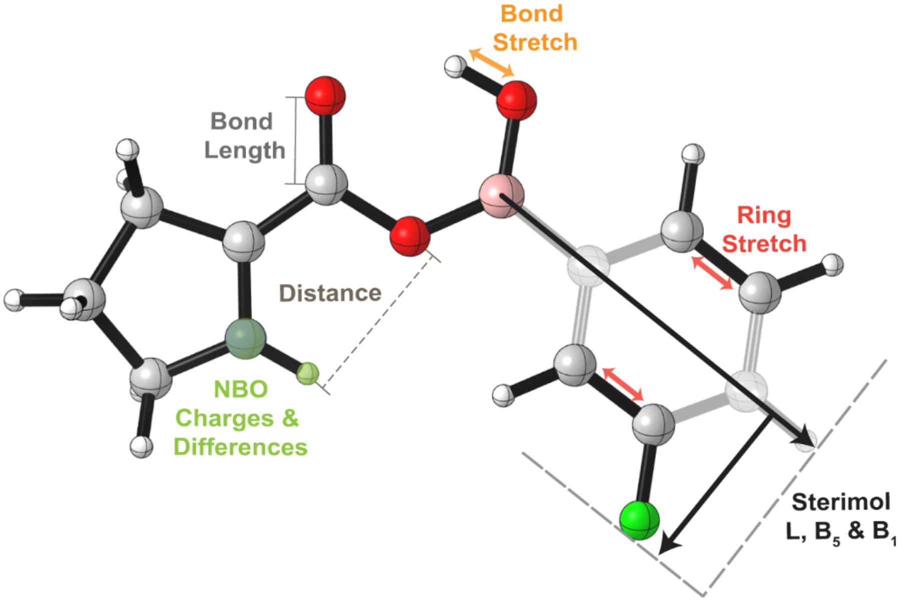

Figure S2. Physical organic parameters used in linear regression.

The Linear regression models were calculated using R (V 3.6.0)[9] and RStudio[10]. Graphic representations were produced using the package ggplot2[11]. Other packages used in code are: tidyr[12], reshape2[13], scales[14], tibble[15], caret[16], plyr[17], dplyr[18], data.table[19], knitr[20], ggrepel[21], and extrafont[22]. CYLview[23] and Avogadro[24] were used in molecular graphic representations.

Model development involved a selection process that evaluates all possible models with a given range for the number of variables. The maximum number of variables is set to be the number of samples divided by 5 whereas the minimum is 1. The resulting models are assessed for their statistical significance and then ranked by a leave-one-out cross-validation. For the best model in each case a general goodness-of-fit measure R^2 is provided together with a leave-one-out cross-validation Q^2, as well as a 3- and 5-fold cross-validation Q^2 (averaged value from 500 iteration of each). To realize this assessment, each parameter was normalized by subtracting its respective mean and dividing by its standard deviation.

The choice of the best performing model was first and foremost based on an internal validation (K-fold cross-validation) in which the data is divided into K subsets (folds) and K models are trained, wherein

each round K-1 folds are used as training data and the remaining set is used as a test set. To evaluate the overall validation, the predicted values from all test sets are measured against the known results, and the Q^2 goodness-of-fit is measured by the models' ability to reproduce results. Leave-one-out cross-validation is a private case of K-fold cross-validation in which the number of folds is the number of samples in the data, such that each sample is treated once as the test set. The Q^2 acts as the validation's R^2 and a good value for Q^2 is a value that is close to the R^2, thus, both measures are used in evaluating the model, where the closer and higher they are, the better the fit.

Parameterization of the molecular library

A molecular library consisting of all the tested boronic acid substitutions was generated for all the proposed structures. In some of the possible structures, the choice of where to position the *ortho* and *meta* substituents holds a substantial effect on physical attributes, as it leads to two distinct rotamers of the same molecule. Thus, we tested two sets that differ with molecules in which the *ortho* and *meta* substituents are positioned such that they are sterically closer to other groups in the structure and those that present less hindrance. In the proline and enamine structure we denote the two sets as "close to the active site" and "far from the active site", while for the non-covalent interaction structures we denote the two sets by the proximity of the boronic acid aryl ring to: (1) the aldehyde aryl ring in the H bonded structure and (2) the aldehyde oxygen in the π interaction structure (Figure S3). A full report of all resulting models is provided in a separate document.

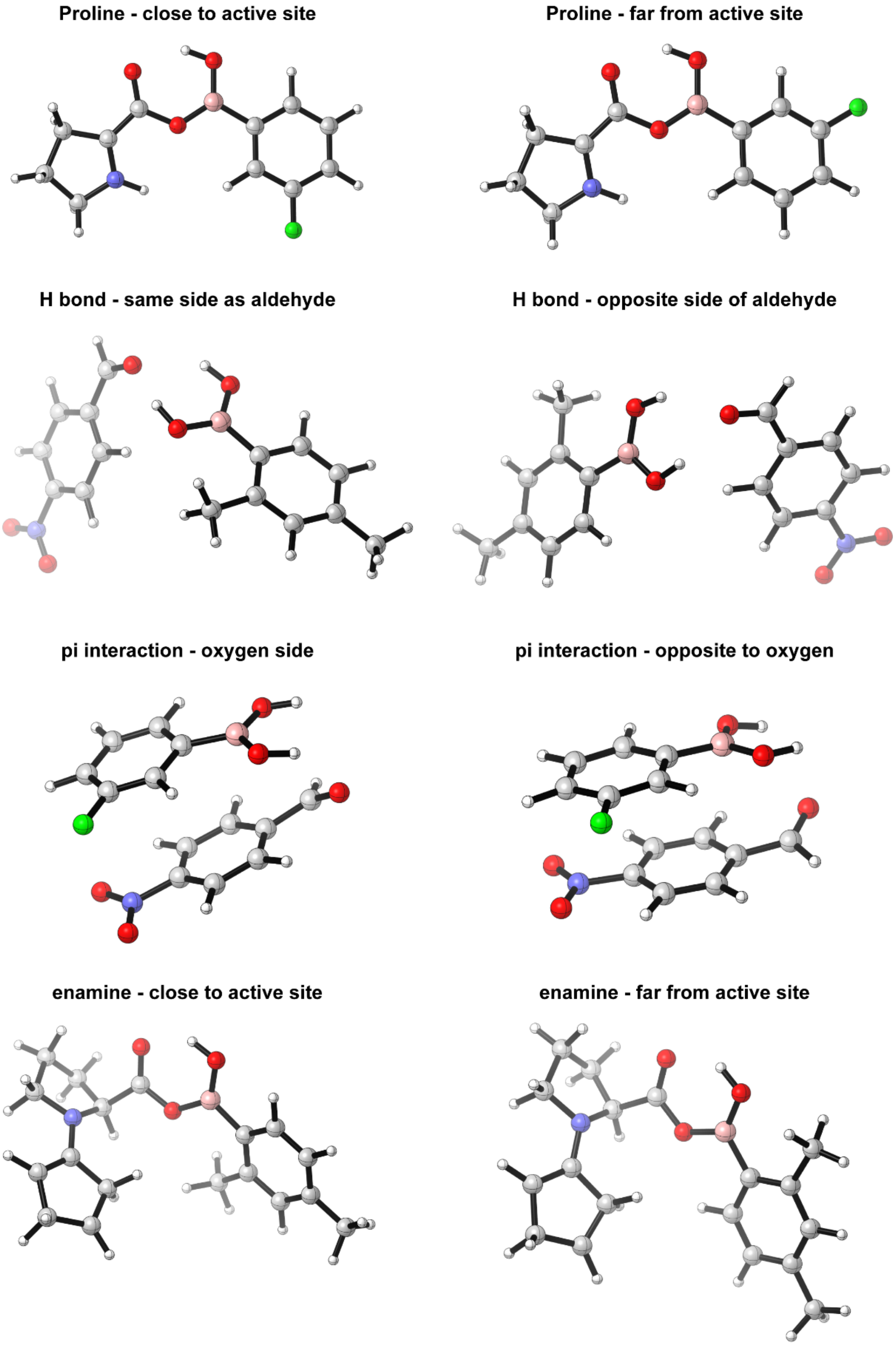

Figure S3. different rotamers used in model selection and respective notations with respect to substituent location.

All of the structures used in the process of model selection included the boronic acid moiety and included at least the same parameters as the simple boronic acid structure. The more complex structures were also parameterized for their unique features. The common parameters (i.e. parameters used for boronic acid s) are illustrated once for the boronic acid structure and are not illustrated on the rest of the structures.

Boronic Acid parameters

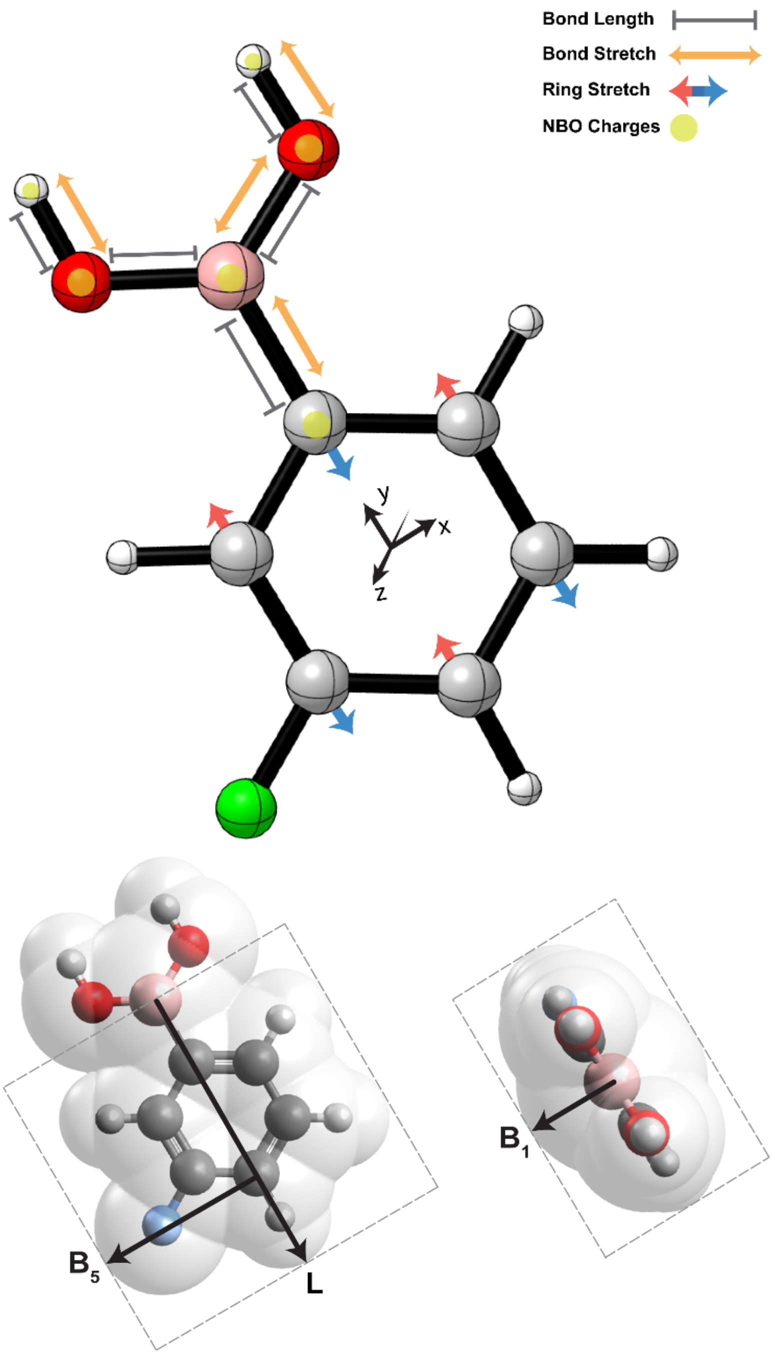

Figure S5. Physical organic parameters used for boronic acid throughout the library.

The parameters chosen for the description of the boronic acids: O–H, B–O and B–C bonds' length, stretching frequency, NBO charge differences (bond wise). The NBO charges were taken for all of the $–B(OH)_2$ moiety atoms, as well as the charge of the carbon on the B–C bond. Two characteristic ring stretching frequencies were considered: (1) the movement of atoms in parallel to the direction of the B–C bond (visualized above) and (2) a perpendicular movement. As mentioned above, Verloop's Sterimol parameters are also measured with the B–C bond as the defining primary axis. Finally, the components of the dipole moment were added to the list of common parameters with the origin at the center of the boronic acid ring.

boronic acid + Cyclopentanone parameters

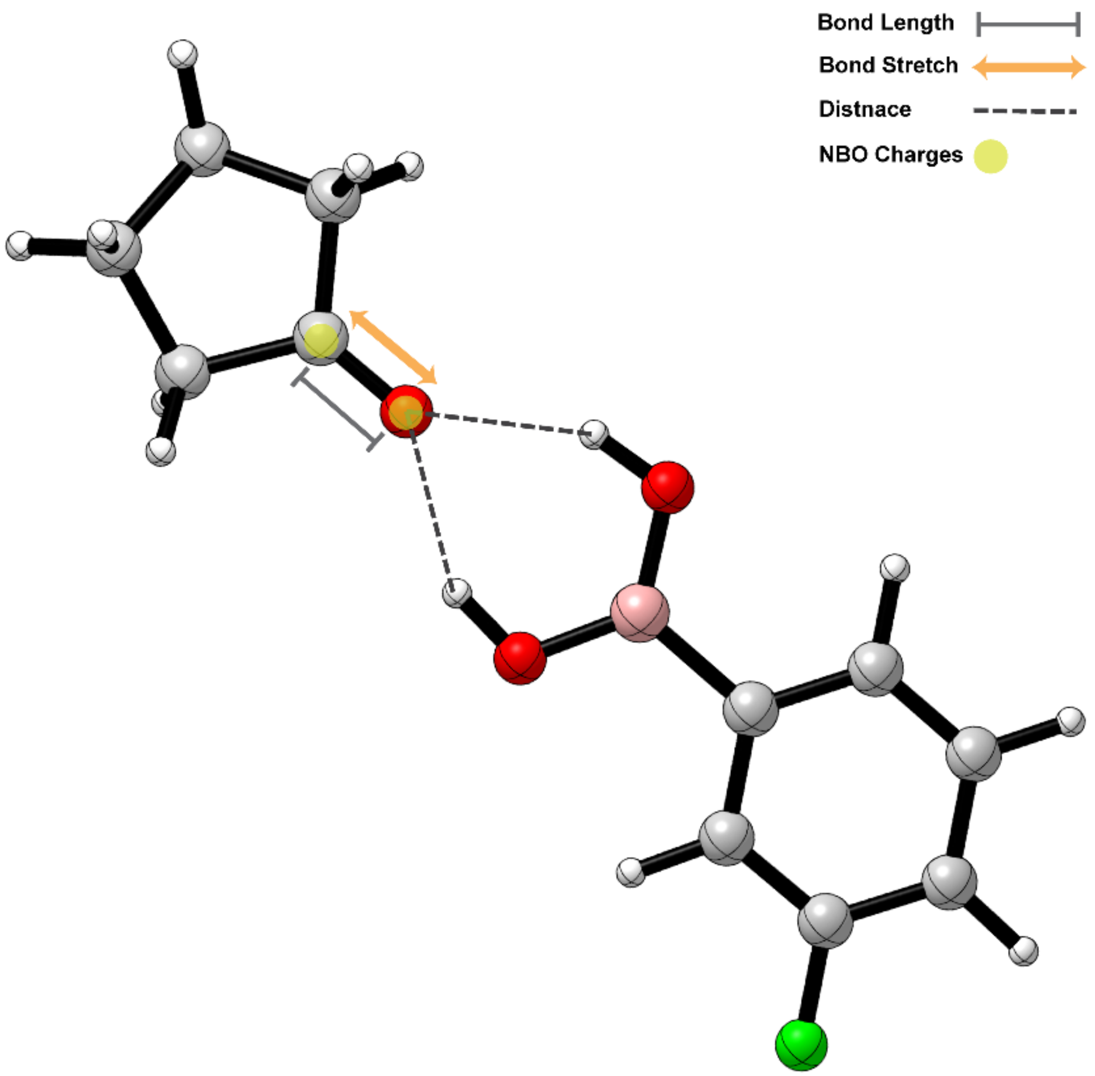

Figure S6. Additional physical organic parameters used in the depiction of the interaction between boronic acid and cyclopentanone.

boronic acid + Proline parameters

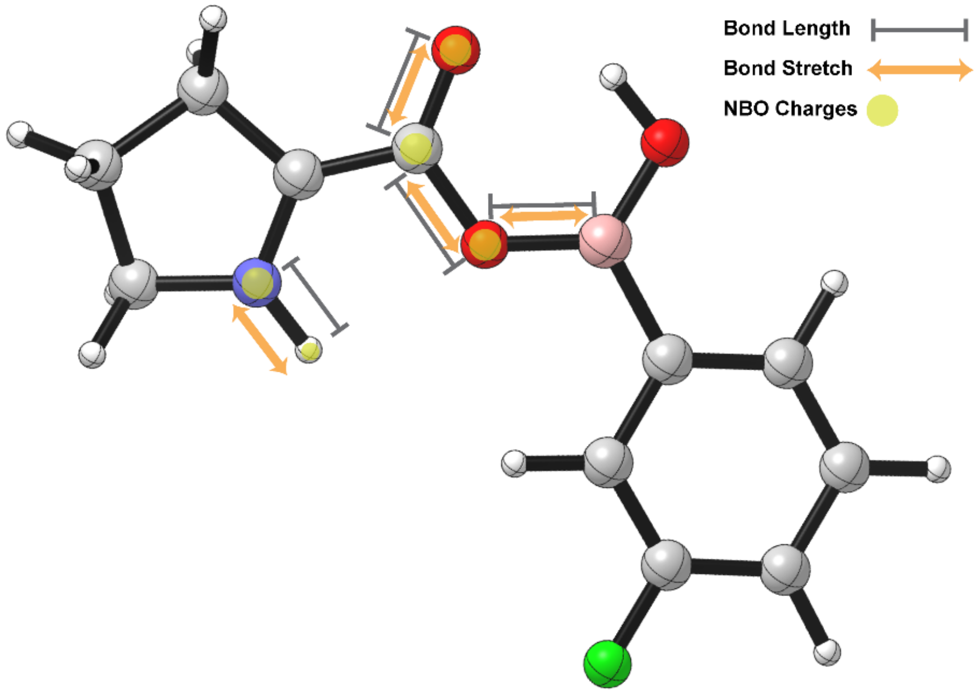

Figure S7. Additional physical organic parameters used in the depiction of the covalent interaction between boronic acid and Proline.

boronic acid + Aldehyde (π interaction) parameters

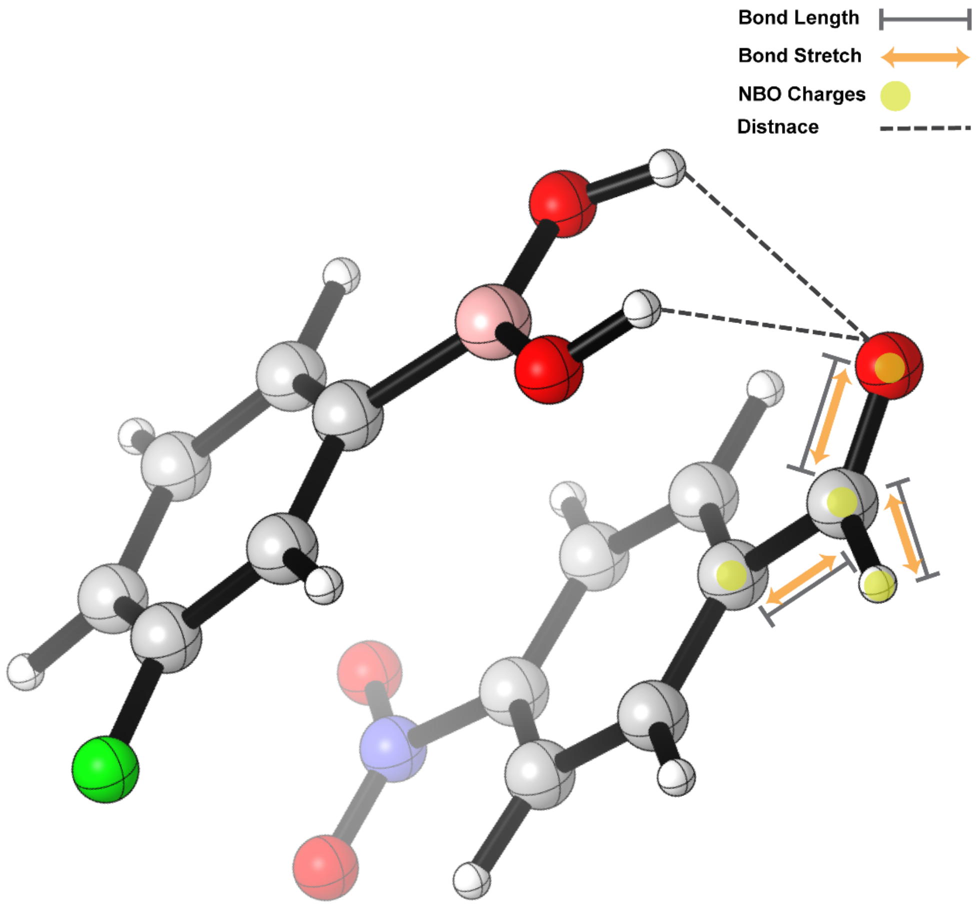

Figure S8. Additional physical organic parameters used in the depiction of the interaction between boronic acid and 4-nitrobenzaldehyde (π-interaction).

boronic acid + 4-nitrobenzaldehyde (H Bond interaction) parameters

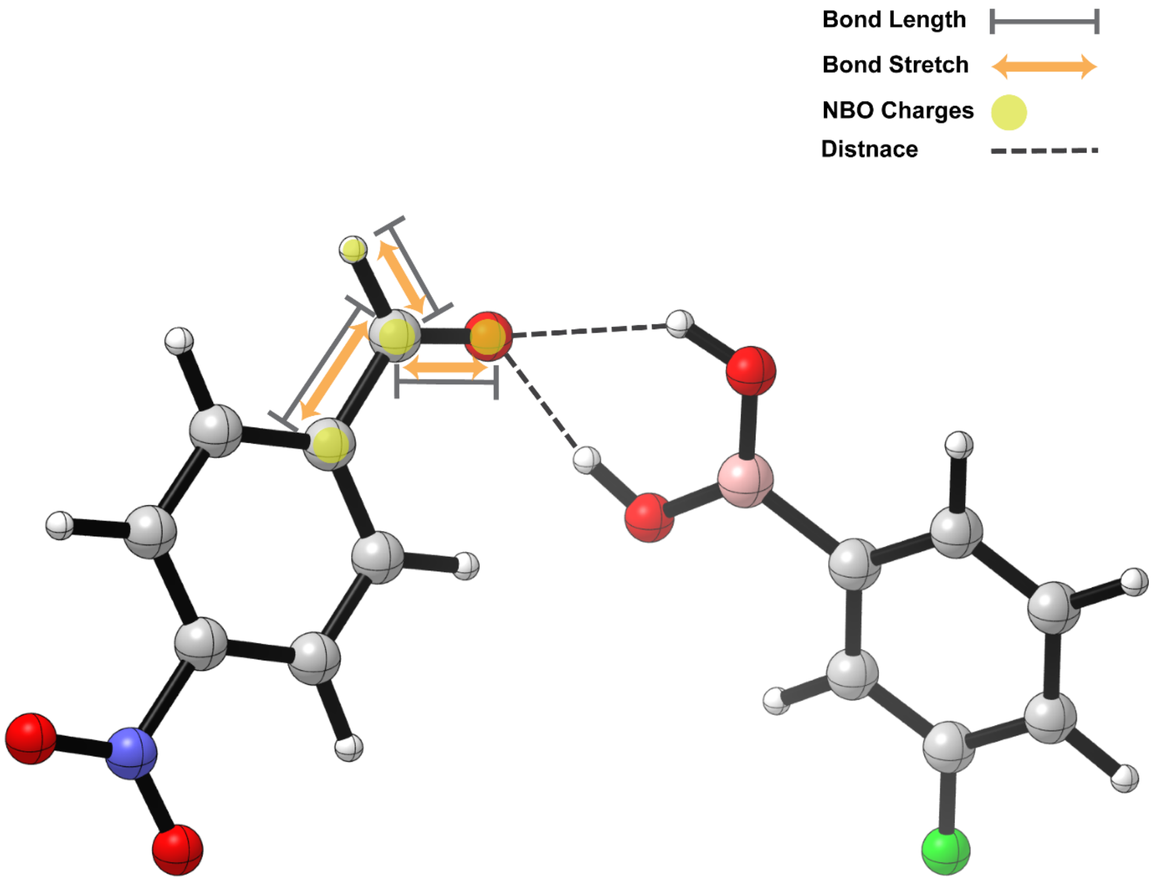

Figure S9. Additional physical organic parameters used in the depiction of the interaction between boronic acid and 4-nitrobenzaldehyde (H-bonding).

Enamine parameters

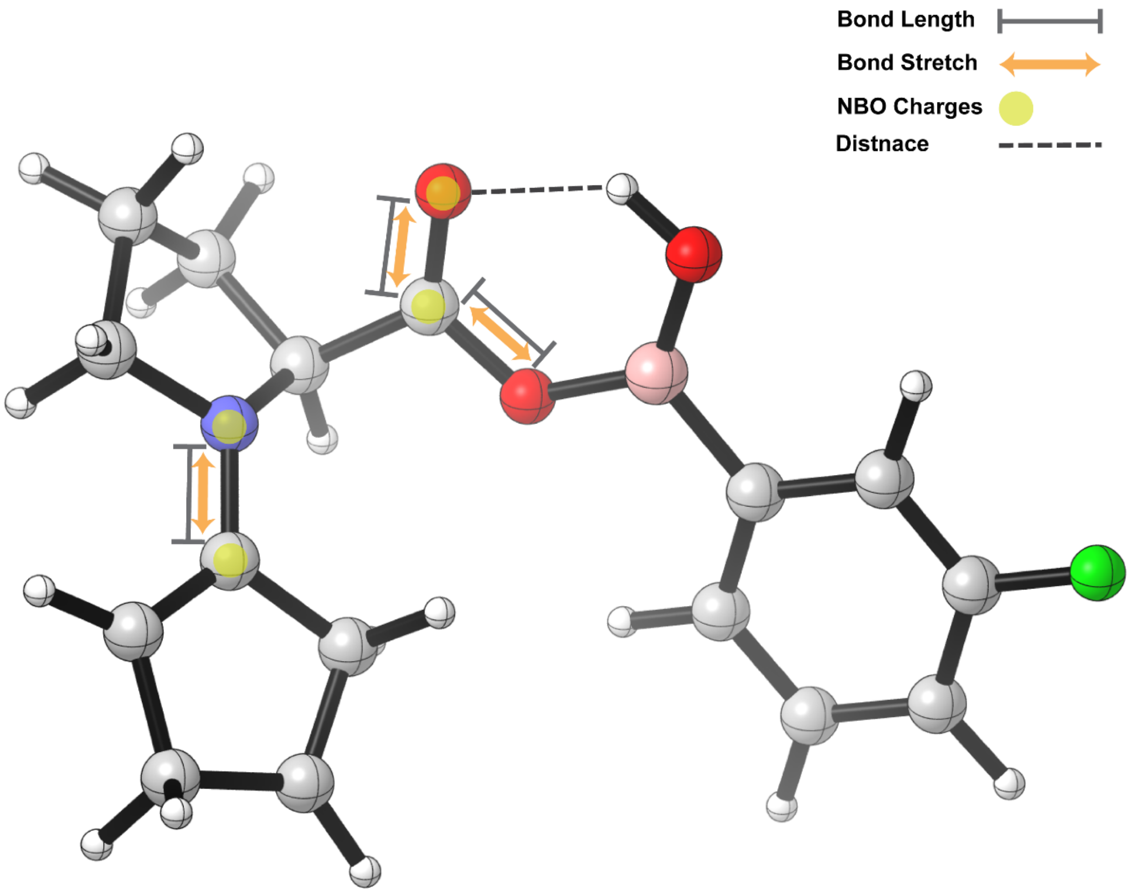

Figure S10. Additional physical organic parameters used in the depiction of the enamine.

Model for the reaction in acetonitrile with the bound boronic acid–proline structure

$$\mathbf{log(d.r.) = 0.12\ B.L_{N\text{-}H} - 0.19\ \Delta q_{C=O} - 0.033\ B_5}$$

$$R^2 = 0.92$$
$$Q^2_{LOO} = 0.87$$
$$Q^2_{5-fold} = 0.85$$
$$Q^2_{3-fold} = 0.83$$

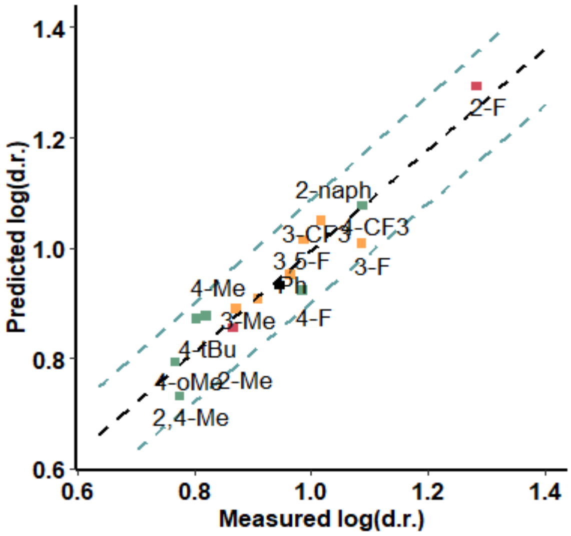

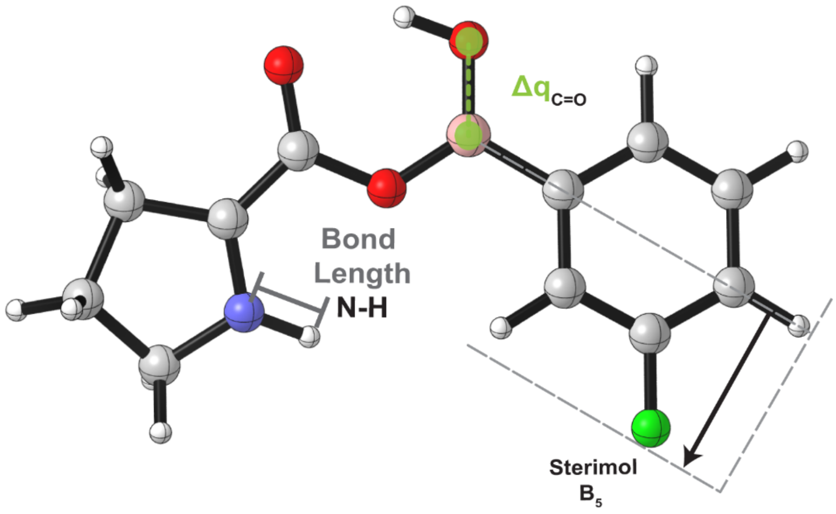

Figure S11. Model, Goodness-of-fit and Parameters for the fitted model of the reaction in acetonitrile using the bound boronic acid–proline structure.

Model for the reaction in $CHCl_3$ with the boronic acid–aldehyde π interaction structure

$$\mathbf{log(d.r.) = -0.32\ B.L_{C=O} - 0.28\ q_C + 0.17\ \Delta q_{O-H}}$$

$$R^2 = 0.91$$
$$Q^2_{LOO} = 0.75$$
$$Q^2_{5-fold} = 0.76$$
$$Q^2_{3-fold} = 0.75$$

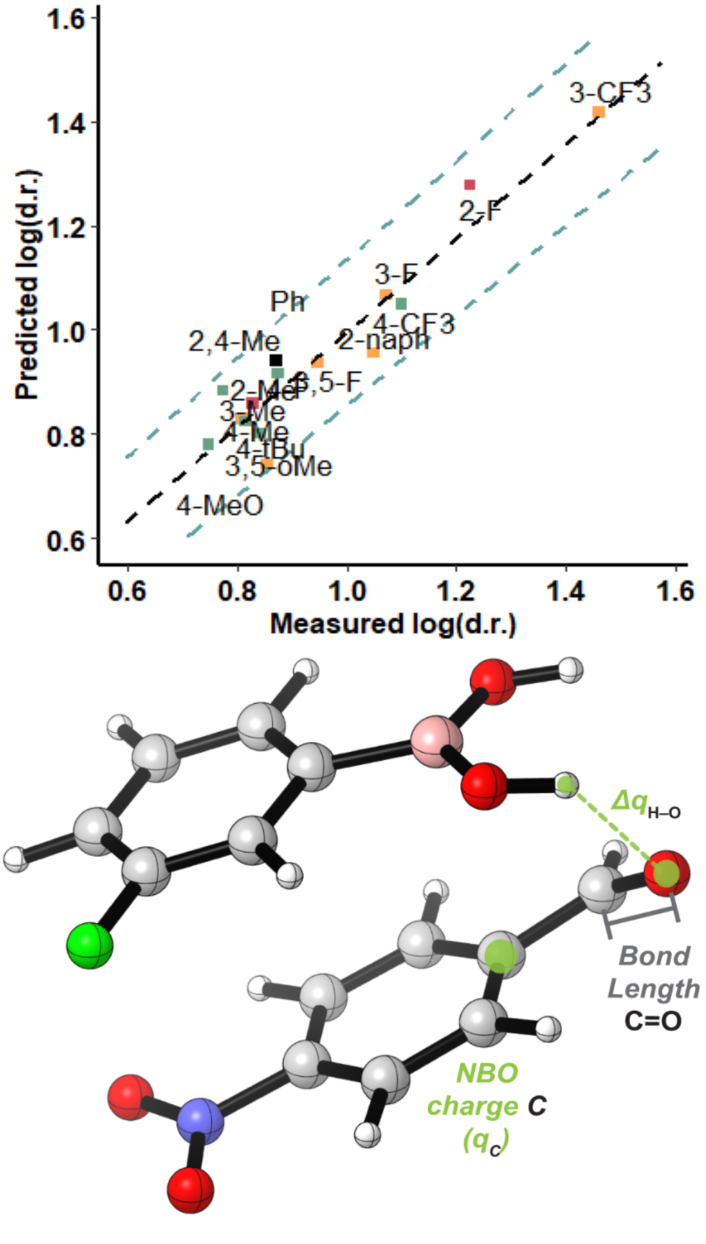

Figure S12. Model, goodness-of-fit and parameters for the fitted model of the reaction in $CHCl_3$ with the boronic acid–aldehyde π interaction structure.

22. Optimized structures – xyz coordinates

This section includes all of the structures used in the mathematical modelling process. The name of each file reflects the substitution of the boronic acid in each of the molecular families. The files are grouped in accordance with their respective family and the basic structure of each family is indicated in the beginning of the group.

Boronic acids - xyz structures

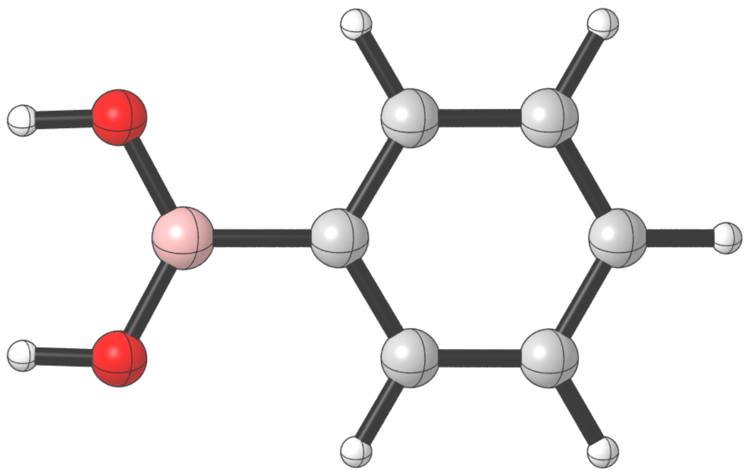

phenyl

16

O	2.372937	-1.206322	-0.000650
B	1.727075	-0.000006	0.000032
C	0.166	-0.000022	-0.000015
C	-0.548436	-1.199809	0.000210
C	-1.935597	-1.202992	0.000242
C	-2.629651	-0.000047	0.000021
C	-1.935545	1.203002	-0.000238
C	-0.548492	1.199852	-0.000253
O	2.372896	1.206377	0.000663
H	3.3323	-1.176434	0.000981
H	-0.004801	-2.136832	0.000450
H	-2.477045	-2.140409	0.000343

H	-3.712496	0.000006	0.000039
H	-2.47712	2.140346	-0.000337
H	-0.004811	2.136849	-0.000524
H	3.332257	1.176154	-0.001016

3-F

16

O	0.1341981278	1.1688432628	0.6971927982
B	-0.0057942905	0.0032534766	-0.0022993595
C	1.0885956318	-1.0984105619	0.179853624
C	2.1750254595	-0.894120475	1.032436924
C	3.1451810211	-1.872850858	1.1928114735
C	3.0474180749	-3.074050521	0.5039193667
C	1.9674086707	-3.2643713621	-0.3370204512
C	0.990832311	-2.306186299	-0.5124422168
O	-1.0431894257	-0.2603838829	-0.8518898434
H	-0.550140898	1.8296574877	0.5687601838
H	2.2542076988	0.04143856	1.5710724742
H	3.984063558	-1.7059699198	1.8558603364
H	3.7866101192	-3.8569962099	0.6068502915
F	1.8694055338	-4.4254516041	-1.0053136889
H	0.1631622435	-2.5011022847	-1.1822928773
H	-1.704245012	0.4286692044	-0.9497014109

3-Me

19

O	0.1082871021	1.1975559971	0.640667993
B	-0.0012112061	-0.0057541036	-0.003478398
C	1.0742032843	-1.0981431906	0.286133811
C	2.117455622	-0.8526209949	1.177846865
C	3.0698533746	-1.8300384079	1.429991979
C	2.9865120476	-3.0586384477	0.793168043
C	1.9555145948	-3.3345622799	-0.103920157
C	1.0118078444	-2.3439068693	-0.343133634
O	-1.0033778076	-0.3019644896	-0.888272284
H	-0.5680303011	1.848763289	0.441624160
H	2.1779957586	0.1088116361	1.672782777
H	3.8786608952	-1.6363098286	2.123170837
H	3.7326473187	-3.8202649818	0.992786156
C	1.8801059809	-4.6727799047	-0.787484512
H	0.2017268245	-2.5392445186	-1.037525165
H	-1.6500176093	0.3898829513	-1.046175456
H	1.0145118439	-4.7288726013	-1.445684454
H	1.8047620529	-5.4806695375	-0.057778587
H	2.7738480898	-4.8564255077	-1.386138873

2-naph

22

O	3.821102	0.772217	0.000583
B	2.910733	-0.249319	-0.000023
C	1.39487	0.121792	-0.000046
C	0.980277	1.481411	-0.000198

C	-0.342409	1.816005	-0.00018
C	-1.342803	0.812108	-0.000081
C	-0.946411	-0.549441	0.000036
C	0.434172	-0.859779	0.000089
O	3.254685	-1.573613	-0.000497
H	4.747023	0.519096	0.000723
C	-3.662971	0.130157	0.000029
C	-2.72411	1.122859	-0.000079
H	-1.632153	-2.594218	0.000249
H	-4.022955	-2.002675	0.00025
H	-4.716277	0.379497	0.000054
H	4.194202	-1.76971	-0.000478
H	0.732182	-1.902752	0.000227
C	-1.943079	-1.556188	0.000137
C	-3.268597	-1.226685	0.000154
H	-3.023602	2.16421	-0.000135
H	-0.649597	2.855328	-0.000286
H	1.737592	2.255549	-0.00035

3-CF3

19

O	0.1291265054	1.1565294904	0.7044487407
B	-0.0077836841	-0.0024330382	-0.0053849429
C	1.0807468744	-1.1093461703	0.1862785746
C	2.1532128956	-0.9154512568	1.0552578479
C	3.1221129696	-1.8958539861	1.228475687
C	3.0280810615	-3.0875603516	0.530323453
C	1.9620209375	-3.2917589015	-0.3394147894

C	0.9963376199	-2.3157131496	-0.512789339
O	-1.0302869543	-0.2583244277	-0.8730624429
H	-0.5485625276	1.8235616248	0.5721058883
H	2.2255976904	0.0173710147	1.6009820922
H	3.949252675	-1.7319743518	1.9063975861
H	3.7759050595	-3.8609328278	0.6554752524
C	1.8925749138	-4.5955966351	-1.0806484811
H	0.170782758	-2.4844346403	-1.1918602514
H	-1.6891120062	0.4315541771	-0.9798597931
F	0.8228961857	-4.6792453907	-1.8756253335
F	2.9743668837	-4.7838302047	-1.8516934385
F	1.8433684322	-5.6398107146	-0.2394749601

3,5-F

16

O	0.1051727197	1.2037084417	0.649915697
B	0.0025528728	0.0124356365	-0.008412531
C	1.0819659488	-1.0817556404	0.291963263
C	2.1051420775	-0.825983802	1.204908969
C	3.0441996048	-1.8067321515	1.452254519
C	3.0143126559	-3.0403993115	0.829847838
C	1.9892963771	-3.2653855321	-0.069560765
C	1.026109477	-2.3179958074	-0.351715954
O	-0.9758844248	-0.2909657853	-0.910430593
H	-0.5639547078	1.8637329819	0.454448251
H	2.1725434135	0.1236953102	1.718268827
F	4.0285407628	-1.5646540416	2.328598838
H	3.7590815617	-3.7953201916	1.037155195

F	1.93887852	-4.4540952699	-0.685950184
H	0.2439039225	-2.5431516387	-1.064064440
H	-1.6298730815	0.3902503614	-1.083096650

3,5-Me

22

O	-0.3947228017	1.244310779	0.40871484
B	0.0090781507	0.005478517	-0.01146318
C	1.3215501995	-0.587241156	0.59027850
C	2.061927598	0.132262822	1.53328304
C	3.2299770393	-0.380982714	2.07693441
C	3.6560724843	-1.642814370	1.66049402
C	2.9472864209	-2.386200015	0.72668265
C	1.7776627745	-1.842166244	0.19892821
O	-0.6775634936	-0.740621048	-0.93139213
H	-1.2050402476	1.581414411	0.02015276
H	1.7122461517	1.110783092	1.84298718
C	4.0308932131	0.390800600	3.08994264
H	4.5693281946	-2.054313646	2.08018921
C	3.4201494188	-3.745977084	0.28952706
H	1.2093476945	-2.407347935	-0.53223056
H	-1.4804144843	-0.348624714	-1.28204897
H	3.5555160216	1.342456039	3.32318240
H	5.0366148069	0.596153526	2.71855588
H	4.135349027	-0.173534526	4.01834020
H	4.3505966247	-4.020147683	0.78515324
H	3.5890819817	-3.771368135	-0.78831750
H	2.6751728755	-4.509549275	0.51989263

3,5-OMe

24

O	0.1431010787	1.180242178	0.68974631
B	0.0236749244	-0.007193577	0.01851671
C	1.0603715226	-1.134556371	0.32960774
C	2.0739220201	-0.909743955	1.27349443
C	2.9870778485	-1.915033375	1.54156602
C	2.9081834899	-3.146310807	0.88292413
C	1.9052647781	-3.359674616	-0.04667810
C	0.978447695	-2.351033660	-0.32440059
O	-0.95127885	-0.259151474	-0.90638453
H	-0.5073847602	1.853653239	0.47838958
H	2.121324127	0.046391230	1.77354701
O	4.0069151779	-1.809375796	2.43319867
H	3.6452703966	-3.896342254	1.12890012
O	1.748785421	-4.518837904	-0.73448538
H	0.2037154378	-2.541967039	-1.05509707
H	-1.5728886674	0.452234532	-1.07559982
C	4.13808231	-0.590931376	3.12860835
C	2.6637068045	-5.559388265	-0.48217329
H	4.9984504335	-0.704885678	3.78279954
H	3.2488919734	-0.380348137	3.72870934
H	4.3107764858	0.240707431	2.44036562
H	2.3698049065	-6.386180215	-1.12325326
H	2.6231296545	-5.877710649	0.56325035
H	3.6856845618	-5.256009920	-0.72572409

3,5-CF3

22

O	0.1335517694	1.159265285	0.7065061692
B	-0.0083149827	0.0037176189	-0.0023166723
C	1.066352643	-1.1159818164	0.2177769015
C	2.1242307124	-0.9240876709	1.1114143301
C	3.0693290828	-1.9149230761	1.3023404353
C	2.9864101656	-3.1190258442	0.6109370798
C	1.9425108872	-3.3130615246	-0.2732476263
C	0.9881607915	-2.3208416072	-0.4705780563
O	-1.010837151	-0.2521876571	-0.8895074522
H	-0.5280599981	1.8406563923	0.5654390293
H	2.200020529	0.0081700498	1.6557161028
C	4.2134973716	-1.7281912998	2.2594429147
H	3.7288834333	-3.8912957417	0.7644546948
C	1.8135268485	-4.5971625594	-1.0442578066
H	0.174907277	-2.4878116514	-1.1673979963
H	-1.6644401448	0.4390303585	-1.0194406857
F	4.1885433246	-0.5348230366	2.855754945
F	4.2011858357	-2.6572770179	3.2247815601
F	5.3952054609	-1.8393682467	1.6378175434
F	2.7800614709	-5.4686359065	-0.7454782753
F	0.643076015	-5.1999258755	-0.7990574557
F	1.8646371282	-4.3823548928	-2.3652370895

2-naph

22

O	0.1269126508	1.1440029884	0.752034431
B	0.0245467678	0.0002382183	0.004752966
C	1.2533470872	-0.949803689	-0.225935556
C	2.6095461002	-0.6758716582	0.154549000
C	3.6128848296	-1.6503688915	-0.107177773
C	3.2599610157	-2.8689376879	-0.734708282
C	1.9687581973	-3.1168124998	-1.095797853
C	0.9749051752	-2.1519345115	-0.839645995
O	-1.1619535047	-0.3829674365	-0.566304524
H	-0.6808698264	1.6560006251	0.839345067
C	3.0165661253	0.5333532482	0.777917727
C	4.323383945	0.7506632247	1.117462798
H	4.0388465495	-3.5989034385	-0.923096999
H	1.7016099477	-4.048615272	-1.577151317
H	-0.0450514962	-2.363407971	-1.133776229
H	-1.9151285286	0.1873874203	-0.398779573
C	5.3100019508	-0.2224720757	0.858295157
C	4.957197467	-1.395914713	0.258079897
H	2.2765059617	1.2898111797	0.986061270
H	4.6051357776	1.6823736205	1.591409314
H	6.3397574924	-0.0353664039	1.133860302
H	5.7013505451	-2.1552876168	0.048321871

2-F

16

O	0.1483617793	1.1675065783	0.6666626478
B	-0.0242810706	-0.0052496853	-0.0199177526
C	1.0582407707	-1.1108419045	0.2251008506
C	2.115802998	-0.8542627828	1.1060235488
C	3.1019627217	-1.7925042958	1.3623175465
C	3.0517516266	-3.029757114	0.7325073511
C	2.0212934768	-3.3223540796	-0.1462816089
C	1.0525792283	-2.3626375969	-0.379768267
O	-1.0620050187	-0.2322446899	-0.8724265254
H	-0.5138644572	1.8460441041	0.5178290011
H	2.150183054	0.1114505577	1.5941621162
H	3.9061052466	-1.5634154967	2.0484632053
H	3.8163775918	-3.7715279618	0.9243639607
H	1.9524355875	-4.275211234	-0.6529541105
F	0.0730721398	-2.6819793985	-1.2348857369
H	-1.687163795	0.4895202196	-0.9739527964

2-Me

19

O	0.083307245	1.05137491	0.7839439668
B	-0.0293415236	-0.09471564	0.0394865943
C	1.1223357506	-1.14774558	0.1734678649
C	2.1723977498	-0.82796524	1.0408452459

C	3.245582395	-1.68171349	1.2392090808
C	3.2814006019	-2.88896049	0.5594965584
C	2.2500513584	-3.22519228	-0.3047809915
C	1.1658707963	-2.37604812	-0.5137114034
O	-1.0952824114	-0.32427104	-0.7896276431
H	-0.6288368451	1.68782197	0.6911324576
H	2.1374021189	0.11662003	1.5689498863
H	4.0444298567	-1.40819131	1.9161455627
H	4.110611821	-3.57127311	0.6996982656
H	2.2847119887	-4.17101914	-0.8324422361
C	0.0852072899	-2.81173283	-1.4683405689
H	-1.7581028668	0.37026992	-0.8148534014
H	-0.0286127274	-2.10242082	-2.2878279193
H	-0.8828842186	-2.86902202	-0.9711290056
H	0.3213033207	-3.79007515	-1.8847522242

2-CF3

19

O	0.2704011508	1.3145407688	0.57674563
B	0.1094776035	0.0727773788	0.04578917
C	1.1284867903	-1.0669054153	0.42755070
C	1.649586117	-1.3012982675	1.70516537
C	2.5864296207	-2.2992722289	1.92928800
C	3.0291266248	-3.0857001392	0.87521477
C	2.5159610672	-2.8846502477	-0.39525704
C	1.5700052773	-1.8915971741	-0.60770196
O	-0.8394583492	-0.2519254263	-0.88162432
H	-0.3879253869	1.9738018546	0.34407765

F	1.7684777433	0.7124374622	2.94669585
H	2.9613967351	-2.4693636373	2.92877644
H	3.7630141374	-3.860457955	1.05380062
H	2.8455506419	-3.5035323615	-1.21973843
H	1.1596172007	-1.7475492913	-1.59939536
H	-1.4146193515	0.4630400099	-1.16559305
C	1.183100495	-0.4868397067	2.88663595
F	-0.1424567514	-0.2845637142	2.86003123
F	1.450673464	-1.0998740292	4.04892036

2,6-F

16

O	0.1083084409	1.2198268965	0.6315775737
B	-0.0239085859	0.0190423372	0.0000126016
C	1.2057871418	-0.9653682008	-0.0030544281
C	2.4228936381	-0.6629652271	0.6113481854
C	3.5177099031	-1.5067754013	0.6243952744
C	3.4154241759	-2.7342965544	-0.0085866679
C	2.2380977426	-3.1012185309	-0.6388897768
C	1.1752150618	-2.2176681544	-0.6204350131
O	-1.1685808422	-0.3712031052	-0.6290095135
H	-0.6658398591	1.7874886027	0.6083276505
F	2.5654947794	0.5098431316	1.2333779059
H	4.4209457674	-1.1893815965	1.1253176353
H	4.2600273049	-3.4104601057	-0.010708644
H	2.1245872771	-4.0508048442	-1.1417780544
F	0.0601114447	-2.6119070729	-1.2400148634
H	-1.8917935182	0.2598943284	-0.6019891519

2,4-Me

22

O	-0.2658187058	1.0592210769	0.8495310678
B	0.0849442868	0.0427387177	-0.0004001505
C	1.1581238156	-1.0149841765	0.4162953298
C	1.838200142	-1.0262856079	1.6468222231
C	2.7739103322	-2.0291505907	1.8923290838
C	3.0641989539	-3.0236613608	0.9663608379
C	2.3878647797	-3.0075837607	-0.2498289441
C	1.4559112261	-2.0203340577	-0.5115481930
O	-0.4805212731	-0.096131537	-1.2429452894
H	-0.932179298	1.6653947605	0.5166673999
C	1.5964392047	0.0059015221	2.7170041231
H	3.2956846235	-2.0333488399	2.8435490719
C	4.0809613546	-4.0905985335	1.2619144702
H	2.5947431681	-3.7719319769	-0.9902442341
H	0.9360274951	-2.018317515	-1.4615014291
H	-1.1373210776	0.5606146083	-1.4838235786
H	0.5546359954	0.0067867225	3.0370145372
H	1.8091187269	1.0106031482	2.3521444040
H	2.226961574	-0.1911685692	3.5830175245
H	4.5034922414	-3.970969915	2.2584883896
H	4.8983308787	-4.057620442	0.5392492378
H	3.6314109159	-5.0830936136	1.1987040382

4-F

16

O	0.1215149019	1.1911654662	0.6584097055
B	-0.0041162107	0.0001680465	-0.0029437489
C	1.0680592296	-1.100933255	0.2586434936
C	2.1250447776	-0.8747039934	1.1432674348
C	3.0843148304	-1.8458071887	1.3840197243
C	2.9708049309	-3.0543414926	0.7231922282
C	1.9433310988	-3.324546262	-0.1607618606
C	0.9958603518	-2.3381184404	-0.3855235146
O	-1.0153429578	-0.281273474	-0.8803305466
H	-0.5511015993	1.8523038829	0.4801515861
H	2.1940426319	0.0799084346	1.6500699921
H	3.9085830253	-1.6865132081	2.0656924352
F	3.8931374703	-4.0012426457	0.9483714527
H	1.9017320404	-4.2873786176	-0.6513629041
H	0.1818749897	-2.5279381907	-1.0742269305
H	-1.6624955509	0.4128074578	-1.024141307

4-Me

19

O	0.083005783	1.21439162	0.643081633
B	0.0003129711	0.01087293	-0.004548946
C	1.076188152	-1.07175853	0.310637217
C	2.0971242713	-0.82475244	1.227798063
C	3.0553181275	-1.78833497	1.508804758

C	3.0226331601	-3.02955611	0.881330613
C	2.0033107687	-3.28157479	-0.036683912
C	1.047680383	-2.32034268	-0.316929513
O	-0.9768435035	-0.29282373	-0.914350339
H	-0.5933334192	1.86041668	0.427732922
H	2.1383001668	0.13638070	1.726179124
H	3.8406344021	-1.57627592	2.225102396
C	4.0554036258	-4.08149711	1.176346933
H	1.9643990241	-4.24457607	-0.533770964
H	0.2627833798	-2.53302475	-1.032853098
H	-1.625017601	0.39358580	-1.087242585
H	4.7731251587	-3.73454903	1.918289309
H	4.604098699	-4.35122000	0.272188834
H	3.5863062606	-4.99112525	1.555414445

4-OMe

20

O	0.1292336244	1.19886215	0.65082492
B	0.0170650212	-0.01149941	0.01797801
C	1.0581976689	-1.11724704	0.34964361
C	2.081891739	-0.89101741	1.26555325
C	3.0208686936	-1.86766844	1.57390561
C	2.9383582884	-3.10964278	0.95056827
C	1.9205461171	-3.36062370	0.02825647
C	0.9997569836	-2.37635709	-0.26222903
O	-0.9656828847	-0.29831440	-0.89263191
H	-0.5294822623	1.85856786	0.42306797
H	2.1490815478	0.07453235	1.75256185

H	3.8005526814	-1.65352807	2.29024684
O	3.7980794779	-4.13238134	1.17508682
H	1.8810980631	-4.33494426	-0.44058414
H	0.2126338232	-2.57781607	-0.97880577
H	-1.5930838955	0.40437118	-1.07613723
C	4.8416843028	-3.92593323	2.09961863
H	5.4061246324	-4.85389148	2.13296594
H	4.4496947378	-3.70289165	3.09549853
H	5.4982916099	-3.11236000	1.78000332

4-tBu

28

O	0.0608347823	1.2228416309	0.5934261553
B	0.0105858159	-0.0123501333	0.0048579947
C	1.0776094622	-1.0704785839	0.4182659658
C	2.059483907	-0.7746328865	1.3601808941
C	3.0121231581	-1.715545905	1.7308957989
C	3.0180540694	-2.9910563777	1.1714898986
C	2.0316494088	-3.2872425426	0.2257215237
C	1.0835042939	-2.3502031403	-0.1429965895
O	-0.9266113067	-0.368560128	-0.9276343579
H	-0.6089320053	1.8527067548	0.3180058736
H	2.0787539484	0.2101739777	1.8117336356
H	3.7548689515	-1.4396223597	2.4664134671
C	4.0454370369	-4.0567358685	1.5484831491
H	2.0040883487	-4.2690520649	-0.2314446127
H	0.3310703317	-2.6081664008	-0.8787144246
H	-1.5707505026	0.3034055361	-1.1616300154

C	5.0428242753	-3.5569619633	2.5933688997
C	3.3192731652	-5.2814048228	2.122771297
C	4.8285841052	-4.472611266	0.2951887875
H	4.5431964448	-3.2715324442	3.5208323305
H	5.6106227022	-2.6993162893	2.2282291727
H	5.7522046986	-4.352603004	2.8269706794
H	2.7511666286	-5.0094756933	3.0140887514
H	4.043812199	-6.0512455974	2.3972999578
H	2.6266606509	-5.7126623193	1.3996388699
H	5.351235864	-3.6161400331	-0.1342562679
H	4.170841236	-4.8850990392	-0.4702224266
H	5.5679478401	-5.2345168964	0.5517459922

4-CF3

19

O	0.1240876792	1.1838124089	0.6713577364
B	-0.0033797059	0.0010123689	0.0006421507
C	1.065267581	-1.1109163087	0.2690437698
C	2.1098475246	-0.8926044443	1.1656043114
C	3.0611567829	-1.8737461406	1.4089940169
C	2.9662236863	-3.0876040701	0.747692134
C	1.9337141307	-3.3292508932	-0.151198668
C	0.9915789518	-2.3435322517	-0.3854385406
O	-1.0017832789	-0.2761478078	-0.8890775257
H	-0.5405961283	1.853343058	0.4936944228
H	2.1767994449	0.0586662512	1.6784706734
H	3.8692173661	-1.6990624857	2.1057257076
C	3.9756099521	-4.1761392281	0.9798352807

H	1.8746647504	-4.2837657356	-0.6590878264
H	0.1842791002	-2.5242111849	-1.0839649714
H	-1.6480085137	0.4173188273	-1.0411394663
F	4.9046869507	-3.8319817826	1.8760589885
F	3.3953626859	-5.3002973449	1.4235060174
F	4.6187046202	-4.5012979162	-0.1508775614

boronic acid + proline - xyz structures

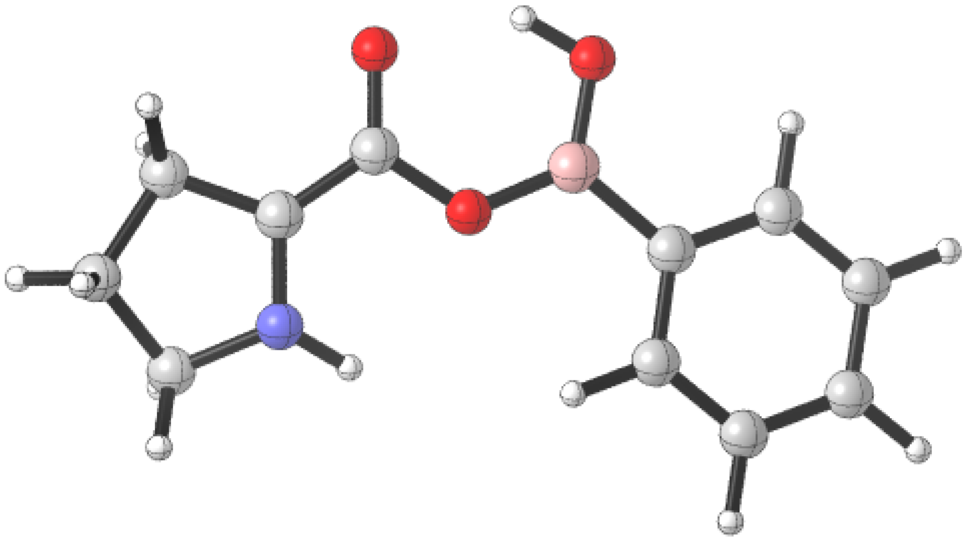

phenyl

29

B	2.4343	-0.2044	-0.0348
C	3.628	-1.2119	-0.0185
C	4.936	-0.7385	-0.1445
C	6.0145	-1.6105	-0.1348
C	5.7998	-2.9756	0.006
C	4.5068	-3.4651	0.1371
C	3.4331	-2.5868	0.123

O	2.6612	1.1218	-0.0868
H	5.0985	0.3272	-0.251
H	7.0226	-1.2291	-0.2353
H	6.6402	-3.6583	0.0153
H	4.3396	-4.5287	0.2505
H	2.4254	-2.9703	0.2323
H	1.8191	1.6096	-0.0784
C	-1.1683	-0.7935	-0.0061
O	1.1472	-0.7499	0.0
C	0.0	0.0	0.0
O	0.0	1.2286	0.0
N	-1.1627	-2.1468	-0.0114
H	-0.3126	-2.6558	-0.1947
C	-2.5757	-0.2897	-0.0335
H	-2.8143	0.0911	-1.0314
C	-3.3862	-1.5556	0.2956
C	-2.4961	-2.7001	-0.2027
H	-2.6221	-3.6214	0.3639
H	-2.6776	-2.9144	-1.2612
H	-3.5105	-1.6411	1.3753
H	-4.3709	-1.5671	-0.1654
H	-2.7336	0.5276	0.6678

3-F

29

B	2.4332	-0.1956	-0.0272
C	3.6309	-1.2026	-0.0109
C	4.9406	-0.7319	-0.122

C	6.015	-1.6098	-0.1124
C	5.8025	-2.9763	0.0116
C	4.5027	-3.4304	0.1241
C	3.4202	-2.5758	0.1148
O	2.6615	1.1294	-0.0716
H	5.1083	0.3334	-0.2165
H	7.0261	-1.2344	-0.2008
H	6.6185	-3.6862	0.0235
F	4.2878	-4.7519	0.2473
H	2.4228	-2.9846	0.2132
H	1.8191	1.6173	-0.066
C	-1.1657	-0.7962	-0.0053
O	1.1514	-0.7475	0.0
C	0.0	0.0	0.0
O	0.0	1.2285	0.0
N	-1.1589	-2.1488	-0.0092
H	-0.3097	-2.6605	-0.1882
C	-2.5739	-0.2942	-0.0291
H	-2.8172	0.0809	-1.0279
C	-3.3813	-1.5594	0.3102
C	-2.493	-2.7052	-0.1881
H	-2.6127	-3.6236	0.3844
H	-2.6806	-2.9259	-1.2441
H	-3.4995	-1.6402	1.3908
H	-4.3686	-1.5737	-0.1452
H	-2.7292	0.5265	0.6687

3-F_far

29

B	2.431118792	-0.2091918697	-0.025188085
C	3.6279451601	-1.2172979708	-0.008236059
C	3.4351023115	-2.5926722156	0.128851229
C	4.5126922972	-3.4665874466	0.143775772
C	5.8066077532	-2.9805082204	0.018512714
C	5.9865429296	-1.6166178207	-0.115774483
C	4.9300606125	-0.7299218573	-0.129710592
O	2.6641427339	1.114962963	-0.074097257
H	2.4291014814	-2.9789378488	0.234184141
H	4.3520168629	-4.5312146369	0.253504693
H	6.6686943485	-3.6336958146	0.025057744
F	7.2374004784	-1.1436337896	-0.235569848
H	5.1179733173	0.3311194305	-0.233860507
H	1.8244707891	1.6071840586	-0.066664732
C	-1.1694979361	-0.7948994708	-0.001600819
O	1.1473708502	-0.7561702243	0.005404538
C	-0.0004096067	-0.0035871342	0.005721009
O	0.0040629118	1.2247612582	0.007890280
N	-1.1673233466	-2.1477811539	-0.009470423
H	-0.3189250055	-2.6600116075	-0.191305492
C	-2.5757331125	-0.2876882607	-0.026812191
H	-2.8148184813	0.0927671079	-1.024681444
C	-3.3886457513	-1.5514613795	0.304523012
C	-2.5027406282	-2.6983332085	-0.195846381
H	-2.6285878446	-3.6188402371	0.372066446
H	-2.6876854719	-2.9132478392	-1.253546926
H	-3.5106450197	-1.6364839867	1.384438583
H	-4.3744198535	-1.5604913975	-0.154209751
H	-2.7302667916	0.5304685728	0.674140318

3-Me

32

B	2.435	-0.208	-0.0398
C	3.6262	-1.218	-0.0229
C	4.9332	-0.75	-0.1572
C	6.0018	-1.635	-0.1433
C	5.7747	-2.9946	0.0082
C	4.4808	-3.4942	0.1474
C	3.4247	-2.5915	0.1291
O	2.6629	1.118	-0.0977
H	5.1037	0.3134	-0.2706
H	7.0147	-1.2669	-0.2471
H	6.6129	-3.6827	0.0223
C	4.2473	-4.9726	0.2993
H	2.4127	-2.9649	0.2474
H	1.8218	1.6069	-0.0875
C	-1.1685	-0.7936	-0.006
O	1.1462	-0.7504	0.0
C	0.0	0.0	0.0
O	0.0	1.2286	0.0
N	-1.1617	-2.1472	-0.0121
H	-0.3112	-2.6545	-0.1984
C	-2.5763	-0.2911	-0.0322
H	-2.8163	0.0899	-1.0297
C	-3.3852	-1.558	0.297
C	-2.4947	-2.7011	-0.2038
H	-2.6198	-3.6235	0.3613
H	-2.6768	-2.9137	-1.2626
H	-3.5076	-1.6447	1.3768

H	-4.3708	-1.57	-0.1623
H	-2.7344	0.5257	0.6697
H	3.2157	-5.1811	0.5808
H	4.4532	-5.4978	-0.6357
H	4.9005	-5.3975	1.0626

3-Me_far

32

B	2.4358008537	-0.2094523179	-0.0445268608
C	3.6255617394	-1.2210700361	-0.023831992
C	3.424129947	-2.5929182659	0.1199422264
C	4.5018017655	-3.4672860792	0.1333207443
C	5.7916303665	-2.9762348365	0.0011504435
C	6.0278152503	-1.6094576495	-0.1411760747
C	4.9351244979	-0.7512146344	-0.150864689
O	2.6644375212	1.1165327531	-0.1042022873
H	2.4166674142	-2.9761957733	0.229184764
H	4.339535687	-4.5319733187	0.2451293325
H	6.6319856736	-3.6618726988	0.008404907
C	7.433406618	-1.0880330006	-0.2641362818
H	5.0957263624	0.3157837826	-0.2615001835
H	1.8229431855	1.6050475516	-0.0976849012
C	-1.1678972488	-0.7930501884	-0.0079258234
O	1.1467891445	-0.7510751554	-0.0050218541
C	0.0010282007	-0.0001134754	-0.0084484061
O	0.0017847615	1.2285635209	-0.0164229706
N	-1.1620146921	-2.1465443701	-0.004214864
H	-0.3117123422	-2.6561868359	-0.185161024

C	-2.5754082777	-0.2898237947	-0.0377241737
H	-2.8148911465	0.0844267812	-1.0378873771
C	-3.3853327884	-1.5538891947	0.3000862651
C	-2.4951949565	-2.7012229065	-0.1917667008
H	-2.620862495	-3.6191685335	0.380405664
H	-2.6771891591	-2.9218974331	-1.2489098806
H	-3.5085791	-1.6327008674	1.3803618534
H	-4.3704896807	-1.5686885974	-0.1599488389
H	-2.7332949502	0.5318230197	0.6584704735
H	7.4384393287	-0.0367457289	-0.5481861761
H	7.9667030414	-1.1824878731	0.6840511255
H	7.996042458	-1.6478449336	-1.0125010894

2-naph_close

35

B	2.4849926113	-0.2358274956	-0.2355473128
C	3.6969930522	-1.2148857681	-0.1254425112
C	5.0125920741	-0.734001914	-0.3690310769
C	6.0944004589	-1.5603081449	-0.2813259987
C	5.936335231	-2.9264429421	0.0616083168
C	4.6318697868	-3.4225070331	0.3135160235
C	3.5334699614	-2.5363624165	0.208968694
O	2.6910116807	1.0729310712	-0.4757578016
H	-4.3008286739	-1.6670284195	0.0474443109
H	-2.5083458162	-3.6203757324	0.7756557494
H	-2.795327864	-0.1210481013	-1.0782272068
C	-3.3016146289	-1.5874138459	0.4688227784
C	-2.4133216832	-2.7786905243	0.0913576947

H	1.8429618515	1.549844241	-0.5004383818
H	-2.6264806399	-3.1291283178	-0.924014268
O	1.2087252878	-0.7848736306	-0.0810062937
C	0.0517094312	-0.0524648125	-0.1400648658
O	0.0349253162	1.1656458891	-0.299705698
N	-1.0817273851	-2.1934539126	0.1642943638
H	-0.2315063163	-2.7139940698	0.0175320898
H	-2.6649894597	0.5322320794	0.5467434497
H	-3.3888837357	-1.5340039272	1.5541201184
H	2.5397870181	-2.9234643499	0.4096620372
C	-1.1052471507	-0.8508229301	-0.0041429863
H	5.1401284835	0.3097714186	-0.6280902012
C	-2.5192697786	-0.3674530247	-0.0482603886
C	4.4705209316	-4.7862096333	0.6609523722
H	7.0932611903	-1.184909352	-0.4708147249
C	7.0365294448	-3.8115803483	0.1631617406
C	5.5506722056	-5.6172562063	0.7519294751
H	3.4713612757	-5.1587266386	0.8547432135
C	6.8498321223	-5.1228027163	0.4991563765
H	8.0316494713	-3.429028329	-0.0303644916
H	5.4171895288	-6.6577001658	1.0182823427
H	7.6986210469	-5.7902152569	0.5741345509

2-naph_far

35

B	2.4644156069	-0.1830828755	-0.327295302
C	3.6795696708	-1.163421995	-0.3026124517
C	3.5241500715	-2.545122999	-0.0104389714

C	4.5934932892	-3.3925705877	0.0056539391
C	5.8977084293	-2.914213764	-0.2740522961
C	6.0727047114	-1.537551517	-0.5665111608
C	4.9407006805	-0.688849424	-0.5700281463
O	2.6518171044	1.1364220718	-0.5206047368
H	-4.2926822939	-1.6987088519	0.1523259188
H	-2.4528396228	-3.655217313	0.7410579649
H	-2.8475962946	-0.0957206705	-0.9725870916
C	-3.2790049441	-1.6223078568	0.5382831901
C	-2.3931563015	-2.7884888123	0.0846000811
H	1.7998691085	1.6057983953	-0.4977744183
H	-2.6412213958	-3.1050122152	-0.9339941987
O	1.1983219913	-0.7488630359	-0.1486899221
C	0.0330316399	-0.0268263615	-0.1380208136
O	-0.000619951	1.1960177554	-0.2512652569
N	-1.0661646783	-2.1902418336	0.128245165
H	-0.216461339	-2.6954119182	-0.0671060647
H	-2.6625371956	0.50071979	0.6689655178
H	-3.3253500327	-1.6089674104	1.6273442099
H	5.0778011136	0.3642229271	-0.7913145003
C	-1.1100759555	-0.8428187718	0.010324955
H	2.5338361107	-2.9233114644	0.2131742705
C	-2.5299394601	-0.3752734043	0.0366894365
C	7.3748551752	-1.0540917638	-0.8453343184
H	4.4637619268	-4.444219809	0.2334960406
C	7.0305703433	-3.7633350477	-0.2693954972
C	8.4496773204	-1.8967069234	-0.8340363526
H	7.5004402514	-0.0011161601	-1.0676728948
C	8.2744100316	-3.2681929463	-0.5422764015
H	6.89323736	-4.8145249928	-0.0450209677
H	9.4407650596	-1.5184500088	-1.0482091547
H	9.1338240688	-3.926191486	-0.5360337911

3-CF3-cat

32

B	2.4321	-0.1893	-0.0273
C	3.6323	-1.1946	-0.0147
C	4.9435	-0.7264	-0.1313
C	6.0188	-1.6013	-0.1313
C	5.803	-2.9675	-0.0106
C	4.5057	-3.4434	0.1085
C	3.4305	-2.5663	0.1077
O	2.6615	1.1353	-0.0694
H	5.1102	0.3398	-0.2253
H	7.0285	-1.2244	-0.2267
H	6.6345	-3.6594	-0.0135
C	4.2438	-4.9112	0.2805
H	2.4242	-2.9553	0.2052
H	1.8188	1.6233	-0.0631
C	-1.1631	-0.7988	-0.0050
O	1.1545	-0.7455	0.0000
C	0.0	0.0	0.0000
O	0.0	1.2285	0.0000
N	-1.1543	-2.1507	-0.0090
H	-0.305	-2.6643	-0.1828
C	-2.5724	-0.2994	-0.0267
H	-2.8189	0.0709	-1.0265
C	-3.3765	-1.5646	0.3201
C	-2.4883	-2.711	-0.1772
H	-2.6022	-3.6266	0.4008

H	-2.6805	-2.938	-1.2309
H	-3.4908	-1.6415	1.4014
H	-4.3652	-1.5822	-0.1319
H	-2.7269	0.5241	0.6681
F	5.2863	-5.6629	-0.0865
F	3.9694	-5.2219	1.5570
F	3.1886	-5.3173	-0.4417

3-CF3-far

32

B	2.4314660788	-0.201120408	-0.0316309321
C	3.6337519007	-1.203945962	-0.0136165646
C	3.4504078325	-2.580275052	0.1334512761
C	4.5281065554	-3.451788885	0.1509384140
C	5.8187529601	-2.958741517	0.0169282522
C	6.0141595889	-1.593240764	-0.1274933595
C	4.9344326182	-0.721365308	-0.1416768232
O	2.662102675	1.122450240	-0.0844765910
H	2.4454302032	-2.969164004	0.2456363004
H	4.3697848798	-4.515464425	0.2704498386
H	6.6680479834	-3.628155491	0.0303676685
C	7.3975075887	-1.033882748	-0.3025005699
H	5.0987007733	0.344536743	-0.2490245553
H	1.8222243829	1.614331116	-0.0762942551
C	-1.1664654901	-0.800022559	-0.0001591109
O	1.1519954441	-0.755238509	0.0026890317
C	0.0002858434	-0.005818513	0.0049297816
O	0.0025531361	1.222207563	0.0066193995

N	-1.1624371612	-2.152770976	-0.0091806063
H	-0.3148881547	-2.664099475	-0.1968325184
C	-2.5734806811	-0.294727649	-0.0205323852
H	-2.8158390083	0.085939461	-1.0175195799
C	-3.3836019068	-1.559667198	0.3128027860
C	-2.4981668128	-2.704963884	-0.1916174331
H	-2.6205300782	-3.626136163	0.3759235072
H	-2.6859830829	-2.919075346	-1.2489112734
H	-3.5016744286	-1.645584942	1.3930704402
H	-4.3709750723	-1.569511002	-0.1423807992
H	-2.7265819396	0.522983162	0.6812257156
F	8.3485777532	-1.927109694	-0.0062802936
F	7.6144499393	-0.638107121	-1.5656076141
F	7.6035148696	0.036573478	0.4739035030

3,5-F

29

B	2.4307	-0.1875	-0.0297
C	3.6368	-1.1885	-0.013
C	4.9359	-0.6942	-0.1315
C	5.9954	-1.5785	-0.1186
C	5.8257	-2.9446	0.0107
C	4.5289	-3.4058	0.1295
C	3.4368	-2.5623	0.1197
O	2.659	1.1364	-0.0769
H	5.1191	0.3672	-0.2322
F	7.2438	-1.1072	-0.2348
H	6.669	-3.6201	0.0196

F	4.3336	-4.7265	0.2592
H	2.4441	-2.9787	0.2237
H	1.8168	1.625	-0.0706
C	-1.1639	-0.7977	-0.0053
O	1.1546	-0.7462	0.0
C	0.0	0.0	0.0
O	0.0	1.2282	0.0
N	-1.1565	-2.1499	-0.0103
H	-0.3076	-2.6619	-0.1895
C	-2.5723	-0.296	-0.0283
H	-2.8155	0.0793	-1.027
C	-3.3793	-1.5615	0.3108
C	-2.4908	-2.7072	-0.1874
H	-2.6093	-3.6251	0.386
H	-2.6788	-2.9288	-1.2431
H	-3.4979	-1.6423	1.3914
H	-4.3663	-1.576	-0.145
H	-2.7275	0.5245	0.6698

3,5-OMe

37

B	2.4343	-0.2084	-0.028
C	3.623	-1.2263	-0.019
C	4.9268	-0.7539	-0.1336
C	5.9925	-1.6447	-0.1302
C	5.7719	-3.0171	-0.0097
C	4.4628	-3.4822	0.1078
C	3.3958	-2.5944	0.1024

O	2.6663	1.1166	-0.0676
H	5.1271	0.3052	-0.226
O	7.2308	-1.0998	-0.2492
H	6.6005	-3.7065	-0.0066
O	4.1481	-4.7997	0.2321
H	2.3941	-2.9902	0.2036
H	1.8259	1.607	-0.0602
C	-1.1669	-0.7956	-0.0058
O	1.1466	-0.7499	0.0
C	0.0	0.0	0.0
O	0.0	1.2287	0.0
N	-1.1574	-2.1487	-0.0073
H	-0.3051	-2.6576	-0.1812
C	-2.5759	-0.2963	-0.0344
H	-2.8174	0.0781	-1.034
C	-3.3818	-1.5631	0.3027
C	-2.4891	-2.7074	-0.1918
H	-2.61	-3.626	0.3803
H	-2.6727	-2.9285	-1.2485
H	-3.5032	-1.6436	1.3831
H	-4.3677	-1.5799	-0.1559
H	-2.7353	0.5242	0.6628
C	5.2001	-5.7344	0.251
H	4.7378	-6.712	0.3606
H	5.7727	-5.7087	-0.6806
H	5.8744	-5.5578	1.0938
C	8.3422	-1.9625	-0.248
H	8.4103	-2.5234	0.6887
H	8.3032	-2.6638	-1.0867
H	9.2193	-1.3291	-0.3511

3,5-OMe

37

B	-0.3861177063	-1.4513084	-0.086532125
C	0.9946281683	-0.7190973	-0.052959072
C	2.1557174828	-1.4842463	0.036815374
C	3.3963728599	-0.8510175	0.069902614
C	3.4756820943	0.5337809	0.009087374
C	2.3183326056	1.2931923	-0.085629683
C	1.0719763356	0.6718304	-0.114630299
O	-0.4455936501	-2.7961464	-0.129731391
H	2.0670840754	-2.5601510	0.078399751
O	4.5827010002	-1.5032786	0.159143763
H	4.4406462091	1.0211871	0.032553822
O	2.5032660416	2.6389560	-0.145634268
H	0.1615340192	1.2473229	-0.196847745
H	-1.3718017058	-3.0926763	-0.163638777
C	-3.7836197915	-0.1197847	0.011572544
O	-1.5298594993	-0.6468981	-0.060188066
C	-2.8090536637	-1.1367543	-0.094007222
O	-3.06862091	-2.3329026	-0.199329726
N	-3.4914205498	1.1960261	0.138781213
H	-2.5529189902	1.4920276	0.356080397
C	-5.2653717619	-0.3171113	0.029579053
H	-5.5726892922	-0.7327269	0.994227502
C	-5.7918088473	1.1162356	-0.160398449
C	-4.676747795	1.9950690	0.418071713
H	-4.6097461593	2.9729725	-0.056275645
H	-4.8012742122	2.1384529	1.496606964
H	-5.9015491749	1.3287877	-1.223936847

H	-6.7490328048	1.2903100	0.325136492
H	-5.5972112272	-1.0123502	-0.739358967
C	1.360547139	3.4527501	-0.251364050
H	1.7191371167	4.4780267	-0.292057602
H	0.7960436647	3.2294665	-1.161099350
H	0.7063620592	3.3327986	0.617343498
C	4.5518295715	-2.9119318	0.216518043
H	4.0060883169	-3.2605581	1.097060653
H	4.0928675339	-3.3348013	-0.680792536
H	5.5874439672	-3.2350579	0.280703611

3,5-CF3-cat

35

B	2.4289	-0.175	-0.0279
C	3.6421	-1.1698	-0.0103
C	4.9432	-0.6833	-0.1284
C	6.0263	-1.5495	-0.1212
C	5.8405	-2.9175	0.008
C	4.5496	-3.4062	0.1297
C	3.4631	-2.544	0.1206
O	2.6573	1.1478	-0.0744
H	5.1033	0.3842	-0.2289
C	7.4097	-0.9728	-0.2473
H	6.6864	-3.59	0.0117
C	4.2937	-4.8788	0.2828
H	2.4624	-2.947	0.2223
H	1.8149	1.6368	-0.0694
C	-1.161	-0.8004	-0.0046

O	1.16	-0.7432	0.0
C	0.0	0.0	0.0
O	0.0	1.228	0.0
N	-1.1532	-2.1517	-0.0109
H	-0.3055	-2.6666	-0.1873
C	-2.5699	-0.2995	-0.0232
H	-2.8168	0.0721	-1.0224
C	-3.3747	-1.5641	0.3235
C	-2.4886	-2.711	-0.1761
H	-2.6014	-3.6266	0.402
H	-2.6826	-2.9375	-1.2295
H	-3.4881	-1.6418	1.4048
H	-4.3638	-1.58	-0.1276
H	-2.7221	0.5235	0.6725
F	7.5301	-0.2242	-1.3503
F	8.3503	-1.9198	-0.3043
F	5.4107	-5.6033	0.1978
F	3.452	-5.3311	-0.6583
F	3.7261	-5.1588	1.4643
F	7.7064	-0.1803	0.7901

2-naph_close

35

B	2.441146397	-0.2290310766	-0.0389229724
C	3.6446797034	-1.2316021601	-0.1275207891
C	4.775607622	-0.7538696609	-0.7500054797
C	5.8962666111	-1.5721778664	-0.9999600016
C	5.8786145914	-2.8813258359	-0.6178457025

C	4.7543231808	-3.4229186743	0.0513219569
C	3.6287564298	-2.5946511551	0.3120321551
O	2.6735123631	1.0979567655	-0.0760040834
H	4.7963215726	0.2815025444	-1.067674657
H	6.7620455053	-1.1590130319	-1.5010843042
H	6.7274124947	-3.5270318494	-0.8111592643
C	4.7465412052	-4.7743688156	0.4754668268
C	2.5483018826	-3.1628617542	1.0385364343
H	1.8333254605	1.5887544329	-0.0662000501
C	-1.1588512099	-0.8069759492	-0.0057924857
O	1.1473908323	-0.7516411265	-0.0000932099
C	-0.0000098625	0.0000307825	-0.00013529
O	0.0000274973	1.2279527093	-0.0003199513
N	-1.1285094667	-2.1601982131	-0.0085087306
H	-0.2699360423	-2.6573592081	-0.1940526087
C	-2.5745277356	-0.3275515753	-0.0319116279
H	-2.8228025159	0.0421314842	-1.0316351112
C	-3.3611590125	-1.6059267217	0.3072852541
C	-2.4534330717	-2.7367641064	-0.1904477445
H	-2.5601884989	-3.6579617473	0.3801979162
H	-2.6355560562	-2.9588484116	-1.2471406305
H	-3.4783462273	-1.6882305430	1.3879949771
H	-4.3479821252	-1.6366608211	-0.1483172308
H	-2.7448401309	0.4911661360	0.6648115189
H	1.7048884651	-2.5384703337	1.2953209865
C	3.6793218841	-5.2928455110	1.1500195109
C	2.5725397514	-4.4690004044	1.4433438149
H	5.6132134152	-5.3880758823	0.2593764034
H	3.6845902983	-6.3256056287	1.4733777614
H	1.7407147925	-4.8733267908	2.006546409

2-naph_far

35

B	2.4397	-0.2184	-0.0528
C	3.597	-1.2781	0.0084
C	4.9537	-1.0224	-0.3781
C	5.9215	-2.0562	-0.2461
C	5.5336	-3.3148	0.2724
C	4.2404	-3.5479	0.6382
C	3.28	-2.5263	0.4968
O	2.6442	1.1111	-0.1275
H	2.2591	-2.7277	0.7978
H	3.9481	-4.5107	1.037
H	6.2859	-4.0887	0.3724
C	7.2622	-1.8186	-0.6353
C	5.3826	0.2227	-0.9082
H	1.789	1.5796	-0.1092
C	-1.1679	-0.7941	-0.008
O	1.1444	-0.7508	0.0
C	0.0	0.0	0.0
O	0.0	1.2295	0.0
N	-1.1582	-2.1475	-0.0203
H	-0.3073	-2.6522	-0.2141
C	-2.5764	-0.2936	-0.031
H	-2.8173	0.0909	-1.0269
C	-3.3828	-1.5634	0.2934
C	-2.4908	-2.7028	-0.213
H	-2.6136	-3.6278	0.3481
H	-2.6729	-2.9111	-1.2726
H	-3.5044	-1.6549	1.3729

H	-4.3686	-1.575	-0.1653
H	-2.7354	0.52	0.6743
H	4.664	1.0215	-1.0117
C	7.6394	-0.6078	-1.1395
C	6.6844	0.4209	-1.2766
H	7.9825	-2.6207	-0.5252
H	8.666	-0.434	-1.4351
H	6.9874	1.3796	-1.6778

2-F_close

29

B	2.4315	-0.225	0.0069
C	3.6378	-1.2258	-0.0036
C	4.9394	-0.712	0.0339
C	6.0538	-1.5345	0.0289
C	5.8895	-2.9132	-0.0152
C	4.6171	-3.4615	-0.0545
C	3.5289	-2.6095	-0.0476
O	2.68	1.0995	0.0239
H	5.0575	0.364	0.0675
H	7.0466	-1.1062	0.0593
H	6.7525	-3.5664	-0.0195
H	4.4536	-4.5298	-0.0901
F	2.31	-3.1867	-0.0887
H	1.8416	1.5946	0.025
C	-1.1496	-0.8179	-0.0042
O	1.149	-0.7502	0.0
C	0.0	0.0	0.0

O	0.0	1.2288	0.0
N	-1.0937	-2.1676	0.0106
H	-0.2149	-2.6517	-0.1018
C	-2.5737	-0.3656	-0.0484
H	-2.8245	-0.0223	-1.0568
C	-3.3376	-1.6517	0.3151
C	-2.4044	-2.778	-0.1475
H	-2.4901	-3.6814	0.4545
H	-2.5837	-3.0412	-1.1953
H	-3.4616	-1.7099	1.3966
H	-4.3202	-1.7123	-0.1466
H	-2.7633	0.4637	0.6306

2-F_far

29

B	1.3533479815	-0.7927666855	0.0000214631
C	0.0000296063	0.0000001333	0.0001229873
C	-1.2483584577	-0.6112568195	0.0179385013
C	-2.4390620093	0.0936829988	0.0330005992
C	-2.3952867192	1.4783908295	0.0365752767
C	-1.1722744297	2.1371969774	0.0215800824
C	-0.0000409928	1.3994260954	0.0000074652
O	1.427310213	-2.1004544484	0.2879275509
F	-1.329731396	-1.9459418323	0.0084711105
H	-3.3733031825	-0.4509161596	0.0418152648
H	-3.3189646428	2.0424107084	0.0514142569
H	-1.1365950675	3.2183110244	0.0280105898
H	0.954205999	1.9117566023	-0.0046025548

H	2.354443715	-2.3961504669	0.2684869674
C	4.7028538671	0.454222234	-0.7471757676
O	2.4794847027	-0.0223762177	-0.3107305294
C	3.7599510912	-0.5124983958	-0.3334640924
O	4.0422303363	-1.6686233951	-0.0306309422
N	4.382386717	1.723578017	-1.0916656146
H	3.4221067961	1.9839850443	-1.2520760043
C	6.1747570853	0.2408718586	-0.8984992682
H	6.372937787	-0.355464932	-1.794481756
C	6.7028758773	1.6782015603	-1.0494009495
H	5.4947614884	3.492827635	-1.3712664923
H	5.516219266	2.3736748375	-2.7465137043
C	5.5185354808	2.4414182648	-1.6535319859
H	6.9318937191	2.0899363072	-0.0663799591
H	7.5957779824	1.7452837456	-1.6662615398
H	6.6013071859	-0.2975255211	-0.054190955

2-Me_close

32

B	2.4379	-0.2212	0.0263
C	3.6693	-1.1933	0.0368
C	4.9259	-0.5798	0.0782
C	6.1015	-1.3135	0.0947
C	6.0347	-2.6974	0.0691
C	4.7991	-3.3266	0.0273
C	3.6121	-2.5992	0.0107
O	2.6528	1.1103	0.0451
H	4.9678	0.5021	0.0979

H	7.0592	-0.8106	0.1272
H	6.9409	-3.2900	0.0816
H	4.7536	-4.4092	0.0071
C	2.3116	-3.3616	-0.0368
H	1.8058	1.5896	0.0349
C	-1.1669	-0.7951	-0.0037
O	1.1459	-0.7542	0.0
C	0.0	0.0000	0.0
O	0.0	1.2287	0.0
N	-1.1576	-2.1473	0.007
H	-0.3029	-2.6616	-0.141
C	-2.5757	-0.2957	-0.039
H	-2.8167	0.0662	-1.0433
C	-3.3823	-1.5578	0.3141
C	-2.4896	-2.7092	-0.1644
H	-2.6086	-3.6186	0.4227
H	-2.6757	-2.9477	-1.2168
H	-3.5049	-1.6237	1.3953
H	-4.3676	-1.5803	-0.1451
H	-2.7346	0.5337	0.6477
H	1.6902	-3.1285	0.8295
H	1.7454	-3.1048	-0.9345
H	2.5008	-4.4342	-0.0467

2-Me_far

32

B	2.4394	-0.2177	-0.0559
C	3.6043	-1.2649	-0.0178

C	4.9485	-0.9406	-0.2841
C	5.9095	-1.9465	-0.2186
C	5.5752	-3.2519	0.1106
C	4.2555	-3.5783	0.3808
C	3.2895	-2.5872	0.31
O	2.6486	1.1108	-0.1338
C	5.3834	0.4563	-0.6394
H	6.9432	-1.6986	-0.4292
H	6.3464	-4.0107	0.1563
H	3.9832	-4.5924	0.643
H	2.2579	-2.8371	0.5266
H	1.7956	1.5822	-0.1144
C	-1.1694	-0.7924	-0.0071
O	1.1437	-0.751	0.0
C	0.0	0.0	0.0
O	0.0	1.2295	0.0
N	-1.1626	-2.146	-0.0187
H	-0.3129	-2.6527	-0.2115
C	-2.577	-0.2894	-0.0304
H	-2.8175	0.0952	-1.0264
C	-3.3859	-1.5574	0.2947
C	-2.4961	-2.6989	-0.2109
H	-2.6207	-3.6231	0.3512
H	-2.6789	-2.9079	-1.2702
H	-3.5076	-1.648	1.3743
H	-4.3718	-1.5675	-0.1639
H	-2.7347	0.5248	0.6745
H	4.8297	0.8391	-1.4963
H	5.1919	1.1469	0.1817
H	6.4473	0.4742	-0.8724

2-CF3-close

32

B	2.4222	-0.1997	0.146
C	3.6833	-1.1515	0.1243
C	4.8673	-0.5557	-0.3159
C	6.0503	-1.2707	-0.4355
C	6.0787	-2.6133	-0.1
C	4.9248	-3.2302	0.361
C	3.7438	-2.5092	0.4704
O	2.6384	1.1258	0.2415
H	4.8479	0.4969	-0.5675
H	6.9476	-0.7772	-0.7858
H	6.995	-3.1824	-0.1848
H	4.947	-4.2726	0.645
C	2.5338	-3.2420	0.9906
H	1.7897	1.6025	0.1806
C	-1.1565	-0.8051	-0.0034
O	1.1575	-0.7449	0.0
C	0.0	0.0000	0.0
O	0.0	1.2301	0.0
N	-1.1342	-2.1528	0.0208
H	-0.2709	-2.6697	-0.0558
C	-2.5703	-0.3187	-0.0451
H	-2.8208	0.0109	-1.0581
C	-3.3619	-1.5778	0.3502
C	-2.4615	-2.7351	-0.1005
H	-2.5564	-3.6223	0.5238
H	-2.6631	-3.0192	-1.1386
H	-3.4761	-1.6125	1.4338

H	-4.3505	-1.6225	-0.1006
H	-2.7316	0.5285	0.6187
F	1.6525	-3.5318	0.0069
F	1.8663	-2.5513	1.9186
F	2.8531	-4.4171	1.5456

2-CF3-far

32

B	2.4332	-0.1989	-0.0433
C	3.6054	-1.2512	0.034
C	4.7864	-1.198	-0.714
C	5.7942	-2.1353	-0.5409
C	5.6458	-3.1493	0.3945
C	4.4806	-3.2322	1.1389
C	3.473	-2.2966	0.9489
O	2.6689	1.1198	-0.0687
H	6.6899	-2.078	-1.1434
H	6.4359	-3.8758	0.5305
H	4.354	-4.0252	1.8648
H	2.5612	-2.3711	1.5296
C	4.9825	-0.1211	-1.753
H	1.8313	1.6156	-0.0769
C	-1.1617	-0.8016	-0.0083
O	1.1528	-0.7493	0.0
C	0.0	0.0	0.0
O	0.0	1.2274	0.0
N	-1.1445	-2.1544	-0.0131
H	-0.289	-2.6567	-0.1917

C	-2.5728	-0.3088	-0.0428
H	-2.808	0.0706	-1.0419
C	-3.375	-1.5813	0.2811
C	-2.4727	-2.7185	-0.2119
H	-2.5941	-3.6409	0.3539
H	-2.6454	-2.9343	-1.2715
H	-3.5057	-1.668	1.3599
H	-4.3566	-1.6005	-0.1861
H	-2.741	0.5069	0.6579
F	3.8641	0.0957	-2.4587
F	5.9338	-0.4558	-2.6385
F	5.352	1.044	-1.2184

2,6-F

29

B	2.4251	-0.2267	-0.1601
C	3.5969	-1.2808	-0.201
C	4.9111	-0.9489	0.1235
C	5.9651	-1.8438	0.1009
C	5.7126	-3.1538	-0.2713
C	4.4284	-3.553	-0.6081
C	3.417	-2.6143	-0.56
O	2.6676	1.0867	-0.2835
H	6.9535	-1.5025	0.3733
H	4.1996	-4.5659	-0.9071
H	6.5223	-3.8705	-0.2996
F	2.1862	-3.0384	-0.9058
F	5.1833	0.3021	0.4999

H	1.8332	1.5845	-0.2151
C	-1.1504	-0.8167	-0.0074
O	1.151	-0.7492	0.0
C	0.0	0.0	0.0
O	0.0	1.2282	0.0
N	-1.0987	-2.167	-0.0266
H	-0.2253	-2.6469	-0.1888
C	-2.5729	-0.3581	-0.0312
H	-2.8265	0.0149	-1.0282
C	-3.3395	-1.6514	0.2986
C	-2.413	-2.7659	-0.2035
H	-2.5023	-3.689	0.3673
H	-2.5951	-2.9917	-1.2595
H	-3.4574	-1.7418	1.3785
H	-4.325	-1.6949	-0.159
H	-2.7565	0.4523	0.6718

2,4-Me_close

35

B	2.4388	-0.2245	0.0264
C	3.6659	-1.1978	0.032
C	4.9256	-0.5929	0.064
C	6.0975	-1.3316	0.0758
C	6.0472	-2.7194	0.0542
C	4.7966	-3.3306	0.0254
C	3.6113	-2.6041	0.013
O	2.655	1.1073	0.0476
H	4.9755	0.4888	0.0828

H	7.057	-0.8285	0.1043
C	7.302	-3.5468	0.0366
H	4.7469	-4.4149	0.0145
C	2.3105	-3.3670	-0.0198
H	1.8089	1.5876	0.0375
C	-1.1676	-0.7948	-0.0039
O	1.1446	-0.7549	0.0
C	0.0	0.0000	0.0
O	0.0	1.2288	0.0
N	-1.1577	-2.1472	0.0075
H	-0.3023	-2.6606	-0.1401
C	-2.5765	-0.2960	-0.0413
H	-2.8163	0.0664	-1.0457
C	-3.3831	-1.5588	0.3099
C	-2.4889	-2.7095	-0.1674
H	-2.6092	-3.6193	0.4186
H	-2.6727	-2.9472	-1.2205
H	-3.5074	-1.6253	1.3908
H	-4.3678	-1.5816	-0.1509
H	-2.7371	0.5327	0.6458
H	7.1909	-4.4444	0.6453
H	7.5362	-3.8672	-0.9811
H	8.1534	-2.9792	0.4104
H	1.6906	-3.1191	0.8434
H	1.7436	-3.1227	-0.9207
H	2.4984	-4.4399	-0.0139

2,4-Me_far

35

B	2.4402	-0.2205	-0.0544
C	3.6016	-1.2676	-0.0151
C	4.9531	-0.9452	-0.2533
C	5.9084	-1.9523	-0.1875
C	5.5859	-3.2736	0.1139
C	4.2547	-3.5853	0.3547
C	3.2877	-2.5942	0.2853
O	2.6496	1.1087	-0.133
C	5.3973	0.4563	-0.5778
H	6.9472	-1.6999	-0.3761
H	3.9757	-4.6039	0.5974
C	6.6608	-4.323	0.1677
H	2.2544	-2.8528	0.4835
H	1.7968	1.58	-0.1149
C	-1.1703	-0.7916	-0.007
O	1.1423	-0.7516	0.0
C	0.0	0.0	0.0
O	0.0	1.2295	0.0
N	-1.1641	-2.1454	-0.0187
H	-0.3148	-2.6517	-0.2139
C	-2.5777	-0.288	-0.0293
H	-2.8189	0.0972	-1.0249
C	-3.3869	-1.5559	0.2955
C	-2.4978	-2.6971	-0.2121
H	-2.6232	-3.6224	0.3481
H	-2.681	-2.9038	-1.2719
H	-3.5076	-1.6475	1.375
H	-4.3732	-1.5653	-0.1624
H	-2.7347	0.526	0.6761
H	6.2609	-5.2822	0.4936
H	7.1163	-4.4614	-0.8147

H	7.4555	-4.0318	0.8563
H	4.8649	0.8502	-1.4432
H	5.1828	1.1353	0.2473
H	6.4668	0.4782	-0.7828

4-F

29

B	2.4340310228	-0.2008722781	-0.0354262195
C	3.62896623	-1.2039958703	-0.0151235949
C	4.937180696	-0.7281330052	-0.1355732935
C	6.0229532651	-1.5892951534	-0.1228968043
C	5.7824815322	-2.9431546694	0.0169169724
C	4.5078839596	-3.4616410474	0.144019812
C	3.4384683227	-2.5796346661	0.1250729571
O	2.6609388077	1.1252877368	-0.0912110524
H	5.0993488894	0.3375008957	-0.2409258218
H	7.0407298042	-1.2359168152	-0.2169482853
F	6.8251142422	-3.7863080770	0.0316449481
H	4.3757635785	-4.5292312868	0.2548030523
H	2.4325500608	-2.9675763558	0.2305011057
H	1.8189916675	1.6132225399	-0.0833862557
C	-1.167771001	-0.7940810535	-0.005075584
O	1.1488987121	-0.7495412947	0.0000118015
C	0.0000870363	-0.0002871386	0.0002085646
O	0.000454304	1.2281778779	0.0001830922
N	-1.1628171077	-2.1473514713	-0.0112884003
H	-0.3141493781	-2.6570730532	-0.1985763847
C	-2.5751395051	-0.2898929406	-0.0286789265

H	-2.8163454895	0.0906204433	-1.02602744
C	-3.3850272829	-1.5554687594	0.3030731911
C	-2.4972279105	-2.7001044776	-0.1987996674
H	-2.6214049258	-3.6216564097	0.3677717526
H	-2.6818278004	-2.9137397231	-1.2568350044
H	-3.5055057607	-1.6412650872	1.3830957001
H	-4.3713865667	-1.5664094001	-0.1544003182
H	-2.7306394027	0.5276205399	0.6728701032

4-Me

32

B	2.4354	-0.2085	-0.0358
C	3.6236	-1.2185	-0.0198
C	4.9368	-0.7564	-0.1397
C	6.0077	-1.6344	-0.1268
C	5.8055	-3.007	0.0086
C	4.4999	-3.473	0.136
C	3.4287	-2.5924	0.1197
O	2.6637	1.1181	-0.089
H	5.1103	0.3082	-0.2412
H	7.0191	-1.2548	-0.2194
C	6.9701	-3.9574	-0.0063
H	4.3253	-4.5366	0.252
H	2.4212	-2.9767	0.2295
H	1.8226	1.6068	-0.08
C	-1.1689	-0.7933	-0.0062
O	1.1457	-0.7505	0.0
C	0.0	0.0	0.0

O	0.0	1.2287	0.0
N	-1.1626	-2.1469	-0.0111
H	-0.3121	-2.6549	-0.1953
C	-2.5765	-0.2904	-0.0333
H	-2.8161	0.09	-1.0311
C	-3.3861	-1.5568	0.2963
C	-2.4956	-2.7006	-0.2031
H	-2.6212	-3.6225	0.3627
H	-2.6772	-2.914	-1.2618
H	-3.509	-1.6427	1.3761
H	-4.3714	-1.5689	-0.1635
H	-2.7347	0.5268	0.668
H	6.7083	-4.9113	0.4502
H	7.291	-4.1556	-1.0315
H	7.8239	-3.5414	0.5288

4-OMe

33

B	2.4361	-0.2094	-0.0324
C	3.6208	-1.2174	-0.0168
C	4.931	-0.757	-0.122
C	6.0177	-1.6222	-0.1146
C	5.7958	-2.9914	0.0047
C	4.4922	-3.4783	0.116
C	3.4296	-2.5992	0.1035
O	2.6633	1.1186	-0.0816
H	5.1047	0.3086	-0.2127
H	7.0185	-1.2248	-0.1999

O	6.7784	-3.9239	0.0237
H	4.3495	-4.5466	0.2115
H	2.4225	-2.9882	0.1976
H	1.8214	1.6058	-0.0736
C	-1.17	-0.7919	-0.0061
O	1.1447	-0.7508	0.0
C	0.0	0.0	0.0
O	0.0	1.2288	0.0
N	-1.166	-2.1457	-0.0102
H	-0.3164	-2.6551	-0.1946
C	-2.5768	-0.2867	-0.0341
H	-2.8152	0.0945	-1.0319
C	-3.3887	-1.5517	0.2949
C	-2.4997	-2.697	-0.2037
H	-2.6276	-3.6188	0.3619
H	-2.6807	-2.91	-1.2626
H	-3.5128	-1.6374	1.3746
H	-4.3737	-1.5622	-0.1657
H	-2.734	0.5307	0.6674
C	8.112	-3.4789	-0.0817
H	8.7332	-4.3697	-0.0459
H	8.2809	-2.9556	-1.0265
H	8.3753	-2.8184	0.7486

4-tBu

41

B	2.4355	-0.208	-0.0268
C	3.6237	-1.2181	-0.0122

C	4.935	-0.7595	-0.1113
C	6.0116	-1.6376	-0.1018
C	5.8179	-3.0123	0.0108
C	4.5019	-3.473	0.1139
C	3.4315	-2.597	0.1018
O	2.6639	1.1189	-0.069
H	5.113	0.3058	-0.1977
H	7.0109	-1.2331	-0.1825
C	6.9711	-4.014	0.0264
H	4.3112	-4.5355	0.2064
H	2.4242	-2.9872	0.1908
H	1.8227	1.6076	-0.0627
C	-1.169	-0.7931	-0.0057
O	1.1458	-0.7505	0.0
C	0.0	0.0	0.0
O	0.0	1.2287	0.0
N	-1.1632	-2.1467	-0.0088
H	-0.3126	-2.6554	-0.1905
C	-2.5765	-0.2897	-0.0344
H	-2.815	0.0902	-1.0326
C	-3.3868	-1.5555	0.2958
C	-2.4961	-2.7003	-0.2008
H	-2.6226	-3.6211	0.3666
H	-2.6769	-2.9157	-1.2593
H	-3.5114	-1.6399	1.3755
H	-4.3714	-1.5678	-0.1655
H	-2.735	0.5282	0.6662
C	6.8152	-4.9858	-1.1519
H	7.6352	-5.7073	-1.153
H	5.8783	-5.5404	-1.0929
H	6.8297	-4.4464	-2.1005
C	6.9408	-4.8033	1.3428

H	7.7621	-5.5229	1.3666
H	7.0459	-4.1321	2.1969
H	6.0069	-5.3538	1.4591
C	8.3334	-3.331	-0.0921
H	8.4236	-2.7749	-1.0269
H	8.5116	-2.6443	0.7372
H	9.1204	-4.0867	-0.0754

4-CF3

32

B	2.4318	-0.1903	-0.0288
C	3.6365	-1.1918	-0.01
C	4.9388	-0.7085	-0.1281
C	6.0272	-1.5698	-0.1161
C	5.8123	-2.9321	0.0199
C	4.524	-3.439	0.1438
C	3.4483	-2.5691	0.127
O	2.6589	1.1343	-0.0764
H	5.0959	0.3581	-0.2303
H	7.0346	-1.1882	-0.209
C	6.9609	-3.9004	0.0298
H	4.374	-4.5058	0.2539
H	2.4438	-2.9603	0.2305
H	1.8165	1.6224	-0.0706
C	-1.1649	-0.7966	-0.0053
O	1.1534	-0.7468	0.0
C	0.0	0.0	0.0
O	0.0	1.2283	0.0

N -1.1582 -2.149 -0.0112

H -0.309 -2.6602 -0.1919

C -2.5731 -0.2944 -0.0279

H -2.8158 0.0829 -1.0261

C -3.3807 -1.56 0.3089

C -2.4925 -2.7053 -0.1907

H -2.6124 -3.6241 0.3811

H -2.6798 -2.9249 -1.2469

H -3.4998 -1.6424 1.3893

H -4.3676 -1.5734 -0.1474

H -2.7282 0.525 0.6714

F 6.9029 -4.7441 -1.0116

F 6.9558 -4.661 1.134

F 8.1475 -3.2904 -0.0242

boronic acid + Cyclopentanone - xyz structures

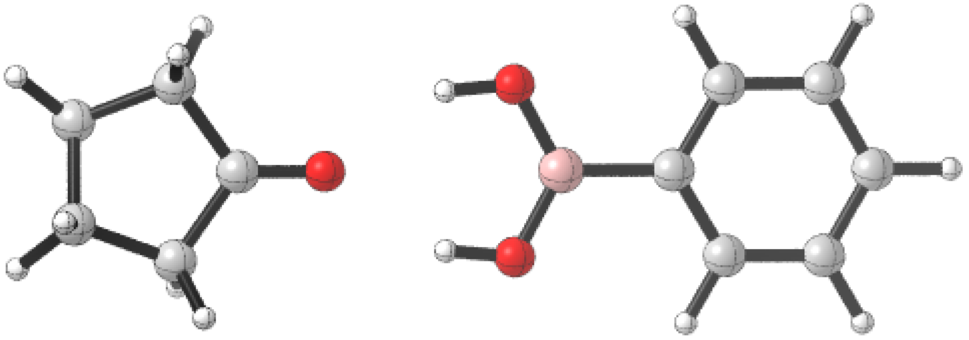

phenyl

30

O 2.5203 -0.1057 -0.0001

B	1.3451	-0.8033	0.0
C	0.0	0.0	0.0
C	0.0	1.3961	0.0
C	-1.1893	2.111	0.0
C	-2.4026	1.4349	0.0
C	-2.4225	0.046	-0.0001
C	-1.2292	-0.662	0.0
O	1.2882	-2.1687	0.0001
H	3.3004	-0.6743	-0.0006
H	0.9485	1.9199	-0.0001
H	-1.1735	3.1937	-0.0001
H	-3.3324	1.9901	-0.0001
H	-3.3682	-0.4814	0.0
H	-1.2405	-1.7455	0.0001
H	2.1588	-2.5859	0.0005
O	4.1762	-2.4941	-0.0006
C	5.2125	-3.1132	-0.0016
C	5.3336	-4.623	0.0152
H	4.6066	-5.0827	-0.6521
H	5.0914	-4.9419	1.0346
C	6.5993	-2.5043	-0.0199
H	6.7639	-2.1391	-1.0393
H	6.6606	-1.6469	0.648
C	6.8054	-4.8888	-0.3049
H	7.1641	-5.8359	0.0924
H	6.9524	-4.905	-1.387
C	7.5313	-3.6748	0.2978
H	8.5347	-3.5413	-0.1008
H	7.6168	-3.7973	1.3798

3-F

30

O	2.5193	-0.1118	0.0028
B	1.3453	-0.8078	0.0
C	0.0	0.0	0.0
C	0.0	1.3958	0.0
C	-1.191	2.1081	-0.0003
C	-2.4069	1.4376	-0.0004
C	-2.394	0.056	-0.0003
C	-1.2226	-0.6724	-0.0002
O	1.2783	-2.171	-0.0028
H	3.2992	-0.6814	0.0022
H	0.9472	1.9201	0.0001
H	-1.1799	3.1904	-0.0004
H	-3.353	1.962	-0.0005
F	-3.5703	-0.5974	-0.0003
H	-1.2615	-1.7543	-0.0003
H	2.1454	-2.596	-0.0029
O	4.1544	-2.5055	-0.0043
C	5.1853	-3.1342	-0.0092
C	5.2925	-4.6447	-0.0023
H	4.5596	-5.0938	-0.6702
H	5.0505	-4.9676	1.016
C	6.5772	-2.5376	-0.024
H	6.7437	-2.165	-1.0405
H	6.647	-1.6864	0.6511
C	6.7613	-4.9215	-0.3278
H	7.1118	-5.8749	0.0618
H	6.9055	-4.9312	-1.4103
C	7.4994	-3.719	0.2824

H	8.5029	-3.5913	-0.1178
H	7.5865	-3.8506	1.3631

3-Me

33

O	2.5203	-0.106	0.0036
B	1.345	-0.8035	0.0000
C	0.0	0.0	0.0000
C	0.0	1.3972	0.0000
C	-1.1936	2.1002	-0.0001
C	-2.4034	1.4156	0.0000
C	-2.4367	0.0252	0.0001
C	-1.2255	-0.663	0.0000
O	1.2897	-2.1694	-0.0033
H	3.3008	-0.674	0.0030
H	0.9461	1.9246	0.0000
H	-1.1891	3.1832	-0.0003
H	-3.3355	1.9696	0.0001
C	-3.741	-0.7251	0.0005
H	-1.2312	-1.7484	0.0000
H	2.1606	-2.5856	-0.0029
O	4.1792	-2.4932	-0.0016
C	5.2165	-3.1105	-0.0038
C	5.3403	-4.6202	0.0062
H	4.614	-5.0781	-0.6630
H	5.0992	-4.9441	1.0244
C	6.6023	-2.499	-0.0178
H	6.7667	-2.1272	-1.0348

H	6.6616	-1.6457	0.6554
C	6.8126	-4.8816	-0.3155
H	7.173	-5.8302	0.0769
H	6.9593	-4.8921	-1.3977
C	7.5364	-3.6697	0.2934
H	8.5395	-3.5323	-0.1050
H	7.6227	-3.7979	1.3746
H	-3.8229	-1.3659	-0.8792
H	-3.8212	-1.3679	0.8789
H	-4.5903	-0.0427	0.0021

2-naph

36

O	2.4453182234	0.1197659493	0.1482160972
B	1.3731352769	-0.7192399779	0.0314459936
C	-0.0663531112	-0.1009075176	0.0283518424
C	-0.2493320435	1.2551957565	0.1401165779
C	-1.5402857473	1.8360821258	0.1389710786
C	-2.672638415	0.9906165208	0.0188198263
C	-2.4721249116	-0.4075736652	-0.0958282964
C	-1.2124466098	-0.9327318263	-0.0910206741
O	1.4940091215	-2.0752291383	-0.0833293809
H	3.2929610355	-0.3419511984	0.1407645684
C	5.7366088139	-4.0576711765	-0.0472930278
H	5.4712356725	-4.481931465	0.9270154392
H	5.0924928961	-4.5220973935	-0.7919340223
C	7.8179506667	-2.9445602124	0.4409012253
H	8.8240845212	-2.6895161393	0.1150177296

H	2.4110315498	-2.3776522739	-0.0735546237
O	4.3873285964	-2.0467188044	0.0378396915
C	5.4758696808	-2.5686633585	0.048384492
H	7.4470206321	-4.0769076598	-1.3667005439
H	6.7443842752	-1.037783272	0.894524834
C	6.7985448517	-1.839274991	0.1599500571
H	6.9812705099	-1.3783470314	-0.8167284093
H	7.8572856974	-3.1486504211	1.5131265948
H	0.6145755599	1.9049702824	0.2320823079
H	7.6658124037	-5.1050528443	0.0457998984
H	-1.0665308207	-2.0023976555	-0.1791862888
C	7.2419423314	-4.1637366328	-0.2974664856
C	-1.7368767746	3.2343720152	0.2537896047
H	-3.3399369757	-1.0506208274	-0.1875089745
C	-3.9639706166	1.5716331649	0.0173290401
C	-2.9947145752	3.7672873037	0.2494236279
H	-0.8668357224	3.8739456459	0.3453473855
C	-4.1223519057	2.9242349214	0.129487158
H	-4.8265707545	0.9221026618	-0.074817031
H	-3.1338284403	4.8370171895	0.3377110521
H	-5.1146487817	3.3568359454	0.1270420858

3-CF3

33

O	2.5167	-0.1142	-0.0007
B	1.3441	-0.8113	0.0
C	0.0	0.0	0.0
C	0.0	1.3968	0.0

C	-1.1863	2.1142	-0.0008
C	-2.4027	1.4446	-0.0016
C	-2.4147	0.0578	-0.0015
C	-1.2258	-0.66	-0.0006
O	1.2717	-2.1727	0.0002
H	3.299	-0.6806	-0.0023
H	0.9493	1.9187	0.0005
H	-1.17	3.1963	-0.0007
H	-3.3346	1.9931	-0.0023
C	-3.7092	-0.7014	-0.0028
H	-1.2474	-1.7438	-0.0006
H	2.1377	-2.6008	-0.0003
O	4.1368	-2.5216	-0.012
C	5.1348	-3.2017	-0.0241
C	5.1653	-4.7155	-0.0111
H	4.4053	-5.1298	-0.6713
H	4.9153	-5.0214	1.0105
C	6.5547	-2.6763	-0.0552
H	6.73	-2.3191	-1.0757
H	6.6739	-1.8257	0.6135
C	6.6154	-5.0681	-0.3469
H	6.9204	-6.0359	0.0453
H	6.7499	-5.091	-1.4306
C	7.4186	-3.9012	0.2502
H	8.4237	-3.8264	-0.1592
H	7.5085	-4.0313	1.3309
F	-3.82	-1.5003	1.0692
F	-4.7778	0.1047	-0.001
F	-3.8203	-1.4959	-1.0781

3,5-F

30

O	2.5177	-0.0773	-0.0028
B	1.3572	-0.7924	0.0
C	0.0	0.0000	0.0
C	0.0	1.3948	0.0
C	-1.2049	2.0674	0.0001
C	-2.4233	1.4149	0.0
C	-2.3926	0.0331	-0.0001
C	-1.2146	-0.6856	0.0
O	1.3049	-2.1546	0.0029
H	3.3077	-0.6333	-0.0033
H	0.9279	1.9510	-0.0001
F	-1.2076	3.4097	0.0002
H	-3.3564	1.9596	0.0
F	-3.563	-0.6244	-0.0002
H	-1.243	-1.7671	0.0001
H	2.1774	-2.5693	0.0035
O	4.1793	-2.4400	0.0004
C	5.2225	-3.0488	0.0013
C	5.3586	-4.5566	0.0254
H	4.6354	-5.0275	-0.6381
H	5.1212	-4.8723	1.0471
C	6.6022	-2.4256	-0.0215
H	6.7616	-2.0644	-1.0432
H	6.6556	-1.5638	0.6415
C	6.8328	-4.8091	-0.2949
H	7.2011	-5.7505	0.107
H	6.9787	-4.8296	-1.3771
C	7.5468	-3.5847	0.3009

H	8.5481	-3.4426	-0.0999
H	7.6352	-3.7009	1.3833

3,5-OMe

38

O	2.5234	-0.0633	-0.0056
B	1.36	-0.7806	0.0
C	0.0	0.0	0.0
C	0.0	1.3938	0.0
C	-1.2114	2.0813	0.0003
C	-2.4117	1.3844	-0.0001
C	-2.4082	-0.0035	-0.0005
C	-1.2036	-0.7029	-0.0001
O	1.3276	-2.1469	0.0057
H	3.3129	-0.6188	-0.0072
H	0.9469	1.914	-0.0002
O	-1.3198	3.4372	0.0009
H	-3.3496	1.9228	0.0
O	-3.6337	-0.5938	-0.0011
H	-1.1753	-1.7829	0.0002
H	2.2057	-2.548	0.0068
O	4.2193	-2.4227	-0.0017
C	5.2634	-3.0286	-0.0017
C	5.4041	-4.536	0.0463
H	4.6752	-5.0194	-0.6018
H	5.1784	-4.8353	1.0754
C	6.6419	-2.4028	-0.0498
H	6.7895	-2.0578	-1.0788

H	6.7002	-1.5304	0.5987
C	6.8753	-4.7901	-0.2859
H	7.2507	-5.7238	0.1275
H	7.0095	-4.8282	-1.3691
C	7.5927	-3.5543	0.2815
H	8.5894	-3.4168	-0.1324
H	7.693	-3.6522	1.3646
C	-0.1266	4.1852	0.002
H	-0.4233	5.231	0.0029
H	0.4725	3.9761	0.8924
H	0.4732	3.9778	-0.8883
C	-3.6778	-2.0013	-0.0022
H	-3.1964	-2.4146	0.8882
H	-4.7306	-2.2724	-0.0032
H	-3.195	-2.4132	-0.8926

3,5-CF3

36

O	2.5131	-0.0995	-0.0066
B	1.3499	-0.8069	0.0
C	0.0	0.0	0.0
C	0.0	1.3929	0.0
C	-1.1925	2.1023	0.0003
C	-2.4106	1.4408	0.0
C	-2.4163	0.0547	-0.0004
C	-1.2268	-0.6597	0.0
O	1.2777	-2.1663	0.0069
H	3.3008	-0.6596	-0.0072

H	0.9452	1.9238	-0.0002
C	-1.135	3.6038	0.0013
H	-3.3382	1.9952	0.0
C	-3.7116	-0.707	-0.0013
H	-1.2468	-1.7435	0.0002
H	2.1441	-2.5948	0.0078
O	4.1368	-2.4725	0.0008
C	5.1742	-3.0922	0.0018
C	5.2945	-4.6007	0.0351
H	4.5638	-5.0687	-0.6223
H	5.058	-4.9069	1.0599
C	6.5595	-2.4833	-0.0301
H	6.7185	-2.1307	-1.055
H	6.6244	-1.6175	0.6266
C	6.7649	-4.8705	-0.289
H	7.1247	-5.8131	0.118
H	6.9066	-4.8999	-1.3715
C	7.4936	-3.6498	0.2962
H	8.4946	-3.5202	-0.1095
H	7.5852	-3.7598	1.3789
F	-2.3496	4.1611	-0.0004
F	-0.4829	4.073	-1.0711
F	-0.4866	4.0716	1.0766
F	-4.7776	0.0989	0.0004
F	-3.8165	-1.4996	-1.0766
F	-3.816	-1.5035	1.071

2-naph

36

O	2.5059	0.1435	-0.0051
B	1.4187	-0.6892	0.0
C	0.0	0.0000	0.0
C	0.0	1.3775	0.0
C	-1.1893	2.1339	0.001
C	-2.396	1.4987	0.0019
C	-2.4651	0.0850	0.0017
C	-1.2641	-0.6782	0.0007
O	1.5666	-2.0489	0.0056
H	3.3483	-0.3268	-0.0036
H	0.9506	1.8954	-0.0008
H	-1.1384	3.2153	0.0011
H	-3.3215	2.0632	0.0028
C	-3.7195	-0.5727	0.0025
C	-1.3855	-2.0935	0.0002
H	2.4929	-2.3244	0.0057
O	4.4534	-2.0222	0.0131
C	5.5274	-2.5733	0.0401
C	6.8717	-1.8761	0.0468
H	6.862	-1.0109	0.7074
H	7.0354	-1.5095	-0.9723
C	5.7446	-4.0717	0.073
H	5.499	-4.3980	1.0893
H	5.0649	-4.5822	-0.6071
C	7.8698	-2.9813	0.3965
H	8.8712	-2.7864	0.0187
H	7.9385	-3.0890	1.4811
C	7.2378	-4.2446	-0.2104
H	7.6469	-5.1631	0.2048
H	7.4109	-4.2609	-1.2886
H	-4.6192	0.0318	0.0034

C	-2.6115	-2.6996	0.0007
C	-3.7965	-1.9352	0.002
H	-0.4875	-2.6913	-0.0007
H	-2.6745	-3.7805	0.0002
H	-4.7594	-2.4297	0.0027

2-F

30

O	2.5313	-0.3333	0.0052
B	1.2929	-0.8959	0.0000
C	0.0	0.0	0.0000
C	0.0	1.3893	0.0000
C	-1.156	2.1506	0.0001
C	-2.3813	1.5037	0.0005
C	-2.4351	0.1155	0.0006
C	-1.2574	-0.6146	0.0002
O	1.0896	-2.2489	-0.0048
H	3.2391	-0.9907	0.0057
F	1.1634	2.0546	-0.0003
H	-1.0766	3.2292	-0.0002
H	-3.2937	2.0863	0.0006
H	-3.3907	-0.3917	0.0007
H	-1.2915	-1.6969	0.0000
H	1.9106	-2.7566	-0.0042
O	3.9242	-2.8784	0.0244
C	4.9311	-3.5419	0.0817
C	6.3422	-2.9937	0.1283
H	6.4069	-2.1319	0.7902

H	6.5739	-2.6478	-0.8848
C	4.9847	-5.0551	0.1185
H	4.6775	-5.3515	1.1272
H	4.273	-5.4903	-0.5809
C	7.2048	-4.1997	0.5042
H	8.2318	-4.1144	0.1559
H	7.2302	-4.3132	1.5902
C	6.458	-5.3879	-0.1233
H	6.754	-6.3451	0.3007
H	6.6587	-5.4232	-1.1963

2-Me

33

O	2.5118	0.092	-0.0121
B	1.4025	-0.7106	0.0
C	0.0	0.0	0.0
C	0.0	1.3984	0.0
C	-1.1753	2.1336	0.0009
C	-2.3895	1.4651	0.0012
C	-2.4121	0.078	0.0007
C	-1.2374	-0.671	0.0003
O	1.501	-2.075	0.0124
H	3.3405	-0.4027	-0.0119
H	0.9536	1.9117	-0.0007
H	-1.1445	3.2157	0.0013
H	-3.3199	2.0196	0.0017
H	-3.3646	-0.4392	0.0005
C	-1.3406	-2.1739	-0.0001

H	2.4161	-2.3842	0.0125
O	4.4058	-2.1103	-0.0019
C	5.4992	-2.6218	-0.0048
C	5.772	-4.1113	0.0285
H	5.0892	-4.6505	-0.6258
H	5.5724	-4.4398	1.0542
C	6.8172	-1.8765	-0.0427
H	6.9353	-1.5083	-1.0675
H	6.797	-1.0094	0.6152
C	7.2604	-4.2314	-0.303
H	7.7161	-5.1326	0.1015
H	7.3987	-4.2461	-1.3863
C	7.8653	-2.9431	0.2787
H	8.8465	-2.714	-0.1315
H	7.9724	-3.0432	1.361
H	-0.8445	-2.6032	-0.8702
H	-0.851	-2.603	0.8739
H	-2.3859	-2.4817	-0.0039

2-CF3

33

O	2.1996	-0.7018	1.0192
B	1.3147	-0.8770	0.0
C	0.0	0.0000	0.0
C	0.0	1.3970	0.0
C	-1.18	2.1268	0.0357
C	-2.397	1.4635	0.0775
C	-2.4251	0.0775	0.0614

C	-1.2383	-0.6406	0.0137
O	1.4608	-1.7902	-0.9993
H	2.996	-1.2443	0.9403
C	1.3034	2.1469	-0.0612
H	-1.1475	3.2076	0.0196
H	-3.3185	2.0295	0.1089
H	-3.3727	-0.4455	0.0789
H	-1.2706	-1.7230	-0.015
H	2.3051	-2.2613	-0.9714
O	4.2755	-1.8511	-0.5184
C	4.7075	-0.8410	-1.0273
H	5.3949	-1.1778	-2.9915
C	4.6668	-0.5196	-2.506
H	3.6858	-0.7526	-2.9199
C	5.357	0.3040	-0.2848
H	4.5355	0.8589	0.185
H	6.0098	-0.0577	0.5078
C	5.0937	0.9477	-2.594
H	5.5793	1.1919	-3.5366
H	4.2205	1.5926	-2.4897
C	6.0218	1.1368	-1.3819
H	6.144	2.1822	-1.1065
H	7.0119	0.7328	-1.6058
F	1.8887	2.2831	1.1321
F	1.1542	3.3817	-0.5572
F	2.1986	1.5248	-0.86

2,6-F

30

O	2.3086	-0.5103	-0.8799
B	1.331	-0.8536	0.0
C	0.0	0.0	0.0
C	0.0	1.3884	0.0
C	-1.1444	2.164	0.0039
C	-2.3732	1.522	-0.0001
C	-2.4441	0.1374	-0.004
C	-1.2618	-0.5792	0.0
O	1.4269	-1.8852	0.8799
H	3.0941	-1.0683	-0.8063
F	1.18	2.0251	0.0222
H	-1.0573	3.2411	0.0125
H	-3.2839	2.106	-0.0001
H	-3.3867	-0.3911	-0.0127
F	-1.3483	-1.9172	-0.0223
H	2.2617	-2.3663	0.8063
O	4.0864	-2.6208	-0.0003
C	5.1025	-3.2731	-0.001
C	5.3889	-4.4529	0.9043
H	4.5156	-5.0956	1.0004
H	5.6021	-4.0379	1.8953
C	6.2935	-3.0417	-0.9073
H	6.5142	-1.98	-1.0029
H	6.004	-3.4077	-1.8982
C	6.6384	-5.0978	0.3022
H	7.2276	-5.6488	1.0321
H	6.3515	-5.7948	-0.4883
C	7.4007	-3.9096	-0.3068
H	8.1462	-4.2155	-1.0377
H	7.9157	-3.3591	0.4835

2,4-Me

36

O	2.5113	0.0825	-0.0089
B	1.3975	-0.7152	0.0
C	0.0	0.0	0.0
C	0.0	1.396	0.0
C	-1.1745	2.1339	0.0012
C	-2.4023	1.4867	0.0018
C	-2.4115	0.0937	0.0016
C	-1.2442	-0.6612	0.0008
O	1.4916	-2.0807	0.0093
H	3.3374	-0.4161	-0.0091
H	0.953	1.9107	-0.0006
H	-1.1372	3.2172	0.0017
C	-3.6959	2.2534	0.0009
H	-3.3675	-0.421	0.0021
C	-1.3564	-2.1634	0.0008
H	2.4055	-2.393	0.0107
O	4.4021	-2.1279	0.005
C	5.5006	-2.6281	0.0102
C	6.8111	-1.8695	-0.0314
H	6.7796	-0.9955	0.6168
H	6.929	-1.5115	-1.0598
C	5.7889	-4.1143	0.0595
H	5.5883	-4.4348	1.0875
H	5.1145	-4.667	-0.5922
C	7.8689	-2.9216	0.3053
H	8.8494	-2.6867	-0.1035

H	7.9727	-3.0091	1.3891
C	7.2797	-4.2221	-0.265
H	7.7433	-5.1143	0.1507
H	7.4226	-4.2468	-1.3475
H	-0.8634	-2.5951	-0.87
H	-0.867	-2.5947	0.8738
H	-2.4031	-2.4662	-0.0013
H	-4.2847	2.0228	-0.889
H	-4.3041	1.9931	0.8692
H	-3.5176	3.3278	0.0206

4-F

30

O	2.5175	-0.1057	-0.0003
B	1.3428	-0.8035	0.0000
C	0.0	0.0	0.0000
C	0.0	1.3962	0.0000
C	-1.1832	2.1198	0.0001
C	-2.3754	1.4214	0.0000
C	-2.4273	0.0407	0.0000
C	-1.2304	-0.66	0.0000
O	1.2831	-2.1685	0.0003
H	3.2975	-0.6747	-0.0008
H	0.9475	1.9211	-0.0002
H	-1.1992	3.2012	0.0000
F	-3.5281	2.1112	0.0000
H	-3.3879	-0.4564	0.0000
H	-1.245	-1.7431	0.0002

H	2.1533	-2.587	0.0007
O	4.1688	-2.4944	0.0003
C	5.2049	-3.1142	0.0012
C	5.3251	-4.6238	0.0196
H	4.5987	-5.0839	-0.6481
H	5.0815	-4.9415	1.0391
C	6.5919	-2.5059	-0.0158
H	6.7579	-2.1418	-1.0354
H	6.6528	-1.6479	0.6513
C	6.7972	-4.8907	-0.2984
H	7.1547	-5.8377	0.1003
H	6.9455	-4.9081	-1.3803
C	7.5229	-3.6765	0.3042
H	8.5269	-3.5439	-0.0934
H	7.6071	-3.7982	1.3863

4-Me

33

O	2.5192	-0.1166	0.0030
B	1.3396	-0.8081	0.0000
C	0.0	0.0	0.0000
C	0.0	1.3955	0.0000
C	-1.1869	2.1126	-0.0034
C	-2.4158	1.4574	-0.0051
C	-2.4223	0.0644	-0.0088
C	-1.2348	-0.6509	-0.0053
O	1.278	-2.1741	-0.0027
H	3.296	-0.6896	0.0023

H	0.9481	1.9202	-0.0009
H	-1.1643	3.1967	-0.0077
C	-3.7041	2.2328	0.0244
H	-3.3708	-0.4611	-0.0170
H	-1.2567	-1.7343	-0.0101
H	2.1471	-2.594	-0.0021
O	4.1674	-2.5136	-0.0012
C	5.2014	-3.1362	-0.0029
C	5.3174	-4.6466	0.0098
H	4.5897	-5.1018	-0.6597
H	5.073	-4.9676	1.0280
C	6.5905	-2.532	-0.0181
H	6.7574	-2.1645	-1.0363
H	6.6539	-1.6767	0.6523
C	6.7887	-4.9164	-0.3095
H	7.1437	-5.8659	0.0855
H	6.9367	-4.9301	-1.3915
C	7.5181	-3.7065	0.2973
H	8.5224	-3.5755	-0.0999
H	7.602	-3.8324	1.3790
H	-4.0394	2.3841	1.0531
H	-3.5839	3.2156	-0.4310
H	-4.496	1.7024	-0.5044

4-OMe

34

O	2.5167	-0.1243	0.0088
B	1.3336	-0.8113	0.0

C	0.0	0.0	0.0
C	0.0	1.4005	0.0
C	-1.1761	2.122	-0.0012
C	-2.4008	1.453	-0.0017
C	-2.4324	0.0619	-0.0011
C	-1.2339	-0.6431	-0.0007
O	1.2686	-2.178	-0.0096
H	3.2915	-0.6996	0.0086
H	0.9484	1.9244	0.0006
H	-1.182	3.2042	-0.0018
O	-3.5067	2.2411	-0.0028
H	-3.3698	-0.4753	-0.001
H	-1.2598	-1.7264	-0.0006
H	2.1371	-2.599	-0.0121
O	4.1545	-2.5346	-0.042
C	5.1706	-3.1795	-0.1338
C	5.2463	-4.689	-0.2385
H	4.4803	-5.0744	-0.9091
H	5.0345	-5.0806	0.7622
C	6.5737	-2.6091	-0.1628
H	6.7093	-2.1699	-1.157
H	6.687	-1.8087	0.5662
C	6.6959	-4.9687	-0.6389
H	7.041	-5.9534	-0.3311
H	6.7996	-4.9041	-1.7243
C	7.4813	-3.826	0.0249
H	8.472	-3.6893	-0.4033
H	7.6052	-4.0352	1.0897
C	-4.7641	1.6075	-0.0033
H	-5.5059	2.4019	-0.0053
H	-4.8951	0.9863	-0.8938
H	-4.8972	0.9887	0.8884

4-tBu

42

O	2.5186360603	-0.1225882468	-0.0026638992
B	1.3377624028	-0.8116346092	0.0000759691
C	0.0000949767	0.0000258068	-0.0000211444
C	0.0000217394	1.3970820756	-0.0001264883
C	-1.183800342	2.1135133705	-0.0000825219
C	-2.4242619308	1.4684613802	-0.0004449123
C	-2.4253277268	0.0758375923	-0.0004616055
C	-1.2349410677	-0.6419603196	-0.0000463523
O	1.2729786742	-2.177285486	0.0032654892
H	3.294073687	-0.697537671	-0.003300606
H	0.9477581931	1.922601846	-0.0003061944
H	-1.1421570206	3.1963744241	0.0000741263
C	-3.7065437341	2.2992311431	-0.000000889
H	-3.359670492	-0.4684941938	-0.0006790713
H	-1.2639112196	-1.7253030939	0.0002662059
H	2.1409546899	-2.5994675821	0.0043035071
O	4.1635353129	-2.5206498637	0.0024424439
C	5.2019387111	-3.1358908482	0.0048451248
C	5.3286161061	-4.6450560392	0.0372932456
H	4.6015643903	-5.1143625577	-0.623136657
H	5.0902299123	-4.9539946661	1.0607187682
C	6.586607427	-2.5222524282	-0.0240729254
H	6.746888799	-2.1674596132	-1.0478479261
H	6.6466875242	-1.6574572497	0.6343617577
C	6.8005938877	-4.908882569	-0.2839302634

H	7.1637876827	-5.8504040602	0.1225648997
H	6.9446105759	-4.9363045296	-1.3662554015
C	7.5237166927	-3.6858238195	0.303558306
H	8.5255636121	-3.5531592479	-0.0993533919
H	7.6126984698	-3.7964400863	1.3864824759
C	-3.7359814526	3.1911647356	-1.2491339275
H	-4.6493308879	3.7904425197	-1.2609285528
H	-2.8849744755	3.8721451482	-1.2750720909
H	-3.7100186721	2.5841547086	-2.1557981278
C	-4.9624185872	1.4278580816	-0.0045874023
H	-5.0105888182	0.7892952443	0.8792441535
H	-5.8474602865	2.0665803317	-0.0049213986
H	-5.0067146717	0.7934911295	-0.8916532758
C	-3.7392711966	3.1833846648	1.2545823607
H	-4.6518609559	3.7836963462	1.2671038851
H	-3.7170855768	2.5705770213	2.1574533828
H	-2.8873004126	3.8628912109	1.287488924

4-CF3

33

O	2.5179	-0.0988	-0.0016
B	1.35	-0.8033	0.0000
C	0.0	0.0	0.0000
C	0.0	1.3965	0.0000
C	-1.1867	2.1102	-0.0017
C	-2.3951	1.4242	-0.0045
C	-2.4223	0.0377	-0.0033
C	-1.2256	-0.6646	-0.0019

O	1.2886	-2.1658	0.0019
H	3.3028	-0.6618	-0.0019
H	0.9468	1.9221	-0.0010
H	-1.1823	3.1927	-0.0067
C	-3.6733	2.2108	0.0340
H	-3.3698	-0.4838	-0.0088
H	-1.2364	-1.7475	-0.0041
H	2.158	-2.5868	0.0027
O	4.1633	-2.4748	0.0022
C	5.2038	-3.0882	0.0029
C	5.3334	-4.5965	0.0289
H	4.6076	-5.0651	-0.6334
H	5.0956	-4.9099	1.0512
C	6.5863	-2.471	-0.0220
H	6.7463	-2.1117	-1.0443
H	6.644	-1.6087	0.6399
C	6.8062	-4.8558	-0.2925
H	7.1709	-5.7983	0.1102
H	6.951	-4.8783	-1.3748
C	7.5261	-3.6338	0.3010
H	8.5277	-3.4966	-0.1009
H	7.615	-3.7489	1.3834
F	-3.6309	3.2754	-0.7789
F	-4.7345	1.4809	-0.3267
F	-3.9282	2.6832	1.2647

Enamine - xyz structures

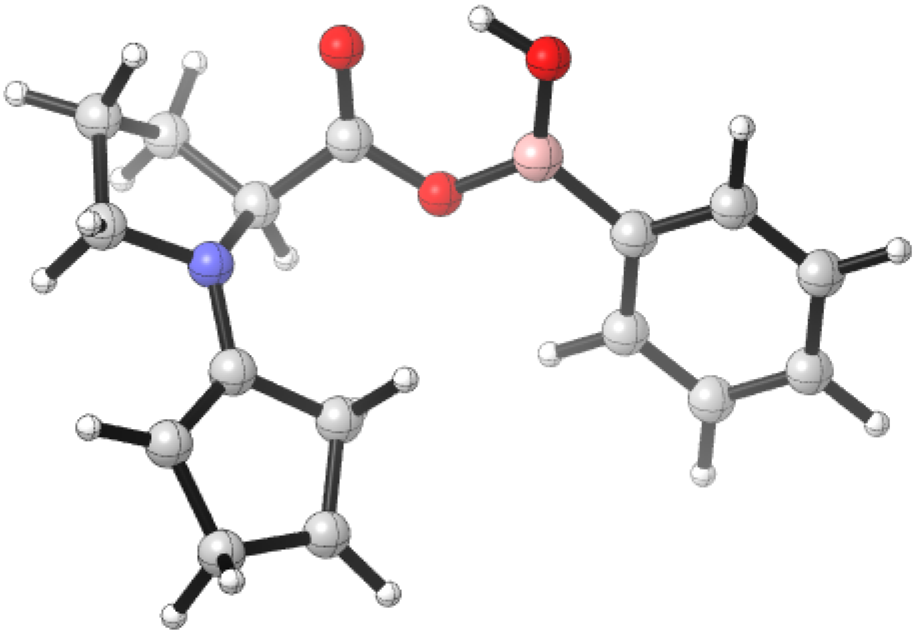

3-F_far

40

O	-1.1232694573	-0.63439543	-0.126282399
B	0.0241772725	0.05404215	-0.013314843
C	1.3965935257	-0.68061451	0.042789223
C	2.5937662231	0.01338574	0.228130050
C	3.8053351404	-0.66182470	0.275273965
C	3.8409928261	-2.04185724	0.136036134
C	2.6486994939	-2.71828109	-0.045687415
C	1.4328980201	-2.06985782	-0.093621772
O	0.0643378803	1.46221292	0.052927611
H	-1.8974546361	-0.05078874	-0.130861994
H	2.5724625916	1.08997258	0.340092976
H	4.7286278839	-0.11644762	0.420310659
H	4.7683300133	-2.59782712	0.166750276
F	2.6821267652	-4.05292151	-0.178947082

H	0.5238470436	-2.64022041	-0.235786959
C	-1.0147579163	2.25965918	0.008557399
C	-2.5766992011	4.85759167	-0.930761208
H	-1.3963369313	5.57197406	0.734670864
H	-2.3992533217	4.17237651	1.153161646
H	-1.0660931857	6.02334417	-1.967764346
H	-1.7586854376	4.73725468	-2.968866702
H	-3.2141790319	5.73881662	-0.893421426
H	-3.1933246527	3.98741061	-1.153198455
N	-0.4245056994	4.08030324	-1.480911056
C	0.838930507	4.18983080	-2.035901662
C	1.9964709036	3.34642679	-1.548769123
C	1.1871351998	4.95622456	-3.079742153
C	3.1107142433	3.79111052	-2.434389217
H	1.767844105	2.27763062	-1.651270739
C	2.633147607	4.76837308	-3.458837032
H	0.5222058153	5.61408405	-3.619483839
H	4.1080465935	3.38184653	-2.395209631
H	3.1974419768	5.70991638	-3.433156796
H	2.7481589796	4.38516881	-4.482712092
H	2.2079833805	3.49865329	-0.481623624
O	-2.1467166304	1.84921935	0.006118840
C	-1.4580125787	4.99705129	-1.952633938
C	-0.6410499268	3.73426503	-0.071103635
H	0.2605466796	3.89070393	0.522057865
C	-1.8016835238	4.62822644	0.366781629

3-F

40

O	-1.1373713954	-0.63332886	-0.12304346
B	0.0121661808	0.05161066	-0.01074785
C	1.3848652184	-0.68312426	0.03836264
C	1.4394663415	-2.07092163	-0.10866481
C	2.6538820116	-2.73970006	-0.07238001
C	3.8357492061	-2.03493574	0.11388834
C	3.7686885538	-0.66281375	0.26102358
C	2.5731194762	0.02462639	0.22693261
O	0.0560716161	1.45948648	0.06001820
H	-1.9090493663	-0.04626384	-0.12324036
H	0.5187537339	-2.62161694	-0.25291314
H	2.6879228222	-3.81493082	-0.18898691
H	4.7982319866	-2.52739660	0.14730835
F	4.9083981705	0.02304309	0.44319372
H	2.5748437372	1.10016111	0.34958031
C	-1.0201897879	2.26106258	0.01712617
C	-2.5722872281	4.86203598	-0.92913257
H	-1.3874372019	5.57730326	0.73260389
H	-2.3958057367	4.18337113	1.15719372
H	-1.0571049423	6.01788624	-1.97034076
H	-1.7561947981	4.73224046	-2.96749328
H	-3.2062194566	5.74592090	-0.89435332
H	-3.1924685992	3.99347332	-1.14809959
N	-0.4239808488	4.07328905	-1.47872211
C	0.8403696552	4.17767616	-2.03373730
C	1.9954383932	3.33198873	-1.54471770
C	1.1911366387	4.94090000	-3.07895674
C	3.1112928911	3.77095600	-2.43097496
H	1.7632572897	2.26365134	-1.64536911
C	2.6370954241	4.74881604	-3.45641645
H	0.5284110396	5.60004596	-3.61991095

H	4.1095923687	3.36518412	-2.38306725
H	3.2034902723	5.68909131	-3.43053586
H	2.7521189429	4.36522933	-4.48012887
H	2.2081329865	3.48623087	-0.47811081
O	-2.1534641426	1.85418791	0.01857848
C	-1.4535730648	4.99338215	-1.95218110
C	-0.640699732	3.73374348	-0.06727213
H	0.2621464033	3.88832150	0.52447675
C	-1.7970950598	4.63391694	0.36861067

3-Me_far

43

O	-1.1370455007	-0.6336924487	-0.1121270487
B	0.015473697	0.0477767398	0.0088928945
C	1.3852083223	-0.683514737	0.0799873344
C	2.5773546889	0.0131998159	0.2751528929
C	3.7858929986	-0.6671889979	0.3349363666
C	3.8102752181	-2.0461904202	0.1983098453
C	2.6348199402	-2.7718321603	0.0032723012
C	1.4353871407	-2.0745578592	-0.0514922603
O	0.0530667771	1.4597551798	0.069935436
H	-1.9058327649	-0.0433942313	-0.1249428719
H	2.5564208321	1.0906339224	0.384780874
H	4.7097467451	-0.1239466788	0.4877530805
H	4.7565168653	-2.5743132894	0.2443299017
C	2.6805347835	-4.268092498	-0.1418182239
H	0.5088396652	-2.618350051	-0.2004313901
C	-1.0216115502	2.2580530322	0.0119018008

C	-2.5715465975	4.860200143	-0.9425189414
H	-1.3944572049	5.5750398492	0.7250636818
H	-2.4042125919	4.180256557	1.1436989987
H	-1.0533238056	6.0159423674	-1.9807618003
H	-1.747773415	4.7275609486	-2.977419604
H	-3.2053634389	5.7443311391	-0.9103367651
H	-3.1909175207	3.9917080326	-1.1638217721
N	-0.4207929121	4.0730614183	-1.4822629496
C	0.8433235499	4.170531558	-2.0365794063
C	1.9960458565	3.3262789369	-1.5397657235
C	1.1968485277	4.9263082995	-3.0866307847
C	3.1133948686	3.7565652887	-2.4285269234
H	1.7631021505	2.2574448714	-1.6302581461
C	2.642476772	4.7276992624	-3.4619616136
H	0.5359515794	5.5830735489	-3.6326758411
H	4.1076547248	3.3402204058	-2.3859166458
H	3.2117924852	5.6664845415	-3.443344076
H	2.757191678	4.3352596635	-4.482432055
H	2.2070506764	3.4887714214	-0.4740692218
O	-2.1564448666	1.8535088715	0.0016116057
C	-1.4490549521	4.9910774604	-1.9614792917
C	-0.6431852189	3.7316876696	-0.0723192665
H	0.2570368361	3.8874957361	0.5230549931
C	-1.8018875685	4.6316726312	0.3583839973
H	1.6821461662	-4.6798259196	-0.2808656632
H	3.1196230408	-4.7324948493	0.7428432216
H	3.2901432622	-4.5583467715	-0.99928758

3-Me

43

O	-1.1475018094	-0.626709421	-0.087169180
B	0.0122206932	0.048831041	-0.013209751
C	1.3808991099	-0.687825519	-0.003721673
C	1.4273629179	-2.074259844	-0.156781857
C	2.6455204982	-2.736598611	-0.165660162
C	3.8254781681	-2.021119302	-0.019173851
C	3.811960767	-0.637165691	0.142218218
C	2.5818460279	0.009703096	0.145975506
O	0.059757591	1.460665950	0.048488280
H	-1.9134248713	-0.032904757	-0.070625095
H	0.5028746501	-2.626212801	-0.273627011
H	2.679818786	-3.811370625	-0.290458227
H	4.7757548183	-2.543538774	-0.031736261
C	5.0945197313	0.127303908	0.324071585
H	2.5541699547	1.086872974	0.269973210
C	-1.0109292207	2.265980316	0.029933954
C	-2.562233527	4.877632552	-0.893290985
H	-1.3471134736	5.585659336	0.749476014
H	-2.3568750544	4.197171874	1.189491295
H	-1.0573396866	6.026288505	-1.957328382
H	-1.7786661373	4.745046346	-2.944333951
H	-3.1913195772	5.764560011	-0.848440505
H	-3.1896807582	4.011963104	-1.102818892
N	-0.4275270913	4.077784911	-1.476672191
C	0.8271509104	4.172596459	-2.053023608
C	1.9832414344	3.316068858	-1.585539996
C	1.1665004023	4.934607049	-3.103242837

C	3.0881091711	3.749781191	-2.488111732
H	1.7414291208	2.250009561	-1.685522631
C	2.6039202306	4.730632157	-3.506151825
H	0.5001170175	5.599623501	-3.632339027
H	4.0797712143	3.325154878	-2.469167075
H	3.1786551735	5.666136592	-3.491749134
H	2.697918529	4.344472680	-4.531100011
H	2.2129768352	3.463838556	-0.521502213
O	-2.1480583253	1.869064874	0.057755346
C	-1.4589378498	5.003810320	-1.933612242
C	-0.624539658	3.737122185	-0.062421857
H	0.2885815842	3.886308312	0.514856256
C	-1.7679410782	4.644666509	0.391914042
H	4.9457184875	1.191669107	0.144078928
H	5.8662139283	-0.230869982	-0.357986943
H	5.4757003656	0.010448613	1.340786467

2-naph_close

46

O	-1.0619801402	-0.6385041368	-0.0814269522
B	0.0821743408	0.0618339334	0.0016050982
C	1.4627581676	-0.651038147	0.0273706718
C	2.6300447434	0.0586833349	0.1750647993
C	3.8918328642	-0.5807728126	0.194393406
C	3.9462110521	-1.9911817296	0.0584952078
C	2.7346684461	-2.7128377371	-0.0898515854
C	1.5346734664	-2.0652579613	-0.1041726228
O	0.1023443508	1.4735159662	0.0594259029

H	-1.8408099613	-0.0612546436	-0.0727146411
H	-3.23039394	5.7122245558	-0.8666120428
H	-3.1963502766	3.9594267154	-1.11730877
H	2.212382146	3.5035018715	-0.5262125236
C	5.0989770823	0.1451683343	0.342373704
C	1.9863415637	3.3491412959	-1.5901593724
C	-0.9845602701	2.2570727834	0.0274751635
C	3.0855109611	3.7968279095	-2.4928628373
H	1.7603413054	2.2793483407	-1.6878801298
C	2.5873059737	4.7678791331	-3.5136598102
H	0.470810255	5.6053266707	-3.6433594148
H	4.0813101078	3.3818427967	-2.4762818909
H	3.1479231072	5.7119302688	-3.5012241819
H	2.6878060304	4.3804334387	-4.5374610203
N	-0.4357645233	4.0735539572	-1.4852803299
C	0.817914181	4.1869129038	-2.060788513
C	-1.4821001697	4.9806617746	-1.9462611906
C	1.1466561431	4.9514619834	-3.1123619629
H	0.2813352196	3.9054139679	0.5080557495
C	-1.7899169926	4.6213843518	0.3794504614
H	-1.0984384596	6.0100031382	-1.97104822
H	-1.7949198074	4.7143616088	-2.9571684093
H	-2.3723268487	4.164305533	1.1763937348
H	0.613332039	-2.6230265469	-0.2175068965
H	2.5969639181	1.1374231839	0.2836907847
H	-1.3881920121	5.5708329535	0.7365480872
O	-2.1132068573	1.836459946	0.0475186045
C	-2.5851987241	4.8367761786	-0.9081458798
C	-0.6278115273	3.7351274434	-0.0696872333
H	2.7817137329	-3.7906415576	-0.1920137394
C	5.2085619273	-2.6312232087	0.0758105789
C	6.303023473	-0.4994778725	0.355299896

H	5.0481348601	1.2229985761	0.4445182626
C	6.3569958586	-1.9048638363	0.2205316839
H	5.2478015621	-3.7089697632	-0.0281849344
H	7.2210583018	0.0622144494	0.4683060724
H	7.3169026498	-2.4048366155	0.2322479751

2-naph_far

46

O	-1.1128772887	-0.65042530	-0.066459880
B	0.0390190754	0.03350707	0.045498057
C	1.4112275405	-0.69400177	0.104064473
C	1.4616569549	-2.05982248	-0.042961167
C	2.6860410044	-2.76602092	-0.005846701
C	3.8894943307	-2.04189329	0.192701708
C	3.8245994885	-0.63491009	0.348052838
C	2.6262816402	0.01619751	0.303687869
O	0.0767545134	1.44491876	0.104512057
H	-1.8830087419	-0.06189685	-0.072921802
H	-3.1893663818	5.71425775	-0.910119802
H	-3.1738138009	3.95826318	-1.139848053
H	2.2263123648	3.46274448	-0.471234463
C	2.7460359313	-4.17307455	-0.160860475
C	2.011250032	3.29103601	-1.534678779
C	-0.9992350416	2.24294749	0.047344411
C	3.1245516504	3.71424342	-2.431850883
H	1.7789840252	2.22116440	-1.615632577
C	2.6476650924	4.67337897	-3.473667963
H	0.5397254758	5.52473701	-3.644158175

H	4.1191437564	3.29859249	-2.389975705
H	3.215711454	5.61305369	-3.468149714
H	2.7580186772	4.26990415	-4.490298864
N	-0.4064447736	4.03686248	-1.473581011
C	0.8554573688	4.12956613	-2.034470800
C	-1.4370491008	4.94882985	-1.959202500
C	1.2035779181	4.87447647	-3.093950694
H	0.2820378729	3.87874524	0.530491867
C	-1.7783974391	4.62044496	0.366842427
H	-1.0416869932	5.97347709	-1.992655750
H	-1.7404701461	4.67287190	-2.970394697
H	-2.3763530348	4.17955626	1.161428344
H	2.5904266265	1.09178796	0.426314858
H	0.5425742496	-2.61607958	-0.193351902
H	-1.3695334896	5.56900379	0.718253079
O	-2.133375191	1.83716329	0.051673584
C	-2.5546284343	4.83053138	-0.933343089
C	-0.6215454414	3.71518950	-0.057658566
H	4.7461346952	-0.08614604	0.503105496
C	5.1154882536	-2.74900700	0.230060790
C	3.943354953	-4.82831422	-0.120409240
H	1.8221495364	-4.71842739	-0.312384867
C	5.1420606349	-4.10639512	0.077597424
H	6.0332451832	-2.19348663	0.381971919
H	3.9806654772	-5.90320912	-0.239857267
H	6.0849388817	-4.63710323	0.107533514

3-CF3-far

43

O	-1.1093911332	-0.63750127	-0.10035008
B	0.0332539095	0.05735220	0.01341522
C	1.4083576863	-0.67282833	0.08077243
C	2.6012107974	0.02520803	0.26242536
C	3.8194654833	-0.64036377	0.32068772
C	3.856360435	-2.01822401	0.19594414
C	2.6716690654	-2.72529034	0.01588842
C	1.4580147105	-2.06448615	-0.04058271
O	0.0710142226	1.46483329	0.07117830
H	-1.8874606951	-0.05908008	-0.11142277
H	2.5738821747	1.10355152	0.36257979
H	4.737848536	-0.08658223	0.46307067
H	4.7986244199	-2.55055732	0.23849006
C	2.7514268956	-4.21974856	-0.11540671
H	0.5405806631	-2.62184471	-0.17909222
C	-1.0110695122	2.25980413	0.01630547
C	-2.5784322643	4.84470254	-0.94681376
H	-1.403681605	5.57631598	0.71501544
H	-2.4031249881	4.17711537	1.14310834
H	-1.0687778515	6.00679272	-1.98903978
H	-1.7555467834	4.71136760	-2.98219779
H	-3.2186410566	5.72421799	-0.91780464
H	-3.1918899765	3.97088476	-1.16360449
N	-0.4228215477	4.06913710	-1.48650835
C	0.8417311858	4.17917261	-2.03931216
C	2.0004729893	3.34142767	-1.54506716
C	1.1903796136	4.94020038	-3.08676930
C	3.1155456548	3.78359220	-2.43100548
H	1.7736656382	2.27165218	-1.64292775
C	2.63763181	4.75403796	-3.46164241
H	0.5252193089	5.59354370	-3.63163405

H	4.1138479171	3.37702642	-2.38774647
H	3.1991437335	5.69729335	-3.43963383
H	2.7559490371	4.36577273	-4.48313872
H	2.2091740632	3.50050563	-0.47828424
O	-2.1408174613	1.84471200	0.01319198
C	-1.457957173	4.97961557	-1.96730722
C	-0.6411027967	3.73452307	-0.07429241
H	0.2587771739	3.89819925	0.51952885
C	-1.8054489103	4.62836830	0.35415033
F	1.552725639	-4.78589233	-0.26594596
F	3.4972796957	-4.58189591	-1.16963506
F	3.3225801058	-4.77819412	0.96201811

3-CF3

43

O	-1.1414020229	-0.63197795	-0.064550515
B	0.0126596202	0.05108462	-0.001967233
C	1.3850833888	-0.68725235	-0.012572959
C	1.4411109993	-2.07388801	-0.176056366
C	2.6562604436	-2.73960624	-0.205434818
C	3.8410355929	-2.02826704	-0.069606699
C	3.795888373	-0.65197654	0.096351723
C	2.579991923	0.01487701	0.127526003
O	0.069271307	1.45630010	0.063577921
H	-1.9113470835	-0.04306439	-0.035044849
H	0.5161793499	-2.62704864	-0.285057607
H	2.6871103751	-3.81290015	-0.337568061
H	4.7950176401	-2.53753310	-0.098336332

C	5.0608758707	0.13552646	0.283332299
H	2.5616689155	1.09010458	0.257691976
C	-1.00289913	2.26641264	0.057649417
C	-2.5556115187	4.86303826	-0.883849790
H	-1.3339892998	5.59236679	0.744857253
H	-2.3410469583	4.20944456	1.207071013
H	-1.0555873275	6.00144637	-1.963147141
H	-1.7787449301	4.71065498	-2.936278791
H	-3.1856211553	5.74963466	-0.847201692
H	-3.1833690144	3.99458988	-1.080934916
N	-0.4210096665	4.05833303	-1.466867907
C	0.8363510251	4.16292485	-2.040445367
C	1.9996915002	3.31753925	-1.569822858
C	1.1700940464	4.92444517	-3.092277829
C	3.1019351453	3.75835636	-2.471998914
H	1.765570847	2.24951353	-1.671668741
C	2.6104114098	4.73392959	-3.491299761
H	0.4982792218	5.58126645	-3.624782862
H	4.1039868546	3.36185722	-2.430265945
H	3.1753061418	5.67526184	-3.475504995
H	2.7099092547	4.34937636	-4.516321016
H	2.2301433944	3.46786935	-0.506229931
O	-2.1369047878	1.86519304	0.103758945
C	-1.4559976708	4.97900334	-1.929139869
C	-0.6143725347	3.73511259	-0.047867205
H	0.3008636591	3.88770657	0.525101620
C	-1.7561829526	4.64699023	0.400985624
F	5.0120740574	1.31268452	-0.355272072
F	5.2862084413	0.41222793	1.576389471
F	6.136707255	-0.51725773	-0.167520222

3,5-F

40

O	-1.1306122507	-0.63646463	-0.111036302
B	0.0151385616	0.05370217	-0.006058033
C	1.38962074	-0.68364648	0.042452513
C	1.4272899892	-2.07182750	-0.094567624
C	2.6470651255	-2.71560221	-0.055030039
C	3.8410964637	-2.03971744	0.117728601
C	3.7728763941	-0.66657459	0.253532244
C	2.5783579768	0.02470788	0.219688918
O	0.0618837143	1.45907831	0.058146412
H	-1.9056698629	-0.05356806	-0.112624477
H	0.5196082068	-2.64439604	-0.230741178
F	2.6895717572	-4.04680078	-0.187921587
H	4.7859528467	-2.56326496	0.145953399
F	4.9151589059	0.01161471	0.423816312
H	2.5818297042	1.10039635	0.333459067
C	-1.0169030281	2.26045639	0.016207070
C	-2.5738677594	4.85665542	-0.932409724
H	-1.3900789857	5.57583589	0.728528982
H	-2.3955985243	4.18049189	1.154732042
H	-1.0606212302	6.01462104	-1.973162516
H	-1.7577607737	4.72810760	-2.970788208
H	-3.2093955658	5.73937290	-0.898176907
H	-3.192660891	3.98693333	-1.150727919
N	-0.4241657998	4.07091371	-1.482144751
C	0.8409599394	4.18095401	-2.035311663
C	1.9977514432	3.33730150	-1.547030587
C	1.1906829406	4.94719680	-3.078483693

C	3.1135180913	3.78172893	-2.430732731
H	1.7680473514	2.26879731	-1.651896069
C	2.6377375044	4.76064001	-3.454371866
H	0.5266064255	5.60514546	-3.619187863
H	4.1140476137	3.38176393	-2.380373235
H	3.2009766946	5.70266041	-3.425044106
H	2.7554102598	4.38022774	-4.478907953
H	2.2087521099	3.48899254	-0.479680365
O	-2.14823754	1.84978541	0.019986831
C	-1.4555919486	4.98958493	-1.955489970
C	-0.639791853	3.73330648	-0.069984895
H	0.2628892962	3.89009029	0.521486590
C	-1.7980740425	4.63139933	0.365465280

3,5-Me

46

O	-1.147025352	-0.61371284	-0.02872614
B	0.0138483842	0.06092202	0.03980895
C	1.3825412584	-0.67462949	0.06514883
C	1.4287946885	-2.06441805	-0.07028631
C	2.6364809001	-2.74855913	-0.06669188
C	3.8124360987	-2.01443646	0.08175741
C	3.8055471482	-0.63225048	0.22674550
C	2.5787558399	0.02490119	0.21345134
O	0.0614194766	1.47400293	0.08115114
H	-1.9116019999	-0.01795857	-0.02208564
H	0.500826461	-2.61346497	-0.18506841
C	2.6921306198	-4.24273457	-0.23088178

H	4.7631089937	-2.53984761	0.08265607
C	5.0879781902	0.13048778	0.41810316
H	2.5544122168	1.10358109	0.32332651
C	-1.0078527342	2.27973089	0.04646534
C	-2.5570453749	4.88151387	-0.91411411
H	-1.3349760916	5.61110334	0.71418983
H	-2.3467592371	4.23193977	1.17791500
H	-1.0521001487	6.00778994	-2.00210824
H	-1.7818221099	4.71336375	-2.96546318
H	-3.1826655666	5.77141318	-0.88083491
H	-3.1884034502	4.01498604	-1.10774993
N	-0.4276195653	4.06504689	-1.49233994
C	0.8237779259	4.14191524	-2.07801581
C	1.9787851314	3.28953629	-1.60053206
C	1.1599927053	4.88164796	-3.14510252
C	3.0792223311	3.69824048	-2.52015924
H	1.731887995	2.22258663	-1.67488685
C	2.5936325003	4.66247442	-3.55337280
H	0.4934135556	5.53989349	-3.68235494
H	4.0675169561	3.26585002	-2.50227409
H	3.1733552299	5.59503420	-3.56001114
H	2.6791092982	4.25676887	-4.57148329
H	2.2168965462	3.45965969	-0.54166293
O	-2.1458829623	1.88526938	0.07914936
C	-1.4572431106	4.98722498	-1.96024389
C	-0.6192834645	3.74849864	-0.07178196
H	0.2965110139	3.90580567	0.49914975
C	-1.7591203543	4.66589987	0.37194697
H	1.6923265282	-4.67402154	-0.23962581
H	3.2563981895	-4.70515189	0.58044314
H	3.18503527	-4.51289257	-1.16674636
H	5.9173276114	-0.35510031	-0.09583566

H	5.3467709998	0.19266797	1.47745581
H	4.9983614584	1.14902203	0.04027569

3,5-OMe

48

O	-1.1322634135	-0.61333151	0.01567624
B	0.0280268138	0.06410187	0.06476048
C	1.396085404	-0.67814581	0.07528089
C	1.4128090844	-2.07601382	-0.04419152
C	2.626574381	-2.74172172	-0.04349720
C	3.8288499415	-2.03533131	0.07607307
C	3.8033866413	-0.65725307	0.19655921
C	2.5827485583	0.02416749	0.19651575
O	0.0767333648	1.47487016	0.09792595
H	-1.8971591392	-0.01765777	0.02932584
H	0.4750615696	-2.60427578	-0.13536589
O	2.7649070317	-4.08666811	-0.15471651
H	4.7476303229	-2.60297514	0.06994016
O	4.9134958971	0.11210220	0.31800130
H	2.5921600172	1.10135641	0.29662331
C	-0.9946496347	2.27957060	0.07462805
C	-2.5589541096	4.87221451	-0.88067318
H	-1.3228204343	5.61262684	0.73202655
H	-2.3267930962	4.23353704	1.21253818
H	-1.0685501957	5.99709141	-1.99030873
H	-1.8035567567	4.69482676	-2.93860792
H	-3.1864817819	5.76072569	-0.84565945
H	-3.1902800622	4.00339415	-1.06394049

N	-0.4321873925	4.05971801	-1.47541503
C	0.8153189083	4.14117088	-2.07000331
C	1.9785612136	3.30011037	-1.59369376
C	1.1398261605	4.87632293	-3.14376749
C	3.0710085404	3.71011596	-2.52168806
H	1.7388498587	2.23115980	-1.66065511
C	2.5731823547	4.66456046	-3.55813059
H	0.4654361298	5.52623029	-3.68155341
H	4.0655448168	3.29324748	-2.49542336
H	3.1459298714	5.60135588	-3.57364558
H	2.6556768517	4.25370598	-4.57452533
H	2.2231479139	3.47753737	-0.53773112
O	-2.1310347041	1.88181107	0.12265856
C	-1.4697535117	4.97549415	-1.93828917
C	-0.6103593345	3.74848090	-0.05201826
H	0.3102589475	3.90911486	0.50993244
C	-1.7481569691	4.66483652	0.39862981
C	1.5884470737	-4.85430967	-0.27976780
C	6.1644697089	-0.53685846	0.32150141
H	1.9084137618	-5.89002598	-0.35556500
H	1.0305234171	-4.57766664	-1.17802027
H	0.9439891786	-4.73403496	0.59481813
H	6.9125986663	0.24416462	0.42654872
H	6.3317534477	-1.07777861	-0.61386952
H	6.2457946869	-1.23377430	1.16005903

3,5-CF3

46

O	-1.1214840602	-0.61797241	0.0191732364
B	0.0232664338	0.07846982	0.0666509725
C	1.4008910201	-0.65746219	0.073395834
C	1.4546117136	-2.04917419	-0.0548918563
C	2.6717712445	-2.70370802	-0.0647488438
C	3.8626405843	-1.99379934	0.0534740819
C	3.8135575905	-0.61954634	0.1844638385
C	2.5930437006	0.04593195	0.1960413503
O	0.0776571313	1.48125295	0.1005465558
H	-1.899841897	-0.03933081	0.0353133669
H	0.5345975814	-2.61169978	-0.1484770967
C	2.7530595413	-4.19804514	-0.2157888149
H	4.812778432	-2.51213565	0.0412318468
C	5.0717584031	0.18942662	0.3338073137
H	2.5740392547	1.12443774	0.3001144495
C	-1.0019402702	2.28630509	0.0755857706
C	-2.5689933728	4.84517804	-0.9394275896
H	-1.3599713234	5.62510876	0.6751221805
H	-2.3589072085	4.24751919	1.1690581768
H	-1.0709194205	5.96723975	-2.0368230173
H	-1.7808829224	4.64980398	-2.983938827
H	-3.2058955399	5.72743699	-0.9279781459
H	-3.1894031552	3.96742258	-1.117725918
N	-0.4267290226	4.03964514	-1.4933763853
C	0.8337675008	4.14292233	-2.0619134789
C	1.9994992265	3.31208818	-1.5714802475
C	1.168581842	4.88731481	-3.1252702757
C	3.1041977588	3.74448259	-2.4749200495
H	1.7710177317	2.24132330	-1.6588952174
C	2.6118718155	4.69864314	-3.5136657393
H	0.4959057176	5.53068673	-3.6727996359
H	4.1079966234	3.35376446	-2.42283782

H	3.1706457046	5.64366079	-3.5115438443
H	2.7188787797	4.29676463	-4.5311242757
H	2.2229763533	3.48085984	-0.5090724512
O	-2.1310703611	1.87567093	0.1324215072
C	-1.4652480037	4.94346760	-1.981752777
C	-0.6232289538	3.75387689	-0.0665014015
H	0.2884453318	3.92840865	0.50600285
C	-1.7739841914	4.66820357	0.3540967878
F	1.5495378544	-4.77093192	-0.2455932487
F	3.387404925	-4.54011327	-1.3449563424
F	3.4378276132	-4.75405143	0.7920073072
F	6.1710218452	-0.53746678	0.1266607264
F	5.0980617965	1.21349795	-0.5296632568
F	5.1696886512	0.72002229	1.5597984041

2-naph_close

46

O	-0.9597249558	-0.3955926411	0.4475170178
B	0.1668862535	0.3106426994	0.2415710905
C	1.5216516601	-0.4364877149	0.020097454
C	2.8317536126	0.1131312302	0.207871906
C	3.9671259805	-0.6699749347	-0.1375875991
C	3.7844515672	-1.979529265	-0.6436276295
C	2.5333275499	-2.5024050509	-0.7902905645
C	1.4074013142	-1.7252448239	-0.4534089136
O	0.1423782815	1.7194496181	0.1452664118
H	-1.7373338999	0.1784222916	0.5225641407
C	3.0638829173	1.401510892	0.7560703428

C	4.331699288	1.8863701577	0.9194584138
H	4.6615231997	-2.5602877139	-0.9047283303
H	2.398807457	-3.5070776104	-1.1691481323
H	0.4187255521	-2.1487374581	-0.5801304415
C	-0.9609809741	2.4813227073	0.1966294435
C	-2.710001624	4.8071864295	-1.0186331463
H	-1.4508415115	5.8579327897	0.3922129934
H	-2.3425690469	4.515432712	1.1318165428
H	-1.3616068342	5.8373850996	-2.3763134139
H	-2.0361778047	4.3513415976	-3.0617515222
H	-3.3952509547	5.6500244276	-1.084182464
H	-3.2893911194	3.8843362832	-1.0331989376
N	-0.5507683631	4.0622804342	-1.5973016291
C	0.6645998024	4.1571533655	-2.2542194881
C	1.925994843	3.5401477872	-1.6932192279
C	0.87765732	4.7239797212	-3.4510525123
C	2.9396513389	3.8837883225	-2.7306422429
H	1.8089738614	2.4589048316	-1.5502690433
C	2.31363072	4.5912376716	-3.8886212702
H	0.1222783623	5.1981998	-4.0601430879
H	3.9709551922	3.5707376575	-2.6836981186
H	2.781290154	5.5650583247	-4.0864562885
H	2.4122646257	4.0233311078	-4.8246702521
H	2.1834136275	3.9345784227	-0.7005759787
O	-2.057604005	2.0550820391	0.4535290835
C	-1.6794693673	4.8129554065	-2.1394405427
C	-0.6624527674	3.9375696653	-0.14096626
H	0.269129398	4.214153382	0.3552221179
C	-1.830916497	4.8479172234	0.2314748655
C	5.451399424	1.1126929845	0.5499882224
C	5.2681947327	-0.1392332187	0.0384029189
H	2.2195685762	2.0082522335	1.0483500266

H	4.4796082021	2.8730596834	1.3399789587
H	6.4492273502	1.5100426987	0.6823253342
H	6.1177130307	-0.7526485066	-0.2376688677

2-naph

46

O	-1.1314805563	-0.521857339	0.3273618225
B	0.0480847259	0.1230660229	0.2886653401
C	1.4454876649	-0.5758829184	0.3190762318
C	1.6542059046	-1.986181956	0.1545546594
C	2.9764070985	-2.5039242908	0.2320238665
C	4.0594692791	-1.6247519136	0.4729462729
C	3.8505915342	-0.2858645443	0.6262929238
C	2.5419623666	0.2290593173	0.5442933718
O	0.0924367453	1.5389124317	0.2302982013
H	-1.8757222231	0.1019765625	0.3345865177
C	0.5997800191	-2.9021350853	-0.0966936789
C	0.8486534891	-4.2377439332	-0.2545044443
H	5.0592730288	-2.0392847564	0.5315933114
H	4.681100842	0.3830996366	0.8102214152
H	2.3961274966	1.294424703	0.6691772757
C	-0.9740722389	2.3465702322	0.197077308
C	-2.5524057487	4.8589926043	-0.9181338569
H	-1.2823768503	5.7228887939	0.6046353056
H	-2.276870867	4.3884157996	1.2140767638
H	-1.0888945184	5.8909638957	-2.1476531311
H	-1.8398323727	4.5142968682	-2.9699095013
H	-3.179105703	5.7484271974	-0.9408001509

H	-3.1877752205	3.9796434962	-1.0183588146
N	-0.4344494555	4.0045125664	-1.4890800676
C	0.8005756418	4.0440581384	-2.1126877717
C	1.9837458746	3.2607227631	-1.5880741776
C	1.0959586033	4.6868334436	-3.252177489
C	3.0521116303	3.5983706585	-2.5720454918
H	1.7573804949	2.1874970122	-1.5521533308
C	2.5229635733	4.4546653138	-3.6762540843
H	0.4036982225	5.2807813765	-3.8303374557
H	4.0491348814	3.1878322564	-2.538278155
H	3.084529578	5.3921330894	-3.7840084644
H	2.5906413715	3.9581047582	-4.6546430275
H	2.2466562732	3.5377753497	-0.557907547
O	-2.1108847774	1.9631225933	0.3166764465
C	-1.48581602	4.8766918755	-2.0033906455
C	-0.5882335358	3.8009366899	-0.0440748824
H	0.3428020777	3.9989232219	0.4883240675
C	-1.7152664244	4.7525575242	0.3566554334
C	2.1617517686	-4.7441466712	-0.1719685808
C	3.2007706334	-3.8918176451	0.0656464238
H	-0.4120981699	-2.5329477057	-0.1599453923
H	0.0268200442	-4.9158922519	-0.445589062
H	2.3407717384	-5.8039340183	-0.2986479168
H	4.2169920802	-4.2624911632	0.1295341615

2-F_far

40

O	-1.3224911703	-0.6382614838	-0.1369084431

B	-0.1406952719	-0.0169402667	-0.0379042599
C	1.2267009302	-0.7684516997	-0.0055394077
C	2.4097572516	-0.0753733064	0.280108803
C	3.6389955466	-0.7113352268	0.3272494645
C	3.7141318482	-2.0765981032	0.0810996613
C	2.566651228	-2.7979833722	-0.2069129529
C	1.3543421262	-2.1334471083	-0.2420586918
O	-0.0343364649	1.3908201759	0.0504547159
H	-2.0603047149	-0.0094054875	-0.1136428985
H	2.349827309	0.9883999285	0.4724114688
H	4.5346230436	-0.1488141224	0.5534414901
H	4.6695175953	-2.5842354322	0.1127373928
H	2.5911434846	-3.8607431684	-0.4048646114
F	0.2672787504	-2.8572130123	-0.5262512356
C	-1.0716702472	2.2409234315	0.0373943099
C	-2.5081924005	4.9089115648	-0.8911250399
H	-1.281812362	5.5702686087	0.7631387593
H	-2.3490763552	4.2223010933	1.1937087927
H	-0.9540134784	5.99854283	-1.9472325922
H	-1.7125209709	4.7392865082	-2.9342953733
H	-3.1023471672	5.8198456789	-0.8523556012
H	-3.1676991224	4.068716987	-1.1056972428
N	-0.3968646006	4.0321869221	-1.4523412938
C	0.8663911894	4.0837166307	-2.0152149324
C	1.9925404514	3.2002514776	-1.5254494272
C	1.2393503702	4.8256073535	-3.0684173081
C	3.1183544616	3.5905978192	-2.4218735544
H	1.7218827089	2.1402061931	-1.613635862
C	2.6746947878	4.5766049517	-3.4530296437
H	0.5983187385	5.5043991915	-3.6111274381
H	4.097824676	3.1400323128	-2.3851842026
H	3.276275646	5.4950872053	-3.4390902944

H	2.7689142113	4.179642449	-4.4737899199
H	2.2168753033	3.357168413	-0.4614673788
O	-2.2234770633	1.8902980036	0.0588277727
C	-1.3919988358	4.9912893129	-1.9221221415
C	-0.6242332219	3.6953512087	-0.042145567
H	0.2870859237	3.8049238956	0.5467810278
C	-1.7357074044	4.6462890228	0.4017236552

2-F

40

O	-1.1569154612	-0.604969105	-0.05477478
B	-0.0004066374	0.072551561	0.05237657
C	1.3452446114	-0.717238709	0.07388389
C	1.3327614329	-2.071337887	-0.28418933
C	2.4897251533	-2.831116936	-0.30946147
C	3.7023701015	-2.247171844	0.03647228
C	3.7541893956	-0.913072083	0.40737088
C	2.5806606686	-0.182315089	0.41769540
O	0.0476332224	1.474984680	0.10924281
H	-1.9217206164	-0.008418051	-0.05343868
H	0.3830451548	-2.519713400	-0.54783383
H	2.4501196322	-3.873495819	-0.59501755
H	4.612858831	-2.832152490	0.02167801
H	4.6796873112	-0.431078344	0.69087067
F	2.6644860724	1.102207991	0.79970770
C	-1.0247365605	2.280506555	0.06872465
C	-2.5636088341	4.860974385	-0.92953679
H	-1.35816899	5.619626866	0.69863651

H	-2.3728703705	4.247071505	1.17537313
H	-1.0568591253	5.972717112	-2.03173422
H	-1.7692063979	4.650763123	-2.96931985
H	-3.190582587	5.750473643	-0.91775869
H	-3.1928634434	3.990910033	-1.11383866
N	-0.4197677021	4.049985988	-1.47004997
C	0.8403526914	4.134868076	-2.03704966
C	2.0078234033	3.331074849	-1.50901647
C	1.1758837269	4.848821629	-3.12188222
C	3.1116920783	3.734327146	-2.42671620
H	1.79095371	2.255978680	-1.54386996
C	2.6223126865	4.655329488	-3.49690773
H	0.5024356391	5.472135135	-3.69139142
H	4.1173086752	3.349765377	-2.36040120
H	3.1780668157	5.602277671	-3.51938264
H	2.7376367903	4.224006505	-4.50162177
H	2.2261987907	3.540302130	-0.45436837
O	-2.1612766852	1.881953808	0.09551032
C	-1.4546313653	4.950567059	-1.96826179
C	-0.6325236261	3.747247364	-0.05038324
H	0.2757849371	3.903326305	0.53217305
C	-1.7780931296	4.668525092	0.36789059

2-Me_far

43

O	-1.3601312355	-0.57653916	-0.021307738
B	-0.1559624791	0.01660454	0.061768158
C	1.1932599087	-0.76346301	0.099280778

C	2.3630925319	-0.04074648	0.359222224
C	3.6042852166	-0.65373971	0.418504785
C	3.6906912101	-2.02100324	0.209093194
C	2.5427944743	-2.75572651	-0.050903820
C	1.2888020717	-2.15412884	-0.107361350
O	-0.0417581292	1.42870897	0.119947378
H	-2.0781339793	0.07645789	-0.002390672
H	2.2917720833	1.02742524	0.522059752
H	4.493064314	-0.07166907	0.624055270
H	4.6512432658	-2.51945814	0.247434534
H	2.6198539213	-3.82402128	-0.214496289
C	0.0841807543	-3.01167581	-0.389338005
C	-1.0677433368	2.28764420	0.091901178
C	-2.49207715	4.95538047	-0.865869178
H	-1.2275351904	5.63374397	0.752473114
H	-2.3027376834	4.30781143	1.228816610
H	-0.9447019407	6.00184523	-1.975242002
H	-1.7383334816	4.73169598	-2.919531786
H	-3.0731682167	5.87503973	-0.834759002
H	-3.166523434	4.11999646	-1.050551963
N	-0.4034800576	4.03961076	-1.447972134
C	0.8480886546	4.05880893	-2.038018373
C	1.9727194973	3.17371193	-1.547975418
C	1.2084247629	4.76870304	-3.117444424
C	3.0839491143	3.52433059	-2.478340916
H	1.6879680487	2.11500814	-1.599439799
C	2.6322481747	4.49071523	-3.524593054
H	0.5651094131	5.44249168	-3.663748967
H	4.0572466279	3.06001148	-2.451007576
H	3.2458979003	5.40113758	-3.545529431
H	2.7005795037	4.06771348	-4.536940908
H	2.2220019416	3.35699819	-0.493820552

O	-2.2252754171	1.95252672	0.128416049
C	-1.3948727224	5.00114924	-1.919371007
C	-0.6068377134	3.73531413	-0.026897999
H	0.3169823398	3.84704571	0.541689534
C	-1.6988405596	4.70847575	0.417601825
H	-0.4615132275	-2.65497085	-1.262603743
H	-0.618442231	-2.98849736	0.443541964
H	0.3866135843	-4.04325189	-0.563302814

2-Me

43

O	-0.2298148816	-0.5058939133	-1.6878603948
B	0.5681169935	-0.0578026773	-0.6996248761
C	1.368969529	-1.0706998891	0.1764673154
C	1.1517561867	-2.42333214	-0.1104136447
C	1.7920835196	-3.4322902406	0.5898137206
C	2.675222248	-3.0934088228	1.6028455255
C	2.9059682155	-1.7585371632	1.9014488636
C	2.2683447521	-0.7337692206	1.2069238429
O	0.6914071811	1.3232259765	-0.4328280582
H	-0.6714290457	0.2189370324	-2.1559069865
H	0.4611811953	-2.6769320307	-0.905086434
H	1.6052049873	-4.4702275215	0.3476428869
H	3.187612819	-3.8659213647	2.1625517511
H	3.5996253137	-1.5030803258	2.6936718621
C	2.5792384757	0.6903280229	1.5928221969
C	0.0478937452	2.3014936692	-1.0913528586
C	-1.8405745437	4.4373148045	0.0314066023

H	-0.101900191	4.7046936156	1.3118915489
H	-0.7077141367	3.0428720519	1.3148029823
H	-1.3818885451	6.2753807758	-1.0951401795
H	-1.9462900691	4.930291093	-2.0971323147
H	-2.4289182324	5.0671072929	0.6955248526
H	-2.4625986193	3.594016634	-0.2683861113
N	0.0272644522	4.7520715755	-1.3881247885
C	0.6653875047	4.9439333304	-2.5921019208
C	2.0309599959	4.3420792253	-2.8335892596
C	0.2453341491	5.6748247547	-3.6329374496
C	2.3567997762	4.8359115595	-4.20281018
H	2.0086202309	3.2435922557	-2.7721091875
C	1.2577664986	5.6895398884	-4.7494083379
H	-0.6947764724	6.2045042691	-3.6812131348
H	3.2642705322	4.58980732	-4.7312975799
H	1.603857309	6.7046446788	-4.9886963902
H	0.8498372499	5.2893852284	-5.6874933159
H	2.7522975526	4.6718234617	-2.0724447691
O	-0.7083061216	2.1215695028	-2.0099823821
C	-1.3515312458	5.1886676452	-1.2152911774
C	0.3623188889	3.6692215877	-0.5045255334
H	1.4164584265	3.6761093304	-0.2216202787
C	-0.5576043101	3.9279712455	0.6981807301
H	1.684823237	1.2183021084	1.9245273449
H	2.9837760035	1.2514487449	0.7501003927
H	3.3094494456	0.7100266286	2.4004551249

2-CF3-close

43

O	-1.0906328574	-0.559088609	-0.01224521
B	0.0330274726	0.163664955	0.10620972
C	1.4258705465	-0.557623893	0.17403693
C	2.4806537494	-0.199988506	1.02103754
C	3.69842879	-0.861656986	0.97515333
C	3.8888062984	-1.897378498	0.07147837
C	2.8531659431	-2.282123917	-0.76435834
C	1.6336058264	-1.622265917	-0.70288203
O	0.0483290761	1.565094720	0.07508010
H	-1.8764783248	0.008577624	-0.05484115
F	2.4842340042	2.120706149	1.51428866
H	4.4912276208	-0.577827691	1.65334147
H	4.840962119	-2.409787524	0.03444826
H	2.9910359429	-3.099837402	-1.45966953
H	0.8210282768	-1.934719963	-1.34715572
C	-1.0444159231	2.346010090	0.03390649
C	-2.6200717532	4.805899620	-1.10549346
H	-1.4883564682	5.704455993	0.50833188
H	-2.4745251525	4.329560514	1.03773376
H	-1.1517350832	5.880821355	-2.30741002
H	-1.769543905	4.422447864	-3.09394187
H	-3.267777544	5.677944147	-1.17092574
H	-3.2265587744	3.911512866	-1.24230579
N	-0.4097541925	4.087464171	-1.51668794
C	0.8584668478	4.192907148	-2.05290133
C	2.0777111937	3.652364293	-1.33960772
C	1.1730070611	4.741457932	-3.23629159
C	3.1729246985	3.967193086	-2.30178945
H	1.9797382453	2.581962602	-1.12403019
C	2.6499403741	4.654136164	-3.52145037
H	0.4668512785	5.177485478	-3.92756470

H	4.2102206216	3.727296229	-2.12884479
H	3.1063178583	5.642528133	-3.66990041
H	2.8653494366	4.091512973	-4.44065450
H	2.2252194112	4.131375831	-0.36183370
O	-2.1682183908	1.915359799	0.06832809
C	-1.4908733292	4.851831705	-2.12947031
C	-0.6820824288	3.819425729	-0.10586236
H	0.1945553543	4.008805521	0.51462314
C	-1.868240009	4.722011621	0.22422709
C	2.3045691947	0.899252465	2.03774545
F	1.0760937447	0.884770176	2.57869412
F	3.1723735694	0.789386984	3.04942354

2-CF3

43

O	-1.1837246508	-0.63787938	0.1085500742
B	-0.0462360144	0.05318189	-0.0162785560
C	1.3692384912	-0.62467923	-0.1022735460
C	1.6512915604	-1.85602729	-0.7092790704
C	2.9287228346	-2.39375247	-0.6815462055
C	3.9569791104	-1.71637062	-0.0403275008
C	3.7074997624	-0.49245711	0.5560268230
C	2.4284011986	0.04471747	0.5136313978
O	-0.0073394105	1.46189200	0.0001305987
H	-1.9576332119	-0.05721387	0.1744347242
F	-0.2662130233	-3.23724060	-0.5995908788
H	3.1234827528	-3.33801870	-1.1697750804
H	4.9508014398	-2.14368905	-0.0197718651

H	4.5055529414	0.04725016	1.0489717600
H	2.2423920006	1.00816910	0.9722181341
C	-1.0830083883	2.26100752	0.0888174188
C	-2.5854842461	4.75680042	-1.0966236340
H	-1.5256388938	5.61404330	0.5828141629
H	-2.5403183858	4.23307497	1.0413220569
H	-1.0387134503	5.88774303	-2.1194694298
H	-1.6241428594	4.50630987	-3.0592402700
H	-3.243902026	5.62015095	-1.1682534537
H	-3.1682334576	3.85734794	-1.2957522263
N	-0.3747248048	4.01446089	-1.4361285489
C	0.9264980248	4.15326238	-1.8883037541
C	2.0857578169	3.43830626	-1.2282796784
C	1.3216324499	4.85181513	-2.9630006067
C	3.246300008	3.88340332	-2.0523893660
H	1.9363348568	2.35191206	-1.2490716115
C	2.8050501007	4.73477630	-3.1980136427
H	0.6697783784	5.41348230	-3.6155403060
H	4.2583777167	3.54504740	-1.8942121972
H	3.30490444	5.71243794	-3.2091637496
H	3.0371547732	4.27553978	-4.1694219125
H	2.1912352148	3.70049993	-0.1664166569
O	-2.2062506159	1.85233418	0.2285776117
C	-1.4013663386	4.85339629	-2.0489430494
C	-0.7070373197	3.73282229	-0.0368421587
H	0.1447346998	3.91683503	0.6199331918
C	-1.8980322303	4.63946495	0.2636322245
C	0.5723976459	-2.62340569	-1.4366392013
F	-0.1551314173	-1.82570168	-2.2281280964
F	1.0904125266	-3.57534938	-2.2264839256

2,6-F

40

O	-1.1460690939	-0.51554237	0.54934295
B	-0.0191305731	0.15698736	0.28833742
C	1.3493985632	-0.58583556	0.09768152
C	1.4413011504	-1.81955814	-0.53987589
C	2.6293257402	-2.49649530	-0.73906161
C	3.8010262693	-1.92278203	-0.27098849
C	3.7790452873	-0.69794061	0.37900341
C	2.5617889277	-0.06682374	0.54153236
O	0.0255031939	1.55258069	0.15819004
H	-1.9062270072	0.08105736	0.63545898
F	0.3227621864	-2.37718748	-1.01223467
H	2.6186676749	-3.44726732	-1.25230487
H	4.742547787	-2.43624052	-0.41284194
H	4.675650724	-0.23201499	0.76176025
F	2.5538207552	1.10661941	1.19198699
C	-1.0321348156	2.37133886	0.26826023
C	-2.5784813546	4.74584930	-1.08855534
H	-1.4275357851	5.76279167	0.43483885
H	-2.4244729293	4.44858705	1.08765605
H	-1.0833958819	5.74955172	-2.30360964
H	-1.721642641	4.28247103	-3.06103781
H	-3.2374176111	5.60227837	-1.21664449
H	-3.1740748455	3.83555886	-1.16202536
N	-0.3909583782	3.95321117	-1.46107307
C	0.8861852302	4.03240158	-1.98979163
C	2.0771850686	3.38980852	-1.31222822
C	1.2264005989	4.60715979	-3.15328445

C	3.1940787155	3.73481965	-2.23795408
H	1.9269453058	2.30902404	-1.20261326
C	2.6969575474	4.45990395	-3.44596789
H	0.5423774201	5.09771260	-3.82989055
H	4.2171573233	3.42983299	-2.08313882
H	3.1941986996	5.42908746	-3.58556990
H	2.8826674989	3.89910572	-4.37320321
H	2.2363013727	3.76226830	-0.29185027
O	-2.1408215998	1.99351283	0.54669629
C	-1.4450565615	4.73151605	-2.10555089
C	-0.6543875021	3.81799422	-0.02588238
H	0.231450977	4.05323180	0.56632951
C	-1.8217374376	4.76442571	0.23950386

2,4-Me_close

46

O	-0.8912309799	-0.54530134	-0.36376314
B	0.2281011833	0.15612646	-0.10368866
C	1.5978218166	-0.57336003	0.02341265
C	2.7603389895	-0.03837411	0.61164985
C	3.9186879337	-0.80560484	0.62996197
C	3.9773572032	-2.08574712	0.08403396
C	2.8257702669	-2.61029448	-0.48877855
C	1.6585074355	-1.86465507	-0.50800541
O	0.199882297	1.56668579	0.01099096
H	-1.673382464	0.02498869	-0.41144182
C	2.7935545442	1.32949612	1.24448331
H	4.8117543593	-0.39373022	1.08907653

C	5.2514648592	-2.88033971	0.14595219
H	2.8430343123	-3.60480583	-0.91839155
H	0.7633775878	-2.28570031	-0.94949618
C	-0.898392356	2.32729634	-0.10150959
C	-2.6250901456	4.89561429	-0.78415330
H	-1.295523585	5.60635764	0.77095159
H	-2.2116141737	4.16820063	1.25019033
H	-1.3072389643	6.10378346	-2.03523665
H	-2.0436330438	4.74471181	-2.89551889
H	-3.2630973633	5.76892288	-0.66239213
H	-3.2539317428	4.02174394	-0.94553051
N	-0.5027791956	4.22728172	-1.55734723
C	0.670392771	4.33958346	-2.27272642
C	1.9445790433	3.66124368	-1.82157453
C	0.8391907328	5.00314594	-3.42654454
C	2.9012765394	4.02734699	-2.90559688
H	1.8077689593	2.57787065	-1.70863826
C	2.2463783647	4.87266816	-3.94931575
H	0.0674814055	5.55033111	-3.94761608
H	3.9304102591	3.70547638	-2.93125937
H	2.7417275194	5.84636348	-4.06581980
H	2.2813262747	4.40540553	-4.94327392
H	2.2676166814	4.02427930	-0.83530380
O	-2.0167138398	1.88557618	-0.17728255
C	-1.6371585286	5.06112455	-1.93278807
C	-0.571336333	3.81643419	-0.15780334
H	0.3779718362	3.97615261	0.35451688
C	-1.7100364751	4.66038163	0.41929526
H	1.9845344891	1.45470218	1.96460941
H	2.6775513656	2.11364472	0.49627170
H	3.7393006604	1.48518051	1.76155127
H	6.1130827805	-2.26088136	-0.10478284

H	5.2229748014	-3.72587746	-0.53987845
H	5.4113140583	-3.27090179	1.15333303

2,4-Me

46

O	-1.1383010125	-0.59988059	-0.0538216965
B	0.0352047707	0.05266290	0.0337270347
C	1.4200361828	-0.65523141	0.0766387025
C	1.5901163513	-2.04138461	-0.0969360555
C	2.8748226638	-2.57530279	-0.0379907354
C	4.0002121371	-1.79224794	0.1913075783
C	3.8257361684	-0.42251659	0.3666873443
C	2.5586250565	0.12710193	0.3072405377
O	0.0760509469	1.47035845	0.0910199044
H	-1.8875185259	0.01708323	-0.0406348506
C	0.4318345724	-2.96986587	-0.3466752999
H	3.0017118924	-3.64371280	-0.1763626914
C	5.3729926321	-2.40071508	0.2514026726
H	4.6866317488	0.21047411	0.5487947649
H	2.4397292478	1.19440514	0.4468663029
C	-0.9913478716	2.27557921	0.059237325
C	-2.555179296	4.88599005	-0.8529054837
H	-1.3088735555	5.60076157	0.7637872983
H	-2.314109885	4.21767396	1.2298197917
H	-1.0688250741	6.01935940	-1.9606437086
H	-1.8128143663	4.72795497	-2.9167477613
H	-3.1783728391	5.77681243	-0.802649026
H	-3.1911729759	4.02228319	-1.0434234929

N	-0.4334058685	4.07620469	-1.4685521221
C	0.8079083637	4.15548023	-2.074211058
C	1.9747627519	3.31243004	-1.6095891104
C	1.1240950528	4.89339969	-3.1487311861
C	3.0574317931	3.71895300	-2.5508687791
H	1.7337162684	2.24324003	-1.6685758318
C	2.5525393102	4.67987455	-3.5778271044
H	0.4461076654	5.54697937	-3.6773751229
H	4.0493660925	3.29507277	-2.542129677
H	3.1281203187	5.61496733	-3.5937505346
H	2.6249064858	4.27375081	-4.5967757592
H	2.2306125993	3.49503705	-0.5569185143
O	-2.1312414906	1.88310226	0.0861766468
C	-1.4720569115	4.99817307	-1.9158429564
C	-0.6044635832	3.74572503	-0.0490980084
H	0.319125868	3.89785578	0.5104468324
C	-1.7381240856	4.65869769	0.4192321526
H	-0.1324592125	-2.67018837	-1.2295844665
H	-0.2703678319	-2.95462872	0.4867668635
H	0.7864283631	-3.98982884	-0.4875745272
H	5.3383793866	-3.47587688	0.0828097139
H	5.8325564948	-2.22260340	1.2252294479
H	6.0261732003	-1.95746004	-0.5021953543

4-F

40

O	-1.1380674013	-0.63379205	-0.143670983
B	0.0135033529	0.04822417	-0.018938312

C	1.3806126307	-0.68594132	0.037062461
C	1.4309033451	-2.07553579	-0.107276676
C	2.6342671342	-2.75981263	-0.065896579
C	3.7942940294	-2.03176621	0.125902639
C	3.7946092458	-0.65808324	0.277372109
C	2.5788284726	0.00584125	0.230177475
O	0.0550551697	1.45795422	0.058573367
H	-1.9084964818	-0.04539806	-0.145730365
H	0.5082397082	-2.62314425	-0.255004400
H	2.6917329725	-3.83384515	-0.177665251
F	4.9634500713	-2.68375674	0.167837603
H	4.7309621363	-0.13836192	0.427156003
H	2.558223234	1.08226314	0.349071527
C	-1.0202581297	2.25850408	0.010435195
C	-2.5715142758	4.86581410	-0.924562346
H	-1.3881378224	5.57155898	0.742361146
H	-2.3972752758	4.17542580	1.157909113
H	-1.0554286803	6.02609557	-1.960358028
H	-1.7543972282	4.74462940	-2.962934435
H	-3.2047818187	5.75000196	-0.885526013
H	-3.1920136666	3.99877579	-1.148428551
N	-0.4233878217	4.07932481	-1.476167001
C	0.8396116484	4.18113844	-2.033090589
C	1.9931493147	3.33118820	-1.547491835
C	1.1909505097	4.94550501	-3.077418626
C	3.1085660368	3.76870055	-2.435299536
H	1.7578811234	2.26359937	-1.648739743
C	2.6353501805	4.74946168	-3.458397507
H	0.5293874787	5.60782978	-3.615820578
H	4.1034182345	3.35319163	-2.398038350
H	3.2049544509	5.68782336	-3.432581269
H	2.7471451579	4.36642809	-4.482670372

H	2.2070313808	3.48359932	-0.480754137
O	-2.1543178141	1.85315767	0.002720226
C	-1.4522103458	5.00160426	-1.946392137
C	-0.6408871009	3.73192679	-0.066873335
H	0.2617116944	3.88376932	0.525958211
C	-1.7975648504	4.63030063	0.372589880

4-Me

43

O	-1.1407399365	-0.6318561589	-0.14350967
B	0.0150714008	0.0453117962	-0.02605492
C	1.38241349	-0.6875937246	0.01689345
C	1.4388757441	-2.0732439100	-0.13934272
C	2.6509284321	-2.7461449888	-0.10968576
C	3.8449239977	-2.0562716540	0.08027396
C	3.7935011126	-0.6719409744	0.24140736
C	2.5841487162	0.0004924223	0.20922861
O	0.0561378593	1.4569942058	0.05438753
H	-1.908255157	-0.0399482449	-0.13854534
H	0.5180509453	-2.6247973891	-0.28691307
H	2.6742826327	-3.8222599210	-0.23540931
C	5.1645850411	-2.7748870588	0.11426021
H	4.7152459549	-0.1213643855	0.39286474
H	2.5657396517	1.0761812624	0.33882779
C	-1.0166578463	2.2583888958	0.01527812
C	-2.5668719019	4.8716729365	-0.90778771
H	-1.3734145421	5.5718044669	0.75429993
H	-2.3836216444	4.1769573611	1.17227989

H	-1.0539993994	6.0303352597	-1.95063943
H	-1.760877029	4.7512152687	-2.95057243
H	-3.198050892	5.7571902764	-0.86409849
H	-3.1902778599	4.0062295652	-1.12974627
N	-0.4235188701	4.0819015032	-1.47152313
C	0.8357804334	4.1787645821	-2.03663846
C	1.9899774464	3.3265041704	-1.55692784
C	1.1826242829	4.9411345487	-3.08420635
C	3.1009689916	3.7600541889	-2.45207830
H	1.7523867242	2.2592190025	-1.65415861
C	2.6241016577	4.7403740537	-3.47400403
H	0.5194426883	5.6046093424	-3.61922945
H	4.09371303	3.3388868329	-2.42256705
H	3.1966516653	5.6771622936	-3.45362149
H	2.7283509316	4.3551163355	-4.49833029
H	2.2107838432	3.4795284209	-0.49178654
O	-2.1528730091	1.8574914856	0.01486168
C	-1.4526754645	5.0065143109	-1.93534755
C	-0.6346762405	3.7315233446	-0.06193873
H	0.2714677032	3.8803581959	0.52623168
C	-1.7867619695	4.6320541545	0.38493473
H	5.0360374868	-3.8464477127	-0.02999043
H	5.667221486	-2.6172867911	1.07011468
H	5.8287584132	-2.4027275695	-0.66769097

4-OMe

44

O	-1.141639729	-0.633277204	-0.144058908

B	0.0142725596	0.044227506	-0.016304115
C	1.3787741946	-0.683859680	0.038334840
C	1.4401153922	-2.068267715	-0.108904346
C	2.6446909072	-2.756622364	-0.071281108
C	3.8277441015	-2.046579617	0.122057363
C	3.791675285	-0.659157491	0.276666443
C	2.5840461458	0.004228297	0.233323529
O	0.0521206861	1.457254649	0.064402676
H	-1.908524467	-0.040732924	-0.145109998
H	0.5205808712	-2.621633673	-0.258081919
H	2.6519066092	-3.830010593	-0.191490324
O	5.0541344843	-2.614147450	0.175276239
H	4.7256858985	-0.134625875	0.428046929
H	2.5668783966	1.080545141	0.356725419
C	-1.0203226716	2.257236544	0.014320283
C	-2.5675042055	4.870312472	-0.919489224
H	-1.3829253389	5.571001643	0.748674862
H	-2.394700328	4.175758155	1.161480704
H	-1.0490709189	6.028696650	-1.954529753
H	-1.7514890033	4.750028514	-2.958198432
H	-3.1990094937	5.755783895	-0.879142272
H	-3.1895998414	4.004715655	-1.144472820
N	-0.4221691092	4.079713609	-1.472613885
C	0.8391758664	4.173568166	-2.033425886
C	1.9897205038	3.319027276	-1.549258407
C	1.1913534328	4.934555022	-3.080273327
C	3.1046997782	3.749036267	-2.441103737
H	1.7496015197	2.252304740	-1.646470631
C	2.6336991179	4.730108705	-3.465028073
H	0.5314389155	5.599341307	-3.617722241
H	4.096270435	3.325400643	-2.408005366
H	3.2085691689	5.665449037	-3.442962404

H	2.7406017574	4.344259247	-4.488866278
H	2.207231147	3.472542199	-0.483510316
O	-2.1567009085	1.856185335	0.005310944
C	-1.4482760224	5.005016921	-1.941373528
C	-0.639165877	3.730706225	-0.063457647
H	0.2642302706	3.881415394	0.528500386
C	-1.7939132849	4.631026779	0.377229983
C	5.1439829332	-4.013601705	0.022841133
H	6.1999199342	-4.259024272	0.094983194
H	4.7625150617	-4.329835094	-0.951415578
H	4.5948758251	-4.532870336	0.812475598

4-tBu

52

O	-1.1241571627	-0.619915867	-0.089646844
B	0.0347534805	0.056727350	-0.004748503
C	1.4016890868	-0.677801979	0.016852883
C	1.4559771102	-2.063243211	-0.122112508
C	2.6669926939	-2.741951263	-0.110445866
C	3.8723090006	-2.059883316	0.044013431
C	3.8166966828	-0.670263961	0.187680427
C	2.6105604338	0.006342723	0.173618003
O	0.0793127937	1.469146081	0.059707082
H	-1.8905083276	-0.026661976	-0.075138888
H	0.532617517	-2.617375289	-0.242418663
H	2.6603573972	-3.816952894	-0.223605480
C	5.2266974228	-2.765408953	0.062412243
H	4.7332463599	-0.106606380	0.312718324

H	2.6015420364	1.083710423	0.290024663
C	-0.9933138466	2.271368314	0.036346449
C	-2.5607907473	4.880344614	-0.872556077
H	-1.3332606918	5.589258833	0.760958348
H	-2.335533357	4.197535480	1.207361287
H	-1.067737551	6.031557705	-1.951014865
H	-1.7966881788	4.748961750	-2.930642959
H	-3.1905509471	5.766451384	-0.821143059
H	-3.1889027451	4.013912385	-1.076743859
N	-0.4309737758	4.084105902	-1.475880379
C	0.816145354	4.175072458	-2.068304165
C	1.9779125957	3.321415695	-1.609980226
C	1.1421800845	4.931574090	-3.126704966
C	3.0702852147	3.747413165	-2.531282444
H	1.7346720582	2.254568229	-1.697277893
C	2.5742629786	4.724968100	-3.546799274
H	0.4692043358	5.594450534	-3.650123803
H	4.0629388547	3.325181596	-2.520192754
H	3.149842842	5.660118895	-3.542577195
H	2.6553274878	4.335373933	-4.571566323
H	2.2223058537	3.478343203	-0.550602458
O	-2.1296908568	1.871695965	0.062043049
C	-1.4676680197	5.008463106	-1.923054246
C	-0.6123301927	3.743654400	-0.059616832
H	0.3059186684	3.896627533	0.508418311
C	-1.7546041104	4.647719562	0.405423066
C	5.0944845165	-4.278722653	-0.106876163
C	6.0943625435	-2.225516297	-1.083419383
C	5.9240387928	-2.488661457	1.401915907
H	4.626953595	-4.537026611	-1.058670815
H	4.5077863864	-4.723339081	0.698935264
H	6.0862973742	-4.733118391	-0.087808665

H	5.6165368487	-2.407197521	-2.047625853
H	7.066736233	-2.722643974	-1.081678012
H	6.2642821898	-1.152842029	-0.987423443
H	5.3224762791	-2.859213349	2.233694617
H	6.091508576	-1.422236943	1.554750368
H	6.8940988319	-2.989580016	1.428209141

4-CF3

43

O	-1.1160134373	-0.63281182	-0.12278478
B	0.0313057557	0.05634581	-0.01924743
C	1.4052540205	-0.68067369	0.02039566
C	1.4506944616	-2.06715606	-0.12332015
C	2.6586243548	-2.74895677	-0.09520195
C	3.8345317904	-2.03661545	0.08105717
C	3.8150286485	-0.65508291	0.23033851
C	2.6045640194	0.01456368	0.19857132
O	0.0748941136	1.46266515	0.05099646
H	-1.8907156042	-0.04958643	-0.11707932
H	0.5261270428	-2.61416830	-0.25947915
H	2.6892356501	-3.82357303	-0.20952873
C	5.1638797523	-2.73736896	0.12684625
H	4.7433125448	-0.11513105	0.36864209
H	2.5843177805	1.09092679	0.31576284
C	-1.0051183967	2.26198324	0.01738891
C	-2.5740634563	4.85959697	-0.91076893
H	-1.3803760198	5.57370816	0.74536280
H	-2.3800021276	4.17430827	1.17196052

H	-1.0697623669	6.02494533	-1.95605616
H	-1.7719380661	4.74241215	-2.95537841
H	-3.2112053749	5.74078241	-0.86816433
H	-3.192429382	3.98948972	-1.12868621
N	-0.4278520299	4.07936700	-1.47926971
C	0.83172455	4.18986303	-2.04407278
C	1.9917243184	3.34170408	-1.57114109
C	1.172487796	4.96012726	-3.08724071
C	3.0993414267	3.78828008	-2.46418310
H	1.7589555896	2.27409734	-1.67817711
C	2.6150134754	4.77213024	-3.47901214
H	0.5043369594	5.62189042	-3.61814417
H	4.0972808987	3.37984043	-2.43329159
H	3.1806964533	5.71273823	-3.45213411
H	2.7212879214	4.39505088	-4.50599851
H	2.2125951879	3.48808671	-0.50498316
O	-2.1361557203	1.85024938	0.02633701
C	-1.4632641599	4.99929699	-1.94086745
C	-0.6317335514	3.73606783	-0.06676845
H	0.2750804718	3.89319247	0.51824913
C	-1.788868154	4.63011686	0.38071321
F	5.0587475198	-4.04975460	-0.09531957
F	6.010627499	-2.24589110	-0.78803637
F	5.7586278449	-2.58365670	1.31831368

phenyl

40

O -1.1412178429 -0.634865011 -0.137182024

B	0.0127265257	0.044326753	-0.020378525
C	1.3804550299	-0.692568337	0.037309569
C	1.4259953194	-2.082360114	-0.101347174
C	2.6332942034	-2.761883734	-0.055931319
C	3.8164059639	-2.058083378	0.132441071
C	3.7901410686	-0.677169127	0.275821114
C	2.5794431539	-0.001763064	0.227422398
O	0.0548685833	1.455315907	0.048213725
H	-1.9095006965	-0.043764442	-0.142273398
H	0.5006178788	-2.626598071	-0.246443454
H	2.6556659855	-3.838416011	-0.166191348
H	4.7603363332	-2.587257531	0.168673867
H	4.7123690193	-0.130413739	0.424824932
H	2.5594346521	1.075464982	0.342089510
C	-1.0193390769	2.256089245	0.003872371
C	-2.5710755415	4.864785108	-0.924157242
H	-1.386606102	5.567867880	0.743231351
H	-2.3952108497	4.170739288	1.156830302
H	-1.0570982536	6.028311277	-1.959684118
H	-1.7553491492	4.747249724	-2.963228164
H	-3.2050417288	5.748412700	-0.883160913
H	-3.1909996827	3.997613165	-1.149084291
N	-0.4222063118	4.081939709	-1.478177161
C	0.8404504978	4.185880983	-2.034863498
C	1.9953442409	3.338404531	-1.548529628
C	1.190480999	4.951012136	-3.079195198
C	3.1103198945	3.778866143	-2.435295369
H	1.7627938478	2.270367977	-1.649790766
C	2.6354517824	4.757624281	-3.459637572
H	0.5273412859	5.611444072	-3.618035576
H	4.1062712215	3.366192821	-2.396608629
H	3.2034761747	5.697016794	-3.434968119

H	2.7481703222	4.373513115	-4.483489073
H	2.2084344437	3.490387774	-0.481723696
O	-2.1544488365	1.852688325	-0.002574507
C	-1.4526395629	5.003310951	-1.946568424
C	-0.6396817672	3.729844737	-0.070052031
H	0.2629067098	3.880102402	0.523158445
C	-1.7960797352	4.627169778	0.371982565

boronic acid + aldehyde (H bond) - xyz structures

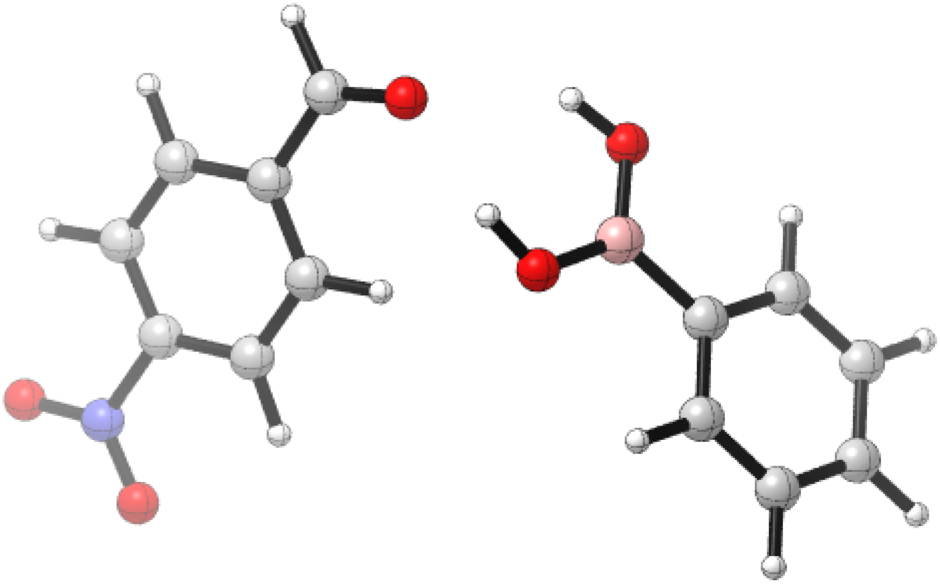

3-F_close

32

O	1.4602948829	2.50634491	-0.02675712
B	0.3021026369	2.00130765	-0.54419544
C	-0.124929057	0.54939299	-0.14355438
C	0.6469342848	-0.20814520	0.73905198
C	0.2644836887	-1.49392317	1.09393920
C	-0.8972612429	-2.04732061	0.57265592
C	-1.6524411315	-1.28801215	-0.30053255

C	-1.2920835839	-0.00810086	-0.66744729
O	-0.5083824359	2.70894354	-1.38980185
H	1.6567993833	3.40856084	-0.30140921
H	1.5538981288	0.22064117	1.14587067
H	0.8695917764	-2.07380017	1.77872548
H	-1.2228219937	-3.04659173	0.82858743
F	-2.777693093	-1.81999613	-0.80807631
H	-1.9148095484	0.54695666	-1.35743409
H	-0.1678597201	3.59287491	-1.57714362
O	0.7222274913	5.28296132	-0.94202558
C	0.3418937055	6.33591633	-0.49062487
H	1.0583072613	7.16095229	-0.33535880
C	-1.055940472	6.62936388	-0.11263216
C	-1.375097281	7.92096286	0.29592713
C	-2.0322420527	5.63393457	-0.15715080
C	-2.6750752435	8.23681815	0.65432524
H	-0.6040888304	8.68124732	0.33119472
C	-3.3324849142	5.93563013	0.20301555
H	-1.7694996838	4.62873552	-0.46218096
C	-3.6220310608	7.23200779	0.59743153
H	-2.9609308056	9.22807938	0.97276918
H	-4.1164171832	5.19303529	0.18569017
N	-5.0158710034	7.55854299	0.98204098
O	-5.8283745565	6.66789007	0.92420387
O	-5.2425983463	8.69398945	1.32659599

3-F_far

32

O	0.490500599	2.701161348	-1.47198809
B	-0.3095729935	2.008165817	-0.60474674
C	0.1339451813	0.573311538	-0.16283892
C	1.3030528838	-0.000151342	-0.66586124
C	1.6975442693	-1.271416153	-0.27392157
C	0.9301010656	-1.993757341	0.62971248
C	-0.225318336	-1.415808044	1.12030383
C	-0.6374743326	-0.154112429	0.74453680
O	-1.4724752224	2.513769756	-0.09833264
H	0.1396918534	3.575936338	-1.68209389
H	1.9022230579	0.559594933	-1.37280053
H	2.6050359878	-1.708077380	-0.67031459
H	1.2090220803	-2.986646022	0.95589718
F	-0.9709274447	-2.112458374	1.99499094
H	-1.5512855068	0.256805695	1.15444842
H	-1.6829849846	3.404352357	-0.39910876
O	-0.7624391049	5.275752232	-1.08217876
C	-0.393047111	6.332756474	-0.63122534
H	-1.1125648585	7.159630073	-0.50218158
C	0.9948591966	6.629178293	-0.22018700
C	1.303679833	7.923639180	0.18711879
C	1.9717010059	5.633396180	-0.23253005
C	2.5941070939	8.242074955	0.57650747
H	0.5321628319	8.684142633	0.19717924
C	3.2622695677	5.937690669	0.15871315
H	1.7163895493	4.626234442	-0.53742267
C	3.5419542012	7.236823136	0.55116120
H	2.8718498433	9.235609024	0.89502742
H	4.0462582875	5.195058190	0.16711657
N	4.9257742403	7.565773399	0.96827142
O	5.7390627786	6.674340785	0.93771552
O	5.1446044872	8.703729638	1.30943195

3-Me_close

35

O	1.471763993	2.54881789	-0.10665142
B	0.3237452124	1.97625423	-0.5796047607
C	-0.094148666	0.57224770	-0.0359537696
C	0.6755741528	-0.07923960	0.9268543982
C	0.2947928806	-1.32506012	1.4062877509
C	-0.8572354859	-1.92948060	0.9266793753
C	-1.6474084819	-1.30463267	-0.0373194537
C	-1.2502088705	-0.05764452	-0.5039452839
O	-0.4824436172	2.59194641	-1.5017137882
H	1.6605715474	3.41818789	-0.4753627255
H	1.5751571859	0.39690207	1.2972976529
H	0.8951636855	-1.82754971	2.1542788663
H	-1.151580999	-2.90315069	1.3034546354
C	-2.8915791138	-1.97582741	-0.5520760128
H	-1.8507303507	0.44367605	-1.2555942844
H	-0.1483814911	3.45638669	-1.7714064085
O	0.7054200848	5.23874806	-1.2451896206
C	0.3077734553	6.27963328	-0.7817659977
H	0.9796482731	7.15407405	-0.7306176156
C	-1.0560123121	6.49690020	-0.2553087359
C	-1.4085342641	7.77574631	0.1657364044
C	-1.9666359249	5.44329667	-0.1753826498
C	-2.678078761	8.02019195	0.6623367414
H	-0.6876331502	8.58199616	0.1024020759
C	-3.2355364845	5.67381410	0.3229882529
H	-1.6767991424	4.45012511	-0.4952719748
C	-3.5606055826	6.95866894	0.7272295686

H	-2.9887261573	8.99920555	0.9951020225
H	-3.9681160513	4.88459053	0.4046707583
N	-4.9213426173	7.20884197	1.2587888638
O	-5.6786659414	6.26998767	1.3046947456
O	-5.1797534735	8.33582406	1.6091568604
H	-3.4075971951	-1.34528402	-1.2747859381
H	-3.5838384044	-2.19550884	0.2626264759
H	-2.6516179323	-2.92248536	-1.0397350088

3-Me_far

35

O	0.3265085166	2.8158944068	-1.8741431494
B	-0.470020606	2.1078325421	-1.0120964489
C	-0.047804905	0.6546194581	-0.6228285792
C	1.1004896727	0.0757938133	-1.1618764178
C	1.4684146741	-1.2158702941	-0.8107906483
C	0.6918399525	-1.9383856745	0.0821443562
C	-0.4617279959	-1.3875468327	0.6388685413
C	-0.8140648191	-0.0939251718	0.27397413
O	-1.6119630572	2.6283253748	-0.4689968511
H	-0.0092168217	3.703880719	-2.0482321375
H	1.7017311335	0.6425534677	-1.8623730553
H	2.3592129709	-1.6628314625	-1.2340458805
H	0.9810906722	-2.9481395859	0.3529705743
C	-1.2888115301	-2.1799558792	1.6142523572
H	-1.7098932223	0.351464169	0.6932676339
H	-1.8026441578	3.5321063691	-0.7415263934
O	-0.8488823415	5.4197888903	-1.3081900301

C	-0.4445572968	6.3956282172	-0.7245687079
H	-1.1194303302	7.2504000859	-0.5440243463
C	0.9313627824	6.5550099135	-0.2093070659
C	1.2886172728	7.7727977959	0.3621427586
C	1.8490138784	5.5073645835	-0.2873769072
C	2.5695131456	7.9626012075	0.8531889549
H	0.562251545	8.5745011124	0.4206509897
C	3.1294756725	5.6832293843	0.2035688995
H	1.5558937316	4.5601039235	-0.7226700844
C	3.458788894	6.9088793385	0.7596481907
H	2.8838340708	8.8938550644	1.3000055916
H	3.8679468152	4.8961918641	0.1651739398
N	4.8317326206	7.100808454	1.283807254
O	5.5954824767	6.1709567407	1.188153498
O	5.092853493	8.1753564509	1.7705077078
H	-2.2122325473	-1.6578046624	1.8608333829
H	-0.7397110389	-2.3504937302	2.5424766539
H	-1.5485933214	-3.1574900533	1.205011289

2-naph_close

38

O	0.435435404	2.5766138	-1.5431658742
B	-0.3938840632	1.9037427	-0.6846451424
C	-0.0135595609	0.4520579	-0.2489215861
C	1.1122424749	-0.1590679	-0.7441725813
C	1.4742216463	-1.4737306	-0.3646436476
C	0.649227577	-2.1736592	0.552358381
C	-0.5101623733	-1.5331687	1.0563380691

C	-0.8295688524	-0.2648531	0.6678920467
O	-1.5407946291	2.4525235	-0.1826715457
H	0.1134163138	3.4626449	-1.7503695888
C	1.9912768162	5.4342989	-0.2236523466
C	2.6796295021	7.9847513	0.7086742987
H	0.6319601661	8.4993226	0.3442020077
C	3.2878518933	5.6852768	0.18536454
H	1.7101160899	4.4493658	-0.5751271604
H	-1.712750664	3.3526887	-0.4791924422
O	-0.7445864352	5.1941055	-1.1076711167
C	-0.3510347319	6.2161897	-0.6006925154
H	-1.0493601081	7.0543768	-0.4329130361
C	1.0416655335	6.4546420	-0.168440898
O	5.2381037806	8.3434094	1.470136911
O	5.7819279746	6.3204210	0.9994826284
C	1.3829544195	7.7196511	0.3008362844
N	4.9912148921	7.2294593	1.0730406541
C	3.6006591394	6.9568051	0.638513457
H	1.744888519	0.3716327	-1.447913977
H	4.0513050909	4.9218676	0.1615614832
H	-1.7185601325	0.2168513	1.0560285978
H	2.9823873215	8.9544212	1.0744002166
C	2.6327624676	-2.1117743	-0.8721886314
H	-1.1384341413	-2.0722806	1.7558922678
C	1.0104135944	-3.4888645	0.9323926467
C	2.956453416	-3.3819298	-0.4878964539
H	3.2576693468	-1.5712330	-1.5733741497
C	2.1342497381	-4.0784835	0.4261493663
H	0.377552313	-4.0206052	1.6330816297
H	3.8434589492	-3.8610187	-0.8816028093
H	2.4004034919	-5.0846609	0.7235206365

2-naph_far

38

O	0.2957261032	2.77765516	-1.8985146736
B	-0.5724088451	2.06862023	-1.1105218975
C	-0.2241375881	0.58402183	-0.7696546336
C	-1.0453572926	-0.16175005	0.0400904551
C	-0.7475260796	-1.50624949	0.3664947859
C	0.4311557048	-2.09439623	-0.1589971332
C	1.2700832827	-1.31591735	-0.9946918466
C	0.9519909665	-0.02203546	-1.2889788312
O	-1.719873704	2.61017462	-0.6021277465
H	0.0055741455	3.68594113	-2.0495290702
C	1.8738486595	5.40804664	-0.2327700096
C	2.6401987829	7.84396683	0.9191895438
H	0.6632949648	8.51455630	0.4366693795
C	3.1467677317	5.54675415	0.2888673397
H	1.5626628714	4.46867618	-0.6725264549
H	-1.8616873114	3.53209506	-0.8417662244
O	-0.8010102782	5.39902706	-1.3176912817
C	-0.3812547765	6.36367070	-0.7262144463
H	-1.0344596667	7.23828236	-0.5623069669
C	0.9864711827	6.48302206	-0.1793592532
O	5.1469337585	7.98422260	1.8944817133
O	5.6003069801	5.96149432	1.335381834
C	1.3663321744	7.69118383	0.3976468707
N	4.8642926469	6.91548369	1.4071776621
C	3.4992346172	6.76361880	0.8499715298
H	-1.948092394	0.28447903	0.443653475
H	3.8618383673	4.73769617	0.2706355819

H	1.5975236393	0.56468881	-1.931106595
H	2.9716633734	8.76674334	1.3711893384
C	-1.5893288506	-2.28190044	1.2011953374
H	2.1687154861	-1.76995686	-1.3960125832
C	0.7298647868	-3.43924639	0.1678518928
C	-1.2758326295	-3.57735625	1.4998265213
H	-2.4885736951	-1.82580534	1.5981928782
C	-0.1010349979	-4.16254321	0.9761280746
H	1.6312408761	-3.88530491	-0.2356113336
H	-1.9253930161	-4.16192859	2.1383117339
H	0.1372083937	-5.19006488	1.2192441334

3-CF3-close

35

O	1.5175492741	2.4099433	-0.148585209
B	0.3115511157	1.8749286	-0.498721898
C	-0.0058326377	0.4063635	-0.056208124
C	0.9156782934	-0.3353533	0.683026490
C	0.6375507069	-1.6372258	1.076583530
C	-0.5733921532	-2.2183953	0.735127635
C	-1.4981717785	-1.4895889	-0.002555532
C	-1.2206906696	-0.1897800	-0.394964783
O	-0.6345454397	2.5594387	-1.209247095
H	1.646490801	3.3203904	-0.436712742
H	1.8625111099	0.1196988	0.947386665
H	1.3631447851	-2.2020482	1.646918002
H	-0.8006845023	-3.2343712	1.031406147
C	-2.8249613934	-2.1121421	-0.328288614

H	-1.9457844297	0.3670536	-0.975077484
H	-0.3507129487	3.4546613	-1.434862924
O	0.5775995484	5.1516254	-0.964777459
C	0.2323463369	6.2336632	-0.555430642
H	0.969259954	7.0498796	-0.463915212
C	-1.146829411	6.5744250	-0.150969354
C	-1.4303204053	7.8934412	0.190719146
C	-2.1413724203	5.5971955	-0.106550968
C	-2.712878973	8.2547271	0.568472703
H	-0.6454270549	8.6394752	0.158077862
C	-3.4242636441	5.9441808	0.274014212
H	-1.9059453464	4.5705753	-0.357178643
C	-3.6786522965	7.2669130	0.599217128
H	-2.9714395914	9.2682487	0.836162979
H	-4.2211693419	5.2170574	0.324455550
N	-5.0542116931	7.6423486	1.004822842
O	-5.8827676784	6.7651220	1.026244849
O	-5.2505945194	8.8007675	1.285400856
F	-3.4071894719	-1.5436138	-1.388943157
F	-2.7138463765	-3.4214318	-0.590185958
F	-3.6892575384	-2.0007047	0.693599412

3-CF3-far

35

O	0.6082216833	2.68946094	-1.6281687945
B	-0.3393699547	1.98266650	-0.9402642983
C	-0.0198329356	0.50386308	-0.535342883
C	1.186576491	-0.09335847	-0.8961547831

C	1.4750645615	-1.40434497	-0.5370555975
C	0.5540619684	-2.13656283	0.1922794773
C	-0.6537778144	-1.55115955	0.5573470032
C	-0.9419089839	-0.24574108	0.1987006547
O	-1.5479603935	2.50374575	-0.5809214747
H	0.3225043036	3.58972617	-1.8299058669
H	1.9056429781	0.48052036	-1.4682108813
H	2.4154994528	-1.85534549	-0.8250609681
H	0.7650085329	-3.15926790	0.4797744651
C	-1.6302975222	-2.37535661	1.3453291807
H	-1.8849073062	0.20114879	0.4857508612
H	-1.6825964073	3.42031702	-0.8456112583
O	-0.6258652462	5.27188880	-1.2912457183
C	-0.3077308618	6.31986626	-0.7832991007
H	-1.0451902937	7.13558365	-0.6916840007
C	1.0387246882	6.61717236	-0.2532132322
C	1.3027878592	7.90460363	0.2043304964
C	2.0215516667	5.62827653	-0.2063087874
C	2.5552155381	8.22322706	0.7026963902
H	0.5266957712	8.65963198	0.1678048624
C	3.2738381222	5.93247887	0.2936317076
H	1.7990108653	4.62573634	-0.5493441012
C	3.5104033225	7.22513580	0.7330722672
H	2.7984652799	9.21165953	1.0626989129
H	4.0607245535	5.19505750	0.3505002957
N	4.8537838548	7.55451907	1.2661340929
O	5.6739010055	6.66900960	1.2830976812
O	5.0352243014	8.68665715	1.6459721296
F	-2.7445374646	-1.70449599	1.6470788901
F	-2.0018420972	-3.47733715	0.675570062
F	-1.0991895187	-2.80008268	2.5025223158

3,5-F

32

O	0.5574900423	2.6701906748	-1.3666499629
B	-0.2935006793	1.9671729611	-0.5610396654
C	0.1069204147	0.5084019684	-0.147765618
C	1.2913126568	-0.0556279448	-0.6220977023
C	1.6227857047	-1.340530653	-0.242824461
C	0.8260214606	-2.0992530929	0.5936991116
C	-0.3414386518	-1.5148708194	1.0477983083
C	-0.7177830793	-0.2340685723	0.69748099
O	-1.4703301684	2.4707025457	-0.090313464
H	0.230615096	3.5572095501	-1.5649831398
H	1.947658579	0.4962061325	-1.2815915757
F	2.7605554954	-1.8852435001	-0.6975391898
H	1.102730727	-3.103944646	0.8791923889
F	-1.1312942815	-2.2311138439	1.8606154017
H	-1.642406062	0.1786843397	1.0781742533
H	-1.6558637366	3.3746800255	-0.3675366686
O	-0.6960288102	5.2317346339	-0.9848530561
C	-0.3512862187	6.3046484826	-0.5515529305
H	-1.0952591381	7.1068680662	-0.40812638
C	1.0362315855	6.6511290564	-0.1819661096
C	1.3129189314	7.9595679689	0.2031497305
C	2.045119333	5.6880862108	-0.2109269458
C	2.6024425782	8.3246302207	0.5527024273
H	0.5171014574	8.694295078	0.2270674616
C	3.3352131808	6.038842637	0.1405559875
H	1.8150040964	4.6692934717	-0.4962309433
C	3.5821378299	7.3510036981	0.5111690043

H	2.8558289786	9.3303408124	0.8528691252
H	4.1435219698	5.3226744487	0.1347766344
N	4.9653387573	7.7301801554	0.8861234705
O	5.8061125566	6.8653131903	0.8433971403
O	5.1553293945	8.8787967436	1.2080263773

3,5-OMe

40

O	0.5549562936	2.67324610	-2.08400762
B	-0.4229525458	2.05839427	-1.34798794
C	-0.1603801079	0.60877229	-0.81800482
C	1.0418640728	-0.03135837	-1.10616226
C	1.2773094027	-1.31536810	-0.63295627
C	0.3170659624	-1.97717175	0.13198383
C	-0.8850020282	-1.32891329	0.41535466
C	-1.1239739281	-0.04419637	-0.05481480
O	-1.6094790602	2.66370812	-1.04425390
H	0.3138043384	3.56759116	-2.35512934
H	1.8036392497	0.45455334	-1.70127456
O	2.4769323169	-1.86612567	-0.95907645
H	0.5004577281	-2.97423995	0.49814458
O	-1.8783285778	-1.89403393	1.15170881
H	-2.0657746413	0.43334952	0.18060563
H	-1.6947067605	3.55894148	-1.38865783
O	-0.5269327063	5.36822220	-1.84051635
C	-0.177007806	6.33478508	-1.20843856
H	-0.795089427	7.24960293	-1.21070598
C	1.0566144093	6.40705716	-0.39754892

C	1.3760809926	7.61464399	0.21628052
C	1.8775381487	5.28982396	-0.24374637
C	2.5234610022	7.72364213	0.98406232
H	0.7252648385	8.47172326	0.09125659
C	3.0236069153	5.38493166	0.52399516
H	1.613560089	4.35269429	-0.71779116
C	3.31930174	6.60154590	1.11743713
H	2.8052363546	8.64418376	1.47278789
H	3.6828437933	4.54213811	0.67027375
N	4.5483255126	6.70684257	1.93895361
O	5.2314507723	5.71706727	2.04228539
O	4.7830348085	7.77685246	2.44885137
C	2.756448931	-3.16803365	-0.50537395
C	-1.683122157	-3.19752755	1.64316381
H	2.0371423911	-3.89105559	-0.90103284
H	2.7541632238	-3.21873332	0.58741835
H	3.7495485159	-3.41241939	-0.87324729
H	-2.5851363719	-3.45443632	2.19244710
H	-0.8239380786	-3.24475040	2.31871712
H	-1.5406560265	-3.91353603	0.82844783

2-naph_close

38

O	0.8016917247	2.4888768449	1.5056193043
B	-0.1572587248	1.8375212504	0.7726549102
C	0.1198988532	0.4593885908	0.0682522281
C	1.2954748425	-0.3451408341	0.2359275616
C	1.4181076964	-1.5585773493	-0.4966919496

C	0.3802407616	-1.9540897785	-1.3740586611
C	-0.7371533712	-1.1859790446	-1.5197317502
C	-0.8585120523	0.0162896127	-0.7948397844
O	-1.4005360485	2.385521493	0.5975746367
H	0.4856390581	3.3336405436	1.8518950119
C	2.355918931	0.0013291102	1.1149821126
C	3.4579675378	-0.797881334	1.2454419158
H	0.4921463869	-2.8811511049	-1.9246064096
H	-1.5308443567	-1.4925825418	-2.1887848082
H	-1.7510144828	0.6159826719	-0.9206098299
H	-1.5156298543	3.2454046446	1.0150738824
O	-0.4663675959	5.0758813325	1.6503713904
C	-0.1579148395	6.1753260549	1.259054517
H	-0.8521402006	7.0229134126	1.3927130231
C	1.119040696	6.5011773269	0.5909755511
C	1.3847112508	7.8326581219	0.2845482817
C	2.0345926773	5.499067695	0.268924578
C	2.5722138164	8.1807252568	-0.3371898306
H	0.6608427335	8.5984956255	0.5360157181
C	3.2214286474	5.8334646462	-0.3565985278
H	1.8124160408	4.4647357102	0.4998452641
C	3.4618330518	7.1679404008	-0.6416527045
H	2.8147396231	9.2027511678	-0.586842167
H	3.9540045187	5.0869980323	-0.6258142611
N	4.7347308375	7.529462213	-1.3095869709
O	5.4994347219	6.6314536866	-1.5655779151
O	4.9195646495	8.6986733624	-1.5513165022
C	3.5749227314	-1.9945635112	0.5088559617
C	2.573114706	-2.3633059996	-0.3411567228
H	2.2814163007	0.911744306	1.6889019798
H	4.2506454352	-0.5105193717	1.9246588258
H	4.4538607334	-2.6158689131	0.622356302

H	2.642672563	-3.2818633307	-0.9120841614

2-naph_far

38

O	1.544386859	2.6012040467	-0.9755483988
B	0.326606653	2.0175856596	-1.1977277793
C	0.0023301847	0.5487691098	-0.7396178238
C	0.8544779923	-0.2929939605	0.0497966525
C	0.4209837892	-1.6077008461	0.3784531917
C	-0.8380791405	-2.0648750628	-0.0788511088
C	-1.640526873	-1.2565819304	-0.8287597358
C	-1.2129868712	0.0465955968	-1.151518695
O	-0.6750863249	2.7027877315	-1.8403725208
H	1.6059414387	3.5064395209	-1.3003828877
C	2.123753513	0.1210865387	0.5336031589
C	2.9011352111	-0.7146736727	1.2870559121
H	-1.1495015048	-3.0703473145	0.1801445364
H	-2.6026042231	-1.6089274682	-1.1778122193
H	-1.8607947048	0.6762544206	-1.748022543
H	-0.4073192491	3.5914936903	-2.1042791395
O	0.5126369182	5.3446290595	-1.6911732829
C	0.1883739678	6.3679989394	-1.1392564222
H	0.8438431537	7.2545445278	-1.1913760642
C	-1.0597084265	6.5468568666	-0.3689731408
C	-1.3498725963	7.8101656631	0.1379239583
C	-1.9236387785	5.4739722104	-0.1481679722
C	-2.5110026991	8.0198149946	0.8630742734
H	-0.6657079502	8.6316689213	-0.0376031482

C	-3.0836210923	5.6698268452	0.5782345144
H	-1.6818435152	4.4926358257	-0.5371126896
C	-3.3497084218	6.9399265651	1.0638962035
H	-2.7712625971	8.9858784811	1.2688977919
H	-3.7763961087	4.8648913725	0.7742516822
N	-4.59417146	7.1527446126	1.840458099
O	-5.3147444916	6.1981373629	2.0029537167
O	-4.8021967548	8.2675522812	2.2569341721
C	2.4643152857	-2.0166984289	1.6068289814
C	1.2496751056	-2.4491781509	1.1599067577
H	2.4743086278	1.1131310605	0.2951893555
H	3.8652962276	-0.3735323231	1.6423307501
H	3.0917409205	-2.6661931635	2.2034689898
H	0.8967679358	-3.4462895828	1.3959528742

2-F_close

32

O	1.4888371673	2.4816556669	-0.03632218
B	0.3120704196	2.0299455988	-0.56919086
C	-0.1284726839	0.5802604467	-0.16365991
C	0.7356949672	-0.2114896139	0.60178057
C	0.4059165233	-1.5019495635	0.98253978
C	-0.8184888555	-2.0376932315	0.60304286
C	-1.7039248975	-1.2841088499	-0.15073265
C	-1.3410262358	0.0001979559	-0.51490631
O	-0.4500484013	2.7856742655	-1.41090861
H	1.7188140409	3.3806223278	-0.29465838
H	1.6876119756	0.2126124274	0.89564122

H	1.0970620063	-2.0891721358	1.57211443
H	-1.0878613646	-3.0447275879	0.89454982
H	-2.6660110711	-1.6687547310	-0.46049009
F	-2.2269260277	0.7013021207	-1.23733247
H	-0.0635686777	3.6573284500	-1.56549403
O	0.8000480769	5.3167402698	-0.84763167
C	0.3874616594	6.3411922456	-0.36072790
H	1.0898214199	7.1558888935	-0.11290962
C	-1.0320331391	6.6154406997	-0.05502232
C	-1.3736525921	7.8822186470	0.41004979
C	-2.0060785816	5.6304787164	-0.22134669
C	-2.6926151541	8.1844507322	0.70421378
H	-0.6045550563	8.6341843205	0.53966123
C	-3.3258918955	5.9190615298	0.07348913
H	-1.7304833995	4.6426501424	-0.56885892
C	-3.636735287	7.1908964982	0.52666668
H	-2.9953621251	9.1565987633	1.06329143
H	-4.1081682051	5.1830532765	-0.03907085
N	-5.0511832841	7.5028770464	0.84107774
O	-5.8608596928	6.6223768473	0.68018570
O	-5.2968916289	8.6177878256	1.23665930

2-F_far

32

O	0.3773108507	2.7067900018	-1.4797722587
B	-0.3663644768	2.0475341338	-0.5344742667
C	0.1346310551	0.6195351705	-0.1216135216
C	1.2283283256	0.0566852765	-0.7900327559

C	1.7138331221	-1.2022283225	-0.4759897005
C	1.1066658291	-1.9389717086	0.5333320121
C	0.0227735501	-1.4162737716	1.220132333
C	-0.4370996293	-0.1564994969	0.879419309
O	-1.4810943301	2.5878350518	0.0294384564
H	0.0195739847	3.5784608988	-1.6901759637
H	1.6961858479	0.6349790757	-1.5769235803
H	2.5595362819	-1.6097046006	-1.013452301
H	1.4763332865	-2.9238564448	0.7883600351
H	-0.4724911268	-1.9620113872	2.011576953
F	-1.4844165885	0.3154810134	1.5679913085
H	-1.6941180197	3.4692496969	-0.2962437863
O	-0.8142334539	5.3149935244	-1.064170701
C	-0.4190895063	6.3496749045	-0.5846421759
H	-1.1223178936	7.1828191427	-0.4126320551
C	0.9816368195	6.6103679398	-0.1927204862
C	1.318989371	7.883688826	0.2562738345
C	1.9424503276	5.6017204563	-0.2645630472
C	2.6223869944	8.1683759363	0.6281288618
H	0.5595132054	8.6542321315	0.3126158054
C	3.245934462	5.8722635794	0.1082455953
H	1.6642870251	4.6108359529	-0.6012567535
C	3.5541624004	7.1513929692	0.542790718
H	2.9222150507	9.1449056712	0.9777128591
H	4.0183269269	5.1184691736	0.0707728664
N	4.9518901652	7.4446740499	0.9390783388
O	5.7514911981	6.5444042702	0.8536065479
O	5.1953729448	8.5650768853	1.3195855194

2-Me_close

35

O	1.3836028501	2.4487252	-0.268389435
B	0.1641096172	1.9151367	-0.5926363138
C	-0.1773453677	0.4948069	-0.0224008386
C	0.7920349785	-0.1120056	0.7828464851
C	0.600780073	-1.3650037	1.3434934906
C	-0.5855305297	-2.0390635	1.1002649257
C	-1.5596299875	-1.4554126	0.303566956
C	-1.3790733065	-0.1970591	-0.2655526199
O	-0.7278060402	2.6007737	-1.3772733662
H	1.5399100277	3.3276359	-0.6290236595
H	1.7164034758	0.4212705	0.9667971345
H	1.3687708388	-1.8107814	1.9622688286
H	-0.7556421837	-3.0192775	1.5281412376
H	-2.484287553	-1.9887298	0.116188299
C	-2.4816315513	0.3711542	-1.120667563
H	-0.3852603635	3.4620819	-1.6488639922
O	0.5563011309	5.1928320	-1.2266481864
C	0.2335709793	6.2560752	-0.7558222693
H	0.9607572956	7.0853815	-0.7142908992
C	-1.1056423451	6.5589584	-0.2091211978
C	-1.3729246853	7.8595277	0.2078934758
C	-2.0779686487	5.5637625	-0.1080097935
C	-2.6178988011	8.1848453	0.7201638154
H	-0.6048671514	8.6195297	0.1288428611
C	-3.3230134234	5.8751026	0.4060265584
H	-1.8533648785	4.5518538	-0.4214189972
C	-3.563032034	7.1802186	0.8047814312

H	-2.8629350693	9.1832934	1.0499741311
H	-4.1017770492	5.1333162	0.5040954629
N	-4.8984047564	7.5167595	1.3527221812
O	-5.7104415707	6.6259224	1.4166910316
O	-5.0828535879	8.6599109	1.6970919492
H	-2.1267515332	0.5996368	-2.125173826
H	-2.8601464383	1.3062604	-0.7072449784
H	-3.3073449113	-0.3356217	-1.1928973891

2-Me_far

35

O	0.4965029284	2.693631913	-1.7162130622
B	-0.340271709	2.0199438111	-0.8605190262
C	0.1057647648	0.5938782708	-0.3856460833
C	1.2942175326	0.0900896985	-0.9244465305
C	1.7851079154	-1.1592855611	-0.5790033194
C	1.0795382065	-1.9335294307	0.3285697588
C	-0.1012344204	-1.4522793954	0.8749113194
C	-0.6058312886	-0.1995459238	0.5343417366
O	-1.5152301117	2.5742714885	-0.4309160222
H	0.1557138366	3.5616935079	-1.9638839024
H	1.8383042979	0.7006655787	-1.6344821242
H	2.7066823394	-1.5247836369	-1.0132262841
H	1.4449890536	-2.9125861417	0.6127270155
H	-0.6477580944	-2.0633013345	1.5837301195
C	-1.8956105328	0.254272887	1.1662142127
H	-1.6779667575	3.4573434748	-0.7807540444
O	-0.7465109471	5.3103148094	-1.4162074003

C	-0.3894633021	6.3415847332	-0.9008618425
H	-1.088089706	7.1931103735	-0.8274564529
C	0.9560577792	6.5754998955	-0.3366530198
C	1.259750495	7.8440043155	0.1488139698
C	1.897690205	5.5475423787	-0.2848829485
C	2.5108806594	8.1043275215	0.682535709
H	0.5151567972	8.6298663911	0.1066841762
C	3.148850163	5.7941237557	0.2493830482
H	1.6454902956	4.5610669038	-0.6537787927
C	3.4251924777	7.0685888755	0.7176855742
H	2.7837609301	9.0761854825	1.0655605032
H	3.904420737	5.0249365401	0.3105494535
N	4.7669828192	7.3360665281	1.2876100977
O	5.5530345977	6.4202311451	1.3053062494
O	4.9822830267	8.4529582154	1.6947984525
H	-2.6576710395	0.4480976752	0.4116961021
H	-1.7614794446	1.1866795659	1.7142206552
H	-2.2679545042	-0.5047643118	1.8530927022

2,4-Me_close

38

O	0.5241947762	2.6553773678	-1.6786657569
B	-0.3348756639	1.9740323329	-0.8534323859
C	0.0079547031	0.5381245309	-0.3326291381
C	1.1747178761	-0.1788568658	-0.6616854583
C	1.3589123744	-1.4505537406	-0.130164552
C	0.4324358184	-2.047605215	0.7205051268
C	-0.7184483859	-1.3381216332	1.0390807656

C	-0.9188214156	-0.0695870522	0.5185106624
O	-1.5184840814	2.5310010872	-0.4436542952
H	0.1917424296	3.5320431673	-1.9101402007
C	2.235410501	0.3787479317	-1.57490444
H	2.2577536283	-2.0007669527	-0.3901227117
C	0.6907038258	-3.4139675899	1.2916375701
H	-1.4600009236	-1.7813559582	1.6934611273
H	-1.8211747604	0.4734883351	0.7713622776
H	-1.6741976433	3.4193140022	-0.7803321036
O	-0.6667323628	5.2882448748	-1.3629252283
C	-0.2957483734	6.2966965885	-0.8137910993
H	-0.9729906858	7.165071769	-0.7372477265
C	1.040713708	6.4810992901	-0.2101028314
C	1.3651519827	7.7291837304	0.3132389596
C	1.9534438888	5.4273964148	-0.1592143173
C	2.6083490415	7.9430389217	0.8849941398
H	0.6429915603	8.5357102831	0.270991794
C	3.1960895985	5.6273381701	0.4129507634
H	1.6847038268	4.4573943028	-0.5586740413
C	3.4936518151	6.8823834005	0.9190316257
H	2.8967971763	8.8979462604	1.2980964902
H	3.9293715801	4.8368986001	0.4750809797
N	4.8261678938	7.0996366901	1.530397626
O	5.5858992107	6.1617627503	1.5489286044
O	5.06112861	8.2009841762	1.967897893
H	2.6637246089	1.2954948573	-1.1688295093
H	1.8229740645	0.6374510735	-2.5496361776
H	3.0361939702	-0.3465353506	-1.7136460899
H	1.1376887906	-4.0737336884	0.5475270553
H	-0.2303262491	-3.8720611184	1.6503009894
H	1.3833552855	-3.353784744	2.1341976131

2,4-Me_far

38

O	1.6189420943	2.6249904715	-0.4982133317
B	0.4755568496	2.0324837321	-0.9630026323
C	0.0634702099	0.5930040208	-0.5067436274
C	0.782734714	-0.1953756184	0.4123431251
C	0.3063023331	-1.4618132832	0.7333896033
C	-0.8592798705	-1.9857239003	0.1807854613
C	-1.562613422	-1.2061943907	-0.7287158442
C	-1.1033219538	0.0586566662	-1.0596918323
O	-0.3602323251	2.6845839079	-1.8375150284
H	1.7585396184	3.5160327509	-0.8372974623
C	2.056165271	0.2815916253	1.0601793614
H	0.8656678343	-2.0668412608	1.4399352504
C	-1.3480463745	-3.3496102869	0.5817310884
H	-2.4690824476	-1.5920143853	-1.1804091252
H	-1.6578501502	0.6556431798	-1.7734213416
H	-0.0458109166	3.5671557954	-2.0675620208
O	0.7617015081	5.3519296066	-1.4433079814
C	0.3502775772	6.3337980421	-0.8749433048
H	0.9977416942	7.2214067525	-0.7667068635
C	-0.9996243973	6.4596493439	-0.2867284285
C	-1.3704789107	7.6819149522	0.2661594344
C	-1.879277014	5.3770067153	-0.2791330611
C	-2.6277849148	7.8408323909	0.8246427946
H	-0.6733722139	8.5112379998	0.2575480401
C	-3.1357922192	5.5221940575	0.2793275927
H	-1.5752397115	4.4274072055	-0.7017808108
C	-3.4797975135	6.7526622301	0.8154006116

H	-2.951986208	8.774236304	1.2597709708
H	-3.8444708878	4.7077983818	0.3077583972
N	-4.8272983241	6.9116126399	1.4117590544
O	-5.5587104647	5.9516158559	1.3900704399
O	-5.1018617108	7.9917438692	1.8783328992
H	2.4430340411	-0.4762407571	1.7403533592
H	1.8939899987	1.2035348331	1.6182221423
H	2.8182710415	0.5045064461	0.3136708457
H	-0.5184680596	-4.0481559179	0.693516753
H	-2.0417118263	-3.7524424759	-0.1554088226
H	-1.8696509186	-3.3043200397	1.5404977139

4-F

32

O	0.4504824417	2.7125573994	-1.4696884778
B	-0.3249522663	2.0162317652	-0.5803477739
C	0.1224073986	0.5808954861	-0.1600048129
C	1.2715224751	-0.0008933614	-0.6998713972
C	1.677415082	-1.2745464829	-0.3317851841
C	0.9121052798	-1.9626614477	0.5905449453
C	-0.2328607718	-1.4297757659	1.1516307254
C	-0.6186283441	-0.1548407384	0.7674124134
O	-1.4698852231	2.5277911633	-0.0365737735
H	0.0948724273	3.5884903882	-1.6651667235
H	1.8542448559	0.5563113626	-1.4232715635
H	2.5637686411	-1.7394122686	-0.7415584618
F	1.2953629997	-3.1958183279	0.9547954313
H	-0.7956679758	-2.0121454717	1.8683632499

H	-1.5131925429	0.2834122739	1.1925478481
H	-1.6820848831	3.4214390006	-0.3265818574
O	-0.7897194729	5.2911431307	-0.9990024465
C	-0.4078415625	6.3348416316	-0.5281583615
H	-1.1255640071	7.1523695635	-0.3417679029
C	0.9946390832	6.6262824487	-0.165853779
C	1.3157558208	7.9094743412	0.2667251505
C	1.9736807052	5.6361420491	-0.2497745882
C	2.6207198755	8.2227482861	0.608854367
H	0.5425591284	8.6654672142	0.3329929735
C	3.2788906755	5.9352271358	0.0937806563
H	1.7086978784	4.6370104282	-0.5722986302
C	3.5705975628	7.2236992920	0.5118154019
H	2.908460992	9.2077814607	0.9444284613
H	4.0651652196	5.1965116260	0.0460479532
N	4.9700807746	7.5478591225	0.8768125973
O	5.7848683233	6.6620671471	0.784587118
O	5.1991994092	8.6767401476	1.2407664414

4-Me

35

O	0.0779969566	2.77717274	-1.7044431308
B	-0.5732046648	2.10718265	-0.7009072479
C	-0.105569339	0.66333517	-0.3388270452
C	0.9320457101	0.04694635	-1.0394532663
C	1.3526481148	-1.23423216	-0.7161922168
C	0.7480283794	-1.94285685	0.3188562909
C	-0.2868221595	-1.33104968	1.0237327938

C	-0.7070579896	-0.05118962	0.6994755144
O	-1.6060786666	2.66082903	0.0045595221
H	-0.2714509885	3.66466612	-1.8514655553
H	1.4133957571	0.58363754	-1.8485313063
H	2.1622937316	-1.69406530	-1.2714827738
C	1.1796580216	-3.34389168	0.6523606782
H	-0.7642240108	-1.86771443	1.8359962009
H	-1.5131092482	0.40996232	1.2577884725
H	-1.8327560491	3.55668327	-0.2662129789
O	-0.9725317886	5.41877976	-1.0426293321
C	-0.482382221	6.39177342	-0.5236673546
H	-1.108130231	7.27399913	-0.3024427101
C	0.9401511538	6.51312882	-0.1416446332
C	1.3850214485	7.72409628	0.3805393874
C	1.8148342367	5.43707793	-0.2924620732
C	2.7106764911	7.87813242	0.7504529662
H	0.6915798465	8.54844286	0.4961658102
C	3.1398379925	5.57731685	0.0770575103
H	1.4544039819	4.49669036	-0.6906299398
C	3.5554114866	6.79649291	0.5876994446
H	3.0925993521	8.80281016	1.1563631172
H	3.8476782193	4.76755219	-0.0205024199
N	4.9759048206	6.95002705	0.9817650484
O	5.6987855398	5.99545842	0.8291136854
O	5.313514751	8.02073699	1.4282672476
H	2.2051088804	-3.52793381	0.3335050019
H	0.5398865611	-4.07279276	0.1495393704
H	1.1120559235	-3.53240447	1.7238559222

4-OMe

36

O	0.1565529692	2.69747793	-1.665979893
B	-0.5490689721	2.05406441	-0.680118935
C	-0.1109078223	0.62198181	-0.256019728
C	0.9570800357	-0.01965538	-0.876703797
C	1.361256048	-1.29788503	-0.509897579
C	0.6799166981	-1.95898772	0.508052502
C	-0.3949337706	-1.33679747	1.145759477
C	-0.7781644446	-0.06815122	0.764425774
O	-1.6160494477	2.63021488	-0.045608138
H	-0.1844990873	3.58044002	-1.853419460
H	1.4874077378	0.49107417	-1.671884104
H	2.194388841	-1.76234264	-1.017035231
O	0.9870597382	-3.20462433	0.945707413
H	-0.9064475092	-1.87503692	1.932799765
H	-1.6140913001	0.40890030	1.261846146
H	-1.8228320023	3.52022360	-0.349308246
O	-0.9146167501	5.35802092	-1.103280106
C	-0.4422948258	6.33554840	-0.576603476
H	-1.079428645	7.21217429	-0.365885397
C	0.9732078714	6.46947779	-0.172670978
C	1.4040266106	7.68857610	0.342284880
C	1.8554768292	5.39641784	-0.298255797
C	2.7237307965	7.85429353	0.728225991
H	0.7045335928	8.51019790	0.439343546
C	3.174390714	5.54825233	0.087760185
H	1.5051767547	4.44881326	-0.688215656
C	3.5765746996	6.77574686	0.589125169

H	3.0949555427	8.78568775	1.128696227
H	3.8877521171	4.74126428	0.009818234
N	4.990829139	6.94192691	0.999726825
O	5.7214686483	5.99041203	0.865298298
O	5.3161253711	8.01888327	1.440232523
C	2.0625064205	-3.87365283	0.328083161
H	2.1395196823	-4.84133783	0.816525075
H	2.9987113068	-3.32454440	0.459964732
H	1.8775815027	-4.01908697	-0.739608010

4-tBu

44

O	0.2457636752	2.6679359304	-1.9521315666
B	-0.6277815535	2.0010321076	-1.1322922875
C	-0.3185315505	0.5197583404	-0.7510923285
C	0.8012465583	-0.1384104872	-1.2526091096
C	1.0777999228	-1.4575731557	-0.9137244859
C	0.2414646033	-2.169575937	-0.0573985972
C	-0.8829866302	-1.5081468902	0.4462107483
C	-1.1575941169	-0.1954011634	0.1076229084
O	-1.7542108158	2.5873183098	-0.6242384773
H	-0.0260947344	3.5774891618	-2.1259121734
H	1.4678075955	0.3905965019	-1.92378891
H	1.9585744917	-1.926713959	-1.3298056862
C	0.5047627096	-3.6203990515	0.3416941938
H	-1.5572639382	-2.0298100039	1.1148828445
H	-2.0372577871	0.2917728025	0.5113634437
H	-1.8758336006	3.5052180201	-0.8888723365

O	-0.7911698166	5.3342931814	-1.3841583139
C	-0.3595179957	6.2685708419	-0.754046281
H	-0.9910540701	7.1572775033	-0.580597667
C	0.9967177469	6.3317924737	-0.1702668926
C	1.3961715932	7.510297293	0.4530686097
C	1.8543616878	5.2336596606	-0.2363246543
C	2.6607874702	7.610203412	1.0087669461
H	0.7156855556	8.3519677315	0.5013895655
C	3.1177766541	5.3197069869	0.3186625124
H	1.5273733915	4.3175553107	-0.7123015696
C	3.4907278099	6.5082593459	0.9254029344
H	3.0069913605	8.5087171937	1.4971337758
H	3.8101547118	4.4913812468	0.2913923336
N	4.8457948732	6.6039399814	1.5179780081
O	5.55813306	5.6334373063	1.4296249562
O	5.145183169	7.6473277799	2.0484433374
C	1.7722353821	-4.1790259189	-0.3047815529
C	0.6632490592	-3.706930432	1.8662366697
C	-0.6820468808	-4.4907755526	-0.0951254654
H	2.6573620111	-3.6144287028	-0.0063116802
H	1.7056232042	-4.1670341723	-1.3941383519
H	1.9140179696	-5.2141819317	0.0101779009
H	1.5005517233	-3.0925210916	2.2018065467
H	0.8524584644	-4.7403583422	2.1650406962
H	-0.2342119214	-3.3653605775	2.3825410025
H	-0.8174762025	-4.4423636938	-1.1769816773
H	-1.6108755624	-4.168195278	0.3758956665
H	-0.5054692771	-5.5319020821	0.1840644638

4-CF3

35

O	0.4286163459	2.6805291	-1.4284422439
B	-0.4448808089	1.9644239	-0.6578492335
C	-0.0967891962	0.4733253	-0.322939227
C	1.0619584422	-0.1173867	-0.8236735036
C	1.3790171307	-1.4367538	-0.530102592
C	0.5259913564	-2.1747271	0.2747979489
C	-0.6367140859	-1.6087580	0.7855336896
C	-0.9413394915	-0.2929043	0.4850420449
O	-1.6029039011	2.4857974	-0.1585403504
H	0.130104441	3.5866290	-1.5793067962
H	1.7219043504	0.4658798	-1.4538541305
H	2.2790571467	-1.8897680	-0.9222361108
C	0.8321894706	-3.6041544	0.6201285521
H	-1.2928534217	-2.2003579	1.4118178945
H	-1.8453609883	0.1558579	0.8774285579
H	-1.7552425627	3.4092140	-0.3878144023
O	-0.7398491472	5.2622008	-0.9226886513
C	-0.3567581794	6.3108432	-0.4631715922
H	-1.0750607863	7.1278494	-0.2783849776
C	1.0474527944	6.6075547	-0.1135678474
C	1.3690897136	7.8955583	0.3039796914
C	2.0279280547	5.6184323	-0.1936086048
C	2.675688519	8.2144195	0.634974799
H	0.5949747244	8.6508342	0.367406596
C	3.334681229	5.9229240	0.138917783
H	1.762984986	4.6153988	-0.5035327173
C	3.6265030201	7.2160391	0.5423597527

H	2.96368564	9.2032820	0.9589642157
H	4.1222511516	5.1853745	0.0939141327
N	5.0278043726	7.5455869	0.8963795878
O	5.8428424849	6.6596930	0.8083620404
O	5.2571037755	8.6782058	1.2478443872
F	1.9646933808	-4.0371011	0.0585044027
F	0.9578946295	-3.7763688	1.9442765755
F	-0.146464591	-4.4298730	0.2219803289

phenyl

32

O	0.3897700032	2.74256966	-1.4770006738
B	-0.3485876852	2.03635829	-0.5637191591
C	0.1357590047	0.60818661	-0.1537953905
C	1.2846721041	0.05459842	-0.722501596
C	1.7214953513	-1.21146677	-0.3607959119
C	1.0102147701	-1.94514909	0.5798293279
C	-0.1356937385	-1.41130644	1.1554420341
C	-0.5669149268	-0.14451239	0.7894038337
O	-1.4838088323	2.53559913	0.0114495969
H	0.0171840104	3.61341842	-1.6629757345
H	1.8359520471	0.62788778	-1.4583111076
H	2.6137038399	-1.62845964	-0.8104553634
H	1.3483589137	-2.93391458	0.8637118432
H	-0.6905649193	-1.98416502	1.887614201
H	-1.4608228053	0.27442489	1.2357827534
H	-1.7138576199	3.42576599	-0.2753587485
O	-0.8475012751	5.32186303	-0.9903173737

C	-0.4374242564	6.36719866	-0.5481273247
H	-1.1303284817	7.21338260	-0.3985387657
C	0.968864275	6.62601338	-0.1745945627
C	1.3218773026	7.91024321	0.2294489329
C	1.9194361668	5.60604679	-0.2194430098
C	2.6303393589	8.19410975	0.5835351555
H	0.5705019844	8.68996208	0.264496289
C	3.2278941316	5.87589874	0.1365196017
H	1.6307590662	4.60753137	-0.5232967128
C	3.5514657586	7.16541008	0.526780373
H	2.9420346179	9.17872410	0.8983095208
H	3.9925289385	5.11348255	0.1189349162
N	4.954246223	7.45749319	0.9057445302
O	5.7440453292	6.54652427	0.8476526182
O	5.2113013431	8.58778096	1.2462759069

boronic acid + aldehyde (pi interaction) - xyz structures

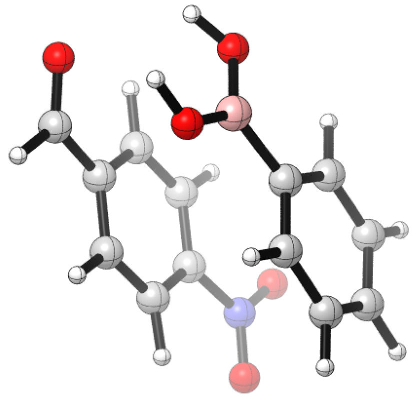

phenyl

32

O	1.2106274651	2.2088190614	-0.0004337685
B	-0.0003688236	1.5623716947	-0.0000840818
C	-0.0002948089	0.0000959059	0.0001733454
C	1.198053489	-0.7178682649	0.0156647494
C	1.2010804723	-2.0997222089	0.1385416677
C	-0.002092889	-2.7881308935	0.2419204952
C	-1.2050336571	-2.0940799032	0.2030071182
C	-1.1991190382	-0.7107918149	0.0842161764
O	-1.1974568081	2.21656047	0.0612550830
H	1.1550461262	3.1691345912	0.0030429506
H	2.1356754417	-0.1780705337	-0.0510940980
H	2.137441499	-2.6427543068	0.1607109342
H	-0.0023817512	-3.8648485958	0.3546896825
H	-2.1418744733	-2.6304979922	0.2832827217
H	-2.1352720506	-0.1645137519	0.0772933314
H	-1.1468255479	3.1518460134	0.2847278839
O	-0.0330575493	3.8937085043	2.3719002976
C	0.6805490427	3.0262090544	2.8090980564
H	1.7535935443	3.217265922	2.9922078120
C	0.2285470558	1.6447635965	3.0915594903
C	1.1838866012	0.6477278041	3.2459751049
C	-1.1312356376	1.3370768975	3.1062831700
C	0.7933257877	-0.6756797289	3.3786164106
H	2.2365524795	0.9028595484	3.2194373005
C	-1.5348221667	0.0256175215	3.2571785515
H	-1.8532408392	2.1307267879	2.9699436208
C	-0.5590449852	-0.9529585615	3.3736525426
H	1.5067941884	-1.4817193255	3.4605325980
H	-2.5767806009	-0.2576129722	3.2547816415
N	-0.9864665905	-2.3656403453	3.4646640437
O	-2.171215335	-2.5938680796	3.3896226603
O	-0.1247896403	-3.2017260944	3.6010325078

3-F_close

32

O	1.2398613955	2.165983175	0.007285195
B	-0.0037258793	1.594810844	-0.067228124
C	-0.1028740302	0.033112291	-0.096850288
C	1.0270704333	-0.764388433	0.084955704
C	0.9122923615	-2.143993718	0.206541836
C	-0.3336038519	-2.751498676	0.137695102
C	-1.4448676443	-1.952502349	-0.066789768
C	-1.3542704756	-0.581412613	-0.182085412
O	-1.1656363793	2.309579933	-0.053552503
H	1.2388439891	3.127669383	0.028068429
H	1.999837514	-0.293320883	0.154226058
H	1.7914629947	-2.754103042	0.369015381
H	-0.4585090409	-3.820366013	0.249231280
F	-2.6513306437	-2.533218623	-0.135974063
H	-2.255303223	0.005279602	-0.311433059
H	-1.082196623	3.232201474	0.209936835
O	-0.1044400408	3.858881215	2.319863864
C	0.6226747099	3.054372753	2.845871757
H	1.6466954356	3.337624005	3.151293190
C	0.2632955427	1.638513741	3.088521454
C	1.2813467831	0.734639888	3.377612773
C	-1.0523460666	1.206055331	2.943551082
C	1.0011715381	-0.616683047	3.478287018
H	2.2995829297	1.087982772	3.487097758
C	-1.3487060167	-0.140463050	3.058234006
H	-1.823654977	1.926743590	2.707653798
C	-0.309716355	-1.021617359	3.304755035

H	1.7691061473	-1.352279370	3.665078783
H	-2.3517779183	-0.519783139	2.929332788
N	-0.6091213553	-2.469388959	3.356147160
O	-1.7597193037	-2.805227004	3.209059896
O	0.3199580499	-3.223403719	3.531697035

3-F_far

32

O	1.2079294788	2.2223392973	-0.0143683901
B	-0.0102092067	1.594260488	-0.0409492161
C	-0.0252605278	0.0294985184	-0.064704475
C	-1.2298695989	-0.6762309108	-0.0534270061
C	-1.244632789	-2.0603413257	0.0523072971
C	-0.052356275	-2.7664281291	0.1532307041
C	1.1344807798	-2.0584712873	0.1230644724
C	1.1719499211	-0.6837006373	0.0110046518
O	-1.2020953152	2.2553309522	0.0144053719
H	1.167805405	3.1830019838	0.016640762
H	-2.1619461484	-0.1270788573	-0.1046484371
H	-2.1839242422	-2.5971062803	0.0770538465
H	-0.0299936244	-3.8425826744	0.2603679934
F	2.2914506972	-2.7328321311	0.2276186602
H	2.1269595671	-0.1729553669	0.0087779108
H	-1.1472111164	3.1873701161	0.2504764742
O	-0.0113381655	3.871100526	2.3585388719
C	0.6927060071	3.0067819263	2.8171671387
H	1.7556266163	3.206674226	3.0442795358
C	0.2424101419	1.6186258657	3.0687922961

C	1.1994247984	0.6264602379	3.2472517143
C	-1.1143425055	1.3014228525	3.0304012676
C	0.8143340874	-0.7013413744	3.3460591256
H	2.2503578584	0.8892173341	3.2666408833
C	-1.5132913537	-0.0153707907	3.1468578036
H	-1.8372786951	2.0912537735	2.8782660317
C	-0.5354679195	-0.9882796975	3.2849656689
H	1.5310897473	-1.5034537906	3.4430094122
H	-2.5523764765	-0.305606289	3.102146549
N	-0.9552696325	-2.4054833484	3.3359422275
O	-2.1360755467	-2.6401195149	3.2259982429
O	-0.0906607568	-3.238040132	3.4739326107

3-Me_close

35

O	1.2390589312	2.207753640	-0.0097949731
B	0.0254207617	1.566816991	-0.0476041965
C	0.0164450698	0.004669196	-0.0611641854
C	1.2084163558	-0.720621395	-0.004567524
C	1.1851835644	-2.102513919	0.1089495923
C	-0.0292970259	-2.775366846	0.1566599483
C	-1.236151531	-2.084044600	0.0736330132
C	-1.1895977449	-0.696208185	-0.0299644298
O	-1.1691530144	2.228637307	-0.014520515
H	1.187006184	3.168202071	-0.0018880269
H	2.1535130303	-0.190864355	-0.0275395304
H	2.1107948825	-2.661056138	0.1704253563
H	-0.0424046228	-3.854057233	0.2648567282

C	-2.5493590961	-2.816896808	0.0857278543
H	-2.119190054	-0.136769986	-0.0640040318
H	-1.1181345342	3.161875279	0.21711437
O	-0.0651440494	3.885460231	2.3369653069
C	0.6464887954	3.025429724	2.7917606852
H	1.7099860904	3.231104116	3.0114404558
C	0.2069571612	1.635248940	3.0495993312
C	1.1728819872	0.653192317	3.231782983
C	-1.1469259194	1.304852870	3.0113602954
C	0.800217394	-0.677479193	3.3393555436
H	2.2212682441	0.926023825	3.2465723352
C	-1.5332492615	-0.014643314	3.1353489647
H	-1.8767466326	2.087137738	2.8530503471
C	-0.5461260261	-0.977526225	3.2813158908
H	1.523661374	-1.472181096	3.4419083738
H	-2.5694568304	-0.315200152	3.0901514611
N	-0.9520949442	-2.397588073	3.3477130213
O	-2.1300415412	-2.644976881	3.2286209365
O	-0.0833906695	-3.220841817	3.5127245282
H	-3.3315791802	-2.210467294	0.5420822824
H	-2.4750203172	-3.748095228	0.6463158366
H	-2.8640368309	-3.058205506	-0.9321880285

3-Me_far

35

O	1.1551716466	2.2567617833	-0.0292948855
B	-0.0741005171	1.6489242663	-0.0827608902
C	-0.1305334597	0.0886309983	-0.1388858279

C	-1.3550149552	-0.5770769501	-0.2174293286
C	-1.4118867572	-1.9595576063	-0.1175074528
C	-0.2459874548	-2.6897641332	0.0705135726
C	0.993106139	-2.055790026	0.1415388865
C	1.0297483612	-0.6697683744	0.0243320312
O	-1.252016363	2.3383147523	-0.0207101018
H	1.1255390356	3.2164743346	0.0267349412
H	-2.265275208	-0.0008635558	-0.33333342
H	-2.364052549	-2.4723865141	-0.1670133625
H	-0.2972006693	-3.7677470152	0.1797184715
C	2.2429479146	-2.8554810323	0.3835567777
H	1.9843998402	-0.1578118394	0.0911335148
H	-1.1760019566	3.2620920136	0.2392732921
O	-0.0664590164	3.8342997224	2.4002675466
C	0.6858485584	3.0073910987	2.8501798854
H	1.7283819706	3.2736769316	3.1041864969
C	0.3305422713	1.58547213	3.0621886361
C	1.3526612452	0.6665507832	3.2809005286
C	-0.9925586122	1.165936785	2.9557468185
C	1.0665481921	-0.6854554727	3.352145627
H	2.3773280157	1.009856374	3.3577039005
C	-1.294538708	-0.1811511754	3.0433244432
H	-1.7661352781	1.8985983187	2.769561322
C	-0.2540084944	-1.0760559173	3.2234316442
H	1.8369852769	-1.4309879693	3.4821932103
H	-2.3044548584	-0.5497391657	2.9431163721
N	-0.5648158035	-2.5208744795	3.2516599848
O	-1.7279105896	-2.8430136644	3.2190779827
O	0.3684746723	-3.2900132202	3.3003235249
H	3.1289205176	-2.2225947808	0.3442294958
H	2.3558611537	-3.645443675	-0.3604856962
H	2.2026573893	-3.3349071747	1.3646171686

2-naph_close

38

O	1.149373769	2.3434105105	-0.1433260099
B	-0.0216609104	1.6295674493	-0.0926266095
C	0.0764835659	0.0740208082	-0.1752794786
C	1.3340684224	-0.567018199	-0.3545623832
C	1.4512668065	-1.9245104881	-0.28669028
C	0.3179764621	-2.7380692956	-0.0284657808
C	-0.9466140921	-2.116299959	0.1342789295
C	-1.0311690588	-0.704635074	0.0496779418
O	-1.2440360381	2.2063319708	0.1011563511
H	1.0486269676	3.2962850368	-0.0561840613
C	-1.1047747283	1.2346552569	3.1664388067
C	0.6880496411	-0.9134782381	3.128611749
H	2.2088855085	0.5674000812	2.8243936868
C	-1.5791007408	-0.0502687389	3.3404807121
H	-1.7782399112	2.0809586724	3.1488166986
H	-1.2260302409	3.1298716073	0.3722979688
O	0.0726161228	3.7527945723	2.4352887554
C	0.7724608605	2.8158537333	2.728300542
H	1.8701192884	2.9268673094	2.7865203715
C	0.2611505932	1.4509733819	2.9916909133
O	-0.3817205134	-3.3788852508	3.3673606176
O	-2.3655769398	-2.6123441707	3.6535821048
N	-1.1801664788	-2.4775761083	3.454721399
C	1.1528459089	0.3845274501	2.9840710699
H	-2.629027303	-0.2610086226	3.4790519346
H	-1.9971993059	-0.2325079965	0.1973579759
C	-0.6686995813	-1.0960819458	3.3085492785

H	2.2082594476	0.0464420982	-0.5370664838
H	1.3454210904	-1.7691354206	3.0870784275
C	-2.0789020027	-2.920697552	0.4105958167
H	2.4138177272	-2.4041630679	-0.4225406375
C	0.4083635685	-4.1455242422	0.0829686499
C	-1.9582540884	-4.2763934483	0.5280887664
H	-3.0400401769	-2.4386223533	0.5464641307
C	-0.7005516887	-4.8951592171	0.3579917525
H	1.3754091599	-4.6177050994	-0.0427507466
H	-2.8249710227	-4.8810540118	0.7597147076
H	-0.6183324583	-5.9697250087	0.4557380528

2-naph_far

38

O	0.9671777102	2.3972789684	-0.1201009069
B	-0.202427058	1.6803401628	-0.103652548
C	-0.122572474	0.1246532321	-0.2154315089
C	-1.3071876505	-0.6641284603	-0.2229429117
C	-1.2503181062	-2.0249476388	-0.1559304641
C	-0.0019215212	-2.694151542	-0.0697978542
C	1.189146762	-1.9251387193	-0.0844229896
C	1.091092629	-0.5139732628	-0.1654937918
O	-1.430199803	2.2510969362	0.0769266411
H	0.8563597791	3.34722681	-0.0187330669
C	-0.8414536134	0.9801557178	2.9645031997
C	1.4016558701	-0.6790234989	3.1673692932
H	2.5477853903	1.1317062723	3.1418775592
C	-1.0109244931	-0.3916586183	3.0229140112

H	-1.6905880984	1.6415119574	2.8573685929
H	-1.4198849185	3.1661417569	0.3764397105
O	-0.2065967743	3.7434062177	2.459675219
C	0.6484844999	2.9751501283	2.8222416175
H	1.6780030077	3.3294759581	3.01429055
C	0.4413455822	1.5202320137	3.0014114018
O	0.942304276	-3.3335195416	3.2210472356
O	-1.1828911952	-3.088691587	3.0494172027
N	-0.0554020158	-2.6588885109	3.1282920371
C	1.5562393053	0.6955470133	3.1202533272
H	-1.9867260955	-0.851340624	2.9752498083
H	2.0049355792	0.0714588275	-0.1515482995
C	0.1171848289	-1.1890335123	3.1105716732
H	-2.2655842814	-0.1601327448	-0.2600911697
H	2.2428702359	-1.3541278225	3.2233946816
C	2.4365108085	-2.5867896732	0.0243394634
H	-2.1570796137	-2.6182543075	-0.1441986994
C	0.0910349404	-4.1003797526	0.0593674697
C	2.4957766079	-3.9458393416	0.1505602157
H	3.3429968465	-1.9925760972	0.0105503096
C	1.3084205172	-4.7105097667	0.1696958019
H	-0.8224555889	-4.6818124533	0.0883313843
H	3.4525655541	-4.4432217331	0.2415808927
H	1.3680276306	-5.7850153035	0.2825905416

3-CF3-close

35

O	1.3067069571	2.11985571	-0.002238128

B	0.0766686764	1.52530181	-0.103574522
C	0.0060369848	-0.03873766	-0.086698043
C	1.1454463641	-0.82057863	0.111930554
C	1.0511739321	-2.19617796	0.272411185
C	-0.1901802645	-2.81729307	0.233091542
C	-1.3274269238	-2.05080299	0.020190454
C	-1.2315867375	-0.67670492	-0.141988816
O	-1.0988632988	2.21278689	-0.155738138
H	1.291854153	3.08149936	-0.020877054
H	2.1132172038	-0.33549024	0.163169960
H	1.9398124072	-2.78920643	0.444381189
H	-0.2775601064	-3.88488814	0.384861338
C	-2.6894758536	-2.68329598	-0.030016162
H	-2.1302912232	-0.08612198	-0.280597382
H	-1.0519939952	3.14314863	0.089190647
O	-0.2324076854	3.84826737	2.210891838
C	0.4951161737	3.07911503	2.786525873
H	1.4969847776	3.40031045	3.126060218
C	0.1675822932	1.65890939	3.050134754
C	1.2036235509	0.79207686	3.385275872
C	-1.1308283787	1.18465095	2.880563409
C	0.9599997842	-0.56424296	3.510034583
H	2.2078376664	1.17788997	3.513259288
C	-1.3919132397	-0.16736461	3.019288107
H	-1.9159119767	1.87707576	2.607702890
C	-0.333913936	-1.01120043	3.313385497
H	1.743525965	-1.27244859	3.734242564
H	-2.3797588721	-0.57965222	2.870629175
N	-0.5905197202	-2.46597600	3.391906548
O	-1.7250572348	-2.84089797	3.217209615
O	0.3555269322	-3.18644125	3.612428135
F	-3.551820633	-2.05768811	0.786154979

F	-3.218368706	-2.61355521	-1.261293880
F	-2.6715350361	-3.96992283	0.317601912

3-CF3-far

35

O	1.2077955116	2.23876907	0.02117762
B	-0.0190175886	1.63327711	-0.03342743
C	-0.0594032574	0.06948782	-0.10022879
C	-1.2753971574	-0.61823981	-0.15652303
C	-1.3215262162	-2.00160840	-0.08742747
C	-0.1446491503	-2.72950149	0.04736023
C	1.06719379	-2.05818661	0.09351447
C	1.1127909197	-0.67188042	0.01245693
O	-1.2032924231	2.30645939	0.02681563
H	1.1858619761	3.19839356	0.08481954
H	-2.1949479493	-0.05002441	-0.23354935
H	-2.2704329218	-2.52024391	-0.11935380
H	-0.1702556853	-3.80755228	0.13075139
C	2.3584513771	-2.80481544	0.26382433
H	2.0675816351	-0.16007735	0.06239643
H	-1.1403709763	3.23134303	0.28794747
O	-0.0071290125	3.82617638	2.43386652
C	0.7001361733	2.96592446	2.89513465
H	1.749976032	3.18310796	3.16346191
C	0.2726979473	1.56226646	3.09685471
C	1.2451646502	0.58608369	3.28427778
C	-1.0746990728	1.21780104	3.00628488
C	0.8865794897	-0.75162185	3.33499493

H	2.2888504171	0.86945970	3.34989299
C	-1.448154455	-0.11056226	3.07411724
H	-1.809818407	1.99586864	2.85176587
C	-0.4538357985	-1.06555534	3.21614843
H	1.6182541113	-1.53930550	3.43514776
H	-2.4786837439	-0.42149089	2.98787627
N	-0.842278098	-2.49250456	3.20417491
O	-2.0210972857	-2.74807809	3.12198844
O	0.0446171922	-3.31125748	3.26194746
F	3.205717971	-2.57020526	-0.74742725
F	2.9995225414	-2.42532500	1.38519814
F	2.1853685736	-4.12432655	0.33924018

3,5-F

32

O	1.1933827531	2.18729151	-0.0235092519
B	-0.0438365486	1.60453057	-0.0736379942
C	-0.1162951429	0.03879966	-0.096557103
C	1.0397939445	-0.71672879	0.0892367986
C	0.9351769474	-2.08899092	0.2172121769
C	-0.2749048652	-2.75277566	0.1537028678
C	-1.4042323279	-1.97979476	-0.0564552946
C	-1.3516065021	-0.60623744	-0.1784783511
O	-1.2161471739	2.29863099	-0.0423885302
H	1.1863799462	3.14840851	0.0170518804
H	2.0105811845	-0.24423101	0.1624323602
F	2.0444686504	-2.80787054	0.4321208303
H	-0.3391842979	-3.82483264	0.2763789348

F	-2.5886963551	-2.59691192	-0.1252841864
H	-2.2669617834	-0.04423394	-0.3100174984
H	-1.141061632	3.22732049	0.2016828138
O	-0.0387138411	3.85222223	2.3195148526
C	0.6777164876	3.03715066	2.8441402558
H	1.7067266988	3.30425401	3.1466070241
C	0.2937382579	1.62743757	3.0863098496
C	1.2945112915	0.69897857	3.3572784357
C	-1.0336633298	1.22630228	2.9572275388
C	0.9848843861	-0.64699855	3.453434651
H	2.3221312747	1.02777751	3.4552834746
C	-1.35949647	-0.11316864	3.068741564
H	-1.7914969074	1.96614400	2.7377906857
C	-0.3376443308	-1.01954970	3.2956019522
H	1.7388360085	-1.40180250	3.62187407
H	-2.3729413189	-0.46843431	2.953532371
N	-0.6720578992	-2.46015710	3.341820142
O	-1.8325075157	-2.76613919	3.203500258
O	0.2400204105	-3.23709097	3.501352422

3,5-OMe

40

O	1.150022883	2.23405445	-0.0427236848
B	-0.0760290485	1.62234671	-0.0993800947
C	-0.1246900478	0.05872947	-0.1543774731
C	1.0422938267	-0.67801758	0.0168861626
C	0.9819141367	-2.06241468	0.1403074252
C	-0.2416347805	-2.72688866	0.0746104254

C	-1.4081652076	-1.98203579	-0.1150964855
C	-1.3504893673	-0.59928069	-0.2307044466
O	-1.2569014609	2.30491488	-0.0374243761
H	1.1168427077	3.19320864	0.0195091028
H	2.0051801794	-0.18939422	0.0918701029
O	2.1628757102	-2.69536695	0.3566945172
H	-0.2896013762	-3.79763435	0.1931194628
O	-2.6428403728	-2.53909686	-0.1714727239
H	-2.2736715375	-0.04616089	-0.3452151608
H	-1.1857002507	3.22987343	0.2194903558
O	-0.0794131847	3.78082880	2.4071963571
C	0.6735490156	2.95566376	2.8589759404
H	1.711793337	3.22664398	3.1251565845
C	0.3254402823	1.52968976	3.0579912751
C	1.3517100482	0.61743302	3.2856050602
C	-0.9929071059	1.10149134	2.9282786084
C	1.0757782694	-0.73717425	3.3425898317
H	2.3730167687	0.96716434	3.3755094283
C	-1.2859494969	-0.24841511	2.9987424883
H	-1.7691570954	1.82888054	2.7330658899
C	-0.2404290707	-1.13546240	3.1908413082
H	1.8515072051	-1.47761550	3.4687850555
H	-2.2904640584	-0.62391626	2.8713647722
N	-0.5397213699	-2.58120794	3.1950362962
O	-1.6969071976	-2.91479600	3.0895144941
O	0.3930359567	-3.34637006	3.2922200542
C	2.1552896367	-4.10359267	0.4551099459
C	-2.7545676504	-3.93392879	0.0105701989
H	3.1900502397	-4.40116587	0.6027416398
H	1.776367713	-4.55848849	-0.4644086311
H	1.5560267214	-4.43702739	1.3054831272
H	-3.8155962407	-4.16121424	-0.04833064

H	-2.3719379186	-4.23473661	0.9891055368
H	-2.2256480585	-4.47795842	-0.7772279611

2-naph_close

38

O	1.1784431686	2.4011935831	-0.2552040136
B	-0.0481884639	1.7897262724	-0.1574845516
C	-0.0463579937	0.21981898	-0.224371792
C	-1.1731370934	-0.6157659844	0.0712475712
C	-0.9734064466	-2.0146773506	0.238714366
C	0.3164638111	-2.5660400133	0.0545085195
C	1.3704800133	-1.767919611	-0.2861196268
C	1.1819709499	-0.3777306049	-0.4108440105
O	-1.186403533	2.5065696933	0.0847461903
H	1.1616315862	3.3579028409	-0.15996335
C	-2.4927695613	-0.1155204518	0.232898149
C	-3.5238540208	-0.9421531381	0.5845314822
H	0.4468285722	-3.6334839698	0.1901347193
H	2.3537291333	-2.1936738409	-0.4404257095
H	2.0384713498	0.2478539571	-0.628776927
H	-1.0406877822	3.4165854481	0.3637318974
O	0.2611603939	3.7005153249	2.4516350349
C	0.8628632733	2.6832931012	2.6888679356
H	1.9674939795	2.6647733106	2.6753581526
C	0.2182414899	1.382263833	2.9857971225
C	1.0033565972	0.2328610025	3.0075069963
C	-1.1564674791	1.3070053799	3.1969096283
C	0.4213941191	-1.0028871256	3.2313172806

H	2.0679882172	0.3051758141	2.8195740765
C	-1.7474306291	0.0828272765	3.4524974193
H	-1.7461417308	2.2128727211	3.1508022371
C	-0.9425195174	-1.0440566654	3.4601705575
H	0.9941063986	-1.9180134571	3.2266794516
H	-2.80882729	-0.0184517552	3.6224916919
N	-1.5700344721	-2.357328585	3.7198187397
O	-2.7287425592	-2.3642763568	4.0613490819
O	-0.8811186132	-3.3411969458	3.5757761532
C	-3.3075069282	-2.3203684168	0.7955880693
C	-2.0605222392	-2.8436794612	0.6116330118
H	-2.6752496386	0.9372385835	0.0843831186
H	-4.5196039333	-0.5348089126	0.7068575887
H	-4.1284703652	-2.9571065135	1.097745216
H	-1.8765731031	-3.9005281724	0.7635848324

2-naph_far

38

O	1.0127431315	2.3857499	-0.17563461
B	-0.1573745865	1.6721244	-0.08903098
C	-0.1789292576	0.1016022	-0.15760989
C	0.9736393025	-0.7517261	-0.13368830
C	0.793987074	-2.1549355	0.01515378
C	-0.5132954177	-2.6943871	0.06857631
C	-1.6041322581	-1.8775547	-0.00293349
C	-1.4256610778	-0.4835596	-0.09998405
O	-1.3575129827	2.2947825	0.12368242
H	0.9015713379	3.3374132	-0.08445317

C	2.3064580442	-0.2634834	-0.19500605
C	3.3758274199	-1.1074494	-0.07480000
H	-0.6284745264	-3.7655166	0.18866104
H	-2.6029090524	-2.2914246	0.04586073
H	-2.2998883295	0.1554170	-0.09663508
H	-1.3018023346	3.2210127	0.37841752
O	0.0541925948	3.8112474	2.43682299
C	0.8273678316	2.9368249	2.73805764
H	1.9103427478	3.1437372	2.81627928
C	0.4366661898	1.5290723	2.97914170
C	1.4252687332	0.5521080	2.97894295
C	-0.9077718047	1.1830102	3.10988627
C	1.0804227778	-0.7875260	3.06835822
H	2.4641071898	0.8343196	2.85689898
C	-1.2638952205	-0.1457605	3.22692115
H	-1.6575578728	1.9622812	3.08906030
C	-0.2593490536	-1.1014572	3.18817452
H	1.8183392293	-1.5751341	3.01053562
H	-2.293750324	-0.4580776	3.31519015
N	-0.6448891869	-2.5281035	3.24851401
O	-1.8212194635	-2.7839766	3.35758260
O	0.2404358287	-3.3483834	3.17809500
C	3.1875317029	-2.4942589	0.10976925
C	1.9220007367	-3.0028438	0.14478576
H	2.470514985	0.7940231	-0.33285615
H	4.3812864853	-0.7084493	-0.12485022
H	4.0441384695	-3.1474031	0.21386527
H	1.7542709374	-4.0641146	0.28394853

2-F_close

32

O	1.2534154382	2.20291344	-0.009086429
B	0.0197479469	1.59853393	-0.036391453
C	0.0228047955	0.03066580	-0.047667275
C	1.2419403383	-0.65818480	-0.039054609
C	1.3063216538	-2.03824636	0.064996643
C	0.1317902888	-2.77597414	0.159236780
C	-1.0961280749	-2.13330963	0.138292268
C	-1.1217807053	-0.75307578	0.036710613
O	-1.1481640742	2.29388757	0.001377610
H	1.2272591049	3.16433808	-0.003764068
H	2.1549778382	-0.07857164	-0.099962846
H	2.264637103	-2.53988764	0.077683942
H	0.1687109025	-3.85325549	0.255468170
H	-2.0286899483	-2.67522835	0.219993336
F	-2.3260172178	-0.16574071	0.056568994
H	-1.0604064415	3.22569366	0.229171240
O	-0.0267896546	3.87348452	2.354476669
C	0.6636271284	2.99874600	2.813174254
H	1.7259517753	3.18696320	3.053832657
C	0.2005450273	1.61249058	3.052915626
C	1.1513412078	0.61978024	3.260986279
C	-1.155616907	1.29678263	2.980858862
C	0.7617344072	-0.70559786	3.365434205
H	2.2018764005	0.88159313	3.302911326
C	-1.5607182242	-0.01782987	3.105823681
H	-1.8736624091	2.08406961	2.796356180
C	-0.5877140943	-0.98990080	3.281439367

H	1.4731826259	-1.50784323	3.490585799
H	-2.5990150704	-0.30503706	3.032260796
N	-1.0103015182	-2.40561785	3.350163236
O	-2.1905459079	-2.64015563	3.236914950
O	-0.1480137346	-3.23828559	3.505393195

2-F_far

32

O	1.2069965061	2.2949319037	-0.0167520214
B	0.0288125565	1.6027533218	-0.0356453586
C	0.027187677	0.0349918957	-0.0397674164
C	-1.1926232977	-0.6486323482	-0.1165952133
C	-1.2706099505	-2.0284803202	-0.0199811389
C	-0.1087304876	-2.7708854647	0.1579007262
C	1.1188970703	-2.1320666296	0.2319605746
C	1.1583224124	-0.752594406	0.1308828395
O	-1.1951851403	2.2106038374	-0.0059474109
H	1.1036493901	3.2516785958	-0.010319047
H	-2.0970544655	-0.0642966219	-0.2337894901
H	-2.2298542314	-2.5257516798	-0.0679546374
H	-0.157601153	-3.8476935515	0.2555281769
H	2.0397544361	-2.6786839644	0.3840128928
F	2.3604646884	-0.1681325048	0.2514645352
H	-1.1831209625	3.1489296025	0.2091218529
O	-0.1302022221	3.8720500157	2.362810853
C	0.6319370173	3.0432550995	2.7927454995
H	1.6883579369	3.2972576843	2.9934999059
C	0.2635097658	1.630411197	3.0446730763

C	1.277954029	0.6946510147	3.2146463915
C	-1.0730151051	1.2368474575	3.0203428549
C	0.9676897033	-0.6507569558	3.3277990888
H	2.3127919754	1.0145019007	3.2135510596
C	-1.3968038789	-0.1006142571	3.1456494906
H	-1.8401491552	1.9846608005	2.8716229726
C	-0.3646294498	-1.0151697344	3.2818626823
H	1.729578841	-1.4097772311	3.424489524
H	-2.4180711731	-0.449626044	3.1116483996
N	-0.7025885145	-2.4531329768	3.3536772145
O	-1.8715147296	-2.7545158388	3.2964119814
O	0.2125174913	-3.2363251578	3.4575491414

2-Me_close

35

O	1.1655480359	2.254750144	0.0044817403
B	-0.0565475756	1.6230353138	-0.0011044721
C	-0.046093043	0.0561128972	-0.0476933473
C	1.2046940548	-0.5695387456	-0.0183343385
C	1.3359517605	-1.9467312877	0.0715252176
C	0.1940796811	-2.731247679	0.1321536361
C	-1.0569551906	-2.1328503176	0.0776059705
C	-1.2009291111	-0.7494615716	-0.0176270243
O	-1.2281862099	2.3197782347	0.0986809526
H	1.1199930598	3.2145741867	0.0417794209
H	2.092775126	0.0503971616	-0.048326382
H	2.3169939708	-2.4032074827	0.1003477684
H	0.2726355605	-3.8074539031	0.2205924576

H	-1.9451130732	-2.7521957444	0.1197897632
C	-2.5903582056	-0.1735456567	-0.076429314
H	-1.1285144515	3.2436659875	0.3529254721
O	0.0074991638	3.8561851059	2.4602498562
C	0.7252242446	2.9725335947	2.8565975682
H	1.8000141432	3.1568374098	3.0365270041
C	0.276377054	1.5812495687	3.0911068555
C	1.2336126257	0.5817524643	3.2151977326
C	-1.0828978652	1.2701607895	3.0942124583
C	0.8462338617	-0.7463677931	3.3002422317
H	2.285711308	0.8401154623	3.2004866096
C	-1.4833020997	-0.0467722887	3.2009402717
H	-1.8067196105	2.0669177974	2.9860330336
C	-0.5056413677	-1.0266199224	3.2816567127
H	1.5612814298	-1.553250293	3.3554346123
H	-2.5246370343	-0.3328749113	3.1924093139
N	-0.9313351155	-2.4418844043	3.3251446967
O	-2.114859271	-2.6694572895	3.2302510818
O	-0.0694243202	-3.280027131	3.4459036631
H	-2.7292791603	0.4297673625	-0.9737193804
H	-2.7804954256	0.4906711937	0.7672009513
H	-3.3323369501	-0.9708182526	-0.0708427943

2-Me_far

35

O	1.2183053235	2.3352843286	0.0005245295
B	0.0579044688	1.6028440942	-0.0594588193
C	0.0974741577	0.0356900506	-0.0608507122

C	-1.1363516353	-0.6234669195	-0.0786287893
C	-1.2362849705	-2.001699418	0.0337119332
C	-0.0784801854	-2.7524563532	0.170898213
C	1.1569062004	-2.1202496917	0.171387298
C	1.2695910113	-0.7367424343	0.0491214963
O	-1.1735144149	2.2005940509	-0.0607330355
H	1.0813996977	3.2879094342	0.0062333172
H	-2.0375202637	-0.0277118832	-0.1601060819
H	-2.2048963349	-2.484277983	0.0313153669
H	-0.1339039776	-3.8278858509	0.2846029079
H	2.057192234	-2.7145476433	0.2750680289
C	2.6443946224	-0.1233467891	0.0594629637
H	-1.1783052057	3.1345233772	0.1726624564
O	-0.24520003	3.9100457509	2.3169429235
C	0.5068430389	3.1016402806	2.8002312212
H	1.5447055032	3.3812367945	3.0583325235
C	0.1562492451	1.6843153465	3.0469230736
C	1.1818856162	0.7735736567	3.2759610062
C	-1.1677385456	1.2588935466	2.9617731346
C	0.900541994	-0.5792893461	3.3758057843
H	2.2056521647	1.1221509465	3.3426857054
C	-1.4651211941	-0.0845443276	3.0865628698
H	-1.9444617521	1.986261934	2.7684379401
C	-0.4196421366	-0.9742221609	3.2747931382
H	1.6737650376	-1.3203913547	3.5130694263
H	-2.475394455	-0.4578944065	3.0101211107
N	-0.7285668668	-2.4185506704	3.3502637498
O	-1.8881160273	-2.7454753047	3.2558056302
O	0.1986136958	-3.179521668	3.4984845924
H	2.7716641698	0.5473626924	0.9106351378
H	2.8165475374	0.479683771	-0.8315575488
H	3.406637967	-0.8998812802	0.1095322583

2,4-Me_close

38

O	1.250216091	2.152440482	0.0405314761
B	-0.0206471107	1.6282836971	-0.0321309916
C	-0.1535199381	0.0702045785	-0.0906628288
C	-1.3821993115	-0.6215530141	-0.1279258527
C	-1.3817281275	-2.0053207662	-0.0090879135
C	-0.2096425897	-2.7462604767	0.140817428
C	0.9993024722	-2.0631908653	0.1455696725
C	1.0178598896	-0.6801136042	0.0292713775
O	-1.1306988588	2.4251942091	0.0200287631
H	1.2823757855	3.1131089327	0.0616744448
C	-2.7039282281	0.0869431426	-0.2606883132
H	-2.3304625718	-2.5325552511	-0.015153711
C	-0.2785925148	-4.2377117417	0.3062053243
H	1.9262838954	-2.6138342248	0.2555709875
H	1.9670059444	-0.1576803514	0.05564538
H	-0.969585902	3.3273578583	0.3173655653
O	-0.041279553	3.856627305	2.4400312771
C	0.669972871	2.9977903263	2.8971708467
H	1.7191942523	3.220313611	3.1652751903
C	0.2565198093	1.5905301825	3.099283828
C	1.2441058551	0.6351444773	3.3132952453
C	-1.0812539776	1.2183350237	2.9847456288
C	0.9110397362	-0.7071535729	3.3731119084
H	2.2801702882	0.941462148	3.3928480898
C	-1.4293232386	-0.1179992787	3.0596513959
H	-1.8281052228	1.9801597127	2.8040873694
C	-0.4206130794	-1.0504688916	3.2368854658

H	1.6540497767	-1.4802162096	3.4985116289
H	-2.4510681269	-0.4517741279	2.9521125788
N	-0.7781825257	-2.4848697228	3.257212231
O	-1.9457322748	-2.7736288998	3.1345423368
O	0.1215156203	-3.2817612826	3.3906207698
H	-2.7180377229	0.7467260172	-1.1274740177
H	-2.898278404	0.721697577	0.6058804635
H	-3.5139599892	-0.6351379092	-0.3541901951
H	-0.9125463183	-4.4988253298	1.1563599114
H	0.7092223582	-4.6623518282	0.4775301289
H	-0.7071340892	-4.7060110523	-0.5819622103

2,4-Me_far

38

O	1.3426092063	2.2100864738	-0.0292143946
B	0.1416174926	1.5513802777	-0.144376392
C	0.077407192	-0.0118801318	-0.1238760908
C	1.1796999964	-0.8598438941	0.0801600849
C	0.9624378349	-2.2282831988	0.2375712874
C	-0.3055169402	-2.796126483	0.1838232546
C	-1.3902164561	-1.9550574646	-0.0555068503
C	-1.193815339	-0.5943082925	-0.2020602508
O	-1.0469248506	2.2285795872	-0.213624882
H	1.2656022988	3.169163735	-0.0468816961
C	2.592534617	-0.3447944626	0.156318646
H	1.81447921	-2.8754895167	0.4179733165
C	-0.513725497	-4.2674978687	0.4025708521
H	-2.3896160737	-2.3720684457	-0.1049778208

H	-2.0487325868	0.0530053204	-0.3575593178
H	-1.0037626365	3.1613560993	0.020316241
O	-0.1817960477	3.9077381207	2.1927307728
C	0.5231727763	3.0934090528	2.7333865126
H	1.5497714991	3.3572957483	3.0474333669
C	0.1306245674	1.687590318	2.9828576955
C	1.1247131585	0.7633625117	3.2876529513
C	-1.1923985119	1.2830357072	2.8226363923
C	0.814990183	-0.582578786	3.3859644253
H	2.1484855731	1.0959018695	3.4115339683
C	-1.5198813376	-0.0545242138	2.9444242621
H	-1.9431745072	2.0195745706	2.5703323157
C	-0.5030849741	-0.9579288426	3.2060959418
H	1.5661699611	-1.3342847445	3.5775895951
H	-2.5293366862	-0.4124894945	2.807771257
N	-0.8377968835	-2.3961801135	3.273794647
O	-1.997092458	-2.7081721986	3.1289696073
O	0.0686558023	-3.1730386431	3.4654421538
H	2.7190085814	0.3385941627	0.9975042447
H	2.8563325631	0.2202352508	-0.7372572078
H	3.2937080133	-1.1708634411	0.2686704345
H	0.4354874798	-4.7920233218	0.4990216477
H	-1.0717531786	-4.7074750022	-0.4254922184
H	-1.0889830416	-4.4383002457	1.3156812472

4-F

32

O	1.2209894743	2.2286793515	-0.0092107719

B	0.0180111688	1.5679495425	-0.0216966797
C	0.0418006929	0.008052083	-0.0492094791
C	1.2527366195	-0.6893145732	-0.0616936649
C	1.2912353934	-2.0723353065	0.0185682738
C	0.0921804009	-2.7573739632	0.1075754675
C	-1.1314263417	-2.1131438301	0.1095774423
C	-1.142436717	-0.7277415226	0.0309096158
O	-1.1882524778	2.2043455192	0.0441435153
H	1.1548572738	3.1880506629	0.0167191518
H	2.1804518037	-0.1327822982	-0.1228719092
H	2.2201148496	-2.6263397329	0.0140135333
F	0.1190223221	-4.0917522736	0.1868453343
H	-2.0402069893	-2.6953228738	0.1832827557
H	-2.0894152469	-0.2012414575	0.04780003
H	-1.1508607534	3.1381633586	0.2764476162
O	-0.0065787197	3.8874764521	2.3628934609
C	0.6877496641	3.0059894113	2.8034034857
H	1.7608629509	3.1788601429	3.0026725653
C	0.208486765	1.6300016243	3.0672109682
C	1.1422687473	0.6107406982	3.2091628147
C	-1.1573514029	1.3508180829	3.0742709629
C	0.7234721623	-0.7063519155	3.3187831596
H	2.2001934079	0.8441409695	3.1911917941
C	-1.5892455763	0.0462442521	3.2042311475
H	-1.8621930049	2.1616936215	2.9500277198
C	-0.6345331292	-0.9543518464	3.30560275
H	1.4194668124	-1.5286583564	3.3904783193
H	-2.6370574915	-0.2145291298	3.1981744228
N	-1.0930218173	-2.3595163404	3.3670069267
O	-2.2843627204	-2.5574883744	3.3082575014
O	-0.2481581207	-3.2172619782	3.4625317701

4-Me

35

O	1.2578050199	2.2265628479	0.0556789356
B	0.034698667	1.6123353364	-0.0571872799
C	-0.0137667536	0.0541240928	-0.1123952314
C	1.1397938304	-0.7127780042	0.0431662393
C	1.0727548661	-2.096409651	0.150157516
C	-0.1504833611	-2.7575827086	0.0978731002
C	-1.3063005364	-1.9957801485	-0.0878651937
C	-1.2382883705	-0.6178974202	-0.1901803253
O	-1.1473264832	2.2982859398	-0.0557558221
H	1.2221454892	3.1871609993	0.0858194178
H	2.1006133055	-0.2144746877	0.1059279479
H	1.9794904941	-2.6732967191	0.2921384243
C	-0.2454826001	-4.2477921972	0.2622437777
H	-2.2667339513	-2.4973081479	-0.1300023779
H	-2.1481283278	-0.040022905	-0.305758021
H	-1.0905692547	3.2199275775	0.2171104214
O	-0.1715611479	3.8502107127	2.3937636624
C	0.5644381773	3.0314904287	2.8839964637
H	1.5926408133	3.3063731633	3.1831041802
C	0.2095787308	1.6083292591	3.0874407409
C	1.2309093259	0.6965519618	3.3343711817
C	-1.1068359042	1.1794794119	2.9383947421
C	0.9532348714	-0.6582909899	3.3897872041
H	2.2502975045	1.0468922751	3.4424489995
C	-1.4003745648	-0.1700541189	3.0083646081
H	-1.8799304881	1.9072383769	2.7320911713
C	-0.3584702814	-1.0584575812	3.215451687

H	1.724543109	-1.3988152203	3.5390277576
H	-2.4033960927	-0.5461954387	2.8727805054
N	-0.6555320734	-2.5064259168	3.2224442417
O	-1.8085935235	-2.8423346453	3.0819752772
O	0.2743604907	-3.2669467804	3.3611549782
H	0.7402474091	-4.697616364	0.3689305609
H	-0.7431670867	-4.7020895908	-0.5962043174
H	-0.8287113027	-4.4962931473	1.1520048266

4-OMe

36

O	1.2091382668	2.2574321166	0.0384531759
B	-0.0115316929	1.6300792669	-0.0403751114
C	-0.0467787576	0.076808906	-0.1181311589
C	1.1167712029	-0.6819490675	-0.027373646
C	1.0874882676	-2.0698227176	0.0514244114
C	-0.1411628696	-2.7262358684	0.0338881209
C	-1.3227342508	-1.9872930247	-0.082913478
C	-1.267334604	-0.6120602734	-0.156645017
O	-1.1991015301	2.3064258976	0.0121320536
H	1.1628994327	3.2171222456	0.0792447506
H	2.0743956555	-0.1745361695	0.0052349909
H	2.0111225756	-2.623492742	0.138391375
O	-0.292193865	-4.0636170106	0.1335452892
H	-2.2623393678	-2.5238751029	-0.0937545046
H	-2.1885194956	-0.0445161701	-0.2216041522
H	-1.140022857	3.2242758158	0.296921638
O	-0.1499815258	3.8454845001	2.4457162427

C	0.6048568475	3.0145043286	2.8839656305
H	1.648148477	3.2788656992	3.136692043
C	0.2523720796	1.5897623895	3.079561369
C	1.2793569258	0.6690793511	3.2575243162
C	-1.07249283	1.1696933842	2.989290537
C	0.9971835794	-0.685612026	3.3017875282
H	2.3048740399	1.012762718	3.3196391497
C	-1.3698944764	-0.1788237158	3.0539016514
H	-1.8506176757	1.9046950903	2.8340988317
C	-0.3238152346	-1.0763414007	3.1905403415
H	1.770995249	-1.4322832842	3.3973007068
H	-2.3811063204	-0.5474752282	2.9668094626
N	-0.6313360801	-2.5228905921	3.1934096038
O	-1.7930019465	-2.8472826445	3.1626767452
O	0.3047875902	-3.2908705749	3.2250711776
C	0.8634416502	-4.8491793644	0.3363931944
H	0.5173021715	-5.8744878103	0.4314507956
H	1.3779026647	-4.5525795864	1.253026726
H	1.5450287042	-4.7719673353	-0.5149947905

4-tBu

44

O	1.0600680803	2.20430383	-0.051776099
B	-0.1455256843	1.54697890	-0.008864057
C	-0.1422237409	-0.01265700	-0.026796943
C	1.0458272426	-0.73825089	0.011340389
C	1.0455099851	-2.12728515	0.064705629
C	-0.1471705778	-2.84965593	0.064045504

C	-1.3407612907	-2.11961395	0.028897464
C	-1.3381442817	-0.73610018	-0.011862314
O	-1.3441200278	2.19589324	0.092238115
H	0.9928667363	3.16372369	-0.056903617
H	1.9902583375	-0.20547117	0.007415007
H	1.9934200781	-2.64599090	0.101133647
C	-0.1830550007	-4.37687655	0.017183273
H	-2.2892572181	-2.64183917	0.023584755
H	-2.2793116715	-0.19836488	-0.029452219
H	-1.2901533706	3.13082633	0.316453426
O	-0.1401479654	3.91636135	2.330155883
C	0.6587573408	3.12147629	2.757158294
H	1.7121867481	3.41394172	2.920650486
C	0.3454383181	1.70469022	3.052602856
C	1.3971624066	0.80746724	3.203181741
C	-0.9761864034	1.26582192	3.083448489
C	1.1399095827	-0.54513470	3.348448977
H	2.4189887371	1.16553027	3.167291703
C	-1.2477512894	-0.07925045	3.247924569
H	-1.7745019513	1.98231419	2.946088790
C	-0.1800709332	-0.95404371	3.362458770
H	1.9295959489	-1.27660232	3.431302400
H	-2.2567480318	-0.46335592	3.260393862
N	-0.4652402749	-2.39710515	3.494207785
O	-1.6235680566	-2.73405893	3.561598084
O	0.4799341809	-3.15257130	3.530433844
C	1.1920208804	-4.99212557	0.279431543
C	-1.1584890581	-4.93976099	1.058551557
C	-0.6506092436	-4.79268329	-1.386294115
H	1.5834665559	-4.67918292	1.249557264
H	1.9126964794	-4.72317038	-0.494598171
H	1.1053870289	-6.07979892	0.284935547

H	-0.8355353463	-4.69816385	2.071220208
H	-1.1995597505	-6.02705908	0.964501028
H	-2.1708105683	-4.55945531	0.924801016
H	0.0237165751	-4.40303760	-2.151130661
H	-1.6529654307	-4.41511602	-1.594263746
H	-0.6722040749	-5.88184698	-1.466099962

4-CF3

35

O	1.1699734243	2.2396486584	-0.0581594794
B	-0.0424151249	1.6021281688	-0.0310990648
C	-0.0430806184	0.0361163927	-0.0723401848
C	1.1574869635	-0.6766304374	-0.1056596104
C	1.1703265879	-2.05938263	-0.0197482836
C	-0.0315692321	-2.7465718418	0.0957426269
C	-1.2400963819	-2.0641515302	0.10484917
C	-1.2373630977	-0.6786819379	0.0229550948
O	-1.2365772861	2.2488571452	0.0878062147
H	1.1253357937	3.1998095151	-0.015719681
H	2.091847343	-0.1331925459	-0.1791621749
H	2.1037722208	-2.6068754068	-0.0299534888
C	-0.018238258	-4.2498088619	0.1580793368
H	-2.1687285367	-2.6118046937	0.1979115268
H	-2.1751768693	-0.1371817304	0.0555663985
H	-1.1820252784	3.1789488959	0.3323295738
O	0.0312224133	3.8616475591	2.3788054904
C	0.7394250942	2.9811281493	2.798723416
H	1.81491137	3.1597595186	2.9791517835

C	0.274385102	1.5992969588	3.0584199174
C	1.2178442508	0.5847451054	3.1670794307
C	-1.0893357715	1.3112320188	3.0959597902
C	0.8098701945	-0.7364497274	3.2684674117
H	2.2736324042	0.8254065954	3.1297898871
C	-1.5096817934	0.0022763616	3.2203541781
H	-1.8022976524	2.118726616	2.9989662044
C	-0.5465912511	-0.9934673878	3.2834891816
H	1.5121736592	-1.5555345193	3.3123841275
H	-2.5556808959	-0.2655948885	3.2364313106
N	-0.9939690081	-2.4031656937	3.3301097322
O	-2.1848791714	-2.6082660317	3.3096355833
O	-0.1394193885	-3.2560198736	3.3721142815
F	1.0580079799	-4.7133705759	0.8009495064
F	-1.0984768952	-4.737885748	0.7692437335
F	0.0092877096	-4.7846915976	-1.0738729409

23. References – Multivariate Modelling Supporting Information

1. Frisch, M. J.; Trucks, G. W.; Schlegel, H. B.; Scuseria, G. E.; Robb, M. A.; Cheeseman, J. R.; Scalmani, G.; Barone, V.; Petersson, G. A.; Nakatsuji, H.; Li, X.; Caricato, M.; Marenich, A. V.; Bloino, J.; Janesko, B. G.; Gomperts, R.; Mennucci, B.; Hratchian, H. P.; Ortiz, J. V.; Izmaylov, A. F.; Sonnenberg, J. L.; Williams-Young, D.; Ding, F.; Lipparini, F.; Egidi, F.; Goings, J.; Peng, B.; Petrone, A.; Henderson, T.; Ranasinghe, D.; Zakrzewski, V. G.; Gao, J.; Rega, N.; Zheng, G.; Liang, W.; Hada, M.; Ehara, M.; Toyota, K.; Fukuda, R.; Hasegawa, J.; Ishida, M.; Nakajima, T.; Honda, Y.; Kitao, O.; Nakai, H.; Vreven, T.; Throssell, K.; Montgomery, J. A., Jr.; Peralta, J. E.; Ogliaro, F.; Bearpark, M. J.; Heyd, J. J.; Brothers, E. N.; Kudin, K. N.; Staroverov, V. N.; Keith, T. A.; Kobayashi, R.; Normand, J.; Raghavachari, K.; Rendell, A. P.; Burant, J. C.; Iyengar, S. S.; Tomasi, J.; Cossi, M.; Millam, J. M.; Klene, M.; Adamo, C.; Cammi, R.; Ochterski, J. W.; Martin, R. L.; Morokuma, K.; Farkas, O.; Foresman, J. B.; Fox, D. J. Gaussian 16, Revision C.01, Gaussian, Inc.: Wallingford CT, **2016**.

2. Zhao, Y.; Truhlar, D. G. The M06 Suite of Density Functionals for Main Group Thermochemistry, Thermochemical Kinetics, Noncovalent Interactions, Excited States, and Transition Elements: Two New Functionals and Systematic Testing of Four M06-Class Functionals and 12 Other Functionals. *Theoretical Chemistry Accounts* **2007**, *120* (1-3), 215–241. https://doi.org/10.1007/s00214-007-0310-x

3. Valero, R.; Gomes, J. R. B.; Truhlar, D. G.; Illas, F. Good Performance of the M06 Family of Hybrid Meta Generalized Gradient Approximation Density Functionals on a Difficult Case: CO Adsorption on MgO(001). *The Journal of Chemical Physics* **2008**, *129* (12), 124710. https://doi.org/10.1063/1.2982923.

4. Weigend, F.; Ahlrichs, R. Balanced Basis Sets of Split Valence, Triple Zeta Valence and Quadruple Zeta Valence Quality for H to Rn: Design and Assessment of Accuracy. *Physical Chemistry Chemical Physics* **2005**, *7* (18), 3297. https://doi.org/10.1039/b508541a.

5. Weigend, F. Accurate Coulomb-Fitting Basis Sets for H to Rn. *Physical Chemistry Chemical Physics* **2006**, *8* (9), 1057. https://doi.org/10.1039/b515623h.

6. Merrick, J. P.; Moran, D.; Radom, L. An Evaluation of Harmonic Vibrational Frequency Scale Factors. *The Journal of Physical Chemistry A* **2007**, *111* (45), 11683–11700. https://doi.org/10.1021/jp073974n.

7. Verloop, A.; Tipker, J. Use of Linear Free Energy Related and Other Parameters in the Study of Fungicidal Selectivity. *Pesticide Science* **1976**, *7* (4), 379–390. https://doi.org/10.1002/ps.2780070410.

8. Glendening, E. D., Reed, A. E., Carpenter, J. E. & Weinhold, F. NBO Version 3.1. included in Gaussian 16, Gaussian, Inc.: Wallingford CT, **2016**.

9. R Core Team. R: A language and environment for statistical computing. R Foundation for Statistical Computing: Vienna, Austria, **2014**. http://www.Rproject.org/

10. RStudio Team. RStudio: Integrated Development for R. RStudio, PBC.: Boston, MA, **2020**. http://www.rstudio.com/.

11. Wickham, H. ggplot2: Elegant Graphics for Data Analysis. Springer-Verlag: New York, **2016**. https://ggplot2.tidyverse.org

12. Wickham, H. and Henry, L. tidyr: Tidy Messy Data. R package version 1.1.0.: **2019**. https://CRAN.R-project.org/package=tidyr

13. Wickham, H. (2007). Reshaping Data with the reshape Package. *Journal of Statistical Software*, **2007**, *21* (12), 1-20. http://www.jstatsoft.org/v21/i12/

14. Wickham, H. and Seidel, D. scales: Scale Functions for Visualization. R package version 1.1.1.: **2020**. https://CRAN.R-project.org/package=scales

15. Muller, K. and Wickham, H. tibble: Simple Data Frames. R package version 3.0.3.: **2020**. https://CRAN.R-project.org/package=tibble

16. Kuhn, M. caret: Classification and Regression Training. R package version 6.0-86.: **2020**. https://CRAN.R-project.org/package=caret

17. Wickham, H. The Split-Apply-Combine Strategy for Data Analysis. *Journal of Statistical Software*, **2011**, *40* (1), 1-29. http://www.jstatsoft.org/v40/i01/.

18. Wickham, H.; Fransois, R.; Henry, L.; Muller, K. dplyr: A Grammar of Data Manipulation. R package version 1.0.0.: **2020**. https://CRAN.R-project.org/package=dplyr

19. Dowle, M. and Srinivasan, A. data.table: Extension of data.frame. R package version 1.12.8.: **2019**. https://CRAN.R-project.org/package=data.table

20. Domencich, T. and McFadden, D. L. Urban Travel Demand: A Behavioral Analysis. North-Holland Publishing Co.: Netherlands, **1975**.

21. Slowikowski, K. ggrepel: Automatically Position Non-Overlapping Text Labels with 'ggplot2'. R package version 0.9.1.:**2021**. https://CRAN.R-project.org/package=ggrepel

22. Chang, W. extrafont: Tools for using fonts. R package version 0.17.: **2014**. https://CRAN.R-project.org/package=extrafont

23. Legault, C. Y. CYLview. Version 1.0b; Université de Sherbrooke: **2009**. http://www.cylview.org.

24. Hanwell, M. D.; Curtis, D. E.; Lonie, D. C.; Vandermeersch, T.; Zurek, E.; Hutchison, G. R. Avogadro: An Advanced Semantic Chemical Editor, Visualization, and Analysis Platform. *Journal of Cheminformatics* **2012**, *4* (1). https://doi.org/10.1186/1758-2946-4-17.
